# Prognostic role of ARID1A negative expression in gastric cancer

**DOI:** 10.1038/s41598-019-43293-5

**Published:** 2019-05-01

**Authors:** Mai Ashizawa, Motonobu Saito, Aung Kyi Thar Min, Daisuke Ujiie, Katsuharu Saito, Takahiro Sato, Tomohiro Kikuchi, Hirokazu Okayama, Shotaro Fujita, Hisahito Endo, Wataru Sakamoto, Tomoyuki Momma, Shinji Ohki, Akiteru Goto, Koji Kono

**Affiliations:** 1 Department of Gastrointestinal Tract Surgery, Fukushima Medical University School of Medicine, Fukushima, 960-1295 Japan; 2 Department of Cellular and Organ Pathology, Graduate School of Medicine, Akita University, Akita, 010-8543 Japan

**Keywords:** Gastric cancer, Tumour biomarkers, Cell growth

## Abstract

*AT-rich interactive domain 1A* (*ARID1A*) functions as a tumor suppressor and several therapeutic targets in *ARID1A*-mutated cancers are under development. Here, we investigated the prognostic value of ARID1A for gastric cancer and its association with expression of PD-L1 and p53. ARID1A expression was examined by immunohistochemistry and negative expression of ARID1A was detected in 39 (19.5%) of 200 cases in a test cohort and in 40 (18.2%) of 220 cases in a validation cohort. Negative expression of ARID1A was associated with worse overall survival in undifferentiated cases, particularly early-stage cases. Negative expression of ARID1A was detected in 11 (50%) of 22 PD-L1-positive cases and in 68 (17.1%) of 398 PD-L1-negative cases in a combined cohort. Negative expression of ARID1A was detected in 45 (22%) of 205 p53-positive cases and in 34 (15.8%) of 215 p53-negative cases in a combined cohort. In addition, expression of EZH2, a potential synthetic lethal target in *ARID1A*-mutated tumors, was detected in 79 ARID1A-negative cases. An ARID1A-knockdown gastric cancer cell line was subjected to microarray analysis, but no actionable targets or pathways were identified. The present results indicate that ARID1A may serve as an early-stage prognostic biomarker for undifferentiated gastric cancer.

## Introduction

*AT-rich interactive domain 1A* (*ARID1A*), located on chromosome 1p36.11, is a subunit of the Switch/Sucrose Non-fermentable (SWI/SNF) chromatin remodeling complex. *ARID1A* has a tumor suppressor function, and inactivating *ARID1A* mutations occur in various types of cancer^1^. In gastric cancer, *ARID1A* is frequently mutated in Epstein-Barr virus (EBV)-positive and microsatellite instability (MSI) subtypes/DNA mismatch repair (MMR) protein deficiency characterized by frequent overexpression of PD-L1/L2, *CDKN2A* methylation, *PIK3CA* mutations, and rare *TP53* mutations^2–5^. Because ARID1A recruits MSH2 (an MMR protein) during DNA replication, inactivation of ARID1A leads to functional impairment of MMR and accumulation of gene mutations^6^. Therefore, evaluation of ARID1A expression in gastric cancer will increase our understanding of impaired MMR and tumor phenotypes, and may lead to development of novel immune checkpoint blockade therapies. Since most *ARID1A* mutations in gastric cancer are truncating mutations that result in lack of ARID1A protein expression, immunohistochemical (IHC) staining can be used as a surrogate marker for *ARID1A* mutations^3,5^.

A meta-analysis including 15 gastric cancer cohorts revealed that low ARID1A expression is associated significantly with worse patient survival and adverse clinicopathological factors, such as lymphatic invasion and lymph node metastasis^7^. Several therapeutic targets in *ARID1A*-mutated cancers are currently under development, including enhancer of zeste homolog 2 (EZH2)^8^. EZH2, a histone methyltransferase subunit of a Polycomb repressor complex, is a potential target in *ARID1A*-mutated tumors in a synthetic lethal manner^9^. Several phase 1 or 2 clinical trials of EZH2 inhibitors for advanced solid tumors are ongoing, and the therapeutic use of EZH2 inhibitors has shown promise in *ARID1A*-mutated gastric cancer^8^.

In the present study, we performed IHC staining for ARID1A in two independent cohorts of patients with surgically resected gastric cancer. To clarify the molecular features of gastric cancer with negative ARID1A expression, we investigated the prognostic value of ARID1A and the relationship between ARID1A and cancer-related molecules, such as p53, MMR status (deficient MMR (dMMR) or proficient MMR (pMMR)), and PD-L1. In addition, we performed microarray analysis to find *ARID1A*-related genes and pathways.

## Results

### Association between ARID1A expression and clinicopathologic variables

**Figure 1:**
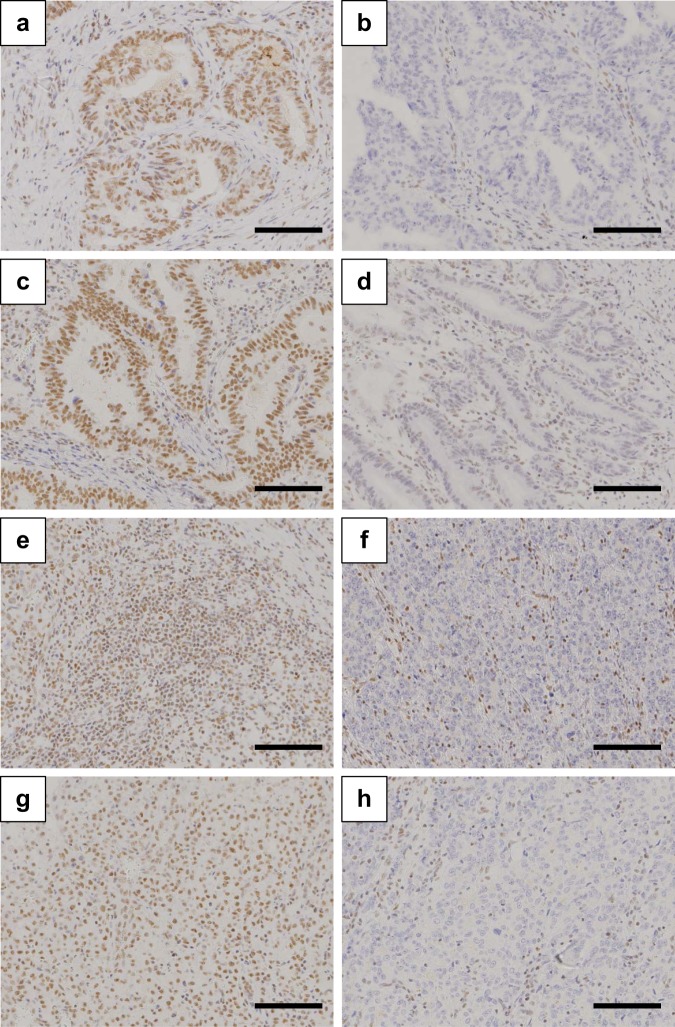
Representative images showing immunohistochemical staining for ARID1A in gastric cancer (**a–h**). (**a,c**) Positive ARID1A staining in differentiated tumor tissues. (**b,d**) Negative ARID1A staining in differentiated tumor tissues. (**e,g**) Positive ARID1A staining in undifferentiated tumor tissues. (**f,h**) Negative ARID1A staining in undifferentiated tumor tissues. Scale bars = 100 μm.

ARID1A expression was evaluated using IHC staining in two independent cohorts of gastric cancer patients (Fig. 1). ARID1A expression was negative in 39 cases (19.5%) in the test cohort, whereas it was negative in 40 cases (18.2%) in the validation cohort. The association of ARID1A expression with the clinicopathological characteristics of gastric cancer patients was investigated (Table 1). ARID1A expression was not associated with age, gender, or histological type in both cohorts. However, negative expression of ARID1A was associated with TNM stage and depth of invasion. Lymphatic invasion and venous invasion were significantly associated with negative ARID1A expression, suggesting that ARID1A plays a role in cancer progression. We also examined the link between ARID1A expression and MMR status (Supplementary Fig. S1). In the test cohort, dMMR was identified in four (10%) of 39 ARID1A-negative tumors compared with eight (5%) of 153 ARID1A-positive tumors in the test cohort, although the difference was not statistically significant (P = 0.255) (Table 1). EBV-positive gastric cancer was frequently identified in ARID1A-negative cases (4 of 16 cases, 25%) compare to ARID1A-positive cases (0 of 21 cases, 0%) (P = 0.028) (Table 1 and Supplementary Fig. S2).

**Table 1:** Clinicopathological characteristics of stomach cancer according to ARID1A expression.

	Test cohort				Validation cohort			
		ARID1A				ARID1A		
	Total (n = 200)	Positive (n = 161)	Negative (n = 39)	P value	Total (n = 220)	Positive (n = 180)	Negative (n = 40)	P value
**Age-year**				0.868				0.486
Mean	67.1	66.9	67.5		64.5	64.1	65.4	
Range	30–90	30–90	42–85		29–91	29–91	40–87	
**Gender-no. (%)**				0.847				0.445
Male	138	110 (68)	28 (72)		155	129 (72)	26 (65)	
Female	62	51 (32)	11 (28)		65	51 (28)	14 (35)	
**Histological type-no. (%)**				0.858				0.008
Differentiated	107	87 (54)	20 (51)		121	107 (59)	14 (35)	
Undifferentiated	93	74 (46)	19 (49)		99	73 (41)	26 (65)	
**TNM Stage-no. (%)**				<0.0001				0.040
I	105	94 (58)	11 (28)		107	94 (52)	13 (33)	
II	32	25 (15)	7 (18)		36	28 (16)	8 (20)	
III	42	27 (17)	15 (39)		52	38 (21)	14 (35)	
IV	21	15 (9)	6 (15)		25	20 (11)	5 (12)	
**Depth of invasion-no. (%)**				<0.0001				0.002
T1	96	88 (55)	8 (21)		75	69 (38)	6 (15)	
T2	22	16 (10)	6 (15)		83	67 (37)	16 (40)	
T3	26	17 (10)	9 (23)		54	38 (21)	16 (40)	
T4	56	40 (25)	16 (41)		8	6 (3)	2 (5)	
**LN metastasis-no. (%)**				0.011				0.220
Positive	85	61 (38)	24 (61)		115	90 (50)	25 (63)	
Negative	115	100 (62)	15 (39)		104	89 (50)	15 (37)	
**Lymphatic invasion-no. (%)**				0.010				<0.0001
Present	123	92 (57)	31 (79)		186	31 (17)	37 (92)	
Absent	77	69 (43)	8 (21)		34	149 (83)	3 (8)	
**Venous invasion-no. (%)**				0.001				0.015
Present	117	85 (53)	32 (82)		164	128 (71)	36 (90)	
Absent	83	76 (47)	7 (18)		56	52 (29)	4 (10)	
**Mismatch repair (MMR)-no. (%)**				0.255				
deficient MMR	12	8 (5)	4 (10)					
proficient MMR	188	153 (95)	35 (90)					
**Epstein-Barr virus (EBV)-no. (%)**				0.028				
Positive	4	0	4 (25)					
Negative	33	21 (100)	12 (75)					

P value from Mann Whitney test or Fisher’s exact test. MMR and EBV status have not evaluated in the validation cohort.

### Prognostic analysis

**Figure 2:**
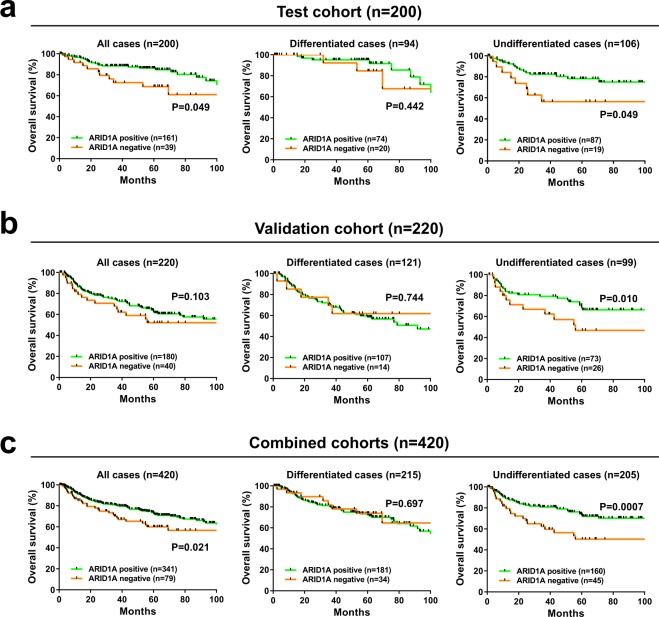
Kaplan-Meier curves showing overall survival of patients with gastric cancer according to ARID1A expression. Kaplan-Meier survival analysis of all cases, differentiated cases, and undifferentiated cases in the test (**a**), validation (**b**), and combined cohorts (**c**).

The prognostic value of ARID1A expression in gastric cancer was investigated next. Overall survival was used as an endpoint for both cohorts. Negative expression of ARID1A was associated with shorter overall survival in the test cohort (P = 0.049) (Fig. 2a). To explore the therapeutic potential of ARID1A, stratified analysis was performed according to histological subtype. Stratification by tumor differentiation showed that ARID1A negativity had no impact on prognosis in differentiated cases in the test cohort (P = 0.442). By contrast, negative expression of ARID1A was significantly associated with shorter overall survival in undifferentiated cases in the test cohort (P = 0.049). To validate these findings, the prognostic value of ARID1A expression was investigated in an independent validation cohort (Fig. 2b). Consistently, negative expression of ARID1A was significantly associated with shorter overall survival in undifferentiated cases in the validation cohort (P = 0.010). To improve the statistical power, the two cohorts were combined (combined cohorts, n = 420), and the results showed that negative expression of ARID1A was significantly associated with shorter overall survival (P = 0.021) especially in undifferentiated cases (P = 0.0007) (Fig. 2c). The Cox proportional hazard model revealed that the prognostic relevance of ARID1A in undifferentiated cases was independent from other clinical characteristics (Table 2). These results were validated by Kaplan-Meier plotter database analysis, which showed that low expression of ARID1A was significantly associated with shorter overall survival in undifferentiated gastric cancer (P = 0.039) (Supplementary Fig. S3).

**Table 2:** Univariate and multivariate Cox regression analysis of ARID1A expression and other clinical covariations.

	Combined cohorts	
	Undifferentiated cases (n = 205)	
	Overall survival	
	HR (95% CI)	P value
**Univariate analysis**		
ARID1A (negative vs. positive)	2.39 (1.40–3.99)	0.0018
Sex (F vs. M)	0.79 (0.45–1.33)	0.374
Age (60≥ vs. <60)	1.66 (0.98–2.95)	0.062
Stage (III, IV vs. I, II)	7.22 (4.17–12.98)	<0.0001
**Multivariate analysis**		
ARID1A (negative vs. positive)	2.01 (1.21–3.42)	0.015
Age (60≥ vs. <60)	1.58 (0.92–2.81)	0.096
Stage (III, IV vs. I, II)	6.65 (3.76–12.19)	<0.0001

**Figure 3:**
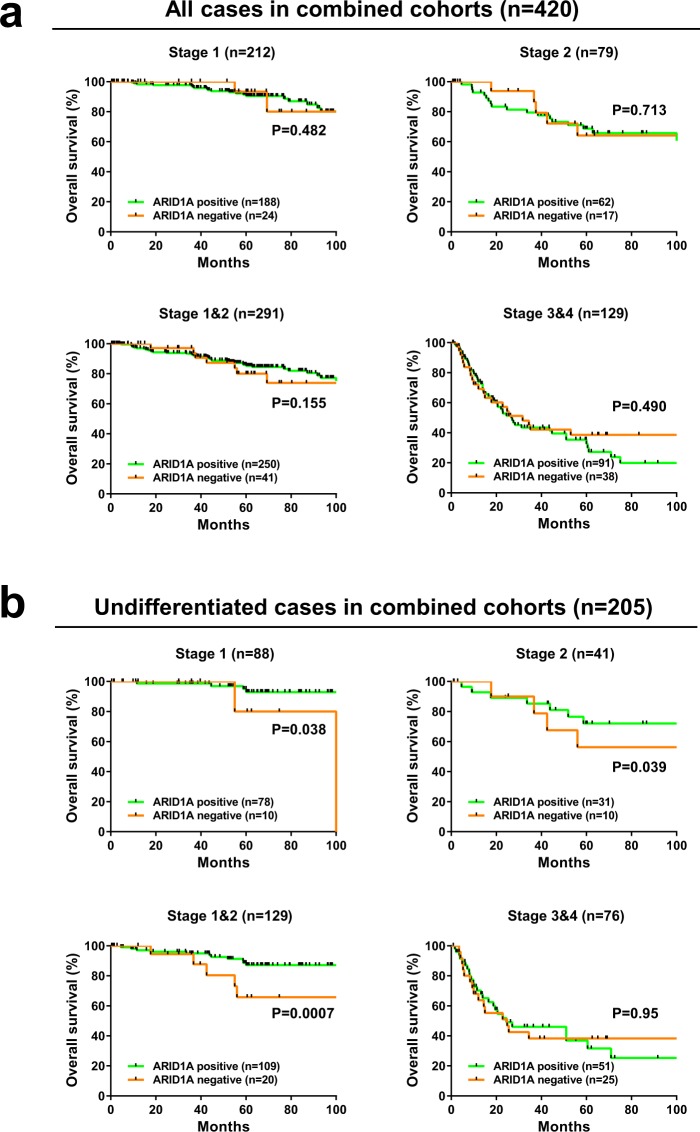
Kaplan-Meier curves showing overall survival of patients with gastric cancer according to ARID1A expression. (**a**) Kaplan-Meier curves showing overall survival of the patients with gastric cancer according to ARID1A expression. Kaplan-Meier survival analysis of all cases in the combined cohort (n = 420) and of TNM stage 1 (n = 212), stage 2 (n = 79), stage 1&2 (n = 291), and stage 3&4 (n = 129) cases stratified according to ARID1A expression. (**b**) Kaplan-Meier curves showing overall survival of patients with gastric cancer according to ARID1A expression. Kaplan-Meier survival analysis of undifferentiated cases in the combined cohorts (n = 205) and of TNM stage 1 (n = 88), stage 2 (n = 41), stage 1&2 (n = 129), and stage 3&4 (n = 76) cases stratified according to ARID1A expression.

Next, we investigated whether ARID1A expression has prognostic value in early-stage gastric cancer. In the combined cohorts, ARID1A had no prognostic value in stage 1, stage 2, or stage 1&2 gastric cancer (Fig. 3a). However, the prognostic value of ARID1A was confirmed in undifferentiated cases, especially in early-stage patients. Negative expression of ARID1A was significantly associated with shorter overall survival in stage 1 (P = 0.038), stage 2 (P = 0.039), or stage 1&2 cases (P = 0.0007) (Fig. 3b). The data indicate that ARID1A expression may serve as a prognostic biomarker to identify high-risk gastric cancer patients, especially in early-stage undifferentiated cases.

### IHC staining for PD-L1 and p53

**Figure 4:**
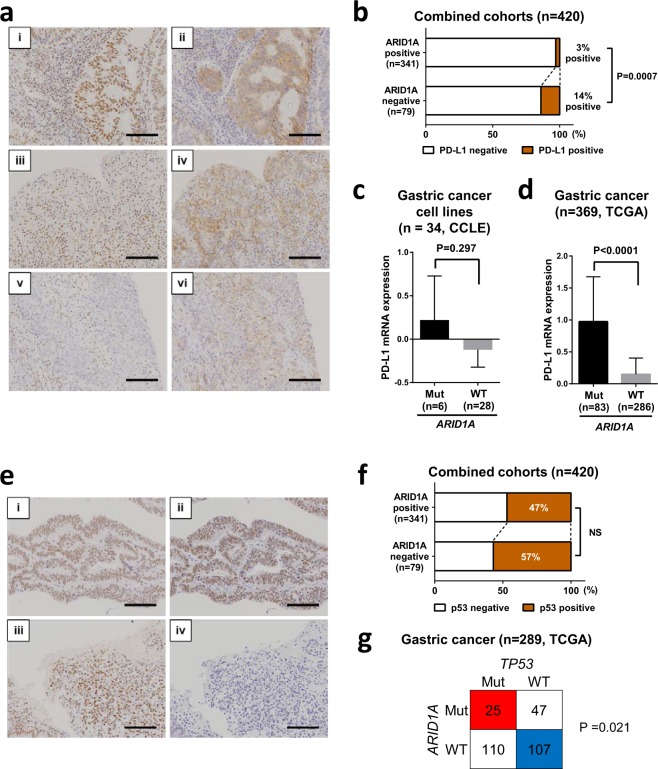
Association between expression of ARID1A and PD-L1 and p53 in gastric cancer. (**a**) Representative images showing immunohistochemical staining of gastric cancer tissue for PD-L1 (i–vi). (i, ii) A case showing positive ARID1A (i) and positive PD-L1 (ii) staining in differentiated tumor tissues. (iii, iv) A case showing positive ARID1A (iii) and positive PD-L1 (iv) staining in undifferentiated tumor tissues. (v, vi) A case showing negative ARID1A (v) and positive PD-L1 (vi) staining in undifferentiated tumor tissues. Scale bars = 100 μm. (**b**) Differences in PD-L1 expression between ARID1A-positive and -negative gastric cancer in the combined cohorts. P = 0.0007, Fisher’s exact test. (**c**) PD-L1 mRNA expression in gastric cancer cell lines with *ARID1A* truncating mutations (Mut, n = 6) and the wild-type (WT, n = 28). The average expression level of PD-L1 was higher in *ARID1A* Mut cell lines than in *ARID1A* WT cell lines. Data were obtained from the Cancer Cell Line Encyclopedia (CCLE). P = 0.297, Mann Whitney test. (**d**) PD-L1 mRNA expression in gastric cancer with *ARID1A* truncating mutations (Mut, n = 83) and wild-type *ARID1A* (WT, n = 286). The average expression level of PD-L1 was higher in *ARID1A* Mut than in *ARID1A* WT cases. Data were provided by The Cancer Genome Atlas (TCGA [Provisional]). P < 0.0001, Mann Whitney test. (**e**) Representative immunohistochemical staining for p53 in gastric cancer. Positive and negative p53 staining in tumor tissues. (i, ii) A case showing positive ARID1A (i) and positive p53 (ii) staining in differentiated tumor tissues. (iii, iv) A case showing positive ARID1A (iii) and negative p53 (iv) staining in undifferentiated tumor tissues. Scale bars = 100 μm. (**f**) Differences in p53 expression between ARID1A-positive and -negative gastric cancer in the combined cohorts. Not significant, Fisher’s exact test. (**g**) Comparison of *ARID1A* mutations and *TP53* mutations in gastric cancer in the TCGA cohort. *TP53* was less frequently mutated in gastric cancer with *ARID1A* truncating mutations. P = 0.021, Fisher’s exact test.

Overexpression of PD-L1 was identified more often in EBV-positive gastric cancer cases with frequent *ARID1A* mutations and rare *TP53* mutations^4^. Therefore, we performed IHC staining for PD-L1 and p53 in the combined cohorts and investigated the correlation with ARID1A expression. Positive staining for PD-L1 at the apical cell surface, cytoplasm, or circumference of malignant cells was observed in gastric cancer cases (Fig. 4a). In the combined cohorts, PD-L1 positivity was observed in 10 of 341 (3%) ARID1A-positive cases and in 11 of 79 (14%) ARID1A-negative cases. PD-L1 was significantly overexpressed in ARID1A-negative gastric cancer in our combined cohorts (P = 0.0007) (Fig. 4b). To confirm this result, PD-L1 expression was examined in publicly accessible datasets using cBioPortal for Cancer Genomics^10,11^. Data from the Cancer Cell Line Encyclopedia (CCLE) showed that PD-L1 mRNA expression was higher in gastric cancer cell lines with *ARID1A* truncating mutation (n = 6) than in wild-type *ARID1A* lines (n = 28) (not statistically significant, P = 0.297) (Fig. 4c)^12^. This result was confirmed using The Cancer Genome Atlas (TCGA [Provisional]) gastric cancer dataset, which showed that PD-L1 mRNA expression was higher in patients with *ARID1A* truncating mutation (n = 83) than in other patients (n = 286) (P < 0.0001) (Fig. 4d)^4^.

IHC staining for p53 was performed in the combined cohorts, and the correlation between ARID1A and p53 expression was investigated (Fig. 4e). The positive expression rate of p53 did not differ according to ARID1A expression status in gastric cancer (P = not significant [NS]) (Fig. 4f). This result suggested that IHC staining for p53 was not useful to confirm TCGA data that *TP53* mutation was less frequent in gastric cancer with *ARID1A* truncating mutations (n = 289) (Fig. 4g).

### Therapeutic utility of EZH2 inhibitors

**Figure 5:**
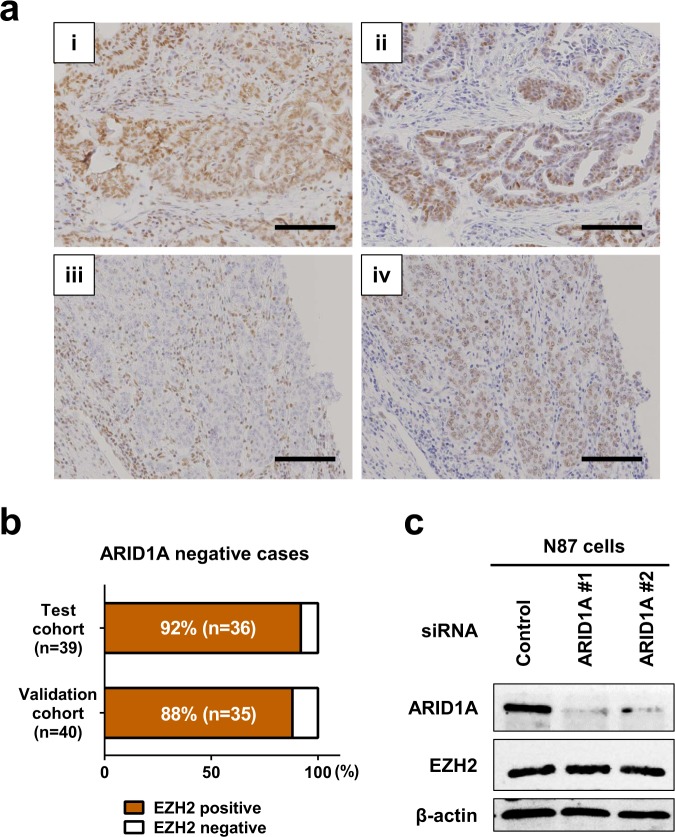
Association between EZH2 and ARID1A expression in gastric cancer. (**a**) Representative images showing immunohistochemical staining of EZH2 in gastric cancer. (i, ii) A case showing positive ARID1A (i) and positive EZH2 (ii) staining in differentiated tumor tissues. (iii, iv) A case showing negative ARID1A (iii) and positive EZH2 (iv) staining in undifferentiated tumor tissues. Scale bars = 100 μm. (**b**) EZH2 expression in ARID1A-negative gastric cancer in the test (n = 39) and validation (n = 40) cohorts. (**c**) Western blot analysis of ARID1A and EZH2 expression in N87 cells in which ARID1A was silenced using two different siRNAs (#1 and #2). ARID1A was downregulated, whereas EZH2 was positively detected. β-actin was used as a loading control. The same cells were used for the microarray analysis in Fig. 6a.

A targeted therapy for ARID1A-deficient tumors was developed based on data demonstrating the synthetic lethality of EZH2 inhibition in *ARID1A*-mutated ovarian cancer cells^9^. Because the response to EZH2 inhibitors often correlates with EZH2 overexpression, we investigated EZH2 expression by IHC staining in gastric cancer (Fig. 5a). To evaluate the therapeutic utility of EZH2 inhibitors, we focused on EZH2 expression in ARID1A-negative cases. EZH2 expression was positive in 36 of 39 ARID1A-negative cases (92%) in the test cohort and in 35 of 40 cases (88%) in the validation cohort (Fig. 5b). These results suggested that EZH2 may be expressed in most gastric cancers with low ARID1A expression.

To examine whether ARID1A deficiency leads to the dysregulation of EZH2 expression, ARID1A expression was knocked down in N87 gastric cancer cells. We confirmed that two siRNA oligonucleotides against ARID1A efficiently downregulated ARID1A expression with no effect on EZH2 expression (Fig. 5c and Supplementary Fig. S4), indicating that ARID1A expression does not directly regulate EZH2 expression.

### Identification of candidate downstream targets of ARID1A

**Figure 6:**
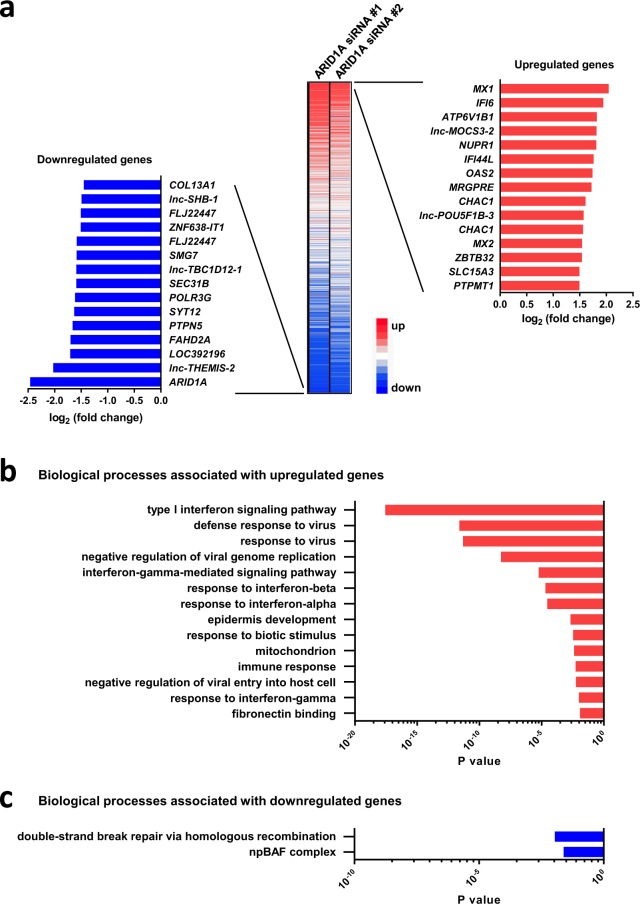
Functional annotation analysis using genes altered by ARID1A knockdown in N87 cells. (**a**) Heatmap showing gene alterations in two different ARID1A knockdown cells relative to control (scrambled siRNA) cells. Two different siRNAs (#1 and #2) were used. The 15 genes showing the greatest upregulation and downregulation are highlighted. (**b**) Gene ontology (GO) terms (biological processes) significantly enriched among upregulated genes in ARID1A knockdown cells relative to control (scrambled siRNA) cells, as determined by DAVID functional annotation analysis. (**c**) GO terms significantly enriched among downregulated genes in ARID1A knockdown cells relative to control (scrambled siRNA) cells, as determined by DAVID functional annotation analysis.

To identify genes regulated by ARID1A in gastric cancer, microarray profiling was performed using RNA isolated from N87 gastric cancer cells treated with two siRNAs against ARID1A (Fig. 5c). The results showed that 162 transcripts were >2-fold upregulated and 142 transcripts were >2-fold downregulated in ARID1A knockdown cells compared with control cells (Fig. 6a and Supplementary Table S1). The genes most significantly upregulated or downregulated by ARID1A knockdown are listed in Fig. 6a. Candidate genes showing a direct link to actionable targets were not identified among upregulated genes (e.g., *MX1*, *IFI6*, *ATP6V1B1*, *Inc-MOCS3-2*, and *NUPR1*). *ARID1A* showed the greatest downregulation, confirming the validity of the experimental system.

The groups of genes showing >2-fold upregulation or downregulation were subjected to Database for Annotation, Visualization and Integrated Discovery (DAVID) gene annotation enrichment analysis to determine significantly enriched gene ontology (GO) terms (Fig. 6b,c and Supplementary Tables S2 and S3). Genes upregulated by ARID1A knockdown were significantly enriched in type 1 interferon signaling pathways [interferon alpha (INF-α) and beta (INF-β)] and virus-related pathways. The *in vitro* experiment suggested that INF-α and INF-β were induced by ARID1A knockdown in gastric cancer cells.

## Discussion

In the present study, we evaluated ARID1A expression by IHC staining and determined the prognostic significance of negative ARID1A expression. Because most *ARID1A* mutations are inactivating mutations leading to decreased ARID1A expression, negative ARID1A expression can be used as a surrogate marker for *ARID1A* mutations (Supplementary Fig. S5)^5^. Here, we found that negative ARID1A expression was associated with poor overall survival of patients with gastric cancer, especially in early-stage undifferentiated cases. Consistent with our result, a systematic meta-analysis of 14 studies demonstrated that negative ARID1A expression predicts poor overall survival in gastric cancer^7^. Further subgroup analysis supported our findings that the prognostic role of ARID1A is significant in Asian populations, and in subgroups with a high proportion of proximal disease, positive EBV infection, and differentiation grade >3^7^. The present results that negative ARID1A expression is associated with lymphatic and venous invasion confirmed the pivotal role of ARID1A as a predictive and prognostic biomarker.

Gastric cancer is a heterogeneous disease associated with complex aberrations of oncogenic pathways^13^. The most well known risk factor for gastric cancer is infection with helicobacter pylori (H. pylori)^14^. ARID1A plays an important role in development of this inflammation-related type of carcinogenesis in that the NF-κB/miR-223-3p/ARID1A axis promotes cell proliferation and migration, leading development of H. pylori-induced gastric cancer^15^. Furthermore, *ARID1A* mutations are highly prevalent in EBV-positive gastric cancer, which is characterized by frequent overexpression of PD-L1/L2 and rare *TP53* mutations^2–4^. A recent report demonstrates that ARID1A deficiency contributes to impaired MMR, thereby increasing the tumor mutation burden^6^. Consistent with previous reports, we showed that PD-L1 is overexpressed in gastric cancer lacking ARID1A expression^2–4^. Therefore, we confirmed that the cohorts used in the present study were suitable for analysis, and that gastric cancer lacking ARID1A expression may be sensitive to PD-1-PD-L1 immune checkpoint therapies^6^.

In addition to immunotherapy, targeted therapies against *ARID1A* mutations could provide significant therapeutic benefits in cancer because *ARID1A* is one of the most frequently mutated genes in various types of cancer^16^. Most *ARID1A* mutations are inactivating mutations, indicating that *ARID1A* itself is a poor therapeutic target^4^. Novel synthetic lethal approaches to the treatment of *ARID1A*-mutated tumors were recently proposed using EZH2 inhibitors, PARP inhibitors, or ATR inhibitors^9,17,18^. For example, EZH2 inhibitors are selective against *ARID1A* inactivation, and the response to EZH2 inhibitors often correlates with gain-of-function mutations in EZH2^9^. In fact, a previous report demonstrated that EZH2 was overexpressed in many solid cancers, suggesting the positive therapeutic efficacy of EZH2 inhibitors for cancers, including gastric cancer^19^.

In the present study, comprehensive gene analysis was performed to identify potential candidate targets or pathways underlying ARID1A deficiency. Despite the identification of several genes induced or suppressed by ARID1A silencing, a direct actionable target was not determined. These results suggest that synthetic lethal targeted therapy is a valid approach to the treatment of *ARID1A*-mutated cancer. Gene enrichment analysis based on microarray results revealed that genes upregulated by *ARID1A* knockdown were significantly enriched in type 1 interferon signaling and virus-related pathways. INF-α and INF-β are induced by EBV infection, contributing to the cellular resistance against virus infection^20–22^. *ARID1A* is highly mutated in EBV-positive gastric cancer, indicating that INF-α and INF-β may be induced by EBV infection as well as by *ARID1A* inactivation itself in *ARID1A*-mutated gastric cancer.

The study has several limitations. First, our study included small number of patients to perform stratified analysis of stage and histological differentiation. Second, we evaluated MMR and EBV status only in the test cohort and found small number of dMMR cases. Third, we evaluated PD-L1 expression in tumor cells rather than in the tumor tissue as a whole and in immune cells. Also, we did not use a combined positive score scoring method that is reproducible, such as that used in the KEYNOTE-059 and KEYNOTE-061 trials^23–25^. Fourth, our experimental analysis was performed using only the N87 gastric cancer cell line in which carcinogenesis is driven by overexpression of HER2. Therefore, the results may apply only to HER2-positive gastric cancers.

In conclusion, the current study shows that ARID1A expression serves as a prognostic biomarker for gastric cancer. The present results warrant further study of the value of ARID1A in gastric cancer.

## Materials and Methods

### Clinical samples of patients

The study included 420 surgical specimens collected from gastric cancer patients who underwent surgical resection at Fukushima Medical University Hospital between January 1991 and December 2014. The test cohort consisted of 200 patients recruited between 2002 and 2014, and the validation cohort consisted of 220 patients recruited between 1991 and 2001. Data regarding age, sex, TNM stage (the 7th classification), and pathological diagnosis, including lymphatic and venous invasion, were retrospectively collected. The carcinomas at the time of primary tumor resection were staged according to the Union for International Cancer Control classification.

The study was approved by the ethics committee of Fukushima Medical University. All patients provided written informed consent. All experiments were carried out in accordance with the approved study plan and relevant guidelines.

### IHC and ISH staining and evaluation

IHC staining was performed using paraffin-embedded histological sections (4 μm thick) using a polymer peroxidase method. Briefly, after deparaffinization and rehydration, the sections were treated with 0.3% hydrogen peroxide in methanol for 30 min to block endogenous peroxidase activity. After rinsing in PBS, the sections were incubated with anti-ARID1A antibody (#12354; D2A8U; 1:500 dilution; Cell Signaling Technology, Danvers, MA, USA), anti-EZH2 antibody (#5246; D2C9; 1:50; Cell Signaling), anti-PD-L1 antibody (#13684; E1L3N; 1:200; Cell Signaling), and anti-p53 antibody (DO-7; 1:100; Dako; Agilent Technologies, Inc., Santa Clara, CA, USA) at 4 °C overnight. An additional wash in PBS was followed by treatment with a peroxidase-labeled polymer conjugated to goat anti-rabbit immunoglobulins (ENvision + kit; Dako) as the secondary antibody for 30 min at room temperature. The staining was visualized with diaminobenzidine, followed by counterstaining with hematoxylin. Protein expression was considered positive when the nucleus of the cancerous tissue and the total field of view were observed at 400× magnification. Loss of ARID1A protein was defined as the absence of nuclear staining in tumor cells in the presence of positive nuclear staining in stroma cells. The case with weak or partial positive staining for ARID1A did not define as positive staining case. Specimen staining was evaluated by investigators blinded to the origin of samples and clinical outcomes. Stained cancer cells were counted per 1000 cancer cells in the maximum field of cancer tissue by two investigators. Staining rates were classified as follows: 0%, 0; 1–10%, 1; and 11–100%, 2. The staining intensity was scored as 0 (negative), 1 (weak), and 2 (strong). The results were expressed as a product of the positivity score and staining intensity score. Positive staining was defined by a score of 2, whereas negative staining was scored at 0 or 1. Evaluation of PD-L1 expression was performed as previously described^26^. Positive expression of PD-L1 protein was defined as positive staining in gastric cancer cells.

IHC staining for MMR proteins was performed for all the cases in the test cohort, as previously described^27^ using primary antibodies specific for MLH1 (ES05, 1:50, Dako), MSH2 (FE11, 1:50, Dako), MSH6 (EP49, 1:200, Dako), and PMS2 (EP51, 1:50, Dako). Loss of a MMR protein was defined as absence of nuclear staining in tumor cells in the presence of positive nuclear staining in normal epithelial cells and lymphocytes. Tumors showing loss of at least one MMR protein were collectively designated as dMMR, and tumors with intact MMR protein expression were designated as pMMR.

Integration of EBV was assessed by *in situ* hybridization (ISH) for the case with increased lymphocyte infiltration (n = 37) in the test cohort, as previously described^28^. EBV-encoded small RNA (EBER)-ISH was performed using the INFORM EBER probe (800–2842, Ventana Medical Systems, Tucson, AZ, USA) and the Discovery XT autostainer (Ventana), according to the manufacturer’s protocol.

### Small interfering RNA (siRNA) transfection

Knockdown experiments were performed using small interfering RNA (siRNA) oligos for ARID1A (s15784 and s15785; Thermo Fisher Scientific Inc., Waltham, MA, USA) and included two target-specific siRNAs and a control siRNA (negative control #1; Thermo Fisher Scientific) according to the manufacturer’s protocol. The gastric cancer cell line NCI-N87 (N87) used in the present study was originally obtained from the American Type Culture Collection (Manassas, VA, USA). Cells were cultured in RPMI-1640 medium (Sigma-Aldrich; Merck KGaA, Darmstadt, Germany). The monolayer cells were maintained in a 37 °C incubator with 5% CO_2_ and observed regularly under a light microscope (magnification, ×40); cells were subcultured when they reached 80–90% confluency. During the exponential growth phase, cells were transiently transfected with siRNA at a final concentration of 5 nM using Lipofectamine RNAiMAX (Thermo Fisher Scientific) according to the manufacturer’s protocol. After 48 h of incubation, cells were used for each experiment.

### Western blotting

Western blot analysis was performed as previously described^29^. N87 cells were washed twice in ice-cold PBS, pelleted by centrifugation (1500 rpm for 5 min) and stored at −80 °C. The pellet was resuspended in radioimmunoprecipitation assay buffer (Thermo Fisher Scientific) with a Halt Protease Inhibitor Single-Use Cocktail (100×; Thermo Fisher Scientific), and centrifuged at 4 °C and 15,000 rpm for 20 min. Total protein concentration was measured using the Bradford method (Bio-Rad Laboratories Inc., Hercules, CA, USA) and a Smart Spec 3000 spectrometer (Bio-Rad Laboratories). Tris-Glycine SDS sample buffer (Thermo Fisher Scientific) and 3-Mercapto-1,2-propandiol (Wako Pure Chemical Industries, Ltd., Osaka, Japan) were added to the total protein samples and heated at 100 °C for 3 min. The 4–20% Tris-Glycine gels (Thermo Fisher Scientific) were loaded with 20 µg of protein samples and electrophoresed at 125 V for 50 min using Tris-Glycine SDS Running buffer in an Invitrogen™ XCell SureLock™ electrophoresis system (Thermo Fisher Scientific). Then, proteins were transferred onto PVDF membranes using the X Cell II™ Blot Module (Thermo Fisher Scientific) at 50 V overnight. The PVDF membranes were then blocked with 5% non-fat skimmed milk. The protein blots were incubated with anti-ARID1A antibody (dilution, 1:1000; catalog no., #12354; Cell Signaling Technology) and anti-EZH2 antibody (dilution, 1:1000; catalog no., #5246; Cell Signaling Technology) for 1 h at room temperature and incubated with goat anti-rabbit HRP secondary antibody (Santa Cruz Biotechnology) for 1 h at room temperature. The protein levels were quantified using primary mouse anti-β-actin antibody (dilution, 1:2000; catalog no., #sc-69789; Santa Cruz Biotechnology) as the internal loading control for 1 h at room temperature. Bound antibodies were detected by SuperSignal West Pico Chemiluminescent Substrate (Thermo Fisher Scientific) and visualized by autoradiography (ImageQuant™ LAS 4000 IR MultiColor imager; Fujifilm Corporation, Tokyo, Japan).

### Analysis of human gastric cancer cell lines and gastric cancer datasets

Publicly accessible datasets of gene expression and mutations were downloaded from the cBioPortal for Cancer Genomics database (http://www.cbioportal.org/). The expression data of PD-L1 and mutation data of *ARID1A* were extracted from CCLE and TCGA provisional data.

### Microarray analysis

Total RNA was isolated from N87 cells using TRIzol (Thermo Fisher Scientific) according to the manufacturer’s protocol. Microarray analysis was performed using the Agilent platform (SuperPrint G3 Human GE Ver 3.0 [Design ID: 72363]) to investigate gene alterations. Data were normalized and analyzed using GeneSpring software (Agilent). The fold change in expression was defined as the ratio of expression in ARID1A knockdown cells to that in control (scrambled siRNA) cells. The raw microarray data are deposited in the Gene Expression Omnibus at the National Center for Biotechnology Information (GSE118273).

### GO analysis

Functional enrichment analysis of genes upregulated or downregulated in ARID1A knockdown cells relative to control cells was performed using DAVID (https://david.ncifcrf.gov/) as previously described^30^.

### Kaplan-Meier plotter database analysis

Kaplan-Meier analysis was performed using publicly available data and the Kaplan-Meier plotter database (http://kmplot.com/analysis/).

### Statistical analysis

The Mann Whitney test and Fisher’s exact test were performed using GraphPad Prism 6 software (GraphPad Software, Inc., San Diego, CA, USA). Survival rate curves were generated using the Kaplan-Meier method and compared by the log-rank test. Logistic regression analysis was performed by JMP software (SAS Institute Inc., Cary, NC, USA). A P value of <0.05 was considered statistically significant.

## Supplementary information

Supplementary Information
