# Supplementary material for: Prognostic role of ARID1A negative expression in gastric cancer
Source: Sci Rep. 2019 May 1;9:6769. doi: 10.1038/s41598-019-43293-5 (PMC6494900; doi:10.1038/s41598-019-43293-5)
Supplement: Supplementary file 1 — Supplementary Information [file 41598_2019_43293_MOESM1_ESM.pdf]

Prognostic role of ARID1A negative expression in gastric cancer

Mai Ashizawa[1], Motonobu Saito[1*], Aung Kyi Thar Min[1], Daisuke Ujiie[1], Katsuharu Saito[1], Takahiro Sato[1], Tomohiro Kikuchi[1], Hirokazu Okayama[1], Shotaro Fujita[1], Hisahito Endo[1], Wataru Sakamoto[1], Tomoyuki Momma[1], Shinji Ohki[1], Akiteru Goto[2] & Koji Kono[1]

[1]Department of Gastrointestinal Tract Surgery, Fukushima Medical University School of Medicine, Fukushima 960-1295, Japan
[2]Department of Cellular and Organ Pathology, Graduate School of Medicine, Akita University, Akita 010-8543, Japan

*Correspondence to: Motonobu Saito
Department of Gastrointestinal Tract Surgery, Fukushima Medical University School of Medicine, 1 Hikarigaoka, Fukushima 960-1295, Japan
E-mail: moto@fmu.ac.jp

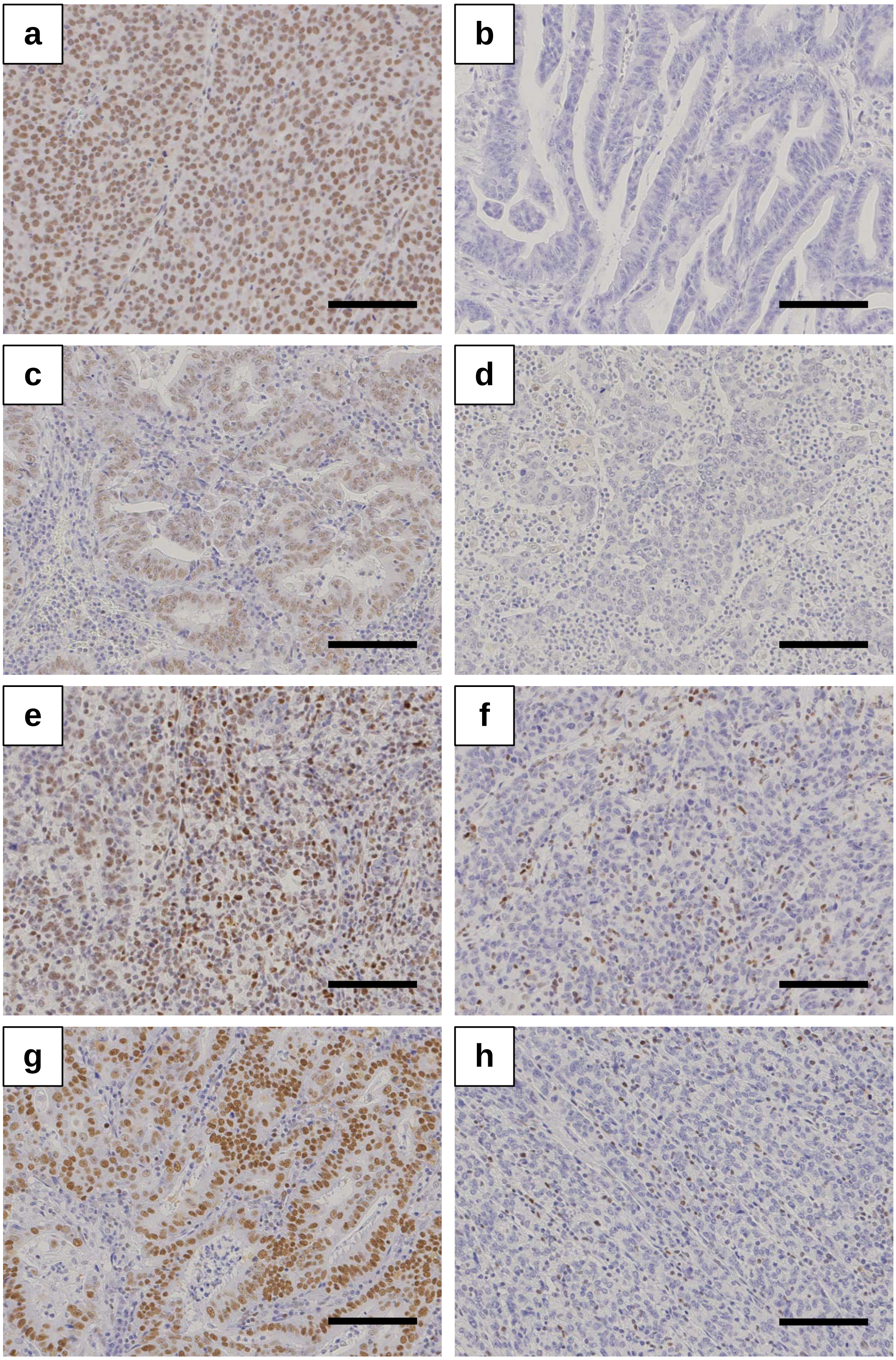

Sup Figure S1. Representative in situ hybridization for Epstein-Barr virus in gastric cancer (a-h). Positive (a) and negative (b) staining for MLH1 staining. Positive (c) and negative (d) staining for PMS2 staining. Positive (e) and negative (f) staining for MSH2 staining. Positive (g) and negative (h) staining for MSH6 staining. Scale bars = 100 μm.

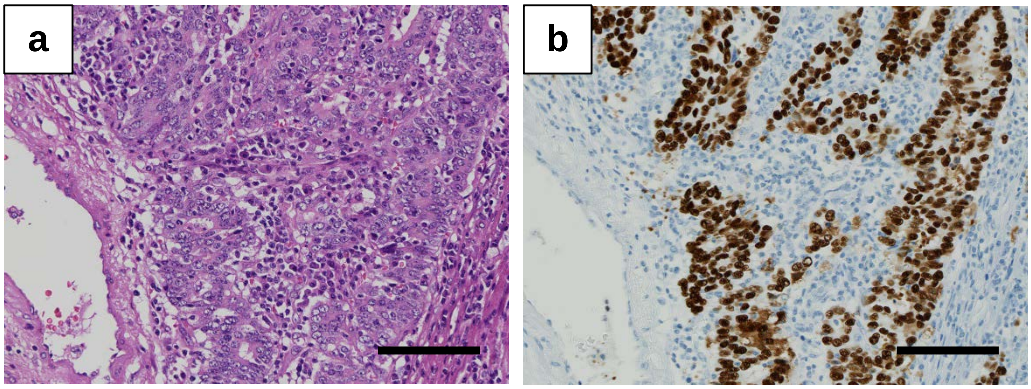

Sup Figure S2. Representative in situ hybridization for Epstein-Barr virus. (a, b) A case with positive EBV (a) and HE staining (b). Scale bars = 100 μm.

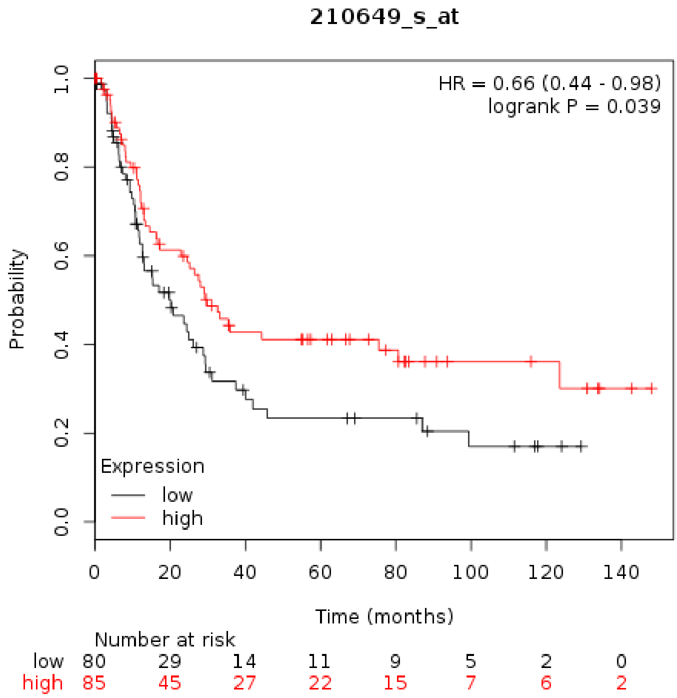

Sup Figure S3. Kaplan-Meier overall survival curves for the patients with poorly differentiated stomach cancer (n = 165) according to ARID1A expression.

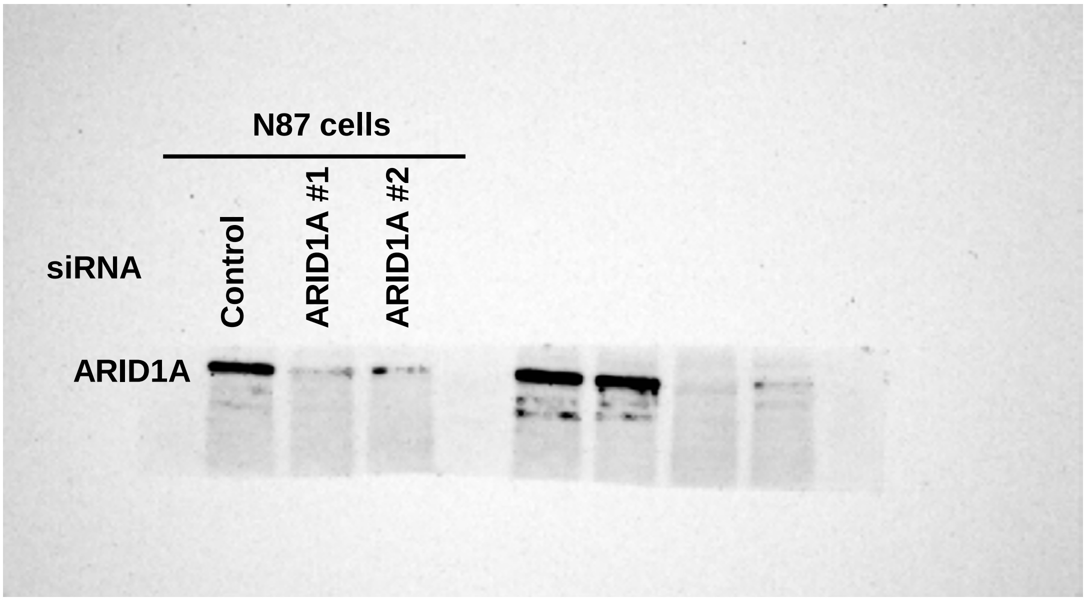

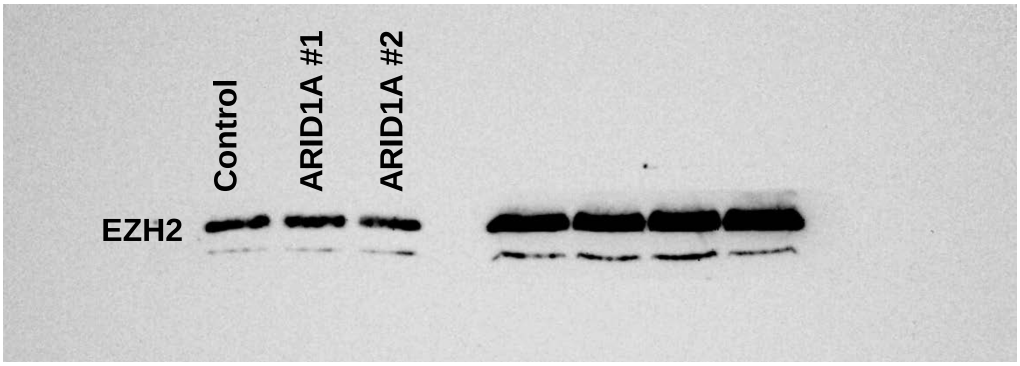

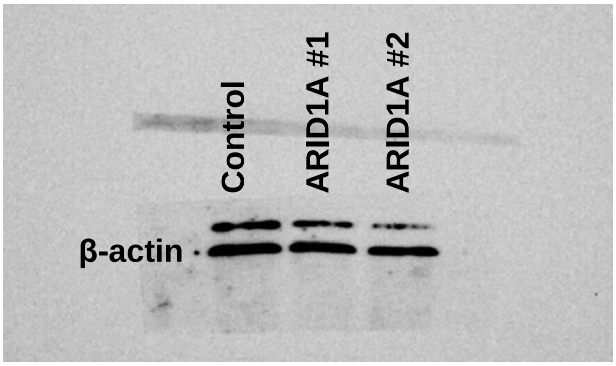

Sup Figure S4. Uncropped blots image of Figure 5c (ARID1A, EZH2, and β-actin).

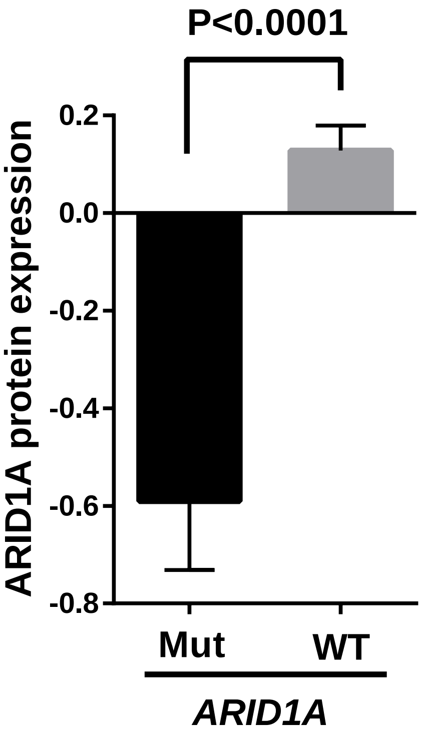

Sup Figure S5. The level of ARID1A protein expressions in stomach cancer cases between *ARID1A* truncating mutations (Mut) and wild-type (WT). Data was provided by The Cancer Genome Atlas (TCGA [Provisional]). P < 0.0001, Mann Whitney test.

Supplementary Table S1. Differentially expressed genes in ARID1A knockdown cells

	Annotation	Normalized Data			Log2 Fold Change			
ProbeName	GeneSymbol	siRNA_control	siRNA_ARID1A#1	siRNA_ARID1A#2	ARID1A#1/Ctrl	ARID1A#2/Ctrl	Average	Regulation
A_23_P17663	MX1	5.019107	6.6971717	7.4307632	1.6780648	2.4116564	2.0448606	up
A_23_P201459	IFI6	4.0532007	5.7906566	6.202137	1.7374558	2.1489363	1.9431961	up
A_23_P142815	ATP6V1B1	-2.799703	-0.677304	-1.281353	2.1223984	1.5183501	1.8203743	up
A_22_P00010051	lnc-MOCS3-2	-2.956832	-0.106299	-2.175813	2.8505335	0.7810199	1.8157767	up
A_24_P270728	NUPR1	0.9810433	3.9248538	1.6584463	2.9438105	0.677403	1.8106068	up
A_23_P45871	IFI44L	1.1368866	2.4772038	3.31804	1.3403172	2.1811533	1.7607353	up
A_23_P204087	OAS2	0.4275131	1.6901956	2.6412716	1.2626824	2.2137585	1.7382205	up
A_24_P315638	MRGPRE	-2.726953	-0.012427	-2.003676	2.7145257	0.7232766	1.7189012	up
A_33_P3376971	CHAC1	2.1827888	4.858581	2.7213125	2.6757922	0.5385237	1.607158	up
A_22_P00012196	lnc-POU5F1B-3	-3.362089	-1.800794	-1.778918	1.5612953	1.5831711	1.5722332	up
A_33_P3376965	CHAC1	-1.818121	0.8286676	-1.343502	2.646789	0.4746194	1.5607042	up
A_23_P6263	MX2	1.8837938	2.9095483	3.94306	1.0257545	2.059266	1.5425103	up
A_23_P131024	ZBTB32	-2.605985	-1.154247	-0.976375	1.4517379	1.6296096	1.5406738	up
A_23_P75786	SLC15A3	-2.536574	-1.633421	-0.451613	0.9031529	2.084961	1.494057	up
A_24_P372562	PTPMT1	3.3888874	4.8976884	4.8668957	1.508801	1.4780083	1.4934047	up
A_23_P129577	TIGD7	-2.244747	-2.437485	0.927484	-0.192738	3.1722307	1.4897464	up
A_33_P3225512	OAS2	0.1125941	0.9959412	2.2062826	0.883347	2.0936885	1.4885178	up
A_32_P27917	KIF26A	-1.383731	0.6023502	-0.40549	1.9860816	0.9782414	1.4821615	up
A_24_P378019	IRF7	3.609726	4.907458	5.2678585	1.2977319	1.6581326	1.4779323	up
A_23_P390621	PACRGL	-3.207687	-0.826482	-2.650415	2.3812044	0.5572722	1.4692383	up
A_33_P3708413	MFAP5	2.3437662	3.8050628	3.7921743	1.4612966	1.4484081	1.4548524	up
A_23_P166797	RTP4	1.1362567	2.3205132	2.8519783	1.1842566	1.7157216	1.4499891	up
A_23_P52266	IFIT1	4.1169767	4.989264	6.1221266	0.8722873	2.0051498	1.4387186	up
A_23_P76743	ASPG	-0.052836	1.792666	0.9768891	1.8455024	1.0297256	1.437614	up
A_33_P3336686	CLIC3	5.6882944	7.384764	6.8606443	1.6964698	1.1723499	1.4344099	up
A_19_P00315804	VLDLR-AS1	-3.023714	-0.704326	-2.502049	2.3193882	0.5216653	1.4205268	up
A_23_P97700	TXNIP	4.560961	6.328261	5.6159096	1.7673001	1.0549488	1.4111245	up
A_24_P557479	XAF1	-0.262383	0.6346135	1.6589117	0.8969965	1.9212947	1.4091456	up
A_23_P57417	MMP11	-3.391971	-1.989587	-1.979502	1.402384	1.4124691	1.4074266	up
A_23_P2674	KRT4	2.6827183	4.147838	4.0240755	1.4651198	1.3413572	1.4032385	up
A_22_P00018971	lnc-NSMCE1-3	-2.783685	-0.809969	-1.956346	1.9737165	0.8273399	1.4005282	up
A_23_P165989	NEURL2	-2.825564	-1.111486	-1.751953	1.7140789	1.0736113	1.3938451	up
A_33_P3839897	RNU4ATAC	-2.138084	-0.283255	-1.211715	1.8548288	0.9263687	1.3905988	up
A_23_P62890	GBP1	-1.451954	0.7422595	-0.875968	2.1942134	0.5759854	1.3850994	up
A_21_P0007114	lnc-FGF8-1	-3.366611	-1.424035	-2.54689	1.9425757	0.819721	1.3811484	up
A_23_P207174	CSH2	-2.983034	-1.274379	-1.945364	1.7086554	1.0376701	1.3731628	up
A_23_P321892	HRG	-3.445	-1.911286	-2.232426	1.5337133	1.2125733	1.3731433	up
A_33_P3400708	BNIPL	-0.426342	1.325952	0.5514379	1.7522936	0.9777794	1.3650365	up
A_32_P227605	RGS9BP	-2.817491	-1.570913	-1.339461	1.2465775	1.4780295	1.3623035	up
A_24_P185117	RILP	-2.229504	-0.883167	-0.869506	1.3463368	1.3599977	1.3531673	up
A_23_P38346	DHX58	1.1446753	2.3590217	2.6223316	1.2143464	1.4776564	1.3460014	up
A_22_P00010750	YTHDF3	-3.112848	-0.828337	-2.718936	2.2845104	0.3939111	1.3392108	up
A_33_P3223522	lnc-SLC25A48-2	-2.54185	-1.454666	-0.954909	1.087184	1.5869408	1.3370624	up
A_23_P370682	BATF2	0.1557813	1.0254302	1.9594021	0.8696489	1.8036208	1.3366349	up
A_33_P3394868	SMIM1	-2.488437	-1.009563	-1.294155	1.478874	1.1942823	1.3365782	up
A_33_P3401826	CMPK2	1.0403266	1.7080727	3.0213404	0.6677461	1.9810138	1.3243799	up
A_22_P00009995	lnc-MLLT4-1	-3.483488	-2.432718	-1.898673	1.0507708	1.5848157	1.3177933	up
A_23_P54918	LDHD	-1.490813	0.2264442	-0.575894	1.7172575	0.9149189	1.3160882	up
A_24_P287043	IFITM2	6.0135393	7.1829143	7.4730654	1.169375	1.4595261	1.3144506	up
A_33_P3377399	lnc-RP11-778D12.2.1-4	-1.469675	-0.002258	-0.319284	1.4674168	1.1503916	1.3089042	up
A_24_P16124	IFITM4P	5.3830967	6.484549	6.888875	1.1014524	1.5057783	1.3036154	up

A_22_P00007720	HLA-DQB1	-2.571675	-0.973018	-1.564447	1.5986576	1.0072284	1.302943	up
A_21_P0002259	lnc-NPAS2-1	-1.993421	-1.284592	-0.098893	0.7088285	1.8945279	1.3016782	up
A_23_P72737	IFITM1	6.819721	7.8814263	8.359805	1.0617051	1.5400839	1.3008945	up
A_24_P28722	RSAD2	1.3025436	2.5285754	2.644207	1.2260318	1.3416634	1.2838476	up
A_23_P211631	FBLN1	-2.710022	-0.770927	-2.096564	1.9390948	0.6134584	1.2762766	up
A_23_P128817	PCK2	-0.716259	1.3732562	-0.266525	2.0895147	0.4497333	1.269624	up
A_23_P105012	HRASLS2	1.2252188	2.5719485	2.411353	1.3467298	1.1861343	1.2664321	up
A_24_P165205	MORN1	-3.642553	-2.383164	-2.377177	1.2593889	1.2653759	1.2623824	up
A_23_P351837	KLHL35	-0.397803	1.1652513	0.5638461	1.5630541	0.9616489	1.2623515	up
A_23_P119196	KLF2	0.0332565	1.0770626	1.5036864	1.0438061	1.4704299	1.257118	up
A_23_P26024	C15orf48	3.5036812	4.8426995	4.6778965	1.3390183	1.1742153	1.2566168	up
A_23_P104318	DDIT4	4.038847	6.156365	4.433094	2.117518	0.3942471	1.2558825	up
A_23_P215634	IGFBP3	0.1327457	1.4851294	1.2813802	1.3523836	1.1486344	1.250509	up
A_33_P3423941	IFITM1	5.5843	6.553148	7.1150637	0.9688478	1.5307636	1.2498057	up
A_33_P3241428	OBSL1	-3.40593	-1.789254	-2.533945	1.6166763	0.8719847	1.2443305	up
A_23_P426944	PAX9	-2.95333	-1.659133	-1.762736	1.2941973	1.190594	1.2423957	up
A_33_P3362063	LINC01124	-2.292203	-0.99824	-1.11238	1.293963	1.1798234	1.2368932	up
A_23_P218047	KRT5	2.395728	4.0026298	3.2423935	1.6069016	0.8466654	1.2267835	up
A_24_P691826	WFDC21P	2.334587	3.4852986	3.6146116	1.1507115	1.2800245	1.215368	up
A_24_P94402	MYCN	-0.052958	1.1873116	1.1367745	1.2402701	1.189733	1.2150016	up
A_23_P201035	GBA	1.5093455	2.8118825	2.6243267	1.302537	1.1149812	1.2087591	up
A_21_P0014021	TTLL8	-2.596482	-1.023906	-1.75581	1.572576	0.8406715	1.2066238	up
A_23_P218997	PDCD6	6.1782494	7.3521886	7.4151583	1.1739392	1.2369089	1.2054241	up
A_23_P819	ISG15	7.3494043	8.291859	8.809743	0.9424543	1.4603386	1.2013965	up
A_24_P114032	EFNA3	-0.792809	0.4538484	0.3626366	1.2466574	1.1554456	1.2010515	up
A_33_P3235147	DLX5	-3.259093	-1.663268	-2.458587	1.5958257	0.8005064	1.198166	up
A_33_P3214012	HMGCLL1	-3.246409	-0.877156	-3.231992	2.3692527	0.0144167	1.1918347	up
A_23_P380614	ATP9A	3.8429594	5.365744	4.7006702	1.5227847	0.8577108	1.1902478	up
A_33_P3390793	TRIM36	-2.17517	-1.094187	-0.888909	1.0809836	1.2862611	1.1836224	up
A_23_P79978	SLC24A3	-3.385478	-2.385088	-2.031039	1.0003896	1.3544388	1.1774142	up
A_21_P0011517	KRT14	1.5324025	3.1780095	2.2334356	1.645607	0.7010331	1.1733201	up
A_23_P128855	SLC39A2	-2.126168	-1.225308	-0.680877	0.9008598	1.4452906	1.1730752	up
A_23_P254254	SGSH	-0.303714	0.8205504	0.8965211	1.1242642	1.2002349	1.1622496	up
A_23_P47208	BANF1	2.9001894	4.0092635	4.115594	1.1090741	1.2154045	1.1622393	up
A_33_P3319886	C19orf45	-3.072934	-1.681306	-2.152049	1.3916283	0.9208851	1.1562567	up
A_22_P00000677	lnc-ADA-1	-3.594842	-2.922015	-1.958106	0.6728263	1.6367354	1.1547809	up
A_33_P3235432	NMNAT1	-0.247253	1.0135694	0.777421	1.2608228	1.0246744	1.1427486	up
A_32_P87697	HLA-DRA	-3.247773	-2.034832	-2.17992	1.2129405	1.067853	1.1403968	up
A_33_P3275495	LOC400743	-1.644088	0.5382781	-1.549332	2.1823664	0.0947561	1.1385613	up
A_33_P3379512	ZNF333	-3.093909	-1.815613	-2.101275	1.2782965	0.9926348	1.1354657	up
A_33_P3437907	FAM171A2	-3.500207	-2.433586	-2.296782	1.0666215	1.2034249	1.1350232	up
A_23_P350551	C12orf57	5.4952707	6.659697	6.5981455	1.1644263	1.1028748	1.1336506	up
A_32_P538180	PAQR7	-2.72698	-1.628753	-1.563345	1.0982268	1.163635	1.1309309	up
A_23_P153320	ICAM1	-1.590603	-0.165743	-0.76007	1.4248605	0.8305335	1.127697	up
A_24_P110062	PTPMT1	0.9135051	1.8810611	2.2000446	0.967556	1.2865396	1.1270478	up
A_24_P91991	NAT8L	0.0338006	1.1086106	1.2128658	1.07481	1.1790652	1.1269376	up
A_19_P00320067	LOC100130417	-0.700833	0.7409387	0.1024642	1.441772	0.8032975	1.1225348	up
A_33_P3262376	OTUD7A	-2.682017	-1.212686	-1.90641	1.469331	0.7756069	1.122469	up
A_23_P150609	IGF2	-1.714591	-0.832469	-0.35459	0.8821216	1.3600011	1.1210613	up
A_23_P87545	IFITM3	7.1596127	8.114851	8.446485	0.9552383	1.2868719	1.1210551	up
A_23_P64828	OAS1	2.7208633	3.5882459	4.087165	0.8673825	1.3663015	1.116842	up
A_24_P227141	ELF5	-2.811908	-1.704335	-1.688455	1.107573	1.1234531	1.1155131	up
A_33_P3374378	GTDC1	-2.778521	-1.640956	-1.687618	1.1375642	1.0909023	1.1142333	up
A_33_P3245178	BEX2	-2.944994	-1.46144	-2.201643	1.4835539	0.743351	1.1134525	up
A_33_P3283611	IFIT3	1.9638443	2.66322	3.4910374	0.6993756	1.5271931	1.1132844	up
A_32_P512061	GBAP1	0.6615262	1.9641318	1.5771155	1.3026056	0.9155893	1.1090975	up

A_19_P00811440	lnc-KLF7-1	-3.221292	-2.4902	-1.735236	0.7310922	1.4860561	1.1085742	up
A_33_P3418175	LYNX1	-3.044308	-1.969734	-1.902998	1.0745738	1.1413095	1.1079417	up
A_23_P346093	TMC8	1.5517817	2.8910031	2.4127197	1.3392215	0.8609381	1.1000798	up
A_32_P141682	EVPLL	-3.005808	-1.676493	-2.134981	1.3293157	0.8708277	1.1000717	up
A_23_P46894	CHAT	-2.297282	-0.989512	-1.409969	1.3077698	0.8873134	1.0975416	up
A_23_P312610	DNAJC4	-2.112759	-0.794133	-1.236505	1.3186259	0.8762541	1.09744	up
A_22_P00005605	DNAJC27-AS1	-3.438201	-2.427147	-2.265436	1.0110545	1.1727655	1.09191	up
A_22_P00014201	ATP2B2-IT2	-2.978924	-1.014582	-2.766012	1.9643421	0.2129123	1.0886272	up
A_23_P43846	FABP6	-3.274521	-2.541304	-1.831424	0.733217	1.4430964	1.0881567	up
A_33_P3308862	NUTM2B	0.622386	1.7558541	1.6632295	1.1334682	1.0408435	1.0871559	up
A_23_P423695	MXD4	0.1041498	1.2591863	1.1146493	1.1550364	1.0104995	1.082768	up
A_33_P3258346	XAF1	-1.947763	-1.238732	-0.496225	0.7090306	1.4515381	1.0802844	up
A_33_P3323501	PHGR1	0.795723	1.9390063	1.8032107	1.1432834	1.0074878	1.0753856	up
A_23_P254507	HOPX	-0.526543	0.4534779	0.6390033	0.9800205	1.165546	1.0727833	up
A_23_P64058	RASGRP2	-3.158774	-1.991345	-2.1892	1.1674294	0.969574	1.0685017	up
A_24_P491397	LDLRAD1	-2.654697	-1.246141	-1.926313	1.4085555	0.7283831	1.0684693	up
A_33_P3417695	ODF3B	0.5565701	1.5134344	1.7360425	0.9568644	1.1794724	1.0681684	up
A_23_P152782	IFI35	1.9037409	2.7588573	3.1808472	0.8551164	1.2771063	1.0661113	up
A_33_P3339865	CALML5	-2.313797	-1.282959	-1.219591	1.0308375	1.0942059	1.0625217	up
A_24_P131580	ALPPL2	4.537369	6.114882	5.084279	1.5775132	0.5469103	1.0622118	up
A_23_P79108	ATP8B3	-2.099901	-0.567238	-1.50965	1.5326629	0.5902515	1.0614572	up
A_23_P55917	SYT3	-3.372303	-2.384384	-2.238011	0.9879191	1.1342924	1.0611058	up
A_19_P00323082	H19	-0.827957	0.3153868	0.1490645	1.1433439	0.9770217	1.0601828	up
A_23_P258393	PPM1M	-3.100766	-1.81131	-2.269952	1.2894561	0.8308141	1.0601351	up
A_23_P142322	CIRBP	1.5650663	2.9283414	2.3105202	1.363275	0.7454538	1.0543644	up
A_21_P0008517	LOC100506476	-2.488742	-1.378099	-1.495293	1.1106436	0.9934495	1.0520465	up
A_23_P58082	CCDC80	-1.57035	-0.136897	-0.899799	1.4334531	0.6705508	1.052002	up
A_23_P19482	DDAH2	2.6746569	3.7731547	3.6685514	1.0984979	0.9938946	1.0461963	up
A_24_P737939	LRRC73	-0.920247	0.0508537	0.2006106	0.9711008	1.1208577	1.0459793	up
A_33_P3398597	EPS8L1	0.6775999	1.8118634	1.634419	1.1342635	0.9568191	1.0455413	up
A_33_P3212754	NUTM2F	0.2085929	1.2612348	1.2454772	1.0526419	1.0368843	1.0447631	up
A_33_P3389230	CXCR3	-0.224023	0.6745133	0.9642067	0.8985362	1.1882296	1.0433829	up
A_23_P106906	PPL	2.4543085	3.7313905	3.2579784	1.277082	0.8036699	1.040376	up
A_24_P245379	SERPINB2	-1.811652	-0.910521	-0.636864	0.9011316	1.1747885	1.0379601	up
A_32_P175715	MEIG1	-2.376365	-1.296177	-1.384273	1.0801878	0.9920917	1.0361397	up
A_23_P401084	ZNF575	-1.451192	-0.459454	-0.378365	0.9917383	1.0728273	1.0322828	up
A_33_P3250887	SYCE3	-3.108312	-1.938041	-2.214581	1.1702704	0.8937311	1.0320008	up
A_23_P38537	KRT16	3.7402716	5.30076	4.242754	1.5604882	0.5024824	1.0314853	up
A_24_P117410	KLHDC7B	-3.020373	-1.669008	-2.309902	1.3513653	0.7104716	1.0309185	up
A_32_P799227	NUTM2B	-2.948385	-1.958453	-1.879154	0.9899321	1.0692306	1.0295813	up
A_33_P3218960	CACNA1H	0.8930454	2.442741	1.4001536	1.5496955	0.5071082	1.0284019	up
A_22_P00011523	lnc-PBX1-1	-1.472623	-0.368637	-0.521317	1.1039863	0.9513068	1.0276466	up
A_23_P171366	USP11	2.2726383	3.5941796	3.001357	1.3215413	0.7287188	1.02513	up
A_24_P52697	H19	0.0827327	1.2392659	0.9733195	1.1565332	0.8905869	1.02356	up
A_23_P88404	TGFB3	-3.238133	-2.115856	-2.320579	1.1222763	0.9175534	1.0199149	up
A_23_P83634	ALOX12B	-3.235538	-2.089649	-2.341979	1.1458888	0.8935583	1.0197235	up
A_33_P6822486	LOC100506844	0.0909152	1.3800273	0.8412051	1.2891121	0.7502899	1.019701	up
A_23_P77328	GCHFR	1.3289447	2.264831	2.4303503	0.9358864	1.1014056	1.018646	up
A_22_P00002654	PIK3CD-AS2	-3.106595	-2.187029	-1.994128	0.9195652	1.1124663	1.0160158	up
A_22_P00024984	lnc-ARHGEF40-1	-3.266096	-2.142395	-2.358474	1.1237011	0.9076223	1.0156617	up
A_21_P0000942	lnc-RER1-1	-2.680442	-1.894199	-1.436869	0.786243	1.2435737	1.0149083	up
A_23_P68740	AIRE	-2.964701	-1.89909	-2.004751	1.0656111	0.9599507	1.0127809	up
A_19_P00321671	lnc-MMRN1-2	4.567233	5.890927	5.2689323	1.3236938	0.7016993	1.0126965	up
A_23_P109488	PIK3IP1	-2.474944	-1.387425	-1.539479	1.0875187	0.9354644	1.0114916	up
A_21_P0002206	lnc-PLEKHH2-2	-3.034401	-1.908527	-2.141516	1.125874	0.8928857	1.0093799	up
A_21_P0010417	lnc-SYNGR1-2	-3.527153	-2.348635	-2.705977	1.1785183	0.8211758	0.9998471	up

A_22_P00011051	lnc-NTRK2-2	-3.576212	-2.320561	-2.834871	1.2556517	0.7413418	0.9984968	up
A_33_P3257165	YPEL1	-2.638311	-1.276387	-2.004263	1.3619242	0.6340485	0.9979863	up
A_21_P0008331	LOC644919	-3.490112	-2.359253	-2.625347	1.1308594	0.8647652	0.9978123	up
A_21_P0012601	lnc-MMRN1-2	5.0045805	6.294951	5.7083883	1.2903705	0.7038078	0.9970892	up
A_33_P3347413	PBXIP1	-2.454829	-1.027675	-1.888473	1.4271538	0.5663555	0.9967546	up
A_23_P314101	SUSD2	1.9692774	2.905541	3.024867	0.9362636	1.0555897	0.9959266	up
A_33_P3336387	PAOX	-2.749222	-1.395352	-2.112069	1.3538692	0.6371524	0.9955108	up
A_22_P00001971	SOX5	-3.258665	-2.582136	-1.945071	0.6765287	1.3135941	0.9950614	up
A_23_P56673	INO80B	-0.920161	0.1802964	-0.03057	1.1004572	0.8895907	0.995024	up
A_23_P98900	CCDC92	0.7739177	2.1315465	1.4060698	1.3576288	0.6321521	0.9948905	up
A_23_P43476	VLDLR	-0.952049	0.6390705	-0.562033	1.5911193	0.3900161	0.9905677	up
A_23_P62709	SPRR3	6.388916	7.3606315	7.397888	0.9717155	1.0089722	0.9903438	up
A_23_P29773	LAMP3	2.296029	3.073464	3.4989233	0.7774348	1.2028942	0.9901645	up
A_23_P116587	OMP	-2.45047	-1.463502	-1.461089	0.9869678	0.9893811	0.9881745	up
A_33_P3398448	PARP10	3.7912169	4.512573	5.043769	0.7213559	1.252552	0.986954	up
A_24_P659836	SYCE3	-2.148029	-1.059519	-1.263979	1.08851	0.8840494	0.9862797	up
A_21_P0005823	lnc-ADAM2-4	-3.281801	-2.477874	-2.113574	0.8039269	1.168227	0.986077	up
A_23_P61524	CCDC71	-0.378461	0.5475397	0.6653862	0.9260006	1.0438471	0.9849239	up
A_23_P129629	MT3	4.7734776	6.014939	5.4995565	1.2414613	0.726079	0.9837702	up
A_23_P166459	LGALS1	6.6195726	7.62773	7.578888	1.0081573	0.9593153	0.9837363	up
A_21_P0007217	lnc-LGALS12-2	4.5118475	5.801226	5.184101	1.2893786	0.6722536	0.9808161	up
A_33_P3339531	CHADL	-1.74203	-0.471253	-1.05408	1.2707767	0.6879501	0.9793634	up
A_23_P411953	PYGO2	3.1076813	4.13314	4.0398455	1.0254588	0.9321642	0.9788115	up
A_33_P3229239	HIST2H2BF	-2.699273	-1.462066	-1.979847	1.2372072	0.7194259	0.9783166	up
A_23_P79122	USE1	1.7521334	2.9800992	2.4795246	1.2279658	0.7273912	0.9776785	up
A_23_P137665	CHI3L1	1.0313001	2.2214627	1.7875576	1.1901627	0.7562575	0.9732101	up
A_23_P103310	S100A7	-2.539797	-1.18814	-1.94801	1.3516569	0.5917869	0.9717219	up
A_23_P139786	OASL	4.297429	5.213718	5.321764	0.9162889	1.0243349	0.9703119	up
A_24_P289260	DACT2	-2.80254	-1.22255	-2.444985	1.5799892	0.3575542	0.9687717	up
A_33_P3417487	SCUBE1	-3.207609	-2.188061	-2.290164	1.0195475	0.9174449	0.9684962	up
A_32_P129669	FRMPD3	-0.527058	0.3785772	0.5013571	0.9056354	1.0284152	0.9670253	up
A_23_P115467	S100A5	-0.555924	0.2385416	0.5829463	0.7944655	1.1388702	0.9666679	up
A_33_P3350673	HOPX	-1.688369	-0.843257	-0.606111	0.8451128	1.0822587	0.9636858	up
A_33_P3305088	LOC100129840	-2.904726	-1.648683	-2.233456	1.2560425	0.6712694	0.963656	up
A_22_P00002902	lnc-C4orf42-1	-3.351716	-2.533247	-2.24332	0.8184688	1.1083965	0.9634327	up
A_21_P0000671	MT1E	2.6969337	3.8294072	3.4892101	1.1324735	0.7922764	0.962375	up
A_33_P3216938	CAND1.11	-0.170218	1.0603542	0.520093	1.2305722	0.690311	0.9604416	up
A_33_P3274245	ENDOV	-2.513248	-1.456354	-1.65371	1.0568943	0.8595376	0.958216	up
A_23_P1962	RARRES3	1.8518915	2.710569	2.9075947	0.8586774	1.0557032	0.9571903	up
A_21_P0009341	WFDC21P	3.8710651	4.7123876	4.9418945	0.8413224	1.0708294	0.9560759	up
A_21_P0000541	LOC100128164	-2.869515	-2.028734	-1.798683	0.8407812	1.0708327	0.955807	up
A_23_P55936	FCGRT	3.291544	4.3709006	4.1225834	1.0793567	0.8310394	0.9551981	up
A_24_P245298	TNFSF12	0.3227005	1.4047394	1.1455002	1.0820389	0.8227997	0.9524193	up
A_23_P202905	TIRAP	-2.68925	-1.713477	-1.761683	0.9757731	0.9275677	0.9516704	up
A_33_P3220698	EPS8L1	6.3363085	7.312732	7.2628727	0.9764237	0.9265642	0.951494	up
A_21_P0009942	LOC102724734	-3.590915	-2.704638	-2.576543	0.8862777	1.0143726	0.9503252	up
A_21_P0002580	DNAJC27-AS1	-2.600929	-1.708781	-1.594639	0.892148	1.0062895	0.9492188	up
A_23_P375165	TEX19	-2.645539	-1.185641	-2.211976	1.4598975	0.4335628	0.9467301	up
A_23_P106806	PRSS27	-0.864934	0.3017101	-0.138629	1.1666441	0.7263045	0.9464743	up
A_33_P3398437	MGA	0.4907336	1.539484	1.3330665	1.0487504	0.8423328	0.9455416	up
A_22_P00014741	SLC30A7	-3.37826	-2.50932	-2.361827	0.8689401	1.0164332	0.9426867	up
A_24_P475349	RAB6B	-3.149712	-2.26426	-2.150109	0.8854518	0.9996023	0.9425271	up
A_33_P3367577	EIF4ENIF1	-2.696905	-1.944743	-1.567426	0.7521615	1.1294789	0.9408202	up
A_22_P00013759	lnc-RP11-90J19.1.1-1	-2.611708	-1.791875	-1.551472	0.8198323	1.0602355	0.9400339	up
A_33_P3422802	ULBP1	0.203958	2.0814137	0.204237	1.8774557	0.0002789	0.9388673	up

A_23_P215669	POLR2J2	1.8301373	2.806069	2.7314777	0.9759316	0.9013405	0.9386361	up
A_23_P359245	MET	4.295638	5.356255	5.112049	1.060617	0.816411	0.938514	up
A_23_P125204	OR10G8	-3.084076	-2.107534	-2.18511	0.9765418	0.8989656	0.9377537	up
A_21_P0013278	LOC728743	-1.74001	-0.928229	-0.678522	0.8117809	1.0614886	0.9366348	up
A_21_P0009450	lnc-LRRC30-1	-0.610636	0.2797999	0.3712792	0.8904362	0.9819155	0.9361759	up
A_21_P0011535	LGALS9	0.6651502	1.3947225	1.8069334	0.7295723	1.1417832	0.9356778	up
A_33_P3368830	LY9	1.6420403	2.5247283	2.6302052	0.8826881	0.9881649	0.9354265	up
A_33_P3367701	TMEM164	-2.543265	-1.36471	-1.853182	1.178555	0.6900835	0.9343193	up
A_33_P3392537	TK2	-1.38605	-0.620873	-0.283171	0.7651768	1.1028795	0.9340282	up
A_21_P0001255	lnc-TNFRSF14-1	-2.620794	-2.126414	-1.247305	0.4943805	1.3734894	0.9339349	up
A_24_P79808	PBXIP1	-1.123178	0.0991335	-0.47767	1.2223115	0.6455083	0.9339099	up
A_33_P3397348	LINC01341	-2.914705	-1.742958	-2.222088	1.1717472	0.6926177	0.9321824	up
A_33_P3282840	RPS29	3.1875286	4.3816333	3.8566008	1.1941047	0.6690722	0.9315884	up
A_23_P130753	DBP	0.6029911	1.8259621	1.2413702	1.222971	0.6383791	0.9306751	up
A_24_P372134	TMEM140	-2.500188	-1.490431	-1.649951	1.0097568	0.8502371	0.929997	up
A_23_P397910	CBLC	1.697175	2.6686683	2.5818682	0.9714932	0.8846932	0.9280932	up
A_21_P0002350	lnc-SMARCAL1-3	-2.15636	-0.198702	-2.260489	1.9576578	-0.104129	0.9267646	up
A_33_P3297444	ABTB1	3.6009274	4.7184052	4.3368254	1.1174779	0.735898	0.926688	up
A_23_P145694	ASNS	6.1079187	7.9342084	6.132778	1.8262897	0.0248594	0.9255746	up
A_23_P132159	USP18	2.319168	2.8994079	3.589446	0.5802398	1.270278	0.9252589	up
A_21_P0011456	PLA2G10	0.4967699	1.3467336	1.4964886	0.8499637	0.9997187	0.9248412	up
A_33_P3293918	SH2D3C	-2.959214	-1.812619	-2.256777	1.1465957	0.7024376	0.9245167	up
A_22_P00001870	LOC101929988	-2.540302	-1.842568	-1.39044	0.6977339	1.1498623	0.9237981	up
A_23_P414211	PAX4	-2.350373	-1.349189	-1.504124	1.001184	0.8462491	0.9237166	up
A_33_P3355944	LINC00908	-1.622113	-0.621415	-0.786417	1.0006986	0.8356967	0.9181977	up
A_32_P25357	CDH15	-2.313493	-0.932052	-1.862454	1.3814406	0.4510384	0.9162395	up
A_33_P3346538	lnc-WWP2-1	-2.762535	-1.748803	-1.944093	1.0137315	0.8184414	0.9160865	up
A_23_P330461	TMC4	3.3864546	4.6104836	3.993637	1.2240291	0.6071825	0.9156058	up
A_23_P19348	CUL7	-0.544072	0.507791	0.2345982	1.0518632	0.7786703	0.9152668	up
A_21_P0000556	LINC00883	-3.50303	-2.917439	-2.260301	0.5855906	1.2427287	0.9141597	up
A_23_P15108	YPEL3	0.1902671	1.3717942	0.8349414	1.1815271	0.6446743	0.9131007	up
A_33_P3412294	ZSCAN2	0.3813672	1.4127522	1.175075	1.031385	0.7937079	0.9125464	up
A_33_P3413905	ADM2	0.6719718	2.153234	1.0092549	1.4812622	0.3372831	0.9092727	up
A_21_P0003286	lnc-ADAMTS9-3	-2.056062	-1.148631	-1.145221	0.9074311	0.910841	0.9091361	up
A_23_P348208	SPRR1A	3.422473	4.495251	4.1674986	1.0727782	0.7450256	0.9089019	up
A_22_P00016954	lnc-TSPYL6-2	-2.909811	-2.475422	-1.526397	0.4343891	1.3834136	0.9089014	up
A_23_P428129	CDKN1C	2.987568	4.1667285	3.622178	1.1791606	0.6346102	0.9068854	up
A_32_P452655	LGALS9C	6.132572	6.800081	7.275119	0.6675086	1.1425467	0.9050277	up
A_33_P3328365	lnc-C21orf90-1	-3.204898	-2.446618	-2.154816	0.7582798	1.0500824	0.9041811	up
A_33_P3290955	KIAA1875	3.720913	4.698726	4.5511007	0.9778132	0.8301878	0.9040005	up
A_32_P74409	C11orf96	-0.046137	1.272038	0.4420376	1.3181748	0.4881744	0.9031746	up
A_33_P3424861	FAM118A	2.7397156	3.6983352	3.5872774	0.9586196	0.8475618	0.9030907	up
A_23_P58538	EPB41L4A-AS1	5.198539	6.478275	5.723274	1.279736	0.5247355	0.9022357	up
A_23_P137856	MUC1	-0.591872	0.4398499	0.178658	1.0317221	0.7705302	0.9011262	up
A_33_P3400374	HELZ2	4.8009157	5.508837	5.8927116	0.7079215	1.0917959	0.8998587	up
A_23_P71880	SPINK4	-0.396317	0.9567919	0.0500417	1.3531094	0.4463592	0.8997343	up
A_23_P324340	DISP2	0.736186	1.7705393	1.5005827	1.0343533	0.7643967	0.899375	up
A_33_P3280784	TMEM254	-2.764472	-1.839508	-1.890904	0.9249647	0.8735688	0.8992667	up
A_33_P3247057	SMIM5	3.5074463	4.7166286	4.096281	1.2091823	0.5888348	0.8990085	up
A_23_P39024	WDR83	1.7579718	2.6299376	2.6757803	0.8719659	0.9178085	0.8948872	up
A_21_P0014633	LOC100505824	-2.094896	-0.982017	-1.418131	1.1128798	0.676765	0.8948224	up
A_23_P101380	B3GNT8	-0.021911	0.7425585	1.0015101	0.7644692	1.0234208	0.893945	up
A_23_P169428	TRUB2	3.68534	4.58246	4.574359	0.89712	0.889019	0.8930695	up
A_33_P3345344	DRGX	-2.29825	-1.149723	-1.662668	1.1485267	0.6355815	0.8920541	up
A_22_P00003384	lnc-CCDC102B-8	-1.446634	-0.42798	-0.681757	1.0186543	0.7648778	0.8917661	up
A_23_P12992	TRMT112	6.97577	7.8167377	7.9154654	0.8409677	0.9396954	0.8903315	up

A_21_P0000264	SNORD74	0.183023	1.5125237	0.6282859	1.3295007	0.4452629	0.8873818	up
A_23_P26511	GDPD3	1.8632727	3.1229777	2.376912	1.2597051	0.5136395	0.8866723	up
A_23_P74097	TCEB3	0.7939105	1.623219	1.7374349	0.8293085	0.9435244	0.8864164	up
A_24_P190472	SLPI	4.6195774	5.8537774	5.1575394	1.2342	0.537962	0.886081	up
A_23_P210465	PI3	-0.221337	1.0288801	0.2993484	1.250217	0.5206852	0.8854511	up
A_22_P00018036	lnc-ZNF236-1	0.8586736	1.6190157	1.868784	0.7603421	1.0101104	0.8852263	up
A_33_P3392000	LINC00963	2.6388102	3.607039	3.440957	0.9682288	0.8021469	0.8851879	up
A_23_P114713	CYP4B1	-2.966326	-1.699404	-2.462897	1.2669225	0.5034289	0.8851757	up
A_23_P91850	IL20RB	1.153213	2.7466187	1.3290787	1.5934057	0.1758657	0.8846357	up
A_33_P3280811	TTC16	-2.247774	-1.404244	-1.322183	0.8435297	0.925591	0.8845604	up
A_33_P3249872	FBLN1	-0.521972	0.5877338	0.1366444	1.1097054	0.6586161	0.8841607	up
A_23_P163492	BAIAP3	-1.242274	-0.15645	-0.560215	1.085824	0.6820598	0.8839419	up
A_19_P00320970	LOC101929696	-2.575394	-1.628737	-1.754774	0.9466574	0.8206203	0.8836389	up
A_33_P3251776	HSD3B7	-0.133355	0.6788931	0.8190517	0.8122478	0.9524064	0.8823271	up
A_22_P00012735	lnc-RAB9B-1	-2.598295	-2.687506	-0.744641	-0.089212	1.8536539	0.8822211	up
A_33_P3531979	LOC283352	0.5552988	1.9398246	0.9345665	1.3845258	0.3792677	0.8818968	up
A_33_P3378284	COX6B2	4.434986	5.3803906	5.252269	0.9454045	0.8172827	0.8813436	up
A_23_P100963	SPNS3	0.5012474	1.4703183	1.2941566	0.9690709	0.7929092	0.88099	up
A_22_P00009644	LOC101929106	-3.095478	-2.115091	-2.314016	0.980387	0.781462	0.8809245	up
A_23_P88678	C15orf27	-3.524459	-2.75773	-2.531685	0.7667289	0.9927738	0.8797514	up
A_23_P96087	H1FX	1.5055017	2.2892098	2.4808054	0.7837081	0.9753037	0.8795059	up
A_23_P37914	SLC5A11	-2.801485	-1.617108	-2.228812	1.1843774	0.5726733	0.8785254	up
A_23_P6413	SELM	-0.782528	0.2627468	-0.071073	1.0452747	0.7114554	0.878365	up
A_23_P66241	MT1M	0.7511149	1.8174543	1.4409556	1.0663395	0.6898408	0.8780902	up
A_21_P0006847	lnc-C10orf126-2	-2.172037	-1.806728	-0.783276	0.3653088	1.388761	0.8770349	up
A_24_P56837	ASB16	-1.109375	-0.269979	-0.197984	0.8393955	0.9113908	0.8753932	up
A_23_P85783	PHGDH	4.2208004	5.752352	4.4357376	1.5315518	0.2149372	0.8732445	up
A_22_P00019654	LOC101929217	-2.765871	-2.020949	-1.768816	0.7449215	0.9970553	0.8709884	up
A_33_P3410385	ARHGAP44	-2.43962	-1.913621	-1.224207	0.5259981	1.2154121	0.8707051	up
A_23_P2283	TAC3	-1.648959	-0.786972	-0.769629	0.8619871	0.8793302	0.8706586	up
A_22_P00014191	lnc-SDR42E1-1	0.0079889	0.9356918	0.8214092	0.9277029	0.8134203	0.8705616	up
A_24_P234732	MXD4	-0.779721	0.2083159	-0.027808	0.9880366	0.7519126	0.8699746	up
A_33_P3406866	NFASC	-3.259063	-2.396924	-2.382351	0.8621392	0.8767123	0.8694258	up
A_24_P354724	TAGAP	-2.185381	-1.480608	-1.152556	0.7047734	1.0328255	0.8687995	up
A_33_P3237634	TSC22D3	3.7159061	5.0394053	4.1231003	1.3234992	0.4071941	0.8653467	up
A_23_P170574	SNAI3	-1.451912	-0.720734	-0.453741	0.7311783	0.9981713	0.8646748	up
A_22_P00018331	lnc-ZNHIT2-1	-2.8028	-2.008294	-1.869378	0.7945056	0.9334221	0.8639639	up
A_33_P3357918	PGLS	5.59192	6.684999	6.226616	1.0930791	0.634696	0.8638876	up
A_23_P309381	HIST2H2AA4	2.0358171	3.164123	2.6348429	1.1283059	0.5990257	0.8636658	up
A_22_P00014606	LOC101927616	-1.334875	-0.47349	-0.469276	0.8613849	0.8655987	0.8634918	up
A_23_P57364	TFF2	-3.176982	-2.640719	-1.987468	0.5362632	1.1895134	0.8628883	up
A_24_P68079	TRANK1	-1.834504	-1.074652	-0.868805	0.7598519	0.9656987	0.8627753	up
A_23_P116557	LGALS9	1.8463345	2.4524426	2.9638453	0.6061082	1.1175108	0.8618095	up
A_21_P0005078	lnc-SOD2-2	-3.134723	-2.325249	-2.221806	0.8094735	0.9129164	0.861195	up
A_23_P115202	CRNN	-3.112321	-2.257523	-2.246483	0.8547981	0.8658378	0.860318	up
A_32_P47754	SLC2A14	2.796996	3.9776387	3.3366528	1.1806426	0.5396566	0.8601496	up
A_24_P374634	STAU2	0.0172615	0.7533727	1.0013266	0.7361112	0.9840651	0.8600881	up
A_21_P0013662	HSPB1	8.779063	9.857082	9.421054	1.0780191	0.6419907	0.8600049	up
A_33_P3271975	LOC100132368	-2.135082	-1.259453	-1.292895	0.875629	0.8421869	0.8589079	up
A_24_P280846	GOLT1A	-0.059369	0.7943568	0.8043203	0.8537259	0.8636894	0.8587077	up
A_23_P1523	RHOD	2.7187386	3.7283597	3.4241705	1.0096211	0.7054319	0.8575265	up
A_23_P88909	SYNGR3	-2.357588	-1.568181	-1.432094	0.7894077	0.9254947	0.8574512	up
A_23_P85015	MAOB	1.1568027	1.8254609	2.201518	0.6686583	1.0447154	0.8566868	up
A_33_P3216150	PRB4	-0.987707	-0.070468	-0.192109	0.9172387	0.795598	0.8564184	up
A_21_P0009404	lnc-ACCN1-1	-2.355459	-1.488058	-1.510396	0.8674016	0.8450632	0.8562324	up
A_33_P3391418	TMEM40	-1.689807	-0.960684	-0.706601	0.7291222	0.9832053	0.8561637	up

A_33_P3393821	C1R	1.1189532	1.8758245	2.0741024	0.7568712	0.9551492	0.8560102	up
A_23_P117582	JDP2	2.986494	4.512078	3.172347	1.5255837	0.185853	0.8557184	up
A_23_P397856	TIRAP	-2.89057	-1.879216	-2.190896	1.011354	0.6996737	0.8555138	up
A_21_P0012109	LOC101929395	-0.969286	-0.411082	0.1829352	0.5582042	1.1522212	0.8552127	up
A_23_P89101	SEPT12	-3.3462	-2.443604	-2.539135	0.9025958	0.807065	0.8548304	up
A_33_P3224819	SLC45A4	0.5776091	1.3658366	1.4983082	0.7882276	0.9206991	0.8544633	up
A_23_P310274	PRSS3P2	3.1612415	4.1003013	3.930729	0.9390597	0.7694874	0.8542736	up
A_33_P3298159	PTGDS	-1.041488	-0.006663	-0.36848	1.0348248	0.673008	0.8539164	up
A_33_P3416150	SBF1P1	2.224039	3.2677588	2.8877535	1.0437198	0.6637144	0.8537171	up
A_33_P3287646	HSPB1	8.801475	9.799494	9.508427	0.9980192	0.7069521	0.8524857	up
A_33_P3236319	lnc-CBWD5-2	-2.723529	-1.866757	-1.875336	0.8567722	0.8481929	0.8524826	up
A_24_P942969	FUT2	1.0576797	2.1056752	1.7144217	1.0479956	0.6567421	0.8523689	up
A_23_P384499	RPTOR	3.4834604	4.3638425	4.3065825	0.8803821	0.823122	0.851752	up
A_23_P250358	HERC6	-0.619025	-0.095841	0.5612049	0.5231838	1.1802297	0.8517068	up
A_23_P120002	SP110	2.6661043	3.2972379	3.7376156	0.6311336	1.0715113	0.8513224	up
A_33_P3392823	USE1	3.6414118	4.790544	4.193468	1.1491323	0.5520563	0.8505943	up
A_23_P56978	PTK6	2.8226023	3.8408866	3.5049238	1.0182843	0.6823216	0.8503029	up
A_33_P3311046	OR2M7	-1.889876	-1.059809	-1.022108	0.8300667	0.8677683	0.8489175	up
A_23_P218770	RAC2	3.2731876	4.145622	4.0959806	0.8724341	0.822793	0.8476136	up
A_33_P3220929	JOSD2	-1.619196	-0.79485	-0.748833	0.8243451	0.8703623	0.8473537	up
A_33_P3215517	RCC2	0.1376457	1.1552858	0.8124361	1.0176401	0.6747904	0.8462153	up
A_23_P4798	ZNF581	1.7863607	3.0529532	2.209485	1.2665925	0.4231243	0.8448584	up
A_33_P3377256	OGFRP1	-2.744598	-1.955452	-1.845668	0.7891455	0.8989301	0.8440378	up
A_33_P3220837	MAFB	0.7956424	1.9661059	1.3130312	1.1704636	0.5173888	0.8439262	up
A_33_P3402615	SLC6A9	3.553175	4.8161197	3.9765167	1.2629447	0.4233418	0.8431432	up
A_23_P78099	VTN	-3.059485	-1.976547	-2.456154	1.0829372	0.6033306	0.8431339	up
A_24_P317762	LY6E	5.541753	6.156405	6.6129208	0.6146522	1.071168	0.8429101	up
A_23_P207675	PLCD3	2.429865	3.3505788	3.1923962	0.9207139	0.7625313	0.8416226	up
A_33_P3249364	TMTC1	-2.601725	-1.684908	-1.835872	0.9168172	0.7658529	0.8413351	up
A_33_P3358295	ABTB1	1.508173	2.543202	2.155239	1.0350289	0.6470661	0.8410475	up
A_23_P111037	HIST1H3A	5.079521	5.9324985	5.9074383	0.8529773	0.8279171	0.8404472	up
A_22_P00023295	lnc-AKIRIN1-1	-3.389214	-2.103502	-2.996353	1.2857118	0.3928607	0.8392862	up
A_21_P0009461	lnc-SEH1L-1	-3.024746	-2.01396	-2.358266	1.0107853	0.6664794	0.8386323	up
A_24_P215882	ARHGDIG	-2.214113	-1.506406	-1.246015	0.7077069	0.9680977	0.8379023	up
A_23_P67896	SCN3A	-2.756881	-2.030907	-1.808359	0.7259734	0.9485214	0.8372474	up
A_22_P00017446	lnc-VPS4A-1	1.7536516	2.6266456	2.5543365	0.872994	0.8006849	0.8368394	up
A_24_P35400	SARDH	-2.785562	-2.155305	-1.743436	0.6302562	1.0421252	0.8361907	up
A_23_P397376	MAF	-2.160752	-1.406817	-1.242788	0.7539349	0.9179645	0.8359497	up
A_33_P3299665	LOC100505530	-1.737269	-0.972122	-0.831416	0.7651472	0.9058533	0.8355003	up
A_24_P293114	SPTBN4	-3.34043	-2.475484	-2.53587	0.8649454	0.8045597	0.8347526	up
A_23_P256205	ABLIM3	-1.390743	-0.397078	-0.718727	0.9936647	0.6720161	0.8328404	up
A_23_P386942	DIRAS1	-2.43765	-1.595157	-1.614951	0.8424931	0.8226991	0.8325961	up
A_21_P0000340	SNORA60	-0.643208	0.2540255	0.1244187	0.897233	0.7676263	0.8324297	up
A_21_P0011684	XLOC_l2_006665	0.430603	1.476531	1.0489583	1.045928	0.6183553	0.8321417	up
A_23_P42802	PDIA4	2.0337753	2.7401395	2.9913082	0.7063642	0.9575329	0.8319485	up
A_23_P106898	ORAI3	-2.404265	-1.407775	-1.738149	0.9964895	0.6661158	0.8313026	up
A_33_P3312301	CIT	-2.570405	-1.623375	-1.855027	0.9470298	0.7153776	0.8312037	up
A_33_P3226377	PRH2	-1.626668	-0.724593	-0.867501	0.9020753	0.7591672	0.8306213	up
A_22_P00022364	LOC100507054	0.1838851	1.0933414	0.9347224	0.9094563	0.7508373	0.8301468	up
A_32_P198325	LRRC75B	-1.745671	-0.738095	-1.095191	1.007576	0.6504803	0.8290282	up
A_23_P68998	MIOX	-2.062556	-1.214263	-1.254038	0.8482928	0.8085175	0.8284051	up
A_21_P0013567	RBPJ	1.3875637	2.147204	2.2842417	0.7596402	0.896678	0.8281591	up
A_33_P3405022	LETM1	0.1887813	1.1201477	0.9133906	0.9313664	0.7246094	0.8279879	up
A_32_P62963	KRT16P2	6.4813976	7.5032954	7.114765	1.0218978	0.6333675	0.8276327	up
A_23_P135381	SP5	4.7268333	5.5279374	5.580065	0.8011041	0.8532314	0.8271678	up
A_32_P93852	BOD1	2.6069565	3.5569758	3.3111324	0.9500194	0.704176	0.8270977	up

A_22_P00001466	lnc-AQP9-1	0.6000748	1.6436691	1.2103829	1.0435944	0.6103082	0.8269513	up
A_21_P0000494	SNORA16B	2.8693962	3.7035422	3.6887293	0.834146	0.8193331	0.8267396	up
A_23_P27734	NPAS1	-2.613081	-1.705736	-1.867103	0.9073455	0.7459781	0.8266618	up
A_22_P00013074	LOC101929237	-2.290295	-1.58213	-1.346191	0.7081642	0.9441032	0.8261337	up
A_24_P8371	SPNS2	3.9512796	5.023686	4.5296144	1.0724063	0.5783348	0.8253706	up
A_23_P122815	CALU	1.356194	1.9750285	2.38721	0.6188345	1.0310159	0.8249252	up
A_23_P48596	RNASE1	1.3218427	1.6826949	2.6108112	0.3608522	1.2889686	0.8249104	up
A_21_P0009324	lnc-CCR7-1	2.6684084	3.6310086	3.3545399	0.9626002	0.6861315	0.8243659	up
A_24_P301837	HRH3	-3.347691	-2.411567	-2.636088	0.9361239	0.7116036	0.8238637	up
A_22_P00016586	LOC101929697	-3.364902	-2.574521	-2.508191	0.790381	0.8567102	0.8235456	up
A_33_P3870056	LOC283335	-0.404218	0.5198855	0.3177352	0.9241033	0.7219529	0.8230281	up
A_23_P218476	B9D2	2.4448328	3.1707568	3.3646078	0.725924	0.919775	0.8228495	up
A_21_P0000277	SNORD37	-1.449547	-0.575126	-0.678869	0.8744206	0.7706776	0.8225491	up
A_22_P00016327	lnc-TMEM14C-1	-3.292793	-2.623238	-2.318203	0.6695554	0.9745908	0.8220731	up
A_23_P125423	C1R	-1.213221	-0.467488	-0.315063	0.7457328	0.8981576	0.8219452	up
A_23_P52031	PGM1	3.4778185	4.340153	4.258876	0.8623347	0.7810574	0.821696	up
A_22_P00000427	BOLA3-AS1	0.5694695	1.3171892	1.4651275	0.7477198	0.895658	0.8216889	up
A_23_P149529	TACSTD2	7.5613575	8.49917	8.26622	0.9378128	0.7048626	0.8213377	up
A_23_P15174	MT1F	0.9882026	1.9195657	1.6993551	0.9313631	0.7111526	0.8212578	up
A_23_P74012	SPRR1A	5.394229	6.437695	5.992647	1.0434661	0.5984182	0.8209422	up
A_22_P00005359	DPYS	-2.518451	-1.709303	-1.68694	0.8091478	0.831511	0.8203294	up
A_23_P386888	LPA	-3.212116	-2.501466	-2.283065	0.7106497	0.9290514	0.8198506	up
A_24_P687582	TADA2B	-2.261001	-1.364142	-1.521074	0.8968592	0.7399273	0.8183932	up
A_22_P00011597	RAB30-AS1	-3.44458	-2.45892	-2.796979	0.9856608	0.6476009	0.8166309	up
A_23_P141992	HSD11B1L	-3.331633	-2.289514	-2.74225	1.0421193	0.5893829	0.8157511	up
A_24_P79070	GNG8	-2.358451	-1.346206	-1.739576	1.0122457	0.618875	0.8155604	up
A_22_P00014771	lnc-SLC36A4-1	-0.882544	-0.053588	-0.083497	0.8289557	0.7990475	0.8140016	up
A_33_P3301034	LRRC28	-3.369362	-2.169797	-2.940946	1.1995654	0.428416	0.8139907	up
A_33_P3251093	SLC36A1	2.3583708	3.3381906	3.0037622	0.9798198	0.6453915	0.8126056	up
A_22_P00015122	lnc-SNX20-5	3.2770796	4.2701335	3.9078226	0.9930539	0.630743	0.8118985	up
A_22_P00013211	ZNRD1-AS1	-3.148322	-2.39177	-2.281906	0.7565525	0.8664165	0.8114845	up
A_21_P0014643	LOC100507420	-2.579021	-1.764634	-1.770458	0.8143873	0.8085632	0.8114753	up
A_33_P3225522	OAS2	2.1454735	2.2043529	3.7084217	0.0588794	1.5629482	0.8109138	up
A_24_P200219	UPK1B	-0.389289	0.5413623	0.3008885	0.9306512	0.6901774	0.8104143	up
A_22_P00001009	LMO7DN	-1.512269	-0.639832	-0.765228	0.872437	0.7470412	0.8097391	up
A_22_P00000775	lnc-ADD3-1	-1.703305	-0.902463	-0.886271	0.8008423	0.8170342	0.8089383	up
A_33_P3354106	KPNA7	-1.895546	-0.667852	-1.505401	1.227694	0.3901458	0.8089199	up
A_23_P33683	MARCH2	-2.352472	-1.490298	-1.598346	0.8621745	0.7541266	0.8081505	up
A_23_P17345	MAFB	-0.513248	0.5403333	0.0493932	1.0535812	0.5626411	0.8081112	up
A_23_P23380	ELOVL1	3.6750517	4.547788	4.418357	0.8727365	0.7433052	0.8080208	up
A_23_P152838	CCL5	0.4360261	1.2098742	1.2778773	0.7738481	0.8418512	0.8078496	up
A_23_P32135	C9orf9	0.2762432	1.3974218	0.7691608	1.1211786	0.4929175	0.8070481	up
A_23_P79587	ALPP	3.8623838	5.1111674	4.2257643	1.2487836	0.3633804	0.806082	up
A_33_P3399593	CCDC134	-2.22152	-1.214657	-1.616531	1.0068622	0.6049881	0.8059252	up
A_22_P00016998	DSCR9	-2.341035	-1.394526	-1.675878	0.9465098	0.6651573	0.8058336	up
A_23_P88767	PLA2G10	2.0154705	2.675403	2.9654264	0.6599326	0.9499559	0.8049443	up
A_33_P3346327	LOC403323	-0.812394	0.0633397	-0.078474	0.8757334	0.7339196	0.8048265	up
A_23_P80382	PRR5	3.679597	4.5707145	4.397872	0.8911176	0.7182751	0.8046963	up
A_33_P3331426	XLOC_l2_008130	-1.023185	-0.132323	-0.305444	0.8908625	0.7177415	0.804302	up
A_33_P3645465	PDCD4-AS1	-0.655846	0.1811652	0.1151013	0.8370113	0.7709475	0.8039794	up
A_21_P0005818	LOC100507420	0.0872197	0.4982333	1.2824912	0.4110136	1.1952715	0.8031426	up
A_22_P00001180	LOC100507530	-2.385598	-1.559189	-1.606474	0.8264084	0.7791233	0.8027659	up
A_21_P0000343	SNORA5C	-2.022351	-1.284254	-1.155247	0.7380972	0.8671045	0.8026009	up
A_23_P39465	BST2	5.9167166	6.5628324	6.874922	0.6461158	0.9582052	0.8021605	up
A_23_P501985	CSF2RA	-1.399307	-0.236614	-0.957871	1.162693	0.4414368	0.8020649	up
A_33_P3212274	F2R	-2.649096	-1.831536	-1.862747	0.81756	0.7863491	0.8019545	up

A_22_P00004713	LOC100506844	-0.853737	0.2369046	-0.342004	1.090642	0.5117331	0.8011875	up
A_23_P376591	CLYBL	-1.771727	-0.513549	-1.427797	1.2581778	0.3439293	0.8010536	up
A_23_P218579	GLB1L	-1.657724	-0.914597	-0.798869	0.7431274	0.8588553	0.8009913	up
A_33_P3263614	ASAP3	-3.351883	-2.533465	-2.568506	0.8184185	0.7833774	0.800898	up
A_33_P3512350	LOC339807	-1.663669	-0.840661	-0.885959	0.8230081	0.7777104	0.8003593	up
A_23_P130689	ELOF1	3.0426607	3.8188195	3.8617897	0.7761588	0.819129	0.7976439	up
A_23_P501877	ZFP64	2.0102892	3.0013509	2.6115055	0.9910617	0.6012163	0.796139	up
A_23_P321223	PMCH	-2.812587	-2.264257	-1.768711	0.5483298	1.0438755	0.7961027	up
A_22_P00014460	AGAP1-IT1	-0.457482	0.3222508	0.354156	0.7797332	0.8116384	0.7956858	up
A_22_P00007187	lnc-GOLGA8J-3	0.8992024	1.9963565	1.3902535	1.0971541	0.4910512	0.7941027	up
A_23_P73637	TEX13B	-3.306181	-2.712835	-2.3115	0.5933459	0.9946802	0.794013	up
A_33_P3419594	PDE9A	1.5070944	2.3389783	2.2632084	0.8318839	0.756114	0.793999	up
A_23_P129101	HEXA	2.766779	3.756164	3.3648348	0.9893851	0.5980558	0.7937205	up
A_33_P3254946	PLEKHH3	2.2599773	3.151527	2.9556742	0.8915496	0.6956968	0.7936232	up
A_24_P318967	PDXK	4.4080095	5.1838326	5.2183228	0.7758231	0.8103132	0.7930682	up
A_22_P00016876	TUSC8	-1.997004	-1.227101	-1.181012	0.7699027	0.8159919	0.7929473	up
A_21_P0013231	XLOC_l2_013459	-2.280855	-1.501874	-1.474002	0.7789817	0.8068533	0.7929175	up
A_32_P45974	C2orf27A	-2.501313	-1.558553	-1.858601	0.9427598	0.6427119	0.7927358	up
A_24_P228026	CYB5D2	-1.120828	-0.213933	-0.44273	0.9068942	0.6780977	0.792496	up
A_33_P3857239	KRT42P	6.742091	7.780764	7.2877836	1.0386729	0.5456924	0.7921827	up
A_23_P89824	TMEM241	0.2069516	0.9429541	1.0535836	0.7360025	0.846632	0.7913172	up
A_32_P215938	GPSM1	3.938548	4.8504663	4.6091824	0.9119182	0.6706343	0.7912762	up
A_33_P3313835	PP14571	1.5704298	2.2588677	2.4628334	0.6884379	0.8924036	0.7904208	up
A_32_P230828	GAS5	5.425991	6.6671157	5.765542	1.2411246	0.339551	0.7903378	up
A_23_P87279	TRPM5	-1.806513	-1.235417	-0.797075	0.5710964	1.009438	0.7902672	up
A_23_P58266	S100P	8.401627	9.352247	9.030905	0.9506207	0.6292782	0.7899494	up
A_21_P0005060	lnc-C6orf192-1	-2.423722	-1.273565	-1.994549	1.1501575	0.4291735	0.7896655	up
A_33_P3327265	TESPA1	-2.538369	-1.738017	-1.75972	0.8003528	0.7786491	0.789501	up
A_23_P138849	KAT5	1.0137262	1.8362346	1.7698612	0.8225083	0.756135	0.7893217	up
A_23_P101742	MRPL54	2.9094687	3.7437549	3.6534595	0.8342862	0.7439909	0.7891386	up
A_33_P3360426	WDR1	0.027813	0.7418199	0.8915553	0.7140069	0.8637424	0.7888746	up
A_21_P0003889	FLJ36777	-2.037839	-1.159618	-1.338923	0.878221	0.698916	0.7885685	up
A_21_P0011840	LOC100507334	-3.385574	-2.590432	-2.607303	0.7951417	0.778271	0.7867063	up
A_24_P191312	SLC1A4	-3.198749	-1.575407	-3.249116	1.6233423	-0.050368	0.7864874	up
A_23_P77669	ZNF821	-1.776079	-1.085067	-0.894418	0.6910119	0.8816614	0.7863367	up
A_22_P00005585	SLC50A1	-0.118454	0.9601126	0.3747492	1.0785666	0.4932032	0.7858849	up
A_24_P419039	PCDH19	-2.805358	-2.182142	-1.857009	0.6232162	0.948349	0.7857826	up
A_23_P116797	C12orf10	3.2090225	3.9940019	3.9949026	0.7849793	0.7858801	0.7854297	up
A_23_P96158	KRT17	8.122621	9.0997925	8.714951	0.9771719	0.59233	0.784751	up
A_33_P3414242	MOG	-0.709088	0.400322	-0.250262	1.1094098	0.4588256	0.7841177	up
A_23_P106204	GSTZ1	1.0372286	1.9565816	1.6853409	0.919353	0.6481123	0.7837327	up
A_23_P23141	H3F3A	7.9420586	8.795765	8.655768	0.8537064	0.7137098	0.7837081	up
A_33_P3235546	RIMS3	-0.600028	0.5334778	-0.166136	1.1335053	0.4338918	0.7836985	up
A_33_P3363120	SCARNA10	-2.105503	-1.207655	-1.436107	0.8978486	0.6693959	0.7836223	up
A_24_P393611	GNAO1	-2.174121	-1.306024	-1.476003	0.8680973	0.6981177	0.7831075	up
A_23_P435477	TMPRSS13	-0.729363	0.4505119	-0.345758	1.1798754	0.383605	0.7817402	up
A_23_P125078	SLC26A11	-0.302891	0.3433566	0.6139851	0.6462479	0.9168763	0.7815621	up
A_24_P161036	ACOT1	3.5825481	4.4098544	4.315502	0.8273063	0.732954	0.7801302	up
A_23_P46936	EGR2	-1.410247	-0.838949	-0.42207	0.5712981	0.9881768	0.7797375	up
A_23_P169293	DMRT1	-2.702528	-1.827186	-2.018527	0.8753414	0.684001	0.7796712	up
A_24_P820037	SLC6A17	-2.025461	-1.195858	-1.296202	0.8296032	0.729259	0.7794311	up
A_24_P206776	CRYAB	-0.556751	0.7226024	-0.278656	1.2793536	0.2780957	0.7787247	up
A_23_P48826	TRIM69	-0.613915	0.2870646	0.0418954	0.9009795	0.6558104	0.7783949	up
A_22_P00019621	lnc-C1QTNF5-1	-3.205693	-2.607062	-2.248437	0.5986309	0.9572558	0.7779434	up
A_21_P0000336	SNORA54	0.88131	1.7036519	1.6136332	0.8223419	0.7323232	0.7773325	up
A_23_P430181	ZBTB3	1.2601881	2.2244706	1.8499575	0.9642825	0.5897694	0.7770259	up

A_23_P119916	WNT6	-2.87851	-2.180466	-2.024283	0.6980441	0.8542266	0.7761353	up
A_23_P109821	TADA3	5.173955	6.0120177	5.888115	0.8380628	0.71416	0.7761114	up
A_22_P00023573	lnc-MAB21L2-1	-1.771448	-0.790687	-1.200546	0.9807615	0.5709019	0.7758317	up
A_23_P322	EFNA4	1.6319494	2.4304976	2.3847303	0.7985482	0.7527809	0.7756646	up
A_23_P99632	RNF31	1.7446947	2.5864596	2.452773	0.8417649	0.7080784	0.7749217	up
A_24_P59667	JAK3	-0.883802	-0.264901	0.0455217	0.6189013	0.9293242	0.7741127	up
A_23_P153372	HSH2D	2.6029987	3.203237	3.5499973	0.6002383	0.9469986	0.7736185	up
A_21_P0000463	SNORD105B	2.714038	3.5776076	3.396593	0.8635697	0.6825552	0.7730625	up
A_33_P3326432	SEPW1	4.5098553	5.325353	5.2398577	0.8154979	0.7300024	0.7727502	up
A_33_P3305093	ZGLP1	-1.310631	-0.311885	-0.766045	0.9987464	0.5445862	0.7716663	up
A_33_P3229512	LOC100996842	-3.0234	-2.291901	-2.212847	0.7314992	0.8105526	0.7710259	up
A_21_P0000249	SNORD24	-2.287527	-1.631249	-1.403437	0.6562781	0.8840895	0.7701838	up
A_33_P3397763	TNFSF9	0.1701531	0.868341	1.0110259	0.6981878	0.8408728	0.7695303	up
A_21_P0007994	LINC00462	-2.78341	-1.838395	-2.189502	0.9450145	0.5939081	0.7694613	up
A_21_P0000318	SNORA24	2.356038	3.6324267	2.6177788	1.2763886	0.2617407	0.7690646	up
A_32_P923011	FAM138A	-0.48176	0.3773665	0.1967855	0.8591266	0.6785455	0.768836	up
A_23_P353524	IVL	4.378395	5.0054955	5.287792	0.6271005	0.9093971	0.7682488	up
A_21_P0012439	TRANK1	-2.914213	-2.492151	-1.800175	0.4220629	1.1140387	0.7680508	up
A_33_P3364289	HSH2D	0.092504	0.7491078	0.970345	0.6566038	0.877841	0.7672224	up
A_23_P210939	EIF6	5.3807964	6.072827	6.2230835	0.6920304	0.8422871	0.7671587	up
A_33_P3589217	SLC25A6	5.1384544	6.1048665	5.7058954	0.9664121	0.567441	0.7669265	up
A_33_P3365037	ERCC1	3.0893564	3.9444509	3.767622	0.8550944	0.6782656	0.76668	up
A_33_P3359704	ZNF703	-2.140535	-1.071154	-1.676974	1.0693808	0.4635611	0.7664709	up
A_23_P388780	DMAP1	1.4013181	2.1436162	2.1911201	0.7422981	0.7898021	0.7660501	up
A_22_P00016154	LARGE-AS1	-2.292382	-1.489235	-1.563434	0.8031464	0.7289481	0.7660472	up
A_23_P24389	CCDC88B	-1.543128	-0.774173	-0.781392	0.7689552	0.7617359	0.7653456	up
A_23_P139260	SLC22A18	3.5974932	4.409538	4.315851	0.8120446	0.718358	0.7652013	up
A_22_P00008559	LOC101927059	-2.685199	-1.660132	-2.181669	1.0250676	0.5035298	0.7642987	up
A_21_P0013514	XLOC_l2_014504	0.8866882	2.6785197	0.623023	1.7918315	-0.263665	0.7640832	up
A_23_P339818	ARRDC4	2.2614555	3.1466012	2.9044504	0.8851457	0.6429949	0.7640703	up
A_33_P3250028	EVI5L	1.3354449	2.3747087	1.8236885	1.0392637	0.4882436	0.7637536	up
A_33_P6805542	lnc-SERPINC1-1	4.6533546	5.828998	5.004305	1.1756434	0.3509502	0.7632968	up
A_23_P76435	GATC	0.5112824	1.333427	1.2148061	0.8221445	0.7035236	0.7628341	up
A_23_P10194	SEZ6L2	4.335911	5.3383555	4.858021	1.0024447	0.52211	0.7622774	up
A_32_P208424	H3F3A	7.5652246	8.43107	8.222796	0.8658457	0.6575718	0.7617088	up
A_33_P3418170	DDX58	0.4563646	0.6911163	1.7437449	0.2347517	1.2873802	0.761066	up
A_23_P46871	SLC29A3	0.3284559	1.2580538	0.9207521	0.9295979	0.5922961	0.760947	up
A_23_P414978	NUDT14	0.5601134	1.4727864	1.1675553	0.912673	0.6074419	0.7600575	up
A_21_P0013099	XLOC_l2_013125	-1.241274	-0.476948	-0.485537	0.7643261	0.7557373	0.7600317	up
A_23_P145681	ACTL6B	-2.783355	-1.843537	-2.205832	0.9398177	0.577523	0.7586703	up
A_21_P0014520	SYNE1-AS1	-3.425093	-2.839409	-2.495175	0.5856843	0.9299181	0.7578012	up
A_33_P3544887	TTC28	0.8817611	1.6933818	1.5857382	0.8116207	0.7039771	0.7577989	up
A_23_P203475	PRKCDBP	4.4530544	5.3581014	5.062701	0.9050469	0.6096468	0.7573469	up
A_33_P6819918	LOC100506844	0.1808319	1.1499271	0.7261	0.9690952	0.5452681	0.7571816	up
A_23_P36018	VSIG2	0.8025646	1.7746301	1.3447285	0.9720655	0.5421639	0.7571147	up
A_23_P345118	PIM1	1.445199	2.2315488	2.172408	0.7863498	0.7272091	0.7567795	up
A_33_P3224307	SCARNA12	3.0100365	3.5871053	3.946331	0.5770688	0.9362946	0.7566817	up
A_24_P787947	YPEL2	-0.715324	0.2542653	-0.171923	0.9695892	0.5434008	0.756495	up
A_23_P386905	UBXN6	0.9316979	1.820991	1.5549479	0.8892932	0.62325	0.7562716	up
A_23_P207367	STAT5A	-2.156535	-1.30685	-1.495306	0.8496852	0.6612291	0.7554572	up
A_23_P31921	ASS1	1.6053619	2.755258	1.966321	1.1498961	0.3609591	0.7554276	up
A_23_P154806	EPB41L1	5.745801	6.672095	6.330097	0.9262939	0.5842962	0.755295	up
A_33_P3299319	RPL28	-0.803747	0.1440244	-0.241008	0.9477711	0.5627389	0.755255	up
A_21_P0009234	lnc-AC003101.1.1-1	-2.026245	-1.148192	-1.394019	0.8780527	0.6322255	0.7551391	up
A_23_P119562	CFD	3.481018	4.2738185	4.198287	0.7928004	0.7172689	0.7550347	up

A_33_P3221177	ZDHHC20	-1.764705	-0.999752	-1.020113	0.7649531	0.7445922	0.7547727	up
A_24_P274270	STAT1	2.6275244	2.937965	3.826497	0.3104405	1.1989727	0.7547066	up
A_33_P3307886	TBXA2R	1.9917002	2.778936	2.7129116	0.7872357	0.7212114	0.7542236	up
A_33_P3381313	FAM160A1	-2.644146	-0.655763	-3.124117	1.988383	-0.479971	0.754206	up
A_33_P3238415	GSG1	-2.943298	-2.196501	-2.182426	0.7467971	0.7608726	0.7538349	up
A_33_P3295358	ANGPTL4	-0.28267	0.7141361	0.227706	0.9968057	0.5103755	0.7535906	up
A_33_P3393088	RAB6C-AS1	-2.015077	-1.16574	-1.35828	0.8493376	0.6567974	0.7530675	up
A_24_P206758	SNX15	-0.737784	0.1321716	-0.101906	0.8699555	0.6358776	0.7529166	up
A_24_P16036	PTRHD1	3.644167	4.5299573	4.262974	0.8857904	0.6188068	0.7522986	up
A_23_P206724	MT1E	3.6864977	4.4945245	4.3826504	0.8080268	0.6961527	0.7520898	up
A_33_P3287113	UVSSA	1.0169015	1.8287687	1.7090449	0.8118672	0.6921434	0.7520053	up
A_32_P147622	MCF2L-AS1	-0.655274	0.3249683	-0.131641	0.9802423	0.5236325	0.7519374	up
A_33_P3387050	C8orf82	5.708894	6.5406566	6.3809147	0.8317628	0.6720209	0.7518919	up
A_33_P3376090	OR1J4	-2.173118	-1.499502	-1.343663	0.6736164	0.8294549	0.7515357	up
A_24_P40978	CYHR1	1.1632438	2.2756648	1.5527167	1.112421	0.389473	0.750947	up
A_33_P3421984	HYAL4	2.9713879	3.799726	3.6448765	0.8283382	0.6734886	0.7509134	up
A_23_P201747	PADI2	-3.470549	-2.68332	-2.757604	0.7872291	0.7129455	0.7500873	up
A_21_P0010402	MIATNB	-1.817507	-0.945537	-1.189361	0.8719702	0.6281457	0.750058	up
A_22_P00001270	lnc-ANKRD53-1	4.987693	5.788301	5.686986	0.8006082	0.6992931	0.7499507	up
A_24_P45446	GBP4	0.1744599	0.6255469	1.2227097	0.451087	1.0482497	0.7496684	up
A_23_P75741	UBE2L6	2.6140223	3.070356	3.6556969	0.4563336	1.0416746	0.7490041	up
A_33_P3313519	CHRFAM7A	-3.224519	-2.544505	-2.406696	0.6800144	0.8178234	0.7489189	up
A_21_P0005720	NCRNA00249	-3.323614	-2.580101	-2.570554	0.7435131	0.7530601	0.7482866	up
A_33_P3775281	LINC01529	-1.39413	-0.573893	-0.718046	0.8202372	0.6760845	0.7481608	up
A_23_P23074	IFI44	1.9795017	2.7176757	2.7371254	0.738174	0.7576237	0.7478988	up
A_33_P3578325	SNORD15A	2.8499937	3.6776233	3.5175562	0.8276296	0.6675625	0.747596	up
A_33_P3348011	TOB2P1	-2.44187	-1.576109	-1.812958	0.8657613	0.6289125	0.7473369	up
A_33_P3370848	ETV2	-0.531048	0.2676911	0.1647782	0.7987394	0.6958265	0.747283	up
A_33_P3311267	KRTAP19-2	1.2632985	2.284346	1.7367616	1.0210476	0.4734631	0.7472553	up
A_21_P0000243	SNORD43	2.2799826	3.605248	2.4488935	1.3252654	0.168911	0.7470882	up
A_23_P90130	NAPSA	-2.075623	-1.514866	-1.142703	0.5607562	0.93292	0.7468381	up
A_32_P209230	CITED4	4.9719925	5.5975575	5.840008	0.6255651	0.8680153	0.7467902	up
A_22_P00004422	lnc-COPZ2-1	-2.63165	-1.67222	-2.097669	0.9594302	0.5339809	0.7467055	up
A_33_P3353051	C6orf48	2.238433	3.385282	2.5845232	1.1468492	0.3460903	0.7464698	up
A_21_P0013952	RBFOX3	1.5708342	2.4252515	2.209115	0.8544173	0.6382809	0.7463491	up
A_24_P270460	IFI27	8.027381	8.754587	8.792506	0.7272062	0.7651253	0.7461658	up
A_33_P3218625	CACFD1	4.7984543	5.59401	5.495117	0.7955556	0.6966629	0.7461093	up
A_23_P156687	CFB	-1.40868	-0.886315	-0.440233	0.5223651	0.9684472	0.7454062	up
A_24_P232790	CCDC177	1.2308283	2.1455588	1.8059001	0.9147306	0.5750718	0.7449012	up
A_22_P00020609	VPS13A-AS1	-1.370976	-0.703959	-0.54889	0.6670175	0.8220859	0.7445517	up
A_23_P26468	RHBDL1	3.3441162	4.0981975	4.0782347	0.7540813	0.7341185	0.7440999	up
A_33_P3363188	FLJ43315	-0.922657	0.8400426	-1.198218	1.7626996	-0.275561	0.7435691	up
A_23_P153571	IGFL2	6.608609	7.1834617	7.5200005	0.5748525	0.9113913	0.7431219	up
A_22_P00012507	LOC102724795	-3.269411	-2.292582	-2.760103	0.9768293	0.5093081	0.7430687	up
A_24_P149036	DPYSL3	0.1878462	1.2169919	0.6447482	1.0291457	0.456902	0.7430239	up
A_23_P111492	MOSPD3	-0.688208	0.2103953	-0.101218	0.898603	0.5869894	0.7427962	up
A_23_P140821	PARD6A	0.5128961	1.2218313	1.2892213	0.7089353	0.7763252	0.7426302	up
A_21_P0014400	DBNDD2	-3.057507	-2.34051	-2.289764	0.7169969	0.7677436	0.7423703	up
A_23_P207387	GHDC	-0.324719	0.339848	0.4943123	0.664567	0.8190312	0.7417991	up
A_23_P152420	GSE1	1.014359	1.8961186	1.615643	0.8817596	0.601284	0.7415218	up
A_23_P420196	SOCS1	-1.379078	-0.645151	-0.633796	0.7339273	0.7452817	0.7396045	up
A_22_P00007673	lnc-HIATL1-1	2.0490322	2.7745757	2.8023357	0.7255435	0.7533035	0.7394235	up
A_22_P00022561	FRY-AS1	-3.049309	-2.477908	-2.14199	0.5714006	0.9073188	0.7393597	up
A_33_P3349591	LOC100129461	-2.641311	-1.776678	-2.027907	0.8646331	0.6134033	0.7390182	up
A_23_P160025	IFI16	-0.554894	-0.321555	0.6880088	0.2333393	1.2429028	0.7381211	up
A_21_P0008433	lnc-MDGA2-2	-3.211926	-2.405361	-2.543647	0.8065651	0.6682792	0.7374221	up

A_33_P3288859	CDPF1	2.0129375	2.7804008	2.7202597	0.7674632	0.7073221	0.7373927	up
A_23_P139648	IAPP	-1.523908	-0.792576	-0.780907	0.7313314	0.7430005	0.7371659	up
A_33_P3227472	SDSL	4.92138	5.6727614	5.643839	0.7513814	0.7224588	0.7369201	up
A_33_P3224730	HMCN2	-3.113416	-2.414547	-2.338472	0.6988692	0.7749448	0.736907	up
A_23_P98252	ARL2	2.2791119	3.1638532	2.8675003	0.8847413	0.5883884	0.7365649	up
A_23_P104641	VPS51	4.0836277	5.0662303	4.5725327	0.9826026	0.488905	0.7357538	up
A_33_P3225086	PEX16	4.387101	5.150641	5.0944004	0.7635398	0.7072992	0.7354195	up
A_33_P3315779	HERC6	-0.466262	-0.130657	0.6689344	0.3356051	1.1351967	0.7354009	up
A_23_P50646	PINLYP	-2.755027	-1.953048	-2.086432	0.8019788	0.6685951	0.735287	up
A_23_P81973	HSD17B8	0.2071881	1.0323148	0.8524513	0.8251267	0.6452632	0.7351949	up
A_22_P00012919	NMNAT1	-2.736722	-1.924096	-2.079693	0.8126266	0.6570289	0.7348278	up
A_33_P3252794	GLI4	1.6834688	2.6509595	2.185522	0.9674907	0.5020533	0.734772	up
A_23_P4353	WSB1	2.5698538	3.3925357	3.2166166	0.8226819	0.6467629	0.7347224	up
A_32_P206899	DNAH2	0.8640494	1.6338887	1.5636144	0.7698393	0.6995649	0.7347021	up
A_33_P3214303	FOXP1	4.464217	5.4448447	4.952853	0.9806275	0.488636	0.7346318	up
A_23_P66050	B3GNT9	-1.466366	-0.716974	-0.746862	0.7493925	0.7195039	0.7344482	up
A_33_P3221971	GDPGP1	0.1376176	0.8537245	0.8902469	0.7161069	0.7526293	0.7343681	up
A_21_P0005689	LOC101929622	5.9387207	6.7990737	6.546912	0.860353	0.6081915	0.7342723	up
A_22_P00009029	SNHG7	4.0349617	5.0566688	4.4814453	1.021707	0.4464836	0.7340953	up
A_32_P460973	HLA-E	5.280616	6.0210004	6.0079784	0.7403846	0.7273626	0.7338736	up
A_33_P3275707	AP1S3	2.8141394	3.4029288	3.6924915	0.5887895	0.8783522	0.7335708	up
A_23_P62081	SCG5	-0.682277	-0.225667	0.3280888	0.4566093	1.0103655	0.7334874	up
A_23_P126593	S100A11	7.0500736	7.9026256	7.664464	0.8525519	0.6143904	0.7334712	up
A_22_P00003420	lnc-CCDC148-1	-3.255193	-2.469719	-2.574227	0.7854745	0.6809664	0.7332205	up
A_23_P325676	ZNF653	-0.744535	0.1162314	-0.138892	0.8607659	0.6056423	0.7332041	up
A_22_P00018112	LOC102724231	0.5576534	1.4954791	1.0861692	0.9378257	0.5285158	0.7331708	up
A_23_P31903	VPS28	6.4736595	7.3877892	7.0257835	0.9141297	0.552124	0.7331269	up
A_21_P0012616	XLOC_l2_010854	0.717134	1.5626378	1.3372188	0.8455038	0.6200848	0.7327943	up
A_22_P00022770	lnc-AF127577.1-4	-3.21238	-2.292487	-2.666747	0.919893	0.5456336	0.7327633	up
A_21_P0011674	XLOC_l2_006595	-3.264049	-2.460489	-2.602238	0.8035593	0.6618106	0.732685	up
A_23_P347541	GRIN3A	-1.769276	-1.110642	-0.962946	0.6586332	0.8063297	0.7324815	up
A_33_P3365142	GAD1	0.3026967	0.919466	1.1498351	0.6167693	0.8471384	0.7319539	up
A_22_P00000221	LOC102724366	-3.023639	-2.383599	-2.20128	0.6400402	0.8223593	0.7311997	up
A_33_P3304754	TPP2	-2.895789	-2.892753	-1.437893	0.0030353	1.4578953	0.7304653	up
A_23_P57868	ACY1	3.4925041	4.3310246	4.1142454	0.8385205	0.6217413	0.7301309	up
A_33_P3220911	BST2	6.8227844	7.36902	7.7367134	0.5462356	0.913929	0.7300823	up
A_21_P0000501	SNORD97	-0.887002	-0.025592	-0.288604	0.8614101	0.5983987	0.7299044	up
A_21_P0000222	SNORD46	1.4249854	2.2185988	2.0911512	0.7936134	0.6661658	0.7298896	up
A_22_P00011709	EMX2OS	-0.908204	-0.199347	-0.157623	0.7088566	0.7505803	0.7297185	up
A_23_P71530	TNFRSF11B	-2.209986	-1.964263	-0.996398	0.2457237	1.2135882	0.729656	up
A_23_P433798	PODNL1	-1.472445	-0.843271	-0.642557	0.6291738	0.8298879	0.7295308	up
A_24_P394533	NEU1	2.1618013	3.132504	2.64987	0.9707027	0.4880686	0.7293856	up
A_32_P55840	LINC01556	-2.268988	-1.367464	-1.712034	0.9015241	0.5569534	0.7292388	up
A_24_P274615	ARRDC3	-0.886845	0.0569544	-0.372253	0.9437995	0.5145917	0.7291956	up
A_33_P3400147	TLE6	-2.060815	-1.109674	-1.553815	0.9511414	0.507	0.7290707	up
A_33_P3329984	GGN	-0.114761	0.7512603	0.4767733	0.8660212	0.5915341	0.7287777	up
A_22_P00009154	C7orf13	-2.063372	-1.382479	-1.28697	0.6808929	0.776402	0.7286475	up
A_21_P0000357	SNORA47	-2.169673	-1.228094	-1.654325	0.9415789	0.515348	0.7284634	up
A_23_P371145	ADPRHL1	-2.856696	-1.826412	-2.430956	1.0302839	0.4257393	0.7280116	up
A_22_P00006229	LOC100507054	-1.272813	-0.460322	-0.629735	0.8124905	0.6430774	0.7277839	up
A_23_P340890	THAP3	1.5377059	2.4897513	2.0406666	0.9520454	0.5029607	0.7275031	up
A_33_P3283971	NFKBIL1	0.6095057	1.536097	1.1367149	0.9265914	0.5272093	0.7269004	up
A_23_P9875	TESK2	-2.07409	-1.343717	-1.350966	0.7303729	0.723124	0.7267485	up
A_24_P48495	LYPD3	2.3148918	3.2204728	2.8619232	0.905581	0.5470314	0.7263062	up
A_21_P0001518	lnc-PTGER3-2	-2.416819	-1.848243	-1.533361	0.5685763	0.8834577	0.726017	up
A_33_P3255509	FCHO1	3.2943573	4.0082326	4.0322123	0.7138753	0.737855	0.7258651	up

A_33_P3392517	LBX2	-1.089418	-0.377543	-0.350588	0.711874	0.7388291	0.7253516	up
A_33_P3292699	EPN1	-3.427074	-2.836734	-2.56703	0.5903399	0.8600435	0.7251917	up
A_33_P3292794	SMUG1	-0.041514	0.4993744	0.8672357	0.5408883	0.9087496	0.724819	up
A_23_P101480	MAST1	-1.599847	-0.888577	-0.862108	0.7112703	0.7377391	0.7245047	up
A_33_P3411397	SLC25A6	8.056904	8.945789	8.616927	0.8888855	0.5600233	0.7244544	up
A_21_P0007869	lnc-RP11-277P12.7.1-3	-3.457782	-2.693494	-2.773191	0.7642882	0.6845913	0.7244398	up
A_33_P3220643	PTRH1	3.5336008	4.344855	4.17091	0.811254	0.6373091	0.7242816	up
A_33_P3384835	LOC101927100	-1.834036	-1.059954	-1.16036	0.7740822	0.6736765	0.7238794	up
A_33_P3319640	HDGFRP2	0.8202028	1.5512986	1.5366211	0.7310958	0.7164183	0.723757	up
A_21_P0000369	SNORA16A	5.884613	6.623548	6.5931787	0.738935	0.7085657	0.7237504	up
A_23_P103011	RAB36	0.6199589	1.0586147	1.628788	0.4386559	1.0088291	0.7237425	up
A_23_P55998	SLC1A5	4.472003	5.2508454	5.140522	0.7788425	0.668519	0.7236807	up
A_19_P00319453	lnc-MMRN1-2	-2.848947	-1.935892	-2.315722	0.9130552	0.5332253	0.7231403	up
A_21_P0000274	SNORD95	-0.465209	0.6304588	-0.115631	1.0956674	0.3495774	0.7226224	up
A_23_P352535	PPP1R16B	1.2758889	2.1732688	1.8223085	0.8973799	0.5464196	0.7218998	up
A_23_P154784	BPIFB1	-2.239405	-1.394115	-1.641096	0.8452902	0.598309	0.7217996	up
A_23_P23048	S100A9	-3.369155	-2.903788	-2.391345	0.4653664	0.9778094	0.7215879	up
A_21_P0000322	SNORA34	3.739482	4.5428424	4.379163	0.8033605	0.6396809	0.7215207	up
A_23_P70398	VEGFA	1.1314964	2.295014	1.409801	1.1635175	0.2783046	0.720911	up
A_33_P3271725	CCDC33	-1.928176	-1.184011	-1.2311	0.7441654	0.6970763	0.7206209	up
A_22_P00020335	LOC100288181	-1.603918	-0.841164	-0.92577	0.762754	0.6781478	0.7204509	up
A_33_P3305758	CAPG	4.1150703	4.8421254	4.828723	0.7270551	0.7136526	0.7203539	up
A_33_P3290040	CPT1C	-1.598584	-0.892605	-0.864599	0.7059789	0.733985	0.7199819	up
A_33_P3393927	MTMR11	-1.810157	-1.003216	-1.178314	0.806941	0.6318431	0.7193921	up
A_23_P52067	GRHL3	2.1204433	2.8755145	2.8033476	0.7550712	0.6829042	0.7189877	up
A_33_P3359344	C1orf86	2.2094765	3.0949893	2.76161	0.8855128	0.5521336	0.7188232	up
A_33_P3386686	LOC100132874	0.6956463	1.6445813	1.1829877	0.948935	0.4873414	0.7181382	up
A_23_P132595	VGLL4	1.7892685	2.676433	2.336358	0.8871646	0.5470896	0.7171271	up
A_33_P3333480	ABHD11-AS1	-1.765581	-1.059953	-1.037037	0.7056279	0.7285438	0.7170858	up
A_21_P0000387	SNORD90	-3.125349	-2.589356	-2.22736	0.5359933	0.8979895	0.7169914	up
A_23_P65442	IRF9	2.2750149	2.89858	3.0844297	0.6235652	0.8094149	0.71649	up
A_23_P15516	TMEM101	2.0436869	2.9990573	2.5211601	0.9553704	0.4774733	0.7164218	up
A_21_P0010773	FLJ43315	-1.653288	-0.040039	-1.83417	1.6132493	-0.180882	0.7161839	up
A_33_P3342917	SYNGR2	1.413445	2.127584	2.131631	0.714139	0.7181859	0.7161625	up
A_22_P00012654	ZNF341-AS1	1.7949858	2.504414	2.5170498	0.7094283	0.722064	0.7157462	up
A_23_P75978	CLPB	1.9221058	2.7908902	2.483656	0.8687844	0.5615501	0.7151673	up
A_32_P123255	SOWAHD	-0.672523	-0.079465	0.1637125	0.5930581	0.8362355	0.7146468	up
A_33_P3248982	FAIM2	-2.368417	-1.780735	-1.527018	0.5876818	0.8413992	0.7145405	up
A_23_P120883	HMOX1	4.9234095	6.5420527	4.7327604	1.6186433	-0.190649	0.7139971	up
A_33_P3417626	ENHO	-1.383166	-1.051137	-0.287895	0.3320293	1.0952706	0.71365	up
A_23_P303101	PCDHGC4	-2.126285	-1.464881	-1.360529	0.6614046	0.7657561	0.7135804	up
A_23_P66813	SIRT7	3.443696	4.247275	4.0666943	0.8035789	0.6229982	0.7132885	up
A_23_P61280	COPG2	-0.471607	0.4093251	0.0736351	0.8809319	0.5452418	0.7130868	up
A_23_P153529	TRPM4	1.8380232	2.684198	2.4173498	0.8461747	0.5793266	0.7127507	up
A_19_P00317789	LOC93622	-2.676461	-2.216244	-1.711482	0.4602172	0.9649792	0.7125982	up
A_33_P3316683	SNX21	-2.856249	-2.196679	-2.090693	0.65957	0.7655563	0.7125632	up
A_33_P3323999	SBF1	6.082773	6.9492927	6.6412764	0.8665195	0.5585032	0.7125113	up
A_23_P208358	RPL28	8.824307	9.677267	9.395191	0.8529596	0.5708838	0.7119217	up
A_32_P66974	PCSK7	1.2897024	2.0783648	1.9245396	0.7886624	0.6348372	0.7117498	up
A_23_P128808	GPR132	-3.255402	-2.010362	-3.077434	1.2450402	0.1779676	0.7115039	up
A_23_P85218	SOX3	-1.800829	-1.111888	-1.066768	0.6889415	0.7340612	0.7115014	up
A_33_P3372044	TPRG1	-2.655149	-1.771878	-2.115522	0.883271	0.5396268	0.7114489	up
A_33_P3281616	CDPF1	1.8197508	2.6130176	2.4492855	0.7932668	0.6295347	0.7114008	up
A_33_P3335682	PPP1R14A	5.3665657	6.1859584	5.969784	0.8193927	0.6032181	0.7113054	up
A_22_P00010811	lnc-NMNAT1-3	-2.284587	-1.597233	-1.550032	0.6873546	0.7345557	0.7109551	up

A_33_P3246829	IL1RN	-0.973216	-0.429527	-0.095008	0.5436893	0.8782082	0.7109487	up
A_21_P0010068	lnc-HRH3-1	-1.813071	-1.050956	-1.153307	0.7621155	0.6597638	0.7109397	up
A_23_P116890	PRB3	0.6262822	1.4798088	1.194077	0.8535266	0.5677948	0.7106607	up
A_33_P3272948	C17orf107	-1.097914	-0.10734	-0.667468	0.9905744	0.4304466	0.7105105	up
A_23_P252808	WBP1	3.2479258	4.138787	3.7779236	0.890861	0.5299978	0.7104294	up
A_23_P159688	TBC1D25	-2.17054	-1.533423	-1.387183	0.6371169	0.7833571	0.710237	up
A_23_P351757	PLCD3	1.1558065	2.0241532	1.707264	0.8683467	0.5514574	0.7099021	up
A_22_P00006422	lnc-FBXO31-1	-3.227893	-2.689071	-2.347085	0.5388219	0.8808074	0.7098147	up
A_23_P110802	CENPH	1.3950562	2.1625085	2.0471897	0.7674522	0.6521335	0.7097929	up
A_23_P14948	MBTPS1	3.0922194	3.897039	3.7060862	0.8048196	0.6138668	0.7093432	up
A_33_P3390637	SLC23A3	-2.721507	-2.081415	-1.943868	0.6400917	0.7776387	0.7088652	up
A_21_P0009321	LOC101929494	-2.905908	-1.986338	-2.407841	0.9195695	0.4980669	0.7088182	up
A_24_P307289	TMEM95	1.9710283	2.704701	2.653657	0.7336726	0.6826286	0.7081506	up
A_23_P54636	ATP6V0D1	4.0986357	4.849914	4.7631187	0.7512784	0.6644831	0.7078808	up
A_23_P331670	PYGB	4.23382	5.0808816	4.802127	0.8470616	0.5683069	0.7076843	up
A_22_P00003850	LINC01272	-1.206394	-0.134032	-0.864506	1.072362	0.3418884	0.7071252	up
A_33_P3338631	NRL	-3.488788	-2.499829	-3.063641	0.9889596	0.4251475	0.7070535	up
A_22_P00009473	PSMG3-AS1	5.745922	6.6042695	6.301038	0.8583474	0.5551157	0.7067316	up
A_22_P00013904	LINC00856	-0.442607	0.085856	0.442297	0.5284629	0.8849039	0.7066834	up
A_24_P259276	ZDHHC24	2.1925058	3.0135093	2.7843094	0.8210034	0.5918036	0.7064035	up
A_33_P3270445	BAI2	-2.367177	-1.830551	-1.492163	0.5366254	0.8750138	0.7058196	up
A_23_P203115	TMEM25	0.0240092	0.664896	0.7941713	0.6408868	0.7701621	0.7055245	up
A_33_P3411925	WDR18	5.9964046	6.8196673	6.5837584	0.8232627	0.5873537	0.7053082	up
A_21_P0011899	XLOC_l2_007876	-3.382367	-2.527441	-2.827066	0.8549268	0.555301	0.7051139	up
A_23_P253052	CD99L2	2.8575077	3.6881766	3.43651	0.8306689	0.5790024	0.7048357	up
A_23_P112103	GSDMD	1.362678	1.9948583	2.139224	0.6321802	0.776546	0.7043631	up
A_24_P280833	LOC100129138	-0.221166	0.7260656	0.2386131	0.9472313	0.4597788	0.7035051	up
A_23_P360316	FUT3	3.1866856	4.1197944	3.6601353	0.9331088	0.4734497	0.7032793	up
A_33_P3702104	MGC34796	-2.026365	-1.350958	-1.295591	0.6754074	0.7307744	0.7030909	up
A_23_P206022	ITGA11	-2.68531	-2.133768	-1.831199	0.5515416	0.8541105	0.702826	up
A_33_P3377750	KLC3	3.2302628	3.9867082	3.8789186	0.7564454	0.6486559	0.7025507	up
A_24_P326708	BRSK2	-1.262044	-0.56757	-0.551692	0.6944747	0.7103524	0.7024136	up
A_33_P3369016	LOC101060085	-2.126353	-1.369671	-1.47852	0.7566814	0.6478329	0.7022572	up
A_24_P5743	ALDH16A1	2.9180155	3.6301627	3.6100216	0.7121472	0.6920061	0.7020767	up
A_23_P74114	ZNF713	3.5052853	4.2420955	4.172574	0.7368102	0.6672888	0.7020495	up
A_21_P0006240	lnc-OLFM1-1	-3.270294	-2.577786	-2.55934	0.692508	0.710954	0.701731	up
A_23_P71379	PSCA	3.3174934	3.9815984	4.0562572	0.6641049	0.7387638	0.7014344	up
A_22_P00006956	TFAP2A-AS1	-3.172498	-2.245722	-2.696875	0.9267762	0.4756227	0.7011994	up
A_33_P3392391	CPT1C	-3.193725	-2.723784	-2.261305	0.4699407	0.93242	0.7011803	up
A_33_P3375368	GPR35	2.8337908	3.6146235	3.4542646	0.7808328	0.6204739	0.7006533	up
A_23_P366682	TPGS1	-0.388426	0.4907312	0.1336975	0.8791571	0.5221233	0.7006402	up
A_23_P74950	RCC2	5.3742046	6.155931	5.9935246	0.7817264	0.6193199	0.7005231	up
A_23_P135257	PRSS3	2.338129	3.1337218	2.9431	0.7955928	0.6049709	0.7002819	up
A_33_P3271395	LOC100129534	-1.257846	-0.489849	-0.625371	0.7679977	0.6324749	0.7002363	up
A_33_P3405995	YBEY	-1.798934	-1.178806	-1.018625	0.6201277	0.7803092	0.7002185	up
A_33_P3294654	RSG1	-2.446019	-1.839129	-1.652714	0.6068902	0.7933054	0.7000978	up
A_23_P46039	FCRLA	-3.046778	-2.510417	-2.184614	0.5363607	0.8621635	0.6992621	up
A_32_P52153	UNC5B-AS1	3.5640955	4.3734803	4.1530857	0.8093848	0.5889902	0.6991875	up
A_32_P223017	MAP3K6	1.4778075	1.9804206	2.3725395	0.5026131	0.894732	0.6986725	up
A_22_P00025928	lnc-RIC3-1	-3.024779	-2.355968	-2.296684	0.6688111	0.7280958	0.6984534	up
A_22_P00002230	TMEM26-AS1	2.3033085	3.206657	2.7954254	0.9033485	0.4921169	0.6977327	up
A_23_P64611	P2RY6	0.0242348	0.4323883	1.01054	0.4081535	0.9863052	0.6972294	up
A_23_P54223	RABGGTA	-0.957365	-0.215795	-0.306114	0.74157	0.6512513	0.6964107	up
A_24_P152649	LOC644189	0.6131744	1.4835749	1.135179	0.8704004	0.5220046	0.6962025	up
A_23_P16058	ZNF296	-0.223062	0.566031	0.3795967	0.7890925	0.6026583	0.6958754	up
A_23_P325726	ACOT11	-1.25772	-0.464464	-0.659602	0.7932553	0.5981178	0.6956866	up

A_23_P250982	ISOC1	3.5982962	4.164389	4.4233665	0.566093	0.8250704	0.6955817	up
A_22_P00015194	LOC100996842	0.2184882	0.9460139	0.8815579	0.7275257	0.6630697	0.6952977	up
A_33_P3362616	PRR3	-2.29559	-1.676659	-1.524321	0.6189308	0.7712688	0.6950998	up
A_23_P15727	FKBP10	3.9593916	4.4255214	4.882802	0.4661298	0.9234104	0.6947701	up
A_24_P100761	BCAS3	-0.111812	0.7387128	0.4271789	0.8505244	0.5389905	0.6947575	up
A_33_P3227716	GATSL3	3.6700087	4.09679	4.631073	0.4267812	0.9610643	0.6939228	up
A_23_P37870	STUB1	4.772188	5.592857	5.3389034	0.8206687	0.5667152	0.693692	up
A_32_P38637	KRBA1	-1.637447	-0.989373	-0.898249	0.6480742	0.7391982	0.6936362	up
A_23_P90463	LSM7	5.658579	6.3591504	6.345108	0.7005715	0.6865292	0.6935504	up
A_21_P0012250	TMEM191B	0.8255258	1.4553461	1.5823398	0.6298204	0.756814	0.6933172	up
A_32_P193288	RPL18A	8.484846	9.297451	9.058737	0.8126049	0.5738907	0.6932478	up
A_24_P105298	H3F3A	8.78908	9.544575	9.419538	0.7554951	0.6304579	0.6929765	up
A_23_P117225	ERCC5	2.450945	3.0945096	3.1921473	0.6435647	0.7412024	0.6923835	up
A_33_P3303015	RPS9	8.377151	9.263939	8.874812	0.8867884	0.4976616	0.692225	up
A_22_P00000195	lnc-ABI3-1	-3.029153	-2.31668	-2.357338	0.7124734	0.6718152	0.6921443	up
A_23_P250462	ATP6AP1	5.3368235	6.227053	5.8307867	0.8902297	0.4939632	0.6920965	up
A_33_P3306177	TAPT1	-1.066549	-0.492605	-0.256718	0.5739436	0.8098311	0.6918874	up
A_19_P00321722	lnc-CCDC71L-1	3.3039885	4.0485682	3.9411545	0.7445798	0.637166	0.6908729	up
A_33_P3384543	POMGNT1	3.8848772	4.540294	4.6104927	0.655417	0.7256155	0.6905162	up
A_33_P3308903	MIB2	2.3327827	3.199738	2.8468084	0.8669553	0.5140257	0.6904905	up
A_33_P3215929	PRR5	0.4849434	1.3333678	1.0163684	0.8484244	0.531425	0.6899247	up
A_21_P0011566	XLOC_l2_005952	3.9204597	4.762094	4.4584675	0.8416343	0.5380077	0.689821	up
A_19_P00315581	LINC01122	4.6396227	5.3959002	5.262907	0.7562776	0.6232843	0.689781	up
A_32_P231179	TEKT4	-0.860137	-0.180544	-0.160913	0.6795931	0.699224	0.6894086	up
A_23_P216282	ARHGEF10	-1.148587	-0.369905	-0.548621	0.7786813	0.5999656	0.6893235	up
A_23_P407614	PYDC1	0.6951613	1.3204312	1.4484463	0.6252699	0.7532849	0.6892774	up
A_33_P3495962	SNORA71A	1.7587204	2.5540614	2.3414326	0.795341	0.5827122	0.6890266	up
A_33_P3383004	PSAPL1	-1.828015	-1.726889	-0.551463	0.1011262	1.2765527	0.6888394	up
A_33_P3422822	GJC2	3.498042	4.203422	4.169503	0.70538	0.6714611	0.6884205	up
A_33_P3219591	RNF213	0.0078883	0.6303644	0.7615695	0.6224761	0.7536812	0.6880787	up
A_33_P3420446	LRRD1	2.7330408	3.5921216	3.2492666	0.8590808	0.5162258	0.6876533	up
A_33_P3227920	SLC16A4	2.5737667	3.7797942	2.7430077	1.2060275	0.169241	0.6876342	up
A_23_P120467	ZFP64	1.647582	2.374248	2.2958336	0.726666	0.6482515	0.6874588	up
A_22_P00001218	lnc-ANKRD11-2	-0.704998	0.1634827	-0.19888	0.8684807	0.5061183	0.6872995	up
A_23_P101551	BCAT2	3.522893	4.227987	4.1919956	0.7050939	0.6691027	0.6870983	up
A_23_P357760	ARSD	-1.540648	-0.875021	-0.83218	0.6656265	0.708468	0.6870472	up
A_23_P68240	GPAT2	-0.933326	-0.295969	-0.197673	0.6373572	0.7356525	0.6865048	up
A_23_P205177	F10	-2.713084	-1.979787	-2.073504	0.7332962	0.6395791	0.6864376	up
A_22_P00002634	lnc-C1orf177-1	0.9343343	1.7694554	1.4718499	0.8351212	0.5375156	0.6863184	up
A_33_P3283669	ATP1A3	0.2657056	1.0540681	0.8494983	0.7883625	0.5837927	0.6860776	up
A_23_P365738	ARC	0.101778	0.9264488	0.6481266	0.8246708	0.5463486	0.6855097	up
A_22_P00012772	lnc-RAI1-1	-2.496137	-1.675824	-1.946009	0.8203135	0.5501285	0.685221	up
A_21_P0000391	SNORD93	-3.296234	-2.756032	-2.46642	0.5402021	0.829814	0.6850081	up
A_24_P296508	SLC43A2	0.3520303	1.3137426	0.7597261	0.9617124	0.4076958	0.6847041	up
A_23_P160546	FAM63A	3.3164158	4.042414	3.9592533	0.7259984	0.6428375	0.684418	up
A_33_P3286278	GRN	7.374406	8.22719	7.888834	0.8527842	0.5144281	0.6836062	up
A_21_P0012868	LOC100128340	-1.469416	-1.02174	-0.55004	0.4476767	0.9193764	0.6835265	up
A_21_P0006169	LOC100506834	-2.964957	-2.259805	-2.303833	0.7051525	0.6611242	0.6831384	up
A_24_P402588	BCL11A	-0.686218	0.2142386	-0.220689	0.9004569	0.465529	0.6829929	up
A_24_P87931	APOL1	1.5122447	2.1971936	2.1929417	0.6849489	0.680697	0.6828229	up
A_24_P280497	FBRSL1	5.39546	6.1883245	5.967682	0.7928643	0.5722218	0.682543	up
A_24_P51683	CDK5R2	-1.103305	-0.393416	-0.448399	0.7098894	0.6549068	0.6823981	up
A_21_P0009377	lnc-NLGN2-1	5.113559	5.884562	5.7063	0.7710033	0.592741	0.6818721	up
A_23_P94591	TMEM141	5.903922	6.5772176	6.5940294	0.6732955	0.6901074	0.6817014	up
A_33_P3375314	ATP9A	1.3625145	2.3103251	1.7778316	0.9478107	0.4153171	0.6815639	up
A_23_P79794	TGIF2	-2.275234	-1.606581	-1.580978	0.6686525	0.6942554	0.6814539	up

A_23_P301372	TAPT1	0.0647721	0.6797376	0.8119426	0.6149654	0.7471705	0.6810679	up
A_23_P142013	XAB2	-1.104558	-0.406526	-0.44076	0.6980314	0.6637974	0.6809144	up
A_33_P3343106	ETV2	1.8664503	2.5381322	2.5565443	0.6716819	0.690094	0.680888	up
A_23_P303455	GPR161	-2.082117	-1.574534	-1.228957	0.5075836	0.8531599	0.6803718	up
A_24_P292470	UCP3	4.5252914	5.323437	5.0875196	0.7981458	0.5622282	0.680187	up
A_23_P501713	IL1F10	-3.047254	-2.279988	-2.454254	0.767266	0.5929997	0.6801329	up
A_33_P3290162	ASB18	-2.299229	-1.390472	-1.847773	0.9087572	0.4514561	0.6801066	up
A_19_P00807670	SOCS2-AS1	-2.267483	-1.868543	-1.306607	0.3989401	0.960876	0.6799081	up
A_32_P183970	C15orf62	0.7288175	1.3639283	1.4534702	0.6351109	0.7246528	0.6798818	up
A_33_P3270628	lnc-EYA3-1	-3.224865	-2.369956	-2.720454	0.8549092	0.5044115	0.6796603	up
A_33_P3364989	ACTL10	-0.275236	0.3916893	0.417038	0.666925	0.6922736	0.6795993	up
A_33_P3312365	LONP1	1.9375248	2.855146	2.379035	0.9176211	0.4415102	0.6795657	up
A_23_P98605	UBXN1	6.6786003	7.500834	7.215131	0.8222337	0.5365305	0.6793821	up
A_22_P00006710	HGC6.3	2.7582226	3.5832458	3.291957	0.8250232	0.5337343	0.6793788	up
A_23_P325562	SLC1A7	-2.369716	-1.464547	-1.916383	0.9051695	0.4533334	0.6792514	up
A_33_P3278313	MSRB3	-0.562354	-0.046215	0.2795944	0.5161386	0.841948	0.6790433	up
A_23_P424734	EXOC3L1	-1.681967	-0.888216	-1.117931	0.7937517	0.5640364	0.678894	up
A_33_P3293768	KRTAP10-6	-3.107966	-2.391519	-2.46696	0.7164469	0.6410055	0.6787262	up
A_23_P35444	INA	-3.134154	-2.429805	-2.481156	0.7043486	0.652998	0.6786733	up
A_22_P00011325	lnc-OST4-2	4.404229	5.193764	4.9719315	0.7895351	0.5677023	0.6786187	up
A_23_P121885	ROPN1L	-2.400247	-1.649337	-1.794061	0.7509098	0.6061854	0.6785476	up
A_23_P55356	VMO1	-3.11957	-2.315568	-2.566555	0.8040025	0.5530148	0.6785086	up
A_23_P119042	NKG7	-3.001466	-2.739054	-1.908187	0.2624116	1.0932791	0.6778454	up
A_23_P369479	MSI2	-0.483481	0.3810124	0.0075336	0.8644939	0.491015	0.6777544	up
A_24_P202567	ITPKC	4.030673	4.784265	4.6319017	0.753592	0.6012287	0.6774104	up
A_33_P6579294	ELP2	0.0831499	0.8727226	0.6482034	0.7895727	0.5650535	0.6773131	up
A_22_P00023623	CTB-178M22.2	-1.940127	-1.273282	-1.253928	0.6668458	0.6861992	0.6765225	up
A_24_P260443	THBS4	-2.709185	-1.780724	-2.284612	0.9284606	0.4245722	0.6765164	up
A_33_P3295148	GLUD1P3	6.2605715	7.035851	6.83823	0.7752795	0.5776587	0.6764691	up
A_22_P00023791	lnc-PCDH12-1	-2.598918	-1.653449	-2.191809	0.9454689	0.4071093	0.6762891	up
A_33_P3345534	KRT14	8.4618435	9.265234	9.010448	0.8033905	0.548605	0.6759977	up
A_33_P3299634	OR1L6	-2.511741	-1.71498	-1.95711	0.7967613	0.554631	0.6756962	up
A_23_P417415	ACOT11	3.269371	3.8550143	4.034913	0.5856433	0.765542	0.6755927	up
A_22_P00008960	LINC00520	-0.989063	0.0476408	-0.674763	1.0367041	0.3143001	0.6755021	up
A_32_P217655	LOC645166	3.5160198	4.2920814	4.090597	0.7760615	0.5745773	0.6753194	up
A_33_P3214803	PRDM13	-0.881937	-0.215372	-0.197908	0.6665654	0.6840286	0.675297	up
A_33_P3393941	MBTPS1	3.1566944	3.9006371	3.7625942	0.7439427	0.6058998	0.6749213	up
A_33_P3257861	SARDH	-0.200313	0.2944379	0.6547442	0.4947505	0.8550568	0.6749036	up
A_33_P3246833	IL1RN	5.037141	5.6778855	5.745226	0.6407447	0.7080851	0.6744149	up
A_23_P166051	RBCK1	1.7255259	2.5925994	2.2071056	0.8670735	0.4815798	0.6743267	up
A_33_P3228573	ASPSCR1	3.67595	4.324269	4.376066	0.6483188	0.7001162	0.6742175	up
A_23_P26649	NMRAL1	2.8850842	3.749555	3.3689508	0.864471	0.4838667	0.6741688	up
A_23_P161439	ADIRF	8.165746	8.9199295	8.759605	0.7541838	0.5938597	0.6740217	up
A_23_P64214	PCSK7	-0.728324	0.0860462	-0.194669	0.8143706	0.5336556	0.6740131	up
A_23_P101054	KRT34	-0.000671	0.8021421	0.5444312	0.8028135	0.5451026	0.6739581	up
A_23_P26629	PYCARD	4.721532	5.3516965	5.4390936	0.6301646	0.7175617	0.6738632	up
A_23_P90484	SARS2	2.273056	2.9987044	2.8945808	0.7256484	0.6215248	0.6735866	up
A_24_P804667	METTL12	0.7139101	1.5465555	1.2277479	0.8326454	0.5138378	0.6732416	up
A_33_P3302165	ABHD8	3.9316454	4.877023	4.3323765	0.9453778	0.4007311	0.6730545	up
A_22_P00023281	SIRPG-AS1	-2.565209	-1.817627	-1.967165	0.7475824	0.5980444	0.6728134	up
A_21_P0006439	lnc-SLC25A6-1	-1.771396	-0.984942	-1.212379	0.7864537	0.5590167	0.6727352	up
A_22_P00001773	LOC101929023	-3.360949	-2.6982	-2.678544	0.6627488	0.6824055	0.6725772	up
A_33_P3382999	PSAPL1	0.060194	0.4345956	1.0308905	0.3744016	0.9706965	0.672549	up
A_21_P0006516	LINC00629	-0.839644	-0.075502	-0.258942	0.7641411	0.5807014	0.6724212	up
A_22_P00013630	lnc-RP11-625H11.1.1-2	-3.322322	-2.850849	-2.448987	0.4714737	0.8733351	0.6724044	up

A_23_P300484	OBSL1	0.6360016	1.4914193	1.1246815	0.8554177	0.4886799	0.6720488	up
A_21_P0006231	lnc-METTL11A-5	-2.604385	-1.848606	-2.016258	0.7557793	0.5881271	0.6719532	up
A_24_P122746	VWA1	-0.736994	-0.227044	0.0969524	0.5099506	0.8339467	0.6719487	up
A_22_P00004142	lnc-CLDN6-2	1.0965462	1.7939429	1.7429948	0.6973968	0.6464486	0.6719227	up
A_33_P3384108	SLC19A1	0.8955379	1.5841799	1.5504746	0.688642	0.6549368	0.6717894	up
A_33_P3290888	CPNE1	3.378746	4.341885	3.7589998	0.9631391	0.3802538	0.6716964	up
A_22_P00024452	lnc-AKT1-2	-3.581491	-2.943934	-2.876514	0.637557	0.7049766	0.6712668	up
A_22_P00008521	lnc-KCNJ12-1	-0.071768	0.7649045	0.4338155	0.8366723	0.5055833	0.6711278	up
A_21_P0011663	GTSCR1	-2.775206	-2.146101	-2.062073	0.6291049	0.7131329	0.6711189	up
A_23_P167983	HIST1H2AC	0.6862245	1.7222166	0.9920869	1.0359921	0.3058624	0.6709273	up
A_33_P3266550	ABO	-0.150908	0.6132484	0.4261937	0.7641568	0.5771022	0.6706295	up
A_21_P0014072	LOC100507670	-2.167775	-1.25283	-1.741641	0.9149456	0.4261346	0.6705401	up
A_33_P3291176	VMAC	-2.032967	-1.240543	-1.484599	0.7924237	0.5483675	0.6703956	up
A_22_P00016771	TRIM31-AS1	0.44911	1.076436	1.161582	0.627326	0.712472	0.669899	up
A_32_P134290	ZCCHC2	0.6087608	1.1575665	1.3995023	0.5488057	0.7907414	0.6697736	up
A_33_P3302428	TNRC6C	2.5626936	3.342154	3.1205177	0.7794604	0.5578241	0.6686423	up
A_33_P3337134	ABLIM2	-3.544847	-2.575837	-3.177662	0.9690101	0.3671844	0.6680973	up
A_33_P3309501	MEG3	-0.604795	0.0449309	0.0805445	0.6497254	0.685339	0.6675322	up
A_23_P38952	ACER1	-2.883521	-2.314858	-2.117663	0.5686631	0.7658584	0.6672608	up
A_19_P00320872	lnc-KIAA0020-1	-3.402637	-2.909169	-2.562587	0.4934676	0.84005	0.6667588	up
A_33_P3404843	SUGP1	-1.582057	-0.740467	-1.090832	0.8415904	0.4912248	0.6664076	up
A_33_P3818959	SAMD11	1.2334623	2.2243295	1.5753207	0.9908671	0.3418584	0.6663628	up
A_24_P142495	KRTAP1-3	4.1996307	5.0970254	4.6343203	0.8973947	0.4346895	0.6660421	up
A_23_P54469	ISL2	-2.647402	-1.983919	-1.978977	0.6634836	0.6684256	0.6659546	up
A_32_P85330	ST20-AS1	-2.462522	-1.777291	-1.817396	0.6852315	0.6451261	0.6651788	up
A_21_P0002352	lnc-AC007557.1.1-3	-2.711578	-2.043737	-2.049392	0.6678405	0.6621862	0.6650133	up
A_24_P343621	ECHDC3	-1.503884	-0.67508	-1.003098	0.8288045	0.5007863	0.6647954	up
A_23_P27571	MCOLN1	0.0608368	0.7640419	0.686872	0.7032051	0.6260352	0.6646202	up
A_21_P0006193	lnc-STX17-1	-0.513885	0.3273878	-0.02618	0.8412728	0.4877048	0.6644888	up
A_33_P3318444	RNF222	3.7520418	4.534718	4.2976284	0.7826762	0.5455866	0.6641314	up
A_23_P354208	WDR81	2.635827	3.404077	3.195033	0.76825	0.559206	0.663728	up
A_23_P64785	ZNF641	-0.413876	0.4098959	0.0897331	0.8237715	0.5036087	0.6636901	up
A_23_P45560	GPR143	-3.182646	-2.492045	-2.546308	0.6906009	0.6363387	0.6634698	up
A_22_P00011393	PPIEL	1.6400833	2.3059554	2.300726	0.6658721	0.6606426	0.6632574	up
A_33_P3239759	PPAN-P2RY11	-0.953911	-0.240791	-0.340873	0.71312	0.6130385	0.6630793	up
A_23_P122375	ZFAND3	-1.867909	-1.170315	-1.239418	0.6975942	0.6284919	0.663043	up
A_33_P3417195	C17orf82	0.2803769	0.8766608	1.0097194	0.5962839	0.7293425	0.6628132	up
A_21_P0000291	SNORD41	-2.437714	-1.682611	-1.867204	0.7551031	0.5705094	0.6628063	up
A_23_P149858	ELOVL3	-3.019281	-2.157417	-2.556951	0.8618639	0.4623301	0.662097	up
A_23_P59452	AOC1	2.1837273	3.0992818	2.5919085	0.9155545	0.4081812	0.6618679	up
A_33_P3350202	MOCS3	3.7050848	4.476937	4.2568903	0.771852	0.5518055	0.6618288	up
A_23_P121533	SPON2	6.43242	7.1067004	7.081563	0.6742806	0.6491432	0.6617119	up
A_24_P390668	FMNL1	-3.025215	-2.254868	-2.472187	0.7703466	0.5530279	0.6616873	up
A_23_P80739	PLCD1	1.3657875	2.2398582	1.8148856	0.8740706	0.4490981	0.6615844	up
A_33_P3224020	ARAFP2	6.513837	7.2033687	7.146942	0.6895318	0.6331053	0.6613186	up
A_23_P82249	ABCB8	-1.984053	-1.171433	-1.47433	0.8126202	0.5097232	0.6611717	up
A_33_P3377380	lnc-PABPC4-2	-0.616324	0.1856594	-0.096139	0.8019834	0.5201845	0.6610839	up
A_22_P00016058	LOC100506047	-1.862583	-1.218983	-1.184754	0.6436	0.6778283	0.6607142	up
A_22_P00024468	lnc-CTNNA2-1	2.6463337	3.282536	3.331112	0.6362023	0.6847782	0.6604903	up
A_19_P00321734	LINC00881	-2.273398	-1.431914	-1.794675	0.8414841	0.4787231	0.6601036	up
A_24_P263937	CCDC23	1.4673276	2.3530946	1.9015408	0.885767	0.4342132	0.6599901	up
A_23_P379649	BMF	-1.229234	-0.116776	-1.022059	1.1124582	0.2071757	0.659817	up
A_24_P335305	OAS3	1.1815124	1.5759659	2.1065187	0.3944535	0.9250064	0.65973	up
A_33_P3602006	ADAT2	6.4698286	7.197724	7.060791	0.7278953	0.5909624	0.6594288	up
A_21_P0000252	SNORD68	2.0462313	2.913487	2.497713	0.8672557	0.4514818	0.6593688	up

A_21_P0005606	lnc-SBDS-10	-0.997834	-0.333492	-0.343547	0.6643419	0.6542869	0.6593144	up
A_23_P61202	FAM207A	2.5467577	3.3368773	3.0751514	0.7901197	0.5283938	0.6592567	up
A_21_P0006253	lnc-PTPRD-3	-2.823218	-2.194036	-2.134088	0.6291821	0.6891305	0.6591563	up
A_23_P51187	PRKCZ	4.775076	5.4621425	5.4060907	0.6870666	0.6310148	0.6590407	up
A_22_P00025249	lnc-CEACAM18-2	-0.456231	0.2339768	0.1711297	0.6902075	0.6273603	0.6587839	up
A_24_P346431	TNS3	3.4863796	4.232796	4.0573997	0.7464166	0.5710201	0.6587183	up
A_33_P3491294	AMDHD2	1.8702574	2.7444034	2.3128376	0.874146	0.4425802	0.6583631	up
A_22_P00013092	lnc-RIC3-1	-2.272826	-1.734805	-1.494348	0.5380211	0.7784777	0.6582494	up
A_23_P377214	HEXIM2	3.560874	4.4715047	3.9664793	0.9106307	0.4056053	0.658118	up
A_23_P129413	DPEP3	2.6301384	3.4263453	3.150157	0.796207	0.5200186	0.6581128	up
A_33_P3360540	AGPAT2	7.7409697	8.524315	8.273781	0.7833452	0.5328112	0.6580782	up
A_33_P3338724	LENG9	1.6077766	2.3097177	2.2217522	0.701941	0.6139755	0.6579583	up
A_33_P3308446	RHOB	2.459319	3.1904788	3.0440035	0.7311597	0.5846844	0.6579221	up
A_33_P3291510	VCY	-3.09797	-2.525797	-2.354606	0.5721726	0.7433641	0.6577684	up
A_21_P0009285	lnc-C1QTNF1-1	-1.632718	-0.936744	-1.013794	0.6959734	0.6189241	0.6574488	up
A_21_P0013246	UPK3B	1.5487967	2.3352656	2.076685	0.786469	0.5278883	0.6571787	up
A_33_P3397865	TNNT1	5.5293436	6.0874352	6.2845507	0.5580916	0.7552071	0.6566494	up
A_22_P00012687	LOC100507156	1.7360468	2.5381055	2.246993	0.8020587	0.5109463	0.6565025	up
A_21_P0011753	XLOC_l2_007063	-0.747188	-0.154998	-0.026547	0.5921898	0.7206411	0.6564155	up
A_33_P3227691	SLC9A5	-2.814124	-2.559648	-1.755933	0.2544768	1.0581915	0.6563342	up
A_23_P59976	GML	-3.349472	-2.676874	-2.709674	0.6725984	0.6397979	0.6561981	up
A_24_P410582	VGLL4	0.8929577	1.75595	1.3414345	0.8629923	0.4484768	0.6557346	up
A_23_P78557	FBXL12	-1.422467	-0.631166	-0.902495	0.7913003	0.5199714	0.6556359	up
A_21_P0013259	GS1-259H13.2	-2.906287	-1.83703	-2.664539	1.0692561	0.2417471	0.6555016	up
A_21_P0009315	lnc-SLC35G3-1	-1.175283	-0.437632	-0.602129	0.7376514	0.573154	0.6554027	up
A_33_P3304878	WDFY4	-2.860679	-2.235123	-2.175621	0.6255562	0.6850584	0.6553073	up
A_33_P3295650	APBA1	-0.067723	0.5273166	0.6477537	0.5950394	0.7154765	0.6552579	up
A_33_P3222380	AHNAK2	2.418683	3.2647367	2.8829145	0.8460536	0.4642315	0.6551426	up
A_23_P117037	LETMD1	1.812624	2.7700696	2.1645517	0.9574456	0.3519278	0.6546867	up
A_21_P0008040	lnc-GSX1-1	-1.940809	-1.044556	-1.527723	0.8962536	0.4130864	0.65467	up
A_33_P3348884	CCDC141	-3.094942	-2.34285	-2.537954	0.7520917	0.5569882	0.6545399	up
A_33_P3328736	CCDC23	2.3873596	3.254784	2.8289576	0.8674245	0.4415979	0.6545112	up
A_23_P150857	SUOX	-0.991096	-0.43575	-0.237786	0.555346	0.7533102	0.6543281	up
A_22_P00019938	LOC101927346	-1.69228	-1.038776	-1.038425	0.6535044	0.6538553	0.6536799	up
A_23_P153383	RPL36	7.6058693	8.296169	8.2226925	0.6903	0.6168232	0.6535616	up
A_23_P251717	WDR45	2.9562464	3.9253888	3.2940083	0.9691424	0.3377619	0.6534522	up
A_24_P72364	C6orf15	0.8189588	1.5264239	1.4182343	0.7074652	0.5992756	0.6533704	up
A_22_P00006389	LOC100130238	-0.046137	0.6153445	0.5990343	0.6614814	0.6451712	0.6533263	up
A_33_P3277965	NFIC	-3.354414	-2.801136	-2.602085	0.553278	0.7523286	0.6528033	up
A_23_P259797	ACSF3	0.5822864	1.3244672	1.1456075	0.7421808	0.5633211	0.652751	up
A_23_P69383	PARP9	2.1715813	2.617888	3.030734	0.4463067	0.8591528	0.6527298	up
A_33_P3282669	POLL	-0.315295	0.4925737	0.1816726	0.807869	0.4969678	0.6524184	up
A_22_P00000388	LINC01258	1.7138767	2.3745284	2.3579035	0.6606517	0.6440268	0.6523392	up
A_33_P3284197	POMGNT1	4.005354	4.627479	4.6876335	0.6221252	0.6822796	0.6522024	up
A_24_P7085	RPL29P2	4.9980173	5.74528	5.5550737	0.7472625	0.5570564	0.6521595	up
A_23_P39223	ZNF2	1.2267418	1.9629025	1.7947707	0.7361608	0.5680289	0.6520948	up
A_23_P171296	MPP1	1.364491	1.8829169	2.1501942	0.5184259	0.7857032	0.6520646	up
A_21_P0005916	lnc-TSNARE1-1	-2.804599	-2.272752	-2.033086	0.5318468	0.7715128	0.6516798	up
A_33_P3258593	PRB1	0.9153457	1.6768847	1.4571128	0.761539	0.5417671	0.6516531	up
A_33_P3293573	VPS37D	1.6277084	2.3420281	2.216405	0.7143197	0.5886965	0.6515081	up
A_33_P3461633	LOC284454	1.1027889	1.7901793	1.7181625	0.6873903	0.6153736	0.651382	up
A_33_P3267081	ZNF771	1.9966211	2.8164716	2.4790182	0.8198504	0.4823971	0.6511238	up
A_23_P145863	S100A11	7.3514204	8.149649	7.8550797	0.7982283	0.5036593	0.6509438	up
A_22_P00013536	lnc-RP11-422N16.3.1-1	0.6472464	1.4401798	1.1559057	0.7929335	0.5086594	0.6507964	up
A_23_P49768	MRPL27	4.349704	4.947949	5.052947	0.5982451	0.7032433	0.6507442	up

A_33_P3277883	LOC100129931	-0.905832	-0.013762	-0.496594	0.8920698	0.4092379	0.6506538	up
A_24_P272313	KIAA1211L	0.9213572	1.8143544	1.3291597	0.8929973	0.4078026	0.6503999	up
A_23_P141362	FZD2	1.7219744	2.5509524	2.1935968	0.8289781	0.4716225	0.6503003	up
A_33_P3420416	LGALS9	1.3736701	1.7823439	2.2652721	0.4086738	0.891602	0.6501379	up
A_32_P88349	LOC730257	1.7101612	2.4017043	2.3187408	0.6915431	0.6085796	0.6500614	up
A_32_P159445	IQSEC2	5.1023273	5.7429996	5.761384	0.6406722	0.6590567	0.6498644	up
A_22_P00003131	lnc-C9orf139-1	-2.682341	-1.960278	-2.1048	0.7220633	0.5775411	0.6498022	up
A_33_P3314673	SYCE1L	-3.102716	-2.639949	-2.266002	0.4627667	0.8367136	0.6497401	up
A_22_P00005768	LINC01201	0.767798	1.3947058	1.4393077	0.6269078	0.6715097	0.6492088	up
A_23_P153964	INHBB	-1.945694	-1.239408	-1.353798	0.7062869	0.5918965	0.6490917	up
A_24_P356130	MAP2K5	-0.069302	0.6737709	0.4856153	0.7430725	0.5549169	0.6489947	up
A_21_P0008083	lnc-UCHL3-5	-2.517333	-1.928783	-1.808404	0.5885508	0.7089288	0.6487398	up
A_22_P00025801	TMEM210	6.7304955	7.4844165	7.2736654	0.753921	0.54317	0.6485455	up
A_23_P357374	PTH2	-1.255744	-0.414062	-0.801196	0.841682	0.4545484	0.6481152	up
A_33_P3315314	MT1HL1	4.200922	4.842897	4.854952	0.6419749	0.6540299	0.6480024	up
A_22_P00024637	lnc-SYNC-2	-2.048466	-1.491947	-1.309269	0.5565186	0.7391973	0.6478579	up
A_33_P3269408	FOXI2	6.961999	7.7098575	7.509391	0.7478585	0.5473919	0.6476252	up
A_24_P356373	HAGHL	0.6909328	1.3193593	1.3570466	0.6284266	0.6661139	0.6472702	up
A_33_P3380263	MTG1	0.413506	0.9999209	1.1215987	0.5864148	0.7080927	0.6472538	up
A_22_P00019852	LOC101927200	-3.112066	-2.133794	-2.796767	0.9782717	0.3152986	0.6467851	up
A_23_P57370	CECR5	2.91539	3.634685	3.4894867	0.719295	0.5740967	0.6466959	up
A_21_P0000732	RAB11B-AS1	2.2520008	2.9320827	2.8648958	0.6800818	0.612895	0.6464884	up
A_24_P306892	B4GALNT3	-2.800322	-2.152001	-2.15596	0.6483212	0.6443617	0.6463414	up
A_24_P194017	NOSIP	2.713729	3.4865623	3.2335577	0.7728334	0.5198288	0.6463311	up
A_22_P00024602	LOC101927437	-1.071051	-0.23629	-0.613257	0.8347616	0.4577937	0.6462777	up
A_33_P3404418	MAP3K6	-0.412751	0.2448511	0.2219381	0.6576018	0.6346889	0.6461453	up
A_22_P00009905	HEIH	1.4641871	2.2285228	1.9921103	0.7643356	0.5279231	0.6461294	up
A_23_P105794	EPSTI1	-1.707477	-1.419132	-0.704423	0.2883453	1.0030546	0.6457	up
A_33_P3256677	CCDC101	1.2142682	2.012508	1.7067318	0.7982397	0.4924636	0.6453517	up
A_33_P3262758	UNC93B1	1.6428194	2.4939466	2.0822315	0.8511272	0.4394121	0.6452696	up
A_33_P3393408	DECR2	-0.868046	-0.248643	-0.197019	0.6194034	0.6710272	0.6452153	up
A_33_P3279124	FAM21C	4.129387	4.912516	4.6360426	0.7831292	0.5066557	0.6448925	up
A_33_P3236858	TGFB1I1	2.0044222	2.6599412	2.638111	0.655519	0.6336889	0.644604	up
A_33_P3327663	SUSD4	1.2918558	2.1217237	1.7510772	0.8298678	0.4592214	0.6445446	up
A_23_P49865	YBX2	-1.654964	-0.930279	-1.091394	0.7246852	0.5635705	0.6441279	up
A_23_P139654	KLRC1	-2.9169	-2.224405	-2.321442	0.6924946	0.5954578	0.6439762	up
A_24_P389959	COPZ1	5.8656473	6.6317277	6.3856316	0.7660804	0.5199843	0.6430323	up
A_21_P0008399	lnc-KIF26A-1	-1.60923	-0.809452	-1.123201	0.799778	0.4860287	0.6429033	up
A_24_P398323	TRIM34	-2.915075	-2.33988	-2.204597	0.5751948	0.7104778	0.6428363	up
A_22_P00012561	LOC100506476	-1.310078	-0.464418	-0.871202	0.8456602	0.4388766	0.6422684	up
A_24_P37939	RPS14	1.2445421	1.9861608	1.7874427	0.7416186	0.5429006	0.6422596	up
A_33_P6809500	LOC100506844	0.1616259	1.1156421	0.4920645	0.9540162	0.3304386	0.6422274	up
A_33_P3218188	SPSB2	2.4168062	3.1785636	2.9393806	0.7617574	0.5225744	0.6421659	up
A_23_P430670	CHST5	-2.41677	-1.891974	-1.657718	0.5247955	0.7590523	0.6419239	up
A_33_P3248602	DUX4	-1.797494	-1.167178	-1.144174	0.6303158	0.6533198	0.6418178	up
A_33_P3420224	ENTPD8	2.0703382	2.872953	2.5501146	0.8026147	0.4797764	0.6411955	up
A_21_P0000809	NAPA-AS1	-1.78405	-0.893126	-1.392598	0.890924	0.3914514	0.6411877	up
A_24_P253818	FLOT2	-3.235809	-2.550297	-2.639156	0.6855123	0.5966528	0.6410825	up
A_23_P166297	ABCG1	-1.480879	-0.525713	-1.153911	0.9551659	0.3269682	0.641067	up
A_33_P3237729	PDLIM4	0.9554324	1.6054139	1.5873885	0.6499815	0.6319561	0.6409688	up
A_21_P0000225	SNORD83B	3.201707	3.9158015	3.7692986	0.7140946	0.5675917	0.6408432	up
A_23_P41145	FAM3D	-0.436263	0.2298136	0.1787376	0.6660767	0.6150007	0.6405387	up
A_21_P0001708	lnc-HMCN1-2	-2.469635	-1.837949	-1.82033	0.631686	0.6493056	0.6404958	up
A_19_P00322939	PVT1	2.5269861	3.2438655	3.0910416	0.7168794	0.5640554	0.6404674	up
A_24_P251962	PCDHB2	0.2194758	0.8182173	0.9015193	0.5987415	0.6820436	0.6403925	up
A_24_P19175	ZNF358	0.3533621	1.0892954	0.8968396	0.7359333	0.5434775	0.6397054	up

A_32_P210642	EGFL7	0.9398122	1.5507298	1.6081915	0.6109176	0.6683793	0.6396484	up
A_23_P340922	ZNF414	-0.943749	-0.237248	-0.371114	0.706501	0.5726352	0.6395681	up
A_23_P408353	HLA-A	4.8327312	5.620853	5.3236475	0.7881217	0.4909163	0.639519	up
A_23_P27107	TM4SF5	-0.822237	-0.040022	-0.325446	0.7822146	0.4967909	0.6395028	up
A_33_P3261353	BCORP1	-0.627017	0.0506244	-0.025891	0.6776409	0.6011252	0.6393831	up
A_33_P3250857	AIDA	-2.866009	-2.078965	-2.374907	0.7870438	0.491102	0.6390729	up
A_22_P00018891	lnc-ERLEC1-1	1.9118462	2.4820733	2.6192732	0.5702272	0.707427	0.6388271	up
A_21_P0004807	LOC101929484	-2.627055	-1.510395	-2.466266	1.1166599	0.1607883	0.6387241	up
A_33_P3280320	WI2-2373I1.2	-2.776553	-2.127673	-2.148051	0.6488793	0.6285014	0.6386904	up
A_22_P00009028	CCDC183-AS1	-1.747264	-1.116389	-1.100842	0.6308751	0.6464224	0.6386488	up
A_24_P96234	QTRT1	-1.938735	-1.255728	-1.344837	0.6830063	0.5938973	0.6384518	up
A_23_P17130	C2orf88	-1.292247	-0.647152	-0.660456	0.6450949	0.6317906	0.6384428	up
A_23_P372255	ITPKB	1.8887053	2.427607	2.626254	0.5389018	0.7375488	0.6382253	up
A_33_P3385957	TTLL1	-1.788066	-1.187978	-1.11183	0.6000886	0.6762366	0.6381626	up
A_33_P3375383	SNAPC2	3.9502506	4.603932	4.5720625	0.6536813	0.6218119	0.6377466	up
A_23_P420209	GCNT3	-3.632191	-2.997979	-2.990914	0.6342123	0.6412773	0.6377448	up
A_33_P3273480	BRSK2	-3.174752	-2.714202	-2.360283	0.4605498	0.8144689	0.6375093	up
A_33_P3293785	KRTAP10-3	-3.049009	-2.261577	-2.562289	0.787432	0.4867199	0.6370759	up
A_24_P245838	MGAT3	3.2881145	3.948299	3.901824	0.6601844	0.6137095	0.6369469	up
A_23_P24311	ALDH3B2	-1.096626	-0.306388	-0.61329	0.7902379	0.483336	0.6367869	up
A_33_P3413701	ERAP1	2.106679	2.6933064	2.7934885	0.5866275	0.6868095	0.6367185	up
A_21_P0000867	ZBTB20-AS1	4.838171	5.5483975	5.401373	0.7102265	0.5632019	0.6367142	up
A_23_P32404	ISG20	6.6714706	7.5427966	7.073189	0.871326	0.4017181	0.6365221	up
A_21_P0000177	C19orf83	-0.343484	0.250073	0.3353968	0.5935574	0.6788812	0.6362193	up
A_23_P107963	FUT1	3.6844044	4.547992	4.0928774	0.8635879	0.408473	0.6360304	up
A_21_P0000262	SNORA27	4.5808983	5.181663	5.252062	0.6007648	0.6711636	0.6359642	up
A_32_P158083	LHFPL3	-3.574161	-3.208743	-2.66899	0.3654187	0.9051709	0.6352948	up
A_23_P376088	LIME1	0.7097626	1.350625	1.3393764	0.6408625	0.6296139	0.6352382	up
A_23_P65651	WARS	2.716919	3.6995578	3.0045023	0.9826388	0.2875834	0.6351111	up
A_23_P85164	DNASE1L1	2.7403555	3.4592261	3.291605	0.7188706	0.5512495	0.6350601	up
A_24_P235266	GRB10	2.091691	3.134026	2.319069	1.042335	0.2273779	0.6348564	up
A_22_P00007888	LINC00092	0.5861855	1.0740399	1.367713	0.4878545	0.7815275	0.634691	up
A_23_P65983	CCDC102A	1.9342194	2.6351848	2.502061	0.7009654	0.5678415	0.6344035	up
A_33_P3318911	INPP5K	2.83776	3.4911056	3.4531612	0.6533456	0.6154013	0.6343734	up
A_23_P163711	FAM57B	1.200562	1.8771448	1.7926731	0.6765828	0.5921111	0.634347	up
A_23_P422071	B3GALT4	1.7276449	2.5490909	2.1748924	0.8214459	0.4472475	0.6343467	up
A_33_P3373364	CLIC4	-0.100404	1.0986128	-0.031336	1.1990166	0.0690675	0.634042	up
A_22_P00018303	SMAD1-AS1	-2.444054	-1.738103	-1.882236	0.7059512	0.5618186	0.6338849	up
A_33_P3245489	ADAMTSL5	6.2474318	7.036737	6.725692	0.7893052	0.47826	0.6337826	up
A_33_P3228128	ERCC1	3.87613	4.5386558	4.4811535	0.6625257	0.6050234	0.6337745	up
A_24_P673786	PIP4K2A	1.0057268	1.9658961	1.3120766	0.9601693	0.3063498	0.6332595	up
A_33_P3307163	RBM20	0.1064572	0.6085148	0.8709078	0.5020576	0.7644506	0.6332541	up
A_23_P60146	PDGFRL	-2.116347	-1.699947	-1.266286	0.4163995	0.8500609	0.6332302	up
A_23_P39076	RRAS	4.7499094	5.299169	5.4667244	0.5492597	0.716815	0.6330373	up
A_33_P3306264	LYPD3	2.5009575	3.3539977	2.9137754	0.8530402	0.412818	0.6329291	up
A_22_P00011268	lnc-OR51B4-3	-2.837754	-1.879287	-2.530597	0.9584665	0.3071563	0.6328114	up
A_23_P372988	PRR22	-2.715892	-2.113259	-2.053177	0.6026335	0.6627154	0.6326745	up
A_33_P3209950	RASGRP2	-0.899049	-0.202253	-0.330513	0.6967964	0.5685358	0.6326661	up
A_23_P120953	SERHL2	-1.865708	-1.238233	-1.228078	0.6274757	0.6376305	0.6325531	up
A_23_P413641	PREX1	-0.681556	-0.366364	0.2679477	0.3151927	0.9495039	0.6323483	up
A_33_P3424507	OR51F1	-1.296648	-0.603965	-0.725432	0.6926827	0.5712156	0.6319492	up
A_33_P3283780	ZBTB7A	-0.422363	0.0206332	0.3983803	0.442996	0.8207431	0.6318696	up
A_21_P0000278	SNORD10	1.3129368	2.0122218	1.8767586	0.699285	0.5638218	0.6315534	up
A_33_P3340808	FLJ46026	-3.04117	-2.50503	-2.314682	0.5361407	0.7264884	0.6313145	up
A_24_P225010	RPS15	8.800328	9.440149	9.423096	0.6398211	0.6227675	0.6312943	up
A_33_P3423721	JPH3	6.1341887	6.886664	6.644211	0.7524753	0.5100222	0.6312487	up

A_32_P511713	C11orf42	-0.972534	-0.30441	-0.378269	0.6681237	0.594265	0.6311944	up
A_33_P3239152	RABEP2	4.439948	5.0403247	5.1017227	0.6003766	0.6617746	0.6310756	up
A_19_P00808834	LINC01478	-1.379753	-0.635072	-0.862786	0.7446809	0.5169663	0.6308236	up
A_23_P250629	PSMB8	2.5378637	3.2026477	3.1346035	0.664784	0.5967398	0.6307619	up
A_24_P175909	MARS	4.89489	5.753979	5.297286	0.8590894	0.4023962	0.6307428	up
A_33_P3302305	BCKDHA	4.269203	5.0343776	4.765402	0.7651744	0.4961987	0.6306865	up
A_33_P3216869	CRABP1	0.2472649	0.8399806	0.9159207	0.5927157	0.6686559	0.6306858	up
A_23_P77493	TUBB3	5.168666	5.689857	5.9088354	0.5211911	0.7401695	0.6306803	up
A_24_P904903	DDX39B	0.5603876	1.310214	1.0718293	0.7498264	0.5114417	0.6306341	up
A_23_P163955	PEMT	2.7571325	3.4082465	3.3664951	0.651114	0.6093626	0.6302383	up
A_21_P0005031	lnc-KHDRBS2-1	-2.795717	-2.178281	-2.152744	0.6174355	0.642973	0.6302042	up
A_33_P3239860	ANKDD1A	-2.934399	-2.144104	-2.464409	0.7902949	0.4699895	0.6301422	up
A_23_P50710	CYP4F2	0.0637002	0.8945766	0.4929004	0.8308764	0.4292002	0.6300383	up
A_19_P00317519	LOC101927043	1.8119364	2.5352998	2.3486156	0.7233634	0.5366793	0.6300213	up
A_21_P0007006	lnc-DYDC1-3	-3.242317	-2.354305	-2.871031	0.8880122	0.3712862	0.6296492	up
A_23_P131060	CYP4F8	1.9724865	2.802567	2.4011736	0.8300805	0.4286871	0.6293838	up
A_23_P406131	TMEM159	3.8625154	4.5906377	4.3923445	0.7281222	0.529829	0.6289756	up
A_23_P76291	PRR4	-0.715973	-0.264663	0.0903668	0.4513106	0.8063402	0.6288254	up
A_23_P410717	CIART	0.3932548	1.2227526	0.8212771	0.8294978	0.4280224	0.6287601	up
A_33_P3268338	DDRGK1	2.4849148	3.1964211	3.0301037	0.7115064	0.5451889	0.6283476	up
A_33_P3389286	SFN	7.892503	8.753801	8.287738	0.8612986	0.3952351	0.6282668	up
A_33_P3263938	LCN15	-3.276519	-2.721912	-2.574621	0.5546074	0.7018983	0.6282529	up
A_33_P3248137	OLFML2A	-3.121942	-2.726438	-2.261091	0.3955045	0.8608513	0.6281779	up
A_21_P0013856	lnc-MAGEA8-2	-2.56707	-1.994629	-1.88374	0.5724411	0.6833296	0.6278854	up
A_32_P18072	lnc-RLBP1-1	-1.228474	-0.535741	-0.666022	0.6927328	0.5624518	0.6275923	up
A_23_P48455	AMN	1.7303209	2.4829345	2.2322683	0.7526135	0.5019474	0.6272805	up
A_33_P3411628	CDKN2A	4.4494762	5.2167954	4.936243	0.7673192	0.4867668	0.627043	up
A_22_P00015215	LINC01152	-3.288584	-2.451286	-2.87238	0.8372974	0.4162035	0.6267505	up
A_23_P156824	HTR1B	-3.521943	-2.47121	-3.31947	1.0507333	0.2024734	0.6266034	up
A_23_P103486	CYP2J2	1.7063351	2.1492286	2.516265	0.4428935	0.8099299	0.6264117	up
A_23_P157147	ZDHHC4	2.4677477	3.3754544	2.812687	0.9077067	0.3449392	0.626323	up
A_21_P0001085	LOC100130417	-3.176038	-2.193755	-2.905886	0.9822826	0.2701514	0.626217	up
A_33_P3310293	PKIG	-1.647522	-1.019291	-1.023366	0.6282301	0.624156	0.6261931	up
A_22_P00009178	LOC340357	-1.92302	-1.215651	-1.37858	0.7073693	0.5444403	0.6259048	up
A_24_P362904	PFKFB4	-0.692264	-0.297297	0.1644368	0.3949676	0.8567009	0.6258342	up
A_23_P359457	DNAH2	-1.779857	-1.072388	-1.236032	0.7074695	0.5438256	0.6256475	up
A_22_P00016555	lnc-TNFRSF10A-1	-1.640853	-1.113749	-0.916667	0.5271034	0.724185	0.6256442	up
A_23_P131899	SDCBP2	2.6428823	3.6279445	2.9090748	0.9850621	0.2661924	0.6256273	up
A_33_P3294966	LOC554206	-2.841019	-2.280918	-2.150182	0.5601015	0.6908376	0.6254696	up
A_24_P305662	TRMT2A	0.7597513	1.346343	1.4240088	0.5865917	0.6642575	0.6254246	up
A_33_P3323323	ZNF497	0.4375486	1.3144093	0.8112631	0.8768606	0.3737145	0.6252875	up
A_23_P431305	FAM69B	-0.262038	0.3477206	0.3785815	0.6097584	0.6406193	0.6251889	up
A_33_P3229953	EEF1A2	2.2814713	3.0205255	2.792326	0.7390542	0.5108547	0.6249545	up
A_23_P206371	NOL3	4.76513	5.5269933	5.252964	0.7618632	0.487834	0.6248486	up
A_23_P23646	USF1	-2.180031	-1.603556	-1.508212	0.5764751	0.6718187	0.6241469	up
A_33_P6469777	SNHG7	6.2291346	7.060618	6.6455564	0.8314834	0.4164219	0.6239526	up
A_33_P3275835	TOR2A	4.5106134	5.194777	5.0738516	0.6841636	0.5632381	0.6237009	up
A_24_P167377	OR10P1	-3.566865	-3.027703	-2.858834	0.5391622	0.7080305	0.6235963	up
A_24_P35891	ZNF219	1.1315508	1.832202	1.6779485	0.7006512	0.5463977	0.6235244	up
A_23_P14667	SLC28A1	-2.084538	-1.562992	-1.359247	0.5215459	0.7252908	0.6234184	up
A_33_P3366336	SBK1	1.0040479	1.7708201	1.4840703	0.7667723	0.4800224	0.6233974	up
A_22_P00016785	LINC00967	1.0388241	1.7061086	1.6182647	0.6672845	0.5794406	0.6233626	up
A_33_P3326423	POLL	2.9034271	3.6576629	3.3954554	0.7542357	0.4920282	0.623132	up
A_22_P00010200	LOC101927895	-2.550245	-1.56776	-2.286704	0.9824851	0.2635405	0.6230128	up
A_23_P59772	CLCN1	-2.322349	-1.62991	-1.76888	0.6924391	0.5534687	0.6229539	up
A_22_P00007974	lnc-IDH3G-1	-1.637451	-1.146109	-0.882941	0.4913425	0.7545104	0.6229265	up

A_24_P275828	PGAP3	6.058547	6.7211337	6.640992	0.6625867	0.5824451	0.6225159	up
A_33_P3216467	FAM83E	0.7542615	1.7395773	1.0139508	0.9853158	0.2596893	0.6225026	up
A_22_P00005662	lnc-EIF3A-1	-2.965992	-2.23311	-2.454831	0.7328823	0.5111613	0.6220218	up
A_22_P00008991	GRTP1-AS1	-1.749576	-1.059176	-1.195996	0.6903997	0.5535803	0.62199	up
A_23_P23850	DAB1	-0.942784	-0.141109	-0.500732	0.8016753	0.4420519	0.6218636	up
A_22_P00020737	lnc-C10orf71-1	3.6220026	4.383099	4.104023	0.7610965	0.4820204	0.6215584	up
A_23_P72584	ACBD7	0.6969256	1.4749789	1.1617284	0.7780533	0.4648027	0.621428	up
A_23_P74088	MMP23B	-3.298483	-2.738836	-2.615297	0.5596466	0.6831861	0.6214163	up
A_23_P160318	COL16A1	1.8761759	2.8522897	2.142705	0.9761138	0.2665291	0.6213214	up
A_33_P3356462	C2CD4A	-3.102804	-2.58238	-2.380892	0.5204234	0.7219122	0.6211678	up
A_23_P107981	SULT2B1	4.836111	5.5467505	5.3676605	0.7106395	0.5315495	0.6210945	up
A_33_P3289596	EFR3B	-3.055702	-2.473127	-2.396506	0.5825756	0.6591961	0.6208859	up
A_24_P327011	PPCDC	-3.139916	-2.646641	-2.391692	0.4932756	0.7482243	0.62075	up
A_33_P3608210	MIR31HG	1.5861588	2.3268132	2.0868988	0.7406545	0.5007401	0.6206973	up
A_33_P3308456	PRAC2	0.863421	1.4623728	1.5055747	0.5989518	0.6421537	0.6205528	up
A_23_P748	IRF6	0.8709173	1.6176915	1.3648682	0.7467742	0.4939508	0.6203625	up
A_23_P18692	ADH5	1.3290844	2.0107818	1.8876419	0.6816974	0.5585575	0.6201274	up
A_21_P0006495	lnc-TIMP1-1	-2.39786	-1.829781	-1.72626	0.568079	0.6716003	0.6198397	up
A_21_P0007467	LOC102723862	-2.984876	-2.382551	-2.347651	0.6023252	0.6372254	0.6197753	up
A_21_P0000223	SNORD15B	2.7632046	3.3878255	3.3780975	0.6246209	0.614893	0.6197569	up
A_33_P3297126	TSNARE1	-2.05701	-1.355213	-1.519296	0.7017965	0.5377135	0.619755	up
A_21_P0012884	LOC728613	0.3399043	1.1822796	0.736681	0.8423753	0.3967767	0.619576	up
A_33_P3291567	TSNARE1	0.3804922	0.7978358	1.2021942	0.4173436	0.821702	0.6195228	up
A_22_P00020414	lnc-TTC7B-2	-0.570784	0.0732288	0.02423	0.6440125	0.5950136	0.619513	up
A_24_P44279	FBXW8	-2.718676	-2.42537	-1.773162	0.2933066	0.945514	0.6194103	up
A_23_P501933	CACNG6	-1.372072	-1.071395	-0.434059	0.3006773	0.9380136	0.6193454	up
A_32_P194072	TECPR1	1.9312601	2.6900096	2.4107895	0.7587495	0.4795294	0.6191394	up
A_23_P90233	ZSCAN5A	1.6703653	2.275045	2.3038511	0.6046796	0.6334858	0.6190827	up
A_23_P363313	SLC16A11	-1.871885	-1.325409	-1.180318	0.5464759	0.6915669	0.6190214	up
A_23_P259207	THNSL2	-0.131166	0.5820169	0.3935294	0.7131834	0.5246959	0.6189397	up
A_22_P00006278	lnc-FAM76A-1	2.7173624	3.4136891	3.2587042	0.6963267	0.5413418	0.6188343	up
A_23_P137543	ZNF362	1.2577157	1.9243522	1.8284893	0.6666365	0.5707736	0.618705	up
A_33_P3310226	LOC100129115	-1.404672	-0.791607	-0.78072	0.6130643	0.6239514	0.6185079	up
A_24_P284805	INPP5J	-2.118024	-1.240089	-1.758971	0.8779359	0.3590531	0.6184945	up
A_33_P3355230	LAIR1	-2.663389	-1.913573	-2.176444	0.7498159	0.4869452	0.6183805	up
A_21_P0012387	XLOC_l2_009811	-2.962831	-2.392587	-2.296927	0.5702438	0.6659041	0.6180739	up
A_22_P00019988	lnc-GTPBP1-2	1.9752264	2.6216874	2.5647545	0.646461	0.5895281	0.6179946	up
A_33_P3321946	MOSPD3	2.2590609	2.9537568	2.800273	0.694696	0.5412121	0.617954	up
A_23_P218423	MRPL10	5.020296	5.7754626	5.501033	0.7551665	0.4807367	0.6179516	up
A_22_P00020612	lnc-CISD2-1	-1.09512	-0.512417	-0.442987	0.5827031	0.6521335	0.6174183	up
A_23_P108280	CYP4F12	1.2486691	2.1834254	1.5484667	0.9347563	0.2997975	0.6172769	up
A_21_P0000384	SNORD85	-2.202961	-1.700681	-1.471084	0.5022802	0.7318778	0.617079	up
A_33_P3420655	KDM4A	1.7862511	2.5530086	2.253109	0.7667575	0.4668579	0.6168077	up
A_22_P00003276	lnc-CALML5-1	-2.447571	-1.780033	-1.881739	0.6675382	0.5658326	0.6166854	up
A_23_P98232	ZFPL1	0.0352755	0.8042955	0.4994631	0.7690201	0.4641876	0.6166039	up
A_33_P3849275	FHL1	3.468192	4.2796006	3.8899736	0.8114085	0.4217815	0.616595	up
A_33_P3311371	PDLIM2	0.569036	1.2909584	1.0801716	0.7219224	0.5111356	0.616529	up
A_22_P00006901	SCARNA10	-0.266284	0.6038427	0.0965886	0.8701272	0.3628731	0.6165001	up
A_33_P3396886	C11orf52	1.4291873	2.0894294	2.0015182	0.6602421	0.572331	0.6162865	up
A_23_P114626	SERPINC1	-3.046618	-2.237692	-2.62321	0.8089261	0.423408	0.6161671	up
A_33_P3241884	SDC3	-3.10472	-2.76507	-2.212272	0.3396506	0.8924487	0.6160496	up
A_24_P23995	RNF187	3.758092	4.415611	4.3319225	0.6575189	0.5738306	0.6156747	up
A_33_P3252834	PHLDA3	3.0767822	3.6869292	3.697815	0.610147	0.6210327	0.6155899	up
A_23_P349406	RIMKLA	0.2216888	0.9186068	0.7557249	0.696918	0.5340362	0.6154771	up
A_33_P3215487	AP5Z1	3.9165878	4.618978	4.445114	0.7023902	0.5285263	0.6154583	up
A_22_P00002048	lnc-BID-2	5.82858	6.6115365	6.2759027	0.7829566	0.4473229	0.6151397	up

A_24_P111054	SLC2A5	-1.8852	-1.144126	-1.396023	0.7410736	0.4891763	0.6151249	up
A_23_P22224	EIF4EBP1	4.449916	5.5395665	4.589958	1.0896506	0.1400423	0.6148465	up
A_22_P00003110	lnc-C9orf103-1	6.249241	7.040152	6.6879435	0.7909112	0.4387026	0.6148069	up
A_33_P3256391	CRB3	3.4117956	4.1623054	3.89085	0.7505097	0.4790545	0.6147821	up
A_23_P350074	SYNE4	0.7350888	1.1470995	1.5525723	0.4120107	0.8174834	0.614747	up
A_33_P3404032	HIST3H2A	-0.627332	0.1125822	-0.138012	0.7399144	0.4893198	0.6146171	up
A_24_P30567	SPPL3	2.490694	3.3174844	2.8930693	0.8267903	0.4023752	0.6145828	up
A_23_P3038	GPX2	6.105564	6.7407365	6.6994286	0.6351724	0.5938644	0.6145184	up
A_24_P379727	CHCHD5	2.35639	2.9894433	2.9520588	0.6330533	0.5956688	0.6143611	up
A_33_P3399468	LOC100133182	6.073491	6.7878547	6.5877275	0.7143636	0.5142365	0.6143	up
A_22_P00024993	LOC102724231	0.266592	1.0658259	0.6954703	0.7992339	0.4288783	0.6140561	up
A_24_P257359	RPL15	8.440395	9.161385	8.9471655	0.7209892	0.5067701	0.6138797	up
A_33_P3248329	ZFAND3	-0.221415	0.678268	0.1066079	0.8996825	0.3280225	0.6138525	up
A_23_P75441	NUDT22	4.218051	4.7425637	4.9210777	0.5245128	0.7030268	0.6137698	up
A_23_P134433	EN2	6.107214	6.821547	6.6202765	0.7143331	0.5130625	0.6136978	up
A_19_P00319633	LOC100294145	-0.614875	-0.013088	0.0106626	0.6017876	0.6255379	0.6136627	up
A_33_P3372910	DDX58	1.5840101	2.0005288	2.3944063	0.4165187	0.8103962	0.6134575	up
A_33_P3326020	KANK3	-0.111737	0.4019613	0.600821	0.5136986	0.7125583	0.6131284	up
A_33_P3272117	ASPSCR1	1.3276076	1.903326	1.9781456	0.5757184	0.650538	0.6131282	up
A_23_P43490	CDKN2A	6.971409	7.741134	7.4278336	0.7697253	0.4564247	0.613075	up
A_21_P0002553	FLJ43879	-0.098524	0.2272282	0.8014317	0.3257518	0.8999553	0.6128535	up
A_22_P00017960	lnc-ZKSCAN1-1	2.286028	3.0167632	2.7801247	0.7307353	0.4940968	0.612416	up
A_22_P00025104	LOC101927472	-1.954508	-1.326435	-1.357991	0.6280732	0.5965171	0.6122952	up
A_23_P97195	MTFR1L	-0.716204	-0.146642	-0.061229	0.5695615	0.6549745	0.612268	up
A_21_P0000188	ELF5	0.5847435	1.0884128	1.3053575	0.5036693	0.720614	0.6121416	up
A_23_P122531	C6orf48	4.0184298	4.919431	4.341668	0.9010015	0.3232384	0.6121199	up
A_24_P89911	NAIF1	1.4663386	2.0673232	2.089384	0.6009846	0.6230454	0.612015	up
A_21_P0003135	lnc-VGLL3-3	-2.513999	-1.639905	-2.164131	0.8740943	0.3498676	0.6119809	up
A_22_P00016235	SNORD57	2.626255	3.314053	3.1620378	0.687798	0.5357828	0.6117904	up
A_23_P140725	IFT140	-0.046872	0.7245154	0.405314	0.7713876	0.4521861	0.6117869	up
A_33_P3401782	C19orf54	-2.80117	-2.101198	-2.277783	0.6999719	0.5233865	0.6116792	up
A_23_P24077	C10orf54	3.8462648	4.765184	4.150613	0.9189191	0.304348	0.6116336	up
A_21_P0009990	lnc-BIRC7-1	-2.256565	-1.684218	-1.605855	0.5723467	0.6507101	0.6115284	up
A_33_P3399755	EPB42	3.3911448	4.1859565	3.8193064	0.7948117	0.4281616	0.6114867	up
A_23_P303891	LCE1C	4.2574053	4.8578506	4.8796835	0.6004453	0.6222782	0.6113618	up
A_23_P15357	LGALS3BP	4.582797	5.219568	5.1681166	0.6367707	0.5853195	0.6110451	up
A_23_P24384	CCDC88B	2.88867	3.4593577	3.5396748	0.5706878	0.6510048	0.6108463	up
A_21_P0005540	lnc-ACTR3C-2	-3.128697	-2.784081	-2.2517	0.3446167	0.8769972	0.6108069	up
A_23_P112548	HDHD3	-0.107604	0.5299921	0.4761281	0.6375957	0.5837317	0.6106637	up
A_21_P0005983	LOC100288842	-2.92625	-2.434732	-2.196831	0.4915173	0.7294183	0.6104678	up
A_23_P253200	RPL15	4.0990143	4.8749003	4.543501	0.7758861	0.4444866	0.6101863	up
A_22_P00008117	lnc-IL25-1	-2.226838	-1.608503	-1.624813	0.6183353	0.6020255	0.6101804	up
A_21_P0010928	lnc-SORCS3-7	-1.541386	-0.840376	-1.02205	0.7010098	0.5193358	0.6101728	up
A_21_P0009926	lnc-STK35-1	4.8079586	5.479242	5.3566246	0.6712833	0.548666	0.6099746	up
A_33_P3251462	C20orf141	3.9514341	4.682075	4.4403534	0.7306409	0.4889193	0.6097801	up
A_33_P3341105	PDE6G	-1.897192	-1.259744	-1.31527	0.6374483	0.5819221	0.6096852	up
A_22_P00010628	lnc-NDUFS8-1	-2.523123	-1.12923	-2.697908	1.3938937	-0.174784	0.6095546	up
A_24_P152468	LOC100128364	2.557228	3.2546563	3.0784407	0.6974282	0.5212126	0.6093204	up
A_23_P74112	IFNLR1	1.5431824	2.3012843	2.002695	0.7581019	0.4595127	0.6088073	up
A_33_P3292130	XLOC_l2_013383	5.6764946	6.465818	6.104642	0.7893233	0.4281473	0.6087353	up
A_21_P0005843	lnc-ASPH-6	-2.559558	-1.919422	-1.982694	0.6401365	0.5768645	0.6085005	up
A_33_P3382351	RNASEK	6.735854	7.3552985	7.3329687	0.6194444	0.5971146	0.6082795	up
A_21_P0005248	lnc-ASB4-3	-2.983197	-2.350772	-2.3991	0.6324246	0.5840969	0.6082608	up
A_33_P6812270	LOC100506844	0.015347	0.8947496	0.352387	0.8794026	0.33704	0.6082213	up
A_33_P3286293	CTNNBIP1	-0.167851	0.561934	0.3185697	0.729785	0.4864206	0.6081028	up
A_33_P3352544	ACOT8	1.2044754	1.8562884	1.7687316	0.651813	0.5642562	0.6080346	up

A_21_P0011562	XLOC_l2_005933	1.3557081	2.066215	1.8609648	0.7105069	0.5052567	0.6078818	up
A_22_P00002137	lnc-BPHL-1	0.0383563	0.6376367	0.653471	0.5992804	0.6151147	0.6071975	up
A_21_P0000346	SNORA36B	-2.287702	-1.697082	-1.664012	0.5906205	0.6236901	0.6071553	up
A_23_P330537	SPSB3	-0.816721	-0.115921	-0.303441	0.7007995	0.5132794	0.6070395	up
A_33_P3401295	CRCT1	1.7432194	2.5371566	2.163334	0.7939372	0.4201145	0.6070259	up
A_21_P0013498	XLOC_l2_014289	-3.388798	-2.771144	-2.792488	0.6176546	0.5963104	0.6069825	up
A_24_P231546	FAM178B	-0.025357	0.4801679	0.6829495	0.5055251	0.7083068	0.606916	up
A_33_P3271455	PXDN	-1.473292	-0.975672	-0.757237	0.4976206	0.7160554	0.606838	up
A_19_P00327081	XLOC_l2_001273	-2.638109	-2.158645	-1.904326	0.4794643	0.7337835	0.6066239	up
A_23_P320553	PPFIA3	0.308115	0.9377189	0.8913407	0.6296039	0.5832257	0.6064148	up
A_23_P132388	SCO2	4.1248627	4.577145	4.885394	0.4522824	0.7605314	0.6064069	up
A_33_P3371954	IGSF8	-0.399142	0.1261725	0.2878647	0.5253143	0.6870065	0.6061604	up
A_19_P00807643	CYP1B1-AS1	-1.872413	-0.986545	-1.546324	0.8858676	0.3260889	0.6059783	up
A_24_P687326	FAM225A	-3.283619	-2.353154	-3.0026	0.930465	0.281019	0.605742	up
A_21_P0000281	SNORD59A	-3.432587	-3.024387	-2.62946	0.4082007	0.8031278	0.6056643	up
A_21_P0012908	XLOC_l2_012046	-1.968491	-1.689461	-1.036411	0.2790294	0.9320793	0.6055543	up
A_23_P208389	AXL	-0.677266	-0.068625	-0.075142	0.6086407	0.6021233	0.605382	up
A_23_P16116	RAB4B	0.3261046	0.9120174	0.9507155	0.5859127	0.6246109	0.6052618	up
A_21_P0000240	SNORD100	0.6922894	1.0335736	1.5610056	0.3412843	0.8687162	0.6050003	up
A_33_P3416231	HOXA9	0.9707069	1.6063724	1.5446553	0.6356654	0.5739484	0.6048069	up
A_33_P3396956	KDF1	-1.358442	-0.743937	-0.763403	0.6145053	0.5950384	0.6047719	up
A_21_P0003162	lnc-RABL3-1	-1.750738	-1.101377	-1.190562	0.6493607	0.5601754	0.604768	up
A_23_P349416	ERBB3	2.4881334	3.3232055	2.8625526	0.835072	0.3744192	0.6047456	up
A_21_P0014466	LOC101928152	-1.560561	-1.171333	-0.740482	0.3892283	0.8200789	0.6046536	up
A_33_P3355266	TINAGL1	5.690419	6.3981843	6.1918535	0.7077651	0.5014343	0.6045997	up
A_23_P415643	ZNF48	2.4537077	2.890533	3.2260742	0.4368253	0.7723665	0.6045959	up
A_24_P132276	QRSL1	-1.508308	-0.835672	-0.971859	0.6726351	0.536448	0.6045416	up
A_23_P41804	NKD2	4.751045	5.3840814	5.326832	0.6330361	0.5757866	0.6044114	up
A_23_P107322	CORO6	0.0818043	0.7115712	0.6607981	0.6297669	0.5789938	0.6043804	up
A_22_P00000643	lnc-ACSM5-1	-1.301658	-0.827738	-0.567473	0.4739204	0.7341852	0.6040528	up
A_21_P0011409	XLOC_l2_004898	-3.192236	-2.420458	-2.756438	0.7717788	0.4357989	0.6037888	up
A_24_P143492	BCAS4	-0.312975	0.1943975	0.3869929	0.5073724	0.6999679	0.6036701	up
A_33_P3406004	YBEY	0.2178249	0.8649206	0.7780285	0.6470957	0.5602036	0.6036496	up
A_33_P3389634	CAPG	5.8393526	6.5025477	6.3833656	0.6631951	0.544013	0.6036041	up
A_22_P00001847	THOC7-AS1	1.3301468	2.0102859	1.8569784	0.6801391	0.5268316	0.6034853	up
A_33_P3302373	SZT2	-2.722397	-2.078915	-2.158966	0.6434827	0.5634313	0.603457	up
A_33_P3220663	STAC3	-1.360474	-0.47423	-1.03993	0.8862438	0.3205438	0.6033938	up
A_33_P3278826	LTK	1.0237966	1.6724286	1.5818019	0.6486321	0.5580053	0.6033187	up
A_22_P00006598	LOC101927675	1.6304693	2.3983445	2.0689592	0.7678752	0.4384899	0.6031826	up
A_32_P34138	FAM25A	2.0173082	2.9988418	2.2418594	0.9815335	0.2245512	0.6030424	up
A_24_P517901	HNRNPA1	2.87191	3.4959311	3.4537134	0.6240211	0.5818033	0.6029122	up
A_23_P4696	PPAN-P2RY11	-2.334877	-1.580565	-1.883462	0.754312	0.4514155	0.6028638	up
A_21_P0008454	lnc-MAP3K9-1	-2.219164	-1.52472	-1.708264	0.6944447	0.5109	0.6026723	up
A_23_P433218	OR7E91P	0.7489982	1.546318	1.1567144	0.7973199	0.4077163	0.6025181	up
A_33_P3240328	PITX1	4.9058256	5.579227	5.437249	0.6734014	0.5314236	0.6024125	up
A_23_P419107	TCP11L2	-2.888586	-1.733449	-2.839137	1.1551373	0.0494497	0.6022935	up
A_19_P00315869	lnc-DET1-1	0.919559	1.5555949	1.4879556	0.6360359	0.5683966	0.6022162	up
A_24_P71700	ZBTB47	1.1523075	1.7020025	1.8070073	0.549695	0.6546998	0.6021974	up
A_33_P3422747	DIS3L2	-1.219235	-0.611818	-0.623159	0.6074171	0.596076	0.6017466	up
A_23_P26386	TPPP3	1.258502	1.6344743	2.0856848	0.3759723	0.8271828	0.6015775	up
A_23_P12405	ESPN	-1.350637	-0.629847	-0.868994	0.7207899	0.4816427	0.6012163	up
A_22_P00008089	TMC3-AS1	-1.144445	-0.444391	-0.642074	0.7000537	0.5023713	0.6012125	up
A_33_P3210622	ASB13	-0.760265	-0.188077	-0.130548	0.5721874	0.6297174	0.6009524	up
A_22_P00023356	lnc-C1orf222-2	3.7080507	4.355699	4.2622623	0.6476483	0.5542116	0.60093	up
A_24_P91472	ALKBH7	2.7503586	3.4385076	3.264041	0.688149	0.5136824	0.6009157	up
A_22_P00023006	lnc-CHRNA6-1	3.3040028	3.9218717	3.8878727	0.6178689	0.5838699	0.6008694	up

A_22_P00016861	LRRC75A-AS1	3.2000437	3.8923297	3.709322	0.692286	0.5092783	0.6007822	up
A_23_P84189	PITPNC1	-2.984259	-2.627026	-2.140313	0.3572326	0.8439462	0.6005894	up
A_33_P3681776	TOLLIP-AS1	-1.096271	-0.327602	-0.664998	0.7686687	0.431273	0.5999708	up
A_23_P119353	RASIP1	-0.090055	0.7518816	0.2678947	0.8419361	0.3579493	0.5999427	up
A_23_P140928	TMC7	-0.992784	-0.389714	-0.396019	0.6030698	0.596765	0.5999174	up
A_33_P3323607	CCDC129	-1.730049	-1.097062	-1.163679	0.632987	0.5663695	0.5996783	up
A_24_P450285	CCDC153	-1.316514	-0.646576	-0.787134	0.6699376	0.5293798	0.5996587	up
A_22_P00018968	lnc-TMEM106C-2	3.9083033	4.564711	4.4509563	0.6564078	0.5426531	0.5995305	up
A_22_P00010905	LOC100294362	-2.380021	-1.826248	-1.734786	0.5537729	0.6452355	0.5995042	up
A_23_P358944	PML	3.9440804	4.4790606	4.608078	0.5349803	0.6639977	0.599489	up
A_21_P0001338	lnc-CDC7-1	-3.356703	-2.624667	-2.890024	0.7320356	0.4666793	0.5993575	up
A_23_P336992	ZFAND2A	2.1336699	2.935307	2.5306358	0.8016372	0.396966	0.5993016	up
A_23_P48237	MCRS1	2.2050772	2.692017	2.916685	0.4869399	0.7116079	0.5992739	up
A_33_P3227252	LINC01000	3.0998945	3.734486	3.6637821	0.6345916	0.5638876	0.5992396	up
A_23_P166421	TBC1D10A	3.3290281	3.997168	3.8591661	0.6681399	0.530138	0.599139	up
A_22_P00023107	lnc-CCDC37-3	-3.253233	-2.828568	-2.480381	0.4246647	0.7728522	0.5987585	up
A_23_P109470	THOC5	1.4605379	2.1344352	1.9836979	0.6738973	0.52316	0.5985286	up
A_32_P22078	RPS9	7.176464	7.962621	7.586995	0.7861571	0.410531	0.5983441	up
A_23_P163567	SMPD3	1.1188855	1.3842602	2.0501137	0.2653747	0.9312282	0.5983014	up
A_24_P21770	YPEL4	-0.250058	0.3835578	0.3126164	0.633616	0.5626745	0.5981453	up
A_21_P0008072	lnc-AL445989.1-2	-0.348436	0.1670036	0.3317576	0.51544	0.6801939	0.597817	up
A_23_P424561	RHOV	2.9700556	3.776013	3.3595743	0.8059573	0.3895187	0.597738	up
A_22_P00013941	lnc-RRP8-1	-1.232324	-0.611931	-0.657307	0.6203928	0.5750175	0.5977051	up
A_23_P428184	HIST1H2AD	5.250868	5.9890637	5.707901	0.7381959	0.4570332	0.5976145	up
A_23_P5778	RAB17	0.7748752	1.4459243	1.2989678	0.6710491	0.5240927	0.5975709	up
A_23_P425750	ARMC6	3.0342693	3.6110806	3.6525812	0.5768113	0.6183119	0.5975616	up
A_33_P3239123	PPIH	-3.020196	-2.606249	-2.239057	0.4139471	0.7811389	0.597543	up
A_33_P3390177	LINC00884	-0.648683	0.0568309	-0.159243	0.705514	0.4894404	0.5974772	up
A_23_P141315	NLE1	3.0755405	3.7051682	3.6406298	0.6296277	0.5650892	0.5973585	up
A_21_P0002930	lnc-TSEN2-1	-3.260652	-2.844888	-2.481788	0.4157643	0.7788637	0.597314	up
A_33_P3321507	TNRC18	5.4581366	6.149551	5.9613476	0.6914144	0.503211	0.5973127	up
A_33_P3415633	TMEM136	-0.431117	0.1290855	0.2028737	0.5602021	0.6339903	0.5970962	up
A_24_P85775	THEMIS2	-1.98991	-1.086053	-1.699651	0.9038572	0.2902589	0.5970581	up
A_23_P156708	TNXB	-1.634259	-0.919081	-1.156138	0.715178	0.4781218	0.5966499	up
A_23_P12784	FRAT2	3.5985298	4.394297	3.9958973	0.7957673	0.3973675	0.5965674	up
A_23_P216708	RFK	1.0694442	1.7410264	1.5906491	0.6715822	0.521205	0.5963936	up
A_33_P3423954	CBX2	-3.304273	-2.467656	-2.948275	0.836617	0.355998	0.5963075	up
A_19_P00319337	LINC00574	-2.204876	-1.668744	-1.548454	0.5361319	0.6564217	0.5962768	up
A_33_P3263625	DUSP8	-3.245084	-2.512119	-2.785524	0.7329652	0.4595597	0.5962625	up
A_33_P3408898	C6orf226	2.231759	2.7181926	2.9375935	0.4864335	0.7058344	0.596134	up
A_23_P141680	BCAS3	0.1761198	0.932796	0.61164	0.7566762	0.4355202	0.5960982	up
A_33_P3404470	UNC93B1	1.6889496	2.4713435	2.098692	0.7823939	0.4097424	0.5960681	up
A_33_P3359683	IL16	-0.473988	0.0629978	0.1805453	0.5369854	0.6545329	0.5957592	up
A_22_P00005726	LOC100287592	-1.205508	-0.508676	-0.710871	0.6968327	0.4946375	0.5957351	up
A_33_P3283713	ABHD14A	2.88513	3.482451	3.479169	0.597321	0.594039	0.59568	up
A_23_P105691	GPRC5D	-2.043245	-1.397832	-1.497967	0.6454125	0.5452781	0.5953453	up
A_32_P189781	LINC00520	-0.128051	1.1065855	-0.172251	1.2346363	-0.0442	0.5952182	up
A_23_P251499	PCOLCE	-1.437729	-0.793882	-0.891174	0.6438465	0.5465546	0.5952005	up
A_23_P67618	ZNF792	-1.530367	-0.844714	-1.025684	0.6856532	0.504683	0.5951681	up
A_23_P382775	BBC3	2.532484	3.322332	2.93293	0.7898479	0.4004459	0.5951469	up
A_23_P4714	MIA	5.8850813	6.283586	6.676862	0.3985047	0.7917805	0.5951426	up
A_33_P3415895	NRBF2	0.5338054	1.0427575	1.2150488	0.5089521	0.6812434	0.5950978	up
A_33_P3230290	ACAP3	4.895711	5.5315156	5.4499073	0.6358047	0.5541964	0.5950005	up
A_23_P258018	MYL5	1.2042341	1.999165	1.5985403	0.7949309	0.3943062	0.5946186	up
A_33_P3395743	VWA1	4.2540636	4.856149	4.840996	0.6020856	0.5869322	0.5945089	up
A_33_P3233273	LRRC61	0.0576539	0.7926078	0.5115023	0.7349539	0.4538484	0.5944011	up

A_22_P00025157	LOC101929683	-3.439312	-2.590424	-3.099486	0.8488884	0.3398264	0.5943574	up
A_33_P3367899	FRAT1	-1.33265	-0.739511	-0.737178	0.5931387	0.5954714	0.5943051	up
A_33_P3369760	GLIPR2	-1.78774	-1.154244	-1.232692	0.6334958	0.5550475	0.5942717	up
A_22_P00014982	lnc-SMC1B-1	-0.864686	-0.285304	-0.255594	0.5793819	0.6090918	0.5942369	up
A_23_P3819	ZNF747	-2.956589	-2.286343	-2.439122	0.6702461	0.5174668	0.5938565	up
A_33_P3316878	CHPF	6.382679	6.982038	6.9709587	0.599359	0.5882797	0.5938194	up
A_33_P3281745	lnc-C10orf71-1	-3.282569	-2.540933	-2.836751	0.741636	0.4458182	0.5937271	up
A_24_P211558	PATZ1	-0.925784	-0.120437	-0.543717	0.8053474	0.3820667	0.5937071	up
A_23_P106822	NOB1	3.730154	4.478746	4.168955	0.7485919	0.4388008	0.5936964	up
A_23_P137865	MTX1	2.9595108	3.5877633	3.518608	0.6282525	0.5590973	0.5936749	up
A_22_P00004440	lnc-COX19-2	-0.044191	0.5771847	0.5211072	0.6213756	0.5652981	0.5933368	up
A_24_P399694	ZCCHC3	1.668931	2.3688579	2.155198	0.6999269	0.4862671	0.593097	up
A_33_P3296846	TMPRSS4	3.8883848	4.358456	4.6044216	0.4700713	0.7160368	0.5930541	up
A_33_P3317850	RIOK3	4.2429724	5.1858573	4.4861317	0.9428849	0.2431593	0.5930221	up
A_33_P3214869	KPNA7	-1.191585	-0.345476	-0.851854	0.8461084	0.3397307	0.5929196	up
A_21_P0000714	SNHG16	5.5039673	5.6931357	6.500416	0.1891685	0.9964485	0.5928085	up
A_23_P82324	CARD11	0.9727387	1.5367751	1.5939789	0.5640364	0.6212401	0.5926383	up
A_23_P164258	PIPOX	-0.914931	-0.281205	-0.363413	0.6337256	0.551518	0.5926218	up
A_22_P00010013	lnc-MME-4	3.5556803	4.2367826	4.0598183	0.6811023	0.504138	0.5926202	up
A_23_P56736	TUBA3D	-0.911681	-0.262087	-0.376434	0.6495943	0.5352473	0.5924208	up
A_23_P16225	BEST2	-2.078107	-1.373633	-1.598153	0.7044735	0.4799542	0.5922139	up
A_33_P3382595	RN7SK	8.050794	8.648922	8.636951	0.5981283	0.5861578	0.5921431	up
A_33_P3352958	ENDOV	-0.376279	0.260571	0.1710625	0.6368504	0.5473418	0.5920961	up
A_33_P3366456	HDGFL1	-0.102614	0.5831966	0.3956847	0.6858106	0.4982987	0.5920546	up
A_33_P3210079	SF3A2	2.8971882	3.5353599	3.4430685	0.6381717	0.5458803	0.592026	up
A_24_P696761	LEMD1	4.4776716	4.9570785	5.1819973	0.4794068	0.7043257	0.5918663	up
A_23_P313031	LOC101930506	-0.197463	0.278553	0.5099487	0.4760156	0.7074113	0.5917134	up
A_21_P0011522	XLOC_l2_005692	1.5503445	2.7278666	1.5561228	1.1775222	0.0057783	0.5916503	up
A_33_P3356811	LCE1E	0.2167978	0.9268451	0.6899858	0.7100473	0.4731879	0.5916176	up
A_33_P3251703	CRIP1	8.514469	9.192755	9.019275	0.6782856	0.5048056	0.5915456	up
A_33_P3708364	LOC644285	6.617076	7.331631	7.0855627	0.7145553	0.4684868	0.591521	up
A_21_P0006521	LOC101928092	0.0186296	0.6699762	0.5500693	0.6513467	0.5314398	0.5913933	up
A_23_P149259	TMEM79	3.5359526	4.1285357	4.1261206	0.5925832	0.590168	0.5913756	up
A_23_P59005	TAP1	4.5710745	5.1493783	5.174753	0.5783038	0.6036787	0.5909913	up
A_23_P56170	MED16	1.4320664	2.1274981	1.9185181	0.6954317	0.4864516	0.5909417	up
A_21_P0010313	lnc-MX1-3	-2.552697	-2.027802	-1.895748	0.524895	0.6569488	0.5909219	up
A_21_P0002566	lnc-SOX11-3	1.7337103	2.3821006	2.267064	0.6483903	0.5333538	0.5908721	up
A_21_P0003710	lnc-FAM53A-1	-1.700438	-0.949352	-1.26998	0.7510858	0.4304576	0.5907717	up
A_23_P257726	C9orf156	0.8507395	1.5586343	1.324162	0.7078948	0.4734225	0.5906587	up
A_33_P3324454	TPP1	4.5396996	5.2599187	5.000656	0.7202191	0.4609566	0.5905879	up
A_33_P3369317	DNAJB5	-3.44257	-2.465347	-3.238808	0.9772229	0.2037611	0.590492	up
A_23_P51761	OR6K2	-1.88555	-1.18634	-1.403923	0.6992102	0.481627	0.5904186	up
A_22_P00023943	OXCT1-AS1	-3.290449	-2.88342	-2.516902	0.4070287	0.7735467	0.5902877	up
A_33_P3403778	ZNF579	5.1314545	5.862968	5.5804844	0.7315135	0.4490299	0.5902717	up
A_23_P89727	CTDP1	-2.224432	-1.648397	-1.619938	0.5760341	0.6044936	0.5902638	up
A_22_P00017108	EMG1	3.9057035	4.5487294	4.4430933	0.6430259	0.5373898	0.5902078	up
A_24_P307580	HTATIP2	-1.497005	-0.935257	-0.878997	0.561748	0.6180077	0.5898778	up
A_24_P11061	CSAG1	-2.071149	-1.373245	-1.589823	0.6979036	0.4813256	0.5896146	up
A_33_P3390217	TTLL9	-3.225164	-2.745521	-2.525605	0.4796436	0.6995597	0.5896016	up
A_33_P3350207	HCFC1R1	3.7704592	4.4725757	4.247509	0.7021165	0.4770498	0.5895832	up
A_33_P3379939	HLA-F	6.7691107	7.458464	7.2587433	0.6893535	0.4896326	0.589493	up
A_23_P38864	RABAC1	5.7624207	6.5259843	6.1773386	0.7635636	0.414918	0.5892408	up
A_23_P157170	DNAJC30	1.6801682	2.3576732	2.1809645	0.677505	0.5007963	0.5891507	up
A_33_P3417459	SCARNA9L	0.7602882	1.468163	1.2302761	0.7078748	0.4699879	0.5889313	up
A_22_P00017379	lnc-UVRAG-2	-0.848756	-0.030315	-0.489445	0.8184414	0.3593111	0.5888763	up
A_33_P3409261	LOC100652758	-1.521219	-0.69642	-1.168628	0.8247991	0.3525906	0.5886948	up

A_23_P109333	C21orf33	3.9993725	4.592483	4.5835648	0.5931106	0.5841923	0.5886514	up
A_23_P14515	ACOT4	0.2627831	1.0301805	0.672637	0.7673974	0.4098539	0.5886257	up
A_23_P47691	TRIM21	-1.216627	-0.943069	-0.313202	0.2735581	0.9034243	0.5884912	up
A_23_P15146	IL32	-0.079984	0.3224015	0.6944909	0.4023852	0.7744746	0.5884299	up
A_22_P00001885	lnc-B3GAT2-2	-1.420881	-0.577154	-1.087805	0.8437276	0.3330765	0.588402	up
A_22_P00002893	lnc-C4orf37-2	-2.835736	-1.761304	-2.733447	1.0744319	0.1022892	0.5883606	up
A_24_P787889	TMEM191B	4.280217	4.7379613	4.9991827	0.4577441	0.7189655	0.5883548	up
A_33_P3318027	CSNK2B	4.188493	4.849403	4.7038403	0.6609101	0.5153475	0.5881288	up
A_33_P3412295	LOC645645	-1.281351	-0.67564	-0.710821	0.6057105	0.5705295	0.58812	up
A_24_P399622	COPE	2.563116	3.1637645	3.1375399	0.6006484	0.5744238	0.5875361	up
A_21_P0009544	lnc-INO80C-1	-1.201931	-0.396834	-0.832851	0.8050971	0.3690801	0.5870886	up
A_33_P3835524	POU2F2	-2.503943	-1.80879	-2.02505	0.6951532	0.4788933	0.5870233	up
A_33_P3353030	UCN	-0.219735	0.6822724	0.0522652	0.9020071	0.2719998	0.5870035	up
A_33_P3236986	CYP17A1-AS1	0.720623	1.232892	1.382174	0.512269	0.661551	0.58691	up
A_21_P0004838	lnc-HIVEP1-2	-2.267475	-1.492226	-1.86901	0.775249	0.3984656	0.5868573	up
A_23_P20480	BRF2	-0.133771	0.6606526	0.2454462	0.7944236	0.3792172	0.5868204	up
A_23_P39550	TMEM163	-0.603252	-0.180584	0.1475258	0.422668	0.7507777	0.5867228	up
A_33_P3234580	ASS1	4.662588	5.5988116	4.8995275	0.9362235	0.2369394	0.5865815	up
A_33_P3218797	PPDPF	5.0877733	5.7955794	5.552991	0.7078061	0.4652176	0.5865119	up
A_33_P3364854	TRPV6	-2.022747	-1.094307	-1.778331	0.9284396	0.2444162	0.5864279	up
A_21_P0014499	SACS-AS1	-2.17263	-1.678007	-1.494713	0.4946232	0.6779175	0.5862703	up
A_33_P3321417	DMAP1	-0.126067	0.3685932	0.5517798	0.4946599	0.6778464	0.5862532	up
A_21_P0014511	RBFADN	-3.344211	-2.754146	-2.761816	0.5900655	0.5823956	0.5862305	up
A_23_P92132	IFRD2	3.8258	4.4845757	4.339347	0.6587758	0.5135469	0.5861614	up
A_23_P27724	SEPW1	6.948059	7.466636	7.6017094	0.5185771	0.6536503	0.5861137	up
A_23_P212475	SHISA5	6.9105444	7.3773837	7.615859	0.4668393	0.7053146	0.586077	up
A_33_P3238166	PXDN	-0.789389	-0.482781	0.0761066	0.3066073	0.8654952	0.5860512	up
A_23_P32722	ZNF408	-1.66822	-1.069345	-1.095214	0.5988751	0.5730052	0.5859401	up
A_22_P00023841	lnc-HLCS-1	-1.122523	-0.628012	-0.445415	0.4945107	0.6771078	0.5858092	up
A_33_P3376249	S100A2	6.1671724	6.6447635	6.861147	0.477591	0.6939745	0.5857828	up
A_23_P38041	NAGPA	2.632082	3.3665195	3.0689983	0.7344375	0.4369164	0.5856769	up
A_23_P16573	DDX49	3.1841917	3.814364	3.7253208	0.6301723	0.5411291	0.5856507	up
A_33_P3278144	ANKRD42	7.4760695	8.12262	8.000532	0.6465502	0.5244627	0.5855065	up
A_24_P98524	PPP3CB	0.6159453	1.2866569	1.116087	0.6707115	0.5001416	0.5854266	up
A_24_P271363	CDS2	2.1136599	3.02607	2.3720007	0.9124103	0.2583408	0.5853756	up
A_22_P00008314	LINC00466	-3.552706	-2.962784	-2.972025	0.589922	0.5806809	0.5853014	up
A_22_P00011440	CTB-178M22.2	-2.964951	-2.24427	-2.515196	0.7206807	0.449755	0.5852178	up
A_21_P0011885	XLOC_l2_007829	-0.363711	0.1862745	0.2563686	0.5499854	0.6200795	0.5850325	up
A_33_P3329839	CDK2AP2	6.245777	6.7899585	6.8715553	0.5441814	0.6257782	0.5849798	up
A_33_P3382162	RFX5	2.7084303	3.5367408	3.049961	0.8283105	0.3415308	0.5849207	up
A_33_P3359308	KLHL29	0.8715138	1.5591502	1.3535986	0.6876364	0.4820848	0.5848606	up
A_24_P49190	C17orf58	0.7383742	1.2660522	1.3803358	0.527678	0.6419616	0.5848198	up
A_22_P00010745	lnc-NIPSNAP3B-1	-1.607884	-0.946707	-1.099795	0.6611767	0.5080886	0.5846327	up
A_23_P501849	RPL13	7.5164785	8.286671	7.9146757	0.7701922	0.3981972	0.5841947	up
A_23_P132341	C22orf46	-1.474728	-0.905752	-0.875389	0.5689759	0.5993385	0.5841572	up
A_33_P3292739	USP17L2	-1.99338	-1.262552	-1.55593	0.7308278	0.4374504	0.5841391	up
A_33_P3347452	RPS6KA2	-1.255368	-0.174429	-1.168412	1.0809393	0.086956	0.5839477	up
A_24_P182281	HHLA3	-2.238233	-1.420973	-1.887939	0.8172603	0.3502946	0.5837774	up
A_21_P0012316	TMEM191B	2.9662037	3.3510098	3.7488184	0.3848062	0.7826147	0.5837104	up
A_33_P3226810	TNFSF10	0.0798645	0.2734709	1.0535192	0.1936064	0.9736548	0.5836306	up
A_24_P272061	RPL13A	9.017773	9.771254	9.431302	0.7534809	0.4135294	0.5835052	up
A_23_P34597	CDA	2.6432486	3.392612	3.060894	0.7493634	0.4176455	0.5835044	up
A_33_P3409277	LOC100128882	-1.955415	-1.331537	-1.412601	0.6238775	0.5428143	0.5833459	up
A_22_P00023909	LOC100507534	-2.7316	-2.240888	-2.055697	0.4907119	0.6759028	0.5833074	up
A_22_P00011546	lnc-PCDH12-1	-3.311455	-2.42266	-3.034056	0.8887947	0.2773986	0.5830966	up
A_33_P3663705	CROCC	-0.056081	0.5283074	0.5256014	0.5843883	0.5816822	0.5830352	up

A_21_P0010300	lnc-TSPEAR-1	-2.010182	-1.593218	-1.261244	0.4169641	0.7489386	0.5829513	up
A_23_P385771	PAOX	0.3727856	1.1389427	0.772243	0.7661572	0.3994575	0.5828073	up
A_22_P00006431	lnc-FBXO47-2	-2.69014	-1.952945	-2.262009	0.7371955	0.4281309	0.5826632	up
A_21_P0012919	XLOC_l2_012162	-3.169993	-2.471251	-2.703469	0.6987419	0.4665244	0.5826331	up
A_33_P3412722	SNX8	1.2444754	2.0637317	1.5904264	0.8192563	0.3459511	0.5826037	up
A_22_P00009163	LOC102723465	0.9502144	2.0141463	1.0514183	1.063932	0.1012039	0.582568	up
A_33_P3259135	NSG1	0.5507536	1.0963922	1.1700258	0.5456386	0.6192722	0.5824554	up
A_33_P3424207	RABEP2	4.099698	4.653382	4.7106094	0.5536838	0.6109114	0.5822976	up
A_22_P00003873	lnc-CEMP1-5	-3.440023	-2.43642	-3.279141	1.0036035	0.1608818	0.5822426	up
A_22_P00022656	LOC101927490	-1.792944	-0.709622	-1.711918	1.083322	0.0810256	0.5821738	up
A_22_P00006290	RAD51-AS1	1.2859192	1.9430327	1.7931023	0.6571136	0.5071831	0.5821483	up
A_23_P57110	SLC52A3	0.2862802	0.8083363	0.9279895	0.5220561	0.6417093	0.5818827	up
A_23_P6196	SCAND1	1.9718237	2.562799	2.5443764	0.5909753	0.5725527	0.581764	up
A_23_P145218	BTN2A3P	0.0414004	0.7433047	0.502852	0.7019043	0.4614515	0.5816779	up
A_33_P3338152	HIF3A	4.8373585	5.553308	5.2845592	0.7159495	0.4472008	0.5815752	up
A_23_P108514	STK16	1.4973202	2.1219344	2.0353594	0.6246142	0.5380392	0.5813267	up
A_23_P106299	SERF2	6.945036	7.6222377	7.430418	0.6772018	0.4853821	0.5812919	up
A_33_P3258362	HBA2	-1.640625	-0.993641	-1.125202	0.6469841	0.5154233	0.5812037	up
A_23_P21838	CNP	5.364788	5.8384786	6.0534706	0.4736905	0.6886826	0.5811865	up
A_21_P0005534	lnc-MRPS33-1	-1.318047	-0.811498	-0.662981	0.5065489	0.655066	0.5808075	up
A_23_P124044	DEAF1	1.842083	2.4807153	2.364828	0.6386323	0.5227451	0.5806887	up
A_24_P105933	VIPR1	0.3784747	1.2391763	0.6790767	0.8607016	0.300602	0.5806518	up
A_33_P3312754	LOC102467146	1.9576292	2.574329	2.5021486	0.6166997	0.5445194	0.5806096	up
A_33_P3417222	CD72	5.7417107	6.378034	6.2664423	0.6363235	0.5247316	0.5805275	up
A_21_P0014233	LOC102723989	-3.205695	-2.440808	-2.809535	0.7648873	0.3961601	0.5805237	up
A_23_P164341	VAMP2	2.8012915	3.3886323	3.3749943	0.5873408	0.5737028	0.5805218	up
A_33_P3320748	FDX1L	3.6052542	4.246986	4.124485	0.6417317	0.5192308	0.5804813	up
A_33_P3867461	TEX41	7.067272	7.6786275	7.616272	0.6113553	0.5489998	0.5801776	up
A_23_P165090	NDUFS7	4.1805687	4.767716	4.753667	0.5871472	0.5730982	0.5801227	up
A_21_P0005693	LOC101929217	-2.249865	-1.673081	-1.666414	0.5767841	0.5834513	0.5801177	up
A_33_P3396370	ACRBP	-2.735973	-1.740316	-2.571649	0.9956567	0.1643236	0.5799901	up
A_33_P3327519	SNORA74A	0.2657976	0.8579507	0.8336105	0.5921531	0.5678129	0.579983	up
A_21_P0000220	SNORD33	1.0276685	1.7280164	1.4872785	0.7003479	0.45961	0.579979	up
A_19_P00319800	LOC100287225	-1.990014	-1.409327	-1.41167	0.5806866	0.5783439	0.5795152	up
A_33_P3221064	LTBP4	4.474387	5.187864	4.9195795	0.7134767	0.4451923	0.5793345	up
A_24_P306443	LOC100233156	-0.933445	-0.398695	-0.309621	0.53475	0.6238246	0.5792873	up
A_33_P3274164	DOT1L	0.1575036	0.7155414	0.7577443	0.5580378	0.6002407	0.5791392	up
A_23_P50815	TTYH1	2.4526634	2.9236188	3.139494	0.4709554	0.6868305	0.5788929	up
A_23_P111188	ZBTB22	-3.197384	-2.519618	-2.717788	0.6777663	0.4795964	0.5786813	up
A_22_P00014899	lnc-SLC9A1-1	-1.172617	-0.53374	-0.65421	0.6388774	0.5184078	0.5786426	up
A_33_P3337627	TRPC6	2.6166906	3.3502374	3.0400858	0.7335467	0.4233952	0.5784709	up
A_33_P3218356	TMEM167B	0.7057948	1.4289484	1.1395483	0.7231536	0.4337535	0.5784536	up
A_33_P3413759	ACER2	-3.016569	-2.505978	-2.370331	0.5105913	0.6462379	0.5784146	up
A_33_P3407636	YWHAE	-0.590609	0.0169463	-0.041438	0.6075549	0.5491705	0.5783627	up
A_33_P3292896	SFXN5	1.0162549	1.702426	1.4862332	0.6861711	0.4699783	0.5780747	up
A_32_P9575	MRPL45	2.2149448	3.0816245	2.5042677	0.8666797	0.2893229	0.5780013	up
A_22_P00003397	LOC102725059	-1.690994	-1.152006	-1.075649	0.5389881	0.6153455	0.5771668	up
A_23_P75800	RAB3IL1	-1.530954	-0.915807	-0.991825	0.6151476	0.5391297	0.5771387	up
A_33_P3879920	TRIM39-RPP21	5.6226273	6.211695	6.1877384	0.5890679	0.5651112	0.5770896	up
A_33_P3336720	HAMP	-1.234575	-0.476565	-0.838416	0.7580094	0.3961592	0.5770843	up
A_23_P112397	FBXW5	4.164591	4.8788943	4.6042204	0.7143035	0.4396296	0.5769665	up
A_21_P0000760	TMPRSS4-AS1	3.466979	4.1475186	3.9402094	0.6805396	0.4732304	0.576885	up
A_22_P00007897	lnc-HSP90AA1-2	3.494625	4.1076274	4.0350714	0.6130023	0.5404463	0.5767243	up
A_33_P3414930	LOC101927202	-2.8553	-2.466102	-2.091067	0.3891983	0.7642334	0.5767158	up
A_33_P3236563	ALDH3B1	-0.578105	0.1943617	-0.198019	0.7724671	0.3800869	0.576277	up
A_21_P0011850	XLOC_l2_007543	-0.896422	-0.109181	-0.531159	0.787241	0.3652625	0.5762518	up

A_22_P00008004	MIR31HG	2.8592958	3.5243068	3.3467007	0.6650109	0.4874048	0.5762079	up
A_33_P3422133	ADAP1	4.1741867	4.728251	4.772485	0.5540643	0.5982981	0.5761812	up
A_22_P00017815	LINC00638	2.7245283	3.293284	3.3081255	0.5687556	0.5835972	0.5761764	up
A_23_P55926	NR1H2	1.3831716	1.9982104	1.9203062	0.6150389	0.5371347	0.5760868	up
A_23_P77415	OSGIN1	-0.165902	0.7997241	0.0201669	0.9656262	0.186069	0.5758476	up
A_21_P0010768	LOC102723908	-3.185345	-2.720666	-2.498524	0.4646797	0.6868217	0.5757507	up
A_24_P358164	RPSAP9	6.89316	7.60883	7.328969	0.7156701	0.4358091	0.5757396	up
A_23_P158533	DEAF1	0.6678019	1.3413224	1.1455135	0.6735206	0.4777117	0.5756161	up
A_33_P3358601	IFITM10	-1.715601	-0.466513	-1.813729	1.2490883	-0.098128	0.57548	up
A_33_P3297244	GAS2L2	-2.694724	-2.211166	-2.027527	0.4835587	0.6671975	0.5753781	up
A_33_P3416503	NUDT8	1.338315	1.8828659	1.9444046	0.5445509	0.6060896	0.5753203	up
A_24_P384397	RAVER1	3.4877043	4.170074	3.9559622	0.6823697	0.4682579	0.5753138	up
A_23_P124642	RASGRP1	1.9234934	2.4672627	2.5303059	0.5437694	0.6068125	0.5752909	up
A_23_P92730	HSPB3	2.6548977	3.3102193	3.1501293	0.6553216	0.4952316	0.5752766	up
A_24_P358591	C2orf70	0.1796799	0.7878437	0.7217832	0.6081638	0.5421033	0.5751336	up
A_33_P3234794	ARHGAP40	-2.143533	-1.605266	-1.53156	0.5382676	0.6119728	0.5751202	up
A_21_P0011762	LOC284379	-2.551681	-1.93489	-2.018597	0.6167905	0.5330842	0.5749373	up
A_23_P13713	PRPH	-1.314614	-0.226832	-1.252806	1.0877824	0.0618081	0.5747953	up
A_33_P3300817	RPS5	9.599626	10.298347	10.050352	0.6987219	0.4507265	0.5747242	up
A_23_P32279	BARX1	-3.24066	-2.396259	-2.935628	0.8444009	0.305032	0.5747165	up
A_23_P47304	CASP5	3.5153675	4.2165713	3.9632292	0.7012038	0.4478617	0.5745327	up
A_23_P113317	P4HTM	0.9125233	1.7440128	1.2300148	0.8314896	0.3174915	0.5744905	up
A_23_P164623	ZBTB45	4.166893	4.7369666	4.7457037	0.5700736	0.5788107	0.5744422	up
A_24_P406814	FAM53B	2.6515894	3.2115388	3.240487	0.5599494	0.5888977	0.5744236	up
A_21_P0002606	lnc-FOXD4L1-1	-3.066539	-2.460238	-2.524091	0.6063011	0.5424488	0.5743749	up
A_21_P0000174	C5orf56	-3.062495	-2.561641	-2.414701	0.5008543	0.6477943	0.5743243	up
A_33_P3330209	AUTS2	-2.921567	-2.465915	-2.228932	0.4556515	0.6926348	0.5741432	up
A_33_P3489675	ARHGAP8	0.8726983	1.5485215	1.3451362	0.6758232	0.4724379	0.5741305	up
A_23_P411296	CEBPB	4.828351	5.8899426	4.9145193	1.0615916	0.0861683	0.5738799	up
A_23_P164999	CDC34	2.9623842	3.4185033	3.6536531	0.4561191	0.6912689	0.573694	up
A_23_P88781	CIAPIN1	3.5641928	4.270944	4.004711	0.7067514	0.4405184	0.5736349	up
A_21_P0000313	SNORA15	0.3185854	0.9020572	0.8821778	0.5834718	0.5635924	0.5735321	up
A_22_P00025286	COQ9	4.4409065	5.019134	5.009516	0.5782275	0.5686092	0.5734184	up
A_23_P149626	PLEKHG5	0.1502109	0.7426639	0.7044678	0.592453	0.5542569	0.573355	up
A_24_P188377	CD55	2.7948923	3.8315558	2.9047632	1.0366635	0.1098709	0.5732672	up
A_23_P427703	MT1L	4.5625935	5.1041703	5.167548	0.5415769	0.6049547	0.5732658	up
A_22_P00023588	CECR3	-0.369239	0.2554603	0.1520014	0.6246996	0.5212407	0.5729702	up
A_23_P500206	IL17RE	0.2515712	0.8317232	0.8172479	0.580152	0.5656767	0.5729144	up
A_33_P3420852	KIRREL2	2.2544184	2.9305205	2.7237968	0.6761022	0.4693785	0.5727403	up
A_33_P3373185	LINC00999	3.16749	3.8049283	3.6755037	0.6374383	0.5080137	0.572726	up
A_33_P3370930	LAMB1	-0.68088	-0.109077	-0.107697	0.5718026	0.5731831	0.5724928	up
A_24_P229884	SMIM22	5.501564	6.130464	6.0167866	0.6289001	0.5152226	0.5720613	up
A_22_P00021588	LOC101930532	-2.558931	-1.94259	-2.031383	0.6163411	0.5275488	0.571945	up
A_22_P00002805	PCBP1-AS1	5.4529505	6.0846915	5.9650764	0.6317411	0.512126	0.5719335	up
A_22_P00008813	LOC102725022	2.6898737	3.3847823	3.138733	0.6949086	0.4488592	0.5718839	up
A_24_P412734	PRSS36	3.355463	4.082418	3.772132	0.7269549	0.4166689	0.5718119	up
A_23_P167168	IGJ	-2.98325	-2.524227	-2.298675	0.4590232	0.6845753	0.5717993	up
A_23_P416314	HRASLS5	5.0750065	5.815188	5.478382	0.7401815	0.4033756	0.5717785	up
A_23_P145935	EPHB6	1.0747275	1.5088143	1.7839007	0.4340868	0.7091732	0.57163	up
A_22_P00025112	lnc-OPN4-2	3.729578	4.4611945	4.1411495	0.7316165	0.4115715	0.571594	up
A_23_P153360	SMIM7	2.6135092	3.4268394	2.943184	0.8133302	0.3296747	0.5715025	up
A_23_P32064	NSMF	1.5616245	2.1332288	2.133009	0.5716043	0.5713844	0.5714943	up
A_24_P766208	RPL3	8.539694	9.344187	8.877587	0.804493	0.3378935	0.5711932	up
A_23_P257609	RPL29	6.338602	7.0084987	6.8110256	0.6698966	0.4724236	0.5711601	up
A_22_P00007179	lnc-GNRHR2-1	-2.766475	-2.710177	-1.680629	0.056299	1.0858462	0.5710726	up
A_33_P3276813	EYS	-0.215393	0.5178399	0.1930695	0.733233	0.4084625	0.5708478	up

A_23_P90143	RPL13A	9.858478	10.588263	10.270348	0.729785	0.41187	0.5708275	up
A_23_P255884	GSN	3.8984156	4.5815434	4.3567553	0.6831279	0.4583397	0.5707338	up
A_33_P3252588	LOC100130093	-1.534208	-0.639415	-1.287699	0.894793	0.2465091	0.5706511	up
A_23_P338890	PTPN1	2.9827442	3.4694219	3.6368303	0.4866777	0.6540861	0.5703819	up
A_33_P3322428	MARK4	-2.020427	-1.518989	-1.381784	0.5014381	0.6386423	0.5700402	up
A_23_P303181	SPHK2	3.7737894	4.4582343	4.2288895	0.6844449	0.4551001	0.5697725	up
A_33_P3293396	LINGO4	-0.281124	0.369163	0.2078929	0.6502872	0.489017	0.5696521	up
A_33_P3334180	PLCH2	-1.960818	-1.46178	-1.320935	0.4990382	0.639883	0.5694606	up
A_33_P3275668	BAG6	6.371332	7.0481896	6.833355	0.6768575	0.4620228	0.5694401	up
A_24_P128683	CNPPD1	0.94209	1.4975705	1.5251384	0.5554805	0.5830483	0.5692644	up
A_23_P14915	CSNK2A2	1.7938576	2.594111	2.1321058	0.8002534	0.3382483	0.5692508	up
A_19_P00319372	LINC00969	-0.011757	0.5940352	0.520915	0.6057921	0.5326719	0.569232	up
A_33_P3340060	RPL13A	8.433098	9.147936	8.856643	0.714838	0.4235449	0.5691914	up
A_21_P0011822	lnc-ZAP70-2	-1.030314	-0.442983	-0.479308	0.5873313	0.5510063	0.5691688	up
A_21_P0011433	BCAP31	6.1310234	6.753053	6.646886	0.6220298	0.5158625	0.5689461	up
A_23_P216610	SUSD1	-0.229671	0.469327	0.209177	0.6989975	0.4388475	0.5689225	up
A_23_P84219	LIPH	3.683422	4.047584	4.4564466	0.364162	0.7730246	0.5685933	up
A_19_P00803441	LOC100499194	-2.31821	-1.629513	-1.869935	0.6886973	0.4482756	0.5684865	up
A_21_P0000299	SNORA19	2.152606	2.7312942	2.7107563	0.5786881	0.5581503	0.5684192	up
A_24_P92367	YDJC	3.872345	4.4296174	4.4518433	0.5572724	0.5794983	0.5683854	up
A_23_P41888	PHYKPL	0.0458717	1.0342627	0.1938171	0.9883909	0.1479454	0.5681682	up
A_23_P321501	DHRS2	3.6285858	3.9479442	4.445238	0.3193584	0.8166523	0.5680053	up
A_24_P253215	EMG1	4.0861387	4.6686435	4.6395826	0.5825048	0.5534439	0.5679743	up
A_22_P00004511	lnc-CPT2-6	-2.371294	-1.663541	-1.943172	0.7077532	0.4281225	0.5679379	up
A_23_P155477	C3orf18	-0.069832	0.4305902	0.5649824	0.500422	0.6348143	0.5676181	up
A_33_P3213006	KRTAP1-4	5.082196	5.7890162	5.510606	0.70682	0.4284096	0.5676148	up
A_33_P3367396	FAM177B	-2.465633	-1.478573	-2.317489	0.9870596	0.1481443	0.5676019	up
A_33_P3788618	ACER2	-2.37	-1.740721	-1.864176	0.6292791	0.5058241	0.5675516	up
A_22_P00022787	LOC101929181	-0.819714	-0.197025	-0.307362	0.6226888	0.5123525	0.5675206	up
A_33_P3331882	CRTC1	1.3057027	1.9446568	1.8017178	0.6389542	0.4960151	0.5674846	up
A_33_P3373850	WDR24	1.791686	2.1196465	2.5985126	0.3279605	0.8068266	0.5673936	up
A_22_P00007260	lnc-GPR144-2	-2.952586	-2.104048	-2.66642	0.8485382	0.286166	0.5673521	up
A_22_P00009011	lnc-LAT2-1	-0.905094	-0.243661	-0.431936	0.6614332	0.4731584	0.5672958	up
A_33_P3265301	GJD3	-3.09759	-2.806379	-2.254301	0.2912111	0.8432889	0.56725	up
A_33_P3276100	URAHP	-1.815366	-0.877346	-1.618982	0.9380202	0.1963844	0.5672023	up
A_22_P00010810	lnc-NMNAT1-2	0.6742811	1.2413406	1.2412844	0.5670595	0.5670033	0.5670314	up
A_23_P57137	AP5S1	1.4770241	2.0830388	2.0046177	0.6060147	0.5275936	0.5668042	up
A_23_P113034	C10orf11	-1.494204	-0.840834	-1.014387	0.6533699	0.4798169	0.5665934	up
A_23_P67952	MYCNOS	0.9709401	1.5802345	1.4948287	0.6092944	0.5238886	0.5665915	up
A_33_P3381899	SYNGR3	-0.59291	-0.012899	-0.039797	0.5800109	0.553113	0.566562	up
A_23_P146187	RRS1	3.1716623	3.575148	3.90059	0.4034858	0.7289276	0.5662067	up
A_23_P17287	IAH1	4.380253	4.856088	5.036339	0.4758353	0.656086	0.5659606	up
A_33_P3389336	LOC101929918	-3.074256	-2.515379	-2.50123	0.558877	0.5730259	0.5659515	up
A_33_P3322864	HES6	-1.665105	-1.085635	-1.112979	0.5794702	0.5521259	0.565798	up
A_33_P3279708	RNU2-1	4.017192	4.812552	4.3534193	0.7953601	0.3362274	0.5657938	up
A_33_P3366039	RPL29	4.5017195	5.122767	5.0122395	0.6210475	0.51052	0.5657838	up
A_21_P0002932	lnc-FBLN2-1	2.1706972	2.8346515	2.6381779	0.6639543	0.4674807	0.5657175	up
A_33_P3210647	COL16A1	-0.079965	0.489605	0.4815826	0.5695701	0.5615478	0.5655589	up
A_33_P3398946	IRF2BP1	-1.97731	-1.408788	-1.414775	0.5685225	0.5625353	0.5655289	up
A_33_P3285456	C1orf68	-3.214458	-2.645301	-2.653339	0.5691569	0.5611188	0.5651379	up
A_33_P3220853	XLOC_l2_007738	-3.206358	-2.417877	-2.864582	0.7884812	0.3417769	0.565129	up
A_24_P250227	NR1D1	0.7476282	1.6729088	0.9525943	0.9252806	0.2049661	0.5651233	up
A_32_P204019	RPL29	7.374386	8.0321045	7.8465786	0.6577187	0.4721928	0.5649557	up
A_33_P3226542	SNORD3B-1	5.794029	6.4009786	6.316926	0.6069493	0.5228968	0.564923	up
A_24_P272290	PXDC1	-3.00925	-2.359803	-2.529457	0.6494465	0.4797924	0.5646194	up
A_33_P3215864	FAM3A	-0.405574	0.3878198	-0.070269	0.7933936	0.3353052	0.5643494	up

A_21_P0013244	DTX2	-2.343082	-1.757034	-1.800499	0.5860486	0.542583	0.5643158	up
A_23_P118462	OVCA2	5.56279	6.192284	6.061186	0.6294942	0.4983959	0.5639451	up
A_23_P11629	TMEM61	-1.278264	-0.804122	-0.624594	0.4741421	0.6536698	0.563906	up
A_23_P166336	TMEM191A	5.573821	5.93026	6.344775	0.3564391	0.7709541	0.5636966	up
A_23_P206454	CENPBD1	1.301105	1.8870792	1.8424196	0.5859742	0.5413146	0.5636444	up
A_33_P3378126	FBXO32	1.2144575	2.0516276	1.5045457	0.8371701	0.2900882	0.5636291	up
A_33_P3294881	CCDC174	2.5292377	3.3351846	2.8499155	0.8059468	0.3206778	0.5633123	up
A_24_P328231	CPSF3L	-1.365269	-0.752308	-0.851676	0.6129608	0.5135927	0.5632768	up
A_23_P134347	CPVL	-3.61799	-3.230062	-2.879638	0.3879278	0.7383518	0.5631398	up
A_24_P365901	TSPAN33	-0.97473	-0.441952	-0.3813	0.5327783	0.59343	0.5631042	up
A_23_P71790	MAMDC4	-1.85101	-1.201328	-1.374558	0.6496825	0.4764519	0.5630672	up
A_21_P0009120	LINC01569	-2.684944	-1.765463	-2.478364	0.9194815	0.2065804	0.563031	up
A_24_P322709	SNTA1	-1.651162	-0.878453	-1.298163	0.7727094	0.3529987	0.5628541	up
A_24_P31003	GFER	1.0975142	1.7690191	1.5515919	0.671505	0.4540777	0.5627914	up
A_21_P0014422	LOC646513	2.9952812	3.6267161	3.4893265	0.6314349	0.4940453	0.5627401	up
A_21_P0014577	LOC100506869	-2.826881	-2.090992	-2.437629	0.7358894	0.3892524	0.5625709	up
A_33_P3332382	LOC100129406	-0.132362	0.5228114	0.3373265	0.6551733	0.4696884	0.5624309	up
A_23_P431939	MR1	1.3325338	2.1865535	1.6028123	0.8540196	0.2702785	0.562149	up
A_23_P143414	ROMO1	6.5094156	6.9989753	7.1440363	0.4895597	0.6346207	0.5620902	up
A_33_P3402304	ZBTB7C	1.0150251	1.8409953	1.313107	0.8259702	0.2980819	0.562026	up
A_33_P3344276	HS1BP3	-1.845618	-1.457245	-1.110193	0.3883729	0.735425	0.561899	up
A_19_P00807869	XLOC_l2_009886	-2.452562	-1.788353	-1.993183	0.6642091	0.4593794	0.5617943	up
A_33_P3380652	ADAM28	-3.051173	-2.116867	-2.862039	0.9343066	0.1891344	0.5617205	up
A_24_P85850	BNIPL	-3.105816	-2.303338	-2.784855	0.8024778	0.3209605	0.5617192	up
A_23_P104323	MGMT	3.122985	3.6129355	3.7560463	0.4899507	0.6330614	0.561506	up
A_21_P0007502	LINC00173	-0.318357	0.2058096	0.2802396	0.5241666	0.5985966	0.5613816	up
A_23_P151497	TRMT61A	0.8862953	1.5011907	1.3938503	0.6148953	0.507555	0.5612252	up
A_23_P129486	MSRB1	3.0545263	3.6935563	3.5374994	0.63903	0.4829731	0.5610016	up
A_22_P00006287	CYP1B1-AS1	-2.151393	-1.270123	-1.910973	0.8812699	0.2404194	0.5608447	up
A_23_P24004	IFIT2	2.115055	2.696775	2.654828	0.5817199	0.539773	0.5607465	up
A_21_P0000033	ANKRD66	2.3048277	2.8985267	2.832428	0.593699	0.5276003	0.5606497	up
A_22_P00012938	lnc-RCSD1-1	-1.419985	-0.937594	-0.781313	0.4823909	0.6386724	0.5605316	up
A_22_P00007049	lnc-GK-3	-0.959303	-0.505711	-0.291834	0.4535928	0.6674695	0.5605311	up
A_22_P00001271	lnc-ANKRD54-1	-3.403781	-3.273685	-2.413052	0.1300955	0.9907289	0.5604122	up
A_24_P311926	HLA-G	7.8037605	8.4218025	8.306181	0.618042	0.5024204	0.5602312	up
A_23_P385206	STX12	1.4837728	1.9368148	2.1511402	0.453042	0.6673675	0.5602047	up
A_22_P00017498	lnc-VWF-2	7.559119	8.270338	7.9681225	0.7112188	0.4090033	0.560111	up
A_21_P0013833	XLOC_l2_015827	-1.926132	-1.434075	-1.298035	0.4920564	0.6280966	0.5600765	up
A_33_P3255949	TYMP	8.408154	9.093151	8.843072	0.6849976	0.4349184	0.559958	up
A_21_P0013605	HMCN2	-2.597597	-2.233776	-1.841566	0.3638213	0.7560315	0.5599264	up
A_22_P00020866	LOC101928850	-2.75342	-2.453156	-1.933918	0.3002641	0.8195019	0.559883	up
A_23_P150510	MED19	3.472825	4.1281075	3.9372797	0.6552825	0.4644547	0.5598686	up
A_32_P101860	TMEM106A	-0.808842	-0.204668	-0.293325	0.6041746	0.5155172	0.5598459	up
A_33_P3385101	TOLLIP	4.3079166	4.87684	4.8580723	0.5689235	0.5501556	0.5595396	up
A_22_P00005332	lnc-DOLPP1-1	4.0340014	4.7278705	4.4591627	0.6938691	0.4251614	0.5595152	up
A_33_P3395074	PLSCR3	0.356616	1.12114	0.7109828	0.764524	0.3543668	0.5594454	up
A_23_P65618	TGM1	0.3329501	1.176971	0.6071348	0.8440208	0.2741847	0.5591028	up
A_33_P3415491	WDR90	1.3227391	1.8880134	1.8755074	0.5652742	0.5527682	0.5590212	up
A_23_P75380	AIP	4.200019	4.80131	4.716769	0.6012912	0.5167503	0.5590208	up
A_23_P142631	FKBP1B	-0.274623	-0.028944	0.5975313	0.2456789	0.8721547	0.5589168	up
A_23_P373568	FUZ	-1.208889	-0.484064	-0.815885	0.7248254	0.3930039	0.5589147	up
A_33_P3379726	CCDC106	1.5783052	2.139526	2.134348	0.5612207	0.5560427	0.5586317	up
A_23_P8083	LY6G6C	0.371201	1.1757927	0.6838336	0.8045917	0.3126326	0.5586121	up
A_21_P0000215	SNORD55	0.282877	0.8666697	0.8157449	0.5837927	0.5328679	0.5583303	up
A_23_P115091	RAB25	3.7680101	4.282269	4.370331	0.5142589	0.6023207	0.5582898	up
A_23_P502312	CD97	5.296874	5.8918624	5.8182774	0.5949884	0.5214033	0.5581958	up

A_23_P134684	HMBOX1	1.8862543	2.7896323	2.0991812	0.903378	0.2129269	0.5581524	up
A_24_P329795	C10orf10	0.4077234	1.1310239	0.8007073	0.7233005	0.3929839	0.5581422	up
A_33_P3232980	MARK4	-2.079495	-1.454044	-1.588673	0.6254511	0.4908223	0.5581367	up
A_22_P00009574	LOC100506113	0.9153171	1.6860967	1.2607498	0.7707796	0.3454328	0.5581062	up
A_19_P00316701	SNHG5	6.0588894	6.9442325	6.289753	0.8853431	0.2308636	0.5581033	up
A_23_P92552	GATB	1.2199368	1.8215322	1.734417	0.6015954	0.5144801	0.5580378	up
A_23_P120435	WFDC3	0.4114828	0.9235964	1.0152626	0.5121136	0.6037798	0.5579467	up
A_21_P0009382	LINC01563	3.6737175	4.3106503	4.152508	0.6369329	0.4787903	0.5578616	up
A_21_P0000221	SNORD32A	0.5626054	1.0892868	1.1516376	0.5266814	0.5890322	0.5578568	up
A_23_P143569	DGCR6L	2.61438	3.1115851	3.2327385	0.4972053	0.6183586	0.5577819	up
A_32_P497434	lnc-ADD1-1	-3.518425	-2.686865	-3.234464	0.8315601	0.2839613	0.5577607	up
A_24_P93896	CNNM2	-2.514159	-1.765451	-2.147754	0.7487085	0.3664057	0.5575571	up
A_32_P54553	USP41	-0.163903	0.1743169	0.6128535	0.3382201	0.7767568	0.5574884	up
A_22_P00003472	LOC102031319	6.407222	6.9988666	6.9305344	0.5916448	0.5233126	0.5574787	up
A_23_P216052	FAM83A	-1.292724	-0.786922	-0.683945	0.5058022	0.6087785	0.5572903	up
A_33_P3309911	PRAMEF5	-2.552594	-1.941352	-2.049386	0.6112413	0.5032082	0.5572248	up
A_24_P63563	KIAA0141	2.7025108	3.4898324	3.0295057	0.7873216	0.3269949	0.5571582	up
A_33_P3217517	EXOC3-AS1	-0.942395	-0.501431	-0.269717	0.4409647	0.6726785	0.5568216	up
A_21_P0000386	SNORD89	0.0759578	0.7748199	0.4897256	0.6988621	0.4137678	0.556315	up
A_19_P00800638	LINC01088	3.590621	4.2219625	4.0718746	0.6313415	0.4812536	0.5562975	up
A_23_P36700	TAPBPL	-1.688077	-1.101	-1.162897	0.5870771	0.5251799	0.5561285	up
A_23_P360240	MYEOV	4.0393963	4.8163924	4.374646	0.7769961	0.3352499	0.556123	up
A_19_P00807507	lnc-MRGPRF-4	-3.022093	-2.393096	-2.53916	0.6289966	0.4829333	0.5559649	up
A_33_P3726064	SPATA3-AS1	-2.255052	-1.755133	-1.643127	0.4999185	0.6119247	0.5559216	up
A_21_P0002201	lnc-GALM-2	-1.698415	-1.210382	-1.074701	0.4880333	0.6237145	0.5558739	up
A_23_P360964	DACT3	-2.135391	-1.306627	-1.852572	0.828764	0.2828193	0.5557916	up
A_22_P00019759	LOC102724050	-3.285508	-2.536979	-2.92253	0.7485292	0.3629787	0.555754	up
A_23_P37983	MT1B	4.3435307	4.872458	4.9261	0.5289273	0.5825691	0.5557482	up
A_33_P3270317	SLC18A3	-0.57996	-0.048269	-0.000338	0.5316911	0.5796223	0.5556567	up
A_33_P3499102	RBM10	1.1262374	1.677547	1.6861544	0.5513096	0.559917	0.5556133	up
A_23_P7896	DUSP22	-0.376354	0.2387786	0.1193519	0.6151323	0.4957056	0.555419	up
A_24_P912048	MGC50722	-1.676017	-0.821436	-1.419883	0.8545814	0.256134	0.5553577	up
A_33_P3385488	RPS18	8.041402	8.74848	8.44494	0.707078	0.4035378	0.5553079	up
A_33_P3363938	NAP1L4	-2.272569	-1.462933	-1.971732	0.8096361	0.300837	0.5552366	up
A_23_P327483	SPATA6	-2.587443	-1.902383	-2.162117	0.6850605	0.4253268	0.5551937	up
A_22_P00001320	ANTXRL	6.4367685	7.1205573	6.863264	0.6837888	0.4264956	0.5551422	up
A_23_P11984	SLC6A9	-1.493098	-0.571788	-1.304488	0.92131	0.1886101	0.55496	up
A_23_P119295	ECSIT	4.435954	5.108669	4.8727226	0.6727147	0.4367685	0.5547416	up
A_24_P228717	RAC2	0.5722599	1.1187811	1.1349983	0.5465212	0.5627384	0.5546298	up
A_23_P112026	IDO1	-2.566513	-2.035457	-1.988381	0.5310566	0.5781324	0.5545945	up
A_24_P270814	CRK	-0.247385	0.3261576	0.2878161	0.5735421	0.5352006	0.5543714	up
A_23_P343963	FAM83F	2.4291658	3.0048804	2.961832	0.5757146	0.5326662	0.5541904	up
A_22_P00021468	lnc-USP24-1	-2.275727	-1.655917	-1.787373	0.6198101	0.4883547	0.5540824	up
A_33_P3248610	ASXL2	-3.063364	-2.374633	-2.644144	0.688731	0.41922	0.5539755	up
A_21_P0011306	SYNGR2	5.9507685	6.3690877	6.6402826	0.4183192	0.6895142	0.5539167	up
A_23_P29153	RSPH14	-1.653162	-1.488031	-0.710503	0.1651306	0.9426584	0.5538945	up
A_24_P247273	PRR33	-1.101132	-0.762616	-0.332242	0.3385158	0.7688899	0.5537028	up
A_33_P3344648	LOC440461	-1.732268	-1.297392	-1.059804	0.4348764	0.6724649	0.5536706	up
A_33_P3348747	DSG3	3.4488316	3.9467883	4.058035	0.4979568	0.6092033	0.55358	up
A_24_P207139	PML	0.6533952	0.9502454	1.4636955	0.2968502	0.8103004	0.5535753	up
A_22_P00010555	lnc-NCOA5-1	-0.890211	-0.186946	-0.48639	0.7032647	0.403821	0.5535429	up
A_22_P00005682	lnc-EIF6-1	-0.393707	0.4360828	-0.116982	0.8297896	0.2767253	0.5532575	up
A_23_P37877	NARFL	1.4520693	1.9336076	2.0768967	0.4815383	0.6248274	0.5531829	up
A_33_P3383866	TREX1	2.433362	2.8650002	3.1079035	0.4316382	0.6745415	0.5530899	up
A_22_P00024345	LINC01269	-0.468671	0.2613483	-0.093181	0.7300191	0.3754902	0.5527547	up
A_33_P3238521	LINC01000	3.5970554	4.248466	4.0510015	0.6514106	0.4539461	0.5526784	up

A_33_P3303031	LOC643549	6.787862	7.421122	7.2599297	0.6332603	0.4720678	0.552664	up
A_22_P00013474	LOC102724940	-0.95351	-0.313356	-0.488516	0.6401534	0.4649935	0.5525734	up
A_33_P3280950	A2M-AS1	-2.212755	-1.733013	-1.587354	0.4797416	0.6254005	0.5525711	up
A_22_P00002558	LOC101928343	-1.919802	-1.278415	-1.456056	0.6413865	0.4637461	0.5525663	up
A_33_P3382423	ZNF428	-0.275884	0.1956253	0.3577123	0.4715095	0.6335964	0.5525529	up
A_33_P3329549	FBRS	1.9488678	2.580274	2.4224815	0.6314063	0.4736137	0.55251	up
A_33_P3266080	TAF6L	3.6152296	4.2232385	4.111699	0.6080089	0.4964695	0.5522392	up
A_21_P0000378	SNORD66	-1.621646	-0.889892	-1.249052	0.7317543	0.3725948	0.5521746	up
A_33_P3402489	OAS3	-2.21568	-1.897456	-1.429575	0.318224	0.7861042	0.5521641	up
A_21_P0000525	LOC285847	-2.236401	-1.740175	-1.628766	0.4962258	0.607635	0.5519304	up
A_33_P3244283	CAMK2N2	2.9362078	3.5391068	3.4370737	0.6028991	0.5008659	0.5518825	up
A_22_P00012456	LINC00176	-2.409959	-1.775908	-1.940378	0.6340509	0.4695811	0.551816	up
A_19_P00320603	lnc-SNCA-2	-3.153231	-2.481715	-2.721132	0.6715164	0.4320991	0.5518078	up
A_32_P162004	RPL29	7.728427	8.343384	8.217039	0.6149569	0.4886122	0.5517845	up
A_23_P147109	TOR4A	3.3390942	3.858437	3.923233	0.5193429	0.5841389	0.5517409	up
A_33_P3538104	CASC22	-2.901078	-2.319309	-2.379586	0.5817688	0.521492	0.5516304	up
A_21_P0006850	lnc-LYZL1-2	-0.456146	0.123394	0.0675669	0.5795398	0.5237126	0.5516262	up
A_19_P00325604	LINC-ROR	-0.309292	0.2197132	0.2645316	0.5290055	0.5738239	0.5514147	up
A_33_P3322373	GBGT1	-0.916844	-0.244545	-0.486504	0.6722984	0.4303403	0.5513194	up
A_23_P150407	CREB3L1	1.7772646	2.3665614	2.2905254	0.5892968	0.5132608	0.5512788	up
A_24_P210082	C11orf71	1.322578	1.8987188	1.8488264	0.5761409	0.5262485	0.5511947	up
A_23_P393758	MVB12A	1.2738061	1.8459044	1.8039489	0.5720983	0.5301428	0.5511205	up
A_24_P48204	SECTM1	-0.215535	0.1699238	0.5005789	0.385459	0.716114	0.5507865	up
A_33_P3327270	TMED7-TICAM2	2.438078	3.0697246	2.907915	0.6316466	0.4698372	0.5507419	up
A_23_P143348	OVOL2	3.5105562	4.2392154	3.8829756	0.7286592	0.3724194	0.5505393	up
A_23_P74526	PPIE	2.430377	3.0776677	2.883894	0.6472907	0.453517	0.5504038	up
A_23_P112798	CRIP2	2.9007215	3.455337	3.4467888	0.5546155	0.5460672	0.5503414	up
A_21_P0000356	SCARNA11	0.9536762	1.4919133	1.5160103	0.5382371	0.5623341	0.5502856	up
A_33_P3257030	P3H4	5.970767	6.5921607	6.4497833	0.6213937	0.4790163	0.550205	up
A_33_P3399064	RNA5-8S5	6.1146784	7.0457616	6.2838535	0.9310832	0.1691752	0.5501292	up
A_23_P204016	CACNB3	1.389504	1.963203	1.9158812	0.573699	0.5263772	0.5500381	up
A_23_P414308	FLCN	0.4449096	1.2268753	0.7628126	0.7819657	0.317903	0.5499344	up
A_33_P3306983	C11orf31	6.660509	7.276031	7.1448317	0.6155219	0.4843226	0.5499222	up
A_33_P3423027	NRL	-2.738405	-2.051578	-2.325959	0.6868265	0.412446	0.5496362	up
A_23_P205200	DHRS12	0.9610128	1.5532103	1.4679074	0.5921974	0.5068946	0.549546	up
A_21_P0011354	XLOC_l2_004844	4.0094986	4.6155705	4.502287	0.606072	0.4927883	0.5494301	up
A_33_P3386181	ZFP64	-0.489376	0.2357097	-0.116041	0.7250853	0.3733344	0.5492098	up
A_23_P101246	VSIG10L	1.0922465	1.0316186	2.2510033	-0.060628	1.1587567	0.5490644	up
A_19_P00804740	TAPT1-AS1	-2.138133	-1.562832	-1.615385	0.5753012	0.5227485	0.5490249	up
A_33_P3379962	HLA-A	8.622793	9.343908	8.999441	0.7211151	0.376648	0.5488815	up
A_23_P50331	FAM32A	3.010005	3.5888743	3.5286474	0.5788693	0.5186424	0.5487559	up
A_33_P3369581	EIF3G	4.2323103	4.8374715	4.724435	0.6051612	0.4921246	0.5486429	up
A_33_P3671506	LOC153811	-1.53608	-0.758876	-1.216262	0.777204	0.319818	0.548511	up
A_24_P95439	CARS	-0.228186	0.8584967	-0.217862	1.0866828	0.010324	0.5485034	up
A_23_P71649	MUSK	-2.522446	-2.358862	-1.589139	0.163584	0.9333072	0.5484456	up
A_22_P00011719	lnc-PEA15-1	-2.353683	-1.872835	-1.737917	0.4808478	0.6157661	0.5483069	up
A_33_P3391727	SIRT7	2.9036407	3.51619	3.3876448	0.6125493	0.484004	0.5482767	up
A_33_P3284596	ARL5C	-2.914935	-2.379738	-2.353609	0.5351968	0.5613263	0.5482615	up
A_23_P354217	TMEM151A	-1.582972	-1.074845	-0.994883	0.5081272	0.5880895	0.5481083	up
A_22_P00012570	lnc-PTP4A2-1	4.549081	5.2349696	4.9593735	0.6858888	0.4102926	0.5480907	up
A_23_P165442	NRBP1	2.732276	3.3577409	3.2029686	0.6254649	0.4706926	0.5480788	up
A_22_P00008747	lnc-KIAA1967-2	1.3605924	2.0037541	1.8133612	0.6431618	0.4527688	0.5479653	up
A_24_P231494	DNPEP	2.1645088	2.697744	2.7271872	0.5332351	0.5626783	0.5479567	up
A_24_P313993	CAPS	2.9503632	3.6615624	3.3350067	0.7111993	0.3846436	0.5479214	up
A_22_P00019422	lnc-C10orf31-1	-2.296593	-1.604415	-1.893029	0.6921778	0.4035635	0.5478707	up
A_24_P173325	UBA52	8.577906	9.219267	9.031937	0.6413612	0.454031	0.5476961	up

A_22_P00011269	lnc-OR51B6-1	-2.614186	-1.901642	-2.231461	0.7125437	0.3827248	0.5476342	up
A_33_P3275435	KMT2E-AS1	0.4157629	1.0902939	0.8364558	0.674531	0.4206929	0.547612	up
A_33_P3361851	RAB7A	5.815894	6.2272906	6.499485	0.4113965	0.6835909	0.5474937	up
A_23_P326296	U2AF1L4	-0.329602	0.3080139	0.1275268	0.6376162	0.457129	0.5473726	up
A_22_P00007822	LOC101929715	-2.682295	-2.29253	-1.978039	0.389765	0.7042558	0.5470104	up
A_33_P3358233	NES	-0.948736	-0.364297	-0.439317	0.5844383	0.5094185	0.5469284	up
A_23_P2431	C3AR1	1.5650864	2.214357	2.0092964	0.6492705	0.4442101	0.5467403	up
A_23_P122896	TFR2	-1.349303	-0.70807	-0.897324	0.641233	0.4519787	0.5466058	up
A_23_P329890	TMEM136	2.8490257	3.364678	3.42634	0.5156522	0.5773144	0.5464833	up
A_33_P3243558	RNF224	-2.577734	-2.196704	-1.865837	0.3810306	0.7118976	0.5464641	up
A_23_P132738	CRYGS	1.4772415	1.9474158	2.0999804	0.4701743	0.6227388	0.5464566	up
A_23_P318604	CYHR1	2.9796934	3.5986333	3.4536104	0.6189399	0.473917	0.5464285	up
A_21_P0012952	LOC100505841	-3.518257	-2.782772	-3.160935	0.7354848	0.357322	0.5464034	up
A_21_P0003727	lnc-GPR125-1	-0.537902	-0.027664	0.0446339	0.5102377	0.5825357	0.5463867	up
A_23_P115573	SHISA4	4.611148	5.2369266	5.0777397	0.6257787	0.4665918	0.5461853	up
A_23_P1029	MFAP2	2.1404266	2.6771207	2.6959705	0.5366941	0.5555439	0.546119	up
A_23_P77965	HEATR6	0.2074933	0.835237	0.6719618	0.6277437	0.4644685	0.5461061	up
A_22_P00003569	SNHG18	0.8101387	1.4122205	1.3001823	0.6020818	0.4900436	0.5460627	up
A_23_P431179	HIST1H4A	0.0239949	0.7502241	0.3897619	0.7262292	0.365767	0.5459981	up
A_22_P00015288	lnc-SPATC1-1	3.1947813	3.718576	3.7628508	0.5237947	0.5680695	0.5459321	up
A_24_P822704	TMEM198	-1.020341	-0.377315	-0.571695	0.6430268	0.4486461	0.5458364	up
A_22_P00004019	lnc-CHMP7-2	1.3479786	1.9841342	1.8034339	0.6361556	0.4554553	0.5458055	up
A_24_P335092	SAA1	-2.762675	-2.242777	-2.191022	0.5198982	0.5716531	0.5457757	up
A_23_P28730	ZNF512B	0.6234441	1.2559171	1.0824313	0.632473	0.4589872	0.5457301	up
A_21_P0000804	HOXD-AS2	0.8672442	1.3635302	1.4620414	0.4962859	0.5947971	0.5455415	up
A_22_P00002410	VASH1	3.4046822	4.0495896	3.850831	0.6449075	0.4461489	0.5455282	up
A_23_P30435	TNIP1	3.8681898	4.5357995	4.2915735	0.6676097	0.4233837	0.5454967	up
A_33_P3253672	KCNH3	4.1949844	4.7253566	4.755293	0.5303721	0.5603085	0.5453403	up
A_33_P3330099	ARSD	1.8996468	2.5627923	2.3267365	0.6631455	0.4270897	0.5451176	up
A_22_P00023898	lnc-PHYHD1-1	-3.114554	-2.410137	-2.729024	0.704417	0.3855302	0.5449736	up
A_22_P00016239	lnc-TMC7-1	-1.784977	-1.147621	-1.332475	0.6373563	0.4525023	0.5449293	up
A_33_P3358893	PRM2	-1.518806	-1.057501	-0.890738	0.4613051	0.628068	0.5446866	up
A_33_P3386364	FANCF	1.6459732	2.1130013	2.2682934	0.4670281	0.6223202	0.5446742	up
A_23_P70566	FKBPL	-1.636712	-1.046849	-1.137274	0.5898628	0.4994378	0.5446503	up
A_22_P00018321	SSPO	-2.764338	-2.139693	-2.299819	0.624645	0.4645188	0.5445819	up
A_23_P54649	TRADD	4.61985	5.2079997	5.120861	0.5881496	0.5010109	0.5445802	up
A_33_P3210379	SCGB3A1	5.4212437	6.025315	5.906267	0.6040711	0.4850235	0.5445473	up
A_33_P3276519	MLST8	1.46315	1.9849257	2.0290928	0.5217757	0.5659428	0.5438592	up
A_33_P3282836	RPS28	9.901338	10.549578	10.34074	0.6482401	0.4394026	0.5438213	up
A_23_P24044	CNNM2	-1.391866	-0.720824	-0.975752	0.6710424	0.4161143	0.5435784	up
A_33_P3345354	RRNAD1	1.5359893	2.1240697	2.0348158	0.5880804	0.4988265	0.5434535	up
A_23_P26945	NAGLU	1.611968	2.2560325	2.0547247	0.6440644	0.4427567	0.5434105	up
A_22_P00014065	LINC01429	-1.638094	-0.9518	-1.237589	0.6862936	0.4005046	0.5433991	up
A_23_P340131	PRSS16	-0.444094	0.3195982	-0.121097	0.7636919	0.3229966	0.5433443	up
A_23_P48070	ING4	0.6476164	1.2338061	1.1478267	0.5861898	0.5002103	0.5432	up
A_23_P155417	ABHD14B	1.0337324	1.4516044	1.702146	0.417872	0.6684136	0.5431428	up
A_33_P3287477	C10orf120	-3.054337	-2.658563	-2.363987	0.3957732	0.6903496	0.5430614	up
A_24_P380132	G3BP2	4.945503	5.465018	5.512041	0.5195146	0.5665379	0.5430262	up
A_22_P00011416	PAK1	-3.187543	-2.796092	-2.492997	0.3914516	0.6945458	0.5429987	up
A_24_P216765	TOMM20	5.067891	5.572577	5.648945	0.5046859	0.5810537	0.5428698	up
A_23_P138480	C10orf95	-0.174221	0.3847952	0.3521905	0.5590158	0.5264111	0.5427134	up
A_33_P3263359	DGKA	-2.413095	-1.747539	-1.993479	0.665556	0.4196162	0.5425861	up
A_33_P3263232	LRRC3	-0.65564	-0.310193	0.0837112	0.3454475	0.7393513	0.5423994	up
A_23_P68910	SSTR3	2.5336733	3.172647	2.9794865	0.6389737	0.4458132	0.5423934	up
A_33_P3705907	FAM207A	2.1172638	2.7271752	2.592125	0.6099114	0.4748612	0.5423863	up
A_23_P54891	C16orf58	1.6693945	2.355062	2.0682688	0.6856675	0.3988743	0.5422709	up

A_21_P0002614	lnc-LYPD6-1	-0.28103	0.372324	0.1496725	0.6533542	0.4307027	0.5420284	up
A_33_P3416479	PHKG1	-3.107151	-2.69063	-2.439766	0.4165213	0.6673853	0.5419533	up
A_22_P00003346	lnc-CBLB-4	-1.71227	-1.283416	-1.057509	0.4288545	0.6547613	0.5418079	up
A_23_P428219	EZH1	0.1878285	0.7873855	0.6716538	0.5995569	0.4838252	0.5416911	up
A_23_P89902	RTN2	-0.575484	0.0792251	-0.146826	0.6547093	0.428658	0.5416837	up
A_23_P150428	OR6M1	-2.636599	-1.87587	-2.313991	0.7607291	0.3226085	0.5416688	up
A_24_P393838	TOMM20	2.3964243	2.7737756	3.1022024	0.3773513	0.7057781	0.5415647	up
A_23_P366812	AQP5	2.2200375	2.7434812	2.779539	0.5234437	0.5595017	0.5414727	up
A_21_P0011714	LILRA6	-0.850777	-0.293961	-0.324754	0.5568156	0.5260229	0.5414193	up
A_23_P259621	LAT2	1.482171	2.2160058	1.8309903	0.7338347	0.3488193	0.541327	up
A_33_P3362153	TMEM238	6.508133	7.089234	7.009392	0.5811009	0.5012589	0.5411799	up
A_23_P44166	DCXR	4.517062	5.083203	5.0331955	0.5661407	0.5161333	0.541137	up
A_32_P409222	ZNF628	1.9545975	2.7089143	2.2818003	0.7543168	0.3272028	0.5407598	up
A_33_P3278220	RABEPK	3.5475073	4.2869935	3.8894491	0.7394862	0.3419418	0.540714	up
A_23_P112774	PTP4A3	0.9928646	1.4908419	1.5762458	0.4979773	0.5833812	0.5406792	up
A_23_P28707	OGFR	5.9504747	6.522905	6.458728	0.5724301	0.5082531	0.5403416	up
A_24_P89413	TRIM39	0.0411263	0.5772729	0.5855956	0.5361466	0.5444694	0.540308	up
A_23_P435444	PCDHGA7	-0.832586	-0.167789	-0.416991	0.6647973	0.4155951	0.5401962	up
A_23_P160167	TSPAN1	6.8240967	7.383325	7.3452454	0.5592284	0.5211487	0.5401886	up
A_23_P47777	MARCH9	1.974721	2.6691499	2.360632	0.6944289	0.385911	0.54017	up
A_23_P88435	FOXN3	-1.990653	-1.787583	-1.113582	0.2030697	0.8770714	0.5400705	up
A_33_P3254996	DSCR9	-2.739907	-2.16171	-2.238117	0.578197	0.5017903	0.5399937	up
A_33_P3393537	PTAFR	3.6561966	4.254087	4.1382275	0.5978904	0.4820309	0.5399606	up
A_33_P3213064	STAT2	4.209202	4.426684	5.071619	0.2174821	0.8624172	0.5399496	up
A_22_P00020980	lnc-C7orf45-1	-0.063175	0.5633574	0.3901234	0.6265321	0.4532981	0.5399151	up
A_22_P00015379	LOC101929494	1.0695615	1.7220602	1.4968348	0.6524987	0.4272733	0.539886	up
A_33_P3344504	APBA2	-3.272358	-2.883486	-2.581619	0.3888729	0.6907394	0.5398061	up
A_19_P00809455	lnc-HES1-3	-3.253959	-2.512366	-2.916099	0.7415927	0.3378599	0.5397263	up
A_23_P334892	TMEM102	0.1405931	0.7226782	0.6379457	0.5820851	0.4973526	0.5397189	up
A_23_P10591	METRNL	5.583682	6.241965	6.0048304	0.6582828	0.4211483	0.5397155	up
A_23_P63379	CA14	-2.129108	-1.619206	-1.559815	0.509903	0.569293	0.539598	up
A_33_P3404950	MINOS1	-3.061127	-2.80727	-2.235814	0.2538574	0.8253129	0.5395851	up
A_33_P3258712	LOC101928000	1.1824808	1.7032928	1.7406607	0.520812	0.5581799	0.5394959	up
A_22_P00008028	lnc-IGFALS-1	-2.269569	-1.761978	-1.698217	0.5075917	0.5713525	0.5394721	up
A_22_P00014497	lnc-SHISA9-1	-0.567921	-0.039311	-0.017608	0.5286098	0.5503125	0.5394611	up
A_23_P761	PSMB4	7.240758	7.8147025	7.745425	0.5739446	0.5046673	0.5393059	up
A_23_P44849	KCTD13	-3.105371	-2.928526	-2.203735	0.1768453	0.9016364	0.5392408	up
A_23_P141960	SERTAD3	-0.034925	0.4709187	0.537477	0.5058436	0.572402	0.5391228	up
A_23_P205913	SLC24A1	-2.127746	-1.633066	-1.544391	0.4946799	0.5833545	0.5390172	up
A_33_P3422248	TMEM200C	3.9356098	4.4934154	4.455511	0.5578055	0.5199013	0.5388534	up
A_22_P00022745	lnc-LRGUK-1	1.4770398	2.2306886	1.8007083	0.7536488	0.3236685	0.5386586	up
A_23_P218486	WDR83OS	5.101803	5.718965	5.5619297	0.6171622	0.4601269	0.5386445	up
A_33_P3300308	MAP1LC3A	-0.664946	-0.050161	-0.20319	0.6147852	0.4617558	0.5382705	up
A_24_P216313	ERGIC3	3.8101387	4.402678	4.294096	0.5925393	0.4839573	0.5382483	up
A_23_P101532	RPS11	9.733178	10.34733	10.195509	0.614152	0.4623308	0.5382414	up
A_33_P3287379	COG8	0.7124443	1.3390341	1.1620212	0.6265898	0.4495769	0.5380833	up
A_22_P00023644	LOC101928668	-1.491251	-0.93551	-0.971055	0.5557404	0.520196	0.5379682	up
A_21_P0006052	lnc-GPR144-1	-1.112455	-0.342107	-0.80695	0.7703476	0.3055048	0.5379262	up
A_33_P3342443	RASA4B	-1.600759	-1.060298	-1.065399	0.5404606	0.5353599	0.5379102	up
A_24_P139620	USP21	-2.298877	-1.610585	-1.911451	0.688292	0.3874264	0.5378592	up
A_24_P370156	MAN2B1	0.2541509	0.9588428	0.6251731	0.7046919	0.3710222	0.5378571	up
A_23_P11005	ADAMTS7	8.975177	9.592825	9.432907	0.6176481	0.4577303	0.5376892	up
A_33_P3303176	MRGPRG	0.3443675	0.9399877	0.8240881	0.5956202	0.4797206	0.5376704	up
A_22_P00010024	SLC12A5	3.8773785	4.4939523	4.3359365	0.6165738	0.4585581	0.5375659	up
A_33_P3671291	SNORA12	-0.468248	-0.232476	0.3710914	0.2357721	0.8393397	0.5375559	up
A_33_P3246613	CCDC78	3.383792	3.9939294	3.8487463	0.6101375	0.4649544	0.5375459	up

A_23_P112482	AQP3	1.1120605	1.566175	1.7327328	0.4541144	0.6206722	0.5373933	up
A_23_P85952	DENND2D	3.6571836	4.421312	3.9672337	0.7641282	0.31005	0.5370891	up
A_33_P3332215	MUC1	-3.593301	-2.56852	-3.543924	1.0247803	0.049377	0.5370786	up
A_22_P00008302	lnc-ITGAL-1	-2.537987	-1.925427	-2.076417	0.61256	0.4615696	0.5370648	up
A_21_P0014702	LOC102724791	-2.42756	-1.86422	-1.916797	0.5633402	0.5107632	0.5370517	up
A_23_P501887	DHPS	1.7313414	2.2790456	2.2574234	0.5477042	0.526082	0.5368931	up
A_24_P937790	CCDC166	-1.834659	-1.124857	-1.470746	0.7098017	0.3639131	0.5368574	up
A_23_P134854	CLDN23	2.116725	2.5261254	2.7807627	0.4094005	0.6640377	0.5367191	up
A_33_P3268464	MPDU1	-2.162545	-1.671779	-1.580239	0.4907656	0.5823059	0.5365357	up
A_23_P85893	GLMP	1.252531	1.8586144	1.7192497	0.6060834	0.4667187	0.536401	up
A_24_P304723	PPIB	5.0281153	5.543057	5.5859575	0.5149417	0.5578423	0.536392	up
A_33_P3300142	ANKFY1	-3.193992	-2.793803	-2.521869	0.4001887	0.6721232	0.5361559	up
A_21_P0011941	XLOC_l2_008163	-3.164637	-2.604046	-2.652936	0.5605915	0.5117014	0.5361464	up
A_22_P00017033	lnc-TTLL11-1	-2.893104	-2.559322	-2.154622	0.333782	0.7384822	0.5361321	up
A_21_P0010455	lnc-C22orf26-5	4.2317476	4.7327485	4.802781	0.5010009	0.5710335	0.5360172	up
A_33_P3388588	LOC400558	5.91545	6.498401	6.4045277	0.5829511	0.4890776	0.5360143	up
A_21_P0012111	XLOC_l2_008546	-1.660689	-1.356758	-0.892747	0.3039312	0.767942	0.5359366	up
A_33_P3318946	HAPLN2	5.200981	5.7476554	5.7260275	0.5466743	0.5250464	0.5358603	up
A_24_P677634	LOC493754	5.5085917	5.963967	6.1248035	0.4553752	0.6162119	0.5357936	up
A_33_P3278573	MAGIX	-0.572482	0.0947733	-0.168477	0.6672554	0.4040055	0.5356305	up
A_23_P37892	GPT2	2.2227793	3.1841836	2.3325233	0.9614043	0.1097441	0.5355742	up
A_33_P3325231	XLOC_l2_001961	-3.505345	-2.706723	-3.232889	0.7986226	0.2724567	0.5355396	up
A_21_P0013280	XLOC_l2_013728	-1.915086	-1.518768	-1.24082	0.396318	0.6742659	0.5352919	up
A_22_P00000668	lnc-ACTR6-1	-1.62468	-0.888073	-1.290825	0.7366076	0.3338547	0.5352311	up
A_24_P938614	CDS1	3.2448988	3.726665	3.833187	0.4817662	0.5882883	0.5350273	up
A_23_P654	ZBTB17	0.8073382	1.4613752	1.2231617	0.654037	0.4158235	0.5349302	up
A_33_P3262205	CPNE1	3.3835325	4.2486186	3.5880384	0.8650861	0.2045059	0.534796	up
A_23_P163227	CKMT1A	6.1528034	6.7084975	6.666629	0.5556941	0.5138254	0.5347598	up
A_23_P136916	WDR13	2.4973564	3.1714702	2.8926992	0.6741138	0.3953428	0.5347283	up
A_21_P0014378	HKR1	-2.626435	-2.046707	-2.13677	0.5797274	0.4896643	0.5346959	up
A_33_P3279640	HCN2	6.5996733	7.244188	7.0244503	0.6445146	0.424777	0.5346458	up
A_32_P46571	RHBDL2	2.2205658	2.7338452	2.7763777	0.5132794	0.5558119	0.5345457	up
A_23_P142146	RPL18	9.065741	9.662901	9.537449	0.5971603	0.4717083	0.5344343	up
A_24_P73370	ULK1	1.9057636	2.7740874	2.1062965	0.8683238	0.2005329	0.5344284	up
A_33_P3296991	FLJ42393	-2.276395	-1.791872	-1.692294	0.4845238	0.5841012	0.5343125	up
A_23_P78734	MYH14	3.700904	4.4166913	4.0536804	0.7157874	0.3527765	0.534282	up
A_24_P42693	CYP4F11	-0.713259	0.331831	-0.690028	1.0450897	0.0232306	0.5341601	up
A_22_P00010235	MT1B	4.4322643	4.9614644	4.970619	0.5292001	0.5383549	0.5337775	up
A_21_P0004478	LOC102724758	-2.115563	-1.565418	-1.59826	0.5501452	0.517303	0.5337241	up
A_33_P3364263	LBH	-2.016705	-1.378458	-1.587575	0.638247	0.4291301	0.5336885	up
A_22_P00014164	LOC646762	-0.59157	0.0359697	-0.151917	0.6275401	0.4396534	0.5335968	up
A_22_P00024094	lnc-BEST4-1	-3.004143	-2.630123	-2.311467	0.3740199	0.6926754	0.5333476	up
A_33_P3271196	AMOTL1	0.8494334	1.3991318	1.3657737	0.5496984	0.5163403	0.5330193	up
A_22_P00003390	lnc-CCDC113-1	4.415839	4.990995	4.9065514	0.5751557	0.4907122	0.532934	up
A_33_P3404989	HIST1H3H	-0.246153	0.3511791	0.222301	0.5973325	0.4684544	0.5328934	up
A_21_P0000206	SNORD21	0.3702202	0.9408174	0.8651385	0.5705972	0.4949184	0.5327578	up
A_23_P56127	SMG9	1.2932563	1.8279967	1.8238964	0.5347405	0.5306401	0.5326903	up
A_19_P00323413	PTPN14	1.6204977	2.4186559	1.8876667	0.7981582	0.267169	0.5326636	up
A_21_P0000310	SNORA12	1.522409	2.0825658	2.027482	0.5601568	0.5050731	0.532615	up
A_23_P20255	COMMD5	3.402646	3.895001	3.9755144	0.4923549	0.5728684	0.5326116	up
A_23_P167401	PCDHB11	1.3716779	1.7651529	2.0434217	0.3934751	0.6717439	0.5326095	up
A_33_P3236868	MT1X	3.3135166	3.8803267	3.8119154	0.5668101	0.4983988	0.5326045	up
A_32_P214340	lnc-AKIRIN1-1	-1.87119	-0.960105	-1.717425	0.9110842	0.1537647	0.5324245	up
A_22_P00020469	lnc-OLFML2A-1	1.7670088	2.4000096	2.1988544	0.6330009	0.4318457	0.5324233	up
A_22_P00015965	lnc-TEFM-2	0.6779532	1.2751737	1.1455541	0.5972204	0.4676008	0.5324106	up
A_23_P42306	HLA-DMA	0.9501586	1.5774016	1.3869734	0.627243	0.4368148	0.5320289	up

A_33_P3294826	CRABP2	-1.201568	-0.545972	-0.793169	0.6555953	0.4083986	0.531997	up
A_33_P3345936	CCDC9	7.0197906	7.6024165	7.500843	0.5826259	0.4810524	0.5318391	up
A_21_P0000332	SNORA45B	0.6428285	1.3195791	1.0296693	0.6767507	0.3868408	0.5317957	up
A_23_P27353	SLC14A2	-3.196062	-2.493499	-2.835101	0.7025635	0.3609607	0.5317621	up
A_21_P0006334	lnc-QSOX2-1	-1.04372	-0.455587	-0.568388	0.5881329	0.4753327	0.5317328	up
A_23_P156970	MEST	2.1430626	2.6534362	2.6958857	0.5103736	0.5528231	0.5315983	up
A_23_P502035	GALT	0.3970838	1.0214939	0.8358564	0.6244102	0.4387727	0.5315914	up
A_32_P538017	TBX10	-3.062268	-2.525736	-2.53564	0.5365317	0.5266278	0.5315798	up
A_22_P00025116	LOC101927267	-0.647121	-0.195154	-0.036119	0.4519672	0.611002	0.5314846	up
A_21_P0000246	SNORD18B	-0.716082	0.3413968	-0.710602	1.0574789	0.0054803	0.5314796	up
A_33_P3253574	OR8B3	-2.861853	-2.357064	-2.30379	0.5047891	0.5580623	0.5314257	up
A_33_P3378545	lnc-DIO2-3	-0.780145	-0.162719	-0.334979	0.6174264	0.4451666	0.5312965	up
A_22_P00014694	SLC25A29	-0.833934	-0.390271	-0.215468	0.4436626	0.6184659	0.5310643	up
A_23_P117424	DCAF11	1.4178276	2.0462317	1.8513737	0.6284041	0.4335461	0.5309751	up
A_33_P3346573	MAP4	-1.058592	-0.539406	-0.515939	0.519186	0.5426536	0.5309198	up
A_33_P3318646	CALY	7.433218	8.099833	7.828101	0.6666145	0.3948832	0.5307488	up
A_22_P00021195	LOC101928504	-1.088626	-0.534466	-0.581409	0.5541606	0.5072174	0.530689	up
A_23_P17330	UCKL1	0.6555629	1.1568441	1.2155337	0.5012813	0.5599709	0.5306261	up
A_24_P314451	F8	-1.445602	-0.869912	-0.960122	0.5756898	0.4854803	0.5305851	up
A_32_P113436	HNRNPA1L2	3.8391151	4.39138	4.3477077	0.5522647	0.5085926	0.5304287	up
A_23_P254498	RANGRF	2.0481396	2.3512177	2.8056269	0.3030782	0.7574873	0.5302827	up
A_23_P159406	SPRR1B	-0.437527	0.0504122	0.1350203	0.4879394	0.5725474	0.5302434	up
A_23_P116091	DPP3	3.643403	4.18033	4.16656	0.5369268	0.5231571	0.5300419	up
A_33_P3372451	DNPH1	6.130602	6.5696626	6.7515593	0.4390607	0.6209574	0.5300091	up
A_32_P38283	TCTE3	-2.671328	-2.03021	-2.252629	0.6411188	0.4186997	0.5299093	up
A_23_P70445	HIST1H3E	5.0260267	5.510548	5.6008234	0.4845214	0.5747967	0.5296591	up
A_23_P149470	NDUFS2	3.6374712	4.1631866	4.170863	0.5257154	0.533392	0.5295537	up
A_24_P218688	ALDH3B1	1.0109434	2.0569959	1.0238953	1.0460525	0.0129519	0.5295022	up
A_21_P0007803	lnc-TMEM132D-2	-3.575523	-3.166852	-2.925366	0.4086711	0.650157	0.5294141	up
A_33_P3262043	BAD	3.498743	4.083489	3.9727488	0.5847459	0.4740057	0.5293758	up
A_19_P00322932	LINC00963	0.6426625	1.6088123	0.7352104	0.9661498	0.0925479	0.5293488	up
A_33_P3418194	LYNX1	3.7066584	4.337217	4.134782	0.6305585	0.4281235	0.529341	up
A_23_P405282	MGC45922	2.9228811	3.5320287	3.3723946	0.6091476	0.4495134	0.5293305	up
A_23_P102117	WNT10A	1.7506666	2.2896695	2.2701979	0.5390029	0.5195313	0.5292671	up
A_23_P87072	PANX3	-3.004257	-2.653894	-2.296212	0.3503633	0.7080443	0.5292038	up
A_33_P3244843	PRKAG3	2.0946188	2.6758523	2.5717058	0.5812335	0.477087	0.5291603	up
A_22_P00006103	lnc-FAM168A-2	-1.718879	-1.280302	-1.099221	0.4385767	0.6196575	0.5291171	up
A_22_P00016775	TRIM52-AS1	3.3022099	3.937614	3.7250376	0.6354041	0.4228277	0.5291159	up
A_23_P164927	SYNGR4	0.9603338	1.7007718	1.2780342	0.740438	0.3177004	0.5290692	up
A_33_P3327687	LOC727993	-1.954706	-1.494223	-1.357054	0.4604831	0.5976524	0.5290678	up
A_33_P3269844	LRRC26	2.5569916	3.0878587	3.0842104	0.5308671	0.5272188	0.529043	up
A_33_P3248008	LOC100652807	-2.031435	-1.426948	-1.57795	0.6044865	0.4534845	0.5289855	up
A_21_P0009067	LOC102467146	-2.431739	-1.870783	-1.934867	0.560956	0.4968724	0.5289142	up
A_23_P44674	CRIP1	6.4113836	7.0233893	6.8571978	0.6120057	0.4458141	0.5289099	up
A_22_P00007880	lnc-HSCB-1	-1.504561	-0.940598	-1.010834	0.5639629	0.4937263	0.5288446	up
A_22_P00001790	LOC101928521	-1.780358	-1.191092	-1.311979	0.5892663	0.468379	0.5288227	up
A_23_P139912	IGFBP6	6.075062	6.7883525	6.4192123	0.7132907	0.3441505	0.5287206	up
A_23_P40453	CBR3	-2.494387	-2.092882	-1.838746	0.4015048	0.6556408	0.5285728	up
A_33_P3261953	LOC100996349	-2.920748	-2.600438	-2.184035	0.3203094	0.7367125	0.5285109	up
A_23_P101642	PTPRH	1.646287	2.7217364	1.627748	1.0754495	-0.018539	0.5284553	up
A_24_P349743	RPSAP58	6.005126	6.745049	6.321782	0.739923	0.3166561	0.5282896	up
A_21_P0005115	RAB44	-2.761201	-2.297555	-2.168398	0.4636464	0.5928028	0.5282246	up
A_22_P00011225	lnc-OPRL1-1	0.0725904	0.6607747	0.5406647	0.5881844	0.4680743	0.5281293	up
A_23_P78782	CA11	0.1048474	0.2872138	0.9787107	0.1823664	0.8738632	0.5281148	up
A_21_P0009367	lnc-TMEM105-2	-0.03178	0.5490966	0.4434748	0.5808764	0.4752545	0.5280654	up
A_33_P3382331	HSPA6	-2.787488	-1.81874	-2.700834	0.9687476	0.0866542	0.5277009	up

A_22_P00007479	lnc-GTPBP1-1	-3.020368	-2.069398	-2.916153	0.9509702	0.1042154	0.5275928	up
A_33_P3379967	HLA-F	1.2672911	1.7027783	1.8867302	0.4354873	0.6194391	0.5274632	up
A_23_P68892	RTCB	4.659666	5.183329	5.190895	0.523663	0.531229	0.527446	up
A_24_P237878	FAM195B	4.718732	5.168758	5.3235636	0.450026	0.6048317	0.5274289	up
A_23_P123848	DAB2IP	3.0127668	3.4636188	3.616705	0.4508519	0.6039381	0.527395	up
A_23_P119266	DNASE2	1.8221331	2.6793628	2.0195475	0.8572297	0.1974144	0.5273221	up
A_22_P00007596	lnc-HDDC3-3	-2.899849	-2.201497	-2.543603	0.6983516	0.3562453	0.5272984	up
A_32_P57728	PMS2P1	3.1157894	3.7605433	3.5253181	0.6447539	0.4095287	0.5271413	up
A_33_P3217028	TM9SF1	0.6604314	1.2006054	1.1744905	0.540174	0.5140591	0.5271165	up
A_33_P3254666	LZTS2	0.2420106	0.8990517	0.6391063	0.6570411	0.3970957	0.5270684	up
A_33_P3229002	C17orf50	-0.48167	-0.049881	0.1405568	0.4317899	0.6222272	0.5270085	up
A_23_P373031	CACNA1C	2.059596	2.7198167	2.4533691	0.6602206	0.3937731	0.5269968	up
A_24_P233915	MED8	1.551189	2.1902947	1.9660492	0.6391058	0.4148603	0.526983	up
A_23_P210763	JAG1	3.2702131	3.659943	3.934266	0.38973	0.664053	0.5268915	up
A_33_P3386765	ABHD14A	2.7420988	3.3249831	3.2127066	0.5828843	0.4706078	0.526746	up
A_32_P62008	RPL10A	8.6759815	9.301687	9.103634	0.6257057	0.4276524	0.526679	up
A_23_P406616	CCDC71L	1.3168435	2.0337653	1.653233	0.7169218	0.3363895	0.5266557	up
A_22_P00009331	LRRC7	-2.279328	-1.752182	-1.753348	0.5271463	0.5259805	0.5265634	up
A_33_P3234490	BOLA2B	2.6180782	3.1425033	3.1466713	0.524425	0.5285931	0.526509	up
A_23_P96827	APCS	-1.953826	-1.414429	-1.440297	0.5393968	0.5135288	0.5264628	up
A_23_P135357	RNMTL1	2.1790123	2.7898374	2.6209927	0.6108251	0.4419804	0.5264027	up
A_33_P3236902	MUC3A	-0.531127	0.0228162	-0.03246	0.5539436	0.4986677	0.5263057	up
A_23_P135611	DALRD3	0.2942128	0.9880176	0.6528606	0.6938047	0.3586478	0.5262263	up
A_33_P3221438	XXYLT1	2.757103	3.276031	3.2906055	0.5189281	0.5335026	0.5262153	up
A_23_P219117	DMTN	-1.762963	-1.470682	-1.002839	0.2922812	0.7601237	0.5262024	up
A_23_P158053	C9orf16	2.816372	3.3104377	3.374648	0.4940658	0.5582762	0.526171	up
A_21_P0000317	SNORA22	0.9368835	1.5183668	1.4076829	0.5814834	0.4707995	0.5261414	up
A_24_P55295	GJA1	-1.71945	-1.996116	-0.390542	-0.276666	1.3289084	0.5261214	up
A_33_P3376154	LOC100128002	0.7101879	1.293531	1.1787052	0.583343	0.4685173	0.5259302	up
A_21_P0011016	LOC643733	2.6959887	3.3982625	3.0454178	0.7022739	0.3494291	0.5258515	up
A_33_P3240333	PITX1	1.9607601	2.590217	2.3828926	0.629457	0.4221325	0.5257948	up
A_33_P3282291	FLJ40039	-0.333807	0.1500506	0.2338181	0.4838572	0.5676246	0.5257409	up
A_23_P423331	NTNG2	-0.843604	-0.345966	-0.290539	0.4976378	0.5530653	0.5253515	up
A_21_P0008304	lnc-FOXG1-6	-0.370381	0.3593254	-0.049517	0.7297068	0.3208642	0.5252855	up
A_23_P50872	NDUFB7	4.5391436	5.1045537	5.0241117	0.5654101	0.4849682	0.5251892	up
A_33_P3372563	ABCC10	-1.211915	-0.693222	-0.680262	0.5186935	0.5316529	0.5251732	up
A_23_P152047	SCAMP5	1.269187	1.5465398	2.0420752	0.2773528	0.7728882	0.5251205	up
A_23_P48581	C14orf93	2.3582878	3.182324	2.5841131	0.8240361	0.2258253	0.5249307	up
A_23_P88580	ARID3B	2.0296726	2.6468883	2.462203	0.6172156	0.4325304	0.524873	up
A_33_P3219459	TMEM240	-1.018288	-0.34375	-0.643214	0.6745386	0.3750744	0.5248065	up
A_23_P210690	TRIB3	4.8861303	6.1535974	4.668253	1.267467	-0.217877	0.5247948	up
A_23_P27215	UBB	7.8739653	8.484905	8.31211	0.61094	0.4381447	0.5245423	up
A_33_P3340309	ZNF768	4.6467867	5.0241055	5.3176346	0.3773189	0.6708479	0.5240834	up
A_22_P00015975	lnc-TELO2-3	-3.094949	-2.448584	-2.693188	0.6463645	0.4017611	0.5240628	up
A_23_P327140	RNF213	-0.835301	-0.739526	0.1169581	0.0957756	0.9522595	0.5240176	up
A_33_P3266010	RIN1	3.7512283	4.171558	4.3788967	0.4203296	0.6276684	0.523999	up
A_22_P00005250	DLX6-AS1	-1.01507	-0.540947	-0.441221	0.4741235	0.5738497	0.5239866	up
A_23_P97064	FBXO6	-1.858698	-1.615131	-1.054365	0.243567	0.8043337	0.5239503	up
A_22_P00024619	lnc-RP11-503N18.3.1-3	3.3419619	3.92588	3.8057346	0.5839181	0.4637728	0.5238454	up
A_23_P501831	FAXDC2	-1.88704	-1.393034	-1.333454	0.4940057	0.553586	0.5237958	up
A_33_P3326483	CRABP1	2.2012653	2.592218	2.8578281	0.3909526	0.6565628	0.5237577	up
A_23_P116037	TM7SF2	1.7409725	2.2842898	2.2450895	0.5433173	0.504117	0.5237172	up
A_24_P273253	AHNAK2	0.9205494	1.7763429	1.11197	0.8557935	0.1914206	0.523607	up
A_21_P0005645	lnc-CLN8-1	-2.798155	-2.023055	-2.526084	0.7750998	0.2720709	0.5235853	up
A_23_P34402	NCSTN	4.004266	4.5327773	4.5229034	0.5285115	0.5186377	0.5235746	up

A_33_P3287039	TFPT	3.5635366	4.0927587	4.0811787	0.529222	0.517642	0.523432	up
A_22_P00022981	ABO	-2.963207	-2.304812	-2.574755	0.6583941	0.3884518	0.523423	up
A_22_P00012807	lnc-RAP1GDS1-3	-1.653235	-0.997008	-1.26294	0.6562276	0.390296	0.5232618	up
A_23_P324384	RPS4Y2	6.0084543	6.6182218	6.445133	0.6097674	0.4366789	0.5232232	up
A_19_P00802413	PIEZO1	7.842409	8.435732	8.295462	0.5933228	0.4530525	0.5231876	up
A_23_P54840	MT1A	5.623336	6.20519	6.087741	0.5818543	0.4644051	0.5231297	up
A_33_P3409996	AP5Z1	-1.072097	-0.375409	-0.722543	0.6966882	0.3495536	0.5231209	up
A_33_P3294002	A4GALT	6.2127066	6.7170415	6.754401	0.5043349	0.5416946	0.5230148	up
A_33_P3256585	MUC8	-3.11361	-2.625709	-2.555556	0.4879015	0.558054	0.5229777	up
A_33_P3263756	ZNF446	2.2059155	2.8656478	2.592083	0.6597323	0.3861675	0.5229499	up
A_33_P3499174	URM1	5.298834	5.9477334	5.6956425	0.6488996	0.3968086	0.5228541	up
A_33_P3296181	CCL3L3	-3.05524	-2.879132	-2.185851	0.1761081	0.8693886	0.5227484	up
A_23_P169470	CDK9	3.0522375	3.5636468	3.5859241	0.5114093	0.5336866	0.522548	up
A_23_P113523	GTPBP6	5.4516983	6.066751	5.881645	0.6150527	0.4299469	0.5224998	up
A_22_P00011315	MLYCD	-1.740168	-1.007459	-1.428136	0.7327094	0.3120322	0.5223708	up
A_33_P3236392	PVRL4	3.6331377	4.181191	4.129717	0.5480533	0.4965792	0.5223162	up
A_33_P7289027	LOC401320	1.4367905	1.9886546	1.9291258	0.5518642	0.4923353	0.5220997	up
A_33_P3303066	BTBD2	1.0444369	1.741952	1.391026	0.697515	0.3465891	0.5220521	up
A_22_P00012023	TTN-AS1	-1.780596	-1.225012	-1.292146	0.555584	0.4884496	0.5220168	up
A_23_P100001	FAM174B	1.345892	1.9852214	1.7505698	0.6393294	0.4046779	0.5220037	up
A_21_P0011716	XLOC_l2_006832	-3.131862	-2.863138	-2.356663	0.2687242	0.7751999	0.5219621	up
A_23_P3592	HSF4	-1.79317	-1.426262	-1.116274	0.3669076	0.6768951	0.5219014	up
A_21_P0000926	LOC100130417	-2.346932	-1.767796	-1.882339	0.5791359	0.4645929	0.5218644	up
A_22_P00012698	lnc-RAB1A-1	0.1878591	0.8014426	0.6178188	0.6135836	0.4299598	0.5217717	up
A_21_P0013865	LOC102725299	-2.081812	-1.85718	-1.262909	0.2246327	0.8189039	0.5217683	up
A_32_P420563	RNF215	-0.911063	-0.270154	-0.508484	0.6409097	0.4025793	0.5217445	up
A_33_P3368358	NEDD9	-1.530214	-1.093809	-0.92337	0.4364052	0.606844	0.5216246	up
A_33_P3226050	GATSL3	4.0838747	4.280585	4.9301777	0.1967101	0.846303	0.5215066	up
A_33_P3294217	UTF1	1.4729466	2.0550294	1.9334116	0.5820828	0.460465	0.5212739	up
A_23_P369328	C10orf35	3.6889458	3.9279666	4.492449	0.2390208	0.803503	0.5212619	up
A_23_P163992	GRB7	5.0795717	5.735271	5.4660053	0.6556993	0.3864336	0.5210664	up
A_24_P277955	FIS1	4.495414	5.1660542	4.866767	0.6706405	0.3713532	0.5209968	up
A_33_P3285715	GLI4	6.8807783	7.4549584	7.3480244	0.5741801	0.4672461	0.5207131	up
A_23_P254944	GSTT1	3.3097181	3.9124007	3.7484446	0.6026826	0.4387264	0.5207045	up
A_23_P152115	NME3	4.1867294	4.6293063	4.784521	0.4425769	0.5977917	0.5201843	up
A_23_P121253	TNFSF10	0.8846574	1.0144591	1.794776	0.1298018	0.9101186	0.5199602	up
A_23_P217208	SLC35A2	-2.003193	-1.70329	-1.263366	0.2999029	0.7398267	0.5198648	up
A_33_P3262118	LUZP1	-2.857357	-2.134791	-2.54024	0.7225664	0.317117	0.5198417	up
A_24_P259607	SUSD4	-2.942221	-2.598269	-2.247328	0.3439524	0.6948926	0.5194225	up
A_21_P0007592	lnc-NAV3-1	-2.219545	-1.504137	-1.896237	0.7154083	0.323308	0.5193582	up
A_21_P0003278	lnc-WNT7A-1	0.9707551	1.4720249	1.5079117	0.5012698	0.5371566	0.5192132	up
A_24_P37887	GPR150	5.42212	6.0774083	5.805197	0.6552882	0.3830767	0.5191824	up
A_23_P84629	RFNG	-0.007563	0.6375136	0.3856974	0.6450768	0.3932605	0.5191686	up
A_23_P62901	BTG2	2.0057077	2.455174	2.5945702	0.4494662	0.5888624	0.5191643	up
A_23_P121506	FAM193A	-1.568211	-0.875306	-1.222814	0.6929054	0.345397	0.5191512	up
A_22_P00011171	lnc-ODF4-2	-3.062717	-2.370497	-2.716658	0.6922207	0.3460598	0.5191403	up
A_21_P0013771	XLOC_l2_015561	2.1704788	2.7174487	2.6617575	0.5469699	0.4912787	0.5191243	up
A_24_P87763	EEF2	6.1429014	6.865277	6.4585934	0.7223754	0.315692	0.5190337	up
A_33_P3400728	RIPK4	-3.500064	-2.648246	-3.313822	0.8518174	0.1862416	0.5190295	up
A_24_P172990	AARS	2.298973	3.1762729	2.4595804	0.8772998	0.1606073	0.5189536	up
A_33_P3246885	DMKN	5.0334883	5.456844	5.6480007	0.4233556	0.6145124	0.518934	up
A_32_P99432	TRAPPC5	5.1199265	5.665834	5.611698	0.5459075	0.4917717	0.5188396	up
A_23_P107483	OR3A3	-2.624417	-2.106549	-2.104662	0.5178683	0.5197547	0.5188115	up
A_33_P3294446	HMBOX1	-3.562557	-3.000994	-3.086536	0.561563	0.4760208	0.5187919	up
A_23_P500886	CLDN15	-2.420059	-1.813039	-1.989729	0.6070199	0.4303303	0.5186751	up
A_33_P3333038	PTMS	7.899132	8.488132	8.347367	0.5890007	0.4482355	0.5186181	up

A_21_P0009629	LINC01478	0.9289069	1.505126	1.3891363	0.5762191	0.4602294	0.5182243	up
A_33_P3344201	AKT1S1	3.2697144	4.086536	3.4891653	0.8168216	0.219451	0.5181363	up
A_23_P212310	ELP6	2.9075356	3.4645238	3.386569	0.5569882	0.4790335	0.5180109	up
A_23_P139722	TNFRSF1A	2.4658737	3.1017509	2.8660088	0.6358771	0.400135	0.5180061	up
A_33_P3250963	TP53TG1	1.0958953	1.7544055	1.4733968	0.6585102	0.3775015	0.5180059	up
A_23_P202696	KBTBD4	2.372614	2.924601	2.8565922	0.5519872	0.4839783	0.5179827	up
A_23_P120504	TMEM74B	-1.302625	-1.054007	-0.515286	0.2486181	0.7873383	0.5179782	up
A_23_P420373	DNMT3A	-3.193672	-2.839969	-2.51167	0.353703	0.6820025	0.5178528	up
A_23_P374389	PWWP2B	3.4119673	4.102494	3.7567291	0.6905265	0.3447619	0.5176442	up
A_33_P3258316	XLOC_l2_013837	3.7062187	4.2763486	4.1713076	0.5701299	0.4650888	0.5176094	up
A_21_P0007497	LINC01234	-0.857718	-0.325802	-0.354541	0.5319157	0.5031767	0.5175462	up
A_33_P3312504	PSD4	2.9478922	3.5671926	3.3636045	0.6193004	0.4157124	0.5175064	up
A_21_P0001553	lnc-DPYD-1	-2.399824	-1.885452	-1.879447	0.5143724	0.5203776	0.517375	up
A_33_P3314231	CRYBA2	-0.507225	0.3942976	-0.374203	0.9015226	0.1330218	0.5172722	up
A_24_P177964	NCKAP5L	2.062027	2.716309	2.442276	0.6542821	0.380249	0.5172656	up
A_33_P3839760	C19orf24	5.399886	5.815807	6.018462	0.4159207	0.6185761	0.5172484	up
A_33_P3395976	CTU1	4.3347178	4.830416	4.873438	0.4956985	0.5387201	0.5172093	up
A_23_P258814	DPH3P1	2.2479887	2.8476176	2.6827745	0.5996289	0.4347858	0.5172074	up
A_23_P126388	SH3BGRL3	5.592757	6.0693755	6.1500607	0.4766183	0.5573034	0.5169609	up
A_23_P127186	TACC2	2.1581783	2.829492	2.520401	0.6713138	0.3622227	0.5167682	up
A_32_P149174	C6orf226	0.49788	0.9281745	1.1009789	0.4302945	0.6030989	0.5166967	up
A_33_P3296067	CDC14C	0.8421211	1.430922	1.286458	0.5888009	0.4443369	0.5165689	up
A_21_P0000502	RNU11	1.3017511	2.0896564	1.5469723	0.7879052	0.2452211	0.5165632	up
A_21_P0009928	lnc-STK35-2	-2.191984	-1.678399	-1.672497	0.5135856	0.5194874	0.5165365	up
A_33_P3411848	CNFN	3.1005058	3.5260282	3.7080154	0.4255223	0.6075096	0.516516	up
A_23_P395075	KDM3A	-1.696878	-1.077689	-1.283308	0.6191888	0.4135695	0.5163791	up
A_33_P3221680	RPS28	9.296579	9.842798	9.78281	0.5462189	0.4862309	0.5162249	up
A_33_P3402868	GRIN2D	3.1704788	3.787951	3.585144	0.6174722	0.4146652	0.5160687	up
A_33_P3259507	FBXO10	-0.018054	0.7977552	0.1980519	0.8158093	0.2161059	0.5159576	up
A_23_P22854	PGPEP1	-2.51616	-1.856865	-2.14367	0.6592951	0.3724899	0.5158925	up
A_23_P144622	GNPDA1	0.6717682	1.3111854	1.0640378	0.6394172	0.3922696	0.5158434	up
A_23_P77562	TMEM219	1.8165035	2.3960423	2.2684984	0.5795388	0.4519949	0.5157669	up
A_33_P3293524	NEURL1	4.6504936	5.3166337	5.015415	0.6661401	0.3649216	0.5155308	up
A_33_P3297305	ELMOD3	-2.847544	-2.086042	-2.578351	0.7615025	0.2691929	0.5153477	up
A_23_P127150	TUBGCP2	3.1329966	3.6843715	3.6122236	0.5513749	0.4792271	0.515301	up
A_33_P3326285	GAS5	8.081652	8.836113	8.357791	0.7544613	0.2761393	0.5153003	up
A_23_P203420	MTA2	3.1028128	3.6019912	3.6341476	0.4991784	0.5313349	0.5152567	up
A_23_P65022	ACADS	-0.403529	0.1400476	0.08322	0.5435762	0.4867487	0.5151625	up
A_23_P408930	OR7E5P	-0.917336	-0.212879	-0.591521	0.7044563	0.3258143	0.5151353	up
A_21_P0010997	LOC283140	-0.052504	0.5542402	0.3709502	0.6067438	0.4234538	0.5150988	up
A_23_P131139	DIRC1	2.0431747	2.7012534	2.4152327	0.6580787	0.3720579	0.5150683	up
A_24_P418044	HLA-J	6.5163603	7.0407596	7.0219145	0.5243993	0.5055542	0.5149768	up
A_22_P00001148	lnc-AMIGO1-1	-0.960954	-0.38546	-0.506674	0.5754933	0.4542794	0.5148864	up
A_33_P3415859	NLRC3	4.750104	5.384023	5.1459007	0.6339192	0.3957968	0.514858	up
A_21_P0000128	MKRN2OS	0.5979104	1.0760489	1.1493244	0.4781385	0.551414	0.5147762	up
A_22_P00012495	lnc-PSD2-1	-3.002559	-2.560391	-2.41526	0.442168	0.5872991	0.5147336	up
A_33_P3380742	TIMM17B	5.609397	6.226064	6.021967	0.6166673	0.41257	0.5146187	up
A_23_P95060	EPHB3	1.5467391	2.3691144	1.7533503	0.8223753	0.2066112	0.5144932	up
A_33_P3223678	LHX3	3.5375013	4.223185	3.8806906	0.6856837	0.3431892	0.5144365	up
A_23_P51679	MEF2D	2.6747866	3.202681	3.1756773	0.5278945	0.5008907	0.5143926	up
A_23_P352799	NPW	0.5248504	0.8520303	1.2254186	0.3271799	0.7005682	0.5138741	up
A_22_P00020566	lnc-VSTM2B-1	-1.563619	-0.990454	-1.109091	0.5731654	0.4545283	0.5138469	up
A_22_P00006862	TCEB3-AS1	1.5583096	2.1943097	1.9495926	0.6360002	0.391283	0.5136416	up
A_22_P00019134	lnc-CBX1-1	-2.315347	-1.626297	-1.977389	0.6890497	0.3379574	0.5135035	up
A_23_P341325	RPL10L	7.173153	7.8860707	7.48707	0.7129178	0.3139172	0.5134175	up
A_33_P3328666	CLTB	2.8859873	3.5597405	3.2388391	0.6737533	0.3528519	0.5133026	up

A_21_P0014073	LOC101927886	-2.257751	-1.750711	-1.738259	0.50704	0.5194917	0.5132659	up
A_23_P33894	MAGED2	1.4319744	2.0207553	1.8696842	0.5887809	0.4377098	0.5132454	up
A_33_P3209581	IQSEC3	1.8600454	2.4732223	2.2731867	0.6131768	0.4131413	0.513159	up
A_23_P200239	YIPF1	2.693799	3.2763705	3.137415	0.5825715	0.4436159	0.5130937	up
A_23_P501822	JUP	7.837219	8.423943	8.276663	0.5867233	0.4394436	0.5130835	up
A_24_P465772	RPSAP52	5.8877897	6.4977994	6.3039093	0.6100097	0.4161196	0.5130646	up
A_33_P3363933	FCRL6	-2.963392	-2.646034	-2.254788	0.317358	0.7086046	0.5129813	up
A_33_P3304527	ZNFX1	-1.578886	-1.016811	-1.115092	0.5620747	0.4637938	0.5129342	up
A_23_P109410	THAP7	1.2033811	1.7121673	1.7203817	0.5087862	0.5170007	0.5128935	up
A_33_P3212665	HTATSF1P2	-1.989697	-1.28499	-1.668745	0.7047067	0.3209519	0.5128293	up
A_23_P100795	STAT3	-0.488217	0.1389923	-0.089945	0.6272092	0.398272	0.5127406	up
A_23_P142776	EIF3F	3.8630075	4.5712705	4.1799974	0.7082629	0.3169899	0.5126264	up
A_33_P3303380	PARD3	-2.990216	-1.724589	-3.230639	1.2656269	-0.240423	0.512602	up
A_33_P3396459	POLR2H	1.8207922	2.4297833	2.2368946	0.6089912	0.4161024	0.5125468	up
A_23_P409623	PPFIBP2	-0.766909	-0.10944	-0.399451	0.6574693	0.3674579	0.5124636	up
A_23_P104583	CORO1B	1.2950387	1.7438912	1.8703938	0.4488525	0.5753551	0.5121038	up
A_23_P363896	LRRC75A-AS1	7.4334917	8.155258	7.735902	0.7217665	0.3024101	0.5120883	up
A_33_P3398000	TTN-AS1	-2.263169	-1.620128	-1.882067	0.6430416	0.3811021	0.5120719	up
A_22_P00018860	lnc-SERHL2-3	8.907745	9.474266	9.365334	0.5665207	0.4575882	0.5120545	up
A_33_P3354646	PNLIPRP1	-3.079267	-2.522571	-2.611963	0.5566952	0.467304	0.5119996	up
A_21_P0009233	lnc-PIPOX-1	-3.06329	-2.348025	-2.754557	0.7152658	0.308733	0.5119994	up
A_33_P3336103	TIGD5	0.2281337	0.7937298	0.6864586	0.5655961	0.4583249	0.5119605	up
A_22_P00004771	lnc-CTU2-2	-2.533881	-2.10104	-1.942828	0.4328411	0.591053	0.511947	up
A_23_P205031	COL4A2	2.845932	3.7430415	2.9725018	0.8971095	0.1265698	0.5118396	up
A_23_P70547	HSPA1L	-3.377106	-2.51733	-3.213302	0.8597758	0.1638036	0.5117897	up
A_23_P32320	ASPSCR1	0.3164487	0.7316423	0.9246607	0.4151936	0.608212	0.5117028	up
A_24_P48162	MPG	3.692994	4.343183	4.0658693	0.6501889	0.3728752	0.5115321	up
A_33_P3546363	TUSC8	5.0036097	5.601092	5.4288206	0.5974822	0.425211	0.5113466	up
A_23_P259692	PSAT1	5.4668646	6.541037	5.4152117	1.0741725	-0.051653	0.5112598	up
A_23_P215491	CCL24	3.620263	4.132582	4.1304493	0.5123191	0.5101862	0.5112527	up
A_33_P3436732	DSCR10	-0.58377	0.0644794	-0.209537	0.6482492	0.3742328	0.511241	up
A_33_P3270636	SHISA5	4.4224977	5.0506673	4.8165903	0.6281695	0.3940926	0.5111311	up
A_22_P00008685	lnc-KIAA1147-1	-2.478825	-2.141988	-1.793449	0.3368371	0.6853755	0.5111063	up
A_33_P3285565	CLDN3	3.4107828	4.0723023	3.7712498	0.6615195	0.360467	0.5109932	up
A_33_P3210848	ELFN1	4.8334303	5.435901	5.2528543	0.6024709	0.4194241	0.5109475	up
A_21_P0007422	lnc-RP11-890B15.2.1-2	-0.36235	0.2088518	0.088007	0.5712023	0.4503574	0.5107799	up
A_32_P218332	ACSF3	2.603654	3.0973048	3.131545	0.4936509	0.5278912	0.510771	up
A_24_P241815	JUNB	1.4415197	1.948194	1.9563761	0.5066743	0.5148563	0.5107653	up
A_23_P35293	GJB5	0.7064924	1.0077353	1.4267688	0.3012428	0.7202764	0.5107596	up
A_23_P213375	PCDHB2	0.4448624	0.7977648	1.1133885	0.3529024	0.6685262	0.5107143	up
A_24_P219552	NFE2L1	4.5899887	5.3356347	4.8655415	0.745646	0.2755528	0.5105994	up
A_33_P3239849	GPX1	5.073842	5.71516	5.453719	0.6413178	0.3798771	0.5105975	up
A_33_P3415216	MPV17	2.836646	3.3293471	3.365097	0.4927011	0.528451	0.510576	up
A_23_P200096	SPSB1	-1.279196	-0.491408	-1.04596	0.7877874	0.2332358	0.5105116	up
A_33_P3213419	LOC100129447	1.7389135	2.3640904	2.1344862	0.6251769	0.3955727	0.5103748	up
A_24_P331128	GNA15	-0.141315	0.4213672	0.3165975	0.5626826	0.4579129	0.5102978	up
A_33_P3302393	KLHL36	0.9514589	1.5995688	1.3237939	0.6481099	0.372335	0.5102224	up
A_19_P00803472	lnc-GDF10-2	1.9409552	2.5603213	2.3420143	0.6193662	0.4010592	0.5102127	up
A_19_P00321715	MIR100HG	-2.272336	-1.962156	-1.562142	0.3101792	0.7101932	0.5101862	up
A_21_P0000382	SNORD71	-1.579634	-1.019921	-1.119031	0.5597129	0.4606028	0.5101578	up
A_33_P3331641	ZNF503-AS2	0.4622541	1.2883377	0.6564798	0.8260837	0.1942258	0.5101547	up
A_23_P127584	NNMT	5.754079	6.637941	5.890359	0.883862	0.1362801	0.510071	up
A_21_P0007452	lnc-AP000679.2.1-1	-2.277432	-1.533953	-2.0008	0.7434793	0.2766328	0.510056	up
A_21_P0007854	lnc-HNF1A-1	-2.291387	-1.649305	-1.913656	0.6420822	0.3777309	0.5099065	up

A_33_P3264072	LOC100128593	-1.230111	-0.778773	-0.661717	0.4513373	0.5683937	0.5098655	up
A_23_P131096	POLRMT	3.351719	3.8649707	3.857892	0.5132518	0.5061731	0.5097125	up
A_22_P00015795	lnc-TARDBP-2	0.0801678	0.5614538	0.6182895	0.4812861	0.5381217	0.5097039	up
A_33_P3309365	SLC25A3P1	2.464466	2.9790502	2.969016	0.5145841	0.50455	0.509567	up
A_33_P3222228	FUT6	-0.216796	0.4422612	0.1432109	0.6590571	0.3600068	0.509532	up
A_33_P3389332	LOC100128531	-2.76632	-2.306381	-2.207514	0.4599392	0.5588057	0.5093725	up
A_23_P137423	IGSF8	0.8392062	1.3003559	1.396677	0.4611497	0.5574708	0.5093103	up
A_23_P414252	SNX8	1.6577225	2.3148465	2.019106	0.657124	0.3613834	0.5092537	up
A_23_P406385	FBXL16	2.5369225	2.894381	3.1979656	0.3574586	0.6610432	0.5092509	up
A_23_P120364	PPDPF	8.169872	8.716612	8.641632	0.5467396	0.4717598	0.5092497	up
A_23_P78802	PRKD2	3.0357132	3.5762153	3.5136642	0.5405021	0.4779511	0.5092266	up
A_22_P00003220	ASCL5	-1.707973	-1.133043	-1.264542	0.5749302	0.4434314	0.5091808	up
A_33_P3304516	ZNFX1	-1.737723	-1.43424	-1.023066	0.3034835	0.7146578	0.5090707	up
A_24_P252078	BTN3A2	1.2294922	1.7038479	1.7732639	0.4743557	0.5437717	0.5090637	up
A_23_P112406	GTF3C5	4.271806	4.7677464	4.793851	0.4959407	0.5220451	0.5089929	up
A_22_P00020762	lnc-AGAP2-2	-1.945551	-1.191104	-1.68206	0.7544479	0.2634916	0.5089698	up
A_22_P00020897	lnc-RPP38-2	-1.404517	-0.937161	-0.854402	0.4673557	0.5501151	0.5087354	up
A_24_P68631	HIST2H2AB	5.419881	5.9610953	5.895694	0.5412145	0.4758129	0.5085137	up
A_22_P00024575	lnc-UMODL1-5	0.6400638	1.187243	1.109889	0.5471792	0.4698253	0.5085022	up
A_33_P3285545	CLDN4	9.313864	9.824831	9.819658	0.5109673	0.5057945	0.5083809	up
A_23_P90359	NRTN	-2.571038	-2.221825	-1.903492	0.3492134	0.6675468	0.5083801	up
A_19_P00321697	LINC00313	-2.682093	-2.178287	-2.169437	0.5038064	0.5126567	0.5082315	up
A_23_P141656	DRG2	1.9334593	2.4687867	2.4143028	0.5353274	0.4808435	0.5080855	up
A_33_P3329419	DNM1	8.102347	8.703537	8.516964	0.6011896	0.4146166	0.5079031	up
A_24_P780052	RPSAP58	6.9887333	7.6688504	7.3242292	0.6801171	0.335496	0.5078065	up
A_33_P3302344	LOC100128320	0.7424922	1.0821915	1.4184008	0.3396993	0.6759086	0.5078039	up
A_21_P0011728	LINC00663	-2.846534	-2.086932	-2.590557	0.7596018	0.2559762	0.507789	up
A_24_P333567	MAN1B1-AS1	-1.128782	-0.394904	-0.847333	0.7338777	0.2814493	0.5076635	up
A_23_P78980	B3GNT3	5.8344297	6.392447	6.2916975	0.5580173	0.4572678	0.5076425	up
A_33_P3398236	COL28A1	-2.715821	-2.338303	-2.078121	0.3775172	0.6376998	0.5076085	up
A_21_P0009528	lnc-PIEZO2-3	-2.652442	-2.094886	-2.194797	0.5575559	0.4576449	0.5076004	up
A_33_P3628675	FAM86B1	-1.90523	-1.370875	-1.424624	0.5343547	0.4806051	0.5074799	up
A_33_P3298539	APOA1	-2.962605	-2.25811	-2.652157	0.7044947	0.3104475	0.5074711	up
A_23_P365817	PPP1R14B	7.1124067	7.7378497	7.501872	0.625443	0.3894653	0.5074542	up
A_23_P251505	NSUN5	3.535575	4.0177827	4.0682697	0.4822078	0.5326948	0.5074513	up
A_23_P63050	UROD	4.720148	5.2815766	5.173545	0.5614286	0.4533968	0.5074127	up
A_23_P80362	NHP2L1	6.5942354	7.146975	7.0562134	0.5527396	0.461978	0.5073588	up
A_23_P418493	ATPAF2	1.3079066	1.9221206	1.7083969	0.6142139	0.4004903	0.5073521	up
A_21_P0002612	lnc-GPR39-2	-1.673566	-1.120165	-1.212306	0.553401	0.4612608	0.5073309	up
A_22_P00025953	lnc-MSMP-1	-2.787727	-2.229578	-2.331306	0.5581496	0.4564216	0.5072856	up
A_23_P104509	FAM53B	-2.249176	-1.755681	-1.728126	0.4934955	0.5210505	0.507273	up
A_22_P00000173	LOC100505940	-0.283307	0.2716107	0.1762781	0.5549173	0.4595847	0.507251	up
A_33_P3272347	lnc-CCDC8-1	4.44602	4.943069	4.963196	0.4970489	0.5171757	0.5071123	up
A_24_P384636	C19orf43	3.1460533	3.6870112	3.6191483	0.5409579	0.4730949	0.5070264	up
A_21_P0000333	SNORA46	-1.29521	-0.692658	-0.884629	0.6025524	0.4105806	0.5065665	up
A_22_P00009701	LOC100131655	2.265421	2.9355464	2.608286	0.6701255	0.342865	0.5064953	up
A_23_P54291	DUOX1	-0.769058	-0.296371	-0.228797	0.4726872	0.5402608	0.506474	up
A_22_P00011599	lnc-PCGF2-1	-1.254117	-0.616404	-0.878933	0.637713	0.3751836	0.5064483	up
A_23_P251548	TEX261	1.8047218	2.2685304	2.3525734	0.4638085	0.5478516	0.5058301	up
A_21_P0000477	SNORA11B	0.7447829	1.2219601	1.2791	0.4771771	0.534317	0.5057471	up
A_33_P3304883	LOC100131372	-2.928155	-2.490046	-2.354851	0.4381094	0.5733049	0.5057072	up
A_33_P3251148	TSPO	8.710253	9.203421	9.2284355	0.4931679	0.5181828	0.5056753	up
A_22_P00003788	lnc-CDK5R1-2	1.8450975	2.5005155	2.2007627	0.6554179	0.3556652	0.5055416	up
A_23_P68942	RPL3	8.940797	9.639257	9.25338	0.6984606	0.312583	0.5055218	up
A_23_P254741	SOD3	1.027185	1.6879725	1.3774362	0.6607876	0.3502512	0.5055194	up
A_23_P325080	PTOV1	1.4019637	1.9809985	1.8338952	0.5790348	0.4319315	0.5054832	up

A_23_P17769	DDT	5.4440594	6.035728	5.8632126	0.5916686	0.4191532	0.5054109	up
A_33_P3237567	HLA-A	-0.267345	-0.361297	0.8371677	-0.093952	1.1045127	0.5052805	up
A_23_P140967	MEFV	-0.364616	0.2717886	0.0094194	0.6364045	0.3740354	0.5052199	up
A_21_P0014671	RPS13	8.638385	9.217749	9.069331	0.5793638	0.4309464	0.5051551	up
A_23_P258912	MYOM2	-0.711369	-0.066612	-0.345819	0.6447573	0.36555	0.5051537	up
A_33_P3298455	LINC01521	-1.818799	-1.499708	-1.127658	0.3190913	0.6911407	0.505116	up
A_21_P0011580	XLOC_l2_006025	0.2276726	0.7636347	0.7019343	0.5359621	0.4742618	0.5051119	up
A_23_P24763	RPS13	9.005106	9.555941	9.464429	0.5508347	0.4593229	0.5050788	up
A_32_P234604	PFN1	3.865759	4.345123	4.396433	0.4793639	0.530674	0.505019	up
A_21_P0009672	LOC400684	-0.443171	0.1582751	-0.034826	0.6014457	0.4083448	0.5048952	up
A_23_P98261	SSSCA1	4.626396	5.1434045	5.119128	0.5170083	0.4927321	0.5048702	up
A_24_P9285	LMAN2	4.48975	5.0189643	4.9701147	0.5292144	0.4803648	0.5047896	up
A_23_P217958	HK1	3.3978872	3.7808862	4.024191	0.3829989	0.6263037	0.5046513	up
A_22_P00017835	lnc-ZC3H3-1	-2.649186	-2.140627	-2.148563	0.5085595	0.5006228	0.5045911	up
A_24_P418619	RPS10	7.306136	7.916721	7.704587	0.6105847	0.3984509	0.5045178	up
A_33_P3663974	THRB	-2.451739	-1.739778	-2.154833	0.7119618	0.2969065	0.5044341	up
A_21_P0000297	SNORD62A	-0.301709	0.2638302	0.1414332	0.5655394	0.4431424	0.5043409	up
A_33_P3394769	TMEM8C	5.838255	6.3956094	6.2893953	0.5573545	0.4511404	0.5042474	up
A_22_P00013923	LOC101927755	-2.955982	-2.38627	-2.517257	0.5697126	0.438726	0.5042193	up
A_23_P320185	NDUFA11	5.7812033	6.4009337	6.1697435	0.6197305	0.3885403	0.5041354	up
A_22_P00013141	lnc-RNASEH1-2	-0.327146	0.3038893	0.0499592	0.6310349	0.3771048	0.5040698	up
A_19_P00808072	lnc-RTL1-2	1.516119	2.097053	1.9429979	0.5809341	0.4268789	0.5039065	up
A_23_P140146	IFI27L2	2.3768024	2.9271407	2.834241	0.5503383	0.4574385	0.5038884	up
A_33_P3361513	NLE1	0.639873	1.1862311	1.1009717	0.5463581	0.4610987	0.5037284	up
A_23_P98571	PPP1R32	-2.91227	-2.637524	-2.179962	0.2747455	0.7323074	0.5035264	up
A_21_P0005002	lnc-CCDC90A-5	2.3882456	3.0535846	2.7298288	0.665339	0.3415833	0.5034611	up
A_22_P00000976	LOC101929946	-3.222094	-2.499134	-2.938211	0.72296	0.2838824	0.5034212	up
A_23_P153022	KRTAP2-4	4.3537407	5.0375085	4.6765604	0.6837678	0.3228197	0.5032938	up
A_23_P259189	CLIC4	0.5604968	1.5518146	0.5754495	0.9913178	0.0149527	0.5031352	up
A_33_P3621701	LOC284933	1.9429836	2.4918923	2.400013	0.5489087	0.4570293	0.502969	up
A_33_P3309429	NUPL2	1.3701539	1.8655477	1.8806248	0.4953938	0.5104709	0.5029323	up
A_23_P24433	CTSF	2.6258564	3.333489	2.9235802	0.7076325	0.2977238	0.5026782	up
A_33_P3343545	NFKBIB	0.2360592	0.7026882	0.7746472	0.466629	0.5385881	0.5026085	up
A_33_P3215797	AHDC1	-0.582662	-0.149908	-0.010348	0.432754	0.5723143	0.5025342	up
A_22_P00015195	LOC100996842	3.1655874	3.6719298	3.6642284	0.5063424	0.498641	0.5024917	up
A_33_P3248024	STARD7-AS1	-1.453194	-1.064163	-0.837278	0.3890314	0.6159158	0.5024736	up
A_23_P342709	FBXO15	-1.836195	-1.650971	-1.016906	0.1852245	0.8192892	0.5022569	up
A_24_P23034	ZNFX1	0.5490737	1.2827334	0.8195539	0.7336597	0.2704802	0.50207	up
A_33_P3308626	MON1B	4.169135	4.7228436	4.6194153	0.5537086	0.4502802	0.5019944	up
A_33_P3391375	LANCL3	2.355404	2.957251	2.757514	0.6018472	0.4021101	0.5019787	up
A_23_P26184	DET1	1.0802407	1.7688355	1.3953896	0.6885948	0.3151488	0.5018718	up
A_24_P416660	FDX1L	3.2376842	3.7451625	3.7334585	0.5074782	0.4957743	0.5016263	up
A_23_P250564	PRKCE	-1.269176	-0.662551	-0.872825	0.6066246	0.3963509	0.5014877	up
A_24_P289404	RPS26	8.389818	8.921446	8.861114	0.5316277	0.4712954	0.5014615	up
A_22_P00011812	lnc-PHACTR4-2	-0.95869	-0.539959	-0.374755	0.4187312	0.5839353	0.5013332	up
A_33_P3209591	AQP3	-1.415354	-1.081474	-0.746574	0.3338804	0.6687803	0.5013304	up
A_23_P55281	HOXB7	-1.067961	-0.680434	-0.452865	0.3875265	0.6150956	0.5013111	up
A_23_P165186	LIN37	2.653204	3.3696008	2.9392176	0.7163968	0.2860136	0.5012052	up
A_33_P3250438	SMIM7	-0.10866	0.167295	0.617661	0.2759552	0.7263212	0.5011382	up
A_33_P3223780	LAMB2	1.9763651	2.82194	2.1330585	0.8455749	0.1566935	0.5011342	up
A_33_P3360341	GATA3	-1.288547	-0.834824	-0.740171	0.453723	0.5483756	0.5010493	up
A_23_P376372	MARVELD3	0.2386122	0.7431426	0.7361121	0.5045304	0.4974999	0.5010152	up
A_21_P0000479	SNORA11D	0.7935209	1.346633	1.2424173	0.553112	0.4488964	0.5010042	up
A_33_P3405459	C20orf195	0.3434382	0.7445741	0.9440007	0.4011359	0.6005626	0.5008493	up
A_24_P4334	RNF38	-1.639248	-0.960862	-1.316368	0.6783867	0.3228803	0.5006335	up
A_33_P3367860	CHRM1	-2.892638	-2.628434	-2.155714	0.2642036	0.7369244	0.500564	up

A_33_P3302312	IER5L	3.0384884	3.604611	3.4734612	0.5661225	0.4349728	0.5005476	up
A_23_P146849	APBA2	-3.666915	-2.7603	-3.572521	0.9066143	0.094394	0.5005041	up
A_33_P3233784	TMEM211	-1.461348	-0.723396	-1.198399	0.7379513	0.262949	0.5004502	up
A_24_P7584	LY6G5C	-1.691425	-1.083064	-1.298894	0.6083612	0.3925309	0.5004461	up
A_22_P00000148	LOC101928760	3.426467	4.027736	3.826048	0.6012693	0.399581	0.5004251	up
A_23_P45488	FAM3A	0.6593771	1.2548275	1.064539	0.5954504	0.4051619	0.5003061	up
A_22_P00006418	ERICH1-AS1	2.8987045	3.4996877	3.29784	0.6009831	0.3991356	0.5000594	up
A_21_P0002061	LOC101927577	-0.17544	0.3876057	0.2615762	0.563046	0.4370165	0.5000313	up
A_21_P0000691	LOC115110	-3.150065	-2.309101	-2.990995	0.8409636	0.1590695	0.5000166	up
A_22_P00003566	lnc-CCT5-1	-1.721972	-1.061863	-1.382123	0.660109	0.339849	0.499979	up
A_21_P0007662	lnc-TMEM132C-7	-3.196589	-2.390257	-3.003007	0.8063316	0.1935821	0.4999568	up
A_23_P142154	GRWD1	4.381419	4.5594087	5.203336	0.1779895	0.8219166	0.499953	up
A_22_P00008311	lnc-ITGB3BP-1	-2.746212	-2.299579	-2.192939	0.4466322	0.5532727	0.4999524	up
A_23_P21134	DDIT3	1.1324115	2.3925567	0.8721509	1.2601452	-0.260261	0.4999423	up
A_33_P3416583	ARAP1	-2.23014	-1.636921	-1.823555	0.5932183	0.4065847	0.4999015	up
A_24_P188878	RPL34	7.4083786	7.907367	7.9091005	0.4989886	0.5007219	0.4998553	up
A_23_P256663	GALR3	6.3747005	6.966722	6.7822905	0.5920215	0.4075899	0.4998057	up
A_21_P0014381	ECH1	-3.340974	-2.760608	-2.921758	0.5803664	0.4192166	0.4997915	up
A_23_P118633	SPATA20	2.4349375	3.00114	2.868063	0.5662026	0.4331255	0.4996641	up
A_24_P360269	RNASET2	4.060891	4.5669503	4.5540867	0.5060592	0.4931955	0.4996274	up
A_23_P301138	IP6K2	2.538803	3.1132236	2.9635773	0.5744205	0.4247742	0.4995973	up
A_21_P0002256	lnc-THNSL2-1	-0.845136	-0.312784	-0.378344	0.532352	0.4667926	0.4995723	up
A_21_P0009077	lnc-GINS2-3	-2.9414	-2.569193	-2.314628	0.3722069	0.6267722	0.4994896	up
A_21_P0006903	lnc-BTRC-2	-0.050126	0.5032907	0.3953524	0.5534163	0.445478	0.4994471	up
A_23_P123393	KCNQ3	0.1262488	0.6776166	0.5733972	0.5513678	0.4471483	0.499258	up
A_23_P88904	NTHL1	2.9640913	3.5393195	3.387107	0.5752282	0.4230156	0.4991219	up
A_33_P3416682	C2orf91	-2.60185	-2.03117	-2.174325	0.5706797	0.4275248	0.4991022	up
A_33_P3257232	TAF10	4.099184	4.661158	4.535327	0.5619741	0.4361429	0.4990585	up
A_21_P0000015	PARP9	1.1282272	1.5860066	1.6685648	0.4577794	0.5403376	0.4990585	up
A_23_P29994	RBPJ	3.042079	3.507873	3.5742226	0.4657941	0.5321436	0.4989689	up
A_33_P3260053	AIF1L	2.7563972	3.3398814	3.1708317	0.5834842	0.4144344	0.4989593	up
A_33_P3283906	NIP7	3.955553	4.4475875	4.4612284	0.4920344	0.5056753	0.4988549	up
A_33_P3238315	RBCK1	2.3105898	2.9652686	2.65337	0.6546788	0.3427801	0.4987295	up
A_24_P91140	RPL23A	4.435046	5.0440164	4.823492	0.6089702	0.3884459	0.498708	up
A_21_P0014544	LOC100507437	-2.436438	-2.111747	-1.763789	0.3246903	0.6726489	0.4986696	up
A_33_P3372332	CYBA	5.5089464	6.131741	5.8834906	0.6227946	0.3745441	0.4986694	up
A_23_P164284	CLDN7	5.1555567	5.766518	5.541791	0.6109614	0.3862343	0.4985979	up
A_23_P63232	CREB3L4	1.9686222	2.6746917	2.2597208	0.7060695	0.2910986	0.498584	up
A_23_P500282	PACS1	1.4602823	2.0574908	1.8602028	0.5972085	0.3999205	0.4985645	up
A_33_P3231923	LOC101927285	2.4252748	2.9533257	2.894247	0.5280509	0.4689722	0.4985116	up
A_23_P145074	PNRC1	3.3947992	4.0458007	3.7406626	0.6510015	0.3458633	0.4984324	up
A_22_P00004763	lnc-CTTN-3	0.2203741	0.7737274	0.6636305	0.5533533	0.4432564	0.4983048	up
A_32_P186731	ISM1	-1.469659	-1.085706	-0.857118	0.3839536	0.6125412	0.4982474	up
A_24_P121171	HINFP	0.765543	1.455368	1.0720563	0.6898251	0.3065133	0.4981692	up
A_21_P0006829	LINC00707	-3.287803	-2.794977	-2.784379	0.492826	0.5034244	0.4981252	up
A_21_P0010942	FAM25A	-0.373771	0.3703227	-0.121689	0.7440939	0.2520819	0.4980879	up
A_22_P00012401	lnc-PRKD1-2	-2.399898	-2.093082	-1.710606	0.3068152	0.6892915	0.4980533	up
A_24_P176714	B9D1	1.4493809	2.0217252	1.8729105	0.5723443	0.4235296	0.497937	up
A_22_P00017763	lnc-YTHDC2-1	-3.199272	-2.533155	-2.869649	0.6661172	0.3296237	0.4978705	up
A_23_P211345	TBX1	-2.389414	-2.10412	-1.679501	0.2852936	0.7099128	0.4976032	up
A_21_P0000358	SNORA71C	0.6329761	1.1732125	1.0876102	0.5402365	0.4546342	0.4974354	up
A_21_P0000376	SNORD32B	-0.449873	0.0381217	0.0569019	0.4879942	0.5067744	0.4973843	up
A_21_P0010415	lnc-APOL1-1	-0.059296	0.5200734	0.355948	0.5793695	0.4152441	0.4973068	up
A_33_P3274851	COX5B	6.6119366	7.106602	7.1117563	0.4946656	0.4998198	0.4972427	up
A_22_P00016940	TRPM2-AS	-1.757345	-1.160518	-1.359754	0.596827	0.3975911	0.4972091	up
A_19_P00804072	RNF213	-1.318641	-1.084098	-0.559274	0.2345433	0.7593675	0.4969554	up

A_23_P154962	RIMBP3	-0.221319	0.1998034	0.3512445	0.4211226	0.5725637	0.4968431	up
A_33_P3320804	IP6K1	5.7347307	6.3898273	6.072997	0.6550965	0.3382664	0.4966815	up
A_21_P0013169	XLOC_l2_013267	-0.964345	-0.458682	-0.476703	0.5056634	0.4876428	0.4966531	up
A_33_P3246774	XLOC_l2_015848	-3.661642	-3.063229	-3.267048	0.5984137	0.3945944	0.4965041	up
A_23_P147888	RPLP2	8.439406	8.981155	8.890473	0.541749	0.451067	0.496408	up
A_33_P3364268	LBH	-0.846554	-0.236948	-0.463347	0.6096053	0.3832064	0.4964058	up
A_21_P0005210	LOC101927354	-1.676183	-0.80131	-1.55852	0.8748736	0.1176634	0.4962685	up
A_23_P257583	DENND2A	-2.464247	-1.889455	-2.046613	0.5747922	0.4176343	0.4962132	up
A_32_P183904	SHF	2.1372175	2.614345	2.6524868	0.4771276	0.5152693	0.4961984	up
A_33_P3751889	TOM1	4.318803	4.9636054	4.6662292	0.6448026	0.3474264	0.4961145	up
A_23_P119143	ICAM5	-2.667842	-2.17872	-2.164751	0.4891229	0.5030913	0.4961071	up
A_24_P116378	GCOM1	1.2037544	1.9314046	1.4681287	0.7276502	0.2643743	0.4960122	up
A_33_P3239143	ZNF497	2.9290113	3.4931674	3.3568392	0.5641561	0.4278278	0.495992	up
A_33_P3351351	ARHGEF10	-1.391622	-0.907542	-0.884004	0.4840794	0.507618	0.4958487	up
A_22_P00007285	SLC45A4	-0.954854	-0.336582	-0.58144	0.6182714	0.373414	0.4958427	up
A_23_P121956	THG1L	2.4517727	3.1294904	2.7657175	0.6777177	0.3139448	0.4958313	up
A_33_P3346635	FAM20C	-3.029816	-2.307174	-2.761258	0.7226419	0.2685573	0.4955996	up
A_22_P00018348	lnc-ZSCAN10-3	-0.145298	0.4990382	0.201396	0.6443367	0.3466945	0.4955156	up
A_19_P00317878	LINC00659	-1.426112	-1.420178	-0.441111	0.0059347	0.9850011	0.4954679	up
A_33_P3454968	LOC645553	5.5969315	6.1973596	5.9873333	0.6004281	0.3904018	0.495415	up
A_21_P0005934	lnc-HMBOX1-1	1.0138164	1.7327013	1.2857385	0.7188849	0.2719221	0.4954035	up
A_23_P420551	CIT	3.3909073	3.9767795	3.7958279	0.5858722	0.4049206	0.4953964	up
A_23_P139795	MSI1	-1.60351	-1.033295	-1.182947	0.5702152	0.4205637	0.4953895	up
A_23_P207517	PDK2	1.8631525	2.4716182	2.2453947	0.6084657	0.3822422	0.495354	up
A_32_P182941	RPS3	7.111576	7.7961135	7.4169216	0.6845374	0.3053455	0.4949415	up
A_22_P00007003	lnc-GGCT-1	2.4159136	2.9238772	2.8977337	0.5079637	0.4818201	0.4948919	up
A_23_P31218	C7orf13	-1.465658	-0.949364	-0.992506	0.5162945	0.4731526	0.4947236	up
A_23_P369343	KLK8	3.1589594	3.648601	3.6587305	0.4896417	0.4997711	0.4947064	up
A_24_P221366	RPS15A	8.937899	9.546099	9.319039	0.6082001	0.3811407	0.4946704	up
A_33_P3277674	FBXL22	-2.678487	-2.128031	-2.240138	0.5504563	0.438349	0.4944027	up
A_23_P93844	TOMM7	8.603173	9.070597	9.124544	0.4674234	0.5213709	0.4943972	up
A_21_P0013971	ZNF385D	-1.397893	-0.916709	-0.890544	0.4811831	0.5073485	0.4942658	up
A_21_P0002496	lnc-CCDC93-3	-1.878627	-1.447931	-1.320916	0.430696	0.5577116	0.4942038	up
A_33_P3825869	CACNA1C	0.8839479	1.6220393	1.1338706	0.7380915	0.2499228	0.4940071	up
A_23_P39840	VAMP5	0.4863772	0.974144	0.9863586	0.4877667	0.4999814	0.4938741	up
A_33_P3259393	HAPLN3	2.6452532	3.032889	3.2449245	0.3876357	0.5996714	0.4936535	up
A_33_P3306659	LOC728254	-2.586589	-2.002502	-2.18343	0.5840862	0.4031591	0.4936227	up
A_23_P83200	AK8	-1.407208	-0.862958	-0.964594	0.54425	0.4426146	0.4934323	up
A_24_P831309	C1orf229	2.3855448	2.8935513	2.864253	0.5080066	0.4787083	0.4933574	up
A_23_P3312	ISLR	-1.133232	-0.598139	-0.681898	0.5350933	0.451334	0.4932137	up
A_33_P3364661	RHOA	0.5381389	1.0798206	0.9825363	0.5416818	0.4443975	0.4930396	up
A_33_P3246108	APOLD1	-1.600345	-1.08997	-1.124763	0.5103746	0.4755817	0.4929781	up
A_23_P219144	CLTA	6.481595	7.0544095	6.894657	0.5728145	0.4130621	0.4929383	up
A_23_P165180	RFXANK	5.4393215	5.8594604	6.004875	0.4201388	0.5655537	0.4928463	up
A_23_P87616	ATP5G2	7.8903627	8.366728	8.399631	0.4763651	0.5092678	0.4928165	up
A_21_P0012248	DGCR5	0.6740241	1.1595187	1.1740909	0.4854946	0.5000668	0.4927807	up
A_23_P116235	MDK	6.1518135	6.756636	6.5323668	0.6048226	0.3805533	0.4926879	up
A_33_P3264815	TLN2	2.2155333	2.9142213	2.5021257	0.698688	0.2865925	0.4926403	up
A_24_P22050	RAB20	0.6164875	1.144187	1.0740538	0.5276995	0.4575663	0.4926329	up
A_33_P3317376	RHOG	0.7282391	1.1988149	1.2425232	0.4705758	0.5142841	0.49243	up
A_23_P41143	APEH	2.432479	2.7274528	3.1223488	0.2949739	0.6898699	0.4924219	up
A_33_P6702964	SLC12A5	-0.401574	0.1772518	0.0042701	0.578826	0.4058442	0.4923351	up
A_22_P00010303	lnc-MTX1-1	-2.906471	-2.357788	-2.470559	0.5486825	0.4359112	0.4922968	up
A_22_P00017346	LOC101928000	0.8556638	1.4066858	1.2891479	0.5510221	0.4334841	0.4922531	up
A_23_P205875	SCAND2P	-0.977789	-0.369597	-0.601865	0.6081924	0.3759241	0.4920583	up
A_22_P00018346	ZNF213-AS1	4.5697374	5.0998397	5.023329	0.5301023	0.4535914	0.4918468	up

A_33_P3397150	PRR36	0.6783891	1.1746044	1.1658301	0.4962153	0.4874411	0.4918282	up
A_23_P304386	HIGD2A	3.3524294	3.780911	3.9074774	0.4284816	0.555048	0.4917648	up
A_33_P3389394	RPPH1	2.7160778	3.3454566	3.0702286	0.6293788	0.3541508	0.4917648	up
A_23_P63816	NRBF2	-0.331089	0.1735101	0.1477733	0.5045986	0.4788618	0.4917302	up
A_23_P304897	BDKRB2	1.4529777	2.2426362	1.6467714	0.7896586	0.1937938	0.4917262	up
A_33_P3238310	MAP3K7CL	-1.967905	-1.470399	-1.482115	0.4975052	0.4857893	0.4916473	up
A_23_P200396	TMEM234	0.3423677	0.8098321	0.8581381	0.4674645	0.5157704	0.4916174	up
A_23_P55319	FLOT2	3.0405645	3.4799304	3.584053	0.4393659	0.5434885	0.4914272	up
A_24_P364954	OGFOD3	0.9076815	1.4141355	1.3836913	0.506454	0.4760099	0.4912319	up
A_23_P352950	PNMA5	-1.051362	-0.674327	-0.446064	0.3770347	0.6052976	0.4911661	up
A_23_P401076	SUSD3	1.5441818	1.6450143	2.425439	0.1008325	0.8812571	0.4910448	up
A_23_P309989	ATG14	-2.808978	-2.087859	-2.54815	0.7211189	0.2608285	0.4909737	up
A_33_P3240053	UFSP1	-0.47383	-0.093257	0.1273766	0.3805733	0.6012068	0.49089	up
A_24_P88850	MRAS	1.7656059	2.095302	2.4176283	0.3296962	0.6520224	0.4908593	up
A_22_P00005052	lnc-DEGS2-4	1.3646216	2.039227	1.6715999	0.6746054	0.3069782	0.4907918	up
A_23_P129829	ORMDL3	5.503868	6.0108	5.9784813	0.5069318	0.4746132	0.4907725	up
A_33_P3377110	CYB561D1	2.876482	3.2771773	3.4572334	0.4006953	0.5807514	0.4907234	up
A_21_P0009773	lnc-BBC3-1	-2.258176	-0.742066	-2.793001	1.5161095	-0.534825	0.4906423	up
A_33_P3401284	RMRP	3.4781637	4.01477	3.9227934	0.5366063	0.4446297	0.490618	up
A_23_P333852	TTLL11	-2.983643	-2.8792	-2.106953	0.1044433	0.8766906	0.490567	up
A_23_P355536	USP54	3.4289503	4.188086	3.6507368	0.7591357	0.2217865	0.4904611	up
A_22_P00011704	lnc-PDZD7-1	1.105844	1.6284599	1.5641403	0.5226159	0.4582963	0.4904561	up
A_33_P3226678	EVPLL	-2.0658	-1.622575	-1.528116	0.4432254	0.537684	0.4904547	up
A_33_P3411075	FSCN1	6.981083	7.2750993	7.6679564	0.2940164	0.6868734	0.4904449	up
A_23_P78742	FLT3LG	-1.250437	-0.755475	-0.764897	0.4949627	0.4855399	0.4902513	up
A_24_P150068	HTR3A	-1.82092	-1.326209	-1.335158	0.4947109	0.4857616	0.4902363	up
A_19_P00315843	SCARNA16	3.5464125	4.206577	3.8664856	0.6601644	0.3200731	0.4901187	up
A_33_P3339276	ACSM5	-1.278624	-0.702377	-0.874688	0.5762467	0.4039359	0.4900913	up
A_23_P56213	GRAMD1A	-0.232908	0.3206949	0.1935453	0.5536027	0.4264531	0.4900279	up
A_23_P342825	FBXW4	1.8104992	2.443253	2.1577063	0.6327539	0.3472071	0.4899805	up
A_21_P0004202	lnc-C5orf38-1	-2.899755	-2.543984	-2.275902	0.3557701	0.6238523	0.4898112	up
A_22_P00020540	lnc-METTL11A-1	-3.053846	-2.509555	-2.618714	0.5442913	0.435132	0.4897116	up
A_33_P3272160	REXO4	5.3272915	5.7167306	5.9169445	0.3894391	0.589653	0.4895461	up
A_33_P3307253	AK5	-3.006402	-2.18541	-2.848302	0.820992	0.1580992	0.4895456	up
A_33_P3407549	SOWAHA	-2.865547	-2.470143	-2.282025	0.3954041	0.5835223	0.4894632	up
A_23_P90014	GPR108	0.9340816	1.4222546	1.4248104	0.488173	0.4907289	0.4894509	up
A_33_P3379377	MARS	0.4219303	1.198358	0.6242514	0.7764278	0.2023211	0.4893744	up
A_24_P419309	SNRNP40	1.021162	1.4842205	1.5368466	0.4630585	0.5156846	0.4893715	up
A_22_P00012024	TTN-AS1	-3.09139	-2.362502	-2.842041	0.728888	0.2493489	0.4891185	up
A_24_P285623	DGUOK	3.6022415	4.167269	4.0152397	0.5650277	0.4129982	0.489013	up
A_23_P35082	SESN2	-1.123872	0.215435	-1.485166	1.3393068	-0.361294	0.4890063	up
A_33_P3336113	TIGD3	-3.565944	-3.050053	-3.103838	0.5158911	0.462106	0.4889986	up
A_22_P00004177	lnc-CLIC5-1	-2.239787	-2.006268	-1.495432	0.2335196	0.7443552	0.4889374	up
A_33_P3336262	CFAP74	-2.250172	-1.53535	-1.98713	0.7148218	0.263042	0.4889319	up
A_22_P00017547	ADARB2-AS1	3.332014	3.8990855	3.7426128	0.5670714	0.4105988	0.4888351	up
A_22_P00008937	C12orf80	-0.107381	0.538753	0.2241402	0.6461344	0.3315215	0.488828	up
A_33_P3259662	ZNF134	-2.409994	-1.838127	-2.004254	0.571867	0.4057403	0.4888036	up
A_23_P20804	FAM219A	2.352888	2.935042	2.7482939	0.5821538	0.3954058	0.4887798	up
A_22_P00003277	CALML3-AS1	-2.939453	-2.534871	-2.366578	0.4045818	0.572875	0.4887284	up
A_24_P117672	SCAF1	0.9511046	1.333147	1.5461011	0.3820424	0.5949965	0.4885194	up
A_33_P3336992	MRPS18B	0.7264376	1.2500052	1.1797771	0.5235677	0.4533396	0.4884536	up
A_21_P0005178	lnc-CITED2-4	-3.228852	-2.615554	-2.865453	0.6132975	0.363399	0.4883482	up
A_23_P114952	TMEM9	4.0430527	4.619759	4.4430017	0.5767064	0.3999491	0.4883277	up
A_23_P256773	TSSC1	4.5911837	5.143291	5.0156317	0.5521073	0.424448	0.4882777	up
A_23_P207632	ATP2A3	-1.676883	-1.348454	-1.029029	0.3284288	0.6478539	0.4881413	up
A_21_P0008252	lnc-TSC22D1-1	0.7448244	1.3248429	1.1410794	0.5800185	0.396255	0.4881368	up

A_19_P00322687	ERVMER34-1	2.273018	2.9097075	2.6125345	0.6366897	0.3395166	0.4881032	up
A_33_P3283626	RNPC3	-2.83005	-2.197668	-2.486311	0.6323824	0.3437386	0.4880605	up
A_23_P139028	POU2F3	-2.208042	-1.697916	-1.742117	0.5101256	0.4659243	0.488025	up
A_23_P74928	MR1	-0.306748	0.4626026	-0.100253	0.769351	0.2064953	0.4879231	up
A_22_P00012085	lnc-PMM2-3	-0.350606	0.296555	-0.021983	0.647161	0.3286228	0.4878919	up
A_33_P3384932	NUDT8	3.3158731	3.7745128	3.8321562	0.4586396	0.516283	0.4874613	up
A_24_P160202	PANX2	-2.891579	-2.426657	-2.381595	0.4649224	0.5099843	0.4874533	up
A_33_P3256510	KCNK12	-0.771501	-0.385909	-0.18244	0.385592	0.5890603	0.4873261	up
A_23_P137016	SAT1	5.0431786	5.8133855	5.247469	0.7702069	0.2042904	0.4872486	up
A_33_P3408244	SPRNP1	3.0599937	3.5767274	3.517746	0.5167337	0.4577522	0.4872429	up
A_33_P3316273	CCL3	-0.603047	-0.138635	-0.093571	0.4644117	0.5094757	0.4869437	up
A_33_P3629247	ANKMY1	-0.106006	0.434094	0.3277469	0.5401001	0.433753	0.4869266	up
A_21_P0003793	lnc-C4orf27-1	-1.22666	-0.759007	-0.720563	0.4676528	0.5060964	0.4868746	up
A_23_P40192	CDH22	3.9621677	4.544144	4.353903	0.5819764	0.3917351	0.4868557	up
A_33_P3344492	SCARNA1	-2.11613	-1.930261	-1.328866	0.1858692	0.7872648	0.486567	up
A_23_P68529	ITPA	4.852232	5.339598	5.337942	0.4873662	0.4857101	0.4865382	up
A_23_P108708	RNF181	4.6823416	5.3088565	5.0286903	0.6265149	0.3463488	0.4864318	up
A_23_P142724	RPL37A	9.655185	10.226282	10.056772	0.5710974	0.4015875	0.4863425	up
A_33_P3238323	RBCK1	0.8771114	1.679421	1.0468869	0.8023095	0.1697755	0.4860425	up
A_23_P64499	MOB2	2.0308628	2.4915566	2.542077	0.4606938	0.5112143	0.4859541	up
A_24_P139943	HS1BP3	1.9772749	2.7123752	2.213911	0.7351003	0.2366362	0.4858682	up
A_33_P3274756	DOPEY1	-1.550594	-1.10016	-1.029373	0.4504342	0.5212212	0.4858277	up
A_23_P21747	CABP5	-0.144319	0.4103956	0.2724867	0.5547147	0.4168057	0.4857602	up
A_22_P00018950	TMEM231	-0.747621	-0.091852	-0.431962	0.6557694	0.3156595	0.4857144	up
A_22_P00002017	lnc-BEGAIN-1	9.053168	9.648905	9.428747	0.5957365	0.3755789	0.4856577	up
A_33_P3299982	USF2	6.4481306	7.054729	6.8127747	0.6065984	0.3646441	0.4856212	up
A_23_P88099	MCF2L	0.6404653	1.3446388	0.9072866	0.7041736	0.2668214	0.4854975	up
A_22_P00022185	TMEM184A	1.0286937	1.7837243	1.2444806	0.7550306	0.2157869	0.4854088	up
A_23_P81492	RPS14	9.120465	9.722717	9.488412	0.602252	0.3679466	0.4850993	up
A_23_P122863	GRB10	1.6351337	2.6811128	1.5593247	1.045979	-0.075809	0.485085	up
A_23_P368195	LSM11	-2.425059	-2.034401	-1.845668	0.3906584	0.579391	0.4850247	up
A_22_P00011520	lnc-PAXIP1-1	1.4417753	1.797523	2.0559864	0.3557477	0.6142111	0.4849794	up
A_33_P6471837	LBX2-AS1	-0.727258	-0.155177	-0.329449	0.5720811	0.397809	0.4849451	up
A_22_P00008800	lnc-KIRREL-1	-2.92607	-2.644006	-2.238376	0.2820633	0.6876938	0.4848785	up
A_23_P149419	GPATCH3	1.2709656	1.9248481	1.5868382	0.6538825	0.3158727	0.4848776	up
A_22_P00005122	lnc-DHX15-1	2.891531	3.4721122	3.2797623	0.5805812	0.3882313	0.4844062	up
A_23_P376735	ZNF524	3.3997784	3.951016	3.8171158	0.5512376	0.4173374	0.4842875	up
A_19_P00318243	CTD-3080P12.3	-1.250071	-0.849536	-0.682037	0.4005346	0.5680332	0.4842839	up
A_24_P181120	PFDN5	6.9353437	7.4917655	7.347412	0.5564218	0.4120684	0.4842451	up
A_24_P7600	FBXL7	-2.484368	-2.416211	-1.58407	0.0681562	0.9002974	0.4842268	up
A_24_P391104	RFX1	2.6345415	3.3348231	2.9025793	0.7002816	0.2680378	0.4841597	up
A_23_P38167	GPRC5C	4.6817408	5.3105006	5.0212355	0.6287599	0.3394947	0.4841273	up
A_33_P3232508	SMARCB1	4.8615437	5.4485583	5.2425566	0.5870147	0.3810129	0.4840138	up
A_23_P79331	TMBIM1	5.037033	5.6215987	5.4204645	0.5845656	0.3834314	0.4839985	up
A_21_P0000840	PXN-AS1	-1.045349	-0.521678	-0.601163	0.5236707	0.4441857	0.4839282	up
A_33_P3394105	SPATA2L	-0.849848	-0.398186	-0.333799	0.4516621	0.5160484	0.4838552	up
A_33_P3391387	LOC100268168	-1.951021	-1.187281	-1.747156	0.7637401	0.2038651	0.4838026	up
A_33_P3222218	NEURL1B	-2.095156	-1.483855	-1.738906	0.611301	0.3562503	0.4837756	up
A_23_P132285	MPST	5.0055876	5.7076097	5.271042	0.7020221	0.2654543	0.4837382	up
A_33_P3423365	GSN	2.9749823	3.493834	3.4235964	0.5188518	0.4486141	0.4837329	up
A_24_P308506	NAT8B	-3.111986	-2.539953	-2.71665	0.5720325	0.3953359	0.4836842	up
A_33_P3292198	SUN5	-3.58624	-3.179124	-3.026105	0.4071155	0.5601354	0.4836254	up
A_33_P3338300	CASZ1	0.8928685	1.4848056	1.268156	0.5919371	0.3752875	0.4836123	up
A_23_P166526	RIBC2	-1.973511	-1.492978	-1.486945	0.4805331	0.4865656	0.4835494	up
A_23_P300124	SMCR8	-1.196564	-0.5722	-0.854024	0.6243639	0.3425398	0.4834519	up
A_23_P213369	KIAA0141	1.151875	1.7502723	1.5200124	0.5983973	0.3681374	0.4832673	up

A_24_P283341	MICAL1	0.3272271	0.8263278	0.7945881	0.4991007	0.467361	0.4832308	up
A_21_P0004507	lnc-PIK3R1-1	-2.501903	-2.070381	-1.967018	0.4315226	0.5348852	0.4832039	up
A_24_P34611	SIX3	-3.108583	-2.645358	-2.60546	0.4632251	0.5031228	0.483174	up
A_33_P3213797	KCNK7	-1.142899	-0.766939	-0.553182	0.3759599	0.5897169	0.4828384	up
A_33_P3232006	UBL4A	3.140193	3.609305	3.6366177	0.4691119	0.4964247	0.4827683	up
A_22_P00013804	NUPR1L	-2.878091	-2.351384	-2.439364	0.5267067	0.4387271	0.4827169	up
A_23_P142294	ETHE1	3.2508955	3.8050075	3.6618242	0.554112	0.4109287	0.4825203	up
A_24_P173124	FLCN	-3.06422	-2.549148	-2.61428	0.5150719	0.4499402	0.482506	up
A_33_P3291636	ZNF74	-0.478195	0.0208893	-0.012401	0.4990845	0.4657941	0.4824393	up
A_23_P104188	ELF3	4.4605236	5.198485	4.687414	0.7379613	0.2268906	0.4824259	up
A_23_P49842	UNC119	1.9270372	2.4413676	2.3773584	0.5143304	0.4503212	0.4823258	up
A_33_P3383606	KCP	2.126585	2.6663032	2.5514545	0.5397182	0.4248695	0.4822938	up
A_33_P3332666	SPHK2	-0.922444	-0.349144	-0.531294	0.5733008	0.39115	0.4822254	up
A_21_P0000892	LOC100507351	-1.045886	-0.348598	-0.77892	0.6972876	0.2669659	0.4821267	up
A_33_P3279831	PNRC1	0.6902771	1.2892151	1.0555878	0.598938	0.3653107	0.4821243	up
A_21_P0005360	lnc-IL6-3	-2.690366	-2.575179	-1.84146	0.1151874	0.8489058	0.4820466	up
A_32_P176550	JMY	-1.723311	-1.038478	-1.444109	0.6848326	0.2792015	0.4820171	up
A_23_P16415	LRP3	4.1714973	4.6776147	4.6291666	0.5061173	0.4576693	0.4818933	up
A_33_P3378935	RBM14	-2.662831	-2.445999	-1.915902	0.2168317	0.7469287	0.4818802	up
A_22_P00010689	DUS2	0.4347844	0.8367691	0.9965277	0.4019847	0.5617433	0.481864	up
A_23_P14975	ENKD1	2.5535202	3.0928702	2.977727	0.53935	0.4242067	0.4817784	up
A_33_P3352687	LINC01001	5.5162144	6.151036	5.844906	0.6348214	0.3286915	0.4817564	up
A_23_P41009	TEX264	0.3142362	0.6334872	0.9584975	0.3192511	0.6442614	0.4817562	up
A_23_P47527	TMEM258	6.916029	7.3537393	7.4418154	0.4377103	0.5257864	0.4817484	up
A_33_P3348313	ELP6	1.1885061	1.7437611	1.5967331	0.5552549	0.408227	0.481741	up
A_23_P216966	PTGS1	-0.792774	0.0374689	-0.659555	0.8302431	0.1332197	0.4817314	up
A_33_P3273584	SCARNA2	-0.923933	-0.655461	-0.228976	0.2684717	0.6949568	0.4817143	up
A_21_P0003887	lnc-STX18-1	-2.853853	-2.462865	-2.281464	0.3909884	0.5723889	0.4816886	up
A_23_P109593	TBC1D22A	2.3269625	2.989345	2.6279535	0.6623826	0.3009911	0.4816868	up
A_21_P0012583	XLOC_l2_010636	1.7572289	2.3302417	2.1474962	0.5730128	0.3902674	0.4816401	up
A_24_P104512	EVPL	3.6654434	4.2154994	4.078641	0.550056	0.4131975	0.4816268	up
A_33_P3781228	CEBPB-AS1	-2.987884	-2.73299	-2.279754	0.2548943	0.7081306	0.4815124	up
A_33_P3268622	LY6D	5.3276815	6.1020303	5.5163565	0.7743487	0.1886749	0.4815118	up
A_33_P3376007	TXLNA	4.5303097	4.9519086	5.071519	0.4215989	0.5412092	0.4814041	up
A_21_P0003914	lnc-ENPP6-1	-2.253484	-1.885664	-1.658614	0.3678203	0.5948696	0.4813449	up
A_22_P00006670	LOC102723924	-2.299963	-1.737534	-1.899728	0.5624294	0.4002352	0.4813323	up
A_23_P120933	ATF4	8.587727	9.365368	8.772689	0.7776413	0.1849623	0.4813018	up
A_33_P3414789	FSD1	-1.29634	-0.633401	-0.99668	0.6629386	0.2996597	0.4812992	up
A_23_P120860	NIPSNAP1	2.7166328	3.0138144	3.3812943	0.2971816	0.6646614	0.4809215	up
A_33_P3284646	FBXL6	4.9283447	5.3666186	5.4515305	0.4382739	0.5231857	0.4807298	up
A_23_P139434	PRB2	-0.954665	-0.382361	-0.565631	0.5723038	0.3890343	0.480669	up
A_33_P3312743	KRTAP4-3	-2.685066	-2.181784	-2.227067	0.5032818	0.4579988	0.4806403	up
A_23_P42288	VWA7	1.0685172	1.5265856	1.5716519	0.4580684	0.5031347	0.4806015	up
A_23_P213000	WDR1	1.5527692	1.9285893	2.138113	0.3758202	0.5853438	0.480582	up
A_33_P3389148	STK11	2.755126	3.304338	3.166936	0.549212	0.4118099	0.480511	up
A_33_P3275722	LY6G6D	0.0581527	0.7651606	0.3120251	0.7070079	0.2538724	0.4804402	up
A_21_P0011028	LOC102724910	0.3853684	0.896431	0.8351784	0.5110626	0.44981	0.4804363	up
A_23_P149249	KRTCAP2	6.496049	6.9849057	6.968013	0.4888568	0.4719639	0.4804103	up
A_32_P14744	RPS15A	8.830036	9.437618	9.182813	0.6075821	0.3527765	0.4801793	up
A_22_P00015424	LOC101927159	-2.219225	-1.580661	-1.897579	0.6385632	0.3216457	0.4801045	up
A_23_P29185	TTC28	-1.311839	-0.90916	-0.754353	0.4026785	0.5574861	0.4800823	up
A_33_P3251617	LINC01118	-1.554276	-1.067793	-1.080641	0.4864831	0.4736352	0.4800592	up
A_22_P00004787	EIF1B-AS1	-2.305828	-1.677379	-1.974211	0.6284494	0.3316169	0.4800332	up
A_23_P14886	GNPTG	3.430726	3.9386783	3.8828392	0.5079522	0.4521132	0.4800327	up
A_22_P00017429	lnc-VMO1-1	-1.530463	-0.94198	-1.159107	0.5884829	0.371356	0.4799194	up
A_23_P433132	KRTCAP3	3.7637196	4.1975513	4.289607	0.4338317	0.5258875	0.4798596	up

A_24_P124370	PARVA	0.6827722	1.3773646	0.9478889	0.6945925	0.2651167	0.4798546	up
A_33_P3360684	CORO1B	0.3889756	0.8670678	0.870533	0.4780922	0.4815574	0.4798248	up
A_33_P3343473	ATRNL1	-2.634917	-1.943157	-2.367285	0.6917596	0.2676323	0.4796959	up
A_23_P48740	DIO2	-2.522922	-2.027816	-2.058693	0.495106	0.4642289	0.4796674	up
A_32_P122226	AMDHD1	-1.865512	-1.171127	-1.600775	0.6943851	0.2647367	0.4795609	up
A_33_P3415211	MPV17	2.8672867	3.375259	3.3183823	0.5079722	0.4510956	0.4795339	up
A_24_P135902	RPS2	9.801854	10.377775	10.184784	0.5759211	0.3829298	0.4794254	up
A_23_P166159	PDRG1	4.315653	4.6751075	4.914772	0.3594546	0.5991192	0.4792869	up
A_22_P00014685	lnc-SLC25A21-1	1.2509322	1.6059203	1.854496	0.3549881	0.6035638	0.479276	up
A_33_P3263284	LOC100130238	1.9904318	2.4459453	2.493433	0.4555135	0.5030012	0.4792573	up
A_33_P3293336	GFRA1	1.3976893	1.7587333	1.99512	0.3610439	0.5974307	0.4792373	up
A_33_P3260134	NTRK3	-2.925099	-2.345071	-2.546661	0.5800283	0.3784387	0.4792335	up
A_23_P18267	NPRL2	1.5534296	1.960218	2.104557	0.4067884	0.5511274	0.4789579	up
A_21_P0006135	lnc-IFNK-1	-0.650964	0.0390201	-0.383133	0.6899843	0.2678313	0.4789078	up
A_22_P00000284	lnc-AC009113.1-1	-2.762534	-2.140952	-2.426329	0.6215818	0.336205	0.4788934	up
A_21_P0000611	FLJ20021	3.5744848	4.292997	3.813672	0.7185121	0.2391872	0.4788497	up
A_23_P215832	ATP5J2	7.1810694	7.695001	7.6247663	0.5139318	0.443697	0.4788144	up
A_24_P112160	UPK3B	2.9207468	3.5758653	3.2230806	0.6551185	0.3023338	0.4787262	up
A_21_P0007446	lnc-RP11-201M22.1.1-2	-3.141702	-2.502653	-2.823328	0.6390495	0.3183739	0.4787117	up
A_23_P122387	MRPS18A	3.9313421	4.3802466	4.439639	0.4489045	0.508297	0.4786007	up
A_33_P3449097	TSPAN10	-0.231976	0.2791081	0.2141123	0.5110841	0.4460883	0.4785862	up
A_33_P3326025	KANK3	1.7230263	2.2188592	2.1843452	0.4958329	0.461319	0.4785759	up
A_22_P00004788	lnc-CXADR-1	-1.996546	-1.51596	-1.520069	0.4805861	0.4764776	0.4785318	up
A_23_P15705	PSMB6	6.3531218	6.8455443	6.8176603	0.4924226	0.4645386	0.4784806	up
A_33_P3390102	GIPC1	7.9740896	8.479916	8.424979	0.505826	0.4508896	0.4783578	up
A_23_P94800	S100A4	5.7548294	5.9947267	6.47141	0.2398973	0.7165804	0.4782388	up
A_24_P184769	WDR20	-1.011861	-0.259258	-0.808119	0.7526026	0.203742	0.4781723	up
A_19_P00326808	HOTAIR	-2.618118	-2.012167	-2.267876	0.6059511	0.3502414	0.4780963	up
A_22_P00015377	TMEM265	2.1338062	2.7352614	2.48849	0.6014552	0.3546839	0.4780695	up
A_19_P00322096	TDRG1	-0.126895	0.3745685	0.3277178	0.5014634	0.4546127	0.4780381	up
A_33_P3416882	ARL9	1.4623761	1.8432832	2.0375347	0.3809071	0.5751586	0.4780328	up
A_33_P3363016	BRF1	0.289525	1.0506124	0.4845023	0.7610874	0.1949773	0.4780323	up
A_24_P304071	IFIT2	-0.523634	-0.120826	0.0295095	0.4028087	0.553144	0.4779763	up
A_23_P96209	REEP4	2.2699833	2.8962903	2.599597	0.626307	0.3296137	0.4779604	up
A_23_P60225	GRHPR	3.8041506	4.4209476	4.143038	0.616797	0.3388872	0.4778421	up
A_24_P763243	EEF1A1	9.743295	10.363808	10.078455	0.620513	0.3351603	0.4778366	up
A_21_P0000308	SNORA9	3.0126562	3.537002	3.4439821	0.5243459	0.4313259	0.4778359	up
A_24_P343377	ATP5J2	7.107891	7.639031	7.532399	0.5311399	0.4245081	0.477824	up
A_23_P250212	SGK223	5.1711664	5.5790496	5.7189054	0.4078832	0.547739	0.4778111	up
A_33_P3288700	FAM207A	1.5400076	2.158733	1.8768435	0.6187253	0.3368359	0.4777806	up
A_33_P3336273	NAV3	-1.795456	-1.13593	-1.499704	0.6595259	0.2957521	0.477639	up
A_21_P0004987	lnc-GMDS-4	-0.520878	0.1027622	-0.189347	0.6236405	0.3315311	0.4775858	up
A_33_P3421571	RAPH1	2.112239	2.583693	2.5955782	0.4714541	0.4833393	0.4773967	up
A_32_P141262	CNOT7	2.0020504	2.5048318	2.4535513	0.5027814	0.4515009	0.4771412	up
A_33_P3262927	PCBP4	-0.386178	0.0128279	0.1690188	0.3990059	0.5551968	0.4771013	up
A_22_P00002296	lnc-C11orf1-1	-1.972591	-1.450203	-1.540844	0.522388	0.4317474	0.4770677	up
A_33_P3252414	TH	0.2515349	0.7071905	0.7499785	0.4556556	0.4984436	0.4770496	up
A_24_P678418	DICER1-AS1	-2.167517	-1.561474	-1.81985	0.6060433	0.3476677	0.4768555	up
A_24_P678104	STMN3	-0.674208	-0.401681	0.0065622	0.2725263	0.6807699	0.4766481	up
A_21_P0011479	XLOC_l2_005465	7.195195	7.687511	7.6561413	0.4923158	0.4609461	0.4766309	up
A_23_P64808	HOXC13	-1.631744	-1.211677	-1.098799	0.4200668	0.5329447	0.4765058	up
A_23_P367013	TAS2R60	-2.162172	-1.650571	-1.720792	0.511601	0.4413796	0.4764903	up
A_24_P264063	LOC729173	-2.589875	-2.103005	-2.123933	0.4868703	0.4659419	0.4764061	up
A_23_P5568	SFT2D3	0.2715206	1.0182137	0.4774184	0.7466931	0.2058978	0.4762955	up
A_22_P00001252	lnc-ANKRD34B-1	-1.020614	-0.540849	-0.548619	0.4797654	0.4719954	0.4758804	up

A_21_P0000325	SNORA37	2.4917154	3.0441232	2.891017	0.5524077	0.3993015	0.4758546	up
A_33_P3381338	TNXB	-1.47808	-0.820899	-1.183599	0.6571817	0.2944818	0.4758317	up
A_21_P0009830	lnc-CSRP2BP-1	-0.008696	0.4751096	0.4590783	0.4838052	0.4677739	0.4757895	up
A_22_P00014382	lnc-SEZ6L2-1	4.070407	4.6517673	4.440572	0.5813603	0.3701649	0.4757626	up
A_22_P00019886	HNF4A-AS1	-0.436434	0.114656	-0.036099	0.5510898	0.4003348	0.4757123	up
A_21_P0008801	lnc-ADAMTS17-1	-2.587076	-2.040778	-2.182041	0.5462978	0.4050345	0.4756662	up
A_33_P3424367	TRIM14	-1.457695	-1.219739	-0.744375	0.2379565	0.7133203	0.4756384	up
A_22_P00013839	lnc-RPL24-1	-2.604786	-1.871548	-2.386843	0.7332387	0.217943	0.4755908	up
A_22_P00002832	lnc-C2orf76-2	-2.295641	-1.733524	-1.906577	0.5621171	0.3890643	0.4755907	up
A_21_P0008478	lnc-SERPINA12-1	-1.170669	-0.832564	-0.558041	0.3381043	0.612628	0.4753661	up
A_23_P393034	HAS3	-3.295181	-2.84415	-2.795482	0.4510303	0.4996982	0.4753642	up
A_23_P100676	SMG6	-1.185467	-0.727587	-0.692704	0.4578795	0.492763	0.4753213	up
A_21_P0004484	LINC01018	1.2556925	1.9601445	1.5015507	0.704452	0.2458582	0.4751551	up
A_23_P68106	TMSB10	8.230369	8.735147	8.675732	0.5047789	0.445363	0.475071	up
A_22_P00000609	lnc-ACOT1-1	-3.504159	-3.038323	-3.019894	0.4658361	0.4842653	0.4750507	up
A_22_P00009815	lnc-MEPCE-1	8.89401	9.468176	9.269928	0.5741663	0.3759184	0.4750424	up
A_32_P352358	LOC650293	-0.434961	0.3469319	-0.266819	0.7818933	0.1681428	0.475018	up
A_23_P64712	TCTN2	-2.338931	-1.92433	-1.803613	0.4146009	0.5353174	0.4749591	up
A_24_P164731	TMED1	1.961276	2.4809937	2.391283	0.5197177	0.430007	0.4748623	up
A_23_P25674	CKB	0.524787	1.3351517	0.6641407	0.8103647	0.1393538	0.4748592	up
A_33_P3274134	TMEM151B	0.0054717	0.5906153	0.3699913	0.5851436	0.3645196	0.4748316	up
A_23_P383915	BTBD16	-1.327236	-0.88613	-0.819017	0.4411054	0.5082183	0.4746618	up
A_21_P0000186	ZNF561-AS1	-2.720644	-2.397612	-2.094657	0.3230324	0.6259878	0.4745101	up
A_22_P00025371	LOC401480	-1.286596	-0.70414	-0.920094	0.5824566	0.3665023	0.4744794	up
A_23_P116512	PRR5L	0.5875292	1.1002755	1.0237317	0.5127463	0.4362025	0.4744744	up
A_21_P0000260	SNORD58A	-0.249361	0.1596751	0.2902565	0.4090362	0.5396175	0.4743269	up
A_23_P159663	UXT	5.770568	6.279451	6.2103357	0.508883	0.4397678	0.4743254	up
A_33_P3375476	KLHDC4	5.304143	5.6772456	5.8794785	0.3731027	0.5753355	0.4742191	up
A_33_P3383856	TREX2	-1.469518	-1.007858	-0.982834	0.4616594	0.4866834	0.4741714	up
A_23_P89884	TRIM28	5.4188166	5.952782	5.833108	0.5339656	0.4142914	0.4741285	up
A_19_P00805950	LOC101928673	0.5273352	1.085824	0.9170332	0.5584889	0.389698	0.4740934	up
A_22_P00008308	FOXD3-AS1	6.424117	6.9824853	6.81345	0.5583682	0.3893328	0.4738505	up
A_33_P3325933	PQBP1	2.0036135	2.4943871	2.4605083	0.4907737	0.4568949	0.4738343	up
A_33_P3305105	VWA5A	-1.109802	-0.380197	-0.891809	0.7296052	0.2179937	0.4737995	up
A_33_P3216448	COL11A2	-0.319353	0.1962729	0.1122136	0.5156255	0.4315662	0.4735959	up
A_24_P37519	LZTFL1	-1.824425	-1.23559	-1.466113	0.5888352	0.3583121	0.4735737	up
A_23_P73763	LAGE3	5.2185173	5.8224416	5.561446	0.6039243	0.3429289	0.4734266	up
A_23_P53588	WNT5B	-3.221511	-3.031738	-2.464432	0.1897731	0.7570787	0.4734259	up
A_33_P3296024	SALL3	0.357532	0.8438344	0.8178163	0.4863024	0.4602842	0.4732933	up
A_23_P415882	TIMM50	4.9431944	5.5232005	5.30958	0.5800061	0.3663855	0.4731958	up
A_21_P0012866	XLOC_l2_011872	-2.520675	-2.12787	-1.96715	0.3928049	0.5535247	0.4731648	up
A_22_P00007594	lnc-HDDC3-1	2.1440096	2.7911916	2.4430733	0.647182	0.2990637	0.4731228	up
A_23_P418031	IFFO2	3.6265717	4.0347333	4.1641035	0.4081616	0.5375319	0.4728467	up
A_23_P200838	KMO	-1.952852	-1.442717	-1.517627	0.5101352	0.4352255	0.4726804	up
A_23_P37718	CNGB1	-3.299171	-2.914185	-2.738803	0.3849864	0.5603681	0.4726772	up
A_23_P160618	SH2D2A	2.3608294	2.7978396	2.868888	0.4370103	0.5080586	0.4725344	up
A_22_P00004017	LOC389641	-0.997633	-0.515939	-0.534308	0.4816947	0.463326	0.4725103	up
A_23_P132027	SPAG4	-2.324477	-2.166926	-1.537087	0.1575513	0.7873898	0.4724705	up
A_33_P3412975	SIX5	3.847518	4.462524	4.177453	0.615006	0.3299351	0.4724705	up
A_23_P83298	PRRX2	1.5502534	1.9782777	2.0670595	0.4280243	0.5168061	0.4724152	up
A_33_P3213747	ZNHIT2	1.3383522	1.795991	1.82547	0.4576387	0.4871178	0.4723783	up
A_23_P169437	LCN2	5.2224236	5.9321733	5.4571867	0.7097497	0.2347632	0.4722564	up
A_33_P3393971	PKP1	0.5426092	1.1543441	0.8751593	0.6117349	0.3325501	0.4721425	up
A_33_P3229122	HIST1H2BF	2.0724974	2.571011	2.518013	0.4985137	0.4455156	0.4720147	up
A_21_P0003866	LOC101929996	-3.460982	-3.011942	-2.966071	0.4490404	0.4949107	0.4719756	up
A_22_P00015999	LOC101928106	-3.243477	-2.954972	-2.588039	0.2885048	0.655438	0.4719714	up

A_24_P185709	EPB41L1	1.0806789	1.7733536	1.3315816	0.6926746	0.2509027	0.4717886	up
A_33_P3288649	HOXA10	3.7481441	4.3473296	4.0924788	0.5991855	0.3443346	0.47176	up
A_23_P75402	NDUFS3	5.555937	6.035682	6.019535	0.4797454	0.4635983	0.4716718	up
A_23_P60240	PIGO	2.2774982	2.6774964	2.8206291	0.3999982	0.5431309	0.4715646	up
A_24_P194714	UBALD2	6.6000433	7.047631	7.0954714	0.4475875	0.4954281	0.4715078	up
A_23_P137848	MRPL24	5.031769	5.562159	5.4442606	0.5303903	0.4124918	0.471441	up
A_23_P138717	RGS10	6.1725464	6.7153535	6.572592	0.5428071	0.4000454	0.4714263	up
A_19_P00319019	SNHG5	5.8782654	6.6010213	6.098068	0.7227559	0.2198029	0.4712794	up
A_33_P3269803	CLSTN3	4.457554	4.97969	4.8779497	0.5221362	0.4203959	0.471266	up
A_23_P5731	FAHD2A	1.6485729	2.013329	2.2256556	0.3647561	0.5770826	0.4709194	up
A_23_P113789	USP19	-1.225306	-0.806178	-0.702687	0.4191279	0.5226188	0.4708734	up
A_33_P3388870	BLOC1S1	2.7421227	3.2800927	3.1458702	0.5379701	0.4037476	0.4708588	up
A_21_P0001989	lnc-TMEM18-11	-1.648814	-0.983782	-1.372352	0.6650319	0.2764621	0.470747	up
A_23_P204144	KRT85	-0.69113	-0.715196	0.2744041	-0.024066	0.9655337	0.4707339	up
A_33_P3313411	ARHGAP33	6.1042747	6.658484	6.491308	0.5542092	0.3870335	0.4706213	up
A_23_P69109	PLSCR1	3.473549	3.7268796	4.161442	0.2533307	0.6878929	0.4706118	up
A_21_P0013468	FAM86B3P	-3.096088	-2.564491	-2.686609	0.5315969	0.4094796	0.4705383	up
A_23_P50052	C18orf12	1.1759973	1.6817756	1.6109824	0.5057783	0.4349852	0.4703817	up
A_23_P20752	CDK20	1.6535969	2.045054	2.2028723	0.3914571	0.5492754	0.4703662	up
A_22_P00003767	lnc-CDK12-2	-1.863225	-1.440198	-1.345645	0.4230266	0.5175796	0.4703031	up
A_33_P3349552	CASKIN1	5.5533323	6.0819287	5.9652233	0.5285964	0.411891	0.4702437	up
A_33_P3262580	ENTPD1	0.3365612	0.9004188	0.7131167	0.5638576	0.3765554	0.4702065	up
A_23_P55136	RANGRF	0.6006994	1.0255952	1.1160846	0.4248958	0.5153852	0.4701405	up
A_24_P398950	UNC45A	3.1309538	3.6199737	3.58218	0.4890199	0.4512262	0.4701231	up
A_33_P3358799	SLC35C2	2.9012222	3.4001393	3.3422565	0.4989171	0.4410343	0.4699757	up
A_24_P251688	ABCF3	4.074316	4.6446843	4.443733	0.5703683	0.3694172	0.4698928	up
A_22_P00017913	LOC100996664	-1.342557	-0.823614	-0.921985	0.5189428	0.4205718	0.4697573	up
A_22_P00017466	lnc-VSIG2-1	-2.127111	-1.720358	-1.594406	0.4067526	0.5327048	0.4697287	up
A_33_P3351279	GPR37L1	-2.271814	-1.728065	-1.876373	0.5437498	0.3954415	0.4695957	up
A_22_P00007064	LOC100505938	4.1303463	4.7266583	4.4730797	0.5963121	0.3427334	0.4695227	up
A_23_P254415	RPSAP58	8.075228	8.660856	8.428629	0.5856285	0.3534012	0.4695148	up
A_21_P0000622	WDR86-AS1	-3.146236	-2.401226	-2.952272	0.7450101	0.1939645	0.4694873	up
A_22_P00002297	TMEM258	5.99199	6.460013	6.4629097	0.4680228	0.4709196	0.4694712	up
A_24_P230282	VCX2	-2.927918	-2.160079	-2.756829	0.767839	0.1710885	0.4694637	up
A_23_P68601	CST7	-0.314936	0.728663	-0.41963	1.0435991	-0.104693	0.4694528	up
A_22_P00025959	PTPN14	-1.949847	-1.152349	-1.808565	0.7974982	0.1412826	0.4693904	up
A_21_P0013078	XLOC_l2_012953	7.1744547	7.6727347	7.614935	0.4982801	0.4404802	0.4693801	up
A_23_P147450	SPG21	5.1725607	5.6016555	5.6822214	0.4290948	0.5096607	0.4693778	up
A_23_P111000	PSMB9	3.4772377	3.907352	3.9857264	0.4301143	0.5084887	0.4693015	up
A_33_P3330109	HLA-C	7.1380587	7.615948	7.5985813	0.4778895	0.4605227	0.4692061	up
A_33_P3338121	LAMB3	7.309531	8.374804	7.1825066	1.0652723	-0.127025	0.4691238	up
A_22_P00009654	lnc-MAT2B-3	2.34379	2.8139386	2.811695	0.4701486	0.467905	0.4690268	up
A_23_P119593	EPHX3	3.252843	3.705727	3.7380066	0.4528842	0.4851637	0.469024	up
A_23_P31873	RAB11FIP1	3.6425972	4.2800536	3.943162	0.6374564	0.3005648	0.4690106	up
A_21_P0000660	LOC100499489	0.7646308	1.3760891	1.0907736	0.6114583	0.3261428	0.4688006	up
A_33_P3256054	MLST8	3.504387	3.9407325	4.005583	0.4363456	0.5011959	0.4687707	up
A_23_P390068	MISP	4.3090706	4.9448514	4.6107435	0.6357808	0.3016729	0.4687269	up
A_22_P00021327	lnc-TMEM85-1	-2.979172	-2.411765	-2.609247	0.5674074	0.3699253	0.4686663	up
A_21_P0009476	lnc-KATNAL2-4	-0.304019	0.1676507	0.16152	0.4716702	0.4655395	0.4686048	up
A_23_P501745	PEX10	-0.374276	0.2684946	-0.080043	0.6427703	0.2942324	0.4685013	up
A_33_P3371718	SAT1	3.9216518	4.694984	4.0851545	0.7733321	0.1635027	0.4684174	up
A_23_P126689	USP21	-1.150948	-0.59798	-0.767085	0.5529685	0.3838635	0.468416	up
A_23_P15542	HSD17B1	2.8986235	3.2322097	3.5018682	0.3335862	0.6032448	0.4684155	up
A_33_P3237784	PORCN	2.8180532	3.3690505	3.2037926	0.5509973	0.3857393	0.4683683	up
A_23_P302094	IMP4	4.317239	4.7861676	4.784767	0.4689288	0.4675283	0.4682286	up
A_21_P0005776	lnc-PTP4A3-1	-3.015631	-2.69768	-2.39722	0.3179514	0.6184111	0.4681813	up

A_33_P3377364	ITGB4	5.8103237	6.507117	6.049674	0.6967931	0.2393503	0.4680717	up
A_33_P3284715	SCARNA7	1.2338481	1.8311133	1.5725188	0.5972652	0.3386707	0.467968	up
A_24_P417162	TBL1X	1.704256	2.5517402	1.7926869	0.8474841	0.0884309	0.4679575	up
A_24_P191013	CYP4A11	-2.356898	-1.811996	-1.966276	0.5449023	0.3906221	0.4677622	up
A_21_P0000493	SCARNA14	2.3639107	2.8803897	2.7829504	0.516479	0.4190397	0.4677594	up
A_24_P142228	RPL13	10.009633	10.543062	10.41169	0.5334292	0.4020567	0.4677429	up
A_23_P391607	ARRDC1	3.0697985	3.6024213	3.4724836	0.5326228	0.4026852	0.467654	up
A_33_P3220612	FAM89B	3.9014807	4.3493	4.3888836	0.4478192	0.4874029	0.4676111	up
A_23_P71270	AZGP1	0.1073017	0.2994165	0.8503079	0.1921148	0.7430062	0.4675605	up
A_33_P3354322	GPX1	6.732423	7.3099055	7.0900517	0.5774827	0.3576288	0.4675558	up
A_23_P98167	UNC93B1	1.2805791	1.8746057	1.6216569	0.5940266	0.3410778	0.4675522	up
A_33_P3262452	PEX26	1.2285705	1.5952182	1.7967644	0.3666477	0.5681939	0.4674208	up
A_23_P371039	NTSR1	2.703395	3.0006251	3.3409166	0.2972302	0.6375217	0.467376	up
A_24_P177604	PPP1R3F	-1.891038	-1.572616	-1.274721	0.3184223	0.6163173	0.4673698	up
A_22_P00006069	lnc-FAM13B-1	-1.263882	-0.670141	-0.923051	0.5937405	0.3408308	0.4672856	up
A_32_P919718	TMEM105	0.9487505	1.3846078	1.4474125	0.4358573	0.498662	0.4672597	up
A_23_P357104	ANXA6	-2.520902	-2.167109	-1.940249	0.3537929	0.5806525	0.4672227	up
A_24_P522631	TMEM201	4.0905504	4.61731	4.498214	0.5267596	0.4076634	0.4672115	up
A_33_P3325068	SIRT3	-2.088356	-1.56655	-1.67588	0.5218062	0.4124765	0.4671414	up
A_24_P115199	FOXK1	0.8434982	1.4766893	1.144557	0.6331911	0.3010588	0.4671249	up
A_23_P325631	SKI	-0.264117	0.1251864	0.280766	0.3893032	0.5448828	0.467093	up
A_23_P209195	C19orf12	0.0087752	0.7145062	0.2367954	0.7057309	0.2280202	0.4668755	up
A_23_P94857	MAN1B1	2.517912	3.0861335	2.883379	0.5682216	0.3654671	0.4668443	up
A_32_P150391	C17orf100	-0.930486	-0.588889	-0.338432	0.3415966	0.5920539	0.4668253	up
A_23_P164691	ICAM3	2.646617	3.1126752	3.1141691	0.4660583	0.4675522	0.4668052	up
A_23_P123454	NUDT18	-0.819944	-0.267252	-0.439108	0.5526924	0.3808355	0.466764	up
A_23_P103226	FAAH	0.1481886	0.8644218	0.3653545	0.7162333	0.217166	0.4666996	up
A_24_P201491	MRPL49	4.4718685	4.935982	4.9410315	0.4641137	0.4691629	0.4666383	up
A_33_P3276638	HMBOX1	0.6066756	1.2769375	0.869544	0.6702619	0.2628684	0.4665651	up
A_23_P51291	PARS2	1.087812	1.6864872	1.4220119	0.5986753	0.3341999	0.4664376	up
A_33_P3222341	PITPNC1	-2.319874	-1.873406	-1.833661	0.4464688	0.4862132	0.466341	up
A_33_P3270852	RPS29	9.340892	9.899189	9.715225	0.5582972	0.3743334	0.4663153	up
A_33_P3269359	SPPL3	0.6048546	1.1944847	0.9478517	0.5896301	0.3429971	0.4663136	up
A_23_P139600	RASAL1	2.9431944	3.4359956	3.3828993	0.4928012	0.4397049	0.4662531	up
A_33_P3330683	XLOC_l2_008285	7.1740303	7.706056	7.574504	0.5320258	0.4004736	0.4662497	up
A_22_P00004473	LOC100129917	1.5953417	2.1871972	1.9358168	0.5918555	0.3404751	0.4661653	up
A_21_P0007088	lnc-C10orf137-1	-0.411862	0.0260453	0.0825534	0.4379072	0.4944153	0.4661613	up
A_23_P367071	UBE2DNL	-1.036434	-0.655099	-0.48572	0.3813353	0.550714	0.4660246	up
A_23_P386241	FAM110A	5.4538946	5.913058	5.9266233	0.4591632	0.4727287	0.465946	up
A_23_P122915	BRI3	7.3038797	7.9018044	7.6377296	0.5979247	0.3338499	0.4658873	up
A_23_P69362	CCDC51	3.1350079	3.7465653	3.455143	0.6115575	0.3201351	0.4658463	up
A_23_P414899	TTC17	-0.104852	0.5526161	0.1693087	0.6574678	0.2741604	0.4658141	up
A_23_P124300	BCO1	-3.043361	-2.809927	-2.34521	0.2334347	0.6981518	0.4657932	up
A_19_P00316427	CDKN2B-AS1	-2.671943	-2.001102	-2.411235	0.6708407	0.2607081	0.4657744	up
A_24_P97931	CSNK2B	4.478341	5.036059	4.852065	0.5577178	0.373724	0.4657209	up
A_33_P3274935	HID1	3.5945978	4.138471	3.982108	0.5438733	0.3875103	0.4656918	up
A_22_P00012889	LINC01234	-1.706067	-1.052804	-1.428346	0.6532631	0.2777209	0.465492	up
A_33_P3285868	CYGB	6.5540266	7.1451507	6.8938637	0.5911241	0.3398371	0.4654806	up
A_22_P00015434	lnc-SSR3-2	-0.721011	-0.043757	-0.467381	0.6772542	0.2536297	0.4654419	up
A_23_P90296	DYRK1B	-0.34003	0.2476282	0.0030003	0.5876579	0.34303	0.4653439	up
A_33_P3403082	LINC00176	1.9231005	2.457272	2.3193626	0.5341716	0.3962622	0.4652169	up
A_21_P0009590	lnc-MBP-1	1.8682079	2.4436498	2.223031	0.5754418	0.3548231	0.4651325	up
A_23_P77529	MSLN	3.64754	4.4264455	3.798708	0.7789054	0.1511679	0.4650366	up
A_22_P00016935	TSPAN33	-2.457831	-2.174312	-1.811356	0.2835188	0.6464746	0.4649967	up
A_23_P123265	SUMF2	0.7101979	1.3541121	0.9960494	0.6439142	0.2858515	0.4648828	up
A_22_P00014977	lnc-SMARCC2-3	0.2604647	0.7791452	0.6714397	0.5186806	0.410975	0.4648278	up

A_19_P00317178	SNHG5	3.0319395	3.750567	3.2425995	0.7186275	0.21066	0.4646437	up
A_23_P204375	LPAR5	0.2235184	0.6960292	0.6801477	0.4725108	0.4566293	0.46457	up
A_21_P0000179	TMEM147	6.9812603	7.460318	7.431327	0.4790578	0.4500666	0.4645622	up
A_33_P3217998	AKR7L	2.880248	3.196735	3.4928493	0.3164868	0.6126013	0.4645441	up
A_32_P11471	FAU	9.226206	9.713512	9.667868	0.4873066	0.4416618	0.4644842	up
A_24_P56130	MYL6	6.813555	7.3797927	7.176196	0.5662379	0.3626413	0.4644396	up
A_33_P3222069	SPHK1	3.0749264	3.5743976	3.504263	0.4994712	0.4293366	0.4644039	up
A_23_P111092	OR2H1	3.7855873	4.2495036	4.25039	0.4639163	0.4648027	0.4643595	up
A_33_P3330125	DIABLO	8.131861	8.6747265	8.517542	0.5428658	0.3856812	0.4642735	up
A_21_P0006174	lnc-C9orf79-1	-1.537173	-1.043924	-1.102153	0.4932489	0.4350195	0.4641342	up
A_24_P48587	PSENEN	4.5879583	5.1035237	5.0005913	0.5155654	0.4126329	0.4640992	up
A_23_P72025	SLC25A20	-0.61645	0.0096331	-0.314372	0.6260829	0.3020783	0.4640806	up
A_24_P405054	SZRD1	1.1605201	1.6324186	1.61656	0.4718986	0.4560399	0.4639692	up
A_33_P3401571	MUC2	-1.395916	-0.929284	-0.934648	0.4666314	0.4612675	0.4639494	up
A_21_P0008130	lnc-SACS-1	-1.03015	-0.548791	-0.583736	0.4813585	0.446414	0.4638863	up
A_23_P208937	TLE6	-1.482402	-1.029614	-1.007445	0.4527884	0.4749565	0.4638724	up
A_33_P3267263	RNVU1-18	6.010419	6.493006	6.4555206	0.4825873	0.4451017	0.4638445	up
A_23_P23017	C1orf123	2.7860775	3.2606883	3.2391043	0.4746108	0.4530268	0.4638188	up
A_23_P117602	GZMB	-2.968618	-2.565303	-2.444382	0.4033146	0.5242355	0.4637751	up
A_33_P3256334	PRB3	0.2521009	0.781045	0.650403	0.528944	0.3983021	0.463623	up
A_22_P00025404	LSP1	4.2842035	4.7573304	4.73831	0.4731269	0.4541063	0.4636166	up
A_32_P18547	YBEY	0.6337056	1.1917696	1.0027962	0.558064	0.3690906	0.4635773	up
A_33_P6571454	ILDR2	0.37395	0.8828702	0.7917857	0.5089202	0.4178357	0.463378	up
A_23_P208866	GMFG	-1.68859	-1.334935	-1.11589	0.3536544	0.5727	0.4631772	up
A_23_P365418	ZNF783	-0.425086	-0.047931	0.1241078	0.3771553	0.5491939	0.4631746	up
A_33_P3217495	SLC5A2	-2.876745	-2.34128	-2.485942	0.535465	0.3908026	0.4631338	up
A_23_P56256	POLR2I	5.7733326	6.218714	6.254216	0.4453816	0.4808836	0.4631326	up
A_24_P329065	BTN3A1	-1.082293	-0.647917	-0.590457	0.4343753	0.4918361	0.4631057	up
A_33_P3405916	LINC00273	-3.248093	-2.737842	-2.832402	0.5102518	0.4156916	0.4629717	up
A_23_P65174	PHF11	1.7121859	2.0482097	2.3019676	0.3360238	0.5897818	0.4629028	up
A_22_P00012003	PLCG1	3.0627604	3.6132865	3.437974	0.5505261	0.3752136	0.4628699	up
A_24_P29723	POR	4.0187025	4.5795064	4.3835125	0.5608039	0.36481	0.462807	up
A_24_P914940	MEF2BNB	0.7121024	1.1696906	1.1800957	0.4575882	0.4679933	0.4627907	up
A_23_P392222	SWI5	2.1345835	2.5405211	2.6542253	0.4059377	0.5196419	0.4627898	up
A_24_P64329	STK32C	1.6733398	1.9261193	2.3460903	0.2527795	0.6727505	0.462765	up
A_23_P138194	NCF2	0.1500011	0.8051224	0.419929	0.6551213	0.269928	0.4625246	up
A_23_P92196	RETNLB	-1.4299	-1.022806	-0.912087	0.407094	0.5178132	0.4624536	up
A_33_P3310696	UBE2J2	3.783742	4.2117696	4.2804985	0.4280276	0.4967566	0.4623921	up
A_23_P87827	UNC119B	3.0780983	3.6263509	3.4544964	0.5482526	0.3763981	0.4623254	up
A_33_P3377851	PNMAL2	-2.89371	-2.887143	-1.975658	0.0065668	0.9180512	0.462309	up
A_21_P0014496	LOC101927752	0.5025096	1.0796156	0.8498874	0.577106	0.3473778	0.4622419	up
A_33_P3258324	LOC102724279	-0.008248	0.588387	0.3194361	0.5966349	0.3276839	0.4621594	up
A_24_P366526	SYNGR2	8.645608	8.955406	9.259981	0.3097982	0.6143732	0.4620857	up
A_33_P3256848	ADAM12	-2.578753	-1.879423	-2.353975	0.6993299	0.2247782	0.462054	up
A_22_P00000283	MIR940	-3.357869	-2.773118	-3.018529	0.5847511	0.3393402	0.4620457	up
A_23_P108294	PPAP2C	3.3588076	3.8883176	3.7533865	0.52951	0.3945789	0.4620445	up
A_23_P394166	CNOT7	2.4044447	2.8596673	2.87317	0.4552226	0.4687252	0.4619739	up
A_22_P00002900	CTBP1-AS2	1.4167428	1.9637961	1.7935266	0.5470533	0.3767839	0.4619186	up
A_23_P15285	METTL22	2.5612202	3.017568	3.028679	0.4563479	0.4674587	0.4619033	up
A_33_P3299140	GTF3C5	-1.715726	-1.225092	-1.282602	0.4906335	0.4331241	0.4618788	up
A_33_P3222769	PRR25	3.2463675	3.8569942	3.5594082	0.6106267	0.3130407	0.4618337	up
A_33_P3229027	RELL2	0.657167	1.0615745	1.1764174	0.4044075	0.5192504	0.461829	up
A_33_P3365357	HYAL2	5.9749784	6.40659	6.4669714	0.4316115	0.491993	0.4618022	up
A_33_P3273369	SH3BP4	-2.896278	-2.715267	-2.153792	0.181011	0.7424862	0.4617486	up
A_33_P3759611	PDE4C	-2.765933	-2.219301	-2.389231	0.5466316	0.3767018	0.4616667	up
A_24_P347880	ALOXE3	-3.238251	-2.562326	-2.99087	0.6759248	0.2473817	0.4616532	up

A_24_P282578	SSR2	3.8930483	4.4929013	4.216399	0.599853	0.3233509	0.461602	up
A_23_P104413	DUX4	7.4358854	8.007616	7.7871647	0.5717306	0.3512793	0.4615049	up
A_21_P0007044	LINC00707	-2.903817	-2.66961	-2.215139	0.2342062	0.6886778	0.461442	up
A_21_P0012445	XLOC_l2_010239	-1.833265	-1.391794	-1.351867	0.4414706	0.4813976	0.4614341	up
A_23_P83192	PHPT1	4.5513306	5.128346	4.897133	0.5770154	0.3458023	0.4614089	up
A_24_P289471	RNASET2	2.9263706	3.3907285	3.3847418	0.4643579	0.4583712	0.4613645	up
A_21_P0004883	lnc-UBR2-1	-0.453562	0.0589323	-0.043386	0.5124941	0.4101758	0.461335	up
A_33_P3407895	RINL	-2.018143	-1.635338	-1.478346	0.3828044	0.5397964	0.4613004	up
A_24_P27977	TRPM2	1.7600298	2.1541686	2.2884073	0.3941388	0.5283775	0.4612582	up
A_23_P16214	FBXW9	1.190938	1.5857167	1.7184525	0.3947787	0.5275145	0.4611466	up
A_32_P20691	CLK3	3.190198	3.8187919	3.483694	0.6285939	0.2934961	0.461045	up
A_24_P20777	RPL37	8.844952	9.323776	9.287967	0.4788246	0.4430151	0.4609199	up
A_33_P3253960	DHRS11	-1.529949	-1.292383	-0.845856	0.237566	0.684093	0.4608295	up
A_23_P30813	HIST1H4K	4.2354517	4.622432	4.769869	0.3869805	0.5344172	0.4606988	up
A_23_P201711	S100A6	8.74372	9.300035	9.1083355	0.5563154	0.3646154	0.4604654	up
A_23_P12989	PRDX5	6.7618694	7.4752965	6.9691696	0.7134271	0.2073002	0.4603636	up
A_24_P23951	BCRP2	1.105248	1.4887714	1.6423721	0.3835235	0.5371242	0.4603238	up
A_23_P44363	CASKIN2	0.0136924	0.52317	0.4245787	0.5094776	0.4108863	0.460182	up
A_33_P3398862	RHOB	2.1947346	2.649076	2.6607132	0.4543414	0.4659786	0.46016	up
A_21_P0000234	SNORD38B	1.9940004	2.501493	2.4067507	0.5074925	0.4127502	0.4601214	up
A_23_P4782	CACNG7	-3.130195	-2.929412	-2.410835	0.200783	0.7193599	0.4600715	up
A_22_P00025578	lnc-UNC93B1-1	1.6450005	2.0912385	2.1188097	0.446238	0.4738092	0.4600236	up
A_23_P119377	CYTH2	1.2151461	1.8043575	1.5458713	0.5892115	0.3307252	0.4599683	up
A_23_P59099	OR11A1	-1.246639	-0.887595	-0.685759	0.3590446	0.5608807	0.4599626	up
A_33_P3257817	LOC102723701	5.9480896	6.49416	6.3217278	0.5460706	0.3736382	0.4598544	up
A_23_P7221	RPL34	8.950955	9.418247	9.403183	0.4672918	0.4522276	0.4597597	up
A_21_P0013504	LOC100996662	-2.520859	-1.941611	-2.180675	0.5792484	0.3401845	0.4597164	up
A_22_P00006553	LOC101929865	-2.445055	-1.989877	-1.980997	0.4551776	0.4640582	0.4596179	up
A_22_P00024545	lnc-LUZP1-1	-1.970794	-1.647751	-1.374752	0.3230429	0.5960422	0.4595425	up
A_33_P3370600	SMIM6	-1.577439	-1.236281	-0.999534	0.3411579	0.5779057	0.4595318	up
A_32_P65533	ZC3H12D	-3.583515	-3.136614	-3.111386	0.4469016	0.4721289	0.4595152	up
A_33_P3243439	GPR162	-2.541784	-2.027624	-2.137037	0.5141606	0.4047475	0.4594541	up
A_21_P0007927	USP12-AS2	-1.469267	-0.788225	-1.231573	0.6810422	0.2376947	0.4593685	up
A_21_P0013085	XLOC_l2_013031	6.120861	6.7031884	6.4570217	0.5823274	0.3361607	0.459244	up
A_33_P3245248	TERC	2.9741936	3.6014953	3.2653456	0.6273017	0.291152	0.4592269	up
A_22_P00001850	PRICKLE2-AS2	-2.109761	-1.747778	-1.55331	0.3619828	0.5564508	0.4592168	up
A_23_P66117	ITFG3	4.2592773	4.692166	4.7447596	0.4328885	0.4854822	0.4591854	up
A_23_P59179	RXRB	0.433506	0.9479642	0.8371959	0.5144582	0.4036899	0.459074	up
A_21_P0010721	LINC00869	-1.118728	-0.634571	-0.684917	0.4841571	0.4338112	0.4589842	up
A_33_P3287997	C9orf106	-3.192936	-2.673577	-2.794502	0.5193596	0.3984344	0.458897	up
A_24_P88079	MUC6	0.132297	0.6188555	0.5634232	0.4865584	0.4311261	0.4588423	up
A_33_P3344332	RPL39	7.0567656	7.5896926	7.4415007	0.532927	0.3847351	0.4588311	up
A_33_P3239879	NAA38	6.83821	7.3520627	7.2419634	0.5138526	0.4037533	0.4588029	up
A_22_P00002022	LOC101930010	-2.785664	-2.399227	-2.254689	0.3864369	0.5309758	0.4587064	up
A_33_P3316587	SLC22A18AS	0.8610249	1.1582384	1.4812102	0.2972136	0.6201854	0.4586995	up
A_33_P3298492	PCBP2	6.4155912	7.0652423	6.6833	0.6496511	0.2677088	0.4586799	up
A_23_P208835	MAP2K2	2.4002151	2.9765887	2.7411604	0.5763736	0.3409452	0.4586594	up
A_22_P00018556	lnc-AAAS-1	-1.104318	-0.56493	-0.726899	0.5393877	0.377419	0.4584034	up
A_33_P3289426	ZNF775	0.4599323	0.7224078	1.1141071	0.2624755	0.6541748	0.4583252	up
A_23_P373687	PUS10	-2.712641	-2.136433	-2.372506	0.5762079	0.3401346	0.4581713	up
A_22_P00002811	lnc-C2orf42-3	-3.263158	-2.617341	-2.992816	0.6458178	0.2703426	0.4580802	up
A_22_P00005559	lnc-EEF1B2-3	-2.963626	-2.287139	-2.723972	0.676487	0.2396543	0.4580706	up
A_33_P3254801	OGFR	3.1392403	3.5274272	3.6671486	0.3881869	0.5279083	0.4580476	up
A_23_P318646	RPS10	8.079612	8.642481	8.432539	0.5628691	0.3529272	0.4578982	up
A_33_P3419165	TFPT	0.1040502	0.5745177	0.5493126	0.4704676	0.4452624	0.457865	up
A_33_P3226167	FAM129B	7.6352262	8.109539	8.076338	0.4743128	0.4411116	0.4577122	up

A_33_P3372580	COG1	3.8662338	4.2749553	4.372916	0.4087215	0.5066824	0.4577019	up
A_33_P3263824	MRPL43	3.8304949	4.311486	4.264904	0.4809909	0.4344091	0.4577	up
A_33_P3419806	RPS29	9.585872	10.114796	9.972267	0.528924	0.3863955	0.4576597	up
A_24_P125871	RIPK4	3.512558	4.121079	3.8189707	0.608521	0.3064127	0.4574669	up
A_23_P113393	APLN	-1.293341	-0.695532	-0.976402	0.5978088	0.3169394	0.4573741	up
A_23_P137948	NENF	5.1368866	5.615165	5.5730667	0.4782786	0.4361801	0.4572294	up
A_24_P357169	EPPK1	1.8338995	2.5306344	2.051608	0.6967349	0.2177086	0.4572217	up
A_21_P0010744	RPS18	10.05708	10.577589	10.450995	0.5205088	0.3939142	0.4572115	up
A_33_P3344951	JMJD7	2.5175114	2.8131604	3.1362095	0.2956491	0.6186981	0.4571736	up
A_22_P00008365	JAG2	-3.058983	-2.668237	-2.535512	0.3907466	0.5234711	0.4571089	up
A_23_P311740	CUL9	2.8514614	3.3922868	3.2247581	0.5408254	0.3732967	0.4570611	up
A_23_P14928	COQ9	3.0129738	3.4426332	3.497428	0.4296594	0.4844542	0.4570568	up
A_32_P14894	RPS10	8.232231	8.779251	8.599323	0.54702	0.3670921	0.457056	up
A_23_P69188	DPH3	3.797699	4.3181148	4.1909065	0.5204158	0.3932076	0.4568117	up
A_22_P00024977	lnc-CETP-1	-0.912602	-0.140347	-0.77134	0.7722549	0.1412611	0.456758	up
A_23_P54692	LOC101927910	-0.326074	0.2093525	0.0518241	0.5354261	0.3778977	0.4566619	up
A_23_P50357	ARHGEF18	5.045577	5.590154	5.4142084	0.5445771	0.3686314	0.4566042	up
A_23_P258978	GOLGA1	-0.345322	0.1634612	0.0590844	0.5087833	0.4044066	0.4565949	up
A_24_P925062	MXRA7	-0.942219	-0.329451	-0.642111	0.6127687	0.300108	0.4564383	up
A_33_P3259058	PSMG4	3.657979	3.899053	4.329774	0.2410741	0.6717949	0.4564345	up
A_33_P3251901	APBB2	-0.474164	0.1132522	-0.148803	0.5874162	0.3253608	0.4563885	up
A_19_P00800549	PMF1	4.9485455	5.415031	5.3944254	0.4664855	0.4458799	0.4561827	up
A_24_P1054	TONSL	-0.164279	0.1257157	0.4580636	0.2899952	0.6223431	0.4561691	up
A_22_P00012254	LOC102724362	3.5297928	3.9924111	3.9794054	0.4626184	0.4496126	0.4561155	up
A_23_P162486	PTPN6	0.1295433	0.6824899	0.4888029	0.5529466	0.3592596	0.4561031	up
A_22_P00022847	lnc-RNF186-1	1.7355986	2.2618556	2.1215477	0.526257	0.3859491	0.4561031	up
A_23_P253375	CUX1	3.3899097	3.9363408	3.7556477	0.5464311	0.3657379	0.4560845	up
A_23_P100499	ROGDI	0.1552787	0.5393496	0.6831994	0.3840709	0.5279207	0.4559958	up
A_23_P382188	STAP2	3.3716755	3.8678956	3.7872896	0.4962201	0.4156141	0.4559171	up
A_24_P339514	CYP2B6	-1.955054	-1.540997	-1.457341	0.4140573	0.4977136	0.4558854	up
A_33_P3372666	PDGFA	0.1641388	0.6475434	0.5921378	0.4834046	0.427999	0.4557018	up
A_23_P375524	LCE1D	8.5966425	9.106625	8.997419	0.5099821	0.4007769	0.4553795	up
A_33_P3392177	CLIC5	4.968807	5.565553	5.282447	0.596746	0.3136396	0.4551928	up
A_23_P258418	TNIP2	0.7329392	1.1644435	1.2117748	0.4315043	0.4788356	0.4551699	up
A_23_P4190	ACSF2	1.1085024	1.9137025	1.2134643	0.8052001	0.1049619	0.455081	up
A_23_P141180	TOM1L2	3.803094	4.288255	4.2280884	0.4851613	0.4249945	0.4550779	up
A_23_P142255	SHD	0.6174369	1.1493988	0.9955082	0.5319619	0.3780713	0.4550166	up
A_22_P00007078	lnc-GLIPR1-3	0.3649359	0.9285445	0.7111912	0.5636087	0.3462553	0.454932	up
A_32_P176911	LINC00239	-0.326307	0.312026	-0.054967	0.6383328	0.2713394	0.4548361	up
A_33_P3306802	LOC100130916	-1.588016	-0.935124	-1.331347	0.6528916	0.256669	0.4547803	up
A_22_P00011843	PHLDA3	1.0468392	1.4850903	1.518127	0.438251	0.4712877	0.4547694	up
A_24_P82466	GAS7	-3.341046	-2.723692	-3.04889	0.6173544	0.2921555	0.454755	up
A_22_P00013523	lnc-RP11-404P21.6.1-2	-2.403207	-1.839832	-2.057162	0.563375	0.3460455	0.4547103	up
A_33_P3292560	SURF1	6.088978	6.6119905	6.4752417	0.5230126	0.3862639	0.4546382	up
A_21_P0007550	lnc-TMEM106C-1	-3.304941	-2.933356	-2.767266	0.3715854	0.5376751	0.4546303	up
A_23_P139929	ERP29	4.6698265	5.3471045	4.90162	0.677278	0.2317934	0.4545357	up
A_23_P319640	LBX2-AS1	0.3071046	0.8542509	0.668951	0.5471463	0.3618465	0.4544964	up
A_33_P3351092	KRTAP20-2	-0.836154	-0.355984	-0.407721	0.4801698	0.4284334	0.4543016	up
A_33_P3213259	CENPBD1P1	0.9291554	1.5719757	1.1949205	0.6428204	0.2657652	0.4542928	up
A_33_P3250068	SNX17	4.7122946	5.450993	4.88212	0.7386985	0.1698256	0.454262	up
A_24_P368943	EVX1	2.98814	3.481194	3.4036074	0.4930539	0.4154673	0.4542606	up
A_23_P8400	CDK5	2.39528	2.8078609	2.8910398	0.412581	0.49576	0.4541705	up
A_33_P3280521	MFAP3L	-3.185332	-2.518165	-2.944287	0.667167	0.2410455	0.4541062	up
A_23_P114466	TBL1Y	1.1271391	1.9252782	1.2371802	0.7981391	0.1100411	0.4540901	up
A_23_P86330	IER5	6.1299047	6.641559	6.526333	0.5116544	0.3964281	0.4540413	up

A_23_P120281	EDAR	1.0156794	1.6731644	1.2659364	0.657485	0.250257	0.453871	up
A_23_P386168	INAFM1	2.0635424	2.6937585	2.341035	0.6302161	0.2774925	0.4538543	up
A_23_P54963	MRPL38	2.9442062	3.4550862	3.3409643	0.51088	0.3967581	0.453819	up
A_32_P5251	RARA	3.805358	4.299869	4.2183037	0.4945111	0.4129458	0.4537284	up
A_24_P569294	MRPS12	5.259612	5.6525035	5.7740707	0.3928914	0.5144587	0.453675	up
A_22_P00015051	SNHG15	0.7328124	1.1683197	1.2045784	0.4355073	0.471766	0.4536367	up
A_23_P76109	RILPL2	3.9139595	4.3340335	4.4011507	0.420074	0.4871912	0.4536326	up
A_22_P00005994	lnc-FABP3-1	-1.93489	-1.25275	-1.709842	0.6821399	0.2250481	0.453594	up
A_23_P4144	COASY	4.2805967	4.664412	4.8039064	0.3838153	0.5233097	0.4535625	up
A_23_P136405	PDCD1	-2.082181	-1.79999	-1.457291	0.2821908	0.6248899	0.4535403	up
A_33_P3798739	LOC286382	-0.299108	0.2585092	0.0503297	0.5576172	0.3494377	0.4535275	up
A_33_P3226070	lnc-CRIPAK-1	-1.729856	-1.085737	-1.466972	0.6441183	0.2628837	0.453501	up
A_33_P3335137	ATG4B	2.3397923	2.8694549	2.7170925	0.5296626	0.3773003	0.4534814	up
A_33_P3264116	NHLRC1	-3.221792	-2.610129	-2.926924	0.6116633	0.294868	0.4532657	up
A_23_P432598	CHMP4C	3.214244	3.745027	3.5898228	0.5307832	0.3755789	0.453181	up
A_21_P0008499	lnc-CDCA4-1	0.2103133	0.7350416	0.5919261	0.5247283	0.3816128	0.4531705	up
A_23_P78871	KCTD15	0.5513935	1.0210705	0.9874477	0.469677	0.4360542	0.4528656	up
A_33_P3330039	PLEKHO1	1.7546186	1.8967938	2.5181465	0.1421752	0.7635279	0.4528515	up
A_23_P163173	LTB4R2	-0.807039	-0.265334	-0.443134	0.5417047	0.363905	0.4528048	up
A_33_P3318564	lnc-LIN28B-1	-1.703692	-1.221345	-1.280881	0.4823475	0.4228115	0.4525795	up
A_23_P406785	C9orf50	-3.038775	-2.566709	-2.605682	0.4720659	0.4330931	0.4525795	up
A_33_P3309662	GPX4	4.3439054	4.9531074	4.639847	0.6092019	0.2959414	0.4525716	up
A_33_P3375790	RFPL4AL1	-3.363006	-2.732862	-3.088108	0.6301439	0.2748973	0.4525206	up
A_33_P3362933	GYPA	2.4185648	2.7674165	2.9747257	0.3488517	0.5561609	0.4525063	up
A_23_P111141	BAG6	3.498417	4.0876765	3.814165	0.5892596	0.3157482	0.4525039	up
A_24_P119545	ITPKB	-2.635222	-2.155436	-2.210015	0.4797862	0.4252071	0.4524967	up
A_33_P3290174	DEGS2	0.9758391	1.3991036	1.457375	0.4232645	0.4815359	0.4524002	up
A_24_P386323	RABEPK	4.825981	5.365969	5.190749	0.539988	0.364768	0.452378	up
A_33_P3215557	ADAMTS7	0.1326046	0.6610985	0.5085578	0.5284939	0.3759532	0.4522236	up
A_21_P0006207	lnc-RAD23B-8	-2.899403	-2.653924	-2.240558	0.2454794	0.6588452	0.4521623	up
A_22_P00000562	lnc-AC127496.3-6	-1.057076	-0.396689	-0.813169	0.6603866	0.2439065	0.4521465	up
A_23_P412321	CCR5	-2.495266	-2.141435	-1.944839	0.3538315	0.5504274	0.4521295	up
A_23_P113623	MRPL45	5.8509073	6.51666	6.0892916	0.6657529	0.2383843	0.4520686	up
A_23_P99967	BBS4	1.197989	1.8230305	1.476963	0.6250415	0.2789741	0.4520078	up
A_22_P00007246	lnc-GPR126-1	-3.036411	-2.293217	-2.875869	0.7431946	0.1605427	0.4518687	up
A_23_P13344	EEF1G	8.934912	9.533251	9.240167	0.5983391	0.3052549	0.451797	up
A_23_P212329	PTPN23	1.67138	2.1870255	2.0592623	0.5156455	0.3878822	0.4517639	up
A_22_P00015554	lnc-STOM-3	1.5245581	1.9852662	1.96733	0.4607081	0.4427719	0.45174	up
A_33_P3280681	LENG8	-1.010076	-0.488508	-0.628275	0.5215678	0.3818011	0.4516845	up
A_22_P00008751	ID2-AS1	-2.197158	-1.648471	-1.842553	0.5486865	0.3546052	0.4516459	up
A_23_P77455	FAM96B	5.09933	5.5241923	5.577759	0.4248624	0.4784288	0.4516456	up
A_23_P119617	SUGP1	2.4019127	2.9510102	2.756092	0.5490975	0.3541794	0.4516385	up
A_23_P1492	AVPI1	4.485717	5.0433774	4.830903	0.5576606	0.3451862	0.4514234	up
A_22_P00010880	lnc-NPB-3	-0.94101	-0.381521	-0.598115	0.5594883	0.3428946	0.4511914	up
A_33_P3293593	TTC9B	-1.040432	-0.806835	-0.371857	0.2335973	0.6685753	0.4510863	up
A_33_P3287745	KAT8	4.196249	4.746424	4.5482407	0.5501752	0.3519917	0.4510834	up
A_33_P3311717	TGIF1	-0.304522	0.1879931	0.1050534	0.4925146	0.409575	0.4510448	up
A_23_P146479	SURF2	4.1844873	4.718695	4.552354	0.5342078	0.3678665	0.4510372	up
A_33_P3248749	RSPH9	2.3580809	2.8951716	2.723055	0.5370908	0.364974	0.4510324	up
A_33_P3379199	UQCR10	5.430278	5.833491	5.9290934	0.403213	0.4988155	0.4510143	up
A_23_P77731	CRYM	0.0894089	1.1230211	-0.042511	1.0336123	-0.131919	0.4508465	up
A_33_P3394972	OSBPL5	-2.135141	-1.755898	-1.612804	0.3792434	0.5223374	0.4507904	up
A_23_P259506	CYSTM1	5.691819	6.0290656	6.2559767	0.3372464	0.5641575	0.450702	up
A_33_P3216570	MUC5AC	-2.027411	-1.70735	-1.446828	0.3200607	0.5805821	0.4503214	up
A_32_P56604	SPERT	-1.483059	-1.352092	-0.713545	0.1309671	0.7695141	0.4502406	up
A_23_P309973	PPP1R36	-2.589464	-2.021966	-2.256743	0.5674975	0.3327205	0.450109	up

A_23_P152963	C17orf59	-0.344157	0.0439487	0.1678882	0.3881054	0.5120449	0.4500752	up
A_23_P50241	CLPTM1	3.1857567	3.6991897	3.572363	0.513433	0.3866062	0.4500196	up
A_33_P3346791	SCAMP3	6.6788874	7.032849	7.2249565	0.3539615	0.5460692	0.4500153	up
A_23_P16078	PAFAH1B3	4.390437	4.8318686	4.848974	0.4414315	0.4585371	0.4499843	up
A_22_P00011983	MIR503HG	-1.408855	-0.915223	-1.002646	0.4936314	0.406209	0.4499202	up
A_21_P0000478	SNORA11C	0.9576812	1.453649	1.3615217	0.4959679	0.4038405	0.4499042	up
A_33_P3319281	ANO7	-1.184145	-0.703261	-0.765337	0.4808836	0.418808	0.4498458	up
A_22_P00002956	lnc-C5orf52-1	-3.546863	-2.740697	-3.4534	0.8061659	0.0934634	0.4498147	up
A_33_P3262515	RASA3	2.3434	2.8053546	2.7810392	0.4619546	0.4376392	0.4497969	up
A_23_P82299	PPP1R35	4.8545256	5.361465	5.247142	0.5069394	0.3926163	0.4497778	up
A_23_P108932	RPL23AP32	-3.27888	-2.997876	-2.660368	0.281004	0.6185124	0.4497582	up
A_23_P107735	CD79A	3.2228842	3.8003678	3.5448942	0.5774837	0.32201	0.4497468	up
A_24_P413437	NONO	3.823866	4.390548	4.1566734	0.5666823	0.3328075	0.4497449	up
A_33_P3399208	HLA-B	3.9816456	4.3574643	4.505313	0.3758187	0.5236673	0.449743	up
A_23_P107454	KRTAP3-1	-1.192083	-1.04789	-0.436856	0.1441932	0.7552271	0.4497101	up
A_33_P3260777	ARF5	6.329999	6.7324157	6.8269014	0.4024167	0.4969025	0.4496596	up
A_21_P0014079	C1orf61	-1.673418	-1.026657	-1.421115	0.6467605	0.2523022	0.4495313	up
A_33_P6812640	LOC101929241	-2.937773	-2.502638	-2.473939	0.4351347	0.4638336	0.4494841	up
A_33_P3214943	SPOCK2	0.9207058	1.4959335	1.2443643	0.5752277	0.3236585	0.4494431	up
A_23_P146885	UTS2R	5.608178	6.198937	5.9162893	0.5907588	0.3081112	0.449435	up
A_32_P184488	PHLDB3	2.980503	3.6125436	3.2472525	0.6320405	0.2667494	0.4493949	up
A_23_P164814	C19orf57	1.1802716	1.7460876	1.5127926	0.5658159	0.332521	0.4491684	up
A_33_P3420496	SNORA53	-1.386838	-1.017852	-0.857506	0.3689861	0.5293326	0.4491594	up
A_22_P00011073	lnc-NUB1-1	-1.557923	-0.960934	-1.256613	0.5969892	0.3013101	0.4491496	up
A_23_P116694	RPS26	10.13717	10.643272	10.52935	0.5061026	0.3921804	0.4491415	up
A_23_P130194	PYCR1	6.540738	7.1237082	6.8557835	0.5829701	0.3150454	0.4490078	up
A_23_P46627	ADIPOR1	3.372219	3.9324985	3.709775	0.5602794	0.3375559	0.4489176	up
A_24_P353300	LIMK2	-0.357409	-0.008574	0.1914873	0.348835	0.5488968	0.4488659	up
A_22_P00004700	LOC102724301	-3.029257	-2.482309	-2.678675	0.5469477	0.3505821	0.4487649	up
A_33_P3824237	LINC00857	1.5831089	2.1288857	1.9347696	0.5457768	0.3516607	0.4487188	up
A_24_P134074	RPS19	10.093268	10.648464	10.435316	0.5551958	0.3420477	0.4486218	up
A_23_P70748	REPS1	4.480423	4.996344	4.861659	0.5159211	0.3812361	0.4485786	up
A_21_P0002226	LOC339807	-2.391043	-1.69864	-2.18629	0.6924024	0.2047532	0.4485778	up
A_22_P00003281	lnc-CALML6-1	-2.049234	-1.860562	-1.340794	0.1886721	0.7084403	0.4485562	up
A_33_P3235716	SPSB3	5.3145504	5.837707	5.6879873	0.5231566	0.3734369	0.4482968	up
A_33_P3368049	C1orf233	1.8237247	2.1151958	2.4282265	0.291471	0.6045017	0.4479864	up
A_23_P118122	RGS11	-0.632866	-0.03711	-0.332657	0.5957561	0.3002091	0.4479826	up
A_21_P0010005	lnc-TASP1-4	-2.286439	-1.505884	-2.1715	0.7805557	0.1149392	0.4477475	up
A_23_P259172	SSR4	6.6873016	7.1952777	7.0747547	0.5079761	0.3874531	0.4477146	up
A_33_P3248654	SARS	6.058525	7.0374684	5.9746923	0.9789434	-0.083833	0.4475553	up
A_23_P61823	RAB24	2.120575	2.8712077	2.2650232	0.7506328	0.1444483	0.4475405	up
A_19_P00319646	LINC00673	3.6492414	4.270039	3.9231634	0.6207976	0.273922	0.4473598	up
A_33_P3346067	LOC388813	-3.322567	-2.900338	-2.850182	0.4222291	0.4723849	0.447307	up
A_23_P46238	CELA2A	-3.374097	-3.097492	-2.756146	0.2766051	0.6179514	0.4472783	up
A_24_P359191	SLC6A6	0.5891304	1.0251169	1.0474405	0.4359865	0.4583101	0.4471483	up
A_33_P3278033	TUSC1	1.2540307	1.7260828	1.6762447	0.4720521	0.422214	0.4471331	up
A_23_P258381	SPSB4	3.112836	3.6633506	3.456461	0.5505147	0.3436251	0.4470699	up
A_21_P0006933	lnc-ZRANB1-2	-1.045402	-0.549541	-0.647538	0.4958615	0.3978639	0.4468627	up
A_33_P7314857	LOC100506085	-1.352336	-0.823729	-0.987314	0.5286074	0.3650222	0.4468148	up
A_33_P3216994	HERC4	-0.096706	0.5526586	0.1472979	0.6493645	0.2440038	0.4466841	up
A_33_P3343981	AATK	1.8928919	2.410231	2.268591	0.5173392	0.375699	0.4465191	up
A_33_P3278774	ZNF44	-0.464147	0.0633688	-0.098694	0.5275159	0.3654528	0.4464843	up
A_22_P00011314	lnc-OSCAR-1	-1.64756	-1.131393	-1.270806	0.5161662	0.3767533	0.4464598	up
A_22_P00017184	DARS-AS1	-1.427451	-0.947054	-1.015123	0.4803968	0.4123278	0.4463623	up
A_22_P00002609	lnc-C1QTNF8-2	-2.725177	-2.117044	-2.440629	0.6081338	0.2845483	0.446341	up
A_23_P119627	NDUFA13	6.260668	6.791283	6.622651	0.5306153	0.3619833	0.4462993	up

A_32_P210572	C1orf53	2.228796	2.635735	2.7139082	0.406939	0.4851122	0.4460256	up
A_33_P3232557	DLGAP3	-3.386658	-2.900787	-2.980812	0.4858708	0.4058454	0.4458581	up
A_24_P233078	PYY2	3.3556156	3.834763	3.7679958	0.4791474	0.4123802	0.4457638	up
A_24_P417596	FLYWCH2	-1.192568	-0.793927	-0.699811	0.3986416	0.4927578	0.4456997	up
A_21_P0008443	lnc-RP11-1085N6.3.1-2	-2.745567	-2.256624	-2.343138	0.4889438	0.4024298	0.4456868	up
A_23_P91430	PXMP4	2.376546	2.8880067	2.7561607	0.5114608	0.3796148	0.4455378	up
A_21_P0006079	LINC01506	-0.810798	-0.345596	-0.38505	0.4652014	0.4257474	0.4454744	up
A_33_P3353242	HSPB1	2.0186663	2.4471107	2.4810648	0.4284444	0.4623985	0.4454215	up
A_21_P0007868	lnc-RBP5-1	-3.133565	-2.224387	-3.152223	0.9091775	-0.018658	0.4452599	up
A_23_P60990	C2orf54	4.615987	5.0040355	5.118451	0.3880487	0.5024643	0.4452565	up
A_33_P3328726	CCDC33	-2.672243	-1.816473	-2.637613	0.8557706	0.0346301	0.4452003	up
A_33_P3376341	LRRC24	0.5104394	1.2941337	0.6170816	0.7836943	0.1066422	0.4451683	up
A_24_P306726	TPT1	8.087187	8.618439	8.446125	0.5312519	0.3589382	0.4450951	up
A_23_P26674	KIAA0430	3.458026	3.9248977	3.8810148	0.4668717	0.4229889	0.4449303	up
A_32_P70724	KDM5B	3.7212667	4.282247	4.049758	0.5609803	0.3284912	0.4447358	up
A_33_P3269218	BAI2	1.1017385	1.3829312	1.7097263	0.2811928	0.6079879	0.4445903	up
A_22_P00000206	lnc-ABT1-2	0.3939242	1.0445457	0.6324568	0.6506214	0.2385325	0.444577	up
A_24_P133584	MFGE8	1.0104647	1.6709623	1.2389007	0.6604977	0.228436	0.4444668	up
A_32_P109922	ZFAT	2.7940588	3.2935538	3.1834965	0.499495	0.3894377	0.4444664	up
A_23_P26223	ASL	2.0617428	2.491115	2.5212727	0.4293723	0.4595299	0.4444511	up
A_23_P135084	RPL7A	9.557624	10.088823	9.91531	0.5311995	0.357686	0.4444428	up
A_33_P3281408	YPEL5	3.9248457	4.5857425	4.1528063	0.6608968	0.2279606	0.4444287	up
A_33_P3214463	MESP2	-1.274028	-0.700234	-0.959217	0.5737939	0.3148108	0.4443023	up
A_23_P34115	IDH3G	2.5767212	3.0061011	3.0359259	0.4293799	0.4592047	0.4442923	up
A_33_P3781394	SCAMP1-AS1	2.6595707	3.0278068	3.179697	0.3682361	0.5201263	0.4441812	up
A_23_P214666	RPS18	9.164001	9.636452	9.579673	0.4724503	0.4156714	0.4440608	up
A_24_P206344	ZNF746	4.1994286	4.7688804	4.5178127	0.5694518	0.3183842	0.443918	up
A_33_P3382849	ACR	-1.744288	-1.298468	-1.302307	0.4458203	0.4419813	0.4439008	up
A_21_P0003270	lnc-DNAJB11-3	-1.022784	-0.630925	-0.527198	0.3918591	0.4955854	0.4437222	up
A_23_P41390	SH3TC1	-1.416197	-0.903823	-1.041224	0.5123739	0.3749728	0.4436734	up
A_23_P42768	SRRM3	-0.920232	-0.262211	-0.690949	0.6580215	0.2292838	0.4436526	up
A_23_P344568	FAM124A	-1.825006	-1.404353	-1.358504	0.4206533	0.4665017	0.4435775	up
A_32_P28939	ALKBH2	4.8573885	5.2249837	5.376872	0.3675952	0.5194836	0.4435394	up
A_21_P0009655	MGC45922	-1.820164	-1.427304	-1.326029	0.3928599	0.4941354	0.4434977	up
A_22_P00007112	lnc-GLUD1-3	-1.634032	-0.924173	-1.456986	0.7098584	0.1770454	0.4434519	up
A_23_P167017	POPDC2	-2.648652	-2.001438	-2.409002	0.6472137	0.2396498	0.4434317	up
A_33_P3309491	PTPRU	6.432518	6.9106364	6.841156	0.4781184	0.408638	0.4433782	up
A_23_P257649	RBP1	6.2472696	6.8532805	6.5277634	0.6060109	0.2804937	0.4432523	up
A_33_P3314594	RAB37	-2.016933	-1.682678	-1.46503	0.3342552	0.5519033	0.4430792	up
A_23_P27515	PLD3	2.3837156	2.9932275	2.6603603	0.6095119	0.2766447	0.4430783	up
A_23_P41267	LOC401127	1.5734434	2.1597342	1.8732738	0.5862908	0.2998304	0.4430606	up
A_23_P340318	C11orf31	7.390833	7.889753	7.7775946	0.49892	0.3867617	0.4428408	up
A_33_P3221960	IL18RAP	-0.234781	0.3822975	0.033545	0.6170788	0.2683263	0.4427025	up
A_22_P00010386	MYL6B	4.805395	5.4149776	5.0810137	0.6095824	0.2756186	0.4426005	up
A_33_P3378360	PRELID1	6.967428	7.401959	7.4180393	0.4345307	0.4506111	0.4425709	up
A_33_P3253804	CEBPD	4.9279623	5.4553657	5.2857	0.5274034	0.3577375	0.4425704	up
A_21_P0009955	lnc-NNAT-1	-1.312981	-0.889156	-0.851881	0.4238253	0.4611006	0.4424629	up
A_23_P148057	ZDHHC9	3.8908243	4.384269	4.2822	0.4934449	0.3913755	0.4424102	up
A_23_P119337	ATF5	0.9944305	1.1145	1.759069	0.1200695	0.7646384	0.442354	up
A_24_P305960	LEMD2	2.3977013	2.950294	2.7297974	0.5525928	0.3320961	0.4423444	up
A_23_P120254	DUSP22	1.963439	2.547833	2.2635412	0.584394	0.3001022	0.4422481	up
A_23_P389588	TCF7L2	-1.352583	-1.008447	-0.812321	0.3441358	0.5402618	0.4421988	up
A_23_P206369	TMEM208	6.5283012	6.9548	6.986021	0.4264989	0.4577198	0.4421094	up
A_24_P43681	DBNL	1.2104468	1.5256405	1.7793903	0.3151937	0.5689435	0.4420686	up
A_33_P3290124	ASB10	-0.263766	0.2500892	0.106472	0.513855	0.3702378	0.4420464	up

A_23_P155360	HDAC11	-0.176794	0.2818914	0.2485805	0.4586854	0.4253745	0.44203	up
A_23_P27075	GABARAP	4.6046352	5.1755548	4.917755	0.5709195	0.3131199	0.4420197	up
A_33_P3282614	C9orf173	0.1773896	0.5662227	0.6725521	0.3888331	0.4951625	0.4419978	up
A_33_P3396891	AVPI1	3.5660849	4.1786294	3.837348	0.6125445	0.2712631	0.4419038	up
A_32_P226078	OAZ3	0.1809564	0.7004261	0.5450249	0.5194697	0.3640685	0.4417691	up
A_23_P149206	B4GALT2	3.0922766	3.5129962	3.5550594	0.4207196	0.4627829	0.4417512	up
A_33_P3233150	ZSWIM4	-0.210113	0.2471032	0.2161474	0.4572158	0.42626	0.4417379	up
A_33_P3384502	LINC01310	-2.733651	-2.204722	-2.379148	0.5289288	0.3545027	0.4417157	up
A_22_P00003471	lnc-CCDC69-1	-0.749954	-0.287498	-0.328981	0.4624558	0.4209728	0.4417143	up
A_21_P0003558	lnc-CRIPAK-1	-2.56863	-2.13621	-2.117808	0.4324207	0.4508219	0.4416213	up
A_24_P316257	NHLRC4	-1.465676	-1.116871	-0.931251	0.3488054	0.5344257	0.4416156	up
A_22_P00023919	lnc-MEGF10-1	0.8152838	1.112256	1.4014187	0.2969723	0.5861349	0.4415536	up
A_22_P00015864	KTN1-AS1	0.8315406	1.2845654	1.2615805	0.4530249	0.4300399	0.4415324	up
A_33_P3280927	SNHG7	3.2223663	3.9120526	3.4155102	0.6896863	0.1931438	0.4414151	up
A_23_P59107	MRPS18B	5.3210936	5.7773423	5.7476482	0.4562488	0.4265547	0.4414017	up
A_33_P3243907	CTSD	4.375971	4.791347	4.8429832	0.4153762	0.4670124	0.4411943	up
A_32_P197621	GEMIN8P4	-1.607508	-0.651655	-1.680973	0.955853	-0.073465	0.4411938	up
A_33_P3409347	C19orf53	-1.268827	-0.910733	-0.744565	0.3580942	0.5242624	0.4411783	up
A_23_P67648	CAPNS1	6.1106415	6.6515546	6.451641	0.5409131	0.3409996	0.4409564	up
A_23_P117274	MIPEP	0.9483881	1.3367572	1.441874	0.3883691	0.4934859	0.4409275	up
A_22_P00003879	lnc-CENPM-1	-3.287045	-3.11494	-2.577346	0.1721046	0.7096987	0.4409017	up
A_33_P3241937	TBC1D16	0.6245489	1.1852894	0.9451971	0.5607405	0.3206482	0.4406944	up
A_22_P00011159	ABO	-0.916023	-0.476504	-0.474387	0.4395189	0.4416366	0.4405777	up
A_24_P89457	CDKN1A	-2.206842	-1.580286	-1.952269	0.6265564	0.2545734	0.4405649	up
A_23_P434301	PTMA	7.024726	7.353685	7.5765724	0.328959	0.5518465	0.4404028	up
A_23_P158024	DCTN3	3.6150198	4.0562634	4.0545635	0.4412437	0.4395437	0.4403937	up
A_33_P3348244	BRI3	5.806752	6.4290547	6.065091	0.6223025	0.2583389	0.4403207	up
A_33_P3229256	NT5C	5.9791737	6.5063014	6.33257	0.5271277	0.3533964	0.4402621	up
A_23_P157072	EIF3B	6.026911	6.4588037	6.4753456	0.4318929	0.4484348	0.4401639	up
A_23_P250516	LOC101928710	-1.875365	-1.629384	-1.241278	0.2459807	0.6340866	0.4400337	up
A_33_P3250383	CNP	-1.412317	-1.175019	-0.769708	0.237298	0.6426091	0.4399536	up
A_33_P3411773	LOC100130285	-0.816525	-0.285385	-0.467914	0.5311408	0.3486114	0.4398761	up
A_19_P00318013	SNHG11	3.3327951	3.8094268	3.7357616	0.4766316	0.4029665	0.4397991	up
A_23_P135271	B4GALT1	2.6589508	3.2412124	2.9561272	0.5822616	0.2971764	0.439719	up
A_33_P3339187	C17orf112	-2.939046	-2.636004	-2.362652	0.3030422	0.5763943	0.4397182	up
A_33_P3215575	ARHGEF10L	2.93005	3.444922	3.2943954	0.5148721	0.3643456	0.4396088	up
A_33_P3313055	NOTCH3	5.6751223	6.359139	5.8702526	0.6840167	0.1951304	0.4395735	up
A_21_P0010660	XLOC_l2_001192	5.076152	5.698837	5.3323774	0.622685	0.2562256	0.4394553	up
A_33_P3343845	CBX7	0.3449907	0.809041	0.7598081	0.4640503	0.4148173	0.4394338	up
A_33_P3344451	C16orf13	6.0057	6.419045	6.4711742	0.4133449	0.4654741	0.4394095	up
A_23_P360209	ND3	7.934249	8.433292	8.313985	0.4990435	0.379736	0.4393897	up
A_23_P146654	BAG1	5.176491	5.764605	5.4670734	0.5881143	0.2905827	0.4393485	up
A_23_P26294	TPSG1	7.954756	8.520903	8.267276	0.5661469	0.31252	0.4393334	up
A_32_P99902	C15orf40	2.3343887	2.8696933	2.6776638	0.5353046	0.3432751	0.4392898	up
A_33_P3279009	HMX1	4.190713	4.7572827	4.5024967	0.5665698	0.3117838	0.4391768	up
A_23_P379034	BAIAP2L2	4.2139482	4.5920224	4.7141733	0.3780742	0.5002251	0.4391496	up
A_22_P00012702	COL4A2-AS1	-2.111664	-1.604677	-1.740423	0.5069866	0.3712411	0.4391139	up
A_23_P131417	HDLBP	4.6456795	5.172686	4.9967413	0.5270066	0.3510618	0.4390342	up
A_33_P3288359	PSMB10	6.6567745	7.0029254	7.188594	0.3461509	0.5318193	0.4389851	up
A_23_P55688	ZNF416	1.72925	2.1630578	2.1732264	0.4338079	0.4439764	0.4388921	up
A_24_P753476	GAS2L1P2	1.246233	1.5978642	1.7717538	0.3516312	0.5255208	0.438576	up
A_33_P3333995	KIFC3	-2.216947	-1.694331	-1.862553	0.5226159	0.354394	0.4385049	up
A_21_P0012657	XLOC_l2_011044	-2.456716	-1.942137	-2.094311	0.5145786	0.3624051	0.4384918	up
A_33_P3304282	RPS15A	8.472621	9.029495	8.792707	0.5568743	0.3200865	0.4384804	up
A_33_P3214466	MESP1	-0.421463	-0.075942	0.1099396	0.345521	0.5314026	0.4384618	up
A_33_P3294608	MVP	7.228735	7.683513	7.6508303	0.4547782	0.4220953	0.4384368	up

A_23_P218412	SPAG7	1.71243	2.2108712	2.0907326	0.4984412	0.3783026	0.4383719	up
A_23_P361773	CCND3	2.6246424	3.0058722	3.1201153	0.3812299	0.4954729	0.4383514	up
A_23_P12128	TSHB	-1.330332	-0.969565	-0.814496	0.3607664	0.5158362	0.4383013	up
A_23_P90172	PPP1R15A	1.5258341	2.4302926	1.4978213	0.9044585	-0.028013	0.4382229	up
A_23_P98282	SPTBN2	1.7838497	2.3815112	2.062605	0.5976615	0.2787552	0.4382084	up
A_21_P0012584	LOC101060498	1.4981265	1.9992557	1.8731241	0.5011292	0.3749976	0.4380634	up
A_33_P3267865	HLA-J	2.05307	2.58948	2.3926458	0.5364099	0.3395758	0.4379928	up
A_23_P26173	TMED3	5.1880255	5.670715	5.581131	0.4826894	0.3931055	0.4378974	up
A_23_P321913	DEF6	0.0959811	0.4084215	0.6593108	0.3124404	0.5633297	0.4378851	up
A_23_P305550	GPR35	1.4786677	1.7295904	2.103385	0.2509227	0.6247172	0.43782	up
A_33_P3417620	ZRSR2	-2.600942	-2.386502	-1.939832	0.2144399	0.6611097	0.4377748	up
A_24_P915806	HNMT	-0.908881	-0.37333	-0.568982	0.5355506	0.3398991	0.4377248	up
A_22_P00012594	SNUPN	2.1362524	2.5797591	2.567813	0.4435067	0.4315605	0.4375336	up
A_23_P24192	RRP12	2.4988232	3.0885205	2.7840328	0.5896974	0.2852097	0.4374535	up
A_23_P163148	VIPAS39	2.9014816	3.3733792	3.3042545	0.4718976	0.4027729	0.4373353	up
A_21_P0012110	LOC101929395	-0.528892	-0.424544	0.2412672	0.1043482	0.7701592	0.4372537	up
A_32_P4364	BCAP31	7.6201324	8.080225	8.034382	0.4600925	0.4142494	0.437171	up
A_23_P68899	TXN2	3.86131	4.318276	4.278528	0.4569659	0.4172182	0.4370921	up
A_23_P115356	AKR7A2	3.9732685	4.277326	4.5433207	0.3040576	0.5700522	0.4370549	up
A_23_P1615	FIBP	2.9529314	3.3129735	3.4668732	0.3600421	0.5139418	0.4369919	up
A_23_P259632	TMEM120A	0.9582253	1.3535099	1.436903	0.3952847	0.4786778	0.4369812	up
A_24_P148235	RPS27	9.955932	10.470036	10.31576	0.5141039	0.359828	0.436966	up
A_33_P3294504	FCRL1	-3.003941	-2.677027	-2.45702	0.3269134	0.5469205	0.436917	up
A_23_P92909	SPINK6	-2.258761	-2.183834	-1.459891	0.0749271	0.7988706	0.4368988	up
A_22_P00000854	lnc-AGMAT-3	-2.832242	-2.503885	-2.286813	0.3283567	0.5454292	0.436893	up
A_24_P408321	OSBPL2	2.5752316	2.9267764	3.097252	0.3515449	0.5220203	0.4367826	up
A_21_P0001666	lnc-CDC42-1	1.7130756	2.1626344	2.136898	0.4495587	0.4238224	0.4366906	up
A_23_P205724	NRDE2	-1.629672	-1.053118	-1.332944	0.5765538	0.2967277	0.4366407	up
A_23_P394043	CAMSAP3	1.0103664	1.4402008	1.4535818	0.4298344	0.4432154	0.4365249	up
A_23_P114144	CCDC22	2.4321747	2.8981233	2.8392057	0.4659486	0.4070311	0.4364898	up
A_33_P3357087	MPV17	4.3909426	4.7891803	4.865655	0.3982377	0.4747124	0.436475	up
A_24_P282108	ZZEF1	1.69349	2.169539	2.0903444	0.476049	0.3968544	0.4364517	up
A_23_P25945	ADCK1	-1.357339	-0.985665	-0.856308	0.3716741	0.5010309	0.4363525	up
A_23_P101960	ZFP36L2	3.7286901	4.3540673	3.9759722	0.6253772	0.247282	0.4363296	up
A_21_P0013399	GS1-259H13.2	-2.447906	-2.121337	-1.901892	0.3265681	0.5460138	0.436291	up
A_23_P142671	ZNF513	-0.200139	0.2266793	0.2456164	0.4268184	0.4457555	0.4362869	up
A_23_P210425	MYL9	0.9231691	1.3376713	1.3812308	0.4145021	0.4580617	0.4362819	up
A_23_P100730	SKAP1	1.3833232	1.7575102	1.8814106	0.374187	0.4980874	0.4361372	up
A_33_P3233125	PSD	2.2752619	2.7545152	2.6682625	0.4792533	0.3930006	0.436127	up
A_33_P3232011	RAB17	4.709958	5.1953225	5.0968103	0.4853644	0.3868523	0.4361084	up
A_21_P0000013	TIMM8A	0.7821398	1.1396732	1.2968073	0.3575335	0.5146675	0.4361005	up
A_23_P15123	UBFD1	3.223195	3.7565475	3.561821	0.5333524	0.3386259	0.4359892	up
A_23_P5163	ATP13A1	1.7396469	2.2627187	2.088276	0.5230718	0.348629	0.4358504	up
A_22_P00025529	lnc-KRTAP5-6-3	-2.414814	-1.886817	-2.07137	0.5279965	0.3434439	0.4357202	up
A_23_P83714	ZNF707	2.276208	2.8414598	2.582119	0.5652518	0.3059111	0.4355814	up
A_33_P3217704	FAM214B	0.3930812	0.8857212	0.7714248	0.49264	0.3783436	0.4354918	up
A_19_P00319829	LOC220729	-1.111418	-0.708403	-0.643502	0.4030147	0.4679155	0.4354651	up
A_23_P95823	NSMCE1	3.5024529	3.9039578	3.971815	0.401505	0.4693623	0.4354336	up
A_24_P38702	NKX2-3	0.0536318	0.5341706	0.4438992	0.4805389	0.3902674	0.4354031	up
A_33_P3401301	RPL39	8.003336	8.485302	8.391745	0.481966	0.3884087	0.4351873	up
A_24_P70888	PLXNB2	4.984104	5.4713044	5.3672295	0.4872003	0.3831253	0.4351628	up
A_33_P3300092	NDST1	5.0412216	5.5236325	5.4288893	0.4824109	0.3876677	0.4350393	up
A_23_P119141	KEAP1	4.1327124	4.6874194	4.4480333	0.5547071	0.315321	0.435014	up
A_23_P208788	C19orf33	8.093648	8.691109	8.365976	0.5974608	0.2723284	0.4348946	up
A_24_P211151	EXOSC5	2.3472986	2.9119601	2.6523218	0.5646615	0.3050232	0.4348424	up
A_23_P34915	ATF3	3.7656326	4.8504844	3.5503864	1.0848517	-0.215246	0.4348028	up

A_22_P00013961	lnc-RTL1-2	2.8260355	3.3665137	3.1551428	0.5404782	0.3291073	0.4347927	up
A_24_P327050	TRAPPC2L	4.824396	5.382897	5.1353903	0.5585008	0.3109942	0.4347475	up
A_24_P808522	RPS14	7.1997175	7.709289	7.559596	0.5095716	0.3598785	0.434725	up
A_32_P72940	RPL35	7.9191284	8.356413	8.351067	0.4372845	0.4319382	0.4346113	up
A_33_P3590259	CXCL14	3.1302786	3.5888925	3.540822	0.4586139	0.4105434	0.4345787	up
A_21_P0000585	TRAPPC1	4.5250673	4.85613	5.0629063	0.3310628	0.5378389	0.4344509	up
A_23_P163647	ECI1	6.7513657	7.460274	6.9108915	0.7089086	0.1595259	0.4342172	up
A_33_P3403075	PRR11	0.9784231	1.5415316	1.2836533	0.5631084	0.3052301	0.4341693	up
A_24_P4054	TRIP6	4.164812	4.6325984	4.565299	0.4677863	0.400487	0.4341366	up
A_21_P0013047	LOC100289495	-2.239281	-1.768597	-1.841736	0.4706836	0.3975444	0.434114	up
A_33_P3320272	TMEM79	2.4981241	2.9792056	2.885087	0.4810815	0.3869629	0.4340222	up
A_21_P0011692	XLOC_l2_006718	-2.561813	-2.160228	-2.095504	0.4015851	0.4663088	0.433947	up
A_23_P29248	TST	6.933962	7.301761	7.433673	0.3677993	0.499711	0.4337552	up
A_33_P3351197	GLIDR	-0.913976	-0.536085	-0.424393	0.3778911	0.4895835	0.4337373	up
A_33_P3410895	POR	6.090474	6.588939	6.459464	0.4984651	0.3689899	0.4337275	up
A_23_P74668	C1orf158	-0.290248	0.1553941	0.1315141	0.4456425	0.4217625	0.4337025	up
A_22_P00004712	LOC100506844	-0.615086	0.0335641	-0.39661	0.6486497	0.2184758	0.4335628	up
A_23_P166823	TNNC1	4.2189264	4.8311315	4.4736624	0.612205	0.254736	0.4334705	up
A_19_P00315705	LOC101927354	-1.566618	-1.065781	-1.200635	0.5008373	0.3659835	0.4334104	up
A_23_P306919	CXorf40B	3.422083	3.9194589	3.791482	0.497376	0.3693991	0.4333875	up
A_24_P219920	FEM1A	-0.334347	0.0268474	0.1711607	0.3611946	0.505508	0.4333513	up
A_21_P0000294	SNORA71B	0.4247646	0.9439426	0.77215	0.5191779	0.3473854	0.4332817	up
A_33_P3247858	MPP1	-3.30493	-2.936766	-2.80658	0.3681641	0.4983499	0.433257	up
A_21_P0002185	lnc-EFR3B-3	-0.533367	-0.221222	0.0207963	0.3121452	0.5541635	0.4331543	up
A_33_P3325643	LINC00487	-2.094368	-1.434499	-1.88795	0.6598682	0.2064171	0.4331427	up
A_33_P3250148	SP8	0.4249311	0.8903341	0.8257527	0.4654031	0.4008217	0.4331124	up
A_21_P0013671	XLOC_l2_015295	2.287053	2.9139724	2.5262928	0.6269193	0.2392397	0.4330795	up
A_33_P3273919	RNF212	-3.199732	-2.717344	-2.816021	0.4823878	0.3837109	0.4330493	up
A_23_P218434	ZNF226	1.4460006	1.9710765	1.7869878	0.5250759	0.3409872	0.4330316	up
A_21_P0011106	LRCOL1	-1.012718	-0.580454	-0.579097	0.4322639	0.4336209	0.4329424	up
A_22_P00005505	LOC101928573	-3.467133	-3.259525	-2.809198	0.207608	0.6579347	0.4327713	up
A_33_P3320718	C16orf13	1.7355976	1.9516702	2.3850155	0.2160726	0.6494179	0.4327452	up
A_23_P5392	TP53I3	3.6321373	4.316219	3.813468	0.6840816	0.1813307	0.4327061	up
A_33_P3243214	C17orf59	-1.505153	-1.159193	-0.985969	0.3459597	0.5191841	0.4325719	up
A_33_P3353073	DEF6	-2.067829	-1.768841	-1.501932	0.2989883	0.565897	0.4324427	up
A_21_P0000334	SNORA49	0.0416694	0.4271555	0.5209866	0.3854861	0.4793172	0.4324017	up
A_23_P70733	TAAR2	-0.165052	0.2433591	0.291275	0.408411	0.456327	0.432369	up
A_23_P252825	SCAP	2.4586916	2.8449445	2.9370508	0.3862529	0.4783592	0.4323061	up
A_21_P0010397	lnc-RGL4-4	-2.821058	-2.060398	-2.717147	0.7606607	0.1039114	0.432286	up
A_23_P373708	KRT18P55	9.184764	9.7330265	9.500997	0.5482626	0.3162327	0.4322476	up
A_24_P382467	SLC39A3	0.1258497	0.6637254	0.4524007	0.5378757	0.326551	0.4322133	up
A_23_P98995	CALCOCO1	1.4157686	2.3200536	1.3757787	0.904285	-0.03999	0.4321475	up
A_24_P336551	BGLAP	0.3928661	0.8701148	0.7799106	0.4772487	0.3870444	0.4321466	up
A_33_P3344574	SFTPA2	1.7990398	2.3160691	2.146267	0.5170293	0.3472271	0.4321282	up
A_33_P3235282	LINC01451	-2.276715	-2.05113	-1.638122	0.2255855	0.6385937	0.4320896	up
A_33_P3294277	CYP4F3	1.9829617	2.8017154	2.0282955	0.8187537	0.0453339	0.4320438	up
A_33_P3286302	TNFRSF14	-0.292052	0.1889401	0.0909934	0.4809923	0.3830457	0.432019	up
A_24_P242688	HADHA	3.79838	4.3236594	4.1369934	0.5252795	0.3386135	0.4319465	up
A_23_P63190	NRAS	1.0859871	1.4078441	1.6276097	0.321857	0.5416226	0.4317398	up
A_24_P376787	ZNF496	-0.883514	-0.508474	-0.39531	0.3750396	0.488204	0.4316218	up
A_24_P3783	HIST1H2BM	3.8873558	4.2826495	4.3551664	0.3952937	0.4678106	0.4315522	up
A_21_P0011458	XLOC_l2_005415	0.0595651	0.5359745	0.4462142	0.4764094	0.3866491	0.4315293	up
A_22_P00015191	lnc-SOX13-3	-1.977471	-1.498223	-1.593841	0.4792485	0.3836308	0.4314396	up
A_33_P3424272	NAA60	5.802355	6.15836	6.3091145	0.3560052	0.5067596	0.4313824	up
A_23_P1956	TMEM223	4.1955395	4.5368667	4.716488	0.3413272	0.5209484	0.4311378	up
A_23_P101829	LPAR2	0.4649639	0.9146347	0.8774571	0.4496708	0.4124932	0.431082	up

A_23_P104607	PSMC3	4.5300293	4.9630237	4.9590235	0.4329944	0.4289942	0.4309943	up
A_23_P89155	CDK3	1.7361183	2.2976184	2.0364094	0.5615001	0.3002911	0.4308956	up
A_23_P406424	RHOC	5.278248	5.696099	5.722129	0.417851	0.443881	0.430866	up
A_33_P3381851	KRTAP10-10	2.277192	2.7237716	2.6922827	0.4465795	0.4150906	0.430835	up
A_21_P0010454	LINC00899	-2.345543	-1.917642	-1.911846	0.4279013	0.4336972	0.4307992	up
A_33_P3423600	OXLD1	4.8131437	5.276961	5.210863	0.4638171	0.3977194	0.4307683	up
A_24_P373885	C19orf25	0.5220051	0.9881163	0.9174085	0.4661112	0.3954034	0.4307573	up
A_33_P3238410	SBF1	-0.602054	-0.051089	-0.291894	0.5509648	0.3101597	0.4305623	up
A_23_P250274	LRRC8A	7.2007303	7.32636	7.9361334	0.1256299	0.7354031	0.4305165	up
A_32_P51905	KIAA1191	4.230427	4.6317735	4.6897707	0.4013467	0.4593439	0.4303453	up
A_23_P255153	RBMX2	0.2660232	0.5235262	0.8691049	0.257503	0.6030817	0.4302924	up
A_23_P144627	PCDHB13	0.761425	1.1851988	1.1981297	0.4237738	0.4367046	0.4302392	up
A_23_P78543	AP1M2	3.2257261	3.589892	3.7219238	0.3641658	0.4961977	0.4301817	up
A_32_P135818	RPS3A	7.8385353	8.355963	8.181345	0.5174274	0.3428097	0.4301186	up
A_23_P94301	TSTA3	4.031843	4.466273	4.457567	0.4344297	0.425724	0.4300768	up
A_33_P3238074	MIEF1	1.0529442	1.5025487	1.4633594	0.4496045	0.4104152	0.4300098	up
A_33_P3589819	MIATNB	-0.067263	0.4481788	0.2772141	0.5154419	0.3444772	0.4299595	up
A_33_P3610123	POLR1A	3.75111	4.249615	4.11244	0.4985051	0.36133	0.4299176	up
A_33_P3372368	KRT8	3.0500927	3.5011392	3.4586763	0.4510465	0.4085836	0.4298151	up
A_23_P81158	ADH1C	-2.777619	-2.225485	-2.470332	0.5521333	0.3072865	0.4297099	up
A_33_P3219870	PNPLA2	4.148473	4.6086783	4.547452	0.4602056	0.3989792	0.4295924	up
A_23_P32029	SLC35D2	0.4792895	1.0314059	0.7862926	0.5521164	0.307003	0.4295597	up
A_33_P3275422	LINC00523	1.5767097	1.9895692	2.0225754	0.4128594	0.4458656	0.4293625	up
A_21_P0011457	XLOC_l2_005403	-2.757618	-2.101798	-2.554841	0.6558206	0.2027776	0.4292991	up
A_21_P0007895	VSIG10	-1.092254	-0.696292	-0.62967	0.3959622	0.4625845	0.4292734	up
A_23_P154585	SNX21	2.2638807	2.6928916	2.693264	0.4290109	0.4293833	0.4291971	up
A_22_P00012482	LOC100506368	-0.051523	0.392221	0.3630853	0.4437442	0.4146085	0.4291763	up
A_33_P3257708	APOA1BP	4.438034	5.050447	4.683796	0.6124129	0.2457619	0.4290874	up
A_23_P81993	C6orf1	4.568987	5.0300937	4.9659443	0.4611068	0.3969574	0.4290321	up
A_23_P165360	ASB1	1.3789878	1.9883208	1.6276269	0.609333	0.2486391	0.4289861	up
A_21_P0003155	LINC00635	-2.079549	-1.624795	-1.67682	0.4547544	0.4027286	0.4287415	up
A_33_P3356577	SIRPB1	-0.212935	0.2075825	0.2239084	0.4205179	0.4368439	0.4286809	up
A_24_P156576	GEMIN8	-0.268024	0.1451688	0.1760979	0.4131928	0.4441218	0.4286573	up
A_23_P105251	GLI1	-0.337147	0.1817818	0.0011635	0.518929	0.3383107	0.4286199	up
A_21_P0000588	HOXB-AS3	0.9466543	1.1378517	1.6125164	0.1911974	0.6658621	0.4285298	up
A_24_P100368	DYNLT3	0.5092187	1.048132	0.827086	0.5389133	0.3178673	0.4283903	up
A_33_P3241582	RPS15A	8.91872	9.445982	9.248126	0.5272617	0.3294058	0.4283338	up
A_23_P54728	FAM173A	3.3897	3.7548852	3.8811626	0.3651853	0.4914627	0.428324	up
A_33_P3354137	MAP4	9.066086	9.585466	9.403275	0.5193806	0.3371887	0.4282846	up
A_23_P33072	MAF1	3.0098648	3.5192704	3.3570175	0.5094056	0.3471527	0.4282792	up
A_24_P160466	GPRIN1	1.6290894	1.8791556	2.235447	0.2500663	0.6063576	0.4282119	up
A_24_P408047	PLEKHA4	5.5096483	5.837276	6.038374	0.3276277	0.5287256	0.4281766	up
A_22_P00000794	lnc-ADSS-2	-2.468194	-2.109548	-1.970801	0.3586457	0.4973931	0.4280194	up
A_23_P49674	ARHGEF15	2.0546255	2.5570846	2.408083	0.5024591	0.3534575	0.4279583	up
A_24_P367211	SOCS7	-0.950969	-0.52169	-0.524457	0.4292789	0.4265118	0.4278953	up
A_19_P00810748	LINC00265	-0.849553	-0.456386	-0.387016	0.393167	0.4625363	0.4278517	up
A_21_P0002398	lnc-KIDINS220-6	0.5833931	1.0751948	0.9472213	0.4918017	0.3638282	0.427815	up
A_21_P0000263	SNORA21	5.1180506	5.540152	5.551469	0.4221015	0.4334183	0.4277599	up
A_23_P129513	MRPL28	4.384162	4.7464643	4.877367	0.3623023	0.4932051	0.4277537	up
A_23_P258088	PACSIN1	1.7256899	2.2512155	2.0555754	0.5255256	0.3298855	0.4277055	up
A_33_P3340862	TMEM88B	7.716154	8.213339	8.074147	0.4971848	0.3579931	0.4275889	up
A_24_P132470	ESYT2	3.1903124	3.553616	3.68217	0.3633037	0.4918575	0.4275806	up
A_22_P00023609	lnc-DUSP1-2	-3.029014	-2.142362	-3.060722	0.8866518	-0.031708	0.4274718	up
A_32_P203013	RPS10P7	7.7021246	8.206562	8.05255	0.5044375	0.3504257	0.4274316	up
A_21_P0012564	XLOC_l2_010508	8.436753	8.981737	8.746219	0.5449839	0.3094654	0.4272246	up
A_23_P27627	ASNA1	3.5744896	4.0382085	3.9649677	0.4637189	0.3904781	0.4270985	up

A_23_P206760	HP	-2.794196	-2.207515	-2.526736	0.5866811	0.2674604	0.4270707	up
A_23_P409951	OAZ1	8.804989	9.305176	9.158646	0.5001869	0.3536568	0.4269218	up
A_24_P178175	GGT1	0.6138997	1.1797123	0.901556	0.5658126	0.2876563	0.4267345	up
A_23_P358009	DNAJC16	-0.656116	-0.251754	-0.207088	0.4043622	0.4490285	0.4266954	up
A_24_P296907	THAP8	-0.731366	-0.006901	-0.602561	0.7244654	0.1288052	0.4266353	up
A_22_P00013913	lnc-RPS27L-2	-2.23457	-1.621024	-1.995086	0.6135459	0.2394838	0.4265149	up
A_24_P31275	ATP1B2	-2.502438	-2.123339	-2.028526	0.3790989	0.4739115	0.4265052	up
A_19_P00315764	LOC644277	0.3800836	0.8233008	0.7896371	0.4432173	0.4095535	0.4263854	up
A_23_P360167	DCTN2	4.711708	5.2905145	4.985549	0.5788064	0.2738409	0.4263237	up
A_24_P239177	MUC4	3.1015587	3.4444346	3.6113186	0.342876	0.5097599	0.4263179	up
A_24_P105747	AP1S3	0.2922382	0.5012465	0.9357405	0.2090082	0.6435022	0.4262552	up
A_23_P140630	LMAN1L	-2.072954	-1.648329	-1.645128	0.4246254	0.4278259	0.4262257	up
A_23_P171255	IGBP1	1.5219135	2.1979613	1.6983008	0.6760478	0.1763873	0.4262176	up
A_33_P3389852	BCAS3	-2.321125	-1.686086	-2.103892	0.6350389	0.2172322	0.4261355	up
A_23_P50081	IMPA2	4.9607105	5.45534	5.318218	0.4946294	0.3575077	0.4260685	up
A_33_P3269718	IVD	3.7400656	4.2301135	4.10203	0.4900479	0.3619642	0.4260061	up
A_33_P3281850	CGREF1	-1.589809	-1.175064	-1.152549	0.4147453	0.4372597	0.4260025	up
A_22_P00009464	LOC102467146	-2.902039	-2.2773	-2.674925	0.6247392	0.2271137	0.4259264	up
A_22_P00019468	lnc-MIXL1-2	0.8131337	1.0964575	1.3816309	0.2833238	0.5684972	0.4259105	up
A_21_P0007354	LOC101927204	-1.873	-1.602222	-1.291973	0.2707772	0.5810266	0.4259019	up
A_33_P3308332	PLEKHB1	2.9809685	3.3641524	3.4492483	0.383184	0.4682798	0.4257319	up
A_23_P380326	SLC39A9	1.2642446	1.6631789	1.7167068	0.3989344	0.4524622	0.4256983	up
A_23_P114057	SEMA4C	4.6855135	5.058539	5.1636543	0.3730254	0.4781408	0.4255831	up
A_33_P3239222	EIF3B	5.9286776	6.33122	6.377057	0.4025426	0.4483795	0.4254611	up
A_23_P251259	GTF2H4	-0.214757	0.2549563	0.1662712	0.4697137	0.3810287	0.4253712	up
A_22_P00004541	PRRT3-AS1	5.835884	6.4032245	6.1192055	0.5673404	0.2833214	0.4253309	up
A_23_P159476	SSNA1	3.445901	3.8621144	3.880313	0.4162135	0.434412	0.4253128	up
A_33_P3362088	P2RX4	3.404643	3.8890128	3.770895	0.4843698	0.366252	0.4253109	up
A_33_P3306207	KLRG2	0.9809766	1.4282107	1.3841987	0.4472342	0.4032221	0.4252281	up
A_23_P39386	HCST	0.8454585	1.2377343	1.3036385	0.3922758	0.45818	0.4252279	up
A_23_P432545	CRACR2B	2.9044619	3.472302	3.1869278	0.5678401	0.2824659	0.425153	up
A_23_P167040	PDIA5	2.859023	3.3677077	3.200635	0.5086846	0.3416119	0.4251483	up
A_21_P0008758	lnc-MAN2C1-3	-1.391564	-0.851162	-1.082036	0.5404019	0.3095288	0.4249654	up
A_33_P3268304	LIMS2	-1.108613	-0.63906	-0.728268	0.4695525	0.3803449	0.4249487	up
A_33_P3219010	PPA2	4.8495865	5.320023	5.2288666	0.4704366	0.3792801	0.4248583	up
A_33_P3245674	ZNF329	0.9955435	1.5668397	1.2739234	0.5712962	0.2783799	0.4248381	up
A_33_P3318960	POLR2F	5.859808	6.2470756	6.32216	0.3872676	0.4623518	0.4248097	up
A_21_P0009950	lnc-SSTR4-2	0.7442555	1.2464056	1.09168	0.5021501	0.3474245	0.4247873	up
A_33_P3249748	THEM6	4.216796	4.569184	4.7138033	0.3523879	0.4970074	0.4246976	up
A_21_P0009763	lnc-ANKRD27-2	-3.340506	-2.974016	-2.858057	0.3664899	0.4824488	0.4244694	up
A_23_P256784	MUC2	-1.737358	-0.97856	-1.647219	0.7587972	0.0901384	0.4244678	up
A_23_P7074	NOA1	4.2535467	4.7384553	4.617552	0.4849086	0.3640051	0.4244568	up
A_33_P3366028	TLDC2	0.4118123	0.4160214	1.2563858	0.004209	0.8445735	0.4243913	up
A_23_P108244	COX6B1	7.5786695	7.970142	8.035841	0.3914723	0.4571714	0.4243219	up
A_33_P3290919	BAG1	4.528144	5.146129	4.758795	0.6179853	0.2306509	0.4243181	up
A_23_P141032	COX4I1	6.9705114	7.418775	7.3708706	0.4482637	0.4003592	0.4243114	up
A_33_P3267502	ANKRD16	3.0312433	3.3841596	3.5268936	0.3529162	0.4956503	0.4242833	up
A_22_P00014620	LINC01356	8.494645	8.979334	8.8584175	0.4846888	0.3637724	0.4242306	up
A_33_P3217009	FEZF1-AS1	-2.496757	-2.217536	-1.927532	0.2792203	0.5692248	0.4242226	up
A_23_P341567	SLC9B2	-2.023675	-1.547561	-1.651538	0.4761143	0.3721371	0.4241257	up
A_19_P00319095	SNHG5	5.7881556	6.4700847	5.9544363	0.6819291	0.1662808	0.4241049	up
A_23_P160154	GALE	1.0643425	1.5125175	1.4643459	0.448175	0.4000034	0.4240892	up
A_33_P3415430	HSPA1B	3.910613	4.4330163	4.2362757	0.5224032	0.3256626	0.4240329	up
A_23_P166807	PCBP4	3.5568228	3.8924508	4.0691957	0.335628	0.512373	0.4240005	up
A_23_P203267	TRIM29	5.087044	5.510141	5.511939	0.4230971	0.4248953	0.4239962	up
A_19_P00315936	LINC00877	-0.043673	0.4471817	0.313231	0.4908543	0.3569036	0.4238789	up

A_22_P00019047	lnc-COG1-1	-3.107586	-2.488742	-2.878794	0.6188448	0.2287927	0.4238187	up
A_23_P137046	NYX	0.8481312	1.2684183	1.2754407	0.4202871	0.4273095	0.4237983	up
A_22_P00012477	LOC401052	-1.218206	-0.706851	-0.882054	0.5113549	0.3361516	0.4237533	up
A_24_P272873	RPL13AP3	8.819568	9.332642	9.1537485	0.5130739	0.3341808	0.4236274	up
A_24_P398064	RGP1	2.6859922	3.0393472	3.179864	0.3533549	0.4938717	0.4236133	up
A_33_P3436316	ASXL1	2.718422	3.1435752	3.1404924	0.4251533	0.4220705	0.4236119	up
A_23_P328323	RAVER2	-0.113577	0.1170502	0.5029326	0.2306275	0.6165099	0.4235687	up
A_33_P3251073	TMEM52	4.295068	4.774565	4.662673	0.4794974	0.3676052	0.4235513	up
A_23_P317756	ACSM3	-0.909527	-0.247299	-0.724689	0.6622281	0.1848378	0.423533	up
A_33_P3327642	AIM1L	3.9002695	4.6023555	4.0451794	0.702086	0.1449099	0.4234979	up
A_22_P00001020	lnc-AL391421.1-3	-1.663629	-1.191882	-1.288404	0.4717469	0.3752251	0.423486	up
A_33_P3308434	MORN1	-1.700538	-0.956968	-1.597163	0.7435694	0.103375	0.4234722	up
A_22_P00001641	FAM181A-AS1	-3.128995	-2.834776	-2.57643	0.2942188	0.5525649	0.4233918	up
A_24_P376441	TAF5L	-2.581982	-1.509021	-2.808191	1.0729606	-0.226209	0.4233757	up
A_22_P00006200	STRIP1	-1.561139	-1.027475	-1.248109	0.5336638	0.3130302	0.423347	up
A_33_P3301752	FLJ21369	-2.806089	-2.446975	-2.318608	0.3591139	0.4874814	0.4232976	up
A_33_P6570282	LINC00674	3.3132124	3.7742753	3.6984024	0.4610629	0.38519	0.4231265	up
A_23_P52101	CYB5R1	2.8856192	3.3934722	3.2240133	0.507853	0.3383942	0.4231236	up
A_23_P46982	MMS19	3.294116	3.6422315	3.7922277	0.3481154	0.4981117	0.4231136	up
A_23_P154188	SAP130	3.2902431	3.6611562	3.765356	0.370913	0.4751129	0.423013	up
A_23_P122924	INHBA	-2.342164	-2.267887	-1.570447	0.0742772	0.7717176	0.4229974	up
A_23_P402610	PFAS	-1.44968	-1.171454	-0.881944	0.2782264	0.5677357	0.422981	up
A_22_P00004635	LOC101927571	-2.159855	-1.739535	-1.734236	0.42032	0.4256191	0.4229696	up
A_23_P60718	ALG12	-0.026035	0.5157609	0.2780495	0.5417957	0.3040843	0.42294	up
A_33_P3248759	WDR24	2.907071	3.1527267	3.507165	0.2456555	0.6000938	0.4228747	up
A_33_P3812815	PKD1	4.515559	5.011747	4.864953	0.4961877	0.3493938	0.4227908	up
A_32_P104518	LINC00476	-2.927113	-2.80827	-2.20052	0.1188438	0.7265937	0.4227188	up
A_33_P3332135	PHOSPHO1	2.174386	2.660544	2.5335646	0.4861579	0.3591785	0.4226682	up
A_23_P50508	PLA2G4C	-1.615562	-0.805888	-1.579946	0.8096743	0.0356164	0.4226453	up
A_22_P00008989	LOC100509303	-0.996691	-0.260312	-0.887831	0.7363787	0.1088595	0.4226191	up
A_22_P00002950	lnc-C5orf47-2	0.7554302	1.256526	1.0993285	0.5010958	0.3438983	0.4224971	up
A_23_P307563	KALRN	-2.051286	-1.462389	-1.795306	0.5888977	0.25598	0.4224389	up
A_33_P3395396	ZNF420	-1.083826	-0.633631	-0.689161	0.4501953	0.3946648	0.42243	up
A_23_P157404	AP1S1	2.6269712	2.9925513	3.1061497	0.3655801	0.4791784	0.4223793	up
A_23_P501010	COL17A1	-0.231225	0.4574566	-0.075292	0.6886811	0.1559329	0.422307	up
A_33_P3271930	PYCR1	-0.383109	0.1505532	-0.07223	0.5336618	0.3108788	0.4222703	up
A_23_P107795	ATP5SL	5.5198536	5.930652	5.9533596	0.4107986	0.433506	0.4221523	up
A_33_P3293760	KRTAP10-8	-0.962228	-0.506524	-0.573677	0.4557037	0.3885508	0.4221273	up
A_23_P56356	PLB1	-2.994516	-2.777151	-2.367682	0.217365	0.6268342	0.4220996	up
A_23_P31532	ZC3HAV1L	0.0361285	0.5251727	0.3909011	0.4890442	0.3547726	0.4219084	up
A_21_P0010046	lnc-SULF2-1	-1.709702	-0.98724	-1.588572	0.7224612	0.1211295	0.4217954	up
A_33_P3388618	TNK1	1.0065145	1.4819202	1.3746777	0.4754057	0.3681631	0.4217844	up
A_23_P133807	TAF8	-1.421001	-0.823554	-1.175085	0.5974474	0.2459164	0.4216819	up
A_32_P86905	KGFLP2	-1.12459	-0.541497	-0.864374	0.5830927	0.2602162	0.4216545	up
A_33_P3411632	TMEM121	0.4902248	0.8506336	0.9730959	0.3604088	0.4828711	0.4216399	up
A_33_P3287661	lnc-C16orf42-2	4.313876	4.945211	4.5255766	0.6313348	0.2117004	0.4215176	up
A_23_P108303	NDUFA7	6.4083805	6.772387	6.8873596	0.3640065	0.4789791	0.4214928	up
A_32_P222383	HMGN2	6.5123653	7.0684104	6.7992983	0.5560451	0.286933	0.421489	up
A_33_P3228564	DOK3	0.5944691	1.099946	0.9319062	0.505477	0.3374372	0.4214571	up
A_23_P259314	RPS4Y1	6.203492	6.70818	6.541605	0.5046878	0.3381128	0.4214003	up
A_23_P61371	TMEM173	3.6819115	4.143848	4.062644	0.4619365	0.3807325	0.4213345	up
A_33_P3390107	RNA18S5	6.0262995	6.5376134	6.357644	0.5113139	0.3313446	0.4213293	up
A_21_P0011990	LINC00607	-2.143424	-1.764893	-1.679338	0.378531	0.4640861	0.4213085	up
A_23_P214969	CITED2	2.1976585	2.938624	2.299244	0.7409654	0.1015854	0.4212754	up
A_22_P00010511	lnc-NBPF3-4	2.4461784	2.9033256	2.8315744	0.4571471	0.385396	0.4212716	up
A_23_P82959	FOXH1	-1.594814	-1.095619	-1.251595	0.4991956	0.3432193	0.4212074	up

A_24_P415601	RNH1	3.4592228	3.902635	3.8581219	0.4434123	0.3988991	0.4211557	up
A_21_P0005202	LOC100507642	-2.519538	-2.216564	-1.98026	0.3029738	0.5392778	0.4211258	up
A_33_P3397147	FAM168B	-0.561557	0.0909114	-0.371937	0.6524687	0.1896205	0.4210446	up
A_32_P169179	MSX2P1	3.149746	3.5205235	3.6209497	0.3707776	0.4712038	0.4209907	up
A_33_P3310976	lnc-CDH4-1	1.4768033	2.0884242	1.7070899	0.6116209	0.2302866	0.4209538	up
A_23_P329740	UBC	9.073858	9.592583	9.397036	0.5187244	0.3231773	0.4209509	up
A_23_P24922	LIPT2	0.1111236	0.6041451	0.4597325	0.4930215	0.348609	0.4208152	up
A_21_P0000258	SNORD26	4.5172176	4.980435	4.895277	0.4632173	0.3780594	0.4206383	up
A_24_P179816	SLC27A3	-0.208825	0.0538044	0.3697748	0.2626295	0.5785999	0.4206147	up
A_21_P0004992	lnc-C6orf146-2	2.3933725	2.87114	2.7568283	0.4777675	0.3634558	0.4206116	up
A_22_P00003321	lnc-CARHSP1-1	1.8219671	2.3413377	2.1437874	0.5193706	0.3218203	0.4205954	up
A_23_P120863	GAL3ST1	0.1928821	0.5134692	0.7133327	0.3205872	0.5204506	0.4205189	up
A_23_P215658	TBRG4	4.467002	4.733089	5.0417585	0.2660871	0.5747566	0.4204218	up
A_33_P3225298	XKR9	0.6950431	1.2313609	0.9995642	0.5363178	0.3045211	0.4204194	up
A_23_P312851	PMEL	0.3464522	0.9012537	0.6324334	0.5548015	0.2859812	0.4203913	up
A_33_P3343073	SMARCB1	1.8264685	2.363606	2.1300688	0.5371375	0.3036003	0.4203689	up
A_21_P0001659	lnc-ACTRT2-1	-2.459135	-2.095738	-1.981936	0.3633978	0.4771998	0.4202988	up
A_23_P8561	RHBDD2	3.5891285	3.9467893	4.072036	0.3576608	0.4829073	0.420284	up
A_24_P170983	ESPNL	-3.512794	-2.973956	-3.211116	0.5388386	0.3016779	0.4202583	up
A_23_P91619	MIF	10.023959	10.436541	10.45178	0.4125814	0.4278212	0.4202013	up
A_22_P00023476	lnc-TELO2-2	-1.847971	-1.412266	-1.443281	0.4357057	0.4046907	0.4201982	up
A_33_P3322859	HES6	2.9599056	3.4282088	3.331293	0.4683032	0.3713875	0.4198453	up
A_23_P68211	SPR	4.3178988	4.6240106	4.8510437	0.3061118	0.533145	0.4196284	up
A_23_P4754	CLPP	5.855116	6.1972775	6.35219	0.3421617	0.4970741	0.4196179	up
A_33_P3343090	MAP1S	5.5107203	6.0281234	5.83251	0.5174031	0.3217897	0.4195964	up
A_23_P207766	ARHGDIA	3.8733873	4.188277	4.39727	0.3148894	0.5238829	0.4193862	up
A_23_P161522	TMEM134	3.6766624	4.1280856	4.063833	0.4514232	0.3871708	0.419297	up
A_33_P3410296	DPYSL4	2.670661	3.2689872	2.9108858	0.5983262	0.2402248	0.4192755	up
A_21_P0006242	lnc-PPP1R26-2	-0.626684	-0.049379	-0.365542	0.5773053	0.2611423	0.4192238	up
A_33_P3570228	TNRC18P1	1.6742029	2.2245145	1.9621868	0.5503116	0.2879839	0.4191477	up
A_33_P3399373	TPRA1	4.650217	5.1648	4.9735193	0.5145831	0.3233023	0.4189427	up
A_24_P376309	PPP1R9B	4.2542133	4.7046704	4.6416025	0.4504571	0.3873892	0.4189231	up
A_33_P8991074	LOC101927768	-2.269502	-1.817635	-1.883732	0.4518676	0.3857698	0.4188187	up
A_32_P393316	RAPGEF3	-0.537899	-0.086363	-0.151802	0.4515352	0.3860965	0.4188159	up
A_23_P31654	RPL8	9.617681	10.119614	9.952887	0.5019331	0.335206	0.4185696	up
A_22_P00012505	lnc-PSMA2-2	-2.479213	-1.867132	-2.254304	0.6120808	0.2249091	0.4184949	up
A_21_P0002976	lnc-GPR27-2	0.5748773	1.0983481	0.8882637	0.5234709	0.3133864	0.4184287	up
A_23_P140830	ELMO3	2.1969976	2.457396	2.773346	0.2603984	0.5763483	0.4183734	up
A_23_P25929	EIF2B2	3.528902	3.9796968	3.9148273	0.4507947	0.3859253	0.41836	up
A_23_P146367	C9orf89	5.7859077	6.108523	6.299999	0.3226152	0.5140915	0.4183533	up
A_22_P00014550	lnc-SIRT5-1	-1.266034	-0.80999	-0.885379	0.4560437	0.3806553	0.4183495	up
A_22_P00011643	lnc-PDCD11-2	1.2588291	1.7524714	1.6018548	0.4936423	0.3430257	0.418334	up
A_33_P3289222	LRRC41	2.0756884	2.5283427	2.4596949	0.4526544	0.3840065	0.4183304	up
A_33_P3213463	NDUFS8	6.385088	6.831841	6.7749805	0.446753	0.3898926	0.4183228	up
A_33_P3417936	HDAC11	-0.994893	-0.706004	-0.447218	0.2888894	0.5476751	0.4182823	up
A_23_P93217	SLC22A7	-2.046179	-1.760443	-1.495423	0.2857361	0.550756	0.418246	up
A_33_P3255716	LOC285095	-0.920775	-0.468873	-0.53624	0.4519024	0.3845358	0.4182191	up
A_33_P3329344	FASN	5.5208683	5.7846684	6.093379	0.2638001	0.5725107	0.4181554	up
A_32_P41496	LOC100132831	0.6786065	1.0944748	1.098988	0.4158683	0.4203816	0.4181249	up
A_23_P39131	GLTSCR2	3.8897371	4.3396153	4.275955	0.4498782	0.3862181	0.4180481	up
A_23_P253123	VGLL1	2.5990915	3.2096505	2.8244553	0.610559	0.2253637	0.4179614	up
A_23_P160881	SMPDL3B	1.4326773	1.9675584	1.7337055	0.5348811	0.3010283	0.4179547	up
A_21_P0000337	SNORA55	-2.204419	-1.628079	-1.944871	0.5763392	0.2595477	0.4179435	up
A_23_P145357	BAK1	2.3645344	2.738688	2.8261166	0.3741536	0.4615822	0.4178679	up
A_23_P143006	PRLH	-2.550788	-1.947943	-2.318022	0.602845	0.2327659	0.4178054	up
A_23_P154500	DNMT3A	1.985219	2.5081487	2.297638	0.5229297	0.3124189	0.4176743	up

A_33_P3378212	MED25	4.541149	4.931103	4.986434	0.3899541	0.4452848	0.4176195	up
A_24_P142151	LRRC28	-1.000918	-0.450351	-0.71628	0.5505672	0.2846379	0.4176025	up
A_33_P3345051	GFY	-2.160859	-1.556834	-1.92972	0.6040254	0.2311392	0.4175823	up
A_22_P00002736	lnc-C20orf96-1	9.259625	9.771378	9.58268	0.5117521	0.3230543	0.4174032	up
A_23_P120931	APOBEC3C	1.5920305	1.8937182	2.1248884	0.3016877	0.5328579	0.4172728	up
A_33_P3228510	EPB41L1	-1.545621	-0.970775	-1.28601	0.5748458	0.2596107	0.4172282	up
A_23_P54477	NOP10	7.56349	7.913388	8.047854	0.3498979	0.4843645	0.4171312	up
A_22_P00015112	lnc-SNX17-1	1.5586996	2.0367036	1.9148359	0.478004	0.3561363	0.4170702	up
A_21_P0001300	lnc-BSND-1	-2.561663	-2.071765	-2.217647	0.489898	0.3440166	0.4169573	up
A_32_P129288	RAB1B	3.948986	4.34996	4.381548	0.4009738	0.4325619	0.4167678	up
A_32_P84605	RPS23	9.195369	9.706987	9.517285	0.5116186	0.3219166	0.4167676	up
A_21_P0007524	lnc-GABARAPL1-1	-2.573027	-2.068691	-2.244238	0.5043359	0.3287885	0.4165622	up
A_33_P3278649	ENTPD2	2.8459406	3.261087	3.2639093	0.4151464	0.4179688	0.4165576	up
A_33_P3215529	TJP2	-2.278417	-1.734481	-1.989423	0.5439363	0.2889943	0.4164653	up
A_33_P3376026	LOC100128770	-2.352916	-1.934736	-1.938265	0.4181805	0.4146514	0.4164159	up
A_22_P00010782	lnc-NKX6-3-1	-2.908033	-2.518174	-2.46507	0.3898594	0.4429631	0.4164113	up
A_22_P00021964	LOC101928813	0.620389	1.0801425	0.9934354	0.4597535	0.3730464	0.4164	up
A_23_P22129	HAP1	-1.833525	-1.261644	-1.572902	0.5718813	0.2606235	0.4162524	up
A_23_P393051	KDF1	3.891096	4.2909236	4.323432	0.3998275	0.4323359	0.4160817	up
A_33_P3352767	MC1R	2.1196156	2.6640944	2.4066858	0.5444789	0.2870703	0.4157746	up
A_23_P17382	ZNF335	1.465847	1.9998689	1.7630854	0.5340219	0.2972384	0.4156301	up
A_23_P430658	HEYL	-0.794728	-0.603005	-0.155252	0.1917229	0.6394758	0.4155993	up
A_22_P00015126	lnc-SNX27-1	-1.453016	-0.921833	-1.1531	0.5311837	0.2999163	0.41555	up
A_22_P00017844	ZCCHC2	-2.069782	-1.844161	-1.464326	0.2256217	0.6054564	0.415539	up
A_19_P00322944	SNHG5	4.5249195	5.245689	4.634988	0.7207694	0.1100683	0.4154189	up
A_24_P318593	SCRN2	1.7255497	2.2562137	2.0255938	0.530664	0.3000441	0.415354	up
A_23_P208674	EMC10	6.1841145	6.614076	6.584654	0.4299617	0.4005394	0.4152505	up
A_23_P210348	COX7A2L	5.7284393	6.2836347	6.0035667	0.5551953	0.2751274	0.4151614	up
A_22_P00014972	LOC101928156	-0.235696	0.0740166	0.284781	0.3097129	0.5204773	0.4150951	up
A_22_P00003181	lnc-C9orf85-1	-1.712835	-1.326335	-1.269203	0.3865004	0.4436317	0.415066	up
A_23_P137984	S100A10	8.344084	8.869123	8.649146	0.5250397	0.3050623	0.415051	up
A_24_P312519	PBLD	-1.137522	-0.533228	-0.911881	0.6042943	0.2256417	0.414968	up
A_21_P0000397	SCARNA18	3.4519367	3.810248	3.9233398	0.3583112	0.4714031	0.4148572	up
A_22_P00002658	lnc-C1orf201-1	0.1264582	0.6407027	0.441906	0.5142446	0.3154478	0.4148462	up
A_21_P0008869	lnc-TM2D3-1	-0.169995	0.1988401	0.2906847	0.3688355	0.46068	0.4147577	up
A_24_P44514	CIB1	4.117687	4.50695	4.5578737	0.3892627	0.4401865	0.4147246	up
A_33_P3410206	VPS9D1	7.5282774	8.03367	7.8520603	0.505393	0.3237829	0.414588	up
A_23_P39453	MEX3D	1.2517776	1.6704392	1.66224	0.4186616	0.4104624	0.414562	up
A_23_P67399	STRN4	3.1681614	3.76162	3.4037485	0.5934587	0.2355871	0.4145229	up
A_33_P3330841	HUS1	-2.235342	-1.906574	-1.735098	0.3287683	0.5002441	0.4145062	up
A_23_P14072	KRT8	8.487782	8.9524555	8.852018	0.464674	0.3642368	0.4144554	up
A_21_P0014285	LINC01234	0.2657619	0.2813501	1.0789738	0.0155883	0.8132119	0.4144001	up
A_23_P27894	SAFB2	1.1147046	1.5661201	1.4920688	0.4514155	0.3773642	0.4143899	up
A_23_P502078	MAPK8IP2	-2.483617	-2.315215	-1.823432	0.1684017	0.6601856	0.4142937	up
A_23_P251680	COMT	4.8704376	4.9757075	5.593255	0.1052699	0.7228174	0.4140437	up
A_33_P3214310	FOXP1	-1.124323	-0.541434	-0.879132	0.5828896	0.2451911	0.4140403	up
A_24_P65199	CDK10	-0.87752	-0.507478	-0.419536	0.3700419	0.4579845	0.4140132	up
A_23_P168993	ADRB3	-3.04205	-2.728584	-2.527514	0.3134663	0.5145366	0.4140015	up
A_22_P00011377	P2RY2	2.088089	2.136891	2.8669882	0.0488019	0.7788992	0.4138506	up
A_23_P45396	HSD17B10	5.8761587	6.1964755	6.383527	0.3203168	0.5073681	0.4138425	up
A_23_P78835	ZNF787	2.941701	3.2901998	3.4207716	0.3484988	0.4790707	0.4137847	up
A_23_P18142	RPL32	9.524153	10.009489	9.866371	0.4853363	0.3422184	0.4137774	up
A_33_P3411427	ZNF837	2.2990751	2.8438325	2.5818558	0.5447574	0.2827807	0.413769	up
A_21_P0013005	NUBP1	0.0725293	0.6139178	0.358562	0.5413885	0.2860327	0.4137106	up
A_33_P3409625	SORBS3	5.595606	6.0348296	5.9837866	0.4392238	0.3881807	0.4137023	up
A_24_P147540	PRR36	6.658472	7.2018447	6.942395	0.5433726	0.2839232	0.4136479	up

A_33_P3793307	LOC339803	0.6120806	1.2098961	0.8410578	0.5978155	0.2289772	0.4133964	up
A_33_P3335629	TMEM208	4.0061045	4.469196	4.369437	0.4630914	0.3633328	0.4132121	up
A_32_P32254	COL6A1	-1.217251	-0.742336	-0.865817	0.4749155	0.3514342	0.4131749	up
A_21_P0011627	XLOC_l2_005871	1.2135196	1.4962635	1.757112	0.2827439	0.5435925	0.4131682	up
A_23_P159191	GAST	3.2097826	3.8255925	3.4200478	0.6158099	0.2102652	0.4130375	up
A_22_P00012701	lnc-RAB1A-4	-2.965514	-2.333137	-2.77196	0.6323769	0.1935537	0.4129653	up
A_33_P3396473	PPP6R2	2.7578363	3.410297	2.9312	0.6524606	0.1733637	0.4129121	up
A_24_P358381	GTPBP6	0.6141114	1.1782355	0.8757834	0.5641241	0.261672	0.4128981	up
A_32_P184796	RPLP0	9.635297	10.200928	9.895409	0.5656309	0.2601118	0.4128714	up
A_21_P0004331	lnc-NEURL1B-4	-3.116434	-2.876982	-2.530204	0.2394519	0.5862303	0.4128411	up
A_24_P314477	TUBB2B	1.5860529	2.2827373	1.7147999	0.6966844	0.128747	0.4127157	up
A_22_P00016688	lnc-TPSAB1-1	8.713681	9.19775	9.055016	0.4840689	0.3413343	0.4127016	up
A_23_P397308	KLC4	-2.029964	-1.679443	-1.555082	0.3505206	0.4748821	0.4127014	up
A_24_P417935	AGAP2-AS1	0.0496693	0.2431126	0.681385	0.1934433	0.6317158	0.4125796	up
A_22_P00010350	SCEL-AS1	-2.064079	-1.477535	-1.825525	0.586544	0.238554	0.412549	up
A_22_P00005758	LOC101929295	-2.630235	-2.375274	-2.060147	0.254961	0.5700882	0.4125246	up
A_33_P3379947	HLA-B	7.6000166	7.987866	8.037087	0.3878493	0.4370709	0.4124601	up
A_33_P3383912	HLA-DRB3	0.1992154	0.670804	0.5525432	0.4715886	0.3533278	0.4124582	up
A_22_P00024512	JARID2-AS1	-3.321463	-2.572675	-3.245374	0.7487876	0.0760889	0.4124383	up
A_33_P3393694	GLTSCR2	7.720377	8.197811	8.067798	0.4774342	0.3474207	0.4124274	up
A_23_P154539	PRPF6	4.239581	4.7674756	4.536398	0.5278945	0.2968168	0.4123557	up
A_33_P3395008	ACOXL	-0.512783	-0.401908	0.2009769	0.1108747	0.7137599	0.4123173	up
A_23_P7083	CTBP1-AS2	-0.103688	0.2810807	0.3361511	0.384769	0.4398394	0.4123042	up
A_22_P00001553	lnc-ARID2-1	-1.098033	-0.715089	-0.656672	0.3829441	0.4413614	0.4121528	up
A_22_P00014555	LOC100130502	-1.401082	-1.082245	-0.895676	0.3188372	0.5054064	0.4121218	up
A_33_P3369696	CCDC94	3.9567528	4.2646832	4.472975	0.3079305	0.516222	0.4120762	up
A_23_P252283	RNF135	1.988287	2.224197	2.576375	0.2359099	0.588088	0.411999	up
A_23_P62115	TIMP1	5.525625	5.855046	6.0201597	0.3294206	0.4945345	0.4119775	up
A_23_P123330	RPL30	9.287385	9.791025	9.607681	0.5036402	0.3202963	0.4119683	up
A_23_P103433	OSCP1	0.651104	1.1664257	0.9596973	0.5153217	0.3085933	0.4119575	up
A_24_P91701	DRICH1	1.4834805	2.1292243	1.6616449	0.6457439	0.1781645	0.4119542	up
A_22_P00002125	lnc-BOLA2B-1	-0.730037	-0.383348	-0.252983	0.3466883	0.4770541	0.4118712	up
A_33_P3319920	KDM4B	3.278202	3.78904	3.5909863	0.510838	0.3127842	0.4118111	up
A_22_P00020486	lnc-RARRES2-3	-1.575225	-1.000944	-1.325886	0.5742817	0.2493396	0.4118106	up
A_22_P00009395	SSSCA1-AS1	-0.46223	0.1818695	-0.282838	0.6440997	0.1793919	0.4117458	up
A_21_P0009837	lnc-CST8-3	-3.132623	-2.732302	-2.70949	0.400322	0.4231336	0.4117278	up
A_33_P3367447	ALDH3B1	0.2059321	0.9376988	0.2972555	0.7317667	0.0913234	0.411545	up
A_33_P3282075	SP6	4.520507	5.199576	4.664505	0.679069	0.1439982	0.4115336	up
A_33_P3242748	ARFRP1	3.9910975	4.3625073	4.4427032	0.3714099	0.4516058	0.4115079	up
A_33_P3380797	FGF3	4.3149548	4.592773	4.8600063	0.2778182	0.5450516	0.4114349	up
A_23_P53081	OSBPL5	3.5605927	3.9417844	4.0022335	0.3811917	0.4416409	0.4114163	up
A_23_P119214	TIMM44	2.8800402	3.4259534	3.1566486	0.5459132	0.2766085	0.4112608	up
A_23_P354175	TMEM129	1.7029476	2.146316	2.0819893	0.4433684	0.3790417	0.4112051	up
A_24_P288954	SRRD	1.8885679	2.3054996	2.2940378	0.4169316	0.4054699	0.4112008	up
A_33_P3298617	XLOC_l2_006578	-1.3662	-0.884822	-1.025741	0.4813776	0.3404589	0.4109182	up
A_23_P345692	IL17D	-0.012983	0.2251067	0.5707431	0.2380896	0.5837259	0.4109077	up
A_21_P0000311	SNORA14A	-2.151842	-1.711214	-1.770727	0.4406285	0.3811154	0.410872	up
A_33_P3243702	KLHL30	-1.466583	-0.966806	-1.144737	0.4997768	0.3218465	0.4108117	up
A_23_P38254	ELAC2	6.047653	6.342335	6.5745506	0.294682	0.5268974	0.4107897	up
A_24_P30314	SCYL1	5.4682226	5.9073253	5.8506413	0.4391027	0.3824186	0.4107606	up
A_33_P3240353	SLC39A4	6.40246	6.683018	6.943348	0.2805581	0.5408878	0.410723	up
A_19_P00322330	LINC01192	-2.430073	-2.03139	-2.007364	0.3986826	0.422709	0.4106958	up
A_21_P0001334	lnc-PKN2-1	-2.99846	-2.552505	-2.623097	0.4459558	0.3753638	0.4106598	up
A_23_P63432	RHBDL2	1.774579	2.1989255	2.1713943	0.4243465	0.3968153	0.4105809	up
A_33_P3224660	ABCD1	-2.097276	-1.689423	-1.683979	0.4078536	0.4132972	0.4105754	up
A_23_P54846	HERPUD1	5.7667017	6.6087832	5.7456837	0.8420816	-0.021018	0.4105318	up

A_24_P915196	C9orf91	0.5640664	1.143671	0.8054514	0.5796046	0.241385	0.4104948	up
A_21_P0009319	LOC101929494	2.3408747	2.887609	2.6151266	0.5467343	0.2742519	0.4104931	up
A_33_P3400763	PLIN4	-2.142816	-1.228668	-2.236102	0.9141479	-0.093286	0.4104309	up
A_33_P3256560	ZER1	3.7455692	4.1478934	4.1640997	0.4023242	0.4185305	0.4104273	up
A_23_P84952	TFE3	0.9231968	1.4182916	1.2489109	0.4950948	0.3257141	0.4104044	up
A_33_P3238543	MAFG-AS1	0.2954316	0.7998276	0.6116357	0.504396	0.3162041	0.4103	up
A_24_P200942	TSC22D4	1.5241947	2.061131	1.8078504	0.5369363	0.2836556	0.410296	up
A_23_P206960	SEC14L1	3.6204853	4.1263223	3.935154	0.505837	0.3146687	0.4102528	up
A_24_P740705	RPS19BP1	4.707515	5.044417	5.1910934	0.3369021	0.4835787	0.4102404	up
A_33_P3334102	ARHGAP27	2.26118	2.5578303	2.7848215	0.2966504	0.5236416	0.410146	up
A_22_P00006365	lnc-FASTK-1	-3.000476	-2.529113	-2.651908	0.4713633	0.348568	0.4099656	up
A_22_P00008943	KRT81	6.136875	6.6369214	6.45675	0.5000463	0.3198748	0.4099605	up
A_21_P0003308	lnc-MCCC1-1	0.9153838	1.4010382	1.2496319	0.4856544	0.3342481	0.4099512	up
A_23_P329016	DPH7	-0.844256	-0.373309	-0.495333	0.4709468	0.3489227	0.4099348	up
A_21_P0002619	lnc-AC007405.7.1-2	1.9393511	2.4050536	2.293517	0.4657025	0.354166	0.4099343	up
A_21_P0002650	lnc-HS1BP3-1	-1.120421	-0.66519	-0.755958	0.4552302	0.3644624	0.4098463	up
A_21_P0013792	LINC00086	-1.712445	-1.318636	-1.286779	0.3938088	0.4256663	0.4097376	up
A_33_P3360097	APRT	6.701005	7.1021705	7.119231	0.4011655	0.4182262	0.4096959	up
A_33_P3259522	CDCP2	-1.375083	-1.002897	-0.927914	0.3721867	0.4471693	0.409678	up
A_22_P00007019	ZNF337-AS1	1.4519405	1.9201818	1.8030262	0.4682412	0.3510857	0.4096634	up
A_23_P213832	SPINK7	0.8881774	1.3721108	1.2234812	0.4839335	0.3353038	0.4096186	up
A_23_P86801	RAPSN	-3.493578	-2.945774	-3.222247	0.5478044	0.2713308	0.4095676	up
A_22_P00009936	HCP5	3.260087	3.6882138	3.6506891	0.4281268	0.3906021	0.4093645	up
A_33_P3278560	ZIK1	-3.263673	-2.851611	-2.857042	0.4120619	0.4066303	0.4093461	up
A_32_P42946	C1orf210	-0.015962	0.2805176	0.5061946	0.2964792	0.5221562	0.4093177	up
A_33_P3332396	TMEM53	0.1773601	0.4897099	0.6835523	0.3123498	0.5061922	0.409271	up
A_23_P119130	RPS19	9.605504	10.15126	9.878252	0.5457563	0.272748	0.4092522	up
A_23_P154894	CSTB	6.965657	7.3965917	7.353159	0.4309344	0.3875017	0.4092181	up
A_23_P52298	NPM3	4.0408974	4.2624125	4.6375895	0.2215152	0.5966921	0.4091036	up
A_24_P916718	ZNF467	-3.12461	-2.762889	-2.668125	0.3617203	0.4564846	0.4091024	up
A_33_P3302320	LINC01251	-3.139556	-2.610306	-2.850623	0.5292499	0.2889333	0.4090916	up
A_23_P171336	NXF3	-1.925172	-1.663934	-1.368275	0.2612376	0.5568972	0.4090674	up
A_24_P414269	ALG3	5.369608	5.872885	5.684252	0.5032773	0.3146439	0.4089606	up
A_21_P0008220	lnc-TUBGCP3-6	-2.660319	-2.074821	-2.428146	0.5854988	0.2321732	0.408836	up
A_23_P100455	MTHFSD	-0.15995	0.3362303	0.161408	0.4961805	0.3213582	0.4087694	up
A_22_P00003446	lnc-CCDC51-1	-0.551901	-0.000523	-0.285923	0.5513783	0.2659774	0.4086778	up
A_33_P3272990	FKBP8	2.4019938	2.8871121	2.7339048	0.4851184	0.3319111	0.4085148	up
A_33_P3335386	FAM83G	4.400028	4.918118	4.698599	0.5180898	0.2985706	0.4083302	up
A_22_P00007346	lnc-GPT2-1	-0.837695	-0.43623	-0.422575	0.4014649	0.4151201	0.4082925	up
A_23_P113237	CRLF2	-2.265807	-1.869837	-1.845192	0.3959703	0.4206147	0.4082925	up
A_33_P3384287	PALM	2.702014	3.013371	3.2070837	0.311357	0.5050697	0.4082134	up
A_22_P00012600	lnc-PTPRCAP-1	0.1411586	0.5403371	0.5583534	0.3991785	0.4171948	0.4081867	up
A_23_P157784	HINT2	3.0741405	3.5916276	3.3729992	0.5174871	0.2988586	0.4081728	up
A_33_P3236651	MAP2K7	5.0047283	5.4656014	5.3599157	0.4608731	0.3551874	0.4080303	up
A_23_P158596	AGTRAP	0.2951174	0.665678	0.7403431	0.3705607	0.4452257	0.4078932	up
A_33_P3344127	HIST1H2AC	3.0866594	3.4722228	3.5168695	0.3855634	0.4302101	0.4078867	up
A_23_P11461	UBE2V1	4.3230085	4.681274	4.7804403	0.3582654	0.4574318	0.4078486	up
A_33_P3275330	NADK	-0.764333	-0.316494	-0.39649	0.4478397	0.3678432	0.4078414	up
A_22_P00014994	LOC102723385	-1.968183	-1.507925	-1.612855	0.4602575	0.3553281	0.4077928	up
A_22_P00019592	MRPL9	3.3602753	3.7902455	3.7456713	0.4299703	0.385396	0.4076831	up
A_22_P00009167	lnc-LMX1A-1	-2.452397	-2.125362	-1.964326	0.3270354	0.488071	0.4075532	up
A_33_P3317392	ADAMTS19	-3.025677	-2.72023	-2.516078	0.3054471	0.5095999	0.4075235	up
A_23_P2873	KLC1	1.2840595	1.8735571	1.5095043	0.5894976	0.2254448	0.4074712	up
A_21_P0008134	lnc-CDX2-1	-2.076544	-1.536444	-1.801722	0.5400996	0.2748218	0.4074607	up
A_33_P3329462	DLEU1-AS1	-0.429117	-0.062454	0.018899	0.3666625	0.4480157	0.4073391	up

A_33_P3342628	HES4	7.2005196	7.714724	7.500963	0.5142045	0.3004437	0.4073241	up
A_23_P162970	IPO4	2.5267677	2.9089665	2.9590492	0.3821988	0.4322815	0.4072402	up
A_23_P141389	RPL27	9.25128	9.702408	9.614516	0.451128	0.3632364	0.4071822	up
A_33_P3418597	GAS2L1	1.609354	1.8766294	2.1564255	0.2672753	0.5470715	0.4071734	up
A_23_P7397	PCDHB10	-2.258053	-2.083559	-1.618241	0.1744943	0.6398115	0.4071529	up
A_23_P146908	STX8	1.7805119	2.134585	2.240674	0.3540731	0.4601622	0.4071176	up
A_22_P00011670	LINC00271	3.0360355	3.614184	3.2720938	0.5781484	0.2360582	0.4071033	up
A_33_P3359354	C1orf86	1.1884832	1.6628399	1.5283289	0.4743567	0.3398457	0.4071012	up
A_33_P3317406	NDUFV1	6.4972677	6.9443173	6.8643856	0.4470496	0.3671179	0.4070838	up
A_23_P74178	HCRTR1	-0.912542	-0.472099	-0.538838	0.440443	0.373704	0.4070735	up
A_21_P0008580	LOC101929151	0.0850482	0.5958781	0.3882985	0.5108299	0.3032503	0.4070401	up
A_21_P0011814	LOC102724689	3.5992746	4.0219207	3.9906158	0.4226461	0.3913412	0.4069936	up
A_24_P161581	SHISA7	-1.227592	-0.704621	-0.936895	0.5229707	0.2906961	0.4068334	up
A_23_P78888	FBL	8.897854	9.286886	9.322315	0.3890324	0.4244614	0.4067469	up
A_33_P3381235	SLCO4A1-AS1	-0.586895	-0.096126	-0.264183	0.4907689	0.3227119	0.4067404	up
A_23_P103905	UFC1	3.453063	3.9047494	3.8147945	0.4516864	0.3617315	0.406709	up
A_24_P941625	ZNF70	-0.833319	-0.39578	-0.457537	0.4375391	0.3757825	0.4066608	up
A_21_P0008744	lnc-ADAM10-2	0.2477713	1.0463519	0.262434	0.7985807	0.0146627	0.4066217	up
A_22_P00014572	lnc-SLA2-2	-1.793973	-1.56336	-1.211548	0.2306132	0.5824251	0.4065192	up
A_24_P218805	HOXC10	0.555131	1.0992332	0.8238368	0.5441022	0.2687058	0.406404	up
A_23_P51548	MGST3	7.01978	7.4817967	7.370184	0.4620166	0.3504038	0.4062102	up
A_23_P171249	IGBP1	4.365587	4.9904695	4.553109	0.6248822	0.1875219	0.4062021	up
A_33_P3409508	MAPK11	-1.638744	-1.152807	-1.312279	0.4859366	0.3264651	0.4062009	up
A_23_P351138	CLDN9	-3.073694	-2.800479	-2.534565	0.2732153	0.5391293	0.4061723	up
A_23_P125348	TRAPPC1	1.736146	2.045505	2.2390337	0.3093591	0.5028877	0.4061234	up
A_33_P3410849	C8orf58	3.4915934	3.971126	3.8241587	0.4795327	0.3325653	0.406049	up
A_33_P3340105	BASP1P1	-2.044457	-1.376646	-1.900412	0.6678114	0.1440458	0.4059286	up
A_23_P50146	SIGLEC15	-0.086549	0.3904614	0.2482429	0.4770107	0.3347921	0.4059014	up
A_23_P34741	ZNF593	4.527913	4.7333837	5.1339855	0.2054706	0.6060724	0.4057715	up
A_33_P3293858	MTUS2	-2.106818	-1.980455	-1.421668	0.1263628	0.6851497	0.4057562	up
A_33_P3418000	RELL1	-2.859582	-2.186598	-2.721087	0.6729834	0.1384943	0.4057388	up
A_23_P30805	HIST1H4J	4.613635	4.937611	5.101118	0.323976	0.487483	0.4057295	up
A_22_P00006108	lnc-FAM171B-1	-2.28226	-1.779102	-1.974033	0.5031571	0.3082266	0.4056919	up
A_22_P00010889	lnc-NPFFR2-2	-2.207899	-1.686778	-1.917679	0.521121	0.2902203	0.4056706	up
A_23_P115785	FANK1	-0.403826	-0.0694	0.0730667	0.3344264	0.476893	0.4056597	up
A_23_P105307	DGKA	0.7559691	1.2115202	1.111331	0.4555512	0.3553619	0.4054565	up
A_33_P3362781	KLHL36	-1.078757	-0.65632	-0.690331	0.4224372	0.3884268	0.405432	up
A_22_P00009101	lnc-LIFR-1	0.4098091	0.7757545	0.8546977	0.3659453	0.4448886	0.405417	up
A_24_P127719	MAFA	0.1866469	0.6748767	0.5090675	0.4882298	0.3224206	0.4053252	up
A_33_P3267822	MICAL3	-2.493211	-2.301873	-1.874026	0.1913381	0.6191843	0.4052612	up
A_22_P00010935	LOC102724872	-2.331745	-2.055808	-1.797344	0.2759371	0.5344014	0.4051692	up
A_23_P163942	SUPT4H1	5.317911	5.6616254	5.7844877	0.3437142	0.4665766	0.4051454	up
A_33_P3277328	LOC730102	-2.989873	-2.466429	-2.703296	0.5234432	0.2865768	0.40501	up
A_23_P124927	RGS14	0.8064423	1.1669455	1.2557011	0.3605032	0.4492588	0.404881	up
A_32_P99753	EFCAB12	-1.608007	-1.272097	-1.134437	0.3359103	0.4735704	0.4047403	up
A_24_P391991	FAM183B	-0.44369	0.0526075	-0.13074	0.4962974	0.3129497	0.4046235	up
A_24_P298027	AXIN2	5.098036	5.4794326	5.525666	0.3813968	0.4276304	0.4045136	up
A_24_P306720	FOXN3-AS1	-0.569342	-0.102651	-0.227076	0.466691	0.3422661	0.4044786	up
A_23_P160240	ACP6	0.4877453	1.0351095	0.7491765	0.5473642	0.2614312	0.4043977	up
A_24_P179351	TPT1	8.769919	9.277954	9.070623	0.5080347	0.300704	0.4043694	up
A_23_P115223	HAX1	4.868944	5.570376	4.9762325	0.7014318	0.1072884	0.4043601	up
A_33_P3240518	AURKAIP1	5.1715097	5.4985337	5.653181	0.327024	0.4816713	0.4043477	up
A_22_P00025022	lnc-LRRC49-2	-1.552287	-1.040708	-1.255194	0.511579	0.2970934	0.4043362	up
A_21_P0008116	lnc-COL4A2-3	0.9951396	1.3675332	1.4313354	0.3723936	0.4361959	0.4042947	up
A_23_P213718	UQCRQ	9.019722	9.439369	9.408591	0.4196472	0.3888693	0.4042583	up
A_33_P3366859	BAGE	-2.861492	-2.189632	-2.724911	0.67186	0.1365812	0.4042206	up

A_33_P3224809	IL17RA	3.8271618	4.197128	4.265459	0.369966	0.4382973	0.4041317	up
A_23_P57521	EIF3L	5.2129726	5.9214883	5.312669	0.7085156	0.0996962	0.4041059	up
A_33_P3215744	PART1	-0.623368	-0.352464	-0.086118	0.2709041	0.53725	0.4040771	up
A_22_P00014450	lnc-SH2D7-5	-0.563161	0.0135827	-0.331835	0.5767436	0.2313256	0.4040346	up
A_33_P3290487	ZNF668	4.61897	5.047028	4.998926	0.4280582	0.3799563	0.4040072	up
A_33_P3261803	FAAH	-0.220159	0.3948503	-0.027176	0.6150088	0.1929827	0.4039958	up
A_21_P0006338	LOC100133077	-3.422884	-3.014694	-3.023099	0.4081895	0.3997843	0.4039869	up
A_33_P3271246	LOC100288842	-2.537056	-2.130664	-2.135649	0.4063914	0.4014065	0.403899	up
A_23_P206510	GLG1	5.8661213	6.3097167	6.2302647	0.4435954	0.3641434	0.4038694	up
A_32_P23010	SDHAF1	4.6852236	5.1284356	5.049718	0.443212	0.3644943	0.4038532	up
A_23_P103282	TMEM59	5.5701323	6.0702853	5.877452	0.5001531	0.3073196	0.4037364	up
A_22_P00017561	LINC00094	0.2584119	0.6821361	0.641861	0.4237242	0.3834491	0.4035866	up
A_23_P502553	TRIM35	1.410142	1.6966043	1.9308224	0.2864623	0.5206804	0.4035714	up
A_21_P0008099	SLITRK5	-2.959761	-2.564401	-2.547992	0.3953598	0.4117684	0.4035641	up
A_24_P406132	MAPK13	2.241025	2.7018304	2.5869255	0.4608054	0.3459005	0.403353	up
A_33_P3266928	LMTK3	3.854391	4.358854	4.1565886	0.5044627	0.3021975	0.4033301	up
A_19_P00315863	LINC00607	-2.667992	-2.590472	-1.938945	0.0775197	0.7290463	0.403283	up
A_23_P12199	FAM46B	-0.863298	-0.035941	-0.884148	0.8273573	-0.02085	0.4032538	up
A_23_P38154	FDXR	5.7655916	6.157519	6.180155	0.3919272	0.4145632	0.4032452	up
A_21_P0000238	SNORD12C	2.5053291	2.8854165	2.9315987	0.3800874	0.4262695	0.4031785	up
A_33_P3338341	PRODH	1.5097594	2.0370421	1.7887039	0.5272827	0.2789445	0.4031136	up
A_22_P00013715	EWSAT1	1.3034425	1.8255696	1.5874863	0.5221272	0.2840438	0.4030855	up
A_23_P310532	C19orf52	2.7942505	3.3106933	3.0839777	0.5164428	0.2897272	0.403085	up
A_33_P3355014	TMEM229B	0.7428169	1.0561543	1.2356	0.3133373	0.4927831	0.4030602	up
A_23_P103398	PSEN2	4.384139	4.821713	4.7523956	0.4375739	0.3682566	0.4029152	up
A_33_P3402035	TRAF4	3.7510633	4.3135886	3.9943304	0.5625253	0.2432671	0.4028962	up
A_23_P323094	PHC1	-2.900459	-2.631196	-2.364217	0.2692633	0.5362423	0.4027528	up
A_33_P3292840	C11orf49	-0.094737	0.2568445	0.3586502	0.3515816	0.4533873	0.4024844	up
A_23_P382602	BCL9	0.6587119	0.9567876	1.1654735	0.2980757	0.5067616	0.4024186	up
A_23_P132248	ZMAT5	0.0621448	0.484899	0.4441848	0.4227543	0.38204	0.4023972	up
A_33_P3417880	NKAPL	-1.39519	-1.020916	-0.964802	0.3742743	0.430388	0.4023311	up
A_22_P00015829	LOC284930	0.1975846	0.6956539	0.5040517	0.4980693	0.3064671	0.4022682	up
A_23_P46170	MED8	2.999999	3.4364057	3.3681164	0.4364066	0.3681173	0.402262	up
A_23_P214876	JARID2	1.5515041	1.9727349	1.9346352	0.4212308	0.383131	0.4021809	up
A_33_P3249076	HOGA1	-3.0802	-2.844236	-2.511806	0.2359648	0.5683944	0.4021796	up
A_21_P0014844	lnc-MRPS25-1	-1.515252	-1.083185	-1.143113	0.4320669	0.3721395	0.4021032	up
A_24_P339416	ARSG	-2.414681	-1.811615	-2.213596	0.6030664	0.2010858	0.4020761	up
A_21_P0000224	SNORD83A	-0.082791	0.3457532	0.2927141	0.4285445	0.3755055	0.402025	up
A_23_P56249	TBCB	5.596465	6.092961	5.903925	0.4964957	0.3074598	0.4019778	up
A_23_P69431	RPL4	8.379356	8.929796	8.632569	0.5504398	0.2532129	0.4018264	up
A_23_P210619	RTFDC1	4.4150248	4.72924	4.904418	0.3142152	0.4893932	0.4018042	up
A_22_P00010069	LOC101927204	-0.719	-0.27208	-0.362561	0.4469204	0.3564391	0.4016798	up
A_21_P0013052	XLOC_l2_012836	-2.737272	-2.164011	-2.507227	0.5732606	0.2300444	0.4016525	up
A_33_P3268649	SLC2A11	-1.395047	-0.894489	-1.092475	0.5005584	0.3025718	0.4015651	up
A_21_P0006310	lnc-C9orf152-1	-1.06532	-0.568086	-0.759438	0.4972343	0.3058825	0.4015584	up
A_22_P00019271	LOC100507091	0.5627394	1.0190701	0.9092917	0.4563308	0.3465524	0.4014416	up
A_23_P115064	CRABP2	3.8039865	4.162951	4.247859	0.3589644	0.4438725	0.4014184	up
A_24_P102821	PTAFR	-2.173547	-2.136212	-1.408065	0.0373349	0.7654824	0.4014087	up
A_33_P7717144	lnc-RNF39-4	-0.904382	-0.427959	-0.578097	0.4764237	0.3262854	0.4013546	up
A_23_P152970	RAPGEFL1	2.3926	2.7758288	2.812005	0.3832288	0.419405	0.4013169	up
A_23_P2884	PIGH	0.6676111	1.0771136	1.0605421	0.4095025	0.392931	0.4012167	up
A_23_P217054	DCAF10	1.020814	1.5638576	1.2800918	0.5430436	0.2592778	0.4011607	up
A_33_P3307447	RPL32P3	6.3186464	6.7663045	6.6733007	0.4476581	0.3546543	0.4011562	up
A_24_P57367	AHCY	4.184492	4.666285	4.5049314	0.4817929	0.3204393	0.4011161	up
A_33_P3420442	CDK20	-1.031226	-0.599792	-0.660428	0.4314337	0.3707976	0.4011157	up
A_23_P79221	ACVR1	0.8429008	1.424809	1.0631943	0.5819082	0.2202935	0.4011009	up

A_23_P122852	SMARCD3	1.825347	2.0965667	2.3562899	0.2712197	0.5309429	0.4010813	up
A_33_P3243264	DNLZ	3.1070614	3.4613557	3.5549078	0.3542943	0.4478464	0.4010704	up
A_23_P205293	EXD2	-1.541434	-1.111061	-1.169996	0.4303732	0.3714376	0.4009054	up
A_33_P3405334	GM2A	3.7341537	4.0968375	4.1731853	0.3626838	0.4390316	0.4008577	up
A_23_P141405	NME2	9.515215	9.937611	9.894501	0.4223957	0.3792858	0.4008408	up
A_23_P39088	PRMT1	5.2910585	5.6912866	5.6924314	0.400228	0.4013729	0.4008005	up
A_23_P203488	SMPD1	-2.257965	-1.701527	-2.012834	0.5564385	0.2451315	0.400785	up
A_23_P254917	LGALS4	-3.190613	-3.104296	-2.475369	0.0863168	0.7152448	0.4007808	up
A_23_P255968	TAAR5	-0.219288	0.2650476	0.097918	0.4843359	0.3172064	0.4007711	up
A_23_P141549	RPS7	9.414722	9.884882	9.745919	0.4701595	0.3311968	0.4006782	up
A_22_P00019311	lnc-MARVELD3-2	1.0434504	1.5333457	1.354877	0.4898953	0.3114266	0.400661	up
A_33_P3248765	ADIPOR1	0.4303203	0.9117971	0.7498798	0.4814768	0.3195596	0.4005182	up
A_33_P3360773	TSSC4	2.6300087	3.0531964	3.007722	0.4231877	0.3777132	0.4004505	up
A_24_P15765	RPS7P5	7.5195103	8.052198	7.7875624	0.5326881	0.2680521	0.4003701	up
A_22_P00017350	GABPB1-AS1	1.5353804	1.8912191	1.9800749	0.3558388	0.4446945	0.4002667	up
A_33_P8912881	LINC00601	3.3701324	3.7551293	3.7856388	0.3849969	0.4155064	0.4002516	up
A_33_P3360301	USP19	2.7897406	3.1050735	3.2749052	0.3153329	0.4851646	0.4002488	up
A_23_P148513	GNG5	5.806616	6.2509274	6.1625547	0.4443116	0.3559389	0.4001253	up
A_23_P208850	RPS16	9.746358	10.230234	10.062696	0.4838762	0.3163385	0.4001074	up
A_24_P917886	MUC5AC	-1.97687	-1.616155	-1.537531	0.3607144	0.4393382	0.4000263	up
A_23_P29036	IFNGR2	0.9861636	1.3225994	1.4492774	0.3364358	0.4631138	0.3997748	up
A_33_P3406408	MANBAL	2.6012354	2.7975554	3.2044268	0.1963201	0.6031914	0.3997557	up
A_19_P00801735	SEPT7-AS1	3.911868	4.4052215	4.2177553	0.4933534	0.3058872	0.3996203	up
A_23_P128246	FICD	0.3690105	1.208478	0.328608	0.8394675	-0.040402	0.3995325	up
A_24_P143440	DYNLRB1	3.0106268	3.3979707	3.4222813	0.3873439	0.4116545	0.3994992	up
A_21_P0007108	lnc-PCBD1-2	-2.006736	-1.601884	-1.612936	0.4048524	0.3938007	0.3993266	up
A_23_P206792	ZNF764	-2.883031	-2.333548	-2.634149	0.5494838	0.2488821	0.3991829	up
A_33_P3282005	VANGL2	-0.879608	-0.215802	-0.745136	0.6638055	0.1344719	0.3991387	up
A_21_P0011149	XLOC_l2_003758	1.3007178	1.7745104	1.6251955	0.4737926	0.3244777	0.3991351	up
A_33_P3290394	IL2RG	-1.95128	-1.629947	-1.474572	0.3213329	0.4767075	0.3990202	up
A_24_P198844	MPDU1	2.1465683	2.8301883	2.2609024	0.68362	0.1143341	0.3989771	up
A_33_P3325704	SPRR2E	1.1906204	1.8445773	1.3343511	0.6539569	0.1437306	0.3988438	up
A_23_P205519	ABHD4	0.2378807	0.890739	0.3826418	0.6528583	0.1447611	0.3988097	up
A_24_P64362	WBP1L	2.0822086	2.550303	2.4115715	0.4680944	0.3293629	0.3987286	up
A_23_P336678	GPHB5	-1.587048	-1.21026	-1.16649	0.3767877	0.420558	0.3986728	up
A_24_P153043	RPL23AP7	8.134914	8.628796	8.438302	0.4938812	0.3033876	0.3986344	up
A_23_P29851	LRPAP1	2.444847	2.8649573	2.8218393	0.4201102	0.3769922	0.3985512	up
A_22_P00006879	PRKAG2-AS1	-3.176095	-2.481663	-3.073494	0.6944325	0.1026011	0.3985168	up
A_33_P3214105	ATF3	-0.028599	0.7505951	-0.010891	0.7791939	0.0177078	0.3984509	up
A_33_P3288754	C19orf48	5.6838045	6.2229657	5.941349	0.5391612	0.2575445	0.3983529	up
A_33_P3344264	DUS3L	2.9241009	3.2778869	3.366969	0.353786	0.4428682	0.3983271	up
A_24_P212152	MZT2B	9.043819	9.44578	9.4384985	0.4019604	0.3946791	0.3983197	up
A_19_P00801042	ZNF90	5.944704	6.409677	6.27633	0.464973	0.3316259	0.3982995	up
A_22_P00017343	lnc-USP47-2	-1.525689	-1.099892	-1.154948	0.4257965	0.3707409	0.3982687	up
A_32_P179676	TOB2	2.838728	3.3074517	3.166482	0.4687238	0.327754	0.3982389	up
A_21_P0005571	lnc-ZKSCAN1-1	3.0448065	3.4167104	3.4693432	0.3719039	0.4245367	0.3982203	up
A_22_P00023999	lnc-C1orf31-1	0.1436019	0.6084728	0.4750605	0.4648709	0.3314586	0.3981648	up
A_33_P3352712	CDR2L	-0.894977	-0.299154	-0.694554	0.5958228	0.2004232	0.398123	up
A_33_P3327702	SYMPK	7.2488966	7.7118335	7.5819397	0.4629369	0.3330431	0.39799	up
A_22_P00020232	lnc-APOA1-1	-1.762995	-1.320617	-1.409451	0.442378	0.3535438	0.3979609	up
A_33_P3241325	MAFG-AS1	-1.529412	-1.308822	-0.954106	0.2205896	0.5753055	0.3979476	up
A_33_P3334895	GRIN2A	-0.952348	-0.636492	-0.472372	0.3158565	0.4799767	0.3979166	up
A_33_P3319581	FIGNL2	1.7516937	2.1394544	2.1595793	0.3877606	0.4078856	0.3978231	up
A_19_P00808668	WDR82	3.7111273	4.1803765	4.037341	0.4692493	0.3262138	0.3977315	up
A_22_P00001217	lnc-ANKRD11-1	-1.564158	-1.073146	-1.25991	0.4910116	0.3042479	0.3976297	up
A_23_P204472	RPLP0	8.922845	9.494627	9.146175	0.5717821	0.2233305	0.3975563	up

A_23_P202156	NFKB2	0.5182309	0.9423451	0.8892036	0.4241142	0.3709726	0.3975434	up
A_33_P3306068	MYCL	-0.09702	0.1387515	0.4622936	0.2357717	0.5593138	0.3975427	up
A_22_P00010667	lnc-NES-1	-0.410103	0.0046492	-0.029977	0.414752	0.380126	0.397439	up
A_21_P0008851	lnc-ITGA11-2	0.0614109	0.5530591	0.3645325	0.4916482	0.3031216	0.3973849	up
A_33_P3347168	TBL1X	2.6587334	3.3172355	2.7948494	0.6585021	0.136116	0.3973091	up
A_23_P10135	NKIRAS2	0.9046478	1.4500031	1.1535587	0.5453553	0.2489109	0.3971331	up
A_33_P3399051	BAIAP2L2	-2.253255	-1.804951	-1.907471	0.4483042	0.3457842	0.3970442	up
A_33_P3325006	TSSC4	5.8633165	6.3229136	6.1977262	0.4595971	0.3344097	0.3970034	up
A_33_P3273364	YIPF2	3.4795256	4.1153636	3.6376667	0.635838	0.1581411	0.3969896	up
A_23_P65930	ZFYVE19	3.4391012	3.8184981	3.8535662	0.3793969	0.414465	0.3969309	up
A_33_P3273148	GADD45GIP1	4.3605967	4.770347	4.7446156	0.4097505	0.3840189	0.3968847	up
A_23_P352389	SPATA32	-2.29323	-1.84562	-1.947088	0.4476099	0.3461423	0.3968761	up
A_23_P202769	DNAJC4	0.5822697	0.9651604	0.993114	0.3828907	0.4108443	0.3968675	up
A_22_P00010856	lnc-NOS2-2	-1.567702	-1.071355	-1.270768	0.496347	0.2969341	0.3966405	up
A_22_P00005316	lnc-DNASE1-3	-2.118241	-1.846986	-1.596242	0.271255	0.5219994	0.3966272	up
A_33_P3427239	LOC100134937	-1.439741	-1.031186	-1.055218	0.408555	0.3845224	0.3965387	up
A_23_P201002	RNF220	5.956871	6.403974	6.302784	0.447103	0.3459129	0.396508	up
A_23_P372860	HIST1H2AC	-0.743581	-0.366016	-0.328147	0.3775644	0.4154339	0.3964992	up
A_33_P3235117	LOC100133286	-0.686248	-0.106411	-0.473097	0.5798369	0.2131505	0.3964937	up
A_22_P00005022	lnc-DDX31-1	-1.825022	-1.412718	-1.444505	0.4123035	0.3805165	0.39641	up
A_22_P00024059	lnc-NDE1-1	1.6922541	2.151319	2.025877	0.459065	0.3336229	0.3963439	up
A_23_P140876	ABCA3	-0.259058	0.122035	0.1525054	0.3810926	0.4115629	0.3963277	up
A_32_P58407	KCND3	-0.290895	0.1530075	0.0578566	0.4439025	0.3487515	0.396327	up
A_23_P34537	EPHX1	3.2586765	3.89741	3.4124527	0.6387334	0.1537762	0.3962548	up
A_33_P3335124	RPS2	8.46942	8.935892	8.795334	0.4664717	0.3259134	0.3961926	up
A_32_P212471	RIIAD1	-2.12735	-1.844043	-1.618302	0.2833066	0.5090475	0.3961771	up
A_33_P3235340	DDX18	3.648034	4.0357556	4.052397	0.3877215	0.4043627	0.3960421	up
A_33_P3220095	ZNF341	2.2455912	2.7417865	2.5413427	0.4961953	0.2957516	0.3959734	up
A_24_P928901	SLC31A1	0.7929802	1.1785007	1.1993442	0.3855205	0.406364	0.3959422	up
A_23_P387630	STARD8	-3.047357	-2.709689	-2.593147	0.3376675	0.4542096	0.3959385	up
A_33_P3342235	CDC42EP5	7.40042	7.8910336	7.7014627	0.4906135	0.3010426	0.395828	up
A_23_P106844	MT2A	7.178585	7.4770646	7.671754	0.2984796	0.4931688	0.3958242	up
A_23_P108265	OR7C2	-0.451226	0.0621319	-0.172983	0.5133581	0.2782435	0.3958008	up
A_22_P00013906	LINC00595	-0.896381	-0.456108	-0.545209	0.4402738	0.351172	0.3957229	up
A_24_P321068	SLC31A1	0.1611066	0.6611118	0.4524198	0.5000053	0.2913132	0.3956592	up
A_22_P00008254	MYO16-AS1	-2.064054	-1.551947	-1.784966	0.5121064	0.2790875	0.395597	up
A_24_P383581	C19orf70	3.670988	4.085607	4.0474586	0.414619	0.3764706	0.3955448	up
A_22_P00005191	lnc-DKK3-1	0.2631521	0.7676444	0.5495672	0.5044923	0.2864151	0.3954537	up
A_23_P210482	ADA	4.3179426	4.65383	4.7729063	0.3358874	0.4549637	0.3954256	up
A_33_P3338116	LAMB2	3.235446	4.0489745	3.212737	0.8135285	-0.022709	0.3954098	up
A_21_P0012880	BRD9	-0.589815	-0.072365	-0.316482	0.5174499	0.2733326	0.3953912	up
A_33_P3349444	NAGA	3.0450344	3.476501	3.4043026	0.4314666	0.3592682	0.3953674	up
A_22_P00022015	lnc-GPR143-1	-2.100022	-1.828558	-1.580757	0.2714644	0.5192652	0.3953648	up
A_33_P3394599	HMG20B	2.101019	2.5582004	2.4345493	0.4571815	0.3335304	0.3953559	up
A_33_P3409513	MAPK11	0.6296601	0.9864397	1.0635624	0.3567796	0.4339023	0.3953409	up
A_21_P0002349	lnc-SMARCAL1-2	-0.972866	-0.718603	-0.436642	0.2542625	0.5362234	0.3952429	up
A_33_P3300680	C19orf12	0.7085452	1.1514492	1.0560727	0.442904	0.3475275	0.3952158	up
A_33_P3215739	LNP1	-1.686373	-1.260508	-1.321826	0.4258652	0.3645477	0.3952065	up
A_24_P81900	SLC2A3	-0.00745	0.6084018	0.1670861	0.6158514	0.1745358	0.3951936	up
A_23_P330419	ACPT	-2.469729	-2.038074	-2.111005	0.4316547	0.3587239	0.3951893	up
A_22_P00007150	SLC38A3	1.8340874	2.406815	2.0516014	0.5727277	0.217514	0.3951209	up
A_23_P36825	GPRC5A	3.0250607	3.632339	3.2078037	0.6072784	0.1827431	0.3950107	up
A_21_P0011491	XLOC_l2_005557	10.110591	10.571533	10.439291	0.4609423	0.3287001	0.3948212	up
A_23_P255523	ALKBH4	1.2430067	1.6282568	1.6473961	0.3852501	0.4043894	0.3948197	up
A_24_P67308	RPL19P12	10.148549	10.586208	10.500517	0.4376593	0.3519678	0.3948135	up
A_21_P0003279	lnc-WNT7A-1	-2.713452	-2.473644	-2.163775	0.2398076	0.5496764	0.394742	up

A_22_P00021246	lnc-DPP4-1	-2.834213	-2.32247	-2.556475	0.5117431	0.2777379	0.3947405	up
A_23_P124190	TRIM34	-1.316176	-1.018993	-0.824028	0.297183	0.4921484	0.3946657	up
A_22_P00000457	OSMR-AS1	-2.008421	-1.500683	-1.727	0.5077376	0.2814207	0.3945792	up
A_32_P54274	DRD5	-3.361373	-2.822157	-3.111507	0.5392153	0.2498658	0.3945405	up
A_23_P162879	APOPT1	2.4237442	2.832666	2.803712	0.4089217	0.3799677	0.3944447	up
A_21_P0002692	lnc-EPHA4-2	-2.92927	-2.395753	-2.674044	0.5335169	0.2552264	0.3943716	up
A_22_P00011639	LOC101927206	3.6002808	4.0646186	3.9246416	0.4643378	0.3243609	0.3943493	up
A_24_P389491	COQ4	0.596148	1.1129484	0.8679786	0.5168004	0.2718306	0.3943155	up
A_23_P60627	ALOX15B	-1.871097	-1.327025	-1.626701	0.5440717	0.2443962	0.394234	up
A_33_P3244640	GRK5	-2.072795	-1.767329	-1.589866	0.3054662	0.4829297	0.3941979	up
A_22_P00007359	lnc-GRAMD4-2	1.0549221	1.3161812	1.5820017	0.2612591	0.5270796	0.3941693	up
A_23_P165698	C2orf49	0.5420251	1.0224876	0.8497458	0.4804626	0.3077207	0.3940916	up
A_23_P108157	TJP3	0.5515595	1.129024	0.7620711	0.5774646	0.2105117	0.3939881	up
A_21_P0000530	LRRC75A-AS1	2.5934248	3.0718617	2.9028816	0.478437	0.3094568	0.3939469	up
A_33_P3339825	EMC10	0.9032822	1.2837696	1.3104267	0.3804874	0.4071446	0.393816	up
A_22_P00004211	LINC01511	-1.395618	-1.052782	-0.950853	0.3428364	0.4447656	0.393801	up
A_33_P3396275	C15orf62	-0.853181	-0.493024	-0.425916	0.3601575	0.4272652	0.3937113	up
A_33_P3396522	POLR2G	6.3945208	6.7469583	6.8294277	0.3524375	0.434907	0.3936722	up
A_21_P0001050	PCAT6	-1.747989	-1.537651	-1.171041	0.2103381	0.5769477	0.3936429	up
A_23_P83599	PRKAR1B	0.0979824	0.4783225	0.5046907	0.3803401	0.4067082	0.3935242	up
A_22_P00000435	lnc-AC074212.3.1-1	2.8025398	3.220696	3.1712456	0.4181562	0.3687058	0.393431	up
A_23_P359540	HIST1H4F	2.8651934	3.1746707	3.3423557	0.3094773	0.4771624	0.3933198	up
A_21_P0012679	LOC101927282	-2.238209	-1.68666	-2.003183	0.5515494	0.2350259	0.3932877	up
A_24_P262738	DDA1	2.1965647	2.6411967	2.5385065	0.4446321	0.3419418	0.3932869	up
A_22_P00002213	LOC102723751	-2.225104	-1.784313	-1.879417	0.4407907	0.3456869	0.3932388	up
A_33_P3423300	TNPO2	4.883444	5.1491027	5.4040766	0.2656589	0.5206327	0.3931458	up
A_33_P3255409	PIH1D1	5.948539	6.3689685	6.314392	0.4204297	0.3658533	0.3931415	up
A_23_P251593	RPL10A	9.561725	10.004969	9.904217	0.443244	0.3424921	0.392868	up
A_33_P3387170	MATN1-AS1	1.1836462	1.6155014	1.5374117	0.4318552	0.3537655	0.3928104	up
A_33_P3320503	RAI1	-2.626704	-2.270366	-2.197447	0.3563378	0.4292567	0.3927972	up
A_22_P00014697	TPT1	3.7743692	4.4116464	3.9226618	0.6372771	0.1482925	0.3927848	up
A_22_P00010040	LOC145783	-1.068673	-0.723295	-0.628517	0.3453779	0.4401565	0.3927672	up
A_23_P146134	DUSP26	-3.372182	-2.814655	-3.144409	0.5575271	0.2277737	0.3926504	up
A_19_P00321132	LOC101927136	-2.342126	-1.908572	-1.990507	0.4335547	0.3516197	0.3925872	up
A_23_P125519	RPS4X	8.867064	9.372803	9.146489	0.5057383	0.2794247	0.3925815	up
A_19_P00322339	LINC00707	-2.596094	-1.651333	-2.755791	0.9447615	-0.159697	0.3925323	up
A_22_P00004698	LOC100287098	0.7997098	1.2136116	1.1708422	0.4139018	0.3711324	0.3925171	up
A_21_P0000910	lnc-MIB2-1	2.120389	2.75098	2.2747307	0.6305909	0.1543417	0.3924663	up
A_23_P8848	INTS9	2.646881	3.1513143	2.927287	0.5044332	0.280406	0.3924196	up
A_24_P85158	SIRT3	1.8712254	2.3383985	2.1881952	0.4671731	0.3169699	0.3920715	up
A_23_P128067	RPL41	10.276671	10.71418	10.623259	0.4375086	0.3465872	0.3920479	up
A_24_P159648	BAIAP2	2.8514624	3.3329997	3.153984	0.4815373	0.3025217	0.3920295	up
A_23_P418597	TNKS1BP1	0.2189937	0.7915454	0.43047	0.5725517	0.2114763	0.392014	up
A_23_P66767	GGT6	-0.374509	-0.065989	0.1008644	0.3085203	0.4753737	0.391947	up
A_23_P60376	EDF1	6.775955	7.2542253	7.0814705	0.4782701	0.3055153	0.3918927	up
A_22_P00000891	lnc-AHNAK-4	-1.631167	-1.144794	-1.333774	0.486373	0.2973933	0.3918831	up
A_33_P3284711	FBXL19-AS1	-3.214613	-2.820628	-2.824853	0.3939848	0.3897598	0.3918723	up
A_23_P117980	KATNB1	-0.010441	0.2740393	0.4883995	0.2844806	0.4988408	0.3916607	up
A_22_P00015127	lnc-SNX27-2	-2.944313	-2.544211	-2.561107	0.4001014	0.3832057	0.3916535	up
A_23_P217068	RPL12	9.762216	10.210955	10.096611	0.4487391	0.3343954	0.3915672	up
A_23_P320717	COG4	1.2000623	1.6005015	1.5826664	0.4004393	0.3826041	0.3915217	up
A_23_P130352	KCTD1	-0.873368	-0.554438	-0.409359	0.3189302	0.4640093	0.3914697	up
A_23_P24375	OTUB1	2.925706	3.2302508	3.403987	0.3045449	0.478281	0.391413	up
A_23_P55802	AP1M1	2.1805239	2.7062602	2.437583	0.5257363	0.2570591	0.3913977	up
A_33_P3261869	NPM2	-1.061615	-0.551225	-0.789372	0.5103893	0.2722421	0.3913157	up

A_33_P3356004	UCKL1-AS1	-2.906417	-2.248054	-2.782277	0.6583633	0.1241403	0.3912518	up
A_33_P3249046	CLDN2	-2.75252	-2.516564	-2.206044	0.2359562	0.5464766	0.3912164	up
A_33_P3289167	ZBTB32	-1.47928	-1.210754	-0.965394	0.2685261	0.5138865	0.3912063	up
A_23_P140698	DNAJC17	0.0947542	0.3253388	0.6462593	0.2305846	0.5515051	0.3910449	up
A_23_P48977	MRPS11	5.158084	5.566962	5.5312805	0.4088779	0.3731966	0.3910372	up
A_22_P00024242	NDUFB2-AS1	-0.713055	-0.325385	-0.318782	0.38767	0.3942733	0.3909717	up
A_33_P3417944	HDAC11	-1.214948	-0.893622	-0.754497	0.3213253	0.4604507	0.390888	up
A_23_P31489	URGCP	1.7146168	2.0643392	2.1465988	0.3497224	0.431982	0.3908522	up
A_23_P208540	NDUFA3	6.8838844	7.2558455	7.293557	0.3719611	0.4096727	0.3908169	up
A_23_P315122	EMX1	0.9284306	1.224163	1.4142609	0.2957325	0.4858303	0.3907814	up
A_23_P168788	PLOD3	5.451788	5.8475375	5.8374777	0.3957496	0.3856897	0.3907197	up
A_33_P3312802	RPL13AP17	0.4242816	0.9042401	0.7254486	0.4799585	0.301167	0.3905628	up
A_22_P00004849	lnc-CYP19A1-1	-1.931205	-1.522499	-1.558807	0.4087062	0.3723974	0.3905518	up
A_22_P00007505	lnc-GYPA-1	-2.280997	-1.556911	-2.224007	0.7240868	0.0569904	0.3905386	up
A_33_P3411204	GCNT2	-2.377778	-2.117676	-1.857126	0.2601018	0.5206523	0.3903771	up
A_33_P3222139	SREBF1	-0.054781	0.3237276	0.3474073	0.378509	0.4021888	0.3903489	up
A_23_P23296	PKP1	0.5260248	0.986752	0.8459864	0.4607272	0.3199616	0.3903444	up
A_32_P148726	LOC728673	-2.271634	-1.574046	-2.188649	0.697588	0.0829849	0.3902864	up
A_24_P356453	COA3	4.5920105	5.0492597	4.915169	0.4572492	0.3231583	0.3902037	up
A_22_P00005789	lnc-EPB41-2	-0.482408	-0.124355	-0.060415	0.3580527	0.4219928	0.3900228	up
A_33_P3365732	TNKS1BP1	0.1450405	0.6940393	0.3760552	0.5489988	0.2310147	0.3900068	up
A_23_P158277	TMCO4	0.5751658	1.013421	0.9168825	0.4382553	0.3417168	0.389986	up
A_33_P3363620	TMEM91	-2.284797	-1.658135	-2.131496	0.6266618	0.1533017	0.3899818	up
A_23_P416686	GPR137	-0.168048	0.2572427	0.1865435	0.4252911	0.3545919	0.3899415	up
A_23_P316960	GRINA	2.593028	2.8606153	3.104967	0.2675872	0.5119391	0.3897631	up
A_22_P00000959	lnc-AL020996.1-1	-3.388248	-2.977231	-3.019786	0.4110177	0.3684619	0.3897398	up
A_23_P79999	ENTPD6	3.685419	4.1602283	3.9898787	0.4748092	0.3044596	0.3896344	up
A_23_P200976	HYI	2.2771273	2.816708	2.516635	0.5395808	0.2395077	0.3895442	up
A_33_P3417437	CD164L2	0.0733938	0.458992	0.4666657	0.3855982	0.3932719	0.3894351	up
A_24_P292964	CDK5RAP3	2.4507847	2.8603215	2.8200502	0.4095368	0.3692656	0.3894012	up
A_33_P3359012	DUSP8	2.8997402	3.4279628	3.1502151	0.5282226	0.2504749	0.3893487	up
A_32_P206839	LOC100288911	-0.235348	-0.028764	0.3366013	0.2065845	0.5719495	0.389267	up
A_24_P179903	AK2	5.5095882	6.0268054	5.7708797	0.5172172	0.2612915	0.3892543	up
A_23_P168403	KCNH2	-0.888458	-0.479564	-0.518873	0.4088941	0.3695846	0.3892393	up
A_22_P00023038	JAG2	1.85042	2.325091	2.1542187	0.4746709	0.3037987	0.3892348	up
A_23_P40347	HM13	4.560423	5.022283	4.876913	0.4618602	0.3164902	0.3891752	up
A_33_P3268472	CTSC	2.0522366	2.306704	2.5759249	0.2544675	0.5236883	0.3890779	up
A_22_P00011688	PDGFRB	-1.763689	-1.202697	-1.546628	0.5609918	0.2170606	0.3890262	up
A_23_P32125	PMPCA	4.3417587	4.6696134	4.791689	0.3278546	0.4499302	0.3888924	up
A_22_P00023121	lnc-RP11-327F22.5.1-4	-3.309416	-2.977673	-2.863558	0.331743	0.4458573	0.3888002	up
A_23_P77568	EIF3C	9.143289	9.597776	9.466371	0.4544878	0.323082	0.3887849	up
A_33_P3409062	TYROBP	-1.337245	-0.697143	-1.199875	0.6401024	0.1373696	0.388736	up
A_33_P3211520	SNAP47	1.375257	1.5991211	1.9288034	0.2238641	0.5535464	0.3887052	up
A_22_P00021185	lnc-RFC5-1	-2.012928	-1.624221	-1.624243	0.3887067	0.3886852	0.388696	up
A_24_P79241	UBOX5	1.1257701	1.4058294	1.6229916	0.2800593	0.4972215	0.3886404	up
A_23_P95851	TUBAL3	-3.261063	-2.989004	-2.755977	0.2720587	0.5050855	0.3885721	up
A_23_P38505	CXCL16	1.6716108	1.9516692	2.1686535	0.2800584	0.4970427	0.3885505	up
A_19_P00315716	SNORA71A	0.5159864	0.998446	0.8104177	0.4824596	0.2944312	0.3884454	up
A_33_P3296366	PODXL2	0.2002783	0.4484277	0.728868	0.2481494	0.5285897	0.3883696	up
A_24_P89426	APOM	-0.890212	-0.244702	-0.759067	0.6455097	0.131145	0.3883274	up
A_23_P41292	CTBP1	1.4064593	1.829042	1.7603436	0.4225826	0.3538842	0.3882334	up
A_23_P35399	ANXA11	5.782648	6.384605	5.957075	0.6019568	0.174427	0.3881919	up
A_23_P5339	TMEM177	4.100916	4.307367	4.670824	0.2064509	0.5699081	0.3881795	up
A_33_P3296577	PRRC2B	2.4360142	2.9677386	2.6805363	0.5317245	0.2445221	0.3881233	up
A_23_P211326	CECR2	-1.260894	-0.743457	-1.002085	0.5174365	0.2588086	0.3881226	up

A_33_P3219475	MSANTD3-TMEFF1	-3.359085	-2.694576	-3.247417	0.6645086	0.1116681	0.3880884	up
A_24_P161959	TMEM179B	2.3674707	2.693108	2.8178844	0.3256373	0.4504137	0.3880255	up
A_24_P91310	PSMC4	4.255685	4.6537385	4.6335945	0.3980537	0.3779097	0.3879817	up
A_23_P35912	CASP4	5.5894356	6.01796	5.936864	0.4285245	0.3474283	0.3879764	up
A_23_P70127	TMED9	3.75148	4.0314736	4.247385	0.2799935	0.4959049	0.3879492	up
A_33_P3274691	SCART1	-2.925661	-2.566766	-2.508658	0.3588951	0.4170024	0.3879488	up
A_24_P344711	AGPAT3	2.2899637	2.8132796	2.5425243	0.5233159	0.2525606	0.3879383	up
A_23_P312344	RBM19	1.8060856	2.3339238	2.0540104	0.5278382	0.2479248	0.3878815	up
A_32_P143000	FAM189A1	-2.304597	-1.755494	-2.077941	0.5491037	0.2266569	0.3878803	up
A_21_P0011921	XLOC_l2_008040	-3.240989	-2.722263	-2.984175	0.5187259	0.2568138	0.3877698	up
A_23_P120316	MTHFD2	4.8542233	5.6226554	4.8611355	0.7684321	0.0069122	0.3876722	up
A_22_P00000382	KRT73-AS1	-1.718243	-1.18136	-1.479845	0.5368834	0.2383986	0.387641	up
A_33_P3349637	PCDH1	7.5058374	7.868444	7.9185057	0.3626065	0.4126682	0.3876374	up
A_21_P0005785	lnc-ERICH1-5	3.1740122	3.5921025	3.5311937	0.4180903	0.3571816	0.3876359	up
A_33_P3309799	TRIM10	-1.178699	-0.83778	-0.744423	0.340919	0.4342756	0.3875973	up
A_23_P122443	HIST1H1C	5.50387	6.057649	5.7252827	0.5537791	0.2214127	0.3875959	up
A_33_P3313597	C20orf181	-2.95829	-2.680592	-2.460819	0.2776978	0.4974711	0.3875845	up
A_23_P432573	MRGPRF	-0.595589	-0.156014	-0.260039	0.4395747	0.3355503	0.3875625	up
A_33_P3252605	CYP2U1	-1.489676	-1.639212	-0.565151	-0.149536	0.9245253	0.3874946	up
A_23_P211522	SYNGR1	1.750638	2.1367383	2.1393986	0.3861003	0.3887606	0.3874304	up
A_23_P62731	MRPS14	4.047738	4.4013896	4.4689035	0.3536515	0.4211655	0.3874085	up
A_33_P3232798	RAB11FIP1	1.5317893	1.9936633	1.8447132	0.461874	0.3129239	0.387399	up
A_33_P3362636	CISD3	5.2940445	5.8075023	5.5553083	0.5134578	0.2612639	0.3873608	up
A_33_P3329078	HBG1	-0.690496	-0.156274	-0.450128	0.5342221	0.2403684	0.3872952	up
A_22_P00025049	ANXA4	-2.594777	-2.161072	-2.253957	0.4337053	0.3408201	0.3872627	up
A_23_P14559	RIPK3	-1.2176	-0.897593	-0.763113	0.3200073	0.4544878	0.3872476	up
A_21_P0010596	RNF223	4.114128	4.702952	4.299657	0.5888238	0.1855288	0.3871763	up
A_23_P75811	SLC3A2	4.489435	5.403809	4.3494005	0.9143739	-0.140035	0.3871696	up
A_33_P3268974	RECQL5	-0.043007	0.5587649	0.1294208	0.6017723	0.1724281	0.3871002	up
A_23_P102258	MRPL53	5.457556	5.8727365	5.81645	0.4151807	0.3588944	0.3870375	up
A_21_P0010421	lnc-PPARA-2	-0.595506	-0.207172	-0.209881	0.3883333	0.3856244	0.3869789	up
A_33_P3406505	ELL	-1.223465	-0.762946	-0.910036	0.4605188	0.3134294	0.3869741	up
A_23_P112429	RPL35	9.835858	10.235683	10.209938	0.3998251	0.3740797	0.3869524	up
A_23_P257593	LOH12CR1	1.1325326	1.5708437	1.468102	0.4383111	0.3355694	0.3869402	up
A_33_P3326927	ZNF19	-0.784192	-0.337669	-0.456922	0.4465227	0.3272705	0.3868966	up
A_24_P371194	MRPL53	3.6014404	4.020375	3.9562845	0.4189344	0.3548441	0.3868892	up
A_21_P0000255	SNORD30	-0.341028	0.0684209	0.0232806	0.4094486	0.3643084	0.3868785	up
A_23_P106773	SULT1A2	3.4734545	3.9288363	3.7917128	0.4553819	0.3182583	0.3868201	up
A_33_P3289352	PYGO2	-0.528585	-0.15274	-0.131002	0.3758454	0.3975835	0.3867145	up
A_22_P00010189	LOC101926937	-1.08421	-0.674518	-0.720555	0.4096918	0.3636551	0.3866735	up
A_24_P322741	IL10RB	1.9981127	2.479981	2.2894373	0.4818683	0.2913246	0.3865964	up
A_33_P3377649	PRSS16	-1.266502	-0.799979	-0.959907	0.4665232	0.3065949	0.386559	up
A_23_P16944	SDC1	6.284831	6.6758733	6.666833	0.3910422	0.3820019	0.3865221	up
A_23_P66719	DHRS13	0.4711595	0.93995	0.7752533	0.4687905	0.3040938	0.3864422	up
A_23_P127613	TRAPPC4	3.6747599	4.1380515	3.9843254	0.4632917	0.3095655	0.3864286	up
A_33_P3248644	QARS	4.9828234	5.4284625	5.309757	0.4456391	0.3269339	0.3862865	up
A_19_P00321511	SNHG5	4.5283766	5.2027698	4.6265135	0.6743932	0.0981369	0.3862651	up
A_23_P138271	ARL8A	4.939542	5.2509847	5.400625	0.3114429	0.4610834	0.3862631	up
A_23_P90444	RBM42	4.529911	5.0261073	4.8061085	0.4961963	0.2761974	0.3861969	up
A_21_P0006511	lnc-HTR2C-1	1.2294364	1.659493	1.5715055	0.4300566	0.3420692	0.3860629	up
A_22_P00015296	EIF3J-AS1	1.2160912	1.5871191	1.6170783	0.371028	0.4009872	0.3860076	up
A_23_P136413	MMP17	1.7500458	2.153504	2.1184568	0.4034581	0.3684111	0.3859346	up
A_22_P00021380	STMND1	-1.608622	-1.017678	-1.427803	0.5909433	0.1808186	0.385881	up
A_33_P3308050	EIF4E2	5.244257	5.504908	5.7551994	0.2606511	0.5109425	0.3857968	up
A_24_P118247	CTU2	0.8593316	1.2180333	1.2722163	0.3587017	0.4128847	0.3857932	up

A_24_P197964	TRIM14	1.9202604	1.9461904	2.6657734	0.0259299	0.745513	0.3857214	up
A_33_P3385775	ZNF703	1.4743953	2.1144156	1.605752	0.6400204	0.1313567	0.3856886	up
A_33_P3351914	NDUFA10	5.800866	6.173777	6.1991625	0.372911	0.3982964	0.3856037	up
A_23_P252981	ACE2	-3.21688	-3.103564	-2.559037	0.1133158	0.6578426	0.3855792	up
A_23_P111005	YIPF3	4.0524054	4.270654	4.6046963	0.2182488	0.5522909	0.3852699	up
A_24_P6083	CHCHD10	4.67311	4.9257665	5.190976	0.2526565	0.5178661	0.3852613	up
A_22_P00002490	C16orf46	-0.214768	0.1988478	0.1421361	0.4136157	0.356904	0.3852599	up
A_23_P207879	CARD14	1.0227828	1.5668674	1.2491317	0.5440846	0.2263489	0.3852167	up
A_23_P100315	DECR2	3.7498589	4.2236953	4.046279	0.4738364	0.2964201	0.3851283	up
A_21_P0012291	ZDHHC8	-1.646715	-1.28659	-1.236772	0.3601255	0.4099431	0.3850343	up
A_22_P00017617	lnc-WFIKKN2-1	-3.551498	-2.746499	-3.586585	0.8049989	-0.035087	0.3849561	up
A_23_P92895	RBM22	2.2764587	2.5968165	2.725706	0.3203578	0.4492474	0.3848026	up
A_33_P3414113	FAAHP1	-2.114473	-1.63312	-1.826227	0.4813538	0.2882462	0.3848	up
A_23_P144877	ATOX1	4.77866	5.221603	5.1050777	0.4429431	0.3264179	0.3846805	up
A_23_P124224	DHX8	2.608386	2.9838252	3.002304	0.3754392	0.393918	0.3846786	up
A_23_P365218	GPR110	-2.800824	-2.388624	-2.44405	0.4122	0.3567743	0.3844872	up
A_33_P3364060	HR	1.5433583	2.0327687	1.822783	0.4894104	0.2794247	0.3844175	up
A_33_P3214209	GUSBP1	-0.217958	0.2015147	0.1313663	0.4194727	0.3493242	0.3843985	up
A_21_P0003999	LOC257396	-1.10399	-0.772135	-0.667089	0.3318548	0.4369016	0.3843782	up
A_32_P90047	C4orf48	5.575179	5.8108115	6.1082153	0.2356324	0.5330362	0.3843343	up
A_33_P3404959	C2orf68	1.4246755	1.9130898	1.7048106	0.4884143	0.2801352	0.3842747	up
A_33_P3410372	NUDT10	-1.596154	-1.13303	-1.290744	0.4631248	0.3054099	0.3842673	up
A_23_P133474	GPX3	0.121469	0.7111664	0.2998471	0.5896974	0.1783781	0.3840377	up
A_23_P100355	PPP4C	3.2990198	3.6802497	3.6857662	0.3812299	0.3867464	0.3839881	up
A_23_P133068	ANK2	-2.78404	-2.159222	-2.640919	0.6248181	0.143121	0.3839696	up
A_24_P93206	TMEM179	-2.346779	-1.998269	-1.927416	0.3485107	0.419363	0.3839369	up
A_24_P7965	ESRRG	-3.193277	-2.842363	-2.776425	0.3509142	0.4168518	0.383883	up
A_24_P83075	LINC00205	-2.624549	-2.136582	-2.344785	0.4879668	0.2797642	0.3838655	up
A_23_P120660	RPS21	10.265805	10.675543	10.623732	0.4097376	0.3579264	0.383832	up
A_33_P3280213	CTSA	3.2155323	3.6742883	3.524331	0.458756	0.3087988	0.3837774	up
A_21_P0002492	FLJ42351	0.4105983	0.689414	0.8993201	0.2788158	0.4887219	0.3837688	up
A_23_P43255	FAM49B	3.7691698	4.1705604	4.1352234	0.4013906	0.3660536	0.3837221	up
A_21_P0000351	SCARNA22	5.3941727	5.840918	5.7148685	0.4467454	0.3206959	0.3837206	up
A_24_P239176	MUC4	4.044779	4.293897	4.562895	0.2491183	0.518116	0.3836172	up
A_23_P16538	ELL	1.5032802	2.1382976	1.6354866	0.6350174	0.1322064	0.3836119	up
A_24_P270144	CD63	8.13922	8.677807	8.367765	0.5385866	0.2285452	0.3835659	up
A_33_P3404411	MIPEP	3.3478413	3.6220431	3.8405285	0.2742019	0.4926872	0.3834446	up
A_24_P288323	DPP9	-2.594893	-2.845245	-1.577898	-0.250352	1.0169957	0.3833219	up
A_23_P24997	CDK4	5.4458084	5.9935517	5.6646633	0.5477433	0.2188549	0.3832991	up
A_24_P53150	TRAF7	3.954464	4.2146573	4.4607935	0.2601934	0.5063295	0.3832614	up
A_23_P115022	TMEM125	6.04813	6.4722705	6.390189	0.4241405	0.3420591	0.3830998	up
A_23_P7066	RPL9	8.503222	9.046534	8.725871	0.5433111	0.2226486	0.3829799	up
A_33_P3361546	TFAP2A	-2.316007	-1.775878	-2.090321	0.5401292	0.2256861	0.3829076	up
A_23_P24987	TSPAN31	0.7435484	1.281456	0.9713988	0.5379076	0.2278504	0.382879	up
A_24_P914495	MARK2	0.4153352	0.8262262	0.7700734	0.4108911	0.3547382	0.3828147	up
A_33_P3254708	ARHGAP40	-2.449258	-1.967991	-2.164907	0.4812667	0.2843511	0.3828089	up
A_32_P92399	COG8	4.5590134	5.101629	4.78187	0.5426154	0.2228565	0.382736	up
A_23_P328740	NEURL3	-0.841942	-0.327256	-0.591627	0.5146861	0.2503147	0.3825004	up
A_33_P3330549	SLC44A2	3.3129387	3.665403	3.7254286	0.3524642	0.4124899	0.3824771	up
A_22_P00013673	lnc-RP11-688I9.2.1-1	-1.828387	-1.589278	-1.302817	0.239109	0.5255694	0.3823392	up
A_33_P3317523	STMN1	5.2559204	5.6501966	5.626197	0.3942761	0.3702765	0.3822763	up
A_22_P00005058	lnc-DENND1A-4	-2.859611	-2.665312	-2.289485	0.1942992	0.5701268	0.382213	up
A_23_P170058	PSMB2	4.427679	4.7593427	4.860422	0.3316636	0.4327431	0.3822033	up
A_21_P0007948	lnc-AL359392.1-2	-0.390327	0.0601425	-0.076594	0.450469	0.3137326	0.3821008	up
A_23_P300150	NFATC1	0.9574809	1.3932347	1.2859173	0.4357538	0.3284364	0.3820951	up

A_33_P3224423	POLM	-1.161965	-0.81964	-0.740294	0.3423257	0.4216714	0.3819986	up
A_32_P52330	LOC113230	1.371561	1.8922777	1.6146903	0.5207167	0.2431293	0.381923	up
A_21_P0000513	VTRNA1-1	-1.947752	-1.494327	-1.63753	0.4534249	0.3102217	0.3818233	up
A_21_P0003242	lnc-EPHA6-1	-1.96972	-1.636341	-1.539566	0.3333793	0.4301543	0.3817668	up
A_33_P3290239	DUOXA1	-1.484111	-0.866725	-1.337989	0.6173854	0.146122	0.3817537	up
A_21_P0000321	SNORA31	2.8616953	3.4825196	3.0043335	0.6208243	0.1426382	0.3817313	up
A_23_P145330	CCHCR1	0.3092756	0.6953645	0.6866236	0.3860889	0.377348	0.3817184	up
A_23_P79816	EMILIN3	-0.847779	-0.394378	-0.537746	0.4534011	0.3100333	0.3817172	up
A_23_P36345	STARD10	2.7213774	3.095058	3.111125	0.3736806	0.3897476	0.3817141	up
A_24_P272917	USP17L25	-1.734453	-1.131728	-1.573767	0.6027246	0.160686	0.3817053	up
A_33_P3333800	NOXO1	0.4561405	0.7311735	0.9444714	0.275033	0.4883308	0.3816819	up
A_33_P3329508	LRRC27	-1.604881	-1.232375	-1.214055	0.3725057	0.3908262	0.3816659	up
A_23_P82296	GNB2	3.8583841	4.278297	4.2016144	0.4199128	0.3432303	0.3815715	up
A_19_P00812911	LINC01137	2.3025398	2.6835608	2.6844263	0.381021	0.3818865	0.3814538	up
A_23_P368934	FBRSL1	3.0920515	3.4361882	3.5107718	0.3441367	0.4187203	0.3814285	up
A_23_P50399	DCAF15	0.0638022	0.5747104	0.3156023	0.5109081	0.2518001	0.3813541	up
A_23_P129835	PPP1R1B	3.288743	3.5000882	3.8399773	0.2113452	0.5512343	0.3812897	up
A_23_P310	MARCKSL1	2.314703	2.6442256	2.747693	0.3295226	0.4329901	0.3812563	up
A_33_P3372501	PPP1R13B	-0.453562	0.0048199	-0.150006	0.4583817	0.303556	0.3809688	up
A_23_P254404	SNX33	-0.777682	-0.503296	-0.290202	0.2743855	0.4874797	0.3809326	up
A_33_P3353996	PPP1R3G	-2.447211	-2.147416	-1.985202	0.2997949	0.462009	0.3809019	up
A_33_P3211679	TSPAN11	0.1244974	0.4778128	0.5329509	0.3533154	0.4084535	0.3808844	up
A_23_P258246	DDB1	5.9297	6.352055	6.268976	0.4223552	0.3392763	0.3808157	up
A_23_P88848	DUS2	0.0998216	0.4404512	0.5206618	0.3406296	0.4208403	0.3807349	up
A_24_P273799	ZNF641	-0.325336	0.1826892	-0.072027	0.5080257	0.2533097	0.3806677	up
A_32_P227317	DDX11L2	-0.744264	-0.396989	-0.330248	0.3472743	0.4140158	0.380645	up
A_33_P3256785	CARM1	4.713704	5.012258	5.176403	0.2985539	0.4626989	0.3806264	up
A_32_P99347	FAM225B	-2.829892	-2.136796	-2.761847	0.6930962	0.0680449	0.3805705	up
A_23_P65427	PSME2	7.628124	7.9124427	8.10494	0.2843185	0.4768162	0.3805673	up
A_21_P0006277	lnc-ALDH1A1-3	-3.174825	-2.759562	-2.828957	0.4152627	0.3458684	0.3805655	up
A_22_P00002484	lnc-C16orf13-1	-1.617371	-1.265181	-1.208449	0.3521905	0.4089217	0.3805561	up
A_22_P00016230	CATIP-AS1	-1.255116	-0.78967	-0.959525	0.4654455	0.2955904	0.380518	up
A_23_P63038	P3H1	0.169219	0.5786796	0.520761	0.4094605	0.351542	0.3805013	up
A_23_P308673	TAOK2	-1.336964	-1.05071	-0.862259	0.2862539	0.4747047	0.3804793	up
A_23_P90311	TICAM1	4.095438	4.5142436	4.437563	0.4188056	0.3421249	0.3804653	up
A_33_P3220415	GALT	-1.337911	-0.779385	-1.135514	0.558526	0.2023974	0.3804617	up
A_32_P181103	GGCX	1.724123	2.2958493	1.9132519	0.5717263	0.1891289	0.3804276	up
A_23_P202708	MADD	2.368167	2.7571645	2.7399225	0.3889976	0.3717556	0.3803766	up
A_33_P3331687	GPSM1	3.2669106	3.7021842	3.5923443	0.4352737	0.3254337	0.3803537	up
A_23_P208847	SIRT6	0.9343009	1.3780475	1.2512331	0.4437466	0.3169322	0.3803394	up
A_21_P0000504	RNU6ATAC	0.9470844	1.2949677	1.3598313	0.3478832	0.4127469	0.3803151	up
A_24_P390928	TRAPPC6A	3.1804209	3.6785016	3.4429579	0.4980807	0.262537	0.3803089	up
A_23_P155376	CRELD1	1.7636623	2.1270576	2.1607876	0.3633952	0.3971252	0.3802602	up
A_23_P129246	PLEKHO2	1.0889835	1.3709049	1.5674901	0.2819214	0.4785066	0.380214	up
A_33_P3257460	DPM3	5.841714	6.0742707	6.3694715	0.2325568	0.5277576	0.3801572	up
A_33_P3363071	ITIH6	-2.336752	-2.011365	-1.901828	0.325387	0.4349241	0.3801556	up
A_23_P304450	GATA6	3.550686	3.9796615	3.8819256	0.4289756	0.3312397	0.3801076	up
A_23_P311358	ZNF282	-1.441827	-0.842182	-1.281286	0.5996451	0.1605411	0.3800931	up
A_23_P369983	FAM98C	3.390232	3.7466235	3.7940083	0.3563914	0.4037762	0.3800838	up
A_33_P3307660	KLHDC4	1.5822878	1.854341	2.0703106	0.2720532	0.4880228	0.380038	up
A_21_P0000199	HOXA6	-1.357789	-0.673453	-1.282178	0.6843352	0.0756102	0.3799727	up
A_23_P104563	CPT1A	2.9815397	3.5344234	3.1884155	0.5528836	0.2068758	0.3798797	up
A_33_P3367994	TAF8	1.9795446	2.6399417	2.07864	0.6603971	0.0990953	0.3797462	up
A_22_P00018324	SSPO	1.8711653	2.3013673	2.2004156	0.430202	0.3292503	0.3797262	up
A_24_P178148	LOC100129455	-2.488751	-2.331583	-1.886522	0.1571684	0.6022289	0.3796986	up
A_33_P3423820	ZC3H3	4.4733114	4.814195	4.8917465	0.3408837	0.4184351	0.3796594	up

A_21_P0000218	SNORD35A	0.5961275	1.0961957	0.8553243	0.5000682	0.2591968	0.3796325	up
A_22_P00020155	lnc-C10orf122-2	-3.18091	-2.804613	-2.798029	0.376297	0.3828809	0.379589	up
A_23_P385199	EPHA10	-3.187877	-2.861152	-2.755741	0.3267257	0.4321363	0.379431	up
A_23_P27133	KRT15	4.517894	4.8528466	4.941716	0.3349528	0.4238224	0.3793876	up
A_33_P3219100	MANBAL	2.7300644	2.9482675	3.2705832	0.2182031	0.5405188	0.3793609	up
A_33_P3299599	NKX2-5	7.5596943	8.082863	7.794977	0.5231686	0.2352829	0.3792257	up
A_23_P19352	CNPY3	2.3621826	2.9239287	2.5588503	0.5617461	0.1966677	0.3792069	up
A_33_P3629131	CDRT3	-0.532552	-0.272548	-0.034152	0.2600036	0.4984002	0.3792019	up
A_23_P168080	SKIV2L	0.3881483	0.904686	0.6299481	0.5165377	0.2417998	0.3791688	up
A_33_P3344673	PPP1R37	-0.912207	-0.355361	-0.710736	0.5568461	0.2014713	0.3791587	up
A_22_P00000322	LOC285191	-1.205605	-0.619607	-1.033417	0.5859976	0.1721878	0.3790927	up
A_23_P215479	CLIP2	2.8657808	3.0774217	3.4119062	0.2116408	0.5461254	0.3788831	up
A_33_P3229335	HIST3H2BB	1.0156455	1.3371906	1.4515882	0.3215451	0.4359427	0.3787439	up
A_24_P160440	MRPL55	5.90872	6.261472	6.3134127	0.3527522	0.4046927	0.3787224	up
A_24_P50753	NUDT4	2.9388866	3.2062364	3.428752	0.2673497	0.4898653	0.3786075	up
A_33_P3317628	PKP3	7.1924343	7.5014577	7.6406193	0.3090234	0.448185	0.3786042	up
A_21_P0000374	SNORD59B	-2.849062	-2.300774	-2.640157	0.5482886	0.2089052	0.3785969	up
A_33_P3298535	ROMO1	6.979595	7.2384286	7.47795	0.2588334	0.4983549	0.3785942	up
A_22_P00003466	MMP25-AS1	-3.092391	-2.906414	-2.521269	0.1859777	0.5711219	0.3785498	up
A_33_P3724155	DERL3	4.1601725	4.57739	4.4998302	0.4172177	0.3396578	0.3784378	up
A_23_P64560	PGAP2	0.7434969	1.0603232	1.1834054	0.3168263	0.4399085	0.3783674	up
A_23_P86493	LBX1	7.7447987	8.235914	8.010228	0.4911156	0.2654295	0.3782725	up
A_22_P00008495	NAPSB	-0.673642	-0.253508	-0.337236	0.4201336	0.3364053	0.3782694	up
A_23_P200252	NTPCR	2.4476566	2.789473	2.8623543	0.3418164	0.4146977	0.378257	up
A_23_P28015	ZNF558	-0.461716	0.0056705	-0.172702	0.4673863	0.2890139	0.3782001	up
A_33_P3260575	CERCAM	0.8412194	1.4444757	0.9942346	0.6032562	0.1530151	0.3781357	up
A_32_P110243	RPS20P27	9.012004	9.541836	9.238407	0.5298319	0.2264032	0.3781176	up
A_23_P101013	TMC6	2.979741	3.5314727	3.1842232	0.5517316	0.2044821	0.3781068	up
A_23_P133923	GPANK1	4.0719757	4.6168656	4.2826138	0.5448899	0.2106381	0.377764	up
A_22_P00020716	lnc-ZNF852-2	-2.975803	-2.648065	-2.548078	0.3277378	0.4277248	0.3777313	up
A_33_P3289780	C14orf2	3.7836666	4.0798345	4.242791	0.2961679	0.4591246	0.3776462	up
A_23_P23194	PINK1	1.2090278	1.6805906	1.4926782	0.4715629	0.2836504	0.3776066	up
A_33_P3263277	EEF1A1	9.490726	9.992138	9.744455	0.5014114	0.2537289	0.3775702	up
A_21_P0005477	lnc-NPVF-4	-1.571475	-1.189339	-1.198604	0.3821354	0.3728709	0.3775032	up
A_21_P0000244	SNORD16	2.8042498	3.1635518	3.1998663	0.359302	0.3956165	0.3774593	up
A_24_P930741	EPHA10	-0.278171	0.1695809	0.0289578	0.447752	0.3071289	0.3774405	up
A_23_P47800	DIABLO	4.5727615	4.941664	4.9587145	0.3689027	0.385953	0.3774278	up
A_21_P0013365	ZNF815P	-3.127253	-2.735296	-2.76439	0.391957	0.3628628	0.3774099	up
A_23_P62371	TAZ	-1.449058	-0.87604	-1.267285	0.5730176	0.1817722	0.3773949	up
A_22_P00003286	lnc-CAMK1G-1	-2.217431	-1.789287	-1.890792	0.4281445	0.3266387	0.3773916	up
A_23_P93658	ASIC3	-2.99576	-2.027614	-3.209213	0.9681463	-0.213453	0.3773467	up
A_23_P169409	RPP25L	5.8752384	6.2345366	6.2704315	0.3592982	0.3951931	0.3772457	up
A_33_P3390853	LINC01420	5.394786	5.657579	5.8864403	0.2627931	0.4916544	0.3772237	up
A_33_P3831730	lnc-NDUFA4-2	-0.283955	0.092299	0.0941105	0.3762541	0.3780656	0.3771598	up
A_23_P106562	GALNS	0.7058821	1.0653076	1.1007619	0.3594255	0.3948798	0.3771527	up
A_21_P0006309	lnc-AL162389.1-1	-3.378164	-2.957334	-3.044727	0.42083	0.333437	0.3771335	up
A_33_P3260307	CARHSP1	3.2001553	3.451839	3.7025414	0.2516837	0.5023861	0.3770349	up
A_22_P00002388	ATP11A	-1.613501	-1.168666	-1.30437	0.4448347	0.3091302	0.3769825	up
A_23_P68155	IFIH1	3.0215454	3.2559028	3.541031	0.2343574	0.5194855	0.3769214	up
A_23_P6891	EIF1B	1.9967384	2.265307	2.481822	0.2685685	0.4850836	0.3768261	up
A_32_P18258	TALDO1	6.561736	7.1037097	6.7733784	0.5419736	0.2116423	0.3768079	up
A_23_P411922	PRPF40B	-0.802726	-0.446955	-0.405076	0.3557706	0.3976502	0.3767104	up
A_33_P3373329	TDRD1	-3.386787	-2.887079	-3.133121	0.4997082	0.2536662	0.3766872	up
A_23_P168864	ZNF16	0.2105398	0.6600118	0.5142822	0.449472	0.3037424	0.3766072	up
A_23_P36187	SYT8	2.3862467	2.7285104	2.7971678	0.3422637	0.4109211	0.3765924	up
A_22_P00022944	lnc-PBX1-2	-2.903485	-2.677933	-2.376033	0.2255514	0.5274515	0.3765014	up

A_33_P3364964	LOC100129596	-0.169576	0.4884048	-0.074582	0.6579804	0.0949941	0.3764873	up
A_22_P00015273	lnc-SPATA17-2	-2.852819	-2.405958	-2.546743	0.4468608	0.3060756	0.3764682	up
A_23_P45945	EIF1	10.250314	10.740728	10.512766	0.4904146	0.2624521	0.3764334	up
A_22_P00018205	lnc-ZNF643-1	-2.54162	-2.202668	-2.127735	0.3389518	0.4138856	0.3764187	up
A_23_P356484	RPS10	9.407052	9.8190975	9.747768	0.4120455	0.3407164	0.3763809	up
A_22_P00017113	lnc-UAP1L1-1	0.0562367	0.4358659	0.4293585	0.3796291	0.3731217	0.3763754	up
A_33_P3775741	RPL32P3	7.917001	8.372623	8.214125	0.4556227	0.2971239	0.3763733	up
A_33_P3270802	CPSF3L	5.1875067	5.59465	5.532899	0.4071431	0.3453922	0.3762677	up
A_23_P9392	C9orf114	0.7621279	1.0082083	1.2685771	0.2460804	0.5064492	0.3762648	up
A_23_P100326	NPRL3	4.2609186	4.720817	4.5534897	0.4598985	0.2925711	0.3762348	up
A_33_P3281710	LOC79999	-3.391605	-3.057386	-2.973374	0.3342187	0.4182313	0.376225	up
A_24_P359117	GPATCH8	1.8064365	2.2073598	2.1576319	0.4009233	0.3511953	0.3760593	up
A_23_P127367	POLD4	4.7704353	5.1715055	5.1213236	0.4010701	0.3508883	0.3759792	up
A_23_P10873	TLR1	-1.643429	-1.185973	-1.348957	0.4574556	0.2944722	0.3759639	up
A_32_P9842	MTMR14	4.821126	5.22308	5.1708145	0.4019542	0.3496885	0.3758214	up
A_21_P0003658	lnc-USP38-1	-0.778321	-0.468819	-0.336324	0.3095026	0.4419975	0.3757501	up
A_23_P124837	LRP1	-0.414729	0.2080584	-0.286035	0.622787	0.1286941	0.3757405	up
A_23_P31840	EEF1D	7.933173	8.468618	8.14914	0.5354452	0.2159672	0.3757062	up
A_23_P417383	ASPRV1	0.2809157	0.4419675	0.8711352	0.1610518	0.5902195	0.3756356	up
A_33_P3324495	ZFP41	2.457302	2.7871728	2.8784637	0.3298707	0.4211617	0.3755162	up
A_23_P53329	FLJ13224	-3.043814	-2.503606	-2.833035	0.5402081	0.2107792	0.3754936	up
A_23_P115955	MRPL21	6.4373684	6.761314	6.864259	0.3239455	0.4268904	0.3754179	up
A_33_P3364808	PTPRC	-2.030205	-1.568699	-1.740988	0.4615059	0.2892165	0.3753612	up
A_23_P41716	GNB2L1	7.5441837	8.055368	7.783395	0.5111847	0.2392111	0.3751979	up
A_23_P107855	FIZ1	0.1594029	0.5397987	0.5292883	0.3803959	0.3698854	0.3751407	up
A_33_P3262555	MEX3D	3.1278906	3.6252465	3.3807173	0.4973559	0.2528267	0.3750913	up
A_24_P48248	C17orf53	-1.005122	-0.458439	-0.801987	0.5466828	0.203135	0.3749089	up
A_23_P215454	ELN	-3.005046	-2.66917	-2.591461	0.3358765	0.4135852	0.3747308	up
A_23_P117683	HYPK	5.639555	5.863726	6.164789	0.2241712	0.5252342	0.3747027	up
A_33_P3424803	HLA-C	8.210633	8.639734	8.530772	0.429101	0.3201389	0.37462	up
A_33_P3409302	COX15	-0.540913	-0.181305	-0.15133	0.3596077	0.3895836	0.3745957	up
A_23_P3849	TRAP1	3.6575003	4.0163684	4.0477	0.3588681	0.3901997	0.3745339	up
A_23_P118894	PRR15L	5.5293674	6.068545	5.7391596	0.5391774	0.2097921	0.3744848	up
A_33_P3411612	TMEM221	-2.310409	-1.879413	-1.9925	0.4309964	0.3179088	0.3744526	up
A_23_P18317	SLC41A3	4.3818417	4.719195	4.793358	0.3373532	0.4115162	0.3744347	up
A_23_P153897	GNG7	-2.175011	-1.769878	-1.831308	0.4051333	0.3437028	0.374418	up
A_22_P00001892	LOC101927583	-2.613287	-2.200744	-2.277043	0.4125428	0.3362439	0.3743933	up
A_21_P0010098	lnc-C20orf166-2	-1.558805	-1.158399	-1.210468	0.4004064	0.3483372	0.3743718	up
A_21_P0011041	XLOC_l2_002910	9.046363	9.597948	9.243424	0.5515852	0.1970615	0.3743234	up
A_33_P3398998	C2orf50	0.1397352	0.4302607	0.5978437	0.2905254	0.4581084	0.3743169	up
A_21_P0006510	lnc-PRPS1-1	-2.41898	-1.738151	-2.351254	0.6808286	0.0677257	0.3742771	up
A_19_P00320966	LINC00861	1.5565042	1.9895654	1.871912	0.4330611	0.3154078	0.3742344	up
A_33_P3247403	TOR3A	0.6893883	1.0309124	1.0962429	0.3415241	0.4068546	0.3741894	up
A_23_P59045	HIST1H2AE	3.557621	3.9594188	3.9039564	0.4017978	0.3463354	0.3740666	up
A_32_P57854	OST4	6.6992226	7.040069	7.106473	0.3408465	0.4072504	0.3740485	up
A_23_P218646	TNFRSF6B	-2.987407	-2.574603	-2.65219	0.4128046	0.3352175	0.374011	up
A_33_P3262089	OR8G2	-2.44046	-2.423429	-1.709526	0.0170317	0.7309341	0.3739829	up
A_22_P00022747	lnc-MTERF-1	-2.00238	-1.276465	-1.98036	0.7259154	0.0220203	0.3739679	up
A_22_P00005038	lnc-DDX60L-1	-3.024073	-2.53103	-2.76933	0.4930437	0.2547431	0.3738934	up
A_32_P86150	CTRB2	2.6754246	3.1719089	2.9264498	0.4964843	0.2510252	0.3737547	up
A_33_P3341586	SIL1	1.4760504	1.799264	1.900343	0.3232136	0.4242926	0.3737531	up
A_33_P3306153	KIAA1841	-1.521908	-0.770033	-1.526571	0.7518749	-0.004663	0.3736057	up
A_22_P00023865	lnc-FKBP3-3	2.2528543	2.761087	2.4917374	0.5082326	0.238883	0.3735578	up
A_22_P00002166	FAM229A	5.2215414	5.8102546	5.379794	0.5887132	0.1582527	0.3734829	up
A_23_P15603	MRM1	0.9844556	1.2277613	1.4880996	0.2433057	0.503644	0.3734748	up
A_23_P400515	KIF17	-3.238935	-2.968372	-2.762642	0.2705627	0.4762921	0.3734274	up

A_23_P118105	NDUFB10	6.5168486	6.880277	6.9001102	0.3634286	0.3832617	0.3733451	up
A_33_P3344039	RABL2A	-0.693213	-0.274279	-0.365494	0.4189344	0.3277192	0.3733268	up
A_23_P112478	NUDT2	2.3520365	2.6953263	2.7552538	0.3432899	0.4032173	0.3732536	up
A_24_P248053	TOP1MT	5.224245	5.6816516	5.513295	0.4574065	0.2890501	0.3732283	up
A_23_P30474	WDR70	1.8899097	2.3068728	2.2193727	0.4169631	0.329463	0.3732131	up
A_22_P00014667	lnc-SLC23A1-1	-3.102964	-2.791865	-2.667643	0.3110993	0.4353211	0.3732102	up
A_22_P00016259	LOC100131564	-0.89613	-0.655483	-0.390371	0.2406468	0.5057583	0.3732026	up
A_23_P67748	RAB11B	-0.674289	-0.260739	-0.341443	0.4135499	0.3328466	0.3731983	up
A_23_P107653	ETFB	7.210701	7.6034856	7.5641365	0.3927846	0.3534355	0.3731101	up
A_24_P322635	ELMO2	1.418715	1.8398194	1.7438178	0.4211044	0.3251028	0.3731036	up
A_23_P215900	SCARA3	2.0284777	2.2548375	2.5483027	0.2263598	0.519825	0.3730924	up
A_33_P3328026	CDK19	2.524807	3.0615144	2.734147	0.5367074	0.2093401	0.3730238	up
A_21_P0010679	XLOC_l2_001362	-0.887519	-0.598034	-0.43097	0.2894845	0.4565487	0.3730166	up
A_32_P208178	RPS3A	9.340586	9.795504	9.631585	0.4549179	0.2909994	0.3729587	up
A_33_P3422931	MRPS34	4.5315495	4.9031243	4.9057264	0.3715749	0.374177	0.3728759	up
A_32_P204218	FAM180A	-1.71429	-1.347657	-1.335199	0.3666325	0.3790908	0.3728616	up
A_21_P0014853	GS1-24F4.2	1.3357501	1.805778	1.6114178	0.4700279	0.2756677	0.3728478	up
A_23_P107166	ACBD4	-1.852412	-1.390032	-1.569147	0.4623804	0.2832656	0.372823	up
A_23_P337550	OTUD5	3.4793549	4.0269427	3.677186	0.5475879	0.1978312	0.3727095	up
A_33_P3311285	LMNA	6.3971233	6.8444324	6.6950617	0.447309	0.2979384	0.3726237	up
A_33_P3315331	CNTD2	-2.249339	-1.840073	-1.913362	0.409266	0.3359771	0.3726215	up
A_24_P598406	RNF216P1	3.1741428	3.3486438	3.7446213	0.1745009	0.5704784	0.3724897	up
A_22_P00020043	HIF1A-AS1	-0.141808	0.3144283	0.146872	0.4562359	0.2886796	0.3724578	up
A_23_P89931	ZNF574	3.1625996	3.5637822	3.5062618	0.4011827	0.3436623	0.3724225	up
A_23_P152024	CSK	1.640626	2.0420613	1.9838467	0.4014354	0.3432207	0.372328	up
A_23_P205686	PSEN1	2.5886345	3.1113954	2.8103905	0.5227609	0.221756	0.3722584	up
A_23_P68866	UQCR10	7.83562	8.19017	8.225568	0.3545504	0.3899479	0.3722491	up
A_24_P74160	SNRPD2	6.9461565	7.21053	7.4262724	0.2643733	0.4801159	0.3722446	up
A_24_P148499	CASP8	-2.04501	-1.906855	-1.438835	0.138155	0.6061745	0.3721647	up
A_22_P00005940	lnc-EXOC3-1	-0.330711	0.0203214	0.0625491	0.3510323	0.39326	0.3721461	up
A_33_P3364051	TOP3B	-0.242587	0.2303577	0.0284987	0.4729443	0.2710853	0.3720148	up
A_23_P138058	NOC2L	3.920763	4.2226357	4.3626738	0.3018727	0.4419107	0.3718917	up
A_23_P214587	TRIM26	2.1089563	2.451119	2.5105743	0.3421626	0.401618	0.3718903	up
A_21_P0000347	SNORA76C	3.760046	4.0743318	4.189192	0.3142858	0.4291458	0.3717158	up
A_21_P0013352	ZNF767P	-3.057972	-2.256867	-3.1158	0.801105	-0.057828	0.3716385	up
A_23_P501996	UBE2V1	4.779627	5.080485	5.2217903	0.300858	0.4421635	0.3715107	up
A_21_P0003596	lnc-DTHD1-2	-2.165165	-1.82193	-1.765533	0.343235	0.3996325	0.3714337	up
A_23_P155147	ZBED4	1.2736444	1.7117686	1.5783458	0.4381242	0.3047013	0.3714128	up
A_23_P31686	CCAR2	0.1561375	0.2750011	0.7800503	0.1188636	0.6239128	0.3713882	up
A_23_P42144	PEX6	-0.279141	0.1469498	0.037323	0.4260907	0.316464	0.3712773	up
A_23_P170534	FUT7	-2.227566	-1.82148	-1.891179	0.406086	0.3363872	0.3712366	up
A_21_P0009320	LOC101929494	2.624362	3.085216	2.9059544	0.4608541	0.2815924	0.3712232	up
A_23_P8095	RNF5	3.1736298	3.7352319	3.3544693	0.5616021	0.1808395	0.3712208	up
A_24_P67699	RPL23A	9.850146	10.260222	10.1819	0.4100752	0.3317537	0.3709145	up
A_24_P147461	SERPINB8	-0.586652	-0.028012	-0.403644	0.55864	0.1830082	0.3708241	up
A_23_P23584	CTNNBIP1	3.1466923	3.601338	3.4336576	0.4546456	0.2869654	0.3708055	up
A_33_P3318187	CENPBD1	1.0317831	1.4112978	1.3937917	0.3795147	0.3620086	0.3707616	up
A_24_P292020	POLR2J	4.77971	5.0831704	5.217767	0.3034606	0.438057	0.3707588	up
A_21_P0001125	lnc-MUTYH-1	-2.15809	-1.946256	-1.628721	0.2118335	0.5293689	0.3706012	up
A_21_P0000785	DNM3OS	0.0200429	0.4015832	0.3793683	0.3815403	0.3593254	0.3704329	up
A_33_P3229181	SLC12A9	4.1846113	4.5984864	4.511095	0.4138751	0.3264837	0.3701794	up
A_22_P00012589	lnc-PTPN23-1	0.4312663	0.8459678	0.756743	0.4147015	0.3254767	0.3700891	up
A_21_P0010595	FBXO25	0.6993294	1.0592742	1.0793757	0.3599448	0.3800464	0.3699956	up
A_23_P115036	IPO13	0.6054792	1.0772457	0.8736081	0.4717665	0.2681289	0.3699477	up
A_24_P154948	GARS	2.911787	3.40301	3.1604586	0.4912229	0.2486715	0.3699472	up
A_21_P0000227	SNORD35B	0.2516379	0.7009664	0.5421	0.4493284	0.290462	0.3698952	up

A_22_P00022956	lnc-INPPL1-1	-2.210457	-2.000475	-1.680722	0.2099824	0.5297351	0.3698588	up
A_33_P3346403	PTMA	9.167248	9.509928	9.564048	0.34268	0.3968	0.36974	up
A_23_P8571	SSC4D	0.059279	0.5570631	0.3009453	0.4977841	0.2416663	0.3697252	up
A_23_P73142	TACO1	2.1896753	2.486197	2.632535	0.2965217	0.4428597	0.3696907	up
A_22_P00023949	RUVBL1-AS1	1.384512	1.7138643	1.7945151	0.3293524	0.4100032	0.3696778	up
A_23_P501372	MIEF2	1.3112278	1.8473248	1.514286	0.5360971	0.2030582	0.3695776	up
A_24_P106542	RSPO3	-2.035485	-1.670458	-1.661456	0.3650265	0.3740292	0.3695278	up
A_19_P00809682	lnc-FAM105B-1	6.3933115	6.7758946	6.749507	0.3825831	0.3561955	0.3693893	up
A_21_P0011035	LOC101928054	-1.790096	-1.371068	-1.470409	0.4190283	0.3196869	0.3693576	up
A_23_P138253	CHTOP	3.8886833	4.3925185	4.1232033	0.5038352	0.23452	0.3691776	up
A_22_P00024782	lnc-CLDN5-1	-0.146887	0.2429366	0.2015624	0.3898234	0.3484492	0.3691363	up
A_23_P17880	DNAL4	0.5079236	0.9490471	0.8049073	0.4411235	0.2969837	0.3690536	up
A_24_P125881	DGCR2	6.322692	6.8294377	6.554038	0.5067458	0.2313461	0.369046	up
A_24_P151498	PRMT2	2.057067	2.4078803	2.4442348	0.3508134	0.3871679	0.3689907	up
A_23_P217088	AK1	4.1153374	4.4163985	4.5521936	0.3010612	0.4368563	0.3689587	up
A_33_P3442605	LOC389641	-2.80741	-2.568068	-2.309101	0.2393415	0.4983084	0.368825	up
A_22_P00016489	LOC102724715	-1.539793	-1.288589	-1.053783	0.251204	0.4860101	0.368607	up
A_22_P00016330	lnc-TMEM151A-1	2.6994371	3.263372	2.8725395	0.5639348	0.1731024	0.3685186	up
A_33_P3359223	C9orf173	1.2901864	1.7695193	1.5477381	0.4793329	0.2575517	0.3684423	up
A_21_P0010489	ZNF605	-1.486066	-1.033029	-1.202348	0.4530377	0.2837181	0.3683779	up
A_33_P3295738	CNGB1	-0.362221	0.157208	-0.14502	0.5194292	0.2172017	0.3683155	up
A_33_P3267562	VAX2	-1.742635	-1.277175	-1.471601	0.4654594	0.2710338	0.3682466	up
A_24_P72479	ARPC1A	5.0548697	5.5647664	5.281451	0.5098968	0.2265816	0.3682392	up
A_33_P3270384	PPP1R14B	7.472048	7.9553294	7.725212	0.4832816	0.2531643	0.368223	up
A_33_P3304688	TNAP	-2.486298	-2.016612	-2.219573	0.4696868	0.2667258	0.3682063	up
A_23_P357101	APOBEC3F	1.0664816	1.3187385	1.5506077	0.2522569	0.4841261	0.3681915	up
A_23_P7941	MRPL2	1.5976992	1.9880605	1.9435587	0.3903613	0.3458595	0.3681104	up
A_23_P34568	ADPRHL2	1.9665565	2.218491	2.4504356	0.2519345	0.4838791	0.3679068	up
A_21_P0007066	lnc-SRGN-1	-1.607118	-1.087278	-1.391161	0.5198398	0.2159567	0.3678982	up
A_24_P338992	EDC3	1.861949	2.1472096	2.3124495	0.2852607	0.4505005	0.3678806	up
A_23_P321320	STK11IP	3.7129583	4.108024	4.053588	0.3950658	0.3406296	0.3678477	up
A_33_P3238250	F11R	3.4655552	3.8678174	3.798893	0.4022622	0.3333378	0.3678	up
A_33_P3269203	SERPINH1	5.8067427	6.1165786	6.2324514	0.3098359	0.4257088	0.3677723	up
A_32_P207124	CT47A11	2.4949875	3.0020442	2.7234335	0.5070567	0.228446	0.3677514	up
A_23_P46903	CAMK2G	-0.02273	0.396627	0.2932987	0.4193568	0.3160286	0.3676927	up
A_33_P3569068	MAD1L1	1.7432108	2.3543487	1.8673906	0.6111379	0.1241798	0.3676589	up
A_23_P78750	SLC17A7	-0.273265	0.0108647	0.177732	0.2841296	0.4509969	0.3675633	up
A_33_P3265549	SLC19A1	-1.43821	-1.078259	-1.063108	0.359951	0.375102	0.3675265	up
A_23_P80473	CHST13	1.5418649	1.9670329	1.8516636	0.425168	0.3097987	0.3674834	up
A_33_P3353979	SYT15	-2.61643	-2.452477	-2.045571	0.1639528	0.5708582	0.3674055	up
A_22_P00014779	lnc-SLC38A8-1	-0.17186	0.1853581	0.2057123	0.3572178	0.3775721	0.3673949	up
A_33_P3334828	INSL3	-1.491489	-1.102177	-1.146356	0.3893118	0.3451328	0.3672223	up
A_33_P3249877	BEST3	0.061779	0.4007378	0.4569831	0.3389587	0.3952041	0.3670814	up
A_23_P49254	HBQ1	-0.94542	-0.554299	-0.602543	0.3911209	0.3428769	0.3669989	up
A_23_P118038	NUTF2	4.041744	4.4399657	4.3774853	0.3982215	0.335741	0.3669813	up
A_33_P3290924	BAG1	2.7876034	3.2784162	3.03055	0.4908128	0.2429466	0.3668797	up
A_33_P3394605	HMG20B	2.4418917	2.798575	2.8188972	0.3566833	0.3770056	0.3668444	up
A_21_P0011212	LINC00367	-2.50223	-2.094532	-2.176249	0.4076984	0.3259811	0.3668398	up
A_33_P3358208	PADI1	5.4919233	5.91326	5.8041887	0.4213367	0.3122654	0.366801	up
A_33_P3224371	PQBP1	2.1867695	2.6518388	2.455284	0.4650693	0.2685146	0.366792	up
A_24_P933011	TMEM200C	-2.704294	-2.026351	-2.648753	0.6779425	0.0555403	0.3667414	up
A_33_P3256914	ITFG3	-1.960712	-1.396736	-1.791433	0.5639763	0.1692791	0.3666277	up
A_23_P36928	POLR1D	4.2159967	4.636663	4.52839	0.4206662	0.3123932	0.3665297	up
A_23_P404667	BIK	4.5560865	5.3040624	4.541106	0.7479758	-0.01498	0.3664977	up
A_23_P367405	PCBD1	6.0375338	6.3672824	6.440694	0.3297486	0.4031601	0.3664544	up
A_33_P3282740	LINC00869	-0.799127	-0.312654	-0.552804	0.4864731	0.2463226	0.3663979	up

A_23_P329361	COG7	1.8731184	2.1671758	2.3117714	0.2940574	0.438653	0.3663552	up
A_21_P0004812	lnc-AL035696.1-4	-1.926053	-1.52535	-1.59413	0.4007034	0.331923	0.3663132	up
A_23_P18292	RPL14	8.284985	8.729286	8.573047	0.4443016	0.2880621	0.3661819	up
A_22_P00015884	lnc-TCEANC2-1	-1.39134	-1.114772	-0.935555	0.2765679	0.4557848	0.3661764	up
A_23_P5131	ISYNA1	2.575613	2.8232045	3.060277	0.2475915	0.484664	0.3661277	up
A_23_P353742	AMBRA1	3.3605776	3.7025466	3.7506752	0.341969	0.3900976	0.3660333	up
A_22_P00017634	lnc-WNT1-1	-1.59167	-1.34036	-1.111104	0.2513104	0.4805665	0.3659384	up
A_33_P3306504	ISYNA1	1.7574034	2.0232234	2.2231426	0.26582	0.4657393	0.3657796	up
A_22_P00007742	LINGO1-AS1	-0.245481	0.1635819	0.0768662	0.4090624	0.3223467	0.3657046	up
A_33_P3691615	lnc-RP11-389E17.1.1-1	-0.148492	0.2826772	0.1516595	0.431169	0.3001514	0.3656602	up
A_22_P00001917	IVD	0.6785817	1.1716757	0.9167199	0.493094	0.2381382	0.3656161	up
A_32_P220307	RPL39	9.07632	9.5661125	9.317699	0.4897928	0.2413797	0.3655863	up
A_22_P00020938	lnc-DHX34-2	-0.550439	0.2018457	-0.571726	0.752285	-0.021286	0.3654993	up
A_24_P32118	ZFYVE26	2.1182728	2.553742	2.413641	0.4354692	0.2953682	0.3654187	up
A_33_P3249716	TMEM178A	0.0796714	0.3808699	0.5091543	0.3011985	0.4294829	0.3653407	up
A_33_P3275801	DES	0.8387828	1.2467346	1.1614108	0.4079518	0.322628	0.3652899	up
A_21_P0012131	MIR646HG	-2.137083	-2.723891	-0.819722	-0.586808	1.3173609	0.3652765	up
A_21_P0000286	SNORD48	-1.8102	-1.448132	-1.442101	0.3620682	0.3680987	0.3650835	up
A_33_P3359368	DHRS4L1	0.1764741	0.5463657	0.5367017	0.3698916	0.3602276	0.3650596	up
A_21_P0000202	MGAT4B	6.28533	6.649319	6.6513844	0.3639894	0.3660545	0.3650219	up
A_23_P218675	WFDC2	-0.738042	-0.289115	-0.457049	0.4489269	0.280993	0.36496	up
A_24_P363896	COL27A1	0.3326674	0.861495	0.5336776	0.5288277	0.2010102	0.364919	up
A_33_P3217147	GPATCH3	-2.174083	-1.706053	-1.912298	0.46803	0.2617855	0.3649077	up
A_23_P153441	HOOK2	2.8793297	3.4590096	3.029358	0.57968	0.1500282	0.3648541	up
A_23_P74435	C1orf50	2.9387016	3.4423337	3.1646671	0.5036321	0.2259655	0.3647988	up
A_23_P13083	BARX2	-2.067295	-1.897303	-1.507764	0.169992	0.5595312	0.3647616	up
A_24_P134834	RHBDD1	-0.42923	-0.081796	-0.047221	0.3474336	0.382009	0.3647213	up
A_23_P372334	CCDC12	4.0188837	4.2259774	4.54111	0.2070937	0.5222263	0.36466	up
A_33_P3252479	SETDB1	0.965446	1.3665042	1.2937012	0.4010582	0.3282552	0.3646567	up
A_33_P3320062	PLD1	0.3534403	0.7047057	0.7314715	0.3512654	0.3780313	0.3646483	up
A_23_P13338	INTS5	-1.935089	-1.669403	-1.471562	0.2656856	0.4635263	0.3646059	up
A_24_P354496	WWC2-AS2	-1.261472	-0.714985	-1.078851	0.5464864	0.1826205	0.3645535	up
A_23_P52647	EHD1	5.1070795	5.5489907	5.394019	0.4419112	0.2869396	0.3644254	up
A_22_P00001797	LOC101927476	-3.100861	-2.436054	-3.036937	0.6648073	0.0639241	0.3643657	up
A_23_P170467	USP3	2.6620007	3.1879601	2.8644047	0.5259595	0.202404	0.3641818	up
A_22_P00020445	lnc-C8orf48-3	-2.140691	-1.979903	-1.573192	0.1607881	0.5674996	0.3641439	up
A_21_P0011376	XLOC_l2_004940	10.096911	10.528709	10.393084	0.431798	0.2961721	0.3639851	up
A_23_P115105	SZRD1	4.4316025	4.7195926	4.8714523	0.2879901	0.4398499	0.36392	up
A_22_P00018539	ERICH6-AS1	0.6956062	1.0613594	1.0574846	0.3657532	0.3618784	0.3638158	up
A_33_P3367855	PIK3R2	5.7935295	6.1150393	6.1995945	0.3215098	0.406065	0.3637874	up
A_21_P0006502	lnc-ZC3H12B-2	0.4221196	0.9734654	0.5980506	0.5513458	0.175931	0.3636384	up
A_33_P3410235	DUOXA1	-0.196664	0.2083316	0.1255722	0.4049959	0.3222365	0.3636162	up
A_32_P81357	FAHD2A	3.251937	3.5172381	3.7138586	0.2653012	0.4619217	0.3636115	up
A_23_P63243	C1orf43	5.085129	5.523233	5.374009	0.4381042	0.2888804	0.3634923	up
A_23_P361448	SESN3	-0.515596	0.2528677	-0.557121	0.7684636	-0.041525	0.3634691	up
A_32_P192970	ALDH4A1	-0.477066	-0.183849	-0.043895	0.2932162	0.4331703	0.3631933	up
A_23_P35414	PPP1R3C	-3.213352	-2.579708	-3.120634	0.6336441	0.0927186	0.3631814	up
A_23_P75500	BRMS1	3.7954712	4.0637507	4.2533827	0.2682796	0.4579115	0.3630955	up
A_19_P00805812	lnc-UQCRFS1-9	-2.42387	-1.789969	-2.331828	0.6339006	0.0920424	0.3629715	up
A_33_P3367171	SLC22A8	-0.73109	-0.416042	-0.320274	0.3150477	0.4108157	0.3629317	up
A_21_P0013656	XLOC_l2_015213	-1.185248	-0.991818	-0.652856	0.19343	0.5323916	0.3629108	up
A_23_P91702	EIF3D	7.05089	7.536885	7.290661	0.4859948	0.2397709	0.3628829	up
A_32_P857658	RPLP1	10.015983	10.401051	10.356602	0.3850679	0.3406191	0.3628435	up
A_23_P107801	C19orf44	1.5787458	1.8767462	2.0064192	0.2980003	0.4276733	0.3628368	up
A_23_P114155	GPKOW	3.1281977	3.4701238	3.5117502	0.3419261	0.3835526	0.3627393	up

A_33_P3283900	CTNS	-3.429534	-3.203283	-2.930382	0.2262511	0.4991527	0.3627019	up
A_32_P783	RPL37A	9.77476	10.205626	10.069042	0.4308653	0.294282	0.3625736	up
A_21_P0007566	lnc-NAB2-1	-1.830887	-1.614587	-1.322083	0.2163005	0.5088048	0.3625526	up
A_24_P418418	RPS17	9.745084	10.219124	9.996141	0.47404	0.2510576	0.3625488	up
A_24_P394246	SHISA5	1.9004078	2.1875634	2.3382912	0.2871556	0.4378834	0.3625195	up
A_22_P00017533	lnc-WDR26-2	-2.404154	-1.95573	-2.127638	0.4484239	0.2765164	0.3624702	up
A_21_P0000498	SNORD1C	-1.668257	-1.32396	-1.287677	0.3442965	0.3805795	0.362438	up
A_23_P42997	CPSF4	3.1575813	3.6489577	3.3910055	0.4913764	0.2334242	0.3624003	up
A_23_P5983	PLTP	1.18641	1.6562405	1.4412699	0.4698305	0.2548599	0.3623452	up
A_22_P00016379	RPARP-AS1	1.5222974	1.7555461	2.013545	0.2332487	0.4912477	0.3622482	up
A_23_P84910	ZNF157	0.7349019	1.336484	0.8577466	0.6015821	0.1228447	0.3622134	up
A_23_P66487	SMARCD2	2.566824	2.7955232	3.0625381	0.2286992	0.4957142	0.3622067	up
A_24_P401739	ARHGAP17	2.0615873	2.482142	2.3654423	0.4205546	0.3038549	0.3622048	up
A_33_P3405680	ARHGEF3-AS1	-2.389727	-1.996344	-2.058774	0.393383	0.3309526	0.3621678	up
A_23_P160582	HYI	0.9467692	1.5150304	1.1028323	0.5682612	0.1560631	0.3621621	up
A_33_P3619819	lnc-USP35-1	7.9305515	8.213234	8.372088	0.2826824	0.4415369	0.3621097	up
A_23_P126393	SETDB1	4.1937284	4.7327876	4.378848	0.5390592	0.1851196	0.3620894	up
A_33_P3364741	MRC2	2.6739664	2.821425	3.2506418	0.1474586	0.5766754	0.362067	up
A_23_P420863	NOD2	-0.697217	0.0072813	-0.677619	0.7044988	0.019598	0.3620484	up
A_33_P3846653	KRT19P2	7.198429	7.6045737	7.5163736	0.4061446	0.3179445	0.3620446	up
A_23_P165148	ILVBL	3.7119055	4.0210567	4.1266775	0.3091512	0.414772	0.3619616	up
A_23_P50839	MBD3	3.1190453	3.5047898	3.4571896	0.3857446	0.3381443	0.3619444	up
A_24_P206121	KCNMB1	0.2255807	0.7960038	0.3789558	0.5704231	0.1533752	0.3618991	up
A_22_P00003960	lnc-CHCHD5-2	-0.386828	-0.015914	-0.034081	0.370914	0.352747	0.3618305	up
A_33_P3270295	FRS3	-3.175607	-2.704891	-2.922669	0.4707158	0.2529378	0.3618268	up
A_23_P126135	MFN2	5.010234	5.290198	5.453905	0.279964	0.4436712	0.3618176	up
A_21_P0000855	LOC100506585	-3.131173	-2.804763	-2.734108	0.3264103	0.3970656	0.361738	up
A_24_P136182	RPS2P32	8.80347	9.201782	9.12854	0.3983126	0.3250704	0.3616915	up
A_33_P3287529	SDF4	1.7677784	2.1462884	2.1126413	0.37851	0.3448629	0.3616865	up
A_33_P3223544	TMBIM4	4.0397778	4.4392047	4.363661	0.3994269	0.3238831	0.361655	up
A_24_P161463	ZFAS1	6.6114902	7.2266335	6.7196493	0.6151433	0.1081591	0.3616512	up
A_22_P00002740	C21orf2	-0.002606	0.4500065	0.2678328	0.4526124	0.2704387	0.3615255	up
A_23_P501339	CDIPT	1.7882633	2.104216	2.1952877	0.3159528	0.4070244	0.3614886	up
A_22_P00001467	ARAP2	-2.907635	-2.68689	-2.405448	0.2207449	0.5021865	0.3614657	up
A_33_P3384284	SUPT20HL1	-1.875923	-1.386578	-1.642816	0.4893451	0.2331071	0.3612261	up
A_33_P3225690	ZNF516	1.3009744	1.9439397	1.3803906	0.6429653	0.0794163	0.3611908	up
A_23_P145238	HIST1H2BK	4.0266027	4.2718616	4.5037174	0.2452588	0.4771147	0.3611867	up
A_23_P165608	SEMA4F	-1.072411	-0.890414	-0.532206	0.1819968	0.5402045	0.3611007	up
A_21_P0000365	SNORD12	0.3253193	0.7698741	0.6029205	0.4445548	0.2776012	0.361078	up
A_22_P00023257	lnc-IFRD2-2	1.0236735	1.4819479	1.287498	0.4582744	0.2638245	0.3610494	up
A_21_P0008121	lnc-SOX1-4	-1.477643	-1.059397	-1.17412	0.4182463	0.3035235	0.3608849	up
A_23_P54816	ATP6V0C	8.960239	9.356691	9.285341	0.396452	0.3251019	0.3607769	up
A_23_P60166	DEPTOR	-0.495646	0.3013544	-0.571353	0.7970009	-0.075707	0.360647	up
A_33_P3359071	ACD	5.4026737	5.7323747	5.7942514	0.329701	0.3915777	0.3606393	up
A_33_P3383955	DDB2	0.6092916	0.8089805	1.1307917	0.1996889	0.5215001	0.3605945	up
A_33_P3353712	RER1	3.4722157	3.900516	3.7650795	0.4283004	0.2928639	0.3605821	up
A_23_P133585	CDK7	3.6538363	4.099502	3.9291897	0.4456658	0.2753534	0.3605096	up
A_22_P00012116	GOLGA3	-1.160968	-0.783483	-0.817607	0.3774853	0.3433604	0.3604229	up
A_33_P3789056	PIK3CD-AS1	-0.830021	-0.524623	-0.414622	0.3053985	0.4153996	0.360399	up
A_32_P1381	HMGN2	4.2073536	4.700599	4.4345694	0.4932456	0.2272158	0.3602307	up
A_23_P316239	CXorf40B	3.9883003	4.441995	4.2550516	0.4536948	0.2667513	0.3602231	up
A_23_P168898	RPS20	9.463672	9.957909	9.689783	0.494237	0.2261114	0.3601742	up
A_23_P94736	ST6GALNAC4	2.0017738	2.4223762	2.3014479	0.4206023	0.299674	0.3601382	up
A_24_P57898	BHLHE23	3.5595274	4.0581574	3.7810745	0.4986301	0.2215471	0.3600886	up
A_33_P3262560	LAMA3	-1.610324	-1.49137	-1.009115	0.1189547	0.6012096	0.3600822	up
A_33_P3399875	C20orf144	-1.117535	-0.789295	-0.725692	0.3282394	0.3918428	0.3600411	up

A_21_P0009274	lnc-MFSD11-2	-2.812493	-2.651917	-2.253295	0.1605763	0.5591989	0.3598876	up
A_23_P9582	TUFM	6.4276075	6.7550416	6.8199453	0.3274341	0.3923378	0.3598859	up
A_23_P42649	POLR2J	4.0984554	4.3611703	4.5554523	0.2627149	0.4569969	0.3598559	up
A_22_P00023572	lnc-CPT2-5	-1.186763	-1.024782	-0.629229	0.1619806	0.5575342	0.3597574	up
A_23_P69670	BLOC1S4	1.3265481	1.647387	1.7250357	0.3208389	0.3984876	0.3596633	up
A_33_P3355281	MIOX	-1.026722	-0.326695	-1.007517	0.700027	0.0192051	0.359616	up
A_22_P00013348	LOC100507420	-0.649547	-0.285284	-0.294585	0.3642635	0.3549619	0.3596127	up
A_33_P3245011	DAK	5.3493004	5.835711	5.581912	0.4864106	0.2326117	0.3595111	up
A_24_P781757	LOC148413	-0.373852	-0.014404	-0.014369	0.3594475	0.3594832	0.3594654	up
A_24_P115971	RIC8A	0.8933935	1.2733955	1.2323041	0.380002	0.3389106	0.3594563	up
A_33_P3322388	SPRR2D	1.2370052	1.6802511	1.5126185	0.4432459	0.2756133	0.3594296	up
A_23_P421221	R3HCC1	1.6111469	1.953054	1.9878864	0.341907	0.3767395	0.3593233	up
A_33_P3264577	DCTN1	3.4589605	3.8463626	3.790038	0.3874021	0.3310776	0.3592398	up
A_24_P389994	NADK	-1.475506	-0.995139	-1.23765	0.4803672	0.2378559	0.3591115	up
A_22_P00021928	lnc-MTRNR2L3-1	-3.236371	-2.659674	-3.094878	0.5766969	0.1414933	0.3590951	up
A_21_P0002360	lnc-CCDC140-5	-3.100711	-2.883756	-2.599546	0.216955	0.5011647	0.3590598	up
A_23_P429560	SSH1	0.9390559	1.3251705	1.2708979	0.3861146	0.331842	0.3589783	up
A_23_P88106	MCF2L	-1.935574	-1.248267	-1.905364	0.6873069	0.0302095	0.3587582	up
A_24_P307854	FBXL18	1.547739	1.920197	1.8927727	0.372458	0.3450337	0.3587458	up
A_33_P3227217	SNORA81	1.1583586	1.5615463	1.4724407	0.4031878	0.3140822	0.358635	up
A_33_P3214096	ATF3	-0.764942	-0.408535	-0.404158	0.3564062	0.3607836	0.3585949	up
A_32_P528311	RTN4RL2	-0.389275	0.1012073	-0.162666	0.4904823	0.2266088	0.3585455	up
A_22_P00000215	PIK3IP1-AS1	-3.057128	-2.916604	-2.480599	0.1405244	0.5765288	0.3585266	up
A_22_P00000471	SRRM2-AS1	-0.601	-0.031951	-0.453033	0.5690494	0.1479678	0.3585086	up
A_21_P0000354	SCARNA8	1.2799354	1.7622256	1.5146217	0.4822903	0.2346864	0.3584883	up
A_22_P00004728	lnc-CTIF-1	-2.068656	-1.619237	-1.801257	0.449419	0.2673993	0.3584092	up
A_33_P3302881	KLHL31	-2.738408	-2.364185	-2.396199	0.374223	0.3422084	0.3582157	up
A_22_P00015052	SNHG15	3.7790222	4.172994	4.1014805	0.3939719	0.3224583	0.3582151	up
A_22_P00012752	lnc-RAD1-2	1.8263397	2.152082	2.217022	0.3257422	0.3906822	0.3582122	up
A_24_P175427	MRRF	0.2200556	0.5079775	0.6484022	0.2879219	0.4283466	0.3581343	up
A_21_P0007907	LINC00540	-1.082282	-0.782617	-0.665915	0.2996645	0.4163666	0.3580155	up
A_33_P3402918	PNKP	3.1212645	3.434811	3.5236568	0.3135467	0.4023924	0.3579695	up
A_23_P217901	TSTD1	4.6038465	4.9406123	4.9828863	0.3367658	0.3790398	0.3579028	up
A_22_P00012509	LOC101927886	-2.108952	-1.892548	-1.609557	0.216404	0.4993949	0.3578994	up
A_23_P81241	NDFIP1	4.9851274	5.4579883	5.2279186	0.4728608	0.2427912	0.357826	up
A_22_P00005633	lnc-EIF1-1	-2.421066	-2.131357	-1.995142	0.2897084	0.4259243	0.3578163	up
A_21_P0010026	lnc-GGTLC1-4	-3.311794	-2.878317	-3.029709	0.4334769	0.2820842	0.3577806	up
A_24_P99795	ISOC2	3.0552473	3.2890983	3.5367832	0.233851	0.4815359	0.3576934	up
A_23_P156209	ZNF622	3.5939302	4.088879	3.814333	0.4949489	0.2204027	0.3576758	up
A_33_P3231407	PYHIN1	-3.255045	-2.932771	-2.862037	0.3222737	0.3930082	0.357641	up
A_24_P361006	NDUFA9	4.080159	4.3571115	4.51847	0.2769523	0.4383106	0.3576314	up
A_21_P0004514	lnc-XRCC4-1	-1.731031	-0.991582	-1.755219	0.7394495	-0.024187	0.3576312	up
A_32_P151544	KRT18	10.278289	10.677126	10.5945	0.3988371	0.3162108	0.3575239	up
A_33_P3222045	EEF2KMT	2.2871351	2.7293077	2.5599842	0.4421725	0.2728491	0.3575108	up
A_33_P3358069	IDH3B	3.7084084	3.974666	4.156995	0.2662578	0.4485865	0.3574221	up
A_33_P3346663	AP2S1	9.373507	9.765711	9.695948	0.3922043	0.3224411	0.3573227	up
A_24_P264943	COMP	-3.439009	-2.928447	-3.234929	0.5105624	0.2040799	0.3573211	up
A_23_P204702	TMBIM6	6.1828976	6.7063336	6.3737755	0.5234361	0.1908779	0.357157	up
A_33_P3424800	HLA-B	7.3819714	7.735686	7.7422953	0.3537145	0.3603239	0.3570192	up
A_33_P3398107	SYS1	1.7099342	2.151342	1.9825583	0.4414077	0.272624	0.3570159	up
A_21_P0013956	PPFIA4	-2.645666	-2.295008	-2.282426	0.3506584	0.3632405	0.3569495	up
A_32_P184464	ROPN1	0.2291975	0.5382533	0.6339498	0.3090558	0.4047523	0.356904	up
A_22_P00014995	LOC102723385	-2.058828	-1.620989	-1.78295	0.437839	0.2758784	0.3568587	up
A_22_P00024564	LOC102724094	2.6266813	2.8713822	3.095499	0.2447009	0.4688177	0.3567593	up
A_33_P3222019	WSCD2	-1.241439	-0.842217	-0.927171	0.3992224	0.3142681	0.3567452	up
A_23_P391689	PET100	6.482748	6.872483	6.80649	0.3897348	0.3237419	0.3567383	up

A_33_P3216853	C2orf15	0.1713853	0.5650001	0.4912052	0.3936148	0.3198199	0.3567174	up
A_33_P3375398	TBL3	2.2054033	2.5572715	2.566887	0.3518682	0.3614836	0.3566759	up
A_23_P334664	PML	-0.444077	-0.441469	0.266448	0.0026078	0.710525	0.3565664	up
A_33_P3321836	PLXNB2	2.2249784	2.579533	2.58352	0.3545547	0.3585415	0.3565481	up
A_23_P55873	CC2D1A	3.0561438	3.3898616	3.4353848	0.3337178	0.379241	0.3564794	up
A_33_P3325935	PQBP1	4.925083	5.329781	5.233329	0.4046979	0.3082457	0.3564718	up
A_21_P0004309	lnc-NDFIP1-2	-2.613075	-2.141576	-2.371694	0.4714992	0.2413814	0.3564403	up
A_23_P137909	HIST3H3	8.071333	8.405245	8.450297	0.3339119	0.3789644	0.3564382	up
A_33_P3352148	AGAP2	-1.274906	-1.021218	-0.81596	0.2536874	0.4589458	0.3563166	up
A_33_P3356776	GOLGA7B	-0.707957	-0.442462	-0.260862	0.2654958	0.4470949	0.3562954	up
A_23_P29096	PDE9A	-0.198367	0.1041608	0.2115011	0.3025279	0.4098682	0.3561981	up
A_23_P217528	KLF8	-2.979873	-2.050621	-3.196772	0.9292522	-0.2169	0.3561763	up
A_33_P3363245	NXPH4	2.7589254	3.1883721	3.0417252	0.4294467	0.2827997	0.3561232	up
A_23_P60537	PRPF4	3.8896418	4.1595674	4.331871	0.2699256	0.4422293	0.3560774	up
A_23_P43557	DENND1A	-0.66596	-0.21256	-0.407304	0.4534001	0.258656	0.3560281	up
A_23_P137403	LRRC41	0.3954973	0.7811918	0.7217736	0.3856945	0.3262763	0.3559854	up
A_22_P00011185	LOC102724000	-3.100978	-2.726875	-2.763231	0.3741028	0.3377473	0.3559251	up
A_21_P0001643	lnc-IRF2BP2-2	-3.263143	-2.59136	-3.223103	0.671783	0.0400403	0.3559116	up
A_21_P0011923	WDPCP	0.9143944	1.2986588	1.2416606	0.3842645	0.3272662	0.3557653	up
A_23_P67042	MOCOS	1.6452045	2.2517056	1.7501707	0.6065011	0.1049662	0.3557336	up
A_24_P211044	CSH1	-1.554166	-1.217972	-1.179001	0.336194	0.375165	0.3556795	up
A_21_P0000355	SCARNA15	-3.246411	-3.121358	-2.66026	0.1250529	0.5861509	0.3556019	up
A_21_P0014585	TMEM44-AS1	-2.71242	-2.439884	-2.273819	0.2725365	0.4386015	0.355569	up
A_23_P79043	TMEM147	5.8805256	6.2557473	6.216381	0.3752217	0.3358555	0.3555386	up
A_33_P3211213	TSEN34	7.063987	7.376887	7.4620924	0.3129001	0.3981056	0.3555028	up
A_22_P00017751	lnc-YIF1A-3	-1.284362	-0.76217	-1.095849	0.522192	0.1885133	0.3553526	up
A_33_P3525263	A2ML1	1.8273621	2.556847	1.8084645	0.729485	-0.018898	0.3552937	up
A_23_P256735	CPQ	0.7720499	1.1490755	1.1054654	0.3770256	0.3334155	0.3552206	up
A_22_P00005313	lnc-DNAJC8-1	-2.761711	-2.423955	-2.38921	0.3377562	0.3725009	0.3551285	up
A_24_P287189	TOLLIP	-2.030913	-1.69047	-1.661191	0.3404427	0.3697224	0.3550825	up
A_23_P211598	PMM1	0.0713878	0.7748461	0.0780911	0.7034583	0.0067034	0.3550808	up
A_21_P0007654	lnc-TMEM132B-2	-2.628847	-2.337744	-2.210021	0.2911029	0.4188263	0.3549646	up
A_23_P313	PEF1	1.9297037	2.2634234	2.3056011	0.3337197	0.3758974	0.3548086	up
A_33_P3259548	WDR5B	-2.427993	-2.019925	-2.126474	0.4080677	0.3015189	0.3547933	up
A_23_P416034	HAUS7	-1.191077	-0.949458	-0.723337	0.2416191	0.4677401	0.3546796	up
A_23_P370569	C12orf66	-1.017398	-0.870483	-0.454956	0.146915	0.5624428	0.3546789	up
A_33_P3319006	C8G	-1.474729	-1.200924	-1.039341	0.2738042	0.4353876	0.3545959	up
A_24_P149704	DAB2IP	-0.813906	-0.450724	-0.468108	0.3631821	0.345798	0.35449	up
A_33_P3392077	TP53I3	4.388362	4.9852424	4.500453	0.5968804	0.1120911	0.3544858	up
A_23_P253421	AUP1	4.867774	5.251205	5.193123	0.383431	0.3253489	0.3543899	up
A_23_P90523	OCEL1	1.1364765	1.3856373	1.5960464	0.2491608	0.4595699	0.3543654	up
A_23_P59153	LSM2	2.950345	3.4136062	3.1955833	0.4632611	0.2452383	0.3542497	up
A_33_P3399560	IFT27	1.3440948	1.5708394	1.8256698	0.2267447	0.481575	0.3541598	up
A_23_P55948	PRR12	2.6063814	3.1231208	2.7977486	0.5167394	0.1913672	0.3540533	up
A_23_P153853	ECH1	3.3070412	3.6368403	3.685073	0.3297992	0.3780317	0.3539155	up
A_33_P3653330	MAP2K4	1.7415123	2.2504334	1.9402599	0.5089212	0.1987476	0.3538344	up
A_21_P0012112	XLOC_l2_008560	9.86255	10.261803	10.170864	0.3992529	0.3083143	0.3537836	up
A_33_P3358019	LOC102723804	-1.707718	-1.420856	-1.287021	0.2868619	0.4206972	0.3537796	up
A_32_P73217	TMEM167B	-0.46486	-0.336112	0.1139469	0.1287484	0.5788074	0.3537779	up
A_21_P0010019	lnc-FOXA2-1	-1.394119	-0.892857	-1.187881	0.5012617	0.2062378	0.3537498	up
A_23_P34968	SCNM1	5.546111	5.8859944	5.9135056	0.3398833	0.3673945	0.3536389	up
A_33_P3272921	ARID3A	0.0391884	0.5362501	0.2493463	0.4970617	0.2101579	0.3536098	up
A_23_P32454	TG	-2.991938	-2.495824	-2.780965	0.496114	0.210973	0.3535435	up
A_33_P3388501	CHIT1	0.6668916	1.0401025	1.0007024	0.3732109	0.3338108	0.3535109	up
A_23_P56604	IL1RL2	-2.957517	-2.367937	-2.840169	0.5895793	0.1173482	0.3534638	up
A_23_P323227	PPP1CA	4.8148575	5.1889806	5.1475925	0.3741231	0.3327351	0.3534291	up

A_23_P325661	ZNF134	-0.190136	0.2359438	0.0904636	0.4260802	0.2806001	0.3533402	up
A_23_P257578	SRA1	3.2362041	3.618136	3.5608377	0.3819318	0.3246336	0.3532827	up
A_22_P00025786	RPARP-AS1	-0.346603	-0.172238	0.185493	0.1743655	0.5320964	0.353231	up
A_21_P0014218	LOC101927067	-2.461254	-2.001548	-2.214532	0.4597063	0.2467222	0.3532143	up
A_21_P0013348	XLOC_l2_014001	-1.258845	-0.726223	-1.085177	0.5326214	0.1736684	0.3531449	up
A_23_P352435	RGS12	-1.045987	-0.575417	-0.810307	0.4705701	0.2356806	0.3531253	up
A_24_P96961	SPSB1	2.8524523	3.456264	2.9548874	0.6038117	0.1024351	0.3531234	up
A_22_P00025856	LOC100132735	-2.320832	-2.017775	-1.917851	0.3030567	0.4029813	0.353019	up
A_24_P348203	LRRC8E	2.0428753	2.4777417	2.3140345	0.4348664	0.2711592	0.3530128	up
A_19_P00320274	ZMIZ1-AS1	-2.427783	-2.331212	-1.818423	0.096571	0.6093602	0.3529656	up
A_24_P76018	ARIH2OS	-3.165507	-2.70118	-2.923919	0.4643269	0.2415881	0.3529575	up
A_23_P324538	LCE3B	-1.754658	-1.499895	-1.303594	0.2547627	0.4510641	0.3529134	up
A_33_P6807856	LINC00689	-2.894184	-2.264082	-2.818468	0.6301012	0.075716	0.3529086	up
A_23_P119698	CD320	4.024434	4.272035	4.4825697	0.247601	0.4581356	0.3528683	up
A_23_P25994	LGMN	0.7495961	1.1951985	1.0097041	0.4456024	0.260108	0.3528552	up
A_33_P3234521	DIS3L	-2.415473	-2.19127	-1.934004	0.2242026	0.4814692	0.3528359	up
A_21_P0000488	SNORD96B	-1.345331	-0.873506	-1.111559	0.4718251	0.2337718	0.3527985	up
A_22_P00014071	PPP1R9B	-1.394257	-0.955302	-1.127786	0.4389548	0.2664709	0.3527129	up
A_33_P3347343	CCDC102B	-2.842753	-2.276078	-2.704156	0.5666752	0.1385968	0.352636	up
A_23_P143303	VPS16	2.5753565	3.014604	2.841361	0.4392476	0.2660046	0.3526261	up
A_24_P357100	RASD2	-2.683742	-2.262594	-2.39966	0.4211476	0.2840819	0.3526148	up
A_19_P00320792	SNHG11	-0.651608	-0.2483	-0.349749	0.4033074	0.3018584	0.3525829	up
A_23_P216402	UCK1	1.2121978	1.4674621	1.6619773	0.2552643	0.4497795	0.3525219	up
A_23_P2967	AP1G2	0.304668	0.7001543	0.6141372	0.3954864	0.3094692	0.3524778	up
A_23_P154234	POLE4	4.3000154	4.798983	4.505934	0.4989677	0.2059183	0.352443	up
A_23_P379550	YARS	2.4485102	3.0460544	2.5558014	0.5975442	0.1072912	0.3524177	up
A_33_P3371785	DTNA	0.3603935	0.6965284	0.7290587	0.3361349	0.3686652	0.3524001	up
A_21_P0003083	lnc-HES1-2	-2.110465	-2.011337	-1.504802	0.0991283	0.6056628	0.3523955	up
A_23_P100632	HN1	8.660856	8.946711	9.079697	0.2858543	0.4188404	0.3523474	up
A_21_P0009526	lnc-PIEZO2-4	-2.722251	-2.327814	-2.412045	0.3944373	0.3102062	0.3523217	up
A_24_P251381	NHEJ1	0.2361116	0.7138438	0.4628048	0.4777322	0.2266932	0.3522127	up
A_23_P81399	SQSTM1	5.8608456	6.697952	5.7281113	0.8371062	-0.132734	0.352186	up
A_21_P0000219	SNORD34	0.3229098	0.7023625	0.6474676	0.3794527	0.3245578	0.3520052	up
A_33_P3369844	CD24	3.6804085	3.8876328	4.1769457	0.2072244	0.4965372	0.3518808	up
A_23_P161156	ZNF438	1.407795	1.7140293	1.8052063	0.3062344	0.3974114	0.3518229	up
A_23_P87011	TAGLN	0.3928738	0.8219638	0.6673527	0.42909	0.2744789	0.3517845	up
A_22_P00011905	lnc-PIK3R1-3	-2.447304	-2.110519	-2.080948	0.3367848	0.3663557	0.3515703	up
A_33_P3224996	MMP25-AS1	-1.409276	-1.031479	-1.084087	0.3777962	0.3251882	0.3514922	up
A_23_P90589	MRPL44	2.950305	3.2172627	3.3863258	0.2669578	0.4360209	0.3514893	up
A_23_P330895	NFKBIB	2.0842457	2.49853	2.3729076	0.4142842	0.288662	0.3514731	up
A_24_P538478	MED28	-2.427858	-1.747437	-2.405411	0.6804214	0.0224476	0.3514345	up
A_32_P194115	SUN2	0.7241578	1.2026892	0.9483361	0.4785314	0.2241783	0.3513548	up
A_22_P00001924	lnc-BAI3-3	0.0676198	0.4892244	0.3486629	0.4216046	0.2810431	0.3513238	up
A_22_P00002486	lnc-C16orf13-3	0.0312843	0.48351	0.2816987	0.4522257	0.2504144	0.35132	up
A_33_P3417502	WNT3A	-1.05356	-0.749596	-0.654914	0.3039637	0.3986454	0.3513045	up
A_33_P3360555	ZNF444	5.309514	5.6543584	5.6672134	0.3448443	0.3576994	0.3512719	up
A_22_P00024006	lnc-FAM168A-2	-3.292841	-3.139914	-2.743381	0.1529262	0.5494597	0.351193	up
A_33_P3378920	RBM14-RBM4	5.986497	6.3913903	6.283804	0.4048934	0.297307	0.3511002	up
A_33_P3396200	ZASP	3.5672684	3.999227	3.8371983	0.4319587	0.2699299	0.3509443	up
A_21_P0001702	lnc-CHD1L-1	-2.764765	-2.301086	-2.526628	0.4636796	0.2381368	0.3509082	up
A_33_P3273020	FKBP2	7.275463	7.6191096	7.6336155	0.3436465	0.3581524	0.3508995	up
A_23_P419602	SREBF2	3.6106033	4.1055474	3.8173513	0.4949441	0.206748	0.3508461	up
A_33_P3301410	EXOSC4	5.127445	5.3996778	5.556821	0.2722325	0.4293757	0.3508041	up
A_33_P3243622	DCAF8	-0.231625	0.1645055	0.0738468	0.3961306	0.3054719	0.3508012	up
A_33_P3352340	KRTAP9-1	-0.87073	-0.594265	-0.445803	0.2764659	0.4249277	0.3506968	up
A_23_P404678	RAB3D	-2.356855	-2.053348	-1.959034	0.3035073	0.397821	0.3506641	up

A_33_P3228460	FXYD3	7.4153605	7.702904	7.8290863	0.2875438	0.4137259	0.3506348	up
A_22_P00002998	LOC102723648	3.0548897	3.4749389	3.3360815	0.4200492	0.2811918	0.3506205	up
A_23_P87902	DYRK4	1.9049568	2.2459016	2.2651358	0.3409448	0.360179	0.3505619	up
A_32_P142700	C22orf15	-0.898382	-0.447817	-0.648209	0.4505644	0.2501731	0.3503687	up
A_33_P3215059	NSMCE1	2.999938	3.2899957	3.4106016	0.2900577	0.4106636	0.3503606	up
A_33_P3399788	SERPINA3	-0.504479	0.1887703	-0.497129	0.6932497	0.0073509	0.3503003	up
A_23_P368909	LINC00346	-2.845837	-2.190593	-2.800796	0.6552441	0.0450411	0.3501426	up
A_21_P0009408	LOC101927471	-1.840037	-1.297453	-1.682403	0.5425835	0.1576338	0.3501086	up
A_19_P00810982	lnc-MACROD1-1	0.122611	0.515923	0.42941	0.393312	0.3067989	0.3500555	up
A_33_P3338793	KCNC3	-2.099806	-1.914206	-1.585371	0.1856008	0.5144353	0.350018	up
A_23_P26314	ZNF319	-1.459611	-1.28914	-0.930216	0.1704707	0.5293942	0.3499324	up
A_24_P86389	HIST1H2AM	5.8873253	6.233252	6.2412195	0.3459268	0.3538942	0.3499105	up
A_23_P316850	ODF3L2	2.2801895	2.8163037	2.4438534	0.5361142	0.1636639	0.349889	up
A_22_P00017016	lnc-TTC4-1	-1.478988	-1.104572	-1.153764	0.3744164	0.3252244	0.3498204	up
A_33_P3604591	SNORA78	-1.91667	-1.597444	-1.536374	0.3192263	0.3802967	0.3497615	up
A_24_P244100	DNAJB12	2.7650528	3.176433	3.0529995	0.4113803	0.2879467	0.3496635	up
A_33_P3408203	TGFA	2.0419426	2.4083228	2.3748875	0.3663802	0.3329449	0.3496625	up
A_21_P0000350	SCARNA20	2.7850742	3.1557508	3.1136532	0.3706765	0.328579	0.3496277	up
A_21_P0013858	TMLHE	0.0072589	0.304873	0.4088421	0.2976141	0.4015832	0.3495987	up
A_19_P00810806	SMIM2-AS1	-0.299361	0.0151291	0.0853009	0.3144903	0.3846622	0.3495762	up
A_33_P3358403	DNLZ	4.728114	5.00971	5.1456013	0.2815957	0.4174871	0.3495414	up
A_23_P208288	ZNF304	0.3563328	0.7917066	0.619885	0.4353738	0.2635522	0.349463	up
A_22_P00015716	lnc-SYT13-2	2.548667	3.0397992	2.7564564	0.4911323	0.2077894	0.3494608	up
A_23_P59616	GTF2IRD2	-1.3998	-0.841584	-1.259219	0.5582161	0.1405816	0.3493989	up
A_24_P346762	FAM214B	-1.593726	-1.002914	-1.485937	0.5908122	0.1077886	0.3493004	up
A_23_P133236	PCDHB14	0.9692106	1.3227234	1.314136	0.3535128	0.3449254	0.3492191	up
A_23_P19576	SF3B5	7.1543913	7.396569	7.6106243	0.2421775	0.456233	0.3492053	up
A_24_P101629	FAM127B	1.7268839	1.8506598	2.3015118	0.123776	0.5746279	0.3492019	up
A_23_P97309	CASP9	1.0958233	1.5511255	1.3388982	0.4553022	0.2430749	0.3491886	up
A_19_P00803334	lnc-GALNTL4-2	-2.990717	-2.384197	-2.899187	0.6065207	0.0915306	0.3490256	up
A_24_P80135	PTPN18	2.5227985	2.736587	3.0068007	0.2137885	0.4840021	0.3488953	up
A_24_P139094	SH3GL1	4.3241367	4.7543797	4.591651	0.430243	0.2675142	0.3488786	up
A_23_P47614	PHLDA2	6.272295	6.495194	6.7470245	0.222899	0.4747295	0.3488143	up
A_23_P12635	FBXL15	4.663472	4.766392	5.2579517	0.1029201	0.5944796	0.3486998	up
A_33_P3387756	LKAAEAR1	2.6581745	3.2031598	2.8105373	0.5449853	0.1523628	0.3486741	up
A_23_P69329	HYAL1	1.5318241	1.9546685	1.80621	0.4228444	0.2743859	0.3486152	up
A_33_P3274811	SPRR2F	0.7963066	1.3868766	0.9029512	0.59057	0.1066446	0.3486073	up
A_23_P405216	ZNF619	-1.671839	-1.417808	-1.228663	0.2540312	0.4431763	0.3486037	up
A_23_P12680	PSAP	5.009203	5.684403	5.0310946	0.6752	0.0218916	0.3485458	up
A_24_P203953	LOC439951	5.0272446	5.508001	5.2435293	0.4807563	0.2162848	0.3485205	up
A_33_P3251932	BCL2L11	0.9307103	1.1919556	1.3664231	0.2612453	0.4357128	0.348479	up
A_32_P30649	ETV5	-0.771822	-0.227947	-0.618761	0.5438743	0.1530604	0.3484674	up
A_23_P1280	ASCC1	1.456768	1.7725191	1.8376913	0.3157511	0.3809233	0.3483372	up
A_23_P39034	SMARCA4	7.0864706	7.5192704	7.350299	0.4327998	0.2638283	0.3483141	up
A_23_P33809	IMP3	5.538349	5.8942533	5.8790407	0.3559041	0.3406916	0.3482978	up
A_24_P75072	SMUG1	2.3643894	2.5990891	2.8262844	0.2346997	0.461895	0.3482974	up
A_22_P00009100	HORMAD2-AS1	-0.61771	-0.194602	-0.344357	0.4231081	0.2733526	0.3482304	up
A_22_P00006187	KCND3-IT1	1.8810453	2.3741364	2.0840034	0.4930911	0.2029581	0.3480246	up
A_23_P315892	ST6GALNAC6	1.6216345	1.8829327	2.056243	0.2612982	0.4346085	0.3479533	up
A_23_P76529	ITGB7	3.894474	4.5430274	3.9417067	0.6485534	0.0472326	0.347893	up
A_23_P132675	UQCRC1	4.5732365	4.8100867	5.0321255	0.2368503	0.458889	0.3478696	up
A_33_P3389363	C19orf54	-1.869789	-1.501623	-1.542346	0.3681665	0.3274436	0.347805	up
A_33_P3268793	ADAT3	2.4551096	2.9355702	2.670146	0.4804606	0.2150364	0.3477485	up
A_23_P161237	GBF1	4.3229837	4.638286	4.703081	0.3153024	0.3800974	0.3476999	up
A_22_P00012656	lnc-PYCARD-1	-2.71189	-2.5605	-2.168146	0.1513891	0.5437434	0.3475663	up
A_32_P47554	HINT1	9.076711	9.531572	9.316971	0.4548616	0.2402601	0.3475609	up

A_23_P170453	CST5	3.274457	3.5462022	3.6976833	0.2717452	0.4232264	0.3474858	up
A_21_P0009057	ZFHX3	-1.094707	-0.948006	-0.546662	0.1467009	0.5480442	0.3473725	up
A_21_P0009977	lnc-TSHZ2-4	-3.263076	-2.650119	-3.181373	0.612957	0.081703	0.34733	up
A_22_P00002559	LOC101928343	-3.03694	-3.188436	-2.190933	-0.151497	0.8460069	0.3472551	up
A_33_P3307363	LPHN2	2.6623592	3.0641742	2.9546185	0.4018149	0.2922592	0.3470371	up
A_33_P3387956	DPP7	3.8787127	4.405104	4.0463724	0.5263915	0.1676598	0.3470256	up
A_23_P215175	ABCF2	2.4423656	2.801004	2.7777386	0.3586383	0.3353729	0.3470056	up
A_33_P3347639	MED18	0.6611824	0.8385763	1.177784	0.1773939	0.5166016	0.3469977	up
A_23_P34018	RPL39	9.187655	9.5986595	9.470564	0.4110041	0.2829084	0.3469563	up
A_23_P314712	CABYR	-1.303513	-1.011468	-0.901761	0.2920451	0.4017525	0.3468988	up
A_24_P409595	MAPK8IP1	-1.949966	-1.65852	-1.547615	0.2914457	0.4023504	0.3468981	up
A_24_P284893	PSMB2	5.6690216	5.9687314	6.062952	0.2997098	0.3939304	0.3468201	up
A_23_P163820	ZNF629	2.2270956	2.4568954	2.6909237	0.2297998	0.4638281	0.3468139	up
A_22_P00016483	LOC148413	2.821126	3.122786	3.2130747	0.3016601	0.3919487	0.3468044	up
A_23_P8452	LFNG	5.2466965	5.4884896	5.698449	0.2417932	0.4517527	0.3467729	up
A_22_P00005305	ENOX1-AS2	-2.696206	-2.065079	-2.634103	0.6311271	0.0621026	0.3466148	up
A_23_P80162	TMPRSS3	0.726099	1.0908222	1.0545073	0.3647232	0.3284082	0.3465657	up
A_23_P327370	PGPEP1	1.1312618	1.4192114	1.5363088	0.2879496	0.4050469	0.3464983	up
A_21_P0000625	LOC100289361	0.1859322	0.2560663	0.8087864	0.0701342	0.6228542	0.3464942	up
A_23_P70991	AIMP2	4.779825	5.2532525	4.999322	0.4734273	0.2194967	0.346462	up
A_22_P00010751	YTHDF3	8.67281	9.055826	8.982439	0.3830166	0.3096294	0.346323	up
A_23_P132260	PES1	2.3937378	2.7630172	2.7169762	0.3692794	0.3232384	0.3462589	up
A_23_P114826	MRPS15	5.2773113	5.6139627	5.6331673	0.3366513	0.3558559	0.3462536	up
A_32_P470728	GSX1	-1.571444	-1.251919	-1.19864	0.3195243	0.3728037	0.346164	up
A_33_P3394978	LOC100128239	-1.995174	-1.464449	-1.833773	0.5307255	0.1614013	0.3460634	up
A_23_P60793	ASMTL-AS1	-2.580542	-2.158809	-2.310226	0.4217327	0.2703157	0.3460242	up
A_23_P128956	ZFYVE1	0.9251456	1.3659401	1.1762385	0.4407945	0.2510929	0.3459437	up
A_23_P54041	THTPA	-0.54786	-0.142708	-0.26119	0.4051514	0.2866693	0.3459103	up
A_22_P00001136	HNRNPKP3	-1.350896	-0.754709	-1.255269	0.5961871	0.0956273	0.3459072	up
A_33_P3378800	AP2M1	8.061584	8.531679	8.283238	0.4700947	0.2216539	0.3458743	up
A_21_P0006528	lnc-GEMIN8-1	-2.708699	-2.160858	-2.564814	0.5478406	0.1438847	0.3458626	up
A_33_P3284586	FBXL8	-1.08815	-0.66519	-0.819538	0.4229598	0.2686119	0.3457859	up
A_22_P00014575	EDNRB-AS1	-0.902696	-0.653526	-0.460428	0.2491703	0.4422679	0.3457191	up
A_22_P00009781	lnc-MED7-1	-1.634066	-1.368506	-1.208237	0.2655602	0.4258289	0.3456945	up
A_23_P118289	BCL7C	2.5383863	2.7358146	3.0323086	0.1974282	0.4939222	0.3456752	up
A_33_P3282898	TLN1	-0.651776	-0.105832	-0.506373	0.5459437	0.1454024	0.3456731	up
A_33_P3394405	LOC727721	-2.101208	-1.865067	-1.646208	0.2361412	0.4549999	0.3455706	up
A_23_P32785	C3orf38	0.7347722	1.0429482	1.1176944	0.308176	0.3829222	0.3455491	up
A_24_P157087	CASP8	0.2599511	0.6376338	0.5733538	0.3776827	0.3134027	0.3455427	up
A_24_P399500	PCP2	-1.764557	-1.166645	-1.671454	0.5979123	0.0931034	0.3455079	up
A_23_P635	PMF1	2.8741493	3.2150474	3.224207	0.340898	0.3500576	0.3454778	up
A_33_P3585268	GNAI2	6.8914633	7.253156	7.2206993	0.3616929	0.329236	0.3454645	up
A_23_P200598	MUL1	2.3318262	2.6888757	2.6655998	0.3570495	0.3337736	0.3454115	up
A_23_P376661	NIT1	1.3225946	1.7422442	1.59373	0.4196496	0.2711353	0.3453925	up
A_33_P3347330	TOMM7	6.9050198	7.244683	7.255807	0.339663	0.3507872	0.3452251	up
A_23_P202206	GSTO2	3.6357079	4.0411963	3.9205484	0.4054885	0.2848406	0.3451645	up
A_33_P3299829	POLR2E	5.549755	5.967832	5.8218946	0.418077	0.2721396	0.3451083	up
A_22_P00015757	lnc-TAF4B-4	-2.225099	-1.81081	-1.94923	0.4142895	0.2758689	0.3450792	up
A_33_P3336384	PAOX	-2.42735	-2.052714	-2.111869	0.3746362	0.3154807	0.3450584	up
A_23_P571	SLC2A1	7.478463	7.868316	7.778674	0.389853	0.300211	0.345032	up
A_24_P219378	CASKIN1	2.610488	2.8516097	3.059186	0.2411218	0.448698	0.3449099	up
A_33_P3216372	IQGAP2	3.1937532	3.5599465	3.5173216	0.3661933	0.3235683	0.3448808	up
A_33_P3212570	PYCARD-AS1	-1.034696	-0.474689	-0.905055	0.5600061	0.1296406	0.3448234	up
A_33_P3407042	B3GALT6	2.9645672	3.2919383	3.3267565	0.3273711	0.3621893	0.3447802	up
A_23_P97932	MSRB2	4.6925125	5.1209693	4.9535503	0.4284568	0.2610378	0.3447473	up
A_33_P3619221	ZMYND8	3.3972397	3.978312	3.5055923	0.5810723	0.1083527	0.3447125	up

A_23_P202658	GSTP1	9.821132	10.199041	10.132557	0.3779097	0.3114252	0.3446674	up
A_33_P3518572	LINC00941	1.7310209	2.03974	2.1115646	0.3087192	0.3805437	0.3446314	up
A_33_P3312682	REXO1	5.2016106	5.589623	5.502675	0.3880124	0.3010645	0.3445385	up
A_33_P3502640	DTX2	2.045989	2.3530803	2.4279604	0.3070912	0.3819714	0.3445313	up
A_23_P27424	ZNF418	-1.110972	-0.645669	-0.887236	0.4653029	0.2237363	0.3445196	up
A_22_P00017240	LOC102724800	-3.329646	-2.938332	-3.032139	0.391314	0.2975071	0.3444105	up
A_32_P113114	ZNF561	0.0325928	0.5181184	0.2358818	0.4855256	0.203289	0.3444073	up
A_33_P3256858	C14orf80	3.9982405	4.38088	4.304387	0.3826394	0.3061466	0.344393	up
A_23_P312863	TUBB4B	5.971546	6.17878	6.452857	0.2072339	0.4813108	0.3442724	up
A_33_P3414851	RNF220	0.675889	1.1100512	0.9301114	0.4341621	0.2542224	0.3441923	up
A_24_P229871	LINC00469	0.2517591	0.7784081	0.4132862	0.526649	0.1615272	0.3440881	up
A_22_P00011853	lnc-PHTF2-1	1.0049667	1.4218802	1.2762113	0.4169135	0.2712445	0.344079	up
A_33_P3577120	TBL3	0.5057564	0.855402	0.8442574	0.3496456	0.338501	0.3440733	up
A_23_P93046	EXOC3	1.5955143	2.0452332	1.8336773	0.449719	0.238163	0.343941	up
A_33_P3217719	TLE1	-1.118137	-0.636891	-0.91249	0.4812455	0.205647	0.3434463	up
A_33_P3233871	F12	5.165082	5.4327025	5.5843134	0.2676206	0.4192314	0.343426	up
A_24_P392022	EEF2KMT	1.3440413	1.864378	1.510427	0.5203366	0.1663857	0.3433611	up
A_23_P214678	CUTA	6.7255936	7.0561795	7.0817194	0.330586	0.3561258	0.3433559	up
A_24_P220618	ZC3H4	4.2639084	4.7149615	4.499468	0.4510531	0.2355595	0.3433063	up
A_32_P331052	RBBP8NL	-0.740082	-0.342237	-0.451394	0.3978448	0.2886882	0.3432665	up
A_23_P3963	CDR2L	4.8971987	5.3654838	5.11539	0.4682851	0.2181912	0.3432381	up
A_33_P3861706	MTMR10	3.7871723	4.029431	4.2313786	0.2422586	0.4442062	0.3432324	up
A_23_P38219	PRPF8	3.241869	3.6070657	3.563013	0.3651967	0.3211441	0.3431704	up
A_23_P332960	TMEM80	3.3463697	3.7300162	3.6489553	0.3836465	0.3025856	0.3431161	up
A_23_P152804	NME1	8.009909	8.335754	8.369897	0.3258457	0.3599882	0.342917	up
A_24_P362317	ADAR	5.7629557	5.9403777	6.2709646	0.1774221	0.508009	0.3427155	up
A_21_P0011135	EIF4A1	5.6992254	5.9959435	6.0878677	0.2967181	0.3886423	0.3426802	up
A_22_P00009205	LOC100506457	-1.691494	-1.351116	-1.346717	0.3403783	0.3447766	0.3425775	up
A_24_P144499	PPIAL4G	8.337319	8.731937	8.627661	0.394618	0.2903414	0.3424797	up
A_22_P00023392	LOC100506526	-0.741811	-0.238572	-0.560237	0.5032392	0.1815743	0.3424067	up
A_33_P3299066	NR4A2	-1.773862	-1.252686	-1.610393	0.5211763	0.1634698	0.3423231	up
A_23_P254165	RAI2	-1.966274	-1.671873	-1.576136	0.2944017	0.3901382	0.3422699	up
A_32_P42197	HNRNPA1	7.979952	8.404299	8.240079	0.4243469	0.2601271	0.342237	up
A_24_P219808	PTOV1	3.76822	4.1633906	4.0574284	0.3951707	0.2892084	0.3421896	up
A_23_P28263	CTDSP1	6.4266806	6.7975917	6.7400627	0.3709111	0.3133822	0.3421466	up
A_21_P0004868	lnc-HIST1H2AI-2	-0.240774	0.1333742	0.0693665	0.3741479	0.3101401	0.342144	up
A_23_P148150	NELFB	3.6680174	3.9779515	4.0423307	0.3099341	0.3743134	0.3421237	up
A_33_P3772937	KRT8P12	6.4144163	6.794598	6.7184124	0.3801818	0.3039961	0.342089	up
A_23_P41588	HARS2	3.190812	3.4640117	3.6017838	0.2731996	0.4109716	0.3420856	up
A_23_P65129	SPRYD3	2.8833818	3.292355	3.1585655	0.4089732	0.2751837	0.3420785	up
A_33_P3260969	PRAP1	2.2468367	2.6399198	2.5378933	0.3930831	0.2910566	0.3420699	up
A_24_P130792	NSUN4	0.5155749	0.9761667	0.7391071	0.4605918	0.2235322	0.342062	up
A_23_P111288	TMEM14C	5.1441936	5.546824	5.425664	0.4026303	0.2814703	0.3420503	up
A_21_P0001124	LOC101929609	-1.384613	-1.043036	-1.042159	0.3415771	0.342454	0.3420155	up
A_23_P426196	MAST3	0.4054103	0.5962234	0.8985925	0.1908131	0.4931822	0.3419976	up
A_23_P78526	CEACAM19	2.3001146	2.6204953	2.6636782	0.3203807	0.3635635	0.3419721	up
A_23_P96542	VMA21	4.2124825	4.5172973	4.5914736	0.3048148	0.3789911	0.341903	up
A_23_P13914	DHX37	3.0765371	3.3696237	3.467245	0.2930865	0.390708	0.3418973	up
A_22_P00007987	LOC101928973	1.4775229	1.7721348	1.8666267	0.2946119	0.3891039	0.3418579	up
A_33_P3272527	MAVS	0.5121903	0.9025807	0.8055072	0.3903904	0.2933168	0.3418536	up
A_23_P151653	APEX1	0.6036811	1.3009119	0.5899401	0.6972308	-0.013741	0.3417449	up
A_32_P162187	C2	-1.141358	-0.805543	-0.793837	0.335815	0.3475218	0.3416684	up
A_22_P00009165	IPO9-AS1	0.2023435	0.4589076	0.6289201	0.2565641	0.4265766	0.3415704	up
A_33_P3381454	C1orf56	0.007504	0.1929174	0.5051885	0.1854134	0.4976845	0.3415489	up
A_22_P00016661	MRGPRF-AS1	0.0271111	0.3089027	0.4284048	0.2817917	0.4012938	0.3415427	up
A_24_P134789	KRTAP10-10	0.4973049	0.7978783	0.8796339	0.3005734	0.382329	0.3414512	up

A_22_P00013517	ERICH6-AS1	0.025033	0.3529558	0.3798618	0.3279228	0.3548288	0.3413758	up
A_24_P203226	PPIAL4B	8.149369	8.548622	8.432818	0.3992529	0.2834492	0.341351	up
A_23_P67453	TNNI3	-2.058328	-1.765571	-1.668389	0.2927575	0.3899393	0.3413484	up
A_33_P3282693	OR3A4P	-1.367096	-1.015769	-1.035986	0.3513274	0.3311105	0.341219	up
A_33_P3854217	KIAA0319L	1.1931653	1.6189408	1.4496298	0.4257755	0.2564645	0.34112	up
A_33_P3306163	LGALS3	5.796468	6.2374396	6.0377245	0.4409719	0.2412567	0.3411143	up
A_33_P3357954	ETV1	-2.290274	-1.864564	-2.033775	0.4257097	0.2564993	0.3411045	up
A_24_P383999	RPS3A	7.787054	8.240593	8.015654	0.4535389	0.2285996	0.3410692	up
A_33_P3382919	LOC100130587	-2.930497	-2.720378	-2.458699	0.2101188	0.4717984	0.3409586	up
A_23_P134295	NUDT1	5.666685	6.03715	5.977972	0.3704648	0.3112869	0.3408759	up
A_23_P71946	BSPRY	4.478737	4.822204	4.816926	0.3434672	0.3381891	0.3408282	up
A_33_P3220149	MAML1	8.329469	8.752226	8.588237	0.4227572	0.2587681	0.3407626	up
A_33_P3388651	ABLIM1	3.7493067	4.204124	3.9759674	0.4548173	0.2266607	0.340739	up
A_23_P20823	WDR34	4.7786036	5.277737	4.9608717	0.4991336	0.1822681	0.3407009	up
A_23_P130158	WNT3	2.0642939	2.4091058	2.4004354	0.3448119	0.3361416	0.3404768	up
A_33_P3284404	SYNGR1	2.1066027	2.4444757	2.449562	0.337873	0.3429594	0.3404162	up
A_24_P386639	BET1L	-0.195592	0.1951213	0.0943952	0.3907137	0.2899876	0.3403506	up
A_22_P00006672	lnc-FOXO6-3	-3.052889	-2.570029	-2.855336	0.4828601	0.1975534	0.3402068	up
A_24_P378987	DHRSX	3.4532614	3.650189	3.9366198	0.1969276	0.4833584	0.340143	up
A_21_P0009753	LINC00662	-0.581809	-0.095565	-0.387795	0.4862437	0.1940141	0.3401289	up
A_32_P115130	MRPL41	5.995764	6.097919	6.5738173	0.1021552	0.5780535	0.3401044	up
A_23_P14774	CTSH	4.1355753	4.534271	4.4170666	0.3986955	0.2814913	0.3400934	up
A_23_P130836	GZMM	0.0796194	0.4383173	0.4007716	0.3586979	0.3211522	0.3399251	up
A_24_P179504	WDR66	-2.64442	-2.560485	-2.048564	0.083935	0.5958562	0.3398956	up
A_23_P146572	NPDC1	5.2469635	5.658776	5.514799	0.4118123	0.2678356	0.339824	up
A_22_P00022263	lnc-AL353597.1-1	1.5097656	1.8828797	1.816122	0.3731141	0.3063564	0.3397353	up
A_24_P122403	TCEB3	1.4520988	1.8795462	1.7041006	0.4274473	0.2520018	0.3397245	up
A_33_P3236591	RLTPR	2.0197592	2.4384027	2.280507	0.4186435	0.2607479	0.3396957	up
A_21_P0005673	LOC389641	-0.982976	-0.902041	-0.384592	0.080935	0.5983834	0.3396592	up
A_24_P26554	LYRM4	-1.209813	-0.693767	-1.046584	0.5160456	0.1632285	0.3396371	up
A_33_P3231820	ASTL	-3.462713	-2.981616	-3.264589	0.4810963	0.1981239	0.3396101	up
A_21_P0013728	FRG1	2.751583	3.0237775	3.1585493	0.2721944	0.4069662	0.3395803	up
A_33_P3231110	USP3	1.8139677	2.3542113	1.9528532	0.5402436	0.1388855	0.3395646	up
A_23_P103968	AKR7A3	2.0430279	2.2502227	2.514925	0.2071948	0.4718971	0.339546	up
A_23_P72853	MFSD5	3.1833029	3.2244706	3.8211193	0.0411677	0.6378164	0.3394921	up
A_23_P127475	CCS	2.0369072	2.4107327	2.3419485	0.3738256	0.3050413	0.3394334	up
A_22_P00006716	lnc-FRMD4B-2	-1.083941	-0.717119	-0.771905	0.3668218	0.3120356	0.3394287	up
A_23_P404685	LCE1A	4.4510593	4.738109	4.842842	0.2870498	0.3917828	0.3394163	up
A_24_P143189	TMSB4X	6.4462395	6.933659	6.6376324	0.4874196	0.1913929	0.3394063	up
A_23_P30464	PRR7	4.8093023	4.9854445	5.3118067	0.1761422	0.5025044	0.3393233	up
A_24_P280803	RPS21	9.215759	9.593828	9.516331	0.3780689	0.3005714	0.3393202	up
A_32_P4626	LOC101928076	-0.204785	0.253367	0.0153947	0.4581523	0.22018	0.3391662	up
A_33_P3278187	SLC35D2	-0.117997	0.312912	0.1293793	0.4309087	0.247376	0.3391423	up
A_24_P219785	CALM3	3.8531656	4.2951236	4.0893345	0.441958	0.2361689	0.3390634	up
A_23_P350059	PFN1P2	3.9315615	4.2140584	4.3270245	0.2824969	0.395463	0.33898	up
A_23_P377434	TNRC18	3.125514	3.6951537	3.2338247	0.5696397	0.1083107	0.3389752	up
A_23_P157513	MOS	-0.936535	-0.397125	-0.798055	0.5394096	0.1384797	0.3389447	up
A_24_P287974	CUEDC1	1.7558918	2.1877608	2.0018778	0.431869	0.245986	0.3389275	up
A_23_P148556	ABCD1	-0.373126	-0.100348	0.0316634	0.2727785	0.4047895	0.338784	up
A_24_P202581	ARHGEF1	0.572391	0.9517431	0.8704567	0.3793521	0.2980657	0.3387089	up
A_33_P3416668	VWA1	-1.309343	-1.04339	-0.897943	0.2659531	0.4114003	0.3386767	up
A_33_P3326772	LAMTOR4	6.2267885	6.479136	6.6517935	0.2523475	0.425005	0.3386762	up
A_22_P00003206	LINC01301	1.6735649	2.0502534	1.9741936	0.3766885	0.3006287	0.3386586	up
A_23_P398372	C9orf69	2.0717134	2.2560916	2.5646038	0.1843782	0.4928904	0.3386343	up
A_23_P308021	CABP4	-2.918268	-2.677815	-2.481473	0.2404528	0.436795	0.3386239	up
A_22_P00014223	RUVBL1-AS1	-1.84529	-1.460997	-1.552345	0.3842931	0.2929444	0.3386188	up

A_21_P0011499	LOC101928203	-1.371609	-0.871982	-1.194024	0.4996271	0.1775851	0.3386061	up
A_22_P00025961	lnc-CNPY3-1	-0.125119	0.2606258	0.1663375	0.3857446	0.2914562	0.3386004	up
A_33_P3272395	RAB19	-1.468222	-1.165768	-1.093518	0.3024545	0.3747039	0.3385792	up
A_22_P00011233	lnc-OR10V1-1	-2.604306	-2.183004	-2.348663	0.4213021	0.2556427	0.3384724	up
A_22_P00006864	lnc-GALM-1	-2.066832	-1.704458	-1.752302	0.3623743	0.3145299	0.3384521	up
A_22_P00024021	lnc-PRDX6-1	-1.951726	-1.569365	-1.657303	0.3823614	0.2944236	0.3383925	up
A_32_P154021	ST8SIA3	-2.695046	-2.290592	-2.422746	0.4044538	0.2723	0.3383769	up
A_23_P153461	LPPR2	-1.264123	-0.812121	-1.039536	0.4520016	0.2245865	0.338294	up
A_22_P00015085	lnc-SNURF-3	-0.904704	-0.511956	-0.620919	0.3927479	0.2837853	0.3382666	up
A_24_P173823	PBX1	2.001464	2.4230304	2.2563334	0.4215665	0.2548695	0.338218	up
A_32_P64475	SDE2	0.1952353	0.5480347	0.5184584	0.3527994	0.3232231	0.3380113	up
A_23_P5601	DOK1	3.4262724	3.8269267	3.701497	0.4006543	0.2752247	0.3379395	up
A_22_P00012377	lnc-PRICKLE4-1	-0.204288	0.2314625	0.0355959	0.435751	0.2398844	0.3378177	up
A_22_P00005910	LINC01252	-1.648333	-1.314533	-1.306498	0.3337994	0.3418346	0.337817	up
A_24_P207479	DEDD2	3.4256954	3.717462	3.8094664	0.2917666	0.3837709	0.3377688	up
A_33_P3371999	TPPP	0.2320347	0.5987158	0.5408802	0.3666811	0.3088455	0.3377633	up
A_23_P103942	DNAJC11	2.6636696	2.8573394	3.1455145	0.1936698	0.4818449	0.3377574	up
A_23_P335495	ANO7	-0.674393	-0.535811	-0.137722	0.1385818	0.5366712	0.3376265	up
A_21_P0000470	SNORD12B	-0.996886	-0.735873	-0.582719	0.261013	0.4141669	0.33759	up
A_22_P00000419	TM4SF19-AS1	-0.759775	-0.471631	-0.372814	0.2881446	0.386961	0.3375528	up
A_21_P0005915	lnc-SLC45A4-1	-0.86755	-0.594624	-0.465449	0.2729263	0.4021015	0.3375139	up
A_33_P3315303	KRT73	1.7586956	2.1117234	2.0805779	0.3530278	0.3218823	0.337455	up
A_23_P103149	ACO2	5.6398973	6.0871854	5.8673153	0.447288	0.227418	0.337353	up
A_33_P3331366	TRIM25	5.607834	5.8374677	6.052799	0.2296338	0.4449654	0.3372996	up
A_33_P3276455	KDM5B	1.7295418	2.1732154	1.9604158	0.4436736	0.2308741	0.3372738	up
A_21_P0013775	ARMCX4	-2.92001	-2.730281	-2.435307	0.189729	0.4847031	0.337216	up
A_23_P211785	ZNF35	-0.048053	0.3043156	0.2739959	0.3523684	0.3220487	0.3372085	up
A_22_P00008766	lnc-KIF16B-1	-1.698161	-1.341786	-1.380138	0.3563752	0.3180227	0.337199	up
A_24_P14731	PCSK1N	2.5551424	2.969089	2.8154907	0.4139466	0.2603483	0.3371475	up
A_21_P0011859	XLOC_l2_007586	1.4083405	1.696073	1.7947493	0.2877326	0.3864088	0.3370707	up
A_33_P3396591	PCED1A	-2.360834	-1.83603	-2.211559	0.5248041	0.1492753	0.3370397	up
A_24_P322395	EGLN2	3.126874	3.4011598	3.5266304	0.2742858	0.3997564	0.3370211	up
A_23_P28434	VAMP8	7.1319113	7.3707805	7.567071	0.2388692	0.4351597	0.3370144	up
A_24_P187651	CLN3	0.9899435	1.3718748	1.2819419	0.3819313	0.2919984	0.3369649	up
A_21_P0013501	XLOC_l2_014331	-0.960142	-0.61316	-0.633305	0.3469825	0.3268371	0.3369098	up
A_23_P303803	C19orf18	-1.719084	-1.646368	-1.11807	0.0727162	0.6010141	0.3368652	up
A_21_P0010921	GLUD1P7	-0.476285	0.0225821	-0.30143	0.4988675	0.1748557	0.3368616	up
A_33_P3395442	lnc-VAMP1-1	-2.27166	-2.09924	-1.77036	0.17242	0.5012999	0.3368599	up
A_23_P73702	MED12	0.1840062	0.3899536	0.651762	0.2059474	0.4677558	0.3368516	up
A_24_P27412	SNUPN	0.1653786	0.6129375	0.3914809	0.4475589	0.2261024	0.3368306	up
A_23_P68511	ANGPT4	-2.68764	-2.796821	-1.904854	-0.109182	0.7827852	0.3368017	up
A_24_P16815	ZMYM3	-1.317735	-0.912293	-1.04958	0.4054418	0.2681546	0.3367982	up
A_33_P3356752	GPER1	-0.749456	-0.645342	-0.180008	0.1041136	0.569448	0.3367808	up
A_21_P0011231	LINC00448	-2.461621	-2.396166	-1.853541	0.065455	0.6080799	0.3367674	up
A_23_P502575	CSNK2A1	3.8290281	4.10022	4.2313538	0.2711921	0.4023256	0.3367589	up
A_23_P255916	RPUSD3	1.7682686	2.0365596	2.1734104	0.268291	0.4051418	0.3367164	up
A_33_P3303291	RIPK3	-2.995493	-2.782627	-2.534967	0.2128658	0.4605265	0.3366962	up
A_22_P00014639	lnc-SLC17A9-1	-2.086676	-1.628003	-1.871966	0.458673	0.2147102	0.3366916	up
A_21_P0005822	lnc-TM2D2-2	-2.497824	-2.168736	-2.153543	0.3290885	0.3442814	0.3366849	up
A_23_P153692	XRCC1	4.7671013	5.2167573	4.990756	0.449656	0.2236548	0.3366554	up
A_23_P8834	EPHX2	1.6699791	2.0265841	1.9866695	0.3566051	0.3166904	0.3366477	up
A_23_P311875	CD6	-1.314762	-1.162157	-0.794086	0.1526051	0.5206761	0.3366406	up
A_23_P76102	GDF11	1.4909062	1.8314948	1.8235602	0.3405886	0.332654	0.3366213	up
A_19_P00803775	lnc-AC136604.1-1	-2.439596	-2.069532	-2.136426	0.3700643	0.3031707	0.3366175	up
A_32_P36235	IER2	8.36617	8.808151	8.5973425	0.4419813	0.2311726	0.3365769	up
A_23_P408996	MBOAT1	1.6717587	2.3928504	1.6238165	0.7210918	-0.047942	0.3365748	up

A_23_P27167	RNASEH1	2.1876364	2.652656	2.3956728	0.4650197	0.2080364	0.3365281	up
A_21_P0012260	XLOC_l2_009328	-0.816433	-0.572515	-0.387574	0.2439175	0.4288588	0.3363881	up
A_33_P3474175	SFTA1P	7.719187	8.1744175	7.9366426	0.4552307	0.2174559	0.3363433	up
A_33_P3263851	LOC400863	-0.415612	-0.129675	-0.029066	0.2859368	0.3865461	0.3362415	up
A_33_P3247489	C11orf85	-3.095369	-2.996065	-2.522323	0.099304	0.5730455	0.3361747	up
A_33_P3278200	MX2	-1.651316	-1.18184	-1.44856	0.4694758	0.2027564	0.3361161	up
A_23_P338233	BCDIN3D	2.5298643	2.9780564	2.7536745	0.4481921	0.2238102	0.3360012	up
A_22_P00018451	lnc-FRMD5-1	0.6380458	1.052794	0.8952413	0.4147482	0.2571955	0.3359718	up
A_24_P318897	SNX21	0.3401361	0.7830253	0.5691171	0.4428892	0.228981	0.3359351	up
A_33_P3250055	MAPK12	-3.019225	-2.481897	-2.884711	0.5373285	0.1345143	0.3359214	up
A_22_P00018345	ZNF205-AS1	-0.395899	-0.058146	-0.062021	0.3377528	0.3338785	0.3358157	up
A_33_P3333455	EMILIN1	1.6042423	2.0324082	1.8476276	0.4281659	0.2433853	0.3357756	up
A_33_P3266419	GLTP	0.3184409	0.6989479	0.6094804	0.380507	0.2910395	0.3357732	up
A_33_P3386506	NFKBIL1	6.926198	7.3928294	7.130945	0.4666314	0.2047472	0.3356893	up
A_23_P8558	ABHD11	1.1956038	1.6952562	1.3672962	0.4996524	0.1716924	0.3356724	up
A_33_P3361891	TMPRSS7	-2.737851	-1.838086	-2.966321	0.899765	-0.22847	0.3356475	up
A_23_P30884	CLIC1	5.7704363	6.2352424	5.976694	0.4648061	0.2062578	0.335532	up
A_23_P35055	NPHS2	-2.190514	-1.540018	-2.169983	0.650496	0.0205302	0.3355131	up
A_23_P253841	HSF1	7.339369	7.7308106	7.618951	0.3914418	0.279582	0.3355119	up
A_22_P00017386	lnc-VAMP1-1	-2.169837	-1.71528	-1.953536	0.4545565	0.2163005	0.3354285	up
A_32_P46981	HSBP1L1	2.6404018	3.103497	2.8480167	0.4630952	0.2076149	0.3353551	up
A_33_P7293840	LINC00271	-3.193459	-3.101495	-2.614991	0.0919645	0.5784681	0.3352163	up
A_23_P205489	SLC7A8	0.3523016	0.79527	0.5797153	0.4429684	0.2274137	0.335191	up
A_23_P168541	C7orf26	2.2354918	2.508245	2.6330633	0.2727532	0.3975716	0.3351624	up
A_23_P200710	PIK3C2B	4.740283	5.1063886	5.0443277	0.3661056	0.3040447	0.3350751	up
A_24_P99216	LRP10	0.7658873	1.1962342	1.0056434	0.430347	0.2397561	0.3350515	up
A_24_P49214	C1orf86	0.7989211	1.1830816	1.084825	0.3841605	0.2859039	0.3350322	up
A_24_P32520	RNF214	0.667676	0.9853354	1.0197177	0.3176594	0.3520417	0.3348506	up
A_23_P50389	NAT14	2.305356	2.6886458	2.5916586	0.3832898	0.2863026	0.3347962	up
A_23_P120845	XBP1	5.65355	6.204862	5.7716455	0.551312	0.1180954	0.3347037	up
A_23_P129659	ZNF689	-0.158061	0.3203969	0.032835	0.4784575	0.1908956	0.3346765	up
A_23_P434518	LFNG	0.4114289	0.7448692	0.7472959	0.3334403	0.3358669	0.3346536	up
A_23_P82588	C7orf55	1.6191349	1.8709149	2.0366335	0.25178	0.4174986	0.3346393	up
A_22_P00004480	LINC00887	1.1968479	1.6505499	1.4123197	0.453702	0.2154717	0.3345869	up
A_33_P3275235	AKT1	2.587408	2.9385324	2.905427	0.3511243	0.3180189	0.3345716	up
A_24_P376339	CCNL2	1.6265087	1.9313293	1.9907541	0.3048205	0.3642454	0.334533	up
A_23_P36157	WDR74	3.7909307	4.100928	4.1498938	0.3099971	0.358963	0.33448	up
A_32_P150300	LOC101928370	-1.884646	-1.55577	-1.544578	0.3288765	0.3400688	0.3344727	up
A_33_P3411388	UNCX	4.8960266	5.3317685	5.1292124	0.4357419	0.2331858	0.3344638	up
A_23_P18205	RAD54L2	1.3983917	1.6668496	1.7985973	0.2684579	0.4002056	0.3343318	up
A_23_P109235	RALY	3.303957	3.4556222	3.820858	0.1516652	0.516901	0.3342831	up
A_23_P203994	PSMD9	5.2350483	5.669908	5.468667	0.4348598	0.2336187	0.3342392	up
A_23_P206830	PAM16	3.575776	3.8182683	4.0017548	0.2424922	0.4259787	0.3342354	up
A_22_P00011487	OPLAH	-0.715745	-0.44272	-0.320589	0.2730246	0.3951559	0.3340902	up
A_33_P3252915	RNF7	-1.628076	-1.496611	-1.091556	0.131465	0.53652	0.3339925	up
A_24_P391568	ARNT	-0.880332	-0.448459	-0.64425	0.4318728	0.2360816	0.3339772	up
A_21_P0011235	LINC00454	0.6854119	1.0745277	0.9641461	0.3891158	0.2787342	0.333925	up
A_24_P205589	ACOT7	4.2225523	4.3605323	4.7523737	0.13798	0.5298214	0.3339007	up
A_23_P250122	FAM20C	-0.704628	-0.458492	-0.283096	0.2461357	0.4215322	0.3338339	up
A_23_P78685	FARSA	3.8381958	4.001348	4.342663	0.1631522	0.504467	0.3338096	up
A_23_P62764	CCDC28B	0.1714306	0.2733302	0.7370477	0.1018996	0.5656171	0.3337584	up
A_33_P3224745	PLA2G15	4.9922905	5.2874446	5.36444	0.2951541	0.3721495	0.3336518	up
A_33_P3228266	CST3	5.1565533	5.416352	5.563944	0.2597985	0.4073906	0.3335946	up
A_23_P53126	LMO2	2.3928347	2.3044124	3.1484318	-0.088422	0.7555971	0.3335874	up
A_23_P68087	ATIC	4.478133	4.901851	4.7213783	0.423718	0.2432451	0.3334816	up
A_22_P00022873	LOC102724532	2.4103403	2.7355695	2.752039	0.3252292	0.3416987	0.3334639	up

A_23_P42829	SND1	5.140254	5.5689893	5.378228	0.4287353	0.2379742	0.3333547	up
A_33_P3360665	ACVR1	-2.001839	-1.460918	-1.876099	0.5409207	0.1257401	0.3333304	up
A_23_P4611	SLC27A5	5.3459797	5.5114045	5.847088	0.1654248	0.5011082	0.3332665	up
A_21_P0003244	lnc-EPHA6-1	0.7337508	1.0826716	1.0513592	0.3489208	0.3176084	0.3332646	up
A_33_P3256272	KRTAP10-5	-2.52086	-1.928865	-2.446528	0.5919952	0.074332	0.3331636	up
A_21_P0007885	LOC643770	-1.299685	-1.131038	-0.802245	0.1686463	0.4974399	0.3330431	up
A_23_P97296	NECAP2	3.0736828	3.1527038	3.66074	0.079021	0.5870571	0.333039	up
A_23_P143535	WDR4	0.836401	1.1057706	1.2330656	0.2693696	0.3966646	0.3330171	up
A_23_P138665	GLUD1	4.2042418	4.4627404	4.611575	0.2584987	0.4073334	0.332916	up
A_23_P2114	LAMTOR1	2.6359453	2.9277492	3.009879	0.2918038	0.3739338	0.3328688	up
A_33_P3289845	IGFL1	2.3658676	2.966682	2.4307365	0.6008143	0.0648689	0.3328416	up
A_33_P3397785	SYT2	-3.17933	-2.696026	-2.997009	0.483304	0.1823206	0.3328123	up
A_23_P132358	MCAT	2.482191	2.9036288	2.7263699	0.4214377	0.2441788	0.3328083	up
A_23_P70701	ARID1B	1.9354181	2.3910222	2.1452856	0.4556041	0.2098675	0.3327358	up
A_24_P210829	NME4	1.7074776	1.9224582	2.1578302	0.2149806	0.4503527	0.3326666	up
A_33_P3230219	TMEM54	7.2733784	7.5663133	7.6456537	0.2929349	0.3722754	0.3326051	up
A_32_P33434	ZNF812	-2.474265	-2.102829	-2.180538	0.3714364	0.2937274	0.3325819	up
A_24_P343255	KLHDC3	1.2141442	1.6244464	1.4688435	0.4103022	0.2546992	0.3325007	up
A_32_P176018	ACTL8	6.3027306	6.561293	6.709141	0.2585626	0.4064102	0.3324864	up
A_23_P53039	LDHC	-2.425111	-2.118247	-2.067022	0.3068638	0.358089	0.3324764	up
A_33_P3362952	lnc-TBC1D29-1	-0.173465	0.1616921	0.1561728	0.3351574	0.329638	0.3323977	up
A_21_P0009433	lnc-USP14-5	-2.279436	-1.9227	-1.97147	0.3567352	0.3079658	0.3323505	up
A_22_P00009403	LUZP1	-1.929709	-1.517682	-1.677103	0.4120274	0.2526064	0.3323169	up
A_23_P1505	LRP5	1.1860495	1.415524	1.6211252	0.2294745	0.4350758	0.3322752	up
A_23_P208900	SEMA6B	-0.744857	-0.217438	-0.607856	0.5274191	0.137001	0.3322101	up
A_23_P320261	DMKN	6.247466	6.652695	6.506633	0.4052291	0.2591667	0.3321979	up
A_22_P00002391	LOC102724153	-2.016857	-1.677485	-1.691842	0.3393722	0.3250146	0.3321934	up
A_33_P3303649	MB	-0.694129	0.0458307	-0.769729	0.7399597	-0.0756	0.33218	up
A_22_P00008019	LOC101929089	-1.464452	-1.16548	-1.099227	0.2989717	0.3652253	0.3320985	up
A_23_P133974	C6orf106	1.9684916	2.1324754	2.4686537	0.1639838	0.5001621	0.332073	up
A_33_P3258660	SCD5	-1.212123	-1.053466	-0.706681	0.1586576	0.5054421	0.3320498	up
A_23_P105028	ATL3	3.0241423	3.3805356	3.3317642	0.3563933	0.307622	0.3320077	up
A_22_P00007674	PCAT7	-1.435581	-0.963438	-1.243947	0.4721437	0.1916342	0.3318889	up
A_22_P00022179	ARHGEF2	-2.633471	-2.264251	-2.339035	0.3692205	0.2944362	0.3318284	up
A_23_P301340	ENTHD2	4.1781616	4.548548	4.471425	0.3703866	0.2932634	0.331825	up
A_32_P25437	SLC12A2	2.8881664	3.0497565	3.3902245	0.1615901	0.502058	0.3318241	up
A_33_P3417432	LOC100129098	-1.764956	-1.523985	-1.342398	0.2409711	0.4225583	0.3317647	up
A_22_P00010068	LOC101927204	0.114996	0.4004774	0.4930162	0.2854815	0.3780203	0.3317509	up
A_22_P00006006	LOC283332	-2.335529	-1.917649	-2.090163	0.4178796	0.2453656	0.3316226	up
A_33_P3225096	MTMR10	-0.211626	0.1108365	0.1291461	0.3224626	0.3407722	0.3316174	up
A_23_P13604	PEBP1	7.225171	7.5123506	7.6008596	0.2871795	0.3756886	0.331434	up
A_22_P00000083	PANDAR	-3.409189	-3.030731	-3.125299	0.3784573	0.2838903	0.3311738	up
A_23_P26457	HBA2	-1.266222	-1.113991	-0.756111	0.1522307	0.5101113	0.331171	up
A_33_P3339051	ZNF428	4.8758707	5.1181045	5.295908	0.2422338	0.4200373	0.3311355	up
A_33_P3219578	ZIC1	-1.391931	-0.963475	-1.158311	0.4284558	0.2336192	0.3310375	up
A_23_P160849	FCER1G	0.9632912	1.1789379	1.4096327	0.2156467	0.4463415	0.3309941	up
A_23_P103276	KTI12	0.5549245	0.7941217	0.9775324	0.2391973	0.4226079	0.3309026	up
A_33_P3384721	NDUFB8	6.5406504	6.879724	6.8632545	0.3390737	0.3226042	0.3308389	up
A_23_P348298	SAC3D1	3.6849794	4.0672126	3.9643087	0.3822331	0.2793293	0.3307812	up
A_24_P200694	RING1	0.4678392	0.964942	0.6321445	0.4971027	0.1643052	0.330704	up
A_21_P0008403	lnc-C14orf79-2	1.270288	1.5363369	1.665483	0.2660489	0.395195	0.330622	up
A_33_P3370076	TFE3	2.2489862	2.654756	2.5044289	0.4057698	0.2554426	0.3306062	up
A_23_P131626	SNRNP200	5.3490553	5.756579	5.6027193	0.4075236	0.253664	0.3305938	up
A_23_P429461	FUK	1.8757267	2.176641	2.235958	0.3009143	0.3602314	0.3305729	up
A_33_P3767773	UBR4	5.147587	5.6109214	5.3453417	0.4633346	0.1977549	0.3305447	up
A_33_P3339036	MECP2	2.401824	2.7686496	2.696002	0.3668256	0.294178	0.3305018	up

A_33_P3354404	KRTAP12-2	-0.219703	0.2068954	0.0146708	0.4265986	0.2343741	0.3304863	up
A_22_P00017823	lnc-ZC3H12B-3	-2.636165	-2.535928	-2.075481	0.1002367	0.5606835	0.3304601	up
A_23_P165007	RASGRP4	-1.452112	-1.145207	-1.098273	0.3069053	0.3538389	0.3303721	up
A_22_P00023546	lnc-PANK4-1	-1.328073	-0.967036	-1.028498	0.3610363	0.2995744	0.3303053	up
A_23_P68072	WDR54	3.8268614	4.0567517	4.2575817	0.2298904	0.4307203	0.3303053	up
A_22_P00022127	lnc-TNFRSF17-2	-1.493392	-1.083686	-1.242619	0.4097056	0.250773	0.3302393	up
A_33_P3411080	FSCN2	-1.32222	-1.001286	-0.982868	0.3209343	0.3393526	0.3301435	up
A_23_P206733	CES1	-0.707424	-0.411342	-0.343271	0.296082	0.3641529	0.3301175	up
A_21_P0000654	LOC646938	-1.586008	-1.149218	-1.36259	0.43679	0.2234178	0.3301039	up
A_24_P241318	DCAF4	2.656744	3.1322837	2.8413029	0.4755397	0.1845589	0.3300493	up
A_23_P67466	PSMD8	6.6476173	6.9946504	6.960681	0.347033	0.3130636	0.3300483	up
A_23_P91350	GPCPD1	0.8797154	1.4034	1.0160766	0.5236845	0.1363611	0.3300228	up
A_22_P00005311	lnc-DNAJC7-1	0.8560014	1.1008592	1.2711735	0.2448578	0.4151721	0.3300149	up
A_33_P3237207	C2orf72	-1.606482	-1.161065	-1.39212	0.4454169	0.2143621	0.3298895	up
A_32_P159612	ASIC2	-2.176776	-1.792602	-1.901172	0.3841734	0.2756033	0.3298884	up
A_19_P00809902	LINC01006	2.0604334	2.4373755	2.3431396	0.3769422	0.2827063	0.3298242	up
A_33_P3791123	ATP5L2	6.968911	7.217035	7.3804035	0.2481237	0.4114924	0.329808	up
A_33_P3240941	BACE1	0.6737256	1.0737743	0.9332752	0.4000487	0.2595496	0.3297992	up
A_23_P56314	UQCR11	7.600794	7.9853578	7.8755608	0.3845639	0.2747669	0.3296654	up
A_21_P0011494	XLOC_l2_005606	-2.059149	-1.601712	-1.85737	0.457437	0.2017789	0.329608	up
A_24_P31421	GLTSCR1	-2.084816	-1.780249	-1.730236	0.3045664	0.3545795	0.3295729	up
A_33_P3258472	SPTBN1	-3.048451	-3.057301	-2.380544	-0.00885	0.6679077	0.329529	up
A_23_P406350	MFSD3	2.2366352	2.4585385	2.6737328	0.2219033	0.4370976	0.3295004	up
A_22_P00022503	lnc-PUM2-1	-2.103746	-1.682031	-1.866598	0.4217157	0.2371483	0.329432	up
A_22_P00003927	lnc-CETP-1	-1.997763	-1.33726	-1.999493	0.6605024	-0.00173	0.329386	up
A_23_P432360	PMM2	3.613163	3.889546	3.9954796	0.2763829	0.3823166	0.3293498	up
A_23_P33791	SSBP2	0.3065457	0.6894889	0.5822158	0.3829432	0.2756701	0.3293066	up
A_22_P00001459	lnc-AQP11-2	1.497611	1.8793488	1.7744083	0.3817377	0.2767973	0.3292675	up
A_23_P22263	BANP	4.8677187	5.209019	5.184907	0.3413005	0.3171883	0.3292444	up
A_23_P314250	FAM78A	-0.10467	0.0465412	0.4026074	0.1512108	0.507277	0.3292439	up
A_21_P0013386	INTS4	0.9185853	1.3274941	1.1680279	0.4089088	0.2494426	0.3291757	up
A_24_P340679	PPIA	7.994692	8.383785	8.263873	0.3890934	0.2691813	0.3291373	up
A_32_P360193	DNHD1	-2.586953	-2.312965	-2.20289	0.273988	0.3840637	0.3290259	up
A_22_P00006968	OSER1-AS1	-0.28432	0.0858007	0.003571	0.3701205	0.2878909	0.3290057	up
A_23_P85441	IGSF9	4.433693	4.906774	4.6185637	0.4730811	0.1848707	0.3289759	up
A_24_P336848	ACYP2	1.9306526	2.3526511	2.1665535	0.4219985	0.2359009	0.3289497	up
A_23_P329772	VPS4A	4.3370724	4.6005187	4.7315054	0.2634463	0.394433	0.3289397	up
A_22_P00013349	LOC100507420	-3.28638	-2.946306	-2.968664	0.3400743	0.3177159	0.3288951	up
A_24_P97770	ERGIC1	-1.743199	-1.462371	-1.366261	0.2808285	0.3769383	0.3288834	up
A_33_P3209214	TNRC18	-1.463489	-1.114634	-1.154732	0.348855	0.3087573	0.3288062	up
A_32_P41487	HMGN2	8.394594	8.856333	8.590412	0.4617386	0.195818	0.3287783	up
A_23_P401675	MARVELD2	3.5874367	3.7287927	4.1035814	0.141356	0.5161448	0.3287504	up
A_33_P3622472	NAA10	3.017662	3.2946696	3.3981266	0.2770076	0.3804646	0.3287361	up
A_24_P317827	TMEM8B	-1.961951	-1.601646	-1.664884	0.3603053	0.2970667	0.328686	up
A_23_P20980	CYC1	5.576006	5.901448	5.9079237	0.3254418	0.3319178	0.3286798	up
A_23_P44195	MSI2	2.2536793	2.5144124	2.650281	0.2607331	0.3966017	0.3286674	up
A_33_P3374504	TXNRD2	-0.587862	-0.066281	-0.452545	0.5215802	0.1353164	0.3284483	up
A_32_P53486	BOLA2B	7.0418463	7.3153815	7.4252033	0.2735353	0.3833571	0.3284462	up
A_23_P43034	ELP3	2.1023855	2.5473237	2.3142958	0.4449382	0.2119103	0.3284242	up
A_24_P122636	BPNT1	-0.887731	-0.625876	-0.492766	0.2618556	0.3949647	0.3284102	up
A_23_P301247	HIST2H2AC	5.3102474	5.573046	5.704221	0.2627988	0.3939734	0.3283861	up
A_33_P3238966	NPLOC4	5.864231	6.0663075	6.3188696	0.2020764	0.4546385	0.3283575	up
A_23_P211878	FLNB	3.023919	3.5145087	3.1898785	0.4905896	0.1659594	0.3282745	up
A_23_P55990	NAPA	2.1133795	2.373385	2.5098772	0.2600055	0.3964977	0.3282516	up
A_23_P5089	ATP5D	4.4087563	4.8344026	4.639599	0.4256463	0.2308426	0.3282444	up
A_22_P00023617	LINC01063	-0.382401	-0.237473	0.1290836	0.1449285	0.5114851	0.3282068	up

A_23_P113005	EFNA1	1.1937103	1.800035	1.2437553	0.6063247	0.050045	0.3281849	up
A_24_P264207	PTMA	9.718642	10.043211	10.050385	0.3245688	0.3317423	0.3281555	up
A_23_P145146	PDCD2	5.376546	5.725655	5.683586	0.3491092	0.3070402	0.3280747	up
A_23_P337875	PAQR3	-2.099004	-1.650752	-1.891416	0.4482527	0.2075887	0.3279207	up
A_33_P3306192	KBTBD13	1.1428595	1.5364375	1.4051094	0.3935781	0.26225	0.327914	up
A_21_P0000610	LINC01060	-2.178251	-1.812317	-1.888489	0.3659339	0.2897625	0.3278482	up
A_23_P33558	LOC729164	-3.081723	-2.687984	-2.819786	0.3937388	0.2619369	0.3278378	up
A_33_P6671337	ZCCHC18	-0.620541	-0.142461	-0.443021	0.4780803	0.1775198	0.3278	up
A_23_P23728	SV2A	-0.811421	-0.386444	-0.580802	0.4249773	0.2306199	0.3277986	up
A_22_P00008114	MYOM3	-1.011889	-0.566566	-0.801951	0.4453225	0.2099381	0.3276303	up
A_24_P303874	C9orf62	4.638673	5.091418	4.841159	0.452745	0.202486	0.3276155	up
A_23_P104046	BPNT1	2.5107136	2.924602	2.7518845	0.4138885	0.2411709	0.3275297	up
A_22_P00016395	lnc-TMEM189-UBE2V1-3	-2.030016	-1.573916	-1.831057	0.4560995	0.1989589	0.3275292	up
A_22_P00004304	lnc-CNTLN-2	2.234336	2.4599805	2.6637325	0.2256446	0.4293966	0.3275206	up
A_22_P00017487	lnc-VWA3B-1	-1.70958	-1.059221	-1.704983	0.6503587	0.0045962	0.3274775	up
A_23_P40718	PARVB	1.0537443	1.3902254	1.3721557	0.3364811	0.3184114	0.3274462	up
A_22_P00014825	lnc-SLC43A1-1	-0.547457	-0.271502	-0.16854	0.2759552	0.3789177	0.3274365	up
A_24_P126305	ZNF500	1.862258	2.2757487	2.1036348	0.4134908	0.2413769	0.3274338	up
A_23_P47282	ST14	4.484229	4.7433543	4.8798695	0.2591252	0.3956404	0.3273828	up
A_32_P220715	MAP1LC3B	4.164797	4.669639	4.314643	0.5048423	0.1498461	0.3273442	up
A_23_P55342	DVL2	1.6643505	2.070271	1.913002	0.4059205	0.2486515	0.327286	up
A_22_P00001849	PRICKLE2-AS1	-2.83603	-2.655059	-2.362429	0.1809707	0.4736004	0.3272855	up
A_33_P3406281	SAMHD1	-0.517138	-0.336047	-0.043762	0.1810904	0.4733758	0.3272331	up
A_33_P3308905	CEP57L1	-1.777865	-1.655236	-1.246044	0.1226296	0.5318213	0.3272254	up
A_21_P0000359	SNORA71D	1.188448	1.527863	1.5034685	0.3394151	0.3150206	0.3272178	up
A_22_P00024462	RARA-AS1	0.630496	0.9801631	0.9352589	0.3496671	0.3047628	0.327215	up
A_33_P3366903	CHST6	3.1589413	3.5605178	3.4117775	0.4015765	0.2528362	0.3272064	up
A_21_P0005195	lnc-GNB2-1	-2.495743	-2.140119	-2.197005	0.3556235	0.2987375	0.3271805	up
A_33_P3228837	CD8A	-0.610596	-0.241627	-0.325316	0.368969	0.2852798	0.3271244	up
A_33_P3286724	PLD2	1.7879677	2.0810103	2.1488476	0.2930427	0.3608799	0.3269613	up
A_23_P9255	SYK	4.110116	4.5438075	4.330344	0.4336915	0.2202282	0.3269599	up
A_24_P113686	ZNHIT6	2.1287756	2.2882447	2.6230917	0.1594691	0.4943161	0.3268926	up
A_33_P3250128	SNX18	2.38591	2.6741958	2.75132	0.2882857	0.3654099	0.3268478	up
A_23_P79134	PLEKHF1	-0.64519	-0.289487	-0.34721	0.3557024	0.2979798	0.3268411	up
A_21_P0010806	XLOC_l2_001496	-1.579771	-1.326169	-1.179711	0.253602	0.4000597	0.3268309	up
A_24_P42624	UNC5C	-3.323962	-2.806071	-3.18831	0.5178909	0.1356514	0.3267711	up
A_22_P00003271	LOC101927043	-1.019519	-0.43809	-0.947461	0.5814285	0.0720577	0.3267431	up
A_32_P8361	MAU2	2.8460865	3.2453132	3.1001196	0.3992267	0.2540331	0.3266299	up
A_21_P0007536	lnc-LRMP-4	-3.372343	-2.91065	-3.18087	0.4616931	0.1914728	0.3265829	up
A_23_P55468	TIMM22	1.3982964	1.7101355	1.7395172	0.3118391	0.3412209	0.32653	up
A_33_P3378915	ARHGEF18	-1.138434	-0.750244	-0.873677	0.3881898	0.2647567	0.3264732	up
A_33_P3311551	WLS	0.0755386	0.4790893	0.3249059	0.4035506	0.2493672	0.3264589	up
A_21_P0014295	PIGBOS1	0.4590788	0.743433	0.8275642	0.2843542	0.3684855	0.3264198	up
A_33_P3709317	SNORA28	1.3784738	1.6081223	1.8016415	0.2296486	0.4231677	0.3264081	up
A_22_P00016248	lnc-TMCO3-1	0.3243332	0.663249	0.6381111	0.3389158	0.3137779	0.3263469	up
A_19_P00805679	LOC730102	-0.309533	0.1044335	-0.07081	0.4139662	0.2387223	0.3263443	up
A_33_P3229196	CD151	7.4555817	7.6947284	7.869006	0.2391467	0.4134245	0.3262856	up
A_23_P26713	RPL23	10.530629	10.915348	10.798443	0.3847189	0.2678137	0.3262663	up
A_33_P3760937	LOC497256	-1.488799	-1.145812	-1.179349	0.3429866	0.3094497	0.3262181	up
A_24_P181672	B3GNTL1	-0.882044	-0.638371	-0.47333	0.2436729	0.4087148	0.3261938	up
A_23_P210726	CDC25B	3.0387678	3.6946921	3.0351572	0.6559243	-0.003611	0.3261568	up
A_24_P28811	CYP4F62P	0.5782399	1.0965691	0.7119942	0.5183291	0.1337543	0.3260417	up
A_23_P500601	TRIM4	3.201024	3.5850358	3.4690876	0.3840118	0.2680636	0.3260377	up
A_33_P3368375	RANBP3	0.0695519	0.5496359	0.2414603	0.4800839	0.1719084	0.3259962	up
A_33_P3420904	PATE3	-1.724693	-1.260107	-1.537296	0.4645867	0.187397	0.3259919	up

A_23_P117694	CORO2B	-3.225251	-2.457028	-3.341595	0.7682228	-0.116344	0.3259393	up
A_22_P00018555	ZNF569	-1.450573	-1.076	-1.173527	0.3745728	0.2770453	0.325809	up
A_33_P3272399	LOC645427	-2.168339	-2.047679	-1.637502	0.1206608	0.5308375	0.3257492	up
A_21_P0009551	lnc-RIT2-1	1.3645964	1.7186246	1.6620088	0.3540282	0.2974124	0.3257203	up
A_24_P49383	AAMDC	3.115058	3.5028815	3.378541	0.3878236	0.2634831	0.3256533	up
A_23_P215484	CCL26	-2.277365	-1.580136	-2.323389	0.6972289	-0.046024	0.3256024	up
A_23_P254816	TCF15	-0.490631	-0.149906	-0.180161	0.340725	0.3104701	0.3255975	up
A_21_P0012457	XLOC_l2_010330	-1.956669	-1.683857	-1.578622	0.2728124	0.3780465	0.3254294	up
A_23_P73420	TRAPPC9	1.9468355	2.0329556	2.5115576	0.0861201	0.5647221	0.3254211	up
A_33_P3355608	PCDHA7	1.3072386	1.6087775	1.6565323	0.3015389	0.3492937	0.3254163	up
A_33_P3383326	LPAR1	-2.574016	-2.306681	-2.190607	0.2673352	0.3834093	0.3253722	up
A_23_P56228	GMIP	2.1719112	2.2923465	2.7021866	0.1204352	0.5302753	0.3253553	up
A_23_P256933	RPL24	9.363899	9.748552	9.629954	0.3846531	0.2660551	0.3253541	up
A_23_P397055	CHPF2	-0.496398	-0.062275	-0.279933	0.4341235	0.216466	0.3252947	up
A_24_P135427	LOC100128398	-3.167222	-2.761325	-2.92253	0.4058971	0.2446919	0.3252945	up
A_23_P55731	CIC	2.1628027	2.6421762	2.3339357	0.4793735	0.171133	0.3252533	up
A_23_P96833	SLAMF9	-0.495008	-0.187883	-0.151659	0.3071241	0.3433485	0.3252363	up
A_23_P54376	STOML1	2.929494	3.3358798	3.1735077	0.4063859	0.2440138	0.3251998	up
A_33_P3217480	HIRIP3	3.4481564	3.7929025	3.7537308	0.3447461	0.3055744	0.3251603	up
A_23_P133770	CCDC167	2.534216	2.6630912	3.0556335	0.1288753	0.5214176	0.3251464	up
A_33_P3246883	DMKN	4.2594976	4.68768	4.4815187	0.4281821	0.2220211	0.3251016	up
A_23_P68121	PSD4	1.2173662	1.5998254	1.4850469	0.3824592	0.2676806	0.3250699	up
A_23_P88046	CARKD	2.918415	3.3803267	3.1064262	0.4619117	0.1880112	0.3249614	up
A_33_P3294821	OTOP1	-2.133528	-1.660557	-1.956843	0.472971	0.1766853	0.3248281	up
A_22_P00011996	lnc-PLCD1-1	-3.087502	-2.539465	-2.98589	0.5480378	0.1016123	0.3248251	up
A_22_P00020161	lnc-BAIAP2-1	-1.999344	-1.672628	-1.676498	0.326716	0.3228464	0.3247812	up
A_19_P00315551	LOC400043	0.9049497	1.4328647	1.02632	0.527915	0.1213703	0.3246427	up
A_23_P33720	FARS2	-0.045553	0.20119	0.3569489	0.2467427	0.4025016	0.3246222	up
A_23_P51660	MUTYH	3.0318365	3.377334	3.3354053	0.3454976	0.3035688	0.3245332	up
A_23_P149496	XKR8	3.357688	3.5270486	3.8372536	0.1693606	0.4795656	0.3244631	up
A_32_P154380	lnc-SLC25A2-1	-2.811776	-2.429213	-2.545825	0.3825629	0.2659507	0.3242568	up
A_23_P352870	PVRL2	1.8254414	2.0852714	2.2140484	0.25983	0.388607	0.3242185	up
A_24_P270890	SPOP	2.458788	2.724506	2.8414974	0.265718	0.3827095	0.3242137	up
A_33_P3253144	DOK3	-2.203949	-1.676561	-2.082916	0.5273881	0.1210332	0.3242106	up
A_23_P34956	MRPL9	5.6488924	6.00083	5.9453106	0.3519378	0.2964182	0.324178	up
A_22_P00014730	lnc-SLC2A4RG-1	-1.368268	-1.146157	-0.942218	0.2221103	0.4260492	0.3240798	up
A_22_P00023975	PAQR9-AS1	-1.0643	-0.829971	-0.650703	0.2343283	0.4135962	0.3239622	up
A_23_P78563	UBL5	7.477953	7.6676364	7.9360437	0.1896834	0.4580908	0.3238871	up
A_23_P47116	RASSF7	1.0924544	1.5539598	1.2783561	0.4615054	0.1859016	0.3237035	up
A_21_P0008784	FAM174B	-2.765694	-2.432022	-2.452078	0.3336716	0.3136156	0.3236436	up
A_23_P250380	MAN2B2	-1.166735	-0.592021	-1.094244	0.5747142	0.0724912	0.3236027	up
A_33_P3289128	ZBTB42	0.8516607	1.0863857	1.2640676	0.234725	0.4124069	0.323566	up
A_33_P3508822	APP	6.008585	6.2686524	6.3954477	0.2600675	0.3868628	0.3234651	up
A_33_P3356607	WIPI2	4.2484207	4.556191	4.5875607	0.3077703	0.3391399	0.3234551	up
A_23_P251562	TUSC2	4.0963745	4.535853	4.3038044	0.4394784	0.2074299	0.3234541	up
A_23_P219197	RGS3	3.3866024	3.6178098	3.802186	0.2312074	0.4155836	0.3233955	up
A_21_P0000251	SNORA65	2.3652477	2.6503577	2.7267818	0.28511	0.3615341	0.3233221	up
A_23_P13701	TMBIM4	1.4760804	1.8445821	1.7540822	0.3685017	0.2780018	0.3232517	up
A_22_P00024147	lnc-CPPED1-3	-2.892864	-2.547064	-2.592422	0.3458002	0.3004425	0.3231213	up
A_23_P417200	TERF2	0.6764846	1.1117597	0.8872614	0.4352751	0.2107768	0.3230259	up
A_33_P3620488	CATSPERG	-0.177538	0.1605258	0.1302776	0.3380642	0.307816	0.3229401	up
A_23_P314798	DCST2	-1.831621	-1.488109	-1.529395	0.3435121	0.3022261	0.3228691	up
A_33_P3335451	PARL	6.6280823	6.9667344	6.935026	0.3386521	0.3069439	0.322798	up
A_23_P18447	PPARGC1A	-2.68218	-2.267809	-2.451112	0.4143715	0.2310689	0.3227202	up
A_23_P14876	SRP14	8.693394	9.01734	9.014881	0.323946	0.3214874	0.3227167	up
A_33_P3309999	DCAF12L2	-0.847797	-0.334955	-0.715214	0.5128417	0.1325827	0.3227122	up

A_23_P68505	FAM217B	3.225912	3.502254	3.594942	0.2763419	0.36903	0.322686	up
A_33_P3384562	PDLIM7	4.028367	4.510102	4.191947	0.4817348	0.1635799	0.3226573	up
A_23_P103720	AGMAT	1.5289049	1.834177	1.868926	0.3052721	0.3400211	0.3226466	up
A_23_P630	MSTO1	-0.650353	-0.386586	-0.269041	0.2637668	0.3813114	0.3225391	up
A_24_P185205	TP53I13	0.553885	0.9560838	0.7967238	0.4021988	0.2428389	0.3225188	up
A_33_P3423740	TMEM164	-1.919607	-1.414648	-1.77968	0.5049586	0.1399264	0.3224425	up
A_23_P134650	PTCD1	4.427329	4.735086	4.7643166	0.3077569	0.3369875	0.3223722	up
A_22_P00008573	lnc-KCNT1-1	-2.525155	-2.155977	-2.249641	0.3691781	0.2755144	0.3223462	up
A_23_P123164	OR6W1P	-2.40532	-2.116485	-2.04947	0.2888346	0.3558497	0.3223422	up
A_21_P0001428	lnc-AC092811.1-1	-2.574444	-2.343005	-2.161221	0.2314384	0.413223	0.3223307	up
A_23_P142310	MKNK2	6.687422	7.226168	6.7931395	0.5387464	0.1057177	0.322232	up
A_33_P3403666	NDOR1	-3.000786	-2.756025	-2.601113	0.244761	0.3996732	0.3222171	up
A_23_P163235	CKMT1A	3.9497194	4.288548	4.2552423	0.3388286	0.3055229	0.3221757	up
A_23_P112859	CST1	-0.712865	-0.251396	-0.530375	0.4614697	0.1824908	0.3219802	up
A_33_P3299314	RPL28	4.5541277	5.141908	4.6102962	0.5877805	0.0561686	0.3219745	up
A_21_P0010088	LOC101927326	2.7597342	3.0884538	3.0748634	0.3287196	0.3151293	0.3219245	up
A_22_P00001348	LINC00667	0.4793057	0.8275518	0.7748971	0.3482461	0.2955914	0.3219187	up
A_33_P3316555	MGST3	6.480706	6.8760734	6.7291355	0.3953672	0.2484293	0.3218982	up
A_33_P3413227	LOC100132363	-1.29483	-0.931788	-1.014182	0.3630414	0.2806482	0.3218448	up
A_21_P0000509	SNAR-G2	9.953706	10.295933	10.255077	0.342227	0.3013716	0.3217993	up
A_21_P0000508	SNAR-D	10.332496	10.702745	10.605747	0.3702498	0.2732515	0.3217506	up
A_33_P3265030	GP1BB	0.1940246	0.4601088	0.5713954	0.2660842	0.3773708	0.3217275	up
A_33_P3405068	NAV1	0.3797464	0.5779614	0.8248944	0.198215	0.445148	0.3216815	up
A_24_P194154	UPF1	5.90265	6.0648303	6.3838234	0.1621804	0.4811735	0.321677	up
A_24_P396197	PRKCSH	1.9858007	2.3310246	2.283926	0.3452239	0.2981253	0.3216746	up
A_23_P316381	ACOX3	0.021966	0.3698335	0.3173523	0.3478675	0.2953863	0.3216269	up
A_21_P0000865	LINC01016	0.1447148	0.5146513	0.4178147	0.3699365	0.2730999	0.3215182	up
A_21_P0011907	XLOC_l2_007931	-2.341181	-1.819058	-2.220333	0.5221238	0.1208484	0.3214861	up
A_23_P83438	UBE2Z	3.8485966	4.30446	4.0356874	0.4558635	0.1870909	0.3214772	up
A_23_P22382	TBC1D10B	5.348095	5.643699	5.695319	0.2956042	0.3472242	0.3214142	up
A_33_P3308232	FAM224A	2.0381374	2.3813567	2.3375206	0.3432193	0.2993832	0.3213012	up
A_23_P313476	FAM120B	0.1678343	0.4278321	0.5504198	0.2599978	0.3825855	0.3212917	up
A_24_P684183	SLC44A4	-0.194054	0.5026064	-0.248171	0.69666	-0.054117	0.3212714	up
A_22_P00010493	lnc-NBAS-1	-1.838234	-1.586998	-1.447073	0.251236	0.391161	0.3211985	up
A_21_P0000544	ATP1A1-AS1	-2.907248	-2.68512	-2.487117	0.2221277	0.420131	0.3211293	up
A_33_P3236993	ARVCF	-2.194928	-1.936733	-1.811058	0.2581949	0.3838701	0.3210325	up
A_23_P383435	ZNHIT3	5.266824	5.651199	5.5244217	0.3843751	0.2575979	0.3209865	up
A_23_P389391	SLC12A4	0.7954893	1.1149111	1.1178474	0.3194218	0.3223581	0.32089	up
A_23_P88753	TSR3	3.530177	3.7928329	3.9090462	0.2626557	0.3788691	0.3207624	up
A_21_P0007235	lnc-P2RY2-2	-2.103708	-1.625901	-1.940201	0.4778075	0.163507	0.3206573	up
A_23_P380928	ARPC4-TTLL3	-0.027863	0.2976236	0.2876873	0.3254862	0.3155499	0.320518	up
A_33_P3372844	DDX56	6.152238	6.3860345	6.559374	0.2337966	0.407136	0.3204663	up
A_23_P251021	HCG9	-1.116216	-0.691166	-0.900391	0.4250498	0.2158251	0.3204374	up
A_23_P119714	BABAM1	3.5021296	3.9586854	3.6861906	0.4565558	0.1840611	0.3203084	up
A_23_P116430	USH1C	0.9573708	1.1790376	1.3761902	0.2216668	0.4188194	0.3202431	up
A_21_P0014480	LOC401068	-1.888491	-1.445643	-1.690904	0.4428477	0.1975875	0.3202176	up
A_23_P81690	COX7A2	8.6453905	8.913963	9.017252	0.2685728	0.3718615	0.3202171	up
A_23_P137209	UBA1	4.3205223	4.772613	4.50879	0.4520907	0.1882677	0.3201792	up
A_23_P8142	CLPS	-1.714532	-1.408031	-1.380728	0.3065019	0.3338041	0.320153	up
A_22_P00023092	lnc-IGFL3-3	-3.111592	-2.307313	-3.275725	0.8042791	-0.164133	0.3200729	up
A_23_P62335	TMLHE	0.539001	0.8431382	0.8748841	0.3041372	0.3358831	0.3200102	up
A_33_P3387561	OSCAR	3.7368345	4.185373	3.9283085	0.4485383	0.191474	0.3200061	up
A_23_P142031	SBK2	-1.793467	-1.639417	-1.307529	0.1540499	0.4859381	0.319994	up
A_23_P90357	TBXA2R	-2.674729	-2.36009	-2.34952	0.3146393	0.3252084	0.3199239	up
A_33_P3379463	LYPLA2	4.273719	4.398105	4.7891216	0.1243863	0.5154028	0.3198946	up

A_22_P00013724	lnc-RP11-817J15.3.1-2	-2.151191	-1.413622	-2.249036	0.7375693	-0.097845	0.3198624	up
A_24_P184732	PAPLN	-2.827723	-2.532277	-2.483464	0.2954459	0.3442586	0.3198522	up
A_23_P45361	GLUD2	2.184659	2.3922515	2.6166983	0.2075925	0.4320393	0.3198159	up
A_33_P3261743	SLC37A2	-2.88544	-2.651937	-2.479341	0.2335031	0.4060988	0.319801	up
A_23_P15202	DHODH	2.832179	3.030458	3.273489	0.1982789	0.4413099	0.3197944	up
A_23_P130169	TBKBP1	1.3157182	1.6387014	1.6322613	0.3229833	0.3165431	0.3197632	up
A_24_P125283	HDAC5	-0.599863	-0.23202	-0.32828	0.3678427	0.2715826	0.3197126	up
A_33_P3388391	GJB4	0.3297854	0.5370932	0.7618876	0.2073078	0.4321022	0.319705	up
A_21_P0011129	TPTE2P6	-1.627094	-1.21344	-1.401403	0.4136548	0.2256908	0.3196728	up
A_21_P0006347	lnc-DCAF10-2	-1.018092	-0.817833	-0.579073	0.2002592	0.4390192	0.3196392	up
A_21_P0007318	LOC440028	-2.256151	-1.851786	-2.021342	0.4043651	0.2348094	0.3195872	up
A_21_P0013781	XLOC_l2_015578	-1.32476	-0.797584	-1.212815	0.5271764	0.1119452	0.3195608	up
A_33_P3305173	RGAG4	-1.817974	-1.814117	-1.182737	0.0038562	0.6352363	0.3195462	up
A_22_P00017575	lnc-WDR63-1	0.7528629	1.0650134	1.0796614	0.3121505	0.3267984	0.3194745	up
A_22_P00006934	lnc-GATAD2B-1	-1.88024	-1.462193	-1.659378	0.4180474	0.2208619	0.3194547	up
A_33_P3372124	TRAFD1	4.402753	4.668367	4.776042	0.265614	0.3732891	0.3194516	up
A_24_P409330	MRPL52	4.5976505	4.991636	4.8425016	0.3939853	0.2448511	0.3194182	up
A_21_P0001449	lnc-SCCPDH-1	-2.930039	-2.515221	-2.706101	0.4148181	0.2239382	0.3193781	up
A_21_P0005333	lnc-PAX4-1	-2.947743	-2.261821	-2.994942	0.6859224	-0.047199	0.3193619	up
A_33_P3228435	FXYD1	-2.970185	-2.789509	-2.512137	0.1806755	0.4580479	0.3193617	up
A_23_P112825	LCMT1	3.609603	4.0209165	3.8369045	0.4113135	0.2273016	0.3193076	up
A_24_P104119	RHOF	0.138164	0.4001403	0.5147009	0.2619762	0.3765369	0.3192565	up
A_23_P123916	LRSAM1	0.5376353	1.1191254	0.5946136	0.58149	0.0569782	0.3192341	up
A_23_P317207	ATXN7L2	0.8153071	1.218153	1.0508571	0.4028459	0.2355499	0.3191979	up
A_23_P102517	PDE6D	1.5011139	1.8673239	1.7732382	0.36621	0.2721243	0.3191671	up
A_24_P941167	APOL6	3.2196198	3.5317569	3.5456648	0.3121371	0.326045	0.3190911	up
A_33_P3412519	PEX26	-1.233305	-1.029355	-0.799108	0.2039499	0.4341965	0.3190732	up
A_33_P3235189	ANKRD13B	0.0748019	0.4008393	0.3866792	0.3260374	0.3118773	0.3189573	up
A_21_P0012379	XLOC_l2_009773	2.1149883	2.4322314	2.4355688	0.3172431	0.3205805	0.3189118	up
A_22_P00014911	lnc-SLCO2B1-3	-2.591334	-2.238168	-2.306797	0.3531656	0.2845368	0.3188512	up
A_33_P3272483	ARFGAP2	5.2145414	5.467833	5.598833	0.2532916	0.3842917	0.3187916	up
A_23_P39110	RUVBL2	5.0171633	5.3443866	5.3274984	0.3272233	0.3103352	0.3187792	up
A_19_P00801752	lnc-SIK1-2	2.1338015	2.5263982	2.3786497	0.3925967	0.2448483	0.3187225	up
A_33_P3818787	LOC344967	-0.988666	-0.81745	-0.522465	0.1712155	0.4662008	0.3187082	up
A_24_P583040	C17orf67	-0.483724	-0.078461	-0.251658	0.4052634	0.2320657	0.3186646	up
A_23_P2474	COPS7A	2.545188	2.7327352	2.9949656	0.1875472	0.4497776	0.3186624	up
A_33_P3284552	COPE	-2.907721	-2.210095	-2.968046	0.6976256	-0.060325	0.3186501	up
A_23_P30254	PLK2	3.6921568	4.057554	3.963931	0.365397	0.2717743	0.3185856	up
A_33_P3333224	GLIS2	2.745431	3.1063151	3.0215645	0.3608842	0.2761335	0.3185089	up
A_23_P259663	ZKSCAN5	-0.652294	-0.31282	-0.354857	0.3394737	0.2974367	0.3184552	up
A_33_P3424462	CNST	-2.616713	-2.37971	-2.217049	0.2370033	0.3996644	0.3183339	up
A_32_P49616	EEF1B2	8.737529	9.198828	8.912783	0.4612989	0.1752539	0.3182764	up
A_23_P101461	CCDC130	4.308524	4.7429442	4.510642	0.4344201	0.2021179	0.318269	up
A_32_P216602	lnc-NAV1-3	2.1152496	2.6076274	2.2592611	0.4923778	0.1440115	0.3181946	up
A_23_P154208	NAGK	2.442624	2.9995408	2.5219736	0.5569167	0.0793495	0.3181331	up
A_22_P00016696	lnc-TRA2A-1	5.3280344	5.779885	5.512327	0.4518504	0.1842928	0.3180716	up
A_24_P148907	MAB21L2	-1.291577	-0.774654	-1.172364	0.5169225	0.1192126	0.3180676	up
A_24_P76854	KRTAP2-1	-0.522507	-0.217735	-0.191314	0.3047714	0.331193	0.3179822	up
A_23_P127013	TCF7L2	-0.915637	-0.539311	-0.656053	0.3763256	0.2595844	0.317955	up
A_21_P0009597	LOC102723376	-1.374042	-1.052542	-1.059691	0.3214998	0.3143506	0.3179252	up
A_23_P422540	USP22	2.4158697	2.841164	2.626339	0.4252944	0.2104693	0.3178818	up
A_33_P3290235	LOC149950	-0.536364	-0.243214	-0.193871	0.29315	0.3424931	0.3178215	up
A_22_P00005603	DNAJC27-AS1	0.0895119	0.1901989	0.6242786	0.100687	0.5347667	0.3177269	up
A_23_P90732	PNKD	5.226227	5.4207673	5.6671047	0.1945405	0.4408779	0.3177092	up
A_24_P291401	TMEM150A	-2.280277	-1.964952	-1.960242	0.3153248	0.3200345	0.3176796	up

A_33_P3290403	IMPA2	1.3182578	1.6071305	1.66465	0.2888727	0.3463922	0.3176324	up
A_33_P3371663	LTK	2.8375816	3.2727647	3.0375185	0.4351831	0.1999369	0.31756	up
A_24_P74371	CTSA	5.5395393	5.9874625	5.72665	0.4479232	0.1871109	0.317517	up
A_23_P345220	SH3PXD2A	-0.961733	-0.494379	-0.794203	0.4673543	0.1675305	0.3174424	up
A_23_P313512	DCP1B	2.3082094	2.5769944	2.6742964	0.268785	0.366087	0.317436	up
A_23_P25194	HRK	1.1575489	1.5826774	1.3672776	0.4251285	0.2097287	0.3174286	up
A_23_P107211	RAB5C	1.1047249	1.4208889	1.423379	0.316164	0.3186541	0.317409	up
A_22_P00000868	LOC100505942	-1.252534	-0.919755	-0.950538	0.3327785	0.3019958	0.3173871	up
A_23_P81926	PSORS1C2	-2.917985	-2.621636	-2.579572	0.2963486	0.3384123	0.3173804	up
A_24_P55496	OSR2	-0.141791	0.1637936	0.1873336	0.3055849	0.3291249	0.3173549	up
A_19_P00322948	SNHG6	4.7935066	5.29833	4.923196	0.5048232	0.1296892	0.3172562	up
A_23_P129704	TERF2IP	2.5769625	2.9836745	2.8043394	0.4067121	0.2273769	0.3170445	up
A_33_P3295091	C1orf140	-1.112819	-0.878209	-0.713428	0.2346106	0.3993917	0.3170011	up
A_22_P00012922	OAZ2	1.05901	1.4919233	1.259954	0.4329133	0.200944	0.3169286	up
A_22_P00008781	lnc-KIF25-2	3.4333258	3.850945	3.6495	0.4176192	0.2161741	0.3168967	up
A_23_P63289	SSU72	5.343916	5.6365323	5.6850843	0.2926164	0.3411684	0.3168924	up
A_19_P00808320	lnc-PPA2-1	5.8294935	6.122812	6.169917	0.2933183	0.3404236	0.3168709	up
A_33_P3351606	MIR124-2HG	-0.425417	0.0140462	-0.231148	0.4394631	0.1942692	0.3168662	up
A_33_P3318343	CTAG2	-1.365482	-0.955983	-1.141377	0.4094992	0.2241049	0.316802	up
A_24_P117177	SNX21	-1.898699	-1.566424	-1.597624	0.3322754	0.301075	0.3166752	up
A_33_P3421490	KIAA1024	-1.766603	-1.670739	-1.229123	0.0958638	0.5374799	0.3166719	up
A_33_P3327772	ATP7A	-0.588807	-0.293979	-0.250464	0.2948279	0.3383431	0.3165855	up
A_22_P00015267	lnc-SPARCL1-1	-1.125085	-0.765293	-0.851759	0.3597922	0.2733264	0.3165593	up
A_23_P16166	PNPLA6	0.9532599	1.2278504	1.3117085	0.2745905	0.3584485	0.3165195	up
A_22_P00004923	lnc-DBH-1	0.1337776	0.4581561	0.4424009	0.3243785	0.3086233	0.3165009	up
A_33_P3260066	BEAN1	-2.07873	-1.692749	-1.831867	0.3859811	0.2468634	0.3164222	up
A_21_P0011765	XLOC_l2_007135	-3.260949	-3.108524	-2.780568	0.1524243	0.4803805	0.3164024	up
A_23_P108835	YPEL5	2.8536081	3.3147016	3.025055	0.4610934	0.1714468	0.3162701	up
A_32_P15320	EEF1A1	6.980027	7.5379014	7.054674	0.5578742	0.074647	0.3162606	up
A_33_P3273436	GALM	-0.387556	-0.074182	-0.0685	0.313374	0.319056	0.316215	up
A_33_P3862354	TRABD	1.0796876	1.4164658	1.375287	0.3367782	0.2955995	0.3161888	up
A_33_P3417141	CCDC124	3.5421515	3.744227	3.9722939	0.2020755	0.4301424	0.3161089	up
A_33_P3393851	ATP2B2	-2.667774	-2.002677	-2.700663	0.6650975	-0.032889	0.3161044	up
A_24_P929754	MKNK2	1.3636036	1.7932363	1.5661793	0.4296327	0.2025757	0.3161042	up
A_33_P3342260	GCK	-2.674655	-2.241069	-2.47615	0.4335856	0.1985052	0.3160454	up
A_33_P3249349	PRAME	-0.881694	-0.630965	-0.500496	0.2507291	0.3811979	0.3159635	up
A_23_P113613	CDCP1	3.1764708	3.5670133	3.4178553	0.3905425	0.2413845	0.3159635	up
A_21_P0013060	XLOC_l2_012870	3.85577	4.1850276	4.1583567	0.3292575	0.3025866	0.315922	up
A_23_P96350	PRAF2	-0.798379	-0.297438	-0.667482	0.5009413	0.1308966	0.3159189	up
A_33_P3413910	ADM2	-0.180449	0.1098723	0.1609807	0.2903214	0.3414297	0.3158755	up
A_23_P43763	PLLP	-2.32901	-2.020446	-2.005825	0.3085637	0.323185	0.3158743	up
A_23_P217712	ARSD	-1.508821	-1.200453	-1.185507	0.3083682	0.3233137	0.315841	up
A_23_P137814	ATP6V0B	5.8325605	6.0419593	6.254816	0.2093988	0.4222555	0.3158271	up
A_24_P376391	PLXND1	2.200142	2.3875833	2.644248	0.1874414	0.4441061	0.3157737	up
A_22_P00017310	MIR99AHG	-3.239987	-2.873078	-2.975391	0.366909	0.264596	0.3157525	up
A_22_P00012899	lnc-RBM34-1	-0.40182	-0.027706	-0.144583	0.374114	0.2572374	0.3156757	up
A_22_P00007431	lnc-GSDMD-3	-2.653714	-2.33546	-2.340627	0.318253	0.313087	0.31567	up
A_33_P3383431	DHRS4	2.2174988	2.5038218	2.5623932	0.2863231	0.3448944	0.3156087	up
A_23_P32938	DDX10	4.013151	4.319469	4.338044	0.3063178	0.324893	0.3156054	up
A_23_P32175	LHX6	0.1847978	0.3745055	0.6262202	0.1897078	0.4414225	0.3155651	up
A_21_P0011831	XLOC_l2_007456	0.4659085	0.570251	0.9926939	0.1043425	0.5267854	0.3155639	up
A_23_P119907	ANKZF1	0.5561733	0.7628613	0.9805841	0.2066879	0.4244108	0.3155494	up
A_23_P257256	GRK6	3.1465225	3.4427953	3.481347	0.2962728	0.3348246	0.3155487	up
A_22_P00019994	lnc-ORC4-1	-2.666393	-2.329236	-2.372879	0.3371575	0.2935143	0.3153359	up
A_24_P373844	KCTD15	0.908	1.2127228	1.2339067	0.3047228	0.3259068	0.3153148	up
A_32_P24581	RPS27A	8.745485	9.117821	9.003762	0.3723354	0.2582769	0.3153062	up

A_24_P136470	BTN2A3P	-0.777377	-0.485161	-0.439008	0.2922163	0.3383694	0.3152928	up
A_23_P372144	CACTIN	2.780511	3.0729384	3.1185722	0.2924275	0.3380613	0.3152444	up
A_21_P0012646	PRDX6	5.260023	5.614372	5.536072	0.3543487	0.2760487	0.3151987	up
A_23_P77048	SLC25A29	1.7073298	1.875556	2.1694708	0.1682262	0.462141	0.3151836	up
A_24_P331904	COMMD4	6.410532	6.707111	6.744298	0.2965789	0.333766	0.3151724	up
A_33_P3221568	ARMC5	1.4128585	1.7426128	1.7133842	0.3297544	0.3005257	0.31514	up
A_33_P3288694	LOC101929243	1.002975	1.2069669	1.4292207	0.2039919	0.4262457	0.3151188	up
A_22_P00007933	ABCC5-AS1	0.1904149	0.5124698	0.4985881	0.3220549	0.3081732	0.315114	up
A_23_P320290	ZNF827	-0.594405	-0.209291	-0.349345	0.3851137	0.2450595	0.3150866	up
A_24_P167825	VPS39	0.4486799	0.8759208	0.6515193	0.4272409	0.2028394	0.3150401	up
A_33_P3264926	SAMD4A	-0.292241	0.1937275	-0.148141	0.4859686	0.1441002	0.3150344	up
A_24_P132008	MSX2	-0.631929	-0.513703	-0.120341	0.1182256	0.5115881	0.3149068	up
A_23_P165921	LINC00029	-2.146514	-1.685805	-1.977553	0.4607091	0.1689611	0.3148351	up
A_33_P3292417	WDR5	5.0689163	5.2888513	5.478486	0.2199349	0.4095697	0.3147523	up
A_33_P3322553	MED25	2.3271065	2.640986	2.6427011	0.3138795	0.3155947	0.3147371	up
A_22_P00023019	lnc-ACSBG1-1	-0.463751	-0.106924	-0.191279	0.3568277	0.2724724	0.3146501	up
A_23_P253068	ANAPC2	-0.58758	-0.255568	-0.290333	0.3320127	0.2972474	0.31463	up
A_33_P3254756	UBA6-AS1	0.7646074	1.0422721	1.1161423	0.2776647	0.3515348	0.3145998	up
A_24_P17302	UBE2J2	-0.793245	-0.341744	-0.615663	0.4515004	0.1775818	0.3145411	up
A_24_P301063	PLEKHG6	0.2035127	0.7492075	0.2868996	0.5456948	0.0833869	0.3145409	up
A_33_P3641714	C19orf66	1.9563885	2.221232	2.320405	0.2648435	0.3640165	0.31443	up
A_22_P00007177	lnc-GNRHR2-1	-2.556132	-2.328443	-2.154968	0.227689	0.4011643	0.3144267	up
A_23_P89910	GEMIN7	4.0282946	4.171304	4.513726	0.1430097	0.4854317	0.3142207	up
A_23_P134477	C7orf50	4.0497437	4.31562	4.4122047	0.2658763	0.3624611	0.3141687	up
A_23_P40025	DAZAP2	5.7682915	6.166531	5.998171	0.3982396	0.2298794	0.3140595	up
A_22_P00000545	LOC100506639	-2.405346	-1.877011	-2.305623	0.5283356	0.0997236	0.3140296	up
A_23_P59069	HIST1H2BO	4.548833	4.8181777	4.9075356	0.2693448	0.3587027	0.3140237	up
A_33_P3310104	SERPINB5	5.309476	5.550014	5.696926	0.2405381	0.3874502	0.3139942	up
A_24_P136641	TOPORS-AS1	2.140459	2.3418527	2.5670528	0.2013936	0.4265938	0.3139937	up
A_22_P00011078	lnc-NUCB1-1	-0.750158	-0.390899	-0.481475	0.3592587	0.2686825	0.3139706	up
A_33_P3299254	VPREB3	-0.253592	0.0919886	0.0286918	0.3455806	0.2822838	0.3139322	up
A_33_P3315906	PTP4A3	1.4836764	1.67837	1.9167967	0.1946936	0.4331203	0.3139069	up
A_23_P200999	ST3GAL3	-2.034791	-1.797096	-1.644849	0.2376943	0.3899412	0.3138177	up
A_33_P3421695	CTNNB1	1.1486616	1.3793764	1.5455489	0.2307148	0.3968873	0.3138011	up
A_33_P3379571	MAP3K9	-0.384664	0.4330158	-0.574857	0.8176794	-0.190193	0.3137431	up
A_21_P0003216	LOC101927440	-0.785216	-0.474589	-0.468363	0.3106265	0.3168531	0.3137398	up
A_33_P3226425	ERP29	1.1944842	1.7302804	1.2861519	0.5357962	0.0916677	0.3137319	up
A_24_P336577	FAM212B	-0.611784	-0.276605	-0.319506	0.3351798	0.2922788	0.3137293	up
A_23_P76961	RPS29	9.740289	10.118229	9.9897785	0.3779402	0.2494898	0.313715	up
A_22_P00008580	LINC01231	-1.873698	-1.684038	-1.435944	0.1896601	0.4377542	0.3137071	up
A_23_P130919	MOB3A	0.284133	0.6803193	0.5152879	0.3961864	0.2311549	0.3136706	up
A_24_P230176	CCDC137	3.905303	4.172644	4.2651834	0.2673411	0.3598805	0.3136108	up
A_33_P3262495	ZNF503	5.2269487	5.5682197	5.5128193	0.3412709	0.2858706	0.3135707	up
A_23_P49499	ST6GALNAC2	-2.298329	-2.143718	-1.825913	0.1546109	0.4724159	0.3135134	up
A_23_P166400	RASL10A	-1.19046	-0.90267	-0.851234	0.2877903	0.3392267	0.3135085	up
A_23_P333029	ERICH5	-1.038819	-0.86175	-0.588884	0.1770687	0.4499345	0.3135016	up
A_22_P00000621	lnc-ACP1-1	-2.25449	-1.793853	-2.088228	0.4606366	0.1662622	0.3134494	up
A_23_P76774	GSC	-0.362334	-0.179162	0.0812354	0.1831718	0.4435692	0.3133705	up
A_22_P00003314	lnc-CAPRIN2-2	-1.403344	-1.218999	-0.961059	0.1843443	0.4422851	0.3133147	up
A_19_P00317412	LINC01137	1.8865013	2.1629348	2.2366714	0.2764335	0.3501701	0.3133018	up
A_33_P3352887	LOC388692	-0.016739	0.3889175	0.2041273	0.4056568	0.2208667	0.3132617	up
A_33_P3369885	MROH1	-0.723226	-0.363679	-0.456273	0.3595467	0.2669535	0.3132501	up
A_21_P0008127	lnc-CRYL1-1	-2.959081	-2.300731	-2.991249	0.6583502	-0.032168	0.313091	up
A_23_P1782	CD82	3.9192963	4.1158175	4.348918	0.1965213	0.4296217	0.3130715	up
A_24_P304154	AMPD3	-1.678978	-1.324441	-1.407384	0.354537	0.2715936	0.3130653	up
A_23_P109171	BFSP1	3.116353	3.5311503	3.3272943	0.4147973	0.2109413	0.3128693	up

A_23_P131299	ZFAND2B	1.9533806	2.3730283	2.1594105	0.4196477	0.2060299	0.3128388	up
A_23_P8013	HIST1H2BL	5.28524	5.549641	5.6463404	0.264401	0.3611002	0.3127506	up
A_23_P106761	CORO1A	0.476284	0.386405	1.1916585	-0.089879	0.7153745	0.3127477	up
A_33_P3314550	RAB3A	-1.097816	-1.082984	-0.487182	0.0148315	0.6106339	0.3127327	up
A_33_P3812669	GABARAPL1	3.6734982	4.3197923	3.6525345	0.6462941	-0.020964	0.3126652	up
A_24_P20200	PLEKHB2	2.6976204	3.049007	2.9713812	0.3513866	0.2737608	0.3125737	up
A_32_P47701	EEF1A1	6.4257393	6.99966	6.476924	0.5739207	0.0511847	0.3125527	up
A_22_P00012891	lnc-RBM22-1	1.5000544	1.8964052	1.7287898	0.3963509	0.2287355	0.3125432	up
A_24_P273666	GNAS	7.6735754	8.057848	7.914212	0.3842726	0.2406368	0.3124547	up
A_33_P3391517	SNX22	-1.641774	-1.25495	-1.403735	0.3868246	0.2380395	0.3124321	up
A_23_P10077	PNPLA2	1.0955343	1.4903893	1.3254738	0.394855	0.2299395	0.3123972	up
A_33_P3354267	AKIRIN1	4.0633755	4.34539	4.4060135	0.2820144	0.342638	0.3123262	up
A_21_P0000259	SNORD25	-1.067692	-0.695777	-0.815155	0.3719149	0.2525373	0.3122261	up
A_23_P421935	ATXN7L3	1.4550681	1.8566875	1.67763	0.4016194	0.2225618	0.3120906	up
A_23_P3574	GFOD2	2.5798855	2.8145413	2.9692745	0.2346559	0.389389	0.3120225	up
A_23_P205697	DLST	4.326771	4.6001954	4.6773796	0.2734246	0.3506088	0.3120167	up
A_23_P18246	XCR1	-1.517754	-1.329925	-1.081722	0.187829	0.4360318	0.3119304	up
A_23_P399501	PKM	8.045168	8.336672	8.377418	0.2915039	0.3322496	0.3118768	up
A_23_P160631	CCT3	7.297262	7.562203	7.6560726	0.2649407	0.3588104	0.3118756	up
A_24_P373126	TRMT61A	-0.416633	-0.110316	-0.099316	0.3063169	0.317317	0.3118169	up
A_33_P3322804	NTRK2	3.5519	3.9148698	3.812563	0.3629699	0.260663	0.3118165	up
A_23_P91221	PKIG	-1.677897	-1.449649	-1.282516	0.2282476	0.3953805	0.3118141	up
A_33_P3247082	SLC6A10P	1.0796919	1.5261984	1.2567568	0.4465065	0.1770649	0.3117857	up
A_21_P0000639	LOC100288123	-2.152809	-1.660065	-2.022043	0.4927445	0.1307659	0.3117552	up
A_24_P336957	HTRA2	2.3312092	2.8558574	2.4296713	0.5246482	0.0984621	0.3115552	up
A_23_P202520	ABLIM1	7.306896	7.7132144	7.5233917	0.4063182	0.2164955	0.3114069	up
A_33_P3326914	RGP1	-0.781624	-0.165339	-0.775224	0.6162858	0.0064001	0.311343	up
A_24_P659036	DENND6B	-1.989182	-1.721002	-1.634686	0.2681794	0.354496	0.3113377	up
A_23_P120776	SLC25A1	4.1902256	4.398517	4.604536	0.2082915	0.4143105	0.311301	up
A_24_P154573	ZBTB49	-3.202768	-2.801207	-2.981742	0.401561	0.2210255	0.3112932	up
A_21_P0000928	LOC115110	-3.01218	-3.029682	-2.37211	-0.017503	0.6400693	0.3112832	up
A_23_P151307	RAPGEF3	-2.194851	-2.229625	-1.53753	-0.034774	0.657321	0.3112733	up
A_24_P416411	PEX12	0.2482925	0.4383097	0.6806464	0.1900172	0.432354	0.3111856	up
A_24_P32887	BRD3	1.6823721	2.1158566	1.8711538	0.4334846	0.1887817	0.3111331	up
A_33_P3243429	GPR152	1.2014575	1.5063677	1.5187798	0.3049102	0.3173223	0.3111162	up
A_22_P00002707	lnc-C20orf196-1	0.9899597	1.2980847	1.3036942	0.308125	0.3137345	0.3109298	up
A_23_P370625	SEPN1	0.9365225	1.2490907	1.2456646	0.3125682	0.3091421	0.3108552	up
A_23_P98382	TIMM8B	7.7843113	8.0126	8.177652	0.2282887	0.3933411	0.3108149	up
A_23_P314070	ARHGAP1	5.8886766	6.1697173	6.229142	0.2810407	0.3404656	0.3107531	up
A_24_P181506	ZNF646	2.888073	3.072782	3.3247986	0.1847091	0.4367256	0.3107173	up
A_33_P3293391	BMS1P6	0.2513018	0.6045003	0.5194531	0.3531985	0.2681513	0.3106749	up
A_24_P106363	MARVELD2	1.1151643	1.2954717	1.5560708	0.1803074	0.4409065	0.310607	up
A_23_P86386	ZNF669	-1.662503	-1.337526	-1.366305	0.3249774	0.2961979	0.3105877	up
A_23_P138541	AKR1C3	4.609082	4.6513524	5.1879387	0.0422702	0.5788565	0.3105633	up
A_23_P32328	LYRM4	4.0070705	4.3645062	4.2707405	0.3574357	0.26367	0.3105528	up
A_33_P3374957	KLF16	3.8313236	4.148272	4.135454	0.3169484	0.3041306	0.3105395	up
A_23_P29684	VILL	2.543044	3.1004634	2.6066952	0.5574193	0.0636511	0.3105352	up
A_23_P340158	ISCU	6.128727	6.550158	6.3283243	0.4214311	0.1995974	0.3105142	up
A_33_P3397279	PPP6R2	-0.363302	0.0717058	-0.177374	0.4350076	0.1859274	0.3104675	up
A_23_P422724	PPIC	3.825035	4.1913247	4.0795135	0.3662896	0.2544785	0.310384	up
A_22_P00018697	LOC100288123	-2.757479	-2.357943	-2.536516	0.3995359	0.2209628	0.3102493	up
A_23_P67391	KPTN	1.0705471	1.5399404	1.2211514	0.4693933	0.1506043	0.3099988	up
A_24_P303480	RAB32	2.9728642	3.3044982	3.261197	0.331634	0.2883329	0.3099835	up
A_23_P82162	SMPD2	1.2920122	1.612268	1.5916953	0.3202558	0.2996831	0.3099694	up
A_21_P0014277	LOC100506606	-2.974597	-2.376704	-2.952659	0.5978935	0.0219381	0.3099158	up
A_33_P3282181	ARHGAP4	5.4014654	5.7104774	5.71227	0.3090119	0.3108044	0.3099082	up

A_23_P129476	VPS9D1	-0.054312	0.2182889	0.292901	0.2726007	0.3472128	0.3099067	up
A_23_P85560	EIF3I	5.633501	5.9345193	5.952223	0.3010182	0.3187218	0.30987	up
A_33_P3413053	NUBP1	4.262931	4.625463	4.520073	0.3625321	0.2571421	0.3098371	up
A_32_P230868	FAM65A	3.7162228	4.1324897	3.9194803	0.4162669	0.2032576	0.3097622	up
A_23_P92261	ECE2	4.955201	5.228741	5.301136	0.27354	0.3459349	0.3097374	up
A_33_P3356220	STARD3	8.753534	9.102476	9.023955	0.3489418	0.270421	0.3096814	up
A_22_P00004590	lnc-CS-1	3.2524996	3.5895228	3.5348349	0.3370233	0.2823353	0.3096793	up
A_23_P47004	DHX32	0.9354272	1.5639992	0.9261961	0.628572	-0.009231	0.3096705	up
A_22_P00022135	FAM110A	1.3712854	1.6532393	1.7086039	0.2819538	0.3373184	0.3096361	up
A_33_P3274105	CLHC1	1.709075	2.0178123	2.0195503	0.3087373	0.3104754	0.3096063	up
A_23_P436281	HIST2H4B	4.435272	4.661551	4.8282003	0.2262788	0.3929281	0.3096035	up
A_24_P226108	RBM47	-2.381773	-2.039669	-2.104782	0.3421035	0.2769904	0.3095469	up
A_24_P246943	PPIA	7.860368	8.236326	8.103483	0.3759584	0.2431154	0.3095369	up
A_33_P3380331	NKIRAS2	0.2006803	0.5291095	0.4911223	0.3284292	0.290442	0.3094356	up
A_23_P82000	TEAD3	1.541573	1.8243728	1.8776274	0.2827997	0.3360543	0.309427	up
A_23_P134419	ZP3	-0.63474	-0.254707	-0.395939	0.380033	0.238801	0.309417	up
A_24_P256155	NKX1-2	1.3021822	1.616231	1.6066813	0.3140488	0.3044992	0.309274	up
A_23_P29747	RPL23A	10.002896	10.330513	10.293785	0.3276167	0.2908888	0.3092528	up
A_33_P3405424	IL4I1	-0.83756	-0.682431	-0.374331	0.1551294	0.4632292	0.3091793	up
A_23_P418431	C6orf164	-3.049498	-2.65263	-2.828087	0.3968685	0.2214112	0.3091399	up
A_21_P0001610	lnc-FMOD-1	-1.915407	-1.6274	-1.585297	0.2880063	0.3301096	0.309058	up
A_23_P77630	MAP1LC3B	0.4829025	1.163744	0.4201698	0.6808415	-0.062733	0.3090544	up
A_33_P3388397	GJB4	-1.743434	-1.712171	-1.156632	0.0312629	0.5868011	0.309032	up
A_24_P45367	NIPAL3	3.0433645	3.4787602	3.226016	0.4353957	0.1826515	0.3090236	up
A_33_P3254460	DLK2	-1.65957	-1.089992	-1.611219	0.5695787	0.0483518	0.3089652	up
A_23_P368996	LRRC56	1.5914183	1.9418702	1.8588257	0.350452	0.2674074	0.3089297	up
A_33_P3286349	DNAAF3	1.9366245	2.0518699	2.4392347	0.1152453	0.5026102	0.3089278	up
A_23_P30024	NFKB1	2.4883642	2.7386823	2.8558693	0.2503181	0.3675051	0.3089116	up
A_33_P3359413	SGTA	3.065257	3.2595105	3.4888048	0.1942534	0.4235477	0.3089006	up
A_22_P00006915	LINC00565	-3.328856	-2.909633	-3.130335	0.4192226	0.1985211	0.3088719	up
A_24_P358328	TPI1P2	6.5398016	6.7878795	6.9093666	0.2480779	0.369565	0.3088214	up
A_33_P3268954	FBLL1	0.7884579	1.0511117	1.1433239	0.2626538	0.354866	0.3087599	up
A_23_P39402	ALKBH6	0.928843	1.2371931	1.2379446	0.3083501	0.3091016	0.3087258	up
A_23_P63870	SAMD8	-0.074691	0.2923927	0.1756373	0.3670836	0.2503281	0.3087058	up
A_33_P3236881	MINOS1-NBL1	7.2039146	7.364822	7.6603613	0.1609073	0.4564467	0.308677	up
A_22_P00002608	SSTR5-AS1	1.4933486	1.7653213	1.8387165	0.2719727	0.3453679	0.3086703	up
A_33_P3214948	SPOCK2	-1.232389	-0.870224	-0.977469	0.362165	0.2549205	0.3085427	up
A_23_P425073	RBMS2	0.1433792	0.5363493	0.3672981	0.3929701	0.2239189	0.3084445	up
A_22_P00018310	lnc-ZNF843-2	-1.902331	-1.614203	-1.573777	0.2881284	0.3285537	0.308341	up
A_23_P87257	MRPL17	3.0764885	3.2980523	3.4715786	0.2215638	0.3950901	0.308327	up
A_23_P84565	POLR3D	0.5982733	1.0340738	0.7791166	0.4358006	0.1808434	0.308322	up
A_23_P144497	RPS3A	9.445214	9.747364	9.759704	0.3021498	0.3144894	0.3083196	up
A_33_P3297415	NRP2	0.6544466	1.0761614	0.8493357	0.4217148	0.1948891	0.3083019	up
A_23_P64954	USP5	3.0435028	3.4155068	3.288086	0.372004	0.2445831	0.3082936	up
A_23_P47879	STAT6	5.331896	5.7212253	5.5591183	0.3893294	0.2272224	0.3082759	up
A_33_P3266923	LMTK2	1.5408893	2.1393437	1.5589838	0.5984545	0.0180945	0.3082745	up
A_23_P40049	CAD	3.3403616	3.7631164	3.5340967	0.4227548	0.1937351	0.3082449	up
A_23_P501134	LIG3	-1.138916	-0.834601	-0.826818	0.3043146	0.312098	0.3082063	up
A_33_P3310430	FAM86B2	3.1810236	3.6787066	3.2997398	0.4976831	0.1187162	0.3081996	up
A_33_P3219811	PTGDS	0.2946329	0.6945396	0.5111232	0.3999066	0.2164903	0.3081985	up
A_23_P71146	POLD2	2.8854504	3.1555285	3.2315836	0.2700782	0.3461332	0.3081057	up
A_33_P3313302	PARP6	-0.394347	-0.077463	-0.095146	0.3168836	0.2992005	0.308042	up
A_33_P3287218	GSTK1	4.565935	4.9212356	4.826686	0.3553004	0.2607508	0.3080256	up
A_33_P3311245	KRTAP19-7	-2.645745	-2.387166	-2.288312	0.2585793	0.3574336	0.3080064	up
A_33_P3235321	SYTL1	-0.934289	-0.543363	-0.709314	0.3909254	0.2249746	0.30795	up
A_22_P00015088	lnc-SNURF-3	-3.220273	-2.961938	-2.862942	0.2583354	0.3573308	0.3078331	up

A_23_P502808	PRIMA1	-0.317606	0.0791245	-0.098697	0.3967309	0.2189097	0.3078203	up
A_33_P3423570	METRN	5.5205193	5.8864207	5.770193	0.3659015	0.2496738	0.3077877	up
A_22_P00024329	MGC16275	-2.582952	-2.368793	-2.181656	0.214159	0.4012959	0.3077275	up
A_33_P3851788	CIRBP-AS1	-0.936544	-0.597355	-0.66057	0.3391895	0.2759743	0.3075819	up
A_33_P3325275	NRSN2	0.3161478	0.6058378	0.6415815	0.28969	0.3254337	0.3075619	up
A_33_P3269740	FAM86B2	3.061512	3.5157447	3.2223816	0.4542327	0.1608696	0.3075512	up
A_33_P3381827	OSBPL2	1.2809839	1.513371	1.6636391	0.2323871	0.3826551	0.3075211	up
A_32_P15799	HMGN2	6.7663927	7.1641717	6.983568	0.397779	0.2171755	0.3074772	up
A_33_P3370284	EPN1	7.1314907	7.553249	7.324606	0.4217582	0.1931152	0.3074367	up
A_22_P00020918	lnc-KLHL35-1	-2.742701	-2.618458	-2.252089	0.1242435	0.4906118	0.3074277	up
A_22_P00000429	lnc-AC073343.1-1	-0.914629	-0.524551	-0.689905	0.3900785	0.2247248	0.3074017	up
A_23_P27367	KDSR	0.4437852	0.778439	0.7238455	0.3346539	0.2800603	0.3073571	up
A_32_P122240	ASCL5	0.4525614	0.8557067	0.6638603	0.4031453	0.2112989	0.3072221	up
A_33_P3210810	LINC00260	-1.744478	-1.412656	-1.461902	0.3318219	0.2825756	0.3071988	up
A_23_P97442	TMEM222	2.3091116	2.6318603	2.6006823	0.3227487	0.2915707	0.3071597	up
A_22_P00003964	RGMB-AS1	-0.226976	0.0650296	0.0952945	0.2920055	0.3222704	0.307138	up
A_33_P3390032	EXOC7	2.84309	3.2343488	3.0660458	0.3912587	0.2229557	0.3071072	up
A_22_P00022774	GPS2	3.04665	3.4594731	3.2478848	0.4128232	0.2012348	0.307029	up
A_33_P3803639	TRAF2	5.625387	5.814383	6.0504313	0.1889958	0.4250441	0.30702	up
A_24_P12401	VEGFA	-0.178633	0.5125313	-0.255759	0.6911645	-0.077126	0.3070192	up
A_33_P3266396	CPTP	4.6571302	4.8802075	5.0480366	0.2230773	0.3909063	0.3069918	up
A_32_P116058	LINC00094	3.1781378	3.4941802	3.4758387	0.3160424	0.2977009	0.3068717	up
A_33_P3284019	FOXP4	7.0844164	7.5579534	7.2245903	0.473537	0.1401739	0.3068554	up
A_33_P3380772	CCM2L	7.345422	7.7755713	7.5289345	0.4301496	0.1835127	0.3068311	up
A_32_P182156	ZNF511	3.8125887	4.0971665	4.14147	0.2845779	0.3288813	0.3067296	up
A_23_P209625	CYP1B1	5.224475	5.611319	5.451023	0.3868442	0.2265482	0.3066962	up
A_33_P3223607	NDOR1	5.2291307	5.5095143	5.562125	0.2803836	0.3329945	0.306689	up
A_23_P147822	EPS8L2	3.0742168	3.3519902	3.409727	0.2777734	0.3355103	0.3066418	up
A_23_P128094	ABCB9	-1.948943	-1.577581	-1.707231	0.3713617	0.2417121	0.3065369	up
A_22_P00003888	lnc-CENPV-2	-0.918595	-0.605495	-0.618764	0.3131003	0.2998309	0.3064656	up
A_33_P3314978	PARD6G-AS1	-2.856813	-2.447284	-2.653458	0.409529	0.2033553	0.3064421	up
A_23_P141044	ZNF688	1.0837569	1.302444	1.4778175	0.2186871	0.3940606	0.3063738	up
A_23_P323783	LSM14B	0.4798093	0.8269401	0.7453127	0.3471308	0.2655034	0.3063171	up
A_23_P16683	TRMT1	4.1947308	4.450722	4.551366	0.2559915	0.3566351	0.3063133	up
A_33_P3319596	TANGO2	4.2478724	4.4861865	4.622163	0.2383142	0.3742905	0.3063023	up
A_22_P00004497	lnc-CPPED1-1	-2.36559	-2.101131	-2.017473	0.2644591	0.3481164	0.3062878	up
A_23_P157333	EPHA1	-0.610358	-0.248807	-0.359336	0.3615503	0.2510214	0.3062859	up
A_33_P3404189	MROH1	-1.276643	-0.839678	-1.101068	0.436965	0.1755753	0.3062701	up
A_33_P3256257	KRTAP4-7	-2.34529	-1.95786	-2.120316	0.3874307	0.2249746	0.3062027	up
A_23_P110712	DUSP1	1.7386332	2.441338	1.6483269	0.7027049	-0.090306	0.3061993	up
A_24_P107336	VPS26B	2.6027927	2.9116187	2.90633	0.308826	0.3035374	0.3061817	up
A_23_P162846	LAMP1	7.6323605	8.043543	7.8335285	0.4111824	0.2011681	0.3061752	up
A_33_P3842886	POLM	2.805317	3.1195207	3.1034174	0.3142037	0.2981005	0.3061521	up
A_24_P10233	DAPK2	-3.563386	-3.288216	-3.226468	0.2751699	0.3369179	0.3060439	up
A_23_P215198	TYW1	3.093501	3.3378854	3.4611683	0.2443843	0.3676672	0.3060257	up
A_23_P46604	NIPAL3	-0.241052	0.1857481	-0.055825	0.4268003	0.1852274	0.3060138	up
A_33_P3255165	ACTA2-AS1	-0.618072	-0.384061	-0.240176	0.2340112	0.3778958	0.3059535	up
A_33_P3245066	SLC35E2B	0.6558976	1.0075569	0.916132	0.3516593	0.2602344	0.3059468	up
A_22_P00016433	LOC101927734	-2.727558	-1.975487	-2.868075	0.7520711	-0.140517	0.3057768	up
A_23_P354734	PCDHGA8	0.6258297	0.8135133	1.0496216	0.1876836	0.4237919	0.3057377	up
A_23_P87323	COMMD9	1.0189476	1.3004289	1.3489337	0.2814813	0.3299861	0.3057337	up
A_33_P3382944	YJEFN3	2.31347	2.739283	2.4991016	0.4258132	0.1856318	0.3057225	up
A_22_P00020576	HOMER2	-0.835671	-0.536993	-0.522978	0.2986779	0.3126926	0.3056853	up
A_23_P21560	FAM49A	-1.367719	-0.662212	-1.462143	0.7055068	-0.094423	0.3055418	up
A_23_P169117	RRAGA	5.3158684	5.6105266	5.632185	0.2946582	0.3163166	0.3054874	up
A_23_P61531	GLB1	2.5332222	3.157906	2.519494	0.6246839	-0.013728	0.3054779	up

A_33_P3313596	CDK11A	1.5139008	1.8771963	1.7613039	0.3632956	0.2474031	0.3053494	up
A_22_P00004408	LMO7-AS1	-1.617999	-1.198309	-1.427084	0.4196906	0.1909156	0.3053031	up
A_33_P3356792	GGT7	-2.645712	-2.163992	-2.516846	0.48172	0.1288664	0.3052932	up
A_22_P00018323	SSPO	1.1271286	1.4227018	1.4420829	0.2955732	0.3149543	0.3052638	up
A_23_P250054	RNF123	-0.066379	0.1921101	0.2856503	0.2584887	0.3520289	0.3052588	up
A_22_P00024766	lnc-EGLN1-1	-2.027789	-1.851079	-1.594002	0.1767101	0.4337874	0.3052487	up
A_33_P3234487	BOLA2B	7.8326445	8.064623	8.211151	0.2319784	0.3785067	0.3052425	up
A_33_P3402454	HAS3	-1.560971	-1.285422	-1.22608	0.2755494	0.3348908	0.3052201	up
A_23_P208482	CLEC4M	-1.011839	-0.628235	-0.785121	0.3836041	0.2267184	0.3051612	up
A_23_P170337	ALDH4A1	2.786747	3.1000419	3.0836859	0.3132949	0.2969389	0.3051169	up
A_23_P155103	ADSL	3.4609413	3.9773965	3.554615	0.5164552	0.0936737	0.3050645	up
A_23_P129786	SREBF1	6.6640196	6.918677	7.0194216	0.2546573	0.355402	0.3050296	up
A_33_P3394352	VPS53	-1.427585	-1.102827	-1.142306	0.3247581	0.2852783	0.3050182	up
A_22_P00015392	lnc-SRGAP3-1	-0.222174	0.2624421	-0.096812	0.4846163	0.1253619	0.3049891	up
A_33_P3659808	PELP1	4.848716	5.2218184	5.085454	0.3731027	0.2367382	0.3049204	up
A_22_P00018322	SSPO	0.5933409	0.8960877	0.9001875	0.3027468	0.3068466	0.3047967	up
A_33_P3267745	C9orf69	-2.275911	-1.857017	-2.085213	0.4188948	0.1906982	0.3047965	up
A_21_P0012087	LOC100506797	-2.607965	-2.092928	-2.513633	0.5150375	0.0943327	0.3046851	up
A_21_P0009130	lnc-CMTM4-1	-0.270819	0.0298333	0.0378599	0.3006525	0.3086791	0.3046658	up
A_23_P133691	RRAGD	1.024982	1.3383017	1.3209534	0.3133197	0.2959714	0.3046455	up
A_33_P3250253	LOC100131150	-0.519705	-0.088215	-0.34192	0.4314895	0.1777849	0.3046372	up
A_32_P145153	RPL31	9.757209	10.144278	9.979404	0.3870688	0.2221956	0.3046322	up
A_22_P00015128	lnc-SNX33-1	-2.407789	-2.033699	-2.172644	0.3740907	0.2351451	0.3046179	up
A_33_P3272291	AKR1C4	-0.70396	-0.429145	-0.36954	0.2748151	0.3344207	0.3046179	up
A_33_P3253528	MED20	-3.047377	-2.695225	-2.790313	0.3521519	0.2570643	0.3046081	up
A_23_P114670	ARHGEF16	3.5563488	3.8239274	3.8979778	0.2675786	0.341629	0.3046038	up
A_33_P3296567	PRRC2B	0.5384512	0.9052405	0.7808013	0.3667893	0.2423501	0.3045697	up
A_23_P114808	MECR	2.4432707	2.8531623	2.6424675	0.4098916	0.1991968	0.3045442	up
A_21_P0005109	lnc-CD83-2	-2.095143	-1.831035	-1.750186	0.2641082	0.3449578	0.304533	up
A_22_P00009924	TAF1A-AS1	-1.293263	-0.933762	-1.043759	0.3595014	0.2495041	0.3045027	up
A_23_P20832	SPTAN1	4.5367203	4.9809422	4.701376	0.444222	0.1646557	0.3044388	up
A_21_P0007709	lnc-KRT80-5	-3.114514	-2.738094	-2.882153	0.3764198	0.2323611	0.3043904	up
A_22_P00004191	LOC101928617	-2.41814	-2.068317	-2.159309	0.349822	0.2588301	0.3043261	up
A_33_P3241482	ZNF346	3.8284655	4.151343	4.1140795	0.3228774	0.285614	0.3042457	up
A_24_P80204	MALL	6.249852	6.649298	6.458809	0.399446	0.2089567	0.3042014	up
A_23_P252783	SLC2A8	4.5842924	4.9992394	4.777689	0.414947	0.1933966	0.3041718	up
A_24_P302802	PCCB	1.1806765	1.4432907	1.5263958	0.2626143	0.3457193	0.3041668	up
A_33_P3229452	FLJ44477	0.8021855	1.1975689	1.0150805	0.3953834	0.2128949	0.3041391	up
A_33_P3284933	IL27	-2.247572	-1.981082	-1.905784	0.266489	0.3417873	0.3041382	up
A_23_P48964	VPS33B	-0.186191	0.1628943	0.0728908	0.3490853	0.2590818	0.3040836	up
A_22_P00000680	lnc-ADAL-1	-2.225858	-1.699143	-2.14453	0.5267153	0.0813274	0.3040214	up
A_33_P3410925	KLF1	-1.010451	-0.751682	-0.661306	0.258769	0.3491449	0.303957	up
A_22_P00023968	LOC101928532	1.978468	2.2618709	2.3027334	0.2834029	0.3242655	0.3038342	up
A_23_P17074	CCDC115	3.8383265	4.145352	4.1385393	0.3070254	0.3002129	0.3036191	up
A_23_P133868	ZKSCAN4	-1.876361	-1.578077	-1.567414	0.2982845	0.3089471	0.3036158	up
A_24_P330773	CALCOCO2	-0.491032	-0.055031	-0.31987	0.4360013	0.1711617	0.3035815	up
A_23_P55107	ULK2	-0.530679	-0.258336	-0.19617	0.2723432	0.3345089	0.303426	up
A_23_P135474	MRPL37	5.6467295	5.926121	5.9740925	0.2793918	0.327363	0.3033774	up
A_23_P3823	BCKDK	3.201435	3.3693194	3.6402178	0.1678844	0.4387827	0.3033335	up
A_33_P3358342	SIAH2	5.1328745	5.641852	5.2304955	0.5089774	0.097621	0.3032992	up
A_33_P3371333	TUBB8	-2.18741	-1.772315	-1.996039	0.4150953	0.1913705	0.3032329	up
A_23_P160787	PEX14	4.3668785	4.572438	4.767517	0.2055593	0.4006386	0.3030989	up
A_32_P60459	OTUD1	1.504919	2.269981	1.3460302	0.7650619	-0.158889	0.3030865	up
A_23_P200404	AK2	4.6802797	5.065844	4.900834	0.3855643	0.2205544	0.3030593	up
A_33_P3877739	SMCR2	0.4813409	0.8451309	0.7235923	0.36379	0.2422514	0.3030207	up
A_33_P3272698	DUSP23	4.8823166	5.191609	5.179034	0.3092923	0.2967176	0.303005	up

A_23_P88817	MLYCD	-1.086841	-0.771912	-0.796027	0.314929	0.2908139	0.3028715	up
A_23_P363174	HIST1H2AL	2.3946543	2.6213536	2.7736788	0.2266994	0.3790245	0.3028619	up
A_23_P31372	MDH2	6.6625414	6.8863444	7.044423	0.223803	0.3818817	0.3028424	up
A_23_P46149	GPR137B	1.4743471	1.4948263	2.059537	0.0204792	0.5851898	0.3028345	up
A_23_P206396	CKLF	5.0942698	5.4417167	5.352462	0.3474469	0.2581921	0.3028195	up
A_22_P00000258	lnc-AC007405.2.1-3	-2.200411	-1.704422	-2.090876	0.4959893	0.1095347	0.302762	up
A_33_P3215288	MIR646HG	-2.214191	-1.942313	-1.880663	0.2718782	0.333528	0.3027031	up
A_24_P186379	FUOM	2.6632252	2.9295945	3.0021915	0.2663693	0.3389664	0.3026679	up
A_32_P157481	ERCC4	-0.248291	0.0894814	0.0192699	0.3377729	0.2675614	0.3026671	up
A_21_P0009739	lnc-S1PR2-1	-0.374004	-0.096635	-0.046066	0.277369	0.3279376	0.3026533	up
A_23_P210920	GSS	4.3234463	4.4399505	4.812214	0.1165042	0.4887676	0.3026359	up
A_22_P00019225	lnc-EIF3A-2	-2.890408	-2.688932	-2.486617	0.2014751	0.4037907	0.3026329	up
A_32_P224850	LOC101927764	1.8760242	2.2915988	2.0655527	0.4155746	0.1895285	0.3025515	up
A_23_P49429	NUDT16L1	3.79984	4.156139	4.0485497	0.3562989	0.2487097	0.3025043	up
A_21_P0009195	lnc-PHB-2	-0.255358	0.1008892	-0.006667	0.356247	0.2486906	0.3024688	up
A_32_P514790	UNK	2.451149	2.7946734	2.7124844	0.3435245	0.2613354	0.3024299	up
A_33_P3279681	PPM1N	-1.157291	-0.96293	-0.746902	0.1943607	0.410388	0.3023744	up
A_22_P00017073	lnc-TUFM-3	-1.191973	-0.961336	-0.817904	0.2306376	0.3740692	0.3023534	up
A_23_P36724	FBXL14	-2.390276	-2.277844	-1.898002	0.1124322	0.4922743	0.3023533	up
A_23_P163361	RLBP1	-1.448474	-1.563503	-0.728819	-0.115029	0.7196546	0.3023126	up
A_23_P127460	SIPA1	3.3759146	3.5516906	3.8046417	0.175776	0.4287272	0.3022516	up
A_23_P11262	F8A1	4.6269264	4.9696956	4.888591	0.3427692	0.2616644	0.3022168	up
A_23_P148737	MYBPH	-1.799609	-1.011465	-1.983346	0.7881441	-0.183736	0.3022039	up
A_23_P201295	CASZ1	-1.335007	-0.819864	-1.245764	0.5151429	0.0892425	0.3021927	up
A_24_P53215	UNC13D	2.0374708	2.69566	1.9834213	0.6581893	-0.054049	0.3020699	up
A_21_P0013846	LINC01420	4.825639	5.055656	5.199666	0.2300172	0.3740273	0.3020222	up
A_21_P0000483	SNORD75	-1.772487	-1.493464	-1.44763	0.2790232	0.3248568	0.30194	up
A_33_P3384260	HNRNPA1	3.3607578	3.793365	3.5319376	0.4326072	0.1711798	0.3018935	up
A_33_P3338360	SCARNA13	2.7434187	2.9593225	3.1312828	0.2159038	0.3878641	0.3018839	up
A_33_P3333360	CDIP1	0.9433489	1.2778087	1.2126222	0.3344598	0.2692733	0.3018665	up
A_24_P340853	PARP10	-1.888516	-1.852643	-1.320686	0.0358729	0.5678301	0.3018515	up
A_21_P0000507	SNAR-B2	10.470572	10.803457	10.741192	0.3328848	0.2706194	0.3017521	up
A_24_P65507	STRA13	6.0627794	6.1091313	6.6199245	0.0463519	0.5571451	0.3017485	up
A_21_P0000474	SNORA38B	-1.299342	-1.138848	-0.856364	0.1604939	0.4429784	0.3017361	up
A_19_P00805954	LOC100129461	-1.622479	-1.223131	-1.41857	0.3993478	0.2039084	0.3016281	up
A_24_P380348	SMAP1	2.1461906	2.350121	2.5454283	0.2039304	0.3992376	0.301584	up
A_32_P62090	CCDC182	-2.646263	-2.505862	-2.183516	0.1404016	0.4627471	0.3015744	up
A_23_P104025	TSEN15	0.8896346	1.2761436	1.1062536	0.3865089	0.216619	0.301564	up
A_23_P431569	LOC100049716	0.2556253	0.6353598	0.4789972	0.3797345	0.223372	0.3015533	up
A_23_P250735	CBX7	2.5846395	3.0470853	2.7252598	0.4624457	0.1406202	0.301533	up
A_21_P0004757	lnc-KLHL31-2	-2.374538	-1.985529	-2.16074	0.38901	0.2137981	0.301404	up
A_19_P00316344	lnc-C9orf69-2	-0.063159	0.0248418	0.4515262	0.0880008	0.5146852	0.301343	up
A_22_P00013894	HCG25	-2.64035	-2.100608	-2.577429	0.5397425	0.0629211	0.3013318	up
A_23_P150350	C11orf1	2.933074	3.2103992	3.258359	0.2773252	0.325285	0.3013051	up
A_33_P3270147	XLOC_l2_001687	-3.176586	-2.832094	-2.91854	0.3444924	0.2580466	0.3012695	up
A_33_P3354464	LOXL1	2.0652866	2.2709222	2.4618683	0.2056356	0.3965817	0.3011086	up
A_33_P3289848	CDX1	0.4347343	0.8337584	0.637907	0.399024	0.2031727	0.3010983	up
A_23_P66038	ZDHHC1	-1.988872	-1.418056	-1.957611	0.570816	0.031261	0.3010385	up
A_21_P0003946	lnc-NDUFS6-1	-2.246527	-1.92822	-1.96281	0.3183069	0.2837167	0.3010118	up
A_33_P3265872	LOC101927497	-1.568821	-1.19677	-1.338912	0.3720503	0.2299085	0.3009794	up
A_33_P3258723	RMND5B	2.1028233	2.5558562	2.2517176	0.453033	0.1488943	0.3009636	up
A_24_P379165	FOXO4	-1.776122	-1.430977	-1.519496	0.3451452	0.2566261	0.3008857	up
A_23_P145376	MAPK13	0.8857966	1.2393284	1.1339879	0.3535318	0.2481914	0.3008616	up
A_33_P3323453	HM13	2.8992662	3.2937179	3.1065273	0.3944516	0.2072611	0.3008564	up
A_23_P27315	EMILIN2	3.8292685	4.186541	4.0736322	0.3572726	0.2443638	0.3008182	up

A_33_P3380462	GPR88	0.258337	0.6115985	0.5066648	0.3532615	0.2483277	0.3007946	up
A_32_P112279	CHTF8	2.9396372	3.2215257	3.2593222	0.2818885	0.319685	0.3007867	up
A_33_P3260100	CCDC167	4.3368273	4.4391904	4.835925	0.1023631	0.4990978	0.3007305	up
A_21_P0000298	SNORD8	-0.707256	-0.101015	-0.712049	0.6062412	-0.004793	0.3007243	up
A_33_P3368328	PHB2	5.777211	6.162167	5.993657	0.3849559	0.2164459	0.3007009	up
A_23_P65768	RSL24D1	3.655839	4.135115	3.777956	0.4792762	0.122117	0.3006966	up
A_33_P3414389	SH2B2	1.7838497	1.9877028	2.1812305	0.2038531	0.3973808	0.300617	up
A_33_P3395823	KCNQ2	2.9542866	3.3256497	3.1840973	0.3713632	0.2298107	0.3005869	up
A_23_P166196	AAR2	3.3884974	3.673697	3.704403	0.2851996	0.3159056	0.3005526	up
A_23_P251767	BANP	-1.291641	-1.041032	-0.941231	0.2506094	0.3504105	0.3005099	up
A_33_P3229869	LOC100128714	-2.866145	-2.470022	-2.661434	0.3961225	0.204711	0.3004167	up
A_23_P152548	SCPEP1	1.430438	1.8759375	1.5857244	0.4454994	0.1552863	0.3003929	up
A_33_P3237644	EFCAB5	-3.004392	-2.895413	-2.512676	0.1089788	0.4917166	0.3003477	up
A_32_P98227	LDB3	-2.816124	-2.554857	-2.476801	0.2612672	0.3393235	0.3002954	up
A_21_P0007799	lnc-SLC15A4-2	-0.51715	-0.228401	-0.205309	0.2887487	0.3118405	0.3002946	up
A_23_P143748	TTLL12	5.0724936	5.3413568	5.404215	0.2688632	0.3317213	0.3002923	up
A_23_P308581	RRP36	5.538952	5.788366	5.8901215	0.249414	0.3511696	0.3002918	up
A_23_P142022	PRPF31	2.6493187	2.8186374	3.0805092	0.1693187	0.4311905	0.3002546	up
A_24_P346886	NDUFB8	7.180503	7.49219	7.469202	0.311687	0.2886992	0.3001931	up
A_33_P3336652	NDUFC2	6.1625967	6.4084897	6.5168076	0.245893	0.3542109	0.3000519	up
A_23_P28652	ATRAID	2.6763763	3.112039	2.8407784	0.4356628	0.164402	0.3000324	up
A_23_P93282	HIST1H3J	3.0906963	3.3728566	3.408412	0.2821603	0.3177156	0.299938	up
A_33_P3275510	C20orf196	1.3180022	1.4963722	1.7394624	0.17837	0.4214602	0.2999151	up
A_24_P20383	ARPC4	1.6065102	1.8510203	1.9618273	0.2445102	0.3553171	0.2999136	up
A_21_P0000169	TMPRSS13	3.0639124	3.5786157	3.1489573	0.5147033	0.0850449	0.2998741	up
A_23_P424316	TCF20	1.7971048	2.0289388	2.1648989	0.2318339	0.367794	0.299814	up
A_22_P00013447	LOC102724966	-2.679581	-2.342861	-2.41676	0.3367195	0.262821	0.2997702	up
A_22_P00000222	LOC100506585	0.1989756	0.536171	0.4613171	0.3371954	0.2623415	0.2997685	up
A_23_P57961	PLXNB1	0.7830086	1.057991	1.1075339	0.2749825	0.3245254	0.2997539	up
A_21_P0001246	LOC101927851	4.1429214	4.577889	4.307369	0.4349675	0.1644478	0.2997077	up
A_33_P3290085	ZNF394	2.237957	2.5137897	2.5614882	0.2758327	0.3235312	0.2996819	up
A_24_P542375	PTMA	8.143434	8.472815	8.413359	0.329381	0.2699251	0.2996531	up
A_21_P0011874	XLOC_l2_007767	-0.69558	-0.304431	-0.487514	0.3911486	0.208066	0.2996073	up
A_23_P6464	PLA2G6	0.1204648	0.4817262	0.3583617	0.3612614	0.2378969	0.2995791	up
A_23_P204630	NTN4	1.3257184	1.9815316	1.2688642	0.6558132	-0.056854	0.2994795	up
A_23_P97606	GSTM5	-1.061492	-0.841126	-0.682957	0.220366	0.3785348	0.2994504	up
A_33_P3267296	FKBP11	5.075329	5.5043797	5.2451677	0.4290509	0.1698389	0.2994449	up
A_24_P42136	KRT18	8.872253	9.235598	9.107689	0.3633442	0.2354355	0.2993898	up
A_33_P3309468	PTPRS	1.3775401	1.4925632	1.861103	0.1150231	0.483563	0.299293	up
A_23_P3982	MIEN1	10.149791	10.426046	10.47209	0.2762556	0.322299	0.2992773	up
A_23_P135465	HARBI1	1.3247781	1.6429949	1.604981	0.3182168	0.2802029	0.2992098	up
A_23_P142969	ZNF2	-2.042004	-1.856482	-1.629107	0.1855221	0.4128966	0.2992094	up
A_23_P256148	AKIRIN1	2.32545	2.528253	2.721016	0.2028031	0.395566	0.2991846	up
A_23_P322043	SMG5	3.7286777	4.066337	3.9892864	0.3376594	0.2606087	0.299134	up
A_23_P96623	OPN1MW	-2.884037	-2.738706	-2.43121	0.1453309	0.452827	0.2990789	up
A_22_P00024677	LOC101927901	-0.659104	-0.376943	-0.343123	0.2821608	0.3159809	0.2990708	up
A_32_P221799	HIST1H2AM	2.966528	3.242684	3.2884836	0.276156	0.3219557	0.2990558	up
A_21_P0013471	XLOC_l2_014171	-1.841335	-1.426421	-1.658198	0.4149141	0.1831374	0.2990258	up
A_22_P00001617	ZBED1	1.2591591	1.447031	1.669281	0.1878719	0.4101219	0.2989969	up
A_33_P3226985	CDK5R1	1.4207926	1.7082086	1.7313137	0.287416	0.3105211	0.2989686	up
A_23_P207213	ALDH3A1	4.0957794	4.311003	4.478424	0.2152238	0.3826447	0.2989342	up
A_24_P212860	C9orf156	-1.093838	-0.695509	-0.894303	0.3983293	0.1995354	0.2989323	up
A_24_P385280	ALDH9A1	2.5838585	2.8459697	2.9195004	0.2621112	0.3356419	0.2988765	up
A_23_P14389	ACIN1	1.2735395	1.6100659	1.5346818	0.3365264	0.2611423	0.2988343	up
A_21_P0002823	LINCR-0002	-0.637333	-0.214619	-0.462473	0.4227138	0.1748595	0.2987866	up
A_21_P0008807	lnc-OR4M2-7	0.2472105	0.7956138	0.2963362	0.5484033	0.0491257	0.2987645	up

A_23_P106694	CHMP1A	4.310917	4.5451293	4.67422	0.2342124	0.3633032	0.2987578	up
A_23_P502930	FASTK	5.1433086	5.563874	5.320257	0.4205651	0.1769486	0.2987568	up
A_33_P3216427	DGKQ	1.427908	1.787261	1.6659303	0.3593531	0.2380223	0.2986877	up
A_33_P3231858	MAP3K14-AS1	-2.668791	-2.477768	-2.262559	0.1910236	0.4062316	0.2986276	up
A_23_P15654	TRAF4	2.59832	2.9666724	2.8271093	0.3683524	0.2287893	0.2985709	up
A_24_P257971	SLC27A4	1.2468333	1.5016041	1.5891619	0.2547708	0.3423286	0.2985497	up
A_21_P0003871	lnc-ZFP42-7	-2.14687	-1.972416	-1.724307	0.1744537	0.4225626	0.2985082	up
A_23_P80136	RRP1	0.6818204	0.9222102	1.038311	0.2403898	0.3564906	0.2984402	up
A_32_P74120	LINC00869	5.747035	6.024509	6.0664225	0.2774739	0.3193874	0.2984307	up
A_22_P00019478	HOXC13-AS	-0.23834	0.140924	-0.020791	0.3792644	0.2175493	0.2984068	up
A_33_P3302687	CARKD	-1.664673	-1.396811	-1.335794	0.2678618	0.3288794	0.2983706	up
A_21_P0007137	LINC01150	1.3246522	1.6643629	1.5813689	0.3397107	0.2567167	0.2982137	up
A_33_P3218089	CLDN24	-3.20265	-2.875423	-2.933467	0.3272278	0.2691832	0.2982055	up
A_33_P3305102	GPR97	0.1084881	0.3346386	0.4786859	0.2261505	0.3701978	0.2981741	up
A_33_P3374643	PAAF1	3.8221292	4.000878	4.2397203	0.1787486	0.4175911	0.2981699	up
A_22_P00005124	NAPA-AS1	1.5030856	1.8826432	1.7194705	0.3795576	0.2163849	0.2979712	up
A_33_P3330911	BCAS1	1.5684662	1.9723792	1.7604537	0.403913	0.1919875	0.2979503	up
A_24_P332971	RPL7L1	3.6143827	3.817594	4.0069637	0.2032113	0.392581	0.2978962	up
A_21_P0005079	lnc-LPA-1	-0.761679	-0.49332	-0.434615	0.2683587	0.3270636	0.2977111	up
A_23_P127721	P2RX3	-1.991962	-1.860981	-1.527576	0.1309815	0.464386	0.2976837	up
A_33_P3406828	MAFIP	-1.053397	-0.743102	-0.768463	0.3102951	0.284934	0.2976146	up
A_33_P3213910	ARHGAP30	-1.621691	-1.219375	-1.428788	0.4023156	0.1929026	0.2976091	up
A_23_P129005	NYNRIN	-1.370595	-1.273376	-0.8727	0.0972195	0.4978952	0.2975574	up
A_23_P108501	EPHA4	0.8466334	1.0699129	1.2184072	0.2232795	0.3717737	0.2975266	up
A_23_P209778	POLR2D	3.366706	3.6720986	3.6562138	0.3053927	0.2895079	0.2974503	up
A_23_P142447	MYO1F	-2.627779	-2.669324	-1.99147	-0.041545	0.6363084	0.2973818	up
A_33_P3264875	GRK6	2.1151829	2.4450002	2.3801079	0.3298173	0.264925	0.2973712	up
A_23_P331479	PPP1R18	2.5499744	2.9843507	2.7103224	0.4343762	0.1603479	0.2973621	up
A_23_P110811	COX7C	8.781133	9.043039	9.113924	0.2619066	0.3327913	0.297349	up
A_23_P95213	SFTPC	-1.695366	-1.4554	-1.34086	0.2399669	0.3545065	0.2972367	up
A_21_P0007706	lnc-ADCY6-1	-2.598287	-2.292396	-2.309717	0.3058913	0.2885697	0.2972305	up
A_23_P33465	CRELD2	5.0532894	5.3025813	5.398444	0.2492919	0.3451548	0.2972233	up
A_33_P3292478	CCL16	1.225368	1.5624266	1.4827514	0.3370585	0.2573834	0.2972209	up
A_24_P280868	FAM86B2	3.4360733	3.9277186	3.538701	0.4916453	0.1026278	0.2971365	up
A_23_P55706	RELB	4.877803	5.3052506	5.0446243	0.4274478	0.1668215	0.2971346	up
A_21_P0013472	XLOC_l2_014182	-1.544716	-1.161573	-1.333644	0.3831434	0.211072	0.2971077	up
A_23_P253029	BOK	3.796279	4.088473	4.0981503	0.2921939	0.3018713	0.2970326	up
A_24_P194081	FXYD5	5.0224676	5.592377	5.046363	0.5699096	0.0238953	0.2969024	up
A_24_P253003	WNT11	6.3894424	6.8186984	6.553957	0.429256	0.1645145	0.2968853	up
A_23_P43238	NAPRT	2.8750372	3.2019067	3.1418505	0.3268695	0.2668133	0.2968414	up
A_23_P408094	MXD1	-0.029278	0.3064709	0.2284951	0.3357487	0.2577729	0.2967608	up
A_24_P147407	STRADA	0.3158364	0.4182782	0.8067908	0.1024418	0.4909544	0.2966981	up
A_23_P24903	P2RY2	3.7198076	3.777741	4.2551355	0.0579333	0.5353279	0.2966306	up
A_33_P3357651	KRTAP10-12	-0.012414	0.404047	0.1640744	0.416461	0.1764884	0.2964747	up
A_23_P71981	ERAL1	2.119914	2.3481336	2.4846363	0.2282195	0.3647223	0.2964709	up
A_23_P135326	POLE3	5.193783	5.544354	5.436125	0.3505712	0.242342	0.2964566	up
A_23_P105044	MRPL23	4.180702	4.372932	4.5813427	0.1922298	0.4006405	0.2964351	up
A_21_P0010050	lnc-PREX1-4	-2.676002	-2.323049	-2.436096	0.3529522	0.2399054	0.2964288	up
A_23_P148546	NAA10	3.4923162	3.730556	3.8468819	0.2382398	0.3545656	0.2964027	up
A_33_P3240767	CIDEC	0.6603265	1.0265956	0.8868322	0.3662691	0.2265058	0.2963874	up
A_23_P165984	ZSWIM3	-0.496809	0.0368285	-0.437715	0.5336375	0.059094	0.2963657	up
A_23_P75149	SFXN4	3.8805342	4.240525	4.1131754	0.3599906	0.2326412	0.2963159	up
A_23_P44505	KLF11	2.0859518	2.659062	2.1053333	0.5731101	0.0193815	0.2962458	up
A_22_P00001854	ADAMTS9-AS1	-1.640854	-1.252121	-1.437111	0.3887339	0.2037435	0.2962387	up
A_32_P67259	SDHA	4.441804	4.8473115	4.6287174	0.4055076	0.1869135	0.2962105	up
A_24_P220485	OLFML2A	-1.961291	-1.597228	-1.733006	0.3640628	0.2282853	0.2961741	up

A_23_P65830	HDDC3	3.6439018	3.8979535	3.9821644	0.2540517	0.3382626	0.2961571	up
A_33_P3266674	ZBTB46	-0.884592	-0.750891	-0.425989	0.1337013	0.4586029	0.2961521	up
A_24_P393461	C1orf43	3.2527933	3.6131024	3.484744	0.3603091	0.2319508	0.2961299	up
A_23_P66110	TSC2	1.2213607	1.7935205	1.2414279	0.5721598	0.0200672	0.2961135	up
A_23_P135104	MRPS2	6.552533	6.726687	6.970605	0.1741538	0.4180718	0.2961128	up
A_32_P218228	FAM109B	-0.773692	-0.424881	-0.530299	0.3488107	0.2433929	0.2961018	up
A_24_P76666	CSNK2A1	0.4958305	0.8548441	0.7289176	0.3590136	0.2330871	0.2960503	up
A_23_P95165	SEMA4B	-0.8302	-0.501854	-0.566608	0.3283453	0.2635913	0.2959683	up
A_22_P00009227	PCOLCE-AS1	1.7199564	2.0443892	1.9873848	0.3244329	0.2674284	0.2959306	up
A_22_P00012987	lnc-RFC4-1	-2.848796	-2.636782	-2.468958	0.2120144	0.3798385	0.2959265	up
A_23_P53176	FOLR1	2.8610363	3.318499	2.9953012	0.4574628	0.134265	0.2958639	up
A_23_P29555	SEC13	7.34752	7.60644	7.680194	0.2589202	0.332674	0.2957971	up
A_33_P3394615	FUNDC2	2.6702032	3.1697717	2.7622042	0.4995685	0.092001	0.2957847	up
A_23_P15045	E4F1	1.8520031	2.1339574	2.1615458	0.2819543	0.3095427	0.2957485	up
A_22_P00024424	APOA1-AS	-2.903361	-2.804454	-2.410835	0.0989068	0.4925253	0.295716	up
A_21_P0013805	LOC389906	3.0378828	3.3332047	3.3339443	0.2953219	0.2960615	0.2956917	up
A_23_P142688	FAM134A	1.6081438	2.0286765	1.7788925	0.4205327	0.1707487	0.2956407	up
A_23_P130488	ERCC2	0.6587205	0.8414555	1.0671554	0.182735	0.4084349	0.2955849	up
A_33_P3267612	MIR143HG	-2.642594	-2.316203	-2.377941	0.3263907	0.264653	0.2955219	up
A_22_P00003232	LINC00900	2.2611113	2.5816445	2.531577	0.3205333	0.2704659	0.2954996	up
A_33_P3265504	EIF5AL1	-0.613494	-0.228605	-0.407507	0.3848891	0.205987	0.2954381	up
A_21_P0000514	VTRNA1-2	-0.30855	-0.031758	0.0055161	0.2767916	0.3140659	0.2954288	up
A_22_P00007780	lnc-HNRNPA3-2	-2.084327	-1.501655	-2.076149	0.5826726	0.0081787	0.2954257	up
A_23_P151368	N6AMT2	2.1358223	2.389277	2.4730463	0.2534547	0.337224	0.2953394	up
A_23_P46378	PIGV	0.8198509	1.1387424	1.0915422	0.3188915	0.2716913	0.2952914	up
A_23_P20107	GSTK1	0.820272	1.1433153	1.0878029	0.3230434	0.2675309	0.2952871	up
A_21_P0011960	LOC100506797	-1.405388	-0.945626	-1.274614	0.4597621	0.130774	0.2952681	up
A_23_P120942	XRCC6	9.032076	9.416914	9.237674	0.3848381	0.2055979	0.295218	up
A_24_P12435	NCOA7	2.5830364	3.0857577	2.6706924	0.5027213	0.087656	0.2951887	up
A_23_P82128	RPS12	9.204692	9.55995	9.439745	0.355258	0.2350531	0.2951555	up
A_23_P169629	SHMT2	5.64396	5.8694663	6.0087376	0.2255063	0.3647776	0.2951419	up
A_23_P24234	OPN4	-1.390511	-1.049477	-1.141498	0.3410335	0.249013	0.2950232	up
A_21_P0004480	lnc-TRIM7-3	-2.782579	-2.525374	-2.449944	0.2572045	0.3326352	0.2949198	up
A_24_P333421	ZNF862	-0.177345	0.1974602	0.0376563	0.3748055	0.2150016	0.2949035	up
A_22_P00019084	LOC101927884	-3.256767	-2.838835	-3.0849	0.4179316	0.1718667	0.2948991	up
A_21_P0011867	XLOC_l2_007656	2.4161053	2.8549695	2.5670357	0.4388642	0.1509304	0.2948973	up
A_24_P681301	UBC	9.856275	10.254406	10.047915	0.3981314	0.1916399	0.2948856	up
A_24_P5550	PHF23	0.2290592	0.6278367	0.4199343	0.3987775	0.1908751	0.2948263	up
A_33_P3350508	FTO	-1.84238	-1.963964	-1.131227	-0.121584	0.711153	0.2947843	up
A_23_P393749	CATSPER3	-0.66973	-0.360224	-0.389712	0.3095064	0.2800183	0.2947624	up
A_23_P258164	CORT	-0.904911	-0.605485	-0.614833	0.2994266	0.2900782	0.2947524	up
A_23_P69339	ACAA1	4.480646	4.8149657	4.7356777	0.3343196	0.2550316	0.2946756	up
A_23_P212447	IFT122	0.8883991	1.2752423	1.0909057	0.3868432	0.2025065	0.2946749	up
A_23_P897	C1orf116	3.5989304	4.0341287	3.7530518	0.4351983	0.1541214	0.2946599	up
A_23_P54622	KIF22	3.5909138	3.8216481	3.9494715	0.2307344	0.3585577	0.294646	up
A_23_P47517	VPS37C	4.6908646	5.110931	4.860052	0.4200664	0.1691876	0.294627	up
A_23_P115215	VPS72	4.014015	4.332681	4.2845783	0.318666	0.2705631	0.2946146	up
A_23_P85703	SOX13	-0.376205	-0.15353	-0.009716	0.2226758	0.3664899	0.2945829	up
A_21_P0004470	lnc-DUSP1-1	-1.987283	-1.858928	-1.52655	0.128355	0.4607329	0.294544	up
A_23_P109877	WDR82	1.9265661	2.3333669	2.1087418	0.4068008	0.1821756	0.2944882	up
A_22_P00016533	LOC101926967	-1.323934	-0.901475	-1.157444	0.4224587	0.1664896	0.2944741	up
A_23_P125408	DOHH	1.6333256	1.7927809	2.0627747	0.1594553	0.4294491	0.2944522	up
A_24_P116535	MMP15	1.2870927	1.6632252	1.4997034	0.3761325	0.2126107	0.2943716	up
A_24_P349616	UBTF	1.2829423	1.5301847	1.6244292	0.2472425	0.3414869	0.2943647	up
A_23_P215253	POLR2J3	4.19139	4.3558173	4.615426	0.1644273	0.424036	0.2942317	up
A_23_P313961	NME6	0.1546755	0.3933845	0.5044074	0.238709	0.3497319	0.2942204	up

A_23_P431346	PRR15	0.353065	0.6852503	0.6092806	0.3321853	0.2562156	0.2942004	up
A_33_P3260430	SPRR2A	1.0172915	1.6554842	0.9674425	0.6381927	-0.049849	0.2941718	up
A_23_P161152	PDSS1	2.5362873	2.7929091	2.867836	0.2566218	0.3315487	0.2940853	up
A_22_P00014701	lnc-SLC25A35-1	0.2109375	0.562078	0.4479423	0.3511405	0.2370048	0.2940726	up
A_23_P49060	SPINT1	4.105277	4.437529	4.361024	0.332252	0.2557468	0.2939994	up
A_23_P104689	RELA	3.075508	3.5339322	3.20498	0.4584241	0.1294718	0.2939479	up
A_24_P50245	HLA-DMA	0.7123456	1.1428633	0.8697124	0.4305177	0.1573668	0.2939422	up
A_33_P3285470	BAI1	-1.313083	-0.596037	-1.442358	0.7170463	-0.129274	0.2938859	up
A_33_P3229177	POLE4	4.2302265	4.6289153	4.4192734	0.3986888	0.1890469	0.2938678	up
A_33_P3379157	MTCH1	3.097393	3.387034	3.3954153	0.2896409	0.2980223	0.2938316	up
A_33_P3345816	GPER1	2.9809113	2.891152	3.6583328	-0.089759	0.6774216	0.2938311	up
A_23_P258071	RNF113A	4.079402	4.4442487	4.3021555	0.3648467	0.2227535	0.2938001	up
A_33_P3246318	RBX1	6.7833576	7.007735	7.1464605	0.2243772	0.3631029	0.29374	up
A_33_P3354678	TRIM78P	-2.705798	-2.449708	-2.374486	0.2560902	0.3313122	0.2937012	up
A_33_P3419234	DCAF4	-0.631998	-0.162365	-0.514229	0.4696326	0.1177692	0.2937009	up
A_33_P3224045	DAPK3	4.135668	4.4527774	4.4059486	0.3171096	0.2702808	0.2936952	up
A_21_P0010376	lnc-ZMAT5-1	-2.510461	-2.057231	-2.376304	0.4532306	0.1341567	0.2936937	up
A_24_P411186	BCL11A	-0.870118	-0.432426	-0.720548	0.4376912	0.1495695	0.2936304	up
A_23_P211285	NDUFV3	4.037732	4.2138658	4.4487867	0.1761336	0.4110546	0.2935941	up
A_22_P00007265	lnc-GPR15-1	-0.843194	-0.399627	-0.699671	0.4435668	0.1435232	0.293545	up
A_23_P86855	MACROD1	2.2688665	2.5807834	2.5439835	0.3119168	0.2751169	0.2935169	up
A_33_P3336233	UBL4A	-1.136412	-0.967783	-0.718077	0.1686287	0.4183345	0.2934816	up
A_33_P3398840	ZBED1	0.0579758	0.2769804	0.4255996	0.2190046	0.3676238	0.2933142	up
A_22_P00005771	lnc-ENPP1-2	0.5199256	0.8503499	0.7759166	0.3304243	0.255991	0.2932076	up
A_23_P44956	RPL35A	9.928794	10.267984	10.176006	0.3391905	0.2472124	0.2932014	up
A_24_P397584	TBCC	1.7861814	2.2208238	1.9375048	0.4346423	0.1513233	0.2929828	up
A_23_P156284	DBN1	4.6260967	4.964578	4.873396	0.3384814	0.2472992	0.2928903	up
A_23_P331235	C5orf38	-0.892355	-0.626378	-0.572596	0.2659774	0.3197594	0.2928684	up
A_21_P0010256	lnc-PKNOX1-2	-0.241999	-0.161518	0.2630243	0.0804815	0.5050235	0.2927525	up
A_33_P3265075	PAAF1	3.7503843	3.9728532	4.1130915	0.2224689	0.3627071	0.292588	up
A_23_P109026	KCNK15	2.8061762	3.0588117	3.1386852	0.2526355	0.332509	0.2925723	up
A_23_P129903	TRIM16L	5.175227	5.586018	5.3495054	0.4107909	0.1742783	0.2925346	up
A_23_P148919	CPT2	2.4742317	2.6490912	2.8844013	0.1748595	0.4101696	0.2925146	up
A_21_P0002876	lnc-RP11-723O4.6.1-1	-0.897723	-0.507943	-0.702485	0.3897805	0.1952381	0.2925093	up
A_23_P153236	CHMP2A	6.38459	6.7444596	6.609728	0.3598695	0.2251377	0.2925036	up
A_24_P83183	NELFA	1.8342056	2.1668048	2.0865717	0.3325992	0.2523661	0.2924826	up
A_22_P00000907	lnc-AIPL1-2	0.6495156	0.9831209	0.9008284	0.3336053	0.2513127	0.292459	up
A_23_P100602	TBCD	2.2493038	2.4707417	2.6127691	0.2214379	0.3634653	0.2924516	up
A_23_P46907	MICU1	0.8314691	1.1236396	1.1240878	0.2921705	0.2926188	0.2923946	up
A_23_P154675	SNRPB	5.7916956	6.018986	6.1491013	0.2272906	0.3574057	0.2923481	up
A_22_P00023755	B4GALT1-AS1	-3.135226	-2.706594	-2.979171	0.4286327	0.1560557	0.2923442	up
A_22_P00012038	MTURN	-0.745171	-0.363633	-0.542035	0.3815384	0.2031364	0.2923374	up
A_23_P54968	ST6GALNAC1	0.2614698	0.3210259	0.7862587	0.059556	0.5247889	0.2921724	up
A_23_P13899	GAPDH	8.13229	8.523449	8.325472	0.3911591	0.193182	0.2921705	up
A_22_P00016173	MIR22HG	0.5772715	0.8671131	0.871767	0.2898417	0.2944956	0.2921686	up
A_23_P17811	SEC14L2	-0.380556	-0.280166	0.1032109	0.10039	0.4837666	0.2920783	up
A_24_P218074	ZNF467	6.077055	6.490688	6.247551	0.4136329	0.170496	0.2920644	up
A_33_P3290622	PDZD11	1.9059849	2.1319728	2.263998	0.2259879	0.3580132	0.2920005	up
A_33_P3415052	NIPAL4	4.864813	5.1879687	5.1255617	0.3231559	0.2607489	0.2919524	up
A_23_P100711	PMP22	1.5573606	1.6689415	2.0296364	0.1115809	0.4722757	0.2919283	up
A_24_P152398	TP53AIP1	-2.860237	-2.418554	-2.718066	0.4416828	0.1421707	0.2919267	up
A_23_P130466	ZNF8	2.278224	2.7029672	2.4373035	0.4247432	0.1590796	0.2919114	up
A_23_P89163	EXOC7	4.243616	4.5720296	4.498994	0.3284135	0.2553778	0.2918956	up
A_33_P3232038	RBAK-RBAKDN	1.506434	1.7991257	1.7974143	0.2926917	0.2909803	0.291836	up
A_33_P3373375	CD81	5.651923	5.6682887	6.219122	0.0163655	0.5671988	0.2917821	up

A_21_P0011109	RPS27	8.877283	9.2000675	9.137991	0.3227844	0.2607079	0.2917461	up
A_33_P3352349	KRTAP9-1	2.42068	2.6537776	2.7710028	0.2330976	0.3503227	0.2917101	up
A_21_P0012915	XLOC_l2_012135	-2.819139	-2.498773	-2.556094	0.3203657	0.2630453	0.2917055	up
A_33_P3356886	DENND2C	-1.581007	-0.775241	-1.803449	0.8057656	-0.222442	0.2916617	up
A_33_P3215023	C6orf89	2.9722385	3.2074609	3.320201	0.2352223	0.3479624	0.2915924	up
A_24_P55148	HIST1H2BJ	2.4769754	2.7517333	2.7853107	0.2747579	0.3083353	0.2915466	up
A_23_P4885	AP2A1	0.4880467	0.8914166	0.6677051	0.4033699	0.1796584	0.2915142	up
A_23_P254852	OTOF	2.734313	3.0276942	3.02396	0.2933812	0.2896471	0.2915142	up
A_24_P128312	ZNF79	-0.661917	-0.076026	-0.664787	0.5858913	-0.00287	0.2915106	up
A_22_P00014568	lnc-SKIV2L2-1	-2.077961	-1.815014	-1.757911	0.2629466	0.3200498	0.2914982	up
A_22_P00016911	RNU4ATAC	9.994491	10.329886	10.242086	0.3353949	0.2475958	0.2914953	up
A_22_P00010560	GPR107	-0.38162	0.0033674	-0.183635	0.3849874	0.1979847	0.291486	up
A_23_P118095	RPL3L	-1.990796	-1.851912	-1.54673	0.1388836	0.4440656	0.2914746	up
A_32_P115375	lnc-SMG6-1	-1.662376	-1.296998	-1.444892	0.3653789	0.217484	0.2914314	up
A_22_P00011896	LINC01014	-2.033613	-1.750018	-1.734538	0.2835951	0.2990751	0.2913351	up
A_22_P00019828	lnc-PLCD3-1	0.7200112	1.1084714	0.9139738	0.3884602	0.1939626	0.2912114	up
A_23_P72117	SMPDL3A	0.6193309	1.0028543	0.8181996	0.3835235	0.1988688	0.2911961	up
A_23_P415401	KLF9	2.705742	3.3241305	2.6696167	0.6183887	-0.036125	0.2911317	up
A_24_P374516	TMSB4X	5.192734	5.5687137	5.3988295	0.3759799	0.2060957	0.2910378	up
A_21_P0008624	lnc-VPS18-1	-0.760212	-0.498355	-0.440052	0.2618575	0.3201604	0.291009	up
A_32_P148710	CFL1	5.6056976	5.8089585	5.9843864	0.2032609	0.3786888	0.2909749	up
A_22_P00000846	ZBED3-AS1	-3.148165	-2.541153	-3.17324	0.6070123	-0.025075	0.2909686	up
A_23_P41599	PCDHB8	-3.018838	-2.903573	-2.552393	0.1152651	0.466445	0.2908551	up
A_24_P21044	PSMG3	3.955162	4.251879	4.240053	0.2967172	0.2848911	0.2908042	up
A_23_P11915	GDAP2	0.722177	1.0591855	0.9667606	0.3370085	0.2445836	0.290796	up
A_23_P78438	ELP2	4.638937	5.0217824	4.8376827	0.3828454	0.1987457	0.2907956	up
A_23_P25121	FKBP11	5.079138	5.4850173	5.254799	0.4058795	0.1756611	0.2907703	up
A_21_P0013323	LOC101929463	-1.724354	-1.185164	-1.682045	0.5391908	0.0423093	0.29075	up
A_32_P222684	PRDM6	-2.636583	-2.402428	-2.289304	0.2341552	0.3472791	0.2907171	up
A_32_P14762	OOEP	0.5476093	1.0791817	0.5973787	0.5315723	0.0497694	0.2906709	up
A_24_P237586	ANKRD37	0.2933927	0.6140161	0.5539613	0.3206234	0.2605686	0.290596	up
A_33_P3248664	VARS	4.05274	4.383823	4.30276	0.3310828	0.25002	0.2905514	up
A_24_P295999	CD4	4.102851	4.5238748	4.2627687	0.4210239	0.1599178	0.2904708	up
A_23_P54991	DYNLL2	4.1666145	4.367065	4.547038	0.2004504	0.3804236	0.290437	up
A_22_P00012142	lnc-POLR2L-1	-2.062244	-1.525379	-2.018463	0.5368648	0.0437813	0.290323	up
A_24_P153840	FGD3	2.6169748	3.1897197	2.6247377	0.5727449	0.0077629	0.2902539	up
A_33_P3292307	UBIAD1	-1.380141	-1.227504	-0.952329	0.1526365	0.4278121	0.2902243	up
A_23_P53724	CLSTN3	-0.824846	-0.376949	-0.692415	0.4478965	0.132431	0.2901638	up
A_23_P16143	GTF2F1	3.1969557	3.4742198	3.4999046	0.2772641	0.302949	0.2901065	up
A_23_P131778	MANBAL	2.6827555	2.767242	3.1784554	0.0844865	0.4956999	0.2900932	up
A_22_P00010310	LOC101927503	-1.065343	-0.626444	-0.924099	0.438899	0.1412449	0.290072	up
A_33_P6461662	VPS11	4.900628	5.247275	5.13404	0.3466468	0.2334118	0.2900293	up
A_24_P370670	ZMYM6NB	1.8399334	2.1569457	2.1028757	0.3170123	0.2629423	0.2899773	up
A_23_P258340	PPIA	8.725062	9.091335	8.9387245	0.3662729	0.2136622	0.2899675	up
A_33_P3262575	BAIAP2L1	4.076792	4.4484305	4.2850733	0.3716388	0.2082815	0.2899601	up
A_21_P0013760	CHTF8	3.8463097	4.028483	4.243685	0.1821733	0.3973751	0.2897742	up
A_23_P133712	CYP39A1	-2.893306	-2.360827	-2.846237	0.5324791	0.0470691	0.2897741	up
A_22_P00015781	lnc-TAOK3-4	-1.74049	-1.384205	-1.517252	0.3562851	0.223239	0.289762	up
A_23_P392126	LYRM9	1.885272	2.281466	2.0685806	0.396194	0.1833086	0.2897513	up
A_33_P3382086	FAM195A	3.7253523	3.9380403	4.0921116	0.212688	0.3667593	0.2897236	up
A_19_P00322934	LINC00963	-0.269788	0.1563311	-0.116485	0.4261189	0.1533027	0.2897108	up
A_24_P167984	ATMIN	2.528038	2.8246713	2.8107643	0.2966332	0.2827263	0.2896798	up
A_19_P00320611	LOC101927151	-1.617445	-1.415065	-1.240486	0.2023792	0.3769584	0.2896688	up
A_23_P208961	MUM1	3.412034	3.6213298	3.7819939	0.2092958	0.3699598	0.2896278	up
A_24_P250335	SNRPA	4.1687346	4.4286885	4.4880085	0.259954	0.319274	0.289614	up
A_33_P3411477	NCCRP1	-1.097309	-0.848345	-0.767176	0.2489634	0.330133	0.2895482	up

A_23_P141208	DHRS7B	2.3424578	2.635448	2.6285353	0.2929902	0.2860775	0.2895339	up
A_23_P132139	C21orf58	0.6166043	1.1184936	0.6937637	0.5018892	0.0771594	0.2895243	up
A_23_P309967	DCTN5	1.1670055	1.3634739	1.54955	0.1964684	0.3825445	0.2895064	up
A_23_P149494	ABHD12	2.4556885	2.8532376	2.6371317	0.3975492	0.1814432	0.2894962	up
A_33_P3303519	CLEC12B	-0.353255	-0.169247	0.0415587	0.1840086	0.394814	0.2894113	up
A_33_P3213493	VAMP2	-2.853966	-2.160967	-2.968143	0.6929982	-0.114177	0.2894104	up
A_23_P4922	C19orf68	3.7641191	4.1763816	3.9306288	0.4122624	0.1665096	0.289386	up
A_23_P113701	PDGFA	0.6374445	0.9286308	0.9249558	0.2911863	0.2875114	0.2893488	up
A_23_P148121	EHBP1L1	2.354661	2.5911984	2.6968164	0.2365375	0.3421555	0.2893465	up
A_23_P8539	DAGLB	0.3229456	0.9034972	0.321065	0.5805516	-0.001881	0.2893355	up
A_33_P3354434	TRIM41	5.135297	5.3880773	5.461137	0.2527804	0.32584	0.2893102	up
A_23_P107036	TMEM11	4.209715	4.4239917	4.5739985	0.2142768	0.3642836	0.2892802	up
A_33_P3326617	FAHD1	-1.918713	-1.491674	-1.767298	0.4270392	0.1514149	0.289227	up
A_33_P3257678	HIST2H3A	6.3367767	6.5891166	6.6627398	0.2523398	0.325963	0.2891514	up
A_23_P390596	PSKH1	2.9100218	3.0332198	3.3648472	0.123198	0.4548254	0.2890117	up
A_23_P214907	MTHFD1L	1.1382251	1.7311416	1.1233249	0.5929165	-0.0149	0.2890081	up
A_33_P3286046	DPCD	3.5407639	3.7312741	3.9281034	0.1905103	0.3873396	0.2889249	up
A_33_P3239405	TMEM9	1.5510163	1.9081316	1.7716546	0.3571153	0.2206383	0.2888768	up
A_23_P62953	PBX1	-1.813786	-1.395912	-1.654069	0.4178739	0.1597166	0.2887952	up
A_32_P139229	ZNF543	0.4389992	0.6989522	0.756618	0.259953	0.3176189	0.2887859	up
A_23_P49816	ADAP2	-1.755908	-1.734503	-1.199789	0.0214052	0.556119	0.2887621	up
A_24_P592012	ZBTB46	-0.9839	-0.81121	-0.579167	0.1726899	0.4047327	0.2887113	up
A_21_P0003556	lnc-PDE6B-1	-3.379044	-3.240096	-2.940624	0.1389484	0.4384208	0.2886846	up
A_22_P00014842	lnc-SLC48A1-1	-2.93733	-2.9803	-2.317001	-0.042971	0.6203287	0.2886789	up
A_23_P256312	MST1R	3.551403	3.9038415	3.7761984	0.3524385	0.2247953	0.2886169	up
A_23_P54205	GPATCH2L	-3.036742	-2.683459	-2.81291	0.3532825	0.2238317	0.2885571	up
A_33_P3412847	LOC101927904	-1.293744	-0.896774	-1.113707	0.3969703	0.1800375	0.2885039	up
A_23_P47357	RPS25	9.699996	9.998587	9.97839	0.2985907	0.2783938	0.2884922	up
A_23_P152107	UBE2I	6.150134	6.436168	6.441084	0.2860341	0.2909498	0.288492	up
A_23_P201778	PTPN7	-0.003329	-0.308613	0.8788266	-0.305285	0.8821554	0.2884355	up
A_24_P125690	MRPL34	5.770088	6.0117984	6.1052475	0.2417102	0.3351593	0.2884347	up
A_33_P3242264	NSUN4	3.2987041	3.5369906	3.6372852	0.2382865	0.3385811	0.2884338	up
A_24_P372932	PEX5	-1.876198	-1.577103	-1.598453	0.2990952	0.2777457	0.2884204	up
A_24_P95038	PPIA	9.491296	9.753296	9.806136	0.2620001	0.3148403	0.2884202	up
A_23_P38015	FBRS	6.492463	6.7233043	6.838396	0.2308412	0.345933	0.2883871	up
A_33_P3381870	DMTN	4.8256254	5.025658	5.2022533	0.2000327	0.3766279	0.2883303	up
A_23_P61633	TNK2	4.9279346	5.260429	5.17198	0.3324943	0.2440453	0.2882698	up
A_33_P3329444	MAMSTR	-0.616832	-0.207809	-0.449328	0.4090238	0.1675038	0.2882638	up
A_23_P107313	SDF2	2.8962278	3.1759753	3.1930008	0.2797475	0.296773	0.2882602	up
A_33_P3266744	SYTL1	3.527257	3.8353505	3.7954807	0.3080936	0.2682238	0.2881587	up
A_22_P00013881	LOC100144595	1.0923882	1.300837	1.4601865	0.2084489	0.3677983	0.2881236	up
A_22_P00004465	lnc-CPEB4-1	1.4106703	1.7775912	1.6199307	0.366921	0.2092605	0.2880907	up
A_33_P3415698	TMIGD2	-0.605845	-0.364652	-0.270958	0.2411938	0.3348875	0.2880406	up
A_22_P00011671	LINC00271	-2.777661	-2.167005	-2.812276	0.6106556	-0.034615	0.2880203	up
A_24_P143138	FGD1	-1.973727	-1.571138	-1.800544	0.4025884	0.1731825	0.2878854	up
A_33_P3291976	TERF1	-3.242197	-2.900338	-3.008368	0.3418596	0.233829	0.2878443	up
A_21_P0000563	SNORA80B	2.288228	2.6564727	2.4955864	0.3682447	0.2073584	0.2878015	up
A_24_P141688	PCBP2	0.2491388	0.5719633	0.5018001	0.3228245	0.2526612	0.2877429	up
A_33_P3382560	RPL23A	8.591379	8.9028225	8.855357	0.3114433	0.263978	0.2877107	up
A_23_P20122	ZC3HAV1	0.0554781	0.2917461	0.3945265	0.236268	0.3390484	0.2876582	up
A_23_P215265	GNA12	-0.319615	-0.059888	-0.004028	0.2597275	0.3155875	0.2876575	up
A_23_P28688	CPSF3	3.505476	3.8468976	3.7392645	0.3414216	0.2337885	0.287605	up
A_21_P0010343	LOC102724679	-1.104443	-0.849555	-0.784187	0.2548881	0.3202558	0.2875719	up
A_21_P0001469	lnc-C1orf195-3	-2.922515	-2.649337	-2.620746	0.2731783	0.301769	0.2874737	up
A_33_P3360728	BLVRB	6.544015	6.9908166	6.6721106	0.4468017	0.1280956	0.2874486	up
A_22_P00014212	LOC101928323	-0.164217	0.1730275	0.0733061	0.337245	0.2375236	0.2873843	up

A_23_P135730	ZNF627	0.9218884	1.3008142	1.117631	0.3789258	0.1957426	0.2873342	up
A_33_P3369371	GPX3	-1.114609	-0.680871	-0.973764	0.4337387	0.1408448	0.2872918	up
A_24_P889720	UBC	8.975582	9.361856	9.163849	0.3862743	0.1882668	0.2872705	up
A_33_P3418798	MSANTD1	-2.412435	-2.054128	-2.196264	0.3583074	0.2161708	0.2872391	up
A_23_P216845	GFI1B	-2.918292	-2.680848	-2.581295	0.2374437	0.3369963	0.28722	up
A_23_P256835	TRAPPC12	2.67488	3.0169063	2.9071732	0.3420262	0.2322931	0.2871597	up
A_33_P3228807	SPEG	-0.29996	-0.07779	0.0521073	0.2221694	0.352067	0.2871182	up
A_33_P3388491	PQLC2	10.135116	10.470512	10.373575	0.3353968	0.2384596	0.2869282	up
A_33_P3354771	MEF2BNB	0.0432067	0.3530893	0.3071284	0.3098826	0.2639217	0.2869022	up
A_33_P3640101	LOC400684	-0.881786	-0.397637	-0.792401	0.484149	0.089385	0.286767	up
A_33_P3333648	OR51I2	-1.717157	-1.436591	-1.424223	0.2805662	0.2929349	0.2867506	up
A_23_P118406	C17orf62	1.2067428	1.3561983	1.6307039	0.1494556	0.4239612	0.2867084	up
A_21_P0012872	XLOC_l2_011873	-2.667244	-2.915291	-1.84579	-0.248047	0.8214538	0.2867035	up
A_23_P67589	C19orf60	5.2004967	5.400187	5.5741577	0.1996903	0.373661	0.2866757	up
A_21_P0010802	XLOC_l2_001448	-1.755506	-1.53658	-1.401125	0.2189255	0.3543801	0.2866528	up
A_19_P00812340	FTL	5.99467	6.671347	5.8912106	0.6766772	-0.103459	0.2866089	up
A_23_P210496	PCIF1	1.1278973	1.4107814	1.4182043	0.2828841	0.290307	0.2865956	up
A_23_P152620	TNFSF13	-1.507877	-1.312588	-1.13013	0.1952887	0.3777471	0.2865179	up
A_32_P32739	NAGS	3.054719	3.3757854	3.3066587	0.3210664	0.2519398	0.2865031	up
A_33_P3412095	PEX7	-1.862924	-1.498946	-1.65391	0.3639774	0.2090135	0.2864954	up
A_22_P00015929	lnc-TCN2-1	1.1800685	1.5352364	1.3978572	0.3551679	0.2177887	0.2864783	up
A_33_P3296852	LOC729162	9.283603	9.579361	9.560786	0.2957583	0.2771835	0.2864709	up
A_21_P0013270	LINC01347	1.9020691	2.286819	2.0901394	0.3847499	0.1880703	0.2864101	up
A_33_P3297030	VPS53	-0.104011	0.2483892	0.1164041	0.3524003	0.2204151	0.2864077	up
A_33_P3244021	MAVS	3.6888332	3.9493241	4.0011063	0.2604909	0.312273	0.286382	up
A_33_P3394993	PREX2	-2.530271	-2.355455	-2.132481	0.1748157	0.3977904	0.286303	up
A_23_P57413	PPM1F	-0.356789	0.1650643	-0.30605	0.5218535	0.0507393	0.2862964	up
A_23_P38482	PHF23	2.90209	3.318954	3.057763	0.4168639	0.155673	0.2862685	up
A_22_P00015081	lnc-SNURF-3	-0.961761	-0.649276	-0.701728	0.3124852	0.2600327	0.2862589	up
A_24_P253723	MIR22HG	0.4543843	0.8909898	0.5902743	0.4366055	0.13589	0.2862477	up
A_22_P00023928	lnc-RP11-293M10.1.1-1	-2.636234	-1.959855	-2.740242	0.6763794	-0.104008	0.2861856	up
A_23_P85392	RER1	3.5478392	3.9457068	3.7222242	0.3978677	0.1743851	0.2861264	up
A_23_P149221	HECTD3	-0.009947	0.4699612	0.0822721	0.479908	0.0922189	0.2860634	up
A_23_P310483	C8orf58	0.476243	1.0080986	0.5165052	0.5318556	0.0402622	0.2860589	up
A_33_P3290602	MMP24-AS1	2.1838064	2.3607469	2.5789127	0.1769404	0.3951063	0.2860234	up
A_19_P00800264	OTUD6B-AS1	3.4297981	3.6675649	3.7640324	0.2377667	0.3342342	0.2860005	up
A_23_P73667	RIBC1	-1.133147	-0.562848	-1.131445	0.5702987	0.0017018	0.2860003	up
A_32_P58074	RPS3A	8.254327	8.600956	8.479668	0.3466291	0.2253408	0.285985	up
A_33_P3225230	SIN3B	4.9858894	5.3899236	5.1537333	0.4040341	0.1678438	0.285939	up
A_24_P119609	MYO1D	-1.160392	-0.973951	-0.775023	0.1864414	0.3853698	0.2859056	up
A_22_P00020012	ANKEF1	-1.437778	-1.106921	-1.196853	0.3308573	0.2409253	0.2858913	up
A_33_P3267482	KIAA1804	4.2387304	4.6391587	4.409871	0.4004283	0.1711407	0.2857845	up
A_23_P17695	SLC37A1	0.342391	0.5479045	0.7082858	0.2055135	0.3658948	0.2857041	up
A_21_P0011339	XLOC_l2_004772	-3.09555	-2.705395	-2.914367	0.3901553	0.1811833	0.2856693	up
A_22_P00010319	lnc-MUC5B-1	0.2025495	0.4535584	0.5228405	0.251009	0.320291	0.28565	up
A_33_P3381751	TIAM1	0.1416001	0.4823785	0.3719831	0.3407784	0.2303829	0.2855806	up
A_33_P3311056	SDHA	6.135866	6.448878	6.3939543	0.3130117	0.2580881	0.2855499	up
A_23_P500873	RAD51D	0.3114219	0.3165774	0.8772755	0.0051556	0.5658536	0.2855046	up
A_24_P372048	YKT6	4.052204	4.3366504	4.3387575	0.2844462	0.2865534	0.2854998	up
A_24_P913227	CDC23	-2.454132	-2.080911	-2.2564	0.3732209	0.1977315	0.2854762	up
A_22_P00000102	SNHG6	5.40526	5.815498	5.5659494	0.4102378	0.1606894	0.2854636	up
A_23_P1043	C1orf106	2.4596987	2.6481805	2.8420916	0.1884818	0.3823929	0.2854373	up
A_22_P00016621	LOC100507316	1.8988504	2.110722	2.257843	0.2118716	0.3589926	0.2854321	up
A_33_P3424222	HLA-DQB1	-1.976047	-1.524822	-1.85644	0.4512253	0.119607	0.2854161	up
A_24_P402690	ITM2C	5.007823	5.327431	5.2589025	0.3196082	0.2510796	0.2853439	up

A_33_P3214298	IMPDH2	3.9588146	4.4024587	4.0858192	0.4436441	0.1270046	0.2853243	up
A_23_P204947	GJB2	4.518875	4.5502896	5.0580006	0.0314145	0.5391254	0.28527	up
A_33_P3259022	SLC6A2	0.2143712	0.7045903	0.2946386	0.4902191	0.0802674	0.2852433	up
A_23_P404005	FAM58A	4.652664	4.814879	5.060771	0.1622148	0.4081068	0.2851608	up
A_21_P0009771	lnc-IGFL3-1	-1.530148	-1.243771	-1.246319	0.286377	0.2838292	0.2851031	up
A_23_P106661	CMTM1	0.0934544	0.5149145	0.2421064	0.4214602	0.1486521	0.2850561	up
A_33_P3317543	GTF2IRD2B	-2.615934	-1.82656	-2.835235	0.7893744	-0.219301	0.2850369	up
A_22_P00003465	MMP25-AS1	3.2954378	3.692409	3.4684668	0.3969712	0.173029	0.2850001	up
A_23_P129334	CLCN7	0.8883843	1.2289081	1.1178441	0.3405237	0.2294598	0.2849917	up
A_22_P00004902	lnc-DALRD3-1	-1.868038	-1.540762	-1.625369	0.3272758	0.2426691	0.2849724	up
A_23_P15669	C17orf85	-0.30541	0.1451144	-0.18621	0.4505248	0.1192007	0.2848628	up
A_33_P3299165	LINC00544	-3.09161	-2.627049	-2.986672	0.4645615	0.1049378	0.2847496	up
A_19_P00322968	LINC00963	1.1108532	1.603631	1.1874456	0.4927778	0.0765924	0.2846851	up
A_24_P46577	ZNRD1	3.7908783	4.1175647	4.033511	0.3266864	0.2426329	0.2846596	up
A_33_P3375541	CD3D	-2.468018	-2.21358	-2.15318	0.2544375	0.3148377	0.2846376	up
A_22_P00012541	LINC00476	0.6029649	0.8983927	0.87676	0.2954278	0.2737951	0.2846115	up
A_33_P3291454	CCDC172	-0.760201	-0.345013	-0.606219	0.4151883	0.1539826	0.2845855	up
A_33_P3224331	DDX3Y	2.1247501	2.451939	2.3663063	0.327189	0.2415562	0.2843726	up
A_22_P00021723	LOC101927078	1.4559774	1.8533168	1.6273084	0.3973393	0.1713309	0.2843351	up
A_24_P355493	LHPP	0.3194094	0.7391114	0.4683194	0.4197021	0.1489101	0.2843061	up
A_24_P280901	RPL21	7.9118347	8.194254	8.197969	0.2824192	0.2861347	0.284277	up
A_24_P47467	TMEM109	5.298237	5.5604997	5.604397	0.2622628	0.30616	0.2842114	up
A_33_P3229873	LRRC27	-0.72308	-0.470524	-0.407447	0.2525554	0.3156328	0.2840941	up
A_21_P0011498	LOC100506281	-2.206819	-1.637691	-2.20786	0.5691285	-0.001041	0.2840439	up
A_23_P362712	ABHD11	1.4620333	1.9782729	1.5138445	0.5162396	0.0518112	0.2840254	up
A_23_P366216	HIST1H2BH	4.938901	5.1312623	5.314576	0.1923614	0.3756752	0.2840183	up
A_19_P00318404	LINC00989	-0.170935	0.2177253	0.0083075	0.3886604	0.1792426	0.2839515	up
A_24_P101391	YBX1	5.9237204	6.2025	6.212756	0.2787795	0.2890358	0.2839077	up
A_33_P3325634	ERICH4	1.2310605	1.5652485	1.4646668	0.334188	0.2336063	0.2838972	up
A_23_P330999	MED23	-0.649405	-0.491005	-0.240187	0.1583996	0.4092178	0.2838087	up
A_23_P104362	ECHS1	6.0921335	6.386597	6.365061	0.2944636	0.2729273	0.2836955	up
A_33_P3404749	FMN1	0.0295835	0.4342136	0.1923266	0.4046302	0.1627431	0.2836866	up
A_23_P27005	DHRS11	0.7368093	1.0312157	1.0095134	0.2944064	0.2727041	0.2835553	up
A_23_P17430	RBM38	1.6977177	1.9284186	2.034068	0.230701	0.3363504	0.2835257	up
A_23_P12866	PSTK	0.827096	1.057816	1.1633844	0.23072	0.3362885	0.2835042	up
A_23_P122233	MRPL22	5.77652	6.0529256	6.0671167	0.2764058	0.290597	0.2835014	up
A_23_P149064	PTPRU	1.2623649	1.6655612	1.4261136	0.4031963	0.1637487	0.2834725	up
A_23_P148785	SFT2D2	0.9673581	1.0290327	1.4726105	0.0616746	0.5052524	0.2834635	up
A_24_P70993	CD99	3.7871447	4.2443967	3.8967714	0.457252	0.1096268	0.2834394	up
A_23_P137381	ID3	-0.139759	0.1056299	0.1817036	0.2453885	0.3214622	0.2834253	up
A_33_P3275741	FCN3	-1.760327	-1.371288	-1.582569	0.3890386	0.1777582	0.2833984	up
A_22_P00021117	lnc-ESCO2-2	-2.305319	-2.193938	-1.850019	0.1113811	0.4553003	0.2833407	up
A_23_P329152	ILF3	3.6914968	3.8794131	4.070259	0.1879163	0.3787623	0.2833393	up
A_24_P643587	ASB8	-1.457368	-1.177347	-1.170842	0.2800207	0.2865257	0.2832732	up
A_23_P67312	ZNF136	-0.524269	-0.218904	-0.263325	0.3053656	0.2609439	0.2831547	up
A_23_P164912	LIN7B	1.7200909	2.1267056	1.8797407	0.4066148	0.1596499	0.2831323	up
A_22_P00011386	lnc-P4HA2-2	0.4402046	0.8333359	0.6132665	0.3931313	0.1730619	0.2830966	up
A_33_P3315874	DHX30	5.577709	5.825003	5.896557	0.247294	0.3188477	0.2830708	up
A_33_P3383436	DHRS4L2	3.047948	3.315579	3.3462934	0.2676311	0.2983456	0.2829883	up
A_21_P0000094	CD33	-2.655393	-2.312834	-2.432021	0.3425591	0.2233722	0.2829657	up
A_23_P53614	BRAP	0.5444627	0.733736	0.9205456	0.1892734	0.3760829	0.2826781	up
A_21_P0007692	LOC102724247	-2.965395	-2.701536	-2.663924	0.2638583	0.3014708	0.2826645	up
A_23_P66599	VPS25	4.1637297	4.4416337	4.4510126	0.277904	0.2872829	0.2825935	up
A_33_P3608172	GPS2	3.6466837	4.090627	3.767909	0.4439435	0.1212254	0.2825844	up
A_22_P00018462	MIA3	-1.141958	-0.817155	-0.901622	0.3248034	0.2403359	0.2825696	up
A_23_P30126	FGFBP1	2.7344923	3.0869975	2.9469328	0.3525052	0.2124405	0.2824728	up

A_33_P3417820	NOL12	3.8457966	4.1619086	4.094495	0.316112	0.2486982	0.2824051	up
A_23_P356330	DESI1	2.7103357	2.9708953	3.0145302	0.2605596	0.3041945	0.282377	up
A_23_P316812	AKR7A2P1	-0.188882	-0.036963	0.2238097	0.1519189	0.4126916	0.2823052	up
A_33_P3265374	HOMER3	5.964014	6.180079	6.3125515	0.2160649	0.3485375	0.2823012	up
A_33_P3343467	LINC00957	-1.682471	-1.373125	-1.427401	0.3093457	0.2550702	0.282208	up
A_23_P115407	GSTM1	-0.594138	-0.413367	-0.210551	0.1807704	0.3835864	0.2821784	up
A_23_P201400	PPOX	1.2108326	1.4891763	1.4968143	0.2783437	0.2859817	0.2821627	up
A_23_P206310	KIAA0513	0.7074704	1.2686963	0.7104874	0.5612259	0.0030169	0.2821214	up
A_33_P3288364	SPATA2L	4.8455143	5.1167483	5.138397	0.271234	0.2928829	0.2820585	up
A_21_P0000026	C15orf40	2.6374865	3.0276456	2.8113403	0.3901591	0.1738539	0.2820065	up
A_22_P00000269	lnc-AC007557.1.1-3	-1.245628	-0.913147	-1.014132	0.3324809	0.2314963	0.2819886	up
A_23_P158662	GPS1	3.7940445	3.904222	4.247802	0.1101775	0.4537573	0.2819674	up
A_32_P20367	RPS7	8.649845	9.002501	8.861084	0.3526554	0.2112389	0.2819471	up
A_23_P67913	GMPPA	0.8157082	0.8814268	1.3138151	0.0657187	0.498107	0.2819128	up
A_33_P3388080	LINC01220	0.1400247	0.3109164	0.5329127	0.1708918	0.3928881	0.2818899	up
A_33_P3339202	LINC01225	-0.138616	0.1541591	0.1323881	0.2927752	0.2710042	0.2818897	up
A_24_P297539	UBE2C	5.720194	5.940335	6.0636415	0.2201409	0.3434477	0.2817943	up
A_32_P21384	RPL17	7.81901	8.181395	8.020162	0.3623848	0.2011519	0.2817683	up
A_32_P30710	RPL23	10.244678	10.584757	10.467711	0.3400793	0.223033	0.2815561	up
A_19_P00316778	lnc-SLC35F5-4	-2.744498	-2.584908	-2.341002	0.1595898	0.4034956	0.2815427	up
A_23_P341443	MNT	4.1132402	4.5257792	4.2635965	0.412539	0.1503563	0.2814477	up
A_23_P65967	SLC38A7	1.7963047	1.9090834	2.2463074	0.1127787	0.4500027	0.2813907	up
A_23_P55174	G6PC3	0.6457171	0.8076353	1.0465155	0.1619182	0.4007983	0.2813582	up
A_23_P148609	PLAC1	-1.795247	-1.302165	-1.725794	0.4930821	0.0694532	0.2812676	up
A_21_P0000543	ATP1A1-AS1	-1.199839	-0.613528	-1.223624	0.5863109	-0.023786	0.2812626	up
A_23_P47247	TCP11L1	-1.119248	-0.572088	-1.10389	0.5471602	0.0153589	0.2812595	up
A_33_P3320301	LINC00493	6.7064066	6.9521985	7.0230074	0.2457919	0.3166008	0.2811964	up
A_33_P3353581	IGBP1	2.8368244	3.2967706	2.9391594	0.4599462	0.102335	0.2811406	up
A_23_P152406	CAPNS2	1.1859112	1.5529456	1.3811283	0.3670344	0.1952171	0.2811258	up
A_21_P0009821	LINC00659	-1.106269	-0.991665	-0.65873	0.1146045	0.4475398	0.2810721	up
A_22_P00003498	LOC100506282	-0.183465	0.1644831	0.0307317	0.3479476	0.2141962	0.2810719	up
A_23_P111843	ZNHIT1	6.3673916	6.5959744	6.7008314	0.2285829	0.3334398	0.2810113	up
A_23_P80336	TOMM22	5.026703	5.28591	5.329418	0.2592073	0.3027153	0.2809613	up
A_33_P3369452	TANGO2	0.7990885	0.9726563	1.1873746	0.1735678	0.3882861	0.2809269	up
A_23_P28638	PLEKHB2	3.0395927	3.3493218	3.2916107	0.3097291	0.252018	0.2808735	up
A_23_P66421	NAT9	3.4073858	3.78202	3.5943298	0.3746343	0.186944	0.2807891	up
A_23_P20022	HILPDA	3.061408	3.3556495	3.328722	0.2942414	0.267314	0.2807777	up
A_24_P342591	RERE	-1.314813	-1.167571	-0.900844	0.1472416	0.4139686	0.2806051	up
A_23_P99226	SIRT4	-2.121927	-1.659382	-2.023286	0.4625454	0.0986409	0.2805932	up
A_21_P0002010	LINC00299	-2.255382	-2.02789	-1.921724	0.2274914	0.3336573	0.2805743	up
A_21_P0003219	lnc-OXNAD1-1	-0.719473	-0.265065	-0.613073	0.4544077	0.1063995	0.2804036	up
A_32_P34876	WDR93	-1.477147	-0.971264	-1.422262	0.5058832	0.0548854	0.2803843	up
A_33_P3359268	HMG20B	5.3726015	5.627299	5.678648	0.2546973	0.3060465	0.2803719	up
A_23_P346302	ZMAT2	4.8499556	5.1892047	5.071335	0.3392491	0.2213793	0.2803142	up
A_33_P3244643	FAM183A	1.1520863	1.5462685	1.3185258	0.3941822	0.1664395	0.2803109	up
A_23_P69970	SOX30	-2.11992	-1.472935	-2.206307	0.6469846	-0.086387	0.2802988	up
A_23_P258093	AGPAT1	2.6523685	2.9950938	2.8701868	0.3427253	0.2178183	0.2802718	up
A_32_P88120	YPEL1	-2.222445	-1.859701	-2.024672	0.3627434	0.1977725	0.2802579	up
A_23_P344531	SYNPO	1.6860733	2.1262517	1.806356	0.4401784	0.1202827	0.2802305	up
A_23_P170901	PACRG	-2.387341	-2.055866	-2.158379	0.3314753	0.2289619	0.2802186	up
A_32_P58425	PHF13	2.9547873	3.2632232	3.206685	0.3084359	0.2518978	0.2801669	up
A_23_P100074	AVEN	2.5510283	2.82513	2.8372154	0.2741017	0.2861872	0.2801445	up
A_23_P33022	POLR2L	7.746668	7.907377	8.146175	0.1607089	0.3995075	0.2801082	up
A_33_P3257222	COMTD1	3.4906807	3.8249364	3.7163925	0.3342557	0.2257118	0.2799838	up
A_23_P54517	TYRO3	2.854127	3.181047	3.0871305	0.32692	0.2330036	0.2799618	up

A_22_P00001139	LOC101928830	-2.148083	-1.70265	-2.033791	0.4454327	0.1142921	0.2798624	up
A_22_P00003270	lnc-CALM2-1	-1.069329	-0.703379	-0.875837	0.3659501	0.1934924	0.2797213	up
A_33_P3389342	ARID5A	-1.490693	-1.086488	-1.33548	0.4042049	0.1552129	0.2797089	up
A_23_P203790	OS9	4.5813456	4.962403	4.759701	0.3810573	0.1783552	0.2797062	up
A_21_P0009917	LOC101927842	-2.771227	-2.265956	-2.71709	0.5052702	0.0541365	0.2797034	up
A_33_P3400429	UBE3A	-1.928355	-1.498915	-1.798633	0.4294395	0.1297216	0.2795806	up
A_23_P150238	C11orf68	2.119381	2.5672698	2.2305565	0.4478889	0.1111755	0.2795322	up
A_23_P111481	SRRT	5.58321	5.957276	5.7679863	0.3740659	0.1847763	0.2794211	up
A_23_P98431	HMBS	5.4055176	5.711057	5.658782	0.3055396	0.2532644	0.279402	up
A_33_P3233459	LINC00994	-2.293405	-1.99808	-2.029956	0.2953243	0.2634487	0.2793865	up
A_22_P00010519	LOC101927172	-2.064795	-1.474813	-2.096011	0.5899816	-0.031216	0.2793827	up
A_22_P00014955	lnc-SLTM-1	-0.210182	0.0991483	0.0392265	0.30933	0.2494083	0.2793691	up
A_23_P55091	FTSJ3	4.3012133	4.554953	4.606084	0.2537398	0.3048706	0.2793052	up
A_32_P112881	SNX12	5.307295	5.5702186	5.602895	0.2629237	0.2955999	0.2792618	up
A_33_P3394312	OR2A2	-2.03088	-1.827046	-1.67656	0.2038336	0.3543196	0.2790766	up
A_23_P41976	CCDC127	2.8229008	3.0345187	3.169218	0.211618	0.3463173	0.2789676	up
A_23_P67529	KCNN4	5.2636404	5.627785	5.457402	0.3641448	0.1937618	0.2789533	up
A_23_P361820	ATG2A	0.736548	0.8409672	1.1899805	0.1044192	0.4534326	0.2789259	up
A_23_P73660	TRAPPC2	0.8954625	1.2196136	1.1290574	0.324151	0.2335949	0.278873	up
A_33_P3391275	LINC00494	-0.9594	-0.772941	-0.588132	0.1864591	0.3712673	0.2788632	up
A_23_P111041	HIST1H2BI	3.9827194	4.2404046	4.2826767	0.2576852	0.2999573	0.2788212	up
A_23_P202683	CDHR5	-3.141646	-2.39071	-3.335007	0.7509358	-0.19336	0.2787877	up
A_23_P10497	ATP9B	1.322042	1.608408	1.5932007	0.286366	0.2711587	0.2787624	up
A_23_P23438	SEMA4A	-1.353677	-1.18001	-0.969825	0.1736674	0.383852	0.2787597	up
A_23_P37949	THOC6	-1.079415	-1.033084	-0.56823	0.0463314	0.5111857	0.2787585	up
A_21_P0011303	PDCD6IPP2	4.471608	4.6803813	4.820304	0.2087731	0.3486958	0.2787345	up
A_23_P11729	ZBTB7B	-0.104656	0.2116542	0.1363187	0.3163099	0.2409744	0.2786422	up
A_23_P86532	BICC1	-0.31089	-0.447094	0.3825607	-0.136204	0.6934505	0.2786233	up
A_33_P3355055	ARL6IP4	3.252655	3.5572581	3.5052547	0.3046031	0.2525997	0.2786014	up
A_33_P3234277	HLA-DPA1	-1.517122	-1.216636	-1.260502	0.3004861	0.2566199	0.278553	up
A_23_P154771	DUSP15	2.2563038	2.5319963	2.5376902	0.2756925	0.2813864	0.2785394	up
A_23_P331908	LOC101060179	0.9496136	1.3933215	1.062932	0.4437079	0.1133184	0.2785132	up
A_24_P370420	WBSCR16	1.2884264	1.5151176	1.6185741	0.2266913	0.3301477	0.2784195	up
A_33_P3283400	LOC100507747	2.1218185	2.4150186	2.3852682	0.2932	0.2634497	0.2783248	up
A_23_P314726	PI4KB	2.3614378	2.8417344	2.4376717	0.4802966	0.0762339	0.2782652	up
A_33_P3349145	TTLL1	0.3259277	0.4254947	0.7828221	0.0995669	0.4568944	0.2782307	up
A_19_P00318284	LINC01122	0.5422764	0.7603512	0.8806539	0.2180748	0.3383775	0.2782261	up
A_24_P61753	CLUH	2.1877098	2.4482226	2.4836464	0.2605128	0.2959366	0.2782247	up
A_23_P65089	DDX47	6.048093	6.2778544	6.374117	0.2297616	0.3260241	0.2778928	up
A_33_P3270793	FAM84A	-2.801171	-3.029818	-2.016822	-0.228646	0.7843494	0.2778516	up
A_32_P57237	RPL21	8.693498	8.997177	8.945424	0.3036795	0.2519264	0.2778029	up
A_23_P256375	STX4	3.355091	3.737842	3.527916	0.382751	0.1728249	0.2777879	up
A_23_P152949	LRRC46	-1.9291	-1.654162	-1.648581	0.2749386	0.2805195	0.277729	up
A_24_P17722	TBC1D9B	5.0286827	5.3241477	5.2885838	0.295465	0.2599011	0.277683	up
A_33_P3400643	LINC00865	-1.121243	-0.882597	-0.804673	0.2386456	0.3165698	0.2776077	up
A_33_P3348091	LTBP4	0.4468927	0.7452626	0.7036672	0.2983699	0.2567744	0.2775722	up
A_32_P104746	ZFYVE28	-0.150935	-0.02793	0.2810173	0.1230049	0.431952	0.2774785	up
A_23_P255672	ABLIM2	-0.35719	-0.062408	-0.097137	0.2947817	0.2600527	0.2774172	up
A_33_P3264269	MOV10	-1.553259	-1.18068	-1.371029	0.3725786	0.1822295	0.2774041	up
A_22_P00021743	lnc-C15orf57-1	-0.547514	-0.371145	-0.169326	0.1763697	0.3781886	0.2772791	up
A_33_P3246083	OXSM	2.9948702	3.2323003	3.3119268	0.2374301	0.3170567	0.2772434	up
A_23_P39656	RNF25	1.2971077	1.5465717	1.6019878	0.249464	0.3048801	0.2771721	up
A_23_P97736	NCDN	1.1616435	1.3858705	1.4916682	0.224227	0.3300247	0.2771258	up
A_22_P00021487	TRABD2B	-2.193729	-1.812773	-2.020522	0.3809562	0.1732078	0.277082	up
A_33_P3215134	ZNF777	0.18536	0.4856405	0.4392314	0.3002806	0.2538714	0.277076	up
A_32_P184518	RPL21	9.119432	9.421091	9.371918	0.3016586	0.2524853	0.277072	up

A_22_P00015592	lnc-STX8-2	-0.728251	-0.489943	-0.412558	0.2383084	0.3156929	0.2770007	up
A_19_P00318152	SNHG11	4.090108	4.527412	4.2067785	0.437304	0.1166706	0.2769873	up
A_22_P00009871	lnc-MFAP4-1	1.292232	1.7746844	1.3637447	0.4824524	0.0715127	0.2769826	up
A_33_P3390391	GLTSCR1	-0.404851	0.0374007	-0.293186	0.4422522	0.1116657	0.2769589	up
A_33_P3380523	PRTFDC1	0.4471211	1.0009832	0.4470921	0.5538621	-2.91E-05	0.2769165	up
A_24_P169073	FAM131C	3.699688	4.068627	3.8845692	0.3689389	0.1848812	0.2769101	up
A_23_P27147	ANAPC11	8.662177	9.02878	8.849371	0.3666029	0.1871939	0.2768984	up
A_23_P154086	BCS1L	3.8988943	4.111735	4.2397327	0.2128406	0.3408384	0.2768395	up
A_33_P3272075	OPRD1	-2.444933	-2.12555	-2.210856	0.3193829	0.234077	0.2767299	up
A_32_P52206	TYW1	1.0996461	1.2743063	1.4784098	0.1746602	0.3787637	0.2767119	up
A_33_P3329088	PRSS8	6.5964193	6.991657	6.7545977	0.3952375	0.1581783	0.2767079	up
A_23_P160559	ECM1	2.6920357	3.0715451	2.8659372	0.3795095	0.1739016	0.2767055	up
A_23_P397238	FKBP1A	2.1036816	2.4022126	2.3583765	0.2985311	0.2546949	0.276613	up
A_23_P215787	HBP1	-0.079721	0.5289822	-0.135215	0.6087036	-0.055494	0.2766049	up
A_22_P00009585	BREA2	-0.877608	-0.562591	-0.639433	0.3150172	0.2381744	0.2765958	up
A_21_P0008178	lnc-PCDH9-3	1.504571	1.8026047	1.7596364	0.2980337	0.2550654	0.2765496	up
A_23_P502142	FYN	0.3993387	0.795207	0.5565424	0.3958683	0.1572037	0.276536	up
A_33_P3390048	INO80E	4.3654394	4.620875	4.6630163	0.2554355	0.2975769	0.2765062	up
A_23_P7655	BNIP1	0.98666	1.3034153	1.2228684	0.3167553	0.2362084	0.2764819	up
A_22_P00003109	lnc-C9orf103-1	5.34186	5.577084	5.6595125	0.2352243	0.3176527	0.2764385	up
A_23_P421011	KAZALD1	0.4104137	0.6234732	0.750217	0.2130594	0.3398032	0.2764313	up
A_22_P00001540	lnc-ARHGEF2-2	-0.768742	-0.419508	-0.565194	0.3492336	0.2035484	0.276391	up
A_23_P369456	SYS1	-0.283438	-0.12577	0.1116619	0.1576681	0.3950996	0.2763839	up
A_33_P3351249	CXCL16	2.3551188	2.5101552	2.7528439	0.1550365	0.3977251	0.2763808	up
A_32_P168886	EIF4H	7.3333664	7.6537175	7.565467	0.3203511	0.2321005	0.2762258	up
A_22_P00012883	lnc-RBM11-2	-0.949205	-0.71099	-0.635118	0.2382145	0.3140869	0.2761507	up
A_22_P00020109	lnc-MPHOSPH8-3	-2.059982	-1.578122	-1.989599	0.4818602	0.0703836	0.2761219	up
A_32_P224149	FKBP15	2.582368	2.8738637	2.8430672	0.2914958	0.2606993	0.2760975	up
A_24_P266285	IQCE	-0.958366	-0.484474	-0.880102	0.4738922	0.0782642	0.2760782	up
A_23_P151662	MAX	0.8086085	1.1058164	1.0633736	0.2972078	0.254765	0.2759864	up
A_23_P132294	GGA1	5.621166	5.913484	5.8807945	0.2923179	0.2596283	0.2759731	up
A_19_P00320723	SRP14-AS1	-2.226374	-1.422284	-2.478644	0.8040905	-0.25227	0.2759104	up
A_21_P0005545	lnc-XRCC2-4	-1.564956	-1.185522	-1.392607	0.3794336	0.172349	0.2758913	up
A_33_P3271171	CNOT3	0.3195176	0.5836601	0.6071458	0.2641425	0.2876282	0.2758853	up
A_24_P813147	TUBB8	3.5663748	3.706781	3.9774199	0.1404061	0.4110451	0.2757256	up
A_23_P118061	CKLF	3.9694824	4.317764	4.1726046	0.3482814	0.2031221	0.2757018	up
A_24_P379353	CCDC24	-1.400631	-1.463107	-0.786789	-0.062476	0.6138415	0.2756827	up
A_33_P3252394	GADD45G	2.5892086	3.0481486	2.6815977	0.45894	0.0923891	0.2756646	up
A_23_P111132	HSPA1A	5.888748	6.238973	6.0896387	0.350225	0.2008905	0.2755578	up
A_23_P2725	RPL21	8.87703	9.13789	9.167283	0.2608595	0.2902527	0.2755561	up
A_23_P129209	IDH2	2.1772375	2.5353045	2.370119	0.358067	0.1928816	0.2754743	up
A_23_P16523	GDF15	7.0330696	7.366218	7.2506313	0.3331485	0.2175617	0.2753551	up
A_23_P130553	SHKBP1	2.4234571	2.7432895	2.6542091	0.3198323	0.230752	0.2752922	up
A_33_P3356245	ZNF416	-0.640353	-0.325798	-0.404344	0.3145547	0.2360086	0.2752817	up
A_32_P94087	RPL21P44	7.5176725	7.835603	7.750271	0.3179307	0.2325983	0.2752645	up
A_24_P941912	DTX3L	1.6706696	1.7171283	2.1747074	0.0464587	0.5040379	0.2752483	up
A_33_P3287119	FAM178B	0.8741298	1.1147194	1.1840348	0.2405896	0.3099051	0.2752473	up
A_33_P3213997	DHRS4L2	4.233796	4.4519114	4.5661154	0.2181153	0.3323193	0.2752173	up
A_21_P0011141	XLOC_l2_003705	-0.071325	0.261385	0.1461368	0.3327098	0.2174616	0.2750857	up
A_23_P88420	NEDD8	7.1593075	7.323625	7.544856	0.1643176	0.3855486	0.2749331	up
A_23_P127394	CRY2	3.884778	4.2968507	4.0225544	0.4120727	0.1377764	0.2749245	up
A_21_P0001790	LOC101927438	-0.482756	-0.075857	-0.339878	0.406899	0.1428781	0.2748885	up
A_21_P0010805	XLOC_l2_001483	-1.614697	-1.432704	-1.24695	0.1819925	0.3677468	0.2748697	up
A_33_P3298587	SBNO2	3.2060862	3.4648728	3.4969864	0.2587867	0.2909002	0.2748435	up
A_33_P3418394	ATG12	-1.70568	-1.45705	-1.404629	0.2486301	0.3010507	0.2748404	up
A_24_P185158	FAM134C	1.2228508	1.4897213	1.5056505	0.2668705	0.2827997	0.2748351	up

A_23_P78268	GLOD4	2.9674845	3.2267318	3.2578135	0.2592473	0.290329	0.2747881	up
A_33_P3309110	ALOX12-AS1	-0.388741	-0.041756	-0.186212	0.3469844	0.2025285	0.2747564	up
A_24_P6428	FAM217B	-2.475586	-2.26463	-2.137111	0.2109561	0.3384757	0.2747159	up
A_33_P3299525	KCTD21	1.0887794	1.4142814	1.3126612	0.3255019	0.2238817	0.2746918	up
A_24_P413920	FAM84A	-0.200066	-0.105022	0.2542601	0.0950437	0.4543257	0.2746847	up
A_33_P3268368	FZR1	1.4780946	1.770143	1.7352695	0.2920485	0.257175	0.2746117	up
A_23_P395566	FBXO31	1.5149179	1.6921153	1.8869085	0.1771975	0.3719907	0.2745941	up
A_23_P136870	MAGEA6	-1.517837	-1.322876	-1.163861	0.1949606	0.3539753	0.274468	up
A_24_P10657	SLC44A2	3.967286	4.163347	4.320134	0.1960607	0.3528481	0.2744544	up
A_24_P357518	RPL21	6.550544	6.835586	6.814295	0.2850423	0.263751	0.2743967	up
A_33_P3391429	ZNF672	1.8724012	2.1209998	2.1725788	0.2485986	0.3001776	0.2743881	up
A_33_P3230399	ZNF784	2.9374447	3.2304206	3.1931362	0.2929759	0.2556915	0.2743337	up
A_24_P398432	ARAP1	1.5273976	1.8552184	1.748146	0.3278208	0.2207484	0.2742846	up
A_22_P00006734	lnc-FSCN2-1	-1.92705	-1.612266	-1.693335	0.3147841	0.2337151	0.2742496	up
A_23_P88710	TMEM87A	5.817894	6.177174	6.007062	0.3592801	0.189168	0.274224	up
A_23_P329870	RHBDF2	4.5705633	4.666371	5.0231686	0.0958076	0.4526053	0.2742064	up
A_22_P00010999	lnc-NSG1.1-1	-2.923986	-2.758289	-2.54159	0.1656978	0.3823965	0.2740471	up
A_33_P3387272	EEF1A1	6.4668655	6.9319806	6.549818	0.4651151	0.0829525	0.2740338	up
A_33_P3231670	RNF208	2.1527805	2.5294318	2.324111	0.3766513	0.1713305	0.2739909	up
A_23_P300740	NPB	-3.089038	-2.972497	-2.657619	0.1165409	0.4314189	0.2739799	up
A_22_P00012194	CASC21	-0.981689	-0.620826	-0.794672	0.3608632	0.1870174	0.2739403	up
A_24_P484797	CIDECP	-0.812307	-0.442489	-0.634362	0.3698177	0.1779451	0.2738814	up
A_24_P330385	SLC22A17	-1.77533	-1.302609	-1.700342	0.4727206	0.0749879	0.2738543	up
A_21_P0011713	XLOC_l2_006821	-1.306008	-1.083162	-0.981253	0.2228465	0.3247557	0.2738011	up
A_23_P123563	RPS6	10.085664	10.413139	10.305782	0.3274756	0.2201185	0.273797	up
A_21_P0012280	VWF	-2.616377	-2.298846	-2.386511	0.3175314	0.2298658	0.2736986	up
A_23_P145388	MTCH1	7.1710863	7.4044623	7.4848995	0.233376	0.3138132	0.2735946	up
A_23_P376557	MMP25	-3.167944	-3.061733	-2.727107	0.1062114	0.4408369	0.2735242	up
A_33_P3398822	ZBED4	-2.796433	-2.443606	-2.602238	0.3528273	0.1941955	0.2735114	up
A_32_P204381	CIAPIN1	2.4231825	2.7853618	2.6079893	0.3621793	0.1848068	0.2734931	up
A_23_P67355	PRRG2	0.2132692	0.5214386	0.4519768	0.3081694	0.2387075	0.2734385	up
A_33_P3291776	NANS	6.162424	6.437226	6.4344473	0.2748017	0.2720232	0.2734125	up
A_24_P191067	CLSTN1	4.0512505	4.4406476	4.208618	0.3893971	0.1573677	0.2733824	up
A_22_P00000467	LOC100506136	-3.111855	-3.107869	-2.569129	0.0039861	0.5427268	0.2733564	up
A_21_P0000324	SNORA36A	-0.156908	-0.061008	0.2938705	0.0958996	0.4507785	0.273339	up
A_33_P3531204	C1QTNF9B-AS1	-0.015216	0.0791631	0.4366469	0.0943794	0.4518633	0.2731214	up
A_22_P00001958	BAZ2B	-1.234385	-0.998022	-0.924533	0.2363634	0.3098517	0.2731075	up
A_23_P64083	SF3B2	5.2144337	5.558724	5.4163246	0.3442903	0.201891	0.2730906	up
A_23_P141194	ICT1	7.067174	7.3480105	7.332467	0.2808366	0.2652931	0.2730649	up
A_33_P3281807	PDDC1	0.2755704	0.556643	0.5405211	0.2810726	0.2649508	0.2730117	up
A_24_P31235	EIF5A	4.618375	4.8332496	4.9494543	0.2148747	0.3310795	0.2729771	up
A_22_P00010255	lnc-MTERFD2-1	-1.870732	-1.510601	-1.684918	0.3601308	0.1858139	0.2729724	up
A_33_P3268466	MPDU1	0.597043	0.9676609	0.772347	0.3706179	0.1753039	0.2729609	up
A_21_P0011081	FAM86C1	1.5685749	1.9581819	1.7248278	0.389607	0.1562529	0.2729299	up
A_23_P79441	C2orf42	0.297267	0.5963402	0.5440111	0.2990732	0.2467442	0.2729087	up
A_33_P3236441	LDHAL6A	3.5707607	3.8615642	3.8257303	0.2908034	0.2549696	0.2728865	up
A_23_P400378	GPBAR1	-2.393893	-2.301196	-1.940819	0.0926969	0.4530745	0.2728857	up
A_33_P3388835	STXBP2	0.8800612	1.1797967	1.1260595	0.2997356	0.2459984	0.272867	up
A_21_P0009396	lnc-SEPT9-1	-3.292892	-2.850278	-3.189958	0.4426138	0.1029334	0.2727736	up
A_24_P262201	SULT1A4	4.2070646	4.483584	4.476077	0.2765193	0.2690125	0.2727659	up
A_22_P00020701	lnc-SLC22A12-2	2.4345322	2.7136006	2.7007742	0.2790685	0.266242	0.2726553	up
A_33_P6817603	IRF6	-0.744912	-0.569051	-0.375638	0.1758609	0.3692746	0.2725678	up
A_33_P3371425	SEPN1	-2.053847	-1.779975	-1.782632	0.2738724	0.2712154	0.2725439	up
A_21_P0012298	XLOC_l2_009539	0.0545688	0.1607895	0.4934211	0.1062207	0.4388523	0.2725365	up
A_33_P3394198	FPGS	1.0152683	1.3420949	1.2333469	0.3268266	0.2180786	0.2724526	up
A_33_P3333030	SUFU	-0.019349	0.2622976	0.2438984	0.2816463	0.263247	0.2724466	up

A_21_P0000239	SNORD101	-1.557556	-1.177452	-1.392795	0.3801041	0.1647611	0.2724326	up
A_33_P3263651	SEMA6B	-3.280942	-2.911041	-3.105979	0.3699012	0.1749625	0.2724319	up
A_32_P218989	YBX1	6.317481	6.5865493	6.593071	0.2690682	0.2755899	0.2723291	up
A_33_P3504659	CASP10	0.3348212	0.5644436	0.6497712	0.2296224	0.31495	0.2722862	up
A_23_P71094	GCC1	0.6478958	1.1020775	0.7381434	0.4541817	0.0902476	0.2722147	up
A_33_P3305399	SETDB1	-1.278725	-0.86876	-1.144519	0.4099655	0.1342058	0.2720857	up
A_33_P3404531	ZCWPW1	-1.140183	-0.771616	-0.964635	0.3685675	0.1755481	0.2720578	up
A_21_P0004231	lnc-FAM105B-3	-3.13111	-2.953344	-2.764808	0.1777661	0.3663023	0.2720342	up
A_21_P0000394	SNORD110	1.2350326	1.57058	1.4434485	0.3355475	0.208416	0.2719817	up
A_24_P165259	PYCR2	4.835946	5.1346374	5.081196	0.2986913	0.2452498	0.2719705	up
A_33_P3274199	TP53I13	4.7845774	5.091742	5.021246	0.3071647	0.2366686	0.2719166	up
A_23_P95879	RPL38	9.811439	10.121471	10.045076	0.3100328	0.2336378	0.2718353	up
A_33_P3267185	ATP2B1	-2.270423	-1.66494	-2.332377	0.6054826	-0.061954	0.2717642	up
A_23_P204277	H2AFJ	7.260502	7.561435	7.5028524	0.3009334	0.2423506	0.271642	up
A_23_P144369	NAP1L5	-0.539652	-0.526547	-0.009554	0.0131044	0.5300975	0.271601	up
A_23_P49924	NT5C3B	3.4997702	3.816382	3.7263374	0.3166118	0.2265673	0.2715895	up
A_23_P128372	FKBP4	4.3823204	4.6293635	4.678417	0.2470431	0.2960968	0.27157	up
A_32_P216426	HNRNPA1L2	4.961735	5.294559	5.171983	0.3328242	0.210248	0.2715361	up
A_32_P191262	ACR	-2.206934	-1.94843	-1.922519	0.2585034	0.2844148	0.2714591	up
A_21_P0010847	CTAGE7P	-2.908206	-2.480008	-2.793498	0.4281976	0.114708	0.2714528	up
A_33_P3334791	GLMP	-1.07375	-0.769159	-0.835697	0.3045907	0.2380529	0.2713218	up
A_24_P254177	SMIM7	2.8876457	3.2943702	3.0233717	0.4067245	0.135726	0.2712252	up
A_23_P49041	TMEM62	0.9327183	1.329843	1.0779972	0.3971248	0.1452789	0.2712019	up
A_23_P84782	THAP4	4.684536	4.917048	4.9944124	0.232512	0.3098764	0.2711942	up
A_21_P0012961	LOC255187	-2.293455	-2.277787	-1.766741	0.0156677	0.5267134	0.2711905	up
A_23_P321703	BCL2A1	3.8146229	4.190535	3.9809875	0.3759122	0.1663647	0.2711384	up
A_23_P10911	PLBD2	-1.442316	-1.375805	-0.966692	0.0665112	0.4756236	0.2710674	up
A_23_P157416	ZNF394	2.6212835	2.9246583	2.8598566	0.3033748	0.2385731	0.2709739	up
A_23_P153767	AKAP8L	3.5666971	3.7669883	3.9082127	0.2002912	0.3415155	0.2709034	up
A_33_P3237552	FIBCD1	-0.943662	-0.903307	-0.442254	0.0403547	0.5014081	0.2708814	up
A_22_P00014270	MFI2-AS1	0.2100301	0.4523053	0.5094762	0.2422752	0.2994461	0.2708607	up
A_23_P34983	JTB	6.589878	6.8707204	6.850564	0.2808423	0.2606859	0.2707641	up
A_23_P344392	OGG1	0.0282922	0.4616942	0.1363311	0.4334021	0.1080389	0.2707205	up
A_23_P122116	DDX41	3.1140156	3.3489442	3.4205236	0.2349286	0.3065081	0.2707183	up
A_33_P3242174	C2orf82	1.754344	1.7654152	2.2846766	0.0110712	0.5303326	0.2707019	up
A_22_P00010241	lnc-MTA3-3	-0.627623	-0.311445	-0.402476	0.3161779	0.2251468	0.2706623	up
A_32_P175739	HK2	3.7863255	3.8982415	4.215435	0.1119161	0.4291096	0.2705128	up
A_21_P0000583	LOC439994	3.0496483	3.3219047	3.3183956	0.2722564	0.2687473	0.2705019	up
A_33_P3216337	FTSJ1	5.2421675	5.6113696	5.4139214	0.3692021	0.1717539	0.270478	up
A_22_P00001558	UBL7-AS1	-1.083674	-0.85391	-0.772616	0.229763	0.3110576	0.2704103	up
A_32_P157945	DSP	5.638176	6.0041337	5.8130245	0.3659577	0.1748486	0.2704032	up
A_33_P3222932	SHISA8	-0.816966	-0.50845	-0.584682	0.3085165	0.2322841	0.2704003	up
A_23_P87603	TARBP2	2.253811	2.5279183	2.5204706	0.2741075	0.2666597	0.2703836	up
A_23_P153797	NCAN	-3.341187	-3.027181	-3.114484	0.3140054	0.2267027	0.270354	up
A_23_P87082	ROBO3	-2.258748	-2.090347	-1.886529	0.1684015	0.3722186	0.27031	up
A_23_P15299	WBP2	2.0516691	2.402225	2.2416801	0.3505559	0.190011	0.2702835	up
A_33_P3274319	TMEM52	0.6582389	1.0182295	0.8387337	0.3599906	0.1804948	0.2702427	up
A_24_P408736	GALNT5	1.7039232	2.015758	1.9325476	0.3118348	0.2286243	0.2702296	up
A_23_P25224	YBX3	5.1583967	5.4840884	5.3730774	0.3256917	0.2146807	0.2701862	up
A_23_P67271	PKN1	1.2285991	1.6281452	1.3693619	0.3995462	0.1407628	0.2701545	up
A_23_P215461	LIMK1	2.211565	2.2411647	2.7222567	0.0295997	0.5106916	0.2701457	up
A_33_P3394203	YIPF3	-1.623299	-1.545908	-1.160583	0.0773916	0.4627161	0.2700539	up
A_23_P354798	COQ10A	0.6040158	0.8878536	0.8602281	0.2838378	0.2562122	0.270025	up
A_23_P127446	DPF2	3.6696243	4.1082044	3.7709417	0.43858	0.1013174	0.2699487	up
A_22_P00025437	MAFG-AS1	-0.22565	0.088994	-0.000416	0.3146439	0.225234	0.2699389	up
A_23_P356565	RRP8	3.5928679	3.7198777	4.0057297	0.1270099	0.4128618	0.2699358	up

A_32_P47643	FAM110C	2.9785194	3.2636704	3.233224	0.285151	0.2547045	0.2699277	up
A_33_P3298043	ABCC6	-1.60441	-1.508306	-1.160719	0.0961046	0.4436908	0.2698977	up
A_33_P3494109	LOC146795	-1.962178	-1.832376	-1.552201	0.1298027	0.409977	0.2698898	up
A_23_P151614	PSME1	1.6015463	1.9308829	1.8119178	0.3293366	0.2103715	0.2698541	up
A_23_P66798	KRT19	11.095647	11.443783	11.287216	0.348136	0.1915693	0.2698526	up
A_33_P3386242	CNKSR1	2.4718323	2.4553895	3.0279293	-0.016443	0.556097	0.2698271	up
A_23_P115346	EIF2D	1.3215251	1.6670728	1.5155263	0.3455477	0.1940012	0.2697744	up
A_21_P0010632	XLOC_l2_001011	-3.251176	-2.66078	-3.302028	0.5903964	-0.050852	0.2697723	up
A_23_P3663	FAM195A	5.635195	5.8140407	5.9957743	0.1788459	0.3605795	0.2697127	up
A_24_P196851	TLN1	1.4302192	1.7948294	1.6050029	0.3646102	0.1747837	0.269697	up
A_22_P00021164	UBAC2-AS1	0.4947596	0.7320585	0.7967711	0.237299	0.3020115	0.2696552	up
A_33_P3371564	FAM86B3P	0.614553	1.033607	0.7346191	0.419054	0.1200662	0.2695601	up
A_21_P0013349	FLNC	-1.099413	-0.310396	-1.349339	0.7890172	-0.249925	0.269546	up
A_33_P3326713	FAM188B	-2.406063	-2.408169	-1.864867	-0.002106	0.5411959	0.2695448	up
A_33_P3377304	TCF7L2	-1.073543	-0.745108	-0.862964	0.3284345	0.2105784	0.2695065	up
A_22_P00019308	lnc-C17orf89-1	-1.721472	-1.352916	-1.551113	0.3685565	0.1703596	0.2694581	up
A_32_P137939	ACTB	9.863231	10.07781	10.187504	0.2145796	0.3242731	0.2694263	up
A_33_P3214705	C19orf60	3.4611273	3.6686711	3.7924185	0.2075439	0.3312912	0.2694175	up
A_24_P213783	RPL31	9.392008	9.710148	9.612553	0.31814	0.2205448	0.2693424	up
A_23_P500410	ATP6V1G2	-3.093787	-2.934176	-2.714747	0.1596112	0.3790405	0.2693259	up
A_24_P260134	NMNAT3	0.9020233	1.185895	1.156682	0.2838717	0.2546587	0.2692652	up
A_23_P147805	UPP1	2.7066278	3.823358	2.1283712	1.1167302	-0.578257	0.2692368	up
A_33_P3408762	LMNA	5.152277	5.471085	5.3719025	0.3188081	0.2196255	0.2692168	up
A_22_P00001214	lnc-ANKRD10-2	-1.140136	-0.744881	-0.997094	0.3952551	0.1430416	0.2691484	up
A_23_P112666	FOXE3	-1.910676	-1.597443	-1.685651	0.3132334	0.2250252	0.2691293	up
A_23_P65584	COQ6	1.0860443	1.5195651	1.1906924	0.4335208	0.1046481	0.2690845	up
A_23_P390097	TTC39B	-0.748616	-0.035997	-0.923183	0.7126184	-0.174567	0.2690256	up
A_23_P300826	C6orf136	0.9853282	1.2145505	1.2941265	0.2292223	0.3087983	0.2690103	up
A_33_P3214056	LINC00869	5.236368	5.494288	5.5164337	0.2579198	0.2800655	0.2689927	up
A_21_P0003454	lnc-CCKAR-1	-1.757274	-1.772822	-1.203832	-0.015548	0.5534425	0.2689471	up
A_23_P41872	B4GALT7	0.0672851	0.4154983	0.2569375	0.3482132	0.1896524	0.2689328	up
A_21_P0005960	lnc-PRAGMIN.1-3	-3.052738	-2.65143	-2.916295	0.4013076	0.1364424	0.268875	up
A_21_P0013248	XLOC_l2_013485	1.2177396	1.53059	1.4426031	0.3128505	0.2248635	0.268857	up
A_23_P7250	CDS1	1.9019556	2.2206006	2.1208973	0.318645	0.2189417	0.2687933	up
A_33_P3415913	IRGQ	-2.246873	-1.679692	-2.276469	0.5671816	-0.029596	0.268793	up
A_23_P141779	CXXC1	1.172173	1.5316434	1.3500915	0.3594704	0.1779184	0.2686944	up
A_33_P3343220	CPTP	4.7401667	4.970059	5.0476103	0.2298923	0.3074436	0.2686679	up
A_23_P145895	TP53TG1	3.649085	3.9240766	3.9114084	0.2749915	0.2623234	0.2686574	up
A_33_P3303772	SLC6A3	-1.781743	-1.4935	-1.532782	0.2882423	0.248961	0.2686017	up
A_24_P932016	HUWE1	1.4493499	1.7895007	1.6463938	0.3401508	0.1970439	0.2685974	up
A_22_P00004105	lnc-CLASP2-1	-2.507358	-2.142339	-2.33528	0.3650191	0.1720786	0.2685488	up
A_23_P66608	KAT2A	3.1405487	3.3250976	3.4929657	0.1845489	0.352417	0.2684829	up
A_32_P168247	COX6A1	9.166704	9.416046	9.454315	0.249342	0.287611	0.2684765	up
A_23_P49155	CDH3	4.5203753	4.5215116	5.0561676	0.0011363	0.5357924	0.2684643	up
A_23_P206759	IST1	0.9010892	1.2910733	1.047925	0.3899841	0.1468358	0.26841	up
A_22_P00015375	LINC01121	-0.899952	-0.459757	-0.803333	0.4401951	0.0966187	0.2684069	up
A_21_P0013655	XLOC_l2_015213	2.806902	2.9826055	3.1679564	0.1757035	0.3610544	0.268379	up
A_24_P227450	ACAD10	0.9384537	1.2880573	1.1256027	0.3496037	0.1871491	0.2683764	up
A_23_P342131	CYB561A3	-1.16225	-0.912838	-0.874943	0.2494125	0.2873073	0.2683599	up
A_33_P3393679	IGSF9B	-1.00096	-0.527377	-0.937858	0.4735827	0.0631022	0.2683425	up
A_23_P39814	CIR1	0.7736211	0.943666	1.1402507	0.1700449	0.3666296	0.2683373	up
A_23_P74716	DEDD	1.332726	1.526329	1.6757312	0.193603	0.3430052	0.2683041	up
A_23_P8640	GPER1	4.135828	4.0998864	4.7083607	-0.035942	0.5725327	0.2682955	up
A_33_P3364607	LOC100130051	-2.677614	-2.536236	-2.282592	0.1413777	0.3950214	0.2681996	up
A_33_P3293266	TMEM175	2.27326	2.631165	2.4516869	0.3579049	0.1784267	0.2681658	up
A_33_P3421163	TP53INP1	-2.553428	-2.221463	-2.349134	0.3319645	0.2042937	0.2681291	up

A_23_P90659	LAPTM4A	7.1233997	7.4832187	7.2997456	0.3598189	0.1763458	0.2680824	up
A_23_P66311	DNASE1	-1.379831	-1.161107	-1.062419	0.2187243	0.3174119	0.2680681	up
A_22_P00009281	LRRC26	7.1183233	7.5079856	7.264756	0.3896623	0.1464329	0.2680476	up
A_33_P3385993	TTLL9	-3.219757	-2.997347	-2.906189	0.2224097	0.3135679	0.2679888	up
A_21_P0006520	lnc-ATP2B3-1	1.5821323	1.6391435	2.0610428	0.0570111	0.4789105	0.2679608	up
A_23_P6869	TMEM115	-0.691627	-0.484721	-0.362725	0.2069054	0.3289018	0.2679036	up
A_22_P00022128	TM4SF1-AS1	-2.835572	-2.476484	-2.658954	0.3590882	0.1766183	0.2678533	up
A_23_P70688	LY86	-1.291102	-1.019466	-1.027078	0.2716351	0.2640233	0.2678292	up
A_22_P00017678	LOC101927885	-0.46626	-0.148636	-0.248252	0.3176241	0.2180085	0.2678163	up
A_33_P3258467	SPTBN1	1.8609219	2.2300076	2.0274296	0.3690858	0.1665077	0.2677968	up
A_22_P00024803	lnc-WWC2-1	10.831157	11.12879	11.068903	0.2976332	0.2377462	0.2676897	up
A_23_P214603	FLOT1	2.795888	3.217958	2.9091415	0.42207	0.1132536	0.2676618	up
A_23_P33759	DHRS3	4.469288	4.776214	4.697633	0.3069263	0.2283449	0.2676356	up
A_23_P105066	ILK	2.2117014	2.4303842	2.5278826	0.2186828	0.3161812	0.267432	up
A_22_P00010971	lnc-NRIP2-1	0.0969577	0.3754435	0.3532314	0.2784858	0.2562738	0.2673798	up
A_33_P3355831	ZNF213	-1.124549	-0.572633	-1.141768	0.5519166	-0.017219	0.267349	up
A_22_P00005658	RALY-AS1	-1.068597	-0.762425	-0.840117	0.3061714	0.2284803	0.2673259	up
A_23_P102988	TXNRD2	-0.791766	-0.277045	-0.771842	0.5147214	0.0199242	0.2673228	up
A_33_P3236272	BANP	-2.719443	-2.395517	-2.508952	0.3239262	0.2104907	0.2672085	up
A_22_P00016922	LINC00941	0.2835441	0.3801003	0.7213807	0.0965562	0.4378367	0.2671964	up
A_23_P90601	STEAP3	2.8377113	3.0157828	3.1939993	0.1780715	0.356288	0.2671797	up
A_33_P3231572	LOC100130456	1.1547947	1.4492979	1.39465	0.2945032	0.2398553	0.2671792	up
A_33_P3456233	LINC01573	-0.828288	-0.36904	-0.753447	0.4592481	0.074841	0.2670445	up
A_33_P3833256	LOC440028	-2.451219	-2.146281	-2.222271	0.3049378	0.2289481	0.266943	up
A_33_P3368039	SLC35B2	2.7007189	2.7799983	3.1552267	0.0792794	0.4545078	0.2668936	up
A_23_P405531	WIZ	4.359314	4.646665	4.6056185	0.2873511	0.2463045	0.2668278	up
A_23_P77779	RPL19	10.108098	10.429478	10.320322	0.3213797	0.212224	0.2668018	up
A_33_P3229083	HIST1H2BK	3.7002048	3.9150748	4.0188637	0.21487	0.3186588	0.2667644	up
A_23_P35820	CFL1	5.920869	6.089927	6.285286	0.1690583	0.3644171	0.2667377	up
A_32_P118258	RPL21	9.577371	9.869455	9.818731	0.2920847	0.2413607	0.2667227	up
A_33_P3363012	PRPF19	7.575691	7.7098594	7.974786	0.1341682	0.3990946	0.2666314	up
A_33_P3882624	POT1	1.9289923	2.1409717	2.2502527	0.2119794	0.3212605	0.2666199	up
A_32_P481377	KRTAP11-1	-1.901168	-1.601223	-1.66791	0.2999454	0.2332583	0.2666018	up
A_23_P46182	RPS8	10.161529	10.427759	10.428398	0.2662306	0.2668695	0.2665501	up
A_23_P92025	CIDEC	-1.086029	-0.733413	-0.905625	0.3526163	0.1804042	0.2665102	up
A_33_P3335902	ARMCX6	0.0044031	0.1011438	0.4405117	0.0967407	0.4361086	0.2664247	up
A_24_P810697	MXRA7	-1.14885	-0.699795	-1.065076	0.4490557	0.0837741	0.2664149	up
A_23_P61551	CD2BP2	0.2471113	0.503551	0.5234661	0.2564397	0.2763548	0.2663973	up
A_23_P323685	HIST1H4H	4.705038	4.9329085	5.0099373	0.2278705	0.3048992	0.2663848	up
A_32_P117313	TRIQK	0.1415772	0.3769364	0.4389219	0.2353592	0.2973447	0.2663519	up
A_23_P111054	HIST1H2BB	3.473547	3.6374211	3.8423376	0.1638742	0.3687906	0.2663324	up
A_24_P244162	PI4K2A	1.1004105	1.3903723	1.3430886	0.2899618	0.2426782	0.26632	up
A_24_P13715	FBXO22	-2.870468	-2.63272	-2.575582	0.2377484	0.2948861	0.2663172	up
A_33_P3320619	HDAC6	-2.289359	-2.11928	-1.926915	0.1700792	0.3624444	0.2662618	up
A_23_P119095	PPP1R13L	5.2192802	5.5360827	5.434924	0.3168025	0.2156439	0.2662232	up
A_33_P3306159	TNRC18	-2.104989	-1.737883	-1.93983	0.3671055	0.1651588	0.2661321	up
A_23_P45496	GDI1	2.9436045	3.0803342	3.3390589	0.1367297	0.3954544	0.2660921	up
A_23_P321466	PEX11G	-1.616726	-1.274673	-1.426683	0.3420529	0.1900435	0.2660482	up
A_23_P216655	TRIM14	2.036912	2.067389	2.538371	0.030477	0.5014591	0.2659681	up
A_33_P3277361	OTX1	-2.605405	-2.360849	-2.3181	0.2445562	0.2873046	0.2659304	up
A_33_P3268634	WIBG	0.5093064	0.7268477	0.8235831	0.2175412	0.3142767	0.265909	up
A_33_P3404954	SLC38A6	3.2004805	3.4135957	3.519042	0.2131152	0.3185616	0.2658384	up
A_23_P110167	MGST2	5.8046417	6.011063	6.129818	0.2064214	0.3251762	0.2657988	up
A_24_P149645	PUF60	5.9429293	6.0393615	6.3777466	0.0964322	0.4348173	0.2656248	up
A_22_P00002540	lnc-C17orf62-1	6.7481194	7.0341516	6.9932117	0.2860322	0.2450924	0.2655623	up
A_23_P331092	C9orf163	1.6714296	1.977407	1.8965597	0.3059773	0.2251301	0.2655537	up

A_21_P0013670	XLOC_l2_015295	-0.44424	-0.038498	-0.318925	0.4057412	0.1253147	0.265528	up
A_21_P0010112	lnc-FOXA2-3	-3.381658	-2.978982	-3.253326	0.4026759	0.1283326	0.2655042	up
A_23_P78248	KRT23	8.237886	8.533779	8.472806	0.2958927	0.2349196	0.2654061	up
A_23_P156739	UQCC2	4.0754404	4.231749	4.449828	0.1563087	0.3743877	0.2653482	up
A_33_P3402565	DSP	3.872612	4.157185	4.1187325	0.2845731	0.2461205	0.2653468	up
A_23_P88680	EMC7	6.207223	6.501902	6.4430637	0.2946792	0.2358408	0.26526	up
A_24_P389038	WDR46	-0.005384	0.1457834	0.3738189	0.1511674	0.3792028	0.2651851	up
A_23_P83045	VCP	7.2171154	7.5723634	7.3921976	0.355248	0.1750822	0.2651651	up
A_21_P0000812	TOB1-AS1	-1.717159	-1.301062	-1.602987	0.4160976	0.1141725	0.2651351	up
A_33_P3220818	SAFB	3.3241549	3.586183	3.59239	0.2620282	0.2682352	0.2651317	up
A_23_P163161	SDR39U1	2.5045042	2.8928285	2.6463642	0.3883243	0.14186	0.2650921	up
A_33_P3398809	MRRF	3.688078	4.0170045	3.8892756	0.3289266	0.2011976	0.2650621	up
A_24_P213643	TSPAN10	-0.483476	-0.129579	-0.307251	0.3538971	0.1762242	0.2650607	up
A_21_P0000301	SNORA5A	0.9508052	1.0930095	1.3384848	0.1422043	0.3876796	0.2649419	up
A_23_P393531	INPP4A	-0.269744	-0.039796	0.0301256	0.2299485	0.29987	0.2649093	up
A_23_P201342	DVL1	6.5897503	6.9094405	6.7998676	0.3196902	0.2101173	0.2649038	up
A_23_P30655	NFKBIE	2.905449	3.1601949	3.1804867	0.254746	0.2750378	0.2648919	up
A_23_P154801	TRPC4AP	1.4482484	1.8332753	1.5930047	0.3850269	0.1447563	0.2648916	up
A_33_P3386132	C2orf49	1.8192816	2.090281	2.0778112	0.2709994	0.2585297	0.2647645	up
A_33_P3328659	CELSR1	7.335043	7.738086	7.4614134	0.4030433	0.1263704	0.2647069	up
A_33_P3236177	ANG	-0.126437	0.2636919	0.0128045	0.3901286	0.1392412	0.2646849	up
A_23_P39755	B3GNT7	-3.118452	-2.790973	-2.916592	0.3274794	0.20186	0.2646697	up
A_23_P86133	RPA2	3.5043745	3.8198562	3.718215	0.3154817	0.2138405	0.2646611	up
A_33_P3232993	MARK2	2.1042871	2.523643	2.2142305	0.4193559	0.1099434	0.2646496	up
A_23_P203173	IL10RA	-2.150172	-2.000493	-1.770673	0.1496787	0.3794985	0.2645886	up
A_33_P3296205	TSC22D1	6.5486326	6.869264	6.757162	0.3206315	0.2085295	0.2645805	up
A_33_P3244753	DRP2	1.0634332	1.388475	1.2672787	0.3250418	0.2038455	0.2644436	up
A_33_P3271387	THAP3	-0.943044	-0.495694	-0.861558	0.4473496	0.0814858	0.2644177	up
A_23_P84344	SIGIRR	3.4651537	3.5727077	3.8863106	0.107554	0.4211569	0.2643554	up
A_24_P244442	BSCL2	1.1008215	1.2494555	1.4808865	0.148634	0.380065	0.2643495	up
A_23_P48109	NINJ2	1.1527152	1.5453944	1.2886076	0.3926792	0.1358924	0.2642858	up
A_23_P207020	WDR45B	6.0763283	6.3499885	6.3311033	0.2736602	0.2547751	0.2642176	up
A_33_P3383205	OK/SW-CL.58	-3.127045	-2.720805	-3.004882	0.4062393	0.1221631	0.2642012	up
A_23_P98248	TRPT1	2.7280579	3.087358	2.8971567	0.3593001	0.1690989	0.2641995	up
A_19_P00319099	lnc-RAD23B-2	-1.257917	-0.958065	-1.029507	0.2998529	0.2284107	0.2641318	up
A_21_P0011561	XLOC_l2_005933	-2.951467	-2.562792	-2.812119	0.3886755	0.1393483	0.2640119	up
A_33_P3219803	PTMA	9.573667	9.8848095	9.790363	0.3111429	0.2166967	0.2639198	up
A_24_P261929	IFI27L1	3.580203	3.8807635	3.8073711	0.3005605	0.2271681	0.2638643	up
A_24_P137997	ZNF34	-1.782065	-1.538009	-1.49843	0.2440562	0.2836356	0.2638459	up
A_21_P0011392	REREP3	-2.072463	-1.681623	-1.935624	0.3908401	0.1368389	0.2638395	up
A_24_P2648	PTPN14	1.5103841	2.007259	1.5411458	0.4968748	0.0307617	0.2638183	up
A_22_P00017038	LOC101927560	-0.673805	-0.292723	-0.527262	0.3810825	0.146543	0.2638128	up
A_33_P3368675	C6orf120	-0.442641	-0.13477	-0.222952	0.3078704	0.2196884	0.2637794	up
A_33_P3297921	MRPL37	4.712718	4.965345	4.987461	0.2526269	0.2747431	0.263685	up
A_33_P3329644	ALG1L2	2.6053934	2.73379	3.0041447	0.1283965	0.3987513	0.2635739	up
A_24_P29445	TMEM14B	4.889797	5.2031226	5.1034546	0.3133254	0.2136574	0.2634914	up
A_23_P39056	KLK7	3.761794	4.032084	4.0184145	0.2702899	0.2566204	0.2634552	up
A_22_P00017037	LOC101927587	-2.324882	-2.003654	-2.119262	0.3212276	0.2056198	0.2634237	up
A_23_P46852	OBFC1	2.9555626	3.373262	3.0646534	0.4176993	0.1090908	0.2633951	up
A_21_P0006626	RRP12	-2.002791	-1.716143	-1.762676	0.2866473	0.2401142	0.2633808	up
A_23_P168951	ZHX2	1.1428075	1.5627694	1.2495785	0.4199619	0.106771	0.2633665	up
A_23_P75889	PSMD13	3.5807877	3.765058	3.9231882	0.1842704	0.3424006	0.2633355	up
A_33_P3361027	TFIP11	4.7239475	4.9685254	5.0058823	0.2445779	0.2819347	0.2632563	up
A_23_P112159	AGO2	4.139099	4.4494033	4.355258	0.3103042	0.2161589	0.2632315	up
A_22_P00010504	LOC101929709	-2.116956	-1.449966	-2.25767	0.6669903	-0.140715	0.2631378	up
A_23_P23855	POLR3GL	3.6918335	3.9674878	3.9424314	0.2756543	0.250598	0.2631261	up

A_23_P151907	PCSK6	-1.317053	-0.914225	-1.193779	0.4028277	0.1232739	0.2630508	up
A_24_P366566	OR5H1	-2.13311	-1.760314	-1.979868	0.3727961	0.1532426	0.2630193	up
A_21_P0000305	PDCL3	3.5608282	3.750525	3.8970127	0.1896968	0.3361845	0.2629406	up
A_33_P3358163	NPAP1	-2.114392	-1.757366	-1.945586	0.3570261	0.1688066	0.2629163	up
A_24_P376294	HNRNPUL1	2.9457932	3.3462539	3.0711174	0.4004607	0.1253243	0.2628925	up
A_23_P77502	PKD1	1.0012555	1.5618043	0.9663138	0.5605488	-0.034942	0.2628036	up
A_33_P3215028	C6orf89	1.3436661	1.5411339	1.6716881	0.1974678	0.328022	0.2627449	up
A_22_P00012110	lnc-POFUT2-3	-3.079387	-2.749923	-2.883416	0.329464	0.1959708	0.2627174	up
A_33_P3285893	SCRN1	-1.823893	-1.371804	-1.750608	0.4520884	0.0732842	0.2626863	up
A_23_P414895	TTC17	-1.415654	-1.000276	-1.30567	0.4153776	0.1099834	0.2626805	up
A_23_P354027	KCTD11	1.9447556	2.1038232	2.3110018	0.1590676	0.3662462	0.2626569	up
A_33_P3395321	HN1	5.9017754	6.0740657	6.2547626	0.1722903	0.3529873	0.2626388	up
A_23_P204417	ITFG2	1.273623	1.4831247	1.589396	0.2095017	0.315773	0.2626374	up
A_23_P310582	ST7L	-0.602109	-0.493172	-0.185824	0.1089377	0.416285	0.2626114	up
A_23_P251893	BRAT1	5.2548523	5.481523	5.553364	0.2266707	0.2985115	0.2625911	up
A_23_P71170	TRPV6	-1.54331	-1.334319	-1.227199	0.2089906	0.3161106	0.2625506	up
A_33_P3257503	SVIL-AS1	0.8981643	1.2372041	1.0841808	0.3390398	0.1860166	0.2625282	up
A_21_P0011772	SIGLEC11	-1.288001	-1.230577	-0.820461	0.0574241	0.4675403	0.2624822	up
A_23_P28772	DBNDD2	4.7631636	4.937511	5.1135416	0.1743474	0.350378	0.2623627	up
A_22_P00008429	SGOL1-AS1	-1.555569	-1.163487	-1.423163	0.3920813	0.1324062	0.2622437	up
A_21_P0012056	LOC101929567	-0.34025	-0.223753	0.0677381	0.116497	0.4079881	0.2622426	up
A_32_P135348	TANC1	4.5884237	4.9667706	4.7345047	0.3783469	0.146081	0.2622139	up
A_23_P328600	SLC8B1	1.9235592	2.194366	2.1771755	0.2708068	0.2536163	0.2622116	up
A_24_P74932	PLP2	4.531085	5.0081115	4.5784655	0.4770265	0.0473804	0.2622035	up
A_23_P39185	RDH13	2.0621758	2.3647037	2.2838898	0.3025279	0.221714	0.262121	up
A_23_P315589	KIAA0930	2.0936193	2.2819886	2.4293547	0.1883693	0.3357353	0.2620523	up
A_33_P3320953	CTXN1	4.285331	4.4696183	4.6247625	0.1842876	0.3394318	0.2618597	up
A_19_P00316820	lnc-WRNIP1-2	-1.474981	-1.285695	-1.140566	0.1892862	0.334415	0.2618506	up
A_21_P0000106	MTRNR2L6	9.783221	10.131857	9.95813	0.3486357	0.1749086	0.2617722	up
A_33_P3415395	SPTLC3	-1.333675	-1.041033	-1.102826	0.2926416	0.2308493	0.2617455	up
A_23_P215132	WDR91	-0.209731	-0.03669	0.1406112	0.1730409	0.3503423	0.2616916	up
A_33_P3398922	MBD1	2.7408485	3.0953107	2.9097433	0.3544622	0.1688948	0.2616785	up
A_33_P3335920	SYNE1	0.957664	1.3267326	1.1119518	0.3690686	0.1542878	0.2616782	up
A_23_P79842	PIGT	5.988899	6.3548303	6.1462374	0.365931	0.1573381	0.2616346	up
A_33_P3258091	RNPEP	6.009634	6.2944903	6.247966	0.2848563	0.2383318	0.2615941	up
A_33_P3235322	SYTL1	-1.67726	-1.368625	-1.462878	0.3086352	0.2143822	0.2615087	up
A_33_P3209869	TCP11L1	-0.6097	-0.210674	-0.485802	0.3990259	0.123898	0.261462	up
A_22_P00014047	SAA3P	-1.502344	-1.233965	-1.247806	0.2683792	0.2545385	0.2614589	up
A_33_P3265290	RPL24	8.62885	8.882565	8.898005	0.2537146	0.2691546	0.2614346	up
A_21_P0000361	SNORA79	1.4276457	1.6934185	1.684732	0.2657728	0.2570863	0.2614296	up
A_33_P3247190	CC2D1B	0.1103005	0.4884605	0.2549887	0.37816	0.1446881	0.2614241	up
A_22_P00007205	MIR17HG	-2.275742	-2.052453	-1.976266	0.2232885	0.2994752	0.2613819	up
A_23_P92362	NDUFC1	7.847596	8.167694	8.050197	0.3200979	0.2026005	0.2613492	up
A_22_P00020803	lnc-CTTNBP2-1	-2.212542	-1.779594	-2.12289	0.4329481	0.0896521	0.2613001	up
A_23_P137073	ZMYM3	-0.476955	-0.156076	-0.275322	0.3208785	0.2016325	0.2612555	up
A_33_P3214432	ZC3HAV1L	3.7989807	4.0705047	4.0496826	0.271524	0.2507019	0.2611129	up
A_21_P0006274	lnc-APBA1-2	4.802368	5.0804896	5.046358	0.2781215	0.2439899	0.2610557	up
A_33_P3392087	IDUA	-0.070724	0.3173218	0.0633254	0.3880458	0.1340494	0.2610476	up
A_23_P107994	TMEM160	4.0797434	4.3082366	4.3733234	0.2284932	0.2935801	0.2610366	up
A_33_P3217649	C9orf116	0.3706856	0.7080345	0.5552993	0.3373489	0.1846137	0.2609813	up
A_24_P269619	DECR1	4.154336	4.4242024	4.406417	0.2698665	0.2520809	0.2609737	up
A_33_P3241051	ADAMTS17	-2.680274	-1.94511	-2.893498	0.7351639	-0.213224	0.2609701	up
A_21_P0013687	XLOC_l2_015410	-2.846418	-2.661179	-2.509795	0.1852391	0.3366237	0.2609314	up
A_23_P143274	NRSN2	-1.73651	-1.470568	-1.480646	0.2659416	0.2558641	0.2609029	up
A_32_P95739	TPI1	7.6142025	7.8630595	7.8871365	0.248857	0.272934	0.2608955	up
A_33_P3228300	RCL1	3.4436245	3.9106555	3.498332	0.467031	0.0547075	0.2608693	up

A_23_P162106	MRPL48	4.982321	5.226662	5.259653	0.2443414	0.2773323	0.2608368	up
A_33_P3314441	FBXL17	-0.134799	-0.113161	0.3652124	0.0216374	0.500011	0.2608242	up
A_22_P00002570	lnc-C17orf97-1	-2.278668	-2.010257	-2.025437	0.2684112	0.2532311	0.2608211	up
A_23_P153941	CAMKMT	-1.955613	-1.868634	-1.521027	0.0869789	0.4345865	0.2607827	up
A_23_P74449	HPDL	4.767701	5.038029	5.0189	0.270328	0.2511988	0.2607634	up
A_23_P9362	PSMB7	7.372222	7.586799	7.6790314	0.2145772	0.3068094	0.2606933	up
A_22_P00014556	LOC100130502	0.8308902	0.8166266	1.3664732	-0.014264	0.535583	0.2606597	up
A_23_P138025	GPN2	1.820878	2.0786986	2.0843334	0.2578206	0.2634554	0.260638	up
A_24_P246173	MYO9B	2.2846928	2.691525	2.3991146	0.4068322	0.1144218	0.260627	up
A_23_P408955	E2F2	3.6745462	3.6955724	4.174362	0.0210261	0.4998159	0.260421	up
A_33_P3245824	PQLC1	3.7791538	4.149734	3.929347	0.3705802	0.1501932	0.2603867	up
A_23_P257795	NDUFA2	6.4613113	6.6106124	6.832756	0.1493011	0.3714447	0.2603729	up
A_21_P0003630	lnc-RAP1GDS1-2	0.1335244	0.55897	0.2288065	0.4254456	0.0952821	0.2603638	up
A_23_P165722	EIF4E2	6.549266	6.6829476	6.9361696	0.1336818	0.3869038	0.2602928	up
A_24_P120109	DHX57	-2.994469	-2.820578	-2.647826	0.1738904	0.346643	0.2602667	up
A_22_P00017888	lnc-ZFAT-3	-0.795336	-0.43536	-0.6349	0.3599753	0.1604357	0.2602055	up
A_33_P3246068	LAMTOR2	5.4399414	5.705174	5.6950674	0.2652326	0.255126	0.2601793	up
A_33_P3285038	CERS5	2.6614084	3.060555	2.7826033	0.3991466	0.1211948	0.2601707	up
A_23_P100501	HMOX2	5.1771936	5.2336793	5.6409826	0.0564857	0.463789	0.2601373	up
A_23_P14543	ALKBH1	0.07582	0.4170647	0.2547622	0.3412447	0.1789422	0.2600935	up
A_23_P45917	CKS1B	7.1903954	7.493424	7.4075127	0.3030286	0.2171173	0.2600729	up
A_23_P210379	ARFGAP1	3.8874702	4.121205	4.1737595	0.2337346	0.2862892	0.2600119	up
A_33_P3382835	NYNRIN	-3.011067	-2.994795	-2.507351	0.0162718	0.503716	0.2599939	up
A_23_P133332	MRPS27	4.3276978	4.6372256	4.5378647	0.3095279	0.2101669	0.2598474	up
A_23_P142239	YIF1B	2.2952728	2.4858012	2.6244183	0.1905284	0.3291454	0.2598369	up
A_23_P155830	TBC1D14	2.1124563	2.3983207	2.3462362	0.2858644	0.2337799	0.2598221	up
A_33_P3418942	NUDT21	2.676591	2.867764	3.0050411	0.1911731	0.3284502	0.2598116	up
A_23_P60458	PPP2R4	3.5802174	3.7945895	3.885438	0.2143722	0.3052206	0.2597964	up
A_22_P00017166	LINC01397	-2.641978	-2.343205	-2.421412	0.2987735	0.2205663	0.2596699	up
A_23_P36408	VPS33A	2.6595335	3.103909	2.7343836	0.4443755	0.0748501	0.2596128	up
A_24_P181055	ST3GAL4	2.6320496	2.830996	2.952304	0.1989465	0.3202543	0.2596004	up
A_21_P0005378	lnc-ABCA13-1	-0.342347	0.0462089	-0.211774	0.3885555	0.1305728	0.2595642	up
A_19_P00318461	LOC101927137	-2.004388	-1.805561	-1.684134	0.1988268	0.3202539	0.2595403	up
A_33_P3238623	DNAJC27-AS1	-2.623612	-2.326675	-2.401503	0.296937	0.2221084	0.2595227	up
A_21_P0000363	SNORA25	0.8160849	1.1322408	1.0189381	0.3161559	0.2028532	0.2595046	up
A_33_P3298942	ATP10B	1.8424463	2.1027284	2.1009827	0.260282	0.2585363	0.2594092	up
A_24_P67946	NUDT4	1.291584	1.3446817	1.7572274	0.0530977	0.4656434	0.2593706	up
A_33_P3409886	VAMP2	0.4926601	0.7909365	0.7130933	0.2982764	0.2204332	0.2593548	up
A_24_P873414	PLEKHB2	5.2051067	5.525766	5.403015	0.3206592	0.1979084	0.2592838	up
A_21_P0012874	LINC00847	-1.605121	-1.072826	-1.618885	0.5322948	-0.013764	0.2592652	up
A_23_P259741	SATB1	1.4516263	1.8680968	1.5536427	0.4164705	0.1020165	0.2592435	up
A_21_P0000265	SNORA45A	-0.925553	-0.506173	-0.826466	0.4193802	0.0990868	0.2592335	up
A_23_P167997	HIST1H2BG	2.4711475	2.7648587	2.6958637	0.2937112	0.2247162	0.2592137	up
A_23_P121265	TWF2	2.15981	2.3557706	2.4822102	0.1959605	0.3224001	0.2591803	up
A_23_P153524	C19orf73	-0.127374	-0.03406	0.2976656	0.0933137	0.4250393	0.2591765	up
A_22_P00006192	MBL1P	-0.759828	-0.298583	-0.702755	0.4612451	0.0570731	0.2591591	up
A_23_P393713	IGHMBP2	-0.374521	-0.106078	-0.124666	0.2684431	0.249855	0.2591491	up
A_21_P0001613	lnc-NUAK2-1	-2.477917	-1.956603	-2.480973	0.5213139	-0.003056	0.2591289	up
A_33_P3211174	PIAS4	-0.905812	-0.469117	-0.824473	0.4366946	0.0813384	0.2590165	up
A_22_P00014756	LOC101060091	-3.062568	-2.708857	-2.898358	0.3537109	0.1642098	0.2589604	up
A_22_P00012847	lnc-RASAL1-1	-2.100416	-1.858632	-1.824387	0.2417836	0.2760286	0.2589061	up
A_23_P31602	BUD31	4.6528215	4.887836	4.935505	0.2350144	0.2826834	0.2588489	up
A_21_P0006522	lnc-ARSD-2	-2.672424	-2.077451	-2.749892	0.5949726	-0.077468	0.2587522	up
A_33_P3227793	CGREF1	2.371356	2.5561118	2.7040882	0.1847558	0.3327322	0.258744	up
A_33_P3301851	MAP3K11	5.6368237	5.800816	5.9902887	0.1639924	0.3534651	0.2587287	up
A_21_P0008865	lnc-KLHL25-9	-2.324463	-1.868702	-2.262867	0.4557605	0.0615959	0.2586782	up

A_22_P00023618	ATP13A5-AS1	-2.929531	-2.668001	-2.673791	0.2615309	0.2557404	0.2586356	up
A_23_P415006	RAB11FIP5	3.1850624	3.5125012	3.3748732	0.3274388	0.1898108	0.2586248	up
A_33_P3377391	LINC01413	-2.278831	-1.911068	-2.129635	0.3677626	0.1491957	0.2584791	up
A_23_P160004	UTY	-2.686644	-2.602526	-2.253834	0.0841186	0.4328101	0.2584643	up
A_23_P57856	BCL6	1.1761942	1.5553465	1.3138976	0.3791523	0.1377034	0.2584279	up
A_32_P129540	LOC728739	2.353488	2.6032023	2.6205997	0.2497144	0.2671118	0.2584131	up
A_32_P38467	SNHG8	7.355877	7.735624	7.492776	0.3797469	0.136899	0.258323	up
A_23_P206684	WWP2	1.3763428	1.6267481	1.6424909	0.2504053	0.2661481	0.2582767	up
A_21_P0001407	lnc-MFSD4-2	-2.139197	-1.883253	-1.878636	0.2559447	0.2605615	0.2582531	up
A_33_P3369039	PRPF31	5.2061405	5.395992	5.5327024	0.1898513	0.3265619	0.2582066	up
A_23_P80626	PRRT3	1.1494942	1.2269297	1.5884686	0.0774355	0.4389744	0.2582049	up
A_22_P00009291	LOC101928813	-0.988809	-0.780634	-0.680633	0.2081742	0.3081756	0.2581749	up
A_23_P33045	RPL26	8.705774	8.995794	8.932097	0.29002	0.2263231	0.2581716	up
A_21_P0010868	XLOC_l2_001945	-1.099988	-0.607095	-1.076621	0.4928923	0.0233669	0.2581296	up
A_33_P3235335	MTMR3	-0.561912	-0.359376	-0.248204	0.2025352	0.3137078	0.2581215	up
A_22_P00015264	SPANXA2-OT1	-0.342159	0.1437178	-0.31188	0.4858766	0.0302787	0.2580776	up
A_23_P393080	CALML3	1.4215102	1.81458	1.5445929	0.3930697	0.1230826	0.2580762	up
A_33_P3299285	FCRLB	-2.201362	-1.997029	-1.889569	0.2043324	0.3117929	0.2580626	up
A_23_P36888	PCED1B	-1.467313	-1.237777	-1.180724	0.2295356	0.2865887	0.2580621	up
A_33_P3423220	TPBGL	-2.68749	-2.502999	-2.355959	0.1844907	0.3315313	0.258011	up
A_24_P391431	TAF9B	0.8368363	1.1146026	1.0748029	0.2777662	0.2379665	0.2578664	up
A_24_P340066	ELF4	4.7306833	4.918076	5.059018	0.1873927	0.3283348	0.2578638	up
A_21_P0008266	lnc-TUBGCP3-6	-2.26225	-2.120481	-1.888415	0.1417687	0.3738346	0.2578017	up
A_33_P3220570	UBTD1	3.0168257	3.283194	3.2660074	0.2663684	0.2491818	0.2577751	up
A_33_P3264846	SAMD9L	-0.656642	-0.86583	0.0680771	-0.209188	0.7247195	0.257766	up
A_22_P00006971	lnc-GDE1-1	-2.687922	-2.405209	-2.455121	0.2827134	0.2328014	0.2577574	up
A_23_P309246	ZSCAN25	-0.185844	0.0188141	0.1249857	0.204658	0.3108296	0.2577438	up
A_33_P3358621	LOC100506036	-1.905449	-1.701106	-1.594418	0.2043438	0.3110313	0.2576876	up
A_23_P64343	TIMM10	4.967602	5.2141814	5.2363453	0.2465797	0.2687435	0.2576616	up
A_21_P0001773	LINC01119	-0.165588	-0.000971	0.1849847	0.1646166	0.3505726	0.2575946	up
A_23_P90533	POP4	4.8105497	5.0036073	5.1325817	0.1930575	0.322032	0.2575448	up
A_33_P3645079	HOXA-AS3	-2.642637	-2.275047	-2.49515	0.3675904	0.1474867	0.2575386	up
A_22_P00025333	MARCH8	-1.534579	-1.371183	-1.182991	0.1633959	0.3515878	0.2574918	up
A_23_P376096	TICAM1	-1.449802	-1.28216	-1.102537	0.1676421	0.3472657	0.2574539	up
A_33_P3331752	MUC3A	-1.618352	-1.48563	-1.236205	0.1327229	0.3821473	0.2574351	up
A_33_P3337161	ADCY4	-0.910909	-0.698118	-0.608855	0.212791	0.3020544	0.2574227	up
A_23_P111240	PHACTR2	2.0116806	2.424706	2.11345	0.4130254	0.1017695	0.2573974	up
A_21_P0000140	LEPROT	1.5071325	1.6600404	1.8690109	0.1529079	0.3618784	0.2573931	up
A_33_P3229067	HIST1H2BN	1.2047906	1.4101644	1.514143	0.2053738	0.3093524	0.2573631	up
A_33_P3248439	MVB12B	2.6965857	2.8323712	3.0754948	0.1357856	0.3789091	0.2573473	up
A_23_P1926	SDHAF2	3.553235	3.620833	4.0003166	0.0675979	0.4470816	0.2573397	up
A_32_P96036	MEX3A	0.6583219	0.8206096	1.0106936	0.1622877	0.3523717	0.2573297	up
A_24_P103025	PCBP1	5.969469	6.2591014	6.194495	0.2896323	0.2250261	0.2573292	up
A_33_P3308914	CIB2	2.3809795	2.55374	2.7227364	0.1727605	0.3417568	0.2572587	up
A_21_P0000375	SNORD19	-3.458165	-3.116521	-3.285513	0.3416436	0.1726522	0.2571479	up
A_23_P165078	ABHD17A	0.7081575	0.9248581	1.0057445	0.2167006	0.2975869	0.2571437	up
A_23_P259393	SFMBT1	0.9256916	1.0832	1.2824583	0.1575084	0.3567667	0.2571375	up
A_22_P00001124	lnc-ALX3-2	-1.475634	-0.999578	-1.437519	0.4760561	0.0381155	0.2570858	up
A_23_P104942	TMX2	4.5618086	4.703771	4.933893	0.1419625	0.3720846	0.2570236	up
A_23_P408830	CAMKK2	1.7994633	1.9559121	2.1569052	0.1564488	0.3574419	0.2569454	up
A_21_P0013524	OTUD6B-AS1	-2.891648	-2.351825	-2.917582	0.5398231	-0.025934	0.2569448	up
A_21_P0007929	lnc-RP11-90M5.1.1-2	-2.693322	-2.511737	-2.36106	0.1815851	0.332262	0.2569236	up
A_33_P3299590	SRRT	1.4039898	1.6990223	1.6227784	0.2950325	0.2187886	0.2569106	up
A_21_P0010092	lnc-NCOA3-9	-1.495112	-1.379713	-1.096893	0.1153994	0.3982186	0.256809	up
A_23_P30745	MEA1	4.899479	5.1566644	5.1558285	0.2571855	0.2563496	0.2567675	up

A_22_P00010998	STX18-AS1	-2.602362	-2.655566	-2.035753	-0.053204	0.5666096	0.2567029	up
A_33_P3249529	PCNX	3.2663326	3.565125	3.480873	0.2987924	0.2145405	0.2566664	up
A_24_P366122	ACBD4	-0.462082	-0.02462	-0.386238	0.4374628	0.0758448	0.2566538	up
A_33_P3310989	UBAP2L	0.5411577	0.6582527	0.9373684	0.117095	0.3962107	0.2566528	up
A_23_P152344	GTF3C1	-0.272693	0.1800761	-0.212416	0.4527693	0.060277	0.2565231	up
A_22_P00001725	LOC101930595	-2.808407	-2.50472	-2.599096	0.3036871	0.2093115	0.2564993	up
A_23_P117992	ATP2C2	1.2172661	1.7815189	1.1659017	0.5642529	-0.051364	0.2564442	up
A_24_P350228	SLC22A23	0.1618938	0.3117075	0.5247488	0.1498137	0.362855	0.2563343	up
A_33_P3394809	PCYT2	3.1211576	3.3763356	3.378622	0.255178	0.2574644	0.2563212	up
A_23_P20494	NDRG1	4.661359	5.31512	4.5201025	0.6537614	-0.141256	0.2562525	up
A_22_P00003781	CDK3	-0.482079	-0.169124	-0.282695	0.3129549	0.1993837	0.2561693	up
A_24_P102895	WDTC1	-0.139106	0.1607924	0.072989	0.2998982	0.2120948	0.2559965	up
A_33_P3827035	ANKRD18DP	-2.986318	-2.870561	-2.590124	0.1157565	0.396194	0.2559752	up
A_24_P53976	GLUL	2.496894	2.9816375	2.5240421	0.4847436	0.0271482	0.2559459	up
A_24_P93371	COMMD4	3.425661	3.6009517	3.7621803	0.1752906	0.3365192	0.2559049	up
A_33_P3294053	ANKRD39	3.493329	3.6093354	3.888958	0.1160064	0.3956289	0.2558177	up
A_33_P3417281	MUC4	0.7749867	1.0235505	1.0380344	0.2485638	0.2630477	0.2558057	up
A_33_P3389261	TMEM194B	-1.092542	-0.795095	-0.878548	0.2974463	0.2139936	0.2557199	up
A_24_P395814	CGB	-1.304179	-0.726577	-1.370598	0.5776019	-0.066419	0.2555914	up
A_21_P0013573	LOC101928195	-1.999564	-1.746485	-1.741577	0.2530789	0.257987	0.255533	up
A_33_P3242703	RAD52	-2.191035	-1.825059	-2.046025	0.3659759	0.1450105	0.2554932	up
A_23_P77401	CPPED1	0.3794856	0.7414742	0.5283604	0.3619885	0.1488748	0.2554317	up
A_33_P3415350	KCNB2	2.2762566	2.824945	2.2380972	0.5486884	-0.038159	0.2552645	up
A_33_P3229288	ACE	-0.322734	-0.023881	-0.111146	0.2988529	0.2115884	0.2552207	up
A_23_P74778	C1orf54	0.8757639	1.3017244	0.9602194	0.4259605	0.0844555	0.255208	up
A_23_P157038	CNPY4	-2.925108	-2.732253	-2.607705	0.1928549	0.3174036	0.2551292	up
A_23_P423197	RXRA	5.2833567	5.696194	5.3806877	0.4128375	0.0973311	0.2550843	up
A_33_P3253249	FTH1	9.763101	10.195356	9.841011	0.4322557	0.0779104	0.2550831	up
A_33_P3222210	ABHD17A	1.0284677	1.2775555	1.2894835	0.2490878	0.2610159	0.2550519	up
A_23_P62605	RPL11	8.920197	9.257517	9.092969	0.3373203	0.1727724	0.2550464	up
A_19_P00320229	FLJ46906	-1.650772	-1.28445	-1.507081	0.366322	0.1436915	0.2550068	up
A_22_P00020040	lnc-SPAG1-4	-0.733192	-0.448098	-0.508363	0.2850943	0.2248287	0.2549615	up
A_23_P83436	PEPD	6.1655426	6.5281186	6.3128347	0.362576	0.1472921	0.2549341	up
A_22_P00014003	lnc-RUNX2-2	-2.083433	-1.945781	-1.71136	0.1376514	0.3720732	0.2548623	up
A_21_P0013910	PTMA	10.188121	10.455196	10.4305	0.2670755	0.2423792	0.2547274	up
A_21_P0014268	LOC100506082	-3.31721	-2.956268	-3.168752	0.3609424	0.1484578	0.2547001	up
A_33_P3382493	SIGLEC14	0.3223801	0.5369644	0.6171207	0.2145844	0.2947407	0.2546625	up
A_33_P3337242	DTNBP1	1.5587149	1.8048754	1.8218455	0.2461605	0.2631307	0.2546456	up
A_23_P321388	RNF19B	-0.730565	-0.353777	-0.59808	0.3767881	0.1324854	0.2546368	up
A_23_P324490	KIAA0355	3.0857735	3.390161	3.2905912	0.3043876	0.2048178	0.2546027	up
A_22_P00022911	lnc-GATAD1-2	-2.673651	-2.134831	-2.703325	0.5388205	-0.029674	0.2545735	up
A_24_P131173	C1orf115	-1.950615	-1.799932	-1.592277	0.1506825	0.3583374	0.2545099	up
A_33_P3349927	WDR60	-3.401789	-2.870114	-3.424592	0.5316746	-0.022804	0.2544355	up
A_23_P983	PRDX6	7.466709	7.776261	7.6656685	0.3095517	0.1989594	0.2542555	up
A_22_P00016064	LARP6	-1.593737	-1.316045	-1.362929	0.2776914	0.2308078	0.2542496	up
A_23_P6802	RRP9	2.8974304	3.0877004	3.2156277	0.19027	0.3181973	0.2542336	up
A_33_P3587376	SNAR-A3	11.108892	11.447323	11.278885	0.3384314	0.1699934	0.2542124	up
A_21_P0013709	LINC01503	-2.247627	-1.977074	-2.009924	0.2705526	0.2377024	0.2541275	up
A_22_P00014211	VIPR1-AS1	-0.774106	-0.605005	-0.43514	0.1691012	0.3389664	0.2540338	up
A_24_P343559	NTRK2	-3.296433	-2.858249	-3.226622	0.4381833	0.0698102	0.2539967	up
A_23_P72387	AFAP1	-3.138917	-2.705602	-3.064294	0.4333153	0.0746229	0.2539691	up
A_24_P142118	THBS1	1.4009595	1.7867861	1.5229521	0.3858266	0.1219926	0.2539096	up
A_23_P206280	GPR56	8.20488	8.331467	8.585999	0.1265869	0.3811188	0.2538528	up
A_23_P116614	ME3	3.2990046	3.6263366	3.4793215	0.327332	0.1803169	0.2538245	up
A_22_P00020569	SDCBP2-AS1	1.3931184	1.6541328	1.6395397	0.2610145	0.2464213	0.2537179	up
A_23_P79836	SERINC3	3.6287146	3.8414197	3.9233885	0.2127051	0.2946739	0.2536895	up

A_23_P417974	AQP11	-2.396862	-1.504635	-2.781779	0.8922262	-0.384917	0.2536545	up
A_22_P00007687	LINC00240	-1.008809	-0.642427	-0.868067	0.3663812	0.1407414	0.2535613	up
A_23_P163258	PARP6	2.835619	3.0870957	3.0911303	0.2514768	0.2555113	0.253494	up
A_23_P145817	MALSU1	4.739231	4.983537	5.00189	0.2443061	0.2626591	0.2534826	up
A_33_P3327097	MTMR1	-2.863334	-2.20332	-3.016481	0.6600146	-0.153147	0.2534338	up
A_21_P0014825	ZNF341-AS1	1.5688086	1.7751951	1.869215	0.2063866	0.3004065	0.2533965	up
A_23_P36513	PRKAG1	0.8366785	0.9967237	1.1832643	0.1600452	0.3465858	0.2533155	up
A_23_P209904	GPC1	6.033781	6.3271556	6.2469816	0.2933745	0.2132006	0.2532876	up
A_24_P203328	TPRXL	3.306038	3.6224246	3.4962091	0.3163867	0.1901712	0.253279	up
A_21_P0000130	GS1-259H13.2	-1.084961	-0.477607	-1.185805	0.6073546	-0.100843	0.2532556	up
A_22_P00016662	lnc-TPCN2-3	-2.503431	-2.14995	-2.350586	0.3534815	0.1528456	0.2531636	up
A_23_P151280	COX14	7.018817	7.153684	7.3902645	0.1348672	0.3714476	0.2531574	up
A_33_P3219055	C15orf61	3.2803316	3.5558867	3.5109892	0.2755551	0.2306576	0.2531064	up
A_23_P319565	PGBD3	-0.547385	-0.212706	-0.375908	0.3346791	0.1714768	0.253078	up
A_33_P3340154	DGCR14	1.4327211	1.6986103	1.6729765	0.2658892	0.2402554	0.2530723	up
A_33_P3216532	EPHB4	1.7035456	2.0644832	1.8486156	0.3609376	0.1450701	0.2530038	up
A_32_P52911	ADI1	2.6906414	3.0043159	2.8827982	0.3136745	0.1921568	0.2529156	up
A_33_P3394234	ZNF133	1.9176579	2.3374457	2.003622	0.4197879	0.0859642	0.252876	up
A_23_P19590	EZR	7.1181097	7.3925824	7.3493357	0.2744727	0.231226	0.2528493	up
A_33_P3277611	TMEM8C	-1.176363	-0.782339	-1.064793	0.3940239	0.1115704	0.2527971	up
A_23_P143987	ATG7	1.2778144	1.5329056	1.5282745	0.2550912	0.2504602	0.2527757	up
A_33_P3286923	TMPRSS5	-1.85179	-1.492759	-1.70529	0.3590307	0.1465001	0.2527654	up
A_23_P157316	C7orf34	-3.371646	-3.047177	-3.190593	0.3244689	0.1810527	0.2527608	up
A_23_P111978	KCNK9	-2.949597	-2.498002	-2.895713	0.4515953	0.0538838	0.2527395	up
A_23_P87709	PLBD1	5.634506	5.862164	5.912262	0.2276578	0.2777557	0.2527068	up
A_23_P93543	PEX7	2.3156357	2.6977744	2.438902	0.3821387	0.1232662	0.2527025	up
A_23_P28279	ACTR1B	2.5699053	2.8579555	2.787219	0.2880502	0.2173138	0.252682	up
A_22_P00013711	lnc-RP11-791J7.2.1-1	-1.158282	-1.114053	-0.697245	0.0442286	0.4610372	0.2526329	up
A_33_P3258191	C16orf91	5.0392437	5.2293754	5.3543186	0.1901317	0.3150749	0.2526033	up
A_33_P3380587	AGO1	-0.200077	0.0116467	0.0933461	0.2117233	0.2934227	0.252573	up
A_22_P00018730	lnc-C3orf71-1	-3.161918	-2.522214	-3.296652	0.6397038	-0.134734	0.2524849	up
A_22_P00017735	lnc-XXbac-B461K10.4.1-2	-1.90312	-1.722368	-1.57896	0.1807518	0.3241592	0.2524555	up
A_21_P0009116	lnc-BANP-1	-0.659327	-0.534584	-0.27928	0.124743	0.3800473	0.2523952	up
A_21_P0013615	XLOC_l2_015098	-1.449	-1.144193	-1.249084	0.3048077	0.1999168	0.2523623	up
A_33_P3222783	SURF4	1.952074	2.173365	2.2354107	0.2212911	0.2833366	0.2523139	up
A_24_P109214	APOC1	-0.019537	0.1137247	0.3517132	0.1332622	0.3712506	0.2522564	up
A_23_P88963	ALDOA	7.3429537	7.631808	7.5584993	0.2888541	0.2155457	0.2521999	up
A_22_P00014120	lnc-SCAP-1	-1.984357	-1.708925	-1.755398	0.2754321	0.2289591	0.2521956	up
A_23_P429383	HOXD9	-3.17875	-2.971121	-2.882001	0.207629	0.2967489	0.2521889	up
A_23_P150147	SSH3	-0.199815	0.1020722	0.0025687	0.3018875	0.202384	0.2521358	up
A_24_P144303	XRCC6P5	-3.125086	-3.082594	-2.663342	0.0424926	0.4617446	0.2521186	up
A_22_P00002305	MIR210	-3.473784	-3.261108	-3.182246	0.2126765	0.2915382	0.2521074	up
A_23_P103476	UBIAD1	1.8245716	1.8973064	2.2559843	0.0727348	0.4314127	0.2520738	up
A_33_P3389942	NHP2	7.996994	8.227982	8.27008	0.2309876	0.2730856	0.2520366	up
A_33_P3412016	SEMA4B	-1.616228	-1.57591	-1.152509	0.040318	0.4637194	0.2520187	up
A_21_P0005320	lnc-EPHB4-1	-0.078002	0.2563877	0.0915542	0.3343902	0.1695566	0.2519734	up
A_23_P68628	NECAB3	0.9613295	1.2965641	1.1299248	0.3352346	0.1685953	0.251915	up
A_33_P3252286	CRLF1	-1.019076	-0.969646	-0.564753	0.0494294	0.4543228	0.2518761	up
A_19_P00805076	LOC102724030	-1.757679	-1.552142	-1.459885	0.2055368	0.2977934	0.2516651	up
A_33_P3290707	MME	-1.801641	-1.344439	-1.755558	0.4572015	0.046083	0.2516422	up
A_23_P152678	B9D1	1.0114441	1.274394	1.2513652	0.2629499	0.2399211	0.2514355	up
A_23_P117778	UBL7	1.33532	1.3416958	1.8316298	0.0063758	0.4963098	0.2513428	up
A_21_P0000197	HIST1H2AG	3.239973	3.4020524	3.580574	0.1620793	0.340601	0.2513402	up
A_22_P00016948	KANTR	-2.135997	-1.907108	-1.862297	0.2288895	0.2737007	0.2512951	up

A_23_P385081	SCAMP2	0.750557	1.0506921	0.9528894	0.3001351	0.2023325	0.2512338	up
A_23_P421175	FAM83H	4.2802935	4.6235757	4.439479	0.3432822	0.1591854	0.2512338	up
A_33_P3351982	COA5	3.274809	3.4947991	3.557229	0.2199903	0.2824202	0.2512052	up
A_23_P54953	SAP30BP	1.7762489	2.053392	2.001503	0.277143	0.2252541	0.2511985	up
A_33_P3210909	SEC13	3.5182438	3.7744632	3.76439	0.2562194	0.2461462	0.2511828	up
A_32_P412313	ASB6	0.4066982	0.5808806	0.7348609	0.1741824	0.3281627	0.2511725	up
A_24_P376322	MBD1	-2.728129	-2.149563	-2.804413	0.5785654	-0.076284	0.2511406	up
A_33_P3302245	TMEM59L	-1.648062	-1.430802	-1.363127	0.2172599	0.284935	0.2510974	up
A_32_P137035	TRIM27	3.51546	3.8570142	3.6759777	0.3415542	0.1605177	0.2510359	up
A_33_P3350393	CES2	-0.023215	0.1807857	0.274435	0.2040005	0.2976499	0.2508252	up
A_24_P409985	TMEM44	-1.377334	-1.097338	-1.155795	0.2799959	0.221539	0.2507675	up
A_24_P412976	TMEM143	2.591425	3.1245131	2.559864	0.5330882	-0.031561	0.2507637	up
A_33_P3244122	HAAO	-1.258835	-1.071342	-0.944888	0.1874929	0.3139472	0.25072	up
A_23_P321034	INADL	0.4701786	1.1497726	0.2919579	0.679594	-0.178221	0.2506866	up
A_23_P30204	HMHB1	1.2248912	1.555831	1.3952837	0.3309398	0.1703925	0.2506661	up
A_23_P53015	TUT1	0.4924536	0.7213044	0.7647123	0.2288508	0.2722588	0.2505548	up
A_21_P0001752	lnc-FAIM3-1	-0.813053	-0.409509	-0.715496	0.4035444	0.0975575	0.250551	up
A_23_P131816	DNTTIP1	4.0854797	4.361917	4.3100224	0.2764373	0.2245426	0.25049	up
A_32_P114215	COMMD6	6.1166563	6.272775	6.461378	0.1561189	0.3447218	0.2504203	up
A_23_P3979	MRPS7	4.5515957	4.8367276	4.76715	0.2851319	0.2155542	0.2503431	up
A_23_P315991	OR10A5	-1.512909	-1.396242	-1.128926	0.1166673	0.3839827	0.250325	up
A_23_P158725	SLC16A3	6.8551455	7.0897765	7.1211157	0.2346311	0.2659702	0.2503006	up
A_23_P83266	ENDOG	2.969614	3.1384282	3.3012257	0.1688142	0.3316116	0.2502129	up
A_33_P3323048	SLC10A7	-2.077541	-1.815885	-1.838842	0.2616563	0.238699	0.2501776	up
A_23_P63067	DAP3	6.629469	6.8600383	6.899249	0.2305694	0.2697802	0.2501748	up
A_33_P3416622	LINC00322	-0.048443	0.3102717	0.0931444	0.3587151	0.1415877	0.2501514	up
A_32_P342064	FTH1	8.794835	9.1311	8.958817	0.3362646	0.1639814	0.250123	up
A_24_P291978	ADCK2	0.066752	0.2805553	0.3530607	0.2138033	0.2863088	0.250056	up
A_22_P00008240	LOC102724190	-0.823865	-0.48226	-0.665475	0.3416052	0.1583901	0.2499976	up
A_23_P36647	TMUB2	2.0559702	2.4073634	2.2043772	0.3513932	0.148407	0.2499001	up
A_21_P0001481	lnc-C1orf201-3	-2.219322	-1.994704	-1.944155	0.224618	0.275167	0.2498925	up
A_21_P0011925	lnc-TEX261-2	-2.913447	-2.681234	-2.645927	0.2322135	0.2675204	0.249867	up
A_33_P3233010	KIR3DL2	1.5843372	1.9138808	1.754519	0.3295436	0.1701818	0.2498627	up
A_22_P00015305	SPINK7	-2.720992	-2.533013	-2.409429	0.187979	0.3115623	0.2497706	up
A_23_P433079	DNAJC14	2.7479315	3.0313478	2.9640303	0.2834163	0.2160988	0.2497575	up
A_23_P256047	ANKEF1	-0.123512	0.2015052	0.0508261	0.325017	0.1743379	0.2496774	up
A_33_P3281018	LINC00689	-0.912402	-0.687266	-0.638202	0.2251358	0.2741995	0.2496677	up
A_23_P134935	DUSP4	0.7901521	1.212924	0.8666811	0.4227719	0.076529	0.2496505	up
A_33_P3361348	ALOX15	-2.832724	-2.418791	-2.747378	0.4139326	0.085346	0.2496393	up
A_19_P00809076	KANSL1-AS1	-0.436918	-0.07791	-0.296664	0.3590078	0.1402535	0.2496307	up
A_24_P20873	HIST1H4I	4.0842085	4.314283	4.3533316	0.2300744	0.2691231	0.2495987	up
A_33_P3303212	CCDC74A	-2.683425	-2.795043	-2.072626	-0.111619	0.6107984	0.2495899	up
A_23_P368896	SNX12	0.1712346	0.5034657	0.3379221	0.332231	0.1666875	0.2494593	up
A_33_P3393245	CMTR1	-1.136169	-0.89236	-0.88119	0.2438097	0.2549796	0.2493947	up
A_23_P36226	SLC25A33	2.3842382	2.5889049	2.678217	0.2046666	0.2939787	0.2493227	up
A_33_P3372074	LLGL2	6.918474	6.9643416	7.371213	0.0458674	0.4527388	0.2493031	up
A_21_P0008862	LOC101929560	-2.390953	-2.165567	-2.117786	0.2253861	0.2731667	0.2492764	up
A_23_P88234	EMC9	3.4765167	3.8202662	3.6311102	0.3437495	0.1545935	0.2491715	up
A_23_P427760	PIWIL4	0.4077473	0.9279289	0.3858476	0.5201817	-0.0219	0.249141	up
A_22_P00010716	LOC102724596	-2.597163	-2.218248	-2.477917	0.3789148	0.119246	0.2490804	up
A_21_P0011326	XLOC_l2_004706	0.0584369	0.3479114	0.2670574	0.2894745	0.2086206	0.2490475	up
A_23_P354547	IRF9	-2.250883	-1.846697	-2.15701	0.4041863	0.0938735	0.2490299	up
A_33_P3297923	TPRN	5.555031	5.7321343	5.8758707	0.1771035	0.3208399	0.2489717	up
A_24_P214858	TREML2	-1.38611	-1.302808	-0.971528	0.0833015	0.4145818	0.2489417	up
A_33_P3210168	PIN1	5.5313387	5.75077	5.8097563	0.2194314	0.2784176	0.2489245	up
A_23_P346673	GPRC5C	-1.955533	-1.869217	-1.544139	0.0863156	0.4113936	0.2488546	up

A_23_P35591	EXOSC1	4.3345814	4.697272	4.4695234	0.3626905	0.1349421	0.2488163	up
A_24_P130936	DDX3Y	0.0985775	0.3079968	0.3866663	0.2094193	0.2880888	0.248754	up
A_21_P0008523	LOC101929080	-3.022483	-2.744352	-2.803135	0.2781313	0.2193477	0.2487395	up
A_33_P3276693	PGF	-0.159147	-0.243105	0.4221048	-0.083958	0.5812521	0.2486472	up
A_33_P3332865	NPEPL1	1.692728	1.8871465	1.9954796	0.1944184	0.3027515	0.248585	up
A_23_P122228	NDUFS6	6.9207907	7.1581874	7.1804028	0.2373967	0.2596121	0.2485044	up
A_24_P217848	HIST1H2AK	6.7060328	6.942577	6.966444	0.2365441	0.2604113	0.2484777	up
A_23_P17826	SLC5A1	-0.05257	0.0288005	0.3629241	0.0813704	0.415494	0.2484322	up
A_33_P3379669	RNF208	0.0697231	0.4695721	0.1667166	0.3998489	0.0969935	0.2484212	up
A_22_P00017233	ENTPD3-AS1	-1.327218	-1.043112	-1.114811	0.2841053	0.2124066	0.248256	up
A_22_P00004022	LOC100506497	-1.58939	-1.391367	-1.290909	0.1980228	0.298481	0.2482519	up
A_33_P3235925	PRKRIP1	3.285555	3.6652236	3.4023113	0.3796687	0.1167564	0.2482126	up
A_33_P3413810	LOC101928038	-3.004826	-2.601857	-2.911551	0.4029689	0.0932751	0.248122	up
A_24_P228667	MRPL40	3.5785265	3.7900243	3.8632631	0.2114978	0.2847366	0.2481172	up
A_33_P3314974	PARD6G-AS1	-0.673371	-0.264102	-0.586568	0.4092693	0.086803	0.2480361	up
A_23_P338168	FBXL19	1.6581573	1.892745	1.9195881	0.2345877	0.2614307	0.2480092	up
A_33_P3318796	FSTL3	2.803978	3.206328	2.897646	0.40235	0.093668	0.248009	up
A_22_P00006296	lnc-FAM83H-1	-1.056077	-0.860081	-0.756134	0.1959953	0.299943	0.2479692	up
A_23_P11705	BSDC1	3.6580982	3.982656	3.8294754	0.3245578	0.1713772	0.2479675	up
A_22_P00015730	SZT2	0.8566852	1.229929	0.9793758	0.3732438	0.1226907	0.2479672	up
A_33_P3397180	LOC100129324	-1.761247	-1.670129	-1.356456	0.0911183	0.4047909	0.2479546	up
A_23_P21495	FCGBP	-1.649182	-1.466711	-1.335765	0.1824718	0.3134174	0.2479446	up
A_21_P0005442	lnc-LRRC61-2	-3.029948	-2.679771	-2.884253	0.3501768	0.1456955	0.2479361	up
A_23_P106433	RCCD1	3.992116	4.339208	4.140891	0.3470922	0.1487751	0.2479336	up
A_23_P214658	PBX2	-1.280972	-1.07526	-0.990858	0.2057123	0.2901144	0.2479134	up
A_23_P395911	FBXO17	0.5982227	0.8951469	0.797122	0.2969241	0.1988993	0.2479117	up
A_33_P3235217	CTPS1	4.298725	4.5230956	4.570118	0.2243705	0.2713928	0.2478817	up
A_32_P94722	BTBD9	1.9016628	1.9930453	2.3060436	0.0913825	0.4043808	0.2478817	up
A_23_P393607	SNAP47	5.086796	5.307055	5.36224	0.2202592	0.275444	0.2478516	up
A_33_P3258600	NYNRIN	-3.090861	-3.127065	-2.558985	-0.036205	0.5318761	0.2478358	up
A_23_P56590	C1D	1.2086091	1.4344177	1.4784527	0.2258086	0.2698436	0.2478261	up
A_23_P96853	FAF1	4.2020235	4.492951	4.4066906	0.2909274	0.2046671	0.2477972	up
A_21_P0014711	EIF4E2	0.6218615	1.0521197	0.6871948	0.4302583	0.0653334	0.2477958	up
A_33_P3256282	KRTAP10-2	-1.889352	-1.424244	-1.859248	0.4651074	0.0301037	0.2476056	up
A_22_P00003759	LOC102725300	-0.571478	-0.333601	-0.31417	0.2378764	0.257308	0.2475922	up
A_24_P48898	APOL2	2.8983278	3.1928678	3.098853	0.2945399	0.2005253	0.2475326	up
A_23_P154840	SOD1	8.217201	8.441238	8.488227	0.2240372	0.2710257	0.2475314	up
A_23_P96777	LRRC47	3.1922388	3.3545308	3.524953	0.162292	0.3327141	0.247503	up
A_24_P944444	MAPKBP1	0.0653081	0.3633747	0.2622323	0.2980666	0.1969242	0.2474954	up
A_33_P3361257	NOP16	5.0111923	5.081428	5.435939	0.0702357	0.4247465	0.2474911	up
A_23_P83159	KLHL9	4.082061	4.1999555	4.459071	0.1178947	0.3770104	0.2474525	up
A_23_P42909	TMEM139	2.0225306	2.477654	2.0622911	0.4551234	0.0397606	0.247442	up
A_33_P3281273	S1PR4	-2.719229	-2.201255	-2.742355	0.5179746	-0.023126	0.2474242	up
A_33_P3243128	COQ5	2.7339287	3.1162028	2.8465023	0.3822742	0.1125736	0.2474239	up
A_33_P3392892	ULK4	-0.829241	-0.362681	-0.801056	0.4665599	0.0281849	0.2473724	up
A_33_P3228739	LRRC3C	-1.366136	-1.064548	-1.173187	0.3015881	0.1929493	0.2472687	up
A_23_P101516	KCNC3	-1.666927	-1.441766	-1.397843	0.2251616	0.2690845	0.247123	up
A_23_P426989	SH3BP5L	3.3273582	3.44865	3.700204	0.1212916	0.3728457	0.2470686	up
A_23_P61268	HGH1	3.341591	3.398542	3.7787695	0.056951	0.4371786	0.2470648	up
A_32_P73452	ANO8	-1.164892	-0.736039	-1.099693	0.428853	0.0651989	0.247026	up
A_33_P3359306	MTA1	3.8989916	4.3132844	3.9787111	0.4142928	0.0797195	0.2470062	up
A_21_P0012426	XLOC_l2_010063	-0.842057	-0.230551	-0.959696	0.6115065	-0.117639	0.2469337	up
A_24_P18917	LMF1	-1.809044	-1.611132	-1.513173	0.1979127	0.2958713	0.246892	up
A_33_P3406458	NANP	-1.775044	-1.620471	-1.435896	0.1545734	0.339148	0.2468607	up
A_23_P217367	ATG4A	0.9309683	1.0420613	1.3135247	0.111093	0.3825564	0.2468247	up
A_23_P77430	PRMT7	5.109459	5.2268786	5.485655	0.1174197	0.3761959	0.2468078	up

A_23_P89343	SNX11	2.2359219	2.304749	2.6606617	0.0688272	0.4247398	0.2467835	up
A_23_P204696	CDKN1B	1.6434107	1.8706989	1.9096289	0.2272883	0.2662182	0.2467532	up
A_24_P308229	AIM1L	1.4878411	2.0247388	1.4444199	0.5368977	-0.043421	0.2467382	up
A_22_P00013783	lnc-RP11-9B6.1.1-2	-2.386841	-2.165131	-2.115225	0.22171	0.2716155	0.2466627	up
A_23_P51906	PFDN2	6.1441364	6.3451123	6.436453	0.2009759	0.2923164	0.2466462	up
A_23_P127793	EML3	0.1406088	0.4192596	0.3551116	0.2786508	0.2145028	0.2465768	up
A_24_P89701	IMPDH1	6.8113537	7.057492	7.058345	0.2461381	0.2469912	0.2465646	up
A_23_P204484	RAB35	4.829213	5.0725446	5.0789204	0.2433314	0.2497072	0.2465193	up
A_23_P146354	POMT1	2.024231	2.2022576	2.3390255	0.1780267	0.3147945	0.2464106	up
A_23_P215419	ICA1	3.4638739	3.8020844	3.6183958	0.3382106	0.1545219	0.2463663	up
A_23_P146946	CST6	4.085823	4.3744264	4.2899446	0.2886033	0.2041216	0.2463624	up
A_33_P3378031	TMEM18	3.0356922	3.3984838	3.1655903	0.3627915	0.1298981	0.2463448	up
A_24_P152635	TMX2	2.7238045	2.9474034	2.992878	0.223599	0.2690735	0.2463362	up
A_33_P3356306	LYG2	-2.627996	-2.376282	-2.387221	0.2517142	0.2407746	0.2462444	up
A_23_P10232	BANK1	-0.998908	-0.919286	-0.586066	0.0796213	0.4128413	0.2462313	up
A_21_P0000511	SNAR-H	10.19777	10.469381	10.418613	0.2716112	0.2208433	0.2462273	up
A_33_P3211793	LOC729732	-1.603677	-1.624702	-1.090354	-0.021025	0.5133228	0.2461491	up
A_33_P3234899	PSMB3	8.324409	8.474026	8.666921	0.1496172	0.3425121	0.2460647	up
A_23_P215517	KLHL7	2.5749168	2.8412347	2.8005743	0.2663178	0.2256575	0.2459877	up
A_33_P3261625	LSM2	5.908428	6.167277	6.141554	0.2588487	0.2331257	0.2459872	up
A_23_P78108	ALDOC	2.4736137	2.5419116	2.8970432	0.0682979	0.4234295	0.2458637	up
A_23_P374695	TEK	-1.191797	-0.818677	-1.073245	0.3731194	0.1185522	0.2458358	up
A_33_P3239839	FBXL18	4.0371904	4.3221536	4.2438183	0.2849631	0.2066279	0.2457955	up
A_21_P0000245	SNORD18A	-1.727439	-0.902441	-2.06089	0.8249984	-0.333451	0.2457736	up
A_33_P3357979	CTBP1	-0.534556	-0.311424	-0.266188	0.2231317	0.2683678	0.2457497	up
A_23_P19479	STK19	0.4836068	0.7786222	0.6799755	0.2950153	0.1963687	0.245692	up
A_23_P431381	C14orf80	1.6071644	1.8957348	1.8099008	0.2885704	0.2027364	0.2456534	up
A_22_P00000995	LOC101927139	-2.397432	-2.285565	-2.018019	0.111867	0.3794136	0.2456403	up
A_33_P3276329	GATSL2	1.2664981	1.5089836	1.5152435	0.2424855	0.2487454	0.2456155	up
A_32_P193646	RBMX	3.215374	3.5904136	3.3315325	0.3750396	0.1161585	0.245599	up
A_33_P3420792	PDAP1	-1.560781	-1.454614	-1.1758	0.1061668	0.3849812	0.245574	up
A_21_P0006395	lnc-PTGES2-1	3.4213095	3.6331973	3.700488	0.2118878	0.2791786	0.2455332	up
A_23_P429449	NKD1	1.8023062	2.1465783	1.9490604	0.3442721	0.1467543	0.2455132	up
A_33_P3320368	TMEM161A	5.496291	5.518399	5.965019	0.0221076	0.4687281	0.2454178	up
A_22_P00001895	LOC100049716	-1.606009	-1.363325	-1.358097	0.2426839	0.2479124	0.2452981	up
A_22_P00002686	lnc-C1orf86-1	-2.74299	-2.557826	-2.437731	0.1851642	0.3052592	0.2452117	up
A_33_P3291414	LOC100996573	-1.409743	-1.071269	-1.257837	0.3384743	0.1519055	0.2451899	up
A_23_P59613	FZD9	-2.090862	-1.667347	-2.024099	0.4235148	0.0667625	0.2451386	up
A_22_P00019317	lnc-NMNAT3-3	0.2072024	0.5282517	0.3764277	0.3210492	0.1692252	0.2451372	up
A_33_P3289696	OSCP1	-2.309924	-2.002968	-2.126713	0.3069563	0.1832109	0.2450836	up
A_33_P3403733	FAM231A	-0.026304	0.1842823	0.2531209	0.2105861	0.2794247	0.2450054	up
A_33_P3387831	CENPM	5.8882647	6.0028567	6.2636337	0.1145921	0.3753691	0.2449806	up
A_24_P286527	IFT22	1.0741377	1.3255625	1.3126683	0.2514248	0.2385306	0.2449777	up
A_21_P0000152	CD44	-1.847011	-1.650814	-1.553387	0.196197	0.2936239	0.2449105	up
A_33_P3322050	DYRK1A	-3.172716	-3.032378	-2.823259	0.1403382	0.349457	0.2448976	up
A_33_P3344991	TBC1D3L	0.9306793	1.1968164	1.1543112	0.2661371	0.2236319	0.2448845	up
A_24_P870620	PTN	-3.194125	-2.781086	-3.117554	0.4130383	0.0765708	0.2448045	up
A_21_P0010059	lnc-CYP24A1-1	-1.857522	-1.483251	-1.742275	0.3742704	0.1152463	0.2447584	up
A_33_P3234103	SETD1A	4.4346857	4.6689277	4.6898327	0.234242	0.255147	0.2446945	up
A_23_P114232	PRDX4	7.019808	7.340903	7.187892	0.321095	0.1680841	0.2445896	up
A_24_P379820	ITM2C	1.4655681	1.7196217	1.7006865	0.2540536	0.2351184	0.244586	up
A_32_P98975	C15orf57	0.91749	1.0821495	1.2419777	0.1646595	0.3244877	0.2445736	up
A_21_P0002294	ZNF112	-2.00065	-1.683291	-1.829094	0.3173595	0.1715565	0.244458	up
A_23_P107684	ZNF324	0.9593377	1.2356753	1.1718721	0.2763376	0.2125344	0.244436	up
A_24_P941217	SGPP2	0.481936	0.7475743	0.7051683	0.2656384	0.2232323	0.2444353	up

A_23_P1519	PITPNM1	0.9443235	1.1336327	1.2437706	0.1893091	0.2994471	0.2443781	up
A_22_P00012015	lnc-PLD1-2	-1.81535	-1.466708	-1.675257	0.3486419	0.1400929	0.2443674	up
A_22_P00002240	lnc-C10orf114-1	2.8597898	3.0542321	3.1539679	0.1944423	0.294178	0.2443101	up
A_23_P202720	SLC35C1	5.242444	5.3607755	5.6125517	0.1183314	0.3701077	0.2442195	up
A_33_P3321642	GRHL2	-2.046876	-1.582716	-2.022619	0.46416	0.0242572	0.2442086	up
A_21_P0002727	lnc-KCNH8-1	-2.419788	-2.030837	-2.320679	0.3889513	0.0991092	0.2440302	up
A_21_P0000499	SNORD82	-3.184975	-3.209701	-2.672255	-0.024726	0.5127196	0.243997	up
A_23_P310350	SHPK	0.5892439	0.560072	1.1063957	-0.029172	0.5171518	0.2439899	up
A_23_P137715	POGK	2.99405	3.1795955	3.2964382	0.1855454	0.3023882	0.2439668	up
A_23_P156732	PHF1	-1.068315	-0.984847	-0.66388	0.0834675	0.4044347	0.2439511	up
A_33_P3252333	SNORD3B-1	8.530716	8.79384	8.755408	0.2631245	0.2246923	0.2439084	up
A_33_P3340869	SCGB1B2P	-3.005393	-2.838022	-2.685042	0.1673718	0.3203511	0.2438614	up
A_23_P40611	TCN2	-0.584526	-0.154905	-0.526679	0.4296207	0.057847	0.2437339	up
A_22_P00017599	SNHG1	1.3193169	1.8519635	1.2740912	0.5326467	-0.045226	0.2437105	up
A_23_P43175	SEPT10	4.5805387	5.076278	4.572219	0.4957395	-0.00832	0.2437098	up
A_23_P145153	PDCD2	3.5999231	3.8231835	3.8638887	0.2232604	0.2639656	0.243613	up
A_23_P78664	DDX39A	6.954919	7.2271066	7.1698666	0.2721877	0.2149477	0.2435677	up
A_23_P57236	GGT7	-1.288133	-1.238232	-0.85104	0.049901	0.4370928	0.2434969	up
A_24_P116669	CANT1	0.4627833	0.7029328	0.7095442	0.2401495	0.2467609	0.2434552	up
A_33_P3302210	LOC100129129	-0.355258	0.0495577	-0.273166	0.4048162	0.0820923	0.2434542	up
A_22_P00012693	FAM204A	-2.628951	-2.703177	-2.067836	-0.074225	0.5611155	0.2434452	up
A_23_P56680	ATP5G3	8.791098	8.904697	9.164363	0.1135998	0.3732653	0.2434325	up
A_33_P3407424	CDC42EP1	6.8666687	7.118057	7.1020956	0.2513881	0.2354269	0.2434075	up
A_22_P00007307	LOC151475	-1.5598	-1.173968	-1.458867	0.3858318	0.1009331	0.2433825	up
A_32_P24376	KRTAP2-3	0.8026252	1.0706177	1.0211787	0.2679925	0.2185535	0.243273	up
A_23_P315206	CCBL1	0.4077063	0.5769668	0.7249441	0.1692605	0.3172379	0.2432492	up
A_23_P425752	TRIM14	-0.523989	-0.517295	-0.044187	0.0066934	0.4798017	0.2432475	up
A_21_P0011873	XLOC_l2_007767	0.6291838	0.8568349	0.8877354	0.2276511	0.2585516	0.2431014	up
A_21_P0005714	lnc-C8orf34-1	1.030901	1.4039316	1.1440578	0.3730307	0.1131568	0.2430937	up
A_24_P41371	CC2D1A	-0.614522	-0.245507	-0.497439	0.3690143	0.1170826	0.2430484	up
A_21_P0005407	LOC101927610	-2.49757	-2.186847	-2.322347	0.3107226	0.1752231	0.2429729	up
A_23_P139486	CDK2AP1	6.160099	6.2945647	6.5113297	0.1344657	0.3512306	0.2428482	up
A_33_P3256095	XLOC_l2_001324	3.2334366	3.5368896	3.4155111	0.303453	0.1820746	0.2427638	up
A_23_P35796	PPP2R5B	0.4596119	0.9263544	0.4783588	0.4667425	0.0187469	0.2427447	up
A_33_P6815452	ITGA9-AS1	-1.791056	-1.575016	-1.522049	0.2160401	0.2690072	0.2425237	up
A_23_P208400	ADCK4	0.2862239	0.3897862	0.6676822	0.1035624	0.3814583	0.2425103	up
A_23_P256172	NDUFAF3	5.562191	5.70857	5.900774	0.146379	0.338583	0.242481	up
A_23_P33216	ATP5B	6.3543625	6.689779	6.503829	0.3354163	0.1494665	0.2424414	up
A_22_P00004848	LOC100133669	-2.641479	-2.065814	-2.732385	0.5756652	-0.090905	0.2423799	up
A_23_P1948	LBHD1	5.6259775	5.7574897	5.9790745	0.1315122	0.353097	0.2423046	up
A_23_P390172	RNASEL	0.9653955	1.0461154	1.3692517	0.08072	0.4038563	0.2422881	up
A_33_P3412233	POMZP3	-0.226368	0.116457	-0.08473	0.3428254	0.1416383	0.2422318	up
A_22_P00016518	PLAC4	-0.273511	0.0656614	-0.128235	0.3391724	0.1452761	0.2422242	up
A_21_P0010501	HOTAIR	-1.653319	-1.347257	-1.475019	0.3060622	0.1783004	0.2421813	up
A_33_P3252884	PHF12	0.5773182	0.9188151	0.7200537	0.3414969	0.1427355	0.2421162	up
A_24_P930926	BAG4	-0.903504	-0.714828	-0.608089	0.1886759	0.2954149	0.2420454	up
A_33_P3258008	UBE3B	-2.242605	-2.118501	-1.882619	0.1241047	0.3599858	0.2420453	up
A_23_P82693	PABPC1	8.800875	9.212248	8.873426	0.4113731	0.0725517	0.2419624	up
A_33_P3405957	BCL9L	1.4409065	1.7624087	1.6031132	0.3215022	0.1622067	0.2418544	up
A_23_P80321	POLR2F	7.074085	7.2422957	7.3895397	0.1682105	0.3154545	0.2418325	up
A_24_P322771	TFF1	-1.348732	-1.072457	-1.141516	0.2762742	0.2072158	0.241745	up
A_23_P3562	SLC7A6OS	2.5782232	2.8798385	2.7600088	0.3016152	0.1817856	0.2417004	up
A_22_P00005218	MEG3	-2.630043	-2.497379	-2.279306	0.1326635	0.3507371	0.2417003	up
A_23_P80342	TAB1	0.4445167	0.7805791	0.591723	0.3360624	0.1472063	0.2416344	up
A_33_P3394297	THNSL2	-3.326477	-2.772409	-3.39733	0.5540688	-0.070853	0.2416081	up
A_22_P00017341	ERVMER34-1	3.441143	3.6635299	3.7018023	0.2223868	0.2606592	0.241523	up

A_33_P3261828	ADAMTS7P1	-2.272801	-1.934823	-2.127783	0.3379788	0.1450181	0.2414985	up
A_33_P3220152	SBNO2	4.01379	4.2627788	4.247652	0.2489886	0.2338619	0.2414253	up
A_33_P3272330	DNMT3A	2.0051403	2.1998448	2.2932358	0.1947045	0.2880955	0.2414	up
A_23_P152428	MARVELD3	1.1737719	1.296042	1.534265	0.1222701	0.3604932	0.2413816	up
A_24_P120934	GADD45G	-0.747246	-0.334582	-0.677297	0.4126635	0.0699487	0.2413061	up
A_32_P4228	LINC00687	-1.554458	-1.316222	-1.310124	0.238236	0.2443333	0.2412846	up
A_22_P00021319	LOC101929533	-0.34884	-0.00488	-0.210434	0.3439603	0.1384058	0.2411831	up
A_23_P100654	ZBTB4	1.5631342	1.9779449	1.6304922	0.4148107	0.067358	0.2410843	up
A_23_P128728	ARG2	0.8437915	1.2553377	0.9143796	0.4115462	0.0705881	0.2410672	up
A_23_P94159	FBXO25	4.2588434	4.4054894	4.5943193	0.146646	0.3354759	0.241061	up
A_22_P00001044	C1orf86	-0.9065	-0.735267	-0.595808	0.1712337	0.3106923	0.240963	up
A_33_P3334398	CA6	-0.991676	-0.79938	-0.702092	0.1922956	0.2895842	0.2409399	up
A_21_P0001290	LINC00853	-0.597341	-0.525491	-0.187351	0.0718498	0.4099903	0.2409201	up
A_33_P3397161	SHANK2-AS3	-0.058722	0.2758741	0.0883985	0.3345962	0.1471205	0.2408583	up
A_33_P3227041	BID	6.503975	6.665565	6.823883	0.1615901	0.3199081	0.2407491	up
A_23_P67299	DOCK6	1.5695448	1.8469734	1.7736125	0.2774286	0.2040677	0.2407482	up
A_33_P3353520	RNF225	-2.5092	-2.266796	-2.270212	0.2424042	0.2389886	0.2406964	up
A_24_P404033	ARPIN	0.1600208	0.3816586	0.4197741	0.2216377	0.2597532	0.2406955	up
A_33_P3218138	POLR2A	5.777426	6.02031	6.01575	0.2428842	0.2383242	0.2406042	up
A_23_P130343	KCTD1	2.281846	2.388311	2.6565485	0.1064649	0.3747025	0.2405837	up
A_23_P310560	NUDT16	0.2488589	0.3268023	0.6520805	0.0779433	0.4032216	0.2405825	up
A_21_P0000289	SNORD45A	0.9958649	1.1102271	1.3626547	0.1143622	0.3667898	0.240576	up
A_21_P0010462	LRRC74B	-0.472562	-0.146732	-0.317323	0.32583	0.1552391	0.2405345	up
A_23_P337033	GMEB2	0.092907	0.4387388	0.2279563	0.3458319	0.1350493	0.2404406	up
A_23_P127088	CUEDC2	3.7588797	4.0126195	3.9858284	0.2537398	0.2269487	0.2403443	up
A_23_P211445	LIMK2	4.0638494	4.3545747	4.253686	0.2907252	0.1898365	0.2402809	up
A_23_P18798	PCDHB9	1.7903528	1.6826596	2.3783646	-0.107693	0.5880117	0.2401593	up
A_32_P132396	FAM86JP	-2.264706	-1.922213	-2.127148	0.3424931	0.137558	0.2400255	up
A_23_P37441	B2M	7.5937176	7.865234	7.802243	0.2715163	0.2085257	0.240021	up
A_23_P303423	SLAIN2	-2.87441	-2.739157	-2.52973	0.1352532	0.3446796	0.2399664	up
A_23_P1594	VEGFB	4.603985	4.849649	4.8380823	0.2456641	0.2340975	0.2398808	up
A_33_P3219146	ODF3	-1.939444	-1.864036	-1.535244	0.075408	0.4042001	0.239804	up
A_23_P341503	C3orf35	-2.801268	-2.580047	-2.542961	0.2212212	0.2583072	0.2397642	up
A_21_P0013830	XLOC_l2_015812	0.9528899	1.2797899	1.105403	0.3269	0.152513	0.2397065	up
A_33_P3237664	HDAC7	-2.836964	-2.917339	-2.277213	-0.080374	0.5597513	0.2396885	up
A_23_P312150	EDN2	-0.917391	-0.488194	-0.867245	0.4291973	0.0501456	0.2396715	up
A_23_P64650	TSFM	4.974307	5.1670747	5.2607803	0.1927676	0.2864733	0.2396204	up
A_23_P208373	CYP2B6	-1.594095	-1.385383	-1.323636	0.2087116	0.2704592	0.2395854	up
A_33_P3402500	TMEM14C	4.974334	5.29318	5.1345596	0.3188462	0.1602259	0.2395361	up
A_33_P3446495	FRMD4B	-1.491238	-1.212355	-1.291232	0.278883	0.200006	0.2394445	up
A_23_P114689	ASAP3	-2.131906	-1.844999	-1.939961	0.2869067	0.1919441	0.2394254	up
A_23_P138776	ACP2	-0.517442	-0.25554	-0.300568	0.2619023	0.2168746	0.2393885	up
A_24_P862886	DRD5	-3.013876	-2.601139	-2.947942	0.4127371	0.0659342	0.2393357	up
A_23_P29836	TMEM42	2.6740742	2.953054	2.8737621	0.2789798	0.199688	0.2393339	up
A_33_P3216913	ABHD15	-0.320302	-0.191814	0.0296035	0.1284876	0.3499055	0.2391965	up
A_23_P211850	ABHD6	2.5712986	2.7859473	2.8349886	0.2146487	0.26369	0.2391694	up
A_33_P3270102	RNF126	0.8892646	1.1352406	1.1215115	0.245976	0.2322469	0.2391114	up
A_21_P0000163	RNF223	-0.009239	0.4870205	-0.027555	0.4962597	-0.018316	0.238972	up
A_24_P59220	POTEF	5.562215	5.710814	5.891514	0.1485992	0.329299	0.2389491	up
A_24_P108242	GOSR2	1.4995055	1.672061	1.8047915	0.1725555	0.3052859	0.2389207	up
A_21_P0007407	lnc-RP11-158I9.5.1-2	1.8212872	2.1238027	1.9966097	0.3025155	0.1753225	0.238919	up
A_23_P218463	SERTAD1	6.216626	6.146687	6.7642145	-0.069939	0.5475884	0.2388246	up
A_21_P0012285	XLOC_l2_009468	-0.756153	-0.593234	-0.441599	0.162919	0.3145537	0.2387364	up
A_23_P348257	NUAK1	3.5712957	3.8208919	3.7991161	0.2495961	0.2278204	0.2387083	up
A_33_P3417640	KLK14	3.099966	3.4983497	3.1789684	0.3983836	0.0790024	0.238693	up

A_23_P31866	MED30	1.659338	1.9721417	1.823853	0.3128038	0.164515	0.2386594	up
A_33_P3362311	C6orf222	-1.582945	-1.078302	-1.610302	0.5046434	-0.027356	0.2386436	up
A_23_P207106	CHRNB1	-3.03038	-2.836913	-2.746618	0.1934664	0.283762	0.2386142	up
A_23_P142403	TM6SF2	-2.406999	-2.308275	-2.028581	0.0987239	0.3784175	0.2385707	up
A_24_P221198	MBLAC1	-0.759525	-0.462135	-0.579964	0.29739	0.1795611	0.2384756	up
A_33_P3312790	MEGF11	-0.801897	-0.53564	-0.591211	0.2662568	0.2106853	0.238471	up
A_22_P00007867	lnc-HS3ST3A1-4	-2.827107	-2.773836	-2.403564	0.0532708	0.4235427	0.2384068	up
A_33_P3307197	PTGFRN	2.2398071	2.4514651	2.504837	0.211658	0.2650299	0.238344	up
A_21_P0004313	lnc-PPARGC1B-1	-1.755608	-1.667171	-1.367428	0.0884371	0.3881798	0.2383084	up
A_33_P3343428	TRAF3IP2	2.6070948	2.7754045	2.9153748	0.1683097	0.30828	0.2382948	up
A_23_P145006	SCGB3A2	0.6671104	0.8862004	0.924561	0.21909	0.2574506	0.2382703	up
A_23_P319492	FAM118B	0.7549081	0.9265208	1.0598316	0.1716127	0.3049235	0.2382681	up
A_24_P79755	AKR1A1	3.0150576	3.1967254	3.3097048	0.1816678	0.2946472	0.2381575	up
A_24_P399980	HEPH	0.1903119	0.4225764	0.4341974	0.2322645	0.2438855	0.238075	up
A_21_P0012925	XLOC_l2_012210	-0.839297	-1.087395	-0.115081	-0.248098	0.7242165	0.2380593	up
A_33_P3243337	MYDGF	6.6470194	7.022036	6.747898	0.3750167	0.1008787	0.2379477	up
A_23_P106463	OAZ2	3.9269657	4.1389422	4.1908617	0.2119765	0.263896	0.2379363	up
A_24_P910297	GOLGA2	1.5987577	1.8832994	1.7898221	0.2845416	0.1910644	0.237803	up
A_33_P3220160	RAC2	0.4477334	0.7947688	0.5762825	0.3470354	0.1285491	0.2377923	up
A_23_P209519	DNAJB2	1.2047925	1.5513558	1.3336287	0.3465633	0.1288362	0.2376997	up
A_33_P3377294	TCF7L2	1.1480927	1.4711151	1.3004527	0.3230224	0.15236	0.2376912	up
A_24_P154006	NDUFB9	8.506094	8.750832	8.736653	0.2447376	0.2305594	0.2376485	up
A_21_P0009360	LINC00673	-1.131087	-0.733255	-1.053731	0.3978314	0.0773563	0.2375939	up
A_23_P78170	MYBBP1A	2.5298347	2.8653598	2.6694603	0.335525	0.1396256	0.2375753	up
A_32_P191859	SETD8	2.0953722	2.2575698	2.4082708	0.1621976	0.3128986	0.2375481	up
A_24_P324563	ABHD17A	2.4042902	2.6275506	2.6560707	0.2232604	0.2517805	0.2375205	up
A_33_P3363153	DNAJC5	3.5403843	3.8246884	3.7311163	0.2843041	0.190732	0.2375181	up
A_23_P15466	SCO1	2.2039957	2.389771	2.4931479	0.1857753	0.2891522	0.2374637	up
A_33_P3318671	LYPLA2	6.1310673	6.106524	6.630495	-0.024543	0.4994278	0.2374423	up
A_33_P3351554	ETNK2	0.5676856	0.7978945	0.8122873	0.2302089	0.2446017	0.2374053	up
A_22_P00007924	TEX26-AS1	-1.809298	-1.637778	-1.506078	0.1715202	0.3032198	0.23737	up
A_23_P139228	ARFIP2	2.2468119	2.6507325	2.3176289	0.4039207	0.070817	0.2373688	up
A_21_P0005099	LINC01128	-0.052117	0.1355972	0.2346463	0.1877141	0.2867632	0.2372387	up
A_32_P104432	LINC00087	-0.565732	-0.551702	-0.105298	0.01403	0.4604335	0.2372317	up
A_33_P3365134	PPP1R13B	1.3918672	1.6251101	1.6329837	0.233243	0.2411165	0.2371798	up
A_23_P1641	RCE1	0.3749323	0.5533199	0.670639	0.1783876	0.2957068	0.2370472	up
A_23_P87973	TRIM13	2.7664032	3.003388	3.0034323	0.2369847	0.2370291	0.2370069	up
A_23_P139471	RPS26	1.0266061	1.2707787	1.2564168	0.2441726	0.2298107	0.2369916	up
A_23_P338505	C19orf40	-2.142289	-1.979676	-1.830948	0.1626129	0.3113413	0.2369771	up
A_23_P338495	MARCH8	-2.821235	-2.51504	-2.653478	0.3061953	0.167757	0.2369761	up
A_33_P3237220	FBXO24	-0.07578	0.2998881	0.0223932	0.3756681	0.0981731	0.2369206	up
A_33_P3363310	KRTAP5-10	-0.015856	0.1960836	0.2456236	0.2119398	0.2614799	0.2367098	up
A_33_P3251538	MAPKAP1	1.4895911	1.6397672	1.8128252	0.1501761	0.3232341	0.2367051	up
A_33_P3209030	ZNF623	-0.858233	-0.617642	-0.625482	0.2405911	0.2327514	0.2366712	up
A_22_P00004217	C1RL-AS1	-0.100441	0.0075974	0.264811	0.1080389	0.3652525	0.2366457	up
A_23_P48307	PABPC3	6.184169	6.5942025	6.2473917	0.4100337	0.0632229	0.2366283	up
A_33_P3337019	LOC728975	-1.819925	-1.743779	-1.422939	0.0761466	0.3969865	0.2365665	up
A_32_P351968	HLA-DMB	-0.920426	-0.817066	-0.550919	0.1033607	0.3695073	0.236434	up
A_24_P277295	RAB43	3.182105	3.1637416	3.6732626	-0.018363	0.4911575	0.236397	up
A_33_P3345255	KBTBD4	0.5097022	0.7476182	0.7444663	0.237916	0.2347641	0.2363401	up
A_21_P0000372	SNORD45C	-2.066649	-1.925232	-1.735431	0.141417	0.3312187	0.2363179	up
A_22_P00021572	lnc-ZFYVE1-1	-2.383061	-1.749715	-2.54385	0.6333466	-0.160788	0.2362793	up
A_24_P303497	MCM9	-2.574984	-2.620646	-2.056937	-0.045662	0.5180476	0.236193	up
A_24_P194420	CCDC134	-1.19855	-0.857823	-1.066916	0.3407264	0.1316338	0.2361801	up
A_21_P0008448	LOC101927856	-0.862834	-0.702056	-0.551255	0.1607771	0.3115783	0.2361777	up
A_33_P3312104	CTC1	-1.984979	-1.792844	-1.704796	0.1921344	0.2801824	0.2361584	up

A_22_P00004869	lnc-CYP7B1-3	-0.008887	0.3382454	0.1162362	0.3471322	0.125123	0.2361276	up
A_21_P0000201	RAB6C	5.353429	5.591492	5.5875273	0.2380633	0.2340984	0.2360809	up
A_33_P3330503	ALDH7A1	0.0079708	0.2174506	0.2706223	0.2094798	0.2626514	0.2360656	up
A_23_P63825	GOT1	3.986208	4.1991515	4.245389	0.2129436	0.259181	0.2360623	up
A_23_P409438	IFNL2	-1.919516	-1.658412	-1.708583	0.2611036	0.2109327	0.2360182	up
A_23_P106024	JAG2	1.4705348	1.8464084	1.5665035	0.3758736	0.0959687	0.2359211	up
A_23_P10463	NDUFS5	8.78627	8.963928	9.080392	0.1776581	0.2941217	0.2358899	up
A_23_P83835	POMK	-1.359359	-1.133887	-1.113077	0.225472	0.2462816	0.2358768	up
A_23_P118306	DNAJA3	4.944479	5.183636	5.1770363	0.2391572	0.2325573	0.2358573	up
A_21_P0011492	SLC22A31	-1.813886	-1.601967	-1.554142	0.2119188	0.2597446	0.2358317	up
A_23_P62607	IL22RA1	1.4917707	1.8106174	1.6445122	0.3188467	0.1527414	0.2357941	up
A_23_P255215	RP9	2.0750408	2.1498866	2.471692	0.0748458	0.3966513	0.2357485	up
A_21_P0000846	SH3PXD2A-AS1	-0.31193	-0.247185	0.0945911	0.0647445	0.4065208	0.2356327	up
A_22_P00001276	lnc-ANKRD6-1	0.3012848	0.6174121	0.4563117	0.3161273	0.1550269	0.2355771	up
A_23_P48610	TMEM55B	2.116949	2.3434567	2.3615046	0.2265077	0.2445555	0.2355316	up
A_23_P135769	ACTB	5.762786	6.0246987	5.971924	0.2619128	0.2091379	0.2355254	up
A_23_P30799	HIST1H3F	4.8087893	4.949452	5.139019	0.1406627	0.3302298	0.2354462	up
A_23_P137586	GMEB1	1.4866676	1.7410946	1.7031107	0.254427	0.2164431	0.235435	up
A_23_P170088	EXD3	-1.464783	-1.235692	-1.22306	0.2290912	0.2417226	0.2354069	up
A_33_P3244317	MESDC2	1.3741531	1.7760186	1.4430428	0.4018655	0.0688896	0.2353776	up
A_23_P423864	PHC2	5.400614	5.4752297	5.796646	0.074616	0.3960323	0.2353241	up
A_23_P353316	SMURF1	-0.376474	-0.248633	-0.033838	0.127841	0.3426366	0.2352388	up
A_23_P350698	ANKFN1	-2.144815	-1.761729	-2.057687	0.3830857	0.0871277	0.2351067	up
A_23_P317056	ND6	3.4110527	3.8169465	3.475357	0.4058938	0.0643044	0.2350991	up
A_24_P268676	BHLHE40	2.1330204	2.5200753	2.2161427	0.3870549	0.0831223	0.2350886	up
A_33_P3299170	LUC7L	5.280402	5.4516625	5.5791883	0.1712604	0.2987862	0.2350233	up
A_23_P80156	PRMT2	0.4839706	0.9104929	0.5274777	0.4265223	0.0435071	0.2350147	up
A_23_P34700	TNNT2	-1.802468	-1.53358	-1.601356	0.2688885	0.2011128	0.2350006	up
A_24_P364087	SERGEF	1.800807	1.7940011	2.2775478	-0.006806	0.4767408	0.2349675	up
A_23_P256682	APEX2	4.402232	4.5062976	4.767928	0.1040654	0.365696	0.2348807	up
A_23_P19673	SGK1	2.1847448	2.339623	2.4996233	0.1548781	0.3148785	0.2348783	up
A_33_P3409506	C9orf85	-0.10728	0.1068583	0.1482739	0.2141385	0.2555542	0.2348464	up
A_23_P4662	BCL3	4.2936506	4.684791	4.3721943	0.3911405	0.0785437	0.2348421	up
A_23_P73540	CCDC120	3.042943	3.4139266	3.1415567	0.3709836	0.0986137	0.2347987	up
A_23_P207125	NLGN2	-0.184845	0.0498323	0.0499363	0.2346768	0.2347808	0.2347288	up
A_23_P89812	CNDP2	2.4515142	2.725511	2.6466837	0.2739968	0.1951695	0.2345831	up
A_22_P00001288	lnc-ANKUB1-1	-2.466516	-2.473997	-1.989883	-0.007481	0.4766331	0.2345761	up
A_32_P218355	C6orf132	5.4960346	5.7887197	5.672477	0.292685	0.1764422	0.2345636	up
A_24_P116871	TXNL4A	3.8377457	4.0475087	4.0970287	0.2097631	0.2592831	0.2345231	up
A_23_P208523	LENG1	-0.190429	0.0264049	0.0617723	0.2168336	0.2522011	0.2345173	up
A_33_P3291294	EXTL3	4.483979	4.6279616	4.8089666	0.1439824	0.3249874	0.2344849	up
A_23_P424712	CCDC142	1.9697771	2.3295398	2.0789165	0.3597627	0.1091394	0.2344511	up
A_32_P158746	RPL17	7.145996	7.4166255	7.344268	0.2706294	0.1982718	0.2344506	up
A_22_P00007282	lnc-GPR19-1	-2.777454	-2.310735	-2.775296	0.4667194	0.0021577	0.2344385	up
A_33_P3315949	CCDC103	-0.571096	-0.045791	-0.627549	0.5253048	-0.056453	0.2344258	up
A_23_P151321	RPL6	8.45358	8.745827	8.630164	0.2922468	0.1765842	0.2344155	up
A_24_P146211	HIST1H2BD	3.24403	3.493031	3.4638014	0.249001	0.2197714	0.2343862	up
A_33_P3350638	KIF22	-0.985014	-0.819626	-0.681842	0.1653876	0.3031712	0.2342794	up
A_23_P355471	TBXA2R	-0.444591	-0.182229	-0.238448	0.262362	0.2061429	0.2342525	up
A_22_P00007101	LOC100506999	-2.925971	-2.569743	-2.813759	0.3562286	0.1122122	0.2342204	up
A_23_P7402	PDZD2	-0.66888	-0.336288	-0.533128	0.3325925	0.1357522	0.2341724	up
A_21_P0005856	lnc-SLCO5A1-3	-2.505723	-2.011773	-2.531521	0.4939506	-0.025798	0.2340764	up
A_21_P0009295	lnc-ALOX12B-1	-0.29179	-0.042903	-0.072651	0.2488866	0.2191391	0.2340128	up
A_22_P00009861	lnc-METTL23-2	0.0075879	0.0868411	0.3963375	0.0792532	0.3887496	0.2340014	up
A_33_P3261914	LOC102724738	-1.497194	-1.352285	-1.174162	0.1449084	0.3230319	0.2339702	up
A_33_P3411097	GRID2IP	-0.858033	-0.53376	-0.714375	0.3242731	0.1436582	0.2339656	up

A_33_P3306113	JMJD4	4.533985	4.740277	4.795583	0.2062917	0.2615976	0.2339447	up
A_33_P3358908	KRTAP9-6	1.514226	1.655983	1.8403578	0.141757	0.3261318	0.2339444	up
A_33_P3278058	PIH1D2	-0.541781	-0.486187	-0.129495	0.055593	0.4122853	0.2339392	up
A_32_P109704	SFSWAP	2.8821964	3.1558194	3.0760574	0.273623	0.193861	0.233742	up
A_22_P00015046	NPR1	-1.890741	-1.882156	-1.43189	0.0085845	0.4588513	0.2337179	up
A_33_P3242458	SLC41A3	1.1628766	1.4313564	1.3617463	0.2684798	0.1988697	0.2336748	up
A_33_P3539223	ARHGEF39	-0.871407	-0.883042	-0.392431	-0.011636	0.4789753	0.2336698	up
A_33_P3218584	POP5	5.2370024	5.320748	5.620244	0.0837455	0.3832417	0.2334936	up
A_23_P166491	SGSM3	1.2037215	1.4210424	1.4532399	0.2173209	0.2495184	0.2334197	up
A_23_P68665	ADRM1	3.060277	3.2651916	3.3221912	0.2049146	0.2619143	0.2334144	up
A_33_P3265376	HOMER3	0.3118925	0.4546127	0.6359472	0.1427202	0.3240547	0.2333875	up
A_24_P134235	KHSRP	-1.134101	-1.122847	-0.678589	0.0112543	0.4555116	0.2333829	up
A_33_P3227686	MROH5	-1.89471	-1.457817	-1.864961	0.4368935	0.0297494	0.2333214	up
A_33_P3361925	PHF8	-0.73184	-0.384511	-0.612573	0.3473291	0.1192665	0.2332978	up
A_23_P24345	SLC39A13	4.4023085	4.7613807	4.5098104	0.3590722	0.107502	0.2332871	up
A_23_P101905	APC2	-1.724747	-0.976556	-2.006404	0.7481909	-0.281657	0.2332668	up
A_21_P0008954	lnc-SETD6-8	-2.851932	-2.976597	-2.260769	-0.124665	0.5911624	0.2332487	up
A_24_P60845	ACHE	-2.352419	-2.191177	-2.047261	0.1612423	0.3051577	0.2332	up
A_23_P1292	ERCC6	-0.342341	0.1579394	-0.376432	0.5002809	-0.034091	0.2330952	up
A_22_P00015348	LOC283335	-0.883059	-0.598901	-0.701027	0.2841582	0.1820321	0.2330952	up
A_22_P00011455	lnc-PAPLN-1	-1.425505	-1.087541	-1.297285	0.3379636	0.1282196	0.2330916	up
A_24_P99071	IPO5	3.3599176	3.7016263	3.484355	0.3417087	0.1244373	0.233073	up
A_22_P00012485	ERVK13-1	-2.381584	-2.252241	-2.044882	0.1293435	0.3367019	0.2330227	up
A_33_P3289207	AAK1	0.8393035	1.118876	1.0257359	0.2795725	0.1864324	0.2330024	up
A_23_P1638	RBM4B	1.4726005	1.7465739	1.6645136	0.2739735	0.1919131	0.2329433	up
A_24_P915371	MYEOV2	7.187524	7.320727	7.5201044	0.133203	0.3325806	0.2328918	up
A_22_P00021960	lnc-SMYD3-2	-2.164656	-1.59423	-2.269307	0.5704265	-0.104651	0.2328877	up
A_33_P3357530	SLC12A7	6.032089	6.206533	6.323262	0.1744437	0.291173	0.2328084	up
A_23_P106056	DAD1	6.044303	6.2038054	6.3504095	0.1595025	0.3061066	0.2328045	up
A_23_P68059	PCGF1	1.5445776	1.866353	1.688376	0.3217754	0.1437984	0.2327869	up
A_23_P85693	GBP2	-1.583257	-1.661868	-1.039129	-0.078611	0.5441279	0.2327585	up
A_19_P00318813	LOC101928738	2.529315	2.6395516	2.884595	0.1102366	0.3552799	0.2327583	up
A_21_P0001741	lnc-HIST2H3PS2-3	0.3084641	0.4971261	0.5852957	0.1886621	0.2768316	0.2327468	up
A_23_P102364	NGEF	3.971427	4.169539	4.2387114	0.198112	0.2672844	0.2326982	up
A_23_P308603	SRC	1.8507185	1.9839978	2.1827755	0.1332793	0.332057	0.2326682	up
A_24_P181677	PQLC1	0.3560872	0.7060866	0.4714055	0.3499994	0.1153183	0.2326589	up
A_22_P00016355	LOC728730	3.8873434	4.2534614	3.986475	0.366118	0.0991316	0.2326248	up
A_33_P3277097	LY6G6E	-1.773939	-1.741093	-1.341575	0.032846	0.4323645	0.2326052	up
A_33_P3261877	LOC100506639	1.7152205	2.0041118	1.891346	0.2888913	0.1761255	0.2325084	up
A_21_P0007015	lnc-OBFC1-2	-2.745562	-2.880464	-2.145743	-0.134902	0.5998189	0.2324582	up
A_33_P3244165	RNA28S5	10.434072	10.727299	10.605726	0.2932272	0.1716547	0.232441	up
A_23_P135914	SF3B3	4.967269	5.1763196	5.223093	0.2090507	0.2558241	0.2324374	up
A_21_P0014863	PPP2R5C	0.0893407	0.2546539	0.3887615	0.1653132	0.2994208	0.232367	up
A_24_P11462	AZIN2	-0.562731	-0.396874	-0.263865	0.1658564	0.2988663	0.2323613	up
A_33_P3575854	LOC642361	2.996337	3.3106418	3.1466494	0.3143048	0.1503124	0.2323086	up
A_21_P0000173	C5orf56	1.4181848	1.5353093	1.7655687	0.1171246	0.347384	0.2322543	up
A_23_P257743	SHB	2.4449987	2.610127	2.744358	0.1651282	0.2993593	0.2322438	up
A_23_P315286	R3HDM4	1.5648136	1.880928	1.7130198	0.3161144	0.1482062	0.2321603	up
A_22_P00002592	lnc-C19orf26-2	-1.779928	-1.520066	-1.575571	0.2598615	0.2043572	0.2321093	up
A_33_P3299739	CLDN19	1.3218546	1.5081048	1.5998096	0.1862502	0.2779551	0.2321026	up
A_24_P51909	CPLX1	-0.668459	-0.326032	-0.546704	0.3424268	0.1217551	0.232091	up
A_23_P152235	IRX3	-2.885819	-2.544845	-2.762657	0.3409731	0.1231613	0.2320672	up
A_23_P76071	B3GNT4	-1.582174	-1.538748	-1.161489	0.043426	0.4206848	0.2320554	up
A_23_P30784	ABT1	-0.296208	-0.115602	-0.012943	0.1806059	0.2832646	0.2319353	up
A_23_P86917	FADD	5.7965984	5.888802	6.1681976	0.0922036	0.3715992	0.2319014	up
A_22_P00010980	DHRS4-AS1	-1.008308	-0.7192	-0.833681	0.2891073	0.1746268	0.2318671	up

A_19_P00320384	CA5BP1	0.0477452	0.2785301	0.2805719	0.2307849	0.2328267	0.2318058	up
A_33_P3466016	LOC200830	0.535831	0.6724916	0.8624945	0.1366606	0.3266635	0.231662	up
A_21_P0009779	LOC100506634	-3.577294	-3.351671	-3.339809	0.2256236	0.2374857	0.2315546	up
A_23_P31085	CCDC28A	2.974434	3.4248013	2.9870653	0.4503675	0.0126314	0.2314994	up
A_23_P200535	TRAPPC3	3.5393639	3.8592334	3.682372	0.3198695	0.1430082	0.2314389	up
A_24_P84396	CEMIP	-1.577418	-1.349206	-1.342798	0.2282114	0.2346196	0.2314155	up
A_33_P3240094	NGEF	-0.796564	-0.866233	-0.264103	-0.06967	0.5324602	0.2313952	up
A_33_P3314579	RAB34	3.765606	4.0022273	3.9917116	0.2366214	0.2261057	0.2313635	up
A_23_P28953	DNMT3B	0.9113112	1.0068154	1.2783031	0.0955043	0.366992	0.2312481	up
A_33_P3288180	CDK11A	2.1867676	2.4132857	2.4227161	0.2265182	0.2359486	0.2312334	up
A_32_P435367	PSMD4	6.1995344	6.4037185	6.4577303	0.2041841	0.2581959	0.23119	up
A_23_P422115	C9orf116	2.1784859	2.5342155	2.2850637	0.3557296	0.1065779	0.2311537	up
A_22_P00025334	LINC01119	-1.881991	-1.577732	-1.724025	0.3042593	0.1579666	0.231113	up
A_22_P00003884	LINC00475	-1.524643	-1.527798	-1.059399	-0.003154	0.4652448	0.2310452	up
A_33_P3422030	FXYD5	-2.701903	-2.338521	-2.603485	0.3633816	0.0984178	0.2308997	up
A_23_P107073	RPA1	4.7637014	4.9761624	5.013027	0.212461	0.2493258	0.2308934	up
A_23_P112162	DGAT1	3.8028584	3.9987292	4.068673	0.1958709	0.2658148	0.2308428	up
A_33_P3449417	KARS	5.672633	5.889405	5.9174976	0.2167716	0.2448645	0.230818	up
A_33_P3250730	ASIC4	1.1900835	1.5233583	1.3184104	0.3332748	0.1283269	0.2308009	up
A_23_P79161	PRELID1	6.586748	6.7858043	6.8492336	0.1990562	0.2624855	0.2307708	up
A_23_P151970	FEM1B	0.086349	0.4878001	0.1462879	0.4014511	0.0599389	0.230695	up
A_24_P506977	SNHG15	4.800931	5.0151753	5.047964	0.2142444	0.2470331	0.2306387	up
A_23_P143551	ATP6V1E1	2.4164324	2.6085267	2.6855135	0.1920943	0.2690811	0.2305877	up
A_33_P3241741	SNORA23	0.9713993	1.2249045	1.1790485	0.2535052	0.2076492	0.2305772	up
A_23_P368225	EME1	0.269156	0.6749487	0.3244567	0.4057927	0.0553007	0.2305467	up
A_21_P0011841	PAFAH1B1	3.084177	3.2787375	3.3506966	0.1945605	0.2665196	0.23054	up
A_22_P00019867	lnc-GSTO2-2	-0.223938	0.2205725	-0.207838	0.44451	0.0160995	0.2303047	up
A_32_P169574	OTUD5	-0.570839	-0.115538	-0.565595	0.4553018	0.0052447	0.2302732	up
A_33_P3383422	DHRS4	1.0189762	1.1604896	1.3378277	0.1415134	0.3188515	0.2301824	up
A_33_P3245709	RALGAPB	2.3940716	2.70571	2.5427675	0.3116384	0.148696	0.2301672	up
A_21_P0002458	lnc-CD207-1	-2.675412	-2.564754	-2.325782	0.1106582	0.3496301	0.2301441	up
A_23_P431933	CAMKK1	-0.150742	0.0762019	0.0824475	0.2269435	0.2331891	0.2300663	up
A_23_P159039	SCRIB	6.106373	6.379641	6.2930956	0.2732682	0.1867228	0.2299955	up
A_33_P3410724	SDHC	5.6857653	5.844053	5.987443	0.1582875	0.3016777	0.2299826	up
A_23_P79545	SUCLG1	3.9360676	4.0979934	4.234087	0.1619258	0.2980194	0.2299726	up
A_33_P3359219	PPAPDC1B	1.7452593	2.006722	1.943594	0.2614627	0.1983347	0.2298987	up
A_33_P3420841	EIF3D	-1.248245	-1.03519	-1.001575	0.2130556	0.2466703	0.2298629	up
A_24_P233570	PSMB1	8.663168	8.884872	8.901128	0.2217045	0.2379599	0.2298322	up
A_24_P161018	PARP14	2.9711847	3.1065526	3.2954254	0.1353679	0.3242407	0.2298043	up
A_33_P3232047	TMEM14B	5.4600763	5.755347	5.6243334	0.2952704	0.1642571	0.2297637	up
A_23_P26759	CANT1	2.4229813	2.449862	2.8556156	0.0268807	0.4326344	0.2297575	up
A_24_P393470	MEF2BNB	-0.898828	-0.925343	-0.412811	-0.026515	0.4860172	0.2297511	up
A_23_P128828	PABPN1	5.172762	5.4334893	5.3715143	0.2607274	0.1987524	0.2297399	up
A_33_P3265714	C2orf61	-3.05084	-2.875216	-2.766993	0.1756244	0.2838473	0.2297359	up
A_33_P3316508	RUNX2	-2.451616	-2.117388	-2.326503	0.3342285	0.1251133	0.2296709	up
A_23_P133227	HARS	4.5153065	4.703263	4.786685	0.1879563	0.2713785	0.2296674	up
A_21_P0000748	TCEB3-AS1	-0.30028	-0.046997	-0.094285	0.2532826	0.2059946	0.2296386	up
A_23_P53736	FBXO21	3.9649267	4.2981973	4.090542	0.3332706	0.1256151	0.2294428	up
A_23_P106708	RPS2	10.578408	10.882992	10.732687	0.3045836	0.1542788	0.2294312	up
A_23_P52610	DDB2	3.6518517	3.8439517	3.9183598	0.1921001	0.2665081	0.2293041	up
A_21_P0009305	lnc-MFAP4-4	-1.179078	-1.005566	-0.894021	0.1735115	0.2850571	0.2292843	up
A_33_P3357573	MAML3	-2.513406	-2.369611	-2.198659	0.1437953	0.3147469	0.2292711	up
A_23_P53891	KLF5	3.8499708	4.167128	3.9913244	0.3171573	0.1413536	0.2292554	up
A_21_P0003255	lnc-P2RY1-3	-3.564521	-3.120543	-3.550049	0.4439778	0.014472	0.2292249	up
A_33_P3355877	NFXL1	-2.453165	-2.134403	-2.313511	0.3187623	0.1396537	0.229208	up
A_23_P201097	GUK1	6.0925884	6.2334294	6.4101496	0.140841	0.3175612	0.2292011	up

A_22_P00008681	NUP50-AS1	2.1821852	2.5787816	2.2439423	0.3965964	0.0617571	0.2291768	up
A_23_P92754	FGFR4	-0.320848	-0.20827	0.0249081	0.1125774	0.3457556	0.2291665	up
A_23_P35456	SH3PXD2A	3.1974335	3.4228654	3.4303036	0.2254319	0.2328701	0.229151	up
A_23_P15582	XYLT2	5.313669	5.447499	5.638115	0.1338296	0.3244457	0.2291377	up
A_23_P252193	ITGA9	-1.753211	-1.520989	-1.527227	0.2322216	0.2259836	0.2291026	up
A_33_P3345132	ZNF578	-2.141594	-1.863657	-1.961461	0.2779374	0.1801333	0.2290354	up
A_23_P103837	UBAP2L	2.35927	2.66752	2.508955	0.30825	0.1496849	0.2289674	up
A_33_P3313635	lnc-BOLA2B-1	-0.991643	-0.873952	-0.651406	0.1176901	0.3402362	0.2289631	up
A_24_P101651	CSAG4	-2.025077	-1.484498	-2.107731	0.5405798	-0.082654	0.2289631	up
A_33_P3240773	CIDECP	-2.593961	-2.408264	-2.321949	0.1856964	0.2720122	0.2288543	up
A_33_P3329467	STYXL1	4.878584	5.0957713	5.119092	0.2171874	0.2405081	0.2288477	up
A_23_P253389	SLC4A2	0.9661646	1.3103828	1.0796318	0.3442183	0.1134672	0.2288427	up
A_32_P232035	LOC100270746	0.6151996	0.411366	1.2766962	-0.203834	0.6614966	0.2288315	up
A_21_P0014358	LOC100507520	-0.707626	-0.362542	-0.595087	0.3450842	0.1125388	0.2288115	up
A_24_P350649	PDPR	2.8729105	3.1214795	3.0819454	0.248569	0.2090349	0.228802	up
A_24_P348989	LILRA1	-2.385181	-1.896923	-2.415999	0.4882588	-0.030818	0.2287207	up
A_24_P401990	ERCC2	0.4055781	0.5565286	0.7120566	0.1509504	0.3064785	0.2287145	up
A_33_P3225843	CCDC149	-0.078032	0.4688253	-0.16748	0.5468574	-0.089448	0.2287045	up
A_24_P100234	MORC2	1.4816399	1.6494899	1.7710028	0.16785	0.2893629	0.2286065	up
A_23_P120227	LBH	1.7072191	1.9863772	1.8852634	0.2791581	0.1780443	0.2286012	up
A_22_P00002626	lnc-C1orf138-1	-1.283938	-1.008501	-1.102199	0.2754369	0.1817384	0.2285876	up
A_23_P202810	OVOL1	3.8784132	4.121856	4.0921326	0.243443	0.2137194	0.2285812	up
A_33_P3799936	ARHGEF10L	5.200285	5.4006896	5.4569902	0.2004046	0.2567053	0.228555	up
A_23_P99891	MESDC1	6.2451353	6.357075	6.5902414	0.1119399	0.3451061	0.228523	up
A_22_P00010050	ADNP-AS1	-0.798932	-0.369232	-0.771594	0.4296999	0.0273385	0.2285192	up
A_23_P315933	ABHD11	5.085492	5.425779	5.201975	0.3402867	0.1164827	0.2283847	up
A_33_P3259423	WDR55	3.2391539	3.4457278	3.4892607	0.206574	0.2501068	0.2283404	up
A_21_P0012309	DGCR5	0.6742029	0.8515048	0.9533839	0.1773019	0.279181	0.2282414	up
A_22_P00003760	lnc-CDIPT-1	-2.878455	-2.679559	-2.620966	0.1988959	0.2574894	0.2281927	up
A_32_P234738	RPL21	7.251525	7.4743667	7.484932	0.2228417	0.233407	0.2281244	up
A_33_P3289005	AP1S3	-0.010057	0.0961423	0.3399706	0.1061993	0.3500276	0.2281134	up
A_33_P3418766	ZNF532	1.63198	2.0632443	1.6569228	0.4312644	0.0249429	0.2281036	up
A_22_P00002697	lnc-C2-2	-1.62436	-1.268225	-1.524358	0.3561344	0.1000013	0.2280679	up
A_24_P304051	GSTO1	7.193948	7.3119903	7.5318527	0.1180425	0.3379049	0.2279737	up
A_22_P00001171	lnc-ANAPC11-2	1.0613399	1.203567	1.375042	0.1422272	0.3137021	0.2279646	up
A_33_P3287879	HIST1H3H	3.9726582	4.096803	4.3044233	0.124145	0.3317652	0.2279551	up
A_23_P24529	C11orf57	0.4901118	0.7404695	0.6956315	0.2503576	0.2055197	0.2279387	up
A_22_P00012866	DANCR	5.548044	5.7874084	5.764488	0.2393642	0.216444	0.2279041	up
A_32_P22501	EIF3J-AS1	0.9426465	1.1684179	1.1726465	0.2257714	0.23	0.2278857	up
A_23_P86021	SELENBP1	3.9392376	4.1947913	4.139435	0.2555537	0.2001972	0.2278755	up
A_21_P0006149	lnc-ANKRD20A1-2	0.533947	0.8579636	0.6656294	0.3240166	0.1316824	0.2278495	up
A_33_P3443165	RAE1	6.5970545	6.8269525	6.822852	0.229898	0.2257977	0.2278478	up
A_33_P3230594	LOC100507547	-0.892001	-0.728688	-0.599649	0.1633124	0.2923513	0.2278318	up
A_22_P00006547	lnc-FKBP2-1	4.803838	5.1647716	4.898265	0.3609338	0.0944271	0.2276804	up
A_21_P0013432	XLOC_l2_013863	-0.658847	-0.403739	-0.458651	0.2551079	0.2001963	0.2276521	up
A_22_P00005871	lnc-ESCO1-2	-1.072836	-0.757204	-0.93321	0.3156323	0.139626	0.2276292	up
A_32_P177097	ZNF557	2.2164364	2.4994407	2.3886461	0.2830043	0.1722097	0.227607	up
A_24_P82106	MMP14	-0.823463	-0.634859	-0.55689	0.1886039	0.266573	0.2275884	up
A_24_P338145	STOML2	3.9549599	4.235893	4.1290407	0.2809329	0.1740809	0.2275069	up
A_22_P00007712	HCG9	-0.266876	0.0363669	-0.11516	0.3032427	0.1517158	0.2274792	up
A_23_P206107	ULK3	0.898653	1.1655011	1.0866594	0.2668481	0.1880064	0.2274273	up
A_24_P318134	ZC3H10	-1.844587	-1.696783	-1.537581	0.1478047	0.3070064	0.2274056	up
A_23_P121945	SNCB	0.7002444	1.0294585	0.8257914	0.3292141	0.1255469	0.2273805	up
A_33_P3235975	CHCHD6	1.7618294	1.8587685	2.1195326	0.0969391	0.3577032	0.2273211	up
A_24_P361167	CHD8	3.291131	3.567594	3.4692974	0.276463	0.1781664	0.2273147	up
A_21_P0002446	lnc-USP34-1	-0.689213	-0.326537	-0.597436	0.3626761	0.0917764	0.2272263	up

A_33_P3248272	UBXN8	-0.840118	-0.496678	-0.729156	0.3434396	0.1109619	0.2272007	up
A_23_P141394	WIPI1	4.0702868	4.509957	4.0848684	0.4396701	0.0145817	0.2271259	up
A_22_P00006264	GAS6-AS1	1.3532844	1.7113118	1.4494977	0.3580275	0.0962133	0.2271204	up
A_22_P00013937	PDXK	-2.867521	-2.559665	-2.721155	0.3078556	0.1463659	0.2271107	up
A_23_P115375	HIST2H3D	7.1235905	7.323974	7.377345	0.2003837	0.2537546	0.2270691	up
A_24_P686965	SH2D5	1.0427604	1.3788052	1.1607866	0.3360448	0.1180263	0.2270355	up
A_33_P3279798	ATP5S	1.5459175	1.8191342	1.7267122	0.2732167	0.1807947	0.2270057	up
A_23_P26810	TP53	-0.151885	0.1269751	0.0232301	0.2788601	0.1751151	0.2269876	up
A_23_P487	UCK2	4.3454695	4.7124076	4.432458	0.3669381	0.0869885	0.2269633	up
A_21_P0003886	LOC101928237	-0.584693	-0.389273	-0.326192	0.1954203	0.2585015	0.2269609	up
A_33_P3590673	SNHG18	1.8570461	1.966392	2.20158	0.1093459	0.3445339	0.2269399	up
A_22_P00003260	NEURL1-AS1	-2.844693	-2.817076	-2.418446	0.027617	0.4262469	0.2269319	up
A_33_P3329255	NDST2	0.9194737	1.1100821	1.182723	0.1906085	0.2632494	0.226929	up
A_33_P3418025	CTSO	1.7716312	1.9626203	2.0344658	0.190989	0.2628346	0.2269118	up
A_33_P3379436	FAM74A4	9.647502	9.945351	9.803218	0.2978487	0.1557159	0.2267823	up
A_24_P274814	TBXAS1	-0.293563	0.0142779	-0.147855	0.3078413	0.1457086	0.2267749	up
A_23_P361469	METTL6	0.0449948	0.318584	0.2248778	0.2735891	0.179883	0.2267361	up
A_33_P3244347	PC	2.3392553	2.7252831	2.4066906	0.3860278	0.0674353	0.2267315	up
A_33_P3403927	SAMD10	2.2494574	2.3800464	2.5723286	0.130589	0.3228712	0.2267301	up
A_23_P77437	PRMT7	0.4104152	0.5923448	0.68191	0.1819296	0.2714949	0.2267122	up
A_23_P38497	SLC25A11	1.9167185	2.1393132	2.147521	0.2225947	0.2308025	0.2266986	up
A_33_P3217594	ZNF818P	-2.178126	-1.867338	-2.035548	0.3107877	0.1425781	0.2266829	up
A_23_P144778	CKMT2	-0.469652	-0.377915	-0.108261	0.0917363	0.3613911	0.2265637	up
A_22_P00001893	lnc-B4GALNT1-2	-0.925356	-0.703824	-0.693791	0.2215328	0.2315655	0.2265492	up
A_21_P0002276	lnc-INSIG2-3	-2.161238	-1.579724	-2.289656	0.5815139	-0.128417	0.2265482	up
A_23_P54179	ZNF410	3.4292593	3.6366277	3.6748734	0.2073684	0.2456141	0.2264912	up
A_32_P194821	RPL21	7.0073357	7.214043	7.2535744	0.2067075	0.2462387	0.2264731	up
A_33_P3384900	SNRPD2P2	1.2111287	1.2732091	1.6019583	0.0620804	0.3908296	0.226455	up
A_33_P3392977	CCNDBP1	2.324463	2.632918	2.4688988	0.308455	0.1444359	0.2264454	up
A_21_P0008550	lnc-BCL11B-1	-0.279898	-0.023253	-0.083793	0.2566443	0.196105	0.2263746	up
A_33_P3554318	TRIM3	-1.700227	-1.632336	-1.315466	0.0678911	0.3847609	0.226326	up
A_19_P00318261	SNORA26	2.4515877	2.627935	2.7277346	0.1763473	0.2761469	0.2262471	up
A_24_P627306	EDEM2	0.8662725	1.0163231	1.1686926	0.1500506	0.3024201	0.2262354	up
A_21_P0012074	XLOC_l2_008128	-2.28639	-2.17802	-1.942369	0.1083705	0.3440218	0.2261962	up
A_33_P3365392	LOC401557	-1.250629	-1.156219	-0.892676	0.0944104	0.3579531	0.2261817	up
A_22_P00003364	lnc-CBR1-1	-2.488066	-2.493505	-2.030281	-0.005439	0.4577856	0.2261735	up
A_23_P36562	ITGA5	0.8314652	1.140737	0.9744911	0.3092718	0.1430259	0.2261488	up
A_24_P209113	NDFIP1	1.986455	2.3420568	2.0830936	0.3556018	0.0966387	0.2261202	up
A_33_P3338335	MIDN	4.1869535	4.4905057	4.335491	0.3035522	0.1485376	0.2260449	up
A_21_P0009271	lnc-GPR142-1	-1.237512	-0.938674	-1.084264	0.2988381	0.1532474	0.2260428	up
A_33_P3351894	MIF-AS1	3.9375505	4.213181	4.113921	0.2756305	0.1763706	0.2260005	up
A_23_P62351	ARMCX6	2.305564	2.4066644	2.656396	0.1011004	0.350832	0.2259662	up
A_22_P00003672	CDC42SE1	4.3900833	4.7516675	4.4803877	0.3615842	0.0903044	0.2259443	up
A_22_P00003017	lnc-C7orf11-1	-1.686049	-1.380169	-1.540187	0.3058801	0.1458616	0.2258709	up
A_23_P148410	FTHL17	6.97713	7.5681844	6.837702	0.5910544	-0.139428	0.2258132	up
A_21_P0004638	HCG14	-1.103958	-0.891079	-0.865376	0.2128787	0.2385826	0.2257307	up
A_33_P3508717	LINC00847	0.6418858	0.82652	0.9085641	0.1846342	0.2666783	0.2256563	up
A_24_P330971	EIF3K	6.785862	6.9481463	7.074665	0.1622844	0.2888031	0.2255437	up
A_23_P218456	ILF3	5.5579195	5.952291	5.614538	0.3943715	0.0566187	0.2254951	up
A_33_P3292886	KRT6A	8.217495	8.279409	8.606497	0.0619144	0.3890019	0.2254581	up
A_23_P117928	BAHD1	3.8021612	4.0805	3.9747305	0.2783389	0.1725693	0.2254541	up
A_23_P72627	HGS	6.61088	6.8037477	6.8688984	0.1928678	0.2580185	0.2254431	up
A_21_P0000213	SNORD42B	-1.087565	-0.858859	-0.865406	0.2287054	0.2221589	0.2254322	up
A_23_P50349	TRIP10	1.2713447	1.4996228	1.4939055	0.2282782	0.2225609	0.2254195	up
A_33_P3421748	SON	2.3445396	2.5415978	2.5982847	0.1970582	0.2537451	0.2254016	up
A_23_P125815	RBM10	4.6216803	4.825047	4.8690014	0.2033668	0.2473211	0.2253439	up

A_22_P00017950	LOC100507461	-2.44089	-2.044912	-2.386357	0.3959775	0.054533	0.2252553	up
A_22_P00002574	lnc-C17orf97-4	0.9892659	0.9793482	1.4495754	-0.009918	0.4603095	0.2251959	up
A_23_P149200	CDC20	3.333784	3.516914	3.6010284	0.1831298	0.2672443	0.2251871	up
A_22_P00015391	THUMPD3-AS1	-1.404764	-1.187218	-1.171986	0.2175465	0.2327781	0.2251623	up
A_33_P3285299	GPRIN2	0.6346016	0.8099656	0.9094343	0.175364	0.2748327	0.2250984	up
A_21_P0010998	LOC283140	-0.636396	-0.539552	-0.283098	0.0968442	0.3532977	0.225071	up
A_33_P3376365	HES2	-0.267155	-0.165866	0.0815783	0.1012883	0.348733	0.2250106	up
A_33_P3299811	FLVCR1-AS1	-1.712584	-1.805419	-1.169757	-0.092836	0.5428262	0.2249952	up
A_33_P3280461	THRAP3	1.3991075	1.7589898	1.4891448	0.3598824	0.0900373	0.2249598	up
A_33_P3626709	LOC100129280	-3.012308	-2.82746	-2.747276	0.1848476	0.2650318	0.2249397	up
A_32_P205110	FOXC1	2.6257267	2.8071132	2.8941727	0.1813865	0.268446	0.2249162	up
A_33_P3383226	GP9	-1.428077	-1.230096	-1.176241	0.1979809	0.2518354	0.2249081	up
A_21_P0011968	XLOC_l2_008270	0.0305386	0.1583128	0.3524919	0.1277742	0.3219533	0.2248638	up
A_23_P164089	RFFL	2.998374	3.363727	3.0827103	0.3653531	0.0843363	0.2248447	up
A_24_P378788	SLC35E1	0.4149437	0.6766529	0.6028776	0.2617092	0.1879339	0.2248216	up
A_23_P60579	MPI	2.203949	2.5378356	2.3196812	0.3338866	0.1157322	0.2248094	up
A_23_P41818	ZFR	2.853857	3.1296535	3.0276003	0.2757964	0.1737433	0.2247698	up
A_33_P3417650	KLK10	0.452363	0.3027844	1.0514703	-0.149579	0.5991073	0.2247644	up
A_33_P3223097	LINC00467	1.939107	2.269527	2.0581884	0.33042	0.1190815	0.2247508	up
A_32_P101301	USP39	6.176096	6.278772	6.522827	0.1026759	0.3467312	0.2247036	up
A_23_P29067	TMPRSS2	-1.972235	-1.660572	-1.83452	0.3116632	0.1377149	0.224689	up
A_32_P106194	TMEM92	-0.910004	-0.801322	-0.569464	0.1086822	0.3405404	0.2246113	up
A_33_P3355090	CDKN2AIPNL	2.1257286	2.3498054	2.3508644	0.2240768	0.2251358	0.2246063	up
A_33_P3316539	SLC7A2	-1.054617	-0.712477	-0.947651	0.3421407	0.1069665	0.2245536	up
A_24_P80532	CCNG2	-2.072547	-1.817239	-1.878837	0.2553077	0.1937099	0.2245088	up
A_33_P3387045	CYB5D1	2.2901936	2.4466195	2.582776	0.156426	0.2925825	0.2245042	up
A_19_P00802550	lnc-C12orf75-1	-0.864696	-0.652562	-0.627864	0.2121339	0.2368321	0.224483	up
A_19_P00319476	lnc-LTBP3-2	-1.242074	-0.949579	-1.085633	0.2924948	0.1564412	0.224468	up
A_33_P3339687	ZNF669	-1.615575	-1.202143	-1.58009	0.4134316	0.0354853	0.2244585	up
A_32_P116271	WDFY3-AS2	-0.268128	0.3100705	-0.397494	0.5781984	-0.129366	0.2244163	up
A_33_P3386932	EEF2KMT	3.6900778	4.0332828	3.7956886	0.343205	0.1056109	0.2244079	up
A_23_P115124	TRIM11	1.807354	2.0181575	2.0451975	0.2108035	0.2378435	0.2243235	up
A_23_P63281	CPTP	0.9953318	1.2459712	1.1932907	0.2506394	0.197959	0.2242992	up
A_24_P9321	HIST1H3I	2.0706682	2.2981658	2.29175	0.2274976	0.2210817	0.2242897	up
A_23_P401098	TTC39C	-0.096421	0.2195106	0.0361681	0.3159318	0.1325893	0.2242606	up
A_23_P389692	KIAA1328	-2.824846	-2.18176	-3.019456	0.643086	-0.19461	0.2242378	up
A_32_P52816	TM7SF3	1.664567	1.9857183	1.7917643	0.3211513	0.1271973	0.2241743	up
A_22_P00017373	lnc-UTS2-1	0.3431644	0.696033	0.4386005	0.3528686	0.0954361	0.2241523	up
A_23_P74330	LINC00626	-1.131926	-0.799937	-1.015843	0.3319888	0.1160822	0.2240355	up
A_23_P115137	LAMTOR5	6.449766	6.581076	6.7663918	0.13131	0.3166256	0.2239678	up
A_23_P93032	ZBED3	3.0048695	3.3158822	3.1415815	0.3110127	0.1367121	0.2238624	up
A_24_P63262	RPN1	2.7950525	2.99326	3.0445576	0.1982074	0.249505	0.2238562	up
A_22_P00024437	LOC340581	-2.014243	-1.675925	-1.904975	0.3383174	0.1092672	0.2237923	up
A_23_P48550	CEP170B	5.5840044	5.8541255	5.761381	0.2701211	0.1773768	0.2237489	up
A_23_P208516	MBOAT7	5.145191	5.315147	5.4224863	0.1699557	0.2772951	0.2236254	up
A_33_P3324884	MICAL1	-1.559104	-1.516885	-1.154255	0.0422196	0.4048491	0.2235343	up
A_23_P383118	ZSWIM5	-0.007827	0.3197703	0.1115723	0.3275976	0.1193996	0.2234986	up
A_23_P16096	PPAN	1.3070331	1.4413791	1.6195621	0.134346	0.3125291	0.2234376	up
A_23_P159833	NDUFA1	7.241358	7.5473104	7.3821898	0.3059526	0.140832	0.2233923	up
A_33_P3325262	SLC6A8	3.4886436	3.8463387	3.577713	0.3576951	0.0890694	0.2233822	up
A_23_P30736	HLA-DOB	-0.32892	-0.183362	-0.027737	0.1455579	0.3011832	0.2233706	up
A_24_P13311	SLC15A4	1.0009508	1.2152214	1.2333779	0.2142706	0.2324271	0.2233489	up
A_33_P3305458	ATF6B	1.110529	1.444377	1.2233539	0.333848	0.1128249	0.2233365	up
A_21_P0010315	lnc-ADARB1-1	-2.175198	-1.947519	-1.956432	0.2276788	0.2187657	0.2232223	up
A_22_P00014870	LOC574538	-1.1416	-0.940626	-0.896269	0.200974	0.2453308	0.2231524	up
A_32_P18250	FUNDC2P2	2.4120836	2.7812304	2.4891958	0.3691468	0.0771122	0.2231295	up

A_23_P211493	TMPRSS6	-2.425324	-2.097355	-2.307052	0.3279696	0.1182728	0.2231212	up
A_23_P74229	STK40	4.828583	5.2238574	4.879484	0.3952746	0.0509014	0.223088	up
A_23_P150064	MMRN2	-1.400635	-1.140969	-1.2142	0.2596664	0.1864352	0.2230508	up
A_24_P370471	ATG10	-0.77922	-0.530307	-0.582212	0.2489123	0.1970081	0.2229602	up
A_23_P20732	GTF3C4	3.2831173	3.5055375	3.5065994	0.2224202	0.2234821	0.2229512	up
A_22_P00022557	lnc-FITM2-1	-0.423576	-0.414344	0.0129442	0.009232	0.4365206	0.2228763	up
A_23_P502371	PHF2	3.0558882	3.4449935	3.11242	0.3891053	0.0565319	0.2228186	up
A_23_P112241	DNAJB5	-0.204844	0.2847939	-0.248934	0.4896383	-0.044089	0.2227745	up
A_19_P00317753	LOC101929563	-2.134897	-1.809979	-2.014285	0.3249183	0.1206126	0.2227654	up
A_33_P3378531	AS3MT	9.406382	9.681372	9.576898	0.2749901	0.170516	0.222753	up
A_24_P398972	COQ7	1.3264194	1.3726583	1.725637	0.0462389	0.3992176	0.2227283	up
A_23_P717	TMEM206	0.9661336	1.108912	1.2687788	0.1427784	0.3026452	0.2227118	up
A_23_P19691	HEBP2	5.644882	5.8736978	5.8614473	0.2288156	0.2165651	0.2226903	up
A_23_P25935	C14orf1	3.0158281	3.0279803	3.448969	0.0121522	0.4331408	0.2226465	up
A_21_P0011764	XLOC_l2_007135	-2.664463	-2.357402	-2.526265	0.3070607	0.1381979	0.2226293	up
A_22_P00014979	lnc-SMARCC2-5	1.2348332	1.431766	1.4830523	0.1969328	0.248219	0.2225759	up
A_24_P417189	DUSP9	-0.699338	-0.429196	-0.524438	0.2701421	0.1749001	0.2225211	up
A_32_P465742	PIP5K1B	-1.736799	-1.38854	-1.640171	0.3482585	0.0966282	0.2224433	up
A_24_P363477	FTSJ2	0.7954745	0.9246993	1.1109853	0.1292248	0.3155108	0.2223678	up
A_22_P00005499	lnc-DZANK1-2	-2.514923	-2.074727	-2.510394	0.4401968	0.004529	0.2223629	up
A_23_P425587	MED11	0.9006763	1.1717129	1.0741882	0.2710366	0.173512	0.2222743	up
A_23_P332374	ZNF175	-0.925347	-0.662326	-0.743834	0.263021	0.1815138	0.2222674	up
A_23_P128554	EBPL	6.6331205	6.8527074	6.858021	0.2195869	0.2249003	0.2222436	up
A_23_P53288	CNPY2	7.022954	7.3657517	7.1244802	0.3427978	0.1015263	0.222162	up
A_32_P150856	LOC407835	3.2377472	3.5893807	3.3304214	0.3516336	0.0926743	0.2221539	up
A_23_P429998	FOSB	-1.151655	-0.757104	-1.101929	0.3945508	0.049726	0.2221384	up
A_23_P64770	DDX23	5.1384563	5.2829967	5.4380465	0.1445403	0.2995901	0.2220652	up
A_32_P50924	HNRNPA1L2	6.531744	6.8094554	6.697912	0.2777114	0.1661682	0.2219398	up
A_23_P88873	GAN	1.6786127	2.0729861	1.7281108	0.3943734	0.0494981	0.2219358	up
A_33_P3388646	ABLIM1	0.1675453	0.5971942	0.1816812	0.4296489	0.0141358	0.2218924	up
A_23_P126089	SPRR2C	0.3735151	0.9732904	0.2173429	0.5997753	-0.156172	0.2218015	up
A_21_P0011774	TMEM221	-3.18464	-2.887537	-3.038513	0.2971027	0.1461265	0.2216146	up
A_33_P3370763	SARM1	-3.552167	-2.975282	-3.685835	0.576885	-0.133667	0.2216088	up
A_33_P3240674	BRD3	1.6549406	2.0119147	1.7410922	0.3569741	0.0861516	0.2215629	up
A_22_P00002553	SNORA76C	8.316117	8.505236	8.570097	0.1891184	0.2539797	0.221549	up
A_33_P3342295	VSX1	6.356991	6.724813	6.4322624	0.3678222	0.0752716	0.2215469	up
A_33_P3359543	ZNF561	3.016923	3.4519782	3.0246992	0.4350553	0.0077763	0.2214158	up
A_23_P139547	TUBA1A	5.820919	5.961493	6.123147	0.140574	0.302228	0.221401	up
A_33_P3246804	STARD3	7.8620214	8.202726	7.9641047	0.3407049	0.1020832	0.2213941	up
A_23_P17512	DTD1	5.2762623	5.4508433	5.54443	0.1745811	0.2681675	0.2213743	up
A_33_P3272553	NCAPH2	0.895061	1.0547643	1.1780677	0.1597033	0.2830067	0.221355	up
A_19_P00811178	MIR503HG	-2.528789	-2.443946	-2.171027	0.0848434	0.3577626	0.221303	up
A_23_P71904	NTMT1	3.4985685	3.554687	3.8850489	0.0561185	0.3864803	0.2212994	up
A_33_P3295009	lnc-AEBP1-1	-1.480336	-1.073286	-1.444791	0.4070506	0.0355454	0.221298	up
A_33_P3222367	SNORA74B	0.1287441	0.2003493	0.4996071	0.0716052	0.370863	0.2212341	up
A_33_P3400217	SLC4A1	-1.377791	-1.120165	-1.193048	0.2576265	0.1847439	0.2211852	up
A_33_P3239338	DGKZ	5.854929	6.0475936	6.104375	0.1926646	0.2494459	0.2210553	up
A_22_P00002994	lnc-C6orf221-1	-1.886086	-1.85898	-1.471105	0.0271053	0.4149804	0.2210429	up
A_22_P00013430	BTG1	4.1101227	4.6988482	3.96348	0.5887256	-0.146643	0.2210414	up
A_23_P154199	PPP1R7	4.6241674	4.816151	4.8739376	0.1919837	0.2497702	0.2208769	up
A_23_P207650	ACADVL	5.784173	6.1192145	5.89087	0.3350415	0.1066971	0.2208693	up
A_24_P151727	NONO	4.905178	5.1543927	5.097685	0.2492147	0.1925068	0.2208607	up
A_22_P00008438	lnc-KAT8-1	-2.587041	-2.208464	-2.523998	0.378577	0.0630434	0.2208102	up
A_22_P00018568	lnc-ST7-1	0.5981746	0.8714604	0.7665057	0.2732859	0.1683312	0.2208085	up
A_33_P3258699	DUX4	-0.996386	-0.853262	-0.697963	0.1431236	0.2984233	0.2207735	up
A_33_P3335940	HN1L	1.6950579	1.7819414	2.0496664	0.0868835	0.3546085	0.220746	up

A_23_P93704	C7orf61	-1.643333	-1.679716	-1.165462	-0.036382	0.4778719	0.2207449	up
A_23_P16252	KLK1	2.4199753	2.5967288	2.6847038	0.1767535	0.2647286	0.220741	up
A_23_P387045	CCDC107	2.1393461	2.3431306	2.377019	0.2037845	0.2376728	0.2207286	up
A_23_P500364	BCL7B	2.2345295	2.491445	2.4190245	0.2569156	0.184495	0.2207053	up
A_24_P391586	OAF	2.0631485	2.297935	2.2697353	0.2347865	0.2065868	0.2206867	up
A_23_P117363	SERPINA6	-1.296239	-1.107896	-1.043479	0.1883435	0.2527609	0.2205522	up
A_19_P00809139	LOC101929709	-3.019593	-2.515803	-3.082306	0.5037894	-0.062713	0.2205381	up
A_23_P85604	WDR26	3.1660385	3.4417682	3.3313227	0.2757297	0.1652842	0.2205069	up
A_23_P35970	SLC37A4	1.8939896	1.9702296	2.258525	0.0762401	0.3645353	0.2203877	up
A_24_P290927	UBAP2L	0.9106913	1.1192908	1.1428027	0.2085996	0.2321115	0.2203555	up
A_23_P500333	SSBP3	-1.918708	-1.454858	-1.94197	0.46385	-0.023262	0.220294	up
A_24_P146892	ORAI1	2.7942429	3.0137467	3.0152626	0.2195039	0.2210197	0.2202618	up
A_23_P339773	TPRG1L	2.0135174	2.2557979	2.2117367	0.2422805	0.1982193	0.2202499	up
A_21_P0000275	SNORD96A	0.0572357	0.4481669	0.1067925	0.3909311	0.0495567	0.2202439	up
A_22_P00002774	OGFRP1	-2.405451	-2.186516	-2.183924	0.218935	0.2215273	0.2202312	up
A_23_P415411	HIST1H4E	5.701207	5.950889	5.8919077	0.249682	0.1907005	0.2201912	up
A_23_P211244	PRMT2	0.2960482	0.6633549	0.3689871	0.3673067	0.0729389	0.2201228	up
A_22_P00004694	RECQL4	-0.872955	-0.586753	-0.718969	0.2862015	0.153986	0.2200937	up
A_23_P362824	CSTF1	4.233615	4.439135	4.468213	0.2055202	0.2345982	0.2200592	up
A_23_P147826	RAP2C	-1.076911	-0.922818	-0.790914	0.1540933	0.2859969	0.2200451	up
A_23_P159539	ASMTL	3.3709517	3.5422997	3.639637	0.1713481	0.2686853	0.2200167	up
A_23_P316460	ZBED6CL	-0.77436	-0.518203	-0.59049	0.2561574	0.1838703	0.2200139	up
A_23_P11843	LRRN2	0.3273249	0.5325093	0.5621109	0.2051845	0.234786	0.2199852	up
A_33_P3223592	APOE	2.245861	2.602181	2.329259	0.3563199	0.0833979	0.2198589	up
A_33_P3423285	lnc-SRGAP3-1	-2.078181	-1.800588	-1.916154	0.2775936	0.1620274	0.2198105	up
A_21_P0010749	LOC100288069	-2.634007	-2.411719	-2.41674	0.2222877	0.217267	0.2197773	up
A_33_P3270863	XDH	0.306695	0.8730116	0.1798606	0.5663166	-0.126834	0.2197411	up
A_23_P12884	GRK5	-0.105865	0.1367364	0.090992	0.2426009	0.1968565	0.2197287	up
A_23_P167856	TMEM63B	0.4596024	0.7040558	0.6545863	0.2444534	0.194984	0.2197187	up
A_33_P3391603	LAMA4	-2.591733	-2.480812	-2.263234	0.1109211	0.3284993	0.2197102	up
A_22_P00022141	lnc-TLCD2-2	1.2320404	1.4424734	1.4609566	0.210433	0.2289162	0.2196746	up
A_23_P8900	COX6C	7.880102	8.034761	8.164512	0.1546593	0.2844095	0.2195344	up
A_23_P60517	FXN	3.4420156	3.674848	3.6481953	0.2328324	0.2061796	0.219506	up
A_22_P00015791	lnc-TARBP2-1	2.5540066	2.7277246	2.8192854	0.173718	0.2652788	0.2194984	up
A_33_P3281686	TSC2	-1.883978	-1.496081	-1.83299	0.387897	0.0509882	0.2194426	up
A_21_P0004456	LOC102546294	-3.12247	-2.430784	-3.375344	0.6916866	-0.252874	0.2194064	up
A_23_P106633	DDX28	3.9863281	4.291041	4.120427	0.3047128	0.134099	0.2194059	up
A_22_P00000494	HLX-AS1	1.4203076	1.5670509	1.7123413	0.1467433	0.2920337	0.2193885	up
A_22_P00018276	LOC101928733	-2.039105	-1.624413	-2.015246	0.4146919	0.0238585	0.2192752	up
A_22_P00008946	lnc-KRT83-1	0.7980042	1.1309214	0.9033108	0.3329172	0.1053066	0.2191119	up
A_33_P3378659	TARP	-0.325171	-0.162071	-0.050062	0.1631002	0.2751093	0.2191048	up
A_33_P3341189	UBE2A	4.2262115	4.4576344	4.432887	0.2314229	0.2066755	0.2190492	up
A_23_P20035	GPR146	-1.756067	-0.92898	-2.145073	0.8270869	-0.389006	0.2190406	up
A_24_P72394	RBAK	-3.106442	-2.887945	-2.886866	0.2184975	0.2195761	0.2190368	up
A_23_P71644	FANCG	4.306095	4.578077	4.472061	0.2719817	0.165966	0.2189739	up
A_33_P3410589	FAM43A	1.640789	2.0435271	1.6759768	0.4027381	0.0351877	0.2189629	up
A_23_P155890	NAA11	1.9904633	2.1109252	2.3078423	0.1204619	0.317379	0.2189205	up
A_23_P316531	GPR25	-0.814197	-0.580126	-0.610683	0.2340703	0.2035141	0.2187922	up
A_32_P82623	AGBL3	-1.337668	-1.059907	-1.17799	0.277761	0.159678	0.2187195	up
A_33_P3358885	LOC100130913	-2.966567	-2.637888	-2.858009	0.3286796	0.1085582	0.2186189	up
A_23_P166686	AMOTL2	5.0490007	5.339765	5.1954126	0.2907643	0.1464119	0.2185881	up
A_23_P55251	ITGA3	3.4766226	3.5416675	3.84875	0.0650449	0.3721275	0.2185862	up
A_22_P00000450	LOC102724330	-3.108232	-2.904762	-2.874595	0.2034702	0.2336369	0.2185535	up
A_23_P346384	MRPL43	-2.501927	-2.599936	-1.966819	-0.098009	0.5351074	0.218549	up
A_21_P0011327	EWSAT1	-2.606056	-2.475489	-2.299553	0.1305673	0.3065035	0.2185354	up
A_22_P00024534	lnc-UBB-2	-2.269715	-2.14583	-1.9566	0.1238849	0.3131146	0.2184998	up

A_21_P0010599	XLOC_l2_000791	0.0355358	0.3771977	0.1307435	0.3416619	0.0952077	0.2184348	up
A_23_P148821	DPH5	2.4593325	2.6722417	2.6832247	0.2129092	0.2238922	0.2184007	up
A_23_P345710	XXYLT1	0.9572353	1.1042409	1.2470031	0.1470056	0.2897677	0.2183867	up
A_21_P0004562	lnc-ANKRA2-3	-1.93765	-1.802971	-1.635863	0.1346788	0.3017869	0.2182329	up
A_23_P115246	FCN3	-3.176978	-2.749982	-3.1676	0.426996	0.0093775	0.2181867	up
A_23_P159920	IKBKG	-0.031044	0.2128735	0.1614127	0.243917	0.1924563	0.2181866	up
A_33_P3273552	KRT83	4.761717	5.0365186	4.9232035	0.2748017	0.1614866	0.2181442	up
A_33_P3275968	SEL1L	0.769762	1.344605	0.6312075	0.5748429	-0.138555	0.2181442	up
A_23_P40295	LAMP5	5.5930233	5.47249	6.1497374	-0.120533	0.5567141	0.2180903	up
A_23_P101297	EML2	1.1534939	1.2375479	1.5055895	0.084054	0.3520956	0.2180748	up
A_23_P36397	CYP27B1	0.2180843	0.4980679	0.374218	0.2799835	0.1561337	0.2180586	up
A_33_P3407937	PLCXD1	0.3445878	0.4714618	0.6537366	0.126874	0.3091488	0.2180114	up
A_24_P8768	MRPL20	6.2507696	6.4694133	6.4681063	0.2186437	0.2173367	0.2179902	up
A_24_P202558	SIPA1L3	-0.351931	-0.289785	0.021893	0.0621457	0.3738241	0.2179849	up
A_23_P47497	MRPL16	5.7985067	5.890734	6.142173	0.0922275	0.3436661	0.2179468	up
A_24_P338788	CSNK1A1L	0.6450915	0.85289	0.873179	0.2077985	0.2280874	0.217943	up
A_22_P00000473	lnc-AC092295.7.1-1	-1.673105	-1.419062	-1.491415	0.2540426	0.1816897	0.2178662	up
A_23_P94095	ANKRD46	1.9639826	2.0225825	2.3410616	0.0586	0.377079	0.2178395	up
A_23_P406135	IFT172	3.4486046	3.617167	3.715642	0.1685624	0.2670374	0.2177999	up
A_23_P148194	ADI1	4.935197	5.0674443	5.2384243	0.1322475	0.3032274	0.2177374	up
A_23_P211814	MAP4	2.3692265	2.636105	2.5377712	0.2668786	0.1685448	0.2177117	up
A_22_P00015897	SNHG6	4.16115	4.4501653	4.3075466	0.2890153	0.1463966	0.217706	up
A_23_P161481	PALD1	0.8126731	0.8651066	1.1955094	0.0524335	0.3828363	0.2176349	up
A_33_P3225567	TRMT2B	1.9262857	2.2377257	2.050108	0.31144	0.1238222	0.2176311	up
A_23_P202427	HKDC1	2.8805933	3.1599336	3.036498	0.2793403	0.1559048	0.2176225	up
A_32_P42925	MYL12B	8.177344	8.382347	8.407577	0.2050028	0.2302322	0.2176175	up
A_23_P310331	RANBP3	3.600811	3.7008262	3.9359713	0.1000152	0.3351603	0.2175877	up
A_23_P86182	MRPS21	6.538889	6.720241	6.7926893	0.1813521	0.2538004	0.2175763	up
A_24_P941824	KIF3B	3.2930603	3.6067023	3.414507	0.313642	0.1214466	0.2175443	up
A_24_P100517	SAPCD2	4.52602	4.78379	4.703124	0.2577701	0.177104	0.217437	up
A_33_P3280531	CRAT	3.5259838	3.901713	3.585085	0.3757291	0.0591011	0.2174151	up
A_19_P00325810	KHDC1	0.1905937	0.4336858	0.3823004	0.2430921	0.1917067	0.2173994	up
A_23_P77000	VASH1	0.0767832	0.0757341	0.5125232	-0.001049	0.43574	0.2173455	up
A_23_P27947	PDCD2L	4.5974855	4.8407474	4.788909	0.2432618	0.1914234	0.2173426	up
A_33_P3255131	KCTD19	0.0605545	0.2544842	0.3012719	0.1939297	0.2407174	0.2173235	up
A_33_P3398897	QDPR	2.6625166	2.7406058	3.019024	0.0780892	0.3565073	0.2172983	up
A_33_P3264731	BTNL10	-3.223384	-3.131395	-2.880928	0.091989	0.3424556	0.2172223	up
A_33_P3347937	HSF1	-1.696877	-1.397385	-1.562058	0.2994919	0.1348195	0.2171557	up
A_33_P3274696	SLC52A2	7.6332884	7.787313	7.9135733	0.1540246	0.2802849	0.2171547	up
A_23_P33433	MAZ	0.6848507	0.9631915	0.8404574	0.2783408	0.1556068	0.2169738	up
A_33_P3562537	RET	-2.255448	-1.841703	-2.235311	0.4137459	0.0201378	0.2169418	up
A_24_P243396	TCOF1	-0.483275	-0.460354	-0.072339	0.0229216	0.4109368	0.2169292	up
A_33_P3267640	HGFAC	-0.814366	-0.564316	-0.630694	0.2500501	0.183672	0.216861	up
A_22_P00014836	LOC101927963	-3.059885	-2.638535	-3.04755	0.4213498	0.0123353	0.2168425	up
A_32_P44316	EEF1A1	6.6922855	7.118774	6.699379	0.4264884	0.0070934	0.2167909	up
A_24_P107897	TXNDC11	2.2325487	2.627708	2.2709303	0.3951592	0.0383816	0.2167704	up
A_21_P0013100	XLOC_l2_013131	1.4063067	1.728107	1.5180235	0.3218002	0.1117168	0.2167585	up
A_21_P0014306	CAPN15	3.8502045	4.081055	4.0528326	0.2308507	0.2026281	0.2167394	up
A_33_P3410459	SCARB2	6.3193893	6.5210605	6.5511045	0.2016711	0.2317152	0.2166932	up
A_23_P259413	CDV3	3.6660995	3.9725108	3.793067	0.3064113	0.1269674	0.2166894	up
A_33_P3417313	PYCRL	-1.169909	-0.903032	-1.003516	0.2668767	0.1663928	0.2166348	up
A_33_P3272479	NPM2	-1.902239	-1.393338	-1.978065	0.5089006	-0.075826	0.2165375	up
A_23_P70998	UMAD1	1.9844599	2.215797	2.1860752	0.2313371	0.2016153	0.2164762	up
A_33_P3242234	F8A2	2.7447224	2.9876332	2.9346638	0.2429109	0.1899414	0.2164261	up
A_23_P314120	CHKB	1.0405893	1.2635264	1.2504067	0.2229371	0.2098174	0.2163773	up

A_23_P215088	ZC3HC1	1.5973854	1.9717355	1.6556811	0.3743501	0.0582957	0.2163229	up
A_23_P79703	HADHB	5.656068	5.7955203	5.949255	0.1394525	0.2931871	0.2163198	up
A_24_P168416	PRDX2	3.5059185	3.6631904	3.7812843	0.1572719	0.2753658	0.2163188	up
A_33_P3294868	LINC00086	-2.059174	-1.838968	-1.846915	0.2202058	0.2122593	0.2162325	up
A_21_P0010519	MST1	-1.108053	-0.948895	-0.834768	0.1591578	0.2732854	0.2162216	up
A_24_P168510	KXD1	2.471074	2.672791	2.701601	0.2017169	0.2305269	0.2161219	up
A_23_P201319	DISP1	0.7437706	1.0877056	0.8319974	0.343935	0.0882268	0.2160809	up
A_23_P65963	BFAR	3.3666363	3.6054654	3.559866	0.2388291	0.1932297	0.2160294	up
A_24_P287691	AP3S2	3.581975	3.7372975	3.8585482	0.1553226	0.2765732	0.2159479	up
A_22_P00020106	lnc-SMG1-2	0.5656633	0.639391	0.9238257	0.0737276	0.3581624	0.215945	up
A_23_P380857	APOL4	-2.098673	-1.868136	-1.897327	0.2305374	0.2013459	0.2159417	up
A_22_P00007292	lnc-GPR27-1	0.5604425	0.7299104	0.8228512	0.1694679	0.2624087	0.2159383	up
A_23_P29303	RRP7A	1.4726596	1.7232285	1.6539669	0.2505689	0.1813073	0.2159381	up
A_21_P0014885	LOC100506257	0.1267967	0.5099025	0.175561	0.3831058	0.0487642	0.215935	up
A_23_P306479	ZBTB11-AS1	-2.051603	-1.716154	-1.955204	0.3354497	0.0963993	0.2159245	up
A_33_P3347037	FAM226A	-3.142089	-2.7959	-3.056437	0.3461888	0.0856516	0.2159202	up
A_21_P0013795	RPL36A	9.729985	9.992163	9.899647	0.2621775	0.1696615	0.2159195	up
A_33_P3244274	RNF208	2.1106558	2.4501586	2.202858	0.3395028	0.0922022	0.2158525	up
A_23_P316741	TSPAN4	1.3337107	1.7496538	1.3494024	0.4159432	0.0156918	0.2158175	up
A_23_P14986	HSD11B2	2.449545	2.774365	2.5563545	0.32482	0.1068096	0.2158148	up
A_33_P3386547	SGPP2	3.1416578	3.5817132	3.1330757	0.4400554	-0.008582	0.2157366	up
A_33_P3370364	PRLHR	0.3180203	0.5971499	0.4700761	0.2791295	0.1520557	0.2155926	up
A_23_P10785	VTI1A	1.6285725	1.834847	1.8534546	0.2062745	0.2248821	0.2155783	up
A_33_P3363082	SCARNA5	0.078352	0.3081546	0.2796831	0.2298026	0.2013311	0.2155669	up
A_33_P3632937	LOC100131262	4.83434	5.04444	5.0553656	0.2100997	0.2210255	0.2155626	up
A_23_P67198	CPAMD8	-3.032561	-2.505961	-3.128085	0.5266008	-0.095523	0.2155387	up
A_23_P109547	MIEF1	2.0382347	2.0926938	2.4147854	0.0544591	0.3765507	0.2155049	up
A_33_P3392740	ATOH8	-2.642932	-2.306024	-2.548883	0.3369079	0.0940492	0.2154785	up
A_24_P273865	MED20	1.6857004	1.9523015	1.8500376	0.2666011	0.1643372	0.2154691	up
A_23_P39364	HOMER3	5.6026955	5.799891	5.836385	0.1971955	0.2336893	0.2154424	up
A_23_P86570	ANXA7	2.7371159	2.9212584	2.983778	0.1841426	0.2466621	0.2154024	up
A_23_P99163	DRAM1	2.836113	3.0979075	3.0050755	0.2617946	0.1689625	0.2153785	up
A_19_P00321577	DLEU1	-0.680286	-0.473505	-0.456355	0.2067809	0.2239308	0.2153559	up
A_33_P3395876	PDE4A	-0.992981	-0.850822	-0.704467	0.142159	0.2885141	0.2153366	up
A_33_P3209895	FAM58A	1.7153969	1.7983098	2.0629435	0.0829129	0.3475466	0.2152298	up
A_23_P32223	CIZ1	3.110054	3.316255	3.3342838	0.2062011	0.2242298	0.2152154	up
A_22_P00019851	lnc-ATP6AP1L-2	-3.219982	-2.877644	-3.131938	0.3423379	0.0880444	0.2151911	up
A_33_P3231981	PARK7	6.2912035	6.5433955	6.469393	0.252192	0.1781893	0.2151907	up
A_24_P382579	OXT	-1.007091	-0.644175	-0.939638	0.3629165	0.0674529	0.2151847	up
A_33_P3408305	CERS3	-0.052421	0.2516518	0.0737758	0.3040729	0.1261969	0.2151349	up
A_32_P191860	SCGB2B2	-1.847321	-1.634938	-1.629435	0.2123833	0.217886	0.2151346	up
A_32_P212373	lnc-EGLN1-1	4.062887	4.3420396	4.213996	0.2791524	0.1511087	0.2151306	up
A_33_P3249185	SAR1A	4.1288433	4.4416814	4.2460785	0.3128381	0.1172352	0.2150366	up
A_33_P3246733	MNT	-1.753931	-1.458992	-1.618863	0.2949395	0.1350684	0.215004	up
A_23_P111452	AGAP3	4.367732	4.6008115	4.5642023	0.2330794	0.1964703	0.2147748	up
A_33_P3386062	LOC283585	-2.208102	-2.111681	-1.874977	0.096421	0.3331251	0.2147731	up
A_21_P0010485	HCG9	0.1620178	0.4829149	0.2706485	0.3208971	0.1086307	0.2147639	up
A_22_P00019065	lnc-RP11-1105G2.3.1-3	-2.279622	-2.104415	-2.025417	0.1752069	0.2542048	0.2147058	up
A_24_P233850	SDHC	1.3545265	1.4904652	1.6477914	0.1359386	0.2932649	0.2146018	up
A_32_P46214	SLC9A9	-2.709394	-3.020832	-1.968917	-0.311439	0.7404764	0.2145189	up
A_21_P0000146	PDK2	-0.059102	0.1110959	0.199564	0.1701975	0.2586656	0.2144315	up
A_23_P153583	PPP5C	1.4559064	1.5933919	1.7472401	0.1374855	0.2913337	0.2144096	up
A_23_P18276	RBM5	3.9095325	4.27587	3.9719372	0.3663373	0.0624046	0.214371	up
A_22_P00008973	lnc-LALBA-1	-1.114157	-0.861323	-0.938514	0.2528338	0.175643	0.2142384	up
A_23_P26954	VAT1	5.4035597	5.795219	5.440358	0.3916593	0.0367985	0.2142289	up

A_24_P860703	SNHG17	3.7237997	3.7877936	4.088249	0.0639939	0.3644495	0.2142217	up
A_23_P83781	CYTH1	4.4459763	4.7285533	4.5916767	0.282577	0.1457005	0.2141387	up
A_23_P127652	ZNF202	-0.157796	0.0339117	0.0786872	0.1917081	0.2364836	0.2140958	up
A_33_P3318841	C5orf63	-0.247643	-0.472637	0.4054184	-0.224994	0.6530609	0.2140334	up
A_23_P316612	GLIS1	-3.196837	-3.134148	-2.831483	0.0626891	0.3653548	0.2140219	up
A_23_P168062	DHX16	1.5320992	1.7091322	1.783104	0.177033	0.2510047	0.2140188	up
A_23_P51231	RUNX3	2.4604626	2.6851497	2.6637564	0.2246871	0.2032938	0.2139905	up
A_23_P7976	HIST1H1E	4.3684216	4.542437	4.622346	0.1740155	0.2539244	0.2139699	up
A_21_P0011848	LOC100506922	1.2976084	1.5014863	1.5215182	0.2038779	0.2239099	0.2138939	up
A_24_P118231	ATP5S	2.0321922	2.3456035	2.146408	0.3134112	0.1142159	0.2138135	up
A_33_P3813128	SEMA3F	1.8755207	2.1480527	2.0305262	0.272532	0.1550055	0.2137687	up
A_33_P3381265	CYB5R2	0.9601889	1.3278885	1.0199528	0.3676996	0.0597639	0.2137318	up
A_23_P93180	HIST1H2BC	2.3797836	2.5943437	2.5926666	0.21456	0.212883	0.2137215	up
A_21_P0009406	lnc-NR1D1-1	-0.608894	-0.211226	-0.579503	0.3976679	0.0293913	0.2135296	up
A_24_P410086	SSBP4	3.7772322	3.7614388	4.2199106	-0.015793	0.4426785	0.2134426	up
A_33_P3399248	UFM1	4.2052193	4.3853736	4.4517136	0.1801543	0.2464943	0.2133243	up
A_23_P55190	EFTUD2	6.010849	6.1999736	6.248296	0.1891246	0.2374468	0.2132857	up
A_22_P00004692	lnc-CTD-2517M22.14.1-1	-1.84709	-1.437717	-1.829978	0.4093728	0.0171123	0.2132425	up
A_21_P0002963	lnc-KCTD6-1	-3.325679	-2.947204	-3.277779	0.378475	0.0479004	0.2131877	up
A_24_P33895	ATF3	-0.708014	0.1848903	-1.17456	0.8929043	-0.466546	0.2131791	up
A_32_P4018	ROR1	1.6091061	1.9025741	1.7419176	0.293468	0.1328116	0.2131398	up
A_23_P52738	DCPS	4.427928	4.600862	4.681261	0.1729341	0.2533331	0.2131336	up
A_23_P201863	CDK18	0.0784259	0.237771	0.3452883	0.1593452	0.2668624	0.2131038	up
A_23_P122724	VNN2	-1.857752	-1.367853	-1.921504	0.4898992	-0.063752	0.2130735	up
A_33_P3283601	LOC389033	0.3994565	0.615303	0.609756	0.2158465	0.2102995	0.213073	up
A_23_P26439	DBNDD1	1.1437793	1.4698629	1.2438102	0.3260837	0.1000309	0.2130573	up
A_21_P0012079	MIR4435-1HG	6.8768635	7.049109	7.130625	0.1722455	0.2537613	0.2130034	up
A_23_P254888	ZYX	1.6393538	1.9755731	1.7291074	0.3362193	0.0897536	0.2129865	up
A_23_P111621	GTF2IRD1	4.607915	4.6874347	4.954196	0.0795198	0.3462811	0.2129004	up
A_32_P118250	DHRS4-AS1	-2.083493	-1.930123	-1.811099	0.1533699	0.2723937	0.2128818	up
A_23_P58953	NQO2	1.0584335	1.5887551	0.9538112	0.5303216	-0.104622	0.2128496	up
A_24_P255874	HCG4B	-1.333838	-1.151751	-1.090366	0.1820865	0.2434712	0.2127788	up
A_33_P3522525	GRPEL1	3.2740383	3.3140206	3.6595821	0.0399823	0.3855438	0.2127631	up
A_22_P00023131	lnc-FANCI-1	-2.184915	-2.066227	-1.878181	0.1186886	0.3067346	0.2127116	up
A_24_P497186	IRF2BP2	1.2806954	1.5206585	1.4659753	0.2399631	0.1852799	0.2126215	up
A_23_P100660	SERPINF1	-3.016994	-2.773643	-2.835172	0.2433505	0.1818218	0.2125862	up
A_24_P602507	HYKK	-0.439112	-0.287572	-0.165555	0.1515398	0.2735572	0.2125485	up
A_22_P00005942	lnc-EXOC3L4-1	-2.487009	-2.481924	-2.067172	0.0050855	0.4198377	0.2124616	up
A_23_P157051	AP4M1	0.3842726	0.5636835	0.6296191	0.1794109	0.2453466	0.2123787	up
A_23_P427114	GORASP1	-2.822336	-2.785733	-2.43428	0.036603	0.3880556	0.2123293	up
A_33_P3385148	C1orf186	2.6473598	2.8913493	2.8279915	0.2439895	0.1806316	0.2123106	up
A_23_P350574	FCRLB	1.2123113	1.5827208	1.266439	0.3704095	0.0541277	0.2122686	up
A_22_P00004393	MAL2	4.9961147	5.2926745	5.1239643	0.2965598	0.1278496	0.2122047	up
A_22_P00018600	lnc-EMILIN2-1	-2.511655	-2.243438	-2.355487	0.2682164	0.1561673	0.2121918	up
A_19_P00801627	ANP32AP1	3.8602839	4.064944	4.0798454	0.2046599	0.2195616	0.2121108	up
A_22_P00006414	lnc-FBXO25-2	-3.082907	-3.044877	-2.696823	0.0380297	0.3860836	0.2120566	up
A_22_P00014799	LINC00673	0.6590056	0.9124546	0.8294845	0.253449	0.1704788	0.2119639	up
A_23_P166775	IL17RC	-0.151043	-0.00647	0.1282692	0.1445737	0.2793126	0.2119431	up
A_23_P104617	GYLTL1B	2.892273	2.9936547	3.2147617	0.1013818	0.3224888	0.2119353	up
A_22_P00005606	lnc-EFR3B-3	1.6792002	1.8551908	1.9268904	0.1759906	0.2476902	0.2118404	up
A_33_P3229276	ZSCAN2	-1.493146	-1.307131	-1.25561	0.1860147	0.237536	0.2117753	up
A_23_P127175	SAR1A	6.0958824	6.435349	6.1797695	0.3394666	0.0838871	0.2116768	up
A_33_P3211404	FBXL18	-1.910658	-1.785486	-1.612505	0.1251717	0.2981525	0.2116621	up
A_19_P00321475	LOC727710	-2.037329	-1.668188	-1.983168	0.3691416	0.0541616	0.2116516	up
A_33_P3238976	TRMT5	3.1480656	3.417067	3.3023405	0.2690015	0.1542749	0.2116382	up

A_22_P00020501	PHKG2	-0.45935	-0.393765	-0.101874	0.0655856	0.3574762	0.2115309	up
A_33_P3317593	AP1B1	7.7849035	7.8726363	8.12019	0.0877328	0.3352861	0.2115095	up
A_24_P171268	RASSF5	1.6951504	1.9294562	1.8837337	0.2343059	0.1885834	0.2114446	up
A_33_P3413845	TIMM13	-0.85082	-0.952965	-0.325789	-0.102146	0.5250301	0.2114422	up
A_23_P111865	ZSCAN21	-1.303567	-0.898824	-1.285533	0.4047427	0.018033	0.2113879	up
A_33_P3385266	ABCC6	-6.48E-05	0.1256685	0.2968311	0.1257334	0.296896	0.2113147	up
A_33_P3413815	LAMB2P1	-1.227383	-1.244387	-0.787898	-0.017004	0.4394851	0.2112403	up
A_22_P00010891	LINC00116	5.598752	5.929418	5.690508	0.3306661	0.0917559	0.211211	up
A_23_P59192	SNRPC	5.8299417	5.988898	6.0933886	0.1589561	0.2634468	0.2112014	up
A_23_P49082	NUBP2	2.5357895	2.7225318	2.7713947	0.1867423	0.2356052	0.2111738	up
A_23_P36322	INPPL1	2.3033104	2.5367103	2.4921303	0.2333999	0.1888199	0.2111099	up
A_33_P3262470	SERP2	-2.729589	-2.541323	-2.495674	0.1882656	0.2339146	0.2110901	up
A_33_P3422113	ZSCAN12P1	2.3646116	2.6302123	2.5211487	0.2656007	0.1565371	0.2110689	up
A_23_P79155	GPR39	1.511127	1.7062654	1.7381101	0.1951385	0.2269831	0.2110608	up
A_33_P3235856	RORB	-1.708006	-1.587755	-1.406174	0.1202512	0.3018327	0.2110419	up
A_23_P44581	NOMO1	3.0986404	3.4149709	3.2043	0.3163304	0.1056595	0.210995	up
A_33_P3347932	HSF1	7.137599	7.3563685	7.3407373	0.2187696	0.2031384	0.210954	up
A_22_P00001555	LOC100288798	-0.992239	-0.769417	-0.793213	0.2228222	0.1990256	0.2109239	up
A_21_P0001254	lnc-MIB2-1	-0.582093	-0.387877	-0.354477	0.1942158	0.2276158	0.2109158	up
A_33_P3405897	PLEKHM1	-0.788123	-0.453212	-0.701231	0.3349114	0.0868926	0.210902	up
A_32_P225604	RPL5	9.339423	9.636339	9.464261	0.296916	0.1248379	0.2108769	up
A_23_P153867	CERS4	-1.062141	-0.928116	-0.774615	0.1340251	0.2875257	0.2107754	up
A_22_P00003762	lnc-CDIPT-2	-0.268041	0.0057826	-0.120423	0.2738233	0.1476173	0.2107203	up
A_23_P149992	PDLIM1	3.3177404	3.5838342	3.473051	0.2660937	0.1553106	0.2107022	up
A_33_P3248900	LINC01002	4.2998905	4.620091	4.4009113	0.3202004	0.1010208	0.2106106	up
A_23_P39237	ZFP36	4.7735004	5.126123	4.8420153	0.3526225	0.0685148	0.2105687	up
A_23_P131935	FERMT1	5.674367	5.793286	5.9765015	0.1189189	0.3021345	0.2105267	up
A_22_P00018452	LOC102723906	-2.426817	-2.259045	-2.173616	0.1677716	0.2532008	0.2104862	up
A_23_P37910	MAPK3	1.580926	1.7762418	1.8065672	0.1953158	0.2256413	0.2104785	up
A_21_P0013912	C19orf68	-1.896835	-1.621925	-1.75084	0.27491	0.1459951	0.2104526	up
A_23_P150009	ZDHHC16	2.8870993	3.0995326	3.0955114	0.2124333	0.2084122	0.2104228	up
A_22_P00024877	LOC100128317	-0.793432	-0.539186	-0.627027	0.2542458	0.1664047	0.2103252	up
A_23_P150249	CCDC85B	3.1686697	3.2258487	3.532113	0.057179	0.3634434	0.2103112	up
A_23_P35848	NADSYN1	2.1438494	2.2663178	2.4418964	0.1224685	0.2980471	0.2102578	up
A_22_P00022421	MIR31HG	-2.564161	-2.463628	-2.244209	0.1005323	0.319952	0.2102421	up
A_21_P0014672	LOC101928069	-1.247026	-1.042069	-1.031516	0.204957	0.2155099	0.2102335	up
A_23_P24926	FNTA	4.8434105	5.079533	5.0275936	0.2361226	0.1841831	0.2101529	up
A_21_P0000307	SNORA2B	2.8655958	2.897408	3.2540855	0.0318122	0.3884897	0.210151	up
A_24_P15391	FOXI3	-2.877227	-2.357035	-2.977122	0.5201914	-0.099895	0.2101482	up
A_21_P0001337	lnc-LRRC8D-2	-1.846638	-1.658711	-1.614309	0.1879277	0.2323289	0.2101283	up
A_33_P3312366	TOP3B	0.3572035	0.7571173	0.3773542	0.3999138	0.0201507	0.2100322	up
A_22_P00005683	MMP24-AS1	-0.895656	-0.64386	-0.727404	0.2517963	0.168252	0.2100241	up
A_23_P149690	PANK4	-0.807594	-0.549964	-0.64519	0.2576304	0.1624041	0.2100172	up
A_23_P78122	MYO18A	1.9603682	2.0771022	2.2636652	0.116734	0.303297	0.2100155	up
A_21_P0007825	lnc-DERA-1	0.3414474	0.5614815	0.5413952	0.2200341	0.1999478	0.209991	up
A_23_P45108	QRICH1	2.3648205	2.522427	2.626728	0.1576066	0.2619076	0.2097571	up
A_32_P155416	ERI3	2.0420837	2.1287913	2.3748636	0.0867076	0.3327799	0.2097437	up
A_21_P0011068	LOC100506691	-0.419367	-0.206419	-0.213033	0.2129483	0.2063341	0.2096412	up
A_33_P3253264	ZBTB48	0.0546479	0.4488788	0.0795884	0.3942308	0.0249405	0.2095857	up
A_24_P293120	ZNF576	2.8895683	2.9690166	3.2292118	0.0794482	0.3396435	0.2095459	up
A_23_P210515	NCOA5	-0.739092	-0.441902	-0.617226	0.2971907	0.1218662	0.2095284	up
A_24_P217834	HIST1H3D	6.0743666	6.2258525	6.3418684	0.1514859	0.2675018	0.2094939	up
A_22_P00014820	lnc-SLC39A8-1	-1.173955	-0.7778	-1.151127	0.3961549	0.0228271	0.209491	up
A_23_P205529	METTL17	1.8379278	2.0842843	2.0104265	0.2463565	0.1724987	0.2094276	up
A_23_P86100	KLHDC9	-1.81119	-1.543878	-1.659773	0.2673121	0.1514168	0.2093644	up
A_33_P3234809	PAX8	-1.090876	-0.732252	-1.03079	0.358624	0.0600853	0.2093546	up

A_33_P3298413	PPIH	4.5155735	4.706902	4.7429304	0.1913285	0.2273569	0.2093427	up
A_21_P0009866	LOC101927820	-3.107686	-2.693381	-3.103418	0.4143052	0.0042682	0.2092867	up
A_24_P398781	IFT43	3.9656992	4.1309943	4.218707	0.1652951	0.2530079	0.2091515	up
A_23_P415827	SEPT8	0.4726377	0.7353249	0.628067	0.2626872	0.1554294	0.2090583	up
A_23_P15375	ARMC7	2.559764	2.90906	2.6285086	0.3492961	0.0687447	0.2090204	up
A_23_P126869	PADI3	-3.191201	-3.006837	-2.957665	0.1843648	0.233536	0.2089504	up
A_33_P3305617	SPTSSB	-2.813345	-2.720139	-2.488698	0.0932062	0.3246474	0.2089268	up
A_23_P58482	MGAT1	3.3973436	3.6039615	3.6085482	0.2066178	0.2112045	0.2089112	up
A_22_P00011107	lnc-NUDT5-1	-0.836144	-0.721415	-0.533083	0.1147294	0.3030615	0.2088955	up
A_33_P3334448	SNORA62	0.8608055	0.9466696	1.1927028	0.0858641	0.3318973	0.2088807	up
A_24_P142024	CHMP4A	2.8377972	3.101912	2.9914083	0.2641149	0.1536112	0.208863	up
A_33_P3358099	CD300E	0.5632949	0.7767196	0.7673883	0.2134247	0.2040935	0.2087591	up
A_21_P0010443	lnc-RPL3-1	-0.184511	0.0878596	-0.03958	0.2723703	0.1449304	0.2086504	up
A_23_P400078	MTHFR	-0.967721	-0.699479	-0.818881	0.2682414	0.14884	0.2085407	up
A_33_P3304293	STAG2	-0.097038	0.3520947	-0.129122	0.4491329	-0.032084	0.2085245	up
A_23_P217028	USP20	0.6203208	0.8563333	0.8012462	0.2360125	0.1809254	0.2084689	up
A_21_P0014331	LOC100505942	-2.402923	-2.321754	-2.067243	0.0811694	0.33568	0.2084247	up
A_23_P309850	RPUSD2	4.5399084	4.7220798	4.774479	0.1821713	0.2345705	0.2083709	up
A_23_P56529	AAMP	3.9075832	4.075749	4.1561337	0.1681657	0.2485504	0.2083581	up
A_21_P0005995	lnc-DMRT2-1	-2.446645	-2.384612	-2.092	0.0620322	0.3546445	0.2083384	up
A_23_P21706	CTPS1	5.009782	5.105108	5.330841	0.095326	0.3210592	0.2081926	up
A_23_P308305	TTC39C	1.5149965	1.6969914	1.7492089	0.1819949	0.2342124	0.2081037	up
A_24_P923251	TGM2	-0.466033	-0.574695	0.0588031	-0.108662	0.5248356	0.2080867	up
A_22_P00002804	lnc-C2orf42-3	-1.708859	-1.561385	-1.440295	0.1474743	0.2685647	0.2080195	up
A_23_P152136	GINS3	3.6764383	3.8769135	3.8917952	0.2004752	0.2153568	0.207916	up
A_23_P102731	SMOX	2.2858524	2.4187407	2.5687456	0.1328883	0.2828932	0.2078908	up
A_33_P3362193	RAD23A	6.6513014	6.952164	6.766203	0.3008628	0.1149015	0.2078822	up
A_21_P0004718	NQO2	-2.396891	-2.146804	-2.231429	0.250087	0.1654613	0.2077742	up
A_23_P165061	AES	4.951313	5.016702	5.3013706	0.0653892	0.3500576	0.2077234	up
A_33_P3398533	PROSER1	-1.144164	-0.992812	-0.880166	0.1513519	0.263998	0.207675	up
A_33_P3236403	CLASRP	4.9726105	5.1987605	5.161645	0.22615	0.1890345	0.2075923	up
A_33_P3415037	VDAC2	6.23715	6.5184255	6.3710556	0.2812753	0.1339054	0.2075903	up
A_24_P277747	CNPY3	1.3148313	1.6606851	1.3841109	0.3458538	0.0692797	0.2075667	up
A_23_P97221	ZNF691	0.8270946	1.0412555	1.0280194	0.2141609	0.2009249	0.2075429	up
A_33_P3256347	TIMM8B	7.257929	7.4777045	7.453228	0.2197757	0.1952992	0.2075374	up
A_23_P168490	HERPUD2	0.1274138	0.3698258	0.3000517	0.2424121	0.1726379	0.207525	up
A_23_P84836	NPEPPS	2.6318245	2.9600701	2.7184687	0.3282456	0.0866442	0.2074449	up
A_23_P45999	FBXO2	2.5640373	2.5456681	2.9971056	-0.018369	0.4330683	0.2073495	up
A_23_P216689	BRD3	2.6373892	2.9903197	2.6991415	0.3529306	0.0617523	0.2073414	up
A_24_P113815	SLC35E2	-0.508683	-0.098885	-0.504162	0.4097982	0.0045204	0.2071593	up
A_23_P157465	UBXN8	1.0030417	1.1554031	1.2649503	0.1523614	0.2619085	0.207135	up
A_33_P3658861	SNORA26	-2.003559	-1.939211	-1.653717	0.0643477	0.3498416	0.2070947	up
A_22_P00017635	lnc-WNT1-2	-2.389458	-2.520739	-1.844021	-0.13128	0.5454373	0.2070785	up
A_33_P3268763	TMUB1	4.985962	5.3401055	5.0459414	0.3541436	0.0599794	0.2070615	up
A_33_P3554053	LINC00106	0.6194973	0.7263441	0.926702	0.1068468	0.3072047	0.2070258	up
A_32_P181020	DXO	0.832078	1.0942554	0.9836974	0.2621775	0.1516194	0.2068985	up
A_23_P163458	EHD4	2.8015566	2.9496284	3.0672035	0.1480718	0.2656469	0.2068594	up
A_23_P86731	ZNF239	2.443779	2.6105533	2.690689	0.1667743	0.2469101	0.2068422	up
A_21_P0000329	SNORA80E	2.7658348	2.9443822	3.000948	0.1785474	0.2351131	0.2068303	up
A_33_P3252048	PPP1R27	-2.512161	-2.615132	-1.995567	-0.102971	0.5165937	0.2068112	up
A_32_P22401	MAP7D1	5.514662	5.5700974	5.8727636	0.0554357	0.3581018	0.2067687	up
A_21_P0002374	lnc-AC112715.2.1-3	-3.137179	-2.88161	-2.979462	0.255569	0.157717	0.206643	up
A_23_P408473	MROH1	1.6599855	2.1242337	1.6090188	0.4642482	-0.050967	0.2066407	up
A_33_P3267814	MICAL3	-1.856329	-1.561441	-1.738206	0.2948885	0.1181235	0.206506	up
A_22_P00025093	PTPN3	1.658062	1.7263122	2.002695	0.0682502	0.3446331	0.2064416	up

A_23_P309198	LINC01547	-1.533629	-1.180635	-1.473805	0.3529944	0.0598245	0.2064095	up
A_32_P89310	PLEKHM3	0.5431938	0.7229238	0.776124	0.1797299	0.2329302	0.2063301	up
A_21_P0000484	SNORD76	1.1187863	1.2892785	1.3609362	0.1704922	0.2421498	0.206321	up
A_23_P116840	ATG101	3.934739	4.1067653	4.1753006	0.1720262	0.2405615	0.2062938	up
A_33_P3248586	DUX4	-1.396681	-1.012041	-1.368746	0.3846402	0.0279346	0.2062874	up
A_23_P118392	RASD1	0.6679363	0.8450432	0.9033976	0.1771069	0.2354612	0.2062841	up
A_23_P12189	DFFA	2.2270088	2.3605466	2.5059958	0.1335378	0.2789869	0.2062624	up
A_23_P152984	ALYREF	6.085099	6.273068	6.309634	0.1879687	0.224535	0.2062519	up
A_23_P124619	S100A14	7.2352953	7.6037517	7.2793303	0.3684564	0.044035	0.2062457	up
A_33_P3322125	DAZAP2	-3.010324	-3.137404	-2.470882	-0.12708	0.5394411	0.2061806	up
A_23_P52373	NDST2	0.0859075	0.2558923	0.3282738	0.1699848	0.2423663	0.2061756	up
A_21_P0007034	lnc-EBF3-4	-1.487362	-1.3957	-1.166698	0.0916624	0.3206639	0.2061632	up
A_33_P3368785	DLGAP1-AS1	3.856619	4.1446404	3.9808636	0.2880216	0.1242447	0.2061331	up
A_24_P333494	FBXO42	1.9543447	2.2600865	2.0607195	0.3057418	0.1063747	0.2060583	up
A_33_P3344308	APTX	3.437477	3.5305781	3.7564478	0.093101	0.3189707	0.2060359	up
A_33_P3372941	TINF2	2.874422	3.0240588	3.1368341	0.1496368	0.2624121	0.2060244	up
A_23_P106505	LCMT2	2.311633	2.5696235	2.4656277	0.2579904	0.1539946	0.2059925	up
A_33_P3292854	CALR	8.281451	8.457907	8.516944	0.1764555	0.2354927	0.2059741	up
A_24_P333019	RNF24	0.2239709	0.3964863	0.4633646	0.1725154	0.2393937	0.2059546	up
A_23_P110345	CHIC2	1.0864072	1.3888879	1.1957846	0.3024807	0.1093774	0.205929	up
A_21_P0005919	lnc-FBXO25-4	-2.423901	-1.807412	-2.628631	0.6164889	-0.20473	0.2058793	up
A_21_P0000300	SNORA48	2.1256065	2.1977963	2.465168	0.0721898	0.3395615	0.2058756	up
A_24_P290770	OGDH	-1.190529	-0.779115	-1.190241	0.4114137	0.0002875	0.2058506	up
A_23_P200203	ECHDC2	5.1924763	5.3349166	5.461691	0.1424403	0.2692146	0.2058275	up
A_19_P00803507	LOC101929666	-2.091815	-1.951866	-1.820484	0.1399488	0.2713304	0.2056396	up
A_22_P00017749	lnc-YIF1A-1	-2.73191	-2.538514	-2.514049	0.1933954	0.2178602	0.2056278	up
A_33_P3225937	CRYZL1	-1.625426	-1.41053	-1.429209	0.2148967	0.1962175	0.2055571	up
A_32_P135336	LOC388242	0.3181763	0.7238612	0.3235865	0.405685	0.0054102	0.2055476	up
A_19_P00321872	LINC00958	-1.217469	-0.968979	-1.055061	0.2484903	0.1624079	0.2054491	up
A_33_P3244181	HSBP1	6.277544	6.402373	6.5635767	0.1248288	0.2860327	0.2054307	up
A_33_P3219303	CEL	-0.595406	-0.251596	-0.528379	0.3438101	0.0670266	0.2054183	up
A_24_P170763	KHNYN	1.9620457	2.0474534	2.2871876	0.0854077	0.3251419	0.2052748	up
A_23_P202773	TEX40	1.16472	1.1359448	1.6037755	-0.028775	0.4390554	0.2051401	up
A_23_P144896	PDLIM7	2.3798122	2.6425138	2.5273542	0.2627015	0.147542	0.2051218	up
A_33_P3215803	EMR1	-2.367201	-2.166925	-2.157252	0.2002759	0.209949	0.2051125	up
A_32_P145867	OCM2	-2.896915	-2.453046	-2.930661	0.4438682	-0.033747	0.2050607	up
A_32_P121674	lnc-PAFAH1B1-2	-1.791143	-1.440465	-1.731827	0.3506784	0.0593162	0.2049973	up
A_33_P3211238	VWCE	-1.197143	-0.807498	-1.176829	0.3896446	0.0203133	0.2049789	up
A_23_P83579	ARNT2	2.2705612	2.262052	2.6887589	-0.008509	0.4181976	0.2048442	up
A_21_P0000035	TMEM217	-1.50534	-1.233995	-1.367055	0.2713451	0.1382842	0.2048147	up
A_22_P00012887	lnc-RBM17-1	-1.951161	-1.503205	-1.989821	0.4479561	-0.038659	0.2046485	up
A_22_P00003002	lnc-C6orf228-2	-1.719196	-1.429739	-1.599682	0.2894578	0.119514	0.2044859	up
A_33_P3399443	SH3TC1	-0.463696	-0.271531	-0.247155	0.1921644	0.2165408	0.2043526	up
A_33_P3230264	GPC3	-0.125572	0.1963363	-0.038851	0.3219085	0.0867209	0.2043147	up
A_32_P80245	ZFP57	1.5083456	1.7813015	1.6440153	0.2729559	0.1356697	0.2043128	up
A_22_P00007297	lnc-GPR3-2	-3.172084	-2.975759	-2.95993	0.1963246	0.2121534	0.204239	up
A_23_P413815	VKORC1L1	3.473299	3.675383	3.6795797	0.2020841	0.2062807	0.2041824	up
A_33_P3324765	CSNK1D	3.470972	3.6261058	3.7241678	0.1551337	0.2531958	0.2041647	up
A_33_P3217689	JMY	-0.706251	-0.161935	-0.842328	0.5443158	-0.136077	0.2041194	up
A_22_P00013618	lnc-RP11-582J16.5.1-3	-0.356553	-0.179361	-0.125708	0.1771922	0.2308455	0.2040188	up
A_23_P41470	DDX60	1.0406938	0.9621067	1.527288	-0.078587	0.4865942	0.2040036	up
A_33_P3327500	UFSP1	-0.84576	-0.673309	-0.610205	0.172451	0.2355557	0.2040033	up
A_23_P98057	ZNF32	0.8891082	1.0605936	1.1256137	0.1714854	0.2365055	0.2039955	up
A_23_P129389	ZDHHC7	2.6970558	2.8467956	2.9552555	0.1497397	0.2581997	0.2039697	up
A_23_P76901	PLEKHG3	5.6602383	5.82017	5.908078	0.1599317	0.2478399	0.2038858	up

A_33_P3364389	SYT8	0.7667308	0.9417353	0.9994679	0.1750045	0.2327371	0.2038708	up
A_33_P3299892	UQCRH	9.227996	9.438383	9.425249	0.2103872	0.1972532	0.2038202	up
A_33_P3347622	MOB1B	2.1391706	2.48846	2.1972055	0.3492894	0.0580349	0.2036622	up
A_23_P147605	C11orf49	2.9474716	3.0482173	3.253912	0.1007457	0.3064404	0.203593	up
A_24_P256243	SSX2	-1.473778	-1.299643	-1.240785	0.1741352	0.2329931	0.2035642	up
A_23_P204364	NOP2	3.3770714	3.4674668	3.6937332	0.0903955	0.3166618	0.2035286	up
A_33_P3277259	PCYT2	1.9607496	2.1443982	2.1841164	0.1836486	0.2233667	0.2035077	up
A_23_P369701	ABHD17C	3.9295912	4.1267104	4.139367	0.1971192	0.2097759	0.2034476	up
A_23_P204436	GIT2	-1.419125	-1.454235	-0.977134	-0.03511	0.4419913	0.2034409	up
A_22_P00013683	lnc-RP11-712L6.5.1-1	0.9946022	1.2654271	1.1305971	0.2708249	0.1359949	0.2034099	up
A_21_P0004860	BTN2A1	0.2969327	0.5197797	0.4808989	0.222847	0.1839662	0.2034066	up
A_33_P3344277	HS1BP3	-1.530315	-1.666839	-0.98702	-0.136524	0.5432954	0.2033856	up
A_23_P111981	LYNX1	-2.028451	-1.783855	-1.866305	0.244596	0.1621461	0.203371	up
A_33_P3380567	SHARPIN	5.007494	5.0723605	5.3491974	0.0648665	0.3417034	0.203285	up
A_23_P145874	SAMD9L	1.1732578	1.0509558	1.7021041	-0.122302	0.5288463	0.2032721	up
A_22_P00003303	lnc-CAND2-1	-3.348475	-3.064665	-3.225759	0.2838099	0.1227157	0.2032628	up
A_21_P0003138	lnc-RPL24-2	-0.996578	-0.752419	-0.83429	0.2441597	0.1622887	0.2032242	up
A_33_P3262028	XLOC_l2_010082	-2.839332	-2.450709	-2.821535	0.3886228	0.0177968	0.2032098	up
A_33_P3216610	TMPRSS4	3.7632952	3.930571	4.002369	0.1672759	0.2390738	0.2031748	up
A_33_P3270197	DIS3L2	-2.378832	-2.280151	-2.071196	0.0986812	0.3076367	0.203159	up
A_21_P0013877	XLOC_l2_015885	9.79564	10.025614	9.971928	0.2299738	0.1762877	0.2031307	up
A_23_P85171	EMD	2.6372232	2.8385787	2.8420658	0.2013555	0.2048426	0.203099	up
A_24_P278192	AK1	0.3258457	0.4638834	0.5939169	0.1380377	0.2680712	0.2030544	up
A_24_P75157	TCP10	-1.62134	-1.315309	-1.521495	0.3060317	0.0998449	0.2029383	up
A_33_P3682006	DBH-AS1	-1.189962	-0.920591	-1.053577	0.269371	0.136385	0.202878	up
A_33_P3897698	LOC100147773	-2.984076	-2.585256	-2.977228	0.3988197	0.0068474	0.2028335	up
A_24_P278367	ACTR1A	3.320403	3.4331012	3.6133661	0.1126981	0.292963	0.2028306	up
A_33_P3288135	CPLX2	-1.28105	-1.007603	-1.149124	0.273447	0.1319265	0.2026868	up
A_32_P158355	ALG1L	2.805193	2.9380193	3.077736	0.1328263	0.272543	0.2026846	up
A_24_P393844	DPH2	3.2504196	3.4527898	3.4532757	0.2023702	0.2028561	0.2026131	up
A_33_P3356290	CADPS	-2.370224	-1.890347	-2.4451	0.4798775	-0.074876	0.2025007	up
A_23_P207600	PSMD11	4.143281	4.317021	4.374526	0.1737399	0.231245	0.2024925	up
A_23_P110005	NICN1	1.3149633	1.5690255	1.4658694	0.2540622	0.1509061	0.2024841	up
A_22_P00014210	MYLK-AS1	-1.579899	-1.067005	-1.687847	0.5128946	-0.107947	0.2024736	up
A_33_P3364582	TNXB	1.2831659	1.4967532	1.4744129	0.2135873	0.191247	0.2024171	up
A_23_P90089	GCDH	2.205924	2.4974093	2.3192186	0.2914853	0.1132946	0.20239	up
A_24_P69095	ENC1	1.1476154	1.1786323	1.5213442	0.0310168	0.3737288	0.2023728	up
A_33_P3229241	HIST2H2BF	1.1821012	1.4302058	1.3386669	0.2481046	0.1565657	0.2023351	up
A_33_P3670415	NAT8L	1.4470053	1.7784357	1.5201554	0.3314304	0.0731502	0.2022903	up
A_24_P379233	GJB3	3.1170292	3.2758431	3.3626413	0.158814	0.2456121	0.202213	up
A_23_P34107	FAM50A	3.3886309	3.608971	3.5727043	0.2203403	0.1840735	0.2022069	up
A_33_P3330175	XLOC_l2_015203	0.5568399	0.7405152	0.7775645	0.1836753	0.2207246	0.2021999	up
A_24_P14010	NMT1	5.0256853	5.114845	5.340767	0.0891595	0.3150816	0.2021205	up
A_24_P876408	C11orf95	0.9293251	1.091754	1.1711044	0.1624289	0.2417793	0.2021041	up
A_23_P218331	CYB561	4.7691355	4.9369473	5.0054903	0.1678119	0.2363548	0.2020834	up
A_23_P380881	ANKRD13B	-0.025583	0.0832429	0.2695894	0.1088257	0.2951722	0.2019989	up
A_21_P0010912	XLOC_l2_002176	8.391862	8.684362	8.503242	0.2925005	0.1113796	0.2019401	up
A_22_P00006435	lnc-FBXO7-1	-2.702975	-2.643835	-2.358387	0.0591397	0.3445878	0.2018638	up
A_23_P202837	CCND1	3.6650114	3.9780402	3.7557087	0.3130288	0.0906973	0.201863	up
A_21_P0011766	XLOC_l2_007147	-2.471544	-2.276422	-2.262957	0.1951222	0.2085869	0.2018546	up
A_23_P36076	SSRP1	7.4869986	7.7446494	7.633048	0.2576509	0.1460495	0.2018502	up
A_24_P353289	GUCD1	4.3123636	4.5362544	4.492152	0.2238908	0.1797886	0.2018397	up
A_23_P104464	ALOX5	0.4537535	1.0054111	0.3054867	0.5516577	-0.148267	0.2016955	up
A_33_P3287685	CNOT3	4.4445133	4.6528907	4.6394377	0.2083774	0.1949244	0.2016509	up
A_33_P3274069	RHBDD3	3.2665424	3.5080724	3.4282732	0.2415299	0.1617308	0.2016304	up

A_33_P3272539	PLEKHG5	2.434144	2.6192212	2.6522799	0.1850772	0.2181358	0.2016065	up
A_23_P374288	CASC3	5.197007	5.5136065	5.2835655	0.3165994	0.0865583	0.2015789	up
A_23_P75299	LHPP	3.1633673	3.356336	3.3735275	0.1929689	0.2101603	0.2015646	up
A_22_P00014166	lnc-SCRN3-2	-0.91538	-0.890942	-0.53678	0.0244374	0.3785992	0.2015183	up
A_23_P123905	EXOSC3	3.4230824	3.4859796	3.763216	0.0628972	0.3401337	0.2015154	up
A_33_P3385782	ZNF713	-1.987361	-1.465613	-2.106155	0.5217476	-0.118795	0.2014763	up
A_23_P149099	DDOST	4.6300764	4.8878007	4.775296	0.2577243	0.1452198	0.2014721	up
A_22_P00011989	LOC101929897	-1.869462	-1.781795	-1.554278	0.0876675	0.3151836	0.2014256	up
A_33_P3295523	RAC3	4.2120943	4.3856406	4.4413614	0.1735463	0.2292671	0.2014067	up
A_24_P272845	DOCK3	-1.696722	-1.353099	-1.637541	0.3436232	0.0591807	0.2014019	up
A_33_P3221748	RUNX3	5.054907	5.191794	5.320795	0.1368871	0.2658882	0.2013876	up
A_23_P201238	SNX27	1.7207556	1.8660231	1.9780798	0.1452675	0.2573242	0.2012959	up
A_23_P153086	RBFA	2.5004148	2.8301482	2.573246	0.3297334	0.0728312	0.2012823	up
A_23_P350107	TRIM56	2.289878	2.471846	2.5104303	0.1819682	0.2205524	0.2012603	up
A_24_P290354	ZFAND5	3.2812815	3.575951	3.3890142	0.2946696	0.1077328	0.2012012	up
A_23_P29803	POLR2H	5.8953123	6.001924	6.191061	0.1066117	0.2957487	0.2011802	up
A_24_P35478	PARD3	0.8488126	1.1667881	0.9331484	0.3179755	0.0843358	0.2011557	up
A_23_P388871	HIST4H4	0.4467711	0.5476856	0.7481275	0.1009145	0.3013563	0.2011354	up
A_24_P37253	LYPD6	-1.371395	-1.450717	-0.890201	-0.079322	0.481194	0.2009358	up
A_33_P3387696	TMBIM4	1.9965677	2.2918677	2.1031094	0.2953	0.1065416	0.2009208	up
A_32_P191527	SETD8	0.6959415	0.85074	0.9429693	0.1547985	0.2470279	0.2009132	up
A_23_P27332	TCF4	-2.510368	-2.510989	-2.108079	-0.000621	0.4022889	0.2008338	up
A_32_P98502	COX5A	6.662298	6.8666368	6.8593464	0.2043386	0.1970482	0.2006934	up
A_33_P3263027	LOC100289120	-1.169007	-1.022663	-0.91401	0.1463447	0.2549973	0.200671	up
A_23_P64860	SELPLG	-1.82423	-1.466691	-1.780463	0.3575397	0.043767	0.2006533	up
A_23_P12858	TBATA	-2.248448	-1.804253	-2.291406	0.4441948	-0.042958	0.2006183	up
A_23_P94053	TRRAP	1.4027824	1.4752045	1.7315931	0.072422	0.3288107	0.2006164	up
A_33_P3427102	TTL	5.5157356	5.6131406	5.8195524	0.097405	0.3038168	0.2006109	up
A_33_P3266530	DUS1L	5.7358923	6.0117793	5.861108	0.275887	0.1252155	0.2005513	up
A_24_P297480	RNF113B	-2.345319	-2.134589	-2.154997	0.2107296	0.1903215	0.2005255	up
A_33_P3340649	KRTAP19-8	-0.566669	-0.256094	-0.47647	0.3105741	0.0901985	0.2003863	up
A_33_P3260426	SPRR2A	-2.123322	-1.692355	-2.153559	0.4309669	-0.030238	0.2003646	up
A_33_P7718819	EXOSC2	9.9870615	10.262188	10.112658	0.2751265	0.1255961	0.2003613	up
A_22_P00016869	LOC101927550	-2.086947	-1.797977	-1.97529	0.28897	0.1116567	0.2003133	up
A_23_P319719	PGAM5	-0.378428	-0.239416	-0.116837	0.1390114	0.261591	0.2003012	up
A_23_P318396	CELF1	2.8282585	3.184567	2.872467	0.3563085	0.0442085	0.2002585	up
A_24_P223384	HIST1H2AB	2.7652702	2.876349	3.0546627	0.1110787	0.2893925	0.2002356	up
A_23_P331700	SRRM3	0.8275909	1.0094872	1.0459642	0.1818962	0.2183733	0.2001348	up
A_23_P162982	DHRS4	1.8916464	2.0479732	2.1355572	0.1563268	0.2439108	0.2001188	up
A_33_P3272823	MAG	-1.27395	-1.03742	-1.110289	0.2365298	0.163661	0.2000954	up
A_33_P3228959	MRPL11	5.31977	5.5470796	5.492593	0.2273097	0.172823	0.2000663	up
A_33_P3366082	EBPL	4.966814	5.1858497	5.1478596	0.2190356	0.1810455	0.2000406	up
A_23_P65481	TEP1	1.5935602	1.7372589	1.8498487	0.1436987	0.2562885	0.1999936	up
A_23_P69326	CADPS	1.5826912	1.9128928	1.6524644	0.3302016	0.0697732	0.1999874	up
A_23_P422044	PCDHGA2	-0.896438	-0.586385	-0.806558	0.3100529	0.08988	0.1999664	up
A_23_P19004	BRD9	1.3361096	1.4917059	1.5804019	0.1555963	0.2442923	0.1999443	up
A_23_P400147	CCDC61	1.4943666	1.6128521	1.7755828	0.1184855	0.2812161	0.1998508	up
A_33_P3237775	NR1H3	5.0376396	5.1605067	5.3144665	0.1228671	0.2768269	0.199847	up
A_22_P00007717	PSMB8-AS1	-1.568579	-1.440361	-1.297139	0.1282177	0.27144	0.1998289	up
A_23_P301877	MED27	1.4563828	1.6337914	1.6786051	0.1774087	0.2222223	0.1998155	up
A_22_P00019589	lnc-ITGAL-2	-1.358695	-1.159967	-1.157794	0.1987286	0.200901	0.1998148	up
A_22_P00012951	lnc-RECQL5-1	-2.135461	-2.095673	-1.775631	0.0397882	0.3598299	0.1998091	up
A_24_P66001	UQCR10	1.5031757	1.6506972	1.7552185	0.1475215	0.2520428	0.1997821	up
A_23_P16063	GRIK5	-2.306801	-1.880854	-2.333201	0.4259477	-0.0264	0.1997738	up
A_23_P106727	RAB11FIP3	2.1639032	2.3953776	2.331932	0.2314744	0.1680288	0.1997516	up
A_21_P0013007	LOC643201	-2.958984	-3.002533	-2.51602	-0.043549	0.4429638	0.1997076	up

A_22_P00015513	LOC101929294	-1.558609	-1.334503	-1.383509	0.2241054	0.1750999	0.1996026	up
A_33_P3329597	BLOC1S3	3.9639463	4.3514934	3.9755993	0.387547	0.0116529	0.1996	up
A_21_P0011263	XLOC_l2_004371	-1.594795	-1.489596	-1.300909	0.1051984	0.2938857	0.199542	up
A_33_P3355247	CCDC69	-0.490586	-0.259023	-0.323084	0.2315631	0.1675019	0.1995325	up
A_23_P216630	SLC44A1	4.834876	5.118342	4.9504194	0.2834659	0.1155434	0.1995046	up
A_23_P68487	BMP7	6.12024	6.0549664	6.5842505	-0.065274	0.4640102	0.1993682	up
A_23_P212339	FYCO1	1.7814274	1.9821286	1.9794598	0.2007012	0.1980324	0.1993668	up
A_32_P6344	MAP2K4	3.1633663	3.5574374	3.1678782	0.3940711	0.0045118	0.1992915	up
A_23_P28697	HAAO	-2.674507	-2.358494	-2.591987	0.3160136	0.0825205	0.199267	up
A_24_P38895	H2AFX	5.381792	5.4979367	5.6641436	0.1161447	0.2823515	0.1992481	up
A_23_P201264	NMNAT1	-1.350658	-1.12551	-1.177424	0.2251473	0.173233	0.1991901	up
A_23_P253177	STK24	5.521044	5.7521434	5.688034	0.2310996	0.1669903	0.1990449	up
A_32_P98298	DUSP8	-1.317804	-1.038045	-1.199734	0.2797589	0.1180701	0.1989145	up
A_23_P41765	IRF1	-0.797465	-0.607941	-0.589316	0.1895247	0.2081494	0.198837	up
A_33_P3355418	AGBL1	-2.846055	-2.479872	-2.81476	0.3661828	0.0312951	0.1987389	up
A_23_P149852	LZTS2	3.3732643	3.5715632	3.5724182	0.1982989	0.1991539	0.1987264	up
A_33_P3366102	KLHL7	1.9283314	2.1948857	2.0590277	0.2665544	0.1306963	0.1986253	up
A_33_P3382513	RPUSD1	5.766263	5.938318	5.9913015	0.1720548	0.2250385	0.1985467	up
A_23_P407115	PIP4K2B	2.8225574	3.0267072	3.0153694	0.2041497	0.192812	0.1984808	up
A_33_P3209404	XLOC_l2_006173	-1.253911	-0.953826	-1.157115	0.3000846	0.0967965	0.1984406	up
A_23_P75622	ATP5L	9.251097	9.398222	9.500832	0.1471252	0.2497349	0.1984301	up
A_33_P3305446	DEFB121	-3.022581	-3.009043	-2.639442	0.0135388	0.3831399	0.1983393	up
A_23_P329924	HCAR2	-3.057617	-2.92483	-2.79428	0.1327877	0.2633371	0.1980624	up
A_23_P149678	PIAS3	3.1985044	3.3939996	3.399067	0.1954951	0.2005625	0.1980288	up
A_33_P3226492	RAB7A	-1.470858	-1.507649	-1.038169	-0.03679	0.4326892	0.1979494	up
A_23_P6762	JAGN1	5.142888	5.282784	5.398631	0.1398959	0.255743	0.1978195	up
A_22_P00004292	lnc-CNOT6-4	-1.077127	-0.862845	-0.895815	0.2142816	0.1813116	0.1977966	up
A_23_P161171	ASAH2	-1.35737	-1.152684	-1.166515	0.2046862	0.190855	0.1977706	up
A_21_P0007887	LOC643770	-1.305305	-1.391159	-0.823954	-0.085854	0.4813514	0.1977489	up
A_23_P104692	PELI3	2.1906147	2.3304286	2.4462824	0.1398139	0.2556677	0.1977408	up
A_22_P00021229	lnc-SHISA4-1	-0.450143	0.0149088	-0.519773	0.4650517	-0.06963	0.197711	up
A_22_P00014659	lnc-SLC22A12-1	-1.930794	-1.739137	-1.727139	0.1916571	0.2036557	0.1976564	up
A_21_P0014205	WBSCR22	-3.580385	-3.257202	-3.508259	0.3231828	0.0721262	0.1976545	up
A_23_P349676	FBXO41	3.8612976	4.01533	4.102501	0.1540322	0.2412033	0.1976178	up
A_23_P129188	CALML4	4.0890865	4.230821	4.342574	0.1417346	0.2534876	0.1976111	up
A_33_P3316064	CA5BP1	-2.176711	-2.086051	-1.872217	0.0906601	0.3044939	0.197577	up
A_24_P832156	FAM120AOS	3.6017609	3.9793668	3.6192617	0.3776059	0.0175009	0.1975534	up
A_21_P0010257	lnc-PKNOX1-2	-2.096957	-2.062233	-1.736629	0.0347238	0.3603287	0.1975262	up
A_22_P00003338	C5orf66	-0.914742	-0.906009	-0.528495	0.0087328	0.3862472	0.19749	up
A_23_P7099	AGA	1.2072282	1.6509366	1.1583452	0.4437084	-0.048883	0.1974127	up
A_23_P502627	ZXDA	-2.602487	-2.441331	-2.368965	0.1611559	0.2335222	0.1973391	up
A_33_P3402076	BAD	-2.885658	-2.805743	-2.571028	0.0799148	0.31463	0.1972724	up
A_23_P160226	MROH7	-0.757522	-0.235119	-0.885397	0.5224028	-0.127875	0.197264	up
A_33_P3329916	RPL6	10.091817	10.343176	10.234898	0.251359	0.1430807	0.1972199	up
A_23_P13425	CD81	6.099265	6.075481	6.5174417	-0.023784	0.4181767	0.1971962	up
A_22_P00016110	THTPA	2.5450163	2.8404965	2.6438284	0.2954803	0.0988121	0.1971462	up
A_23_P387523	ZBTB40	1.9289932	2.2202754	2.031828	0.2912822	0.1028347	0.1970584	up
A_23_P411113	CNTNAP1	-1.897284	-1.56901	-1.831465	0.3282738	0.0658188	0.1970463	up
A_23_P117506	DHRS7	4.101264	4.3712134	4.225355	0.2699494	0.1240912	0.1970203	up
A_24_P42389	OTUD6A	-1.715588	-1.488821	-1.548395	0.2267675	0.1671929	0.1969802	up
A_33_P3370787	EPHB2	1.0399289	1.3524852	1.1211929	0.3125563	0.081264	0.1969101	up
A_33_P3297468	SLC34A3	0.516324	0.7808948	0.6455593	0.2645707	0.1292353	0.196903	up
A_23_P122579	DAXX	1.6203537	1.774457	1.8597708	0.1541033	0.2394171	0.1967602	up
A_21_P0011915	XLOC_l2_008013	4.302556	4.53657	4.461918	0.234014	0.1593618	0.1966879	up
A_23_P54170	MED6	2.0036335	2.1170564	2.2835808	0.1134229	0.2799473	0.1966851	up
A_23_P126939	RAB29	0.022222	0.1074891	0.3303251	0.0852671	0.3081031	0.1966851	up

A_21_P0006611	lnc-PFKP-11	-2.094306	-1.805034	-1.990216	0.2892718	0.1040902	0.196681	up
A_23_P122775	RTN4IP1	3.0694914	3.1397138	3.3926086	0.0702224	0.3231173	0.1966698	up
A_33_P3418158	SLC2A8	0.5596294	0.8733163	0.6392565	0.3136869	0.079627	0.1966569	up
A_33_P3398912	SLC2A6	3.4989204	3.4304042	3.9607067	-0.068516	0.4617863	0.196635	up
A_21_P0010844	XLOC_l2_001760	1.5572548	1.7646437	1.74301	0.2073889	0.1857553	0.1965721	up
A_22_P00021105	lnc-C16orf13-1	0.738842	0.9818664	0.8889613	0.2430244	0.1501193	0.1965718	up
A_23_P252775	APTX	2.2844257	2.5227761	2.4392185	0.2383504	0.1547928	0.1965716	up
A_23_P150379	MPZL2	2.4765263	2.4342084	2.9118433	-0.042318	0.435317	0.1964996	up
A_33_P3315554	ANKMY1	2.0808115	2.375443	2.178959	0.2946315	0.0981474	0.1963894	up
A_19_P00321342	CCDC171	-1.152788	-1.052525	-0.860448	0.1002631	0.2923398	0.1963015	up
A_22_P00008088	lnc-IL15RA-1	-1.04395	-0.86511	-0.830206	0.1788392	0.2137437	0.1962914	up
A_23_P332326	ARHGEF19	2.83041	3.1546798	2.8986855	0.3242698	0.0682755	0.1962726	up
A_21_P0013757	XLOC_l2_015478	9.55034	10.055758	9.437434	0.5054178	-0.112906	0.1962562	up
A_23_P26557	C16orf59	3.7681284	4.0074296	3.9213247	0.2393012	0.1531963	0.1962488	up
A_21_P0000269	SNORA52	-2.182416	-1.945966	-2.026428	0.2364497	0.1559882	0.196219	up
A_23_P207967	CTIF	0.0961995	0.282599	0.3020835	0.1863995	0.205884	0.1961417	up
A_33_P3414487	WBSCR22	5.083009	5.363629	5.194623	0.2806201	0.1116142	0.1961172	up
A_24_P339664	NCLN	5.0668917	5.150657	5.37535	0.0837655	0.3084583	0.1961119	up
A_33_P3227264	CDK11A	5.088133	5.265364	5.303114	0.1772313	0.2149811	0.1961062	up
A_23_P143016	ARID5A	0.7959757	1.0271168	0.956913	0.2311411	0.1609373	0.1960392	up
A_24_P396650	RPS6KA1	1.599143	1.7829423	1.8074145	0.1837993	0.2082715	0.1960354	up
A_33_P3341490	KRT42P	-1.35816	-1.232959	-1.091316	0.1252008	0.2668438	0.1960223	up
A_33_P3261610	POLR3GL	2.2288694	2.5055804	2.3441687	0.276711	0.1152992	0.1960051	up
A_21_P0001995	LINC01304	-0.206276	-0.0046	-0.015991	0.2016759	0.1902852	0.1959805	up
A_33_P3636590	SUCLG2	0.7984238	0.9393997	1.0493917	0.140976	0.250968	0.195972	up
A_24_P34545	ING5	0.863698	1.2419963	0.8772678	0.3782983	0.0135698	0.1959341	up
A_23_P130731	TBC1D17	0.9527845	1.0999179	1.1974959	0.1471334	0.2447114	0.1959224	up
A_33_P3356255	ANXA11	-1.316974	-0.951619	-1.290534	0.3653555	0.0264406	0.1958981	up
A_23_P160828	C1orf159	0.1784506	0.2940779	0.4545202	0.1156273	0.2760696	0.1958485	up
A_23_P328545	GABRP	-2.405372	-2.420105	-1.99895	-0.014733	0.4064217	0.1958442	up
A_21_P0004402	lnc-C5orf43-3	0.9735303	1.1356401	1.2030821	0.1621099	0.2295518	0.1958308	up
A_24_P382489	SLC27A1	3.4791975	3.4347434	3.9152813	-0.044454	0.4360838	0.1958149	up
A_24_P182122	ND1	5.8189545	6.174456	5.8545094	0.3555017	0.0355549	0.1955283	up
A_33_P3215953	MPZL1	2.5098076	2.7875495	2.6230555	0.2777419	0.1132479	0.1954949	up
A_23_P163639	ANKRD11	2.5780869	2.9585853	2.5884104	0.3804984	0.0103235	0.195411	up
A_33_P3323481	PMF1-BGLAP	-1.074176	-0.650181	-1.107371	0.423995	-0.033195	0.1953998	up
A_23_P101505	KLK11	5.8304033	5.9955173	6.055977	0.1651139	0.2255735	0.1953437	up
A_33_P3279241	ZNF608	-1.756621	-1.442958	-1.679621	0.313663	0.0770001	0.1953316	up
A_33_P3367017	BRMS1	1.9858437	2.1423287	2.2199678	0.1564851	0.2341242	0.1953046	up
A_21_P0011715	ZNF865	0.5011025	0.6674171	0.725348	0.1663146	0.2242456	0.1952801	up
A_22_P00016144	PSMA3-AS1	2.3154478	2.3641257	2.657218	0.0486779	0.3417702	0.195224	up
A_24_P8494	SEC61B	6.807892	6.9319215	7.0743046	0.1240296	0.2664127	0.1952212	up
A_33_P3243153	GFPT1	3.7957735	4.349014	3.6328907	0.5532403	-0.162883	0.1951788	up
A_33_P3424067	SUV420H1	-3.077662	-2.846493	-2.918758	0.231169	0.1589041	0.1950365	up
A_32_P78783	ZNF778	-1.043346	-0.64393	-1.05271	0.399416	-0.009363	0.1950264	up
A_19_P00810045	MLX	1.183958	1.316782	1.4411755	0.1328239	0.2572174	0.1950207	up
A_21_P0013476	XLOC_l2_014196	-2.467389	-2.230512	-2.314254	0.2368777	0.1531353	0.1950065	up
A_32_P68746	SMU1	2.623643	2.8002028	2.8370237	0.1765599	0.2133808	0.1949704	up
A_24_P68247	TRIM4	-1.279509	-1.066222	-1.102865	0.2132869	0.1766439	0.1949654	up
A_24_P385313	PTPRF	6.5635195	6.9325194	6.584361	0.369	0.0208416	0.1949208	up
A_23_P501795	MED22	0.7983756	0.8685165	1.1179781	0.0701408	0.3196025	0.1948717	up
A_33_P3406449	SLC15A4	0.8209128	0.998539	1.0329943	0.1776261	0.2120814	0.1948538	up
A_24_P769672	C12orf73	2.5733423	2.6522212	2.884139	0.0788789	0.3107967	0.1948378	up
A_24_P313822	PAK4	1.2000499	1.443788	1.3458881	0.2437382	0.1458383	0.1947882	up
A_23_P39067	SPIB	-2.842082	-2.756975	-2.537899	0.0851069	0.3041832	0.194645	up
A_23_P40588	HSCB	1.743474	2.016541	1.8596802	0.273067	0.1162062	0.1946366	up

A_23_P126917	IGSF3	-3.068565	-2.845309	-2.90265	0.2232559	0.1659153	0.1945856	up
A_22_P00002037	HAR1B	-3.409784	-3.23227	-3.198175	0.1775136	0.2116089	0.1945612	up
A_23_P146077	ZNF395	2.5183372	2.8881345	2.5375757	0.3697972	0.0192385	0.1945179	up
A_23_P77073	SPPL2A	2.910079	3.248135	2.9608955	0.3380561	0.0508165	0.1944363	up
A_23_P141146	FBXL20	2.2613134	2.727837	2.1836605	0.4665237	-0.077653	0.1944354	up
A_24_P391526	MAGED1	3.3686047	3.491387	3.6346655	0.1227822	0.2660608	0.1944215	up
A_23_P216489	GNE	4.99759	5.0196815	5.3641987	0.0220914	0.3666086	0.19435	up
A_22_P00022940	lnc-JMJD7-PLA2G4B-5	-1.5777	-1.320916	-1.44588	0.256784	0.1318197	0.1943018	up
A_23_P117082	HEBP1	4.3118296	4.5330625	4.4791594	0.2212329	0.1673298	0.1942813	up
A_23_P140648	CYFIP1	4.6777973	4.908262	4.8358564	0.2304645	0.1580591	0.1942618	up
A_33_P3230990	SCUBE1	-2.348208	-2.321553	-1.98638	0.0266557	0.3618288	0.1942422	up
A_33_P3330384	BET1L	-1.504043	-1.254597	-1.365061	0.2494459	0.1389818	0.1942139	up
A_21_P0000237	SNORA10	3.3569546	3.5210166	3.581214	0.164062	0.2242594	0.1941607	up
A_33_P3237927	ARHGAP4	-1.117999	-0.963708	-0.884055	0.1542902	0.2339439	0.1941171	up
A_24_P314179	ETS2	3.0382023	3.0781808	3.3863974	0.0399785	0.3481951	0.1940868	up
A_33_P3279720	ARFRP1	-1.18845	-0.983555	-1.005193	0.2048955	0.1832576	0.1940765	up
A_21_P0002558	lnc-DTYMK-2	-3.433688	-2.803174	-3.676284	0.6305144	-0.242596	0.1939591	up
A_24_P215804	CKLF	0.8602181	1.167664	0.9406862	0.307446	0.0804682	0.1939571	up
A_24_P184305	BBS1	-0.210735	0.0841503	-0.118042	0.2948856	0.0926929	0.1937892	up
A_23_P10685	HSPBP1	5.67309	5.708591	6.024988	0.035501	0.3518982	0.1936996	up
A_32_P19840	lnc-EPHA1-1	1.779666	2.0910182	1.8556376	0.3113523	0.0759716	0.1936619	up
A_23_P28084	STX10	3.4506102	3.8192172	3.469221	0.368607	0.018611	0.193609	up
A_21_P0010890	XLOC_l2_002033	-1.520593	-1.478519	-1.175567	0.0420737	0.3450265	0.1935501	up
A_23_P69468	NDUFB4	7.952607	8.094612	8.197512	0.142005	0.2449045	0.1934547	up
A_23_P132121	SIK1	0.9961562	1.1598616	1.2193394	0.1637054	0.2231832	0.1934443	up
A_23_P55421	CBX8	0.2274098	0.4270454	0.4144702	0.1996355	0.1870604	0.1933479	up
A_23_P27810	ZNF607	3.079298	3.2077441	3.3375034	0.1284461	0.2582054	0.1933258	up
A_22_P00003343	lnc-CBFA2T3-2	-1.640024	-1.445746	-1.447945	0.1942782	0.1920795	0.1931789	up
A_23_P164228	ATP5G1	5.8551254	6.0026727	6.093793	0.1475473	0.2386675	0.1931074	up
A_24_P349207	LOC441081	-0.742503	-0.400448	-0.698351	0.3420544	0.0441513	0.1931028	up
A_24_P226008	MGLL	4.5394316	4.8947787	4.5700827	0.3553472	0.0306511	0.1929991	up
A_33_P3295803	RAB2B	-0.882328	-0.751524	-0.627184	0.1308045	0.2551441	0.1929743	up
A_22_P00001253	lnc-ANKRD34B-2	0.3198204	0.500875	0.5246272	0.1810546	0.2048068	0.1929307	up
A_23_P14804	TSPAN3	5.3612194	5.583059	5.5252075	0.2218394	0.1639881	0.1929138	up
A_23_P38830	ZNF552	-1.527851	-1.371691	-1.298204	0.1561604	0.2296467	0.1929035	up
A_33_P3344086	HIST1H2AJ	6.9424524	7.08439	7.1862354	0.1419377	0.243783	0.1928604	up
A_33_P3333177	LOC644083	-3.294805	-3.159937	-3.044026	0.1348672	0.2507782	0.1928227	up
A_23_P29079	PFKL	2.7670174	3.036138	2.8832388	0.2691207	0.1162214	0.1926711	up
A_24_P56467	GMPR2	4.242774	4.1957927	4.675062	-0.046981	0.4322882	0.1926534	up
A_23_P26583	NLRC5	-1.256389	-1.188159	-0.939374	0.0682302	0.3170142	0.1926222	up
A_24_P74571	CBY1	-0.040884	0.2133322	0.0901194	0.2542167	0.1310039	0.1926103	up
A_23_P53018	HRASLS5	-0.048072	0.144341	0.1447353	0.1924129	0.1928072	0.19261	up
A_33_P3346473	PHF21A	0.1076651	0.021687	0.5788369	-0.085978	0.4711719	0.1925969	up
A_33_P3337144	ZNF704	-1.975483	-1.598333	-1.967496	0.3771501	0.007987	0.1925685	up
A_33_P3369213	LINC00999	-2.685072	-2.182769	-2.802288	0.5023038	-0.117216	0.192544	up
A_23_P15402	SAT2	4.2926025	4.5998893	4.3703737	0.3072867	0.0777712	0.192529	up
A_24_P159948	ARFRP1	0.3476496	0.5175605	0.5626798	0.1699109	0.2150302	0.1924706	up
A_33_P3351259	LOC100130560	-1.665628	-1.58895	-1.357497	0.0766778	0.3081303	0.192404	up
A_33_P3314813	C11orf94	-1.53037	-1.336628	-1.339312	0.1937423	0.1910586	0.1924005	up
A_33_P3317825	NRAS	-0.290578	-0.131168	-0.065224	0.1594105	0.2253547	0.1923826	up
A_23_P62588	CALML6	-3.404539	-3.131199	-3.29317	0.2733407	0.1113696	0.1923552	up
A_21_P0014312	SNHG19	6.4473267	6.458427	6.820822	0.0111003	0.3734951	0.1922977	up
A_33_P3314276	ASPHD1	4.4466124	4.5125957	4.7651777	0.0659833	0.3185654	0.1922743	up
A_23_P309803	ZNF777	2.8079748	3.0092187	2.991232	0.2012439	0.1832571	0.1922505	up
A_23_P371129	SLX4	1.0336995	1.2092528	1.2425709	0.1755533	0.2088714	0.1922123	up

A_22_P00023552	LOC101929021	-0.600386	-0.306604	-0.510202	0.2937822	0.0901837	0.191983	up
A_23_P104804	ZBTB16	-2.275735	-1.921812	-2.246051	0.3539228	0.0296841	0.1918035	up
A_23_P103532	GPR161	-1.040591	-0.823474	-0.874126	0.2171164	0.1664653	0.1917908	up
A_23_P321959	SFT2D1	4.588416	4.755672	4.8047047	0.1672559	0.2162886	0.1917722	up
A_23_P165494	GORASP2	5.204529	5.3725266	5.420005	0.1679978	0.215476	0.1917369	up
A_33_P3239122	PPIH	-1.910614	-1.552837	-1.885102	0.3577766	0.0255113	0.191644	up
A_22_P00003920	CES2	-0.01167	0.2630172	0.0969195	0.2746873	0.1085897	0.1916385	up
A_33_P3362371	RTN3	0.8398681	1.1500397	0.9128609	0.3101716	0.0729928	0.1915822	up
A_33_P3377261	RNF7	3.5747147	3.6981435	3.834384	0.1234288	0.2596693	0.1915491	up
A_21_P0000284	SNORD52	0.266304	0.481575	0.4340439	0.215271	0.1677399	0.1915054	up
A_33_P3239634	NFYA	-0.903155	-0.860405	-0.562984	0.0427499	0.3401704	0.1914601	up
A_21_P0014059	PIGC	-2.995378	-2.879854	-2.728168	0.1155248	0.2672103	0.1913675	up
A_33_P3258320	ZNF562	-0.20514	0.1234756	-0.151036	0.3286157	0.0541039	0.1913598	up
A_33_P3319161	FXN	3.0168695	3.305328	3.1110516	0.2884584	0.094182	0.1913202	up
A_33_P3236310	NOBOX	0.6938686	0.917181	0.8531504	0.2233124	0.1592817	0.1912971	up
A_23_P62634	RHCE	-1.585059	-1.316865	-1.470752	0.2681942	0.1143069	0.1912506	up
A_33_P3280418	MGC72080	2.4473257	2.8647246	2.4123678	0.4173989	-0.034958	0.1912205	up
A_33_P3285092	LOC100129763	-2.170805	-1.778074	-2.181105	0.3927302	-0.0103	0.191215	up
A_23_P67162	PIN1	3.7255936	3.8512273	3.9823217	0.1256337	0.2567282	0.1911809	up
A_22_P00003135	lnc-C9orf147-1	-2.24133	-2.213631	-1.886787	0.0276992	0.3545432	0.1911212	up
A_23_P140960	NDUFAB1	7.227977	7.3978486	7.4403105	0.1698718	0.2123337	0.1911027	up
A_33_P3413188	LINC00087	-0.131299	-0.041102	0.1606431	0.0901971	0.2919421	0.1910696	up
A_23_P25835	FNTB	2.052289	2.297048	2.1896229	0.2447591	0.1373339	0.1910465	up
A_24_P336276	SLCO3A1	0.5264235	0.7546544	0.6801171	0.228231	0.1536937	0.1909623	up
A_33_P3529860	KDM2B	0.4955549	0.6640191	0.7089624	0.1684642	0.2134075	0.1909359	up
A_24_P220897	SPATA24	-2.033331	-1.720791	-1.96409	0.3125396	0.0692406	0.1908901	up
A_32_P186981	RPL17	7.891941	8.116388	8.04925	0.2244473	0.1573086	0.1908779	up
A_23_P164035	PSMC5	3.773014	3.943707	3.9839697	0.1706929	0.2109556	0.1908243	up
A_23_P146644	ANXA2	9.254221	9.499226	9.390768	0.2450047	0.1365471	0.1907759	up
A_22_P00018545	lnc-TM6SF1-2	0.6243882	0.8397613	0.7905316	0.215373	0.1661434	0.1907582	up
A_23_P214079	SPINK1	0.6978183	1.6890149	0.0881381	0.9911966	-0.60968	0.1907582	up
A_33_P3386726	PI4KB	2.4262047	2.7625127	2.4713717	0.336308	0.045167	0.1907375	up
A_33_P3326349	SMG5	-0.060706	0.2055287	0.0544095	0.2662344	0.1151152	0.1906748	up
A_23_P62907	ATF6	2.9235306	3.0710654	3.157341	0.1475349	0.2338104	0.1906726	up
A_23_P201979	CREM	-0.382479	-0.071823	-0.311878	0.3106561	0.0706015	0.1906288	up
A_21_P0000845	MIPEPP3	-1.572791	-1.487968	-1.276372	0.0848227	0.2964187	0.1906207	up
A_21_P0002208	lnc-PRKCE-1	-2.248936	-1.86692	-2.249787	0.3820157	-0.000851	0.1905822	up
A_33_P3306217	TRIM39	-1.732788	-1.595884	-1.488637	0.1369042	0.2441506	0.1905274	up
A_32_P93328	NDUFB2-AS1	-0.572005	-0.400179	-0.362891	0.1718254	0.2091141	0.1904697	up
A_33_P3281033	MACROD2	-1.01733	-0.806712	-0.847218	0.2106185	0.1701126	0.1903656	up
A_22_P00005135	LOC440117	-2.485737	-2.341286	-2.249489	0.1444507	0.236248	0.1903493	up
A_24_P26177	VPRBP	-0.712799	-0.326647	-0.718269	0.3861518	-0.005471	0.1903405	up
A_23_P28598	DLX2	-3.137135	-2.637784	-3.255863	0.4993508	-0.118727	0.1903117	up
A_23_P108662	MOGS	5.4941025	5.680633	5.688184	0.1865306	0.1940813	0.1903059	up
A_32_P100430	LOC101927974	0.6844239	0.7351027	1.0143452	0.0506787	0.3299213	0.1903	up
A_22_P00004279	ALG14	1.3581233	1.5899863	1.5067945	0.231863	0.1486712	0.1902671	up
A_33_P3265855	PRR5-ARHGAP8	3.2379065	3.443215	3.4130983	0.2053084	0.1751919	0.1902502	up
A_33_P3415820	THBS1	-1.787686	-1.593448	-1.601635	0.1942387	0.1860519	0.1901453	up
A_33_P3413987	SERPING1	-2.599328	-2.358664	-2.459887	0.2406647	0.139441	0.1900529	up
A_33_P3274478	UNC119B	-1.559078	-1.438045	-1.300018	0.1210327	0.2590599	0.1900463	up
A_23_P141965	HAUS8	4.3938637	4.650724	4.517027	0.2568603	0.1231632	0.1900117	up
A_33_P3390477	MRPL4	6.637003	6.7061515	6.9478426	0.0691485	0.3108397	0.1899941	up
A_33_P3274768	LOC100132790	-0.578306	-0.446456	-0.33028	0.1318498	0.2480259	0.1899378	up
A_23_P334709	FKBP9	5.618288	6.0290093	5.587393	0.4107213	-0.030895	0.189913	up
A_24_P319364	F11R	6.428296	6.656813	6.579526	0.2285171	0.1512299	0.1898735	up
A_23_P409386	SLC25A22	3.8822699	3.9847646	4.159481	0.1024947	0.2772112	0.189853	up

A_22_P00016991	lnc-TTC26-2	-0.504627	-0.26375	-0.365868	0.2408772	0.1387591	0.1898181	up
A_23_P203737	INTS4	3.3262053	3.5822015	3.4497461	0.2559962	0.1235409	0.1897686	up
A_21_P0009287	lnc-FSCN2-1	-1.309355	-1.281585	-0.957711	0.0277696	0.351644	0.1897068	up
A_23_P148308	RBM3	3.898857	4.1943088	3.9825773	0.2954516	0.0837202	0.1895859	up
A_33_P3370424	NOTCH1	0.7873092	0.9576683	0.9960351	0.1703591	0.2087259	0.1895425	up
A_23_P212715	CBLB	0.5679169	0.9545884	0.5599661	0.3866715	-0.007951	0.1893604	up
A_23_P128706	DYNC1H1	5.1056557	5.445868	5.144107	0.3402124	0.0384512	0.1893318	up
A_23_P51884	PUSL1	4.6756916	4.8052382	4.924802	0.1295466	0.2491102	0.1893284	up
A_23_P92093	CELSR3	-2.083221	-1.629545	-2.158301	0.4536753	-0.075081	0.1892972	up
A_23_P101332	KRI1	4.0220623	4.153605	4.2690887	0.1315427	0.2470264	0.1892846	up
A_22_P00007695	lnc-HIST2H2AA3-1	-0.641768	-0.333581	-0.571472	0.3081875	0.0702963	0.1892419	up
A_23_P203891	NCOR2	7.1019506	7.16388	7.418477	0.0619292	0.3165264	0.1892278	up
A_33_P3262181	APOBEC3F	1.5671158	1.647069	1.8655758	0.0799532	0.29846	0.1892066	up
A_23_P71889	ODF2	-0.121639	0.0569282	0.0780258	0.1785674	0.1996651	0.1891162	up
A_21_P0007223	LOC101927056	-0.352985	-0.121748	-0.206228	0.2312365	0.1467571	0.1889968	up
A_32_P101699	LOC729887	2.3586445	2.4132266	2.6819372	0.0545821	0.3232927	0.1889374	up
A_23_P43150	ZHX1	0.2782807	0.7858815	0.1484108	0.5076008	-0.12987	0.1888654	up
A_22_P00004443	lnc-COX6C-1	-2.459775	-2.220056	-2.321823	0.2397189	0.1379519	0.1888354	up
A_24_P189739	DUSP16	-1.202034	-1.110042	-0.916558	0.0919914	0.2854753	0.1887333	up
A_23_P326844	NOP9	-1.628789	-1.609962	-1.270319	0.0188279	0.3584704	0.1886492	up
A_33_P3398583	LINC00951	-2.618229	-2.223077	-2.636345	0.3951514	-0.018116	0.1885176	up
A_33_P3360753	KCNQ2	-1.658283	-1.43488	-1.505039	0.223403	0.153244	0.1883235	up
A_23_P360874	LRWD1	4.7507305	4.864865	5.013195	0.1141343	0.2624645	0.1882994	up
A_33_P3346826	IL32	-0.974213	-0.501988	-1.069898	0.4722247	-0.095685	0.1882701	up
A_33_P3405789	TULP3	2.8504915	2.936358	3.1411266	0.0858665	0.2906351	0.1882508	up
A_23_P209183	COLGALT1	2.7965279	2.9335628	3.0359573	0.1370349	0.2394295	0.1882322	up
A_33_P3238433	ALDH3A1	4.6469555	4.76603	4.9043074	0.1190743	0.2573519	0.1882131	up
A_23_P16798	PFN4	-2.795856	-2.289375	-2.926019	0.5064807	-0.130163	0.1881589	up
A_33_P3377005	LMNTD2	0.6386428	0.9371729	0.7163892	0.2985301	0.0777464	0.1881382	up
A_23_P120056	RTKN	2.4534454	2.497624	2.7855034	0.0441785	0.332058	0.1881182	up
A_32_P6172	LINC01003	-0.095243	0.0663915	0.1192927	0.161634	0.2145352	0.1880846	up
A_24_P20630	LEF1	2.3812437	2.1281152	3.0105143	-0.253129	0.6292706	0.188071	up
A_32_P133670	ANP32A	1.8938589	2.101954	2.0618248	0.2080951	0.1679659	0.1880305	up
A_22_P00018268	LOC101928824	-2.166038	-1.821097	-2.135	0.3449407	0.0310378	0.1879892	up
A_33_P3313595	CDK11B	3.2956266	3.450292	3.5169249	0.1546655	0.2212982	0.1879818	up
A_21_P0003167	lnc-TPRA1-2	-2.337422	-2.253	-2.045889	0.0844228	0.291533	0.1879779	up
A_23_P320658	BUB3	4.706991	4.9393334	4.850545	0.2323422	0.1435537	0.187948	up
A_23_P170352	MRPL12	4.830598	4.9347873	5.1022377	0.1041894	0.2716398	0.1879146	up
A_23_P2066	APIP	-0.582817	-0.377942	-0.411868	0.2048755	0.170949	0.1879122	up
A_22_P00009454	LOC101927668	-3.095894	-2.77461	-3.041356	0.3212843	0.0545383	0.1879113	up
A_21_P0005579	lnc-TMEM140-1	-2.384898	-1.929636	-2.464407	0.4552627	-0.079508	0.1878772	up
A_24_P637982	C1orf122	5.2628727	5.3133426	5.5880585	0.0504699	0.3251858	0.1878278	up
A_22_P00007954	LOC102724450	-2.940857	-3.212567	-2.293502	-0.27171	0.6473553	0.1878226	up
A_23_P103617	ANXA9	3.8012094	4.1160264	3.861889	0.314817	0.0606794	0.1877482	up
A_24_P398130	USP6NL	2.3172684	2.5449872	2.4649248	0.2277188	0.1476564	0.1876876	up
A_23_P361405	HYAL3	0.8132176	0.901577	1.1000905	0.0883594	0.2868729	0.1876161	up
A_24_P119577	PHACTR4	1.6016083	1.9670987	1.6113334	0.3654904	0.0097251	0.1876078	up
A_24_P4877	ZCRB1	1.9398279	1.9663854	2.2884617	0.0265574	0.3486338	0.1875956	up
A_23_P201047	THBS3	-0.752194	-0.455823	-0.673429	0.296371	0.0787649	0.1875679	up
A_21_P0012400	NUDT16P1	-0.091945	0.0070577	0.1841779	0.0990024	0.2761226	0.1875625	up
A_24_P229536	MIR99AHG	-1.598309	-1.303185	-1.518337	0.2951236	0.0799718	0.1875477	up
A_21_P0011143	XLOC_l2_003732	-2.537843	-2.235735	-2.465093	0.3021076	0.0727491	0.1874284	up
A_33_P3354935	CSF1	-1.260151	-1.042718	-1.102762	0.2174335	0.1573892	0.1874113	up
A_23_P54055	AJUBA	2.156002	2.573011	2.1137295	0.4170089	-0.042273	0.1873682	up
A_23_P140602	NGRN	2.2168236	2.188249	2.6200256	-0.028574	0.4032021	0.1873138	up
A_24_P275873	TBCD	5.201541	5.4292054	5.3483906	0.2276645	0.1468496	0.1872571	up

A_23_P251660	ABCF1	3.5704203	3.658959	3.8562899	0.0885387	0.2858696	0.1872041	up
A_33_P3256550	MST152	-1.424323	-1.27867	-1.195607	0.1456533	0.2287164	0.1871848	up
A_24_P376129	DFNB31	-1.781945	-1.540363	-1.649165	0.2415819	0.1327801	0.187181	up
A_23_P19313	TBP	1.5234408	1.6299834	1.7912564	0.1065426	0.2678156	0.1871791	up
A_21_P0010556	XLOC_l2_000416	-3.416035	-2.964268	-3.493456	0.4517667	-0.077421	0.1871728	up
A_24_P416645	KLK13	-1.165954	-0.961422	-0.996387	0.2045322	0.1695676	0.1870499	up
A_23_P214950	PERP	8.386006	8.72501	8.421089	0.3390036	0.0350828	0.1870432	up
A_23_P31116	ACOT13	4.020109	4.2298265	4.184456	0.2097173	0.1643467	0.187032	up
A_23_P100642	PNMT	1.495233	1.7349672	1.629538	0.2397342	0.134305	0.1870196	up
A_24_P399888	CENPM	2.981083	3.0629334	3.2732162	0.0818505	0.2921333	0.1869919	up
A_23_P129157	NEIL1	0.4205327	0.7154079	0.4996223	0.2948751	0.0790896	0.1869824	up
A_23_P206901	NDE1	5.4250746	5.396501	5.827573	-0.028574	0.4024983	0.1869624	up
A_23_P3355	POLG	3.3738527	3.5188203	3.6027794	0.1449676	0.2289267	0.1869471	up
A_23_P62188	ZC4H2	2.1335087	2.352796	2.2880564	0.2192874	0.1545477	0.1869175	up
A_23_P216017	ENTPD4	-0.080156	0.1239343	0.08954	0.2040906	0.1696963	0.1868935	up
A_22_P00016633	LOC102724564	-1.776008	-1.375633	-1.80265	0.4003749	-0.026642	0.1868665	up
A_24_P127235	BCR	2.4705038	2.615703	2.6989326	0.1451993	0.2284288	0.1868141	up
A_33_P3375170	LOC100131242	-2.465141	-2.28049	-2.276197	0.1846509	0.1889436	0.1867973	up
A_23_P154358	PROM2	2.7931337	2.9587026	3.001048	0.1655688	0.2079144	0.1867416	up
A_23_P139396	C11orf73	5.1384993	5.334295	5.3160496	0.1957955	0.1775503	0.1866729	up
A_33_P3368706	NFYC	3.678667	3.948954	3.7817116	0.270287	0.1030445	0.1866658	up
A_23_P254353	NOXA1	1.1451273	1.478138	1.1854353	0.3330107	0.040308	0.1866593	up
A_23_P130149	ENO3	0.1680875	0.4612956	0.2479515	0.2932081	0.079864	0.1865361	up
A_22_P00016681	lnc-TPPP2-1	-1.744351	-1.902395	-1.213262	-0.158044	0.5310893	0.1865227	up
A_33_P3291877	ARID1B	-0.225407	-0.071554	-0.006253	0.1538529	0.2191534	0.1865032	up
A_21_P0000233	SNORD38A	1.3968511	1.6920595	1.474638	0.2952085	0.0777869	0.1864977	up
A_22_P00005078	lnc-DEXI-1	-2.816939	-2.630069	-2.630907	0.1868696	0.1860316	0.1864506	up
A_22_P00016482	LOC148413	-0.825964	-0.827597	-0.451524	-0.001634	0.3744392	0.1864028	up
A_23_P345564	OPRL1	-3.009442	-3.04802	-2.598075	-0.038578	0.4113662	0.186394	up
A_21_P0010564	XLOC_l2_000471	-3.2721	-2.732492	-3.439021	0.539609	-0.166921	0.186344	up
A_22_P00017805	lnc-ZBTB25-1	-1.687491	-1.551035	-1.451267	0.1364565	0.2362242	0.1863403	up
A_32_P2392	GOLGA8A	-2.311001	-1.78742	-2.461932	0.523581	-0.15093	0.1863254	up
A_23_P127385	MYBPC3	-1.689043	-1.62443	-1.381025	0.0646129	0.3080177	0.1863153	up
A_33_P3295838	C17orf53	-0.948767	-0.722125	-0.802791	0.2266421	0.1459761	0.1863091	up
A_33_P3344618	TFEB	3.459117	3.4692292	3.821619	0.0101123	0.3625021	0.1863072	up
A_33_P7363082	CWC15	3.4454222	3.612183	3.6512747	0.1667609	0.2058525	0.1863067	up
A_24_P297888	MTAP	1.7615719	2.1133018	1.782248	0.3517299	0.0206761	0.186203	up
A_33_P3337277	LOC100129846	-2.380399	-1.928536	-2.459891	0.4518633	-0.079492	0.1861857	up
A_33_P6817068	NDUFA6-AS1	-1.854289	-1.6076	-1.728666	0.2466888	0.1256228	0.1861558	up
A_23_P26687	TMEM186	3.5493631	3.6067948	3.8640518	0.0574317	0.3146887	0.1860602	up
A_33_P3423791	GRHL1	-1.738275	-1.375466	-1.729067	0.3628087	0.0092077	0.1860082	up
A_21_P0012386	LOC101929607	-2.567382	-2.433951	-2.328814	0.1334312	0.2385681	0.1859996	up
A_23_P164179	TOB1	3.2868204	3.2294707	3.7161026	-0.05735	0.4292822	0.1859663	up
A_23_P159956	MID2	0.080617	0.3500996	0.1829615	0.2694826	0.1023445	0.1859136	up
A_33_P3361457	IFNAR2	1.9009142	2.0136056	2.1599731	0.1126914	0.259059	0.1858752	up
A_33_P3260614	PLCB2	1.2349854	1.1354799	1.7061548	-0.099505	0.4711695	0.185832	up
A_32_P41292	VDAC2	6.161134	6.354344	6.339448	0.1932101	0.1783142	0.1857622	up
A_23_P78053	FAM117A	1.7810011	1.9626012	1.9709206	0.1816001	0.1899195	0.1857598	up
A_21_P0004254	lnc-GPBP1-3	-1.841589	-1.772448	-1.539328	0.0691409	0.3022609	0.1857009	up
A_23_P105227	ME3	-0.586762	-0.275949	-0.526244	0.310812	0.0605178	0.1856649	up
A_33_P3251054	RNF38	3.0772552	3.395598	3.1301975	0.3183427	0.0529423	0.1856425	up
A_23_P73511	ARAF	0.8689232	0.8465929	1.262526	-0.02233	0.3936029	0.1856363	up
A_23_P150583	SCGB1A1	-1.570963	-1.438502	-1.332265	0.1324611	0.238698	0.1855795	up
A_23_P13873	MLF2	4.7238655	4.875672	4.943142	0.1518064	0.2192764	0.1855414	up
A_19_P00321781	LOC100506499	-0.766933	-0.635804	-0.527057	0.1311283	0.2398753	0.1855018	up
A_21_P0012320	LOC101929374	-1.760566	-1.445961	-1.704308	0.3146048	0.0562582	0.1854315	up

A_23_P313278	MUC3A	-1.133133	-1.014257	-0.881464	0.1188769	0.2516699	0.1852734	up
A_33_P3296789	HDAC8	0.2783818	0.4986486	0.4283919	0.2202668	0.1500101	0.1851385	up
A_24_P131522	ANTXR1	-0.216768	-0.31143	0.2478976	-0.094662	0.4646654	0.1850018	up
A_23_P42198	HIST1H3G	5.0596886	5.182363	5.3068657	0.1226745	0.2471771	0.1849258	up
A_21_P0007856	lnc-ZNF664-2	-2.450577	-2.199294	-2.332015	0.2512825	0.1185617	0.1849221	up
A_23_P367676	SIN3A	2.185566	2.4208083	2.3201475	0.2352424	0.1345816	0.184912	up
A_23_P218608	EIF5B	2.609724	2.7195773	2.869647	0.1098533	0.259923	0.1848881	up
A_33_P3287611	KRT3	-0.311147	-0.17641	-0.076244	0.134737	0.2349029	0.1848199	up
A_21_P0008276	LOC101927702	-2.762972	-2.761931	-2.394385	0.0010409	0.3685873	0.1848141	up
A_22_P00015401	KMT2E-AS1	2.1051016	2.2723737	2.307372	0.1672721	0.2022705	0.1847713	up
A_23_P135616	STX18	1.7841063	2.0979571	1.839467	0.3138509	0.0553608	0.1846058	up
A_23_P50137	MEX3C	3.3110418	3.5551	3.4361153	0.2440581	0.1250734	0.1845658	up
A_33_P3319572	FAM71E2	-1.474252	-1.188351	-1.391056	0.2859006	0.0831962	0.1845484	up
A_23_P166087	RASSF2	0.1766639	0.4167156	0.3056788	0.2400518	0.129015	0.1845334	up
A_33_P3555368	FLJ26086	0.2351623	0.4158812	0.4235086	0.1807189	0.1883464	0.1845326	up
A_33_P3421275	NKX6-3	-2.917636	-3.012115	-2.454097	-0.094479	0.4635387	0.1845298	up
A_23_P93383	RGL2	1.4744186	1.6862879	1.6314902	0.2118692	0.1570716	0.1844704	up
A_33_P3211078	HMGA2	1.8734846	2.1966367	1.9192619	0.3231521	0.0457773	0.1844647	up
A_23_P46369	RAB13	2.1375484	2.3225627	2.321353	0.1850143	0.1838045	0.1844094	up
A_23_P394064	PTRF	5.2943373	5.4053235	5.5520325	0.1109862	0.2576952	0.1843407	up
A_23_P9523	RBKS	3.142767	3.4547534	3.199254	0.3119865	0.0564871	0.1842368	up
A_19_P00319640	LINC01104	0.1001096	0.2148647	0.3537941	0.1147552	0.2536845	0.1842198	up
A_33_P3388958	SNX32	-2.092207	-1.860088	-1.955948	0.2321191	0.1362591	0.1841891	up
A_22_P00025243	lnc-SMG6-1	0.357295	0.6020184	0.4808674	0.2447233	0.1235724	0.1841478	up
A_21_P0010141	MIR99AHG	1.0177073	1.3524337	1.0511093	0.3347263	0.033402	0.1840641	up
A_24_P218587	MED17	0.6694512	0.7357554	0.9711952	0.0663042	0.301744	0.1840241	up
A_33_P3268507	CEACAM1	-0.984606	-1.001982	-0.599217	-0.017376	0.3853884	0.1840062	up
A_33_P3372859	DDX54	2.6328192	2.6882615	2.945343	0.0554423	0.3125238	0.1839831	up
A_21_P0007425	lnc-TSSC4-2	-2.636441	-2.380451	-2.524612	0.2559893	0.1118288	0.1839091	up
A_33_P3238157	MYO18A	0.9360328	1.1604185	1.0794392	0.2243857	0.1434064	0.1838961	up
A_33_P3394347	CLRN1-AS1	-0.842358	-0.711073	-0.605855	0.1312847	0.2365031	0.1838939	up
A_23_P64837	SMAGP	2.6280184	2.8928018	2.7308683	0.2647834	0.10285	0.1838167	up
A_22_P00003497	lnc-CCDC90B-1	0.6682854	0.875865	0.8283019	0.2075796	0.1600165	0.1837981	up
A_32_P222149	LRRC72	-3.460487	-3.150677	-3.402779	0.3098099	0.0577076	0.1837587	up
A_33_P3266410	MAZ	-1.077371	-0.698276	-1.08909	0.3790951	-0.011719	0.1836879	up
A_33_P3305487	REM2	-1.267581	-1.323099	-0.844737	-0.055519	0.4228439	0.1836627	up
A_22_P00016017	LOC101928117	-2.106537	-1.835784	-2.010006	0.2707534	0.0965309	0.1836421	up
A_33_P3325914	TAPBP	0.3189006	0.5110612	0.4938445	0.1921606	0.1749439	0.1835523	up
A_33_P3363425	FRMD3	-2.129987	-2.109845	-1.783057	0.0201421	0.3469305	0.1835363	up
A_33_P3410700	ATP8	7.304492	7.5810885	7.3949575	0.2765966	0.0904655	0.183531	up
A_24_P328969	YIPF5	1.3323989	1.6046734	1.427001	0.2722745	0.0946021	0.1834383	up
A_22_P00013169	lnc-RNF13-2	0.8909864	1.2886686	0.8599329	0.3976822	-0.031054	0.1833143	up
A_21_P0003828	lnc-TBC1D19-4	-1.224759	-0.838089	-1.244924	0.3866696	-0.020164	0.1832526	up
A_32_P43050	FRG1	4.058094	4.176868	4.3057604	0.1187739	0.2476664	0.1832202	up
A_21_P0000022	BRI3	0.7424636	0.9195733	0.9317513	0.1771097	0.1892877	0.1831987	up
A_23_P87310	LMO1	-3.445369	-3.051994	-3.472719	0.3933744	-0.027351	0.1830118	up
A_23_P141479	SUPT6H	2.753728	2.958755	2.914585	0.2050271	0.1608572	0.1829422	up
A_24_P14634	EMID1	-0.237233	-0.22194	0.1132507	0.0152936	0.3504839	0.1828887	up
A_24_P232696	SMARCD1	3.1252174	3.1472316	3.4689112	0.0220141	0.3436937	0.1828539	up
A_32_P185317	MNX1-AS1	3.295106	3.297132	3.658764	0.0020261	0.363658	0.182842	up
A_23_P389102	MYO1D	2.728302	2.760776	3.0613556	0.032474	0.3330536	0.1827638	up
A_32_P163858	SCD	3.104968	3.2002778	3.375125	0.0953097	0.2701569	0.1827333	up
A_22_P00011449	lnc-PANK4-2	-2.614336	-2.436337	-2.426911	0.1779993	0.1874247	0.182712	up
A_24_P401637	LOC100130856	0.9745774	0.9958224	1.3186607	0.021245	0.3440833	0.1826642	up
A_23_P330908	DERL1	1.7443619	1.8891544	1.9648046	0.1447926	0.2204428	0.1826177	up
A_23_P252335	MIS18A	3.428791	3.488995	3.7337904	0.060204	0.3049994	0.1826017	up

A_23_P420417	TLCD1	2.8770132	3.1877437	2.9314709	0.3107305	0.0544577	0.1825941	up
A_22_P00015797	lnc-TAS1R2-1	-1.20479	-0.904202	-1.140244	0.3005877	0.0645461	0.1825669	up
A_21_P0013574	MTHFD1L	2.614623	3.039927	2.5542421	0.4253039	-0.060381	0.1824615	up
A_24_P258955	ZNF317	3.0226269	3.14117	3.2689953	0.1185432	0.2463684	0.1824558	up
A_24_P333733	ATP6V0A1	5.9793644	6.0960946	6.227377	0.1167302	0.2480125	0.1823714	up
A_24_P315921	lnc-PABPC4-2	0.6254768	1.0327749	0.5826807	0.4072981	-0.042796	0.182251	up
A_23_P153745	IFI30	5.4593897	5.6016555	5.6815844	0.1422658	0.2221947	0.1822302	up
A_33_P3397288	EDN3	-2.494702	-2.178904	-2.446042	0.3157973	0.0486598	0.1822286	up
A_22_P00021652	LOC101927963	-2.431152	-2.066777	-2.43112	0.3643746	3.195E-05	0.1822033	up
A_33_P3344229	HIST1H4A	3.9839735	4.1570497	4.1752844	0.1730762	0.1913109	0.1821935	up
A_33_P3272773	ZNF217	2.009677	2.130735	2.252945	0.121058	0.243268	0.182163	up
A_23_P102404	CCT7	7.2156973	7.33948	7.4562073	0.1237826	0.24051	0.1821463	up
A_23_P215060	PODXL	5.40487	5.7175727	5.456423	0.3127027	0.0515528	0.1821277	up
A_23_P66180	CACNG3	-2.931525	-2.841178	-2.657729	0.0903468	0.2737961	0.1820714	up
A_23_P218807	ZC3H7B	1.9410448	1.9148755	2.331314	-0.026169	0.3902693	0.18205	up
A_33_P3419733	DNAJC5	0.6739082	0.9330726	0.7788014	0.2591643	0.1048932	0.1820288	up
A_21_P0000100	MTRNR2L1	9.1539345	9.358728	9.313138	0.2047939	0.1592035	0.1819987	up
A_33_P3290082	HPS4	-1.774258	-1.464721	-1.720057	0.3095369	0.0542007	0.1818688	up
A_23_P88703	TP53BP1	2.8302078	3.0739608	2.9501257	0.243753	0.1199179	0.1818354	up
A_32_P179740	ATXN7L1	-1.186845	-1.152102	-0.858138	0.0347433	0.3287072	0.1817253	up
A_32_P725218	METTL2A	0.5898714	0.8823624	0.6608238	0.292491	0.0709524	0.1817217	up
A_23_P203743	GAB2	-0.151343	0.2741351	-0.213414	0.4254785	-0.06207	0.181704	up
A_33_P3294404	AKIRIN1	1.6873007	1.8105593	1.9274445	0.1232586	0.2401438	0.1817012	up
A_22_P00016082	lnc-THBS3-1	-3.42506	-3.108903	-3.377942	0.3161569	0.0471177	0.1816373	up
A_23_P106481	EMC4	5.226753	5.3824544	5.4342947	0.1557012	0.2075415	0.1816213	up
A_23_P11025	ZNF185	2.9840956	3.4341588	2.8971968	0.4500632	-0.086899	0.1815822	up
A_33_P3372466	TECR	4.2172394	4.2606883	4.5369396	0.0434489	0.3197002	0.1815746	up
A_23_P210538	ELMO2	-1.982666	-2.026471	-1.575879	-0.043805	0.4067869	0.1814909	up
A_24_P65864	NSFL1C	2.0065117	2.1677241	2.2082233	0.1612124	0.2017117	0.181462	up
A_23_P122876	TAF6	5.1875687	5.4407496	5.2972765	0.253181	0.1097078	0.1814444	up
A_23_P165952	ACTR5	0.4321613	0.6251864	0.60185	0.1930251	0.1696887	0.1813569	up
A_23_P251945	DCTN4	2.9863777	3.2772985	3.0581465	0.2909207	0.0717688	0.1813447	up
A_33_P3324086	MCUR1	4.9343386	5.0284305	5.2029104	0.0940919	0.2685719	0.1813319	up
A_33_P3388636	LOC643549	-2.951688	-2.852249	-2.688602	0.0994384	0.2630863	0.1812624	up
A_24_P21887	PLCH2	-2.078497	-1.895271	-1.8992	0.1832256	0.1792965	0.1812611	up
A_33_P3221563	ARMC5	3.9843311	4.0084853	4.322654	0.0241542	0.3383226	0.1812384	up
A_33_P3400547	MED21	2.1818924	2.1678262	2.558343	-0.014066	0.3764505	0.1811922	up
A_23_P6596	HES1	0.9644399	1.139852	1.1513882	0.1754122	0.1869483	0.1811802	up
A_19_P00320954	LINC00211	-0.099424	0.2051444	-0.041645	0.3045683	0.0577793	0.1811738	up
A_21_P0001306	lnc-PRKAA2-1	-3.11291	-2.712633	-3.151031	0.4002769	-0.038121	0.181078	up
A_33_P3242863	NT5M	-0.001589	0.2180853	0.1408224	0.2196746	0.1424117	0.1810432	up
A_23_P6836	IP6K2	-1.297031	-0.930174	-1.301814	0.3668575	-0.004783	0.1810374	up
A_24_P336113	CABIN1	1.4598966	1.5665245	1.714869	0.1066279	0.2549725	0.1808002	up
A_23_P162746	CRYL1	1.7326956	1.6936646	2.1333113	-0.039031	0.4006157	0.1807923	up
A_23_P405148	MFF	6.1438684	6.258508	6.3906918	0.1146398	0.2468233	0.1807315	up
A_33_P3399433	C20orf27	7.3793726	7.4429336	7.6770763	0.063561	0.2977037	0.1806324	up
A_23_P142708	LMAN2L	2.7110424	2.9126935	2.8705025	0.2016511	0.1594601	0.1805556	up
A_23_P132669	GLT8D1	1.6787558	1.8619146	1.8566847	0.1831589	0.1779289	0.1805439	up
A_23_P29257	H1F0	4.777293	4.8444943	5.071024	0.0672011	0.2937307	0.1804659	up
A_23_P399001	CXXC5	5.3098574	5.564365	5.4161434	0.2545075	0.1062861	0.1803968	up
A_33_P3408757	FOXO6	3.1194277	3.286755	3.3128633	0.1673274	0.1934357	0.1803815	up
A_33_P3325009	RGS4	8.670874	8.895021	8.80748	0.2241478	0.1366062	0.180377	up
A_33_P3303136	SERPINB6	4.5583076	4.826609	4.65075	0.2683015	0.0924425	0.180372	up
A_23_P76538	TESC	7.8080177	7.8529525	8.123814	0.0449348	0.3157959	0.1803653	up
A_23_P145289	GNL1	1.9338427	2.173121	2.055273	0.2392783	0.1214304	0.1803544	up
A_21_P0000613	TFAP2A-AS1	-2.165537	-1.7849	-2.185548	0.3806377	-0.020011	0.1803134	up

A_33_P3356325	ETV6	-0.54506	-0.278114	-0.451702	0.2669458	0.0933576	0.1801517	up
A_33_P3322288	AZI2	0.3644285	0.6266589	0.4624739	0.2622304	0.0980454	0.1801379	up
A_24_P144601	POU5F1	-2.883692	-2.415243	-2.992055	0.4684496	-0.108363	0.1800433	up
A_24_P58337	FTH1	8.915	9.3850765	8.804888	0.4700766	-0.110112	0.1799822	up
A_22_P00003244	lnc-CADPS-1	1.9335318	2.2310328	1.9959011	0.2975011	0.0623693	0.1799352	up
A_23_P211106	SETD4	-0.486833	-0.30028	-0.313567	0.186553	0.1732659	0.1799095	up
A_23_P100704	MAPK7	1.0308399	1.3296614	1.0917482	0.2988215	0.0609083	0.1798649	up
A_22_P00004325	SLC26A4-AS1	-2.895919	-2.664477	-2.767669	0.231442	0.1282508	0.1798464	up
A_23_P142187	HIF3A	-2.60622	-2.262121	-2.59074	0.3440993	0.0154796	0.1797894	up
A_33_P3299761	KRT23	4.7117147	4.9528956	4.829789	0.2411809	0.1180744	0.1796277	up
A_33_P3246163	RPL5	9.491674	9.7460575	9.596475	0.2543831	0.1048002	0.1795917	up
A_33_P3240543	AGAP3	3.034356	2.8758798	3.5520067	-0.158476	0.5176506	0.1795871	up
A_23_P76851	PRMT5	4.053235	4.2938905	4.1716537	0.2406554	0.1184187	0.1795371	up
A_23_P23102	ZSCAN20	0.4659276	0.7694821	0.5212259	0.3035545	0.0552983	0.1794264	up
A_23_P169112	CPSF1	1.1665316	1.4050074	1.2867312	0.2384758	0.1201997	0.1793377	up
A_24_P184388	LRTOMT	-0.147517	-0.036647	0.1001978	0.1108699	0.247715	0.1792924	up
A_23_P40936	NR2C2	2.0467129	2.3092713	2.1426687	0.2625585	0.0959559	0.1792572	up
A_23_P106998	MRPS23	3.3158836	3.378159	3.6121225	0.0622754	0.2962389	0.1792572	up
A_22_P00016721	lnc-TRAPPC6B-1	-1.79108	-1.430702	-1.793082	0.3603778	-0.002002	0.1791878	up
A_33_P3341901	TSR2	4.3818607	4.4082437	4.713849	0.0263829	0.3319883	0.1791856	up
A_23_P210900	ACSS2	1.3886828	1.3605967	1.7749171	-0.028086	0.3862343	0.179074	up
A_23_P380766	CDC42BPB	4.0384665	4.3043027	4.130763	0.2658362	0.0922966	0.1790664	up
A_24_P100673	EMC4	5.809289	5.947208	6.0294914	0.137919	0.2202025	0.1790607	up
A_24_P49260	SPTLC3	0.1771207	0.5864739	0.1257768	0.4093533	-0.051344	0.1790047	up
A_24_P152188	PRICKLE2	-2.0325	-2.162706	-1.544469	-0.130206	0.4880314	0.1789128	up
A_23_P363406	ANGEL1	-0.112575	-0.024883	0.1575561	0.0876918	0.2701311	0.1789114	up
A_21_P0000036	RDM1	-0.561584	-0.309386	-0.456124	0.2521977	0.1054592	0.1788285	up
A_22_P00022693	SH3TC1	0.4819942	0.6378851	0.6837435	0.1558909	0.2017493	0.1788201	up
A_22_P00024845	lnc-HSPB6-1	-0.156638	0.051806	-0.007467	0.2084436	0.1491704	0.178807	up
A_22_P00022232	RCL1	4.5188265	4.743153	4.652066	0.2243266	0.1332398	0.1787832	up
A_22_P00020726	lnc-ZNF8-1	2.7671213	2.8820071	3.0097656	0.1148858	0.2426443	0.1787651	up
A_33_P3271634	HLA-DPB1	-3.245171	-2.742698	-3.390169	0.5024726	-0.144998	0.1787373	up
A_24_P169688	MICB	-0.332154	-0.212651	-0.094206	0.119503	0.2379479	0.1787255	up
A_24_P71373	SLC9A1	-0.868235	-0.514934	-0.864171	0.3533015	0.0040646	0.178683	up
A_23_P214330	SERPINB1	5.816394	6.113706	5.876362	0.2973123	0.059968	0.1786401	up
A_23_P128919	LGALS3	7.832431	8.145527	7.876601	0.3130961	0.0441704	0.1786332	up
A_22_P00016183	lnc-TLE3-6	-0.8504	-0.718859	-0.624931	0.1315417	0.2254696	0.1785057	up
A_23_P101707	PLIN3	0.3019862	0.5635929	0.3972988	0.2616067	0.0953126	0.1784596	up
A_33_P3416772	C1orf159	1.603817	1.6943588	1.8700705	0.0905418	0.2662535	0.1783977	up
A_22_P00005063	lnc-DENND5A-1	0.1913023	0.5593991	0.1799741	0.3680968	-0.011328	0.1783843	up
A_23_P138725	MARVELD1	5.4623985	5.346967	5.934577	-0.115431	0.4721785	0.1783736	up
A_23_P12363	ROR1	2.8528032	3.071094	2.9912071	0.2182908	0.1384039	0.1783473	up
A_23_P49220	TCF25	2.7064152	2.9477515	2.8217344	0.2413364	0.1153193	0.1783278	up
A_23_P18604	LAP3	3.0967817	3.1145864	3.4356155	0.0178046	0.3388338	0.1783192	up
A_24_P76740	CC2D1B	1.2866578	1.6088247	1.321096	0.3221669	0.0344381	0.1783025	up
A_23_P169017	DEFB103B	-0.73658	0.3245549	-1.441129	1.0611353	-0.704549	0.1782932	up
A_23_P398044	CCDC97	4.963175	5.1172385	5.16564	0.1540637	0.2024651	0.1782644	up
A_33_P3216292	TBC1D2	5.8222246	5.9475064	6.053426	0.1252818	0.2312012	0.1782415	up
A_23_P132263	EIF4ENIF1	2.6854496	2.8760467	2.851327	0.1905971	0.1658773	0.1782372	up
A_23_P36689	LRRC23	0.3913631	0.5568504	0.5823102	0.1654873	0.1909471	0.1782172	up
A_33_P3317576	DHFRL1	-0.034933	0.0356202	0.2509189	0.0705528	0.2858515	0.1782022	up
A_23_P39561	UBE2F	2.4241343	2.531201	2.6734457	0.1070666	0.2493115	0.178189	up
A_33_P3315284	KRT74	-1.725799	-2.013181	-1.082085	-0.287382	0.643714	0.1781659	up
A_22_P00014841	lnc-SLC48A1-1	-0.616969	-0.446429	-0.431351	0.1705399	0.1856184	0.1780791	up
A_24_P225534	RHBDL2	0.7985692	1.0176392	0.9356427	0.21907	0.1370735	0.1780717	up
A_22_P00022599	LINC01526	-2.313465	-2.31014	-1.960666	0.0033243	0.3527985	0.1780614	up

A_33_P3240318	PITX3	1.7351885	1.9606528	1.865613	0.2254643	0.1304245	0.1779444	up
A_21_P0010927	LINC00502	-0.695384	-0.548374	-0.486575	0.1470094	0.2088089	0.1779091	up
A_23_P337201	SRP72	6.079755	6.190042	6.325266	0.1102872	0.2455111	0.1778991	up
A_33_P3388745	LOC100132207	-1.847535	-1.814609	-1.524676	0.0329261	0.3228588	0.1778924	up
A_22_P00007714	lnc-HLA-DMA-1	-0.569361	-0.466703	-0.316249	0.1026583	0.2531123	0.1778853	up
A_23_P408376	HSPA12A	3.562357	3.6182475	3.862174	0.0558906	0.2998171	0.1778538	up
A_23_P101111	CTDP1	0.8592057	1.0675383	1.0065303	0.2083325	0.1473246	0.1778286	up
A_32_P506600	RAN	9.033244	9.218531	9.203498	0.1852865	0.1702538	0.1777701	up
A_33_P3854030	MBD5	-2.582357	-2.530931	-2.278254	0.0514257	0.3041024	0.1777641	up
A_23_P62868	EXOSC10	3.545289	3.6660676	3.7799711	0.1207786	0.2346821	0.1777303	up
A_23_P502343	ADAM33	-1.438912	-1.356275	-1.166158	0.0826378	0.2727547	0.1776962	up
A_33_P3324692	ALG1L2	1.3375607	1.3885198	1.6419468	0.0509591	0.3043861	0.1776726	up
A_23_P85180	TMEM187	-0.265206	0.108995	-0.284487	0.3742013	-0.01928	0.1774604	up
A_23_P103661	YY1AP1	3.9927063	4.2381806	4.102021	0.2454743	0.1093149	0.1773946	up
A_21_P0014334	LOC101928557	-1.126747	-0.965199	-0.933536	0.1615481	0.1932106	0.1773794	up
A_23_P166333	UFD1L	5.3977594	5.4613605	5.688879	0.063601	0.2911196	0.1773603	up
A_33_P3315764	TP53	-1.512035	-1.110874	-1.558648	0.4011607	-0.046613	0.1772738	up
A_23_P130040	PHB	4.5412846	4.4928355	4.9442596	-0.048449	0.4029751	0.177263	up
A_22_P00017667	GMDS-AS1	-3.017463	-2.854179	-2.826582	0.1632843	0.1908813	0.1770828	up
A_23_P365149	BCKDK	1.2667241	1.3369246	1.5506792	0.0702004	0.2839551	0.1770778	up
A_21_P0006415	lnc-MBTPS2-1	-1.955218	-1.606484	-1.949811	0.3487339	0.0054073	0.1770706	up
A_33_P3228558	ARHGAP27	6.000348	6.142862	6.2118893	0.1425138	0.2115412	0.1770275	up
A_24_P324640	ZNF544	1.5510345	1.6631913	1.7928638	0.1121569	0.2418294	0.1769931	up
A_21_P0011560	XLOC_l2_005921	-1.199325	-0.943641	-1.101046	0.2556844	0.0982795	0.1769819	up
A_33_P3288659	ACTL8	4.466674	4.557383	4.7298946	0.0907092	0.2632208	0.176965	up
A_33_P3341970	NEGR1	-0.55895	-0.397083	-0.366997	0.1618671	0.1919537	0.1769104	up
A_23_P52207	BAMBI	8.891182	8.8566265	9.27946	-0.034555	0.388278	0.1768613	up
A_24_P288448	RASSF2	-2.408139	-2.348774	-2.113838	0.0593653	0.2943015	0.1768334	up
A_23_P65518	DACT1	-0.207472	0.0615115	-0.122854	0.2689838	0.0846186	0.1768012	up
A_33_P3373560	SPTAN1	1.4322791	1.6992197	1.5188422	0.2669406	0.0865631	0.1767519	up
A_23_P131846	SNAI1	1.5263114	1.647254	1.7587051	0.1209426	0.2323937	0.1766682	up
A_33_P3313221	ZNF691	-1.588859	-1.424845	-1.399545	0.1640143	0.1893139	0.1766641	up
A_21_P0011758	XLOC_l2_007097	-0.05581	0.155489	0.0860925	0.2112989	0.1419025	0.1766007	up
A_33_P3258265	SEMA6C	-0.653196	-0.348465	-0.604904	0.3047314	0.0482922	0.1765118	up
A_33_P3230488	RPS6KB2	5.811141	6.0125337	5.962756	0.2013927	0.1516151	0.1765039	up
A_24_P226278	JADE2	1.3106513	1.2982612	1.6760063	-0.01239	0.365355	0.1764824	up
A_24_P304987	SAP30BP	3.2865067	3.2265897	3.6993027	-0.059917	0.412796	0.1764395	up
A_22_P00004521	lnc-CR392000.1-2	2.4993134	2.6703515	2.6810656	0.1710382	0.1817522	0.1763952	up
A_23_P311192	SPTLC1	-0.55869	0.0627561	-0.827399	0.6214461	-0.268709	0.1763685	up
A_33_P3303577	TMEM110	2.4362097	2.5852447	2.6398687	0.149035	0.2036591	0.176347	up
A_23_P82412	NSUN5	4.008363	4.173244	4.1959047	0.1648812	0.187542	0.1762116	up
A_22_P00007027	lnc-GIT1-1	-1.432086	-1.282886	-1.228951	0.1492	0.203135	0.1761675	up
A_23_P138514	COMMD3	5.929945	6.119761	6.0924187	0.189816	0.1624737	0.1761448	up
A_21_P0003290	lnc-MINA-3	8.051176	8.280598	8.174014	0.2294216	0.122838	0.1761298	up
A_21_P0009212	lnc-PAFAH1B1-2	-2.780678	-2.041014	-3.168135	0.7396636	-0.387457	0.1761032	up
A_32_P6221	FAM86C2P	-0.579574	-0.444734	-0.362405	0.13484	0.2171693	0.1760046	up
A_21_P0006290	lnc-SEMA4D-1	-2.199256	-1.981869	-2.064673	0.2173867	0.134583	0.1759849	up
A_33_P3271111	NINJ1	5.757744	5.837806	6.0296297	0.0800624	0.2718859	0.1759741	up
A_24_P324314	MXRA7	-1.128085	-0.913789	-0.990509	0.2142959	0.1375761	0.175936	up
A_19_P00803850	LINC01539	-1.873532	-1.806774	-1.588487	0.0667577	0.2850452	0.1759014	up
A_23_P6771	LMCD1	1.8241339	2.153151	1.8469038	0.3290172	0.0227699	0.1758935	up
A_24_P245358	ATP5A1	6.1527834	6.250954	6.4062614	0.0981708	0.2534781	0.1758244	up
A_32_P209094	FGGY	1.9924402	2.2712293	2.06524	0.278789	0.0727997	0.1757944	up
A_23_P66402	MED9	1.5405703	1.7754102	1.6571007	0.2348399	0.1165304	0.1756852	up
A_23_P34835	LMNA	6.769932	6.9477906	6.9434423	0.1778588	0.1735106	0.1756847	up
A_33_P3210160	ZNF865	10.346423	10.518921	10.525173	0.1724978	0.17875	0.1756239	up

A_33_P3212575	NNAT	0.0477376	0.2979255	0.1486969	0.2501879	0.1009593	0.1755736	up
A_22_P00020270	lnc-MBLAC1-1	-3.063678	-2.882603	-2.893663	0.1810756	0.1700156	0.1755456	up
A_24_P211565	C1QTNF6	0.7596536	1.1340466	0.7362986	0.374393	-0.023355	0.175519	up
A_24_P278172	ZFAND5	1.5738039	1.8701987	1.6283121	0.2963948	0.0545082	0.1754515	up
A_22_P00010595	lnc-NDST1-1	-0.961201	-0.756467	-0.815115	0.2047339	0.1460857	0.1754098	up
A_21_P0012148	XLOC_l2_008632	0.4179974	0.7315226	0.4550786	0.3135252	0.0370812	0.1753032	up
A_23_P32707	ESPL1	2.6671133	2.8225007	2.8621855	0.1553874	0.1950722	0.1752298	up
A_33_P3215412	C2CD4C	1.8885574	2.020866	2.1065521	0.1323085	0.2179947	0.1751516	up
A_33_P3253460	LOC100130654	2.3188305	2.5527053	2.4351902	0.2338748	0.1163597	0.1751173	up
A_24_P302998	ATP5I	7.140685	7.208259	7.423276	0.067574	0.2825909	0.1750824	up
A_21_P0011802	MSH6	0.1039467	0.4245248	0.1335282	0.3205781	0.0295815	0.1750798	up
A_24_P106953	PTGES2	2.1730995	2.3004537	2.3958158	0.1273542	0.2227163	0.1750352	up
A_33_P3244803	ACOX1	4.1206064	4.1555724	4.4356613	0.034966	0.3150549	0.1750104	up
A_23_P94921	SLC20A2	4.5632963	4.80671	4.669792	0.2434135	0.1064959	0.1749547	up
A_23_P126103	CTH	0.8180294	1.5294247	0.4562359	0.7113953	-0.361794	0.1748009	up
A_23_P102109	TUBA4A	6.1500187	6.0748315	6.574727	-0.075187	0.4247084	0.1747606	up
A_23_P145197	BYSL	4.8522673	5.0024266	5.0516157	0.1501594	0.1993485	0.1747539	up
A_22_P00010128	LOC101927907	-2.718282	-2.570502	-2.516678	0.1477797	0.2016037	0.1746917	up
A_33_P3250398	CTSZ	1.4896216	1.6630001	1.6655111	0.1733785	0.1758895	0.174634	up
A_24_P85317	CHD2	-2.89882	-2.449318	-2.999217	0.4495025	-0.100397	0.1745528	up
A_21_P0000640	LINC00460	-0.671836	-0.676896	-0.317695	-0.00506	0.3541408	0.1745405	up
A_33_P3365002	TUBB2A	2.279543	2.4457421	2.4624157	0.1661992	0.1828728	0.174536	up
A_23_P55127	ADPRM	-0.519277	-0.318001	-0.371678	0.2012763	0.1475987	0.1744375	up
A_23_P123424	CHRNB3	-1.930276	-1.815831	-1.695859	0.1144452	0.234417	0.1744311	up
A_21_P0000254	SNORD31	-0.973388	-0.730359	-0.867642	0.2430296	0.1057463	0.1743879	up
A_23_P87580	ANP32D	1.4822745	1.6939921	1.6193304	0.2117176	0.1370559	0.1743867	up
A_24_P44931	MPND	0.5015445	0.7356653	0.6161847	0.2341209	0.1146402	0.1743805	up
A_33_P3373214	ZHX1-C8orf76	3.1582355	3.0294728	3.6356106	-0.128763	0.477375	0.1743062	up
A_33_P3278868	HEATR5A	0.5761809	0.5516563	0.9489889	-0.024525	0.372808	0.1741416	up
A_23_P138139	OMA1	0.1905241	0.3933668	0.3359561	0.2028427	0.145432	0.1741374	up
A_24_P185394	GSK3A	5.940051	6.121675	6.106661	0.1816239	0.1666098	0.1741169	up
A_24_P116805	STAT3	-0.14307	0.1300211	-0.067978	0.2730913	0.0750918	0.1740916	up
A_22_P00012248	lnc-PPP1R12B-1	-1.434474	-1.070267	-1.450505	0.3642063	-0.016031	0.1740875	up
A_33_P3273230	HORMAD1	-0.700995	-0.399629	-0.65422	0.3013668	0.0467758	0.1740713	up
A_24_P22976	ARRDC2	2.2330017	2.4560866	2.3580313	0.2230849	0.1250296	0.1740572	up
A_23_P43273	EXT1	0.7604427	1.1571383	0.7112594	0.3966956	-0.049183	0.1737561	up
A_23_P202104	PPIF	6.1997223	6.3121476	6.4347706	0.1124253	0.2350483	0.1737368	up
A_21_P0009788	lnc-ZNF793-1	-0.259459	-0.110057	-0.061531	0.1494012	0.197928	0.1736646	up
A_33_P3304501	CDX2	4.6289606	4.8901467	4.715103	0.2611861	0.0861425	0.1736643	up
A_23_P114839	FHL3	0.2029023	0.2858343	0.4672523	0.082932	0.2643499	0.173641	up
A_23_P116942	LAG3	-0.835211	-0.973475	-0.349808	-0.138264	0.4854026	0.1735694	up
A_22_P00018073	lnc-ZNF33A-2	-1.363863	-0.865345	-1.515245	0.498518	-0.151382	0.1735678	up
A_24_P364838	SLC9A3R2	2.0630808	2.2864003	2.1867943	0.2233195	0.1237135	0.1735165	up
A_23_P115922	EIF4EBP2	5.776925	6.156859	5.7439604	0.3799338	-0.032965	0.1734846	up
A_23_P89589	PER1	0.971653	1.2222166	1.0679483	0.2505636	0.0962954	0.1734295	up
A_23_P8380	C7orf49	0.7904301	0.9750919	0.9525652	0.1846619	0.1621351	0.1733985	up
A_23_P140057	TNFRSF19	-1.794859	-1.929996	-1.312941	-0.135136	0.4819188	0.1733913	up
A_23_P207319	MAP3K14	2.9963055	3.0897279	3.2494469	0.0934224	0.2531414	0.1732819	up
A_23_P97990	HTRA1	1.9607029	2.0275931	2.2403622	0.0668902	0.2796593	0.1732748	up
A_22_P00024976	lnc-LAT2-1	-2.391376	-2.029023	-2.407214	0.3623524	-0.015838	0.1732571	up
A_33_P3214456	PDSS2	-0.042951	0.0919528	0.1686134	0.1349039	0.2115645	0.1732342	up
A_21_P0010663	XLOC_l2_001206	1.8129587	1.9438844	2.028494	0.1309257	0.2155352	0.1732304	up
A_33_P3228375	XPO5	-1.878714	-1.796402	-1.614612	0.0823126	0.264102	0.1732073	up
A_24_P60972	TSTD2	0.4230757	0.7768297	0.4156857	0.353754	-0.00739	0.173182	up
A_23_P16157	KHSRP	6.608843	6.7263455	6.837661	0.1175027	0.2288179	0.1731603	up
A_33_P3507542	LEPREL2	0.4339404	0.3743377	0.8398528	-0.059603	0.4059124	0.1731548	up

A_23_P89249	ERBB2	9.606814	9.62105	9.938633	0.0142355	0.3318186	0.173027	up
A_23_P115683	HPS6	6.126565	6.258202	6.340974	0.1316371	0.2144089	0.173023	up
A_21_P0001153	LOC100131564	-0.262703	-0.176814	-0.002678	0.0858889	0.260025	0.1729569	up
A_22_P00011910	IDH1-AS1	-1.200319	-1.282888	-0.771922	-0.082569	0.4283967	0.172914	up
A_23_P18887	MCCC2	1.624567	1.9530711	1.6418443	0.3285041	0.0172772	0.1728907	up
A_24_P83586	MMACHC	0.6815085	0.885313	0.8231912	0.2038045	0.1416826	0.1727436	up
A_33_P3265803	DEFB108B	-2.175264	-1.543724	-2.461423	0.6315408	-0.286159	0.1726911	up
A_33_P3395952	COL20A1	-1.639409	-1.436681	-1.496904	0.2027278	0.1425047	0.1726162	up
A_33_P3494748	TMEM65	0.9528871	0.8696237	1.3813658	-0.083263	0.4284787	0.1726077	up
A_23_P126752	CAPZB	1.8126278	1.9843926	1.9859247	0.1717649	0.1732969	0.1725309	up
A_33_P3261620	GHSR	-1.760719	-1.553715	-1.62272	0.2070041	0.1379991	0.1725016	up
A_23_P102925	PWP2	2.2623863	2.4653115	2.40444	0.2029252	0.1420536	0.1724894	up
A_23_P162087	TMEM9B	4.670928	4.8680587	4.818614	0.1971307	0.147686	0.1724083	up
A_21_P0004989	lnc-GMDS-1	-2.039064	-1.933069	-1.800316	0.1059947	0.2387476	0.1723712	up
A_23_P420361	BRK1	7.4210443	7.550837	7.635992	0.1297927	0.2149477	0.1723702	up
A_23_P4909	SNRNP70	3.8518066	3.9080758	4.1402016	0.0562692	0.2883949	0.1723321	up
A_24_P396720	PPP1CB	3.3471022	3.4335895	3.605195	0.0864873	0.2580929	0.1722901	up
A_23_P112311	TRIM32	2.2164507	2.4275947	2.3498697	0.211144	0.133419	0.1722815	up
A_21_P0000216	SNORD36C	0.9124613	1.1970377	0.9724364	0.2845764	0.0599751	0.1722758	up
A_33_P3397840	WIPF3	-1.358779	-1.219388	-1.15369	0.139391	0.2050886	0.1722398	up
A_21_P0000304	SNORA13	1.4801865	1.5366211	1.7682247	0.0564346	0.2880383	0.1722364	up
A_23_P84775	PLRG1	3.0279846	3.3068156	3.0934696	0.278831	0.065485	0.172158	up
A_19_P00803997	NUP50-AS1	-1.221313	-0.924431	-1.173883	0.2968817	0.04743	0.1721559	up
A_23_P168576	POMZP3	-0.951597	-0.811182	-0.74828	0.1404147	0.2033167	0.1718657	up
A_22_P00013910	SLC37A4	1.2304788	1.240282	1.5642767	0.0098033	0.3337979	0.1718006	up
A_24_P334640	PAQR8	-3.13535	-2.973768	-2.953392	0.161582	0.1819575	0.1717697	up
A_23_P366936	KRT6C	5.6272955	5.5972505	6.0007877	-0.030045	0.3734922	0.1717236	up
A_33_P3312182	PROSER2	-0.290682	-0.086583	-0.151387	0.2040997	0.1392951	0.1716974	up
A_23_P149664	TMEM183B	4.2892475	4.560161	4.361699	0.2709136	0.0724516	0.1716826	up
A_23_P128532	C12orf45	3.810485	3.9463983	4.0178556	0.1359134	0.2073708	0.1716421	up
A_23_P410998	RAB5B	1.1172462	1.2687593	1.3088832	0.1515131	0.191637	0.1715751	up
A_22_P00011743	lnc-PER2-1	-1.268293	-0.971071	-1.222367	0.2972217	0.0459261	0.1715739	up
A_22_P00008304	lnc-ITGAL-3	-2.471457	-2.666518	-1.93328	-0.195062	0.5381765	0.1715573	up
A_24_P21715	RAD9A	1.4598823	1.7427678	1.5200806	0.2828856	0.0601983	0.1715419	up
A_23_P156319	LARP1	6.341647	6.596316	6.429818	0.2546687	0.088171	0.1714199	up
A_33_P3331095	ATXN7L1	-2.172946	-2.302609	-1.700851	-0.129663	0.4720945	0.1712158	up
A_33_P3384432	TFDP2	4.2854185	4.5861936	4.3269978	0.3007751	0.0415792	0.1711771	up
A_23_P2181	CYB5R2	3.8686953	4.097833	3.981862	0.2291379	0.1131668	0.1711524	up
A_24_P74487	SMIM11	1.5658875	1.6886535	1.785408	0.122766	0.2195206	0.1711433	up
A_23_P81859	HIST1H2AH	6.2297916	6.350138	6.451662	0.1203465	0.2218704	0.1711085	up
A_21_P0013016	XLOC_l2_012415	-2.043701	-1.82281	-1.922537	0.2208905	0.1211638	0.1710272	up
A_33_P3318237	PPP2R5B	-1.318902	-1.134065	-1.161714	0.1848369	0.1571879	0.1710124	up
A_23_P29630	SPCS1	5.0078316	5.2064214	5.1511726	0.1985898	0.1433411	0.1709654	up
A_23_P211748	GTF2E1	2.4909506	2.647595	2.6762266	0.1566443	0.185276	0.1709602	up
A_23_P80353	L3MBTL2	0.3894496	0.6018453	0.5188923	0.2123957	0.1294427	0.1709192	up
A_23_P92536	CNGA1	-2.717713	-2.856398	-2.237238	-0.138685	0.4804752	0.170895	up
A_33_P3278293	METTL16	3.317418	3.4273534	3.5492325	0.1099353	0.2318144	0.1708748	up
A_24_P168760	GTPBP1	2.3455296	2.5957856	2.4370222	0.2502561	0.0914927	0.1708744	up
A_23_P164826	RNASEH2A	4.990946	5.140795	5.1826296	0.1498494	0.1916838	0.1707666	up
A_24_P576506	SPRN	-3.028429	-2.807114	-2.908262	0.2213147	0.120167	0.1707408	up
A_22_P00001179	LOC100507530	-0.845325	-0.711849	-0.637419	0.1334758	0.2079053	0.1706905	up
A_19_P00323103	UBE2E3	-0.025096	-0.200372	0.4914522	-0.175276	0.5165482	0.1706359	up
A_33_P3762733	D21S2088E	-1.084355	-0.959104	-0.868351	0.1252518	0.2160049	0.1706283	up
A_23_P22926	GNB1	4.752101	4.8592305	4.986209	0.1071296	0.234108	0.1706188	up
A_33_P3257943	RPL26	8.401177	8.563358	8.5802	0.1621809	0.1790228	0.1706018	up
A_24_P400355	GET4	4.5223846	4.7323184	4.6536226	0.2099338	0.131238	0.1705859	up

A_23_P169178	TESK1	4.6382484	4.7207866	4.896838	0.0825381	0.2585897	0.1705639	up
A_23_P112634	SMIM14	1.0853744	1.4312172	1.0805335	0.3458428	-0.004841	0.170501	up
A_32_P200238	UCA1	-0.182107	-0.246562	0.2232785	-0.064456	0.4053855	0.170465	up
A_24_P356509	RAD51D	-0.949684	-1.001229	-0.557212	-0.051545	0.3924713	0.1704631	up
A_33_P3398634	TFEB	4.5504923	4.63668	4.8052254	0.0861878	0.2547331	0.1704605	up
A_23_P154256	ELMOD3	1.2974782	1.5051174	1.430737	0.2076392	0.1332588	0.170449	up
A_24_P58529	TUBA1C	6.244134	6.3713746	6.457711	0.1272407	0.2135773	0.170409	up
A_22_P00014361	CWC15	3.5998678	3.666947	3.8734846	0.0670791	0.2736168	0.1703479	up
A_23_P368126	AHCYL1	5.468419	5.6653705	5.612108	0.1969514	0.1436892	0.1703203	up
A_23_P151179	C12orf43	0.6457644	0.7963181	0.8358083	0.1505537	0.1900439	0.1702988	up
A_23_P117380	FAM181A	-1.087433	-0.680231	-1.154057	0.4072022	-0.066624	0.1702893	up
A_21_P0000348	SNORA80A	3.6269417	3.7638469	3.8304844	0.1369052	0.2035427	0.170224	up
A_33_P3409518	TUBBP5	2.497672	2.598431	2.737342	0.100759	0.2396698	0.1702144	up
A_19_P00322966	LINC01278	-2.524889	-2.543653	-2.165725	-0.018764	0.3591635	0.1701995	up
A_23_P34527	FLAD1	2.5679684	2.66962	2.8066683	0.1016517	0.2386999	0.1701758	up
A_24_P85200	ELMSAN1	2.3762455	2.5617328	2.5309916	0.1854873	0.1547461	0.1701167	up
A_21_P0005447	PAXIP1-AS1	-1.94239	-1.789584	-1.754983	0.1528053	0.1874065	0.1701059	up
A_23_P378526	RTEL1	1.107997	1.1194029	1.4365969	0.0114059	0.3285999	0.1700029	up
A_23_P94795	TEAD4	3.018402	3.2848134	3.0918188	0.2664113	0.0734167	0.169914	up
A_23_P33613	DCTPP1	5.714466	5.7572007	6.0114574	0.0427346	0.2969914	0.169863	up
A_23_P101093	COPZ2	-0.4169	-0.322665	-0.171506	0.0942349	0.2453938	0.1698143	up
A_33_P3230709	KIF1C	6.7364664	6.9124007	6.89972	0.1759343	0.1632538	0.169594	up
A_33_P3292126	XLOC_l2_013837	0.5969787	0.7443318	0.7887764	0.1473532	0.1917977	0.1695755	up
A_23_P1014	LINC00467	2.809451	3.0171847	2.9408255	0.2077336	0.1313744	0.169554	up
A_23_P155441	RFT1	1.4558873	1.7546921	1.4961252	0.2988048	0.0402379	0.1695213	up
A_23_P76249	KRT6B	3.4043894	3.5851436	3.5625744	0.1807542	0.158185	0.1694696	up
A_23_P170608	TSPYL2	-0.595824	-0.192823	-0.659969	0.4030008	-0.064145	0.1694279	up
A_23_P257423	ALG14	2.2113304	2.406919	2.3545933	0.1955886	0.1432629	0.1694257	up
A_23_P374149	CMTR1	0.9551606	1.1569748	1.0921459	0.2018142	0.1369853	0.1693997	up
A_22_P00009515	lnc-MAN2C1-4	-2.865606	-2.890815	-2.501691	-0.025209	0.3639157	0.1693535	up
A_23_P379630	SLC38A10	1.3366623	1.660778	1.3511791	0.3241158	0.0145168	0.1693163	up
A_33_P3295640	APBA3	-1.655823	-1.510911	-1.462112	0.1449122	0.1937118	0.169312	up
A_23_P380724	FASTKD5	4.6046495	4.77704	4.770832	0.1723905	0.1661825	0.1692865	up
A_21_P0010220	LOC101930100	-2.241965	-1.95195	-2.193504	0.2900157	0.048461	0.1692383	up
A_33_P3286387	EHMT1	-0.946694	-0.707842	-0.847141	0.238852	0.0995526	0.1692023	up
A_23_P374844	GAL	6.6424074	6.680886	6.9423323	0.0384784	0.2999249	0.1692016	up
A_23_P29594	RPL39L	1.3894181	1.5897679	1.5272055	0.2003498	0.1377873	0.1690686	up
A_24_P114183	FDPS	6.8081675	6.6939015	7.260565	-0.114266	0.4523974	0.1690657	up
A_23_P379026	GTPBP2	3.0554037	3.3561387	3.0927382	0.300735	0.0373344	0.1690347	up
A_33_P3263379	SNORD17	1.71171	1.9476724	1.8135915	0.2359624	0.1018815	0.1689219	up
A_33_P3286492	HNF4G	0.0351243	0.048173	0.3598356	0.0130486	0.3247113	0.16888	up
A_33_P3398697	PICK1	5.5062256	5.570029	5.7801065	0.0638032	0.273881	0.1688421	up
A_23_P396062	RAB40C	1.768302	1.9403453	1.9339209	0.1720433	0.1656189	0.1688311	up
A_33_P3403643	LOC101059906	-0.67611	-0.358141	-0.656451	0.3179693	0.0196595	0.1688144	up
A_21_P0013558	XLOC_l2_014720	5.9930477	6.120069	6.2035666	0.1270213	0.2105188	0.1687701	up
A_33_P3333603	SMOC1	-2.25252	-2.361978	-1.805615	-0.109458	0.4469042	0.1687229	up
A_23_P42265	APOM	0.5468998	0.968667	0.4625483	0.4217672	-0.084352	0.1687078	up
A_21_P0000492	SNAR-F	9.897935	10.117731	10.01553	0.2197962	0.1175947	0.1686955	up
A_21_P0000101	MTRNR2L2	9.926948	10.194126	9.997107	0.2671785	0.070159	0.1686688	up
A_23_P159227	ADAM15	2.7100382	2.7307649	3.0264692	0.0207267	0.3164311	0.1685789	up
A_19_P00802464	lnc-WDR27-1	-2.381635	-2.688997	-1.737137	-0.307362	0.6444979	0.1685682	up
A_23_P144054	PRKCD	1.7961807	2.011063	1.9183083	0.2148824	0.1221275	0.168505	up
A_23_P5211	MUC16	-2.96654	-2.930548	-2.665543	0.0359914	0.3009973	0.1684943	up
A_23_P365086	ANKEF1	-0.648937	-0.250657	-0.710258	0.3982801	-0.061321	0.1684797	up
A_23_P314086	RNF126	4.707427	4.7565475	4.9952297	0.0491204	0.2878027	0.1684616	up
A_23_P162322	WNT10B	0.1989565	0.2068043	0.5279059	0.0078478	0.3289495	0.1683986	up

A_32_P206949	TMEM17	0.0745192	0.7813478	-0.295663	0.7068286	-0.370183	0.1683231	up
A_22_P00015507	lnc-STAT6-2	-1.462189	-1.184461	-1.403286	0.2777281	0.0589032	0.1683156	up
A_33_P3265016	PEX6	-0.572969	-0.408374	-0.401159	0.1645951	0.1718106	0.1682029	up
A_23_P17706	IL17RA	2.0429058	2.0713162	2.3508196	0.0284104	0.3079138	0.1681621	up
A_33_P3270203	DIS3L2	-1.344001	-1.160286	-1.191468	0.1837153	0.1525326	0.168124	up
A_24_P91165	CACNB1	0.0800276	0.370626	0.1256475	0.2905984	0.04562	0.1681092	up
A_23_P109768	RNF7	5.784333	5.901174	6.0036373	0.1168408	0.2193041	0.1680725	up
A_32_P40377	LOC389906	3.4319067	3.547936	3.652011	0.1160293	0.2201042	0.1680667	up
A_24_P124973	NDNL2	-0.351997	-0.137448	-0.230497	0.2145486	0.1215	0.1680243	up
A_24_P703830	NANOS3	-0.467703	-0.095047	-0.504506	0.3726559	-0.036803	0.1679263	up
A_33_P3217103	RBFOX2	0.6869178	0.8514977	0.8581247	0.1645799	0.171207	0.1678934	up
A_33_P3220939	PCID2	2.4982004	2.7212481	2.6109009	0.2230477	0.1127005	0.1678741	up
A_24_P276791	LRRC42	0.4572892	0.7831249	0.4671259	0.3258357	0.0098367	0.1678362	up
A_33_P3323939	LIPN	-1.746943	-1.520825	-1.63744	0.2261176	0.1095033	0.1678104	up
A_23_P56288	LENG8	0.0431199	0.1311545	0.2906861	0.0880346	0.2475662	0.1678004	up
A_24_P13032	UBE2D1	2.105585	2.3077397	2.2389393	0.2021546	0.1333542	0.1677544	up
A_24_P188071	TUBA1C	9.069126	9.1814	9.292194	0.1122742	0.2230682	0.1676712	up
A_24_P145629	SERINC2	0.8188191	1.0162382	0.9567123	0.1974192	0.1378932	0.1676562	up
A_24_P102981	DNAJB2	5.564646	5.842707	5.621852	0.2780614	0.0572062	0.1676338	up
A_21_P0012048	LOC102723927	-1.267647	-0.970624	-1.229452	0.2970238	0.0381956	0.1676097	up
A_23_P41246	MFSD10	1.6627636	1.979321	1.681385	0.3165574	0.0186214	0.1675894	up
A_23_P375281	TRPV1	0.7662921	1.6032362	0.2644215	0.8369441	-0.501871	0.1675367	up
A_22_P00008239	lnc-IRF2BP2-3	1.329124	1.5358953	1.4573503	0.2067714	0.1282263	0.1674988	up
A_33_P3341154	INTS1	-2.149223	-1.940361	-2.023095	0.2088623	0.1261282	0.1674953	up
A_24_P184555	PXN	6.0918894	6.2447295	6.2739487	0.1528401	0.1820593	0.1674497	up
A_21_P0004241	lnc-AC091435.2-1	-0.787913	-0.571847	-0.669293	0.2160664	0.1186199	0.1673431	up
A_33_P3329153	CDK13	0.2437882	0.3847809	0.4373632	0.1409926	0.1935749	0.1672838	up
A_21_P0014236	C9orf3	1.3845463	1.3682251	1.7352142	-0.016321	0.350668	0.1671734	up
A_22_P00016257	lnc-TMED5-1	1.4621205	1.5662446	1.6922793	0.1041241	0.2301588	0.1671414	up
A_23_P21673	FOCAD	3.9853754	4.2605624	4.044338	0.275187	0.0589628	0.1670749	up
A_33_P3332145	RPN2	5.7086506	5.8791137	5.8722525	0.1704631	0.1636019	0.1670325	up
A_24_P159036	RPL36AL	8.82391	9.010845	8.970817	0.1869354	0.1469069	0.1669211	up
A_23_P348636	FOXJ1	1.9787235	1.8403478	2.4508944	-0.138376	0.4721708	0.1668975	up
A_21_P0011489	XLOC_l2_005553	-2.051065	-2.097547	-1.670846	-0.046482	0.3802195	0.1668688	up
A_33_P3323822	GATAD2B	0.9472404	1.1166592	1.1115284	0.1694188	0.164288	0.1668534	up
A_33_P3398401	CRBN	2.3344917	2.4722733	2.5301647	0.1377816	0.195673	0.1667273	up
A_23_P217968	SUV420H1	-0.09731	0.0860734	0.0524273	0.1833835	0.1497374	0.1665604	up
A_23_P71830	ZBTB26	-2.860119	-1.926505	-3.460806	0.9336135	-0.600687	0.1664633	up
A_23_P117727	MTFMT	1.3061604	1.4667697	1.4784675	0.1606093	0.172307	0.1664581	up
A_23_P213093	OCIAD1	3.941908	4.209637	4.0069914	0.2677293	0.0650835	0.1664064	up
A_33_P3262789	REEP6	0.8579044	1.1196575	0.9288468	0.2617531	0.0709424	0.1663477	up
A_22_P00003512	lnc-CCL1-1	-0.7196	-0.349658	-0.756966	0.3699417	-0.037366	0.1662877	up
A_23_P139669	SLC2A3	-1.999284	-1.547444	-2.118579	0.4518395	-0.119295	0.1662722	up
A_21_P0012380	LINC00960	-1.934629	-1.849795	-1.686981	0.0848346	0.2476482	0.1662414	up
A_21_P0007420	lnc-ZBTB44-1	-2.944304	-2.733532	-2.822699	0.2107713	0.1216042	0.1661878	up
A_23_P88134	CINP	1.129663	1.2848864	1.3066816	0.1552234	0.1770186	0.166121	up
A_23_P303317	ZXDC	1.5403962	1.8689127	1.5438876	0.3285165	0.0034914	0.1660039	up
A_23_P359738	EPC2	2.8106766	3.112783	2.84056	0.3021064	0.0298834	0.1659949	up
A_23_P210048	HDAC4	2.1935997	2.3811116	2.3379526	0.1875119	0.1443529	0.1659324	up
A_24_P15621	SLC6A10P	3.1990871	3.4553466	3.2742271	0.2562594	0.07514	0.1656997	up
A_33_P3329949	ANXA7	4.3919964	4.4868197	4.628479	0.0948234	0.2364826	0.165653	up
A_23_P388855	KAT6B	-0.87446	-0.751917	-0.665729	0.1225433	0.2087317	0.1656375	up
A_22_P00006743	FSIP1	-2.898707	-2.661724	-2.804448	0.2369828	0.094259	0.1656209	up
A_22_P00005174	LINC01501	-3.220635	-2.945696	-3.164519	0.2749386	0.0561154	0.165527	up
A_33_P3403615	FKBP1A	3.0669756	3.218307	3.2466612	0.1513314	0.1796856	0.1655085	up
A_23_P72537	AIFM1	5.526335	5.63705	5.7466087	0.1107154	0.220274	0.1654947	up

A_33_P6817354	FGD5-AS1	2.481906	2.5860286	2.7086935	0.1041226	0.2267876	0.1654551	up
A_22_P00010972	lnc-NRIP2-1	-1.014952	-0.783007	-0.916009	0.231945	0.0989423	0.1654437	up
A_23_P4007	FXR2	-0.713884	-0.678622	-0.418452	0.0352621	0.2954321	0.1653471	up
A_23_P159650	COX7B	7.409976	7.4842305	7.6663904	0.0742545	0.2564144	0.1653345	up
A_33_P3384392	ADRBK1	4.4524736	4.420011	4.815504	-0.032463	0.3630304	0.1652839	up
A_23_P17844	PVALB	-2.811905	-2.499379	-2.793937	0.3125253	0.0179677	0.1652465	up
A_19_P00813352	lnc-DYM-1	-0.80062	-0.686747	-0.584184	0.113873	0.2164364	0.1651547	up
A_33_P3305885	WNK2	-1.910317	-2.017578	-1.472887	-0.107261	0.4374304	0.1650848	up
A_21_P0003925	LINC01333	-2.533873	-2.067595	-2.669998	0.4662786	-0.136125	0.1650767	up
A_33_P3260317	NR6A1	-0.906195	-0.27304	-1.209437	0.6331549	-0.303242	0.1649563	up
A_23_P156061	LNPEP	0.5255041	0.7395654	0.6413264	0.2140613	0.1158223	0.1649418	up
A_22_P00008886	LOC101927418	-2.538091	-2.497014	-2.249299	0.0410779	0.2887929	0.1649354	up
A_24_P221883	PARL	3.8342314	4.0070443	3.99125	0.1728129	0.1570187	0.1649158	up
A_24_P521994	KLHL24	0.2303486	0.6327834	0.1575427	0.4024348	-0.072806	0.1648145	up
A_23_P147918	S100A16	7.27017	7.4472003	7.4225054	0.1770301	0.1523352	0.1646826	up
A_23_P410312	C12orf76	1.9358673	2.2658658	1.9349899	0.3299985	-0.000877	0.1645606	up
A_24_P316489	NDUFA10	1.6376629	1.7660623	1.8382969	0.1283994	0.200634	0.1645167	up
A_22_P00015071	lnc-SNTG2-4	-2.87097	-2.484446	-2.928607	0.3865242	-0.057637	0.1644436	up
A_33_P3315268	KRT78	-0.511975	-0.488501	-0.206568	0.0234742	0.3054066	0.1644404	up
A_23_P384517	GYG1	4.33712	4.523976	4.4790373	0.1868558	0.1419172	0.1643865	up
A_24_P307869	LLGL2	2.8164377	3.0082107	2.9533043	0.1917729	0.1368666	0.1643198	up
A_33_P3390643	MMACHC	-0.165633	0.1323834	-0.135097	0.2980166	0.0305362	0.1642764	up
A_22_P00017049	lnc-TUBA1A-1	-1.393107	-1.19038	-1.267501	0.2027273	0.1256056	0.1641665	up
A_33_P3392525	ARL4D	-1.013079	-0.893765	-0.804132	0.1193137	0.2089477	0.1641307	up
A_22_P00001957	SRP54-AS1	-1.239861	-1.040047	-1.111468	0.1998143	0.1283927	0.1641035	up
A_33_P3281905	LINC00930	1.2134557	1.397901	1.3571849	0.1844454	0.1437292	0.1640873	up
A_23_P3042	PPP2R5E	3.5613928	3.8396592	3.6112986	0.2782664	0.0499058	0.1640861	up
A_33_P3237944	AP4M1	-0.977902	-0.735575	-0.89206	0.2423272	0.0858421	0.1640847	up
A_23_P170518	DYM	3.0983467	3.3099256	3.2148447	0.2115789	0.116498	0.1640384	up
A_21_P0009131	lnc-CDH16-1	-2.666515	-2.565545	-2.43953	0.10097	0.2269843	0.1639771	up
A_23_P74269	SRM	3.5724602	3.6049256	3.867938	0.0324655	0.2954779	0.1639717	up
A_23_P212458	SEC61A1	2.9176655	3.1398072	3.023387	0.2221417	0.1057215	0.1639316	up
A_23_P251695	NXT1	5.2151318	5.344192	5.413907	0.1290603	0.1987753	0.1639178	up
A_22_P00008241	LOC102724190	-2.648467	-2.575281	-2.393902	0.0731862	0.2545657	0.1638759	up
A_21_P0008404	LOC102723354	2.7743845	2.8618674	3.0146208	0.0874829	0.2402363	0.1638596	up
A_21_P0014119	NCK1-AS1	-0.27698	-0.354416	0.1281619	-0.077436	0.4051423	0.1638532	up
A_23_P103690	FAM189B	2.2522802	2.1754632	2.6566486	-0.076817	0.4043684	0.1637757	up
A_23_P170290	TMEM57	0.5286622	0.7394738	0.6450372	0.2108116	0.116375	0.1635933	up
A_23_P1206	RPS24	9.356495	9.515131	9.524986	0.1586361	0.1684914	0.1635637	up
A_33_P3318292	SFPQ	5.5048504	5.6883492	5.6484337	0.1834989	0.1435833	0.1635411	up
A_23_P4494	DSC2	1.271769	1.576746	1.2938299	0.3049769	0.0220609	0.1635189	up
A_23_P126908	TNFRSF14	1.8718691	2.2055135	1.8651171	0.3336444	-0.006752	0.1634462	up
A_33_P3239295	PRODH	-1.316521	-0.967187	-1.339022	0.3493338	-0.022501	0.1634164	up
A_33_P3331188	ARHGAP23	-0.587988	-0.471895	-0.37728	0.1160936	0.2107081	0.1634009	up
A_21_P0001911	NIFK-AS1	-1.447299	-1.424399	-1.143441	0.0229001	0.3038583	0.1633792	up
A_33_P3297621	EMC1	0.8477058	1.0169225	1.0051956	0.1692166	0.1574898	0.1633532	up
A_23_P59798	MKRN1	5.5630302	5.8356433	5.6167917	0.2726131	0.0537615	0.1631873	up
A_32_P171061	ASCL2	-2.083302	-1.606338	-2.233974	0.476964	-0.150671	0.1631463	up
A_23_P127663	PRRG4	-0.305451	-0.322908	0.0382133	-0.017457	0.3436642	0.1631033	up
A_24_P365515	FOXA2	0.2888274	-0.05929	0.9630914	-0.348117	0.674264	0.1630733	up
A_23_P121499	WFS1	3.3830442	3.6547961	3.4373894	0.2717519	0.0543451	0.1630485	up
A_33_P3314468	IMMT	1.2015238	1.3860936	1.34303	0.1845698	0.1415062	0.163038	up
A_23_P94660	TBC1D13	4.3572025	4.6135755	4.4269047	0.2563729	0.0697022	0.1630375	up
A_33_P3239455	GTF2IRD2B	-2.321304	-2.348479	-1.968058	-0.027174	0.3532467	0.1630361	up
A_23_P207842	RARA	-0.213998	-0.026002	-0.076361	0.1879959	0.1376376	0.1628168	up
A_33_P3227324	MIER2	3.034707	3.096703	3.2983294	0.061996	0.2636223	0.1628091	up

A_21_P0002920	lnc-CPN2-3	-3.528357	-3.141509	-3.589758	0.3868482	-0.0614	0.1627239	up
A_33_P3334590	MTMR10	-0.514901	-0.546633	-0.157755	-0.031732	0.3571458	0.1627071	up
A_21_P0003243	lnc-EPHA6-1	-0.673216	-0.452841	-0.568411	0.2203746	0.104805	0.1625898	up
A_23_P22994	NFYC	1.8049803	1.9960837	1.9390373	0.1911035	0.134057	0.1625803	up
A_21_P0010295	lnc-CSTB-1	-2.308644	-2.436769	-1.855376	-0.128125	0.4532676	0.1625711	up
A_24_P48069	DOK4	3.5928736	3.688006	3.8226624	0.0951324	0.2297888	0.1624606	up
A_21_P0009794	lnc-VN1R2-1	-1.239187	-0.937533	-1.215946	0.3016543	0.0232415	0.1624479	up
A_23_P142421	KMT2B	2.646492	3.0065732	2.6111279	0.3600812	-0.035364	0.1623585	up
A_24_P324405	ANKRD11	2.9838057	3.3296056	2.962675	0.3457999	-0.021131	0.1623347	up
A_23_P82351	BBS9	0.4834142	0.6635909	0.6277967	0.1801767	0.1443825	0.1622796	up
A_23_P315843	NCOA5	-0.582623	-0.290974	-0.549743	0.2916484	0.0328794	0.1622639	up
A_23_P366366	SCRN1	4.281885	4.6142974	4.273917	0.3324122	-0.007968	0.1622221	up
A_22_P00020312	lnc-FKBP2-1	4.6776752	4.9273515	4.7524014	0.2496762	0.0747261	0.1622012	up
A_23_P141126	GALK1	-0.083292	-0.12533	0.2830281	-0.042038	0.3663206	0.1621413	up
A_33_P3288023	NCKIPSD	-1.359347	-1.095864	-1.298639	0.2634831	0.060708	0.1620955	up
A_21_P0002160	lnc-RPS7-1	-1.445165	-1.288098	-1.278295	0.1570664	0.1668696	0.161968	up
A_23_P404094	THAP7-AS1	-1.455605	-1.35387	-1.233518	0.1017351	0.2220874	0.1619112	up
A_23_P204448	ANAPC5	6.099699	6.2719684	6.2511683	0.1722693	0.1514692	0.1618693	up
A_33_P3249509	NFIC	1.6992798	1.8005595	1.921668	0.1012797	0.2223883	0.161834	up
A_23_P212749	HTT	0.450336	0.5515003	0.6727972	0.1011643	0.2224612	0.1618128	up
A_23_P503233	EDARADD	5.4428654	5.5540786	5.655178	0.1112132	0.2123127	0.161763	up
A_33_P3364348	JPH1	2.9386635	3.040546	3.160305	0.1018825	0.2216415	0.161762	up
A_23_P156531	PSMB1	6.967511	7.2229447	7.035598	0.2554336	0.0680866	0.1617601	up
A_33_P3214339	PLCXD2	-1.903434	-1.606384	-1.87697	0.29705	0.026464	0.161757	up
A_33_P3399291	GUK1	4.6718655	4.7196803	4.9473896	0.0478148	0.2755241	0.1616695	up
A_23_P63178	TAF12	3.8613243	4.1365104	3.9094028	0.2751861	0.0480785	0.1616323	up
A_24_P35169	GATAD1	1.2983713	1.5042882	1.4156885	0.2059169	0.1173172	0.161617	up
A_24_P307827	WHAMM	2.7307959	2.8979297	2.886879	0.1671338	0.1560831	0.1616085	up
A_23_P153945	GTDC1	1.3549867	1.5300446	1.503128	0.1750579	0.1481414	0.1615996	up
A_23_P86504	C10orf76	3.4675922	3.6061664	3.6520796	0.1385741	0.1844873	0.1615307	up
A_19_P00316185	LOC101927686	-0.111719	0.039248	0.0603356	0.1509666	0.1720543	0.1615105	up
A_21_P0000383	SNORD84	-1.733207	-1.360086	-1.783423	0.3731213	-0.050216	0.1614528	up
A_23_P382488	LRRC16B	-2.102912	-2.153302	-1.729641	-0.05039	0.3732715	0.1614409	up
A_32_P62571	RBM8A	2.864255	2.9241595	3.126976	0.0599046	0.2627211	0.1613128	up
A_33_P3335233	HKDC1	0.6550522	0.9252701	0.7073627	0.2702179	0.0523105	0.1612642	up
A_23_P334870	TMEM217	-2.676452	-1.913746	-3.116633	0.762706	-0.440181	0.1612625	up
A_23_P109881	ITIH4	-1.731086	-1.545564	-1.594117	0.1855221	0.1369686	0.1612453	up
A_23_P42353	ETV7	-1.755151	-1.989596	-1.198281	-0.234446	0.55687	0.1612122	up
A_33_P3241108	DNAJC11	-2.118076	-2.028525	-1.885384	0.089551	0.2326927	0.1611218	up
A_33_P3209915	PTAR1	-1.280287	-0.984428	-1.254032	0.2958593	0.0262551	0.1610572	up
A_33_P3236676	C9orf152	3.2658634	3.6263957	3.2273922	0.3605323	-0.038471	0.1610305	up
A_23_P5903	SLCO4A1	4.2518606	4.1336503	4.6920557	-0.11821	0.4401951	0.1609924	up
A_24_P276888	CENPO	1.565073	1.7544851	1.6975989	0.1894121	0.1325259	0.160969	up
A_23_P27795	SPINT2	8.267008	8.391844	8.464076	0.124836	0.1970682	0.1609521	up
A_23_P98183	HRAS	3.0732737	3.2075143	3.260765	0.1342406	0.1874914	0.160866	up
A_23_P136012	FBXO8	0.5931149	0.8466101	0.6613469	0.2534952	0.0682321	0.1608636	up
A_24_P375819	HTR7P1	-2.464367	-2.405889	-2.201155	0.0584781	0.2632129	0.1608455	up
A_33_P3337259	WASH5P	-0.566231	-0.426138	-0.38474	0.1400929	0.1814909	0.1607919	up
A_23_P17471	PCED1A	-1.291845	-0.798533	-1.463712	0.4933114	-0.171867	0.1607223	up
A_33_P3345708	CREB3L4	0.8388453	1.2400203	0.7590156	0.401175	-0.07983	0.1606727	up
A_23_P416036	HAUS7	1.6506824	1.8223896	1.8001261	0.1717072	0.1494436	0.1605754	up
A_21_P0013622	CLCN3	-2.530524	-2.422098	-2.31784	0.1084263	0.2126844	0.1605554	up
A_24_P294931	PPP2R5D	3.7137022	3.9240108	3.82446	0.2103086	0.1107578	0.1605332	up
A_23_P28869	PTPRA	3.3397446	3.3563561	3.644187	0.0166116	0.3044424	0.160527	up
A_23_P206359	CDH1	5.679123	6.079596	5.599635	0.4004731	-0.079488	0.1604927	up
A_23_P29495	CTNNB1	4.9801054	5.0213494	5.2596807	0.041244	0.2795754	0.1604097	up

A_33_P3240200	DPCD	5.662838	5.787994	5.8581085	0.1251559	0.1952705	0.1602132	up
A_23_P97394	BCAR3	2.228795	2.4888873	2.2890024	0.2600923	0.0602074	0.1601498	up
A_22_P00000741	LOC642852	2.2589931	2.5215669	2.3165941	0.2625737	0.057601	0.1600873	up
A_24_P364236	NDUFC2	2.1142483	2.1921487	2.3565044	0.0779004	0.2422562	0.1600783	up
A_33_P3312119	C6orf99	1.066545	1.1609974	1.2922235	0.0944524	0.2256784	0.1600654	up
A_33_P3226177	CYFIP2	-1.063519	-1.292686	-0.51425	-0.229167	0.5492687	0.1600506	up
A_33_P7796746	TONSL	10.032088	10.278374	10.105793	0.2462854	0.0737047	0.1599951	up
A_23_P154315	MRPS9	5.963688	5.9839377	6.2634153	0.0202498	0.2997274	0.1599886	up
A_23_P410224	C6orf52	-2.277795	-2.253272	-1.982367	0.0245235	0.2954288	0.1599761	up
A_22_P00000430	ZNF316	1.1825395	1.2958541	1.3891387	0.1133146	0.2065992	0.1599569	up
A_33_P3396270	SLC9A4	1.2906084	1.4875922	1.4133387	0.1969838	0.1227303	0.159857	up
A_33_P3209229	RAB26	0.8296289	1.051475	0.9272728	0.2218461	0.0976439	0.159745	up
A_21_P0014001	NNT-AS1	-0.731339	-0.533687	-0.609568	0.1976523	0.1217709	0.1597116	up
A_23_P212397	LARS2	1.8954668	2.0324702	2.077817	0.1370034	0.1823502	0.1596768	up
A_23_P203406	GANAB	4.8408127	4.960791	5.040101	0.1199784	0.1992884	0.1596334	up
A_33_P3374623	ABCA7	1.0507464	1.1743975	1.2462854	0.123651	0.195539	0.159595	up
A_23_P20427	RHOBTB2	-0.197864	-0.257947	0.1812477	-0.060082	0.3791118	0.1595147	up
A_33_P3323368	LINC00921	-2.53231	-2.422217	-2.32352	0.1100926	0.2087891	0.1594409	up
A_32_P220472	ZFAND6	4.9128494	5.0334954	5.1110306	0.120646	0.1981812	0.1594136	up
A_24_P82493	DCAKD	-1.077977	-1.186278	-0.651011	-0.108301	0.4269667	0.1593328	up
A_24_P350644	PRRC2B	3.6200829	3.8511014	3.7077227	0.2310185	0.0876398	0.1593292	up
A_23_P83351	DFNB31	-0.988816	-0.791956	-0.867059	0.1968603	0.1217566	0.1593084	up
A_24_P282210	CAPN15	4.947423	5.2318387	4.981574	0.2844157	0.0341511	0.1592834	up
A_23_P217609	RPL36A	9.53646	9.736177	9.655308	0.1997175	0.1188479	0.1592827	up
A_23_P110504	CLPTM1L	4.516453	4.7681565	4.5833044	0.2517037	0.0668516	0.1592777	up
A_21_P0013320	XLOC_l2_013873	0.6158786	0.8310814	0.7192226	0.2152028	0.103344	0.1592734	up
A_22_P00014975	SMARCC2	3.2236223	3.3334975	3.4322548	0.1098752	0.2086325	0.1592538	up
A_23_P16242	ZNF20	-0.454289	-0.379178	-0.210899	0.0751109	0.2433896	0.1592503	up
A_19_P00322357	LOC101928738	0.2170372	0.4981332	0.2544026	0.281096	0.0373654	0.1592307	up
A_23_P200507	CNIH4	6.225148	6.332275	6.436452	0.1071267	0.2113037	0.1592152	up
A_23_P59787	LUC7L2	2.438159	2.612844	2.5818272	0.174685	0.1436682	0.1591766	up
A_22_P00011823	LOC101927934	-0.142918	-0.095767	0.1281519	0.0471511	0.2710695	0.1591103	up
A_22_P00018253	CLUHP3	0.5221515	0.6755376	0.6868038	0.1533861	0.1646524	0.1590192	up
A_23_P114656	HDAC1	5.607445	5.6874976	5.84527	0.0800529	0.2378254	0.1589391	up
A_33_P3490794	LINC00924	-1.941523	-1.651185	-1.914007	0.290338	0.0275164	0.1589272	up
A_33_P3285354	C11orf95	1.1695213	1.3231058	1.3337684	0.1535845	0.164247	0.1589158	up
A_23_P58102	EXOSC7	3.204793	3.3888917	3.3384838	0.1840987	0.1336908	0.1588948	up
A_33_P3240787	LOC100131910	-3.232334	-2.98643	-3.160458	0.2459037	0.0718756	0.1588897	up
A_33_P3460043	BAALC-AS2	0.1572781	0.2989106	0.3332257	0.1416326	0.1759477	0.1587901	up
A_23_P15829	TOP3A	0.7010055	0.8321085	0.8873544	0.131103	0.1863489	0.158726	up
A_22_P00025328	lnc-C11orf41-1	-2.008668	-1.786038	-1.913883	0.2226291	0.0947847	0.1587069	up
A_24_P89843	CYHR1	1.7647581	2.1227355	1.7241077	0.3579774	-0.04065	0.1586635	up
A_23_P432591	CCDC125	2.1322803	2.1609912	2.420886	0.0287108	0.2886057	0.1586583	up
A_23_P11644	SPRR2D	0.0104537	0.7388191	-0.400643	0.7283654	-0.411097	0.1586342	up
A_23_P145024	ADRB2	-3.233863	-2.83098	-3.319485	0.4028833	-0.085621	0.158631	up
A_21_P0014170	LOC100506885	-3.285306	-3.046774	-3.206603	0.2385316	0.0787032	0.1586174	up
A_33_P3315239	ZNF7	-2.401475	-2.39205	-2.093731	0.0094249	0.3077431	0.158584	up
A_23_P67992	C1D	2.88978	3.0655723	3.0311117	0.1757922	0.1413317	0.1585619	up
A_33_P3411315	KRTAP3-3	4.973007	5.263022	5.0001106	0.2900147	0.0271034	0.1585591	up
A_22_P00020287	lnc-CPVL-4	-2.185809	-1.968234	-2.086402	0.2175746	0.0994062	0.1584904	up
A_21_P0012477	XLOC_l2_010493	0.1782022	0.3634343	0.3099151	0.1852322	0.1317129	0.1584725	up
A_33_P3225685	B3GAT3	2.4776402	2.7945962	2.4775887	0.316956	-5.15E-05	0.1584523	up
A_21_P0014172	HIST1H4H	0.682756	1.3409672	0.3413911	0.6582112	-0.341365	0.1584232	up
A_21_P0000598	FBXW4	2.0085335	2.2118754	2.122033	0.203342	0.1134996	0.1584208	up
A_22_P00017432	lnc-VN1R2-1	-0.979852	-0.773914	-0.869086	0.2059379	0.1107659	0.1583519	up
A_22_P00022855	LOC100996437	-2.876135	-2.564149	-2.87146	0.3119867	0.0046754	0.158331	up

A_23_P435501	SERINC3	1.608655	1.6646895	1.8692436	0.0560346	0.2605887	0.1583116	up
A_33_P3288805	KLC2	-2.301174	-2.202929	-2.082942	0.0982451	0.2182317	0.1582384	up
A_24_P242036	RRP7B	1.7572966	2.0025601	1.8282819	0.2452636	0.0709853	0.1581245	up
A_24_P189997	PCSK6	-2.020306	-1.756788	-1.967618	0.2635179	0.0526876	0.1581028	up
A_33_P3343812	OR1L6	-1.179986	-1.152157	-0.891727	0.0278287	0.288259	0.1580439	up
A_33_P3243657	TIMM10B	2.7644472	2.7497926	3.0950975	-0.014655	0.3306503	0.1579978	up
A_33_P3347108	TTC5	3.3771734	3.4244647	3.6458607	0.0472913	0.2686873	0.1579893	up
A_33_P3224867	GSG1	-2.972674	-2.855308	-2.774285	0.1173668	0.1983895	0.1578782	up
A_23_P432512	PPIL2	0.8059397	0.8957396	1.031888	0.0897999	0.2259483	0.1578741	up
A_23_P355364	LRCH3	-1.944459	-1.673642	-1.899544	0.2708168	0.0449147	0.1578658	up
A_23_P134827	ASH2L	1.822999	2.0602117	1.9014635	0.2372127	0.0784645	0.1578386	up
A_23_P340333	ITPRIP	3.3231287	3.477054	3.48487	0.1539254	0.1617413	0.1578333	up
A_33_P3421759	SON	1.0193701	1.1169229	1.2373967	0.0975528	0.2180266	0.1577897	up
A_33_P3354990	SYNJ2	-1.414432	-1.163575	-1.349725	0.2508569	0.0647073	0.1577821	up
A_21_P0004128	lnc-EMB-3	-1.845937	-1.681417	-1.694978	0.1645198	0.150959	0.1577394	up
A_23_P76034	PVRL1	0.1647048	0.1927967	0.4520369	0.0280919	0.2873321	0.157712	up
A_21_P0008531	lnc-MBIP-2	1.2289982	1.585125	1.1882801	0.3561268	-0.040718	0.1577044	up
A_32_P92783	STIP1	2.8757992	2.9143605	3.1526299	0.0385613	0.2768307	0.157696	up
A_23_P250035	SDHA	2.7984915	3.0189748	2.893239	0.2204833	0.0947475	0.1576154	up
A_33_P3354151	BOLA1	1.7357874	1.813498	1.9730682	0.0777106	0.2372809	0.1574957	up
A_33_P3380693	ADAM15	7.7863684	7.798001	8.089574	0.0116324	0.3032055	0.157419	up
A_23_P126757	INTS3	1.1003284	1.3518076	1.1636128	0.2514792	0.0632844	0.1573818	up
A_33_P6607913	CWC15	3.876298	4.0148015	4.052536	0.1385036	0.1762381	0.1573708	up
A_23_P10374	R3HDM2	2.8938122	3.1201453	2.9821978	0.2263331	0.0883856	0.1573594	up
A_23_P259580	TAPBP	-2.245342	-2.350647	-1.825412	-0.105305	0.41993	0.1573126	up
A_24_P147765	FOXRED2	2.4764795	2.8799853	2.387519	0.4035058	-0.088961	0.1572726	up
A_23_P111804	PARP12	2.6869984	2.4742637	3.2141953	-0.212735	0.5271969	0.1572311	up
A_23_P154688	SLC4A11	3.1812239	3.2775016	3.399356	0.0962777	0.218132	0.1572049	up
A_23_P394567	KIAA1467	-0.956554	-1.002064	-0.596777	-0.04551	0.3597779	0.1571341	up
A_19_P00320434	LOC100996579	0.7442431	0.8272381	0.975452	0.0829949	0.2312088	0.1571019	up
A_23_P154522	MTA3	0.8935213	1.0849795	1.0162373	0.1914582	0.122716	0.1570871	up
A_23_P20316	CA3	-2.92628	-2.381203	-3.157191	0.5450766	-0.230912	0.1570826	up
A_32_P109572	HNRNPL	4.1373034	4.238069	4.350518	0.1007657	0.2132149	0.1569903	up
A_23_P129433	SLC9A5	-0.651471	-0.486189	-0.502866	0.1652823	0.1486049	0.1569436	up
A_21_P0007796	lnc-SLC15A4-5	-2.501865	-2.315181	-2.374667	0.1866849	0.1271987	0.1569418	up
A_33_P3217465	MID1IP1	5.604617	5.7693925	5.753627	0.1647754	0.1490097	0.1568925	up
A_23_P48747	DHRS1	1.2784777	1.4806013	1.3899975	0.2021236	0.1115198	0.1568217	up
A_23_P3221	SQRDL	6.205763	6.2762856	6.448784	0.0705228	0.243021	0.1567719	up
A_23_P14636	MFAP1	3.7875996	3.8877006	4.0010386	0.100101	0.213439	0.15677	up
A_33_P3390521	UBE2Z	0.4337697	0.6680565	0.5129738	0.2342868	0.0792041	0.1567454	up
A_33_P3285824	C4orf3	5.748418	5.7226872	6.0876093	-0.025731	0.3391914	0.1567304	up
A_23_P252653	STK25	3.7241936	3.898479	3.8633108	0.1742854	0.1391172	0.1567013	up
A_24_P410017	POTEI	5.87689	5.9515576	6.1155224	0.0746675	0.2386322	0.1566498	up
A_23_P428827	AKIRIN2	1.1167917	1.3139787	1.232893	0.197187	0.1161013	0.1566441	up
A_32_P88231	ERICH2	-2.363223	-2.473464	-1.939735	-0.110241	0.4234886	0.1566238	up
A_21_P0000497	SNORD1B	-1.47456	-1.130764	-1.505135	0.3437963	-0.030575	0.1566107	up
A_33_P3245006	DAK	-0.314078	-0.131999	-0.182992	0.1820798	0.1310868	0.1565833	up
A_33_P3296862	C16orf89	-2.142568	-2.064749	-1.90727	0.0778189	0.2352982	0.1565585	up
A_23_P165247	DAZAP1	3.919588	4.192698	3.9595318	0.2731099	0.0399437	0.1565268	up
A_21_P0010451	lnc-C22orf26-4	-1.814609	-1.846616	-1.46955	-0.032007	0.3450589	0.1565259	up
A_24_P41021	FBXO33	0.5991488	0.7668667	0.7444196	0.1677179	0.1452708	0.1564944	up
A_32_P93149	DDX10	-2.112658	-2.081188	-1.831223	0.0314703	0.281435	0.1564527	up
A_33_P3346222	MSH5	-2.295283	-2.063174	-2.214658	0.2321091	0.0806251	0.1563671	up
A_23_P69521	CCNI	6.1106367	6.355696	6.1781883	0.2450595	0.0675516	0.1563056	up
A_23_P6303	U2AF1	7.004589	7.138223	7.1835527	0.1336341	0.1789637	0.1562989	up
A_33_P3271273	HOXB2	-0.150766	-0.109005	0.1198473	0.0417609	0.2706137	0.1561873	up

A_22_P00021754	lnc-DDX19A-1	-1.989352	-1.58502	-2.081336	0.4043322	-0.091984	0.1561742	up
A_33_P3246007	APOA1BP	5.3724775	5.605001	5.452203	0.2325234	0.0797253	0.1561244	up
A_23_P356109	ATXN7L1	-2.428546	-2.063752	-2.481162	0.3647938	-0.052616	0.1560891	up
A_22_P00012112	LOC100287896	-1.440076	-1.155342	-1.412728	0.2847347	0.0273485	0.1560416	up
A_33_P3273233	LOC100129215	-2.031895	-1.901	-1.85071	0.1308947	0.1811843	0.1560395	up
A_23_P20075	NPC1L1	-1.612169	-1.242064	-1.670207	0.3701048	-0.058038	0.1560335	up
A_33_P3281196	MRPS36	3.0907822	3.1919436	3.3016672	0.1011615	0.2108851	0.1560233	up
A_24_P67988	FRMD8	4.9038515	5.0477147	5.0719395	0.1438632	0.168088	0.1559756	up
A_33_P3322654	RNPEPL1	6.068349	6.140574	6.30807	0.0722251	0.2397213	0.1559732	up
A_22_P00007240	lnc-GPR12-1	1.2843189	1.4041228	1.4764013	0.1198039	0.1920824	0.1559432	up
A_21_P0004591	MLLT4-AS1	-2.660398	-2.352005	-2.656966	0.3083928	0.0034323	0.1559125	up
A_23_P424597	C19orf25	2.1134024	2.1289067	2.4096317	0.0155044	0.2962294	0.1558669	up
A_23_P126197	SRSF4	4.5783396	4.5306764	4.9376993	-0.047663	0.3593597	0.1558483	up
A_23_P106720	TBL3	1.7496786	1.887692	1.9231253	0.1380134	0.1734467	0.15573	up
A_21_P0013608	LINC01573	-2.060685	-1.722874	-2.087073	0.3378105	-0.026388	0.1557112	up
A_24_P336759	MCL1	2.7168636	2.7049108	3.0399618	-0.011953	0.3230982	0.1555727	up
A_22_P00005439	lnc-DUOXA1-2	-2.530132	-2.686316	-2.062835	-0.156184	0.4672968	0.1555564	up
A_23_P70060	PPAP2A	2.8458824	2.8983216	3.1044903	0.0524392	0.2586079	0.1555235	up
A_23_P142424	IGFLR1	2.6843138	2.9156623	2.763935	0.2313485	0.0796213	0.1554849	up
A_21_P0007329	lnc-CCDC34-1	0.8661385	1.1464672	0.8967619	0.2803288	0.0306234	0.1554761	up
A_24_P67395	KRT8	3.8863087	4.112375	3.9708471	0.2260661	0.0845385	0.1553023	up
A_21_P0011671	XLOC_l2_006578	-2.435047	-2.315208	-2.244543	0.1198387	0.1905034	0.155171	up
A_33_P3405349	C7orf43	-1.19811	-1.019268	-1.066614	0.1788416	0.131496	0.1551688	up
A_23_P28090	STX10	1.1749868	1.4575047	1.2026982	0.2825179	0.0277114	0.1551146	up
A_21_P0002469	lnc-SFTPB-1	-1.102012	-0.902267	-0.991601	0.1997457	0.1104112	0.1550784	up
A_33_P3346866	KANSL2	5.4741783	5.614261	5.644207	0.1400828	0.1700287	0.1550558	up
A_24_P258235	OR5L2	-0.035621	0.26123	-0.02243	0.2968507	0.0131907	0.1550207	up
A_23_P388681	ELAVL1	1.6349106	1.6802721	1.8995552	0.0453615	0.2646446	0.1550031	up
A_33_P3259017	CSNK1G2	4.0623446	4.096046	4.3385773	0.0337014	0.2762327	0.1549671	up
A_33_P3350634	KIF22	3.4996033	3.6576953	3.6512575	0.158092	0.1516542	0.1548731	up
A_23_P865	FRRS1	1.0918951	1.3346767	1.1586976	0.2427816	0.0668025	0.1547921	up
A_23_P368101	SAP30L	3.0683012	3.3974118	3.048667	0.3291106	-0.019634	0.1547382	up
A_32_P141923	NACA2	7.4707117	7.693193	7.557704	0.2224813	0.0869923	0.1547368	up
A_23_P106174	PSEN1	-0.597542	-0.394488	-0.491155	0.2030544	0.1063876	0.154721	up
A_23_P66891	CDC42EP4	2.9501352	3.2028604	3.0068464	0.2527251	0.0567112	0.1547182	up
A_33_P3421733	EIF3C	3.3002272	3.5060287	3.403778	0.2058015	0.1035509	0.1546762	up
A_23_P423457	SERINC5	1.5409493	1.745501	1.6457181	0.2045517	0.1047688	0.1546602	up
A_23_P96688	SUV420H1	-2.125356	-1.72837	-2.213206	0.396986	-0.08785	0.1545681	up
A_33_P3281795	MGLL	4.0145416	4.3628163	3.9754028	0.3482747	-0.039139	0.154568	up
A_22_P00001894	lnc-B4GALNT3-1	-2.594608	-2.660949	-2.219301	-0.066341	0.3753068	0.1544827	up
A_32_P100641	LINC01233	-1.81124	-1.619426	-1.6941	0.191814	0.1171398	0.1544769	up
A_33_P3421867	MDGA1	-1.063044	-0.853543	-0.963746	0.2095008	0.099298	0.1543994	up
A_24_P336584	RASSF5	-1.326135	-1.221418	-1.122137	0.1047173	0.2039986	0.1543579	up
A_24_P879563	WAC-AS1	2.2628708	2.2820163	2.5522394	0.0191455	0.2893686	0.1542571	up
A_22_P00021268	lnc-UBE2Z-1	-1.46724	-1.376688	-1.249374	0.0905528	0.2178664	0.1542096	up
A_24_P216087	PAF1	4.04432	4.2453866	4.151578	0.2010665	0.1072578	0.1541622	up
A_23_P101699	HDGFRP2	-0.589131	-0.410571	-0.459398	0.1785603	0.1297331	0.1541467	up
A_32_P228501	CDC26	4.2841644	4.2912936	4.5852385	0.0071292	0.301074	0.1541016	up
A_23_P149042	PYCR2	1.474072	1.6509318	1.6053944	0.1768599	0.1313224	0.1540911	up
A_22_P00018910	lnc-LAMA1-5	-3.553718	-3.191577	-3.607679	0.3621411	-0.053961	0.15409	up
A_21_P0014397	RALY-AS1	2.079811	2.2146163	2.2531443	0.1348052	0.1733332	0.1540692	up
A_33_P3405434	VWA5B1	-1.556761	-1.264024	-1.541501	0.292737	0.0152597	0.1539984	up
A_23_P131771	TPD52L2	1.9886866	2.2488914	2.036357	0.2602048	0.0476704	0.1539376	up
A_24_P315500	NFYC-AS1	1.3580108	1.676661	1.3471761	0.3186503	-0.010835	0.1539078	up
A_32_P177955	STX17-AS1	1.7369528	1.9061422	1.8755703	0.1691895	0.1386175	0.1539035	up
A_23_P60283	XPA	2.0398626	2.2838874	2.1036444	0.2440248	0.0637817	0.1539032	up

A_23_P360079	NCKAP5	-0.835068	-0.47987	-0.882474	0.3551979	-0.047406	0.1538959	up
A_23_P106887	FUS	4.368616	4.628817	4.4161806	0.260201	0.0475645	0.1538827	up
A_23_P140527	FOXB1	0.3978295	0.7316961	0.3716631	0.3338666	-0.026166	0.1538501	up
A_24_P132813	OPALIN	-3.009324	-2.922062	-2.78891	0.0872624	0.2204139	0.1538382	up
A_24_P417526	FRG1B	2.132146	2.2023797	2.369525	0.0702338	0.2373791	0.1538064	up
A_23_P93623	ATP6V1F	5.7113953	5.6322675	6.0981016	-0.079128	0.3867064	0.1537893	up
A_33_P3570223	ACTG1P20	8.696305	8.88628	8.813764	0.1899748	0.1174583	0.1537166	up
A_23_P70355	SERPINB6	2.1792974	2.460339	2.2055902	0.2810416	0.0262928	0.1536672	up
A_33_P3593774	PIK3R3	-0.307533	-0.035323	-0.272566	0.2722096	0.0349665	0.1535881	up
A_21_P0007400	lnc-IL18-1	-1.801865	-1.702765	-1.593796	0.0991001	0.2080684	0.1535842	up
A_21_P0013440	LOC102725415	-2.395546	-2.280918	-2.203022	0.1146281	0.1925242	0.1535761	up
A_24_P341222	SMIM20	2.4957857	2.6203322	2.6783876	0.1245465	0.1826019	0.1535742	up
A_24_P225518	CPTP	-0.962676	-0.888293	-0.730076	0.0743823	0.2325993	0.1534908	up
A_23_P217319	FGF13	-2.672114	-2.340132	-2.69712	0.3319824	-0.025006	0.153488	up
A_24_P29975	ARPC5L	4.7005844	4.774999	4.9330482	0.0744147	0.2324638	0.1534393	up
A_23_P21316	PRUNE	-2.458081	-2.233709	-2.375593	0.2243722	0.0824883	0.1534302	up
A_23_P154058	EIF2B4	2.7013235	2.865581	2.8438644	0.1642575	0.1425409	0.1533992	up
A_23_P313828	CENPV	-2.804164	-3.27564	-2.025915	-0.471476	0.7782495	0.1533868	up
A_23_P92520	ANP32C	0.3662281	0.4546471	0.5845666	0.088419	0.2183385	0.1533787	up
A_23_P201079	PRDM2	-1.914833	-1.95626	-1.566662	-0.041427	0.3481703	0.1533716	up
A_23_P13033	RBM4	2.525382	2.6295953	2.7278814	0.1042132	0.2024994	0.1533563	up
A_32_P205553	RPL26L1	5.82251	5.8886285	6.0630426	0.0661187	0.2405329	0.1533258	up
A_19_P00808453	COX5A	7.7744055	7.887524	7.9679184	0.1131187	0.1935129	0.1533158	up
A_23_P168868	PTDSS1	7.2167606	7.3589473	7.381151	0.1421866	0.1643906	0.1532886	up
A_23_P151506	PLEK2	4.605056	4.668843	4.847843	0.063787	0.2427874	0.1532872	up
A_24_P173234	ZNF613	1.2476797	1.3394356	1.4624524	0.0917559	0.2147727	0.1532643	up
A_21_P0010303	lnc-C21orf67-5	-0.541671	-0.335893	-0.441148	0.2057781	0.1005235	0.1531508	up
A_33_P3376636	OPA3	0.6107798	0.8140898	0.7137113	0.20331	0.1029315	0.1531208	up
A_24_P135748	GRTP1	1.6727343	1.8843794	1.7673297	0.2116451	0.0945954	0.1531203	up
A_33_P3239084	EEF2KMT	1.0661068	1.2440634	1.1942868	0.1779566	0.12818	0.1530683	up
A_33_P3235568	CAPZB	3.0497313	3.1460233	3.2595453	0.096292	0.2098141	0.153053	up
A_23_P160177	ATP1A4	2.307598	2.507913	2.4133196	0.200315	0.1057215	0.1530182	up
A_24_P108738	SCARF2	2.1966267	2.5261378	2.17307	0.3295112	-0.023557	0.1529772	up
A_21_P0001092	lnc-TTC34-3	-2.158451	-1.990276	-2.020877	0.1681752	0.1375737	0.1528745	up
A_23_P36484	RDH16	-2.957613	-2.868653	-2.74089	0.0889602	0.2167232	0.1528417	up
A_24_P383478	ESR1	-2.471668	-2.689667	-1.948062	-0.217999	0.5236063	0.1528038	up
A_23_P156788	STX11	-2.828705	-2.381285	-2.970604	0.4474194	-0.1419	0.1527598	up
A_23_P416965	FAM149A	-0.170345	0.1408558	-0.176107	0.3112006	-0.005763	0.152719	up
A_22_P00011158	ABO	1.3539705	1.5171404	1.4960499	0.1631699	0.1420794	0.1526246	up
A_23_P20970	ATG12	3.477582	3.6120286	3.6481056	0.1344466	0.1705236	0.1524851	up
A_23_P256413	CMTM7	4.98209	4.9758143	5.293332	-0.006276	0.3112421	0.1524832	up
A_33_P3356320	C17orf97	-1.99175	-1.99939	-1.679246	-0.00764	0.3125033	0.1524317	up
A_33_P3235990	PTPN21	-0.376577	-0.176273	-0.272139	0.2003036	0.1044378	0.1523707	up
A_22_P00006314	lnc-FAM91A1-1	-3.351224	-2.863469	-3.534243	0.4877553	-0.183018	0.1523685	up
A_23_P154986	GGT1	-1.879686	-1.70575	-1.748911	0.1739368	0.1307755	0.1523561	up
A_24_P126557	RAVER1	-0.731611	-0.632006	-0.526624	0.0996056	0.2049875	0.1522965	up
A_21_P0008938	lnc-RP11-327F22.5.1-6	-2.052406	-2.063542	-1.736748	-0.011136	0.3156581	0.152261	up
A_23_P369733	RANBP10	3.0636272	3.2752738	3.1564264	0.2116466	0.0927992	0.1522229	up
A_24_P391368	ATXN10	4.0312138	4.1172843	4.2493887	0.0860705	0.2181749	0.1521227	up
A_24_P302685	ARHGEF4	-2.924667	-2.594341	-2.950934	0.3303261	-0.026267	0.1520296	up
A_23_P252155	STRBP	3.1681938	3.3771615	3.263134	0.2089677	0.0949402	0.1519539	up
A_33_P3301620	RNF32	2.9774618	3.2160687	3.0427542	0.2386069	0.0652924	0.1519496	up
A_33_P3406686	DCTN1	-2.110633	-1.844331	-2.073171	0.2663021	0.0374618	0.1518819	up
A_33_P3305915	GGA1	0.941618	1.0805044	1.1064429	0.1388865	0.164825	0.1518557	up
A_21_P0000212	SNORA68	0.9181547	1.1604509	0.9795399	0.2422962	0.0613852	0.1518407	up

A_21_P0000740	HIVEP3	-0.769814	-0.745992	-0.490035	0.0238218	0.279779	0.1518004	up
A_22_P00014959	LOC100288181	-2.166359	-1.627224	-2.401897	0.5391345	-0.235538	0.1517983	up
A_21_P0003830	lnc-TBC1D1-1	-2.620609	-2.730094	-2.207538	-0.109485	0.4130704	0.1517929	up
A_33_P3715177	MEGF8	3.381549	3.6343713	3.4322262	0.2528224	0.0506773	0.1517499	up
A_24_P244470	RNF41	-0.145212	0.0393038	-0.026234	0.184516	0.118978	0.151747	up
A_24_P309317	PSAP	3.3273163	3.79571	3.1623516	0.4683938	-0.164965	0.1517146	up
A_23_P52127	ACBD6	5.2335377	5.2972198	5.473283	0.0636821	0.2397451	0.1517136	up
A_22_P00007455	lnc-GTDC1-4	-2.025844	-1.996688	-1.751658	0.0291557	0.2741866	0.1516712	up
A_24_P404245	PCYT2	2.1315327	2.3290658	2.2371674	0.1975331	0.1056347	0.1515839	up
A_23_P99917	WDR73	3.738452	3.8198285	3.9601316	0.0813766	0.2216797	0.1515281	up
A_22_P00009583	FAM83H-AS1	2.8628483	3.0141234	3.0146055	0.1512752	0.1517572	0.1515162	up
A_33_P3305775	lnc-HDDC3-4	-1.995032	-1.777425	-1.909629	0.217607	0.0854034	0.1515052	up
A_33_P3372886	DDX51	3.6537209	3.697187	3.9132462	0.0434661	0.2595253	0.1514957	up
A_24_P14367	PTBP1	1.3058367	1.380919	1.5337319	0.0750823	0.2278953	0.1514888	up
A_23_P140423	NDUFB1	6.4440165	6.623215	6.567775	0.1791987	0.1237583	0.1514785	up
A_21_P0009852	LINC01273	-1.940773	-1.935381	-1.643259	0.0053921	0.2975135	0.1514528	up
A_33_P3227880	BTN3A2	-1.094477	-0.960197	-0.925931	0.1342807	0.1685462	0.1514134	up
A_22_P00003499	LOC100506282	-2.377981	-2.481953	-1.971223	-0.103972	0.4067578	0.1513928	up
A_32_P160896	FTMT	-2.608819	-2.491303	-2.423707	0.117516	0.1851122	0.1513141	up
A_23_P150343	SLN	-2.079659	-2.05181	-1.804882	0.0278487	0.2747769	0.1513128	up
A_23_P69437	YEATS2	4.4319963	4.518724	4.6478596	0.0867276	0.2158632	0.1512954	up
A_23_P42335	FANCE	3.310546	3.4020143	3.5215569	0.0914683	0.2110109	0.1512396	up
A_24_P290013	FAM86B3P	0.8287425	1.1665692	0.7933736	0.3378267	-0.035369	0.1512289	up
A_21_P0000257	SNORD27	1.0110245	1.1794543	1.1447468	0.1684299	0.1337223	0.1510761	up
A_23_P66306	ALG1	3.7245016	3.7019506	4.04914	-0.022551	0.3246384	0.1510437	up
A_33_P3215999	AFMID	0.7063127	0.8664217	0.848032	0.160109	0.1417193	0.1509142	up
A_23_P27066	UBE2G1	1.8963289	2.0132565	2.0811806	0.1169276	0.1848517	0.1508896	up
A_32_P98732	GCM1	-1.848652	-1.736805	-1.658793	0.1118465	0.1898594	0.1508529	up
A_23_P20722	SNAPC4	4.921116	5.0977664	5.0459194	0.1766505	0.1248035	0.150727	up
A_22_P00023569	lnc-FAM168A-1	0.0313745	0.0824623	0.2817206	0.0510879	0.2503462	0.150717	up
A_21_P0010525	XLOC_l2_000123	7.9045534	7.907642	8.202874	0.0030885	0.2983208	0.1507046	up
A_24_P162373	ZNRF3	6.024415	6.0469794	6.303215	0.0225644	0.2788	0.1506822	up
A_21_P0002702	PRKAR2A-AS1	-1.441869	-1.566256	-1.016182	-0.124387	0.4256873	0.1506503	up
A_23_P34496	TMEM39B	2.303833	2.2957177	2.6130733	-0.008115	0.3092403	0.1505625	up
A_23_P500433	CARD9	-0.949118	-0.372418	-1.224732	0.5766997	-0.275614	0.1505427	up
A_22_P00010449	CLUAP1	-0.237864	0.0049663	-0.179692	0.2428303	0.0581718	0.150501	up
A_33_P3361388	MYCBPAP	-3.38006	-3.24832	-3.210941	0.1317401	0.1691196	0.1504299	up
A_23_P123866	UBAP1	2.6566954	2.9182563	2.6959763	0.2615609	0.0392809	0.1504209	up
A_23_P90062	DNAJB1	4.8373585	4.947699	5.02775	0.1103406	0.1903915	0.1503661	up
A_33_P3361337	TMEM184A	-0.494851	-0.03809	-0.650889	0.4567609	-0.156038	0.1503613	up
A_22_P00019960	lnc-CALML6-1	0.8789263	1.1198225	0.9386363	0.2408962	0.05971	0.1503031	up
A_33_P3214199	ZNF532	0.0352359	0.3819513	-0.010957	0.3467155	-0.046193	0.1502612	up
A_33_P3471712	LOC284600	10.022615	10.237513	10.108223	0.2148981	0.0856085	0.1502533	up
A_23_P401700	APBB1IP	-2.008019	-1.846644	-1.868893	0.1613755	0.1391263	0.1502509	up
A_33_P3293798	KRTAP10-1	0.0152321	0.1301441	0.2007995	0.114912	0.1855674	0.1502397	up
A_23_P60286	EIF4B	3.0005388	3.2279	3.0735912	0.2273612	0.0730524	0.1502068	up
A_23_P168551	SLC29A4	-1.558529	-1.459567	-1.357082	0.0989628	0.201447	0.1502049	up
A_23_P124486	PTPN9	-0.878068	-0.639168	-0.816582	0.2389002	0.0614858	0.150193	up
A_23_P162228	NACA	8.034606	8.306202	8.063086	0.271596	0.0284796	0.1500378	up
A_22_P00006058	LINC00087	-1.815157	-1.852943	-1.477333	-0.037787	0.3378234	0.1500182	up
A_23_P150876	VPS37B	4.7297115	5.0429654	4.716426	0.3132539	-0.013286	0.1499841	up
A_32_P416583	NLRC5	-0.514953	-0.386342	-0.343662	0.1286106	0.1712904	0.1499505	up
A_33_P3299865	HIST1H4K	5.897315	6.042044	6.0521803	0.1447291	0.1548653	0.1497972	up
A_21_P0011740	LOC101927522	-1.660354	-1.532618	-1.488518	0.1277356	0.1718359	0.1497858	up
A_33_P3369567	LSP1	1.464982	1.74614	1.4832878	0.281158	0.0183058	0.1497319	up
A_33_P3228325	SP100	2.194025	2.38018	2.307253	0.1861548	0.1132278	0.1496913	up

A_22_P00022336	lnc-ADAMTS18-6	-1.478049	-1.335535	-1.321284	0.1425142	0.156765	0.1496396	up
A_23_P97632	EPRS	5.4974766	5.870809	5.42328	0.3733325	-0.074197	0.1495678	up
A_22_P00005190	lnc-DISP2-2	-2.38425	-2.402201	-2.067176	-0.017951	0.3170743	0.1495619	up
A_22_P00019143	lnc-DHX15-1	-2.183442	-2.176327	-1.891531	0.0071154	0.2919116	0.1495135	up
A_33_P3403132	NR2F6	6.9176893	7.004001	7.13015	0.0863118	0.2124605	0.1493862	up
A_32_P57810	RNF157	-2.11106	-2.32064	-1.602768	-0.209581	0.5082912	0.1493553	up
A_23_P6708	NCBP2-AS2	6.6917543	6.6970906	6.985093	0.0053363	0.2933388	0.1493375	up
A_33_P3314151	CRACR2A	-0.563177	-0.478893	-0.348809	0.0842843	0.2143683	0.1493263	up
A_33_P3283213	MGC57346	-2.365328	-2.293102	-2.139007	0.0722263	0.2263212	0.1492738	up
A_33_P3687198	HRK	1.164012	1.4492807	1.1771712	0.2852688	0.0131593	0.149214	up
A_22_P00009218	lnc-LPPR5.1-1	-1.631155	-1.485912	-1.478013	0.1452427	0.1531425	0.1491926	up
A_23_P64799	AAAS	1.7141695	1.6910863	2.0355768	-0.023083	0.3214073	0.1491621	up
A_24_P116351	SIVA1	5.5705175	5.698323	5.7409906	0.1278052	0.1704731	0.1491392	up
A_22_P00015563	lnc-STOX2-1	-2.723927	-2.510055	-2.639561	0.2138722	0.0843666	0.1491194	up
A_23_P136325	WIPF2	2.1666527	2.4459214	2.1854992	0.2792687	0.0188465	0.1490576	up
A_23_P29029	URB1-AS1	0.9519024	0.9693904	1.232296	0.017488	0.2803936	0.1489408	up
A_21_P0001029	lnc-PRRC2C-1	-2.983346	-2.686702	-2.982187	0.2966435	0.0011585	0.148901	up
A_33_P3312877	CCDC107	3.4708347	3.5376554	3.701808	0.0668206	0.2309732	0.1488969	up
A_32_P198303	DNAJC24	-0.594079	-0.413903	-0.476475	0.1801758	0.1176033	0.1488895	up
A_23_P27744	FBXO46	3.5564623	3.691341	3.7193432	0.1348786	0.1628809	0.1488798	up
A_33_P3365864	BSDC1	-0.935825	-0.784473	-0.789426	0.1513524	0.1463995	0.148876	up
A_24_P289636	NGRN	3.6612177	3.864038	3.7561035	0.2028203	0.0948858	0.1488531	up
A_23_P51699	ARHGEF2	0.5050993	0.6119962	0.6954169	0.1068969	0.1903176	0.1486073	up
A_22_P00015995	TEX261	-1.611409	-1.468422	-1.457236	0.1429868	0.1541724	0.1485796	up
A_23_P305692	ELMOD2	-0.79193	-0.642682	-0.644143	0.1492481	0.1477866	0.1485174	up
A_23_P161125	MOV10	1.8371782	1.9182096	2.0531797	0.0810313	0.2160015	0.1485164	up
A_22_P00009735	BTBD9	-2.81102	-2.706247	-2.618805	0.104773	0.1922147	0.1484939	up
A_22_P00025412	lnc-UCHL5-2	-3.240321	-3.077375	-3.106288	0.162946	0.134033	0.1484895	up
A_23_P119102	VASP	4.791526	5.056068	4.8236656	0.2645421	0.0321398	0.1483409	up
A_32_P91250	UBE2L3	5.1017246	5.276714	5.223282	0.1749892	0.1215572	0.1482732	up
A_23_P420269	INTS2	-2.889801	-2.950834	-2.532312	-0.061033	0.3574889	0.1482277	up
A_23_P305140	C10orf32	2.5404196	2.7151084	2.6621037	0.1746888	0.1216841	0.1481864	up
A_21_P0008715	lnc-GABRB3-1	-1.562633	-1.295365	-1.533754	0.2672677	0.0288792	0.1480734	up
A_24_P237175	CST2	-0.793885	-0.530142	-0.761497	0.2637429	0.0323877	0.1480653	up
A_23_P166023	PFDN4	3.290205	3.3948388	3.4813395	0.1046338	0.1911345	0.1478841	up
A_23_P156667	PPP1R10	2.7508307	3.0605922	2.7368307	0.3097615	-0.014	0.1478808	up
A_21_P0000055	LYRM4	0.2631397	0.5895815	0.2324591	0.3264418	-0.030681	0.1478806	up
A_33_P3257628	COG4	1.1811862	1.4202175	1.2378626	0.2390313	0.0566764	0.1478538	up
A_24_P345377	GOLPH3L	3.194479	3.4321666	3.2523127	0.2376876	0.0578337	0.1477606	up
A_23_P132405	ACAD9	5.602602	5.679216	5.821492	0.0766139	0.2188902	0.147752	up
A_33_P3374190	INPP5A	-1.097942	-0.89538	-1.005015	0.2025619	0.0929265	0.1477442	up
A_24_P25346	CIRH1A	4.44728	4.547952	4.6420593	0.1006722	0.1947794	0.1477258	up
A_23_P77593	VAC14	-0.725751	-0.639123	-0.51694	0.086628	0.2088108	0.1477194	up
A_23_P200386	KHDRBS1	5.4611425	5.5525966	5.665104	0.091454	0.2039614	0.1477077	up
A_21_P0002497	lnc-C2orf76-1	-1.66491	-1.393078	-1.641397	0.271832	0.0235133	0.1476727	up
A_33_P3252989	ZSWIM8	3.262948	3.5801435	3.2410946	0.3171954	-0.021853	0.147671	up
A_24_P36847	DHX9	2.9823446	3.0531383	3.2067099	0.0707936	0.2243652	0.1475794	up
A_24_P250650	RABL2A	4.0752983	4.2980313	4.1476374	0.222733	0.0723391	0.147536	up
A_23_P304171	KIAA0226	3.640584	3.7030778	3.8731174	0.0624938	0.2325335	0.1475136	up
A_23_P160720	BATF3	0.4627032	0.146482	1.0738893	-0.316221	0.611186	0.1474824	up
A_22_P00018341	ZRANB1	0.1683936	0.3872066	0.2443676	0.2188129	0.075974	0.1473935	up
A_33_P3314643	SPEF1	-0.358192	-0.140893	-0.280746	0.217299	0.077446	0.1473725	up
A_24_P701776	ARHGEF35	0.2761231	0.2437758	0.6031346	-0.032347	0.3270116	0.1473322	up
A_21_P0000897	LOXL1-AS1	-0.629763	-0.442594	-0.522342	0.1871686	0.1074209	0.1472948	up
A_21_P0000385	SNORD88C	-0.360967	-0.276403	-0.151069	0.0845637	0.209898	0.1472309	up
A_33_P3388283	HMGCL	-2.838341	-2.70198	-2.680257	0.1363614	0.1580846	0.147223	up

A_24_P88800	COX7A2L	2.5547743	2.8539672	2.549943	0.2991929	-0.004831	0.1471808	up
A_33_P3869455	ATXN8	-0.72915	-0.620145	-0.543856	0.1090045	0.1852942	0.1471493	up
A_19_P00802954	LOC441239	-2.865881	-2.663745	-2.773783	0.2021356	0.0920975	0.1471165	up
A_24_P6903	ACTBL2	1.8585005	1.8665848	2.1445875	0.0080843	0.286087	0.1470857	up
A_33_P3239854	ALKBH5	0.4232779	0.5470929	0.5936279	0.1238151	0.1703501	0.1470826	up
A_23_P49412	PRR14	0.5413313	0.5937991	0.7829256	0.0524678	0.2415943	0.1470311	up
A_23_P116414	PLA2G16	6.927165	6.9742303	7.174095	0.0470653	0.2469301	0.1469977	up
A_23_P55011	SLC38A10	5.2105417	5.4271502	5.2878513	0.2166085	0.0773096	0.1469591	up
A_23_P106389	SEMA7A	-0.316644	-0.254755	-0.084676	0.0618897	0.2319684	0.146929	up
A_23_P413788	FUT11	-0.870051	-0.674284	-0.772011	0.1957669	0.0980401	0.1469035	up
A_23_P91390	THBD	3.5005188	4.047704	3.2470179	0.5471854	-0.253501	0.1468422	up
A_21_P0000209	SNORD4B	-1.190207	-1.132085	-0.954654	0.0581222	0.2355533	0.1468377	up
A_21_P0003116	lnc-CDC25A-1	-0.910992	-0.873413	-0.654922	0.0375791	0.2560697	0.1468244	up
A_23_P31536	SSBP1	5.5663557	5.5940623	5.832288	0.0277066	0.2659321	0.1468194	up
A_33_P3263666	ANKRD9	3.4816093	3.5213609	3.7354202	0.0397515	0.2538109	0.1467812	up
A_22_P00000346	NR2F2-AS1	-2.661462	-2.673556	-2.35605	-0.012094	0.3054118	0.1466587	up
A_21_P0001800	lnc-TNP1-3	-2.41538	-2.292972	-2.244513	0.1224079	0.1708674	0.1466377	up
A_22_P00004984	LOC90768	-1.598811	-1.5297	-1.374858	0.0691109	0.2239533	0.1465321	up
A_23_P144005	SSUH2	-0.739008	-0.386072	-0.798885	0.3529367	-0.059877	0.1465299	up
A_22_P00010173	FGD5-AS1	3.6021547	3.6681695	3.8291235	0.0660148	0.2269688	0.1464918	up
A_33_P3317211	MECP2	-1.191365	-1.0173	-1.072462	0.1740646	0.1189027	0.1464837	up
A_33_P3210085	NET1	3.7944002	3.8842025	3.9975204	0.0898023	0.2031202	0.1464612	up
A_19_P00317444	LOC389602	-0.948468	-0.699567	-0.904477	0.2489014	0.0439916	0.1464465	up
A_19_P00812723	lnc-ZADH2-1	-2.562078	-2.447225	-2.384164	0.1148529	0.1779141	0.1463835	up
A_32_P54137	UQCRH	8.582191	8.694008	8.763026	0.1118164	0.1808348	0.1463256	up
A_23_P45864	TNR	-2.599509	-1.907438	-2.998994	0.6920712	-0.399485	0.1462933	up
A_33_P3341239	CDK16	6.027878	5.992848	6.3554316	-0.03503	0.3275538	0.1462619	up
A_23_P163278	PARP16	0.0536404	0.4675121	-0.067847	0.4138718	-0.121487	0.1461923	up
A_24_P196372	AGBL5	-0.924972	-0.673927	-0.883666	0.2510452	0.041306	0.1461756	up
A_23_P34176	WWC3	-2.866582	-2.87662	-2.564202	-0.010037	0.3023806	0.1461716	up
A_24_P212234	SLC6A18	-2.263853	-2.2564	-1.979024	0.0074522	0.2848287	0.1461405	up
A_24_P182764	ATG4B	3.3438635	3.4374108	3.5425243	0.0935473	0.1986609	0.1461041	up
A_23_P130304	TXNL4A	6.414756	6.5027623	6.6188593	0.0880065	0.2041035	0.146055	up
A_24_P134816	BCL9L	0.9593391	1.3289099	0.8818364	0.3695707	-0.077503	0.146034	up
A_21_P0010267	lnc-TPTE-3	3.4020634	3.56247	3.53372	0.1604066	0.1316567	0.1460316	up
A_21_P0000800	RNASEH1-AS1	-0.334449	-0.253573	-0.123335	0.0808754	0.2111135	0.1459944	up
A_24_P630490	DFNB59	-3.278946	-2.837621	-3.428332	0.4413259	-0.149386	0.1459701	up
A_32_P224522	SLC25A23	2.3477468	2.3229823	2.6643276	-0.024765	0.3165808	0.1459081	up
A_23_P26847	SOX9	0.9796791	1.093163	1.1579018	0.1134839	0.1782227	0.1458533	up
A_22_P00021796	lnc-TM7SF2-1	-0.24266	-0.097985	-0.095709	0.1446748	0.1469502	0.1458125	up
A_21_P0010893	NUTM2B-AS1	1.4967895	1.5670509	1.7180905	0.0702615	0.2213011	0.1457813	up
A_23_P134517	PURB	2.1672907	2.3862824	2.239812	0.2189918	0.0725212	0.1457565	up
A_23_P218654	ZGPAT	4.263397	4.307511	4.510681	0.0441136	0.2472839	0.1456988	up
A_24_P500891	AK2	5.4370565	5.4871154	5.678195	0.0500588	0.2411385	0.1455987	up
A_33_P3346102	TAOK3	1.5418706	1.7446766	1.6302114	0.202806	0.0883408	0.1455734	up
A_23_P168019	ZKSCAN3	-3.086212	-2.735454	-3.145937	0.3507578	-0.059726	0.1455162	up
A_33_P3277178	SSPO	-1.724659	-1.631705	-1.526635	0.0929542	0.1980238	0.145489	up
A_33_P3370751	SMURF2	-1.219972	-1.28473	-0.864306	-0.064758	0.3556657	0.1454537	up
A_23_P150281	TP53I11	-0.758062	-0.811605	-0.413721	-0.053543	0.3443418	0.1453993	up
A_22_P00003909	lnc-CERK-1	2.8237476	2.729464	3.2087107	-0.094284	0.384963	0.1453397	up
A_23_P20196	ARPC1B	4.720811	4.7466426	4.9854736	0.0258317	0.2646627	0.1452472	up
A_33_P3413463	BRF1	3.9692707	4.0305734	4.1984463	0.0613027	0.2291756	0.1452391	up
A_23_P132793	MANF	5.994335	6.0510325	6.2281	0.0566974	0.2337647	0.145231	up
A_33_P3221761	KMT2B	-1.755904	-1.553675	-1.667748	0.202229	0.0881567	0.1451929	up
A_22_P00018132	lnc-ZNF503-2	-0.250389	-0.215654	0.0052562	0.0347347	0.2556453	0.14519	up
A_22_P00009356	lnc-LRRTM1-2	-2.126756	-2.033858	-1.929436	0.0928974	0.19732	0.1451087	up

A_33_P3339103	POLR1C	4.861068	5.0174146	4.994871	0.1563468	0.1338034	0.1450751	up
A_23_P12911	C11orf24	4.830515	4.8290343	5.122113	-0.001481	0.2915983	0.1450589	up
A_23_P53162	STIM1	0.7941008	0.8985543	0.9797049	0.1044536	0.1856041	0.1450288	up
A_23_P37391	CCDC85C	3.6806355	3.6985068	3.9526281	0.0178714	0.2719927	0.144932	up
A_21_P0001705	LINC00869	4.405448	4.5351377	4.565523	0.1296897	0.1600752	0.1448824	up
A_24_P43723	GJC3	-2.84932	-3.073038	-2.335883	-0.223718	0.5134368	0.1448594	up
A_23_P74359	CSRP1	3.6302605	3.5501285	3.9999323	-0.080132	0.3696718	0.1447699	up
A_24_P159181	PDCD7	3.5633726	3.6111732	3.805066	0.0478005	0.2416935	0.144747	up
A_23_P256021	LAS1L	5.063452	5.1741405	5.242235	0.1106887	0.1787834	0.1447361	up
A_24_P296457	PHKG2	-0.227748	-0.246671	0.0805383	-0.018923	0.3082862	0.1446817	up
A_24_P272761	DENND1A	-0.424452	-0.103636	-0.455925	0.320816	-0.031473	0.1446714	up
A_22_P00005663	LOC102724671	-2.096492	-1.970935	-1.932782	0.1255565	0.1637101	0.1446333	up
A_24_P6125	KCNJ4	-3.302938	-2.638463	-3.678181	0.664475	-0.375243	0.1446158	up
A_23_P107933	VRK3	1.2353945	1.0461044	1.7137661	-0.18929	0.4783716	0.1445408	up
A_23_P56567	GEMIN6	5.0544024	5.0817523	5.3161335	0.0273499	0.2617312	0.1445405	up
A_21_P0002044	lnc-SIX2-3	-2.78682	-2.885651	-2.398968	-0.098832	0.3878512	0.1445098	up
A_23_P156289	OSMR	-1.720866	-1.321391	-1.831349	0.3994751	-0.110483	0.144496	up
A_32_P56392	RBMX	-0.245733	0.1960564	-0.39888	0.4417896	-0.153147	0.1443214	up
A_33_P3235642	RBBP5	-1.26013	-1.313154	-0.918472	-0.053024	0.3416576	0.1443167	up
A_23_P121182	HACL1	3.3488083	3.5412374	3.4450102	0.1924291	0.0962019	0.1443155	up
A_33_P3269924	HIP1R	-0.931726	-0.75182	-0.823059	0.1799064	0.1086669	0.1442866	up
A_19_P00321009	LINC01133	2.0296125	2.2717748	2.075983	0.2421622	0.0463705	0.1442664	up
A_33_P3267948	SLC25A23	-1.513942	-1.182195	-1.557196	0.3317471	-0.043254	0.1442466	up
A_21_P0004304	lnc-CXXC5-1	-1.524908	-1.583549	-1.178069	-0.05864	0.346839	0.1440992	up
A_23_P395426	DIDO1	2.1732798	2.2791696	2.3551702	0.1058898	0.1818905	0.1438901	up
A_23_P97860	LIPA	4.6813326	4.690802	4.959611	0.0094695	0.2782784	0.1438739	up
A_33_P3271316	RPP25	-0.435539	-0.223085	-0.360252	0.2124538	0.0752869	0.1438704	up
A_21_P0000586	TTC3P1	0.4877091	0.4743156	0.7888384	-0.013393	0.3011293	0.143868	up
A_21_P0006547	lnc-KIAA1210-2	-1.474625	-1.540972	-1.120585	-0.066347	0.3540402	0.1438468	up
A_24_P390070	SRSF9	4.3017483	4.4347005	4.456479	0.1329522	0.1547308	0.1438415	up
A_23_P11744	WASH1	5.1768236	5.30946	5.331852	0.1326366	0.1550283	0.1438324	up
A_24_P381136	PACSIN3	1.2047877	1.2601752	1.4369063	0.0553875	0.2321186	0.143753	up
A_24_P175563	ACBD5	-3.277588	-2.95375	-3.313988	0.323838	-0.0364	0.143719	up
A_33_P3278347	B3GALT5-AS1	2.124632	2.159162	2.37753	0.0345302	0.2528982	0.1437142	up
A_23_P168443	EPHB4	-0.65333	-0.547511	-0.471778	0.1058192	0.1815519	0.1436856	up
A_23_P28969	CHMP4B	4.398733	4.420881	4.6638927	0.0221477	0.2651596	0.1436536	up
A_23_P209694	PAPOLG	1.5947886	1.7495856	1.7272658	0.1547971	0.1324773	0.1436372	up
A_22_P00007462	PKN2-AS1	-2.864251	-2.630041	-2.811386	0.2342103	0.052865	0.1435376	up
A_33_P3327058	ARHGEF2	-1.793539	-1.589304	-1.710814	0.2042341	0.0827251	0.1434796	up
A_23_P106741	PSMD7	5.7428846	5.858231	5.914442	0.1153464	0.1715574	0.1434519	up
A_24_P329487	FAM84B	2.598011	2.9787564	2.5040312	0.3807454	-0.09398	0.1433828	up
A_23_P384761	GATA4	0.3694525	0.3808584	0.6448045	0.0114059	0.275352	0.143379	up
A_23_P396626	SYNRG	-0.742933	-0.83662	-0.362786	-0.093688	0.3801465	0.1432295	up
A_23_P129118	PDCD7	1.6803169	1.8463569	1.8006635	0.1660399	0.1203465	0.1431932	up
A_21_P0013802	TMLHE	0.8697739	0.9991307	1.026763	0.1293569	0.1569891	0.143173	up
A_33_P3246418	MDFI	6.150834	6.0939555	6.494048	-0.056879	0.343214	0.1431677	up
A_33_P3284290	ZNF778	-1.905812	-1.674679	-1.850631	0.231133	0.0551806	0.1431568	up
A_33_P3273732	lnc-BRD3-1	-0.516892	-0.292524	-0.454965	0.2243686	0.0619273	0.1431479	up
A_23_P412389	FGF18	-2.090643	-1.770066	-2.124929	0.3205771	-0.034286	0.1431456	up
A_23_P91015	SMYD5	2.1502838	2.119248	2.4675426	-0.031036	0.3172588	0.1431115	up
A_23_P251095	PDHA1	6.0205717	6.1370416	6.190299	0.1164699	0.1697273	0.1430986	up
A_23_P99642	SLC7A7	0.2526465	0.3664455	0.4249272	0.1137991	0.1722808	0.1430399	up
A_21_P0009533	lnc-RP11-863N1.2.1-1	-0.784392	-0.669911	-0.612814	0.1144805	0.1715775	0.143029	up
A_33_P3322945	SPDYC	-2.981982	-2.896106	-2.781808	0.085876	0.2001736	0.1430248	up
A_23_P37545	AAGAB	4.688529	4.7111573	4.9518976	0.0226283	0.2633686	0.1429985	up

A_23_P26325	CCL17	-3.152902	-3.111759	-2.908082	0.0411427	0.2448199	0.1429813	up
A_23_P323563	PLEKHG2	2.3905516	2.4364696	2.6305838	0.045918	0.2400322	0.1429751	up
A_24_P142269	HIRIP3	-0.723971	-0.796032	-0.365984	-0.072062	0.3579874	0.1429629	up
A_33_P3258510	LYPLA2	0.6212874	0.5665412	0.9619474	-0.054746	0.3406601	0.142957	up
A_24_P224776	LSM4	4.7398243	4.8015723	4.963929	0.061748	0.2241049	0.1429265	up
A_33_P3326892	lnc-AC112512.1-1	-2.530492	-2.408047	-2.367131	0.1224453	0.1633611	0.1429032	up
A_23_P53198	DGAT2	0.8236775	0.9634566	0.9696412	0.1397791	0.1459637	0.1428714	up
A_33_P3293474	OPN3	-1.35517	-1.077531	-1.347209	0.2776384	0.0079608	0.1427996	up
A_33_P3298387	PLK1	-0.322804	-0.213911	-0.146286	0.1088929	0.1765184	0.1427057	up
A_23_P24616	SIAE	0.4187198	0.5981002	0.524693	0.1793804	0.1059732	0.1426768	up
A_21_P0014173	LOC102724889	-2.670652	-2.500077	-2.555931	0.1705747	0.1147213	0.142648	up
A_23_P98402	SIDT2	2.7491446	2.8300986	2.9534454	0.0809541	0.2043009	0.1426275	up
A_33_P3256894	FAM21C	0.9945207	1.2592053	1.0149779	0.2646847	0.0204573	0.142571	up
A_33_P3313622	MIR17HG	0.7052617	1.0075688	0.6880779	0.3023071	-0.017184	0.1425617	up
A_21_P0000342	SNORA5B	0.0971322	-0.05127	0.5305734	-0.148403	0.4334412	0.1425192	up
A_23_P387943	CASP2	1.7046976	1.9127321	1.78162	0.2080345	0.0769224	0.1424785	up
A_32_P223173	LOC102725053	1.4120331	1.5880184	1.5209274	0.1759853	0.1088944	0.1424398	up
A_33_P3251984	MRGPRF	-2.462196	-2.243059	-2.396474	0.2191365	0.0657213	0.1424289	up
A_33_P3334908	DHRSX	-1.877503	-2.052035	-1.418318	-0.174532	0.4591847	0.1423264	up
A_33_P3302796	U2AF2	7.68083	7.57188	8.074318	-0.10895	0.3934879	0.1422689	up
A_33_P3258946	SIRT2	-0.644065	-0.463724	-0.539934	0.1803412	0.1041312	0.1422362	up
A_23_P126605	TMOD4	-2.461654	-2.08192	-2.556983	0.3797343	-0.095329	0.1422027	up
A_24_P108779	KCTD17	0.1080737	-0.009439	0.5099516	-0.117512	0.4018779	0.1421828	up
A_21_P0011241	XLOC_l2_004180	-2.934856	-2.880523	-2.704844	0.054333	0.2300112	0.1421721	up
A_33_P3323448	HM13	-2.661614	-2.767472	-2.271651	-0.105858	0.3899627	0.1420524	up
A_32_P18440	ARID5B	2.2840233	2.5071416	2.3449516	0.2231183	0.0609283	0.1420233	up
A_32_P420009	ALS2CL	-0.56821	-0.431527	-0.42109	0.136683	0.14712	0.1419015	up
A_24_P403734	ZNF385A	3.8344297	3.990107	3.9624367	0.1556773	0.1280069	0.1418421	up
A_24_P302374	CLCN6	1.0052795	1.360704	0.9333859	0.3554244	-0.071894	0.1417654	up
A_22_P00004110	lnc-CLCN3-1	-0.658256	-0.448206	-0.584853	0.2100501	0.0734034	0.1417267	up
A_33_P8922891	LOC101927751	-1.970867	-2.068677	-1.589726	-0.09781	0.3811402	0.141665	up
A_33_P3276323	RAD21-AS1	-2.955744	-2.795939	-2.832304	0.1598051	0.1234396	0.1416223	up
A_33_P3280044	ANKRD11	5.02756	5.365744	4.9725866	0.3381839	-0.054974	0.1416051	up
A_23_P42116	PPT2	1.6943645	1.6105089	2.0614214	-0.083856	0.3670569	0.1416006	up
A_33_P6820772	PDCD4-AS1	-1.838663	-1.699858	-1.694272	0.1388044	0.1443906	0.1415975	up
A_23_P59836	METTL2B	3.0047808	2.9902415	3.3023357	-0.014539	0.297555	0.1415079	up
A_22_P00001377	ILF3-AS1	-0.012589	-0.174359	0.4320426	-0.161769	0.4446321	0.1414313	up
A_23_P394917	SRCAP	-1.123178	-0.85976	-1.103791	0.2634177	0.0193868	0.1414022	up
A_33_P3350566	CACNA1I	-2.439714	-2.561277	-2.035533	-0.121563	0.4041805	0.1413088	up
A_23_P257091	NUP214	2.0597792	2.0120473	2.3901005	-0.047732	0.3303213	0.1412947	up
A_23_P500300	TRIM15	2.9718046	2.899549	3.3263397	-0.072256	0.3545351	0.1411397	up
A_19_P00321089	LINC00963	0.2739935	0.5180373	0.3122163	0.2440438	0.0382228	0.1411333	up
A_22_P00001898	lnc-B4GALT3-1	-2.413474	-1.976545	-2.568166	0.4369292	-0.154692	0.1411187	up
A_23_P202501	RNLS	-1.172114	-1.774964	-0.28705	-0.60285	0.8850646	0.1411073	up
A_21_P0000703	ZNF695	-1.187801	-0.69579	-1.397681	0.4920111	-0.20988	0.1410656	up
A_21_P0010986	U2AF1	4.5994043	4.680985	4.7999105	0.0815806	0.2005062	0.1410434	up
A_23_P216920	NEK6	2.0963268	2.0613656	2.4128904	-0.034961	0.3165636	0.1408012	up
A_33_P3300267	VIT	-1.941533	-1.805059	-1.796427	0.1364741	0.1451058	0.14079	up
A_21_P0012427	XLOC_l2_010064	0.7277842	1.1024194	0.6346388	0.3746352	-0.093145	0.1407449	up
A_33_P3325871	FLJ38668	-1.58563	-1.380745	-1.509037	0.204885	0.0765929	0.140739	up
A_33_P3275199	JRK	1.7213078	1.8124137	1.9115925	0.0911059	0.1902847	0.1406953	up
A_22_P00024841	NSFL1C	1.5314646	1.7163815	1.6278739	0.184917	0.0964093	0.1406631	up
A_23_P81650	C5orf15	3.9190063	4.092359	4.0268774	0.1733527	0.1078711	0.1406119	up
A_23_P24586	ACCS	-0.852654	-0.635956	-0.788196	0.2166982	0.0644584	0.1405783	up
A_24_P625382	YBX3	4.6134443	4.7945857	4.7133865	0.1811414	0.0999422	0.1405418	up
A_23_P500734	POGZ	1.9233484	2.0430312	2.0847368	0.1196828	0.1613884	0.1405356	up

A_21_P0001252	lnc-TMEM88B-4	-1.460602	-1.355188	-1.285098	0.1054134	0.1755042	0.1404588	up
A_23_P138760	CLCF1	0.2687998	0.4943285	0.3239007	0.2255287	0.0551009	0.1403148	up
A_23_P123256	PDAP1	3.6097422	3.6233172	3.8767061	0.0135751	0.266964	0.1402695	up
A_22_P00010239	lnc-MTA2-1	-2.482091	-2.356452	-2.32738	0.1256383	0.154711	0.1401746	up
A_32_P524014	UTRN	2.6211414	2.6937604	2.8288145	0.072619	0.2076731	0.140146	up
A_33_P3368540	SRGAP3	0.9626026	1.0432262	1.1621695	0.0806236	0.1995668	0.1400952	up
A_23_P145	HMGCL	1.334506	1.507143	1.4419913	0.172637	0.1074853	0.1400611	up
A_33_P3651948	NEO1	-1.079937	-0.978544	-0.90121	0.1013932	0.1787267	0.1400599	up
A_23_P76015	ARHGEF17	2.3386192	2.6255364	2.3317738	0.2869172	-0.006845	0.1400359	up
A_24_P92256	SGSM2	-1.571966	-1.393027	-1.470909	0.1789384	0.1010566	0.1399975	up
A_23_P74653	NUDC	4.145626	4.179477	4.391575	0.0338511	0.2459488	0.1399	up
A_23_P96041	TMEM164	4.346545	4.4483485	4.524541	0.1018033	0.1779957	0.1398995	up
A_33_P3398526	BCL2L11	1.0240335	1.2265253	1.1012669	0.2024918	0.0772333	0.1398625	up
A_33_P3405921	GAS8	2.6329088	2.8267512	2.718563	0.1938424	0.0856543	0.1397483	up
A_33_P3221303	CCR10	-3.141298	-2.669959	-3.333141	0.471339	-0.191843	0.1397479	up
A_19_P00812715	XLOC_l2_010118	-2.014385	-1.964443	-1.78489	0.0499425	0.2294955	0.139719	up
A_22_P00015773	LOC100996579	-0.036381	0.2095251	-0.002852	0.2459064	0.0335288	0.1397176	up
A_23_P166536	BRD1	3.3247814	3.4750857	3.4538908	0.1503043	0.1291094	0.1397069	up
A_33_P3358253	INPPL1	1.5582132	1.7145128	1.6813021	0.1562996	0.1230888	0.1396942	up
A_33_P3366124	KANTR	3.2783957	3.3405452	3.4956322	0.0621495	0.2172365	0.139693	up
A_33_P3570208	LPP-AS2	-1.447099	-1.279861	-1.335014	0.1672378	0.1120844	0.1396611	up
A_23_P132277	MCM5	4.7736893	4.886267	4.940338	0.1125779	0.1666489	0.1396134	up
A_21_P0003579	lnc-CPEB2-5	2.1791525	2.3737216	2.2637625	0.1945691	0.08461	0.1395895	up
A_23_P324754	CEMIP	3.038043	3.1452785	3.2099342	0.1072354	0.1718912	0.1395633	up
A_23_P22119	PLEC	5.4247713	5.439201	5.6894608	0.0144296	0.2646895	0.1395595	up
A_22_P00007228	lnc-GPER-3	-2.499426	-2.15551	-2.564255	0.3439159	-0.064829	0.1395434	up
A_19_P00318587	LINC01023	1.7707748	1.7782946	2.0420704	0.0075197	0.2712956	0.1394076	up
A_21_P0011813	TMEM128	1.5558472	1.7662015	1.6242685	0.2103543	0.0684214	0.1393878	up
A_22_P00001875	LOC100419583	0.1933394	0.0521617	0.613256	-0.141178	0.4199166	0.1393695	up
A_23_P58983	TBC1D22B	0.0008454	0.3276615	-0.04742	0.3268161	-0.048265	0.1392753	up
A_24_P112377	DDX31	-1.673734	-1.592556	-1.476475	0.0811777	0.197259	0.1392183	up
A_21_P0011605	AMZ2P1	0.6044927	0.8356299	0.6516757	0.2311373	0.047183	0.1391602	up
A_23_P3911	PLXDC1	-0.722859	-0.466266	-0.701193	0.2565932	0.0216661	0.1391296	up
A_23_P170649	SBSPON	0.8437095	0.8704233	1.0952525	0.0267138	0.2515431	0.1391284	up
A_22_P00009293	lnc-LRRC32-5	-2.441465	-2.531443	-2.073308	-0.089978	0.3681574	0.13909	up
A_22_P00001741	C11orf95	-1.322444	-1.248711	-1.118025	0.0737324	0.2044187	0.1390755	up
A_23_P212042	MFI2	-0.699921	-0.422009	-0.699744	0.2779117	0.0001769	0.1390443	up
A_23_P152028	TRIP4	3.0469666	3.1991725	3.1728249	0.1522059	0.1258583	0.1390321	up
A_23_P355439	HIST1H2AA	-2.456906	-2.311065	-2.324701	0.1458406	0.1322045	0.1390226	up
A_24_P49199	GLDN	-0.953726	-0.377964	-1.251512	0.5757628	-0.297785	0.1389887	up
A_24_P65910	UQCC1	2.0169687	1.9718347	2.3399744	-0.045134	0.3230057	0.1389358	up
A_21_P0014676	OR7E47P	-1.798148	-1.411213	-1.907231	0.3869352	-0.109083	0.1389263	up
A_23_P208698	GYS1	3.491065	3.6962132	3.563736	0.2051482	0.0726709	0.1389096	up
A_24_P322611	DIDO1	1.7024364	1.7777467	1.904892	0.0753102	0.2024555	0.1388829	up
A_23_P10936	KRTAP4-1	0.7117548	0.5552483	1.1458826	-0.156507	0.4341278	0.1388106	up
A_24_P141332	CAMK2G	3.8236694	4.0353904	3.8894424	0.2117209	0.065773	0.138747	up
A_32_P7823	MAPK1IP1L	2.0851355	2.0388503	2.4088755	-0.046285	0.32374	0.1387274	up
A_23_P160862	HNRNPU	5.5955563	5.7939763	5.674549	0.1984201	0.0789928	0.1387064	up
A_23_P124733	COQ2	-0.135927	0.0122943	-0.006836	0.148221	0.1290908	0.1386559	up
A_23_P90273	CHST8	-2.48346	-2.019935	-2.669745	0.4635248	-0.186285	0.1386199	up
A_23_P102965	BCL2L13	2.987298	3.198711	3.053115	0.2114129	0.0658169	0.1386149	up
A_23_P109133	AVP	-1.480294	-1.020903	-1.662562	0.4593916	-0.182268	0.138562	up
A_23_P204423	PPP1CC	3.550661	3.9016051	3.4767208	0.350944	-0.07394	0.1385019	up
A_33_P3276703	VGF	2.3889236	2.423531	2.6312914	0.0346074	0.2423677	0.1384876	up
A_22_P00015589	STX6	1.3813004	1.562408	1.4771309	0.1811075	0.0958304	0.138469	up
A_33_P3394828	NOC4L	4.1223707	3.9335685	4.588106	-0.188802	0.4657354	0.1384666	up

A_23_P8582	FAM126A	-1.119024	-0.556757	-1.404378	0.5622678	-0.285354	0.1384571	up
A_23_P71419	COPS5	4.6759424	4.7738767	4.854826	0.0979342	0.1788836	0.1384089	up
A_19_P00320314	FLJ32255	0.579175	0.8701544	0.5649018	0.2909794	-0.014273	0.1383531	up
A_22_P00012087	lnc-PMM2-5	2.6520195	2.8677506	2.7129793	0.2157311	0.0609598	0.1383455	up
A_24_P256380	WLS	0.4109268	0.5567012	0.5417557	0.1457744	0.1308289	0.1383016	up
A_23_P58280	GAR1	5.978612	6.0675874	6.1661215	0.0889754	0.1875095	0.1382425	up
A_23_P24444	DHCR7	6.58278	6.4147696	7.0272493	-0.16801	0.4444695	0.1382296	up
A_33_P3363898	TUBG1	6.078142	6.072059	6.3606434	-0.006083	0.2825012	0.1382091	up
A_22_P00010807	LINC00970	-2.292169	-2.031243	-2.27677	0.2609258	0.0153985	0.1381621	up
A_23_P66563	SMG8	1.823555	2.0004668	1.922885	0.1769118	0.09933	0.1381209	up
A_22_P00017851	lnc-ZCCHC7-3	-1.9349	-1.725061	-1.868532	0.2098393	0.0663681	0.1381037	up
A_23_P69493	RHOA	4.1598635	4.3015513	4.2942133	0.1416879	0.1343498	0.1380188	up
A_23_P18739	TMEM184C	-0.042109	0.0579658	0.1338277	0.1000752	0.1759372	0.1380062	up
A_24_P216681	LSM10	3.799347	3.8671355	4.007559	0.0677886	0.2082119	0.1380003	up
A_21_P0003110	lnc-GLB1-1	-2.486057	-2.521291	-2.175074	-0.035234	0.3109827	0.1378745	up
A_33_P3378665	TC2N	0.0650954	0.0003071	0.4055557	-0.064788	0.3404603	0.137836	up
A_24_P167473	ARPC3	7.002205	7.0832934	7.1965103	0.0810885	0.1943054	0.137697	up
A_23_P304682	EMP2	5.288886	5.3657565	5.48738	0.0768704	0.198494	0.1376822	up
A_21_P0000367	SNORA32	1.9386854	2.056271	2.096405	0.1175857	0.1577196	0.1376526	up
A_24_P295806	CHID1	1.7586775	1.8554292	1.9370909	0.0967517	0.1784134	0.1375825	up
A_23_P42241	OR5V1	-1.615315	-1.679328	-1.276182	-0.064012	0.3391337	0.1375608	up
A_23_P351734	NPHP4	0.6600156	0.7738881	0.8211756	0.1138725	0.16116	0.1375163	up
A_24_P118376	CEACAM20	1.326549	1.5169377	1.411149	0.1903887	0.0846	0.1374943	up
A_33_P3369956	ATXN2L	1.1841497	1.4488473	1.1943903	0.2646976	0.0102406	0.1374691	up
A_33_P3229397	CCT4	7.275795	7.411179	7.415262	0.1353841	0.1394672	0.1374257	up
A_23_P209394	CFLAR	2.4991312	2.557736	2.7153234	0.0586047	0.2161923	0.1373985	up
A_24_P50248	FAM110C	-0.793834	-0.397573	-0.915363	0.3962603	-0.121529	0.1373656	up
A_33_P3364626	TMEM194B	-1.180643	-1.010077	-1.076498	0.1705656	0.1041446	0.1373551	up
A_33_P3336780	ABCB8	0.5304785	0.6151533	0.720408	0.0846748	0.1899295	0.1373022	up
A_33_P3417452	ZGLP1	1.3414211	1.6075397	1.3498983	0.2661185	0.0084772	0.1372979	up
A_23_P100220	ESRP2	4.715987	4.7568307	4.9496775	0.0408435	0.2336903	0.1372669	up
A_23_P436138	MAX	-1.362604	-1.148628	-1.302146	0.2139759	0.0604572	0.1372166	up
A_23_P162120	NUMA1	1.4148598	1.5669045	1.5371618	0.1520448	0.1223021	0.1371734	up
A_22_P00001223	lnc-ANKRD13B-1	-1.18917	-1.148016	-0.95603	0.0411539	0.23314	0.1371469	up
A_33_P3295066	RPL23P8	8.957302	9.185734	9.003043	0.2284317	0.0457411	0.1370864	up
A_24_P281580	GATS	-1.251413	-1.139278	-1.089522	0.1121359	0.161891	0.1370134	up
A_23_P250156	IGF2BP2	2.314086	2.4410434	2.4610558	0.1269574	0.1469698	0.1369636	up
A_23_P333705	NEK3	0.6349063	0.5226803	1.0210443	-0.112226	0.386138	0.136956	up
A_23_P52939	SLC43A1	-1.139805	-1.030043	-0.975854	0.1097627	0.1639514	0.136857	up
A_21_P0000370	SNORD6	-0.781757	-0.630499	-0.659447	0.1512585	0.1223102	0.1367843	up
A_33_P3327333	IRF3	3.0063972	3.0340343	3.2522917	0.027637	0.2458944	0.1367657	up
A_21_P0014651	LOC100129203	-1.495762	-1.450623	-1.267397	0.0451388	0.2283649	0.1367519	up
A_23_P39799	LOXL3	-1.466072	-1.34085	-1.317822	0.1252213	0.1482501	0.1367357	up
A_33_P3338186	HEXDC	3.533781	3.789948	3.5510244	0.2561669	0.0172434	0.1367052	up
A_22_P00017934	lnc-ZFP90-2	-2.967776	-2.703654	-2.958615	0.2641225	0.009161	0.1366417	up
A_23_P60657	RPRD2	2.0286646	2.292212	2.03831	0.2635474	0.0096455	0.1365964	up
A_22_P00000261	ERICH2	0.3657169	0.4577842	0.5466886	0.0920672	0.1809716	0.1365194	up
A_33_P3284951	MCM5	3.117199	3.2840667	3.2233667	0.1668677	0.1061678	0.1365178	up
A_33_P3293753	KRTAP10-9	-1.799323	-1.604353	-1.721327	0.1949697	0.0779963	0.136483	up
A_23_P211196	LINC01547	-2.302567	-2.055619	-2.276646	0.2469478	0.0259209	0.1364343	up
A_23_P156156	ZCCHC9	2.1684074	2.3498836	2.2596855	0.1814761	0.0912781	0.1363771	up
A_23_P83278	CHMP5	4.356428	4.410341	4.575245	0.0539126	0.2188168	0.1363647	up
A_24_P276531	SNIP1	-0.116994	-0.090733	0.1294346	0.0262618	0.246429	0.1363454	up
A_23_P131683	MAPRE3	-1.449029	-1.27436	-1.351069	0.1746693	0.097961	0.1363151	up
A_23_P210608	ZNF217	2.3074484	2.5268693	2.360653	0.2194209	0.0532045	0.1363127	up
A_23_P96633	CT55	-2.208459	-1.896801	-2.247575	0.3116589	-0.039115	0.1362718	up

A_23_P9293	TJP2	4.842144	5.0267835	4.929942	0.1846395	0.0877981	0.1362188	up
A_24_P11436	TTC22	-1.885962	-1.734096	-1.765414	0.151866	0.1205478	0.1362069	up
A_33_P3209433	RELA	1.2773995	1.5415072	1.2856145	0.2641077	0.008215	0.1361613	up
A_33_P3290368	LINC00894	-2.059851	-2.124079	-1.723358	-0.064228	0.3364935	0.1361327	up
A_23_P12343	GSTM3	4.1306543	4.241795	4.2917376	0.1111407	0.1610832	0.136112	up
A_33_P3396746	KIF26B	-1.081497	-1.041828	-0.849009	0.0396686	0.2324882	0.1360784	up
A_23_P13524	TMEM126A	5.060069	5.162046	5.23024	0.1019769	0.1701708	0.1360738	up
A_21_P0014102	LOC643072	1.5545912	1.3550329	2.026206	-0.199558	0.4716148	0.1360283	up
A_33_P3225091	NUMBL	3.154603	3.2722955	3.3088121	0.1176925	0.1542091	0.1359508	up
A_33_P3316786	DACH1	-0.375584	-0.321572	-0.157829	0.0540118	0.2177553	0.1358836	up
A_24_P187948	BID	6.543124	6.597217	6.7607794	0.0540929	0.2176552	0.135874	up
A_23_P125771	HCFC1	-1.416985	-1.274044	-1.288332	0.142941	0.1286531	0.135797	up
A_24_P134266	BSG	4.1937857	4.2645593	4.394598	0.0707736	0.2008123	0.135793	up
A_23_P44617	COPG1	1.4180346	1.607748	1.4998507	0.1897135	0.0818162	0.1357648	up
A_22_P00003268	lnc-CALM1-5	-2.74826	-2.678679	-2.54644	0.0695806	0.2018194	0.1357	up
A_23_P207058	SOCS3	-1.187073	-0.889879	-1.212915	0.297194	-0.025842	0.1356761	up
A_22_P00009296	lnc-LRRC36-1	0.077137	0.2261224	0.1993942	0.1489854	0.1222572	0.1356213	up
A_24_P163405	TRIM27	1.0193391	1.2355037	1.0743299	0.2161646	0.0549908	0.1355777	up
A_23_P166	MOB3C	0.5469155	0.6674666	0.6975184	0.1205511	0.1506028	0.135577	up
A_21_P0003226	lnc-TRAK1-1	-1.496509	-1.18849	-1.533596	0.3080187	-0.037086	0.1354661	up
A_24_P400376	CHCHD2	5.875291	5.9676566	6.0538435	0.0923657	0.1785526	0.1354592	up
A_23_P394836	INF2	2.8720245	3.0926871	2.9222698	0.2206626	0.0502453	0.1354539	up
A_32_P122402	LOC441455	0.8385797	1.0847387	0.8633099	0.2461591	0.0247302	0.1354446	up
A_22_P00001341	MIR612	-1.326007	-1.168161	-1.212981	0.157846	0.1130257	0.1354358	up
A_23_P31399	PON2	5.5553875	5.379297	6.002345	-0.176091	0.4469576	0.1354334	up
A_33_P3297141	WDR48	1.4006519	1.4371448	1.6348314	0.0364928	0.2341795	0.1353362	up
A_24_P287780	CHMP1A	1.4662666	1.6060524	1.5971122	0.1397858	0.1308456	0.1353157	up
A_21_P0014785	VIM-AS1	-0.992692	-1.012193	-0.702562	-0.019502	0.2901297	0.135314	up
A_23_P60101	ZNF696	1.8886957	2.036635	2.0112972	0.1479392	0.1226015	0.1352704	up
A_33_P3228385	ATPIF1	6.4698896	6.691813	6.5183706	0.2219234	0.048481	0.1352022	up
A_23_P37778	FHOD1	1.4484906	1.4814291	1.6858964	0.0329385	0.2374058	0.1351721	up
A_23_P101655	ACTN4	5.723138	5.823443	5.8928614	0.1003051	0.1697235	0.1350143	up
A_22_P00012424	LOC101927071	-1.23038	-1.06084	-1.129898	0.1695399	0.100482	0.135011	up
A_24_P284324	TMEM104	2.8792582	2.8346076	3.1939268	-0.044651	0.3146687	0.1350091	up
A_23_P71241	SEC61G	5.570032	5.709375	5.700696	0.1393428	0.1306639	0.1350033	up
A_33_P3423270	TMEM40	-0.257188	-0.250577	0.0061064	0.0066109	0.2632947	0.1349528	up
A_33_P3357470	REPS2	-1.444786	-1.042708	-1.577208	0.4020782	-0.132422	0.1348281	up
A_33_P3349776	LSM14B	0.3489113	0.5734053	0.393991	0.224494	0.0450797	0.1347868	up
A_23_P414913	GLIPR2	0.6826491	0.413619	1.2212372	-0.26903	0.5385881	0.134779	up
A_23_P40315	DZANK1	-1.081614	-0.955719	-0.938163	0.1258946	0.1434503	0.1346724	up
A_33_P3359160	FAM189B	3.6473284	3.4853606	4.078558	-0.161968	0.4312296	0.1346309	up
A_24_P40907	PPAPDC2	0.9498138	0.7489142	1.4199028	-0.2009	0.470089	0.1345947	up
A_22_P00020679	lnc-MFSD6-1	2.629383	2.7639136	2.7640142	0.1345305	0.1346312	0.1345809	up
A_19_P00321284	LINC00630	-1.898308	-1.904752	-1.622943	-0.006444	0.2753649	0.1344607	up
A_21_P0000242	SNORD54	-2.262735	-1.981469	-2.275141	0.2812662	-0.012406	0.1344303	up
A_23_P341223	KLHL21	3.6434698	3.9428287	3.6129694	0.2993588	-0.0305	0.1344292	up
A_33_P3545065	SH3GL1P1	1.4266586	1.6064568	1.5156817	0.1797981	0.0890231	0.1344106	up
A_23_P395585	C3orf22	-2.214421	-2.042936	-2.117094	0.1714845	0.0973268	0.1344056	up
A_23_P381461	LRRC45	1.3396263	1.4342499	1.5136299	0.0946236	0.1740036	0.1343136	up
A_21_P0010713	lnc-PROK1-1	-1.199508	-1.127328	-1.003108	0.0721803	0.1964006	0.1342905	up
A_23_P215956	MYC	6.2486067	6.479469	6.28619	0.2308621	0.0375834	0.1342227	up
A_23_P252306	ID1	6.938176	7.1801744	6.9645977	0.2419982	0.0264215	0.1342099	up
A_23_P143474	ATP5O	8.22843	8.359852	8.365421	0.131422	0.1369915	0.1342068	up
A_33_P3375710	lnc-VAMP3-1	-1.500632	-1.718851	-1.014008	-0.218219	0.4866238	0.1342022	up
A_21_P0002639	lnc-TMEM18-6	-1.445475	-1.293671	-1.328917	0.1518035	0.1165576	0.1341806	up
A_23_P207399	NBR1	4.538312	4.910628	4.43412	0.3723159	-0.104192	0.1340621	up

A_23_P373079	PATL1	1.512857	1.5763907	1.717443	0.0635338	0.204586	0.1340599	up
A_33_P3265950	CBFA2T2	-0.785614	-0.583378	-0.719841	0.2022357	0.065773	0.1340044	up
A_24_P122524	WDR3	2.9065752	2.978458	3.102664	0.0718827	0.1960888	0.1339858	up
A_21_P0002779	TM4SF1-AS1	-2.650579	-2.473953	-2.559329	0.1766255	0.0912495	0.1339375	up
A_23_P15564	AMZ2	4.258277	4.4833665	4.300871	0.2250896	0.042594	0.1338418	up
A_23_P58353	HNRNPD	6.9320908	7.0630584	7.068802	0.1309676	0.1367111	0.1338394	up
A_23_P58647	CTNNA1	5.4404497	5.651788	5.496766	0.2113385	0.0563164	0.1338274	up
A_24_P641130	PRRC2A	1.3764081	1.56633	1.454073	0.1899219	0.0776649	0.1337934	up
A_33_P3320127	RAB8A	4.3997107	4.531094	4.5357656	0.1313834	0.136055	0.1337192	up
A_23_P76914	SIX1	2.2275677	2.2383924	2.4841404	0.0108247	0.2565727	0.1336987	up
A_23_P20606	NIPSNAP3A	2.4973364	2.6198316	2.6422043	0.1224952	0.1448679	0.1336815	up
A_33_P3384825	CTDNEP1	4.235053	4.2856355	4.451832	0.0505824	0.2167788	0.1336806	up
A_23_P348121	FOSL2	1.5102077	1.7408018	1.5468907	0.2305942	0.0366831	0.1336386	up
A_33_P3214293	PPP3R1	0.7038541	0.7398439	0.9350624	0.0359898	0.2312083	0.133599	up
A_22_P00005314	lnc-DNASE1-1	-1.774762	-1.240333	-2.04208	0.5344296	-0.267318	0.1335559	up
A_24_P606538	GGNBP1	-2.675714	-2.720853	-2.363474	-0.045139	0.3122404	0.1335508	up
A_24_P414371	PPP3CA	3.421115	3.567986	3.5413027	0.1468711	0.1201878	0.1335294	up
A_33_P3263664	KRTAP6-2	-2.744934	-2.648696	-2.574217	0.0962381	0.1707172	0.1334777	up
A_24_P239811	UBXN11	-0.580127	-0.25561	-0.637805	0.3245173	-0.057678	0.1334195	up
A_23_P319617	CHST7	1.1566119	1.2254024	1.3545108	0.0687904	0.1978989	0.1333446	up
A_23_P49009	LPCAT4	-0.006017	-0.267277	0.5219178	-0.26126	0.5279346	0.1333373	up
A_23_P105313	EIF2B1	3.7267952	3.8508391	3.869134	0.1240439	0.1423388	0.1331913	up
A_33_P3240258	RPN2	6.45452	6.598495	6.5767536	0.1439748	0.1222334	0.1331041	up
A_23_P201368	CTBS	1.767415	1.858748	1.9421606	0.0913329	0.1747456	0.1330392	up
A_24_P285032	IDS	-0.56989	-0.279987	-0.593859	0.2899032	-0.023969	0.132967	up
A_23_P218892	EIF4G1	4.714608	4.835276	4.859872	0.1206679	0.1452637	0.1329658	up
A_23_P64689	PAN2	-1.095163	-0.760128	-1.164289	0.3350353	-0.069126	0.1329548	up
A_23_P402787	UBALD1	1.868289	2.0673804	1.9350586	0.1990914	0.0667696	0.1329305	up
A_21_P0000090	TSFM	2.262765	2.3325868	2.4586706	0.0698218	0.1959057	0.1328638	up
A_22_P00001364	lnc-AP001631.10.1-1	-1.886948	-1.776906	-1.731461	0.1100426	0.1554875	0.1327651	up
A_22_P00008544	lnc-KCNMB1-3	-3.431564	-3.231457	-3.366287	0.2001069	0.0652769	0.1326919	up
A_32_P23795	XLOC_l2_010916	3.0633888	3.3622537	3.029704	0.2988648	-0.033685	0.1325901	up
A_23_P406105	GCN1L1	6.630069	6.720224	6.805064	0.0901551	0.1749954	0.1325753	up
A_19_P00320351	LOC642852	-1.048855	-0.745006	-1.087687	0.3038497	-0.038831	0.1325092	up
A_33_P3326046	RNF167	2.351982	2.5156293	2.4531355	0.1636472	0.1011534	0.1324003	up
A_23_P250607	PLS3	3.910266	4.135485	3.9498262	0.2252193	0.0395603	0.1323898	up
A_24_P395621	RUNDC1	0.961762	1.0591884	1.129025	0.0974264	0.167263	0.1323447	up
A_33_P3271480	LOC90784	2.1779108	2.2830405	2.3373604	0.1051297	0.1594496	0.1322897	up
A_21_P0012549	RPL32P3	-3.060248	-2.994252	-2.86172	0.0659959	0.1985283	0.1322621	up
A_23_P87664	SART3	2.2305908	2.306398	2.4191952	0.0758071	0.1886044	0.1322057	up
A_23_P92202	GMPPB	0.2820602	0.2216106	0.6069021	-0.06045	0.324842	0.1321962	up
A_23_P81048	STIM2	0.0563984	0.0863009	0.2906704	0.0299025	0.234272	0.1320872	up
A_33_P3370060	ARHGAP23	-1.3357	-1.114315	-1.292925	0.2213855	0.0427747	0.1320801	up
A_22_P00010155	MRPL46	3.8222342	4.030597	3.8779907	0.2083631	0.0557566	0.1320598	up
A_23_P38190	ORMDL3	0.3416214	0.4104972	0.5368428	0.0688758	0.1952214	0.1320486	up
A_33_P6803961	NOP14-AS1	-0.865256	-0.640457	-0.826045	0.2247996	0.0392113	0.1320055	up
A_33_P3305482	EIF2B3	3.1105728	3.072969	3.4120083	-0.037604	0.3014355	0.1319158	up
A_32_P180971	LOC728323	3.537137	3.7426085	3.59507	0.2054715	0.0579329	0.1317022	up
A_33_P3379341	LOC391322	-1.567181	-1.411549	-1.459448	0.155632	0.1077328	0.1316824	up
A_22_P00016784	LOC101929099	-0.977396	-0.865108	-0.826323	0.1122875	0.151073	0.1316803	up
A_23_P93258	HIST1H3B	5.6967173	5.7401023	5.916686	0.043385	0.2199688	0.1316769	up
A_19_P00320664	FLJ46906	-0.109409	-0.067762	0.1121922	0.041647	0.2216015	0.1316242	up
A_24_P926367	THRAP3	5.782529	5.936147	5.892104	0.1536183	0.1095753	0.1315968	up
A_23_P40880	CMTM8	1.9257154	1.9101129	2.2044191	-0.015603	0.2787037	0.1315506	up
A_23_P93823	RFC2	3.1819744	3.2367425	3.3901882	0.0547681	0.2082138	0.1314909	up

A_32_P75299	TOMM5	7.3211527	7.2715135	7.633581	-0.049639	0.3124285	0.1313946	up
A_32_P64038	LOC400590	3.3550396	3.471438	3.5012817	0.1163983	0.1462421	0.1313202	up
A_33_P3362321	FHL2	2.357563	2.568399	2.4092903	0.2108359	0.0517273	0.1312816	up
A_21_P0001244	LOC101927851	0.2013083	0.2993069	0.3658624	0.0979986	0.1645541	0.1312764	up
A_23_P85903	TLR5	-1.4053	-1.319253	-1.229004	0.0860467	0.1762958	0.1311712	up
A_22_P00003291	NDST2	-1.086447	-1.05949	-0.851071	0.0269575	0.2353759	0.1311667	up
A_21_P0011500	LOC100288069	1.1220946	1.3114853	1.194921	0.1893907	0.0728264	0.1311085	up
A_22_P00002552	MGC57346-CRHR1	1.3068161	1.412118	1.4636116	0.1053019	0.1567955	0.1310487	up
A_24_P139901	GYPC	-2.135329	-1.951905	-2.05667	0.183424	0.0786595	0.1310418	up
A_24_P942068	TANC2	1.0867157	1.2141914	1.2211866	0.1274757	0.1344709	0.1309733	up
A_21_P0013292	EIF4H	6.98166	7.1852546	7.039942	0.2035947	0.0582819	0.1309383	up
A_23_P402751	COX2	8.847931	9.140579	8.817105	0.2926483	-0.030826	0.1309114	up
A_22_P00014840	lnc-SLC47A2-3	-2.482539	-2.363085	-2.340374	0.1194539	0.1421652	0.1308095	up
A_23_P425516	TSPAN17	1.025701	1.1580124	1.1549664	0.1323113	0.1292653	0.1307883	up
A_33_P7380829	TSR1	9.791974	10.010622	9.834747	0.218648	0.0427732	0.1307106	up
A_21_P0000181	CAMTA1	1.2633481	1.3602934	1.4278188	0.0969453	0.1644707	0.130708	up
A_24_P182539	ATG4C	-0.722871	-0.58104	-0.60339	0.1418304	0.1194811	0.1306558	up
A_23_P93690	MCM7	7.6996	7.767099	7.8933935	0.0674987	0.1937933	0.130646	up
A_23_P213336	FGF1	-1.746032	-1.397721	-1.83308	0.3483105	-0.087048	0.1306312	up
A_33_P3294297	RNF126	3.8967228	3.912417	4.1421566	0.0156941	0.2454338	0.130564	up
A_23_P118435	SUMO2	6.429598	6.542112	6.5781984	0.112514	0.1486006	0.1305573	up
A_23_P159110	SLC35E3	0.2748861	0.501471	0.3091207	0.2265849	0.0342345	0.1304097	up
A_23_P156748	ANKS1A	2.9447727	2.9321027	3.218254	-0.01267	0.2734814	0.1304057	up
A_23_P120414	YWHAB	7.020358	7.117245	7.1842194	0.0968871	0.1638613	0.1303742	up
A_22_P00012802	lnc-RANBP9-1	-1.195895	-1.185762	-0.945293	0.0101333	0.2506023	0.1303678	up
A_32_P149251	DNAJC18	-1.259314	-0.836381	-1.421514	0.4229336	-0.1622	0.130367	up
A_23_P59738	MYL7	-2.618964	-2.36473	-2.612522	0.2542336	0.0064418	0.1303377	up
A_33_P3378835	SLC9A3R1	6.236949	6.214804	6.5197535	-0.022145	0.2828045	0.1303299	up
A_32_P389118	HEATR5B	-0.476597	-0.260461	-0.43217	0.216136	0.0444264	0.1302812	up
A_33_P3356910	TCEAL6	0.3362784	0.2658339	0.6672239	-0.070445	0.3309455	0.1302505	up
A_33_P3378785	FAM99A	0.4143043	0.6135998	0.4753547	0.1992955	0.0610504	0.130173	up
A_21_P0012244	XLOC_l2_009285	-1.216693	-1.064837	-1.108241	0.151856	0.1084523	0.1301541	up
A_33_P3272558	NCAPH2	6.0922184	6.2251925	6.219368	0.1329742	0.1271496	0.1300619	up
A_21_P0000360	SNORA77	0.487195	0.470861	0.7635641	-0.016334	0.2763691	0.1300175	up
A_23_P90369	TMEM259	5.422598	5.508349	5.5968695	0.0857511	0.1742716	0.1300113	up
A_23_P12079	KCNC4	-2.634501	-2.676594	-2.332477	-0.042093	0.3020239	0.1299656	up
A_33_P3381328	FAM201A	0.1021795	0.591857	-0.127582	0.4896774	-0.229762	0.1299579	up
A_23_P70897	ZCWPW1	2.4787436	2.7992349	2.4181156	0.3204913	-0.060628	0.1299317	up
A_24_P239731	B4GALT5	3.9239597	4.0391555	4.068219	0.1151958	0.1442595	0.1297276	up
A_24_P229164	HIP1R	0.6987124	0.8026171	0.8542318	0.1039047	0.1555195	0.1297121	up
A_21_P0005567	lnc-LAT2-1	-1.073295	-0.756212	-1.130996	0.3170824	-0.057701	0.1296906	up
A_33_P3366053	ADPRH	-0.13511	-0.058956	0.0481153	0.0761547	0.1832256	0.1296902	up
A_24_P830667	RPL21	6.938614	7.1105895	7.025957	0.1719756	0.0873432	0.1296594	up
A_33_P3219596	LINC00925	-1.61592	-1.404653	-1.567875	0.2112665	0.0480447	0.1296556	up
A_23_P126623	PGD	2.9303408	2.8479486	3.2719908	-0.082392	0.34165	0.1296289	up
A_33_P3216438	SPATA21	-0.914722	-0.601804	-0.968405	0.3129182	-0.053682	0.1296179	up
A_33_P3340007	CTAGE10P	-2.295411	-1.933681	-2.397965	0.3617306	-0.102553	0.1295886	up
A_33_P3360611	MPP2	0.8098269	0.8690291	1.0097799	0.0592022	0.1999531	0.1295776	up
A_22_P00012332	LOC101928812	-2.537121	-2.578696	-2.236479	-0.041575	0.3006415	0.1295332	up
A_21_P0011702	XLOC_l2_006789	0.5885639	0.6364913	0.7996826	0.0479274	0.2111187	0.129523	up
A_22_P00021084	lnc-KANSL3-1	-0.777249	-0.544934	-0.750624	0.2323155	0.0266252	0.1294703	up
A_24_P80138	PDCL3	3.862132	3.8840766	4.099118	0.0219445	0.2369862	0.1294653	up
A_24_P103686	AGPAT6	2.045023	2.2250247	2.1238213	0.1800017	0.0787983	0.1294	up
A_23_P502224	CYB5R3	8.006425	7.838577	8.432994	-0.167848	0.426569	0.1293604	up
A_33_P3228285	ANKRD13D	3.774002	3.8363872	3.9702682	0.0623851	0.1962662	0.1293256	up
A_21_P0006969	SFTA1P	-0.762888	-0.498846	-0.768327	0.2640419	-0.005439	0.1293013	up

A_33_P3258330	GDPD5	-1.546331	-1.343661	-1.490425	0.2026701	0.0559063	0.1292882	up
A_21_P0007311	lnc-C11orf89-1	1.7684231	1.9309735	1.864419	0.1625505	0.0959959	0.1292732	up
A_23_P76488	EMP1	7.903632	7.9174743	8.14832	0.0138421	0.244688	0.1292651	up
A_23_P65031	DYNLL1	8.006589	8.053917	8.217749	0.047328	0.2111597	0.1292438	up
A_23_P44112	LAT	0.9259272	1.0936646	1.0165434	0.1677375	0.0906162	0.1291769	up
A_23_P503182	ABR	3.8543901	3.8704267	4.0965805	0.0160365	0.2421904	0.1291134	up
A_24_P119685	OBSCN	-0.542784	-0.070431	-0.756961	0.472353	-0.214178	0.1290877	up
A_23_P23206	MAD2L2	1.9016466	1.9344802	2.126955	0.0328336	0.2253084	0.129071	up
A_24_P343233	HLA-DRB1	-0.348974	-0.172036	-0.267787	0.1769385	0.0811877	0.1290631	up
A_23_P409541	POLR1D	1.8535681	1.9504447	2.0146523	0.0968766	0.1610842	0.1289804	up
A_23_P46455	DNAJC8	1.4356008	1.5963464	1.5327249	0.1607456	0.0971241	0.1289349	up
A_33_P3284919	SEMA6C	1.1358185	1.3197517	1.2097454	0.1839333	0.0739269	0.1289301	up
A_23_P84448	TUBA4A	4.5085297	4.415171	4.8596067	-0.093359	0.3510771	0.1288593	up
A_33_P3248108	ZNF618	-1.696633	-1.73916	-1.396537	-0.042527	0.3000956	0.1287844	up
A_33_P3235204	ELMOD3	-1.377521	-1.217734	-1.279752	0.1597867	0.0977688	0.1287777	up
A_23_P258221	ABCC5	3.923214	4.1255183	3.978383	0.2023044	0.0551691	0.1287367	up
A_22_P00003255	lnc-CALCOCO2-5	-2.851835	-2.957895	-2.488372	-0.10606	0.3634627	0.1287013	up
A_24_P406301	NDUFB2	8.38665	8.747835	8.282664	0.3611851	-0.103986	0.1285996	up
A_23_P19322	SAYSD1	1.1312041	1.257462	1.2621188	0.1262579	0.1309147	0.1285863	up
A_33_P3300893	MADD	0.8755512	0.9108605	1.0973344	0.0353093	0.2217832	0.1285462	up
A_23_P403745	MTSS1L	0.7747197	0.9756589	0.830853	0.2009392	0.0561333	0.1285362	up
A_33_P3311205	PRPF40B	-2.852569	-2.627617	-2.820573	0.2249522	0.0319958	0.128474	up
A_24_P932736	HMBOX1	-2.495487	-2.156385	-2.577782	0.3391018	-0.082295	0.1284034	up
A_24_P84419	VAV2	2.819419	2.912191	2.9833136	0.092772	0.1638947	0.1283333	up
A_33_P3226775	MUC20	0.68086	1.0667305	0.5516338	0.3858705	-0.129226	0.1283221	up
A_33_P3408221	SEC14L1P1	-0.200948	-0.023057	-0.122297	0.1778908	0.0786514	0.1282711	up
A_23_P107661	PPP2R1A	3.825594	4.0271964	3.8804674	0.2016025	0.0548735	0.128238	up
A_22_P00023960	lnc-BATF3-3	-2.765543	-2.363271	-2.91137	0.402272	-0.145827	0.1282226	up
A_23_P381449	SP2	0.8543506	1.132741	0.832386	0.2783904	-0.021965	0.1282129	up
A_23_P79962	MKKS	2.9971933	3.32871	2.9218426	0.3315167	-0.075351	0.128083	up
A_22_P00002482	GDPGP1	0.0594006	0.1242886	0.2506642	0.064888	0.1912637	0.1280758	up
A_21_P0001433	lnc-RNF187-1	-2.720128	-2.710249	-2.473867	0.0098786	0.2462611	0.1280699	up
A_33_P3253440	PQLC3	-1.599972	-1.388612	-1.555257	0.2113595	0.0447149	0.1280372	up
A_32_P319200	GGTLC2	-1.327618	-1.153022	-1.246275	0.1745963	0.0813437	0.12797	up
A_33_P3312544	GABRB1	-2.230463	-2.071508	-2.133485	0.1589556	0.0969782	0.1279669	up
A_24_P365506	FERMT1	0.9839039	0.9510779	1.2726469	-0.032826	0.288743	0.1279585	up
A_23_P143120	ADAM17	1.6208754	1.9468722	1.5507731	0.3259969	-0.070102	0.1279473	up
A_33_P3217559	DLK1	-1.623633	-1.330252	-1.661445	0.2933807	-0.037812	0.1277845	up
A_33_P3303810	LAD1	7.6185246	7.538154	7.9544163	-0.08037	0.3358917	0.1277606	up
A_24_P245246	PIP4K2B	5.8978186	6.0442224	6.006878	0.1464038	0.1090593	0.1277316	up
A_32_P171328	UBE2S	6.551759	6.5444827	6.8144407	-0.007276	0.262682	0.127703	up
A_23_P201636	LAMC2	4.8889694	5.145554	4.8877325	0.2565846	-0.001237	0.1276739	up
A_23_P70168	TARS	5.6584454	5.8843303	5.687789	0.2258849	0.0293436	0.1276143	up
A_19_P00322599	KIAA1671	0.012053	0.2715139	0.0077424	0.2594609	-0.004311	0.1275752	up
A_33_P3302255	ITM2B	5.180784	5.3326526	5.2840166	0.1518683	0.1032324	0.1275504	up
A_23_P128323	SCNN1A	2.6152916	2.5223498	2.9632835	-0.092942	0.3479919	0.1275251	up
A_33_P3348614	TBL1XR1	2.600213	2.7263083	2.7291574	0.1260953	0.1289444	0.1275199	up
A_23_P252700	UNC50	3.3506632	3.509272	3.4470825	0.1586089	0.0964193	0.1275141	up
A_23_P140050	SUPT20H	0.2111349	0.4821429	0.1950369	0.271008	-0.016098	0.127455	up
A_23_P351275	UPP1	0.6543794	1.9098096	-0.346436	1.2554302	-1.000815	0.1273074	up
A_23_P202117	PCGF5	1.6322746	1.6165771	1.9025745	-0.015697	0.2702999	0.1273012	up
A_24_P239606	GADD45B	3.646926	4.019379	3.5290718	0.3724532	-0.117854	0.1272995	up
A_22_P00006293	lnc-FAM83F-1	7.092822	7.0283027	7.4119396	-0.064519	0.3191176	0.1272991	up
A_21_P0011266	LINC00639	-1.699214	-1.387608	-1.756271	0.3116064	-0.057057	0.1272747	up
A_33_P3344291	LOC100130698	-1.521052	-1.304658	-1.482974	0.2163944	0.0380778	0.1272361	up
A_23_P29204	MTFP1	3.1786242	3.225819	3.3857565	0.047195	0.2071323	0.1271636	up

A_21_P0009118	lnc-C16orf55-1	-2.12573	-2.044207	-1.952993	0.0815229	0.1727371	0.12713	up
A_21_P0013587	XLOC_l2_014835	0.8901057	1.0769587	0.957489	0.1868529	0.0673833	0.1271181	up
A_23_P171223	CXorf56	-0.33293	-0.283635	-0.128016	0.049295	0.2049146	0.1271048	up
A_23_P98580	FADS2	-0.178837	-0.258334	0.1548505	-0.079497	0.3336873	0.1270952	up
A_19_P00807614	lnc-WDR7-8	-2.967429	-2.588426	-3.092625	0.3790035	-0.125196	0.1269039	up
A_23_P91114	PREB	3.85182	3.8534217	4.1039877	0.0016017	0.2521677	0.1268847	up
A_33_P3304212	PLEKHG3	5.336273	5.4055305	5.5207415	0.0692573	0.1844683	0.1268628	up
A_24_P261005	SMIM19	2.5534897	2.8199534	2.5405073	0.2664638	-0.012982	0.1267407	up
A_23_P82929	NOV	4.6517696	4.8133216	4.743675	0.161552	0.0919056	0.1267288	up
A_33_P3261505	EPG5	-1.217805	-1.040247	-1.141974	0.1775575	0.0758314	0.1266944	up
A_23_P10815	PUM1	4.06417	4.2276015	4.153887	0.1634316	0.0897169	0.1265743	up
A_19_P00316202	lnc-TP53INP1-1	-0.00081	0.1400557	0.1114492	0.1408653	0.1122589	0.1265621	up
A_21_P0000717	METTL23	3.7895403	3.9552903	3.8768759	0.16575	0.0873356	0.1265428	up
A_33_P3399492	SDHA	3.0109177	3.1506228	3.1242876	0.1397052	0.1133699	0.1265376	up
A_23_P170186	OPLAH	3.7393894	3.798573	3.9331617	0.0591836	0.1937723	0.126478	up
A_32_P6015	MNX1	2.7556229	2.9691043	2.7950506	0.2134814	0.0394278	0.1264546	up
A_23_P35791	RPS6KA4	3.583912	3.629273	3.7914505	0.045361	0.2075386	0.1264498	up
A_23_P24716	TMEM132A	5.5153103	5.6592374	5.624216	0.1439271	0.1089058	0.1264164	up
A_33_P3303865	FAM120C	0.8087945	0.9954653	0.8748741	0.1866708	0.0660796	0.1263752	up
A_33_P3226665	ZNF419	-0.033074	0.4053569	-0.218828	0.4384313	-0.185754	0.1263387	up
A_21_P0000268	SNORA69	-1.870399	-1.856572	-1.63161	0.0138268	0.2387886	0.1263077	up
A_33_P3285156	BAIAP2-AS1	2.6581764	2.7348828	2.8338556	0.0767064	0.1756792	0.1261928	up
A_21_P0007218	BCAS4	-1.594525	-1.542371	-1.394457	0.0521541	0.200068	0.126111	up
A_33_P3414482	GOT2	7.882328	7.87649	8.140318	-0.005838	0.2579899	0.126076	up
A_33_P3327539	TBC1D9	-0.391004	-0.142092	-0.387802	0.2489119	0.003202	0.1260569	up
A_23_P137689	OLFML2B	-1.978648	-1.956916	-1.748301	0.0217319	0.2303472	0.1260395	up
A_21_P0013318	CRCP	0.7614036	0.7259927	1.0485392	-0.035411	0.2871356	0.1258624	up
A_32_P34387	SPATA7	-1.5745	-1.373357	-1.524074	0.2011428	0.0504265	0.1257846	up
A_23_P255805	ZNF7	2.769001	2.9154124	2.8741484	0.1464114	0.1051474	0.1257794	up
A_33_P3376478	CYP17A1	-0.57346	-0.450367	-0.445002	0.1230922	0.128458	0.1257751	up
A_33_P3211473	XPO7	4.8094397	4.8663726	5.0039177	0.0569329	0.194478	0.1257055	up
A_23_P250455	HDAC6	1.5518236	1.7054453	1.6496105	0.1536217	0.0977869	0.1257043	up
A_33_P3234222	TSPO2	-0.77801	-0.542974	-0.761989	0.2350359	0.0160213	0.1255286	up
A_23_P112412	TEX10	4.45741	4.5094066	4.6564617	0.0519967	0.1990519	0.1255243	up
A_33_P3235098	NUTM2D	-0.864879	-0.817518	-0.661238	0.0473609	0.2036414	0.1255012	up
A_33_P3364508	C7orf43	0.4178934	0.5248342	0.5617375	0.1069408	0.1438441	0.1253924	up
A_21_P0010798	LYPD8	-1.607646	-1.298674	-1.665936	0.3089719	-0.05829	0.1253412	up
A_33_P3242783	LOC440082	-2.348647	-2.077019	-2.369674	0.2716279	-0.021028	0.1253	up
A_32_P516342	ANKRD33	-0.507961	-0.44206	-0.323369	0.0659008	0.1845923	0.1252465	up
A_23_P31477	NUDCD3	1.9250488	1.8858728	2.214614	-0.039176	0.2895651	0.1251946	up
A_23_P200874	CEP85	0.1229897	0.1692853	0.3269587	0.0462956	0.203969	0.1251323	up
A_23_P431853	ND2	6.963422	7.258044	6.918936	0.2946219	-0.044486	0.1250679	up
A_22_P00012676	LOC101929542	-2.603501	-2.356654	-2.600354	0.2468479	0.0031478	0.1249979	up
A_23_P94932	MMADHC	5.781643	5.9823403	5.830858	0.2006974	0.0492153	0.1249564	up
A_23_P142974	ARHGAP25	0.3695178	0.6572461	0.3314591	0.2877283	-0.038059	0.1248348	up
A_19_P00806320	ARPC2	3.640295	3.6666856	3.8634977	0.0263906	0.2232027	0.1247966	up
A_23_P100868	MYO19	3.3833246	3.2780128	3.738227	-0.105312	0.3549023	0.1247952	up
A_33_P3394021	KLHL12	-2.183282	-2.028217	-2.088923	0.1550651	0.0943589	0.124712	up
A_24_P43588	MAD2L1BP	3.68534	3.774983	3.8450232	0.089643	0.1596832	0.1246631	up
A_21_P0000247	SNORD18C	-0.12285	0.3264604	-0.322938	0.4493103	-0.200088	0.1246111	up
A_24_P58177	C14orf159	-1.817165	-1.768921	-1.61619	0.048244	0.2009745	0.1246092	up
A_33_P3300600	OR7G1	-1.980781	-1.633721	-2.078668	0.3470597	-0.097887	0.1245863	up
A_33_P3292387	LRRIQ3	0.4182358	0.3595676	0.7257905	-0.058668	0.3075547	0.1244433	up
A_33_P3213551	SHARPIN	5.0895634	5.104508	5.323414	0.0149446	0.2338505	0.1243975	up
A_33_P3220182	TRIM15	-2.000577	-2.112779	-1.639667	-0.112202	0.3609104	0.1243542	up
A_23_P7325	BST1	-3.30029	-2.833738	-3.518384	0.466552	-0.218094	0.1242291	up

A_24_P318073	RPUSD4	1.9285812	2.2437692	1.8618021	0.3151879	-0.066779	0.1242044	up
A_21_P0010094	lnc-ARFGEF2-2	-1.314772	-1.072236	-1.309077	0.2425365	0.0056953	0.1241159	up
A_21_P0000775	ENO1-AS1	-2.598257	-2.49465	-2.453851	0.1036072	0.1444056	0.1240064	up
A_32_P41553	TSACC	0.1288381	0.3012815	0.2042904	0.1724434	0.0754523	0.1239479	up
A_22_P00002938	LOC648987	-1.467671	-1.301752	-1.386045	0.1659193	0.0816259	0.1237726	up
A_33_P3229246	HIST2H2BE	-0.935209	-0.69091	-0.932052	0.2442989	0.0031571	0.123728	up
A_21_P0011258	XLOC_l2_004315	-1.968567	-1.87866	-1.811302	0.0899072	0.1572657	0.1235864	up
A_23_P357929	SLC35D1	-0.872422	-0.979864	-0.517879	-0.107442	0.3545427	0.1235502	up
A_21_P0009080	lnc-COX4NB-1	-2.483106	-2.16999	-2.549208	0.3131158	-0.066102	0.1235068	up
A_33_P3290338	PARP1	4.5829973	4.594074	4.818902	0.0110765	0.2359047	0.1234906	up
A_21_P0009536	lnc-SS18-1	-1.664195	-1.425088	-1.656408	0.2391071	0.0077872	0.1234472	up
A_23_P145777	NDUFA4	8.655585	8.631622	8.926385	-0.023963	0.2707996	0.1234183	up
A_24_P263956	ATXN7L3B	2.87642	2.8373847	3.1621761	-0.039035	0.2857561	0.1233604	up
A_21_P0007747	LOC643770	-0.893709	-0.837299	-0.703556	0.0564103	0.1901536	0.123282	up
A_19_P00320555	LOC101929709	-2.109901	-2.059685	-1.91362	0.0502162	0.196281	0.1232486	up
A_32_P30831	RAD51-AS1	-0.418417	-0.142929	-0.447607	0.2754879	-0.029191	0.1231487	up
A_23_P371824	TUFT1	5.6483364	5.8678784	5.674863	0.219542	0.0265265	0.1230342	up
A_23_P213840	ATP6V0E1	5.090539	5.269072	5.1580496	0.1785331	0.0675106	0.1230218	up
A_23_P105900	BTF3P11	6.411824	6.646479	6.4232063	0.2346549	0.0113821	0.1230185	up
A_23_P64129	HTATIP2	3.9148426	4.2429023	3.832736	0.3280597	-0.082107	0.1229765	up
A_21_P0000161	USB1	0.3110933	-0.072694	0.940805	-0.383788	0.6297116	0.122962	up
A_33_P3361398	C19orf26	-1.565319	-1.366147	-1.518694	0.199172	0.0466251	0.1228986	up
A_23_P103897	SPATA1	-3.14544	-3.057589	-2.987575	0.0878511	0.1578653	0.1228582	up
A_33_P3422289	lnc-TMED5-1	0.7872725	0.8448939	0.9752755	0.0576215	0.1880031	0.1228123	up
A_32_P189790	DKFZp779M0652	-3.096164	-2.911027	-3.035843	0.1851368	0.0603201	0.1227285	up
A_32_P505133	C19orf47	-0.737644	-0.634326	-0.595603	0.1033187	0.1420407	0.1226797	up
A_23_P376627	D2HGDH	3.332756	3.338366	3.5724049	0.00561	0.2396488	0.1226294	up
A_19_P00810894	FLJ40288	-2.357619	-2.222131	-2.247892	0.135488	0.1097264	0.1226072	up
A_33_P3374117	LOC101927507	-2.46623	-2.485571	-2.201713	-0.019342	0.2645171	0.1225877	up
A_23_P50504	FTL	6.162508	6.7506747	5.819416	0.5881667	-0.343092	0.1225374	up
A_33_P3350823	ZNF639	0.5306869	0.5378795	0.7685375	0.0071926	0.2378507	0.1225216	up
A_24_P305467	GATAD2A	1.8007021	1.6333494	2.2130785	-0.167353	0.4123764	0.1225119	up
A_22_P00006607	lnc-FOXA1-2	0.5687332	0.6657829	0.7167015	0.0970497	0.1479683	0.122509	up
A_19_P00322109	LOC401320	-2.105545	-1.879612	-2.086535	0.2259326	0.0190096	0.1224711	up
A_22_P00014818	LOC101927809	-1.848072	-1.757083	-1.694132	0.0909891	0.1539397	0.1224644	up
A_23_P39602	NCOA1	1.5543523	1.7576532	1.5959711	0.203301	0.0416188	0.1224599	up
A_24_P246351	FAM71E1	0.6377425	0.3711929	1.1490355	-0.26655	0.5112929	0.1223717	up
A_33_P3213892	PRDM15	-3.041341	-2.64682	-3.191137	0.394521	-0.149796	0.1223626	up
A_33_P3356701	ZDHHC12	5.2403927	5.2388663	5.4860907	-0.001526	0.245698	0.1220858	up
A_22_P00015685	lnc-SYNC-2	-2.950652	-2.923245	-2.733922	0.0274072	0.2167296	0.1220684	up
A_21_P0001689	MGC27382	-1.103915	-1.087009	-0.876696	0.0169058	0.2272191	0.1220624	up
A_21_P0000894	IQCH-AS1	-1.930401	-2.147087	-1.469621	-0.216685	0.4607801	0.1220474	up
A_23_P154875	BACE2	4.1051216	4.2148213	4.2394857	0.1096997	0.1343641	0.1220319	up
A_23_P370434	C1QBP	7.075595	7.1910834	7.204115	0.1154885	0.12852	0.1220043	up
A_23_P86900	B4GAT1	1.5132298	1.7739401	1.4965053	0.2607102	-0.016725	0.1219928	up
A_33_P3213752	CDK5RAP1	4.633752	4.6631703	4.847868	0.0294185	0.2141161	0.1217673	up
A_33_P3360392	LINC00092	-1.516641	-1.339374	-1.450474	0.1772666	0.0661664	0.1217165	up
A_22_P00013451	lnc-RP11-295K3.1.1-1	-0.988308	-0.919954	-0.81325	0.0683537	0.1750584	0.121706	up
A_32_P231617	TM4SF1	3.4718323	3.62468	3.5623589	0.1528478	0.0905266	0.1216872	up
A_23_P129458	SDR42E1	2.3941202	2.4842505	2.5472736	0.0901303	0.1531534	0.1216419	up
A_23_P202219	CALHM2	2.309061	2.4096007	2.4517221	0.1005397	0.1426611	0.1216004	up
A_33_P3245412	TRMT10B	-0.257737	-0.346194	0.0738635	-0.088458	0.3316002	0.1215713	up
A_23_P24796	FAM160A2	3.3105412	3.3652778	3.4988804	0.0547366	0.1883392	0.1215379	up
A_33_P3219785	SOWAHC	6.7906733	6.9669614	6.8574085	0.1762881	0.0667353	0.1215117	up
A_24_P56689	ZNF205	0.8226652	1.037859	0.850358	0.2151938	0.0276928	0.1214433	up

A_23_P141847	UBE2M	3.3347673	3.282063	3.6303005	-0.052704	0.2955332	0.1214144	up
A_33_P3233165	CFAP36	-2.190818	-1.346202	-2.792608	0.8446164	-0.601789	0.1214136	up
A_23_P108657	WDSUB1	0.9004741	1.130332	0.9133787	0.2298579	0.0129046	0.1213813	up
A_23_P412214	RAP1GAP2	-1.427092	-1.153387	-1.458096	0.273705	-0.031004	0.1213503	up
A_22_P00004109	lnc-CLCN3-1	-2.200759	-2.047888	-2.111004	0.1528711	0.0897555	0.1213133	up
A_24_P178300	PRR21	-2.099532	-1.932665	-2.023877	0.1668668	0.075655	0.1212609	up
A_21_P0013581	LOC642236	1.8207035	1.90486	1.9790134	0.0841565	0.1583099	0.1212332	up
A_24_P282416	ABL1	-1.593737	-1.38623	-1.558828	0.2075067	0.0349083	0.1212075	up
A_23_P155351	BTD	-3.083169	-3.224633	-2.699538	-0.141465	0.3836308	0.121083	up
A_22_P00008393	EHD4-AS1	0.3882561	0.5702415	0.4484124	0.1819854	0.0601563	0.1210709	up
A_33_P3250595	ITSN1	-1.175036	-1.122628	-0.985317	0.0524082	0.1897192	0.1210637	up
A_23_P118607	PTGES3L-AARSD1	2.7754698	2.901773	2.891241	0.1263032	0.1157713	0.1210372	up
A_32_P197489	KLF13	5.6682568	5.838406	5.7401323	0.1701493	0.0718756	0.1210125	up
A_33_P3368453	MICA	4.38713	4.364614	4.651659	-0.022516	0.2645292	0.1210067	up
A_23_P87635	RITA1	4.0094233	3.9419246	4.3188744	-0.067499	0.3094511	0.1209762	up
A_22_P00000650	lnc-ACSS3-2	-0.55226	-0.433983	-0.428833	0.1182766	0.1234264	0.1208515	up
A_32_P780862	BMP8B	-1.161555	-1.15871	-0.922745	0.0028453	0.2388101	0.1208277	up
A_23_P85053	ZRSR2	1.7520161	2.0096412	1.7360082	0.2576251	-0.016008	0.1208086	up
A_33_P3323699	NPPA	-0.539746	-0.219619	-0.618265	0.320127	-0.078519	0.1208038	up
A_23_P258272	SDCCAG3	4.0698433	4.14119	4.240019	0.0713468	0.1701756	0.1207612	up
A_24_P945228	CYP4V2	-2.784094	-2.619068	-2.707657	0.165026	0.076437	0.1207315	up
A_23_P209449	FZD7	3.1991663	3.473681	3.1660967	0.2745147	-0.03307	0.1207225	up
A_23_P258190	AKR1B1	3.9654207	4.0596576	4.112462	0.0942369	0.1470413	0.1206391	up
A_23_P5976	ZSWIM1	-0.73478	-0.673283	-0.555007	0.0614972	0.1797724	0.1206348	up
A_23_P141520	C17orf49	2.6921396	2.2036138	3.4218426	-0.488526	0.729703	0.1205885	up
A_33_P3319113	PDPR	-1.588963	-1.460133	-1.476699	0.12883	0.1122637	0.1205468	up
A_24_P391604	KRTAP6-3	0.2818575	0.619133	0.1855373	0.3372755	-0.09632	0.1204777	up
A_32_P116660	RBM43	-0.746971	-0.321381	-0.931642	0.42559	-0.184671	0.1204593	up
A_24_P148796	MST1	-0.544775	-0.233611	-0.615124	0.3111644	-0.070349	0.1204078	up
A_21_P0009795	lnc-EPN1-1	-0.204208	-0.063689	-0.10406	0.1405191	0.1001487	0.1203339	up
A_21_P0009402	lnc-AC027763.2.1-1	-1.79801	-1.564852	-1.79056	0.2331576	0.0074501	0.1203039	up
A_23_P130418	NDUFV2	6.7632256	6.938172	6.8288813	0.1749463	0.0656557	0.120301	up
A_23_P80040	PROCR	4.643367	4.474296	5.053031	-0.169071	0.4096642	0.1202967	up
A_33_P3265704	UFD1L	7.2436666	7.2961984	7.4316597	0.0525317	0.1879931	0.1202624	up
A_23_P400459	TRMT10B	0.0431151	0.0999851	0.2264757	0.05687	0.1833606	0.1201153	up
A_23_P136238	TMEM203	3.8777122	3.9285083	4.0670958	0.050796	0.1893835	0.1200898	up
A_21_P0003642	lnc-SYNPO2-2	-2.582582	-3.036378	-1.888668	-0.453796	0.6939139	0.1200591	up
A_23_P427622	SPPL3	0.9489279	1.1959496	0.9418788	0.2470217	-0.007049	0.1199863	up
A_21_P0001470	lnc-C1orf195-2	-1.235642	-1.005143	-1.226171	0.2304983	0.0094709	0.1199846	up
A_33_P3294222	RLTPR	-2.333828	-2.344728	-2.083211	-0.0109	0.250617	0.1198584	up
A_21_P0000295	SNORA67	4.8235846	4.81675	5.0700817	-0.006835	0.2464972	0.1198313	up
A_21_P0006549	lnc-APLN-1	1.4707942	1.5136023	1.6675968	0.0428081	0.1968026	0.1198053	up
A_23_P217755	SHROOM2	-0.847261	-0.661442	-0.79355	0.1858196	0.0537114	0.1197655	up
A_32_P382427	PSMG3-AS1	-0.196705	0.0195327	-0.173627	0.2162376	0.0230775	0.1196575	up
A_33_P3231252	NHLH2	-0.871378	-0.640147	-0.863313	0.2312312	0.0080647	0.119648	up
A_23_P58763	PELO	5.239539	5.2317023	5.48666	-0.007837	0.2471209	0.119642	up
A_24_P58054	SLC9A8	1.201633	1.3502579	1.292275	0.1486249	0.090642	0.1196334	up
A_23_P421032	SEC14L4	-0.296915	-0.340415	-0.014224	-0.043499	0.2826915	0.119596	up
A_22_P00013710	HAS2-AS1	0.9104967	0.7290168	1.3308988	-0.18148	0.4204021	0.1194611	up
A_23_P27840	SUPT5H	3.7047548	3.981852	3.6665716	0.2770972	-0.038183	0.119457	up
A_22_P00013207	lnc-RNF24-2	-0.119872	0.0337939	-0.034741	0.153666	0.0851312	0.1193986	up
A_23_P143143	ID2	4.1343107	4.284364	4.2230015	0.1500535	0.0886908	0.1193721	up
A_23_P9152	RCL1	4.692067	4.8549533	4.7678795	0.1628861	0.0758123	0.1193492	up
A_23_P65870	FBXO22	3.0707664	3.3006845	3.0795012	0.229918	0.0087347	0.1193264	up
A_22_P00010993	lnc-NRXN1-2	-2.794453	-2.431155	-2.9191	0.3632977	-0.124648	0.119325	up

A_22_P00009274	lnc-LRRC14-1	-1.503292	-1.304431	-1.463615	0.1988616	0.0396771	0.1192694	up
A_33_P3373469	PSMD2	8.334801	8.460957	8.447169	0.1261559	0.1123686	0.1192622	up
A_21_P0012404	LINC01210	-1.108254	-0.944085	-1.034005	0.1641693	0.0742493	0.1192093	up
A_33_P3318327	FLJ31713	-1.846001	-1.39602	-2.057651	0.4499817	-0.211649	0.1191661	up
A_24_P312189	MEPCE	2.0371637	2.0537095	2.2588577	0.0165458	0.221694	0.1191199	up
A_24_P373333	FAM192A	2.3352013	2.5380235	2.3704052	0.2028222	0.0352039	0.1190131	up
A_33_P3217119	OSCP1	-0.384675	-0.374536	-0.156792	0.010139	0.2278824	0.1190107	up
A_22_P00013852	LOC400620	-0.561255	-0.353629	-0.531006	0.2076263	0.0302491	0.1189377	up
A_21_P0009632	lnc-LMAN1-1	-3.151546	-2.81824	-3.247003	0.3333066	-0.095457	0.1189246	up
A_21_P0011928	PEBP1	3.9916763	4.101807	4.119343	0.1101308	0.1276665	0.1188986	up
A_23_P83098	ALDH1A1	5.828017	5.977801	5.91601	0.1497836	0.0879927	0.1188881	up
A_23_P103256	CFHR3	-1.606306	-1.618607	-1.356365	-0.012301	0.2499404	0.1188195	up
A_21_P0001088	lnc-AL590822.1-1	-2.525466	-2.467304	-2.346027	0.0581625	0.1794388	0.1188006	up
A_23_P4160	NBR2	-0.614749	-0.489964	-0.502042	0.1247859	0.1127071	0.1187465	up
A_23_P12463	QSOX1	4.433261	4.6696486	4.434264	0.2363877	0.0010033	0.1186955	up
A_23_P90997	OTOS	-1.37708	-1.348287	-1.168671	0.0287929	0.2084088	0.1186008	up
A_21_P0010510	LINC01134	-2.531829	-2.335649	-2.490815	0.1961799	0.0410137	0.1185968	up
A_33_P3401322	ISG20L2	3.0065336	2.943233	3.306942	-0.063301	0.3004084	0.1185539	up
A_21_P0001802	SPATA3-AS1	-2.036904	-1.905055	-1.931989	0.1318493	0.1049151	0.1183822	up
A_24_P290373	CXorf56	-0.406892	-0.25689	-0.32032	0.1500025	0.0865722	0.1182873	up
A_33_P3465703	SNORA60	-2.128243	-1.918554	-2.10141	0.2096887	0.0268331	0.1182609	up
A_33_P3350853	PAXIP1-AS1	-2.736854	-2.936805	-2.300606	-0.199952	0.4362478	0.1181481	up
A_23_P43800	BOP1	4.614957	4.587212	4.878933	-0.027745	0.2639761	0.1181157	up
A_24_P385611	SP100	1.7849903	1.8975611	1.9086151	0.1125708	0.1236248	0.1180978	up
A_24_P267522	ZNF585A	-3.633433	-3.302178	-3.728503	0.3312547	-0.09507	0.1180924	up
A_24_P365954	THAP11	0.9494329	1.0143833	1.1203642	0.0649505	0.1709313	0.1179409	up
A_33_P3371224	ANO10	1.5649366	1.5770535	1.7886877	0.0121169	0.2237511	0.117934	up
A_23_P152727	PLEKHM1	4.2477684	4.365542	4.36586	0.1177735	0.1180916	0.1179326	up
A_23_P301328	PDXK	-2.955281	-2.42715	-3.247672	0.528131	-0.292391	0.1178701	up
A_22_P00012790	lnc-RALGPS1-1	-2.024647	-1.975546	-1.838044	0.0491014	0.1866036	0.1178525	up
A_23_P130531	CDC37	3.9773521	4.080693	4.109707	0.1033406	0.1323547	0.1178477	up
A_22_P00006932	lnc-GATAD1-2	-2.808668	-2.477662	-2.904047	0.3310063	-0.095379	0.1178137	up
A_23_P106002	NFKBIA	3.6003695	3.8181882	3.618085	0.2178187	0.0177155	0.1177671	up
A_22_P00022917	lnc-ALDH3A2-2	-1.727206	-1.753781	-1.465184	-0.026575	0.262022	0.1177235	up
A_32_P827528	S1PR2	1.947566	1.4765048	2.6539812	-0.471061	0.7064152	0.117677	up
A_23_P39375	KLHL26	-0.165592	-0.152002	0.0561647	0.0135899	0.2217565	0.1176732	up
A_23_P50455	POLD1	4.5410852	4.680465	4.6369762	0.13938	0.095891	0.1176355	up
A_24_P925314	GM2A	-0.415567	-0.479043	-0.116823	-0.063475	0.2987447	0.1176348	up
A_33_P3243248	ZNFX1	-1.434354	-1.206497	-1.427103	0.2278566	0.0072508	0.1175537	up
A_23_P110062	EIF2B5	2.8506222	2.8964915	3.039569	0.0458694	0.1889467	0.117408	up
A_23_P24485	NFRKB	-1.703055	-1.64793	-1.52344	0.0551248	0.179615	0.1173699	up
A_21_P0001493	lnc-PPT1-1	-2.941807	-2.634857	-3.014121	0.3069499	-0.072313	0.1173183	up
A_23_P75283	RBP4	-1.719723	-1.817235	-1.387638	-0.097512	0.3320856	0.1172869	up
A_23_P69720	ANXA5	5.75031	5.904676	5.830473	0.154366	0.080163	0.1172645	up
A_23_P204355	ZNF384	2.1661358	2.3848462	2.181943	0.2187104	0.0158072	0.1172588	up
A_23_P107421	TK1	8.436145	8.611738	8.494994	0.1755934	0.0588493	0.1172214	up
A_23_P29124	GP1BB	-2.494985	-2.468732	-2.286839	0.0262532	0.2081461	0.1171997	up
A_21_P0012984	lnc-FGF10-3	-1.429041	-1.166371	-1.457319	0.26267	-0.028278	0.1171961	up
A_33_P3209279	SASH1	-2.011661	-1.788353	-2.000609	0.2233076	0.0110517	0.1171796	up
A_33_P3474328	PPP1R11	-0.386641	-0.232487	-0.306464	0.1541538	0.0801768	0.1171653	up
A_33_P3262949	FERMT1	-2.550266	-2.831359	-2.034863	-0.281093	0.5154033	0.1171551	up
A_23_P1676	TMEM218	2.980031	3.0135512	3.180808	0.0335202	0.2007771	0.1171486	up
A_23_P218817	CPT1B	0.3044848	0.5119519	0.3313026	0.2074671	0.0268178	0.1171424	up
A_22_P00009257	lnc-LRP5-1	1.3230686	1.3988609	1.48143	0.0757923	0.1583614	0.1170769	up
A_33_P3365810	MRPL12	4.565215	4.592322	4.7722454	0.0271068	0.2070303	0.1170685	up
A_23_P70045	H2AFY	7.18814	7.288647	7.321745	0.1005073	0.133605	0.1170561	up

A_19_P00327354	LINC00963	0.1312957	0.4145851	0.082087	0.2832894	-0.049209	0.1170404	up
A_23_P3602	NUDT7	-0.78265	-0.850356	-0.480943	-0.067706	0.3017073	0.1170008	up
A_33_P3263307	RANGAP1	7.818368	7.895722	7.9748583	0.077354	0.1564903	0.1169221	up
A_19_P00316815	LINC00630	-2.303519	-2.271196	-2.102136	0.0323236	0.2013836	0.1168536	up
A_23_P502269	POLDIP3	4.290411	4.518783	4.295741	0.2283721	0.0053301	0.1168511	up
A_32_P18824	BRD7	6.0098276	6.0513854	6.201928	0.0415578	0.1921005	0.1168292	up
A_33_P3402116	AGO1	4.4871035	4.533733	4.6741085	0.0466294	0.187005	0.1168172	up
A_33_P3290748	LOC648044	-2.355811	-2.130565	-2.347464	0.2252462	0.0083475	0.1167969	up
A_33_P3213767	CCDC157	-0.200027	-0.023423	-0.143097	0.1766043	0.0569301	0.1167672	up
A_23_P131020	GATAD2A	2.8003063	3.0910144	2.743044	0.2907081	-0.057262	0.1167228	up
A_21_P0008701	lnc-LRRK1-2	1.1566906	1.4142437	1.1325736	0.2575531	-0.024117	0.1167181	up
A_23_P203819	GOLGA3	3.4552755	3.6204839	3.5233784	0.1652083	0.0681028	0.1166556	up
A_32_P155247	FTL	6.4053974	7.037499	6.006542	0.6321015	-0.398855	0.1166232	up
A_24_P127075	TMEM135	-2.020557	-1.788292	-2.01978	0.2322655	0.0007777	0.1165216	up
A_23_P138835	CAPN1	3.470932	3.5765557	3.5982609	0.1056237	0.1273289	0.1164763	up
A_23_P13753	NFE2	-0.70171	-0.252768	-0.917851	0.4489427	-0.21614	0.1164012	up
A_23_P60028	TCEB1	7.9268017	7.9031224	8.183219	-0.023679	0.2564173	0.116369	up
A_32_P430359	DDX54	6.4268866	6.4960465	6.590317	0.06916	0.1634302	0.1162951	up
A_23_P47790	METTL1	2.9878836	3.0883708	3.119956	0.1004872	0.1320725	0.1162798	up
A_33_P3661631	PCBD2	2.0430536	2.289393	2.0292053	0.2463393	-0.013848	0.1162455	up
A_23_P129695	VASN	3.6845198	3.8292375	3.7722712	0.1447177	0.0877514	0.1162345	up
A_33_P3292851	UBE2I	-0.634933	-0.513188	-0.52421	0.1217451	0.1107235	0.1162343	up
A_21_P0014945	DUX4	-2.270209	-2.107522	-2.200444	0.1626868	0.0697651	0.116226	up
A_33_P3240229	CREBBP	2.651579	2.776713	2.7588263	0.125134	0.1072474	0.1161907	up
A_33_P3318926	UNC13B	-0.16333	-0.022439	-0.071854	0.1408911	0.091476	0.1161835	up
A_21_P0005470	lnc-NDUFA4-1	-2.673147	-2.888045	-2.226147	-0.214898	0.4470003	0.1160513	up
A_24_P154080	ECE1	-1.311213	-1.225522	-1.16486	0.085691	0.1463532	0.1160221	up
A_24_P207727	MED15	1.9331322	2.018992	2.079237	0.0858598	0.1461048	0.1159823	up
A_24_P89971	SURF4	4.5755672	4.7327065	4.6503086	0.1571393	0.0747414	0.1159403	up
A_22_P00023002	lnc-PCYOX1-1	0.2853117	0.4248834	0.3776116	0.1395717	0.0922999	0.1159358	up
A_24_P12413	TRAM2	0.321064	0.2973638	0.5765629	-0.0237	0.2554989	0.1158993	up
A_24_P407717	GRB2	2.8776073	2.806127	3.1806993	-0.07148	0.303092	0.1158059	up
A_23_P80098	GART	4.9026146	4.902207	5.134609	-0.000408	0.2319946	0.1157935	up
A_21_P0011972	XLOC_l2_008313	0.5484624	0.6942968	0.6340079	0.1458345	0.0855455	0.11569	up
A_23_P138524	CPXM2	-2.677878	-2.552191	-2.572192	0.1256869	0.1056862	0.1156865	up
A_33_P3256232	MIIP	4.3100853	4.2480836	4.6034536	-0.062002	0.2933683	0.1156833	up
A_24_P338648	ARL1	3.784398	3.8016152	3.9984083	0.0172172	0.2140102	0.1156137	up
A_23_P105002	ROM1	1.9696302	1.9474368	2.2230482	-0.022193	0.253418	0.1156123	up
A_33_P3405360	PDCL	-0.746397	-0.695632	-0.566053	0.0507646	0.1803432	0.1155539	up
A_22_P00008037	LINC01280	-2.796744	-2.777644	-2.584776	0.0191002	0.211968	0.1155341	up
A_33_P3314341	KIAA1958	-2.900169	-2.761901	-2.807575	0.138268	0.0925942	0.1154311	up
A_32_P738377	AATBC	-1.389656	-1.139664	-1.408897	0.2499914	-0.019242	0.1153748	up
A_23_P258689	DNAAF5	2.8239489	2.941051	2.9375496	0.1171021	0.1136007	0.1153514	up
A_24_P132039	RNF14	0.1504579	0.3059278	0.2256022	0.1554699	0.0751443	0.1153071	up
A_33_P3356216	ARCN1	2.1869345	2.226565	2.37788	0.0396304	0.1909456	0.115288	up
A_24_P341187	GBA2	-0.079393	-0.003997	0.0755425	0.0753956	0.1549354	0.1151655	up
A_23_P24157	PYROXD2	0.015203	0.1218271	0.1388445	0.1066241	0.1236415	0.1151328	up
A_33_P3239587	MXRA7	6.00928	6.1387725	6.1099434	0.1294923	0.1006632	0.1150777	up
A_21_P0008405	lnc-C14orf79-2	-2.127737	-1.917246	-2.108202	0.2104907	0.0195351	0.1150129	up
A_21_P0014608	LOC102724021	-2.914577	-2.86868	-2.730457	0.0458972	0.1841199	0.1150086	up
A_22_P00010120	LOC101928069	0.6256423	0.653976	0.8273048	0.0283337	0.2016625	0.1149981	up
A_23_P102937	SUMO3	3.7962894	4.2887607	3.5334797	0.4924712	-0.26281	0.1148307	up
A_33_P3381318	FAM160A1	0.496738	0.8026815	0.4203849	0.3059435	-0.076353	0.1147952	up
A_33_P3322415	MARK4	-1.318258	-1.229323	-1.177638	0.0889354	0.1406202	0.1147778	up
A_33_P3408962	SCN8A	-1.06509	-1.087388	-0.813407	-0.022297	0.2516832	0.1146929	up
A_23_P255569	DUS1L	3.8140783	4.00635	3.8511667	0.1922717	0.0370884	0.1146801	up

A_23_P65757	CCNB2	5.744936	5.7032285	6.0159636	-0.041708	0.2710276	0.11466	up
A_33_P3385983	ZNF20	-0.675844	-0.453565	-0.66912	0.2222796	0.0067239	0.1145017	up
A_21_P0014848	TMEM165	-3.031211	-2.59659	-3.236982	0.4346206	-0.205771	0.1144248	up
A_24_P73075	TTC12	0.3014484	0.5511599	0.2804976	0.2497115	-0.020951	0.1143804	up
A_23_P62270	KDM5C	2.3107529	2.324205	2.5260391	0.0134521	0.2152863	0.1143692	up
A_23_P94819	RPH3AL	0.6036282	0.7259212	0.7100258	0.122293	0.1063976	0.1143453	up
A_23_P207742	THRA	5.799073	6.066109	5.7607164	0.267036	-0.038357	0.1143396	up
A_23_P252536	MRPS26	5.5293083	5.476395	5.810897	-0.052913	0.2815886	0.1143377	up
A_24_P295877	CEP104	-0.993414	-0.772983	-0.985232	0.2204313	0.0081825	0.1143069	up
A_22_P00018466	LOC102725086	-2.789512	-2.917097	-2.433315	-0.127584	0.3561974	0.1143065	up
A_33_P3320533	POLR1A	-2.371999	-2.531177	-1.984296	-0.159177	0.3877034	0.1142631	up
A_33_P3347320	ADIRF-AS1	-1.038306	-0.551079	-1.297027	0.487227	-0.258721	0.114253	up
A_22_P00021145	LOC101927557	-3.02371	-2.75174	-3.067318	0.2719696	-0.043608	0.1141806	up
A_22_P00016496	lnc-TMEM99-5	1.9932499	2.0311065	2.1837301	0.0378566	0.1904802	0.1141684	up
A_23_P349127	CDAN1	-0.422036	-0.679195	0.0634432	-0.25716	0.4854789	0.1141596	up
A_23_P3453	RPAP1	3.6188202	3.5074105	3.9584923	-0.11141	0.3396721	0.1141312	up
A_33_P3254412	ULK2	-3.080772	-3.055793	-2.877489	0.0249789	0.2032836	0.1141312	up
A_23_P91590	RANBP1	6.7001724	6.698649	6.9299088	-0.001523	0.2297363	0.1141064	up
A_32_P806841	ARL4A	1.1430335	1.2166929	1.2975607	0.0736594	0.1545272	0.1140933	up
A_21_P0006884	lnc-EIF5AL1-1	-1.494028	-1.195883	-1.564261	0.2981443	-0.070233	0.1139555	up
A_21_P0000285	SNORD49A	1.5460243	1.7356701	1.5842214	0.1896458	0.038197	0.1139214	up
A_22_P00024629	lnc-CDKAL1-2	-2.787293	-2.72185	-2.624961	0.0654428	0.1623313	0.1138871	up
A_33_P3416767	MAFG	1.194984	1.3428831	1.2747221	0.1478992	0.0797381	0.1138186	up
A_23_P105625	COQ5	3.367838	3.6115785	3.3516788	0.2437406	-0.016159	0.1137908	up
A_24_P322908	USP27X	0.324985	0.2727461	0.604744	-0.052239	0.2797589	0.11376	up
A_21_P0001725	lnc-ELOVL1-1	-2.692443	-2.31727	-2.84016	0.3751731	-0.147717	0.1137282	up
A_33_P3303355	ZCCHC18	0.486186	0.6043077	0.5954785	0.1181216	0.1092925	0.1137071	up
A_33_P3330179	LOC100131372	-2.46024	-2.10781	-2.585372	0.3524299	-0.125132	0.1136489	up
A_23_P130027	EPN3	3.7630014	3.6982217	4.0549555	-0.06478	0.291954	0.1135871	up
A_23_P887	IKBKE	-1.880673	-1.761335	-1.772839	0.119338	0.1078339	0.1135859	up
A_33_P3287710	DLG5-AS1	0.0443258	0.1879172	0.1278892	0.1435914	0.0835633	0.1135774	up
A_33_P3244141	NDEL1	3.278716	3.4755645	3.3089523	0.1968484	0.0302362	0.1135423	up
A_24_P321626	POLR3B	1.327249	1.3274598	1.5540423	0.0002108	0.2267933	0.113502	up
A_22_P00001425	lnc-APITD1-1	-2.680575	-2.594987	-2.539182	0.0855882	0.141393	0.1134906	up
A_33_P3345812	GPER1	0.3888464	0.1700797	0.8344488	-0.218767	0.4456024	0.1134179	up
A_33_P3266780	PODXL2	-0.087104	-0.163461	0.2160292	-0.076357	0.303133	0.1133881	up
A_33_P3219260	PDZD11	3.5900164	3.662994	3.7436028	0.0729775	0.1535864	0.113282	up
A_23_P350886	GLTSCR1L	0.6574969	0.9879236	0.5535684	0.3304267	-0.103929	0.1132491	up
A_22_P00018564	LOC101928725	-0.643179	-0.702175	-0.357754	-0.058996	0.2854257	0.113215	up
A_23_P104116	RBM8A	7.117341	7.1503463	7.310692	0.0330052	0.1933508	0.113178	up
A_21_P0013589	LINC01504	-2.836306	-2.985002	-2.461347	-0.148696	0.3749597	0.1131319	up
A_33_P3395971	CTU1	1.6668196	1.5902543	1.9696274	-0.076565	0.3028078	0.1131213	up
A_33_P3244194	PTPLB	-1.428299	-1.195429	-1.434995	0.2328706	-0.006695	0.1130877	up
A_33_P3242080	DDX19A	3.8629208	3.8724065	4.079487	0.0094857	0.2165661	0.1130259	up
A_22_P00023904	lnc-GPR182-1	-2.411682	-1.990378	-2.60698	0.4213033	-0.195298	0.1130027	up
A_23_P103414	YTHDF2	5.3583946	5.396338	5.5463285	0.0379434	0.1879339	0.1129386	up
A_32_P107746	ENSA	3.5932894	3.8258834	3.5864697	0.232594	-0.00682	0.1128871	up
A_23_P432610	N4BP1	1.7470903	1.8155026	1.9044304	0.0684123	0.1573401	0.1128762	up
A_23_P65230	TMTC4	1.2597604	1.2585912	1.486568	-0.001169	0.2268076	0.1128192	up
A_23_P27677	IRF3	3.13299	3.1499028	3.341648	0.0169129	0.2086582	0.1127856	up
A_23_P208870	AKT2	3.7539778	4.0550075	3.6784267	0.3010297	-0.075551	0.1127393	up
A_21_P0013888	TPTE2	-0.701536	-0.739247	-0.438359	-0.037712	0.2631764	0.1127324	up
A_23_P212284	POC1A	1.995223	2.2493272	1.9663095	0.2541041	-0.028913	0.1125953	up
A_21_P0004273	lnc-XRCC4-1	-1.35564	-0.839776	-1.646414	0.5158634	-0.290774	0.1125445	up
A_24_P289726	PSMD3	5.346635	5.4262633	5.4919624	0.0796285	0.1453276	0.112478	up
A_33_P3351175	WNK2	2.5311136	2.4900947	2.7970543	-0.041019	0.2659407	0.1124609	up

A_22_P00016054	DLGAP1-AS1	1.8446407	2.138867	1.7753248	0.2942262	-0.069316	0.1124551	up
A_32_P129527	ERMARD	1.7506418	1.8828311	1.8433313	0.1321893	0.0926895	0.1124394	up
A_23_P111635	STYXL1	1.8872595	1.9937396	2.005618	0.1064801	0.1183586	0.1124194	up
A_23_P110196	HERC5	0.3612523	0.3745198	0.5728216	0.0132675	0.2115693	0.1124184	up
A_33_P3357935	STRC	-0.973928	-0.883318	-0.839701	0.0906096	0.1342268	0.1124182	up
A_23_P317244	SPACA3	-0.746377	-0.347944	-0.920035	0.3984327	-0.173658	0.1123874	up
A_33_P3315856	DHX34	3.543746	3.503943	3.8082666	-0.039803	0.2645207	0.1123588	up
A_33_P3273624	HYAL3	-0.793337	-0.661273	-0.700818	0.1320643	0.0925193	0.1122918	up
A_21_P0007715	lnc-CTDSP2-4	-0.762764	-0.687113	-0.614008	0.0756512	0.148756	0.1122036	up
A_21_P0014166	LOC100506379	-3.128054	-2.91119	-3.120603	0.2168634	0.0074511	0.1121572	up
A_22_P00003842	LOC100129083	-2.726464	-2.661055	-2.567574	0.0654087	0.15889	0.1121494	up
A_33_P3331652	EMC9	-0.131945	0.1781101	-0.21809	0.3100553	-0.086144	0.1119554	up
A_21_P0007546	LOC100288798	-2.566557	-2.546544	-2.362747	0.0200129	0.2038097	0.1119113	up
A_23_P51202	ZNF436	-0.130528	0.0937996	-0.131061	0.2243271	-0.000534	0.1118968	up
A_24_P267592	SAMHD1	-2.322731	-2.805956	-1.615731	-0.483225	0.7069998	0.1118872	up
A_24_P305570	RIN2	3.0659895	3.2747931	3.0809135	0.2088037	0.014924	0.1118638	up
A_23_P129556	IL4R	4.0250387	4.0053763	4.2683268	-0.019662	0.243288	0.1118128	up
A_33_P3209491	TNS1	-0.167232	-0.121994	0.0110221	0.045238	0.1782541	0.1117461	up
A_23_P17420	BCAS1	-1.088929	-1.025001	-0.929488	0.0639281	0.159441	0.1116846	up
A_23_P416774	CLIC5	2.806367	2.8013415	3.0347157	-0.005025	0.2283487	0.1116617	up
A_24_P17870	HCP5	1.4123473	1.2239408	1.8238935	-0.188406	0.4115462	0.1115699	up
A_33_P3389827	PROM2	3.8177414	3.8266892	4.0318565	0.0089478	0.2141151	0.1115315	up
A_23_P45475	GLA	5.15744	5.22047	5.3174314	0.0630298	0.1599913	0.1115105	up
A_23_P215790	EGFR	1.8059826	2.1075377	1.7273436	0.3015552	-0.078639	0.1114581	up
A_24_P475814	OPA3	4.1774073	4.434712	4.1429567	0.2573047	-0.034451	0.1114271	up
A_22_P00008734	SNHG17	1.1746049	1.3169208	1.2551222	0.1423159	0.0805173	0.1114166	up
A_23_P63847	SUPV3L1	2.313034	2.3402324	2.508646	0.0271983	0.195612	0.1114051	up
A_21_P0011603	AMZ2	2.8159056	2.9692082	2.8853617	0.1533027	0.0694561	0.1113794	up
A_21_P0007309	lnc-GLB1L2-3	-0.014484	0.0936403	0.1001334	0.1081243	0.1146174	0.1113708	up
A_21_P0005640	lnc-FBXO25-3	1.3712521	1.6724572	1.2927608	0.3012052	-0.078491	0.111357	up
A_23_P206856	WDR59	1.8716488	2.0926166	1.8732948	0.2209678	0.001646	0.1113069	up
A_23_P434352	CAST	5.089299	5.353152	5.0480595	0.2638526	-0.04124	0.1113064	up
A_32_P466514	IRF2BPL	-1.480123	-1.335023	-1.402617	0.1450996	0.0775065	0.1113031	up
A_23_P344988	ICK	0.6849647	0.6044474	0.9880104	-0.080517	0.3030458	0.1112642	up
A_21_P0000102	MTRNR2L2	8.770753	8.9994545	8.764555	0.2287016	-0.006198	0.1112518	up
A_24_P291826	SYTL3	1.8934822	2.2551217	1.7542434	0.3616395	-0.139239	0.1112003	up
A_33_P3214597	RP9P	-0.355575	-0.248016	-0.240802	0.1075592	0.1147728	0.111166	up
A_33_P3523501	LOC374890	-1.517592	-1.302152	-1.510741	0.2154403	0.0068512	0.1111457	up
A_23_P137391	ENO1	6.268505	6.3625636	6.396695	0.0940585	0.12819	0.1111243	up
A_23_P147025	RAB33A	-2.664212	-2.303273	-2.802908	0.3609388	-0.138696	0.1111214	up
A_32_P221256	MGC70870	2.6208868	2.7556653	2.70819	0.1347785	0.0873032	0.1110408	up
A_23_P9061	EIF3H	8.733949	8.896276	8.793588	0.1623278	0.059639	0.1109834	up
A_24_P397150	GAK	2.7220516	2.8081622	2.8578815	0.0861106	0.1358299	0.1109703	up
A_23_P169887	GTF3C2	4.9460335	5.0894494	5.024418	0.1434159	0.0783844	0.1109002	up
A_23_P65466	RAB2B	-1.055877	-1.01838	-0.87161	0.0374975	0.184267	0.1108823	up
A_23_P102508	SLC5A6	2.9501	3.0680146	3.0538568	0.1179147	0.1037569	0.1108358	up
A_23_P107465	KRT31	-1.32045	-1.774177	-0.64509	-0.453727	0.6753602	0.1108165	up
A_23_P147984	TRIP12	5.1565924	5.3084664	5.2262545	0.1518741	0.0696621	0.1107681	up
A_33_P3318861	DYTN	2.7644033	2.9510226	2.7992973	0.1866193	0.034894	0.1107566	up
A_22_P00022830	lnc-ROPN1B-1	-2.28726	-2.025209	-2.327844	0.2620502	-0.040585	0.1107327	up
A_23_P99138	MRPL51	7.4534483	7.587295	7.5410023	0.1338468	0.087554	0.1107004	up
A_23_P121345	TBCCD1	-0.014557	0.0221262	0.1699748	0.0366836	0.1845322	0.1106079	up
A_33_P3326553	TMEM82	2.3639927	2.4936147	2.4554262	0.129622	0.0914335	0.1105278	up
A_23_P251974	ZFYVE9	-2.385832	-2.022056	-2.52857	0.3637767	-0.142738	0.1105195	up
A_33_P3323559	CRYAA	-0.320128	-0.28548	-0.133787	0.0346479	0.1863408	0.1104944	up
A_23_P154643	BMP7	2.2979155	2.1062655	2.7105541	-0.19165	0.4126387	0.1104944	up

A_32_P122940	LOC642852	-0.22173	-0.027981	-0.194567	0.1937485	0.0271626	0.1104555	up
A_23_P61447	ETFDH	2.721466	3.0199084	2.6439142	0.2984424	-0.077552	0.1104453	up
A_21_P0000276	SNORD87	0.0568485	0.2646689	0.0699153	0.2078204	0.0130668	0.1104436	up
A_23_P92860	CCNO	3.1372795	3.1565871	3.338605	0.0193076	0.2013254	0.1103165	up
A_23_P35205	RCAN3	1.0140624	1.2471247	1.0016294	0.2330623	-0.012433	0.1103146	up
A_23_P214474	TOMM6	6.0973444	5.891725	6.5233507	-0.205619	0.4260063	0.1101935	up
A_23_P370924	SMIM4	4.8128233	4.7730317	5.072958	-0.039792	0.2601347	0.1101716	up
A_32_P218025	LOC100506253	-1.821713	-2.087062	-1.336051	-0.26535	0.4856615	0.1101558	up
A_23_P111303	SCAF8	2.7296677	2.8187242	2.8609219	0.0890565	0.1312542	0.1101553	up
A_33_P3380101	MAP7D1	4.828514	4.7731977	5.1041374	-0.055316	0.2756233	0.1101534	up
A_32_P56713	BCR	1.920373	1.9015064	2.159381	-0.018867	0.239008	0.1100707	up
A_33_P3292844	NEUROG3	1.1648521	1.3078871	1.2417941	0.1430349	0.076942	0.1099885	up
A_33_P3297888	CCDC125	2.0792093	2.1089206	2.269456	0.0297112	0.1902466	0.1099789	up
A_32_P95223	FDPSP2	4.0186825	3.879046	4.3782015	-0.139637	0.359519	0.1099412	up
A_23_P9458	POLR1E	1.7929478	1.9741225	1.8314271	0.1811748	0.0384793	0.109827	up
A_23_P385063	DNAJB6	2.0434866	2.212422	2.0941591	0.1689353	0.0506725	0.1098039	up
A_33_P3382177	TIMP2	6.180007	6.559958	6.019494	0.379951	-0.160513	0.109719	up
A_33_P3364459	GMPR2	0.6127691	0.429348	1.0155177	-0.183421	0.4027486	0.1096637	up
A_23_P153197	TGIF1	3.1333904	3.3974338	3.0886726	0.2640433	-0.044718	0.1096628	up
A_23_P348063	SYNGR1	-0.072211	-0.024741	0.099607	0.0474701	0.1718178	0.1096439	up
A_21_P0000061	CGREF1	0.0025048	0.2048712	0.0194097	0.2023664	0.0169048	0.1096356	up
A_33_P3404697	PSTK	1.7479019	1.7489262	1.9659586	0.0010242	0.2180567	0.1095405	up
A_23_P202138	SFXN3	3.01824	3.050322	3.2048187	0.0320821	0.1865788	0.1093304	up
A_24_P47681	CAND1	3.0381231	3.227097	3.0676556	0.1889739	0.0295324	0.1092532	up
A_23_P214739	FBXL4	2.315506	2.524671	2.3246508	0.2091651	0.0091448	0.1091549	up
A_23_P106973	SEPT9	8.425046	8.538139	8.530167	0.1130934	0.1051207	0.109107	up
A_19_P00319655	CTD-3080P12.3	-2.886639	-2.983521	-2.571588	-0.096882	0.3150506	0.1090845	up
A_23_P5221	ZNF333	-1.495451	-1.219156	-1.553595	0.2762942	-0.058144	0.1090751	up
A_33_P3793702	LINC00964	-2.155294	-1.872225	-2.220321	0.2830691	-0.065027	0.1090213	up
A_22_P00019831	LOC101927137	-2.377336	-2.090029	-2.446725	0.2873066	-0.069389	0.1089587	up
A_33_P3315510	ANKS1A	-2.394865	-2.144059	-2.42779	0.2508059	-0.032926	0.10894	up
A_24_P268160	DRAM2	1.8498249	2.0380406	1.8794832	0.1882157	0.0296583	0.108937	up
A_23_P67725	LMNB2	2.0440931	2.0694995	2.236538	0.0254064	0.1924448	0.1089256	up
A_33_P3256105	DUX4	-0.820416	-0.755567	-0.667532	0.0648484	0.152884	0.1088662	up
A_24_P148811	RUVBL1	0.6086774	0.6023192	0.8326364	-0.006358	0.223959	0.1088004	up
A_23_P319859	EYA2	3.865079	3.7852168	4.1625004	-0.079862	0.2974215	0.1087797	up
A_22_P00017762	CCNT2-AS1	0.1175795	0.2625146	0.1901298	0.1449351	0.0725503	0.1087427	up
A_33_P3221019	ZAN	-2.670503	-2.776333	-2.347268	-0.10583	0.3232353	0.1087028	up
A_23_P214487	HIST1H4C	9.202403	9.280205	9.341948	0.0778017	0.1395445	0.1086731	up
A_22_P00002248	FLJ37035	0.1486659	0.3170757	0.197505	0.1684098	0.0488391	0.1086245	up
A_23_P30069	DDX60L	1.3177786	1.148099	1.7046943	-0.16968	0.3869157	0.108618	up
A_24_P80181	TMEM127	3.473298	3.643435	3.5203886	0.1701369	0.0470905	0.1086137	up
A_23_P91293	VAPB	2.9618254	3.0858912	3.0549622	0.1240659	0.0931368	0.1086013	up
A_22_P00025851	lnc-NUFIP2-1	-2.142934	-1.966592	-2.102212	0.1763415	0.0407214	0.1085315	up
A_23_P80377	PACSIN2	3.1519175	3.273332	3.247551	0.1214147	0.0956335	0.1085241	up
A_22_P00013992	lnc-RUNDC3A-1	-1.496678	-1.361867	-1.414454	0.1348105	0.0822244	0.1085174	up
A_24_P399630	PRKACA	1.3848319	1.4419103	1.5447669	0.0570784	0.159935	0.1085067	up
A_21_P0008037	LOC101928839	-2.656337	-2.574019	-2.521694	0.0823178	0.1346428	0.1084803	up
A_23_P103131	MIEF1	4.952051	4.984573	5.1364365	0.0325217	0.1843853	0.1084535	up
A_23_P57658	HRASLS	0.4073038	0.5349321	0.4965677	0.1276283	0.0892639	0.1084461	up
A_33_P3350094	PATL2	-2.256086	-1.907709	-2.387694	0.3483772	-0.131608	0.1083844	up
A_23_P259127	ESRP1	2.377349	2.6574893	2.3138409	0.2801404	-0.063508	0.1083162	up
A_23_P413761	SRSF3	6.8932104	7.0212073	6.9817934	0.1279969	0.088583	0.10829	up
A_23_P145657	STAG3	2.8850956	2.817391	3.1692495	-0.067705	0.2841539	0.1082246	up
A_33_P3374559	GUSBP11	-1.109928	-1.062102	-0.941331	0.0478258	0.1685972	0.1082115	up
A_22_P00023982	KBTBD11-OT1	-1.594251	-1.994152	-0.97804	-0.399901	0.6162109	0.1081548	up

A_33_P3233774	ZNF174	-0.570409	-0.370409	-0.554116	0.2000003	0.016293	0.1081467	up
A_23_P114445	MAGEE1	-1.495933	-1.608703	-1.166905	-0.112771	0.3290277	0.1081285	up
A_23_P380917	SEC11A	6.5693407	6.629559	6.7253466	0.0602183	0.1560059	0.1081121	up
A_23_P97892	LDB1	-0.645177	-0.621742	-0.452426	0.0234351	0.1927509	0.108093	up
A_33_P3559102	LOC440864	-1.833544	-1.583483	-1.867441	0.250061	-0.033897	0.1080818	up
A_33_P3346108	RUFY4	-2.270055	-1.964926	-2.359051	0.3051291	-0.088996	0.1080667	up
A_33_P3383189	SP9	0.3468766	0.5021281	0.4077382	0.1552515	0.0608616	0.1080565	up
A_24_P330691	SUMO2	6.8431253	6.904288	6.998067	0.0611625	0.1549416	0.108052	up
A_33_P3297399	KCTD9	0.351943	0.3594809	0.560494	0.0075378	0.2085509	0.1080444	up
A_33_P3386262	CDT1	4.36497	4.3687263	4.577298	0.003756	0.212328	0.108042	up
A_33_P3305368	DDX31	0.9907064	1.0837674	1.113606	0.093061	0.1228995	0.1079803	up
A_22_P00012775	lnc-RALB-1	-2.761736	-2.564396	-2.743487	0.1973395	0.0182483	0.1077939	up
A_22_P00004297	CNPY3	-0.274302	0.1694169	-0.502442	0.4437184	-0.22814	0.107789	up
A_32_P213661	CCDC122	-0.49407	-0.308727	-0.463837	0.1853423	0.0302324	0.1077874	up
A_23_P106682	EMP2	6.8584356	6.8301606	7.1022596	-0.028275	0.243824	0.1077745	up
A_33_P3681213	PREPL	2.1147852	2.4327245	2.0123434	0.3179393	-0.102442	0.1077487	up
A_19_P00319981	LINC01503	-3.217256	-3.020151	-3.199021	0.1971049	0.0182354	0.1076702	up
A_24_P117954	INO80C	0.0228171	0.0734878	0.187479	0.0506706	0.1646619	0.1076663	up
A_22_P00004542	PARD3-AS1	-2.864097	-2.047508	-3.46536	0.8165891	-0.601263	0.1076632	up
A_23_P104109	RPS6KC1	1.1516066	1.3628154	1.1557002	0.2112088	0.0040936	0.1076512	up
A_23_P215735	ST7	0.2510538	0.3093596	0.4079609	0.0583057	0.1569071	0.1076064	up
A_23_P128543	MED4	4.8683043	4.911324	5.040433	0.0430198	0.1721287	0.1075742	up
A_23_P135722	BTC	-0.965901	-0.761823	-0.954856	0.2040782	0.0110445	0.1075614	up
A_23_P71864	FAM73B	0.8069735	0.9516997	0.877192	0.1447263	0.0702186	0.1074724	up
A_23_P164536	PIK3C3	2.023491	2.0315251	2.2303934	0.0080342	0.2069025	0.1074684	up
A_21_P0000270	SNORA63	3.1249285	3.0456967	3.419094	-0.079232	0.2941656	0.1074669	up
A_33_P3230526	MPRIP	-0.958341	-0.788995	-0.91297	0.1693459	0.0453715	0.1073587	up
A_23_P30200	PCDHB16	-1.654191	-1.58688	-1.506832	0.0673113	0.1473589	0.1073351	up
A_23_P309865	ZNF449	-1.421147	-1.374322	-1.253326	0.0468245	0.1678209	0.1073227	up
A_22_P00014116	lnc-SCAMP5-1	-1.649219	-1.642653	-1.441284	0.006566	0.2079353	0.1072507	up
A_24_P941268	CA5B	-1.05407	-0.939993	-0.953862	0.1140761	0.1002073	0.1071417	up
A_23_P146811	FBXO38	0.4776254	0.5730891	0.5963087	0.0954638	0.1186833	0.1070735	up
A_23_P53557	LTBR	4.0158825	4.1629233	4.0827475	0.1470408	0.066865	0.1069529	up
A_33_P3226099	OTOP2	-2.960997	-2.988574	-2.719777	-0.027577	0.2412198	0.1068214	up
A_33_P3697530	SEMA4D	-0.881576	-1.060453	-0.489066	-0.178877	0.3925095	0.1068161	up
A_24_P391260	PTTG1IP	2.6222115	2.777082	2.68091	0.1548705	0.0586987	0.1067846	up
A_33_P3338693	SNAP25	-0.688169	-0.519195	-0.643711	0.1689735	0.0444574	0.1067154	up
A_33_P3407985	TMEM99	-0.541935	-0.58881	-0.282049	-0.046874	0.2598867	0.1065063	up
A_24_P124672	DYNLL1	7.2622976	7.3270955	7.410366	0.0647979	0.1480684	0.1064332	up
A_21_P0002043	LOC100130502	-1.992592	-1.991404	-1.780915	0.0011883	0.2116771	0.1064327	up
A_24_P16214	LINC00665	-0.354874	-0.346346	-0.150651	0.0085278	0.2042232	0.1063755	up
A_23_P365719	TAPBP	3.3917341	3.2430634	3.753087	-0.148671	0.3613529	0.1063411	up
A_33_P3393350	RHOT2	5.219779	5.35772	5.2944746	0.1379409	0.0746956	0.1063182	up
A_23_P126716	ATPIF1	7.6845493	7.838926	7.74275	0.1543765	0.0582008	0.1062887	up
A_23_P168567	GTF2I	6.417904	6.4451966	6.6031733	0.0272927	0.1852694	0.106281	up
A_24_P77676	HSPA9	4.4112415	4.4685664	4.566324	0.0573249	0.1550827	0.1062038	up
A_21_P0013251	PMS2	2.6184816	2.7626147	2.6866856	0.1441331	0.0682039	0.1061685	up
A_23_P26704	MFSD11	2.4688263	2.6171327	2.532814	0.1483064	0.0639877	0.1061471	up
A_21_P0011008	XLOC_l2_002651	-2.540471	-2.321429	-2.547227	0.2190428	-0.006756	0.1061435	up
A_33_P3373541	ELAC2	-0.607415	-0.598682	-0.40399	0.0087328	0.2034249	0.1060789	up
A_22_P00013909	SLC37A4	-0.933465	-0.888885	-0.765977	0.0445805	0.1674881	0.1060343	up
A_23_P207981	SOCS6	-2.574632	-2.444105	-2.493214	0.1305268	0.0814183	0.1059725	up
A_24_P91566	BMP7	1.1619806	1.0486121	1.4872456	-0.113369	0.3252649	0.1059482	up
A_32_P72394	PRKAR1B	-0.636872	-0.341941	-0.720099	0.2949309	-0.083226	0.1058524	up
A_22_P00012859	LINC01317	-1.062372	-1.030726	-0.882395	0.0316463	0.1799769	0.1058116	up
A_24_P276490	LYPLA2	0.5822411	0.4440689	0.9319935	-0.138172	0.3497524	0.1057901	up

A_33_P3316026	USF2	1.1307473	1.3670883	1.1058288	0.236341	-0.024919	0.1057112	up
A_23_P160968	LAMC2	1.8141079	2.0458035	1.7938261	0.2316957	-0.020282	0.1057069	up
A_33_P3382399	KRTAP3-1	-0.951426	-1.125324	-0.56613	-0.173899	0.3852959	0.1056986	up
A_23_P18413	TIMMDC1	5.829832	5.9961867	5.8747234	0.1663547	0.0448914	0.105623	up
A_24_P460419	LRRC10B	-1.671032	-1.709787	-1.421097	-0.038754	0.2499356	0.1055906	up
A_23_P351342	PLEKHM2	-0.491076	-0.313773	-0.45734	0.1773028	0.0337362	0.1055195	up
A_33_P3352664	SP7	-1.300455	-1.140922	-1.24895	0.159533	0.0515046	0.1055188	up
A_23_P26777	NUP85	4.784357	4.8829684	4.896723	0.0986114	0.1123657	0.1054885	up
A_23_P135184	RALGDS	3.1060495	3.0267463	3.3962831	-0.079303	0.2902336	0.1054652	up
A_22_P00008113	lnc-IL21R-1	-2.004227	-1.83079	-1.966743	0.1734366	0.0374837	0.1054602	up
A_21_P0000172	CSPG5	-1.761365	-1.768805	-1.543346	-0.007439	0.21802	0.1052904	up
A_23_P79302	LYPD6B	0.3217177	0.5072684	0.3466411	0.1855507	0.0249233	0.105237	up
A_33_P3259428	WDR55	3.112276	3.0489068	3.386119	-0.063369	0.2738428	0.1052368	up
A_23_P128734	ERH	6.7511883	6.826692	6.8859634	0.0755038	0.1347752	0.1051395	up
A_21_P0008496	lnc-RP11-736N17.6.1-1	-2.767983	-2.571163	-2.754529	0.1968207	0.013454	0.1051374	up
A_19_P00321259	LINC01133	2.567133	2.6868653	2.6576624	0.1197324	0.0905294	0.1051309	up
A_33_P3363560	TMEM51	3.3043032	3.3378348	3.4810266	0.0335317	0.1767235	0.1051276	up
A_24_P945283	DLG3	4.312481	4.2468314	4.5883503	-0.06565	0.2758694	0.1051099	up
A_21_P0006572	LOC101928359	-1.367491	-1.410661	-1.114127	-0.04317	0.2533636	0.1050966	up
A_23_P79818	OSER1	3.930685	4.2484937	3.8230429	0.3178086	-0.107642	0.1050832	up
A_22_P00017996	lnc-ZNF131-2	-2.317551	-2.314264	-2.110714	0.0032876	0.2068377	0.1050626	up
A_33_P3249439	NCAPH2	6.043276	6.1824994	6.1141653	0.1392236	0.0708895	0.1050565	up
A_33_P3307486	SYNRG	-2.091104	-1.803895	-2.168351	0.2872095	-0.077246	0.1049815	up
A_33_P3309929	HDAC3	4.35874	4.342196	4.5851946	-0.016544	0.2264547	0.1049554	up
A_23_P48997	PSTPIP1	-2.120023	-1.992086	-2.038138	0.1279373	0.0818849	0.1049111	up
A_33_P3256793	KIAA1324	-2.18253	-1.95645	-2.198819	0.2260799	-0.016289	0.1048956	up
A_23_P150092	SEPHS1	4.4777985	4.524776	4.640484	0.0469775	0.1626854	0.1048315	up
A_21_P0001142	lnc-SLC44A5-4	-0.15174	0.0073514	-0.101252	0.159091	0.050488	0.1047895	up
A_19_P00321310	LOC100506127	-2.350658	-2.199754	-2.292004	0.1509037	0.0586538	0.1047788	up
A_23_P303548	NOL4L	-2.159151	-1.702226	-2.40658	0.4569244	-0.24743	0.1047473	up
A_33_P3296682	NDUFB5	5.4078646	5.424377	5.6008186	0.0165124	0.1929541	0.1047332	up
A_21_P0003497	LINC01365	-1.258739	-1.063877	-1.244475	0.1948624	0.0142636	0.104563	up
A_22_P00018257	lnc-ZNF726-1	-0.683963	-0.636881	-0.522013	0.0470824	0.1619506	0.1045165	up
A_21_P0000200	MCPH1	1.4231782	1.6908693	1.364336	0.2676911	-0.058842	0.1044245	up
A_23_P58293	UBE2D3	3.9449177	4.083339	4.015276	0.1384215	0.0703583	0.1043899	up
A_23_P65532	PELI2	-2.342777	-2.21302	-2.263771	0.1297565	0.0790062	0.1043813	up
A_33_P3382730	LARP4B	0.7193613	0.8150525	0.8324018	0.0956912	0.1130405	0.1043658	up
A_33_P3372682	GTSE1-AS1	-0.676297	-0.826483	-0.317424	-0.150186	0.3588729	0.1043434	up
A_21_P0003911	lnc-DCTD-1	-0.019292	0.4593773	-0.28939	0.4786692	-0.270098	0.1042857	up
A_21_P0011401	LOC100996255	-0.865017	-0.522948	-0.998671	0.3420692	-0.133654	0.1042078	up
A_23_P12329	APH1A	2.7991362	2.8089786	2.9977064	0.0098424	0.1985703	0.1042063	up
A_23_P89215	FN3K	-2.245694	-2.193189	-2.089876	0.0525045	0.1558175	0.104161	up
A_24_P257416	CXCL2	-0.845097	-0.689364	-0.792511	0.1557331	0.0525866	0.1041598	up
A_23_P207927	C17orf80	1.6872349	1.7320485	1.8507023	0.0448136	0.1634674	0.1041405	up
A_22_P00015994	ATP6V1B1-AS1	-2.328292	-2.379104	-2.069255	-0.050812	0.259037	0.1041123	up
A_24_P53519	CHAF1A	4.34604	4.3925815	4.5075235	0.0465417	0.1614838	0.1040127	up
A_22_P00018124	ZNF32-AS1	-2.724823	-2.802902	-2.43895	-0.078079	0.2858725	0.1038966	up
A_23_P202945	ZBTB44	0.8755331	1.1945763	0.7642312	0.3190432	-0.111302	0.1038706	up
A_32_P221991	RALGPS1	-0.882742	-0.718242	-0.839571	0.1645002	0.0431714	0.1038358	up
A_24_P302695	MRPL30	4.930092	4.9337935	5.1338177	0.0037017	0.2037258	0.1037137	up
A_23_P254271	TUBB6	7.296296	7.2306676	7.569294	-0.065629	0.2729979	0.1036847	up
A_22_P00004265	lnc-CMTM5-1	0.3465757	0.4652529	0.4348707	0.1186771	0.088295	0.1034861	up
A_24_P381975	TMEM231	-3.298124	-2.851272	-3.538095	0.4468522	-0.239971	0.1034405	up
A_23_P425304	SUFU	-0.38185	-0.329536	-0.227386	0.0523138	0.1544638	0.1033888	up
A_22_P00000560	lnc-AC127496.3-4	-1.457115	-1.505167	-1.202292	-0.048052	0.2548232	0.1033857	up

A_33_P3384845	POFUT1	-2.480997	-2.278373	-2.476929	0.2026241	0.0040684	0.1033462	up
A_23_P125643	ASB9	1.550045	1.8418412	1.4649372	0.2917962	-0.085108	0.1033442	up
A_32_P192545	TCEAL6	1.1259079	1.0442972	1.4142017	-0.081611	0.2882938	0.1033416	up
A_21_P0011389	XLOC_l2_005076	-2.695857	-2.954553	-2.230538	-0.258696	0.4653187	0.1033113	up
A_22_P00021143	lnc-CCDC166-1	-1.730145	-1.577004	-1.676669	0.1531405	0.0534754	0.103308	up
A_23_P94365	ADCK5	1.7576828	1.9578052	1.764091	0.2001224	0.0064082	0.1032653	up
A_33_P3222917	CD276	6.4716864	6.4829574	6.6668463	0.011271	0.1951599	0.1032155	up
A_21_P0001238	lnc-EGLN1-1	4.4179544	4.5349374	4.5071974	0.1169829	0.0892429	0.1031129	up
A_33_P3215333	NFATC2	-1.700853	-1.473815	-1.721684	0.2270384	-0.020831	0.1031039	up
A_24_P235049	MTHFD1L	2.7221107	3.0663257	2.5840797	0.3442149	-0.138031	0.103092	up
A_23_P161918	CCDC86	6.491848	6.4119496	6.777796	-0.079898	0.2859478	0.1030247	up
A_24_P860797	PAIP2B	0.5936909	0.7045808	0.6886911	0.1108899	0.0950003	0.1029451	up
A_23_P47226	YIF1A	6.514448	6.2529216	6.981803	-0.261527	0.4673548	0.1029141	up
A_23_P154065	TUBA4A	7.218769	7.12686	7.5164404	-0.091909	0.2976713	0.1028812	up
A_33_P3390918	FSD2	-3.123166	-3.066881	-2.973727	0.0562851	0.1494391	0.1028621	up
A_33_P3333488	HAGH	4.6739273	4.7375474	4.815996	0.0636201	0.1420689	0.1028445	up
A_33_P3385842	CCDC7	-1.294563	-0.943941	-1.439728	0.3506227	-0.145164	0.1027291	up
A_23_P360804	CPNE5	-0.246859	-0.212026	-0.076303	0.034833	0.1705551	0.102694	up
A_23_P91468	PSMA7	6.56783	6.727655	6.613386	0.1598249	0.0455561	0.1026905	up
A_23_P6398	AP1B1	1.5134177	1.4962196	1.7359772	-0.017198	0.2225595	0.1026807	up
A_23_P116387	INCENP	3.0190592	2.7766004	3.466878	-0.242459	0.4478188	0.10268	up
A_23_P119344	TEAD2	-1.507648	-1.345483	-1.464463	0.1621647	0.0431852	0.102675	up
A_22_P00010666	lnc-NEO1-1	-1.191302	-1.049972	-1.1273	0.1413307	0.064002	0.1026664	up
A_22_P00013057	LOC284648	-0.029645	0.1881104	-0.042108	0.2177553	-0.012463	0.1026461	up
A_23_P161324	NUDT13	-2.809987	-2.370682	-3.044013	0.4393051	-0.234026	0.1026394	up
A_33_P3306397	CIAO1	3.050642	3.0562882	3.2502308	0.0056462	0.1995888	0.1026175	up
A_23_P80773	SRPRB	5.053645	5.290215	5.022297	0.2365699	-0.031348	0.1026108	up
A_33_P3406998	DYRK1A	-0.691399	-0.464593	-0.713133	0.2268057	-0.021734	0.102536	up
A_22_P00000848	ZBED3-AS1	1.6921749	1.7013755	1.8879738	0.0092006	0.1957989	0.1024997	up
A_33_P3334037	WBP11	3.2574978	3.3216643	3.3982716	0.0641665	0.1407738	0.1024702	up
A_22_P00018241	LOC101927905	-3.046372	-2.992742	-2.895138	0.0536306	0.1512346	0.1024326	up
A_23_P138881	ACTN3	-0.957589	-1.067097	-0.643298	-0.109509	0.3142905	0.102391	up
A_32_P44775	C9orf85	0.8347006	1.0687284	0.805419	0.2340279	-0.029282	0.1023731	up
A_33_P3342563	PITPNM3	2.7347775	2.8357768	2.8385124	0.1009994	0.103735	0.1023672	up
A_21_P0000495	SNORA57	5.6184187	5.5950656	5.846422	-0.023353	0.2280035	0.1023252	up
A_23_P41066	RASSF1	3.612399	3.7716737	3.6577673	0.1592746	0.0453682	0.1023214	up
A_23_P82913	TRMT12	3.7918558	3.8775887	3.9107552	0.0857329	0.1188993	0.1023161	up
A_33_P3241081	AKNAD1	-2.431311	-2.605932	-2.05207	-0.174621	0.3792405	0.1023096	up
A_23_P87560	BTG1	1.2644997	1.6843581	1.0492568	0.4198585	-0.215243	0.1023078	up
A_23_P302134	FLJ37453	-0.470617	-0.303314	-0.433428	0.1673026	0.037189	0.1022458	up
A_23_P33093	ST6GALNAC5	-2.619792	-2.405129	-2.630056	0.2146635	-0.010264	0.1021999	up
A_33_P3314192	PACSIN2	2.306324	2.4562879	2.360692	0.1499639	0.054368	0.1021659	up
A_33_P3281465	SLC5A6	2.1201181	2.2914958	2.152872	0.1713777	0.0327539	0.1020658	up
A_23_P139297	TRIM44	1.6832924	1.6441817	1.9264317	-0.039111	0.2431393	0.1020143	up
A_23_P128073	SMARCC2	5.4675293	5.493402	5.6455526	0.0258727	0.1780233	0.101948	up
A_23_P54576	KIFC3	-0.279781	-0.113195	-0.242557	0.1665855	0.0372238	0.1019046	up
A_33_P3318288	CFH	-0.41469	-0.440692	-0.185119	-0.026002	0.2295709	0.1017842	up
A_23_P169050	MRPS28	5.081918	5.1702046	5.1970816	0.0882869	0.1151638	0.1017253	up
A_33_P3391596	LAMA4	-2.280357	-1.915019	-2.442317	0.3653383	-0.16196	0.1016892	up
A_23_P157352	MRPS33	3.956478	4.0673556	4.048937	0.1108775	0.0924587	0.1016681	up
A_33_P3386955	MRPS5	6.1307764	6.206658	6.2581825	0.0758815	0.1274061	0.1016438	up
A_23_P46844	TRIM8	3.3481102	3.601461	3.297989	0.2533507	-0.050121	0.1016147	up
A_23_P167227	HADH	5.3213663	5.4705586	5.375388	0.1491923	0.0540218	0.1016071	up
A_23_P143147	TCFL5	1.9558115	2.1011147	2.0136185	0.1453033	0.057807	0.1015551	up
A_23_P111487	SRRT	1.0424671	1.1876559	1.1003141	0.1451888	0.057847	0.1015179	up
A_33_P3308387	CASP2	-0.284363	-0.079614	-0.286192	0.2047491	-0.001829	0.1014602	up

A_33_P3282384	SIMC1	-1.058835	-1.120618	-0.79429	-0.061783	0.2645445	0.1013806	up
A_33_P3280721	WLS	2.145503	2.2200723	2.273491	0.0745692	0.1279879	0.1012785	up
A_23_P89062	CLUHP3	0.6790209	0.8028069	0.7577167	0.123786	0.0786958	0.1012409	up
A_22_P00024715	GRAMD1A	0.0134916	0.0681896	0.1610766	0.054698	0.1475849	0.1011415	up
A_22_P00002539	PLSCR3	-1.275638	-1.23394	-1.115328	0.0416985	0.1603098	0.1010041	up
A_23_P85777	GNPAT	3.2520723	3.5073876	3.1985264	0.2553153	-0.053546	0.1008847	up
A_21_P0007046	lnc-GATA3-1	-2.927944	-3.077487	-2.576633	-0.149543	0.3513115	0.1008843	up
A_33_P3240747	LOC100130899	2.4143066	2.281342	2.748866	-0.132965	0.3345594	0.1007974	up
A_32_P66364	PPP1R1C	0.7446857	0.9169622	0.7737036	0.1722765	0.0290179	0.1006472	up
A_23_P154566	TOX2	2.3435307	2.4273057	2.4610338	0.083775	0.1175032	0.1006391	up
A_33_P3354683	PIGS	5.894602	5.789636	6.200838	-0.104966	0.3062363	0.1006353	up
A_23_P85598	ADCK3	1.4415503	1.7618508	1.3223529	0.3203006	-0.119197	0.1005516	up
A_22_P00003640	lnc-CD80-1	-2.971261	-2.955422	-2.786011	0.0158384	0.1852498	0.1005441	up
A_24_P328492	SOCS5	1.0328422	1.2357001	1.0309992	0.202858	-0.001843	0.1005075	up
A_23_P112187	FIBCD1	-0.352658	-0.317271	-0.187282	0.035387	0.1653762	0.1003816	up
A_23_P160992	FMO4	-1.518542	-1.82987	-1.006513	-0.311328	0.5120292	0.1003506	up
A_33_P3221843	COQ3	0.6483278	0.4219775	1.075284	-0.22635	0.4269562	0.1003029	up
A_21_P0000603	ASB9P1	-0.455294	-0.218675	-0.491499	0.2366195	-0.036205	0.1002073	up
A_23_P209731	ARMC9	3.0333757	2.941935	3.3251677	-0.091441	0.2917919	0.1001756	up
A_23_P217114	ALAD	-1.150809	-1.037183	-1.064158	0.1136265	0.0866509	0.1001387	up
A_23_P89460	AATF	4.654298	4.86599	4.6428175	0.2116923	-0.01148	0.100106	up
A_24_P56894	CRLF3	-0.977336	-0.898156	-0.856461	0.0791802	0.1208754	0.1000278	up
A_33_P3348973	PIKFYVE	-1.593898	-1.453727	-1.534061	0.1401711	0.0598373	0.1000042	up
A_33_P3271599	CCDC125	0.9942908	1.0084519	1.1799712	0.0141611	0.1856804	0.0999208	up
A_33_P3399864	ZNF792	-2.28542	-1.959442	-2.411576	0.3259783	-0.126156	0.099911	up
A_33_P3294159	CALY	-1.631905	-1.437987	-1.626054	0.1939178	0.0058513	0.0998845	up
A_23_P369899	TMEM158	0.8823514	0.8930721	1.0713859	0.0107207	0.1890345	0.0998776	up
A_23_P1682	TMEM45B	2.8528929	3.0485978	2.8566236	0.1957049	0.0037308	0.0997179	up
A_22_P00018457	lnc-CCDC51-1	-0.623183	-0.571003	-0.476103	0.0521808	0.1470799	0.0996304	up
A_21_P0002481	lnc-TGFBRAP1-3	-2.623306	-2.49164	-2.555906	0.131666	0.0673997	0.0995328	up
A_33_P3348159	MKLN1	1.833334	1.961349	1.9043655	0.128015	0.0710316	0.0995233	up
A_21_P0004551	LINC01019	-2.696532	-2.676871	-2.517162	0.0196612	0.1793699	0.0995156	up
A_23_P205778	GNB5	0.2163801	0.1890268	0.4425454	-0.027353	0.2261653	0.099406	up
A_33_P3271301	TCF20	-0.870307	-0.909898	-0.631917	-0.03959	0.2383909	0.0994003	up
A_23_P383278	PYCRL	2.4369116	2.53375	2.5383883	0.0968385	0.1014767	0.0991576	up
A_23_P89799	ACAA2	3.6030579	3.849671	3.554716	0.246613	-0.048342	0.0991356	up
A_23_P161399	MXI1	1.5753794	1.7773752	1.571537	0.2019959	-0.003842	0.0990767	up
A_23_P121326	CYB561D2	1.0481267	1.0582161	1.23592	0.0100894	0.1877933	0.0989413	up
A_23_P10559	AATK	3.2946014	3.461635	3.3252554	0.1670337	0.030654	0.0988438	up
A_24_P4816	GABARAPL1	0.4535356	1.0466142	0.0581069	0.5930786	-0.395429	0.098825	up
A_23_P114774	PMVK	4.0606213	4.0255704	4.293231	-0.035051	0.2326098	0.0987794	up
A_23_P24633	THYN1	3.1191835	3.290996	3.1449099	0.1718125	0.0257263	0.0987694	up
A_23_P88865	CMTM3	1.5757589	1.7799354	1.5690212	0.2041764	-0.006738	0.0987194	up
A_21_P0005187	lnc-DLL1-4	-1.823783	-1.446908	-2.003244	0.3768759	-0.179461	0.0987077	up
A_33_P3239864	ANKDD1A	-1.53377	-1.317005	-1.553145	0.2167654	-0.019375	0.0986953	up
A_23_P502047	CHRD	-1.36147	-1.084415	-1.441135	0.2770543	-0.079665	0.0986948	up
A_33_P3316522	DEFB124	-1.620505	-1.319603	-1.724134	0.3009024	-0.103629	0.0986366	up
A_23_P429581	TMEM67	-1.902654	-1.72661	-1.88149	0.1760435	0.0211639	0.0986037	up
A_22_P00024189	lnc-RNLS-1	-2.441922	-1.93644	-2.750221	0.5054817	-0.308299	0.0985914	up
A_32_P133244	PARN	2.4989796	2.6191373	2.5759544	0.1201577	0.0769749	0.0985663	up
A_23_P144465	PAPSS1	1.4942136	1.4496975	1.7355657	-0.044516	0.2413521	0.098418	up
A_24_P103922	CTBP2	2.859295	3.0550685	2.86034	0.1957736	0.0010452	0.0984094	up
A_23_P133814	HIST1H3C	6.355587	6.399874	6.5080748	0.0442872	0.1524878	0.0983875	up
A_32_P83049	EFR3B	2.2690897	2.091619	2.6432714	-0.177471	0.3741818	0.0983555	up
A_33_P3382606	FBXW12	-2.735731	-2.789206	-2.48572	-0.053475	0.250011	0.0982679	up
A_33_P3396635	SSX3	-1.314684	-1.011063	-1.422281	0.3036213	-0.107597	0.0980122	up

A_23_P408913	TTC30B	-0.059545	-0.023706	0.1006279	0.0358386	0.1601725	0.0980055	up
A_33_P3326256	MRPS16	3.5076752	3.5967402	3.614585	0.0890651	0.1069098	0.0979874	up
A_33_P3361701	UBAP2L	2.303115	2.3856673	2.416524	0.0825524	0.113409	0.0979807	up
A_23_P25204	SLC25A3	8.905465	9.21648	8.790401	0.3110151	-0.115064	0.0979757	up
A_22_P00000865	LOC101927609	-1.126398	-1.127401	-0.929456	-0.001003	0.1969419	0.0979693	up
A_22_P00011856	lnc-PHYHD1-1	-2.845552	-2.81027	-2.685036	0.0352821	0.160517	0.0978996	up
A_33_P3372727	SEMA5A	2.084548	2.107613	2.2572517	0.0230651	0.1727037	0.0978844	up
A_23_P94998	LETM1	1.6716871	1.6579099	1.8811188	-0.013777	0.2094317	0.0978272	up
A_23_P48771	C14orf159	0.2149367	0.2139463	0.4114685	-0.00099	0.1965318	0.0977707	up
A_23_P55376	KANSL1	3.186966	3.318688	3.2507505	0.131722	0.0637846	0.0977533	up
A_23_P105545	VAMP1	-2.907281	-2.899996	-2.719131	0.0072854	0.1881506	0.097718	up
A_33_P3393766	C17orf96	5.5804443	5.471461	5.8848524	-0.108984	0.3044081	0.0977123	up
A_33_P3352562	MFSD12	1.1925869	1.2515206	1.3285675	0.0589337	0.1359806	0.0974572	up
A_19_P00318600	LOC648987	-0.954208	-0.901916	-0.811676	0.0522919	0.1425319	0.0974119	up
A_23_P217475	IDS	3.3022623	3.4187417	3.3805037	0.1164794	0.0782414	0.0973604	up
A_33_P3264121	TBCA	7.8577595	7.9402966	7.969941	0.0825372	0.1121817	0.0973594	up
A_23_P160689	LRIF1	-0.495045	-0.337421	-0.458125	0.1576238	0.0369205	0.0972722	up
A_32_P59302	HIVEP3	-0.996907	-1.103665	-0.695722	-0.106758	0.3011856	0.0972137	up
A_23_P422511	SLC25A45	-1.785815	-1.802519	-1.574705	-0.016705	0.2111096	0.0972025	up
A_23_P379945	CCDC88C	0.9089117	0.9545465	1.0574179	0.0456347	0.1485062	0.0970705	up
A_23_P75609	CEP164	0.5414634	0.6576285	0.6194267	0.1161652	0.0779634	0.0970643	up
A_24_P824592	RBMX	-0.249936	-0.000333	-0.305521	0.2496028	-0.055585	0.0970089	up
A_33_P3332487	FANK1	-2.088863	-1.496936	-2.486813	0.5919275	-0.397949	0.096989	up
A_33_P3333146	RAP1GAP2	-2.259441	-2.008023	-2.316935	0.2514186	-0.057493	0.0969626	up
A_33_P3281151	FUBP1	3.994215	4.1770663	4.005275	0.1828513	0.0110598	0.0969555	up
A_23_P76841	NGDN	3.7809305	3.8411508	3.9145918	0.0602202	0.1336613	0.0969408	up
A_23_P339954	NPIPB9	5.7837286	6.079986	5.6813326	0.2962575	-0.102396	0.0969307	up
A_23_P154832	ATP5J	6.5392685	6.519275	6.7529488	-0.019993	0.2136803	0.0968435	up
A_23_P68486	FAM210B	1.8968649	1.8951406	2.092245	-0.001724	0.1953802	0.096828	up
A_23_P204503	PRKAB1	0.8242412	0.9665351	0.8755255	0.1422939	0.0512843	0.0967891	up
A_23_P8185	DYNLT1	6.817499	6.894261	6.934022	0.0767617	0.1165228	0.0966423	up
A_24_P216253	DLGAP4	2.4046516	2.4751363	2.5274515	0.0704846	0.1227999	0.0966423	up
A_24_P322847	POLR3H	4.521682	4.3647723	4.871786	-0.156909	0.3501043	0.0965974	up
A_23_P341065	SELO	2.2173033	2.2034278	2.4241562	-0.013875	0.2068529	0.0964887	up
A_23_P170978	ZNF692	3.4242907	3.479795	3.5616884	0.0555043	0.1373978	0.096451	up
A_23_P160200	ZFP69B	-0.326616	-0.400019	-0.060327	-0.073403	0.2662892	0.0964432	up
A_22_P00022674	lnc-POLR1E-1	-1.492706	-1.330273	-1.462491	0.1624327	0.0302153	0.096324	up
A_23_P6099	PLCB1	-0.176956	-0.015796	-0.145561	0.1611595	0.0313945	0.096277	up
A_33_P3292525	TMEM201	2.9820013	2.9037719	3.2527666	-0.078229	0.2707653	0.0962679	up
A_33_P3239569	RPS21	-0.387531	-0.373423	-0.209132	0.0141087	0.1783991	0.0962539	up
A_24_P39211	ING1	1.5786772	1.7056608	1.6441555	0.1269836	0.0654783	0.096231	up
A_21_P0011049	XLOC_l2_003050	0.8354545	0.7750502	1.0882912	-0.060404	0.2528367	0.0962162	up
A_23_P397417	CNEP1R1	1.935214	1.871428	2.1911764	-0.063786	0.2559624	0.0960882	up
A_23_P130787	QPCTL	-0.381139	-0.264919	-0.305283	0.11622	0.0758567	0.0960383	up
A_33_P3325355	TOMM34	5.6256485	5.7371697	5.7061167	0.1115212	0.0804682	0.0959947	up
A_33_P3265359	HES6	2.9782438	2.750218	3.3980722	-0.228026	0.4198284	0.0959013	up
A_21_P0011074	XLOC_l2_003253	-2.7205	-2.268508	-2.980871	0.4519916	-0.260371	0.0958104	up
A_21_P0008498	lnc-TMEM179-2	-0.719076	-0.788678	-0.457883	-0.069602	0.2611933	0.0957956	up
A_33_P3306843	ZNF836	-2.199966	-2.009615	-2.198972	0.190351	0.0009937	0.0956724	up
A_23_P68884	DRG1	4.3792048	4.4681277	4.4815483	0.088923	0.1023436	0.0956333	up
A_32_P156963	ACTG1	7.4706907	7.539285	7.593298	0.0685945	0.1226072	0.0956008	up
A_22_P00000483	IPO5P1	-1.149864	-1.115727	-0.992951	0.0341368	0.1569128	0.0955248	up
A_24_P416177	ADCY7	2.8745852	3.014852	2.9250498	0.1402669	0.0504646	0.0953658	up
A_23_P354908	NMNAT2	-2.083947	-1.888973	-2.088338	0.1949744	-0.004391	0.0952916	up
A_23_P4425	FLII	3.144906	3.2152085	3.2651749	0.0703025	0.1202688	0.0952857	up
A_22_P00017486	TMEM88B	-2.859854	-2.739622	-2.789583	0.1202326	0.0702715	0.095252	up

A_21_P0000250	SNORD36A	2.40631	2.4187198	2.584241	0.0124097	0.1779308	0.0951703	up
A_33_P3401990	VPREB3	-1.581463	-1.386391	-1.586484	0.1950717	-0.005022	0.0950251	up
A_33_P3399268	IL15RA	-0.861655	-0.866834	-0.666649	-0.005179	0.1950059	0.0949135	up
A_23_P371613	CHCHD1	5.760113	5.75909	5.9509573	-0.001023	0.1908445	0.0949109	up
A_24_P416997	APOL3	-3.439609	-3.289805	-3.399687	0.1498034	0.0399218	0.0948626	up
A_21_P0000799	RNASEH1-AS1	-0.78999	-0.657815	-0.732535	0.132174	0.0574546	0.0948143	up
A_23_P46309	RCC1	2.0747128	2.0582242	2.2806187	-0.016489	0.2059059	0.0947087	up
A_23_P215296	CDK13	2.7306995	2.8348632	2.815774	0.1041637	0.0850744	0.094619	up
A_33_P3322724	POLR3A	2.9630404	2.9803462	3.1348324	0.0173059	0.171792	0.0945489	up
A_24_P944827	ATG7	-0.22802	-0.336491	0.0693307	-0.10847	0.2973509	0.0944402	up
A_22_P00013104	lnc-RIMS3-2	-1.560636	-1.497929	-1.434513	0.0627065	0.126123	0.0944147	up
A_33_P3269636	SBSN	1.0686398	1.234272	1.0918355	0.1656323	0.0231957	0.094414	up
A_23_P217917	GSTM4	-1.496026	-1.722547	-1.080801	-0.226521	0.4152255	0.0943522	up
A_33_P3399840	USP39	5.3302	5.3720183	5.4768705	0.0418181	0.1466703	0.0942442	up
A_32_P132438	AKAP1	5.6934576	5.7859387	5.7891817	0.0924811	0.0957241	0.0941026	up
A_24_P160401	CDCP1	1.0502634	1.1852269	1.1033611	0.1349635	0.0530977	0.0940306	up
A_23_P122439	BTN2A2	-0.745644	-0.572034	-0.73154	0.1736097	0.0141034	0.0938566	up
A_21_P0010444	lnc-NFAM1-1	-2.338663	-1.896914	-2.592748	0.4417486	-0.254085	0.0938317	up
A_23_P153628	YIF1B	1.7732515	1.5571766	2.1769123	-0.216075	0.4036608	0.0937929	up
A_24_P81841	CDKN1B	2.418374	2.6222863	2.4019327	0.2039123	-0.016441	0.0937355	up
A_21_P0011959	XLOC_l2_008221	-2.646042	-2.468814	-2.635851	0.1772273	0.0101902	0.0937088	up
A_21_P0010029	lnc-FAM182B-1	-2.349069	-2.151054	-2.359688	0.1980143	-0.010619	0.0936975	up
A_22_P00015032	lnc-SMUG1-6	-1.568817	-1.35801	-1.592264	0.2108069	-0.023448	0.0936797	up
A_23_P1819	OR8B8	-0.192739	-0.023697	-0.17444	0.1690416	0.0182986	0.0936701	up
A_22_P00018477	lnc-THOC5-1	-1.795468	-2.049972	-1.353778	-0.254503	0.44169	0.0935934	up
A_23_P22625	SLC9A6	2.8827906	3.034266	2.91827	0.1514754	0.0354795	0.0934775	up
A_33_P3220827	PEA15	1.9752073	2.0299172	2.1073914	0.0547099	0.132184	0.093447	up
A_23_P61487	LRRC20	3.4268398	3.3573775	3.6831894	-0.069462	0.2563496	0.0934436	up
A_23_P500271	IRF5	2.695775	2.573769	3.0045862	-0.122006	0.3088112	0.0934026	up
A_23_P23839	LGR6	3.7307978	3.9142365	3.7341194	0.1834388	0.0033216	0.0933802	up
A_33_P3691916	FAM13A	0.231348	0.2693791	0.380003	0.0380311	0.1486549	0.093343	up
A_23_P75839	TSG101	3.8231554	3.8486705	3.984291	0.0255151	0.1611357	0.0933254	up
A_33_P3336878	TMEM180	-0.308691	-0.301956	-0.128855	0.0067348	0.1798363	0.0932856	up
A_23_P370142	PAFAH1B2	4.372863	4.4330115	4.499235	0.0601487	0.1263723	0.0932605	up
A_24_P218814	RDH5	2.224904	2.2355676	2.400693	0.0106635	0.1757889	0.0932262	up
A_33_P3220857	C9orf64	-2.346301	-1.926249	-2.58028	0.4200525	-0.233979	0.0930367	up
A_32_P169131	ALG1	2.9649754	2.8869681	3.228959	-0.078007	0.2639837	0.0929883	up
A_23_P157215	BZW2	6.75194	6.7789693	6.9108267	0.0270295	0.1588869	0.0929582	up
A_33_P3258801	SLURP1	-1.994326	-1.84337	-1.959436	0.1509557	0.0348902	0.0929229	up
A_32_P173298	SF3B3	0.9880362	1.1321859	1.0297079	0.1441498	0.0416718	0.0929108	up
A_23_P373724	PPFIBP1	4.4740925	4.491223	4.6426363	0.0171304	0.1685438	0.0928371	up
A_23_P37988	CPNE2	0.4738698	0.4696264	0.6637335	-0.004243	0.1898637	0.0928102	up
A_33_P3251322	MTMR14	-0.730841	-0.737528	-0.538645	-0.006688	0.1921954	0.0927539	up
A_22_P00014894	lnc-SLC7A5-1	2.8637486	3.0047908	2.908207	0.1410422	0.0444584	0.0927503	up
A_33_P3276112	lnc-CTD-2517M22.14.1-1	-3.244338	-3.045597	-3.257657	0.1987405	-0.013319	0.0927107	up
A_24_P291598	USP4	2.8654833	2.9658866	2.9504232	0.1004033	0.08494	0.0926716	up
A_24_P396375	ECE1	1.3414297	1.4718151	1.3962951	0.1303854	0.0548654	0.0926254	up
A_33_P3267543	DPM2	-3.07235	-2.964829	-2.994625	0.1075213	0.0777247	0.092623	up
A_23_P431789	ARF4	5.1535454	5.321652	5.1703806	0.1681066	0.0168352	0.0924709	up
A_23_P164737	MED26	3.8999739	4.0230317	3.9618235	0.1230578	0.0618496	0.0924537	up
A_23_P7783	KDM3B	2.8713446	2.883018	3.0444012	0.0116735	0.1730566	0.092365	up
A_33_P3317305	AGAP1	-0.549874	-0.346175	-0.568902	0.2036991	-0.019028	0.0923357	up
A_33_P3365932	WASH1	1.8147707	1.9284272	1.8857594	0.1136565	0.0709887	0.0923226	up
A_33_P3364373	KRTAP5-2	0.1975818	0.4845119	0.0951781	0.2869301	-0.102404	0.0922632	up
A_33_P3351851	HIST1H4L	1.275558	1.202414	1.5331602	-0.073144	0.2576022	0.0922291	up

A_23_P350451	PRDM1	-0.447222	-0.189754	-0.520278	0.2574678	-0.073056	0.0922058	up
A_33_P3271051	CYTH3	4.298209	4.3993278	4.381201	0.1011186	0.0829916	0.0920551	up
A_23_P18579	PTTG2	4.983178	4.9861665	5.164236	0.0029883	0.1810579	0.0920231	up
A_24_P295379	GPR107	4.3886013	4.4248776	4.5363493	0.0362763	0.147748	0.0920122	up
A_24_P267997	TOR1AIP1	0.2346711	0.2429519	0.4104066	0.0082808	0.1757355	0.0920081	up
A_22_P00015588	LOC102724791	-2.185985	-2.083762	-2.104341	0.1022229	0.0816445	0.0919337	up
A_23_P356101	FBXO11	0.3174748	0.4231834	0.3955956	0.1057086	0.0781207	0.0919147	up
A_33_P3222243	MED15P9	0.0320148	0.1784239	0.0693359	0.146409	0.0373211	0.0918651	up
A_33_P3233105	MLLT4	0.0881453	0.2664523	0.0935278	0.1783071	0.0053825	0.0918448	up
A_23_P4522	TXNL1	5.8038044	5.8691773	5.9220552	0.0653729	0.1182509	0.0918119	up
A_33_P3387931	CENPP	1.4115872	1.4729023	1.5338497	0.0613151	0.1222625	0.0917888	up
A_23_P122947	AVL9	0.8816819	1.2262502	0.7206798	0.3445683	-0.161002	0.091783	up
A_23_P61810	BAIAP2	2.62817	2.6288571	2.8108501	0.0006871	0.1826801	0.0916836	up
A_33_P7431456	HLA-E	9.358257	9.536737	9.363067	0.1784802	0.0048094	0.0916448	up
A_33_P3220202	PKN3	3.247263	3.3141713	3.3636332	0.0669084	0.1163702	0.0916393	up
A_24_P337419	TIGD6	-2.73643	-2.891617	-2.397971	-0.155188	0.3384588	0.0916356	up
A_24_P172993	UBE2O	3.6855145	3.655808	3.8984737	-0.029706	0.2129593	0.0916264	up
A_33_P3211054	ZNF684	-0.129122	-0.02845	-0.046677	0.1006718	0.0824451	0.0915585	up
A_24_P205019	ZNF250	0.6759591	0.8123264	0.7226505	0.1363673	0.0466914	0.0915294	up
A_23_P49021	WDR61	3.2667046	3.4403958	3.2759428	0.1736913	0.0092382	0.0914648	up
A_23_P215406	RAC1	4.875016	4.9412274	4.9917145	0.0662112	0.1166983	0.0914547	up
A_33_P3285024	TMEM203	2.468583	2.517303	2.6027365	0.0487199	0.1341534	0.0914366	up
A_24_P36299	ARHGAP35	-1.947311	-1.973907	-1.738006	-0.026595	0.2093058	0.0913553	up
A_22_P00019874	lnc-NPY5R-4	0.8660412	1.0168304	0.8979507	0.1507893	0.0319095	0.0913494	up
A_33_P3714482	LRP5L	-2.277021	-2.182073	-2.189284	0.0949485	0.0877378	0.0913432	up
A_23_P159101	SNRNP35	1.770957	1.823039	1.9015112	0.0520821	0.1305542	0.0913181	up
A_24_P653603	C17orf89	7.2059765	7.2157845	7.3787346	0.0098081	0.1727581	0.0912831	up
A_33_P3350493	CLPSL2	-0.54474	-0.571631	-0.335298	-0.02689	0.2094421	0.0912759	up
A_23_P502609	PIGQ	2.2476473	2.3379102	2.339901	0.0902629	0.0922537	0.0912583	up
A_23_P80778	DIRC2	3.2621574	3.5916467	3.1151342	0.3294892	-0.147023	0.091233	up
A_21_P0000089	ZFYVE28	-3.293235	-2.883513	-3.520564	0.4097221	-0.227329	0.0911967	up
A_23_P25073	MRPS35	5.8175592	5.8437495	5.9737263	0.0261903	0.156167	0.0911787	up
A_33_P3369663	SUN1	1.6524515	1.8775568	1.6096821	0.2251053	-0.042769	0.0911679	up
A_21_P0003088	lnc-AC069257.9.1-6	-1.629161	-1.477876	-1.59812	0.1512857	0.0310411	0.0911634	up
A_23_P12140	RPL5	6.077015	6.2824607	6.0538607	0.2054458	-0.023154	0.0911458	up
A_24_P194000	SAE1	6.643482	6.62802	6.841222	-0.015462	0.1977396	0.0911386	up
A_33_P3353622	RBAK	-2.425567	-2.418601	-2.250385	0.0069654	0.1751819	0.0910736	up
A_23_P71415	WDYHV1	1.556591	1.6431623	1.6521039	0.0865712	0.0955129	0.091042	up
A_23_P134237	RARRES2	-2.020604	-2.477761	-1.381394	-0.457157	0.6392098	0.0910264	up
A_32_P45009	IDH1	4.218148	4.3777413	4.240591	0.1595931	0.0224428	0.091018	up
A_21_P0000772	OSER1-AS1	-1.952276	-1.698381	-2.024136	0.2538948	-0.07186	0.0910175	up
A_33_P6812319	NDUFV3	-0.129535	-0.252223	0.1751494	-0.122688	0.3046846	0.0909984	up
A_33_P3297255	MXD3	2.962654	2.9710116	3.136259	0.0083575	0.173605	0.0909812	up
A_33_P3354181	KCMF1	0.6243882	0.7883182	0.642354	0.1639299	0.0179658	0.0909479	up
A_24_P23445	RNU12	0.7125721	0.7546029	0.8523579	0.0420308	0.1397858	0.0909083	up
A_23_P131435	CD302	-0.022563	-0.041304	0.177979	-0.018741	0.2005425	0.0909009	up
A_19_P00322797	SEC14L1	1.0792012	1.0549893	1.2851782	-0.024212	0.205977	0.0908825	up
A_23_P122001	MSH3	1.6731634	1.7589965	1.7689672	0.0858331	0.0958037	0.0908184	up
A_23_P25706	CLMN	-0.581482	-0.44856	-0.532837	0.1329222	0.048645	0.0907836	up
A_33_P3224795	IKZF5	-0.874169	-0.751613	-0.81528	0.1225562	0.0588889	0.0907226	up
A_23_P345928	METTL25	-0.362031	-0.551527	0.0088835	-0.189496	0.3709145	0.0907095	up
A_22_P00000928	TMEM9B-AS1	-2.939367	-2.492364	-3.205088	0.4470031	-0.265721	0.090641	up
A_23_P3502	ZC3H18	1.829998	1.8313193	2.0098515	0.0013213	0.1798534	0.0905874	up
A_24_P310616	SLC35A2	3.3518944	3.4438205	3.4411182	0.0919261	0.0892239	0.090575	up
A_33_P3340565	PRSS53	-0.713537	-0.604344	-0.641715	0.1091929	0.0718222	0.0905075	up

A_21_P0014709	PAX8-AS1	2.8961067	2.883016	3.0901222	-0.013091	0.1940155	0.0904624	up
A_32_P50522	FKBP1A	3.765399	3.7602658	3.951456	-0.005133	0.1860571	0.090462	up
A_23_P307430	LSM12	3.0755415	2.9433808	3.3886127	-0.132161	0.3130713	0.0904553	up
A_23_P390384	ZNF630	0.0053444	0.231082	-0.039521	0.2257376	-0.044865	0.0904362	up
A_23_P165840	ODC1	7.5298147	7.876448	7.364012	0.3466334	-0.165803	0.0904152	up
A_22_P00008020	LOC101929089	-0.015059	0.1373963	0.0130033	0.1524553	0.0280623	0.0902588	up
A_33_P3267460	RFX3	-3.227645	-3.041203	-3.233687	0.1864421	-0.006042	0.0902002	up
A_33_P3272189	MFSD9	2.4669666	2.5296626	2.5846376	0.062696	0.117671	0.0901835	up
A_21_P0013338	XLOC_l2_013931	-2.736496	-2.808569	-2.484252	-0.072073	0.2522442	0.0900854	up
A_21_P0012658	XLOC_l2_011048	-2.375275	-2.432886	-2.138164	-0.057612	0.2371111	0.0897497	up
A_24_P98914	PFKM	5.326872	5.3009734	5.5322495	-0.025898	0.2053776	0.0897396	up
A_23_P92948	PRRC1	2.6532755	2.6394944	2.8464098	-0.013781	0.1931343	0.0896766	up
A_19_P00322898	CA5BP1	-2.592658	-2.69922	-2.306745	-0.106563	0.2859123	0.0896747	up
A_24_P322444	NUP62	6.8342743	6.890673	6.9572153	0.0563989	0.122941	0.0896699	up
A_23_P78289	FAM104A	4.4037447	4.4455256	4.5412607	0.041781	0.137516	0.0896485	up
A_23_P78944	AMH	-2.174652	-2.053652	-2.116474	0.1209998	0.0581775	0.0895886	up
A_32_P203515	SPECC1L	3.708705	3.735681	3.8609009	0.0269761	0.1521959	0.089586	up
A_23_P101351	ZNF426	2.5318842	2.6202683	2.6226597	0.0883842	0.0907755	0.0895798	up
A_23_P82674	GBAS	1.1148982	1.2382689	1.1706738	0.1233707	0.0557756	0.0895731	up
A_23_P433990	SPG7	3.2096891	3.3421645	3.2563305	0.1324754	0.0466414	0.0895584	up
A_24_P97825	CCDC69	-2.409924	-2.880372	-1.760482	-0.470448	0.6494422	0.089497	up
A_21_P0006346	lnc-PRSS3-1	-2.728799	-2.748047	-2.530579	-0.019248	0.1982207	0.0894864	up
A_22_P00015397	lnc-SRP54-1	-1.048515	-1.042743	-0.875446	0.0057716	0.173069	0.0894203	up
A_33_P3352019	SCARA3	-0.515467	-0.302193	-0.550267	0.2132735	-0.0348	0.0892367	up
A_21_P0011654	NPIPA2	3.7791986	4.006842	3.7299366	0.2276435	-0.049262	0.0891907	up
A_33_P3351052	POU3F1	1.0083051	1.0801396	1.1147766	0.0718346	0.1064715	0.0891531	up
A_23_P12147	C1orf74	0.2070518	0.2636428	0.3287292	0.056591	0.1216774	0.0891342	up
A_24_P163537	TMED4	2.6114302	2.6644092	2.7367182	0.052979	0.125288	0.0891335	up
A_23_P17204	ANAPC1	3.736761	3.734263	3.9175234	-0.002498	0.1807623	0.0891321	up
A_24_P88801	NPHP1	-0.40579	-0.309243	-0.324126	0.0965467	0.0816641	0.0891054	up
A_21_P0001273	lnc-LDLRAP1-1	-0.89903	-0.581462	-1.038403	0.3175678	-0.139372	0.0890977	up
A_22_P00024264	lnc-ADD1-1	0.3695226	0.5732579	0.3438616	0.2037354	-0.025661	0.0890372	up
A_23_P38446	TNFAIP1	5.1191587	5.228248	5.1880293	0.1090894	0.0688705	0.08898	up
A_22_P00003881	lnc-CENPP-2	-2.205409	-2.215596	-2.017317	-0.010187	0.1880922	0.0889527	up
A_24_P35228	GRHL2	1.7563992	2.0070662	1.6834526	0.2506671	-0.072947	0.0888603	up
A_24_P107859	SPRED1	0.7633891	0.8713145	0.8330979	0.1079254	0.0697088	0.0888171	up
A_23_P396777	PCGF3	-1.539712	-1.403932	-1.497975	0.1357799	0.0417361	0.088758	up
A_33_P3359115	LMBRD1	3.8644028	3.9149995	3.9913025	0.0505967	0.1268997	0.0887482	up
A_23_P309361	HENMT1	4.2406254	4.4941945	4.1645393	0.2535691	-0.076086	0.0887415	up
A_33_P3215178	PIGQ	-0.210572	-0.047843	-0.195838	0.1627288	0.0147333	0.0887311	up
A_24_P23921	C17orf98	-0.953863	-0.785519	-0.944819	0.168344	0.0090442	0.0886941	up
A_22_P00012113	lnc-POLD3-2	-1.387297	-1.35514	-1.242097	0.0321569	0.1451998	0.0886784	up
A_23_P82334	SLC25A13	2.948347	3.127151	2.9468546	0.1788039	-0.001493	0.0886557	up
A_33_P3410836	HIST1H4D	4.7545652	4.755506	4.9309015	0.0009408	0.1763363	0.0886385	up
A_23_P16110	OR7E24	1.062612	1.2832928	1.0191393	0.2206807	-0.043473	0.088604	up
A_23_P390443	SHISA7	-2.843755	-2.532508	-2.977807	0.3112474	-0.134052	0.0885978	up
A_23_P89621	CBX4	-0.167997	0.2854986	-0.444318	0.4534955	-0.276321	0.0885871	up
A_23_P120566	RRBP1	3.5113935	3.531324	3.6686	0.0199304	0.1572065	0.0885685	up
A_22_P00005925	HOXA-AS3	-2.583553	-2.346695	-2.643332	0.2368586	-0.059779	0.0885398	up
A_24_P122337	SYTL4	-1.582202	-1.391433	-1.595943	0.1907687	-0.013741	0.0885139	up
A_22_P00024555	NSUN3	-3.360677	-3.12052	-3.423854	0.2401564	-0.063177	0.0884895	up
A_23_P40108	COL9A3	5.7716455	5.9274983	5.792714	0.1558528	0.0210686	0.0884607	up
A_24_P286898	MAPK8	-0.43339	-1.459151	0.7692738	-1.025761	1.2026639	0.0884514	up
A_23_P8432	COPS6	5.964082	6.0585284	6.046386	0.0944467	0.082304	0.0883753	up
A_33_P3289034	PKD2L1	-2.251999	-2.244676	-2.08258	0.0073228	0.1694193	0.088371	up
A_33_P3380682	TBPL1	0.8653088	0.9496355	0.9576478	0.0843267	0.092339	0.0883329	up

A_21_P0013896	TTTY14	-0.723159	-0.572305	-0.697391	0.1508546	0.0257688	0.0883117	up
A_22_P00019616	lnc-KBTBD5-1	-0.421712	-0.385518	-0.281288	0.0361943	0.1404243	0.0883093	up
A_23_P71148	BLVRA	5.293153	5.2051225	5.557741	-0.08803	0.2645884	0.088279	up
A_23_P102420	CCT4	8.561961	8.610346	8.689985	0.0483847	0.1280241	0.0882044	up
A_33_P3645888	SNORA71C	-1.483328	-1.390464	-1.400061	0.092864	0.0832672	0.0880656	up
A_21_P0004360	LOC100505625	-2.166963	-1.783028	-2.374783	0.383935	-0.20782	0.0880574	up
A_32_P69368	ID2	3.0639668	3.2020497	3.1018028	0.138083	0.0378361	0.0879595	up
A_23_P78771	NUCB1	1.0273237	0.940012	1.2904053	-0.087312	0.2630816	0.0878849	up
A_24_P675386	C11orf58	0.0135326	0.2442498	-0.041599	0.2307172	-0.055131	0.0877929	up
A_22_P00001477	JRK	-2.613107	-2.325023	-2.725617	0.288084	-0.11251	0.0877868	up
A_33_P3302892	NID2	-2.940045	-2.8568	-2.847734	0.0832443	0.0923104	0.0877774	up
A_23_P347508	COA7	3.5533323	3.5175986	3.7644167	-0.035734	0.2110844	0.0876753	up
A_33_P3502315	CRLS1	4.6183195	4.609884	4.802046	-0.008436	0.1837263	0.0876453	up
A_33_P3249259	TGM6	-2.548117	-2.178916	-2.742209	0.3692012	-0.194092	0.0875546	up
A_24_P305678	PITPNB	4.7766333	4.755567	4.972369	-0.021066	0.1957359	0.0873349	up
A_33_P3263696	ATG13	0.1308403	0.2941427	0.1420388	0.1633024	0.0111985	0.0872505	up
A_33_P3329477	RPL13P5	1.4393778	1.5433049	1.5099206	0.1039271	0.0705428	0.087235	up
A_21_P0014210	LOC101927172	-1.627652	-1.319024	-1.761832	0.3086281	-0.13418	0.087224	up
A_33_P3379456	SH3YL1	4.826255	4.8731112	4.9537134	0.0468564	0.1274586	0.0871575	up
A_23_P259012	BAP1	1.22049	1.4086246	1.2066255	0.1881347	-0.013865	0.0871351	up
A_21_P0014823	LOC100287160	-2.555695	-2.025988	-2.911174	0.529707	-0.355479	0.087114	up
A_33_P3410935	C17orf89	6.827757	6.871162	6.9585533	0.0434051	0.1307964	0.0871007	up
A_23_P143958	RPL22L1	9.337799	9.5732975	9.276445	0.2354984	-0.061354	0.0870724	up
A_23_P8339	MRPL18	6.2593565	6.3082223	6.384571	0.0488658	0.1252146	0.0870402	up
A_33_P3403748	UQCC3	6.371646	6.302422	6.6149406	-0.069224	0.2432947	0.0870354	up
A_33_P3571120	AP3D1	3.0381851	3.334292	2.9160433	0.2961068	-0.122142	0.0869825	up
A_22_P00009983	LOC101928782	-2.316002	-2.130892	-2.32716	0.1851103	-0.011157	0.0869765	up
A_21_P0007382	lnc-GPR83-2	1.1864567	1.4727492	1.0740271	0.2862926	-0.11243	0.0869315	up
A_23_P251705	ARHGEF9	-0.671519	-0.349532	-0.819669	0.3219867	-0.14815	0.0869184	up
A_23_P311616	JMJD6	1.8684015	2.044232	1.866395	0.1758304	-0.002007	0.0869119	up
A_24_P743802	ZNF618	2.6958313	2.7110152	2.854412	0.0151839	0.1585808	0.0868824	up
A_22_P00023942	SLC2A1-AS1	-2.345605	-2.281947	-2.235544	0.063658	0.1100614	0.0868597	up
A_21_P0001544	lnc-BARHL2-1	-2.512087	-2.141119	-2.709441	0.3709688	-0.197354	0.0868075	up
A_33_P3341365	RNF216	2.9351397	2.974935	3.0688887	0.0397954	0.133749	0.0867722	up
A_24_P72750	SPIN1	3.6138067	3.8003478	3.6008005	0.1865411	-0.013006	0.0867674	up
A_32_P141724	COMMD7	1.3960166	1.5507388	1.4144969	0.1547222	0.0184803	0.0866013	up
A_33_P3229380	LINC01347	1.2415233	1.4472361	1.2082615	0.2057128	-0.033262	0.0862255	up
A_33_P3342653	ADAM33	-1.824047	-1.747315	-1.728459	0.0767322	0.0955873	0.0861597	up
A_23_P306215	FAM84A	0.3404813	0.0325785	0.8206959	-0.307903	0.4802146	0.0861559	up
A_23_P141893	PPM1N	2.0027962	2.0676541	2.1102028	0.064858	0.1074066	0.0861323	up
A_23_P57819	TATDN2	3.3289194	3.2769847	3.5531168	-0.051935	0.2241974	0.0861313	up
A_23_P256542	FAM162A	5.8787003	6.004414	5.924862	0.1257138	0.0461617	0.0859377	up
A_23_P321349	SAAL1	1.0157266	1.0958595	1.1073055	0.080133	0.091579	0.085856	up
A_22_P00006098	lnc-FAM164C-1	-1.807879	-1.32723	-2.116944	0.4806485	-0.309065	0.0857916	up
A_33_P3309231	HSCB	8.755875	8.923622	8.759632	0.1677475	0.0037575	0.0857525	up
A_22_P00024912	LOC102723366	-1.967833	-2.104008	-1.660374	-0.136175	0.3074594	0.0856423	up
A_33_P3259740	LOC400661	-2.549099	-2.292152	-2.634796	0.2569461	-0.085697	0.0856243	up
A_21_P0003137	MTRNR2L2	8.625964	8.746815	8.676338	0.1208506	0.050374	0.0856123	up
A_33_P3365047	RBBP9	-1.413937	-1.435558	-1.221149	-0.021621	0.1927881	0.0855834	up
A_33_P3521643	PPP1R12C	5.395052	5.4783235	5.482793	0.0832715	0.0877409	0.0855062	up
A_33_P3265965	CFAP97	-0.34213	-0.35447	-0.158862	-0.01234	0.1832681	0.0854642	up
A_23_P206293	GPR114	-0.538076	-0.651347	-0.253947	-0.113271	0.2841291	0.085429	up
A_23_P347432	DVL1	1.5291162	1.7841129	1.4449415	0.2549968	-0.084175	0.0854111	up
A_22_P00002441	LOC102723354	3.1806211	3.1868339	3.3452015	0.0062127	0.1645804	0.0853965	up
A_23_P134953	PLIN2	1.0237508	1.2497754	0.9684119	0.2260246	-0.055339	0.0853429	up
A_23_P210948	UQCC1	-0.032938	-0.167717	0.2724214	-0.134779	0.3053589	0.08529	up

A_21_P0006369	LOC100288842	-2.271067	-2.278013	-2.093562	-0.006946	0.177505	0.0852795	up
A_23_P127948	ADM	2.6905842	3.175507	2.376011	0.4849229	-0.314573	0.0851748	up
A_21_P0006384	lnc-DIRAS2-1	-2.646865	-2.431843	-2.691585	0.2150214	-0.044721	0.0851504	up
A_33_P3289236	HPR	-2.724519	-2.184192	-3.094593	0.5403268	-0.370074	0.0851265	up
A_22_P00011635	MIR143HG	-2.109269	-2.066383	-1.981967	0.0428863	0.1273027	0.0850945	up
A_23_P3775	OGFOD1	3.8853617	3.9713712	3.9694805	0.0860095	0.0841188	0.0850642	up
A_32_P489130	BRWD3	-1.171937	-0.855506	-1.318346	0.3164301	-0.14641	0.0850103	up
A_24_P307014	BRD2	5.4995184	5.5895047	5.5793285	0.0899863	0.0798101	0.0848982	up
A_24_P636318	FAM168B	2.3156233	2.40523	2.3957663	0.0896068	0.080143	0.0848749	up
A_22_P00015329	lnc-SPPL3.1-3	0.627646	-0.029502	1.4543548	-0.657148	0.8267088	0.0847805	up
A_24_P116242	KLHDC2	3.0331469	3.3008122	2.9349794	0.2676654	-0.098167	0.084749	up
A_21_P0006695	lnc-RPP30-6	-2.857919	-2.746873	-2.799531	0.1110461	0.0583875	0.0847168	up
A_33_P3415923	FMNL3	-0.492297	-0.518901	-0.296366	-0.026604	0.1959314	0.0846639	up
A_23_P32036	NMRK1	2.3160753	2.3792634	2.4221048	0.0631881	0.1060295	0.0846088	up
A_33_P3361442	C19orf73	-1.041465	-1.110984	-0.802764	-0.06952	0.2387004	0.0845904	up
A_23_P17307	MRGBP	3.4276295	3.453507	3.57088	0.0258775	0.1432505	0.084564	up
A_33_P3257150	CDC42	3.2853575	3.4464793	3.2933264	0.1611219	0.0079689	0.0845454	up
A_24_P382630	MTG2	1.2491984	1.0184579	1.6489534	-0.230741	0.399755	0.0845072	up
A_33_P3316928	PELI1	2.625041	2.647574	2.7715006	0.0225329	0.1464596	0.0844963	up
A_23_P125265	KPNA2	7.8775005	7.9206367	8.003339	0.0431361	0.1258383	0.0844872	up
A_24_P344295	RNF167	0.1877809	0.3629651	0.1815591	0.1751843	-0.006222	0.0844812	up
A_24_P115700	COX15	-0.972386	-0.94802	-0.827874	0.0243659	0.1445117	0.0844388	up
A_33_P3255914	MYLIP	2.2553673	2.3980813	2.281394	0.142714	0.0260267	0.0843704	up
A_32_P191084	KCTD5	3.1595335	3.1032329	3.3844585	-0.056301	0.224925	0.0843122	up
A_24_P126471	SLC12A9	-1.46994	-1.36189	-1.409376	0.1080504	0.0605645	0.0843074	up
A_23_P122532	C6orf47	0.1581159	0.1284499	0.356307	-0.029666	0.1981912	0.0842626	up
A_32_P703	LOC646626	-0.110974	0.0701194	-0.123549	0.1810932	-0.012575	0.0842593	up
A_33_P3419419	PIK3C2B	-2.338795	-2.400605	-2.108634	-0.06181	0.2301612	0.0841756	up
A_24_P329924	SIK3	1.3113575	1.4542384	1.3367028	0.1428809	0.0253453	0.0841131	up
A_32_P168349	C6orf25	-2.050401	-2.142012	-1.790581	-0.091611	0.25982	0.0841045	up
A_33_P3347040	DPP9-AS1	4.452531	4.7012553	4.371913	0.2487245	-0.080618	0.0840533	up
A_22_P00016479	lnc-TMEM86A-2	1.0924606	1.1360092	1.216825	0.0435486	0.1243644	0.0839565	up
A_23_P31055	NUP43	4.916836	4.998283	5.0031815	0.0814471	0.0863457	0.0838964	up
A_33_P3290343	CYP1B1	3.1961594	3.3356633	3.2243853	0.139504	0.0282259	0.0838649	up
A_24_P19828	TOE1	1.8476372	2.0251021	1.8378572	0.177465	-0.00978	0.0838425	up
A_33_P3242014	PHC1	0.7290425	0.7037416	0.9219189	-0.025301	0.1928763	0.0837877	up
A_23_P116264	NRGN	0.6668577	0.5571494	0.9440813	-0.109708	0.2772236	0.0837576	up
A_24_P911676	SOX4	1.3449106	1.4612436	1.395823	0.116333	0.0509124	0.0836227	up
A_32_P34920	FOXD1	2.8804064	3.0868125	2.8412323	0.2064061	-0.039174	0.083616	up
A_23_P151436	ALG5	6.1454782	6.187941	6.2702074	0.0424628	0.1247292	0.083596	up
A_22_P00007528	LINC00577	-0.163754	-0.121901	-0.038467	0.0418534	0.1252875	0.0835705	up
A_33_P3219469	SCAF11	3.2417974	3.3118072	3.3389168	0.0700097	0.0971193	0.0835645	up
A_23_P34546	SDCCAG8	-0.941856	-0.811141	-0.905451	0.1307154	0.0364046	0.08356	up
A_33_P3266520	SPPL2B	-1.288011	-1.062625	-1.346346	0.2253866	-0.058335	0.0835259	up
A_23_P94703	TOR1B	1.3749213	1.5070367	1.4096656	0.1321154	0.0347443	0.0834298	up
A_24_P466374	EYA3	0.6777821	0.6550932	0.86729	-0.022689	0.189508	0.0834095	up
A_24_P252575	RABGAP1	2.8371773	2.902267	2.9389048	0.0650897	0.1017275	0.0834086	up
A_23_P46725	EPC1	2.2689867	2.3351989	2.3695202	0.0662122	0.1005335	0.0833728	up
A_24_P381029	GLRX3	5.962061	6.0485244	6.0423317	0.0864635	0.0802708	0.0833671	up
A_23_P9823	MLXIP	3.6053772	3.560079	3.8173647	-0.045298	0.2119875	0.0833447	up
A_24_P192727	KAZALD1	3.5892773	3.458857	3.8862562	-0.13042	0.296979	0.0832794	up
A_33_P3531828	LARS	3.8483448	4.078132	3.7849398	0.2297874	-0.063405	0.0831912	up
A_21_P0000694	NIFK-AS1	1.4534698	1.4798956	1.5932198	0.0264258	0.13975	0.0830879	up
A_24_P82419	H3F3C	7.4508867	7.5030456	7.5647955	0.0521588	0.1139088	0.0830338	up
A_24_P168574	GNAS	1.0040841	1.2773805	0.8968477	0.2732964	-0.107236	0.08303	up
A_33_P3385656	FNDC9	-1.672555	-1.391203	-1.787861	0.2813511	-0.115307	0.0830221	up

A_33_P3299898	CTSLP2	0.9024291	0.8734703	1.0973334	-0.028959	0.1949043	0.0829728	up
A_32_P103633	MCM2	4.8449183	5.148048	4.7075815	0.3031297	-0.137337	0.0828965	up
A_23_P142174	FOXA3	-0.422601	-0.54277	-0.136663	-0.120169	0.2859378	0.0828843	up
A_23_P3514	CFAP20	4.2841635	4.2645173	4.469537	-0.019646	0.1853733	0.0828636	up
A_23_P383132	HIC2	3.439312	3.5042062	3.540083	0.0648942	0.100771	0.0828326	up
A_23_P131526	C2orf47	4.124976	4.238874	4.1764927	0.1138978	0.0515165	0.0827072	up
A_33_P3411991	RABGAP1	0.9155531	1.0966673	0.8996325	0.1811142	-0.015921	0.0825968	up
A_23_P1072	ATP1A1	6.2888184	6.4402986	6.302492	0.1514802	0.0136738	0.082577	up
A_33_P3210965	TCTN1	2.9165344	3.0949025	2.9032125	0.1783681	-0.013322	0.0825231	up
A_23_P83917	KDM2A	2.125719	2.2154737	2.2009306	0.0897546	0.0752115	0.0824831	up
A_23_P147277	TSEN54	4.328923	4.301022	4.521576	-0.027901	0.1926527	0.0823758	up
A_23_P168847	FBXO16	0.0789132	0.0922647	0.2301536	0.0133514	0.1512404	0.0822959	up
A_22_P00007317	LINC01146	-0.790271	-0.609397	-0.806563	0.1808739	-0.016292	0.0822909	up
A_23_P76622	DCT	-1.633848	-1.48397	-1.619288	0.149878	0.0145593	0.0822186	up
A_33_P3313846	ZDHHC8	-2.749978	-2.558565	-2.776972	0.1914127	-0.026994	0.0822092	up
A_21_P0009073	lnc-MPHOSPH6-2	0.6706138	0.8082805	0.6973624	0.1376667	0.0267487	0.0822077	up
A_24_P21752	TBRG1	0.252974	0.5670943	0.1031995	0.3141203	-0.149775	0.0821729	up
A_23_P40194	DDX27	5.610134	5.6994834	5.684885	0.0893493	0.0747509	0.0820501	up
A_24_P18802	VPS18	2.6535816	2.7175612	2.7536602	0.0639796	0.1000786	0.0820291	up
A_23_P170774	E2F6	3.8029947	3.8298135	3.9401674	0.0268188	0.1371727	0.0819957	up
A_23_P381203	KIAA0556	-0.997666	-0.768093	-1.063362	0.2295737	-0.065695	0.0819392	up
A_22_P00003780	lnc-CDK2AP1-1	-0.319883	-0.186537	-0.289663	0.1333456	0.0302196	0.0817826	up
A_23_P133902	PSORS1C1	-1.761921	-1.151807	-2.20854	0.6101136	-0.446619	0.0817474	up
A_22_P00017050	lnc-TUBA1C-1	-0.689671	-0.590369	-0.625633	0.0993018	0.0640373	0.0816696	up
A_23_P47857	TM7SF3	5.0422764	5.1316113	5.116132	0.089335	0.0738554	0.0815952	up
A_23_P164559	CHAMP1	1.904479	2.2102494	1.7618814	0.3057704	-0.142598	0.0815864	up
A_23_P146497	PPP1R26	2.6291924	2.8661666	2.5553522	0.2369742	-0.07384	0.0815671	up
A_23_P131365	NDUFS1	5.8432646	5.9289165	5.9206924	0.0856519	0.0774279	0.0815399	up
A_23_P77813	FN3KRP	4.2627134	4.429671	4.258769	0.1669574	-0.003944	0.0815065	up
A_33_P3267280	CTDSP2	0.0250921	0.4191079	-0.205953	0.3940158	-0.231045	0.0814855	up
A_33_P3324860	ETV3	-2.357064	-2.198243	-2.353053	0.1588204	0.0040104	0.0814154	up
A_24_P409494	BCL2L13	0.856421	0.8793902	0.9960847	0.0229692	0.1396637	0.0813165	up
A_24_P169343	AAED1	0.2380619	0.5103412	0.1284037	0.2722793	-0.109658	0.0813105	up
A_33_P3577142	lnc-ANKRD11-5	0.6604347	0.6059189	0.8774476	-0.054516	0.2170129	0.0812485	up
A_23_P47058	CUZD1	-1.241238	-1.140435	-1.179544	0.1008029	0.0616937	0.0812483	up
A_23_P104734	STT3A	3.1322165	3.2816358	3.1452618	0.1494193	0.0130453	0.0812323	up
A_33_P3227345	FKBP1A	0.8103905	0.7511625	1.0320082	-0.059228	0.2216177	0.0811949	up
A_22_P00009075	lnc-LETM2-1	-0.564724	-0.504487	-0.462677	0.0602374	0.1020474	0.0811424	up
A_23_P325438	XPO6	2.0712175	2.0813632	2.2233486	0.0101457	0.1521311	0.0811384	up
A_22_P00010879	MAFG-AS1	-1.699854	-1.488294	-1.749196	0.2115598	-0.049342	0.081109	up
A_32_P206698	CKS1B	4.002248	4.072387	4.0942717	0.0701394	0.0920239	0.0810816	up
A_22_P00024077	LOC102724115	-2.663763	-2.357625	-2.8078	0.3061378	-0.144037	0.0810504	up
A_23_P117797	CLN6	2.6974878	2.5576978	2.9993086	-0.13979	0.3018208	0.0810154	up
A_19_P00808208	CDCA7L	1.2366695	1.3874149	1.2479153	0.1507454	0.0112457	0.0809956	up
A_24_P303974	CDC123	4.612834	4.714442	4.6729736	0.1016078	0.0601397	0.0808737	up
A_33_P3289204	AAK1	-0.527856	-0.330135	-0.563871	0.197721	-0.036015	0.0808532	up
A_22_P00025224	MED6	2.1821566	2.1755672	2.3503857	-0.006589	0.1682291	0.0808198	up
A_21_P0013198	PSPHP1	4.94298	5.051227	4.9963684	0.1082473	0.0533886	0.0808179	up
A_21_P0000641	LINC00959	-1.522806	-1.382857	-1.501199	0.1399488	0.0216069	0.0807779	up
A_23_P203947	DDX11	1.9651594	1.9829793	2.1086187	0.0178199	0.1434593	0.0806396	up
A_23_P553	TARS2	2.7550278	2.7727246	2.898552	0.0176969	0.1435242	0.0806105	up
A_22_P00016609	lnc-TOLLIP-1	-1.537786	-1.411708	-1.502852	0.1260777	0.0349336	0.0805056	up
A_23_P230	TTC4	0.8001008	0.8269372	0.9342308	0.0268364	0.13413	0.0804832	up
A_23_P72697	GPIHBP1	-2.500757	-2.280284	-2.560404	0.2204731	-0.059646	0.0804133	up
A_24_P371758	MRPL36	5.533725	5.5700746	5.65812	0.0363498	0.1243954	0.0803726	up
A_23_P4572	MYL12A	7.316845	7.394279	7.4001513	0.0774341	0.0833063	0.0803702	up

A_24_P72646	BIN3-IT1	-2.396352	-2.372084	-2.259921	0.0242682	0.1364317	0.0803499	up
A_22_P00003816	HID1-AS1	-2.222813	-2.109855	-2.175117	0.112958	0.0476961	0.080327	up
A_22_P00000379	LOC101929408	-0.076025	0.0087304	-0.000183	0.0847559	0.0758429	0.0802994	up
A_33_P3338559	RBBP4	4.8476505	5.012154	4.843706	0.1645036	-0.003944	0.0802796	up
A_22_P00013823	MID1IP1-AS1	-1.421012	-1.300038	-1.381465	0.1209736	0.039547	0.0802603	up
A_23_P372874	S100A13	6.562193	6.6030507	6.6818104	0.0408578	0.1196175	0.0802376	up
A_32_P117422	PMS2P4	2.5088043	2.6230426	2.554987	0.1142383	0.0461826	0.0802104	up
A_23_P75921	TRAF6	-0.058915	0.0151339	0.0273447	0.0740485	0.0862594	0.0801539	up
A_22_P00012285	TCERG1L-AS1	-0.277815	-0.210862	-0.184543	0.0669532	0.0932722	0.0801127	up
A_23_P126727	NOL9	0.2857957	0.4831605	0.2486196	0.1973648	-0.037176	0.0800943	up
A_32_P44453	INPP1	1.7922735	1.7445521	2.0000591	-0.047721	0.2077856	0.0800321	up
A_23_P135499	CLIC4	0.3786836	0.9321032	-0.014712	0.5534196	-0.393396	0.0800119	up
A_23_P161446	GDI2	4.049898	4.1654096	4.0943775	0.1155114	0.0444794	0.0799954	up
A_33_P3257538	HYKK	-0.930452	-0.863091	-0.837833	0.0673609	0.0926189	0.0799899	up
A_33_P3317752	KRTAP4-7	-1.772947	-1.565385	-1.820581	0.207562	-0.047634	0.0799642	up
A_24_P48177	ST3GAL2	0.2561398	0.4884453	0.1837525	0.2323055	-0.072387	0.0799592	up
A_22_P00010175	FGD5-AS1	1.1842623	1.1119876	1.4163938	-0.072275	0.2321315	0.0799284	up
A_24_P334130	FN1	-2.358399	-2.323269	-2.233736	0.0351303	0.1246631	0.0798967	up
A_22_P00011611	lnc-PCM1-4	-0.974992	-0.752515	-1.037718	0.222477	-0.062726	0.0798755	up
A_23_P378288	IKZF4	-0.162656	0.1507635	-0.316372	0.3134198	-0.153716	0.0798519	up
A_23_P389525	COMMD1	4.2150993	4.352551	4.237219	0.1374517	0.0221195	0.0797856	up
A_23_P151133	TSPAN9	-1.43882	-1.44857	-1.269512	-0.009749	0.1693082	0.0797794	up
A_33_P3423949	CBX2	4.4633284	4.4373765	4.648758	-0.025952	0.1854296	0.0797389	up
A_23_P120153	RNF149	5.0904827	5.255754	5.084567	0.1652713	-0.005916	0.0796778	up
A_33_P3220381	TRAPPC13	-0.388875	-0.095053	-0.523399	0.2938223	-0.134524	0.0796492	up
A_23_P87049	SORL1	5.9526577	6.1838746	5.8806314	0.2312169	-0.072026	0.0795953	up
A_19_P00330076	lnc-SIK1-5	-1.263314	-1.281051	-1.086503	-0.017737	0.1768112	0.0795372	up
A_21_P0010633	LINC01057	-2.309322	-2.168832	-2.290796	0.1404901	0.0185263	0.0795082	up
A_23_P151297	TNS2	-1.380236	-1.046713	-1.554781	0.3335223	-0.174545	0.0794888	up
A_24_P93855	FOXP4	-0.498508	-0.452317	-0.38612	0.0461917	0.1123881	0.0792899	up
A_23_P58877	GOPC	1.8297882	1.8810787	1.936882	0.0512905	0.1070938	0.0791922	up
A_22_P00021552	lnc-CGN-1	-1.542603	-1.299209	-1.627669	0.2433939	-0.085066	0.0791638	up
A_23_P119789	TMEM185B	1.4691968	1.5012846	1.5954185	0.0320878	0.1262217	0.0791547	up
A_33_P3289045	GSTT2B	-1.940265	-1.716861	-2.005475	0.2234039	-0.065209	0.0790973	up
A_24_P386334	SH3GLB2	2.0243664	2.1642642	2.0426598	0.1398978	0.0182934	0.0790956	up
A_24_P323072	GNRH2	2.5244122	2.44025	2.7667274	-0.084162	0.2423153	0.0790765	up
A_24_P311771	ZFR	1.5671377	1.6720338	1.6203861	0.1048961	0.0532484	0.0790722	up
A_21_P0005152	lnc-GMDS-3	-2.514778	-2.561905	-2.309531	-0.047127	0.2052469	0.0790597	up
A_23_P364890	RNF40	1.4893522	1.8086891	1.3280859	0.3193369	-0.161266	0.0790353	up
A_33_P3217347	PJA1	4.074936	4.104469	4.2034597	0.0295329	0.1285238	0.0790284	up
A_33_P3243168	MZF1	3.9610777	4.2056956	3.8745117	0.2446179	-0.086566	0.079026	up
A_23_P22765	NDUFB11	6.5016937	6.1478066	7.0135965	-0.353887	0.5119028	0.0790079	up
A_23_P330262	PIP5K1C	-0.235333	-0.230561	-0.082096	0.0047722	0.1532369	0.0790045	up
A_23_P162142	TSKU	3.4254894	3.4593244	3.5496044	0.0338349	0.124115	0.078975	up
A_33_P3424295	TRAF2	-0.120274	-0.016317	-0.066402	0.1039567	0.0538712	0.0789139	up
A_23_P106859	EMC8	5.758338	5.764583	5.909896	0.0062451	0.1515579	0.0789015	up
A_33_P3376821	GZMA	-2.029345	-2.155435	-1.745631	-0.126091	0.2837133	0.0788114	up
A_22_P00022395	RNF24	1.1974087	1.3662419	1.1860952	0.1688333	-0.011313	0.0787599	up
A_33_P3502914	ZDHHC8P1	-3.128653	-3.21986	-2.879951	-0.091207	0.2487021	0.0787475	up
A_33_P3712341	CXCL12	-2.140942	-2.211458	-1.913087	-0.070516	0.2278547	0.0786696	up
A_23_P106737	LUC7L	2.7586803	2.784985	2.889615	0.0263047	0.1309347	0.0786197	up
A_21_P0006496	GAGE2B	-0.00141	0.0725966	0.0818234	0.0740061	0.0832329	0.0786195	up
A_23_P69242	SUMF1	4.960539	5.2066283	4.871682	0.2460895	-0.088857	0.0786164	up
A_23_P21033	GMPS	6.458976	6.519359	6.5557604	0.0603833	0.0967846	0.078584	up
A_23_P144453	MRFAP1	5.1306114	5.19459	5.2237244	0.0639787	0.0931129	0.0785458	up
A_22_P00012577	STAM-AS1	-3.276517	-3.192534	-3.203564	0.0839829	0.0729532	0.0784681	up

A_21_P0005198	lnc-WBSCR16-1	-1.729428	-1.662489	-1.639575	0.0669384	0.0898528	0.0783956	up
A_22_P00021083	LINC00644	-0.067232	0.1075244	-0.085281	0.1747561	-0.01805	0.0783532	up
A_32_P78816	PSPH	5.7728806	6.0347342	5.6676683	0.2618537	-0.105212	0.0783207	up
A_23_P303203	CHMP7	-0.483758	-0.396289	-0.414629	0.0874691	0.069129	0.078299	up
A_24_P75230	MYL12B	7.2078867	7.242973	7.3293734	0.0350862	0.1214867	0.0782864	up
A_23_P92994	MFF	3.181034	3.2109694	3.307145	0.0299354	0.126111	0.0780232	up
A_23_P251916	RMDN1	4.6948433	4.7701955	4.7755013	0.0753522	0.080658	0.0780051	up
A_22_P00011858	lnc-PHYHIP-2	-2.53951	-2.213718	-2.709351	0.3257916	-0.169841	0.0779755	up
A_24_P365807	EFNB1	6.0495596	6.1154847	6.139471	0.0659251	0.0899115	0.0779183	up
A_23_P60339	C9orf64	0.0196552	0.0428538	0.1520734	0.0231986	0.1324182	0.0778084	up
A_23_P218375	ITGAE	1.7725363	1.6679668	2.0325327	-0.104569	0.2599964	0.0777135	up
A_33_P3369461	AMIGO1	1.5684872	1.3177028	1.9745913	-0.250784	0.4061041	0.0776599	up
A_24_P413988	TGOLN2	4.4552183	4.6531157	4.4126015	0.1978974	-0.042617	0.0776403	up
A_21_P0000306	SNORA2A	-0.129079	-0.16084	0.0578098	-0.03176	0.1868892	0.0775645	up
A_23_P28878	C20orf27	3.116231	3.1125598	3.2749596	-0.003671	0.1587286	0.0775287	up
A_19_P00800667	LINC00398	-1.922731	-1.822184	-1.868224	0.1005478	0.0545073	0.0775275	up
A_23_P60534	TOR2A	2.2901268	2.2799745	2.4552593	-0.010152	0.1651325	0.0774901	up
A_23_P387071	ZNF830	2.3083467	2.4090743	2.3625736	0.1007276	0.0542269	0.0774772	up
A_23_P320113	SRXN1	5.031945	5.1618276	5.0569744	0.1298823	0.0250292	0.0774558	up
A_23_P67971	GALM	0.612226	0.5740976	0.8052049	-0.038128	0.1929789	0.0774252	up
A_33_P3531206	C1QTNF9B-AS1	0.4781652	0.3072205	0.8039522	-0.170945	0.3257871	0.0774212	up
A_23_P102842	NFS1	3.2458038	3.0376153	3.6088085	-0.208189	0.3630047	0.0774081	up
A_33_P3226643	ATG9A	-0.082127	-0.18216	0.1726742	-0.100033	0.2548008	0.0773838	up
A_22_P00001621	SGMS1-AS1	-1.162056	-0.964778	-1.204588	0.197278	-0.042531	0.0773735	up
A_23_P79927	NOP56	4.1334066	4.244189	4.1773148	0.1107822	0.0439081	0.0773451	up
A_23_P153251	ZNF274	-0.183936	0.0400829	-0.253287	0.2240186	-0.069351	0.0773337	up
A_22_P00016718	lnc-TRAPPC12-3	1.7821712	1.8974814	1.8214893	0.1153102	0.0393181	0.0773141	up
A_33_P3317123	SEC24C	5.6594563	5.243048	6.2304344	-0.416408	0.5709782	0.0772851	up
A_33_P3273290	ZNF28	0.4931445	0.3280335	0.8127446	-0.165111	0.3196001	0.0772445	up
A_21_P0000784	TMEM147-AS1	-0.961207	-0.822251	-0.945678	0.1389561	0.0155292	0.0772426	up
A_22_P00016776	CTC-338M12.4	2.263402	2.3722596	2.3090115	0.1088576	0.0456095	0.0772336	up
A_33_P3271925	ARFGEF2	1.7945738	2.097179	1.6464257	0.3026052	-0.148148	0.0772285	up
A_24_P942354	PITPNA	4.3314247	4.29917	4.5180016	-0.032255	0.1865768	0.0771611	up
A_33_P3407780	PPM1F	5.0740767	5.024776	5.2773457	-0.049301	0.203269	0.0769842	up
A_33_P3356926	APBB3	-2.758148	-2.510367	-2.852001	0.2477808	-0.093853	0.0769641	up
A_32_P155364	RPL7	9.033441	9.170292	9.050472	0.1368513	0.0170317	0.0769415	up
A_22_P00004149	LOC101928035	-1.133687	-0.990803	-1.1227	0.1428843	0.0109873	0.0769358	up
A_24_P753454	NDUFA6-AS1	-1.856469	-1.390924	-2.168171	0.4655447	-0.311703	0.076921	up
A_33_P3888365	RSBN1	1.0056238	1.1465354	1.0185275	0.1409116	0.0129037	0.0769076	up
A_23_P109928	PSMD6	3.3306122	3.3224893	3.4924345	-0.008123	0.1618223	0.0768497	up
A_23_P143484	PIGP	2.4219751	2.45365	2.543912	0.0316749	0.1219368	0.0768058	up
A_33_P3376404	WDR13	-1.066912	-0.844375	-1.135885	0.222537	-0.068973	0.0767822	up
A_33_P3256532	RBM48	-0.570934	-0.50303	-0.485432	0.0679045	0.0855026	0.0767035	up
A_33_P3370959	BTN1A1	-2.869645	-2.807489	-2.778661	0.0621555	0.0909841	0.0765698	up
A_33_P3280666	PHF21B	0.3873749	0.6221767	0.3056841	0.2348018	-0.081691	0.0765555	up
A_23_P429020	HKR1	0.1628146	0.2255592	0.2530747	0.0627446	0.09026	0.0765023	up
A_22_P00023170	LOC100506085	-2.573421	-2.652846	-2.340994	-0.079425	0.2324271	0.076501	up
A_22_P00010813	SMG7-AS1	-2.289937	-2.279281	-2.147717	0.0106556	0.14222	0.0764378	up
A_22_P00015940	ENTPD1-AS1	-1.483263	-1.263297	-1.550486	0.2199659	-0.067224	0.0763712	up
A_23_P255869	PLGRKT	2.3106718	2.3535905	2.420494	0.0429187	0.1098223	0.0763705	up
A_21_P0000087	MCPH1	1.2076983	1.1744485	1.393568	-0.03325	0.1858697	0.0763099	up
A_23_P93988	ARHGEF5	2.1587467	1.9955392	2.474535	-0.163208	0.3157883	0.0762904	up
A_33_P3385870	CCNG2	-2.662023	-2.464439	-2.707099	0.1975834	-0.045076	0.0762535	up
A_24_P911094	RABL2A	-2.419285	-2.445991	-2.240076	-0.026706	0.1792097	0.0762519	up
A_24_P346368	TMEM248	4.1159716	4.0905576	4.2934856	-0.025414	0.1775141	0.07605	up
A_24_P469641	RNF216	1.548625	1.4749193	1.774282	-0.073706	0.225657	0.0759757	up

A_33_P3253653	GPR155	10.206353	10.358158	10.206392	0.1518049	3.91E-05	0.075922	up
A_24_P337334	DCTD	2.5274067	2.6491132	2.5574818	0.1217065	0.0300751	0.0758908	up
A_33_P3210827	RAD51D	-1.385868	-1.609406	-1.010666	-0.223538	0.3752017	0.0758317	up
A_22_P00003395	LOC102725059	0.0434895	0.1181135	0.1204209	0.0746241	0.0769315	0.0757778	up
A_23_P83923	ZNF672	3.1931276	3.2076297	3.3300838	0.014502	0.1369562	0.0757291	up
A_33_P3295870	FAM166A	0.6718555	0.8094082	0.685636	0.1375527	0.0137806	0.0756667	up
A_33_P3211804	RUNX1	2.9143734	2.9291377	3.0509377	0.0147643	0.1365643	0.0756643	up
A_22_P00004421	lnc-COPZ2-1	-1.080188	-0.988691	-1.020374	0.0914974	0.059814	0.0756557	up
A_23_P204395	AACS	3.1225443	3.2125144	3.1838503	0.0899701	0.061306	0.0756381	up
A_23_P256107	HPSE	0.3003984	0.3851895	0.3666863	0.0847912	0.066288	0.0755396	up
A_19_P00320989	LINC00894	-3.283938	-3.028351	-3.388577	0.2555878	-0.104639	0.0754745	up
A_23_P323166	SRRM2	6.24298	6.3732944	6.26359	0.1303144	0.0206099	0.0754621	up
A_21_P0000515	VTRNA1-3	2.9531765	2.9346004	3.1226664	-0.018576	0.1694899	0.0754569	up
A_33_P3284077	NUP133	-0.577377	-0.655266	-0.348594	-0.077889	0.2287831	0.0754471	up
A_33_P3308137	FAM221A	-0.706101	-0.76673	-0.49466	-0.060628	0.2114415	0.0754066	up
A_23_P115331	AHDC1	0.6198521	0.6652441	0.725266	0.045392	0.1054139	0.075403	up
A_22_P00007581	HDAC9	-2.364067	-2.016541	-2.561049	0.3475261	-0.196982	0.075272	up
A_21_P0006497	lnc-FAM156B-1	-2.847668	-2.857486	-2.687316	-0.009818	0.1603525	0.0752671	up
A_23_P371787	SUSD6	3.62465	3.7965856	3.6031141	0.1719356	-0.021536	0.0751998	up
A_23_P65455	HAUS4	0.5049753	0.6657705	0.4944606	0.1607952	-0.010515	0.0751402	up
A_23_P216549	RUSC2	1.2954221	1.4384308	1.302546	0.1430087	0.0071239	0.0750663	up
A_33_P3258223	MCM7	2.0668697	2.1998181	2.0840435	0.1329484	0.0171738	0.0750611	up
A_32_P42574	C1orf198	5.700742	5.7191005	5.8324614	0.0183587	0.1317196	0.0750391	up
A_24_P43391	TMEM165	1.8193455	1.8717918	1.9168587	0.0524464	0.0975132	0.0749798	up
A_32_P155091	ATXN2L	6.41171	6.4912057	6.482092	0.0794959	0.0703821	0.074939	up
A_23_P94782	CAPN8	1.3115077	1.2455788	1.5272641	-0.065929	0.2157564	0.0749137	up
A_32_P170481	LOC100240735	0.6202087	0.5811954	0.8090415	-0.039013	0.1888328	0.0749097	up
A_21_P0000774	STK4-AS1	-2.610653	-2.512601	-2.558955	0.0980523	0.0516982	0.0748752	up
A_21_P0014703	LINC01128	-2.015415	-2.694487	-1.186705	-0.679072	0.8287106	0.0748193	up
A_33_P3363674	NFYC	2.5051212	2.5996242	2.5602245	0.0945029	0.0551033	0.0748031	up
A_23_P350172	RNF169	2.0471506	2.2432513	2.0005846	0.1961007	-0.046566	0.0747674	up
A_33_P3398927	MBD1	0.7779937	0.8952098	0.8100791	0.1172161	0.0320854	0.0746508	up
A_23_P135239	TLE1	3.26412	3.3710666	3.306446	0.1069465	0.042326	0.0746362	up
A_32_P228699	PRDM10	0.3603935	0.506762	0.363205	0.1463685	0.0028114	0.07459	up
A_24_P313334	SERF2	1.4042892	1.3627667	1.5949826	-0.041523	0.1906934	0.0745854	up
A_21_P0013102	LOC102723724	0.9147468	0.927206	1.0513668	0.0124593	0.13662	0.0745397	up
A_21_P0008447	lnc-SGPP1-1	-2.212066	-2.161611	-2.113453	0.0504544	0.0986123	0.0745333	up
A_23_P157715	PPP1R16A	6.0955305	6.0650134	6.275074	-0.030517	0.1795435	0.0745132	up
A_33_P3267270	TCEANC2	-2.317402	-2.204525	-2.281299	0.1128771	0.0361028	0.07449	up
A_22_P00023500	LINC01475	-1.15993	-1.049635	-1.121319	0.1102948	0.0386114	0.0744531	up
A_23_P12173	CRTC2	3.8837776	4.0184436	3.8979015	0.134666	0.0141239	0.0743949	up
A_24_P244410	ANAPC15	1.8661261	1.9608245	1.9201736	0.0946984	0.0540476	0.074373	up
A_24_P305933	TMCC3	-0.540259	-0.432345	-0.499496	0.1079135	0.0407624	0.074338	up
A_32_P215318	ACACA	1.0766263	0.8709779	1.4309468	-0.205648	0.3543205	0.0743361	up
A_23_P75790	MYRF	4.371091	4.4811845	4.4096603	0.1100936	0.0385695	0.0743315	up
A_23_P50799	OR10H2	0.6174836	0.7519245	0.6316938	0.1344409	0.0142102	0.0743256	up
A_24_P14531	TM9SF4	3.3951035	3.1325603	3.8062925	-0.262543	0.4111891	0.0743229	up
A_33_P3335257	CATSPERD	-0.881347	-0.808597	-0.805527	0.0727496	0.0758195	0.0742846	up
A_24_P56330	TEX29	-3.205262	-2.770096	-3.492204	0.4351666	-0.286942	0.0741124	up
A_23_P26570	TMED6	-2.448514	-2.476183	-2.272685	-0.027669	0.1758294	0.07408	up
A_24_P36745	CXorf38	2.4556332	2.4594007	2.5999928	0.0037675	0.1443596	0.0740635	up
A_23_P70571	SLC39A7	2.1276665	2.0701575	2.3332834	-0.057509	0.205617	0.074054	up
A_23_P6344	SDF2L1	7.8213634	7.5948167	8.19588	-0.226547	0.3745165	0.0739849	up
A_24_P942730	ZSCAN29	2.0273552	2.1061206	2.096528	0.0787654	0.0691729	0.0739691	up
A_33_P3273459	SPATA21	-1.340535	-1.346857	-1.186297	-0.006322	0.1542378	0.0739577	up
A_24_P47547	RAN	6.5235643	6.5826726	6.612342	0.0591083	0.0887775	0.0739429	up

A_32_P20454	COPA	1.6255646	1.8533621	1.5456357	0.2277975	-0.079929	0.0739343	up
A_23_P103099	RBFOX2	0.97016	1.0530787	1.0348501	0.0829186	0.0646901	0.0738044	up
A_24_P118489	WHAMM	2.8467312	2.9371514	2.9039087	0.0904202	0.0571775	0.0737989	up
A_23_P73012	C9orf3	3.3054724	3.423036	3.3354702	0.1175637	0.0299978	0.0737808	up
A_23_P44155	CD96	-3.192604	-2.907743	-3.330102	0.2848611	-0.137498	0.0736814	up
A_24_P922357	C22orf39	0.4488607	0.6293817	0.4156828	0.180521	-0.033178	0.0736716	up
A_23_P329573	ITGB2	-0.439797	-0.091192	-0.641077	0.3486052	-0.201279	0.073663	up
A_23_P156049	HEXB	4.1714725	4.2362447	4.2539034	0.0647721	0.0824308	0.0736015	up
A_23_P3994	TMEM256	3.4709024	3.5692887	3.5196447	0.0983863	0.0487423	0.0735643	up
A_24_P104091	ORAOV1	3.8849545	3.9575677	3.9593782	0.0726132	0.0744238	0.0735185	up
A_23_P360179	NEURL4	1.000277	0.9916005	1.1558585	-0.008677	0.1555815	0.0734525	up
A_23_P100141	UNKL	1.0883937	1.1763334	1.1473293	0.0879397	0.0589356	0.0734377	up
A_24_P902052	DANCR	5.044389	5.1214204	5.114232	0.0770316	0.0698433	0.0734375	up
A_21_P0003289	lnc-MINA-3	-2.487708	-2.19744	-2.631179	0.2902675	-0.143471	0.0733984	up
A_33_P3272361	CTNNBL1	5.468113	5.5703015	5.5126066	0.1021886	0.0444937	0.0733411	up
A_23_P200143	DCAF8	-0.503245	-0.352391	-0.507524	0.1508536	-0.004279	0.0732875	up
A_23_P37347	SNW1	4.416644	4.4674363	4.5123568	0.0507922	0.0957127	0.0732524	up
A_22_P00016170	DCP1A	-1.70412	-1.718496	-1.543291	-0.014376	0.1608286	0.0732262	up
A_23_P124892	KISS1	-1.525374	-1.489627	-1.414694	0.0357471	0.1106806	0.0732138	up
A_33_P3235053	TRIM47	6.224368	6.2549863	6.34006	0.0306182	0.1156921	0.0731552	up
A_33_P3414591	TWIST1	0.7322564	0.7062807	0.9044561	-0.025976	0.1721997	0.073112	up
A_23_P143650	DUSP18	-0.459534	-0.053742	-0.719158	0.4057913	-0.259624	0.0730834	up
A_33_P3269208	STX19	0.8887253	1.0352297	0.8883843	0.1465044	-0.000341	0.0730817	up
A_33_P3270404	ATG9A	2.3449984	2.2755675	2.5605907	-0.069431	0.2155924	0.0730808	up
A_33_P3218768	UPF2	0.641542	0.7093024	0.7198472	0.0677605	0.0783052	0.0730329	up
A_21_P0013342	CCZ1	-2.118893	-2.128415	-1.963306	-0.009522	0.1555872	0.0730325	up
A_33_P3379371	RYK	4.7060013	4.6717486	4.8862963	-0.034253	0.180295	0.0730212	up
A_33_P3312975	CLASP1	-0.625428	-0.486891	-0.617953	0.1385369	0.0074754	0.0730062	up
A_23_P211504	KDELR3	2.6913242	2.5957637	2.9328365	-0.095561	0.2415123	0.0729759	up
A_23_P97573	PRPF3	1.7110147	1.6305676	1.9373503	-0.080447	0.2263355	0.0729442	up
A_23_P253464	FAM175A	-0.866738	-0.405376	-1.182244	0.4613619	-0.315506	0.0729277	up
A_22_P00010091	LOC102725254	-1.733154	-1.724887	-1.595696	0.0082669	0.1374583	0.0728626	up
A_23_P411806	SLC44A1	0.8897171	1.0232844	0.9018703	0.1335673	0.0121531	0.0728602	up
A_33_P3369834	REEP6	-1.335659	-1.276267	-1.249507	0.0593915	0.0861521	0.0727718	up
A_33_P3313065	OR5C1	-3.241447	-2.998002	-3.339402	0.2434442	-0.097955	0.0727446	up
A_23_P1199	NUDT5	5.477807	5.4844356	5.616624	0.0066285	0.1388168	0.0727227	up
A_23_P142389	LSR	3.6980495	3.8453288	3.696185	0.1472793	-0.001864	0.0727074	up
A_33_P3391865	INF2	0.8873019	1.0896573	0.8302522	0.2023554	-0.05705	0.0726528	up
A_33_P3278435	COA4	5.1958733	5.1443696	5.3926144	-0.051504	0.1967411	0.0726187	up
A_23_P130653	RTBDN	0.0225058	0.2403712	-0.050168	0.2178655	-0.072674	0.0725958	up
A_21_P0000473	SNORA84	-0.082881	0.1069789	-0.127588	0.1898603	-0.044707	0.0725768	up
A_23_P122674	PAK1IP1	1.8920813	1.8208542	2.1083765	-0.071227	0.2162952	0.0725341	up
A_22_P00009616	LOC100507554	-2.261735	-2.305732	-2.072681	-0.043997	0.1890545	0.0725288	up
A_33_P3277012	DFFB	-2.335478	-2.205062	-2.320907	0.1304162	0.0145717	0.0724939	up
A_23_P157970	INVS	1.298316	1.3375468	1.4040184	0.0392308	0.1057024	0.0724666	up
A_33_P3256680	MFHAS1	-2.43113	-2.699161	-2.018353	-0.268032	0.412777	0.0723727	up
A_24_P156267	SOX12	-2.984128	-2.908902	-2.914862	0.0752256	0.0692656	0.0722456	up
A_23_P158880	STARD5	-1.095287	-0.982595	-1.063695	0.1126928	0.0315928	0.0721428	up
A_23_P128967	ALDH6A1	0.6640849	0.7477703	0.7245774	0.0836854	0.0604925	0.072089	up
A_32_P40375	LOC441528	0.4318609	0.6151056	0.3927741	0.1832447	-0.039087	0.0720789	up
A_23_P428248	TTC21A	-1.554558	-1.403685	-1.561369	0.1508732	-0.006812	0.0720308	up
A_23_P376599	RALBP1	6.019705	6.0625186	6.1209	0.0428138	0.1011953	0.0720046	up
A_33_P3240244	C2CD4D	-2.091086	-2.02477	-2.013445	0.0663166	0.0776415	0.071979	up
A_21_P0012651	EEF2KMT	-0.54989	-0.447412	-0.508485	0.1024785	0.0414047	0.0719416	up
A_32_P8813	LINC00926	-1.575587	-1.507412	-1.499922	0.0681748	0.0756655	0.0719202	up
A_23_P99785	ZC2HC1C	0.0222907	0.1394129	0.0488434	0.1171222	0.0265527	0.0718374	up

A_33_P3306024	SIGLEC16	2.9584494	2.9643054	3.0961533	0.005856	0.1377039	0.07178	up
A_33_P3398564	ACAP3	3.0394173	3.1938834	3.0284996	0.1544662	-0.010918	0.0717742	up
A_22_P00002526	lnc-C17orf48-1	-2.504666	-2.285124	-2.580731	0.2195418	-0.076066	0.0717381	up
A_23_P169873	TRPC2	-2.829192	-2.90013	-2.614842	-0.070938	0.2143495	0.0717058	up
A_22_P00001724	lnc-ATG7-2	3.9386654	3.950852	4.0698786	0.0121865	0.1312132	0.0716999	up
A_23_P331253	XPNPEP1	1.3965678	1.440321	1.4960294	0.0437531	0.0994616	0.0716074	up
A_23_P22915	SLC30A7	1.4755988	1.6592741	1.435009	0.1836753	-0.04059	0.0715427	up
A_23_P140668	IDH3A	1.5766916	1.4664493	1.8299618	-0.110242	0.2532702	0.0715139	up
A_23_P105519	WBP11	3.9796515	4.043283	4.0590105	0.0636315	0.0793591	0.0714953	up
A_24_P1773	LONP2	0.2590723	0.1916642	0.4693112	-0.067408	0.2102389	0.0714154	up
A_21_P0006818	lnc-AKR1E2-5	-1.288401	-1.42631	-1.007731	-0.137909	0.2806697	0.0713804	up
A_33_P3359027	CCDC42B	-2.761391	-2.913146	-2.467002	-0.151755	0.294389	0.0713172	up
A_33_P3257312	ATG2A	4.530237	4.439755	4.7632456	-0.090482	0.2330084	0.0712631	up
A_23_P37560	PEX11A	0.6629992	0.6940107	0.7744036	0.0310116	0.1114044	0.071208	up
A_24_P565556	ALG1	0.7598138	0.6085572	1.053473	-0.151257	0.2936592	0.0712013	up
A_21_P0010889	NUTM2A-AS1	1.2755008	1.2658596	1.4273944	-0.009641	0.1518936	0.0711262	up
A_22_P00017738	lnc-YBEY-1	0.4168973	0.6183972	0.3576145	0.2014999	-0.059283	0.0711086	up
A_23_P161022	SNAPIN	2.6687279	2.3105092	3.1691523	-0.358219	0.5004244	0.0711029	up
A_23_P81087	INTS12	1.6818781	1.5986614	1.9070206	-0.083217	0.2251425	0.0709629	up
A_23_P149281	EPHA2	1.7656374	1.8733039	1.7996316	0.1076665	0.0339942	0.0708303	up
A_21_P0002505	lnc-C2orf27B-3	1.4535594	1.6434422	1.4051604	0.1898828	-0.048399	0.0707419	up
A_21_P0011678	CS	4.752038	4.5631833	5.0821037	-0.188855	0.3300657	0.0706055	up
A_23_P218505	LHB	0.5818968	0.7359037	0.5689106	0.154007	-0.012986	0.0705104	up
A_23_P66158	KNOP1	3.9999905	3.8892283	4.2516413	-0.110762	0.2516508	0.0704443	up
A_19_P00320056	HCG18	0.7641263	0.8104038	0.8587279	0.0462775	0.0946016	0.0704396	up
A_24_P365327	LSM14A	5.1328564	5.264609	5.141944	0.1317525	0.0090876	0.07042	up
A_21_P0013371	LOC100506098	-1.323204	-1.431793	-1.07384	-0.108589	0.2493644	0.0703876	up
A_23_P90855	FARP2	-1.48369	-1.624547	-1.202068	-0.140857	0.2816215	0.0703824	up
A_23_P49975	KRT10	7.391697	7.286038	7.6380205	-0.105659	0.2463236	0.0703323	up
A_24_P83922	SNRPC	-1.154928	-1.0434	-1.125978	0.1115274	0.0289502	0.0702388	up
A_33_P3452297	LINC00317	-1.0097	-0.958044	-0.920912	0.0516562	0.0887885	0.0702224	up
A_33_P3383233	NDRG2	2.3947392	2.2532277	2.6765156	-0.141511	0.2817764	0.0701325	up
A_33_P3369505	MANEAL	0.7741599	0.7091603	0.9792109	-0.065	0.205051	0.0700257	up
A_23_P70318	ENPP4	2.6997786	2.760466	2.7789822	0.0606875	0.0792036	0.0699456	up
A_22_P00011998	ITGA9-AS1	-1.454469	-1.532506	-1.236703	-0.078036	0.2177663	0.069865	up
A_33_P3230976	PREP	0.8569365	1.0021191	0.8512192	0.1451826	-0.005717	0.0697327	up
A_23_P351535	DBIL5P	-2.830288	-2.414017	-3.107138	0.4162705	-0.27685	0.0697101	up
A_22_P00013889	LOC101927060	-2.111455	-1.535445	-2.54806	0.5760098	-0.436606	0.0697019	up
A_33_P3402474	ATAT1	1.0075231	0.9074836	1.2469616	-0.100039	0.2394385	0.0696995	up
A_24_P63019	IL1R2	1.4027157	1.3418665	1.6029286	-0.060849	0.200213	0.0696819	up
A_21_P0000808	UBL7-AS1	-0.660462	-0.433053	-0.748662	0.2274094	-0.088199	0.0696051	up
A_33_P3627001	PEBP4	-0.985513	-0.720343	-1.111512	0.2651701	-0.125999	0.0695853	up
A_22_P00024951	LOC727993	-1.52308	-1.409165	-1.49784	0.113915	0.0252395	0.0695772	up
A_23_P122563	PFDN6	3.245617	3.0961833	3.5340033	-0.149434	0.2883863	0.0694764	up
A_23_P61050	MLKL	3.9852686	3.9353404	4.174075	-0.049928	0.1888065	0.0694392	up
A_33_P3301524	XRCC3	2.9229145	2.87291	3.11172	-0.050004	0.1888056	0.0694005	up
A_24_P22939	ADAMTSL5	-0.291555	-0.202864	-0.241596	0.0886912	0.0499587	0.069325	up
A_21_P0000236	SNORA64	2.2439976	2.2303023	2.3961782	-0.013695	0.1521807	0.0692427	up
A_24_P22488	AXIN1	2.1398335	2.2913327	2.12679	0.1514993	-0.013043	0.0692279	up
A_23_P364517	SELK	4.341119	4.503865	4.316679	0.162746	-0.02444	0.0691531	up
A_23_P142407	ZNF101	1.4759259	1.3833241	1.7067242	-0.092602	0.2307982	0.0690982	up
A_23_P92082	TKT	7.0945415	7.1540513	7.1731443	0.0595098	0.0786028	0.0690563	up
A_23_P43810	LTBP1	-1.949628	-1.840449	-1.920824	0.1091795	0.0288043	0.0689919	up
A_22_P00007204	MIR17HG	-1.309909	-0.899846	-1.582008	0.4100628	-0.2721	0.0689817	up
A_33_P3342076	PRDM2	-0.97564	-0.864578	-0.948776	0.1110616	0.0268636	0.0689626	up
A_23_P127964	PRCP	5.4835386	5.57937	5.5255995	0.0958314	0.0420609	0.0689461	up

A_33_P3339109	POLR1C	-0.31108	-0.314491	-0.169837	-0.00341	0.1412435	0.0689166	up
A_23_P40657	GCAT	-0.455205	-0.262242	-0.510407	0.1929622	-0.055203	0.0688796	up
A_24_P388528	ST6GAL1	3.162386	3.3182569	3.144205	0.1558709	-0.018181	0.068845	up
A_33_P3296831	TP73	-2.445223	-2.063663	-2.689107	0.3815601	-0.243885	0.0688378	up
A_33_P3264224	CDC42BPG	0.2495403	0.3680105	0.2687411	0.1184702	0.0192008	0.0688355	up
A_24_P186346	ANAPC1	1.9844284	2.2263055	1.8802099	0.2418771	-0.104218	0.0688293	up
A_21_P0014809	FAM136A	4.832762	4.940334	4.8624783	0.1075721	0.0297165	0.0686443	up
A_23_P352266	BCL2	-1.026837	-0.963847	-0.952606	0.0629902	0.0742312	0.0686107	up
A_21_P0013818	XLOC_l2_015752	-1.21652	-1.310167	-0.985767	-0.093647	0.2307529	0.0685527	up
A_23_P34628	GTF2B	2.0785446	2.089229	2.2049627	0.0106845	0.1264181	0.0685513	up
A_33_P3264419	YTHDF3	2.925354	2.974112	3.0135927	0.048758	0.0882387	0.0684984	up
A_23_P134274	POP7	7.725816	7.6864905	7.90211	-0.039325	0.1762943	0.0684845	up
A_23_P136547	PIGG	2.144762	2.3300433	2.096445	0.1852813	-0.048317	0.0684822	up
A_23_P162547	MYL2	-1.2891	-0.738925	-1.702413	0.5501747	-0.413313	0.0684307	up
A_24_P144377	DEXI	4.756852	4.71547	4.935051	-0.041382	0.1781988	0.0684082	up
A_33_P3360216	HIST1H2AI	2.864542	2.874456	2.9913988	0.0099139	0.1268568	0.0683854	up
A_22_P00007879	lnc-HSCB-1	-2.268768	-2.360539	-2.040329	-0.091771	0.2284398	0.0683346	up
A_24_P374652	NUCKS1	2.1954288	2.2828603	2.2446222	0.0874314	0.0491934	0.0683124	up
A_33_P3373459	CHST12	2.7952938	2.6389213	3.0881128	-0.156373	0.292819	0.0682232	up
A_33_P3224858	EIF2AK2	1.374157	1.1865726	1.6980777	-0.187584	0.3239207	0.0681682	up
A_23_P251173	EIF2AK1	4.206259	4.385918	4.162796	0.1796594	-0.043463	0.0680983	up
A_22_P00001173	LOC100506603	-1.700405	-1.754173	-1.51046	-0.053768	0.1899452	0.0680885	up
A_33_P3334220	ACACB	-0.145247	-0.225389	0.0710702	-0.080143	0.2163167	0.0680869	up
A_21_P0005816	lnc-RP11-419C23.1.1-2	-2.143021	-2.172818	-1.977052	-0.029796	0.1659689	0.0680863	up
A_32_P210252	RPL22	6.4636116	6.643536	6.41982	0.1799245	-0.043792	0.0680664	up
A_24_P97145	KIDINS220	0.3347416	0.529192	0.2762704	0.1944504	-0.058471	0.0679896	up
A_33_P3407103	YTHDF1	-1.063574	-1.178755	-0.812645	-0.115181	0.2509294	0.0678742	up
A_23_P13885	ATN1	1.8507633	1.9653888	1.8718472	0.1146255	0.0210838	0.0678546	up
A_23_P387184	NHSL1	-0.159707	-0.055292	-0.128703	0.1044149	0.031004	0.0677094	up
A_23_P168276	TBPL1	3.8673992	3.9367166	3.9334545	0.0693173	0.0660553	0.0676863	up
A_23_P395374	HIST1H4D	4.3949184	4.3981442	4.527048	0.0032258	0.1321297	0.0676777	up
A_24_P134653	OFD1	3.0119667	3.0736785	3.0855627	0.0617118	0.073596	0.0676539	up
A_23_P142590	ILKAP	0.603931	0.6882334	0.6549349	0.0843024	0.0510039	0.0676532	up
A_33_P3393106	FAM188A	-1.798136	-1.81528	-1.645696	-0.017143	0.1524406	0.0676487	up
A_23_P140994	DDX19B	1.7292576	1.8500814	1.7435899	0.1208239	0.0143323	0.0675781	up
A_33_P3305780	CCNI2	-0.251652	-0.298804	-0.06935	-0.047152	0.182302	0.0675752	up
A_33_P3220565	UBTD2	1.4736276	1.5044804	1.5778646	0.0308528	0.1042371	0.0675449	up
A_22_P00011959	LOC100506100	0.5860062	0.594976	0.712091	0.0089698	0.1260848	0.0675273	up
A_33_P3309039	CPSF7	4.7362146	4.8706594	4.736512	0.1344447	0.0002975	0.0673711	up
A_23_P76364	CD9	5.9535027	5.997535	6.044097	0.0440326	0.0905943	0.0673134	up
A_24_P38951	RELT	3.7951965	3.7127757	4.012228	-0.082421	0.2170315	0.0673053	up
A_23_P139998	UBAC2	2.7136421	2.8597999	2.7020483	0.1461577	-0.011594	0.067282	up
A_23_P215566	AHR	3.370922	3.4165845	3.459793	0.0456624	0.088871	0.0672667	up
A_21_P0006358	lnc-HIATL1-2	-2.185727	-2.086884	-2.150321	0.0988429	0.0354056	0.0671242	up
A_23_P126803	ARPC5	5.6691475	5.5313964	5.941085	-0.137751	0.2719374	0.0670931	up
A_21_P0008205	lnc-GPR183-2	-1.312936	-1.205402	-1.286354	0.1075335	0.0265822	0.0670578	up
A_33_P3317317	KRTAP5-4	1.3862448	1.3005314	1.6060395	-0.085713	0.2197948	0.0670407	up
A_22_P00020277	lnc-GCNT3-1	-2.28198	-2.133833	-2.296093	0.1481471	-0.014113	0.0670171	up
A_19_P00322938	LOC401320	-1.296968	-1.050765	-1.409226	0.2462034	-0.112257	0.066973	up
A_23_P112801	CHP1	6.4175997	6.5599504	6.409189	0.1423507	-0.00841	0.0669701	up
A_24_P157926	TNFAIP3	-1.267523	-1.074469	-1.326724	0.1930547	-0.059201	0.066927	up
A_22_P00001665	lnc-ASH1L-2	-1.632639	-1.511337	-1.620326	0.1213021	0.0123134	0.0668077	up
A_22_P00020330	lnc-ANGEL1-2	-1.700489	-1.650293	-1.617497	0.0501957	0.0829916	0.0665936	up
A_23_P13073	PUS3	3.7148695	3.6606174	3.9021702	-0.054252	0.1873007	0.0665243	up
A_33_P3387365	PXMP4	-0.780089	-0.798344	-0.628829	-0.018255	0.1512604	0.0665028	up

A_23_P300781	CNOT4	2.2732725	2.2684722	2.4106245	-0.0048	0.137352	0.0662758	up
A_24_P345209	DYRK3	-0.972887	-0.790479	-1.022781	0.1824074	-0.049895	0.0662563	up
A_22_P00006575	MAP3K14-AS1	-2.555643	-2.840194	-2.138649	-0.284551	0.4169943	0.0662218	up
A_33_P3275751	SEC14L1	3.357689	3.3647046	3.4831	0.0070157	0.125411	0.0662134	up
A_33_P3368452	MICA	2.372734	2.3291478	2.548705	-0.043586	0.175971	0.0661924	up
A_22_P00015574	lnc-STUB1-1	-0.185911	-0.244474	0.0049696	-0.058563	0.1908808	0.066159	up
A_24_P218006	DNAJC30	0.7701287	0.8584905	0.8140836	0.0883617	0.0439549	0.0661583	up
A_23_P116602	USP35	2.4943113	2.4450364	2.6758156	-0.049275	0.1815043	0.0661147	up
A_33_P3360675	PRKAR1B	1.5284567	1.6522899	1.5367799	0.1238332	0.0083232	0.0660782	up
A_32_P110016	LOC101928424	-3.553723	-3.285431	-3.68994	0.268292	-0.136216	0.0660378	up
A_33_P3401093	FBXO36	-1.365668	-1.478191	-1.121112	-0.112524	0.2445555	0.066016	up
A_23_P60472	UBAP2	4.064973	4.057283	4.204652	-0.00769	0.139679	0.0659945	up
A_23_P16032	TRAPPC2B	2.685648	2.7587972	2.7443447	0.0731492	0.0586967	0.065923	up
A_33_P3314466	IMMT	0.5099897	0.5610337	0.5907769	0.051044	0.0807872	0.0659156	up
A_23_P342600	STK35	-1.072251	-1.036828	-0.975864	0.0354233	0.0963869	0.0659051	up
A_21_P0013308	XLOC_l2_013846	-2.530471	-2.241396	-2.687756	0.2890749	-0.157284	0.0658953	up
A_33_P3286608	PGBD2	0.5390706	0.6077986	0.6020489	0.068728	0.0629783	0.0658531	up
A_23_P137143	DKC1	5.081311	5.1305394	5.163769	0.0492282	0.0824575	0.0658429	up
A_22_P00017059	ADAM8	-1.325297	-1.157196	-1.361758	0.1681008	-0.036461	0.06582	up
A_33_P3244322	BCL2L12	2.293727	2.4455209	2.273508	0.151794	-0.020219	0.0657876	up
A_22_P00010644	lnc-NEDD1-4	-2.530718	-2.496451	-2.433448	0.0342677	0.0972707	0.0657692	up
A_33_P3278540	MRPL14	5.063282	5.0383687	5.2197266	-0.024913	0.1564446	0.0657656	up
A_24_P410952	PEA15	1.6656399	1.716721	1.7458339	0.0510812	0.080194	0.0656376	up
A_33_P3308055	EIF4E2	3.8867779	3.9388585	3.96597	0.0520806	0.0791922	0.0656364	up
A_22_P00020240	lnc-TRPT1-2	-3.253083	-3.033996	-3.340979	0.2190869	-0.087895	0.0655958	up
A_22_P00025822	LOC100130264	-2.610301	-2.135155	-2.954397	0.4751453	-0.344097	0.0655243	up
A_33_P3299421	ZNF530	-0.894098	-0.863728	-0.793496	0.0303698	0.1006022	0.065486	up
A_24_P135444	AMFR	1.9533577	1.7887907	2.2487364	-0.164567	0.2953787	0.0654058	up
A_33_P3751230	lnc-MIB2-1	-3.113642	-2.69452	-3.401956	0.4191217	-0.288314	0.0654037	up
A_33_P3344292	SAMD4A	-2.500027	-2.675366	-2.193921	-0.175339	0.3061063	0.0653838	up
A_33_P3377519	HOXA6	0.4721494	0.8386922	0.236371	0.3665428	-0.235778	0.0653822	up
A_24_P107941	USB1	4.982294	4.9154572	5.179866	-0.066837	0.1975718	0.0653675	up
A_23_P94683	NFX1	1.3099766	1.3946218	1.3560276	0.0846453	0.046051	0.0653481	up
A_24_P124662	MAPKAPK5	3.4674883	3.4316678	3.633974	-0.03582	0.1664858	0.0653327	up
A_19_P00317871	PVT1	1.1942921	1.2826748	1.2363558	0.0883827	0.0420637	0.0652232	up
A_23_P71316	RBPMS	-2.276243	-2.224082	-2.198051	0.0521607	0.0781918	0.0651763	up
A_33_P3217834	SMARCAL1	3.5139828	3.5030417	3.6552181	-0.010941	0.1412354	0.0651472	up
A_23_P331895	TTYH3	3.1957378	3.0277848	3.4939804	-0.167953	0.2982426	0.0651448	up
A_32_P29784	TPM3	6.9599695	6.912454	7.137622	-0.047515	0.1776524	0.0650685	up
A_33_P3350728	FARP2	-1.677843	-1.818881	-1.406768	-0.141038	0.2710748	0.0650182	up
A_23_P70020	PFDN1	5.2494507	5.2939897	5.334922	0.044539	0.0854712	0.0650051	up
A_23_P315789	RFXAP	-0.450228	-0.230037	-0.540465	0.220191	-0.090237	0.0649769	up
A_22_P00005366	lnc-DRD5-1	-2.466525	-2.457284	-2.345828	0.0092416	0.1206975	0.0649695	up
A_33_P3253234	IQSEC2	2.9735708	2.7577834	3.3192549	-0.215787	0.3456841	0.0649483	up
A_23_P168629	RBM28	2.6909924	2.7650123	2.746848	0.0740199	0.0558558	0.0649378	up
A_32_P149536	SUMO2	6.3452225	6.3539357	6.466341	0.0087132	0.1211185	0.0649159	up
A_22_P00015739	LOC101928994	-2.825747	-2.687476	-2.834359	0.1382709	-0.008612	0.0648296	up
A_23_P46315	DENND2C	3.0512753	3.2721686	2.9599915	0.2208934	-0.091284	0.0648048	up
A_23_P214533	ZSCAN31	-0.370004	-0.094552	-0.515943	0.2754517	-0.145939	0.0647562	up
A_24_P183128	PLAC8	2.759431	3.1341672	2.513977	0.3747363	-0.245454	0.0646412	up
A_23_P116624	SLC6A5	-3.05704	-2.869825	-3.115014	0.1872149	-0.057974	0.0646205	up
A_23_P212497	ACAD11	-0.551159	-0.312517	-0.660576	0.2386417	-0.109417	0.0646124	up
A_23_P411102	DLG4	-1.798953	-1.615487	-1.853267	0.1834655	-0.054314	0.0645757	up
A_23_P346421	ZNF532	-0.06699	0.1424837	-0.147313	0.2094736	-0.080323	0.0645754	up
A_23_P69226	EMC3	2.4338655	2.2904782	2.706358	-0.143387	0.2724924	0.0645525	up
A_23_P64932	RIC8B	-2.129691	-2.18937	-1.940915	-0.059679	0.188776	0.0645486	up

A_23_P162211	MANSC1	1.2521086	1.2916069	1.3416705	0.0394983	0.0895619	0.0645301	up
A_22_P00012284	lnc-PPP2R2D-3	-1.878112	-1.891474	-1.735819	-0.013362	0.1422925	0.064465	up
A_21_P0010854	AKR1C8P	1.3056145	2.0858026	0.6543222	0.7801881	-0.651292	0.0644479	up
A_33_P3411741	SIDT2	-1.071962	-1.272566	-0.742593	-0.200604	0.3293691	0.0643823	up
A_19_P00317937	PPP4R1L	-1.604219	-1.60714	-1.472568	-0.002921	0.1316509	0.0643651	up
A_24_P264664	CHD2	2.19936	2.407516	2.1199179	0.2081561	-0.079442	0.064357	up
A_24_P217330	POMGNT2	0.2118187	0.0083084	0.5439072	-0.20351	0.3320885	0.0642891	up
A_33_P3369146	ATP2B3	-1.477106	-1.419132	-1.406538	0.0579743	0.0705686	0.0642715	up
A_23_P209538	KANSL3	-0.251479	-0.109848	-0.264578	0.1416316	-0.013099	0.0642664	up
A_33_P3367830	EFEMP2	-2.044142	-2.166583	-1.793189	-0.12244	0.2509532	0.0642564	up
A_23_P107744	S1PR5	-2.422676	-2.595949	-2.121086	-0.173272	0.3015904	0.064159	up
A_23_P101615	ZNF565	1.2110014	1.2342014	1.316112	0.0232	0.1051106	0.0641553	up
A_22_P00010335	lnc-MYBBP1A-2	-2.001153	-1.976859	-1.897262	0.0242934	0.1038909	0.0640922	up
A_33_P3262069	OR2AG2	-1.533305	-1.593091	-1.345367	-0.059786	0.1879377	0.0640757	up
A_23_P14473	CNIH1	6.4587545	6.549229	6.4964046	0.0904746	0.0376501	0.0640624	up
A_23_P377291	TGFA	-2.522162	-2.755806	-2.160444	-0.233643	0.3617184	0.0640376	up
A_23_P369641	MROH6	0.3709488	0.3644071	0.5055447	-0.006542	0.1345959	0.0640271	up
A_22_P00002802	LOC100133985	-0.576157	-0.450917	-0.57342	0.1252394	0.0027366	0.063988	up
A_32_P232620	lnc-PARN-3	-2.543062	-2.642088	-2.316261	-0.099026	0.2268009	0.0638872	up
A_21_P0007163	lnc-APOA4-1	-2.175938	-2.010818	-2.21336	0.1651201	-0.037422	0.0638492	up
A_33_P3331346	TRIM65	3.2695675	3.373201	3.293579	0.1036334	0.0240116	0.0638225	up
A_23_P502882	CBFA2T2	0.3314271	0.4780088	0.3122187	0.1465817	-0.019208	0.0636866	up
A_24_P135276	USP42	4.1826286	4.2341485	4.2583885	0.0515199	0.0757599	0.0636399	up
A_23_P62840	YRDC	4.5831833	4.532682	4.7609196	-0.050501	0.1777363	0.0636175	up
A_21_P0002212	LOC100506235	-1.790197	-1.631279	-1.822033	0.1589184	-0.031836	0.0635412	up
A_33_P3290687	DNAJC1	3.7255716	3.8778281	3.7003965	0.1522565	-0.025175	0.0635407	up
A_33_P3368895	lnc-UXS1-4	-1.38816	-1.540219	-1.109028	-0.152059	0.2791319	0.0635364	up
A_33_P3292621	SLC27A1	-3.500607	-3.204217	-3.66993	0.2963901	-0.169323	0.0635333	up
A_33_P3369311	BBX	2.7293758	2.7837596	2.80198	0.0543838	0.0726042	0.063494	up
A_24_P202717	PLEKHJ1	2.3730516	2.2048192	2.66827	-0.168232	0.2952185	0.063493	up
A_23_P89762	PHLPP1	0.7215023	0.7211266	0.8488603	-0.000376	0.127358	0.0634911	up
A_24_P101201	PDIA3	5.845813	5.929255	5.8893404	0.0834422	0.0435276	0.0634849	up
A_24_P260639	HIST1H1D	4.4843493	4.414637	4.6810217	-0.069712	0.1966724	0.0634801	up
A_23_P75220	SLC25A28	1.6620531	1.523355	1.92768	-0.138698	0.2656269	0.0634644	up
A_21_P0000490	SNORD105	-1.758338	-1.841048	-1.548842	-0.08271	0.2094955	0.0633926	up
A_23_P203888	MMP19	-0.281144	-0.249047	-0.186512	0.0320973	0.0946326	0.063365	up
A_24_P417007	POLR2F	0.700202	0.5950813	0.9320374	-0.105121	0.2318354	0.0633574	up
A_33_P3369565	APTX	-0.051136	0.042109	-0.017668	0.0932446	0.0334678	0.0633562	up
A_24_P256404	LOC100133616	-0.62725	-0.443401	-0.684568	0.1838489	-0.057318	0.0632653	up
A_33_P3388882	FAM66D	0.3927555	0.4863782	0.4255595	0.0936227	0.032804	0.0632133	up
A_33_P3346841	YTHDF2	3.7118664	3.6886573	3.861477	-0.023209	0.1496105	0.0632007	up
A_23_P61398	PIM3	6.0471506	6.0822825	6.1383915	0.0351319	0.0912409	0.0631864	up
A_33_P3213204	MAGEF1	2.9831667	2.9379745	3.1546955	-0.045192	0.1715288	0.0631683	up
A_23_P341275	POP1	4.6343317	4.545296	4.8496704	-0.089036	0.2153387	0.0631516	up
A_24_P324011	KCTD2	0.2703056	0.4546776	0.2121835	0.184372	-0.058122	0.0631249	up
A_24_P21447	SURF6	1.3271227	1.3882914	1.3921881	0.0611687	0.0650654	0.063117	up
A_21_P0008230	lnc-SGCG-5	-2.56332	-2.722405	-2.278209	-0.159085	0.2851112	0.0630131	up
A_33_P3408711	FLNA	-0.773294	-0.584775	-0.835929	0.188519	-0.062635	0.0629418	up
A_22_P00014384	SF3B3	3.9074535	3.948627	3.9919863	0.0411735	0.0845327	0.0628531	up
A_24_P67142	SON	0.7643952	0.7896648	0.8648186	0.0252695	0.1004233	0.0628464	up
A_23_P27822	GPATCH1	0.326839	0.260366	0.5189886	-0.066473	0.1921496	0.0628383	up
A_33_P3405754	CEP104	-0.812974	-0.640661	-0.859726	0.1723127	-0.046752	0.0627801	up
A_21_P0007763	MAPKAPK5-AS1	4.871291	4.836597	5.031391	-0.034694	0.1601	0.0627029	up
A_33_P3309075	TBC1D8	-0.987146	-0.799982	-1.048932	0.1871643	-0.061786	0.0626893	up
A_23_P204252	M6PR	2.050685	2.1214604	2.1050367	0.0707755	0.0543518	0.0625637	up
A_23_P63897	CTBP2	6.9053993	6.9344916	7.0013676	0.0290923	0.0959683	0.0625303	up

A_32_P215179	NPIPA1	1.0985675	1.3696618	0.9524798	0.2710943	-0.146088	0.0625033	up
A_22_P00000867	AGRN	7.1758175	7.222921	7.2535086	0.0471034	0.0776911	0.0623972	up
A_23_P128147	TUBA1B	9.286287	9.356583	9.340568	0.0702953	0.0542803	0.0622878	up
A_23_P137361	WRAP73	0.6935344	0.5904446	0.9211106	-0.10309	0.2275763	0.0622432	up
A_24_P65098	TMEM87A	-1.344851	-1.027907	-1.537326	0.3169441	-0.192475	0.0622344	up
A_24_P410408	KRT83	-2.513576	-2.297162	-2.605613	0.2164137	-0.092037	0.0621883	up
A_33_P3232945	F2RL1	2.6870718	2.7537856	2.7447205	0.0667138	0.0576487	0.0621812	up
A_24_P201739	SH2B3	-0.908531	-0.835667	-0.857037	0.0728641	0.0514941	0.0621791	up
A_23_P100517	TMEM170A	1.4720407	1.556632	1.5117593	0.0845914	0.0397186	0.062155	up
A_24_P139773	PPP1R8	1.5912943	1.5604773	1.7463188	-0.030817	0.1550245	0.0621037	up
A_23_P118690	ZNF207	4.422371	4.4312863	4.5374756	0.0089154	0.1151047	0.06201	up
A_24_P941376	ZNF473	0.1839895	0.1890049	0.3029923	0.0050154	0.1190028	0.0620091	up
A_23_P407074	DNM2	3.2261171	3.0769506	3.4992037	-0.149167	0.2730866	0.06196	up
A_33_P3246505	MAP3K8	1.5547395	1.500804	1.7325425	-0.053936	0.177803	0.0619338	up
A_33_P3269806	ZNF616	-0.017824	0.0155334	0.0725851	0.0333576	0.0904093	0.0618835	up
A_23_P141302	ZNF18	1.2811227	1.3343697	1.351512	0.053247	0.0703893	0.0618181	up
A_33_P3243502	LOC729732	0.3218322	0.2835927	0.4836025	-0.038239	0.1617703	0.0617654	up
A_33_P3221868	TMEM216	1.4540534	1.3354583	1.6961365	-0.118595	0.2420831	0.061744	up
A_22_P00024695	TMEM147-AS1	-0.490531	-0.424977	-0.432605	0.0655532	0.0579252	0.0617392	up
A_21_P0010461	LOC101928824	-2.46923	-2.073473	-2.741551	0.3957574	-0.272321	0.0617181	up
A_33_P3365805	CLN5	2.261571	2.2146459	2.4319077	-0.046925	0.1703367	0.0617058	up
A_23_P87150	LPXN	-0.989128	-0.873877	-0.980969	0.1152511	0.0081592	0.0617051	up
A_24_P155502	INHBC	-1.280255	-1.212065	-1.225221	0.0681901	0.0550346	0.0616124	up
A_21_P0006000	lnc-C9orf93-1	-2.320797	-2.044307	-2.474465	0.2764902	-0.153668	0.061411	up
A_33_P3398917	C15orf59	0.0878234	0.0964437	0.2019362	0.0086203	0.1141129	0.0613666	up
A_19_P00811843	ARHGAP32	0.1625028	0.2477107	0.1999941	0.0852079	0.0374913	0.0613496	up
A_33_P3372688	BHLHA9	-3.172904	-2.963975	-3.259165	0.2089291	-0.086261	0.0613343	up
A_23_P23575	SLC39A1	2.3144798	2.31457	2.436882	9.012E-05	0.1224022	0.0612462	up
A_32_P207180	ARL16	2.93005	3.070128	2.9122906	0.1400781	-0.017759	0.0611594	up
A_22_P00007734	VPS11	-0.794078	-0.691856	-0.774041	0.102222	0.0200377	0.0611298	up
A_23_P53152	AKIP1	0.1097846	-0.037243	0.3789172	-0.147027	0.2691326	0.0610526	up
A_21_P0004503	LOC257396	-0.274636	-0.152651	-0.274532	0.121985	0.0001035	0.0610442	up
A_33_P3353552	SLC48A1	3.224904	3.1873097	3.384552	-0.037594	0.1596479	0.0610268	up
A_22_P00003786	ATG9B	-0.576621	-0.434344	-0.596943	0.1422768	-0.020322	0.0609772	up
A_24_P225961	DAG1	2.3685274	2.4358573	2.423111	0.0673299	0.0545836	0.0609567	up
A_24_P210399	TM9SF2	3.8738565	3.960445	3.9091043	0.0865884	0.0352478	0.0609181	up
A_22_P00006571	lnc-FMN1-3	-1.166599	-0.997604	-1.213789	0.1689949	-0.04719	0.0609026	up
A_23_P259098	ZSCAN16	-1.220365	-1.203454	-1.115517	0.016911	0.1048484	0.0608797	up
A_33_P3254121	RNASET2	-1.114777	-0.801315	-1.306544	0.3134613	-0.191768	0.0608468	up
A_19_P00319854	SNAI3-AS1	-2.759178	-2.442035	-2.954649	0.3171434	-0.19547	0.0608366	up
A_24_P306561	TCF25	4.8518457	4.9585853	4.8665323	0.1067395	0.0146866	0.0607131	up
A_33_P3251672	ABCA2	-2.988965	-2.985446	-2.871122	0.0035191	0.1178432	0.0606811	up
A_23_P163682	RHBDF1	3.2277699	3.1860986	3.390788	-0.041671	0.1630182	0.0606735	up
A_23_P108404	AGAP1	2.5162172	2.6771493	2.4765682	0.1609321	-0.039649	0.0606415	up
A_23_P76969	SIPA1L1	3.2049665	3.319428	3.2117414	0.1144614	0.0067749	0.0606182	up
A_19_P00318909	XLOC_l2_010751	-3.014802	-3.13498	-2.773397	-0.120178	0.2414053	0.0606134	up
A_21_P0010592	LOC100288069	-1.488651	-1.337722	-1.51838	0.1509285	-0.029729	0.0605998	up
A_22_P00013902	lnc-RPS24-2	-1.08552	-1.070849	-0.978991	0.0146704	0.1065288	0.0605996	up
A_24_P158718	DTX4	-1.556043	-1.501387	-1.489513	0.0546565	0.0665298	0.0605931	up
A_23_P145584	UBE2H	0.8057728	1.1033354	0.6293721	0.2975626	-0.176401	0.060581	up
A_19_P00322220	SNHG17	2.0247316	1.899992	2.2704792	-0.12474	0.2457476	0.060504	up
A_24_P262407	THRA	1.4099956	1.5227804	1.4181051	0.1127849	0.0081096	0.0604472	up
A_33_P3397520	KRTAP10-12	-2.110299	-2.115126	-1.984616	-0.004827	0.1256833	0.0604283	up
A_23_P215449	BAZ1B	2.8704538	2.797752	3.0638027	-0.072702	0.1933489	0.0603235	up
A_23_P154108	DYNC1I2	4.381671	4.3689523	4.514909	-0.012719	0.1332378	0.0602596	up
A_22_P00014070	lnc-SAMD14-1	-1.96233	-1.841085	-1.963084	0.1212444	-0.000754	0.0602453	up

A_33_P3363665	NFYC	1.373776	1.4548693	1.4130697	0.0810933	0.0392938	0.0601935	up
A_19_P00316326	MAP3K13	2.7679281	2.6456609	3.0105515	-0.122267	0.2426233	0.060178	up
A_33_P3301174	TMED2	6.425578	6.527268	6.444127	0.1016898	0.018549	0.0601194	up
A_22_P00023679	lnc-QPCT-3	-3.154687	-2.910597	-3.27856	0.2440903	-0.123873	0.0601087	up
A_33_P3399090	DIXDC1	-0.488121	-0.392372	-0.463813	0.0957489	0.0243077	0.0600283	up
A_24_P98086	GNA12	3.332407	3.3854814	3.3992834	0.0530744	0.0668764	0.0599754	up
A_22_P00021154	lnc-HIST4H4-2	0.1638465	0.132668	0.3149567	-0.031178	0.1511102	0.0599658	up
A_24_P300483	C7orf25	-0.621462	-0.465665	-0.657528	0.155797	-0.036066	0.0598655	up
A_23_P253158	EP400	-0.015343	0.0323362	0.0566444	0.0476794	0.0719876	0.0598335	up
A_33_P3409392	FZD6	3.0771456	3.1585422	3.1153975	0.0813966	0.0382519	0.0598242	up
A_24_P134319	ADNP	3.9410582	3.9737096	4.0277815	0.0326514	0.0867233	0.0596874	up
A_24_P371425	ATRIP	-0.732585	-0.875984	-0.469899	-0.143399	0.2626863	0.0596437	up
A_23_P148273	MAGT1	3.0083103	3.0702033	3.0656738	0.061893	0.0573635	0.0596282	up
A_33_P3244921	VWA9	2.2480068	2.1968207	2.4183302	-0.051186	0.1703234	0.0595686	up
A_32_P133884	TUSC1	1.8936653	1.9015546	2.0048962	0.0078893	0.1112309	0.0595601	up
A_33_P3217393	CD276	-0.120022	-0.092683	-0.028319	0.0273385	0.0917025	0.0595205	up
A_33_P3226407	LOC649305	-1.350864	-1.445645	-1.137058	-0.094781	0.2138062	0.0595124	up
A_33_P3321136	DENND3	-2.585672	-2.301524	-2.751067	0.2841475	-0.165396	0.059376	up
A_23_P143643	ASCC2	0.7825828	0.7535505	0.9301786	-0.029032	0.1475959	0.0592818	up
A_24_P7652	MAP7D2	-2.936187	-2.921813	-2.832156	0.0143747	0.1040318	0.0592033	up
A_33_P3225273	QSOX1	-1.178846	-0.894071	-1.345496	0.2847753	-0.16665	0.0590627	up
A_23_P403284	OTX1	1.6991949	1.7656813	1.7507381	0.0664864	0.0515432	0.0590148	up
A_21_P0000352	SCARNA4	-0.955483	-0.910395	-0.882564	0.0450873	0.0729184	0.0590029	up
A_23_P149613	FMO1	-2.943083	-3.036047	-2.732347	-0.092964	0.2107356	0.0588859	up
A_33_P3313245	AMACR	-1.72926	-1.723724	-1.617172	0.0055361	0.1120882	0.0588121	up
A_33_P3815560	WDR6	1.9730339	1.97895	2.0843105	0.0059161	0.1112766	0.0585964	up
A_24_P381224	SYVN1	3.0796995	3.2754064	3.0011644	0.1957068	-0.078535	0.0585859	up
A_33_P3403107	PRR19	-0.066575	-0.133044	0.1169958	-0.066469	0.1835709	0.0585511	up
A_22_P00000406	LOC102724508	-0.811916	-0.789225	-0.717578	0.0226913	0.0943384	0.0585148	up
A_33_P3393801	PDZK1IP1	-2.263612	-2.185512	-2.224739	0.0781002	0.0388734	0.0584868	up
A_21_P0004531	LOC101929719	-2.817282	-2.828081	-2.689735	-0.010799	0.1275475	0.0583744	up
A_22_P00006487	LOC100506674	0.8203163	0.8734264	0.8839531	0.0531101	0.0636368	0.0583735	up
A_23_P416581	GNAZ	3.0240288	2.8990874	3.265499	-0.124941	0.2414703	0.0582645	up
A_23_P35316	ZNF695	-3.221487	-2.988003	-3.338573	0.233484	-0.117085	0.0581993	up
A_32_P385587	ALAS2	-2.691964	-2.371085	-2.896488	0.3208785	-0.204525	0.058177	up
A_23_P38468	TAX1BP3	1.4993811	1.4888663	1.6262445	-0.010515	0.1268635	0.0581744	up
A_23_P14302	LINC00341	0.6927586	0.8903012	0.611424	0.1975427	-0.081335	0.058104	up
A_24_P416159	RBL2	0.5907955	0.5918245	0.7059388	0.001029	0.1151433	0.0580862	up
A_33_P3239287	CHD3	3.6810274	4.025413	3.4527817	0.3443856	-0.228246	0.0580699	up
A_33_P3359250	MAD2L2	-1.928423	-1.768996	-1.971816	0.1594267	-0.043393	0.0580168	up
A_23_P112512	SLC25A51	1.0195556	1.1861205	0.968976	0.1665649	-0.05058	0.0579927	up
A_21_P0005351	lnc-CHST12-1	-0.93961	-0.7878	-0.975513	0.1518102	-0.035903	0.0579534	up
A_23_P397969	FOXK2	2.8122158	3.007691	2.732565	0.1954751	-0.079651	0.0579121	up
A_23_P348138	MKL1	1.2629337	1.2313781	1.4098773	-0.031556	0.1469436	0.057694	up
A_33_P3319463	NCOA4	1.8982239	1.8545213	2.057269	-0.043703	0.1590452	0.0576713	up
A_23_P136986	APOOL	2.376461	2.5290217	2.3392115	0.1525607	-0.03725	0.0576556	up
A_21_P0008535	lnc-DLGAP5-2	-1.967994	-1.747932	-2.073009	0.2200618	-0.105015	0.0575235	up
A_23_P398854	DOK7	5.7453337	5.460237	6.14542	-0.285097	0.4000864	0.0574949	up
A_23_P152272	RNPS1	4.305583	4.3017154	4.4244356	-0.003868	0.1188526	0.0574925	up
A_23_P404565	SLC2A11	-2.622082	-2.461116	-2.668098	0.1609666	-0.046016	0.0574754	up
A_23_P323924	ZFYVE27	5.9571447	5.9467864	6.0824327	-0.010358	0.125288	0.0574648	up
A_21_P0004866	LINC00240	0.0869556	0.2346506	0.0540886	0.1476951	-0.032867	0.0574141	up
A_33_P3210904	SEC13	-0.546514	-0.521481	-0.456729	0.0250335	0.0897846	0.057409	up
A_33_P3233550	RTN4	7.6810913	7.7306714	7.7462664	0.0495801	0.0651751	0.0573776	up
A_23_P146637	SIGMAR1	5.682373	5.6434274	5.836009	-0.038946	0.153636	0.0573452	up
A_23_P15305	PRPSAP1	3.812748	3.9414334	3.7987251	0.1286855	-0.014023	0.0573313	up

A_24_P348925	CCNK	1.5947056	1.6855974	1.6184163	0.0908918	0.0237107	0.0573013	up
A_24_P146670	SLK	1.637826	1.9326234	1.4576302	0.2947974	-0.180196	0.0573008	up
A_23_P402000	ZNF527	-1.314926	-1.305057	-1.210274	0.0098686	0.1046519	0.0572603	up
A_22_P00010979	DHRS4-AS1	0.6504674	0.5681911	0.8470449	-0.082276	0.1965776	0.0571506	up
A_33_P3336437	TTLL5	-1.61318	-1.685214	-1.427043	-0.072034	0.1861372	0.0570517	up
A_33_P3383283	CASP10	3.339775	3.4902158	3.3034296	0.1504407	-0.036345	0.0570476	up
A_33_P3377194	ADRA1A	1.0973601	1.2179604	1.0907059	0.1206002	-0.006654	0.056973	up
A_33_P3264780	CDK8	-0.210322	-0.257315	-0.049441	-0.046992	0.160881	0.0569444	up
A_23_P423389	CREB3	1.912384	1.9687696	1.9694395	0.0563855	0.0570555	0.0567205	up
A_23_P13683	ZBTB39	-0.798129	-0.547431	-0.935593	0.2506976	-0.137464	0.056617	up
A_23_P208416	SLC35E1	3.1129103	3.3633828	2.975504	0.2504726	-0.137406	0.0565331	up
A_23_P120270	MCFD2	4.1539545	4.44039	3.9805727	0.2864356	-0.173382	0.0565269	up
A_23_P14273	ZFYVE21	4.391369	4.321857	4.573925	-0.069512	0.1825562	0.0565221	up
A_23_P51376	NKAIN1	-1.363887	-1.692091	-0.922713	-0.328204	0.4411736	0.0564849	up
A_23_P147641	TCEA2	1.0455112	1.2766814	0.9272695	0.2311702	-0.118242	0.0564642	up
A_23_P34066	IL9R	-0.838779	-0.809686	-0.754983	0.0290923	0.0837956	0.0564439	up
A_33_P3294237	MTF1	2.1883354	2.208713	2.2807913	0.0203776	0.0924559	0.0564168	up
A_23_P422831	FAM189A2	0.0667944	0.2127571	0.0335822	0.1459627	-0.033212	0.0563753	up
A_23_P158007	ZDHHC12	3.0038843	2.8398433	3.280653	-0.164041	0.2767687	0.0563638	up
A_33_P3418917	RUSC1-AS1	1.3314605	1.1864934	1.5890083	-0.144967	0.2575479	0.0562904	up
A_23_P29922	TLR3	-0.381718	-0.460985	-0.189962	-0.079267	0.1917558	0.0562446	up
A_23_P115046	EIF2B3	2.2679958	2.1801963	2.468254	-0.0878	0.2002583	0.0562294	up
A_23_P117546	SOS2	-0.018599	0.2118254	-0.136882	0.2304244	-0.118283	0.0560706	up
A_23_P71433	UBE2W	3.00173	3.1364198	2.9791698	0.1346898	-0.02256	0.0560648	up
A_33_P3280779	TMEM254	-2.227627	-2.140133	-2.203021	0.0874934	0.0246058	0.0560496	up
A_23_P167497	EFNA5	-2.349014	-2.454554	-2.131424	-0.10554	0.2175899	0.0560249	up
A_23_P391275	RCAN3	0.7288961	1.011282	0.5585322	0.2823858	-0.170364	0.056011	up
A_24_P273143	LINC00152	5.7719793	5.8188586	5.83712	0.0468793	0.0651407	0.05601	up
A_33_P3213377	IMMP2L	0.7097564	0.6278167	0.9036355	-0.08194	0.1938791	0.0559697	up
A_24_P287826	RFWD3	2.7777004	2.718287	2.9488611	-0.059413	0.1711607	0.0558736	up
A_24_P71153	PAFAH2	-0.936114	-0.917106	-0.843523	0.0190086	0.0925918	0.0558002	up
A_33_P3359590	DAZAP1	-2.696117	-2.51416	-2.76657	0.1819563	-0.070453	0.0557516	up
A_24_P216654	SOAT1	-0.506139	-0.512518	-0.388265	-0.006379	0.1178737	0.0557473	up
A_23_P23171	AGO4	-0.259224	-0.283214	-0.12376	-0.02399	0.1354642	0.055737	up
A_21_P0010287	lnc-RSPH1-4	-1.308937	-1.214342	-1.292152	0.0945945	0.0167842	0.0556893	up
A_33_P3418371	EPWW6493	-0.861043	-0.914057	-0.696672	-0.053014	0.1643705	0.0556781	up
A_23_P209933	TGOLN2	3.350091	3.4716182	3.3398876	0.1215272	-0.010203	0.0556619	up
A_23_P153026	GAA	5.787219	5.8702903	5.8154116	0.0830712	0.0281925	0.0556319	up
A_23_P56140	CSNK1G2	2.0224028	1.8998985	2.2560282	-0.122504	0.2336254	0.0555606	up
A_23_P359904	PDDC1	4.9152746	4.941734	4.999896	0.0264592	0.0846214	0.0555403	up
A_23_P143692	SH3BP1	-0.45384	-0.71472	-0.081916	-0.26088	0.3719239	0.0555217	up
A_21_P0000312	SNORA14B	-1.725235	-1.590176	-1.750005	0.1350589	-0.02477	0.0551445	up
A_23_P152791	SLC16A6	0.5334411	0.348033	0.8291121	-0.185408	0.295671	0.0551314	up
A_23_P130482	ZNF211	-1.166579	-1.121667	-1.101262	0.0449119	0.0653172	0.0551145	up
A_23_P166633	ITGB5	3.7344866	3.7334094	3.845746	-0.001077	0.1112595	0.0550911	up
A_33_P3666884	PNPLA7	0.4492345	0.7328291	0.2758045	0.2835946	-0.17343	0.0550823	up
A_23_P118888	PAFAH1B1	3.6254826	3.7309213	3.630206	0.1054387	0.0047235	0.0550811	up
A_23_P254081	LIAS	0.1270104	0.1659732	0.198185	0.0389628	0.0711746	0.0550687	up
A_23_P70409	POLR1C	2.9825315	3.032188	3.0428896	0.0496564	0.060358	0.0550072	up
A_24_P56484	BRMS1L	-1.167156	-1.117793	-1.106671	0.0493627	0.0604844	0.0549235	up
A_23_P255601	TMEM185A	1.037365	1.056531	1.1279149	0.019166	0.0905499	0.054858	up
A_23_P5831	HPCAL1	2.6092825	2.4721417	2.8560953	-0.137141	0.2468128	0.054836	up
A_23_P313632	FUT8	1.2615781	1.2508864	1.3818994	-0.010692	0.1203213	0.0548148	up
A_23_P327426	TIPRL	4.5223417	4.5627046	4.591481	0.0403628	0.0691395	0.0547512	up
A_21_P0013753	XLOC_l2_015451	6.141986	6.1222816	6.271158	-0.019704	0.1291723	0.054734	up
A_24_P42501	ACOT9	3.097229	3.3419662	2.9619112	0.2447372	-0.135318	0.0547097	up

A_24_P382026	MIF4GD	0.7165885	0.7476587	0.794867	0.0310702	0.0782785	0.0546744	up
A_23_P148015	AXIN2	4.315755	4.455975	4.284773	0.1402202	-0.030982	0.0546191	up
A_23_P66872	GEMIN4	3.3613186	3.2036624	3.62817	-0.157656	0.2668514	0.0545976	up
A_23_P93499	TFB1M	3.1410685	3.0982308	3.292964	-0.042838	0.1518955	0.054529	up
A_23_P91764	TNFRSF13C	-2.884414	-2.639947	-3.01989	0.244467	-0.135476	0.0544955	up
A_22_P00006335	lnc-FANCI-1	-1.060705	-0.880957	-1.131497	0.1797476	-0.070792	0.0544777	up
A_33_P3268487	ABAT	2.0204296	2.0225987	2.1271067	0.0021691	0.1066771	0.0544231	up
A_33_P3332492	FANK1	-0.423022	-0.198832	-0.538381	0.2241898	-0.115359	0.0544152	up
A_22_P00017754	lnc-YIF1A-6	-2.565167	-2.858702	-2.162892	-0.293535	0.4022749	0.0543698	up
A_23_P97853	TMEM254	1.5007472	1.5783653	1.5318642	0.0776181	0.031117	0.0543675	up
A_23_P60248	TXN	9.345625	9.358906	9.441002	0.0132809	0.095377	0.0543289	up
A_21_P0004717	lnc-SERPINB6-2	-2.81241	-2.798299	-2.717951	0.0141103	0.0944591	0.0542847	up
A_23_P27035	TMEM98	4.133971	4.345426	4.0310173	0.2114549	-0.102954	0.0542505	up
A_21_P0011380	lnc-STOML1-1	-2.006094	-1.790358	-2.113351	0.2157354	-0.107258	0.0542388	up
A_23_P211126	DYRK1A	1.7896366	2.0390754	1.6486573	0.2494388	-0.140979	0.0542297	up
A_33_P3225882	BET1L	-1.52795	-1.245989	-1.701651	0.2819605	-0.173701	0.0541298	up
A_32_P187663	ZNF596	-2.209649	-2.195764	-2.115295	0.0138855	0.0943537	0.0541196	up
A_22_P00005678	EIF4ENIF1	1.31843	1.3919802	1.3530922	0.0735502	0.0346622	0.0541062	up
A_33_P3270451	TXNDC5	0.7244997	0.8828082	0.6742911	0.1583085	-0.050209	0.05405	up
A_23_P37514	C15orf39	3.8569736	3.7610412	4.0608587	-0.095932	0.2038851	0.0539763	up
A_23_P38011	DHX38	1.7473364	1.9218793	1.6806049	0.1745429	-0.066731	0.0539057	up
A_24_P283928	NABP2	1.1223483	1.1220465	1.2303991	-0.000302	0.1080508	0.0538745	up
A_24_P173754	C1orf21	-1.56037	-1.923872	-1.089171	-0.363502	0.471199	0.0538485	up
A_21_P0000489	SNORD104	3.9224472	3.9137683	4.0387077	-0.008679	0.1162605	0.0537908	up
A_23_P123974	DTYMK	6.772045	6.7707	6.880947	-0.001345	0.108902	0.0537784	up
A_33_P3261937	RNASE13	-1.512239	-1.312084	-1.605014	0.2001543	-0.092775	0.0536895	up
A_33_P3349495	TTTY16	-1.656176	-1.542002	-1.66299	0.1141744	-0.006814	0.0536804	up
A_19_P00322711	LINC00662	0.5787015	0.5956531	0.6690788	0.0169516	0.0903773	0.0536644	up
A_23_P104073	S100A3	-0.885174	-0.779932	-0.883169	0.1052423	0.0020056	0.0536239	up
A_21_P0001664	LOC100505887	-2.951915	-2.79016	-3.006491	0.1617544	-0.054576	0.0535893	up
A_23_P28538	MRPS5	2.856845	2.9578233	2.8630114	0.1009784	0.0061665	0.0535724	up
A_33_P3298567	ZNF692	-2.121223	-1.801513	-2.333867	0.3197098	-0.212644	0.0535327	up
A_33_P3308132	LOC102725127	0.818748	0.852448	0.8919449	0.0337	0.0731969	0.0534484	up
A_21_P0013535	XLOC_l2_014123	-2.541044	-2.59207	-2.383223	-0.051026	0.1578209	0.0533973	up
A_23_P206140	DNAJA4	4.9383106	5.0817466	4.9016657	0.143436	-0.036645	0.0533955	up
A_33_P3303464	SWSAP1	-1.493435	-1.492538	-1.387761	0.0008979	0.1056743	0.0532861	up
A_23_P145089	HSP90AB1	5.969576	6.0526576	5.9929733	0.0830817	0.0233974	0.0532396	up
A_23_P115762	ECD	3.1099672	3.0067306	3.3196812	-0.103237	0.2097139	0.0532386	up
A_23_P309515	KCTD18	-1.875474	-1.732957	-1.911594	0.1425166	-0.036121	0.0531979	up
A_33_P3414058	MEN1	0.5559096	0.634738	0.5832863	0.0788283	0.0273767	0.0531025	up
A_33_P3212645	NOTCH2NL	4.111188	4.1434646	4.184985	0.0322766	0.0737972	0.0530369	up
A_33_P3261084	IDH3B	2.5132294	2.4534936	2.6790152	-0.059736	0.1657858	0.053025	up
A_23_P165668	SLC35F5	-2.548406	-2.649907	-2.340866	-0.101501	0.2075408	0.0530199	up
A_24_P56270	DYRK2	2.9757261	3.2543359	2.8031254	0.2786098	-0.172601	0.0530045	up
A_23_P42738	FAM220A	2.0556831	2.1172214	2.1000395	0.0615382	0.0443563	0.0529473	up
A_24_P152404	C10orf76	0.3842278	0.2038798	0.6704187	-0.180348	0.286191	0.0529215	up
A_23_P3527	POLR2C	3.8211164	3.796845	3.9512138	-0.024271	0.1300974	0.052913	up
A_33_P3318763	LOC101927497	-2.500181	-2.006621	-2.887947	0.4935598	-0.387766	0.0528969	up
A_23_P42331	HMGA1	5.5496197	5.6566863	5.54834	0.1070666	-0.00128	0.0528934	up
A_33_P3251289	VIMP	2.773902	3.0302205	2.6231852	0.2563186	-0.150717	0.0528009	up
A_33_P3382380	UBQLN4	1.5141821	1.6363797	1.4975758	0.1221976	-0.016606	0.0527957	up
A_23_P132936	SPCS3	5.066284	4.9950585	5.243002	-0.071226	0.1767178	0.0527461	up
A_33_P3404126	PCGF3	0.562901	0.364615	0.8665838	-0.198286	0.3036828	0.0526984	up
A_22_P00003668	lnc-CDC42EP1-1	-2.7092	-3.003007	-2.310058	-0.293807	0.399142	0.0526676	up
A_22_P00008401	lnc-JPH1-3	-1.256903	-1.34362	-1.065005	-0.086717	0.1918984	0.0525906	up
A_23_P61881	ARIH2	4.038724	4.001186	4.181388	-0.037538	0.142664	0.052563	up

A_23_P3532	LITAF	3.7410917	3.6922421	3.8950472	-0.04885	0.1539555	0.0525529	up
A_33_P3210278	SYNE2	3.994197	4.066687	4.0267973	0.0724902	0.0326004	0.0525453	up
A_22_P00021447	lnc-NPB-1	-2.44473	-2.567007	-2.217391	-0.122277	0.2273395	0.0525314	up
A_33_P3231363	STK3	-1.074608	-0.754911	-1.289412	0.3196974	-0.214804	0.0524468	up
A_33_P3411885	LOC100128851	0.6053906	0.5802016	0.7348766	-0.025189	0.1294861	0.0521486	up
A_33_P3223472	AMZ2	4.232584	4.279177	4.290242	0.0465932	0.0576582	0.0521257	up
A_24_P67189	SPEM1	-2.877941	-2.562709	-3.088979	0.315232	-0.211038	0.0520971	up
A_19_P00808774	BAIAP2-AS1	-1.214072	-1.145831	-1.178153	0.0682411	0.0359192	0.0520802	up
A_23_P252322	ATP5E	9.528354	9.53348	9.627056	0.005126	0.0987024	0.0519142	up
A_33_P3369786	UBE3D	-2.355415	-2.484203	-2.122811	-0.128788	0.232604	0.0519081	up
A_23_P21425	GPR119	-0.524903	-0.400779	-0.545318	0.1241236	-0.020415	0.0518541	up
A_33_P3220277	RRBP1	2.660142	2.5904279	2.8335238	-0.069714	0.1733818	0.0518339	up
A_23_P133629	CDC23	3.307004	3.3000283	3.4175787	-0.006976	0.1105747	0.0517995	up
A_24_P191656	ACAD9	2.2324162	2.2981544	2.2702694	0.0657382	0.0378532	0.0517957	up
A_22_P00001001	lnc-AL353698.1-1	1.4151793	1.2871327	1.6466928	-0.128047	0.2315135	0.0517335	up
A_23_P203255	API5	-0.281635	-0.379201	-0.080618	-0.097566	0.2010174	0.0517256	up
A_23_P363968	C1RL	-0.41579	-0.237545	-0.490678	0.1782446	-0.074888	0.0516782	up
A_23_P39813	TTC31	-1.508007	-1.946436	-0.966263	-0.438428	0.5417442	0.0516579	up
A_33_P3339536	EWSR1	4.488702	4.3933015	4.687333	-0.0954	0.1986313	0.0516155	up
A_23_P73457	RUFY1	4.4249268	4.534661	4.418292	0.1097341	-0.006635	0.0515497	up
A_21_P0013240	GTF2IRD1	-0.697586	-0.756239	-0.53584	-0.058653	0.1617465	0.0515468	up
A_33_P3300430	FBN3	0.0836592	0.4487257	-0.178526	0.3650665	-0.262185	0.0514407	up
A_33_P3239884	NCAM2	-2.264469	-2.231386	-2.194795	0.033083	0.0696743	0.0513786	up
A_23_P259594	AKAP7	-0.464128	-0.192749	-0.632821	0.271379	-0.168694	0.0513427	up
A_33_P3428642	DMWD	0.6931114	0.5792899	0.9096074	-0.113822	0.216496	0.0513372	up
A_22_P00020611	lnc-PRL-2	-1.438475	-1.328042	-1.44641	0.1104331	-0.007935	0.0512493	up
A_23_P40039	AMMECR1L	3.7954302	3.794784	3.8985033	-0.000646	0.1030731	0.0512135	up
A_33_P3280945	SNHG3	6.086771	5.974794	6.3011713	-0.111977	0.2144003	0.0512116	up
A_33_P3683362	lnc-RNF125-2	-2.433826	-2.792968	-1.972274	-0.359141	0.4615526	0.0512056	up
A_23_P108751	FHL2	5.901532	6.0049014	5.900569	0.1033692	-0.000963	0.051203	up
A_23_P399797	SMAD5-AS1	-2.562458	-2.61671	-2.405913	-0.054253	0.1565449	0.0511461	up
A_24_P409346	NPEPPS	0.864789	1.0077095	0.8241334	0.1429205	-0.040656	0.0511324	up
A_33_P3323101	ZNF33A	1.2267342	1.3848925	1.1706767	0.1581583	-0.056057	0.0510504	up
A_33_P3296303	CHMP5	4.328431	4.4160924	4.342677	0.0876613	0.014246	0.0509536	up
A_33_P3419720	MLH1	4.0938597	4.1826673	4.1069355	0.0888076	0.0130758	0.0509417	up
A_21_P0008617	lnc-THBS1-2	-2.706935	-2.753015	-2.558982	-0.04608	0.1479523	0.0509362	up
A_21_P0000014	FAN1	-2.820628	-2.953575	-2.585956	-0.132948	0.2346714	0.0508618	up
A_33_P3300975	HOXC4	2.9192228	2.7546554	3.1854105	-0.164567	0.2661877	0.0508101	up
A_23_P137470	SIPA1L2	2.6621342	2.9359717	2.4898558	0.2738376	-0.172278	0.0507796	up
A_21_P0005923	LOC100506990	-1.364971	-1.279925	-1.348536	0.0850463	0.0164356	0.050741	up
A_21_P0013882	XLOC_l2_015894	-2.646207	-2.51538	-2.675654	0.1308277	-0.029446	0.0506908	up
A_24_P272225	ASH1L-AS1	1.965766	1.85994	2.1729326	-0.105826	0.2071667	0.0506704	up
A_32_P448360	ASB16-AS1	0.0442867	0.0315499	0.1581993	-0.012737	0.1139126	0.0505879	up
A_23_P10156	CHMP6	0.7188501	0.5939002	0.9448228	-0.12495	0.2259727	0.0505114	up
A_22_P00018347	ZNF213-AS1	-1.772183	-1.458039	-1.985316	0.3141437	-0.213133	0.0505052	up
A_23_P148372	CSTF2	3.1661043	3.2031226	3.2300863	0.0370183	0.063982	0.0505002	up
A_24_P230721	WDR73	0.655026	0.6741753	0.7365289	0.0191493	0.0815029	0.0503261	up
A_33_P3305790	NOS3	-0.384014	-0.463075	-0.20434	-0.079061	0.1796742	0.0503068	up
A_21_P0000464	SNORD123	-2.906295	-2.881595	-2.830686	0.0246999	0.0756092	0.0501546	up
A_24_P23245	NDUFA6	6.604767	6.6533494	6.6561785	0.0485826	0.0514116	0.0499971	up
A_22_P00001478	JRK	1.6370687	1.6593766	1.7147017	0.0223079	0.0776329	0.0499704	up
A_23_P102122	ARPC2	7.641076	7.5693784	7.812584	-0.071698	0.1715078	0.0499051	up
A_33_P3403418	CTNNA1	2.633296	2.698748	2.6676035	0.0654521	0.0343075	0.0498798	up
A_22_P00007735	VPS11	-0.579613	-0.373945	-0.68562	0.2056685	-0.106007	0.0498309	up
A_23_P64990	RAD52	0.0379529	0.0821223	0.0932884	0.0441694	0.0553355	0.0497525	up
A_24_P551842	CYTB	9.282012	9.470989	9.19251	0.1889772	-0.089502	0.0497375	up

A_24_P230948	PER3	-0.836601	-0.559517	-1.014335	0.2770844	-0.177733	0.0496755	up
A_23_P170820	PUM1	4.5477753	4.607631	4.5872526	0.0598559	0.0394774	0.0496666	up
A_23_P89543	KIAA0753	1.1177745	1.1537375	1.181109	0.0359631	0.0633345	0.0496488	up
A_23_P18993	MIER3	-2.799337	-2.923342	-2.576232	-0.124005	0.2231047	0.0495498	up
A_23_P61569	VAMP4	-1.547539	-1.616686	-1.379305	-0.069147	0.1682334	0.0495431	up
A_23_P114084	PHEX	-2.801646	-2.683758	-2.820498	0.117888	-0.018852	0.0495179	up
A_24_P42436	ARHGAP32	-0.36678	-0.298977	-0.335568	0.0678024	0.0312114	0.0495069	up
A_33_P3389638	NOP14	4.1198034	4.0916057	4.2469864	-0.028198	0.127183	0.0494926	up
A_33_P3381948	WTIP	3.0208368	2.916615	3.2240038	-0.104222	0.203167	0.0494726	up
A_22_P00002209	LINC00847	-2.736892	-2.414931	-2.960111	0.3219612	-0.223219	0.049371	up
A_23_P422193	SUV39H1	1.0077453	0.7993584	1.3146577	-0.208387	0.3069124	0.0492628	up
A_33_P3282390	GRM4	-3.056451	-2.766016	-3.248517	0.2904356	-0.192066	0.0491849	up
A_24_P150486	SPTLC2	0.0062709	0.1052251	0.0056667	0.0989542	-0.000604	0.049175	up
A_33_P3388865	LRRC10	-2.1823	-2.051651	-2.214736	0.1306491	-0.032435	0.0491068	up
A_23_P396666	TBC1D2B	2.9834547	2.9872804	3.0776205	0.0038257	0.0941658	0.0489957	up
A_24_P827491	PA2G4	2.803276	2.7442222	2.9600983	-0.059054	0.1568222	0.0488842	up
A_24_P416961	ARVCF	-0.074199	-0.110609	0.0598989	-0.03641	0.1340976	0.0488436	up
A_33_P3335746	SNAPC5	-1.665734	-1.531045	-1.702772	0.1346898	-0.037037	0.0488262	up
A_24_P320254	HNRNPH1	5.757471	5.8837085	5.7288017	0.1262374	-0.028669	0.048784	up
A_23_P352358	KIAA2013	3.4661522	3.394217	3.635622	-0.071935	0.1694698	0.0487673	up
A_19_P00322737	ZNF254	-2.563646	-2.360863	-2.668932	0.2027829	-0.105286	0.0487486	up
A_24_P415280	SEC61A2	-1.326932	-1.144121	-1.41232	0.1828108	-0.085388	0.0487113	up
A_24_P9090	HNRNPDL	4.0647955	4.119222	4.1077394	0.0544267	0.042944	0.0486853	up
A_23_P202750	C11orf54	0.980998	1.1337667	0.9255633	0.1527686	-0.055435	0.048667	up
A_23_P93389	NUDT3	1.3721719	1.4340968	1.407546	0.0619249	0.0353742	0.0486495	up
A_33_P3349546	DCAF17	1.8155298	2.043982	1.6842785	0.2284522	-0.131251	0.0486004	up
A_23_P255317	RNF14	1.4387875	1.4089913	1.5657067	-0.029796	0.1269193	0.0485616	up
A_23_P70148	NNT	0.2584505	0.2644343	0.3495197	0.0059838	0.0910692	0.0485265	up
A_22_P00010918	LINC01011	-3.039171	-2.814132	-3.167185	0.2250388	-0.128015	0.0485121	up
A_21_P0012703	XLOC_l2_011415	-1.819032	-1.491758	-2.049497	0.3272739	-0.230465	0.0484045	up
A_33_P3243707	KLHL20	0.6260967	0.7058506	0.643137	0.0797539	0.0170403	0.0483971	up
A_33_P3330283	SP1	2.2374983	2.2114158	2.36032	-0.026083	0.1228218	0.0483696	up
A_24_P320759	PRKRIP1	0.8231063	0.9200482	0.822885	0.096942	-0.000221	0.0483603	up
A_33_P3806721	KDM8	1.0239224	1.0206399	1.1238527	-0.003283	0.0999303	0.0483239	up
A_32_P197561	EBF1	-0.418117	-0.739866	0.0002246	-0.32175	0.4183412	0.0482957	up
A_33_P3226600	GPAA1	6.506069	6.4262414	6.68231	-0.079828	0.1762409	0.0482066	up
A_24_P928217	FAM134A	-0.9657	-0.816399	-1.018616	0.1493015	-0.052916	0.048193	up
A_24_P106681	YWHAG	7.095806	6.977019	7.310912	-0.118787	0.215106	0.0481594	up
A_22_P00006201	SMKR1	-0.279649	-0.493416	0.0304289	-0.213767	0.3100781	0.0481555	up
A_23_P106835	BBS2	1.7542162	1.8085394	1.7960682	0.0543232	0.041852	0.0480876	up
A_33_P3407414	LINC01278	-0.812456	-0.970291	-0.558464	-0.157835	0.2539921	0.0480783	up
A_33_P3542911	SNAR-C4	-0.173245	-0.056109	-0.194345	0.117136	-0.0211	0.0480182	up
A_33_P3370404	PANX1	3.772313	3.7296615	3.910985	-0.042652	0.1386719	0.0480101	up
A_23_P302568	SLC30A3	-1.049763	-1.163662	-0.839983	-0.113899	0.2097793	0.04794	up
A_23_P390722	TOR1AIP2	-0.064114	-0.180562	0.1481805	-0.116447	0.2122946	0.0479236	up
A_23_P102890	MRPS6	6.353035	6.154469	6.6472692	-0.198566	0.2942343	0.0478342	up
A_23_P153050	ALKBH5	2.5048256	2.4746194	2.630557	-0.030206	0.1257315	0.0477626	up
A_33_P3499692	LOC645261	2.4909582	2.6342835	2.4430895	0.1433253	-0.047869	0.0477283	up
A_33_P3864411	ACTR3BP5	-0.697345	-0.568514	-0.730788	0.1288309	-0.033443	0.047694	up
A_23_P90895	PPP1R21	-1.224063	-1.156712	-1.196075	0.0673513	0.0279884	0.0476699	up
A_33_P3422701	YEATS2	-2.523558	-2.424629	-2.527256	0.0989289	-0.003698	0.0476155	up
A_23_P135454	AFG3L2	2.0586576	2.189693	2.0228357	0.1310353	-0.035822	0.0476067	up
A_23_P68401	CTNNBL1	1.7842598	1.9157166	1.7479186	0.1314569	-0.036341	0.0475578	up
A_23_P250294	ABHD5	2.4585161	2.5443711	2.4677172	0.085855	0.0092011	0.047528	up
A_33_P3601163	ILF3-AS1	3.8949451	3.6509023	4.233982	-0.244043	0.3390369	0.047497	up
A_23_P167201	SEPSECS	-2.731318	-2.660628	-2.707171	0.0706892	0.0241463	0.0474178	up

A_33_P3329878	RNF216	2.8315115	2.76131	2.9963236	-0.070201	0.1648121	0.0473053	up
A_21_P0000248	SNORA66	-1.292576	-1.461234	-1.029346	-0.168658	0.2632299	0.0472858	up
A_22_P00011411	USP27X-AS1	-2.754788	-2.904805	-2.510211	-0.150017	0.2445767	0.04728	up
A_21_P0014256	STK32C	3.2862358	3.2748313	3.3920279	-0.011405	0.105792	0.0471938	up
A_22_P00022877	lnc-IL15RA-1	-0.500814	-0.568383	-0.338882	-0.067569	0.161932	0.0471816	up
A_23_P218111	SERPINA1	3.2016525	3.3153253	3.1823263	0.1136727	-0.019326	0.0471733	up
A_24_P95822	NPTN	2.571683	2.5682158	2.6694107	-0.003467	0.0977278	0.0471303	up
A_24_P280762	KHDC1	1.1442013	1.1508112	1.2317758	0.0066099	0.0875745	0.0470922	up
A_33_P3257714	RPS23	4.052454	4.1614013	4.0376854	0.1089473	-0.014769	0.0470893	up
A_23_P128974	BATF	3.9006948	3.88442	4.0111065	-0.016275	0.1104116	0.0470684	up
A_19_P00801867	LOC100129434	-2.447348	-2.368885	-2.431815	0.0784638	0.0155332	0.0469985	up
A_23_P113417	C17orf70	2.104147	2.0954614	2.2067995	-0.008686	0.1026526	0.0469835	up
A_33_P3369766	MED4	2.478695	2.4721208	2.579031	-0.006574	0.1003361	0.046881	up
A_23_P162165	KCTD14	-0.028788	-0.355613	0.391779	-0.326825	0.420567	0.0468712	up
A_19_P00812587	RPL5	7.4526234	7.612536	7.386422	0.1599126	-0.066201	0.0468557	up
A_23_P215154	NUB1	1.6687851	1.702693	1.7284164	0.0339079	0.0596313	0.0467696	up
A_23_P112061	HGSNAT	3.6115074	3.6065612	3.7099628	-0.004946	0.0984554	0.0467546	up
A_22_P00017616	TOB1-AS1	-0.745159	-0.468154	-0.928775	0.2770047	-0.183616	0.0466945	up
A_24_P191417	NAB1	2.7622213	2.8748755	2.7429028	0.1126542	-0.019319	0.0466678	up
A_23_P3368	FURIN	0.3755198	0.4160619	0.4282742	0.0405421	0.0527544	0.0466483	up
A_23_P368681	GIMAP2	-1.449296	-1.339716	-1.465718	0.1095791	-0.016423	0.0465782	up
A_23_P152353	EARS2	0.9823413	0.8849092	1.1727171	-0.097432	0.1903758	0.0464718	up
A_23_P217120	EHMT1	2.7344646	2.8865504	2.6752224	0.1520858	-0.059242	0.0464218	up
A_33_P3345663	PPP1R21	-0.853856	-0.849303	-0.765759	0.0045533	0.0880966	0.046325	up
A_24_P398898	MORF4L1	6.7220917	6.914994	6.6216784	0.1929021	-0.100413	0.0462444	up
A_33_P3226710	C7orf13	-0.719553	-0.684992	-0.661636	0.0345607	0.0579171	0.0462389	up
A_33_P3289296	TMEM37	0.9467931	0.8257413	1.1602783	-0.121052	0.2134852	0.0462167	up
A_33_P3258061	PALM3	3.5068474	3.6189675	3.4871407	0.1121202	-0.019707	0.0462067	up
A_33_P3268005	FAM120AOS	-0.401385	-0.146475	-0.563927	0.2549095	-0.162542	0.0461838	up
A_23_P77714	CLUAP1	1.8011093	2.005282	1.6892991	0.2041726	-0.11181	0.0461812	up
A_22_P00009322	lnc-LRRC57-1	-2.720081	-2.612202	-2.735599	0.1078794	-0.015517	0.046181	up
A_33_P3321657	HSPG2	4.430479	4.591203	4.362093	0.1607242	-0.068386	0.046169	up
A_23_P9761	CNTROB	0.4938135	0.4120431	0.6678648	-0.08177	0.1740513	0.0461404	up
A_22_P00001620	LOC101928809	-2.651869	-2.53752	-2.67407	0.1143484	-0.022201	0.0460738	up
A_22_P00011020	lnc-NT5C-2	-3.07835	-3.043222	-3.021434	0.0351284	0.0569162	0.0460223	up
A_24_P835500	ITPRIPL2	4.356529	4.2750173	4.5300455	-0.081512	0.1735163	0.0460021	up
A_23_P425066	CRYBB2	2.7776194	2.6829133	2.964262	-0.094706	0.1866427	0.0459683	up
A_23_P48717	NPC2	7.6952944	7.821858	7.6604357	0.1265636	-0.034859	0.0458524	up
A_23_P137057	SLC25A5	8.248217	8.362705	8.225397	0.1144886	-0.02282	0.0458345	up
A_23_P43566	NDUFA8	7.3305664	7.3604913	7.3922815	0.0299249	0.0617151	0.04582	up
A_32_P831181	BRI3BP	2.6556568	2.6330342	2.769824	-0.022623	0.1141672	0.0457723	up
A_24_P16610	ZNRF1	0.0472813	-0.090237	0.2762704	-0.137518	0.2289891	0.0457356	up
A_23_P117599	AHSA1	5.012822	4.9680657	5.1490145	-0.044756	0.1361923	0.045718	up
A_23_P122041	PPP2CA	3.6339445	3.55615	3.8028202	-0.077795	0.1688757	0.0455406	up
A_23_P67127	TMEM145	-1.344557	-1.488967	-1.109072	-0.14441	0.2354851	0.0455375	up
A_23_P350555	TCP10L	1.4852233	1.5294285	1.5319824	0.0442052	0.0467591	0.0454822	up
A_22_P00025843	lnc-CHAD-5	-2.094653	-1.982053	-2.116337	0.1125999	-0.021685	0.0454576	up
A_22_P00003284	MIR205HG	0.1724949	0.2937079	0.1421247	0.121213	-0.03037	0.0454214	up
A_24_P218979	CDCA3	3.266367	3.2915578	3.3320112	0.0251908	0.0656443	0.0454175	up
A_33_P3379046	LOC729770	-0.551625	-0.412508	-0.600003	0.1391172	-0.048378	0.0453696	up
A_23_P150080	RPP38	4.0030413	3.9321208	4.164588	-0.07092	0.1615467	0.0453131	up
A_23_P154605	SULF2	3.8704605	3.7472568	4.084258	-0.123204	0.2137976	0.0452969	up
A_23_P91657	EWSR1	3.5989952	3.7477307	3.5408487	0.1487355	-0.058146	0.0452945	up
A_33_P3367073	LOC101928991	-0.179147	-0.180657	-0.087536	-0.001511	0.0916104	0.0450499	up
A_21_P0000528	PRR26	-2.183098	-2.368446	-1.907707	-0.185348	0.2753906	0.0450214	up
A_23_P37191	PSMB5	5.5462465	5.295222	5.887311	-0.251025	0.3410645	0.0450199	up

A_22_P00011640	lnc-PDCD1-2	-2.14323	-2.354942	-1.841617	-0.211712	0.3016133	0.0449508	up
A_33_P3284472	DDX51	0.9822559	0.9152722	1.1391292	-0.066984	0.1568732	0.0449448	up
A_23_P207850	TNS4	6.33123	6.1504483	6.6018896	-0.180782	0.2706595	0.0449388	up
A_24_P240065	CHMP3	3.8033	3.917316	3.7791567	0.1140161	-0.024143	0.0449364	up
A_33_P3611762	TRAM2-AS1	-0.043178	0.0351019	-0.031675	0.0782795	0.0115027	0.0448911	up
A_23_P406928	ND5	5.3990316	5.5745654	5.31326	0.1755338	-0.085772	0.0448811	up
A_23_P30666	TNFRSF21	3.3797512	3.4590106	3.390234	0.0792594	0.0104828	0.0448711	up
A_23_P304543	NFX1	1.674119	1.7292552	1.708683	0.0551362	0.034564	0.0448501	up
A_22_P00009478	lnc-MAGEA4-1	-0.791166	-0.669863	-0.822771	0.1213031	-0.031604	0.0448494	up
A_22_P00022417	lnc-SFI1-1	-1.746818	-1.422944	-1.981217	0.3238745	-0.234399	0.0447378	up
A_23_P205818	PPIP5K1	1.3368316	1.3244262	1.4386826	-0.012405	0.101851	0.0447228	up
A_23_P28057	THOP1	6.044219	6.098884	6.078991	0.0546651	0.0347719	0.0447185	up
A_23_P8119	ZBTB9	-0.957222	-1.084009	-0.741	-0.126787	0.2162223	0.0447176	up
A_32_P235159	MSL3P1	3.0347643	3.0821557	3.0767994	0.0473914	0.0420351	0.0447133	up
A_24_P111912	FAM172A	2.2884922	2.3746848	2.2914925	0.0861926	0.0030003	0.0445964	up
A_23_P257278	CDK5RAP1	0.9937577	1.1143227	0.962347	0.1205649	-0.031411	0.0445771	up
A_33_P3238058	LINC01101	-1.045378	-0.897469	-1.104136	0.1479096	-0.058758	0.0445759	up
A_33_P3401452	CHD6	-2.675885	-2.373279	-2.889406	0.3026061	-0.213521	0.0445426	up
A_22_P00020522	RNF216-IT1	-2.024111	-2.029711	-1.929561	-0.005599	0.0945501	0.0444753	up
A_33_P3400263	CHRAC1	1.81423	2.0547204	1.6626301	0.2404904	-0.1516	0.0444453	up
A_33_P3262020	C8G	-0.688501	-0.682423	-0.605842	0.0060778	0.0826588	0.0443683	up
A_33_P3332348	RN7SL1	7.421856	7.428674	7.5036135	0.0068183	0.0817575	0.0442879	up
A_19_P00322828	MIAT	-1.649532	-1.398674	-1.811918	0.2508583	-0.162385	0.0442364	up
A_24_P381604	ITM2B	3.1207438	3.118815	3.2110023	-0.001929	0.0902586	0.0441649	up
A_33_P3327158	LOC100131432	-1.877682	-1.772708	-1.894427	0.1049748	-0.016745	0.0441148	up
A_23_P13183	EXT2	3.6151333	3.5257678	3.7926798	-0.089365	0.1775465	0.0440905	up
A_23_P6935	CD47	3.6331873	3.5365176	3.8180208	-0.09667	0.1848335	0.0440819	up
A_33_P3667484	ACTG1P4	4.662321	4.607329	4.805458	-0.054992	0.143137	0.0440724	up
A_22_P00017071	lnc-TUFM-1	-0.750085	-0.791503	-0.620864	-0.041418	0.129221	0.0439017	up
A_24_P282237	PRIM2	-1.86032	-2.086223	-1.546618	-0.225903	0.3137026	0.0439	up
A_22_P00014743	LOC102606465	0.7079258	0.9150305	0.588522	0.2071047	-0.119404	0.0438504	up
A_22_P00025225	lnc-BRAT1-1	-0.059215	0.0266256	-0.057368	0.0858402	0.0018463	0.0438433	up
A_33_P3769406	PIGU	0.3154974	0.3497734	0.3689017	0.034276	0.0534043	0.0438402	up
A_22_P00017171	lnc-UBLCP1-3	-1.15457	-1.143614	-1.077919	0.0109558	0.0766511	0.0438035	up
A_22_P00025816	LOC100505978	-1.989673	-2.007381	-1.884442	-0.017708	0.1052303	0.0437613	up
A_23_P69497	CLEC3B	-1.725535	-1.859247	-1.504339	-0.133712	0.2211962	0.0437422	up
A_33_P3303956	HOXA-AS3	-0.573518	-0.313759	-0.746011	0.2597594	-0.172493	0.0436332	up
A_23_P4679	ERF	1.7743788	1.7528982	1.8831244	-0.021481	0.1087456	0.0436325	up
A_23_P11390	VCY	-1.89899	-1.670666	-2.040107	0.2283239	-0.141118	0.0436032	up
A_23_P147383	GPAA1	2.239297	2.2354798	2.3300962	-0.003817	0.0907993	0.0434911	up
A_21_P0004859	BTN2A1	-1.444581	-1.266686	-1.535613	0.1778956	-0.091032	0.0434318	up
A_33_P3219121	KALRN	-2.963659	-3.012832	-2.827699	-0.049174	0.1359599	0.0433931	up
A_33_P3279841	MAPK8IP3	4.7457724	4.6340547	4.9441547	-0.111718	0.1983824	0.0433323	up
A_23_P133543	KLHL3	-2.545925	-2.350511	-2.654689	0.1954145	-0.108763	0.0433255	up
A_32_P186027	ANP32A	2.8899107	2.9798493	2.886487	0.0899386	-0.003424	0.0432575	up
A_33_P3357580	MRTO4	2.747119	2.8401904	2.7405558	0.0930715	-0.006563	0.0432541	up
A_33_P3298634	NFS1	-0.589717	-0.943061	-0.149982	-0.353344	0.4397345	0.043195	up
A_23_P41365	SMR3A	-2.005655	-1.972863	-1.952221	0.0327926	0.0534339	0.0431132	up
A_23_P65797	KLHL25	4.8608255	4.790302	5.0175705	-0.070524	0.156745	0.0431106	up
A_33_P3251896	APBB2	-2.454838	-2.561604	-2.261859	-0.106766	0.1929786	0.0431062	up
A_33_P3402570	CCDC59	2.4957066	2.376101	2.7015162	-0.119606	0.2058096	0.043102	up
A_23_P60180	ABL1	1.8651304	1.8252273	1.9912024	-0.039903	0.1260719	0.0430844	up
A_23_P162336	CNOT2	4.652293	4.638471	4.752248	-0.013822	0.0999546	0.0430663	up
A_32_P103291	SMYD3	3.1771307	3.1708274	3.2695408	-0.006303	0.0924101	0.0430534	up
A_21_P0000115	CNPY2	4.244605	4.1576104	4.4175663	-0.086995	0.1729612	0.0429833	up
A_33_P3247237	CIZ1	0.0807529	0.2340202	0.0134168	0.1532674	-0.067336	0.0429657	up

A_23_P127068	SEMA4G	0.142683	-0.343635	0.7148604	-0.486318	0.5721774	0.0429297	up
A_24_P408801	ZNF587B	-1.645105	-1.483329	-1.721062	0.1617765	-0.075957	0.0429099	up
A_24_P392958	TBC1D20	-0.522138	-0.722455	-0.236028	-0.200316	0.2861099	0.0428967	up
A_23_P171258	ABCB7	2.146925	2.2473693	2.1321611	0.1004443	-0.014764	0.0428402	up
A_23_P78571	COX6B2	-1.283336	-1.235911	-1.245081	0.0474248	0.0382552	0.04284	up
A_23_P146004	CBLL1	0.6614499	0.4877482	0.9207993	-0.173702	0.2593494	0.0428238	up
A_33_P3811287	HUWE1	2.9296618	3.0271363	2.917673	0.0974746	-0.011989	0.042743	up
A_24_P230938	MORN4	0.9441929	0.8072376	1.1665797	-0.136955	0.2223868	0.0427158	up
A_23_P209477	PPM1G	4.314234	4.3727746	4.3410435	0.0585408	0.0268097	0.0426753	up
A_22_P00001367	DLGAP1-AS2	-0.680603	-0.598825	-0.677131	0.0817781	0.0034714	0.0426247	up
A_23_P3450	TUBGCP4	1.957017	1.9522729	2.0469675	-0.004744	0.0899506	0.0426033	up
A_24_P399362	PSMG2	2.8394814	2.957746	2.8063612	0.1182647	-0.03312	0.0425723	up
A_23_P93311	DDR1	2.956707	2.9740338	3.0244923	0.0173268	0.0677853	0.042556	up
A_32_P175301	DENND3	2.5363379	2.6791368	2.4785967	0.1427989	-0.057741	0.0425289	up
A_33_P3340294	RBM33	-0.749416	-0.586935	-0.826927	0.1624813	-0.07751	0.0424855	up
A_23_P151746	ISCA2	4.568491	4.67921	4.5423145	0.1107192	-0.026176	0.0422714	up
A_21_P0003709	lnc-FAM53A-2	-1.707212	-1.582437	-1.747696	0.1247759	-0.040483	0.0421462	up
A_24_P244356	NLRX1	2.7910337	2.52006	3.1462154	-0.270974	0.3551817	0.042104	up
A_23_P123622	NPR2	-0.219705	-0.038046	-0.317319	0.1816592	-0.097614	0.0420225	up
A_24_P19752	PEX19	2.2080126	2.1070952	2.3927174	-0.100917	0.1847048	0.0418937	up
A_23_P46928	PFKP	5.8333187	5.750363	6.00002	-0.082956	0.1667013	0.0418727	up
A_33_P3286616	IRAK1	1.7885303	1.8284307	1.8322387	0.0399003	0.0437083	0.0418043	up
A_23_P135164	UAP1L1	-0.342095	-0.221154	-0.37954	0.1209412	-0.037445	0.041748	up
A_23_P38181	GGA3	2.7797613	2.7197318	2.9232397	-0.06003	0.1434784	0.0417244	up
A_24_P929388	TMEM169	-2.591329	-2.68133	-2.41791	-0.090001	0.1734192	0.0417092	up
A_23_P69738	RASL11B	0.1565714	-0.037874	0.4343624	-0.194445	0.277791	0.0416729	up
A_33_P3401673	GIT1	-1.864344	-2.275835	-1.369585	-0.411491	0.4947591	0.0416341	up
A_23_P1585	ESRRA	5.732976	5.813825	5.7353773	0.0808492	0.0024014	0.0416253	up
A_23_P254179	ADNP	3.4676542	3.663447	3.3550158	0.1957927	-0.112638	0.0415771	up
A_33_P3367332	FAM213B	4.51007	4.4223733	4.6809072	-0.087697	0.1708374	0.0415704	up
A_23_P409626	HNRNPUL2	0.1645398	0.1286674	0.2834849	-0.035872	0.1189451	0.0415363	up
A_24_P272967	AVL9	-0.478079	-0.241986	-0.631103	0.236093	-0.153024	0.0415344	up
A_33_P3377209	ENSA	2.9706964	2.9258857	3.0984497	-0.044811	0.1277533	0.0414712	up
A_24_P38572	NOL6	0.915473	1.0533385	0.860507	0.1378655	-0.054966	0.0414498	up
A_23_P77833	PGS1	-0.036839	-0.099881	0.1089792	-0.063041	0.1458187	0.0413888	up
A_23_P313223	C11orf84	-0.656024	-0.806062	-0.423222	-0.150038	0.2328019	0.0413821	up
A_33_P3211633	WDR3	0.573401	0.486228	0.7432203	-0.087173	0.1698194	0.0413232	up
A_23_P70231	ALDH7A1	4.1066246	4.116424	4.1792145	0.0097995	0.0725899	0.0411947	up
A_33_P3324186	LOC642366	-1.429385	-1.51021	-1.266184	-0.080825	0.1632013	0.0411882	up
A_33_P3386117	RER1	3.4067354	3.0932384	3.8024235	-0.313497	0.3956881	0.0410955	up
A_23_P65370	GLRX5	5.5165415	5.532948	5.5822897	0.0164065	0.0657482	0.0410774	up
A_24_P257348	ARL6IP5	3.035781	3.0876517	3.0659332	0.0518708	0.0301523	0.0410116	up
A_33_P3361182	PDCD11	0.9040756	1.0539331	0.8362045	0.1498575	-0.067871	0.0409932	up
A_24_P203689	KIF7	-1.599711	-1.415884	-1.701619	0.1838269	-0.101908	0.0409594	up
A_23_P355311	PTCH2	-2.064231	-2.091413	-1.955157	-0.027182	0.1090741	0.0409461	up
A_33_P3356846	MTX3	-2.629577	-2.713021	-2.464358	-0.083444	0.1652196	0.040888	up
A_23_P25735	PSMA6	6.29947	6.326471	6.353633	0.0270009	0.054163	0.0405819	up
A_23_P6546	TUBGCP6	0.9001494	0.8592343	1.0221252	-0.040915	0.1219759	0.0405304	up
A_33_P3318668	CYSRT1	5.69785	5.759167	5.717512	0.061317	0.0196619	0.0404894	up
A_33_P3413216	TSPAN4	0.0449719	0.262394	-0.091509	0.217422	-0.136481	0.0404706	up
A_22_P00018284	lnc-ZNF8-1	-1.96947	-1.940262	-1.917749	0.0292082	0.0517211	0.0404646	up
A_24_P337058	CCNL1	1.9289198	2.1391606	1.7995739	0.2102408	-0.129346	0.0404475	up
A_23_P40956	GHRL	-2.384743	-2.05099	-2.637633	0.3337526	-0.25289	0.0404311	up
A_23_P15101	TMC5	1.0510154	1.0580802	1.1245823	0.0070648	0.0735669	0.0403159	up
A_33_P3884230	NFIX	-0.880423	-0.645624	-1.034648	0.2347989	-0.154225	0.040287	up
A_33_P3336810	NUDT17	-1.652103	-1.886336	-1.337397	-0.234233	0.3147059	0.0402362	up

A_24_P203407	FITM2	0.0968566	0.1202278	0.1539431	0.0233712	0.0570865	0.0402288	up
A_21_P0011541	LRRC37A11P	-2.943577	-2.691826	-3.114967	0.2517514	-0.17139	0.0401807	up
A_23_P142750	EIF2AK2	4.9607	4.711822	5.2899237	-0.248878	0.3292236	0.0401728	up
A_23_P159671	PHKA2	2.8158588	2.8024478	2.9095783	-0.013411	0.0937195	0.0401542	up
A_23_P134176	SOD2	4.248048	4.277881	4.298127	0.0298333	0.0500793	0.0399563	up
A_23_P332509	NBAS	1.7275343	1.9982648	1.5366721	0.2707305	-0.190862	0.0399342	up
A_32_P155776	POTEKP	6.6689672	6.633059	6.7846527	-0.035908	0.1156855	0.0398886	up
A_32_P182662	AIDA	-1.309038	-1.137209	-1.401166	0.1718288	-0.092128	0.0398505	up
A_21_P0001486	lnc-TAF12-3	-1.093754	-0.822784	-1.285216	0.2709699	-0.191462	0.0397542	up
A_24_P322229	RASL10B	-0.712961	-0.742711	-0.603795	-0.029749	0.1091661	0.0397084	up
A_23_P153256	ZNF773	1.6103754	1.5554299	1.7446547	-0.054945	0.1342793	0.0396669	up
A_24_P222835	S100PBP	0.0916247	0.0769968	0.1855764	-0.014628	0.0939517	0.0396619	up
A_33_P3334635	RPA2	1.896575	2.040298	1.8321424	0.143723	-0.064433	0.0396452	up
A_33_P3258013	UBE3B	2.6403894	2.5593371	2.800706	-0.081052	0.1603165	0.0396321	up
A_24_P320171	TRMT44	-1.977737	-2.027435	-1.848786	-0.049698	0.1289506	0.0396261	up
A_23_P25503	FNDC3A	2.8756819	3.150309	2.6802816	0.2746272	-0.1954	0.0396135	up
A_24_P922631	C5orf58	1.1183434	1.2511306	1.0647159	0.1327872	-0.053627	0.0395799	up
A_22_P00001196	OSGEPL1-AS1	-1.730398	-1.845535	-1.536164	-0.115137	0.1942344	0.0395486	up
A_32_P749354	EIF1AD	4.0616884	3.94939	4.2530756	-0.112298	0.1913872	0.0395443	up
A_33_P6433406	PTK6	9.987573	10.082588	9.971487	0.0950155	-0.016086	0.039465	up
A_21_P0007129	LOC101929295	1.351901	1.8050809	0.9776301	0.4531798	-0.374271	0.0394545	up
A_23_P97250	GON4L	4.049487	4.087621	4.0901423	0.0381341	0.0406551	0.0393946	up
A_23_P20894	EHMT1	-0.710962	-0.47845	-0.864815	0.232512	-0.153852	0.0393298	up
A_33_P3222648	STK4	3.1711264	3.1355424	3.2853384	-0.035584	0.114212	0.039314	up
A_23_P45699	FUBP1	3.9613447	4.1055627	3.8957329	0.144218	-0.065612	0.0393031	up
A_23_P203900	SCARB1	7.423603	7.2997594	7.626052	-0.123844	0.2024488	0.0393026	up
A_24_P633349	lnc-ITGAL-2	-1.341169	-1.377532	-1.226296	-0.036363	0.1148734	0.0392554	up
A_24_P126181	NR2C2AP	3.0720768	2.9481473	3.2745104	-0.12393	0.2024336	0.039252	up
A_24_P913716	B3GNT7	1.6427174	1.7343707	1.6295404	0.0916534	-0.013177	0.0392382	up
A_33_P3356502	RALB	4.6954174	4.5779033	4.891403	-0.117514	0.1959858	0.0392358	up
A_23_P128783	EAPP	2.7877092	2.9833407	2.6705036	0.1956315	-0.117206	0.0392129	up
A_23_P19291	TUBB2A	5.40129	5.289183	5.59181	-0.112107	0.1905203	0.0392067	up
A_22_P00017348	lnc-USP7-1	-0.461372	-0.607516	-0.236907	-0.146143	0.2244659	0.0391612	up
A_21_P0012264	CRYBB2P1	2.4775162	2.2922502	2.7410612	-0.185266	0.263545	0.0391395	up
A_23_P52147	TBCE	4.443159	4.5032325	4.461197	0.0600734	0.0180378	0.0390556	up
A_23_P114903	HSPA6	-1.32537	-0.644815	-1.92794	0.6805553	-0.60257	0.0389929	up
A_23_P309224	AGK	-0.756918	-1.031317	-0.404577	-0.274399	0.3523407	0.038971	up
A_24_P144881	RNF6	-2.656333	-2.727896	-2.506876	-0.071563	0.149457	0.0389469	up
A_23_P134395	TBL2	4.7495136	4.614819	4.9620743	-0.134695	0.2125607	0.038933	up
A_23_P379020	GNRHR2	-2.441422	-2.761503	-2.043481	-0.320082	0.3979402	0.0389292	up
A_33_P3619171	PMAIP1	3.4011297	3.4162793	3.4638224	0.0151496	0.0626926	0.0389211	up
A_33_P3230788	MUSK	-1.143002	-0.75339	-1.45483	0.3896112	-0.311829	0.0388913	up
A_21_P0000273	SNORA41	-0.272922	-0.328822	-0.139316	-0.0559	0.1336055	0.0388527	up
A_23_P420293	C11orf45	-1.57489	-1.651819	-1.420274	-0.076929	0.1546164	0.0388436	up
A_23_P55064	WRAP53	-1.108875	-1.288324	-0.851773	-0.179449	0.257102	0.0388267	up
A_33_P3245784	ZNF620	-2.279567	-2.060563	-2.420979	0.2190042	-0.141412	0.0387961	up
A_23_P389919	WHSC1	1.8389511	1.9598641	1.7956071	0.120913	-0.043344	0.0387845	up
A_23_P163380	MTHFS	5.1239796	5.1852756	5.1402473	0.061296	0.0162678	0.0387819	up
A_23_P105138	CAT	1.3263316	1.4382386	1.2919059	0.111907	-0.034426	0.0387406	up
A_23_P59888	NACAP1	4.0527	4.152493	4.030341	0.099793	-0.022359	0.038717	up
A_23_P254120	FBXO9	2.6576576	2.6727014	2.720046	0.0150437	0.0623884	0.0387161	up
A_33_P3408844	LOC100133091	0.7128878	0.7693596	0.7337828	0.0564718	0.020895	0.0386834	up
A_23_P120921	ANKRD54	0.0361562	-0.179727	0.3293066	-0.215883	0.2931504	0.0386338	up
A_19_P00322962	SPIN3	-2.359294	-2.040636	-2.600998	0.3186588	-0.241704	0.0384774	up
A_33_P3307307	KMT2A	-0.71697	-0.587443	-0.769656	0.1295276	-0.052686	0.0384209	up
A_23_P37327	ABCD4	1.8501339	1.9016547	1.8753395	0.0515208	0.0252056	0.0383632	up

A_24_P277349	SEC31A	3.571745	3.4832273	3.7369137	-0.088518	0.1651688	0.0383255	up
A_23_P37415	SECISBP2L	-0.443701	-0.470498	-0.34037	-0.026797	0.1033306	0.0382669	up
A_33_P3671378	CERCAM	-0.926353	-0.691587	-1.084656	0.2347651	-0.158304	0.0382307	up
A_23_P12062	TRIT1	3.2090006	3.167707	3.3266735	-0.041294	0.1176729	0.0381897	up
A_24_P158385	ZMYND19	1.9496679	1.9256482	2.0499935	-0.02402	0.1003256	0.0381529	up
A_32_P84009	CMTM4	4.889241	4.742247	5.112381	-0.146994	0.2231398	0.0380728	up
A_33_P3235940	KLK6	8.939211	9.059179	8.89538	0.1199684	-0.043831	0.0380688	up
A_33_P3246715	CENPVP2	-3.013319	-2.722447	-3.228072	0.2908719	-0.214753	0.0380592	up
A_33_P3340718	KDM6A	0.3927221	0.4858632	0.3756681	0.0931411	-0.017054	0.0380435	up
A_23_P32054	CAMSAP1	-0.232068	-0.057051	-0.331065	0.1750169	-0.098997	0.0380099	up
A_33_P3379091	SYNGR1	0.0396686	0.0691514	0.0861211	0.0294828	0.0464525	0.0379677	up
A_19_P00804777	LINC00662	1.539814	1.8848853	1.2706714	0.3450713	-0.269143	0.0379643	up
A_22_P00003425	lnc-CCDC23-1	-1.273683	-1.289004	-1.182468	-0.015321	0.0912147	0.0379469	up
A_23_P319270	CEP131	4.146781	4.25215	4.1172667	0.1053691	-0.029514	0.0379274	up
A_33_P3419970	NPB	1.941288	2.1989522	1.7593069	0.2576642	-0.181981	0.0378416	up
A_32_P192474	PRRT1	-1.590798	-1.526805	-1.579124	0.063993	0.0116739	0.0378335	up
A_33_P3343182	IQCA1	-2.965582	-3.030482	-2.825306	-0.0649	0.140276	0.0376879	up
A_24_P85181	RBSN	2.7898283	3.013739	2.6412516	0.2239108	-0.148577	0.037667	up
A_33_P3326682	SDHC	1.6211939	1.5355563	1.7821503	-0.085638	0.1609564	0.0376594	up
A_33_P3222659	lnc-WDR73-5	-2.144909	-2.075979	-2.138616	0.0689306	0.0062938	0.0376122	up
A_23_P74320	SCYL3	0.8774204	1.0058737	0.82409	0.1284533	-0.05333	0.0375614	up
A_22_P00023931	lnc-MKL1-1	-0.828233	-0.744859	-0.83649	0.0833745	-0.008257	0.0375588	up
A_33_P3382629	lnc-GFM1-1	-2.269342	-2.316314	-2.147293	-0.046973	0.1220489	0.0375382	up
A_33_P3399870	CERS6	3.6522074	3.6720386	3.7074022	0.0198312	0.0551949	0.037513	up
A_23_P140563	TTC23	-0.118248	-0.158668	-0.002948	-0.040419	0.1153002	0.0374405	up
A_23_P57347	PCNT	1.6002226	1.7998171	1.4754581	0.1995945	-0.124764	0.037415	up
A_23_P28318	NDUFAF7	3.2985945	3.4140134	3.2579746	0.1154189	-0.04062	0.0373995	up
A_24_P237753	NUP188	1.2750897	1.2339334	1.3909574	-0.041156	0.1158676	0.0373557	up
A_21_P0007170	lnc-OR51E1-1	-2.821503	-2.810904	-2.757394	0.0105991	0.0641091	0.0373541	up
A_24_P160104	TUBA8	2.1277666	2.0630689	2.2671041	-0.064698	0.1393375	0.0373199	up
A_22_P00016780	lnc-TRIM41-3	0.8494949	0.9925718	0.7810555	0.1430769	-0.068439	0.0373187	up
A_22_P00016656	LOC101927355	-2.316897	-2.48408	-2.075164	-0.167183	0.2417331	0.0372752	up
A_23_P388190	DIDO1	1.8227339	2.1544108	1.5656042	0.331677	-0.25713	0.0372736	up
A_22_P00018010	LOC101928238	-2.586772	-2.262141	-2.837208	0.3246307	-0.250436	0.0370973	up
A_33_P3683076	MAGED2	-2.000066	-1.714327	-2.211625	0.285739	-0.211559	0.0370901	up
A_23_P111297	RPP40	1.713561	1.8315287	1.6697316	0.1179676	-0.043829	0.0370691	up
A_21_P0009964	lnc-NCOA3-10	0.4872599	0.6186848	0.4299488	0.1314249	-0.057311	0.0370569	up
A_33_P6623568	PARN	-1.525348	-1.47225	-1.504361	0.0530982	0.020987	0.0370426	up
A_22_P00025453	lnc-TIGIT-6	-2.794809	-2.929523	-2.586157	-0.134715	0.208652	0.0369687	up
A_33_P3405763	TMPPE	-2.180905	-2.074944	-2.213002	0.1059608	-0.032097	0.036932	up
A_21_P0013071	XLOC_l2_012925	-1.699354	-2.009441	-1.315526	-0.310087	0.3838286	0.036871	up
A_33_P3343442	ZNF316	5.0239677	4.954454	5.1671906	-0.069514	0.1432228	0.0368545	up
A_32_P140706	PMS2	0.0433397	0.2101965	-0.049891	0.1668568	-0.093231	0.0368128	up
A_23_P109636	LRIG1	1.6464796	1.654974	1.7115793	0.0084944	0.0650997	0.036797	up
A_23_P106675	PLCG2	0.4000254	0.4903617	0.3831987	0.0903363	-0.016827	0.0367548	up
A_24_P134356	BTBD3	2.0923138	2.2587962	1.9992876	0.1664825	-0.093026	0.0367281	up
A_21_P0009971	lnc-CEBPB-1	-0.698085	-0.585386	-0.73733	0.1126995	-0.039245	0.0367274	up
A_33_P3393341	LPIN3	3.8633413	3.8379235	3.9620705	-0.025418	0.0987291	0.0366557	up
A_22_P00021758	LOC642366	-1.204128	-1.239458	-1.0955	-0.03533	0.1086288	0.0366495	up
A_33_P3422812	C7orf73	2.2464828	2.3350863	2.2311554	0.0886035	-0.015327	0.036638	up
A_23_P110879	TRAF3IP2	1.4769483	1.4294877	1.5975428	-0.047461	0.1205945	0.036567	up
A_23_P43613	NDUFB6	5.8964434	5.799072	6.066924	-0.097372	0.1704807	0.0365546	up
A_23_P58036	MCCC1	3.9649162	4.055919	3.9469328	0.0910029	-0.017983	0.0365098	up
A_23_P386420	GTF2H3	1.4135003	1.3185539	1.581356	-0.094946	0.1678557	0.0364547	up
A_23_P156017	GOLPH3	3.2414417	3.2819295	3.273734	0.0404878	0.0322924	0.0363901	up
A_23_P118353	SRP68	2.378518	2.4991574	2.3303576	0.1206393	-0.048161	0.0362394	up

A_24_P690924	SEC23B	2.5811033	2.7143245	2.5201511	0.1332212	-0.060952	0.0361345	up
A_33_P3219601	ABL2	0.6878033	0.7643061	0.6834302	0.0765028	-0.004373	0.0360649	up
A_33_P3372742	KRTAP5-11	0.4068756	0.4968162	0.3890238	0.0899406	-0.017852	0.0360444	up
A_22_P00003761	lnc-CDIPT-1	0.1758542	0.270092	0.153554	0.0942378	-0.0223	0.0359688	up
A_24_P285480	MRPL33	6.123932	6.15537	6.1642895	0.0314384	0.0403576	0.035898	up
A_23_P99204	MAPKAPK5-AS1	2.0331192	1.9414067	2.196497	-0.091712	0.1633778	0.0358326	up
A_33_P3397755	MAPKAP1	0.7036347	0.7240477	0.7546878	0.0204129	0.051053	0.035733	up
A_22_P00001167	LOC101927256	-2.054022	-2.056103	-1.980604	-0.002081	0.0734181	0.0356684	up
A_23_P163117	C14orf169	3.9803886	3.933247	4.0988636	-0.047142	0.118475	0.0356667	up
A_33_P6806144	LINC00476	-0.636227	-0.171556	-1.02966	0.4646707	-0.393433	0.0356188	up
A_23_P334608	GUSB	4.7117996	4.660394	4.8343105	-0.051405	0.1225109	0.0355527	up
A_23_P213592	RNF44	2.8438807	2.981936	2.7767649	0.1380553	-0.067116	0.0354698	up
A_23_P104098	SYT2	-2.99968	-2.87185	-3.056606	0.1278298	-0.056926	0.0354519	up
A_23_P43898	EPHX4	2.4269876	2.4804206	2.4444466	0.0534329	0.0174589	0.0354459	up
A_24_P401174	KIAA0556	-2.247948	-2.325411	-2.099639	-0.077462	0.1483088	0.0354232	up
A_24_P407259	SOX21	-1.816773	-1.833521	-1.729295	-0.016748	0.0874777	0.0353646	up
A_21_P0010870	FAM21C	-0.711127	-0.495972	-0.855776	0.2151551	-0.14465	0.0352528	up
A_23_P210643	NELFCD	3.1034546	3.1100698	3.1672144	0.0066152	0.0637598	0.0351875	up
A_33_P3386835	SLC20A2	1.0079646	0.832439	1.2538037	-0.175526	0.2458391	0.0351567	up
A_21_P0013523	OTUD6B-AS1	3.9747381	3.9444432	4.075225	-0.030295	0.1004868	0.0350959	up
A_23_P30315	TRIM7	2.5938444	2.5110793	2.746501	-0.082765	0.1526566	0.0349457	up
A_32_P41405	THOC3	5.7805843	5.5943065	6.036742	-0.186278	0.2561579	0.03494	up
A_33_P3345389	C20orf194	0.1676598	0.3831148	0.021884	0.2154551	-0.145776	0.0348396	up
A_33_P3294133	EIF4B	6.3792877	6.403032	6.42513	0.0237441	0.0458422	0.0347931	up
A_21_P0004750	lnc-SUPT3H-1	-0.166695	-0.064897	-0.198921	0.1017981	-0.032227	0.0347857	up
A_22_P00018851	CELF6	2.0409212	2.2082992	1.9430695	0.167378	-0.097852	0.0347631	up
A_24_P126262	BAGE4	-2.92571	-2.692148	-3.089752	0.2335613	-0.164042	0.0347594	up
A_23_P20384	LSM1	3.984336	3.9601216	4.0780554	-0.024214	0.0937195	0.0347526	up
A_24_P126425	FAM188B	-0.913541	-1.129841	-0.628057	-0.2163	0.2854848	0.0345926	up
A_24_P262355	PHB	5.304818	5.269218	5.409542	-0.0356	0.1047239	0.0345619	up
A_24_P57730	MRPL52	0.8845096	0.7447324	1.0933399	-0.139777	0.2088304	0.0345266	up
A_24_P124992	PSMA4	5.546773	5.5724654	5.5900574	0.0256925	0.0432844	0.0344884	up
A_23_P23815	SLC30A1	1.9991045	2.1393847	1.9277115	0.1402803	-0.071393	0.0344436	up
A_33_P3259801	TMEM189	2.0628557	2.0816865	2.1128845	0.0188308	0.0500288	0.0344298	up
A_19_P00809119	CASC15	0.3336282	0.3684468	0.3676338	0.0348187	0.0340056	0.0344121	up
A_23_P149649	SDHB	3.9868984	4.053478	3.9889717	0.0665793	0.0020733	0.0343263	up
A_33_P3370226	RPL7	9.242383	9.355944	9.197472	0.1135607	-0.044911	0.0343246	up
A_24_P41042	RITA1	2.0064173	1.8404388	2.2410402	-0.165978	0.234623	0.0343223	up
A_24_P150580	RASL12	-2.881693	-2.944867	-2.74994	-0.063174	0.1317525	0.034289	up
A_33_P3248794	NAB2	5.4998417	5.3062663	5.76196	-0.193575	0.2621183	0.0342715	up
A_23_P403424	JMJD7-PLA2G4B	4.0103283	4.098059	3.9910965	0.0877309	-0.019232	0.0342495	up
A_22_P00015031	HOXC-AS3	2.1985168	2.0380664	2.4273968	-0.16045	0.2288799	0.0342147	up
A_23_P60488	ODF2	1.8477116	1.956099	1.8076777	0.1083875	-0.040034	0.0341768	up
A_21_P0000205	C2orf48	-0.291819	-0.196541	-0.319057	0.0952783	-0.027237	0.0340204	up
A_24_P256307	ARF3	5.8093843	5.706056	5.980714	-0.103328	0.1713295	0.0340006	up
A_21_P0000593	SNORA70D	0.4479322	0.5393348	0.4244356	0.0914025	-0.023497	0.033953	up
A_24_P267293	SARM1	0.0658727	0.1334419	0.0661831	0.0675693	0.0003104	0.0339398	up
A_33_P3228315	CARD14	-0.206576	-0.047449	-0.297828	0.1591272	-0.091252	0.0339375	up
A_33_P3258997	TRMT2B	-1.040033	-0.98915	-1.023189	0.0508828	0.0168438	0.0338633	up
A_24_P252996	FOLR3	-2.201512	-2.138477	-2.19693	0.0630355	0.0045829	0.0338092	up
A_23_P24755	STX5	2.4604673	2.2942157	2.6943283	-0.166252	0.233861	0.0338047	up
A_23_P110846	CNOT8	1.6368904	1.6969619	1.6443434	0.0600715	0.007453	0.0337622	up
A_21_P0011014	N6AMT1	-0.383093	-0.26243	-0.436284	0.1206627	-0.053191	0.0337358	up
A_24_P323545	MYH14	0.267828	0.4317288	0.1713576	0.1639009	-0.09647	0.0337152	up
A_33_P3311170	SLC7A5P1	1.7157459	1.9372764	1.5613365	0.2215304	-0.154409	0.0335605	up
A_33_P3347465	ZC3H18	1.9303846	1.9207997	2.006959	-0.009585	0.0765743	0.0334947	up

A_33_P3219429	SMNDC1	0.9986267	0.9609737	1.1032586	-0.037653	0.1046319	0.0334895	up
A_23_P147495	BCORL1	0.8797579	0.9336443	0.892848	0.0538864	0.0130901	0.0334883	up
A_21_P0005977	lnc-ZFAT-5	-2.489821	-2.560579	-2.352104	-0.070758	0.1377177	0.03348	up
A_23_P52531	FAM24B	0.5585213	0.5683451	0.6156564	0.0098238	0.0571351	0.0334795	up
A_33_P3270599	TPM2	5.469885	5.457124	5.549554	-0.012761	0.079669	0.0334542	up
A_23_P45059	DOCK1	1.4723654	1.4657655	1.5456305	-0.0066	0.0732651	0.0333326	up
A_24_P62800	KLHDC8A	-2.589092	-2.388243	-2.723332	0.2008486	-0.13424	0.0333041	up
A_22_P00001008	LINC00857	-2.002544	-2.261333	-1.677153	-0.258789	0.3253913	0.0333011	up
A_33_P3409672	DEPDC5	-0.348442	-0.256717	-0.373641	0.0917244	-0.025199	0.0332627	up
A_23_P258570	PSMD10	1.6234541	1.6783352	1.635097	0.0548811	0.0116429	0.033262	up
A_33_P3331085	SEC24A	0.2306271	0.2791023	0.248672	0.0484753	0.0180449	0.0332601	up
A_33_P3216933	SIK2	-0.965991	-0.873473	-0.992038	0.0925179	-0.026047	0.0332353	up
A_24_P341535	GOLGA2P7	-0.033368	0.1492596	-0.149544	0.1826277	-0.116176	0.0332258	up
A_33_P3351836	C2orf73	-2.323494	-2.1781	-2.402459	0.1453948	-0.078965	0.0332149	up
A_21_P0012006	LOC101927661	-2.173909	-2.073977	-2.207589	0.0999327	-0.033679	0.0331266	up
A_33_P3259938	lnc-RNF208-1	-0.832987	-0.71808	-0.881898	0.1149077	-0.048911	0.0329983	up
A_23_P92349	FGFRL1	2.1559782	2.4142537	1.9634619	0.2582755	-0.192516	0.0328796	up
A_23_P124122	PXMP2	3.3370037	3.3792758	3.3604898	0.0422721	0.0234861	0.0328791	up
A_23_P355824	MGRN1	5.1438637	4.9151235	5.438198	-0.22874	0.2943344	0.0327971	up
A_23_P9465	FPGS	2.1811247	2.2910514	2.1367292	0.1099267	-0.044395	0.0327656	up
A_24_P945293	CHMP3	-0.81892	-0.766847	-0.805826	0.0520735	0.0130944	0.032584	up
A_33_P3404701	SEL1L	-1.007739	-0.469301	-1.4811	0.5384378	-0.473362	0.0325382	up
A_23_P416191	TAS2R31	-2.077426	-1.93122	-2.158568	0.1462059	-0.081142	0.0325317	up
A_19_P00322023	BLCAP	-2.354721	-2.106909	-2.537504	0.2478118	-0.182783	0.0325142	up
A_23_P126888	KIF21B	-2.129773	-2.002763	-2.191755	0.1270099	-0.061982	0.0325139	up
A_19_P00320440	LINC00662	-2.03411	-1.592646	-2.410622	0.4414635	-0.376513	0.0324754	up
A_22_P00008153	lnc-ILK-1	-1.189391	-1.251497	-1.062339	-0.062106	0.1270518	0.0324728	up
A_23_P218237	LCAT	-1.358233	-1.378159	-1.273464	-0.019926	0.0847693	0.0324216	up
A_21_P0005521	KMT2E-AS1	2.116023	1.8263388	2.4704447	-0.289684	0.3544216	0.0323687	up
A_23_P86943	SRPR	2.697197	3.0421271	2.4169617	0.3449302	-0.280235	0.0323474	up
A_33_P3241204	CALHM3	2.0762148	2.0876565	2.129445	0.0114417	0.0532303	0.032336	up
A_22_P00001663	lnc-ASH1L-1	-3.06899	-2.759252	-3.314059	0.3097382	-0.245069	0.0323347	up
A_33_P3215948	MPZL2	5.4161673	5.295506	5.6013584	-0.120661	0.1851912	0.0322649	up
A_24_P268196	LZIC	-0.324877	-0.096273	-0.489376	0.2286038	-0.164499	0.0320525	up
A_23_P48175	TMEM106C	5.6071024	5.5687385	5.7095575	-0.038364	0.1024551	0.0320456	up
A_23_P14284	VTI1B	5.943207	6.064635	5.8858604	0.121428	-0.057346	0.0320408	up
A_33_P3298577	SBNO1	-0.883671	-1.068691	-0.634769	-0.18502	0.2489028	0.0319414	up
A_22_P00002610	LMF1	-2.542341	-2.40874	-2.612157	0.1336007	-0.069816	0.0318923	up
A_23_P326691	RBBP4	3.7843046	3.9012647	3.7311277	0.1169601	-0.053177	0.0318916	up
A_23_P5441	ABCB6	4.548562	4.685475	4.475279	0.1369128	-0.073283	0.0318148	up
A_24_P357536	FBXO11	1.1077561	1.240088	1.0390167	0.1323319	-0.068739	0.0317962	up
A_24_P411815	MPLKIP	3.0968246	3.0823655	3.174737	-0.014459	0.0779123	0.0317266	up
A_23_P253571	PHF5A	2.4348774	2.4476438	2.4855614	0.0127664	0.050684	0.0317252	up
A_33_P3410409	LAMP2	3.6084452	3.6206431	3.6596928	0.012198	0.0512476	0.0317228	up
A_22_P00018423	lnc-RP11-410N8.4.1-2	4.1686907	4.1762223	4.223979	0.0075316	0.0552883	0.03141	up
A_23_P59481	UBE3C	2.9491415	3.0334945	2.9274492	0.084353	-0.021692	0.0313303	up
A_33_P3302791	LOC730338	-0.022596	0.0916662	-0.074339	0.1142626	-0.051743	0.0312598	up
A_33_P3411296	PURA	3.8431997	3.8379693	3.9109335	-0.00523	0.0677338	0.0312517	up
A_22_P00020220	lnc-SARM1-2	-2.481268	-2.646155	-2.253969	-0.164887	0.227299	0.0312059	up
A_23_P342668	AKAP17A	4.8331184	4.8703713	4.85826	0.0372529	0.0251417	0.0311973	up
A_33_P3335248	SDCCAG3	2.0908737	2.2253003	2.0186663	0.1344266	-0.072207	0.0311096	up
A_21_P0000280	SNORD60	0.470819	0.5081697	0.4955049	0.0373507	0.0246859	0.0310183	up
A_23_P138435	ZMIZ1	4.0962	4.135035	4.1189604	0.0388351	0.0227604	0.0307977	up
A_23_P114929	MPC2	5.032073	5.0571723	5.06855	0.0250993	0.0364771	0.0307882	up
A_23_P163467	C15orf52	6.6070604	6.7085614	6.567069	0.101501	-0.039991	0.0307548	up

A_23_P26254	NDUFAF1	4.09196	3.8570704	4.3882875	-0.23489	0.2963276	0.0307191	up
A_23_P8281	IFNGR1	3.3689232	3.4738326	3.3254385	0.1049094	-0.043485	0.0307124	up
A_33_P3396239	SNRPD3	4.848671	4.7737317	4.9848356	-0.074939	0.1361647	0.0306127	up
A_21_P0011414	XLOC_l2_005100	-0.594831	-0.590415	-0.538095	0.0044155	0.0567355	0.0305755	up
A_23_P159741	BCOR	0.2366328	0.3946486	0.1397047	0.1580157	-0.096928	0.0305438	up
A_23_P46222	TRIM46	-1.31855	-1.334661	-1.241377	-0.016111	0.0771728	0.0305309	up
A_32_P357301	PPHLN1	-1.649339	-1.624081	-1.613576	0.0252581	0.0357628	0.0305104	up
A_33_P3368339	NUTM2G	-1.575571	-1.616504	-1.473775	-0.040933	0.1017957	0.0304313	up
A_23_P69058	MLH1	4.0792303	3.9622712	4.2569475	-0.116959	0.1777172	0.0303791	up
A_24_P278299	ASB13	4.0904827	3.791287	4.4502726	-0.299196	0.3597899	0.030297	up
A_33_P3301306	KMT2C	1.9100428	2.0419135	1.8387375	0.1318708	-0.071305	0.0302827	up
A_22_P00007889	HAO2-IT1	-2.825355	-2.491979	-3.098215	0.3333767	-0.27286	0.0302583	up
A_23_P201996	WAC	5.3028555	5.3822026	5.2840023	0.0793471	-0.018853	0.030247	up
A_23_P88381	NUMB	2.2556562	2.171286	2.4004917	-0.08437	0.1448355	0.0302327	up
A_33_P3421520	IKZF5	-0.093211	0.0118756	-0.137849	0.1050863	-0.044638	0.0302241	up
A_33_P3210368	BCL7C	-3.084573	-2.856579	-3.252231	0.227994	-0.167658	0.0301682	up
A_24_P212127	ZNF33A	0.8241701	1.0127144	0.6958909	0.1885443	-0.128279	0.0301325	up
A_22_P00024064	OGFR-AS1	-2.29893	-1.682492	-2.85514	0.6164384	-0.556209	0.0301146	up
A_21_P0008850	LOC102723493	-2.664575	-2.261547	-3.007393	0.403028	-0.342818	0.0301049	up
A_24_P34944	PCDHGA12	-2.022105	-1.913648	-2.070535	0.1084571	-0.04843	0.0300136	up
A_33_P3389023	SRD5A3	-1.198912	-1.099844	-1.237955	0.0990677	-0.039043	0.0300124	up
A_33_P3298057	ABCC5	-0.349639	-0.337071	-0.302217	0.0125685	0.0474219	0.0299952	up
A_23_P95130	SLC37A3	3.2822943	3.129027	3.4953423	-0.153267	0.213048	0.0298903	up
A_23_P103201	PNRC2	1.4794793	1.643897	1.3748226	0.1644177	-0.104657	0.0298805	up
A_23_P59202	TAF11	2.5682735	2.5157332	2.6805525	-0.05254	0.1122789	0.0298693	up
A_24_P330822	HNF1B	-2.698921	-2.548083	-2.790089	0.1508374	-0.091168	0.0298345	up
A_33_P3329592	BLOC1S3	-1.819632	-1.709886	-1.869979	0.109746	-0.050347	0.0296993	up
A_33_P3335371	MAML3	-1.59204	-1.451468	-1.673266	0.1405716	-0.081226	0.0296726	up
A_24_P199655	VANGL1	5.1152096	4.9791293	5.310543	-0.13608	0.1953335	0.0296266	up
A_23_P65157	COX17	6.8055334	6.642731	7.027564	-0.162802	0.2220306	0.0296142	up
A_23_P152181	POLR3E	3.3221836	3.3254752	3.377984	0.0032916	0.0558004	0.029546	up
A_33_P3311740	ZNF774	-2.414278	-2.258995	-2.510634	0.1552832	-0.096356	0.0294638	up
A_23_P70249	CDC25C	1.0251966	1.0897751	1.0194478	0.0645785	-0.005749	0.0294149	up
A_32_P190416	MAP7	-0.013541	-0.037746	0.0692878	-0.024205	0.0828285	0.0293117	up
A_32_P29806	CRADD	2.9367237	2.8290172	3.1029425	-0.107707	0.1662188	0.0292561	up
A_23_P64404	FADS3	0.7095261	0.9927011	0.4847293	0.283175	-0.224797	0.0291891	up
A_21_P0014188	LOC401317	-2.369454	-2.063206	-2.61734	0.3062482	-0.247886	0.0291811	up
A_23_P419624	BLCAP	3.3568506	3.3283153	3.4435587	-0.028535	0.0867081	0.0290864	up
A_23_P42884	MRPS24	7.929241	7.736508	8.180129	-0.192733	0.2508879	0.0290773	up
A_33_P3302632	HIST1H2BE	2.230174	2.1660829	2.3522692	-0.064091	0.1220951	0.029002	up
A_21_P0012118	XLOC_l2_008599	-2.729485	-2.818463	-2.582587	-0.088978	0.1468978	0.0289598	up
A_24_P228579	NDUFAF5	3.7228336	3.6199317	3.8836508	-0.102902	0.1608172	0.0289576	up
A_22_P00015555	LOXL1-AS1	-1.266872	-1.31226	-1.163685	-0.045388	0.1031866	0.0288992	up
A_23_P131208	NR4A2	-1.143715	-0.832062	-1.397596	0.3116531	-0.253881	0.0288861	up
A_33_P3332006	CBX1	3.9278288	4.0634503	3.8499422	0.1356216	-0.077887	0.0288675	up
A_23_P77818	ATP5H	8.165469	8.147276	8.241347	-0.018193	0.0758781	0.0288424	up
A_33_P3481987	SLC16A12	9.636218	9.681835	9.648091	0.0456171	0.0118732	0.0287452	up
A_22_P00005381	lnc-DRGX-1	-1.381994	-1.236419	-1.470182	0.1455751	-0.088188	0.0286934	up
A_23_P43425	C9orf40	1.2300615	0.9848599	1.5326071	-0.245202	0.3025456	0.028672	up
A_33_P3255766	NIF3L1	1.8320255	1.849906	1.8714457	0.0178804	0.0394201	0.0286503	up
A_22_P00001149	PCED1B-AS1	-2.892669	-2.765265	-2.962774	0.1274035	-0.070105	0.0286492	up
A_33_P3340085	POLDIP3	1.1108632	1.2620335	1.0169792	0.1511703	-0.093884	0.0286431	up
A_33_P3550894	GATA2	-1.437273	-1.440935	-1.376385	-0.003663	0.0608878	0.0286126	up
A_23_P368278	FCHSD2	1.5049944	1.5076342	1.5595307	0.0026398	0.0545363	0.0285881	up
A_32_P199884	HORMAD1	-1.062256	-1.00149	-1.065881	0.0607667	-0.003624	0.0285711	up
A_22_P00016791	lnc-TRIM63-1	-2.434439	-2.412907	-2.398869	0.0215323	0.0355706	0.0285515	up

A_23_P346982	DTWD2	0.5070953	0.502285	0.5689468	-0.00481	0.0618515	0.0285206	up
A_33_P3286334	SH3BP1	-0.975042	-1.059866	-0.833283	-0.084824	0.1417589	0.0284677	up
A_33_P3424472	TNPO3	-1.449457	-1.536202	-1.305804	-0.086744	0.1436534	0.0284545	up
A_23_P79931	ATRN	1.4869094	1.641027	1.3896832	0.1541176	-0.097226	0.0284457	up
A_22_P00020365	lnc-TSR1-1	-1.705545	-1.677257	-1.67694	0.0282874	0.028604	0.0284457	up
A_23_P250478	PDK3	0.4925375	0.2965035	0.7452078	-0.196034	0.2526703	0.0283182	up
A_33_P3316313	MTERF4	1.4695339	1.7173762	1.278326	0.2478423	-0.191208	0.0283172	up
A_21_P0012255	LOC102724248	-2.01596	-2.07791	-1.89743	-0.06195	0.1185308	0.0282905	up
A_21_P0010545	LAMTOR5-AS1	0.2077503	0.1049523	0.3670678	-0.102798	0.1593175	0.0282598	up
A_33_P3396646	BEND3	-1.066278	-0.936912	-1.139179	0.1293664	-0.072901	0.0282328	up
A_23_P154235	NMI	3.3941574	3.4054952	3.4391003	0.0113378	0.0449429	0.0281403	up
A_33_P3216664	LOC151174	-1.409952	-1.229376	-1.534343	0.1805759	-0.124391	0.0280924	up
A_23_P15348	MPRIP	2.9188423	3.0965962	2.7972708	0.1777539	-0.121572	0.0280912	up
A_23_P109034	SDC4	4.6337786	4.7241216	4.5995045	0.090343	-0.034274	0.0280345	up
A_23_P127233	SMNDC1	4.2647552	4.177217	4.408251	-0.087538	0.1434956	0.0279787	up
A_23_P201764	DHDDS	-2.281734	-2.457968	-2.04963	-0.176234	0.2321038	0.0279347	up
A_23_P26117	MAN2C1	0.2086191	0.3583169	0.1145139	0.1496978	-0.094105	0.0277963	up
A_24_P115007	ALDH5A1	0.1660795	0.0982471	0.2894073	-0.067832	0.1233277	0.0277476	up
A_33_P3334843	PLD1	-2.739973	-2.582566	-2.841897	0.1574073	-0.101923	0.0277419	up
A_22_P00012848	RASAL2	-1.86241	-1.98374	-1.685749	-0.12133	0.1766615	0.0276659	up
A_23_P45786	COL9A2	-2.067067	-2.005933	-2.073128	0.0611339	-0.006061	0.0275364	up
A_33_P3420068	GOLGA7	0.7367535	0.6454873	0.8829689	-0.091266	0.1462154	0.0274746	up
A_33_P3390570	KMT2A	3.6243105	3.665195	3.6381588	0.0408845	0.0138483	0.0273664	up
A_23_P141974	TPM4	4.9760494	4.957749	5.0489874	-0.018301	0.072938	0.0273187	up
A_33_P3421363	ZNF169	-0.15348	-0.085054	-0.167417	0.0684257	-0.013937	0.0272446	up
A_23_P201672	METTL13	5.2519016	5.1593404	5.398814	-0.092561	0.1469126	0.0271757	up
A_33_P3289820	IQSEC1	4.1341934	4.265714	4.0570107	0.1315208	-0.077183	0.027169	up
A_23_P58443	ANKHD1-EIF4EBP3	1.0740881	1.2992945	0.9031305	0.2252064	-0.170958	0.0271244	up
A_22_P00003387	lnc-CCDC107-2	-2.473949	-2.417197	-2.476788	0.056752	-0.002839	0.0269564	up
A_24_P386771	PPP1CC	3.434534	3.5553737	3.3675642	0.1208396	-0.06697	0.0269349	up
A_24_P196592	MMP28	0.5213661	0.5134955	0.583106	-0.007871	0.0617399	0.0269346	up
A_24_P30194	IFIT5	3.0394983	2.8469691	3.285842	-0.192529	0.2463436	0.0269072	up
A_23_P18490	MAEA	1.2540836	1.4181275	1.1436677	0.1640439	-0.110416	0.026814	up
A_21_P0007500	lnc-CD9-1	-2.289855	-1.842042	-2.684077	0.4478126	-0.394222	0.0267953	up
A_32_P206401	QSOX2	1.6347294	1.7411804	1.5818043	0.106451	-0.052925	0.026763	up
A_23_P16139	CHERP	0.395402	0.6261811	0.2178254	0.2307792	-0.177577	0.0266013	up
A_24_P120970	ARRDC1-AS1	-0.932107	-0.935895	-0.875165	-0.003788	0.0569425	0.0265775	up
A_33_P3319155	ACP1	3.9568014	3.9921384	3.974513	0.035337	0.0177116	0.0265243	up
A_23_P4944	CALM3	4.3910923	4.3714595	4.4637136	-0.019633	0.0726213	0.0264943	up
A_23_P89780	LAMA3	4.5196295	4.6532865	4.438775	0.133657	-0.080854	0.0264013	up
A_23_P45524	NGFRAP1	5.8434315	5.9571033	5.7825623	0.1136718	-0.060869	0.0264013	up
A_33_P3304603	ZMIZ1	0.0421038	0.0693669	0.0675702	0.0272632	0.0254664	0.0263648	up
A_23_P12849	FBXO18	1.05477	0.9220996	1.2401485	-0.13267	0.1853786	0.0263541	up
A_24_P277807	SNX3	4.2576904	4.4085746	4.159442	0.1508842	-0.098248	0.0263178	up
A_24_P322474	PDE4A	4.019636	3.8367677	4.2549505	-0.182868	0.2353144	0.0262229	up
A_24_P353619	ALPL	-3.091998	-3.011848	-3.119882	0.0801506	-0.027883	0.0261337	up
A_23_P76557	NDUFA12	5.931983	5.880774	6.035431	-0.051209	0.1034479	0.0261195	up
A_23_P145904	H2AFV	7.385007	7.425122	7.3970833	0.0401149	0.0120764	0.0260956	up
A_33_P3351536	PTK2B	1.9427204	1.86766	2.069788	-0.07506	0.1270676	0.0260036	up
A_23_P122805	TMEM209	2.3079834	2.3711877	2.2965803	0.0632043	-0.011403	0.0259006	up
A_23_P120125	COLEC11	-0.813231	-0.714296	-0.860393	0.0989342	-0.047162	0.0258861	up
A_21_P0008656	lnc-ARID3B-1	-0.758195	-0.639702	-0.824954	0.1184926	-0.066759	0.025867	up
A_24_P130865	PHF8	-0.225525	-0.311731	-0.087734	-0.086206	0.1377907	0.0257921	up
A_21_P0003966	lnc-MTRR-2	-2.251798	-2.447307	-2.004733	-0.195509	0.2470646	0.0257776	up
A_33_P3304252	MRPL32	4.784753	4.7735486	4.8475046	-0.011204	0.0627518	0.0257738	up

A_24_P361457	ENDOV	-0.107871	0.1835089	-0.347768	0.2913795	-0.239897	0.0257411	up
A_23_P87591	YEATS4	2.4332647	2.496698	2.4212675	0.0634332	-0.011997	0.025718	up
A_23_P121702	OCIAD2	7.110859	6.948796	7.3241367	-0.162063	0.2132778	0.0256074	up
A_23_P32021	FANCC	1.8853655	1.9484639	1.8733807	0.0630984	-0.011985	0.0255568	up
A_23_P45166	PROSC	1.7730398	1.6671863	1.9299631	-0.105854	0.1569233	0.0255349	up
A_33_P3389376	MDM4	-0.071334	0.0851932	-0.176807	0.1565275	-0.105473	0.0255275	up
A_23_P24948	KCNE3	2.749587	3.0485244	2.5016575	0.2989373	-0.24793	0.0255039	up
A_24_P274795	CDCA7L	3.8701057	3.9958777	3.7952595	0.125772	-0.074846	0.0254629	up
A_24_P410389	BCL2L13	-1.682802	-1.681218	-1.633627	0.0015845	0.0491748	0.0253797	up
A_19_P00813554	KTN1-AS1	-0.552218	-0.67536	-0.378348	-0.123142	0.1738696	0.0253637	up
A_33_P3401621	CCNB1	3.5217838	3.4627767	3.6313877	-0.059007	0.1096039	0.0252984	up
A_23_P121196	TMEM43	2.045003	1.9595685	2.1810274	-0.085434	0.1360245	0.025295	up
A_23_P155868	PGRMC2	3.2128067	3.3586154	3.1175184	0.1458087	-0.095288	0.0252602	up
A_21_P0000380	SNORD69	-1.235426	-1.113043	-1.307411	0.1223826	-0.071985	0.0251987	up
A_33_P3270509	SIAH1	0.5619741	0.7437701	0.4305139	0.1817961	-0.13146	0.0251679	up
A_24_P203056	BCL7A	1.234292	1.126627	1.3922706	-0.107665	0.1579785	0.0251567	up
A_21_P0000615	LINC00184	-3.02262	-2.878562	-3.116448	0.144058	-0.093828	0.025115	up
A_23_P63798	KLF6	4.116085	4.2746997	4.0076456	0.1586146	-0.108439	0.0250876	up
A_24_P350200	LOC101929612	9.026699	9.185089	8.918386	0.1583901	-0.108313	0.0250387	up
A_33_P3286422	FANCA	-0.531826	-0.531919	-0.481696	-9.25E-05	0.0501299	0.0250187	up
A_23_P138461	C10orf2	1.0660262	0.8945298	1.2870951	-0.171496	0.2210689	0.0247862	up
A_23_P15734	KRT9	0.3723807	0.3536	0.4407263	-0.018781	0.0683456	0.0247824	up
A_22_P00010973	lnc-NRIP2-2	-0.043555	-0.051699	0.0138173	-0.008143	0.0573726	0.0246146	up
A_33_P3231267	lnc-ITGA2-1	-1.551	-1.663716	-1.38922	-0.112716	0.1617799	0.0245318	up
A_23_P266	PIN1P1	1.8040972	1.8985796	1.758595	0.0944824	-0.045502	0.0244901	up
A_24_P11131	SRRM1	3.642109	3.6465287	3.6865625	0.0044198	0.0444536	0.0244367	up
A_24_P96762	TRNAU1AP	0.6023793	0.8330908	0.4204984	0.2307115	-0.181881	0.0244153	up
A_23_P380318	EGR4	-0.544974	-0.690779	-0.35061	-0.145805	0.1943636	0.0242794	up
A_24_P271527	JOSD1	4.9662457	5.0856757	4.8953276	0.1194301	-0.070918	0.024256	up
A_23_P90419	PBX4	2.3104239	2.4273343	2.2418222	0.1169105	-0.068602	0.0241544	up
A_33_P8911753	ITGA9-AS1	-1.68335	-1.851431	-1.467316	-0.168081	0.2160335	0.0239761	up
A_33_P3256773	INIP	1.6938753	1.570395	1.8651695	-0.12348	0.1712942	0.0239069	up
A_23_P62752	NPPB	-2.910974	-2.599931	-3.174217	0.3110428	-0.263244	0.0238996	up
A_33_P3305023	WWOX	-0.426735	-0.725519	-0.08026	-0.298783	0.3464756	0.0238462	up
A_23_P29318	SAMM50	5.2373	5.0803227	5.4419355	-0.156977	0.2046356	0.0238292	up
A_24_P141019	ST7	-0.786042	-0.787419	-0.737099	-0.001377	0.0489435	0.0237834	up
A_23_P11995	PRDX1	7.89618	7.8042874	8.035543	-0.091893	0.1393633	0.0237353	up
A_32_P105549	ANXA8L1	4.4478626	4.547933	4.3950434	0.1000705	-0.052819	0.0236256	up
A_23_P5301	TFCP2L1	1.241529	1.2532892	1.2769871	0.0117602	0.0354581	0.0236092	up
A_23_P27285	MPPE1	3.1199322	3.1512275	3.1358223	0.0312953	0.0158901	0.0235927	up
A_23_P336015	NOC2L	0.9846363	0.9157968	1.1006498	-0.06884	0.1160135	0.023587	up
A_23_P26928	CCDC103	0.6272187	0.8340864	0.467484	0.2068677	-0.159735	0.0235665	up
A_21_P0000109	MTRNR2L9	4.302703	4.4504495	4.2020426	0.1477466	-0.10066	0.0235431	up
A_33_P3215933	MPZL3	-0.454168	-0.33548	-0.525972	0.1186886	-0.071804	0.0234423	up
A_23_P29575	MRPS36	5.9593353	5.888929	6.0765553	-0.070406	0.1172199	0.0234067	up
A_23_P328206	DNMBP	1.4924283	1.4231229	1.6085377	-0.069305	0.1161094	0.023402	up
A_33_P3275000	ZBTB40	-2.545932	-2.486632	-2.558544	0.0592999	-0.012612	0.023344	up
A_32_P21255	SLC30A4	-0.92491	-0.847803	-0.955429	0.077107	-0.030519	0.0232942	up
A_24_P110799	LSS	3.1208	2.949307	3.3388767	-0.171493	0.2180767	0.0232918	up
A_24_P401768	AMOTL1	-1.604708	-1.571825	-1.59101	0.0328832	0.0136981	0.0232906	up
A_21_P0011364	WHAMMP1	1.02671	1.1164069	0.9835787	0.0896969	-0.043131	0.0232828	up
A_23_P343411	AGRN	4.0089474	4.045712	4.018735	0.0367646	0.0097876	0.0232761	up
A_23_P128698	SPRY2	0.6252627	0.8121619	0.4847121	0.1868992	-0.140551	0.0231743	up
A_24_P383523	SAMD4A	0.6013756	0.8191433	0.4299007	0.2177677	-0.171475	0.0231464	up
A_22_P00003529	lnc-CCNB2-1	-3.070678	-2.930652	-3.16448	0.1400263	-0.093802	0.0231124	up
A_23_P255503	PAFAH1B2	1.6821642	1.5521536	1.858304	-0.130011	0.1761398	0.0230646	up

A_23_P109345	PTTG1IP	5.6611185	5.6870656	5.681202	0.0259471	0.0200834	0.0230153	up
A_23_P146217	BAG4	-1.63528	-1.547694	-1.676848	0.0875859	-0.041568	0.0230088	up
A_23_P31335	ZNF12	2.64923	2.7161307	2.6282806	0.0669007	-0.020949	0.0229757	up
A_24_P327499	TTI1	0.3740764	0.3653836	0.4287109	-0.008693	0.0546346	0.0229709	up
A_22_P00013157	TTC39A-AS1	-2.81055	-2.894879	-2.680349	-0.084329	0.1302004	0.0229355	up
A_23_P47034	HHEX	-0.165964	-0.182218	-0.104013	-0.016254	0.0619507	0.0228484	up
A_24_P66125	STAG2	1.9081821	2.1160674	1.7459149	0.2078853	-0.162267	0.022809	up
A_23_P111228	COQ3	1.2648997	1.1505013	1.4249005	-0.114398	0.1600008	0.0228012	up
A_22_P00003431	BBOX1-AS1	-0.755164	-0.529701	-0.935099	0.2254629	-0.179935	0.022764	up
A_23_P210719	NSFL1C	1.2796307	1.2762752	1.3284898	-0.003356	0.0488591	0.0227518	up
A_24_P327815	STIP1	1.9473925	1.7873702	2.152894	-0.160022	0.2055016	0.0227397	up
A_33_P3324949	IGF2BP2-AS1	-1.826549	-1.802067	-1.805568	0.0244813	0.0209804	0.0227308	up
A_22_P00019607	PCBP4	-1.092152	-1.056712	-1.08216	0.0354404	0.0099921	0.0227163	up
A_21_P0012835	XLOC_l2_011649	-0.667855	-0.703805	-0.586473	-0.035951	0.0813823	0.0227158	up
A_21_P0012956	LOC101926941	-2.620797	-2.871265	-2.325132	-0.250468	0.295665	0.0225986	up
A_23_P146512	GOLM1	4.1572056	4.2575297	4.1018887	0.1003242	-0.055317	0.0225036	up
A_23_P103110	MAFF	0.9242635	1.4479136	0.4455261	0.5236502	-0.478737	0.0224564	up
A_24_P161144	ZNF843	-0.497738	-0.387632	-0.562999	0.110106	-0.065261	0.0224226	up
A_23_P22499	GNL3L	2.6641045	2.7880483	2.5849714	0.1239438	-0.079133	0.0224054	up
A_24_P93703	TMEM198B	-0.559994	-0.587306	-0.487873	-0.027311	0.0721211	0.0224049	up
A_19_P00802936	BRK1	7.5261297	7.4778914	7.6191597	-0.048238	0.09303	0.0223958	up
A_23_P3186	CCNK	1.7701235	1.8282189	1.7564392	0.0580955	-0.013684	0.0222056	up
A_23_P73848	TTTY14	0.008399	0.0273247	0.0338264	0.0189257	0.0254273	0.0221765	up
A_33_P3210443	LINC00637	-1.72899	-1.706412	-1.707225	0.0225778	0.0217643	0.022171	up
A_23_P150255	RBM14	4.938119	4.8890634	5.0314426	-0.049056	0.0933237	0.0221341	up
A_23_P356616	ABTB2	1.4941082	1.7063918	1.3260608	0.2122836	-0.168047	0.0221181	up
A_21_P0000847	LOC100630918	-1.271976	-1.323113	-1.176618	-0.051138	0.0953574	0.0221097	up
A_23_P158829	ARRB2	2.0632124	2.1024022	2.0682144	0.0391898	0.005002	0.0220959	up
A_23_P133058	MRFAP1L1	2.2562218	2.3043933	2.2522097	0.0481715	-0.004012	0.0220797	up
A_21_P0003024	LOC101928948	1.6961298	1.551774	1.8846083	-0.144356	0.1884785	0.0220614	up
A_23_P333063	SMARCE1	1.8988628	1.9035625	1.9382839	0.0046997	0.0394211	0.0220604	up
A_19_P00322299	ZSWIM6	0.3933339	0.5776739	0.2530756	0.18434	-0.140258	0.0220408	up
A_33_P3367692	CFH	-2.831286	-2.899058	-2.719465	-0.067772	0.1118212	0.0220244	up
A_22_P00014366	CBR3-AS1	-1.861883	-1.85258	-1.827151	0.0093031	0.0347319	0.0220175	up
A_33_P3705884	LINC00662	0.8672972	1.1548629	0.6236925	0.2875657	-0.243605	0.0219805	up
A_24_P382001	POLR3K	4.5758467	4.4703298	4.725272	-0.105517	0.1494255	0.0219543	up
A_24_P945194	PDCD6IP	0.6896391	0.734179	0.6890049	0.0445399	-0.000634	0.0219529	up
A_23_P72138	MRPS22	5.0847254	5.0946937	5.1185055	0.0099683	0.0337801	0.0218742	up
A_33_P3251796	PDSS1	1.6600218	1.6410241	1.7227435	-0.018998	0.0627217	0.021862	up
A_23_P166248	RCAN1	2.8913622	3.0958877	2.7305079	0.2045255	-0.160854	0.0218356	up
A_23_P45517	PPP2R3B	0.823163	0.899724	0.790247	0.076561	-0.032916	0.0218225	up
A_23_P71319	FDFT1	6.150735	5.9606357	6.384342	-0.190099	0.2336073	0.021754	up
A_23_P10442	OSBPL1A	2.3956146	2.461636	2.3730726	0.0660214	-0.022542	0.0217397	up
A_23_P155857	NUDT6	0.8205566	0.8312845	0.8532014	0.0107279	0.0326448	0.0216863	up
A_23_P48628	ZBTB25	-0.166395	-0.080906	-0.208563	0.0854888	-0.042169	0.0216601	up
A_23_P217135	ZNF275	3.3462791	3.1364684	3.599227	-0.209811	0.2529478	0.0215685	up
A_23_P122796	HDDC2	0.5413694	0.3633018	0.7625694	-0.178068	0.2212	0.0215662	up
A_33_P3356711	ING3	-1.779163	-1.596945	-1.918267	0.1822186	-0.139104	0.0215573	up
A_32_P28223	LOC101928837	-0.123766	-0.128924	-0.07555	-0.005158	0.0482163	0.021529	up
A_21_P0000766	TEKT4P2	1.6665115	1.5483742	1.8276129	-0.118137	0.1611013	0.021482	up
A_33_P3304963	LRRC27	-1.54927	-1.570104	-1.485855	-0.020833	0.0634151	0.0212908	up
A_21_P0005892	lnc-EXT1-2	-2.438035	-2.249286	-2.58421	0.1887488	-0.146175	0.0212868	up
A_33_P3342096	ZHX3	-0.188569	-0.20094	-0.133643	-0.012372	0.0549254	0.021277	up
A_23_P218068	PLEKHA5	0.6762314	0.7695456	0.6253119	0.0933142	-0.05092	0.0211973	up
A_33_P3315190	MICAL2	0.0458469	-0.077419	0.2113347	-0.123266	0.1654878	0.021111	up
A_23_P200493	LBR	6.100013	6.2130704	6.029022	0.1130576	-0.070991	0.0210335	up

A_23_P256855	XPO5	1.7096815	1.5703955	1.8909788	-0.139286	0.1812973	0.0210056	up
A_22_P00015594	lnc-STXBP1-1	-2.390069	-2.334916	-2.403339	0.0551529	-0.01327	0.0209416	up
A_22_P00009058	lnc-LENG9-1	-2.35549	-2.103612	-2.565614	0.2518783	-0.210124	0.0208772	up
A_23_P15798	KRTAP4-12	-1.842732	-1.688937	-1.954813	0.1537952	-0.11208	0.0208576	up
A_23_P380181	LMO4	2.4304113	2.6393094	2.2629786	0.2088981	-0.167433	0.0207326	up
A_23_P342185	WAPAL	0.8373051	1.0127783	0.7032199	0.1754732	-0.134085	0.020694	up
A_32_P62211	LOC101928433	-1.01764	-0.635883	-1.358088	0.3817568	-0.340448	0.0206544	up
A_23_P152125	MVD	3.0617437	2.807942	3.3568535	-0.253802	0.2951098	0.020654	up
A_23_P142125	HRC	-1.896646	-2.174375	-1.577752	-0.277729	0.3188939	0.0205823	up
A_23_P60296	OSTF1	4.3078604	4.316193	4.340686	0.0083327	0.0328255	0.0205791	up
A_22_P00015621	TMEM180	-2.693651	-2.782178	-2.563997	-0.088526	0.1296539	0.0205637	up
A_33_P3381259	lnc-DNAI1-1	-0.845835	-0.803539	-0.847065	0.0422964	-0.001229	0.0205336	up
A_19_P00800555	lnc-UQCRFS1-7	2.5587807	2.9995074	2.1590662	0.4407268	-0.399714	0.0205061	up
A_24_P253251	SLC7A1	3.2210855	3.5800986	2.9030628	0.3590131	-0.318023	0.0204952	up
A_23_P150018	DUSP5	0.6782312	0.834579	0.5628147	0.1563478	-0.115417	0.0204656	up
A_33_P3396214	KREMEN2	1.557147	1.4075165	1.7476926	-0.149631	0.1905456	0.0204575	up
A_23_P152858	COPRS	6.6661243	6.6164784	6.7566338	-0.049646	0.0905094	0.0204318	up
A_33_P3242965	ATAD3C	0.9173331	0.8329492	1.0424986	-0.084384	0.1251655	0.0203907	up
A_23_P101208	CYB5A	4.4052725	4.220225	4.6310377	-0.185048	0.2257652	0.0203588	up
A_33_P3388331	FARP1	-1.023552	-1.16851	-0.83788	-0.144959	0.1856718	0.0203567	up
A_21_P0007110	lnc-WAPAL-1	-1.856932	-1.987022	-1.686278	-0.13009	0.1706538	0.020282	up
A_33_P3212982	PLEKHM1	1.475492	1.4678082	1.5236516	-0.007684	0.0481596	0.0202379	up
A_24_P921321	PTPRJ	0.608304	0.7707815	0.4862714	0.1624775	-0.122033	0.0202224	up
A_24_P355876	TMBIM6	8.086373	8.112064	8.101056	0.025691	0.0146828	0.0201869	up
A_33_P3319982	lnc-TMC7-1	-1.847443	-1.848261	-1.806358	-0.000818	0.0410843	0.020133	up
A_33_P3244882	AGO3	2.1244688	2.154612	2.134573	0.0301433	0.0101042	0.0201237	up
A_23_P371239	CMIP	1.4517708	1.3512225	1.592515	-0.100548	0.1407442	0.020098	up
A_23_P92687	DAP	2.8115292	2.833549	2.8295794	0.0220199	0.0180502	0.020035	up
A_33_P3213695	CAB39L	-0.663143	-0.494431	-0.791794	0.1687121	-0.128651	0.0200305	up
A_33_P3323074	AGPAT4	-1.367572	-1.42546	-1.269752	-0.057888	0.0978208	0.0199664	up
A_23_P29924	TMEM128	1.2479782	1.2535505	1.2822747	0.0055723	0.0342965	0.0199344	up
A_33_P3407524	SUV39H1	-0.594455	-0.693404	-0.455846	-0.098948	0.1386089	0.0198302	up
A_33_P3371175	CYP20A1	2.002285	2.140308	1.9038191	0.1380229	-0.098466	0.0197785	up
A_23_P166899	DNAJB11	5.1753407	5.09598	5.2942524	-0.07936	0.1189117	0.0197756	up
A_23_P259166	TCEAL4	4.711111	4.6553874	4.806097	-0.055724	0.094986	0.0196311	up
A_33_P3418010	NUP62	4.592843	4.534695	4.6900244	-0.058148	0.0971813	0.0195167	up
A_24_P263910	LINC01588	0.1187105	0.2370195	0.0394096	0.118309	-0.079301	0.0195041	up
A_33_P3226832	F3	3.8348846	3.6978517	4.010911	-0.137033	0.1760263	0.0194967	up
A_23_P369471	LCE3A	-3.090676	-3.123921	-3.018566	-0.033245	0.0721095	0.0194322	up
A_33_P3237150	BMP2	-2.392643	-2.260805	-2.485629	0.1318378	-0.092986	0.019426	up
A_33_P3331726	LINC00657	2.648219	2.6442542	2.6910172	-0.003965	0.042798	0.0194166	up
A_23_P106258	SLC25A47	-2.822861	-2.714765	-2.892491	0.1080954	-0.069631	0.0192324	up
A_22_P00012255	lnc-PPP1R14D-1	-0.795105	-0.417084	-1.134693	0.3780208	-0.339589	0.0192161	up
A_32_P71943	DSTYK	1.9482145	1.9941058	1.9406776	0.0458913	-0.007537	0.0191772	up
A_24_P138713	HEATR3	0.9404073	1.1098971	0.8091331	0.1694899	-0.131274	0.0191078	up
A_21_P0013848	FOXN3	0.4365959	0.3351598	0.5761428	-0.101436	0.1395469	0.0190554	up
A_22_P00014305	SMIM2-AS1	-2.219068	-2.44588	-1.954402	-0.226813	0.2646651	0.0189263	up
A_24_P79054	TGFB1	1.5146346	1.2664638	1.8006315	-0.248171	0.2859969	0.018913	up
A_33_P3315801	CDK12	1.6448841	1.5901656	1.7374163	-0.054718	0.0925322	0.0189068	up
A_24_P67027	CLK2	0.4700298	0.5607014	0.4167938	0.0906715	-0.053236	0.0187178	up
A_21_P0012960	LOC255187	-2.26115	-2.258119	-2.227198	0.0030313	0.033952	0.0184916	up
A_21_P0000899	LOC100506990	-2.559508	-2.420152	-2.662	0.1393564	-0.102492	0.0184323	up
A_32_P73821	CSDE1	5.20385	5.2834487	5.1610584	0.0795989	-0.042791	0.0184038	up
A_23_P124327	NSD1	2.17173	2.0771947	2.3026638	-0.094535	0.1309338	0.0181992	up
A_23_P66137	SOX8	2.7846022	2.5446782	3.0608816	-0.239924	0.2762795	0.0181778	up
A_24_P140608	HBEGF	-1.544323	-1.367091	-1.685267	0.1772327	-0.140943	0.0181448	up

A_33_P3407105	YTHDF1	-0.874871	-0.964878	-0.748574	-0.090007	0.1262965	0.0181448	up
A_23_P376799	FAM21C	3.2224798	3.342442	3.138792	0.1199622	-0.083688	0.0181372	up
A_23_P18806	YIPF5	1.5281343	1.5255976	1.5668483	-0.002537	0.0387139	0.0180886	up
A_23_P312646	C9orf142	3.6404762	3.4817472	3.835247	-0.158729	0.1947708	0.0180209	up
A_33_P3296372	EIF2B3	4.0028963	3.8374705	4.204317	-0.165426	0.2014208	0.0179975	up
A_23_P306655	AMIGO3	-0.854375	-0.789615	-0.883153	0.0647602	-0.028777	0.0179915	up
A_23_P56894	CYP20A1	-0.58628	-0.39809	-0.738764	0.1881895	-0.152484	0.0178528	up
A_33_P3394489	CHKB-AS1	-0.672675	-0.668172	-0.641477	0.0045033	0.031198	0.0178506	up
A_33_P3371984	IGSF1	-2.002507	-1.749374	-2.22	0.2531328	-0.217494	0.0178196	up
A_33_P3332393	TMEM53	-2.292194	-2.67545	-1.873693	-0.383256	0.4185019	0.0176231	up
A_33_P3327587	LOC100127904	-2.011817	-2.119697	-1.868798	-0.10788	0.1430192	0.0175697	up
A_23_P80694	ACTR8	-1.020183	-1.235165	-0.770078	-0.214982	0.2501049	0.0175617	up
A_21_P0000600	IFT27	-1.682224	-1.499584	-1.829856	0.1826396	-0.147632	0.0175037	up
A_23_P170491	TRAIP	2.6625881	2.6664767	2.6936264	0.0038886	0.0310383	0.0174634	up
A_23_P317796	MOGAT3	-1.882122	-2.013186	-1.716133	-0.131064	0.1659889	0.0174623	up
A_23_P83463	SEP15	6.0607605	6.0295625	6.126833	-0.031198	0.0660725	0.0174372	up
A_24_P193498	TM2D3	0.757185	0.6974821	0.8517008	-0.059703	0.0945158	0.0174065	up
A_23_P415558	ZNF212	0.9434485	0.9034901	1.0180345	-0.039958	0.0745859	0.0173137	up
A_19_P00810523	NDUFAF2	4.675294	4.5042872	4.8808403	-0.171007	0.2055464	0.0172699	up
A_23_P167389	ARAP3	3.8295393	3.6827035	4.0107574	-0.146836	0.1812182	0.0171912	up
A_23_P219084	ZNF3	1.999443	1.910296	2.122923	-0.089147	0.1234798	0.0171664	up
A_33_P3293675	SLC12A7	-3.486519	-3.250259	-3.688455	0.2362602	-0.201936	0.0171622	up
A_23_P128351	CRACR2A	-0.410264	-0.420133	-0.366084	-0.009869	0.0441804	0.0171556	up
A_22_P00012272	lnc-PPP1R3D-2	-1.665719	-1.886989	-1.41037	-0.22127	0.2553492	0.0170395	up
A_21_P0009392	lnc-RGS9-1	-0.778811	-0.698764	-0.824804	0.0800467	-0.045994	0.0170264	up
A_23_P160809	COG2	3.0776234	3.0168157	3.1724434	-0.060808	0.09482	0.0170062	up
A_23_P137238	KDM5D	0.3925028	0.4737492	0.3452196	0.0812464	-0.047283	0.0169816	up
A_21_P0004310	lnc-ARHGAP26-4	-3.123891	-2.995415	-3.218478	0.1284757	-0.094587	0.0169442	up
A_21_P0000684	LOC100506548	1.4823389	1.6957984	1.3025794	0.2134595	-0.17976	0.01685	up
A_21_P0000062	C22orf39	-2.224071	-2.323962	-2.090548	-0.099891	0.133523	0.0168159	up
A_23_P208143	ZNF397	-0.420732	-0.353794	-0.454158	0.0669375	-0.033427	0.0167553	up
A_22_P00011469	lnc-PAQR3-1	-2.378356	-2.39833	-2.324884	-0.019974	0.0534723	0.016749	up
A_21_P0004995	lnc-ECI2-3	-1.019973	-0.957296	-1.049246	0.0626774	-0.029273	0.0167022	up
A_23_P147388	KIF13B	2.8911772	2.745195	3.070486	-0.145982	0.1793089	0.0166633	up
A_24_P37264	RNF144A	-1.570285	-1.436744	-1.67062	0.1335406	-0.100335	0.0166028	up
A_24_P200000	STEAP3	0.5010886	0.4843307	0.5508618	-0.016758	0.0497732	0.0165076	up
A_21_P0001717	lnc-NLRP3-1	-0.84786	-1.320283	-0.342701	-0.472422	0.5051589	0.0163684	up
A_33_P3324206	HR	1.6659794	1.7620182	1.6025643	0.0960388	-0.063415	0.0163119	up
A_23_P154874	DSCR3	0.2418523	0.1622596	0.3539872	-0.079593	0.1121349	0.0162711	up
A_24_P43810	FAM83A	-2.731458	-2.721269	-2.709318	0.0101888	0.0221398	0.0161643	up
A_23_P162579	HSPB8	-0.05798	0.3532848	-0.436997	0.4112644	-0.379018	0.0161233	up
A_33_P3216237	BZW2	5.7472982	5.722675	5.80408	-0.024623	0.0567818	0.0160792	up
A_23_P203299	RCN1	5.072624	5.286609	4.8906956	0.213985	-0.181929	0.0160282	up
A_33_P3271800	SIRT2	0.2145572	0.2540603	0.2070823	0.0395031	-0.007475	0.0160141	up
A_23_P135690	CC2D1A	10.698201	10.712059	10.716272	0.0138578	0.0180712	0.0159645	up
A_32_P12327	USP46-AS1	-1.147261	-0.943745	-1.31893	0.203516	-0.171669	0.0159235	up
A_32_P209989	MRPL46	4.092016	4.194654	4.02122	0.1026378	-0.070796	0.0159209	up
A_23_P26895	TUBD1	1.0185566	1.0484581	1.0203581	0.0299015	0.0018015	0.0158515	up
A_33_P3414799	SH3BP2	-2.209717	-1.97039	-2.417573	0.2393274	-0.207855	0.015736	up
A_21_P0013089	XLOC_l2_013056	-0.305348	-0.189325	-0.389966	0.1160235	-0.084617	0.0157032	up
A_23_P9443	C9orf78	3.421689	3.394238	3.4805136	-0.027451	0.0588245	0.0156868	up
A_19_P00807046	lnc-ST3GAL1-1	0.7320371	0.7368736	0.7585702	0.0048366	0.0265331	0.0156848	up
A_33_P3398111	ZC3H18-AS1	-2.775842	-2.657426	-2.862903	0.1184158	-0.087062	0.015677	up
A_33_P3247180	CC2D1B	-0.20221	0.0479379	-0.421009	0.2501483	-0.218799	0.0156748	up
A_33_P3274304	WIPF2	5.5211544	5.6102147	5.463419	0.0890603	-0.057735	0.0156624	up
A_24_P345451	CYBRD1	0.7743001	0.9541097	0.6255975	0.1798096	-0.148703	0.0155535	up

A_23_P145016	BRD8	4.1390696	4.323195	3.9860334	0.1841254	-0.153036	0.0155447	up
A_22_P00006112	NR2F1-AS1	-1.290431	-0.839972	-1.709821	0.4504585	-0.41939	0.0155342	up
A_33_P3254320	SH2D3A	4.419653	4.482804	4.3874044	0.0631509	-0.032248	0.0154512	up
A_24_P227211	KLC2	-0.075539	-0.120959	0.0006056	-0.04542	0.0761447	0.0153623	up
A_32_P25273	HSPD1	8.923061	8.92705	8.949787	0.0039883	0.0267258	0.015357	up
A_23_P53788	MTIF3	3.5009289	3.451285	3.5811472	-0.049644	0.0802183	0.0152872	up
A_23_P354297	CHTF18	4.567336	4.6099753	4.5552588	0.0426393	-0.012077	0.015281	up
A_23_P118536	SLFN12	-0.578132	-0.49025	-0.635504	0.0878825	-0.057372	0.0152555	up
A_23_P122197	CCNB1	5.8971167	5.7637734	6.0609283	-0.133343	0.1638117	0.0152342	up
A_23_P81248	TAF7	5.886696	5.832022	5.9718065	-0.054674	0.0851107	0.0152185	up
A_23_P406341	AFAP1L2	1.7293587	1.622036	1.8671036	-0.107323	0.1377449	0.0152111	up
A_24_P351420	ZDHHC3	3.455719	3.3311496	3.6107073	-0.124569	0.1549883	0.0152094	up
A_33_P3253598	GYG2P1	-0.883834	-0.976757	-0.760518	-0.092923	0.1233168	0.015197	up
A_23_P48713	PTGR2	-0.042717	0.0237694	-0.078824	0.0664859	-0.036108	0.0151892	up
A_33_P3254306	FLCN	-2.624986	-2.394063	-2.825552	0.2309229	-0.200567	0.0151782	up
A_23_P117068	SNRPF	7.2434855	7.1753755	7.3419075	-0.06811	0.0984221	0.015156	up
A_33_P3377199	PRDX1	8.839584	8.64554	9.0637455	-0.194044	0.2241612	0.0150585	up
A_23_P348264	LETM2	-1.433199	-1.388499	-1.447859	0.0446997	-0.01466	0.0150199	up
A_22_P00006353	lnc-FARP1-1	-0.542082	-0.729857	-0.324307	-0.187775	0.2177749	0.0150001	up
A_21_P0010079	lnc-C20orf196-2	-1.630294	-1.618119	-1.612492	0.0121756	0.0178022	0.0149889	up
A_22_P00011997	lnc-PLCD1-1	-2.124095	-1.86074	-2.357596	0.2633557	-0.2335	0.0149277	up
A_33_P3333527	MSTO1	3.1383972	2.8518958	3.4547062	-0.286501	0.316309	0.0149038	up
A_21_P0005020	PSORS1C3	-2.69903	-2.465313	-2.903068	0.233717	-0.204038	0.0148396	up
A_24_P243776	ZNF277	0.2995901	0.496562	0.1321917	0.1969719	-0.167398	0.0147867	up
A_32_P110872	GGACT	-0.046156	-0.298738	0.2359858	-0.252582	0.2821417	0.0147798	up
A_24_P238744	POTEM	6.4846	6.4273887	6.5713634	-0.057211	0.0867634	0.014776	up
A_22_P00015371	lnc-SRBD1-1	-0.868854	-1.068835	-0.63937	-0.199981	0.2294846	0.0147519	up
A_24_P811704	PPFIBP1	3.1169682	3.056048	3.207384	-0.06092	0.090416	0.0147479	up
A_23_P71727	CKS2	7.0168695	6.8220487	7.2411766	-0.194821	0.2243071	0.0147431	up
A_24_P416131	COTL1	6.044656	6.0020223	6.1166945	-0.042634	0.0720387	0.0147026	up
A_22_P00000579	lnc-AC136604.1-1	-0.181395	-0.482739	0.1491737	-0.301344	0.3305688	0.0146124	up
A_33_P3311979	ZFPM1	-0.362408	-0.148699	-0.546895	0.2137089	-0.184487	0.0146108	up
A_33_P3397486	ENTPD4	0.8399568	0.9318366	0.7771559	0.0918798	-0.062801	0.0145395	up
A_21_P0008219	lnc-TUBGCP3-6	-0.867281	-0.683144	-1.022401	0.1841373	-0.15512	0.0145085	up
A_22_P00024240	lnc-FPGS-1	-1.140561	-1.102164	-1.150046	0.0383968	-0.009485	0.0144558	up
A_33_P3385002	ELK1	-3.163845	-3.052372	-3.246504	0.1114728	-0.08266	0.0144066	up
A_33_P3423425	ZNF770	3.1596365	3.181438	3.1665936	0.0218015	0.0069571	0.0143793	up
A_23_P19182	REEP2	1.6089964	0.8417101	2.4049873	-0.767286	0.7959909	0.0143523	up
A_21_P0011595	XLOC_l2_006101	-2.87644	-2.589155	-3.1352	0.2872841	-0.25876	0.014262	up
A_32_P11894	C12orf65	2.4860535	2.5808034	2.4197931	0.0947499	-0.06626	0.0142448	up
A_24_P928969	PTPN3	1.6762295	1.4823723	1.8984823	-0.193857	0.2222529	0.0141978	up
A_23_P259292	C1QTNF5	-0.057796	0.1809239	-0.268188	0.2387199	-0.210392	0.014164	up
A_33_P3728167	CDH5	-2.217294	-1.935766	-2.470587	0.281528	-0.253294	0.0141173	up
A_33_P3287203	SLC22A14	-0.901786	-0.740165	-1.035223	0.1616206	-0.133437	0.014092	up
A_22_P00024561	lnc-ROM1-2	-2.180776	-2.084895	-2.248591	0.0958805	-0.067815	0.0140327	up
A_24_P57528	SLC39A11	3.9941597	3.8456502	4.1704273	-0.14851	0.1762676	0.0138791	up
A_24_P944427	SETD5	-2.335345	-2.823209	-1.819755	-0.487864	0.5155897	0.013863	up
A_23_P83110	CDK5RAP2	3.0800495	3.1971765	2.9906063	0.1171269	-0.089443	0.0138419	up
A_24_P942805	PGP	0.8144851	0.6699495	0.9866557	-0.144536	0.1721706	0.0138176	up
A_24_P830690	PDPK1	0.4848905	0.5441265	0.4532104	0.0592361	-0.03168	0.013778	up
A_23_P23443	EFHD2	3.9538822	3.8923154	4.042985	-0.061567	0.0891027	0.013768	up
A_22_P00014615	LINC00508	-0.403859	-0.262029	-0.518169	0.1418295	-0.11431	0.0137596	up
A_24_P347480	NEK9	3.4646626	3.5068402	3.4499655	0.0421777	-0.014697	0.0137403	up
A_24_P138361	EIF3M	6.1724644	6.142678	6.2295437	-0.029787	0.0570793	0.0136464	up
A_33_P3211327	TMEM262	-1.757991	-1.797405	-1.691405	-0.039413	0.066586	0.0135863	up
A_22_P00000870	lnc-AGRP-3	-1.579789	-1.45889	-1.673631	0.1208987	-0.093842	0.0135286	up

A_33_P7518452	LRIG2	-1.208126	-1.18389	-1.205614	0.0242362	0.002512	0.0133741	up
A_21_P0011710	XLOC_l2_006812	-1.001302	-0.687408	-1.288603	0.3138938	-0.287301	0.0132964	up
A_23_P388433	C4orf3	3.6715117	3.3194203	4.050103	-0.352091	0.3785915	0.0132501	up
A_33_P3341329	ZCRB1	3.3581982	3.3246064	3.4182549	-0.033592	0.0600567	0.0132325	up
A_33_P3211734	SIKE1	3.525445	3.660954	3.416357	0.135509	-0.109088	0.0132105	up
A_23_P215037	WTAP	3.9740639	3.8535876	4.120841	-0.120476	0.1467772	0.0131505	up
A_24_P124558	HOXC8	0.5425162	0.3733964	0.7377005	-0.16912	0.1951842	0.0130322	up
A_22_P00007299	GPR37L1	-0.581178	-0.575159	-0.561366	0.0060186	0.0198116	0.0129151	up
A_23_P167599	FAM134B	-0.010477	-0.05097	0.0556827	-0.040493	0.0661597	0.0128336	up
A_23_P421423	TNFAIP2	5.433506	5.25767	5.6349487	-0.175836	0.2014427	0.0128033	up
A_33_P3365553	LINC00937	-1.580741	-1.775868	-1.360153	-0.195127	0.2205873	0.0127301	up
A_22_P00014167	lnc-SCRN3-3	-1.461126	-1.390895	-1.506067	0.0702314	-0.04494	0.0126455	up
A_23_P139919	CHST11	0.2662554	0.4237585	0.133996	0.1575031	-0.132259	0.0126219	up
A_21_P0007523	DDX11-AS1	0.4037595	0.3603163	0.4722867	-0.043443	0.0685272	0.012542	up
A_24_P19810	PPCS	3.4133234	3.5174928	3.3341808	0.1041694	-0.079143	0.0125134	up
A_23_P118174	PLK1	2.4857254	2.5398984	2.4564934	0.054173	-0.029232	0.0124705	up
A_21_P0003588	STIM2	-1.092109	-1.041646	-1.117771	0.0504627	-0.025662	0.0124004	up
A_33_P3700794	METTL1	2.1432562	2.205357	2.1057386	0.0621009	-0.037518	0.0122917	up
A_32_P1173	MB21D1	1.6251612	1.3431234	1.931695	-0.282038	0.3065338	0.012248	up
A_23_P332042	RECQL5	1.4088173	1.3854251	1.4566054	-0.023392	0.0477881	0.012198	up
A_33_P3368925	LRFN3	-1.899894	-2.113717	-1.661685	-0.213823	0.2382097	0.0121933	up
A_24_P335620	SLC7A5	5.1364613	5.374124	4.922991	0.2376628	-0.21347	0.0120962	up
A_22_P00002543	lnc-C17orf63-1	0.2034297	0.1468077	0.2841859	-0.056622	0.0807562	0.0120671	up
A_24_P68908	LOC344887	1.2095561	2.1345296	0.3086987	0.9249735	-0.900857	0.012058	up
A_32_P184727	KPNB1	6.89859	6.843374	6.9778814	-0.055216	0.0792913	0.0120375	up
A_21_P0000253	SNORD29	-0.063603	-0.05262	-0.050645	0.0109835	0.0129581	0.0119708	up
A_33_P3211198	NCMAP	-0.671522	-0.738754	-0.580581	-0.067232	0.0909414	0.0118547	up
A_33_P3341424	MYO10	1.9043455	1.8658419	1.9663725	-0.038504	0.062027	0.0117617	up
A_23_P76731	MOK	0.8301621	0.9439611	0.7398772	0.1137991	-0.090285	0.0117571	up
A_32_P222695	ARHGEF37	0.4955525	0.6336379	0.3809128	0.1380854	-0.11464	0.0117228	up
A_33_P3376463	NR2C2	0.0391378	0.1803427	-0.078788	0.1412048	-0.117926	0.0116396	up
A_23_P63219	POGZ	2.2080956	2.1988091	2.2406301	-0.009286	0.0325346	0.0116241	up
A_23_P52082	INTS7	1.6854124	1.7956605	1.5981841	0.1102481	-0.087228	0.0115099	up
A_23_P337934	FBLIM1	1.0680532	1.0160694	1.1427646	-0.051984	0.0747113	0.0113637	up
A_23_P44643	ANAPC7	1.9127512	1.7848139	2.0633783	-0.127937	0.1506271	0.0113449	up
A_23_P141917	TYK2	0.2607808	0.314466	0.2297654	0.0536852	-0.031015	0.0113349	up
A_22_P00013948	lnc-RSPH1-1	0.3700509	0.433712	0.3285861	0.0636611	-0.041465	0.0110981	up
A_23_P104651	CDCA5	5.8586855	5.8194337	5.9200926	-0.039252	0.0614071	0.0110776	up
A_33_P3252630	ZNF254	-1.699204	-1.561838	-1.814426	0.1373658	-0.115222	0.0110717	up
A_22_P00023682	lnc-PLEKHA3-2	-2.974661	-3.15428	-2.77298	-0.179618	0.2016809	0.0110313	up
A_33_P3396089	SLC38A6	-2.071856	-2.241604	-1.880076	-0.169748	0.1917796	0.0110157	up
A_22_P00012736	ATP2A1-AS1	0.7712336	0.7543602	0.81007	-0.016873	0.0388365	0.0109816	up
A_33_P3394933	P4HA2	0.9145474	1.0052366	0.8457365	0.0906892	-0.068811	0.0109391	up
A_24_P216421	DTNB	-2.199777	-1.748378	-2.629627	0.4513989	-0.42985	0.0107745	up
A_24_P183264	BTF3	6.1461945	6.2740397	6.0398903	0.1278453	-0.106304	0.0107706	up
A_23_P17490	TMEM230	3.4000168	3.5390038	3.2825508	0.1389871	-0.117466	0.0107605	up
A_24_P15754	TOMM40	3.1553783	3.0921798	3.2400036	-0.063199	0.0846252	0.0107133	up
A_23_P59261	TPBG	2.4002638	2.2932172	2.5286608	-0.107047	0.128397	0.0106752	up
A_33_P3352307	RPS6KA1	-0.261197	-0.29567	-0.20541	-0.034472	0.0557866	0.0106571	up
A_21_P0008254	lnc-LCP1-1	-1.323739	-1.40738	-1.218872	-0.083641	0.1048675	0.0106132	up
A_21_P0000659	UBAC2-AS1	0.589756	0.5738568	0.6268778	-0.015899	0.0371218	0.0106113	up
A_23_P345460	PLEKHG4	2.7664566	2.545383	3.0084734	-0.221074	0.2420168	0.0104716	up
A_32_P214284	LOC100129516	-1.813755	-2.128885	-1.477722	-0.31513	0.3360329	0.0104513	up
A_23_P383977	SNAPC5	1.3587098	1.2194629	1.5187998	-0.139247	0.16009	0.0104215	up
A_33_P3275290	GLT8D1	0.4488769	0.5143914	0.4041381	0.0655146	-0.044739	0.0103879	up
A_23_P24104	PLAU	0.6205125	0.8356004	0.426158	0.2150879	-0.194355	0.0103667	up

A_24_P145047	ZNF609	-0.01985	-0.200208	0.1811819	-0.180358	0.2010317	0.0103369	up
A_33_P3216433	TPM3P9	0.6735158	0.6608081	0.7067285	-0.012708	0.0332127	0.0102525	up
A_33_P3312509	ADAMTSL2	3.3346663	3.5355763	3.1539745	0.2009101	-0.180692	0.0101092	up
A_22_P00018020	lnc-ZNF205-1	-0.850114	-0.981617	-0.698467	-0.131502	0.1516476	0.0100727	up
A_33_P3332885	BTN2A1	-0.262595	-0.241295	-0.263824	0.0212998	-0.001229	0.0100353	up
A_33_P3413962	PFKP	4.5070972	4.436706	4.5975075	-0.070391	0.0904102	0.0100095	up
A_22_P00009393	MCM3AP	0.8128734	0.3120523	1.3337078	-0.500821	0.5208345	0.0100067	up
A_23_P23457	FBLIM1	-1.771575	-2.016433	-1.506715	-0.244859	0.2648597	0.0100005	up
A_21_P0014918	HERC2P4	-0.647096	-0.439796	-0.834433	0.2072992	-0.187337	0.0099812	up
A_22_P00014477	lnc-SHARPIN-2	1.0730057	0.9595523	1.2063036	-0.113453	0.1332979	0.0099223	up
A_33_P3219895	KLC2	5.0397196	4.8307242	5.268551	-0.208995	0.2288313	0.009918	up
A_21_P0000040	ZBTB24	-0.666547	-0.677397	-0.635863	-0.010849	0.0306845	0.0099175	up
A_32_P88310	LOC730183	0.3184829	0.3102827	0.3463693	-0.0082	0.0278864	0.0098431	up
A_24_P916141	DCAF7	3.2976952	3.0666184	3.5482893	-0.231077	0.2505941	0.0097587	up
A_23_P33196	COL5A2	-1.488692	-1.433946	-1.523925	0.0547466	-0.035233	0.009757	up
A_33_P3251283	CTAGE4	-0.137911	-0.003041	-0.253388	0.1348701	-0.115477	0.0096965	up
A_24_P96474	LDOC1L	3.4070253	3.4245896	3.408801	0.0175643	0.0017757	0.00967	up
A_23_P137705	TBX19	-0.331492	-0.439879	-0.203804	-0.108387	0.1276889	0.0096509	up
A_24_P279797	MRI1	0.2897406	0.2963667	0.3022561	0.0066261	0.0125155	0.0095708	up
A_22_P00000282	MIR940	2.0758543	2.0564046	2.11442	-0.01945	0.0385656	0.009558	up
A_33_P3277614	OLA1	6.3663588	6.3640704	6.3877583	-0.002288	0.0213995	0.0095556	up
A_33_P3268343	PGAM4	6.8932447	6.7317758	7.0737257	-0.161469	0.180481	0.009506	up
A_22_P00004847	LOC100133669	-2.731634	-2.830288	-2.614004	-0.098653	0.1176305	0.0094886	up
A_21_P0007321	lnc-SOX6-1	-0.342149	-0.250629	-0.414904	0.0915203	-0.072754	0.009383	up
A_23_P3856	ZFP1	-0.486739	-0.372762	-0.581981	0.1139765	-0.095243	0.009367	up
A_33_P3361417	C6orf57	0.7669263	0.5201016	1.0324664	-0.246825	0.2655401	0.0093577	up
A_24_P842006	C16orf93	-2.49557	-2.600465	-2.372152	-0.104895	0.1234176	0.0092611	up
A_33_P3372869	DDX52	-0.371011	-0.492245	-0.23146	-0.121234	0.1395512	0.0091586	up
A_23_P46924	BUB3	3.4634	3.453092	3.4919596	-0.010308	0.0285597	0.0091259	up
A_24_P392480	MYPOP	-1.246667	-1.06433	-1.410803	0.1823378	-0.164135	0.0091011	up
A_22_P00011141	LOC101928273	-2.292612	-2.311884	-2.255356	-0.019273	0.037256	0.0089917	up
A_23_P68730	PDXK	4.031026	3.7553716	4.3244286	-0.275654	0.2934027	0.0088742	up
A_24_P206343	MYO1G	-1.334444	-0.95249	-1.698731	0.3819532	-0.364288	0.0088327	up
A_33_P3351101	TYSND1	-1.232746	-1.514751	-0.933149	-0.282005	0.2995968	0.0087957	up
A_23_P398491	C15orf57	-0.24096	-0.2687	-0.195647	-0.02774	0.0453134	0.0087869	up
A_22_P00015029	HOXC-AS1	-1.424928	-1.723313	-1.109043	-0.298385	0.3158851	0.00875	up
A_24_P270033	MPZL3	3.737916	3.8001752	3.693139	0.0622592	-0.044777	0.0087411	up
A_21_P0012508	AADACP1	-2.244914	-2.388913	-2.083478	-0.144	0.1614356	0.008718	up
A_33_P3248072	SIRPB1	2.280222	2.0769296	2.5008745	-0.203292	0.2206526	0.0086801	up
A_23_P130764	KCNJ14	1.9656382	1.9788203	1.9695778	0.0131822	0.0039396	0.0085609	up
A_33_P3276918	FAM64A	0.4362688	0.3023109	0.587316	-0.133958	0.1510472	0.0085447	up
A_32_P85539	HCFC2	-0.398711	-0.293506	-0.486877	0.1052046	-0.088166	0.0085192	up
A_33_P3213645	ERN2	0.9669228	1.0839148	0.8669109	0.116992	-0.100012	0.0084901	up
A_21_P0000595	SNORA70E	1.9796696	2.0995975	1.8766756	0.1199279	-0.102994	0.008467	up
A_33_P3347928	CCNL1	2.2511473	2.3255973	2.1936064	0.07445	-0.057541	0.0084546	up
A_22_P00000004	lnc-WISP1-1	-1.538771	-1.274282	-1.786366	0.2644892	-0.247595	0.0084472	up
A_23_P167269	TMA16	2.5226088	2.5539937	2.508113	0.0313849	-0.014496	0.0084445	up
A_23_P92642	ANKHD1	2.805273	2.9477239	2.679657	0.1424508	-0.125616	0.0084174	up
A_24_P807031	ATP6AP1L	-1.80016	-1.552299	-2.031205	0.2478614	-0.231045	0.0084083	up
A_33_P3343552	CAPN10	-1.189362	-1.39183	-0.970121	-0.202467	0.2192411	0.0083869	up
A_23_P216476	ZBTB5	2.9997606	3.0804772	2.9358063	0.0807166	-0.063954	0.0083811	up
A_23_P202345	ADO	0.6227922	0.4272847	0.8350477	-0.195508	0.2122555	0.008374	up
A_33_P3241591	LOC100131864	-2.505957	-2.694946	-2.300289	-0.18899	0.2056675	0.0083389	up
A_33_P3383756	WHSC1L1	-0.88539	-0.911271	-0.84285	-0.02588	0.0425401	0.0083299	up
A_33_P3333820	UBE2R2	2.2010765	2.3785644	2.0402203	0.1774879	-0.160856	0.0083158	up
A_23_P38959	VAV1	-3.212918	-3.222347	-3.187054	-0.009429	0.0258641	0.0082176	up

A_33_P3423859	PPP1R3D	1.9436474	1.9741693	1.9295311	0.0305219	-0.014116	0.0082028	up
A_23_P95923	ZNF562	1.3991275	1.4590931	1.3554664	0.0599656	-0.043661	0.0081522	up
A_23_P331813	ZNF687	0.7916341	0.5668583	1.0327015	-0.224776	0.2410674	0.0081458	up
A_23_P127128	DNAJC1	4.168724	4.16091	4.192749	-0.007814	0.024025	0.0081055	up
A_33_P3306442	QRICH2	-2.426415	-2.232275	-2.604435	0.1941409	-0.17802	0.0080605	up
A_33_P3247988	PCSK4	-0.96339	-0.918166	-0.992748	0.0452237	-0.029358	0.0079329	up
A_23_P14340	KIAA0391	1.4520531	1.4052167	1.5146546	-0.046836	0.0626016	0.0078826	up
A_24_P339869	ZBTB21	0.0686417	0.0860934	0.0669498	0.0174518	-0.001692	0.00788	up
A_24_P29001	LSM3	6.443001	6.4651256	6.436615	0.0221248	-0.006386	0.0078695	up
A_33_P6814367	PAX8-AS1	-2.222465	-2.391871	-2.037322	-0.169406	0.185143	0.0078683	up
A_21_P0009754	lnc-UQCRFS1-9	-0.608027	-0.423713	-0.776607	0.1843143	-0.16858	0.0078673	up
A_22_P00011243	lnc-OR1F1-1	-0.867514	-0.942154	-0.777287	-0.07464	0.0902271	0.0077937	up
A_23_P33856	STAG2	0.2090845	0.4297643	0.0039015	0.2206798	-0.205183	0.0077484	up
A_24_P295601	ATG13	1.0458665	0.9191446	1.1879911	-0.126722	0.1421247	0.0077014	up
A_23_P155301	NEK11	-1.373692	-1.28686	-1.445331	0.0868321	-0.071639	0.0075967	up
A_32_P25050	RDH10	3.6210442	3.5912538	3.6659937	-0.02979	0.0449495	0.0075796	up
A_33_P3346098	TAOK2	1.6590433	1.5705752	1.7626524	-0.088468	0.1036091	0.0075705	up
A_23_P210886	BCL2L1	2.6711416	2.525146	2.8322582	-0.145996	0.1611166	0.0075605	up
A_33_P3396877	RPP38	3.2483444	3.136166	3.3755102	-0.112178	0.1271658	0.0074937	up
A_22_P00009887	lnc-MFSD4-1	-1.745065	-1.478217	-1.997129	0.2668481	-0.252064	0.0073919	up
A_21_P0000315	SNORA18	2.0090656	1.9752336	2.0576801	-0.033832	0.0486145	0.0073912	up
A_23_P58588	SLIT3	-2.595346	-2.829429	-2.346482	-0.234083	0.2488644	0.0073907	up
A_33_P3594214	OR7E12P	1.2788677	1.4228091	1.1494966	0.1439414	-0.129371	0.0072851	up
A_33_P3342126	PRDM8	-1.509105	-1.512998	-1.490985	-0.003893	0.0181198	0.0071132	up
A_33_P3390057	TM4SF1	6.846319	6.8048882	6.901925	-0.041431	0.0556059	0.0070875	up
A_23_P41204	FAM131A	-1.023104	-1.111395	-0.920714	-0.088292	0.1023898	0.0070491	up
A_32_P10272	PMS2P5	1.9124994	1.9192424	1.9196539	0.006743	0.0071545	0.0069487	up
A_33_P3334185	AGTPBP1	-1.384529	-1.291094	-1.464135	0.0934353	-0.079606	0.0069149	up
A_24_P181585	LRRC59	5.661436	5.5754848	5.7611475	-0.085951	0.0997114	0.00688	up
A_22_P00000913	lnc-AK1-1	-2.315491	-2.307131	-2.310105	0.0083606	0.0053864	0.0068735	up
A_32_P32391	OR7E156P	1.4960365	1.6559434	1.3497849	0.1599069	-0.146252	0.0068276	up
A_24_P382765	NHP2L1	4.5853205	4.6033397	4.580921	0.0180192	-0.004399	0.00681	up
A_21_P0007029	FLJ37035	-0.85542	-0.318058	-1.379253	0.5373621	-0.523834	0.0067642	up
A_23_P100441	FAM192A	2.9088573	2.8738732	2.9573565	-0.034984	0.0484991	0.0067575	up
A_33_P3316310	MTERF4	0.1050434	0.3024502	-0.078875	0.1974068	-0.183918	0.0067441	up
A_23_P57059	STAU1	4.739049	4.4817576	5.0098057	-0.257291	0.2707567	0.0067327	up
A_23_P135966	SLC25A14	3.3074799	3.1678834	3.460413	-0.139596	0.1529331	0.0066683	up
A_24_P636974	lnc-SEPT7L-1	-2.09731	-2.000607	-2.180688	0.0967026	-0.083379	0.006662	up
A_22_P00005497	DYX1C1	1.5080085	1.4871297	1.5421977	-0.020879	0.0341892	0.0066552	up
A_23_P79942	PANK2	3.3418026	3.3727765	3.32413	0.0309739	-0.017673	0.0066507	up
A_33_P3255741	ZNF81	-1.939008	-1.557168	-2.307585	0.3818398	-0.368577	0.0066313	up
A_23_P124024	MED10	1.7445698	1.7080493	1.7943411	-0.03652	0.0497713	0.0066254	up
A_24_P295590	RASSF4	-2.674961	-2.54186	-2.794818	0.133101	-0.119858	0.0066217	up
A_23_P34606	MTOR	0.7241492	0.7854519	0.6760669	0.0613027	-0.048082	0.0066102	up
A_21_P0000309	SNORA11	-1.497896	-1.547617	-1.435185	-0.049721	0.0627117	0.0064955	up
A_23_P201596	AMPD2	2.2260733	2.07404	2.3910933	-0.152033	0.16502	0.0064933	up
A_23_P29225	FBXO7	5.0446987	4.9548926	5.147481	-0.089806	0.1027823	0.0064881	up
A_23_P210015	PTPN18	0.7465868	0.3656392	1.140419	-0.380948	0.3938322	0.0064423	up
A_23_P91697	LARGE	1.6788654	1.7597098	1.6107507	0.0808444	-0.068115	0.0063648	up
A_23_P373649	TIMM23	4.483946	4.3644834	4.6160393	-0.119462	0.1320934	0.0063155	up
A_33_P3231542	ZFHX3	2.668562	2.880506	2.4692135	0.2119441	-0.199348	0.0062978	up
A_23_P84922	HDAC8	-0.423226	-0.366296	-0.467575	0.0569301	-0.044349	0.0062907	up
A_24_P165423	RBP7	-2.215481	-2.553998	-1.864393	-0.338517	0.3510876	0.0062853	up
A_23_P258251	ENOX2	1.9754639	2.1705527	1.792923	0.1950889	-0.182541	0.006274	up
A_23_P120146	TGFBRAP1	3.0563793	3.0129237	3.112257	-0.043456	0.0558777	0.006211	up
A_23_P257956	ILF2	5.744193	5.648826	5.8518934	-0.095367	0.1077004	0.0061667	up

A_23_P140309	MAPK1IP1L	5.4665127	5.3039804	5.641341	-0.162532	0.1748285	0.0061481	up
A_23_P119448	PPP6R1	-0.278282	-0.39254	-0.151786	-0.114258	0.1264954	0.0061188	up
A_22_P00009064	lnc-LEO1-4	-3.177078	-3.082422	-3.259631	0.0946565	-0.082553	0.0060518	up
A_23_P206077	AEN	1.6593218	1.5838008	1.7467041	-0.075521	0.0873823	0.0059307	up
A_21_P0009752	lnc-UQCRFS1-9	-0.443458	-0.186338	-0.688818	0.2571197	-0.24536	0.0058799	up
A_24_P156388	TTC38	0.4961042	0.4342375	0.5695295	-0.061867	0.0734253	0.0057793	up
A_22_P00003891	lnc-CEP152-1	0.350523	0.0597124	0.6528039	-0.290811	0.3022809	0.0057352	up
A_33_P3243649	LOC100131831	-0.835583	-0.663639	-0.996151	0.1719446	-0.160568	0.0056884	up
A_22_P00012139	POLR2C	-0.640056	-0.78844	-0.480336	-0.148384	0.1597199	0.0056682	up
A_23_P118749	DERL2	3.1165056	3.065639	3.1786814	-0.050867	0.0621758	0.0056546	up
A_33_P3417339	SHROOM3	0.2481432	0.169764	0.3377576	-0.078379	0.0896144	0.0056176	up
A_23_P429977	KCNQ1	1.1229019	1.2280607	1.0289702	0.1051588	-0.093932	0.0056136	up
A_21_P0014077	LOC100505666	-0.949644	-0.753518	-1.134608	0.1961265	-0.184964	0.0055814	up
A_23_P92967	MOCS2	2.4380074	2.3243728	2.56256	-0.113635	0.1245527	0.0054591	up
A_24_P163113	CDV3	5.318343	5.465387	5.182212	0.1470437	-0.136131	0.0054562	up
A_21_P0014039	FBRSL1	-0.217435	-0.221297	-0.202664	-0.003862	0.014771	0.0054545	up
A_33_P3415062	KIAA1671	-0.141415	-0.163424	-0.108615	-0.022009	0.0328002	0.0053954	up
A_33_P3284508	CD14	0.9430766	0.8230767	1.0738525	-0.12	0.1307759	0.005388	up
A_24_P68819	PPP1R21	0.3204064	0.4070735	0.2444744	0.0866671	-0.075932	0.0053675	up
A_33_P3421794	DHX34	-2.648353	-2.732629	-2.553373	-0.084276	0.0949805	0.0053524	up
A_33_P3249773	TATDN3	0.6465368	0.6862741	0.6174612	0.0397372	-0.029076	0.0053308	up
A_32_P103837	FAM60A	3.2734108	3.0929723	3.4644356	-0.180439	0.1910248	0.0052931	up
A_23_P86195	SLC44A3	2.5033169	2.5412984	2.4758692	0.0379815	-0.027448	0.0052669	up
A_22_P00007338	LOC100506314	-0.782339	-0.773711	-0.780559	0.0086279	0.00178	0.005204	up
A_23_P60016	PTTG3P	1.2722654	1.2363749	1.3185072	-0.035891	0.0462418	0.0051756	up
A_23_P213085	SLC10A7	-0.9904	-1.115232	-0.855298	-0.124832	0.1351023	0.0051353	up
A_23_P129221	FAH	2.8477736	2.643355	3.062316	-0.204419	0.2145424	0.0050619	up
A_33_P3339336	FAM86FP	0.909729	0.8572006	0.9723697	-0.052528	0.0626407	0.0050561	up
A_23_P122052	GPX8	2.438179	2.6543221	2.2320461	0.2161431	-0.206133	0.0050051	up
A_23_P130064	SNF8	5.3209515	5.104646	5.5471764	-0.216305	0.2262249	0.0049598	up
A_33_P3273136	MCUR1	2.8696527	2.826919	2.9222918	-0.042734	0.052639	0.0049527	up
A_33_P3211809	RUNX1	-0.827188	-0.850127	-0.794634	-0.02294	0.0325532	0.0048068	up
A_33_P3235987	PIN4	3.4136076	3.4297996	3.4067965	0.016192	-0.006811	0.0046904	up
A_33_P3340792	DHDDS	-1.411324	-1.345594	-1.467699	0.0657301	-0.056375	0.0046775	up
A_33_P3343972	RSPH1	-0.605241	-0.873122	-0.328009	-0.267881	0.2772317	0.0046752	up
A_24_P180680	LAPTM4B	7.5433445	7.5034127	7.5925865	-0.039932	0.049242	0.0046551	up
A_22_P00009527	lnc-MAOA-1	-0.94011	-0.932846	-0.938123	0.0072637	0.001987	0.0046253	up
A_24_P136683	CA5BP1	-0.983353	-1.004157	-0.953374	-0.020804	0.0299788	0.0045874	up
A_33_P3210059	SF3A1	3.3275814	3.2786012	3.385685	-0.04898	0.0581036	0.0045617	up
A_19_P00808883	Q7Z2X8	-1.211128	-1.219559	-1.19394	-0.00843	0.0171881	0.0043788	up
A_23_P117580	ENTPD5	-2.570765	-2.538131	-2.594812	0.0326343	-0.024048	0.0042933	up
A_24_P109554	CNTRL	-2.750616	-2.67142	-2.821258	0.0791962	-0.070642	0.0042771	up
A_33_P3372667	lnc-VIP-1	-2.435024	-2.286242	-2.575348	0.1487825	-0.140324	0.0042294	up
A_22_P00010734	lnc-NIF3L1-1	-0.419557	-0.503909	-0.326785	-0.084352	0.092772	0.00421	up
A_24_P15062	ZNF490	-1.465672	-1.363321	-1.559673	0.1023512	-0.094001	0.0041752	up
A_23_P48099	NUP37	3.926445	3.9685311	3.8926668	0.0420861	-0.033778	0.004154	up
A_23_P257971	AKR1C1	2.8106794	3.533277	2.0963697	0.7225976	-0.71431	0.0041439	up
A_33_P3419098	PLEC	0.0009265	-0.120936	0.1310048	-0.121862	0.1300783	0.004108	up
A_24_P414376	KLF3	3.273203	3.4311757	3.1233969	0.1579728	-0.149806	0.0040834	up
A_33_P3404775	PABPC4	5.9193306	5.92034	5.9264774	0.0010095	0.0071468	0.0040781	up
A_23_P1602	CDC42EP2	3.0115318	2.9067388	3.1244373	-0.104793	0.1129055	0.0040562	up
A_23_P593	GPBP1L1	2.952837	3.1385818	2.7751484	0.1857448	-0.177689	0.0040281	up
A_32_P170749	STAG3	3.0127525	2.9994283	3.033967	-0.013324	0.0212145	0.0039451	up
A_22_P00015967	lnc-TEFM-5	-1.58606	-1.640797	-1.523488	-0.054737	0.0625725	0.0039177	up
A_23_P203957	TMTC1	-0.418197	-0.202291	-0.626335	0.2159057	-0.208138	0.0038838	up
A_33_P3364379	YIF1A	3.4183407	3.03976	3.804593	-0.378581	0.3862524	0.0038359	up

A_22_P00019235	LOC101559451	-0.612641	-0.710493	-0.507258	-0.097852	0.1053829	0.0037653	up
A_23_P131375	PQLC3	0.4498143	0.4961581	0.4109907	0.0463438	-0.038824	0.0037601	up
A_23_P42514	C6orf62	4.0972586	3.9692674	4.2327223	-0.127991	0.1354637	0.0037363	up
A_33_P3270485	CBX6	6.8539896	6.7985177	6.9169064	-0.055472	0.0629168	0.0037224	up
A_33_P3369079	CHKB-AS1	-0.065698	-0.127206	0.0032148	-0.061508	0.068913	0.0037024	up
A_33_P3284686	SSBP3	-1.590845	-1.65774	-1.516635	-0.066895	0.0742097	0.0036573	up
A_23_P109442	HPS4	3.1489048	3.2912984	3.0136023	0.1423936	-0.135303	0.0035455	up
A_23_P64630	RNF26	4.4172735	4.236282	4.605097	-0.180992	0.1878233	0.0034158	up
A_24_P287075	MAP4K2	-0.22352	-0.254401	-0.185825	-0.030881	0.0376949	0.003407	up
A_23_P152768	TUBG1	2.0742044	1.9422116	2.2129526	-0.131993	0.1387482	0.0033777	up
A_32_P128209	LINC01554	-1.691493	-1.739735	-1.636546	-0.048242	0.0549474	0.0033526	up
A_23_P28420	OLA1	3.9894361	4.0424066	3.9431095	0.0529704	-0.046327	0.0033219	up
A_33_P3398091	ZNF562	1.6863775	1.7447104	1.6345124	0.0583329	-0.051865	0.0032339	up
A_23_P67980	KLF7	-2.106689	-2.127273	-2.079702	-0.020584	0.0269871	0.0032017	up
A_33_P3423435	ZNF710	-1.37348	-1.708999	-1.031865	-0.335519	0.3416152	0.0030482	up
A_24_P371628	ANKH	-0.287594	-0.404413	-0.164712	-0.116819	0.1228819	0.0030315	up
A_33_P3294986	LIPE	4.0390234	3.911756	4.172288	-0.127267	0.1332645	0.0029986	up
A_32_P192615	TAF1	2.2285013	2.3454785	2.1174908	0.1169772	-0.111011	0.0029833	up
A_23_P169934	RILPL1	0.6885352	0.5681911	0.8148127	-0.120344	0.1262775	0.0029666	up
A_23_P14493	DNAAF2	2.3721542	2.2322583	2.5179415	-0.139896	0.1457872	0.0029457	up
A_24_P93967	FMR1	2.6468496	2.6705089	2.6288958	0.0236592	-0.017954	0.0028527	up
A_23_P255286	SMPD4	7.3166637	7.1515937	7.4872713	-0.16507	0.1706076	0.0027688	up
A_24_P9883	AP5B1	-0.520792	-0.578178	-0.457888	-0.057386	0.0629039	0.002759	up
A_33_P3263387	TTLL4	0.1792884	0.1430774	0.220654	-0.036211	0.0413656	0.0025773	up
A_33_P3390539	GYLTL1B	0.6128144	0.5403819	0.6903868	-0.072433	0.0775723	0.0025699	up
A_23_P364478	FAM175B	0.5082932	0.4094977	0.6121464	-0.098795	0.1038532	0.0025289	up
A_23_P61180	PLCXD1	2.7230434	2.8213868	2.629733	0.0983434	-0.09331	0.0025165	up
A_24_P418809	GNAS	6.725032	6.7950253	6.659812	0.0699935	-0.06522	0.0023868	up
A_24_P576174	DCP1A	1.5917654	1.5880032	1.6002183	-0.003762	0.0084529	0.0023453	up
A_23_P12896	FANCF	1.4183841	1.2541099	1.5872827	-0.164274	0.1688986	0.0023122	up
A_33_P3392325	CDC16	4.898267	4.917995	4.883157	0.0197282	-0.01511	0.0023091	up
A_23_P103070	YWHAH	3.4433289	3.287335	3.6039238	-0.155994	0.1605949	0.0023005	up
A_23_P201248	SLC26A9	2.4997673	2.456039	2.5478811	-0.043728	0.0481138	0.0021927	up
A_24_P81947	CORO1C	4.075815	3.8547025	4.301243	-0.221113	0.2254276	0.0021574	up
A_24_P123119	EHHADH	-2.116839	-2.202427	-2.026997	-0.085588	0.0898414	0.0021265	up
A_33_P3315258	CHD1L	0.2246485	0.2629395	0.1905899	0.038291	-0.034059	0.0021162	up
A_33_P3242778	LOC100129888	-1.807457	-1.714042	-1.896663	0.0934153	-0.089205	0.002105	up
A_23_P80278	SFI1	2.675889	2.6675181	2.6883469	-0.008371	0.0124578	0.0020435	up
A_33_P3390576	KMT2A	-0.934765	-0.833163	-1.032437	0.1016016	-0.097672	0.0019646	up
A_21_P0010840	XLOC_l2_001689	-1.663892	-1.604177	-1.71977	0.0597153	-0.055879	0.0019183	up
A_22_P00015572	lnc-STT3B-1	-0.242747	-0.213328	-0.268377	0.0294189	-0.02563	0.0018947	up
A_24_P198820	CDK12	3.928297	3.8756433	3.9847002	-0.052654	0.0564032	0.0018747	up
A_33_P3253747	CYP1A2	-1.940645	-1.714831	-2.16278	0.2258139	-0.222136	0.0018392	up
A_24_P282762	TLK1	-1.231511	-0.876072	-1.583324	0.3554387	-0.351812	0.0018132	up
A_24_P166407	HIST1H4B	6.5417976	6.5138326	6.573372	-0.027965	0.0315743	0.0018046	up
A_23_P212159	NUP210	1.520544	1.6523738	1.392199	0.1318297	-0.128345	0.0017424	up
A_32_P90080	ARMC10	3.014554	3.083054	2.9494543	0.0685	-0.0651	0.0017002	up
A_24_P921477	METTL23	1.4397693	1.5403628	1.3425517	0.1005936	-0.097218	0.001688	up
A_24_P38276	FZD1	1.9119177	1.8421855	1.9850245	-0.069732	0.0731068	0.0016873	up
A_24_P408424	MYH9	5.578059	5.4701524	5.689233	-0.107907	0.1111736	0.0016334	up
A_21_P0000362	SNORA1	0.8968239	0.8158093	0.9809852	-0.081015	0.0841613	0.0015733	up
A_22_P00003769	lnc-CDK17-1	-2.649791	-2.769755	-2.52672	-0.119965	0.123071	0.0015532	up
A_23_P146084	GSR	1.7548418	1.8624654	1.6502833	0.1076236	-0.104558	0.0015326	up
A_33_P3217609	TBC1D22A	-2.124699	-1.739367	-2.506986	0.3853326	-0.382287	0.0015227	up
A_21_P0001367	lnc-WDR3-2	-2.760503	-2.936793	-2.58124	-0.176289	0.1792634	0.001487	up
A_24_P181944	PHF20	0.0381284	0.221982	-0.142752	0.1838536	-0.180881	0.0014865	up

A_23_P18078	RARRES1	-1.239776	-1.199938	-1.276645	0.0398378	-0.036869	0.0014844	up
A_23_P96976	ZCCHC17	3.4340353	3.426169	3.444809	-0.007866	0.0107737	0.0014536	up
A_22_P00015024	SMTN	0.3695478	0.3069067	0.4349141	-0.062641	0.0653663	0.0013626	up
A_33_P3236102	IER5L	3.1968966	3.052652	3.3437328	-0.144245	0.1468363	0.0012958	up
A_22_P00013526	LINC00421	-2.502695	-2.425209	-2.577675	0.0774863	-0.07498	0.0012531	up
A_33_P3243544	ESRRA	2.9043465	2.9217181	2.8891735	0.0173717	-0.015173	0.0010993	up
A_24_P284523	MAP3K10	1.5960894	1.709672	1.4846344	0.1135826	-0.111455	0.0010638	up
A_33_P3287907	ZBED3-AS1	-1.920045	-1.72578	-2.112376	0.1942654	-0.192331	0.0009673	up
A_33_P3316045	INTS1	6.615596	6.5528703	6.6801853	-0.062726	0.0645895	0.000932	up
A_22_P00011068	lnc-NUAK1-2	-1.931285	-2.080587	-1.780321	-0.149302	0.1509638	0.0008309	up
A_33_P3423830	TCAF1	-1.986405	-2.198008	-1.773422	-0.211602	0.2129831	0.0006905	up
A_33_P3416563	AIG1	2.9282494	2.8188443	3.0390244	-0.109405	0.110775	0.000685	up
A_23_P167805	B3GAT2	-1.921683	-2.154068	-1.688028	-0.232385	0.233655	0.000635	up
A_33_P3423931	LCN9	-2.027329	-2.3329	-1.720621	-0.305571	0.3067083	0.0005685	up
A_33_P3397655	SYNPO	-0.794513	-0.516642	-1.071722	0.2778711	-0.277209	0.0003309	up
A_33_P3871347	SNED1	-0.536548	-0.636287	-0.436333	-0.099739	0.1002154	0.0002382	up
A_24_P63030	SFXN5	0.1895456	0.0755782	0.3039808	-0.113967	0.1144352	0.0002339	up
A_23_P255257	DCAF12	0.890564	0.9292216	0.8523235	0.0386577	-0.03824	0.0002086	up
A_23_P90453	KRTDAP	-0.302446	-0.289783	-0.314746	0.0126634	-0.0123	0.0001817	up
A_33_P3325467	SMIM12	2.3584518	2.2419353	2.4752817	-0.116517	0.1168299	0.0001566	up
A_21_P0000108	MTRNR2L8	6.1883183	6.2478776	6.1290665	0.0595593	-0.059252	0.0001538	up
A_33_P3350227	MED22	-0.623133	-0.563914	-0.682066	0.0592189	-0.058932	0.0001433	up
A_23_P141636	EIF4A3	6.5265093	6.5251956	6.5280676	-0.001314	0.0015583	0.0001223	up
A_21_P0011036	LOC374443	0.8826652	0.7967606	0.968647	-0.085905	0.0859818	3.862E-05	up
A_33_P3217213	PDLIM7	1.0490122	1.1059461	0.9920135	0.0569339	-0.056999	-3.24E-05	down
A_23_P166269	FAM3B	-1.631843	-1.546971	-1.716851	0.0848713	-0.085009	-6.87E-05	down
A_24_P204244	ANXA2P1	6.806817	6.9013524	6.7120466	0.0945354	-0.09477	-0.000118	down
A_33_P3334515	NDRG2	3.7895746	3.6015334	3.9772272	-0.188041	0.1876526	-0.000194	down
A_23_P131227	TTC27	3.7826586	3.7128024	3.8520184	-0.069856	0.0693598	-0.000248	down
A_33_P3342106	ZHX3	-1.030023	-1.240443	-0.820244	-0.210421	0.2097783	-0.000321	down
A_21_P0000331	SNORA44	0.2758918	0.1067605	0.4443283	-0.169131	0.1684365	-0.000347	down
A_22_P00009113	THAP9-AS1	-1.541997	-1.321758	-1.762964	0.2202392	-0.220967	-0.000364	down
A_23_P135995	ARV1	2.4464893	2.3598952	2.5323257	-0.086594	0.0858364	-0.000379	down
A_19_P00320570	lnc-UBLCP1-4	-0.291335	-0.420577	-0.162869	-0.129241	0.1284657	-0.000388	down
A_22_P00009791	lnc-MEGF10-1	1.0955219	0.9044666	1.2857122	-0.191055	0.1901903	-0.000432	down
A_33_P3232504	CYSRT1	5.8726654	5.903091	5.8413286	0.0304255	-0.031337	-0.000456	down
A_32_P148345	ANXA2	4.3664274	4.4401307	4.291807	0.0737033	-0.07462	-0.000458	down
A_24_P356601	HEXIM1	2.0795755	1.9956326	2.162487	-0.083943	0.0829115	-0.000516	down
A_23_P171117	MORC4	2.006319	2.0150266	1.9965382	0.0087075	-0.009781	-0.000537	down
A_23_P409516	FAM122A	1.5635614	1.5650382	1.560842	0.0014768	-0.002719	-0.000621	down
A_21_P0007437	LOC101927495	1.3400502	1.2877469	1.3909516	-0.052303	0.0509014	-0.000701	down
A_33_P6703419	ATG9B	0.1371789	0.1569514	0.1158705	0.0197725	-0.021308	-0.000768	down
A_24_P190877	TRMT1L	-1.248682	-1.2447	-1.254349	0.0039821	-0.005667	-0.000843	down
A_33_P3343210	SLC25A37	1.8754282	1.9249625	1.8241343	0.0495343	-0.051294	-0.00088	down
A_23_P41437	UFSP2	2.680544	2.7796245	2.5796652	0.0990806	-0.100879	-0.000899	down
A_33_P3293213	RGL3	0.3217716	0.434125	0.2074904	0.1123533	-0.114281	-0.000964	down
A_24_P15043	KLHL18	1.2981215	1.1400394	1.4541907	-0.158082	0.1560693	-0.001006	down
A_33_P3335590	CCDC64B	4.743058	4.878465	4.6055794	0.135407	-0.137479	-0.001036	down
A_23_P200056	NADK	0.9313073	0.7453647	1.1150823	-0.185943	0.183775	-0.001084	down
A_23_P65699	SPG11	2.942297	3.0273995	2.8549652	0.0851026	-0.087332	-0.001115	down
A_32_P103945	B4GALT4	1.3959365	1.3355546	1.4540701	-0.060382	0.0581336	-0.001124	down
A_33_P3239532	ZNF3	-1.361434	-1.462184	-1.262959	-0.100751	0.0984745	-0.001138	down
A_23_P334021	IGF2R	3.2701092	3.3281236	3.2095547	0.0580144	-0.060555	-0.00127	down
A_24_P169773	CTAGE4	2.40588	2.4814754	2.327609	0.0755954	-0.078271	-0.001338	down
A_23_P314584	MAPKAPK3	3.6364918	3.0916514	4.178645	-0.54484	0.5421534	-0.001343	down
A_23_P401055	SOX2	2.4423914	2.526301	2.3557863	0.0839095	-0.086605	-0.001348	down

A_23_P152330	DOC2A	-2.163716	-2.333108	-1.997068	-0.169391	0.1666484	-0.001372	down
A_23_P38427	RAB11FIP4	2.340973	2.1335998	2.5454416	-0.207373	0.2044687	-0.001452	down
A_23_P91814	SLC25A38	0.6788755	0.443161	0.9116602	-0.235714	0.2327848	-0.001465	down
A_23_P211738	UBP1	3.49831	3.468924	3.5246563	-0.029386	0.0263462	-0.00152	down
A_23_P29282	NPTXR	-2.321533	-2.576456	-2.069858	-0.254923	0.2516756	-0.001624	down
A_23_P342091	OARD1	3.2793207	3.4778857	3.077218	0.198565	-0.202103	-0.001769	down
A_33_P3355296	D2HGDH	-0.132851	-0.041376	-0.227906	0.091475	-0.095055	-0.00179	down
A_24_P328504	SP140	0.9445772	0.8519368	1.0335479	-0.09264	0.0889707	-0.001835	down
A_33_P6608390	YTHDF3	-1.653968	-1.780823	-1.530928	-0.126855	0.1230407	-0.001907	down
A_33_P3258452	C12orf5	0.9528546	0.7418556	1.1600204	-0.210999	0.2071657	-0.001917	down
A_32_P18470	TCEAL5	0.7289524	0.5658388	0.8882265	-0.163114	0.1592741	-0.00192	down
A_21_P0004659	LOC101928353	3.8696508	3.995954	3.739297	0.1263032	-0.130354	-0.002025	down
A_33_P3420078	LRP11	-1.139159	-0.97933	-1.303094	0.1598291	-0.163935	-0.002053	down
A_24_P382253	RPRD1A	2.0271807	2.0417295	2.0084248	0.0145488	-0.018756	-0.002104	down
A_33_P3243929	FLJ42022	-2.915465	-2.721508	-3.113917	0.1939569	-0.198452	-0.002248	down
A_23_P70480	HIST1H4L	7.5318985	7.5017667	7.557461	-0.030132	0.0255623	-0.002285	down
A_23_P130974	KIAA1683	-1.242392	-1.215098	-1.274509	0.0272942	-0.032117	-0.002412	down
A_24_P40529	TMLHE	-0.907484	-1.052088	-0.767905	-0.144604	0.1395788	-0.002513	down
A_33_P3424467	CNST	0.1437202	0.2485685	0.0338435	0.1048484	-0.109877	-0.002514	down
A_33_P3386429	SENP6	-2.051659	-1.957786	-2.150585	0.093873	-0.098926	-0.002527	down
A_33_P3238920	INPP5F	-0.567962	-0.594115	-0.546868	-0.026154	0.0210934	-0.00253	down
A_23_P30363	P4HA2	5.057824	5.098228	5.0122347	0.0404038	-0.045589	-0.002593	down
A_22_P00012764	ZNF462	-2.116518	-2.034276	-2.204027	0.082242	-0.087508	-0.002633	down
A_33_P3348494	PCID2	5.019536	5.033361	5.000429	0.0138249	-0.019107	-0.002641	down
A_33_P3310070	FOXK2	-0.689399	-0.683436	-0.700923	0.0059633	-0.011523	-0.00278	down
A_23_P416305	RPL27A	-1.778084	-1.828828	-1.73293	-0.050744	0.0451541	-0.002795	down
A_22_P00003880	lnc-CENPP-1	-0.079601	-0.049167	-0.115777	0.0304341	-0.036176	-0.002871	down
A_24_P307974	TAF8	0.7128964	0.8984761	0.5214853	0.1855798	-0.191411	-0.002916	down
A_23_P66766	RPAIN	3.1045685	3.2346253	2.9686213	0.1300569	-0.135947	-0.002945	down
A_22_P00002971	lnc-C6orf112-2	-2.710358	-2.814994	-2.611783	-0.104635	0.0985754	-0.00303	down
A_22_P00002481	lnc-C15orf57-2	-2.304678	-2.133488	-2.48207	0.1711891	-0.177392	-0.003102	down
A_21_P0000756	LINC01012	-0.301202	-0.313358	-0.295437	-0.012156	0.0057654	-0.003195	down
A_21_P0001900	GACAT1	-0.217969	-0.135784	-0.307082	0.0821857	-0.089113	-0.003464	down
A_22_P00017352	GABPB1-AS1	-0.471411	-0.39339	-0.556398	0.0780206	-0.084988	-0.003484	down
A_21_P0000034	COX11	2.004304	1.9801145	2.0214758	-0.024189	0.0171719	-0.003509	down
A_33_P3369034	BCAR1	6.6231174	6.5558105	6.6833363	-0.067307	0.0602188	-0.003544	down
A_21_P0007476	LINC00592	-3.012375	-3.053792	-2.978178	-0.041417	0.0341966	-0.00361	down
A_33_P3474250	TRIM44	1.8061666	1.9505954	1.6544237	0.1444287	-0.151743	-0.003657	down
A_33_P3281468	STARD9	-2.856082	-3.092609	-2.626902	-0.236527	0.2291794	-0.003674	down
A_22_P00005112	lnc-DHRS7B-3	-1.097508	-1.071503	-1.130964	0.0260043	-0.033457	-0.003726	down
A_23_P321855	ARHGEF7	2.239544	2.2898164	2.1817904	0.0502725	-0.057754	-0.003741	down
A_32_P195065	SEMA4F	-0.469703	-0.937838	-0.009181	-0.468134	0.4605217	-0.003806	down
A_23_P133245	IK	4.7269573	4.622679	4.823597	-0.104278	0.0966396	-0.003819	down
A_32_P9468	COX19	1.9453516	1.8262215	2.0567493	-0.11913	0.1113977	-0.003866	down
A_23_P101796	SYDE1	-2.61025	-2.884613	-2.343652	-0.274363	0.2665982	-0.003882	down
A_21_P0000876	TMEM161B-AS1	0.8452697	0.8182268	0.864522	-0.027043	0.0192523	-0.003895	down
A_22_P00021161	lnc-MYBBP1A-2	-0.628511	-0.786423	-0.478395	-0.157913	0.150115	-0.003899	down
A_24_P79153	SCAMP4	-0.265315	-0.317779	-0.220741	-0.052464	0.0445743	-0.003945	down
A_23_P214638	EHMT2	1.0948186	1.2181897	0.9635401	0.1233711	-0.131279	-0.003954	down
A_33_P3391578	RPL23AP64	-1.592801	-1.666499	-1.527013	-0.073698	0.0657878	-0.003955	down
A_33_P3603812	FRMD8	-2.328504	-2.4007	-2.264547	-0.072196	0.0639572	-0.004119	down
A_33_P3393135	HIST1H2AI	-0.628641	-0.736791	-0.528845	-0.10815	0.0997958	-0.004177	down
A_23_P201647	SMG7	2.0496407	1.8831172	2.207755	-0.166523	0.1581144	-0.004205	down
A_33_P3272080	QRFP	0.3584838	0.3908157	0.3175044	0.0323319	-0.040979	-0.004324	down
A_23_P121011	CSRNP1	-0.901502	-0.731044	-1.080957	0.1704578	-0.179455	-0.004499	down
A_23_P94174	MRPL15	4.406273	4.381799	4.4217224	-0.024474	0.0154495	-0.004512	down

A_19_P00806930	XLOC_l2_003627	-1.285039	-1.541363	-1.037828	-0.256324	0.247211	-0.004556	down
A_21_P0014815	lnc-TTR-1	-1.508617	-1.651245	-1.375218	-0.142628	0.133399	-0.004614	down
A_21_P0009252	lnc-ABI3-1	-2.320525	-2.505802	-2.144509	-0.185277	0.1760159	-0.004631	down
A_33_P3397443	PKMYT1	5.742714	5.7901816	5.685933	0.0474677	-0.056781	-0.004657	down
A_23_P10858	ANKLE2	1.2517524	1.3717985	1.1223207	0.1200461	-0.129432	-0.004693	down
A_24_P393372	PACS2	2.0282974	2.1256356	1.921299	0.0973382	-0.106998	-0.00483	down
A_24_P332623	CTAGE11P	1.5831728	1.7073393	1.449336	0.1241665	-0.133837	-0.004835	down
A_22_P00003080	lnc-C8orf56-1	-2.98874	-3.186122	-2.801034	-0.197382	0.1877062	-0.004838	down
A_22_P00022160	lnc-TXLNB-3	-2.275886	-2.431766	-2.129864	-0.15588	0.1460218	-0.004929	down
A_22_P00009992	RFX2	-1.972856	-2.10207	-1.853597	-0.129214	0.1192594	-0.004977	down
A_22_P00004926	PRR7-AS1	-2.333465	-2.083942	-2.59298	0.2495222	-0.259515	-0.004997	down
A_33_P3352134	ATG16L1	-1.592805	-1.711816	-1.483833	-0.119011	0.1089726	-0.005019	down
A_24_P38347	DPYSL2	0.9931274	0.8896318	1.0864134	-0.103496	0.093286	-0.005105	down
A_33_P3417745	IQGAP1	6.0227776	5.9307933	6.1045275	-0.091984	0.0817499	-0.005117	down
A_33_P3543013	OR5AK4P	-2.507851	-2.537703	-2.488238	-0.029852	0.0196133	-0.005119	down
A_33_P3242304	TNP2	-2.314632	-2.166479	-2.473126	0.1481531	-0.158494	-0.005171	down
A_33_P3287348	CHN2	-2.845292	-2.760969	-2.940094	0.0843227	-0.094802	-0.00524	down
A_33_P3383029	MXI1	1.4762626	1.6579618	1.2839751	0.1816993	-0.192287	-0.005294	down
A_23_P99614	BTBD6	5.2471466	5.2727904	5.2108335	0.0256438	-0.036313	-0.005335	down
A_24_P21410	NOL6	1.3692131	1.1780653	1.5495992	-0.191148	0.1803861	-0.005381	down
A_23_P5325	ERCC3	2.0482588	2.0453973	2.0403576	-0.002861	-0.007901	-0.005381	down
A_24_P942112	POLR1B	-0.256969	-0.354646	-0.170063	-0.097677	0.0869064	-0.005385	down
A_23_P69249	ACTL6A	5.1472683	4.9741616	5.3095694	-0.173107	0.1623011	-0.005403	down
A_23_P432056	RTN4RL1	-1.920382	-1.699566	-2.152023	0.2208152	-0.231642	-0.005413	down
A_23_P153301	CEACAM5	-1.348529	-1.514066	-1.193898	-0.165537	0.1546311	-0.005453	down
A_24_P377489	CCZ1	4.8779135	4.81128	4.9335375	-0.066634	0.055624	-0.005505	down
A_33_P3337272	NRARP	5.639723	5.6285872	5.639778	-0.011136	5.531E-05	-0.00554	down
A_24_P46093	SLC6A6	2.3772373	2.2096686	2.533679	-0.167569	0.1564417	-0.005563	down
A_24_P142442	POLDIP2	5.0625563	5.275987	4.837942	0.2134309	-0.224614	-0.005592	down
A_22_P00015532	lnc-STK24-1	-0.354503	-0.575302	-0.144965	-0.220799	0.2095375	-0.005631	down
A_21_P0000126	TGFBR3L	-1.980677	-2.306633	-1.665998	-0.325955	0.3146796	-0.005638	down
A_23_P158938	AP3M1	1.1805377	1.0521622	1.2976098	-0.128376	0.1170721	-0.005652	down
A_21_P0002188	lnc-SLC4A1AP-1	-1.63459	-1.733155	-1.54737	-0.098565	0.0872207	-0.005672	down
A_21_P0008402	lnc-C14orf79-2	-1.22023	-1.075837	-1.376182	0.1443925	-0.155952	-0.00578	down
A_33_P6472709	SYNJ2-IT1	-3.182085	-2.967676	-3.408069	0.2144089	-0.225984	-0.005788	down
A_21_P0005656	LOC100506990	-1.041431	-1.210419	-0.884066	-0.168989	0.1573644	-0.005812	down
A_33_P3406961	XLOC_l2_013467	-0.421557	-0.547042	-0.307882	-0.125485	0.1136746	-0.005905	down
A_23_P158775	UMOD	-1.634021	-1.414033	-1.866009	0.2199884	-0.231988	-0.006	down
A_33_P3297978	MYO1E	4.4937706	4.3772035	4.5983152	-0.116567	0.1045446	-0.006011	down
A_33_P3209162	APOC4	-2.845608	-2.772637	-2.930625	0.0729713	-0.085016	-0.006022	down
A_22_P00002636	lnc-C1orf177-2	-1.478497	-1.537009	-1.432089	-0.058512	0.0464072	-0.006052	down
A_21_P0014606	LOC101929516	-0.785429	-0.791883	-0.791131	-0.006454	-0.005702	-0.006078	down
A_33_P3281036	HCG11	3.6680164	3.7006364	3.6231585	0.03262	-0.044858	-0.006119	down
A_21_P0011183	XLOC_l2_003992	-1.456461	-1.316646	-1.608575	0.1398149	-0.152115	-0.00615	down
A_22_P00000603	lnc-ACCSL-1	0.5605245	0.4070129	0.701714	-0.153512	0.1411896	-0.006161	down
A_23_P97365	LRRC8D	2.3967009	2.2673984	2.513545	-0.129303	0.1168442	-0.006229	down
A_23_P217151	KRBOX4	-0.485602	-0.314646	-0.669241	0.1709561	-0.18364	-0.006342	down
A_24_P45005	NPEPL1	2.988634	2.9519982	3.0125675	-0.036636	0.0239334	-0.006351	down
A_24_P151582	TEF	0.6087985	0.4700756	0.7347741	-0.138723	0.1259756	-0.006374	down
A_24_P942211	SLC35E2	2.0327883	2.1321998	1.9205112	0.0994115	-0.112277	-0.006433	down
A_24_P294851	TRIM38	-0.846429	-0.857699	-0.848048	-0.01127	-0.001618	-0.006444	down
A_24_P37409	DUSP2	1.4983196	1.5603299	1.4233346	0.0620103	-0.074985	-0.006487	down
A_23_P99186	UBE3B	-1.649372	-1.755444	-1.556286	-0.106072	0.0930858	-0.006493	down
A_23_P214211	RARS2	5.3424788	5.366076	5.305765	0.0235972	-0.036714	-0.006558	down
A_21_P0011029	LINC01347	-1.814072	-1.603853	-2.037499	0.2102184	-0.223427	-0.006604	down
A_23_P77590	VAC14	2.0768948	1.9055891	2.2348423	-0.171306	0.1579475	-0.006679	down

A_32_P516818	PQLC2L	-1.512586	-1.837167	-1.201374	-0.324581	0.3112121	-0.006685	down
A_24_P329600	UBQLN1	4.00875	4.0220447	3.9820824	0.0132947	-0.026668	-0.006686	down
A_21_P0014618	LOC101928521	-2.644328	-2.947166	-2.354902	-0.302838	0.2894259	-0.006706	down
A_23_P137035	PIR	5.071435	5.090059	5.0393324	0.0186238	-0.032103	-0.006739	down
A_22_P00025549	lnc-NEDD4L-2	-1.591141	-1.618918	-1.576999	-0.027777	0.014142	-0.006818	down
A_22_P00011218	lnc-OPN4-1	-0.541238	-0.547772	-0.548448	-0.006534	-0.007209	-0.006871	down
A_22_P00018048	ZNF275	-0.589711	-0.774854	-0.418314	-0.185143	0.1713963	-0.006874	down
A_23_P41854	CARD6	2.1693974	2.0774856	2.2473412	-0.091912	0.0779438	-0.006984	down
A_24_P277615	TRIM7	-2.495378	-2.428633	-2.576178	0.0667446	-0.080801	-0.007028	down
A_23_P100486	VKORC1	3.1431847	2.6542506	3.6180515	-0.488934	0.4748669	-0.007034	down
A_22_P00004317	PSMD5-AS1	0.4451675	0.506022	0.3702302	0.0608544	-0.074937	-0.007041	down
A_23_P15272	ABCC6	0.93469	0.7858496	1.0693779	-0.14884	0.1346879	-0.007076	down
A_24_P374962	STAG3L2	1.0521765	1.083807	1.0063143	0.0316305	-0.045862	-0.007116	down
A_21_P0004481	lnc-NKD2-3	-0.511732	-0.665541	-0.372171	-0.15381	0.1395607	-0.007124	down
A_24_P135406	KCTD9	2.0902386	1.9702153	2.1959515	-0.120023	0.1057129	-0.007155	down
A_23_P120973	FAM118A	0.0627527	0.0423188	0.068624	-0.020434	0.0058713	-0.007281	down
A_33_P3305840	SRSF7	5.5991726	5.4501004	5.733673	-0.149072	0.1345005	-0.007286	down
A_24_P309645	TPCN1	1.4801502	1.5913033	1.3543944	0.1111531	-0.125756	-0.007301	down
A_33_P3346302	UBAP2	1.7221785	1.6505837	1.7791495	-0.071595	0.0569711	-0.007312	down
A_33_P3288039	ATP9B	-0.818192	-0.705342	-0.945666	0.1128497	-0.127474	-0.007312	down
A_33_P3417810	NOL10	4.2150126	4.1193633	4.2960186	-0.095649	0.0810061	-0.007322	down
A_21_P0006148	lnc-FAM27D1.1-3	-2.813614	-2.798394	-2.84355	0.0152206	-0.029935	-0.007357	down
A_33_P3340164	NDUFB5	2.7764387	2.7799726	2.75803	0.0035338	-0.018409	-0.007437	down
A_24_P129277	NOD1	-0.27357	-0.23574	-0.326309	0.0378294	-0.05274	-0.007455	down
A_23_P50775	LRFN3	4.1805763	3.860124	4.485998	-0.320452	0.3054218	-0.007515	down
A_33_P3323136	ENKUR	-1.024123	-1.051144	-1.012166	-0.027021	0.0119576	-0.007532	down
A_21_P0011770	LINC01535	1.5349483	1.5637007	1.4910831	0.0287523	-0.043865	-0.007556	down
A_23_P214882	MTRF1L	0.3684096	0.3177552	0.403872	-0.050654	0.0354624	-0.007596	down
A_24_P205364	SHMT1	-1.699183	-1.528884	-1.884855	0.1702991	-0.185672	-0.007686	down
A_23_P39647	SLC4A3	-0.106345	-0.071286	-0.156782	0.035059	-0.050437	-0.007689	down
A_24_P317835	INPP5E	2.9889603	2.950994	3.011548	-0.037966	0.0225878	-0.007689	down
A_23_P309292	HECTD4	0.086443	0.0980039	0.0594249	0.0115609	-0.027018	-0.007729	down
A_33_P3239148	ZNF548	-1.122373	-0.729698	-1.53053	0.3926749	-0.408157	-0.007741	down
A_33_P3242603	ASH1L-AS1	1.306674	1.1292052	1.4686103	-0.177469	0.1619363	-0.007766	down
A_23_P153930	ACVR2A	0.0552764	0.052877	0.04211	-0.002399	-0.013166	-0.007783	down
A_21_P0010069	lnc-RP11-93B14.6.1-4	-1.643648	-1.415172	-1.887817	0.2284761	-0.244169	-0.007847	down
A_33_P3359724	C1orf194	-2.286005	-2.271508	-2.316198	0.0144968	-0.030193	-0.007848	down
A_33_P3217958	FCHSD2	-3.102236	-3.211286	-3.008939	-0.10905	0.0932975	-0.007876	down
A_23_P344037	CHFR	-0.735593	-0.71235	-0.774614	0.0232425	-0.039021	-0.00789	down
A_32_P187599	SERBP1	6.7160273	6.6784863	6.7376995	-0.037541	0.0216722	-0.007934	down
A_24_P320545	PTK7	-0.003471	0.0207872	-0.043607	0.0242581	-0.040136	-0.007939	down
A_23_P77661	ZNF720	0.6873484	0.6368828	0.7218933	-0.050466	0.0345449	-0.00796	down
A_23_P395524	PWP1	5.795313	5.7910037	5.7836523	-0.004309	-0.011661	-0.007985	down
A_23_P254031	TTF1	1.9395256	1.8806157	1.9821911	-0.05891	0.0426655	-0.008122	down
A_22_P00009989	lnc-MLH3-1	-2.607956	-2.329057	-2.903225	0.2788994	-0.29527	-0.008185	down
A_23_P115316	TOR3A	3.650012	3.4917111	3.7919092	-0.158301	0.1418972	-0.008202	down
A_23_P317691	UIMC1	3.5543814	3.5212607	3.5710526	-0.033121	0.0166712	-0.008225	down
A_33_P3260373	ISY1-RAB43	-2.25102	-2.568488	-1.950096	-0.317468	0.3009238	-0.008272	down
A_19_P00317647	LINC01278	-1.274678	-1.534346	-1.031566	-0.259668	0.2431126	-0.008278	down
A_23_P128624	CARS2	6.4888325	6.524568	6.4363537	0.0357356	-0.052479	-0.008372	down
A_22_P00023466	lnc-ANKRD10-2	-0.265427	-0.347394	-0.200309	-0.081967	0.0651178	-0.008425	down
A_33_P3354881	LOC100506753	-0.745853	-0.766466	-0.742193	-0.020612	0.0036607	-0.008476	down
A_23_P500799	CASP6	3.2150898	3.1589704	3.2541256	-0.056119	0.0390358	-0.008542	down
A_33_P3317589	GFRA4	-2.502677	-2.688553	-2.334021	-0.185876	0.1686559	-0.00861	down
A_21_P0009249	lnc-ARL4D-1	-3.248329	-3.188863	-3.325041	0.0594661	-0.076712	-0.008623	down

A_23_P32233	KLF4	1.4070754	1.6418028	1.1546717	0.2347274	-0.252404	-0.008838	down
A_33_P3214481	P4HA1	1.6038666	1.478241	1.7116809	-0.125626	0.1078143	-0.008906	down
A_23_P150286	PSMA1	5.7549477	5.8372684	5.654788	0.0823207	-0.10016	-0.008919	down
A_33_P3297302	ELMOD3	-2.140071	-2.196392	-2.101593	-0.056321	0.0384784	-0.008921	down
A_23_P68949	ST13	4.7471485	4.709082	4.767332	-0.038066	0.0201836	-0.008941	down
A_23_P431981	HMGXB4	1.9714174	1.9444747	1.9803677	-0.026943	0.0089502	-0.008996	down
A_33_P3406899	TRAK1	1.7446918	1.8850923	1.5862699	0.1404004	-0.158422	-0.009011	down
A_33_P3349469	ATAD3A	5.8399773	5.709472	5.9524126	-0.130505	0.1124353	-0.009035	down
A_33_P3231367	ATXN10	1.9694672	1.9264607	1.9943476	-0.043006	0.0248804	-0.009063	down
A_24_P222599	PDPK1	1.7435541	1.7280521	1.7408862	-0.015502	-0.002668	-0.009085	down
A_23_P25525	GTF3A	5.603202	5.3554378	5.8327103	-0.247764	0.2295084	-0.009128	down
A_19_P00320971	LINC01192	-2.085764	-1.960068	-2.229765	0.1256957	-0.144001	-0.009153	down
A_23_P169838	SUN1	4.6656866	4.7355256	4.5775394	0.069839	-0.088147	-0.009154	down
A_23_P36972	ZIC2	-2.174584	-2.065927	-2.301589	0.1086578	-0.127004	-0.009173	down
A_23_P67829	UXS1	3.0313559	2.9752707	3.069086	-0.056085	0.0377302	-0.009177	down
A_23_P11160	MAP7D3	0.1252542	0.0761781	0.1559472	-0.049076	0.0306931	-0.009192	down
A_33_P3284253	GYG2	0.036273	-0.271496	0.3255744	-0.307769	0.2893014	-0.009234	down
A_33_P3278407	MLLT4	3.9073362	3.9924273	3.8036203	0.0850911	-0.103716	-0.009312	down
A_23_P168828	KLF10	3.0062695	3.0960212	2.8978872	0.0897517	-0.108382	-0.009315	down
A_33_P3282973	BAALC	-1.683086	-1.704922	-1.679891	-0.021836	0.0031958	-0.00932	down
A_32_P80850	COL14A1	-0.386866	-0.419125	-0.373271	-0.032259	0.0135951	-0.009332	down
A_23_P11279	ALG13	1.8891802	1.6491098	2.1105824	-0.24007	0.2214022	-0.009334	down
A_23_P149375	THEM4	0.5667424	0.4732876	0.6413984	-0.093455	0.074656	-0.009399	down
A_21_P0000131	CAMTA1	4.671109	4.5562415	4.7671423	-0.114868	0.0960331	-0.009417	down
A_33_P3209476	CCM2	1.3654323	1.0200944	1.6919346	-0.345338	0.3265023	-0.009418	down
A_23_P11408	PRY2	0.9716497	1.21596	0.7084332	0.2443104	-0.263217	-0.009453	down
A_23_P114164	UBQLN2	2.7451372	2.5289197	2.9423504	-0.216218	0.1972132	-0.009502	down
A_33_P3380346	WHSC1	-1.003729	-1.117387	-0.909144	-0.113658	0.0945849	-0.009537	down
A_33_P3287922	TTI1	4.4638042	4.332046	4.5763845	-0.131758	0.1125803	-0.009589	down
A_24_P379750	MXD1	1.520627	1.5712328	1.4507494	0.0506058	-0.069878	-0.009636	down
A_23_P167308	RNF4	3.9229155	3.8834567	3.9430952	-0.039459	0.0201797	-0.00964	down
A_23_P154938	HIRA	4.399003	4.222324	4.556368	-0.176679	0.1573649	-0.009657	down
A_23_P78014	TUBG2	1.5318508	1.4422746	1.6020675	-0.089576	0.0702167	-0.00968	down
A_22_P00020402	lnc-RALGAPA2-2	-2.677514	-2.107136	-3.267303	0.5703783	-0.589788	-0.009705	down
A_33_P3324065	FBF1	-2.167753	-1.99385	-2.361136	0.173903	-0.193383	-0.00974	down
A_19_P00320158	SEC14L1	1.2349167	1.0753837	1.3749332	-0.159533	0.1400166	-0.009758	down
A_23_P152919	NUP88	3.3517504	3.4715514	3.2123652	0.119801	-0.139385	-0.009792	down
A_33_P3234667	ZKSCAN1	1.3852477	1.5435925	1.207233	0.1583448	-0.178015	-0.009835	down
A_23_P140511	CLPX	2.9222517	2.9223504	2.9024553	9.871E-05	-0.019796	-0.009849	down
A_23_P164797	ZNF580	3.0409365	2.9069648	3.1551247	-0.133972	0.1141882	-0.009892	down
A_23_P97623	RRAGC	2.3556967	2.5246882	2.1667852	0.1689916	-0.188911	-0.00996	down
A_23_P500501	FGFR3	0.9854097	0.8425484	1.1082559	-0.142861	0.1228461	-0.010008	down
A_33_P3230688	CDK10	3.983036	3.877543	4.0684414	-0.105493	0.0854054	-0.010044	down
A_23_P209987	POLR1B	2.8079357	2.6602569	2.9354506	-0.147679	0.1275148	-0.010082	down
A_23_P14124	RASL11A	-1.619074	-1.749598	-1.508764	-0.130524	0.1103096	-0.010107	down
A_33_P3299110	ARHGAP42	-1.684489	-1.866128	-1.52315	-0.18164	0.1613393	-0.01015	down
A_32_P17635	SRSF8	3.157095	2.956777	3.3370218	-0.200318	0.1799269	-0.010195	down
A_33_P3336422	ZBED5-AS1	1.3372903	1.2193007	1.4348412	-0.11799	0.0975509	-0.010219	down
A_24_P388252	PPP3R1	1.5832376	1.2544518	1.891489	-0.328786	0.3082514	-0.010267	down
A_33_P3374049	KIAA2013	1.15594	0.9384694	1.3528032	-0.217471	0.1968632	-0.010304	down
A_23_P74581	SNHG12	4.221546	4.3773847	4.045061	0.1558385	-0.176485	-0.010323	down
A_23_P138137	OMA1	0.9087248	0.7771792	1.0196185	-0.131546	0.1108937	-0.010326	down
A_23_P435941	SAMD1	1.1892214	1.2591505	1.0983219	0.0699291	-0.090899	-0.010485	down
A_23_P7684	CCNJL	-0.153565	-0.186014	-0.142223	-0.032449	0.0113416	-0.010554	down
A_22_P00019085	lnc-CLCNKB-2	-2.640351	-2.635745	-2.666073	0.004606	-0.025722	-0.010558	down
A_23_P369987	AREL1	2.753971	2.8706374	2.6160984	0.1166663	-0.137873	-0.010603	down

A_33_P3244083	KIF13B	-0.729394	-0.91387	-0.566261	-0.184476	0.1631327	-0.010672	down
A_19_P00322088	LOC388210	-3.165446	-3.103834	-3.248453	0.0616119	-0.083007	-0.010697	down
A_23_P163408	SCAPER	-0.867111	-0.792058	-0.963615	0.0750532	-0.096504	-0.010725	down
A_23_P31143	TPD52L1	5.72536	5.7709765	5.6582375	0.0456166	-0.067122	-0.010753	down
A_23_P413634	ZNF329	-0.78582	-0.595358	-0.99787	0.1904616	-0.21205	-0.010794	down
A_23_P166122	POLR3F	0.2300892	0.2492704	0.1889305	0.0191813	-0.041159	-0.010989	down
A_23_P320159	TCEANC	-0.754376	-0.86862	-0.662113	-0.114244	0.0922632	-0.01099	down
A_33_P3564399	SH3GL1P2	-1.317626	-1.270159	-1.387151	0.0474663	-0.069525	-0.011029	down
A_23_P436526	SLC25A42	-0.888042	-0.912985	-0.885158	-0.024943	0.0028839	-0.01103	down
A_23_P138856	DRAP1	6.274208	6.3415856	6.1847363	0.0673776	-0.089472	-0.011047	down
A_23_P134744	RNF122	-0.005308	-0.089177	0.0564342	-0.083869	0.0617418	-0.011064	down
A_23_P212213	THUMPD3	2.5039387	2.3929973	2.5925598	-0.110941	0.0886211	-0.01116	down
A_23_P132910	RBM47	2.396449	2.2314305	2.5388308	-0.165019	0.1423817	-0.011318	down
A_24_P233488	LIF	-2.408324	-2.203774	-2.635534	0.20455	-0.22721	-0.01133	down
A_23_P121657	HS3ST1	0.9905772	0.8930092	1.0654597	-0.097568	0.0748825	-0.011343	down
A_23_P19852	IQCE	4.267598	4.0772176	4.4352074	-0.190381	0.1676092	-0.011386	down
A_33_P3304528	SENP1	1.8193951	1.8520689	1.7638702	0.0326738	-0.055525	-0.011425	down
A_22_P00014796	LINC00673	0.8626175	0.8584976	0.843677	-0.00412	-0.01894	-0.01153	down
A_23_P54900	UBN1	3.7692862	3.738563	3.7769136	-0.030723	0.0076275	-0.011548	down
A_23_P36183	GTF2H1	1.9814396	1.9376445	2.0020704	-0.043795	0.0206308	-0.011582	down
A_23_P125624	ACOT9	5.0938435	5.266228	4.8981657	0.1723847	-0.195678	-0.011647	down
A_23_P374902	CLDND2	-1.791475	-1.967558	-1.638707	-0.176083	0.1527686	-0.011657	down
A_32_P411592	MBD6	2.1702404	2.2142034	2.1029425	0.043963	-0.067298	-0.011667	down
A_33_P3245922	MAP3K4	2.9905872	2.98313	2.97466	-0.007457	-0.015927	-0.011692	down
A_22_P00002639	lnc-C1orf185-2	-3.083442	-3.079564	-3.110946	0.0038779	-0.027504	-0.011813	down
A_22_P00003503	LOC100129931	-2.564056	-2.551168	-2.600595	0.0128877	-0.036538	-0.011825	down
A_33_P3387796	PHLDB1	2.272336	2.321797	2.1991205	0.0494609	-0.073215	-0.011877	down
A_32_P494620	LHFPL5	-2.352077	-2.452527	-2.275455	-0.10045	0.076622	-0.011914	down
A_23_P200443	SHC1	4.371517	4.286947	4.432193	-0.08457	0.0606756	-0.011947	down
A_33_P3229477	MPP7	-0.812387	-0.842301	-0.806531	-0.029914	0.005857	-0.012028	down
A_23_P87013	TAGLN	-0.105699	-0.02038	-0.215083	0.0853186	-0.109384	-0.012033	down
A_23_P44546	DFFB	-1.28174	-1.330626	-1.256956	-0.048885	0.0247846	-0.01205	down
A_24_P228637	SETD4	1.677597	1.5025301	1.8284807	-0.175067	0.1508837	-0.012092	down
A_23_P52826	BUD13	3.0458937	2.9241672	3.1432314	-0.121727	0.0973377	-0.012194	down
A_23_P120594	ACSS1	-0.583114	-0.474137	-0.716687	0.1089773	-0.133573	-0.012298	down
A_23_P73548	GRIPAP1	1.8050928	1.7961545	1.7890153	-0.008938	-0.016078	-0.012508	down
A_33_P3383656	DUSP28	2.1782274	2.2100253	2.1213694	0.0317979	-0.056858	-0.01253	down
A_23_P28105	TSN	2.64544	2.4884772	2.7773123	-0.156963	0.1318722	-0.012545	down
A_32_P94660	LRRC37B	0.0054202	0.057116	-0.071402	0.0516958	-0.076822	-0.012563	down
A_33_P3308764	RXRG	-3.021839	-2.827862	-3.241246	0.1939771	-0.219407	-0.012715	down
A_23_P208013	ZNF407	-1.124229	-1.055682	-1.218323	0.0685477	-0.094093	-0.012773	down
A_33_P3234197	TRIM45	1.2139525	1.1385264	1.2637777	-0.075426	0.0498252	-0.0128	down
A_23_P170498	NTAN1	2.6766157	2.5979505	2.7296095	-0.078665	0.0529938	-0.012836	down
A_24_P307626	ATP1A4	2.3335667	2.3160377	2.3251219	-0.017529	-0.008445	-0.012987	down
A_23_P47073	WDR37	2.1245565	2.3092427	1.9137526	0.1846862	-0.210804	-0.013059	down
A_33_P3331242	G3BP1	6.4951286	6.5847096	6.3793106	0.089581	-0.115818	-0.013119	down
A_33_P3329607	RAB1B	1.2367196	1.2314487	1.2156491	-0.005271	-0.02107	-0.013171	down
A_33_P3278068	ELF1	2.8230038	2.7982612	2.8213987	-0.024743	-0.001605	-0.013174	down
A_33_P3294162	PMPCB	-0.928565	-1.00939	-0.87411	-0.080825	0.0544548	-0.013185	down
A_32_P515088	KDSR	-0.393262	-0.361911	-0.451087	0.0313516	-0.057824	-0.013236	down
A_24_P721898	BTBD7	1.9706268	2.1288505	1.7857141	0.1582236	-0.184913	-0.013345	down
A_21_P0000662	RAB6C-AS1	-0.789304	-0.682391	-0.922918	0.1069126	-0.133615	-0.013351	down
A_24_P254705	ZNF695	-0.450552	-0.292679	-0.63531	0.1578736	-0.184758	-0.013442	down
A_21_P0004813	lnc-IRF4-2	-2.675241	-2.593806	-2.783714	0.081435	-0.108473	-0.013519	down
A_24_P210675	NDE1	-0.481951	-0.614975	-0.37604	-0.133025	0.1059108	-0.013557	down
A_23_P41166	B3GALNT1	0.4933972	0.4248185	0.534852	-0.068579	0.0414548	-0.013562	down

A_33_P3876985	DESI2	2.7297792	2.7385373	2.6938925	0.0087581	-0.035887	-0.013564	down
A_23_P412409	PRRC2C	2.7347975	2.7286391	2.7138014	-0.006158	-0.020996	-0.013577	down
A_23_P133956	KIFC1	1.067862	0.8445354	1.263721	-0.223327	0.195859	-0.013734	down
A_22_P00009026	lnc-LCLAT1-2	0.3870168	0.3158608	0.4306221	-0.071156	0.0436053	-0.013775	down
A_24_P223124	FNDC3B	3.7142801	3.617309	3.783677	-0.096971	0.069397	-0.013787	down
A_33_P3242124	HAUS8	0.8712583	0.9538732	0.7610359	0.0826149	-0.110222	-0.013804	down
A_33_P3405114	ENHO	-0.200088	-0.234681	-0.193174	-0.034593	0.0069132	-0.01384	down
A_23_P119943	IGFBP2	5.099492	4.927357	5.2437973	-0.172135	0.1443052	-0.013915	down
A_21_P0011033	XLOC_l2_002855	-2.737457	-2.551134	-2.951631	0.1863227	-0.214175	-0.013926	down
A_24_P219053	GPALPP1	-1.616171	-1.766974	-1.493307	-0.150802	0.1228643	-0.013969	down
A_22_P00003393	lnc-CCDC117-1	-2.054833	-2.350175	-1.787532	-0.295343	0.2673006	-0.014021	down
A_23_P78383	C18orf8	0.9346685	0.8351917	1.0060501	-0.099477	0.0713816	-0.014048	down
A_33_P3267320	RFT1	-2.580019	-2.413253	-2.77509	0.1667659	-0.195071	-0.014153	down
A_23_P55518	SMAD7	2.4055414	2.475244	2.3073301	0.0697026	-0.098211	-0.014254	down
A_32_P2634	XLOC_l2_001592	-0.014352	-0.116965	0.0595984	-0.102613	0.0739503	-0.014331	down
A_33_P3303372	PARD3	3.8949347	3.97302	3.7881393	0.0780854	-0.106795	-0.014355	down
A_23_P255104	LHFPL2	1.6904764	1.5088797	1.8433104	-0.181597	0.1528339	-0.014381	down
A_22_P00007146	LOC101927450	-2.797977	-2.953169	-2.671549	-0.155192	0.1264279	-0.014382	down
A_23_P73801	TCEAL1	1.277267	1.2549877	1.2707739	-0.022279	-0.006493	-0.014386	down
A_32_P12610	E2F6	1.1533246	1.1571941	1.1206555	0.0038695	-0.032669	-0.0144	down
A_33_P3380751	ST8SIA1	-1.48926	-1.554369	-1.452997	-0.06511	0.036263	-0.014423	down
A_22_P00016450	lnc-TMEM63A-1	2.1624565	2.0958343	2.200221	-0.066622	0.0377646	-0.014429	down
A_23_P28625	WDR12	3.8607645	3.6799893	4.0126514	-0.180775	0.1518869	-0.014444	down
A_23_P59602	MIOS	2.713519	2.7071977	2.690835	-0.006321	-0.022684	-0.014503	down
A_24_P82880	TPM4	4.1708775	4.168628	4.1440725	-0.002249	-0.026805	-0.014527	down
A_21_P0011648	XLOC_l2_006267	-1.886095	-1.702903	-2.098596	0.1831918	-0.212501	-0.014655	down
A_23_P58466	SMN1	5.1152153	4.9292254	5.271777	-0.18599	0.1565619	-0.014714	down
A_33_P3399263	IL15RA	-0.059686	-0.154641	0.0058165	-0.094954	0.0655026	-0.014726	down
A_22_P00011473	LINC00514	-2.391703	-2.333734	-2.479151	0.0579696	-0.087448	-0.014739	down
A_22_P00009020	lnc-LCE5A-1	-2.104129	-2.142417	-2.09543	-0.038288	0.0086985	-0.014795	down
A_23_P845	TOMM40L	2.3937092	2.199573	2.5580826	-0.194136	0.1643734	-0.014881	down
A_24_P156501	EBF1	-2.711019	-2.97356	-2.47836	-0.262541	0.2326598	-0.014941	down
A_23_P151209	CSRNP2	1.4762344	1.3530321	1.569417	-0.123202	0.0931826	-0.01501	down
A_33_P3540143	IL17RA	0.06743	-0.154689	0.2593622	-0.222119	0.1919322	-0.015093	down
A_24_P4705	PPME1	1.3292212	1.1791763	1.4485645	-0.150045	0.1193433	-0.015351	down
A_23_P60324	UBAC1	4.5152836	4.619221	4.380539	0.1039376	-0.134745	-0.015404	down
A_22_P00010826	lnc-NNT-1	-0.664763	-0.498933	-0.861451	0.1658301	-0.196687	-0.015429	down
A_21_P0000679	LINC01578	-0.272042	0.0332747	-0.608265	0.3053169	-0.336223	-0.015453	down
A_21_P0011474	CHEK2	1.672946	1.6032996	1.711586	-0.069646	0.03864	-0.015503	down
A_22_P00006209	TMEM44-AS1	0.4891586	0.6673989	0.2798433	0.1782403	-0.209315	-0.015538	down
A_22_P00011922	lnc-PITPNC1-1	-1.277916	-1.147985	-1.439281	0.129931	-0.161365	-0.015717	down
A_33_P3232937	GNG12-AS1	-1.972847	-2.15584	-1.821365	-0.182993	0.1514816	-0.015756	down
A_33_P3273409	SLC35E1	5.166418	5.275825	5.025465	0.109407	-0.140953	-0.015773	down
A_22_P00010277	F11-AS1	-0.487378	-0.550938	-0.455506	-0.06356	0.0318718	-0.015844	down
A_24_P100351	GNL3L	5.447276	5.354222	5.508626	-0.093054	0.0613499	-0.015852	down
A_33_P3235400	HDGF	3.870141	4.053365	3.6549616	0.1832242	-0.215179	-0.015978	down
A_23_P30275	PCYOX1L	2.3477583	2.0731645	2.590294	-0.274594	0.2425356	-0.016029	down
A_21_P0014120	LOC100507291	-0.484508	-0.360559	-0.640518	0.1239486	-0.15601	-0.016031	down
A_23_P48339	IFT88	-0.757909	-0.672225	-0.87566	0.0856843	-0.117751	-0.016033	down
A_23_P434944	ILF3	1.4215894	1.424582	1.3865085	0.0029926	-0.035081	-0.016044	down
A_23_P142950	GIGYF2	4.136816	4.081964	4.1595736	-0.054852	0.0227575	-0.016047	down
A_33_P3415042	ACLY	4.2918844	4.2190566	4.332593	-0.072828	0.0407085	-0.01606	down
A_21_P0000168	ATF7	1.3253727	1.1745872	1.4439816	-0.150785	0.118609	-0.016088	down
A_21_P0010695	LINC00339	-0.211582	-0.459116	0.0037451	-0.247534	0.2153273	-0.016104	down
A_23_P100392	TCEB2	3.810361	3.584394	4.004099	-0.225967	0.193738	-0.016114	down
A_24_P191588	ALAS1	3.1733751	3.1976418	3.116808	0.0242667	-0.056567	-0.01615	down

A_24_P385585	TMEM18	-0.93584	-0.501291	-1.40278	0.4345489	-0.466939	-0.016195	down
A_33_P3390202	SPATA25	-2.63324	-2.452311	-2.846684	0.1809294	-0.213444	-0.016257	down
A_33_P3334343	HERC2	-0.450116	-0.354406	-0.578681	0.0957093	-0.128565	-0.016428	down
A_23_P358917	CYP3A7	3.1536798	2.8382611	3.4361877	-0.315419	0.2825079	-0.016455	down
A_23_P145957	TPK1	-1.871364	-2.431269	-1.344475	-0.559906	0.5268889	-0.016508	down
A_24_P185029	SH2B1	-0.078481	-0.225466	0.0353975	-0.146985	0.1138783	-0.016553	down
A_24_P176493	ATMIN	1.0363417	0.8467035	1.1926489	-0.189638	0.1563072	-0.016665	down
A_33_P3232562	RBBP4	1.6620035	1.7834353	1.5070753	0.1214318	-0.154928	-0.016748	down
A_33_P6443530	ZNF34	-1.454355	-1.432501	-1.50974	0.0218539	-0.055386	-0.016766	down
A_23_P16976	ANXA4	4.1440086	4.1679854	4.086397	0.0239768	-0.057611	-0.016817	down
A_23_P7679	NUP155	2.4699593	2.4058537	2.500267	-0.064106	0.0303078	-0.016899	down
A_23_P300076	IQCG	-2.021137	-2.204715	-1.871384	-0.183578	0.1497531	-0.016912	down
A_24_P16378	HGSNAT	-2.869254	-2.784908	-2.987465	0.0843463	-0.118211	-0.016932	down
A_23_P131646	RPIA	5.2759104	5.2278285	5.290085	-0.048082	0.0141745	-0.016954	down
A_23_P252062	PPARG	3.6110268	3.5941834	3.5938492	-0.016843	-0.017178	-0.01701	down
A_21_P0000353	SCARNA23	1.3357162	1.3840895	1.2532697	0.0483732	-0.082447	-0.017037	down
A_21_P0014503	INTS6-AS1	0.2994838	0.2401362	0.3246388	-0.059348	0.0251551	-0.017096	down
A_33_P3279109	ZNF75A	0.49193	0.4464188	0.503222	-0.045511	0.011292	-0.01711	down
A_32_P147078	SLC8A1	-2.901093	-2.681352	-3.155059	0.2197406	-0.253967	-0.017113	down
A_33_P3389658	PDE4D	-1.093072	-1.265981	-0.954429	-0.17291	0.1386428	-0.017133	down
A_19_P00802713	LOC101929580	-2.513988	-2.532466	-2.529867	-0.018479	-0.015879	-0.017179	down
A_24_P415327	HPS1	1.2361851	1.0968022	1.3409681	-0.139383	0.1047831	-0.0173	down
A_23_P141484	FAM222B	4.110756	3.9194999	4.2673674	-0.191256	0.1566114	-0.017322	down
A_33_P3243093	RGS5	-0.970275	-0.962455	-1.012842	0.0078197	-0.042567	-0.017374	down
A_33_P3268313	PGAM2	-2.452632	-2.891944	-2.048255	-0.439312	0.4043765	-0.017468	down
A_33_P3371727	SAT1	-2.027366	-1.787644	-2.302234	0.2397218	-0.274868	-0.017573	down
A_24_P68311	SYNE2	0.3936682	0.4975138	0.2546582	0.1038456	-0.13901	-0.017582	down
A_21_P0012850	LOC101926941	-2.551948	-2.826461	-2.31263	-0.274513	0.2393177	-0.017598	down
A_24_P184445	MMP19	-2.053224	-2.048916	-2.092798	0.0043082	-0.039574	-0.017633	down
A_24_P269432	BET1	0.6196151	0.5396681	0.6642461	-0.079947	0.044631	-0.017658	down
A_23_P21485	PID1	-1.983327	-2.330948	-1.671206	-0.347621	0.3121219	-0.017749	down
A_23_P255076	RWDD2A	-0.137415	-0.078127	-0.232234	0.059288	-0.094819	-0.017765	down
A_23_P305205	KIAA0895	-3.163543	-3.016184	-3.346433	0.1473594	-0.18289	-0.017765	down
A_22_P00023080	LRIG2	1.117239	1.198369	1.0005779	0.08113	-0.116661	-0.017766	down
A_23_P64232	ZPR1	3.9742126	4.023351	3.8892994	0.0491385	-0.084913	-0.017887	down
A_23_P207336	PPY	-2.453633	-2.535134	-2.407918	-0.081501	0.0457153	-0.017893	down
A_23_P93082	NUS1	2.2748117	2.1537433	2.360056	-0.121068	0.0852442	-0.017912	down
A_33_P3329784	ANKLE2	1.3641539	1.369679	1.3227835	0.0055251	-0.04137	-0.017923	down
A_24_P372012	ICA1	1.6761942	1.7073174	1.6090832	0.0311232	-0.067111	-0.017994	down
A_22_P00021593	lnc-C12orf66-3	-2.727434	-2.788307	-2.702653	-0.060873	0.024781	-0.018046	down
A_23_P75453	MEN1	1.2040648	1.1372366	1.234745	-0.066828	0.0306802	-0.018074	down
A_23_P131723	YWHAQ	7.1717215	7.149922	7.1572514	-0.0218	-0.01447	-0.018135	down
A_22_P00011852	lnc-PHTF2-1	-1.856654	-2.015349	-1.734325	-0.158696	0.1223288	-0.018183	down
A_22_P00002362	lnc-C12orf45-1	-3.006348	-2.997422	-3.051825	0.0089269	-0.045476	-0.018275	down
A_23_P2143	SPCS2	5.2926903	5.3089623	5.23983	0.0162721	-0.05286	-0.018294	down
A_21_P0013894	XLOC_l2_015938	2.6527252	2.704041	2.5646744	0.0513158	-0.088051	-0.018368	down
A_23_P259521	WDR41	3.3383942	3.312447	3.3274727	-0.025947	-0.010921	-0.018434	down
A_32_P151875	ZNF398	2.727975	2.723207	2.6957703	-0.004768	-0.032205	-0.018486	down
A_23_P395404	FGF20	3.277254	2.9210086	3.5963888	-0.356246	0.3191347	-0.018555	down
A_24_P142473	CTDNEP1	1.3080091	1.2197576	1.3589334	-0.088252	0.0509243	-0.018664	down
A_23_P386764	DOLPP1	2.8135662	2.5991273	2.9905043	-0.214439	0.1769381	-0.01875	down
A_23_P204751	ASIC1	2.774334	2.581513	2.9294863	-0.192821	0.1551523	-0.018834	down
A_21_P0013993	FAM104B	-0.160168	-0.076389	-0.281633	0.0837789	-0.121465	-0.018843	down
A_33_P3403392	AACS	-0.285296	-0.240208	-0.36807	0.0450878	-0.082774	-0.018843	down
A_22_P00015030	HOXC-AS2	-2.064856	-1.958571	-2.208846	0.1062846	-0.14399	-0.018853	down
A_23_P257945	HCCS	2.9699707	2.8146005	3.0875044	-0.15537	0.1175337	-0.018918	down

A_33_P3237574	HLA-A	7.492134	7.560882	7.3855066	0.068748	-0.106627	-0.01894	down
A_21_P0011956	XLOC_l2_008221	-0.920245	-0.862805	-1.015617	0.0574398	-0.095372	-0.018966	down
A_33_P3391455	SCN5A	-2.982826	-2.818729	-3.1849	0.1640968	-0.202074	-0.018988	down
A_24_P148450	UBE2E3	2.6678514	2.6650872	2.632537	-0.002764	-0.035315	-0.019039	down
A_24_P337397	ANKRA2	-0.593212	-0.44403	-0.780564	0.1491818	-0.187352	-0.019085	down
A_23_P502747	RASAL2	-0.541558	-0.516863	-0.604467	0.0246954	-0.062909	-0.019107	down
A_32_P155506	UBE2E2	1.054965	1.0172896	1.0543666	-0.037675	-0.000598	-0.019137	down
A_21_P0013339	XLOC_l2_013931	-0.007633	0.0962768	-0.149927	0.10391	-0.142294	-0.019192	down
A_21_P0000290	SNORD44	-0.592783	-0.585101	-0.638912	0.0076814	-0.046129	-0.019224	down
A_23_P133424	SKP1	7.071479	6.986218	7.118271	-0.085261	0.046792	-0.019234	down
A_24_P333326	CTAGE5	0.1225424	0.2075672	-0.000963	0.0850248	-0.123505	-0.01924	down
A_23_P132378	CELSR1	1.5325723	1.4836888	1.5429225	-0.048883	0.0103502	-0.019267	down
A_33_P3364696	STAMBP	1.7003336	1.6556125	1.7064552	-0.044721	0.0061216	-0.0193	down
A_32_P144596	TNKS	-0.830012	-0.858429	-0.840218	-0.028417	-0.010206	-0.019311	down
A_24_P411863	KMT2E	-1.304789	-1.281056	-1.367225	0.0237331	-0.062436	-0.019351	down
A_22_P00015927	lnc-TCL1B-2	-0.759692	-0.613122	-0.944966	0.1465693	-0.185275	-0.019353	down
A_33_P8915099	LOC101928336	-2.115449	-2.13129	-2.138317	-0.015842	-0.022868	-0.019355	down
A_33_P3238280	ESYT3	-2.055541	-2.152692	-1.99713	-0.097151	0.0584102	-0.01937	down
A_21_P0005007	lnc-DEK-1	-2.571879	-2.684485	-2.498045	-0.112607	0.0738335	-0.019387	down
A_23_P417148	RAI1	2.52629	2.4235902	2.590211	-0.1027	0.063921	-0.019389	down
A_24_P335358	PUS1	3.0216599	3.000606	3.0038948	-0.021054	-0.017765	-0.019409	down
A_21_P0013580	LOC102725126	-2.763753	-2.868095	-2.698245	-0.104342	0.0655088	-0.019416	down
A_19_P00804093	CCDC149	-2.593961	-2.488058	-2.739035	0.1059029	-0.145075	-0.019586	down
A_33_P3321205	BEGAIN	-0.646298	-0.84472	-0.487086	-0.198422	0.1592116	-0.019605	down
A_33_P3398331	MMP24	-0.249738	0.0725637	-0.611334	0.3223019	-0.361596	-0.019647	down
A_23_P29046	CBR1	3.2081041	3.1813588	3.1953344	-0.026745	-0.01277	-0.019758	down
A_33_P3317253	PTER	1.3476386	1.3916993	1.2639351	0.0440607	-0.083704	-0.019821	down
A_23_P398294	HIP1R	4.6445913	4.6028094	4.6467075	-0.041782	0.0021162	-0.019833	down
A_23_P391926	LPHN1	1.2334747	1.1079531	1.3193088	-0.125522	0.085834	-0.019844	down
A_24_P129417	BMP1	-1.010827	-1.113344	-0.948049	-0.102517	0.062778	-0.019869	down
A_33_P3267431	LOC642648	-2.287995	-2.250013	-2.36579	0.037982	-0.077795	-0.019907	down
A_33_P3284518	UHRF1BP1L	-0.288655	-0.23906	-0.378252	0.0495949	-0.089597	-0.020001	down
A_24_P234116	C1orf56	4.234024	4.032539	4.395483	-0.201485	0.161459	-0.020013	down
A_23_P313389	UGCG	1.5217052	1.4663734	1.5369773	-0.055332	0.0152721	-0.02003	down
A_23_P80839	MAP6D1	3.9518414	3.6235614	4.240034	-0.32828	0.2881928	-0.020044	down
A_33_P3287825	CCDC136	-1.082124	-1.217754	-0.986677	-0.13563	0.0954471	-0.020092	down
A_33_P3242743	ARFRP1	-0.046832	-0.341988	0.2080641	-0.295156	0.2548957	-0.02013	down
A_24_P323084	LINC00482	0.3340159	0.361331	0.2663188	0.0273151	-0.067697	-0.020191	down
A_24_P14464	WFDC2	-2.87029	-2.979433	-2.801582	-0.109143	0.0687084	-0.020217	down
A_22_P00015751	lnc-TAF1C-1	-1.045476	-1.169855	-0.961647	-0.124379	0.0838294	-0.020275	down
A_22_P00016354	LOC728730	2.0740662	2.0990949	2.0084648	0.0250287	-0.065601	-0.020286	down
A_24_P243749	PDK4	-1.976959	-2.06153	-1.933012	-0.084571	0.0439467	-0.020312	down
A_33_P3303385	NCAPD2	5.3774023	5.3211646	5.392935	-0.056238	0.0155325	-0.020353	down
A_23_P97423	UBE2Q1	1.3802233	1.2492504	1.4704809	-0.130973	0.0902576	-0.020358	down
A_23_P131990	VSX1	-1.958201	-1.816762	-2.140471	0.141439	-0.18227	-0.020415	down
A_21_P0011293	XLOC_l2_004594	-1.374891	-1.224659	-1.565959	0.1502318	-0.191068	-0.020418	down
A_32_P181077	DOCK8	0.9534822	0.9745169	0.8915644	0.0210347	-0.061918	-0.020442	down
A_23_P19164	TTC1	3.6309872	3.5731864	3.647871	-0.057801	0.0168839	-0.020458	down
A_33_P3278362	ANKRD2	-0.954407	-1.195189	-0.754548	-0.240782	0.1998591	-0.020461	down
A_23_P4041	PCTP	3.7522202	3.7347598	3.7286835	-0.01746	-0.023537	-0.020499	down
A_33_P3415191	ATP8B1	3.2068424	3.1760898	3.196494	-0.030753	-0.010348	-0.02055	down
A_33_P3365676	PTPLAD1	6.1595182	5.968393	6.30947	-0.191125	0.1499519	-0.020587	down
A_24_P818010	LOC729737	2.9421625	3.0658135	2.7771902	0.123651	-0.164972	-0.020661	down
A_23_P31109	GINM1	3.4615192	3.6791282	3.2025528	0.2176089	-0.258966	-0.020679	down
A_19_P00320718	PCAT19	-2.181953	-2.354574	-2.050695	-0.17262	0.131259	-0.020681	down
A_23_P330788	IQSEC2	1.8590508	1.6628995	2.013506	-0.196151	0.1544552	-0.020848	down

A_24_P234856	CAMLG	-1.831115	-1.585214	-2.118713	0.2459016	-0.287598	-0.020848	down
A_21_P0013228	INTS4	-1.012101	-1.001269	-1.064933	0.0108314	-0.052832	-0.021	down
A_21_P0009816	lnc-FOXA2-8	-3.143991	-3.081923	-3.248109	0.0620685	-0.104118	-0.021025	down
A_23_P20777	RBM18	3.9812727	3.8278737	4.0926027	-0.153399	0.11133	-0.021034	down
A_22_P00001182	LOC100287015	0.4081321	0.1901193	0.5840573	-0.218013	0.1759253	-0.021044	down
A_23_P409168	NBEAL2	1.5756254	1.4625611	1.6465578	-0.113064	0.0709324	-0.021066	down
A_23_P64567	PPME1	-0.040056	-0.285457	0.1630397	-0.245401	0.2030954	-0.021153	down
A_22_P00025715	lnc-PDZK1IP1-1	-0.340674	-0.386373	-0.337294	-0.045698	0.0033808	-0.021159	down
A_23_P117146	PRDM4	0.6134687	0.6833572	0.5011544	0.0698886	-0.112314	-0.021213	down
A_24_P418408	FAM89A	3.2679281	3.0972257	3.3959513	-0.170702	0.1280232	-0.02134	down
A_23_P124570	FER	-0.3037	-0.383137	-0.266948	-0.079437	0.0367522	-0.021343	down
A_23_P78762	HSD17B14	-2.455553	-2.591736	-2.36206	-0.136183	0.093493	-0.021345	down
A_23_P121527	KLHL5	-0.050637	0.1963873	-0.34041	0.2470245	-0.289773	-0.021374	down
A_33_P3281741	RFX8	-3.074228	-2.776829	-3.414466	0.2973988	-0.340239	-0.02142	down
A_23_P209944	RETSAT	-0.106411	-0.059319	-0.196466	0.047092	-0.090055	-0.021482	down
A_23_P11353	ATP6AP2	4.496708	4.5925508	4.3578205	0.0958428	-0.138887	-0.021522	down
A_23_P422794	NSMCE2	3.5088797	3.4360852	3.5385208	-0.072794	0.0296412	-0.021577	down
A_23_P134167	PDSS2	2.2178774	2.2327142	2.15977	0.0148368	-0.058107	-0.021635	down
A_23_P114282	MCTS1	6.0554457	5.988215	6.0793495	-0.067231	0.0239038	-0.021663	down
A_23_P48121	ADIPOR2	4.158345	3.870646	4.402663	-0.287699	0.244318	-0.021691	down
A_33_P3424297	SELT	3.2702923	3.3591723	3.13801	0.0888801	-0.132282	-0.021701	down
A_22_P00013159	RNF112	-3.04044	-2.803882	-3.320418	0.2365584	-0.279978	-0.02171	down
A_19_P00807748	LOC102725166	-1.371266	-1.380221	-1.405735	-0.008955	-0.034469	-0.021712	down
A_24_P329635	TSC1	2.798336	2.8218846	2.7312784	0.0235486	-0.067058	-0.021755	down
A_33_P3789894	SNORA75	-0.768616	-0.570705	-1.010122	0.1979108	-0.241506	-0.021798	down
A_33_P3289286	TMEM57	-2.436325	-2.646134	-2.270113	-0.209809	0.1662126	-0.021798	down
A_22_P00019752	lnc-ANKRD34B-2	0.132123	0.2219424	-0.001468	0.0898194	-0.133591	-0.021886	down
A_23_P372096	NOL4L	1.6259251	1.7156949	1.4923596	0.0897698	-0.133565	-0.021898	down
A_33_P3846177	B4GALNT1	1.1497464	0.8890066	1.3665586	-0.26074	0.2168121	-0.021964	down
A_33_P3276475	CHMP1B	2.7189798	2.5692353	2.8247614	-0.149745	0.1057816	-0.021981	down
A_24_P204675	NPIPB5	5.6097975	5.7821403	5.393483	0.1723428	-0.216314	-0.021986	down
A_23_P13797	C12orf49	1.273643	1.2870736	1.2162066	0.0134306	-0.057436	-0.022003	down
A_23_P202269	ANK3	2.28261	2.3576694	2.163412	0.0750594	-0.119198	-0.022069	down
A_32_P850562	ZNF337-AS1	-1.718885	-2.162338	-1.319573	-0.443454	0.3993111	-0.022071	down
A_22_P00014280	lnc-SEPT11-1	-3.245163	-2.973975	-3.560499	0.2711883	-0.315336	-0.022074	down
A_33_P3248953	C5orf63	-1.385047	-1.187899	-1.626357	0.1971483	-0.241309	-0.02208	down
A_22_P00012658	LINCR-0002	-2.234699	-2.26552	-2.248065	-0.030821	-0.013366	-0.022094	down
A_23_P418015	MAPRE2	1.3534412	1.2731562	1.389534	-0.080285	0.0360928	-0.022096	down
A_24_P384755	NOL10	-0.07355	-0.285584	0.0941234	-0.212034	0.1676736	-0.02218	down
A_33_P3272828	RAD1	0.2246723	0.260139	0.1448197	0.0354667	-0.079853	-0.022193	down
A_24_P36097	RPAIN	1.8486462	1.9686027	1.684269	0.1199565	-0.164377	-0.02221	down
A_24_P849801	RPL22	2.6184807	2.90237	2.2898026	0.2838893	-0.328678	-0.022394	down
A_23_P93269	ZNF165	2.8058586	2.9271855	2.6396055	0.1213269	-0.166253	-0.022463	down
A_22_P00000181	lnc-ABHD12B-3	-2.509567	-2.326587	-2.737638	0.1829808	-0.22807	-0.022545	down
A_24_P2948	RUSC1	2.1083307	1.743217	2.428235	-0.365114	0.3199043	-0.022605	down
A_21_P0014669	ZNF518A	-2.429766	-2.899408	-2.005338	-0.469641	0.424428	-0.022607	down
A_33_P3404623	MKS1	-1.819072	-1.711243	-1.972364	0.1078291	-0.153292	-0.022731	down
A_23_P161352	PTPLA	2.3632555	2.3400226	2.340889	-0.023233	-0.022367	-0.0228	down
A_23_P303260	STX7	0.6409688	0.6853309	0.5508027	0.0443621	-0.090166	-0.022902	down
A_24_P239017	UBE2MP1	3.1610537	3.0051455	3.2711334	-0.155908	0.1100798	-0.022914	down
A_24_P233917	EFCAB14	3.8980303	3.87501	3.8751917	-0.02302	-0.022839	-0.022929	down
A_19_P00321110	TIAM2	-0.195857	-0.186698	-0.250898	0.0091586	-0.055042	-0.022942	down
A_23_P500956	B3GNT2	0.6725493	0.602006	0.6971326	-0.070543	0.0245833	-0.02298	down
A_33_P3302957	PLEKHG4	1.4446692	1.2404256	1.6029382	-0.204244	0.1582689	-0.022987	down
A_33_P3350726	PPARG	2.08354	2.066032	2.0550585	-0.017508	-0.028481	-0.022995	down
A_33_P7699408	LOC101928973	0.5030603	0.5138421	0.4462881	0.0107818	-0.056772	-0.022995	down

A_23_P218706	ZNF343	-0.211948	-0.210012	-0.259923	0.001936	-0.047976	-0.02302	down
A_33_P3334313	ACTR3B	-0.291785	-0.380661	-0.248961	-0.088876	0.0428247	-0.023026	down
A_23_P394259	MPP2	-2.143283	-2.299768	-2.032875	-0.156485	0.1104088	-0.023038	down
A_33_P3313779	CCDC64	-2.882937	-2.702806	-3.109269	0.1801314	-0.226331	-0.0231	down
A_23_P143817	MYLK	-2.101487	-1.887184	-2.362079	0.2143025	-0.260592	-0.023145	down
A_33_P3260623	PLCB3	-0.643879	-0.765123	-0.568948	-0.121244	0.0749307	-0.023157	down
A_22_P00009106	FAM155A-IT1	0.6809597	0.8214173	0.4940653	0.1404576	-0.186894	-0.023218	down
A_23_P86216	PSMA5	6.490181	6.464004	6.469904	-0.026177	-0.020277	-0.023227	down
A_32_P36046	C2orf68	0.8964615	0.8997273	0.846509	0.0032659	-0.049953	-0.023343	down
A_23_P15073	JMJD8	1.840725	1.7974577	1.8372927	-0.043267	-0.003432	-0.02335	down
A_23_P154507	ITGB1BP1	5.2865343	5.223331	5.3029385	-0.063203	0.0164042	-0.0234	down
A_24_P28295	RABGAP1L	1.914259	2.0170846	1.7645979	0.1028256	-0.149661	-0.023418	down
A_33_P3362521	CCDC174	2.6000853	2.7575946	2.3957272	0.1575093	-0.204358	-0.023424	down
A_33_P3421827	HBZ	-0.023266	-0.066794	-0.026624	-0.043529	-0.003358	-0.023443	down
A_24_P722216	DHTKD1	0.351615	0.4115768	0.2447357	0.0599618	-0.106879	-0.023459	down
A_33_P3298062	ABCC5	2.7630396	2.7741504	2.7049541	0.0111108	-0.058085	-0.023487	down
A_23_P13998	GOLGA2P5	0.7787905	0.827374	0.6831799	0.0485835	-0.095611	-0.023514	down
A_23_P201279	UBE4B	2.6063604	2.554195	2.6114006	-0.052166	0.0050402	-0.023563	down
A_33_P3555009	ZNF528-AS1	3.1228523	2.9253297	3.2730408	-0.197523	0.1501885	-0.023667	down
A_23_P201808	PPAP2B	-0.176558	-0.049117	-0.351394	0.1274409	-0.174837	-0.023698	down
A_23_P89030	C16orf95	1.8606358	1.6494122	2.024372	-0.211224	0.1637363	-0.023744	down
A_24_P133162	STPG1	-0.636735	-0.40975	-0.911306	0.2269859	-0.274571	-0.023793	down
A_21_P0006968	SFTA1P	-0.378024	-0.240345	-0.563335	0.1376791	-0.185311	-0.023816	down
A_22_P00024043	DGAT1	0.3298297	0.0845156	0.5274916	-0.245314	0.1976619	-0.023826	down
A_22_P00015003	lnc-SMNDC1-3	-2.085805	-2.129562	-2.089943	-0.043757	-0.004138	-0.023947	down
A_23_P36939	NUFIP1	-1.308584	-1.10898	-1.556091	0.1996045	-0.247507	-0.023951	down
A_21_P0008530	lnc-NOVA1-1	-1.118392	-1.038053	-1.246675	0.0803385	-0.128284	-0.023973	down
A_24_P20032	RFX5	2.5947523	2.4496207	2.6918497	-0.145132	0.0970974	-0.024017	down
A_24_P194508	SPIN2B	1.5659866	1.6132584	1.4705014	0.0472717	-0.095485	-0.024107	down
A_23_P70583	VPS52	0.0428538	0.0396643	-0.002291	-0.00319	-0.045145	-0.024167	down
A_23_P425104	BRD4	0.7428584	0.7638745	0.6734924	0.0210161	-0.069366	-0.024175	down
A_23_P31747	C8orf76	3.8608427	3.7351804	3.9381514	-0.125662	0.0773087	-0.024177	down
A_24_P350245	DOCK5	0.300807	0.1552186	0.398026	-0.145588	0.097219	-0.024185	down
A_21_P0014493	TRIM52-AS1	-0.303387	-0.509734	-0.145584	-0.206347	0.1578035	-0.024271	down
A_23_P211167	C21orf2	-2.36011	-2.414167	-2.354698	-0.054057	0.0054128	-0.024322	down
A_33_P3214720	ZC3H12A	1.8629122	1.8150525	1.8617849	-0.04786	-0.001127	-0.024493	down
A_22_P00024157	LOC102724352	-0.942387	-1.011841	-0.92199	-0.069454	0.0203967	-0.024529	down
A_23_P132444	TCEA1	3.5010042	3.535831	3.4169998	0.0348268	-0.084004	-0.024589	down
A_23_P12477	SCMH1	-1.07519	-1.133978	-1.065685	-0.058788	0.0095048	-0.024642	down
A_33_P3369790	GGN	-2.145083	-1.998403	-2.341176	0.1466804	-0.196092	-0.024706	down
A_23_P8522	TMEM106B	2.3123732	2.374227	2.201007	0.0618539	-0.111366	-0.024756	down
A_33_P3386671	RORC	-2.163644	-2.206094	-2.170779	-0.04245	-0.007135	-0.024793	down
A_23_P13663	FAM60A	5.4167824	5.2047725	5.578965	-0.21201	0.1621828	-0.024914	down
A_33_P3311076	CYB5A	3.6148548	3.4204698	3.7593699	-0.194385	0.144515	-0.024935	down
A_24_P298174	CBX1	3.3190746	3.397039	3.191226	0.0779643	-0.127849	-0.024942	down
A_33_P3287959	RASA4	-0.004778	-0.007635	-0.051847	-0.002857	-0.047069	-0.024963	down
A_22_P00023425	lnc-SCN8A-2	-0.507632	-0.634931	-0.430265	-0.127299	0.0773668	-0.024966	down
A_33_P3293009	NKAIN4	-1.83427	-1.94616	-1.77286	-0.111891	0.06141	-0.02524	down
A_24_P30923	SNN	2.3730726	2.2581754	2.4374533	-0.114897	0.0643806	-0.025258	down
A_33_P3388016	TMA7	5.31044	5.0538373	5.516449	-0.256603	0.2060089	-0.025297	down
A_24_P55225	RSPH9	-3.141005	-3.093508	-3.239271	0.0474973	-0.098266	-0.025384	down
A_21_P0013517	XLOC_l2_014549	4.293023	4.183433	4.351783	-0.10959	0.0587597	-0.025415	down
A_33_P3281532	RMND5A	0.2032633	0.2418475	0.1137276	0.0385842	-0.089536	-0.025476	down
A_23_P377882	KCNH2	-1.667648	-1.648767	-1.737885	0.0188818	-0.070236	-0.025677	down
A_23_P63980	LRFN4	4.237053	3.8744626	4.5482855	-0.36259	0.3112326	-0.025679	down
A_23_P348183	C6orf223	-2.34922	-2.306652	-2.443171	0.0425682	-0.093951	-0.025691	down

A_33_P3221119	GON4L	2.263795	2.238027	2.238078	-0.025768	-0.025717	-0.025742	down
A_23_P38618	PIGL	0.8709574	0.7487969	0.9415269	-0.12216	0.0705695	-0.025795	down
A_23_P143845	TIPARP	3.7429466	3.816626	3.6175737	0.0736795	-0.125373	-0.025847	down
A_32_P7015	TSPAN15	1.6611605	1.7372594	1.5333467	0.0760989	-0.127814	-0.025857	down
A_33_P3262541	ADAMTSL2	-0.948324	-0.88779	-1.060675	0.060534	-0.112351	-0.025908	down
A_19_P00801917	lnc-FARS2-2	2.7869253	2.6831498	2.8388538	-0.103776	0.0519285	-0.025923	down
A_32_P118586	DENND6A	2.907135	2.7873273	2.974761	-0.119808	0.067626	-0.026091	down
A_33_P3290829	LOC101059906	-3.248119	-3.300491	-3.248001	-0.052372	0.000118	-0.026127	down
A_24_P316430	NT5E	-0.072828	-0.043443	-0.154472	0.0293851	-0.081644	-0.026129	down
A_23_P388900	SLC22A15	0.217423	0.4374766	-0.054899	0.2200537	-0.272322	-0.026134	down
A_23_P77228	CRTC3	0.5806871	0.6274037	0.4815798	0.0467167	-0.099107	-0.026195	down
A_22_P00020037	lnc-ZPBP-1	-2.94324	-2.890869	-3.048052	0.0523706	-0.104813	-0.026221	down
A_24_P83738	ASTN2	2.0275898	1.8829803	2.1196795	-0.144609	0.0920897	-0.02626	down
A_23_P413585	FOXD2	-2.557078	-2.429853	-2.736853	0.1272247	-0.179775	-0.026275	down
A_33_P3217123	BCL2L12	7.1759186	7.2409253	7.0582876	0.0650067	-0.117631	-0.026312	down
A_33_P3245947	LRRC45	-2.14795	-2.28007	-2.068566	-0.13212	0.0793839	-0.026368	down
A_23_P70670	CD83	1.6659803	1.7230501	1.556149	0.0570698	-0.109831	-0.026381	down
A_33_P3333050	SLC9A4	0.883739	0.838624	0.8760634	-0.045115	-0.007676	-0.026395	down
A_24_P317719	WHSC1L1	0.085084	-0.045683	0.1628161	-0.130767	0.0777321	-0.026518	down
A_23_P358542	KIFC2	0.0754857	0.1274662	-0.029684	0.0519805	-0.10517	-0.026595	down
A_24_P357037	UBE2G2	3.598753	3.4050374	3.7391415	-0.193716	0.1403885	-0.026664	down
A_23_P101992	MARCO	-1.99018	-1.803352	-2.230443	0.1868272	-0.240263	-0.026718	down
A_23_P374767	UBE2D4	1.5597343	1.5896392	1.4763317	0.0299048	-0.083403	-0.026749	down
A_24_P131222	ATP13A2	1.6520185	1.4183803	1.8321257	-0.233638	0.1801071	-0.026766	down
A_24_P225679	IRS1	2.1647978	1.935617	2.3403788	-0.229181	0.175581	-0.0268	down
A_22_P00008052	LOC645553	0.2602034	0.42558	0.0411797	0.1653767	-0.219024	-0.026824	down
A_21_P0014015	CNP	-1.370148	-1.302694	-1.491389	0.0674539	-0.121241	-0.026893	down
A_23_P153676	TLE2	0.9652004	0.9640865	0.9125266	-0.001114	-0.052674	-0.026894	down
A_33_P3279441	GOSR2	2.6606693	2.6530633	2.6143665	-0.007606	-0.046303	-0.026954	down
A_33_P3313555	PSPC1	3.0120277	3.0603461	2.9097643	0.0483184	-0.102263	-0.026973	down
A_24_P235316	VDAC3	4.206936	4.152411	4.2074556	-0.054525	0.0005198	-0.027003	down
A_33_P3270519	STRIP1	-1.420393	-1.406315	-1.48854	0.0140777	-0.068147	-0.027035	down
A_33_P3367575	EIF4ENIF1	-1.047417	-0.91942	-1.229685	0.1279974	-0.182268	-0.027135	down
A_33_P3254555	MCM9	-1.189902	-1.241269	-1.192839	-0.051367	-0.002937	-0.027152	down
A_33_P3272209	MFSD6	3.25461	3.1884665	3.2663603	-0.066144	0.0117502	-0.027197	down
A_23_P253586	DOPEY2	-2.764308	-2.732801	-2.850416	0.0315073	-0.086107	-0.0273	down
A_33_P3259722	TMEM222	0.45785	0.5176306	0.3433032	0.0597806	-0.114547	-0.027383	down
A_23_P164638	ZNF419	-1.371	-1.274543	-1.522363	0.0964565	-0.151363	-0.027453	down
A_33_P3349774	LSM14B	-2.639958	-2.441475	-2.893392	0.1984828	-0.253434	-0.027476	down
A_33_P3362869	DNAJC19	4.6378975	4.692452	4.5283213	0.0545545	-0.109576	-0.027511	down
A_33_P3345608	EDRF1	-0.594786	-0.808795	-0.43581	-0.214009	0.1589761	-0.027516	down
A_22_P00007597	CRTC3-AS1	-2.992671	-3.023032	-3.01737	-0.030361	-0.024699	-0.02753	down
A_23_P365189	CAMTA2	-1.781472	-1.918252	-1.699831	-0.13678	0.0816417	-0.027569	down
A_23_P60591	DNAJC7	3.1075478	3.0952792	3.0646257	-0.012269	-0.042922	-0.027595	down
A_23_P411162	PER2	-0.597524	-0.73352	-0.516813	-0.135996	0.0807109	-0.027643	down
A_24_P186065	DHFRL1	-0.010489	-0.188107	0.1118269	-0.177619	0.1223154	-0.027652	down
A_33_P3399768	LINC01160	-1.791915	-1.819666	-1.81948	-0.027751	-0.027565	-0.027658	down
A_33_P3269578	PBRM1	-1.084814	-1.058889	-1.166091	0.0259242	-0.081277	-0.027676	down
A_33_P3373745	BRD4	-0.430094	-0.377522	-0.538179	0.0525727	-0.108085	-0.027756	down
A_21_P0006147	LOC101927042	-2.26476	-2.745081	-1.840011	-0.480321	0.4247494	-0.027786	down
A_23_P3552	LOC102723428	-0.877204	-0.807389	-1.002733	0.0698152	-0.125529	-0.027857	down
A_23_P151471	CUL4A	4.624771	4.5780683	4.615572	-0.046703	-0.009199	-0.027951	down
A_33_P3416473	YAE1D1	-0.089857	-0.152122	-0.083726	-0.062264	0.0061307	-0.028067	down
A_22_P00018673	lnc-WDR5-2	-0.685615	-0.746393	-0.68114	-0.060779	0.0044742	-0.028152	down
A_22_P00021963	lnc-NLRP12-2	-1.78077	-1.888674	-1.729582	-0.107904	0.0511875	-0.028358	down
A_33_P3593546	MARK3	3.9807568	4.0424914	3.8622465	0.0617347	-0.11851	-0.028388	down

A_21_P0001096	lnc-DNAJC11-1	-1.150952	-1.094401	-1.264308	0.056551	-0.113356	-0.028403	down
A_21_P0012116	ZNF850	-0.566272	-0.63496	-0.55442	-0.068688	0.0118518	-0.028418	down
A_23_P30223	SRD5A1	-0.906929	-1.165748	-0.70497	-0.258819	0.2019587	-0.02843	down
A_21_P0014372	ZNF649-AS1	-3.179615	-3.063738	-3.352375	0.1158774	-0.17276	-0.028441	down
A_23_P35883	FOXRED1	0.9052072	0.9193187	0.8341527	0.0141115	-0.071054	-0.028471	down
A_24_P392060	SIMC1	2.8122454	2.5535626	3.0139074	-0.258683	0.2016621	-0.02851	down
A_23_P208310	CD3EAP	4.1582823	3.9918733	4.2676554	-0.166409	0.1093731	-0.028518	down
A_19_P00320218	LINC01237	-1.484208	-1.486892	-1.538664	-0.002685	-0.054457	-0.028571	down
A_33_P3271430	MAP3K3	2.8466034	2.935186	2.7008524	0.0885825	-0.145751	-0.028584	down
A_21_P0012415	XLOC_l2_010013	-2.334766	-2.530777	-2.195941	-0.196011	0.1388249	-0.028593	down
A_23_P328034	C20orf96	0.0936999	0.2134147	-0.083241	0.1197147	-0.176941	-0.028613	down
A_23_P59630	PMS2P3	1.7055454	1.6279068	1.7257562	-0.077639	0.0202107	-0.028714	down
A_24_P823684	HSP90AB1	5.4983015	5.5725045	5.3666573	0.074203	-0.131644	-0.028721	down
A_22_P00004627	AATBC	-1.144596	-1.10451	-1.242151	0.0400858	-0.097555	-0.028734	down
A_23_P219060	GPSM3	-0.525097	-0.544004	-0.563669	-0.018907	-0.038572	-0.028739	down
A_33_P3334108	ZNF740	0.2524209	0.3570547	0.0902352	0.1046338	-0.162186	-0.028776	down
A_23_P15944	ELAC1	0.3179798	0.3779717	0.2004275	0.0599918	-0.117552	-0.02878	down
A_23_P41987	GFRA3	-2.042869	-2.193505	-1.949901	-0.150635	0.092968	-0.028834	down
A_24_P936122	STX16	3.232297	3.339294	3.0675735	0.106997	-0.164723	-0.028863	down
A_23_P214727	GPR63	-2.088471	-2.24075	-1.993925	-0.152279	0.0945463	-0.028866	down
A_23_P22096	PTK2	1.80931	1.8003874	1.7604895	-0.008923	-0.04882	-0.028872	down
A_23_P146728	ALG2	2.956108	2.8657937	2.9884663	-0.090314	0.0323582	-0.028978	down
A_23_P110571	MAST4	1.3475623	1.416091	1.2208157	0.0685287	-0.126747	-0.029109	down
A_32_P755542	TAMM41	3.852252	3.5228333	4.1233587	-0.329419	0.2711067	-0.029156	down
A_23_P117494	MTHFD1	5.870779	5.861672	5.8213577	-0.009107	-0.049421	-0.029264	down
A_23_P381577	ZNF25	-2.274592	-2.377062	-2.230778	-0.10247	0.0438137	-0.029328	down
A_23_P87500	ORMDL2	6.295619	6.1982102	6.334345	-0.097409	0.0387259	-0.029341	down
A_21_P0014061	LINC00083	-0.58085	-0.673558	-0.546869	-0.092708	0.0339804	-0.029364	down
A_33_P3418091	ISLR2	-1.620497	-1.534092	-1.765898	0.0864058	-0.145401	-0.029497	down
A_33_P3251198	PLCB1	-0.594535	-0.512407	-0.735671	0.0821281	-0.141135	-0.029504	down
A_21_P0007070	lnc-RPP30-2	-0.562352	-0.397345	-0.786569	0.1650071	-0.224217	-0.029605	down
A_23_P380208	VEPH1	-0.987554	-1.207368	-0.826973	-0.219814	0.1605802	-0.029617	down
A_23_P141738	SS18	0.3081293	0.2130561	0.3438811	-0.095073	0.0357518	-0.029661	down
A_33_P3312039	RAD23B	2.2676105	2.40105	2.0747156	0.1334395	-0.192895	-0.029728	down
A_32_P201496	POLR3H	2.0831928	1.9798446	2.1270685	-0.103348	0.0438757	-0.029736	down
A_23_P73780	IRAK1	3.2703762	3.2460093	3.2350626	-0.024367	-0.035314	-0.02984	down
A_33_P3881262	CSF3R	-1.236869	-1.381846	-1.151652	-0.144977	0.0852175	-0.02988	down
A_23_P23221	GADD45A	3.5916748	3.5103087	3.613162	-0.081366	0.0214872	-0.029939	down
A_21_P0000597	CTSLP8	1.2253389	1.0005035	1.3902736	-0.224835	0.1649346	-0.02995	down
A_33_P3339860	PLEKHA2	4.355254	4.339378	4.3111115	-0.015876	-0.044143	-0.03001	down
A_23_P61426	MSRA	3.1531487	3.353887	2.8923674	0.2007384	-0.260781	-0.030021	down
A_23_P257131	PEX13	2.2500763	2.485495	1.9545383	0.2354188	-0.295538	-0.03006	down
A_22_P00005536	FLJ38668	-2.645133	-2.430315	-2.920106	0.214818	-0.274974	-0.030078	down
A_23_P259135	NCOA6	3.5987024	3.576363	3.5608587	-0.022339	-0.037844	-0.030092	down
A_23_P59418	NRF1	-0.88661	-1.044088	-0.789544	-0.157478	0.0970664	-0.030206	down
A_24_P241276	EXOSC8	5.114377	5.11002	5.05826	-0.004357	-0.056117	-0.030237	down
A_33_P3390708	TMEM2	1.7061825	1.6114678	1.7403674	-0.094715	0.0341849	-0.030265	down
A_22_P00018315	lnc-ZNF852-2	-1.363589	-1.256643	-1.531065	0.106946	-0.167476	-0.030265	down
A_23_P95050	ARFIP1	-0.404269	-0.621285	-0.247811	-0.217017	0.1564579	-0.030279	down
A_23_P150394	FXYD6	-2.955068	-2.605055	-3.365691	0.3500133	-0.410623	-0.030305	down
A_23_P75330	HNRNPF	4.118289	3.9684663	4.2073555	-0.149823	0.0890665	-0.030378	down
A_23_P71867	IL11RA	-0.73801	-0.709276	-0.82757	0.0287337	-0.08956	-0.030413	down
A_23_P375	CDCA8	5.111356	4.8858542	5.2759895	-0.225502	0.1646338	-0.030434	down
A_21_P0003274	lnc-AC069257.9.1-6	-1.819906	-2.230482	-1.470395	-0.410576	0.3495112	-0.030532	down
A_23_P94860	NUDT9	4.0298347	3.9337173	4.0648317	-0.096118	0.034997	-0.03056	down

A_33_P3244818	RSBN1L	1.7793188	1.8030396	1.6944056	0.0237207	-0.084913	-0.030596	down
A_24_P664939	CNBP	1.9792728	1.945518	1.9517574	-0.033755	-0.027515	-0.030635	down
A_21_P0005573	lnc-PIK3CG-2	0.4134874	0.6210976	0.1445928	0.2076101	-0.268895	-0.030642	down
A_23_P37068	TRAF3	-0.447899	-0.471634	-0.48554	-0.023735	-0.037641	-0.030688	down
A_23_P42935	BRAF	0.539166	0.5875382	0.4293666	0.0483723	-0.109799	-0.030714	down
A_33_P3232828	SRSF3	5.5366783	5.4089217	5.6028767	-0.127757	0.0661984	-0.030779	down
A_24_P73389	STK24	0.6514702	0.680203	0.5611424	0.0287328	-0.090328	-0.030797	down
A_33_P3317979	MYO5B	-2.228437	-2.050109	-2.468402	0.1783276	-0.239965	-0.030819	down
A_24_P166645	REPIN1	2.0178928	1.9884343	1.9855556	-0.029459	-0.032337	-0.030898	down
A_22_P00006145	lnc-FAM18A-1	-1.182511	-1.445992	-0.980915	-0.263481	0.2015958	-0.030943	down
A_23_P27983	APLP1	-2.07906	-2.05366	-2.166773	0.0253992	-0.087713	-0.031157	down
A_24_P129632	DLG5	3.039609	3.106586	2.9101982	0.066977	-0.129411	-0.031217	down
A_23_P86252	PIGC	3.2410965	3.0349326	3.3842592	-0.206164	0.1431627	-0.031501	down
A_32_P11230	LINC00999	2.8973398	2.924295	2.8073483	0.0269551	-0.089992	-0.031518	down
A_23_P254801	PLCG1	-0.436393	-0.406967	-0.528862	0.0294256	-0.092469	-0.031522	down
A_24_P310009	USP43	0.0453978	-0.035703	0.0634398	-0.0811	0.0180421	-0.031529	down
A_23_P150693	FJX1	2.3280134	2.1452422	2.4475422	-0.182771	0.1195288	-0.031621	down
A_33_P3335596	C17orf62	0.099062	-0.02992	0.1646915	-0.128982	0.0656295	-0.031676	down
A_23_P162702	HECTD4	2.2512255	2.2978854	2.1411963	0.0466599	-0.110029	-0.031685	down
A_23_P57547	SLC25A17	3.411005	3.3259053	3.4327145	-0.0851	0.0217094	-0.031695	down
A_33_P3290945	PTCD3	0.8023439	0.7255392	0.8156433	-0.076805	0.0132995	-0.031753	down
A_21_P0008786	lnc-RGMA-9	-1.83113	-1.659175	-2.067131	0.1719546	-0.236001	-0.032023	down
A_23_P160869	LRIG2	-0.203359	-0.242065	-0.229002	-0.038706	-0.025642	-0.032174	down
A_24_P50908	TRIM11	1.9297276	1.629704	2.1653557	-0.300024	0.2356281	-0.032198	down
A_24_P13230	RAB6A	2.5088472	2.5282903	2.4249926	0.019443	-0.083855	-0.032206	down
A_23_P21785	NSUN3	-0.053677	-0.120583	-0.051302	-0.066906	0.0023742	-0.032266	down
A_33_P3252236	N6AMT1	0.8517799	0.775867	0.8630524	-0.075913	0.0112724	-0.03232	down
A_24_P291133	UHMK1	-0.628087	-0.590582	-0.730433	0.0375047	-0.102346	-0.032421	down
A_33_P3636080	MLH3	-0.548642	-0.408179	-0.754166	0.1404634	-0.205523	-0.03253	down
A_24_P161261	SPDYE2	-2.538262	-2.399037	-2.74277	0.139225	-0.204508	-0.032641	down
A_23_P107775	TMEM190	-2.768627	-2.481753	-3.12095	0.2868745	-0.352322	-0.032724	down
A_21_P0014847	CYB561D2	-0.479254	-0.631451	-0.392656	-0.152197	0.0865974	-0.0328	down
A_24_P50801	NRP2	0.4278321	0.1385436	0.6515117	-0.289289	0.2236795	-0.032804	down
A_23_P79769	BIRC7	-2.776582	-2.642306	-2.976749	0.1342766	-0.200166	-0.032945	down
A_24_P74981	KDM1A	0.4006314	0.3497887	0.3853788	-0.050843	-0.015253	-0.033048	down
A_32_P8251	PHRF1	5.822584	5.7298627	5.8491697	-0.092721	0.0265856	-0.033068	down
A_24_P390833	MPPE1	-0.35795	-0.481838	-0.300232	-0.123888	0.0577178	-0.033085	down
A_23_P318581	CFAP97	3.992836	3.961329	3.9581194	-0.031507	-0.034717	-0.033112	down
A_33_P3318642	MC1R	-2.931796	-2.636363	-3.293544	0.2954323	-0.361749	-0.033158	down
A_33_P3240115	SEPT8	-0.884131	-0.944108	-0.890564	-0.059977	-0.006433	-0.033205	down
A_23_P215111	ATP6V0A4	-0.193903	0.0409837	-0.495208	0.2348871	-0.301304	-0.033209	down
A_24_P298420	PRMT5	0.889678	0.9425359	0.7702127	0.0528579	-0.119465	-0.033304	down
A_33_P3252369	TMEM182	0.0947466	0.1475515	-0.024679	0.0528049	-0.119425	-0.03331	down
A_24_P931964	LOC100128816	-1.652242	-1.583102	-1.788004	0.06914	-0.135763	-0.033311	down
A_32_P172141	CDON	-0.921182	-0.945745	-0.963277	-0.024563	-0.042096	-0.033329	down
A_33_P3347161	TBL1X	0.1118794	0.4358163	-0.278759	0.3239369	-0.390639	-0.033351	down
A_23_P144677	LNPEP	2.6071835	2.5368056	2.6107578	-0.070378	0.0035744	-0.033402	down
A_22_P00016106	lnc-THSD4-4	-2.378079	-2.579479	-2.243507	-0.2014	0.1345716	-0.033414	down
A_33_P3229725	ARSA	1.9659758	2.0188184	1.8462715	0.0528426	-0.119704	-0.033431	down
A_23_P111112	VARS2	3.3330317	3.2435918	3.355402	-0.08944	0.0223703	-0.033535	down
A_23_P12503	NUP133	3.5638885	3.5703845	3.490285	0.006496	-0.073604	-0.033554	down
A_24_P195476	PMS2	2.1404781	2.0247283	2.1891136	-0.11575	0.0486355	-0.033557	down
A_33_P3238171	ZDHHC8	-1.886939	-1.705105	-2.135939	0.1818337	-0.249	-0.033583	down
A_33_P3314386	TOR1AIP1	2.5170221	2.5835996	2.3832474	0.0665774	-0.133775	-0.033599	down
A_24_P270525	GTF2A2	5.4765425	5.2435174	5.6423073	-0.233025	0.1657648	-0.03363	down
A_23_P193	EXO5	2.4523	2.4053369	2.4318724	-0.046963	-0.020428	-0.033695	down

A_23_P319583	RIMS3	3.239482	3.0869603	3.3245983	-0.152522	0.0851164	-0.033703	down
A_33_P3651994	ERCC8	1.8295422	1.8924055	1.6991091	0.0628634	-0.130433	-0.033785	down
A_23_P24555	PHLDB1	1.483911	1.4267173	1.4734697	-0.057194	-0.010441	-0.033818	down
A_24_P15292	SPRTN	0.7486491	0.6205521	0.8089132	-0.128097	0.0602641	-0.033916	down
A_22_P00014561	lnc-SIX6-1	-2.272877	-2.145385	-2.468284	0.1274927	-0.195406	-0.033957	down
A_21_P0000379	SNORD67	-2.588397	-2.554734	-2.690165	0.0336633	-0.101768	-0.034052	down
A_19_P00811264	lnc-TSHZ1-1	-0.498677	-0.409974	-0.655513	0.0887027	-0.156836	-0.034067	down
A_23_P171077	EBP	6.5621204	6.2490973	6.8069572	-0.313023	0.2448368	-0.034093	down
A_24_P915007	NACC1	4.8747435	4.6846642	4.996628	-0.190079	0.1218843	-0.034097	down
A_24_P347488	PSMC1	5.9306593	5.894939	5.898102	-0.03572	-0.032557	-0.034139	down
A_23_P63660	FAM213A	5.0521584	4.9141955	5.1217966	-0.137963	0.0696383	-0.034162	down
A_32_P15421	RNF185	-0.970909	-0.876526	-1.133662	0.0943828	-0.162753	-0.034185	down
A_23_P104151	TMEM69	2.0555487	2.0981612	1.944519	0.0426126	-0.11103	-0.034209	down
A_32_P71788	FKBP4	2.777852	2.6219087	2.8651056	-0.155943	0.0872536	-0.034345	down
A_33_P3237517	ZNF292	-1.197348	-0.823116	-1.640486	0.3742313	-0.443138	-0.034453	down
A_22_P00003430	lnc-CCDC33-2	0.1065254	0.0392594	0.1047144	-0.067266	-0.001811	-0.034539	down
A_33_P3367642	LMF2	5.208193	5.1144376	5.2328234	-0.093755	0.0246305	-0.034562	down
A_33_P3252177	EID2B	-2.270976	-2.227339	-2.383821	0.0436368	-0.112845	-0.034604	down
A_22_P00025917	LOC100506603	1.611887	1.378057	1.7764826	-0.23383	0.1645956	-0.034617	down
A_23_P5415	NIF3L1	4.1690855	4.04015	4.228586	-0.128935	0.0595007	-0.034717	down
A_33_P3376828	CMTM7	1.1180439	0.9051457	1.2614202	-0.212898	0.1433764	-0.034761	down
A_33_P3271350	IKBKAP	-0.115477	-0.082338	-0.218154	0.0331388	-0.102678	-0.03477	down
A_21_P0001245	LOC101927851	-0.636253	-0.729213	-0.612836	-0.092959	0.0234175	-0.034771	down
A_33_P3401267	FEZ2	-1.511528	-1.173466	-1.919264	0.3380618	-0.407736	-0.034837	down
A_23_P147729	SLC35E3	0.3993726	0.4142141	0.3146954	0.0148416	-0.084677	-0.034918	down
A_21_P0000241	SNORA33	1.1893191	0.9018192	1.4069667	-0.2875	0.2176476	-0.034926	down
A_24_P84880	LOC148709	2.6168633	2.5227237	2.6411257	-0.09414	0.0242624	-0.034939	down
A_33_P3230583	LOC642236	-1.982857	-2.020451	-2.015197	-0.037593	-0.03234	-0.034966	down
A_23_P40952	RAF1	3.168662	3.1425376	3.124733	-0.026124	-0.043929	-0.035027	down
A_24_P233878	GDAP2	-0.742351	-0.822078	-0.73297	-0.079727	0.0093803	-0.035173	down
A_23_P167789	FAM53C	2.793851	2.7978902	2.7194366	0.0040393	-0.074414	-0.035187	down
A_32_P226149	YWHAZ	6.308672	6.3461375	6.200822	0.0374656	-0.10785	-0.035192	down
A_23_P17503	KIF16B	1.5840645	1.512126	1.5854645	-0.071939	0.0014	-0.035269	down
A_32_P163247	CD8A	-1.069996	-1.098287	-1.112247	-0.028291	-0.042251	-0.035271	down
A_33_P3416221	NDUFA10	-0.690776	-0.669764	-0.782664	0.0210123	-0.091888	-0.035438	down
A_33_P3216232	ITGB1BP1	3.0501442	2.7106233	3.3186522	-0.339521	0.268508	-0.035506	down
A_23_P372660	GAPVD1	1.5078068	1.3670969	1.5774293	-0.14071	0.0696225	-0.035544	down
A_24_P191847	PTCD2	-1.907507	-1.911064	-1.97509	-0.003557	-0.067583	-0.03557	down
A_23_P105862	FRY	-0.726775	-0.718002	-0.806694	0.0087733	-0.079918	-0.035573	down
A_33_P3229328	NBPF6	-1.420651	-1.198458	-1.713991	0.2221923	-0.29334	-0.035574	down
A_24_P233944	CEPT1	-0.280602	-0.266006	-0.366456	0.014596	-0.085854	-0.035629	down
A_24_P873764	BCR	3.863719	3.6945567	3.9616146	-0.169162	0.0978956	-0.035633	down
A_23_P132175	RTN4R	3.8997555	3.518846	4.2093754	-0.380909	0.3096199	-0.035645	down
A_33_P3327454	RSPRY1	3.6567326	3.538445	3.7037182	-0.118288	0.0469856	-0.035651	down
A_23_P363275	WDR66	-2.896854	-2.940989	-2.924167	-0.044135	-0.027313	-0.035724	down
A_23_P342934	TLE3	0.0574689	0.1271858	-0.083907	0.0697169	-0.141376	-0.03583	down
A_24_P406060	RNF144B	-2.382389	-2.073576	-2.763052	0.3088126	-0.380663	-0.035925	down
A_24_P189458	NSMCE4A	2.3652086	2.325626	2.3329315	-0.039583	-0.032277	-0.03593	down
A_24_P323114	ANXA2P3	2.416422	2.4860435	2.27493	0.0696216	-0.141492	-0.035935	down
A_24_P924862	RAPH1	1.1994419	1.2051868	1.121768	0.0057449	-0.077674	-0.035964	down
A_21_P0000148	NDUFB6	6.2857637	6.100221	6.3993654	-0.185543	0.1136017	-0.03597	down
A_23_P213678	PAM	5.9488745	5.979344	5.846367	0.0304694	-0.102508	-0.036019	down
A_33_P3377994	WNK4	-1.16314	-1.179071	-1.219408	-0.015931	-0.056267	-0.036099	down
A_33_P3210099	ALPK3	-1.512507	-1.315661	-1.781575	0.196847	-0.269067	-0.03611	down
A_33_P3233005	MBTD1	0.1695514	0.1709714	0.0958881	0.00142	-0.073663	-0.036122	down
A_23_P135778	PPP2R2D	2.3433037	2.2727866	2.3411846	-0.070517	-0.002119	-0.036318	down

A_33_P3211153	ANKS3	3.3838959	3.3062005	3.3888988	-0.077695	0.005003	-0.036346	down
A_23_P42498	SNX3	5.9389753	5.9921746	5.81293	0.0531993	-0.126045	-0.036423	down
A_21_P0010867	ZNF37BP	-1.5036	-1.534305	-1.545771	-0.030705	-0.042171	-0.036438	down
A_23_P370588	HOXB8	-1.796971	-2.128839	-1.538184	-0.331868	0.2587872	-0.03654	down
A_22_P00008737	lnc-KIAA1755-6	1.079165	1.0202093	1.064919	-0.058956	-0.014246	-0.036601	down
A_23_P409462	DCBLD1	-0.150802	-0.272158	-0.102687	-0.121356	0.0481148	-0.036621	down
A_23_P101737	DOT1L	0.3142905	0.3144035	0.2408662	0.000113	-0.073424	-0.036656	down
A_33_P3342633	DHRS12	-1.109725	-1.42856	-0.864318	-0.318836	0.2454062	-0.036715	down
A_23_P11237	TAF1	0.1737437	0.2555223	0.0182137	0.0817785	-0.15553	-0.036876	down
A_23_P218079	SLC38A2	3.414772	3.4967647	3.2589102	0.0819926	-0.155862	-0.036935	down
A_33_P3269588	GABPA	-0.157671	-0.167169	-0.222176	-0.009498	-0.064505	-0.037002	down
A_24_P25234	MTL5	-1.270086	-1.31203	-1.302496	-0.041944	-0.03241	-0.037177	down
A_23_P5912	YTHDF1	6.357606	6.250319	6.390539	-0.107287	0.0329332	-0.037177	down
A_23_P98532	ZDHHC5	3.9035902	3.747995	3.9846678	-0.155595	0.0810776	-0.037259	down
A_33_P3294392	EDC4	5.3975153	4.9973226	5.723176	-0.400193	0.3256607	-0.037266	down
A_33_P3342862	ZNF664-FAM101A	-2.654241	-2.582649	-2.800398	0.0715921	-0.146157	-0.037282	down
A_24_P941336	TSR1	3.2793016	3.1455665	3.338358	-0.133735	0.0590563	-0.037339	down
A_23_P17275	DNAJC27	-1.39288	-1.198175	-1.662293	0.194705	-0.269414	-0.037354	down
A_23_P201655	MYCBP	4.5610256	4.3334737	4.7136908	-0.227552	0.1526651	-0.037443	down
A_23_P51410	SMYD3	-0.486015	-0.530011	-0.516994	-0.043995	-0.030979	-0.037487	down
A_23_P144697	RAD1	1.6643734	1.7914119	1.462265	0.1270385	-0.202108	-0.037535	down
A_23_P128991	SLIRP	8.336505	8.193111	8.404763	-0.143394	0.0682583	-0.037568	down
A_24_P98613	TSPAN14	2.3160563	2.1937299	2.3631582	-0.122326	0.047102	-0.037612	down
A_23_P113184	FTO	3.029808	2.9438195	3.0405245	-0.085989	0.0107164	-0.037636	down
A_23_P76823	ADSSL1	1.2718687	1.4356313	1.0328326	0.1637626	-0.239036	-0.037637	down
A_23_P391228	MANEAL	3.8959398	3.5920215	4.124551	-0.303918	0.228611	-0.037654	down
A_33_P3379644	ALDH1A1	2.006836	1.9559064	1.9824362	-0.05093	-0.0244	-0.037665	down
A_33_P3383524	LINC00923	1.1840658	1.3002348	0.9924908	0.116169	-0.191575	-0.037703	down
A_23_P91919	SERP1	4.99434	5.047111	4.866062	0.0527711	-0.128278	-0.037753	down
A_33_P3211569	ERBB3	2.0091639	1.7246757	2.2181034	-0.284488	0.2089396	-0.037774	down
A_24_P298013	GTPBP2	-0.963658	-0.672137	-1.330918	0.2915216	-0.36726	-0.037869	down
A_22_P00010081	WAC-AS1	-1.65968	-1.56704	-1.828163	0.0926404	-0.168483	-0.037921	down
A_23_P93772	HOXA5	1.411439	1.7615466	0.9854264	0.3501077	-0.426013	-0.037952	down
A_33_P3714477	LINC00996	-2.587002	-2.656301	-2.593765	-0.069299	-0.006763	-0.038031	down
A_23_P376188	BEND7	-0.082175	-0.161957	-0.078463	-0.079782	0.0037127	-0.038034	down
A_33_P3368500	IFT52	3.4761448	3.4586911	3.4172707	-0.017454	-0.058874	-0.038164	down
A_24_P77681	PAIP1	3.0235186	3.0414548	2.9290323	0.0179362	-0.094486	-0.038275	down
A_33_P3320548	NUPL2	2.1991987	1.9490886	2.372735	-0.25011	0.1735363	-0.038287	down
A_33_P3374563	SPATA7	0.2232218	0.2221017	0.147748	-0.00112	-0.075474	-0.038297	down
A_24_P122732	SLC41A1	3.4763107	3.1437764	3.7322254	-0.332534	0.2559147	-0.03831	down
A_23_P58529	DIMT1	4.836178	4.7272687	4.8684464	-0.108909	0.0322685	-0.03832	down
A_33_P3413958	OR7E47P	-0.090717	0.0532913	-0.311381	0.1440082	-0.220665	-0.038328	down
A_23_P156471	CDC5L	2.910862	2.7583919	2.9865303	-0.15247	0.0756683	-0.038401	down
A_33_P3236133	SHQ1	-1.576159	-1.621188	-1.607959	-0.045029	-0.0318	-0.038414	down
A_23_P20743	TMEM246	4.4098034	4.1960635	4.546646	-0.21374	0.1368427	-0.038449	down
A_33_P3294578	PRKCI	3.504918	3.4754996	3.4574394	-0.029418	-0.047479	-0.038449	down
A_21_P0011448	XLOC_l2_005314	0.0311046	0.009563	-0.024331	-0.021542	-0.055436	-0.038489	down
A_24_P98385	RNF20	-0.273222	-0.521883	-0.101679	-0.248661	0.1715426	-0.038559	down
A_33_P3243554	SEPT6	-1.038172	-1.314428	-0.839278	-0.276256	0.1988945	-0.038681	down
A_33_P3352941	FBXO22-AS1	-1.141388	-1.175292	-1.184971	-0.033904	-0.043583	-0.038743	down
A_33_P3215128	IBA57	0.0207281	-0.089717	0.0536313	-0.110445	0.0329032	-0.038771	down
A_23_P89455	SLC35B1	3.5006504	3.3003793	3.6233397	-0.200271	0.1226893	-0.038791	down
A_21_P0001665	lnc-APITD1-1	0.5415483	0.2999029	0.7055183	-0.241645	0.16397	-0.038838	down
A_23_P115919	PHYH	1.7809572	1.8024774	1.6817017	0.0215201	-0.099256	-0.038868	down
A_24_P406754	LOXL4	4.1315336	4.2180867	3.9672432	0.0865531	-0.16429	-0.038869	down
A_23_P118002	UQCRC2	6.0359344	5.826923	6.1671925	-0.209012	0.131258	-0.038877	down

A_33_P3215768	GALNT6	2.263276	2.243196	2.205573	-0.02008	-0.057703	-0.038892	down
A_33_P3323722	ARL4C	3.1534243	3.041905	3.187089	-0.111519	0.0336647	-0.038927	down
A_33_P3359748	SARS	-1.695582	-1.620623	-1.848416	0.0749593	-0.152833	-0.038937	down
A_33_P3349827	RBM3	1.7392836	1.7506337	1.6499577	0.0113502	-0.089326	-0.038988	down
A_23_P29975	C4orf19	0.2272511	0.2484994	0.1280012	0.0212483	-0.09925	-0.039001	down
A_23_P153586	UQCRFS1	7.8449087	7.871403	7.740287	0.0264945	-0.104622	-0.039064	down
A_23_P152305	CDH11	-2.832318	-2.285085	-3.457683	0.5472329	-0.625365	-0.039066	down
A_22_P00014096	lnc-SASS6-2	-2.926887	-3.009369	-2.922557	-0.082483	0.0043294	-0.039077	down
A_33_P3217073	GHITM	6.9408216	6.953833	6.8495865	0.0130115	-0.091235	-0.039112	down
A_32_P14850	NPIPB15	5.7532997	5.927221	5.5011387	0.1739211	-0.252161	-0.03912	down
A_22_P00015826	lnc-TBC1D22A-2	-1.956286	-2.09104	-1.899916	-0.134754	0.0563703	-0.039192	down
A_23_P130856	CEP89	0.4084497	0.3780432	0.3604703	-0.030406	-0.047979	-0.039193	down
A_22_P00020400	LOC730183	0.3329973	0.1642499	0.4232607	-0.168747	0.0902634	-0.039242	down
A_32_P41065	TMCC1	3.3337955	3.4003053	3.1887722	0.0665097	-0.145023	-0.039257	down
A_33_P3375576	MAP7	2.385439	2.251369	2.4409552	-0.13407	0.0555162	-0.039277	down
A_22_P00003871	CELF6	-1.840099	-1.837504	-1.921297	0.0025954	-0.081198	-0.039301	down
A_23_P88893	DEF8	6.0169935	5.927172	6.028078	-0.089821	0.0110846	-0.039368	down
A_24_P96403	RUNX1	3.1445074	2.9833636	3.2268267	-0.161144	0.0823193	-0.039412	down
A_24_P236935	KLK6	5.538539	5.715057	5.2831554	0.176518	-0.255384	-0.039433	down
A_23_P374294	CCDC7	-2.86692	-2.934582	-2.878183	-0.067662	-0.011262	-0.039462	down
A_22_P00016415	ADAT1	-0.639015	-0.643115	-0.713875	-0.004099	-0.07486	-0.039479	down
A_24_P920125	PPIA	1.6247864	1.2849445	1.8854675	-0.339842	0.2606812	-0.03958	down
A_24_P152968	AKR1C1	1.5795527	2.2218642	0.8578639	0.6423116	-0.721689	-0.039689	down
A_22_P00021231	LOC101928346	-2.303429	-2.369886	-2.316373	-0.066457	-0.012944	-0.0397	down
A_33_P3420254	DNAAF5	1.2560558	1.1507134	1.2818475	-0.105342	0.0257916	-0.039775	down
A_23_P428842	TMEM44	1.0176659	0.9996171	0.956069	-0.018049	-0.061597	-0.039823	down
A_24_P307175	SMYD4	1.7207594	1.6085773	1.7532353	-0.112182	0.032476	-0.039853	down
A_22_P00003163	lnc-C9orf53-1	-2.311792	-2.197417	-2.505964	0.1143749	-0.194172	-0.039899	down
A_23_P322845	PPAPDC1B	0.4553461	0.5580196	0.2726789	0.1026735	-0.182667	-0.039997	down
A_22_P00009059	LENG8-AS1	-1.281004	-1.338911	-1.303245	-0.057908	-0.022242	-0.040075	down
A_22_P00014220	lnc-SEC24C-1	0.0052404	-0.092459	0.0227141	-0.0977	0.0174737	-0.040113	down
A_21_P0010748	SEPT14	-0.043416	0.1208773	-0.287948	0.1642928	-0.244533	-0.04012	down
A_24_P238250	LGALS7	0.7322593	0.657063	0.7271271	-0.075196	-0.005132	-0.040164	down
A_21_P0013299	XLOC_l2_013808	-2.661734	-3.160343	-2.24354	-0.498608	0.4181941	-0.040207	down
A_33_P3242543	MAOA	1.363883	1.2184119	1.4289203	-0.145471	0.0650373	-0.040217	down
A_22_P00014194	lnc-SDSL-4	-2.716501	-2.847525	-2.665934	-0.131024	0.0505667	-0.040229	down
A_33_P3408983	APC	0.2934208	0.2395849	0.2667942	-0.053836	-0.026627	-0.040231	down
A_23_P105562	VWF	-0.817313	-0.520762	-1.194465	0.2965512	-0.377152	-0.040301	down
A_22_P00003546	PIGBOS1	1.581027	1.4994507	1.5819983	-0.081576	0.0009713	-0.040303	down
A_23_P7582	TCF7	3.2538948	3.17024	3.2569027	-0.083655	0.0030079	-0.040323	down
A_23_P63929	WDR11	2.0723581	2.1574292	1.9066324	0.0850711	-0.165726	-0.040327	down
A_23_P21436	PHF19	0.7660394	0.4436016	1.0074692	-0.322438	0.2414298	-0.040504	down
A_33_P3489228	PTPN20B	-2.502729	-2.52837	-2.558208	-0.025641	-0.055479	-0.04056	down
A_23_P373598	MAFK	2.642641	2.7730107	2.4311218	0.1303697	-0.211519	-0.040575	down
A_22_P00007866	LOC100506974	-1.227532	-1.193403	-1.342828	0.0341291	-0.115295	-0.040583	down
A_23_P144096	CISH	-0.774213	-1.045816	-0.58385	-0.271603	0.1903629	-0.04062	down
A_23_P251795	GPC2	0.475811	0.4020791	0.4681053	-0.073732	-0.007706	-0.040719	down
A_32_P415151	WDR27	-2.519163	-2.505905	-2.613892	0.0132582	-0.094728	-0.040735	down
A_23_P99540	ZFP36L1	6.481098	6.498236	6.382002	0.017138	-0.099096	-0.040979	down
A_24_P247978	ZNF589	-2.133402	-2.240258	-2.108529	-0.106856	0.0248737	-0.040991	down
A_23_P65240	COL4A1	-2.094767	-2.055452	-2.216088	0.0393152	-0.121321	-0.041003	down
A_23_P2998	OXA1L	2.580593	2.295545	2.7835703	-0.285048	0.2029772	-0.041035	down
A_32_P70927	PAGE2	-3.084233	-2.979638	-3.270939	0.1045952	-0.186706	-0.041055	down
A_33_P3372285	ZNF81	-2.053221	-2.040099	-2.148457	0.0131221	-0.095236	-0.041057	down
A_24_P913339	SLC35F6	1.5775232	1.4928694	1.5796266	-0.084654	0.0021033	-0.041275	down
A_23_P215675	COA1	3.2744503	3.1702266	3.2961159	-0.104224	0.0216656	-0.041279	down

A_33_P3388938	SNAI3-AS1	-2.881013	-2.982962	-2.861664	-0.101949	0.0193491	-0.0413	down
A_33_P3258824	NOTCH2	1.7145443	1.7057943	1.6405897	-0.00875	-0.073955	-0.041352	down
A_33_P3239347	NKX3-1	-0.900399	-0.90252	-0.981013	-0.00212	-0.080614	-0.041367	down
A_33_P3230339	TTF1	2.0423079	1.8351026	2.166751	-0.207205	0.1244431	-0.041381	down
A_23_P204847	LCP1	-0.177782	-0.496673	0.0582247	-0.318891	0.2360067	-0.041442	down
A_23_P101237	C18orf21	2.4036312	2.3258662	2.3985052	-0.077765	-0.005126	-0.041445	down
A_21_P0010891	NUTM2B-AS1	2.1565485	1.9642205	2.2659407	-0.192328	0.1093922	-0.041468	down
A_32_P182473	ZNF625	2.0233803	1.9765449	1.9870358	-0.046835	-0.036345	-0.04159	down
A_33_P3378101	AGBL4	-2.527444	-2.657841	-2.480361	-0.130397	0.0470836	-0.041657	down
A_22_P00015349	LOC283335	-0.031405	-0.005429	-0.140856	0.0259762	-0.109451	-0.041737	down
A_24_P322369	VPS4B	3.3285532	3.3542418	3.2193317	0.0256886	-0.109221	-0.041766	down
A_24_P131589	CD86	-0.167006	-0.138625	-0.279291	0.0283809	-0.112286	-0.041952	down
A_23_P45976	RAP1GAP	-2.328893	-2.272476	-2.46943	0.0564172	-0.140537	-0.04206	down
A_23_P426021	SEL1L3	3.8420334	3.6936383	3.906273	-0.148395	0.0642395	-0.042078	down
A_22_P00022181	lnc-SUMO1-1	-0.05625	-0.202648	0.0059776	-0.146398	0.0622277	-0.042085	down
A_23_P88201	PPP2R3C	2.5675821	2.6232705	2.4276505	0.0556884	-0.139932	-0.042122	down
A_33_P3412149	CLCC1	1.7055321	1.7119241	1.614872	0.006392	-0.09066	-0.042134	down
A_21_P0005869	lnc-TMEM64-1	-0.627327	-0.601644	-0.7373	0.0256834	-0.109973	-0.042145	down
A_33_P3214586	ARTN	-1.065647	-1.074054	-1.141616	-0.008407	-0.075969	-0.042188	down
A_23_P159305	TAF15	4.4748507	3.8066897	5.0584936	-0.668161	0.583643	-0.042259	down
A_33_P3283237	YY2	-0.657486	-0.574904	-0.824647	0.082581	-0.167161	-0.04229	down
A_33_P3217153	GRIA4	-1.615157	-1.811925	-1.503191	-0.196769	0.1119657	-0.042402	down
A_23_P88589	NR2F2	4.32837	4.1468406	4.425091	-0.18153	0.0967207	-0.042404	down
A_23_P47377	HSD17B12	4.320627	4.2274117	4.3290157	-0.093215	0.0083885	-0.042413	down
A_33_P3390758	HSPA8	6.9338865	6.853168	6.92962	-0.080719	-0.004267	-0.042493	down
A_23_P107307	SLC13A2	-1.285974	-1.370477	-1.286657	-0.084503	-0.000683	-0.042593	down
A_33_P3362353	ARAF	0.1239748	0.0267301	0.135921	-0.097245	0.0119462	-0.042649	down
A_24_P785293	SNHG1	5.657522	5.880717	5.348774	0.2231946	-0.308748	-0.042777	down
A_33_P3313030	TXNDC15	-1.052467	-1.05939	-1.131152	-0.006922	-0.078685	-0.042804	down
A_24_P120115	CFLAR	0.03406	0.0626364	-0.080342	0.0285764	-0.114402	-0.042913	down
A_22_P00000129	RIMKLB	-0.501689	-0.290813	-0.798497	0.210876	-0.296807	-0.042966	down
A_23_P311895	CLIC5	0.045238	-0.167724	0.1721625	-0.212962	0.1269245	-0.043019	down
A_24_P266728	SF1	5.8730984	5.719402	5.9406624	-0.153697	0.067564	-0.043066	down
A_24_P294821	SYNJ2	-1.858078	-1.955542	-1.847146	-0.097464	0.010932	-0.043266	down
A_23_P930	APH1A	1.3256774	1.052908	1.5118737	-0.272769	0.1861963	-0.043287	down
A_23_P258698	MANBA	1.4804611	1.4950671	1.3791537	0.014606	-0.101307	-0.043351	down
A_21_P0008899	lnc-NME4-1	-2.932444	-2.829864	-3.12182	0.1025798	-0.189376	-0.043398	down
A_21_P0009813	LOC101927820	-2.48044	-2.748353	-2.299346	-0.267913	0.1810944	-0.043409	down
A_23_P784	LZIC	3.4018173	3.4778104	3.2388935	0.0759931	-0.162924	-0.043465	down
A_23_P141863	ZNF544	1.1882553	1.187448	1.1020851	-0.000807	-0.08617	-0.043489	down
A_24_P802145	IRS1	0.1908374	-0.06825	0.3628326	-0.259087	0.1719952	-0.043546	down
A_22_P00024701	MFSD11	-1.036241	-1.074833	-1.084745	-0.038592	-0.048504	-0.043548	down
A_33_P3531857	DARS	5.3728695	5.2588563	5.3997717	-0.114013	0.0269022	-0.043555	down
A_23_P132718	SEMA3B	4.552253	4.762504	4.2547464	0.2102513	-0.297506	-0.043628	down
A_23_P26124	RORA	1.3360958	1.3283124	1.2564311	-0.007783	-0.079665	-0.043724	down
A_22_P00022623	lnc-EIF4E1B-1	-2.381205	-2.471521	-2.378338	-0.090316	0.0028667	-0.043725	down
A_22_P00009215	BZRAP1-AS1	-2.216517	-2.475377	-2.045268	-0.25886	0.1712489	-0.043806	down
A_33_P3353343	SRRM2	-0.189786	0.0075898	-0.474891	0.1973763	-0.285104	-0.043864	down
A_23_P87964	ESD	1.426023	1.2877574	1.4764986	-0.138266	0.0504756	-0.043895	down
A_33_P3445805	PPP6R3	5.444543	5.313045	5.488226	-0.131498	0.0436831	-0.043907	down
A_33_P3367481	ARHGAP35	2.0720816	2.1181483	1.9378853	0.0460668	-0.134196	-0.044065	down
A_23_P171095	USP27X	-1.665834	-1.950074	-1.469865	-0.28424	0.1959682	-0.044136	down
A_23_P133694	SLC29A1	3.212182	2.9778256	3.358221	-0.234356	0.146039	-0.044159	down
A_21_P0009376	lnc-NLGN2-1	-1.982105	-1.958714	-2.093873	0.0233912	-0.111768	-0.044188	down
A_23_P55779	CYP2A13	-2.860654	-3.007051	-2.802682	-0.146397	0.0579715	-0.044213	down
A_23_P98844	ARHGEF25	-2.925307	-3.084838	-2.854366	-0.159531	0.0709415	-0.044295	down

A_23_P154488	PNPT1	3.8892183	3.608605	4.0810947	-0.280613	0.1918764	-0.044369	down
A_33_P3394183	ZNF324B	1.7810478	1.5178413	1.9554281	-0.263206	0.1743803	-0.044413	down
A_24_P7179	DIP2A	-2.88767	-3.137709	-2.726609	-0.250039	0.1610611	-0.044489	down
A_24_P303193	HNRNPA0	1.1776695	1.0490351	1.2171516	-0.128634	0.0394821	-0.044576	down
A_22_P00000924	LOC101929549	-2.186537	-2.013343	-2.44897	0.1731939	-0.262433	-0.04462	down
A_19_P00320948	FAM200B	1.0303602	0.8564935	1.1149726	-0.173867	0.0846124	-0.044627	down
A_23_P141447	RDM1	1.309535	1.2831831	1.2466063	-0.026352	-0.062929	-0.04464	down
A_24_P62237	CERK	1.6840096	1.7694345	1.5092449	0.0854249	-0.174765	-0.04467	down
A_33_P3396492	CDH24	-0.791717	-0.778404	-0.894558	0.0133128	-0.102841	-0.044764	down
A_33_P3563369	SNORA10	3.6356678	3.487369	3.6941824	-0.148299	0.0585146	-0.044892	down
A_32_P35969	CHRNA7	-0.197196	-0.441001	-0.043294	-0.243806	0.1539016	-0.044952	down
A_21_P0012381	XLOC_l2_009790	-2.789242	-2.777902	-2.890554	0.0113406	-0.101311	-0.044985	down
A_23_P164387	SMARCE1	5.848365	5.8223233	5.784361	-0.026042	-0.064004	-0.045023	down
A_24_P156886	DDX59	-1.011425	-1.163965	-0.949035	-0.152541	0.0623894	-0.045076	down
A_33_P3354203	NELL1	-2.485098	-2.341301	-2.719082	0.1437969	-0.233984	-0.045094	down
A_24_P160001	FKBP1A	2.3249016	2.207892	2.351718	-0.11701	0.0268164	-0.045097	down
A_24_P342807	SLC30A6	-0.430865	-0.670808	-0.281308	-0.239943	0.1495566	-0.045193	down
A_19_P00808120	FAM200B	-0.598541	-0.562701	-0.724861	0.0358396	-0.12632	-0.04524	down
A_33_P3341656	MEF2D	2.6129484	2.5057702	2.6296215	-0.107178	0.0166731	-0.045253	down
A_23_P97021	AGO3	2.4879818	2.3557067	2.5296955	-0.132275	0.0417137	-0.045281	down
A_23_P136355	HHAT	0.1288824	-0.448275	0.615449	-0.577157	0.4865665	-0.045295	down
A_33_P6505283	LOC100507316	2.8449678	2.52346	3.075882	-0.321508	0.2309141	-0.045297	down
A_24_P64344	BLNK	-0.056462	-0.136069	-0.067484	-0.079607	-0.011022	-0.045315	down
A_33_P3209615	KRTAP4-8	-1.27358	-1.489609	-1.14825	-0.216029	0.1253305	-0.045349	down
A_23_P500381	HTR7	-0.794566	-1.087363	-0.592697	-0.292797	0.201869	-0.045464	down
A_33_P3314176	FAM46C	1.8462286	2.1759105	1.42557	0.3296819	-0.420659	-0.045488	down
A_23_P46275	ATP5F1	6.586769	6.541455	6.5408125	-0.045314	-0.045957	-0.045635	down
A_33_P3220530	SRSF6	5.729537	5.593885	5.77384	-0.135652	0.0443029	-0.045675	down
A_24_P379693	DNPEP	0.4261985	0.1921473	0.5688796	-0.234051	0.1426811	-0.045685	down
A_33_P3240538	TUBE1	1.4948668	1.6746283	1.2237139	0.1797614	-0.271153	-0.045696	down
A_33_P3333863	CCZ1	1.8876524	1.7037463	1.9801378	-0.183906	0.0924854	-0.04571	down
A_23_P365226	FAM161B	-2.453912	-2.426812	-2.572493	0.0270998	-0.118581	-0.045741	down
A_33_P3349716	ZNF766	3.4635038	3.4985766	3.3369455	0.0350728	-0.126558	-0.045743	down
A_33_P3417260	NMRK1	1.7817583	1.6659498	1.8060431	-0.115808	0.0242848	-0.045762	down
A_22_P00011215	EML2	-2.079229	-2.028532	-2.22178	0.0506973	-0.142551	-0.045927	down
A_33_P3441060	TMEM242	1.4180884	1.1856489	1.558557	-0.23244	0.1404686	-0.045985	down
A_23_P146798	SEPHS2	4.3557634	4.2415032	4.377782	-0.11426	0.0220184	-0.046121	down
A_33_P3238196	ZNF33A	-0.224689	0.034328	-0.575948	0.2590165	-0.351259	-0.046121	down
A_23_P45389	RAB9A	3.6294127	3.6831088	3.4834433	0.0536962	-0.145969	-0.046137	down
A_33_P3268910	SPRY1	2.0194693	1.9436984	2.0028505	-0.075771	-0.016619	-0.046195	down
A_21_P0014693	lnc-PRR11-1	-2.028889	-2.109143	-2.041085	-0.080254	-0.012196	-0.046225	down
A_19_P00320731	LOC101927043	-2.557175	-2.50479	-2.702063	0.0523846	-0.144888	-0.046252	down
A_23_P205997	APH1B	-1.905242	-1.942739	-1.960304	-0.037497	-0.055063	-0.04628	down
A_23_P65741	DIS3L	2.0383177	1.9187284	2.0651598	-0.119589	0.0268421	-0.046374	down
A_23_P65609	GTF2A1	1.5473876	1.58212	1.4197598	0.0347323	-0.127628	-0.046448	down
A_21_P0014572	lnc-CACNB3-1	-1.966133	-2.165343	-1.85985	-0.19921	0.1062832	-0.046463	down
A_33_P3238671	C20orf196	-0.504821	-0.777708	-0.324894	-0.272887	0.1799269	-0.04648	down
A_23_P387552	RARG	3.6910648	3.4666257	3.8224516	-0.224439	0.1313868	-0.046526	down
A_24_P90900	CTRL	-2.00527	-2.131666	-1.972032	-0.126396	0.0332375	-0.046579	down
A_24_P110914	TPT1-AS1	1.6022491	1.6777568	1.4335809	0.0755076	-0.168668	-0.04658	down
A_33_P3311863	AP2A2	-1.550133	-1.892977	-1.300458	-0.342844	0.2496758	-0.046584	down
A_24_P193295	RAB15	5.66148	5.7125163	5.5172157	0.0510364	-0.144264	-0.046614	down
A_33_P3300635	PFKFB2	3.1079407	3.1660352	2.9565096	0.0580945	-0.151431	-0.046668	down
A_33_P3369336	GCG	-2.087862	-2.22301	-2.046147	-0.135148	0.0417147	-0.046717	down
A_23_P362637	UBR2	2.4719553	2.499617	2.3505564	0.0276618	-0.121399	-0.046869	down
A_33_P3213557	CCZ1	0.3163118	0.3152504	0.2235699	-0.001061	-0.092742	-0.046902	down

A_33_P3404588	FGD4	0.5500307	0.5307608	0.4752035	-0.01927	-0.074827	-0.047049	down
A_21_P0000599	MKRN1	1.272038	1.3418231	1.1081476	0.0697851	-0.16389	-0.047053	down
A_23_P374689	GAD1	-0.451038	-0.878094	-0.118204	-0.427056	0.3328338	-0.047111	down
A_23_P160503	GLRX2	3.2782593	3.0336976	3.428546	-0.244562	0.1502867	-0.047138	down
A_23_P131202	HES6	-1.523558	-1.843204	-1.298408	-0.319645	0.2251506	-0.047247	down
A_23_P133755	PREP	3.9342318	3.593491	4.180415	-0.340741	0.2461834	-0.047279	down
A_23_P259272	WSB2	5.8523436	5.788015	5.8221064	-0.064329	-0.030237	-0.047283	down
A_23_P27606	IL27RA	1.3041306	0.9732418	1.5403571	-0.330889	0.2362266	-0.047331	down
A_33_P3364463	NKAIN3	-3.074967	-2.943443	-3.301198	0.1315243	-0.22623	-0.047353	down
A_24_P297537	MAMSTR	-1.741674	-1.440895	-2.137233	0.3007793	-0.395559	-0.04739	down
A_23_P127579	PTS	4.7341166	4.5156455	4.8575716	-0.218471	0.1234551	-0.047508	down
A_24_P58331	DCAKD	-1.207071	-1.278595	-1.230622	-0.071524	-0.023551	-0.047537	down
A_22_P00005087	DGAT1	1.5636892	1.2593637	1.7729173	-0.304326	0.209228	-0.047549	down
A_21_P0006941	LOC102723337	-2.280411	-2.055688	-2.60024	0.2247224	-0.319829	-0.047553	down
A_21_P0014186	LOC101927811	-0.058681	0.0423169	-0.254818	0.1009975	-0.196137	-0.04757	down
A_23_P341349	CAPN10	2.3806915	2.3216243	2.3445187	-0.059067	-0.036173	-0.04762	down
A_23_P90790	METAP1D	1.0921092	1.1607604	0.9281836	0.0686512	-0.163926	-0.047637	down
A_21_P0001496	lnc-PDZK1IP1-1	-2.370503	-2.506664	-2.329755	-0.136161	0.0407479	-0.047706	down
A_33_P3340990	AKTIP	1.0343056	1.101798	0.8713942	0.0674925	-0.162911	-0.047709	down
A_22_P00012788	lnc-RALGDS-1	-2.301348	-2.324985	-2.37323	-0.023638	-0.071882	-0.04776	down
A_23_P57401	LZTR1	0.5054851	0.4046512	0.5106545	-0.100834	0.0051694	-0.047832	down
A_32_P72341	TRIM59	2.6185894	2.5903955	2.5510998	-0.028194	-0.06749	-0.047842	down
A_33_P3286041	POLR3E	3.6410847	3.5996108	3.586833	-0.041474	-0.054252	-0.047863	down
A_33_P3396612	BMS1P20	4.5454273	4.390387	4.6046743	-0.15504	0.059247	-0.047897	down
A_23_P318300	ZAK	2.542715	2.5862794	2.403101	0.0435643	-0.139614	-0.048025	down
A_33_P3333317	OPTN	1.690383	1.8353877	1.4490628	0.1450048	-0.24132	-0.048158	down
A_19_P00327415	LOC101927405	-1.564869	-1.715471	-1.510654	-0.150602	0.0542159	-0.048193	down
A_23_P8196	ME1	0.8182087	1.0872054	0.4527736	0.2689967	-0.365435	-0.048219	down
A_23_P127079	PPRC1	4.735963	4.691956	4.6833773	-0.044007	-0.052586	-0.048296	down
A_23_P24135	TACR2	-1.474958	-1.3734	-1.673124	0.1015582	-0.198166	-0.048304	down
A_33_P3333587	FBXL20	4.542941	4.515653	4.4735804	-0.027288	-0.069361	-0.048324	down
A_24_P48318	CWC25	1.7908258	1.8108654	1.6741376	0.0200396	-0.116688	-0.048324	down
A_23_P52761	MMP7	-0.108109	0.1343603	-0.44737	0.2424693	-0.339261	-0.048396	down
A_23_P367816	SNX29	2.3143806	2.321363	2.210391	0.0069823	-0.10399	-0.048504	down
A_33_P3299487	AIM1L	-1.70335	-1.648759	-1.855169	0.0545912	-0.151819	-0.048614	down
A_33_P3216316	TRIM41	-2.471355	-2.56382	-2.476385	-0.092464	-0.005029	-0.048747	down
A_23_P32903	OCRL	3.0962238	2.8903413	3.2045374	-0.205883	0.1083136	-0.048784	down
A_33_P3298861	SLC12A8	-1.063168	-1.20583	-1.018119	-0.142662	0.0450487	-0.048806	down
A_23_P55123	COX10	-0.435291	-0.569488	-0.398777	-0.134197	0.0365133	-0.048842	down
A_23_P6624	UMPS	6.264473	6.1302495	6.3009415	-0.134223	0.0364685	-0.048877	down
A_33_P3233135	KIF16B	-0.065558	-0.385934	0.1569653	-0.320376	0.2225232	-0.048927	down
A_33_P3263193	RHNO1	3.0392027	2.8973298	3.083064	-0.141873	0.0438614	-0.049006	down
A_24_P377775	RGS3	-1.965941	-1.98206	-2.047916	-0.016119	-0.081975	-0.049047	down
A_23_P35609	UROS	2.4996452	2.3381963	2.5629349	-0.161449	0.0632896	-0.04908	down
A_33_P3417865	ZFP36L1	6.27094	6.156673	6.2870264	-0.114267	0.0160866	-0.04909	down
A_23_P363399	SLC38A1	5.584465	5.744575	5.326091	0.16011	-0.258374	-0.049132	down
A_23_P75083	ZMYND11	1.2621202	1.2066531	1.2192707	-0.055467	-0.04285	-0.049158	down
A_23_P144816	VDAC1	6.868438	6.9749885	6.663515	0.1065507	-0.204923	-0.049186	down
A_22_P00004758	LOC101927932	-2.592006	-2.192753	-3.089719	0.3992536	-0.497713	-0.04923	down
A_33_P3433156	TRMU	2.160427	1.9961858	2.2261915	-0.164241	0.0657644	-0.049238	down
A_22_P00008392	lnc-JMJD7-PLA2G4B-2	-1.779201	-1.763245	-1.893662	0.0159564	-0.11446	-0.049252	down
A_23_P343237	ANGEL2	-1.047946	-1.27049	-0.923918	-0.222544	0.1240277	-0.049258	down
A_33_P3217020	RAPH1	-1.845753	-1.924205	-1.865827	-0.078452	-0.020074	-0.049263	down
A_23_P418373	BCL2L2	2.53168	2.3623118	2.6024847	-0.169368	0.0708046	-0.049282	down
A_33_P3224055	LOC100132481	-2.09115	-2.146939	-2.133953	-0.055789	-0.042803	-0.049296	down

A_33_P3242136	FGF1	-2.131169	-2.161518	-2.199501	-0.030349	-0.068332	-0.049341	down
A_33_P3422466	MORN3	-2.007667	-1.827112	-2.287008	0.1805553	-0.279341	-0.049393	down
A_23_P53541	CHD4	3.456295	3.4355617	3.3781738	-0.020733	-0.078121	-0.049427	down
A_23_P400298	PRSS22	3.8184385	3.8547425	3.683259	0.036304	-0.13518	-0.049438	down
A_21_P0000592	SNORA70G	1.2446198	1.3245292	1.0657301	0.0799093	-0.17889	-0.04949	down
A_24_P100742	ADD1	-0.886001	-0.802443	-1.068548	0.0835581	-0.182547	-0.049495	down
A_33_P3350074	SLC25A19	5.762478	5.582933	5.8430223	-0.179545	0.0805445	-0.0495	down
A_23_P8801	CYP3A5	2.9844427	2.7135205	3.1562748	-0.270922	0.1718321	-0.049545	down
A_33_P3299386	ZNF550	-1.141974	-1.17228	-1.210803	-0.030307	-0.068829	-0.049568	down
A_23_P95302	RFC5	5.006795	4.844401	5.069936	-0.162394	0.0631409	-0.049627	down
A_21_P0005184	lnc-WDR27-1	-2.726555	-2.575781	-2.976643	0.1507735	-0.250088	-0.049657	down
A_33_P3228450	FXYD3	4.1153927	3.7036338	4.4278183	-0.411759	0.3124256	-0.049667	down
A_24_P365365	TCF3	4.0557413	3.7835097	4.2285814	-0.272232	0.1728401	-0.049696	down
A_33_P3338909	ARPC5	6.5174465	6.270165	6.665016	-0.247282	0.1475697	-0.049856	down
A_33_P3228609	LINC01123	-0.650889	-0.651701	-0.749836	-0.000813	-0.098948	-0.04988	down
A_23_P12620	TNKS2	-0.006425	0.0126896	-0.125326	0.0191145	-0.118901	-0.049893	down
A_22_P00005233	LOC154449	-0.941103	-1.197005	-0.785126	-0.255902	0.1559773	-0.049962	down
A_23_P407992	CRAMP1L	-2.488101	-2.409433	-2.666693	0.0786676	-0.178593	-0.049963	down
A_24_P122050	PSMF1	2.6162062	2.3850236	2.7473545	-0.231183	0.1311483	-0.050017	down
A_22_P00015390	lnc-SRGAP3-1	1.0664287	1.1290703	0.9036932	0.0626416	-0.162735	-0.050047	down
A_19_P00806499	SPG20-AS1	-2.863849	-2.900344	-2.927495	-0.036495	-0.063646	-0.050071	down
A_33_P3393170	CAPN5	3.569868	3.366075	3.6734543	-0.203793	0.1035862	-0.050103	down
A_23_P23411	PRCC	0.5770249	0.5468259	0.506978	-0.030199	-0.070047	-0.050123	down
A_23_P110076	WDR53	1.8755941	1.6762619	1.9746399	-0.199332	0.0990458	-0.050143	down
A_32_P56249	USP30-AS1	-1.678162	-1.926866	-1.529813	-0.248704	0.1483488	-0.050178	down
A_32_P45738	PGAM1	6.1523256	5.9251027	6.27917	-0.227223	0.1268444	-0.050189	down
A_22_P00017991	CARD8-AS1	0.5254254	0.4760265	0.4744263	-0.049399	-0.050999	-0.050199	down
A_23_P218317	NARF	3.695383	3.636541	3.6537752	-0.058842	-0.041608	-0.050225	down
A_33_P3340847	CARD6	0.0628548	-0.015728	0.0405698	-0.078582	-0.022285	-0.050434	down
A_23_P105957	ACTN1	4.7264214	4.5832047	4.768606	-0.143217	0.0421848	-0.050516	down
A_23_P166826	DCP1A	3.6652136	3.561047	3.6683369	-0.104167	0.0031233	-0.050522	down
A_24_P42066	GGT3P	-2.170041	-2.28609	-2.15529	-0.116049	0.0147505	-0.050649	down
A_23_P71752	ZFAND5	5.269409	5.2348123	5.2027016	-0.034597	-0.066708	-0.050652	down
A_21_P0013268	XLOC_l2_013594	0.7090511	0.7153459	0.6013732	0.0062947	-0.107678	-0.050692	down
A_22_P00010460	lnc-NADSYN1-1	0.0868855	-0.071993	0.1443701	-0.158879	0.0574846	-0.050697	down
A_33_P3264272	SYF2	-1.876132	-2.085384	-1.768352	-0.209253	0.1077795	-0.050737	down
A_24_P303524	MICALL2	3.7842941	3.6370797	3.8297548	-0.147214	0.0454607	-0.050877	down
A_23_P54556	MKL2	2.910574	2.7649283	2.9544096	-0.145646	0.0438356	-0.050905	down
A_33_P3283136	TMEM209	2.283945	2.1936955	2.2722015	-0.09025	-0.011744	-0.050997	down
A_33_P3321611	PHACTR4	-2.233037	-2.329203	-2.238996	-0.096166	-0.005959	-0.051062	down
A_23_P101407	C3	-2.30163	-2.219131	-2.486307	0.0824986	-0.184677	-0.051089	down
A_22_P00004478	LOC100507250	-2.952122	-3.160461	-2.846136	-0.208338	0.1059864	-0.051176	down
A_24_P932418	AP2A2	2.1710672	1.7214661	2.5182838	-0.449601	0.3472166	-0.051192	down
A_23_P150852	ESYT1	2.0260706	2.0855412	1.8641834	0.0594707	-0.161887	-0.051208	down
A_23_P110606	MAML1	0.6361876	0.7153549	0.4543667	0.0791674	-0.181821	-0.051327	down
A_33_P3386532	MRPL38	-0.864846	-0.812514	-1.019886	0.0523324	-0.155039	-0.051353	down
A_33_P3277140	MINOS1	6.8693886	6.7305512	6.9053364	-0.138837	0.0359478	-0.051445	down
A_24_P127021	ST13	3.723648	3.567473	3.7769108	-0.156175	0.0532627	-0.051456	down
A_23_P50418	ZNF791	1.1654105	1.2641597	0.9635916	0.0987492	-0.201819	-0.051535	down
A_23_P335905	STK36	-0.677373	-0.693549	-0.764307	-0.016176	-0.086934	-0.051555	down
A_24_P335901	ZNF266	0.7755942	0.7007818	0.7472863	-0.074812	-0.028308	-0.05156	down
A_23_P124760	MED24	3.052908	2.8847222	3.1177511	-0.168186	0.0648432	-0.051671	down
A_33_P3209356	RFWD2	2.6292849	2.5808616	2.574254	-0.048423	-0.055031	-0.051727	down
A_33_P3263902	MXI1	-2.131066	-2.204227	-2.161522	-0.073161	-0.030456	-0.051809	down
A_33_P3416124	LOC100130872	-2.642297	-2.659673	-2.728551	-0.017376	-0.086254	-0.051815	down
A_23_P89570	ZMYND15	0.260211	0.1438437	0.2729197	-0.116367	0.0127087	-0.051829	down

A_33_P3342613	ZDHHC8	5.6804476	5.52041	5.7368126	-0.160038	0.056365	-0.051836	down
A_24_P920979	ASB7	0.3905835	0.3778777	0.2995567	-0.012706	-0.091027	-0.051866	down
A_33_P3237699	RMND5A	1.6573524	1.5948267	1.6160188	-0.062526	-0.041334	-0.05193	down
A_23_P204158	RNFT2	1.1979098	1.0087118	1.2832069	-0.189198	0.0852971	-0.05195	down
A_22_P00012212	PRR34-AS1	4.6927786	4.5408645	4.740761	-0.151914	0.0479822	-0.051966	down
A_33_P3784253	PAK1	3.5133667	3.4513078	3.4710598	-0.062059	-0.042307	-0.052183	down
A_24_P338187	HNRNPH3	3.7578049	3.7397695	3.671399	-0.018035	-0.086406	-0.052221	down
A_33_P3275948	STARD3NL	1.9764385	1.699997	2.148405	-0.276442	0.1719666	-0.052238	down
A_22_P00013897	lnc-RPS21-2	-0.965172	-0.981157	-1.053845	-0.015985	-0.088673	-0.052329	down
A_21_P0005594	lnc-GNA12-1	2.7851925	2.5142264	2.9513721	-0.270966	0.1661797	-0.052393	down
A_23_P140256	PNP	6.1609964	5.792789	6.423851	-0.368207	0.2628546	-0.052676	down
A_21_P0004468	lnc-FBXW11-2	3.0186405	2.8458314	3.0859375	-0.172809	0.067297	-0.052756	down
A_21_P0002045	LOC100130502	0.9668112	1.0743198	0.753768	0.1075087	-0.213043	-0.052767	down
A_23_P217009	C9orf24	-1.625933	-1.845167	-1.512236	-0.219234	0.1136975	-0.052768	down
A_24_P603224	SF3A3	4.870969	4.6273685	5.009021	-0.2436	0.138052	-0.052774	down
A_33_P3214501	AEN	0.586412	0.3929763	0.6742411	-0.193436	0.0878291	-0.052803	down
A_33_P3234864	UTRN	0.3208351	0.2233276	0.3124857	-0.097507	-0.008349	-0.052928	down
A_23_P161488	TIAL1	3.1176949	3.152536	2.9769316	0.0348411	-0.140763	-0.052961	down
A_24_P934563	MTCH2	1.6350403	1.6793427	1.4847765	0.0443025	-0.150264	-0.052981	down
A_23_P163580	CENPT	-1.846349	-1.724812	-2.073941	0.1215372	-0.227592	-0.053027	down
A_23_P2801	ELF1	0.7028222	0.6368218	0.6627035	-0.066	-0.040119	-0.05306	down
A_23_P318284	GPD1L	0.7183476	0.6754751	0.6550994	-0.042872	-0.063248	-0.05306	down
A_22_P00010205	MSH3	-0.354604	-0.365773	-0.449722	-0.011169	-0.095118	-0.053144	down
A_24_P414256	TMA7	5.881936	5.627731	6.029793	-0.254205	0.1478567	-0.053174	down
A_32_P187617	TDRD3	-1.312583	-1.307062	-1.424472	0.0055218	-0.111889	-0.053184	down
A_24_P117942	TOMM20L	0.8519635	0.8133116	0.784215	-0.038652	-0.067749	-0.0532	down
A_33_P3236157	UNG	6.787184	6.7045627	6.7633495	-0.082621	-0.023834	-0.053228	down
A_33_P3289745	LOC399900	-2.014114	-1.708246	-2.426699	0.3058682	-0.412586	-0.053359	down
A_23_P129064	GATM	3.005332	2.7738118	3.1301165	-0.23152	0.1247845	-0.053368	down
A_22_P00003818	COX10-AS1	-2.064956	-2.563832	-1.672862	-0.498877	0.3920941	-0.053391	down
A_24_P861009	BRWD1	0.9384217	0.8836346	0.8863678	-0.054787	-0.052054	-0.053421	down
A_22_P00005150	lnc-DHX38-5	0.3478603	0.2304974	0.3582883	-0.117363	0.010428	-0.053468	down
A_23_P209619	ATL2	4.919862	4.9346204	4.798046	0.0147586	-0.121816	-0.053529	down
A_33_P3335391	FAM83G	-2.694529	-2.460582	-3.035646	0.2339475	-0.341117	-0.053585	down
A_33_P3388482	TMEM120B	-1.942457	-2.200548	-1.79168	-0.258091	0.1507769	-0.053657	down
A_33_P3386237	TAT-AS1	-0.935663	-1.004005	-0.974724	-0.068342	-0.039062	-0.053702	down
A_24_P245815	ASPHD2	-0.473122	-0.587058	-0.466736	-0.113935	0.0063863	-0.053775	down
A_33_P3389153	STK10	1.7469759	1.4993281	1.8869982	-0.247648	0.1400223	-0.053813	down
A_24_P694760	ERC1	1.1657891	1.1601086	1.0636497	-0.005681	-0.102139	-0.05391	down
A_33_P3218252	SMG1	3.6443396	3.5648637	3.6154957	-0.079476	-0.028844	-0.05416	down
A_23_P146284	SQLE	5.962264	5.494307	6.321804	-0.467957	0.35954	-0.054209	down
A_33_P3310780	CTTN	6.3016653	6.169991	6.3248653	-0.131674	0.0232	-0.054237	down
A_33_P3317168	EPS8L3	3.823905	3.6115594	3.927351	-0.212346	0.103446	-0.05445	down
A_32_P103614	NEBL-AS1	-1.662063	-1.565604	-1.867521	0.0964589	-0.205458	-0.0545	down
A_33_P3271105	PABPC4	-1.392848	-1.358522	-1.536172	0.0343256	-0.143325	-0.0545	down
A_23_P310022	KIAA1217	1.7895136	1.8259454	1.6438675	0.0364318	-0.145646	-0.054607	down
A_33_P3318414	HMHA1	3.7536058	3.8590589	3.538725	0.105453	-0.214881	-0.054714	down
A_21_P0000320	SNORA30	0.1650071	-0.095004	0.3154192	-0.260011	0.1504121	-0.054799	down
A_23_P379475	DHCR24	5.6352816	5.195416	5.9653845	-0.439866	0.3301029	-0.054881	down
A_33_P3235370	ZNF250	-1.180347	-1.146362	-1.324143	0.0339847	-0.143797	-0.054906	down
A_23_P113283	ZMAT3	-1.22305	-1.435257	-1.120685	-0.212206	0.1023655	-0.05492	down
A_24_P134195	MYADM	0.397943	0.4033175	0.2824059	0.0053744	-0.115537	-0.055081	down
A_23_P113803	KATNA1	3.4275723	3.323874	3.4210014	-0.103698	-0.006571	-0.055135	down
A_19_P00322260	LOC101929709	-0.794859	-0.640379	-1.059657	0.15448	-0.264797	-0.055159	down
A_23_P43504	ABCA2	0.194056	-0.008897	0.286634	-0.202953	0.0925779	-0.055187	down
A_23_P387471	MICB	-1.821516	-1.986287	-1.76715	-0.164772	0.0543656	-0.055203	down

A_23_P26336	ZC3H7A	2.811246	2.834375	2.6776905	0.023129	-0.133555	-0.055213	down
A_23_P94118	GTF2E2	2.4737272	2.4243073	2.412609	-0.04942	-0.061118	-0.055269	down
A_23_P354387	MYOF	6.2037783	6.17469	6.122322	-0.029088	-0.081456	-0.055272	down
A_23_P89056	NUP93	3.1132383	3.046453	3.0694609	-0.066785	-0.043777	-0.055281	down
A_21_P0014261	LOC102723539	-1.724753	-1.662931	-1.897209	0.0618219	-0.172455	-0.055317	down
A_23_P426809	ARHGEF11	-0.502965	-0.52482	-0.591757	-0.021855	-0.088791	-0.055323	down
A_33_P3217534	RBM33	-2.210342	-2.226353	-2.305101	-0.016011	-0.094759	-0.055385	down
A_23_P501547	ADCY6	1.4467893	1.2996373	1.4830337	-0.147152	0.0362444	-0.055454	down
A_22_P00021565	SNORA78	6.4965305	6.094957	6.7871532	-0.401574	0.2906227	-0.055475	down
A_23_P120170	TIGD1	1.7927494	1.6322927	1.8422394	-0.160457	0.04949	-0.055483	down
A_23_P44932	EIF2A	3.2307892	3.1631646	3.187334	-0.067625	-0.043455	-0.05554	down
A_24_P100277	CYB5R3	0.5849447	0.1918736	0.8669281	-0.393071	0.2819834	-0.055544	down
A_32_P167791	ST13	4.010111	3.8236165	4.085415	-0.186494	0.075304	-0.055595	down
A_24_P142743	CNN2	4.028185	3.6513867	4.293749	-0.376798	0.265564	-0.055617	down
A_33_P3245631	TTC39A	-2.336551	-2.747309	-2.03705	-0.410758	0.2995009	-0.055629	down
A_23_P165402	SF3B6	6.6646547	6.5155845	6.70222	-0.14907	0.0375652	-0.055753	down
A_23_P206661	NQO1	3.2108717	3.3161368	2.994073	0.1052651	-0.216799	-0.055767	down
A_33_P3375145	LURAP1L	-0.194398	0.113852	-0.614224	0.3082504	-0.419826	-0.055788	down
A_32_P43465	ZNF182	0.4361281	0.3821316	0.3784809	-0.053997	-0.057647	-0.055822	down
A_24_P99838	ZNF223	0.1408901	0.0861216	0.0839977	-0.054769	-0.056892	-0.05583	down
A_33_P3302115	CAPRIN1	-0.698846	-0.799805	-0.709569	-0.100958	-0.010723	-0.055841	down
A_33_P3272461	METTL5	4.852085	4.7009645	4.8915167	-0.151121	0.0394316	-0.055845	down
A_21_P0010703	lnc-C1orf177-2	-1.255312	-1.566458	-1.056026	-0.311146	0.1992865	-0.05593	down
A_23_P90099	TMEM205	4.5140867	4.6479335	4.2683764	0.1338468	-0.24571	-0.055932	down
A_23_P167081	REST	1.0316825	0.9840875	0.9672184	-0.047595	-0.064464	-0.05603	down
A_24_P927189	OXNAD1	0.9108386	0.8944688	0.8151121	-0.01637	-0.095726	-0.056048	down
A_24_P175187	SAMD9	0.095499	0.0069752	0.0714612	-0.088524	-0.024038	-0.056281	down
A_33_P3399618	KLHL33	-1.812783	-1.797594	-1.940591	0.0151896	-0.127808	-0.056309	down
A_23_P27180	NSRP1	1.5739594	1.5598192	1.4753866	-0.01414	-0.098573	-0.056356	down
A_21_P0003715	lnc-CYTL1-1	-1.708553	-1.892509	-1.637358	-0.183956	0.0711956	-0.05638	down
A_22_P00001222	NDUFV2	3.79873	3.7922368	3.6922998	-0.006493	-0.10643	-0.056462	down
A_33_P3462960	DNAJC3	2.4506063	2.425919	2.3621883	-0.024687	-0.088418	-0.056553	down
A_33_P3283300	FAM204A	-0.594654	-0.38531	-0.917378	0.2093444	-0.322724	-0.05669	down
A_22_P00017079	lnc-TUSC5-2	-2.692643	-3.034231	-2.464531	-0.341588	0.2281127	-0.056738	down
A_23_P39718	FEZ2	2.3168364	2.4524941	2.067668	0.1356578	-0.249168	-0.056755	down
A_23_P163697	SYT17	-1.155759	-1.108276	-1.316815	0.047483	-0.161056	-0.056787	down
A_23_P366230	LATS1	-1.871991	-2.164684	-1.692942	-0.292693	0.1790485	-0.056822	down
A_23_P60499	ZNF462	3.1157522	3.0513587	3.0664492	-0.064394	-0.049303	-0.056848	down
A_33_P3309319	PIDD1	2.9445114	2.6966624	3.0786371	-0.247849	0.1341257	-0.056862	down
A_24_P95029	TAX1BP1	4.2864037	4.291985	4.1670284	0.0055814	-0.119375	-0.056897	down
A_23_P161507	MTL5	-0.309093	-0.342998	-0.388994	-0.033905	-0.079901	-0.056903	down
A_23_P165598	DAPL1	-0.919686	-1.458389	-0.494817	-0.538703	0.4248695	-0.056917	down
A_23_P150365	REXO2	6.5475807	6.604359	6.37679	0.0567784	-0.170791	-0.057006	down
A_24_P352116	SNHG7	1.5444956	1.3873281	1.587501	-0.157167	0.0430055	-0.057081	down
A_33_P3288074	lnc-ATG2B-2	-1.829959	-2.012019	-1.762106	-0.18206	0.0678535	-0.057103	down
A_22_P00014053	DGKD	0.6595016	0.703352	0.5014286	0.0438504	-0.158073	-0.057111	down
A_24_P389608	PROSER2	-1.895569	-2.172598	-1.733136	-0.277029	0.1624332	-0.057298	down
A_23_P396867	HM13	-0.014817	-0.164395	0.0201478	-0.149578	0.0349646	-0.057307	down
A_32_P51084	NUP205	4.4277306	4.360905	4.3798113	-0.066825	-0.047919	-0.057372	down
A_23_P250644	CDKN2AIPNL	2.161378	2.0558467	2.1521559	-0.105531	-0.009222	-0.057377	down
A_21_P0000666	LOC100129361	0.6770868	0.4017482	0.8376093	-0.275339	0.1605225	-0.057408	down
A_21_P0011538	XLOC_l2_005731	-1.645722	-1.766371	-1.64005	-0.120649	0.005672	-0.057489	down
A_23_P201066	SUSD4	-1.148033	-1.578547	-0.832498	-0.430514	0.3155346	-0.05749	down
A_33_P3324786	TCFL5	0.4255772	0.2961955	0.4395981	-0.129382	0.0140209	-0.05768	down
A_23_P366394	ZAK	0.0644927	0.0352535	-0.021799	-0.029239	-0.086292	-0.057765	down
A_21_P0011175	CASC4	-0.169184	-0.264594	-0.189557	-0.09541	-0.020373	-0.057892	down

A_33_P3404651	TTC7A	2.224761	2.053141	2.2804356	-0.17162	0.0556746	-0.057973	down
A_22_P00017308	MIR99AHG	1.0329642	1.0538435	0.8961372	0.0208793	-0.136827	-0.057974	down
A_21_P0013723	LOC102724238	2.407875	2.529986	2.169711	0.1221108	-0.238164	-0.058027	down
A_33_P3253807	CEBPG	4.620139	4.6214414	4.502657	0.0013022	-0.117482	-0.05809	down
A_21_P0000617	LIMD1-AS1	-2.446811	-2.438926	-2.570929	0.0078845	-0.124118	-0.058117	down
A_23_P145965	TPST1	1.1121607	1.1049232	1.003056	-0.007237	-0.109105	-0.058171	down
A_21_P0011780	LINC01534	-2.509178	-2.753623	-2.381213	-0.244445	0.1279652	-0.05824	down
A_24_P397247	SDAD1	2.0828705	1.9908137	2.0583	-0.092057	-0.02457	-0.058314	down
A_22_P00008145	lnc-IL6-2	-2.43003	-2.556625	-2.420186	-0.126595	0.0098438	-0.058376	down
A_23_P251303	SEC16A	2.6586971	2.5725818	2.6280556	-0.086115	-0.030642	-0.058378	down
A_33_P3363168	SSH2	2.2717657	2.2304826	2.1962252	-0.041283	-0.075541	-0.058412	down
A_23_P94754	TNFSF15	-0.685046	-0.619374	-0.867569	0.0656714	-0.182523	-0.058426	down
A_23_P58521	ERCC8	0.2335963	0.034276	0.3159337	-0.19932	0.0823374	-0.058491	down
A_33_P3321711	TOMM34	3.430749	3.330565	3.4138088	-0.100184	-0.01694	-0.058562	down
A_23_P131954	SNX5	5.7163506	5.787997	5.527424	0.0716462	-0.188927	-0.05864	down
A_23_P105664	CCDC59	3.6698313	3.4805617	3.7416916	-0.18927	0.0718603	-0.058705	down
A_23_P433188	GZF1	0.771337	0.7030702	0.7221513	-0.068267	-0.049186	-0.058726	down
A_23_P65041	RACGAP1P	-0.521266	-0.879911	-0.280203	-0.358645	0.2410631	-0.058791	down
A_23_P144476	SPRY1	0.3741412	0.2498894	0.3807349	-0.124252	0.0065937	-0.058829	down
A_33_P3246580	KIAA1244	-1.078721	-0.933404	-1.341847	0.1453161	-0.263126	-0.058905	down
A_22_P00007424	UBXN8	-1.353321	-1.317195	-1.507377	0.0361257	-0.154056	-0.058965	down
A_23_P320578	RGS16	2.9503336	2.6531596	3.129507	-0.297174	0.1791735	-0.059	down
A_33_P3312384	MIR99AHG	-2.276075	-2.241615	-2.428673	0.0344608	-0.152598	-0.059068	down
A_24_P33156	AFMID	-0.519258	-0.565106	-0.591574	-0.045849	-0.072316	-0.059083	down
A_21_P0000256	SNORD28	-0.813921	-0.888386	-0.857778	-0.074465	-0.043857	-0.059161	down
A_23_P301995	LIN9	-0.014919	-0.029745	-0.118544	-0.014826	-0.103625	-0.059226	down
A_22_P00020910	LOC100507564	-2.151115	-2.231123	-2.189585	-0.080007	-0.03847	-0.059238	down
A_21_P0000208	SNORD22	0.8076181	0.7000775	0.7966213	-0.107541	-0.010997	-0.059269	down
A_23_P353056	C2CD2L	3.0616302	3.0077987	2.996911	-0.053832	-0.064719	-0.059275	down
A_24_P922252	SPATA5L1	-2.327813	-2.506748	-2.267577	-0.178935	0.060236	-0.05935	down
A_33_P3282394	MLLT1	4.4976044	4.2692933	4.607005	-0.228311	0.1094008	-0.059455	down
A_23_P399146	ZNF549	-1.659176	-1.767695	-1.669593	-0.108519	-0.010417	-0.059468	down
A_23_P257905	CENPB	2.1954613	2.1584926	2.1134357	-0.036969	-0.082026	-0.059497	down
A_23_P65357	SYNJ2BP	2.6935396	2.5084357	2.7595873	-0.185104	0.0660477	-0.059528	down
A_23_P53567	CCDC53	3.3105154	3.3436885	3.158206	0.0331731	-0.152309	-0.059568	down
A_22_P00010329	lnc-MYB-1	-1.899132	-2.6775	-1.239932	-0.778368	0.6592002	-0.059584	down
A_23_P52058	TARBP1	1.0404449	0.9676819	0.9940271	-0.072763	-0.046418	-0.05959	down
A_21_P0011403	GOLGA8IP	0.3548145	0.3577542	0.2324653	0.0029397	-0.122349	-0.059705	down
A_23_P103631	EBNA1BP2	7.2985353	7.1350913	7.3425474	-0.163444	0.0440121	-0.059716	down
A_33_P3220422	POM121L12	-1.28909	-1.408659	-1.289158	-0.119569	-6.82E-05	-0.059819	down
A_23_P76918	TIMM9	5.481654	5.490311	5.353097	0.008657	-0.128557	-0.05995	down
A_33_P3289145	COPS8	2.844347	2.683311	2.8854275	-0.161036	0.0410805	-0.059978	down
A_33_P3376017	lnc-KIAA0319L-1	-0.241645	-0.503511	-0.099765	-0.261867	0.1418796	-0.059994	down
A_24_P22079	FOXO1	0.9632883	1.0263577	0.7802258	0.0630693	-0.183063	-0.059997	down
A_33_P3220475	FLJ23867	0.7540736	0.7024365	0.6856055	-0.051637	-0.068468	-0.060053	down
A_33_P3343432	THEM4	0.6884728	0.5655813	0.6911936	-0.122891	0.0027208	-0.060085	down
A_23_P76882	CCNB1IP1	2.878378	2.5261235	3.1103868	-0.352254	0.2320089	-0.060123	down
A_24_P235305	ZNF706	0.2283101	0.3021321	0.0341992	0.073822	-0.194111	-0.060144	down
A_22_P00020201	LINC01585	-1.581842	-1.711191	-1.572804	-0.129349	0.009038	-0.060155	down
A_21_P0005650	lnc-ERI1-1	-2.309774	-2.554261	-2.185619	-0.244487	0.124155	-0.060166	down
A_22_P00010710	lnc-NFYB-1	-2.787319	-2.130838	-3.564188	0.6564803	-0.776869	-0.060194	down
A_23_P369666	ZMYND8	0.4699163	0.4109621	0.4084344	-0.058954	-0.061482	-0.060218	down
A_23_P162589	VDR	4.6560106	4.654038	4.5375147	-0.001973	-0.118496	-0.060234	down
A_24_P277657	GMPR	-0.885989	-1.033417	-0.859092	-0.147428	0.0268974	-0.060265	down
A_23_P21548	ARHGAP10	1.072649	0.8900805	1.1344485	-0.182569	0.0617995	-0.060385	down
A_23_P426472	ZNF45	0.0817523	0.1083002	-0.065578	0.0265479	-0.14733	-0.060391	down

A_22_P00008116	lnc-IL23A-2	0.6113954	0.3731022	0.728827	-0.238293	0.1174316	-0.060431	down
A_33_P3279431	GOSR1	1.9708748	1.7674308	2.0534334	-0.203444	0.0825586	-0.060443	down
A_23_P145247	ZSCAN26	-0.78034	-0.883415	-0.798256	-0.103075	-0.017916	-0.060496	down
A_21_P0007039	LOC102725247	-2.318477	-2.57607	-2.181952	-0.257593	0.1365247	-0.060534	down
A_24_P289170	CCDC88C	-2.488514	-2.460025	-2.638103	0.0284882	-0.14959	-0.060551	down
A_23_P4400	KRTAP4-11	0.2568665	0.142838	0.2497754	-0.114028	-0.007091	-0.06056	down
A_23_P359636	BROX	0.2854395	0.1803908	0.2693238	-0.105049	-0.016116	-0.060582	down
A_23_P92499	TLR2	0.1539617	-0.217502	0.4041834	-0.371464	0.2502217	-0.060621	down
A_23_P251342	GSK3B	2.6190548	2.493671	2.623084	-0.125384	0.0040293	-0.060677	down
A_23_P77103	SORD	3.8059893	3.6121974	3.878296	-0.193792	0.0723066	-0.060743	down
A_24_P724040	SNRPB2	3.0021505	2.8686957	3.0140953	-0.133455	0.0119448	-0.060755	down
A_23_P147431	LYN	2.6815987	2.5543227	2.687356	-0.127276	0.0057573	-0.060759	down
A_33_P3309289	ST3GAL4	-1.570297	-1.14506	-2.117075	0.4252367	-0.546779	-0.060771	down
A_33_P3325131	CANX	3.7040863	3.6388087	3.6477995	-0.065278	-0.056287	-0.060782	down
A_33_P3395146	LOC646762	2.9461498	2.966227	2.804347	0.0200772	-0.141803	-0.060863	down
A_33_P3369419	GON4L	-1.547027	-1.562437	-1.6534	-0.01541	-0.106372	-0.060891	down
A_24_P108451	GPI	7.5146465	7.274058	7.6334467	-0.240589	0.1188002	-0.060894	down
A_33_P3358957	PAPL	0.5172448	0.432282	0.4804092	-0.084963	-0.036836	-0.060899	down
A_22_P00018583	LOC145845	-1.891981	-2.11896	-1.787254	-0.226979	0.1047273	-0.061126	down
A_24_P385134	SCD5	1.6010141	1.5190864	1.5605955	-0.081928	-0.040419	-0.061173	down
A_23_P110941	GSTA4	1.884655	1.9365096	1.7102809	0.0518546	-0.174374	-0.06126	down
A_33_P3369239	QKI	0.9296198	0.8819661	0.8547521	-0.047654	-0.074868	-0.061261	down
A_23_P66948	GAREM	0.3062611	0.3572264	0.1327415	0.0509653	-0.17352	-0.061277	down
A_32_P219279	ELFN2	-0.094308	-0.316569	0.0053916	-0.22226	0.0997	-0.06128	down
A_23_P85640	INPP5B	-0.556532	-0.568248	-0.667458	-0.011715	-0.110925	-0.06132	down
A_23_P158570	ACADSB	-0.750459	-0.997243	-0.626333	-0.246784	0.1241255	-0.061329	down
A_33_P3262665	MAP7D3	0.6190915	0.2970333	0.8184876	-0.322058	0.1993961	-0.061331	down
A_33_P3369401	CYP24A1	-0.31043	-0.547644	-0.195957	-0.237214	0.1144729	-0.061371	down
A_23_P406227	SNAI3-AS1	-0.461818	-0.486578	-0.559865	-0.024759	-0.098046	-0.061403	down
A_21_P0005621	LOC101927815	0.081224	-0.028857	0.0683227	-0.110081	-0.012901	-0.061491	down
A_22_P00008601	KDM4C	-2.109026	-2.062018	-2.279155	0.0470076	-0.170129	-0.061561	down
A_21_P0000753	DLG1-AS1	-1.129065	-1.265976	-1.115293	-0.136911	0.0137725	-0.061569	down
A_24_P276816	RCOR3	1.2952437	1.5269885	0.9402733	0.2317448	-0.35497	-0.061613	down
A_23_P209652	INO80D	-1.711095	-1.869999	-1.675615	-0.158904	0.03548	-0.061712	down
A_24_P316102	MLLT10	1.5069795	1.6067514	1.2836022	0.099772	-0.223377	-0.061803	down
A_22_P00008132	ASMTL-AS1	-2.163577	-2.302608	-2.148404	-0.139031	0.0151725	-0.061929	down
A_24_P139191	ITCH	-0.675299	-0.767314	-0.707241	-0.092015	-0.031942	-0.061978	down
A_23_P14458	L3HYPDH	-0.042013	-0.196766	-0.011504	-0.154753	0.0305085	-0.062122	down
A_33_P3259183	FAM78B	-0.005723	-0.444927	0.3092222	-0.439203	0.3149457	-0.062129	down
A_23_P102582	C20orf24	7.3669577	7.1956906	7.41389	-0.171267	0.0469322	-0.062167	down
A_23_P49448	FA2H	4.393758	4.3652673	4.297901	-0.028491	-0.095857	-0.062174	down
A_33_P3257330	DCBLD1	-0.321286	-0.43973	-0.32719	-0.118444	-0.005904	-0.062174	down
A_24_P53282	CPD	0.1712451	0.2325411	-0.014568	0.061296	-0.185813	-0.062258	down
A_23_P58396	PDGFC	3.791483	3.8230214	3.6354074	0.0315385	-0.156075	-0.062268	down
A_23_P131449	C2orf81	-0.178787	-0.230639	-0.251515	-0.051852	-0.072728	-0.06229	down
A_32_P42964	NUPR1L	-0.81764	-1.070644	-0.689219	-0.253004	0.1284213	-0.062291	down
A_23_P355385	PPP6C	4.8206053	4.6445775	4.872018	-0.176028	0.0514126	-0.062308	down
A_21_P0000011	AMDHD2	0.4259367	0.5052161	0.221684	0.0792794	-0.204253	-0.062487	down
A_24_P921366	CALD1	2.456954	2.4880033	2.300766	0.0310493	-0.156188	-0.062569	down
A_23_P200560	CDC42	5.2474356	5.3950553	4.9745665	0.1476197	-0.272869	-0.062625	down
A_33_P3241299	VPS9D1-AS1	-2.431077	-2.697968	-2.289607	-0.266891	0.14147	-0.062711	down
A_23_P31135	ACAT2	5.9466496	5.5225854	6.245228	-0.424064	0.2985783	-0.062743	down
A_23_P109895	SLC26A6	-0.099731	-0.247232	-0.077894	-0.147501	0.0218372	-0.062832	down
A_24_P354715	NT5E	0.543921	1.0474968	-0.085372	0.5035758	-0.629293	-0.062859	down
A_23_P49459	LOC81691	-0.125222	0.1178308	-0.494214	0.243053	-0.368992	-0.062969	down
A_33_P3325087	NSRP1	0.3107982	0.16148	0.3340554	-0.149318	0.0232573	-0.06303	down

A_24_P54485	CCDC115	-0.191926	-0.241933	-0.268034	-0.050007	-0.076108	-0.063057	down
A_33_P3379801	DNAAF2	0.0224361	0.0026522	-0.084147	-0.019784	-0.106584	-0.063184	down
A_24_P369898	MYO15B	-0.181753	0.1098886	-0.60022	0.2916412	-0.418467	-0.063413	down
A_23_P160537	AUNIP	2.6411467	2.6528974	2.502489	0.0117507	-0.138658	-0.063453	down
A_23_P51646	PLK3	2.6911383	2.5553231	2.6999865	-0.135815	0.0088482	-0.063483	down
A_33_P3404922	KIAA1217	-1.491422	-1.347255	-1.762658	0.1441665	-0.271236	-0.063535	down
A_22_P00015633	lnc-SUMO1-1	0.0393662	-0.149476	0.1010175	-0.188842	0.0616512	-0.063595	down
A_23_P55880	ZNF564	0.3525677	0.413156	0.1647487	0.0605884	-0.187819	-0.063615	down
A_32_P120895	LYSMD2	3.6056013	3.2843518	3.799611	-0.321249	0.1940098	-0.06362	down
A_33_P3414669	RLIM	-0.242353	-0.433774	-0.178204	-0.191421	0.0641499	-0.063635	down
A_24_P355267	SLC25A25	3.2435827	3.2237115	3.136076	-0.019871	-0.107507	-0.063689	down
A_23_P90333	ZNF404	-2.102819	-1.963511	-2.36972	0.1393075	-0.266902	-0.063797	down
A_21_P0005787	LOC101927815	-0.613799	-0.413628	-0.941581	0.200171	-0.327782	-0.063806	down
A_22_P00000167	DNMBP-AS1	-2.988109	-2.866131	-3.237729	0.1219788	-0.24962	-0.063821	down
A_22_P00023378	TSPAN33	-2.687247	-2.921676	-2.580486	-0.23443	0.1067607	-0.063835	down
A_24_P233960	SNX27	2.1123743	1.8828173	2.2141342	-0.229557	0.1017599	-0.063899	down
A_23_P121926	SEPP1	2.3887281	2.7021184	1.9474936	0.3133903	-0.441235	-0.063922	down
A_23_P107513	CEP76	1.5107293	1.5016599	1.39188	-0.009069	-0.118849	-0.063959	down
A_23_P153640	ANKRD27	2.657589	2.676207	2.511012	0.0186181	-0.146577	-0.063979	down
A_22_P00000684	FANK1-AS1	-2.895628	-3.143037	-2.776242	-0.247409	0.1193864	-0.064011	down
A_23_P501538	HOXA3	1.2383189	1.3477888	1.0006304	0.1094699	-0.237689	-0.064109	down
A_23_P61778	NXN	-1.49964	-1.692859	-1.43465	-0.193219	0.06499	-0.064115	down
A_23_P123539	PPP2R2A	2.9251614	2.791243	2.9308395	-0.133918	0.0056782	-0.06412	down
A_21_P0007135	lnc-NAALADL1-1	-3.073836	-3.256984	-3.018942	-0.183147	0.0548937	-0.064127	down
A_21_P0000512	SNAR-I	-2.899177	-2.827597	-3.099197	0.0715807	-0.20002	-0.06422	down
A_33_P3291394	GPN1	3.2696428	3.1286006	3.2821617	-0.141042	0.0125189	-0.064262	down
A_24_P876522	GPX8	2.2160254	2.2342649	2.0690145	0.0182395	-0.147011	-0.064386	down
A_23_P93431	AIG1	4.2121325	4.116874	4.1785574	-0.095258	-0.033575	-0.064417	down
A_23_P328237	SPTY2D1	-1.237566	-1.536675	-1.067293	-0.299109	0.1702728	-0.064418	down
A_23_P74344	TADA1	1.0120482	1.0208521	0.8743868	0.0088038	-0.137661	-0.064429	down
A_23_P342751	LINC01198	-0.419641	-0.635828	-0.332333	-0.216187	0.0873079	-0.06444	down
A_24_P941643	PLCB1	-1.14619	-1.118844	-1.30242	0.0273461	-0.15623	-0.064442	down
A_23_P155052	APOL6	-2.32931	-2.635812	-2.151889	-0.306502	0.1774206	-0.064541	down
A_23_P45940	TFB2M	3.331582	3.222755	3.311224	-0.108827	-0.020358	-0.064593	down
A_24_P282309	MYOF	2.0469542	1.9607244	2.003871	-0.08623	-0.043083	-0.064656	down
A_33_P3358521	RIC1	-0.371236	-0.419082	-0.452767	-0.047845	-0.081531	-0.064688	down
A_33_P3365616	BTF3L4	3.6099577	3.559526	3.5309858	-0.050432	-0.078972	-0.064702	down
A_32_P165113	WDR92	2.889244	2.7524161	2.8966618	-0.136828	0.0074177	-0.064705	down
A_24_P116606	WWOX	-2.045138	-2.334729	-1.884959	-0.289591	0.1601791	-0.064706	down
A_33_P3273906	OR10G4	-2.257562	-2.26788	-2.376748	-0.010319	-0.119186	-0.064753	down
A_33_P3289721	CDRT1	-1.994172	-1.711329	-2.406533	0.2828431	-0.412361	-0.064759	down
A_24_P196534	PKNOX1	1.8377666	1.6711335	1.8747883	-0.166633	0.0370216	-0.064806	down
A_23_P29638	NCKIPSD	-1.062933	-1.140341	-1.115312	-0.077407	-0.052378	-0.064893	down
A_23_P98092	OAT	4.0125303	3.815083	4.080166	-0.197447	0.0676355	-0.064906	down
A_33_P3301219	FAM27C	0.8209019	0.6997218	0.8122139	-0.12118	-0.008688	-0.064934	down
A_23_P118246	GINS2	4.801278	4.5798326	4.8928223	-0.221446	0.0915442	-0.064951	down
A_33_P3415345	ALS2	-1.270363	-1.605063	-1.065702	-0.3347	0.2046614	-0.065019	down
A_24_P32627	LINC01183	-0.971117	-1.19921	-0.873103	-0.228093	0.0980134	-0.06504	down
A_23_P102950	RSPH1	0.7421722	0.8062348	0.548008	0.0640626	-0.194164	-0.065051	down
A_24_P246467	ATF2	-0.758208	-0.553326	-1.093207	0.2048821	-0.335	-0.065059	down
A_33_P3258056	PALM3	-0.209682	-0.079226	-0.470256	0.1304555	-0.260574	-0.065059	down
A_23_P97906	NT5C2	2.0550146	1.8795605	2.1000824	-0.175454	0.0450678	-0.065193	down
A_21_P0009056	LINC01572	-1.687943	-1.831845	-1.674579	-0.143903	0.0133634	-0.06527	down
A_19_P00331839	LOC100132356	-2.666702	-2.814736	-2.649266	-0.148034	0.0174356	-0.065299	down
A_33_P3282566	TMEM104	1.4281206	1.147162	1.5784469	-0.280959	0.1503263	-0.065316	down
A_23_P165891	TAF1B	1.0341816	0.9776616	0.95996	-0.05652	-0.074222	-0.065371	down

A_23_P42042	LYRM2	1.8474741	1.870328	1.693851	0.0228539	-0.153623	-0.065385	down
A_23_P139820	SLC11A2	1.7944651	1.8543706	1.6034317	0.0599055	-0.191033	-0.065564	down
A_23_P74914	URB2	2.5680618	2.27521	2.7297392	-0.292852	0.1616774	-0.065587	down
A_23_P145514	IL20RA	2.4101257	2.3744054	2.314662	-0.03572	-0.095464	-0.065592	down
A_23_P122545	NELFE	4.3061447	4.0225005	4.458517	-0.283644	0.1523724	-0.065636	down
A_33_P3210900	SEC13	-2.034726	-2.461697	-1.739104	-0.426971	0.2956219	-0.065675	down
A_33_P3317664	MGAT1	2.409443	2.2813745	2.4061565	-0.128068	-0.003286	-0.065677	down
A_24_P157342	BRK1	4.4943113	4.3739257	4.4830866	-0.120386	-0.011225	-0.065805	down
A_21_P0014329	CSNK2A2	2.9288797	2.758513	2.9676094	-0.170367	0.0387297	-0.065819	down
A_23_P207445	MAP2K6	0.8532381	0.6542649	0.9205098	-0.198973	0.0672717	-0.065851	down
A_24_P363802	PSMD5	2.5515652	2.387385	2.584033	-0.16418	0.0324678	-0.065856	down
A_23_P48358	PCCA	3.6005697	3.4648118	3.6045465	-0.135758	0.0039768	-0.065891	down
A_19_P00319465	MAVS	2.006812	1.8126836	2.0689993	-0.194129	0.0621872	-0.065971	down
A_33_P3230698	KIF1A	1.0864973	0.8776493	1.1633983	-0.208848	0.076901	-0.065974	down
A_33_P3261019	SLC9A2	0.0744538	-0.093662	0.1104717	-0.168116	0.0360179	-0.066049	down
A_23_P47077	BAG3	2.1748438	2.1325078	2.0850477	-0.042336	-0.089796	-0.066066	down
A_24_P152527	VAT1	0.5801559	0.6954889	0.3326445	0.1153331	-0.247511	-0.066089	down
A_33_P3391656	TP53RK	2.1684828	1.8927546	2.3119202	-0.275728	0.1434374	-0.066145	down
A_33_P3408632	LMF2	-1.182904	-1.387825	-1.110344	-0.204921	0.0725594	-0.066181	down
A_21_P0000235	SNORA6	0.6889801	0.4668613	0.7785816	-0.222119	0.0896015	-0.066259	down
A_23_P139143	STX3	1.043963	1.3057871	0.6494832	0.2618241	-0.39448	-0.066328	down
A_24_P62860	STAM2	-0.496709	-0.746222	-0.379961	-0.249513	0.1167479	-0.066382	down
A_33_P3225507	OR10G2	-2.061093	-2.063589	-2.191385	-0.002495	-0.130292	-0.066394	down
A_22_P00022810	POFUT1	0.1792421	0.1033554	0.1222787	-0.075887	-0.056963	-0.066425	down
A_33_P3390441	KPNB1	6.2421703	6.0867877	6.264514	-0.155383	0.0223436	-0.066519	down
A_32_P170925	TXNRD3	0.6715283	0.5206189	0.6893754	-0.150909	0.0178471	-0.066531	down
A_24_P385336	HIPK1	-1.634114	-1.841641	-1.559844	-0.207527	0.0742698	-0.066628	down
A_33_P3279610	COX6A2	-2.451733	-2.377397	-2.659351	0.0743365	-0.207618	-0.066641	down
A_21_P0007829	LINC00941	-0.9039	-1.127456	-0.813797	-0.223556	0.0901032	-0.066726	down
A_21_P0008046	AHSA2	-1.768506	-1.635982	-2.034486	0.1325245	-0.26598	-0.066728	down
A_23_P25913	DNAL1	1.2814951	1.3276	1.101862	0.0461049	-0.179633	-0.066764	down
A_23_P152818	KAT7	0.6933713	0.4420538	0.8111248	-0.251318	0.1177535	-0.066782	down
A_19_P00802433	LINC01564	-1.907677	-1.724013	-2.225001	0.1836643	-0.317324	-0.06683	down
A_23_P412186	ZNF252P	-0.359707	-0.318693	-0.534424	0.0410147	-0.174717	-0.066851	down
A_32_P720220	LINC01559	-0.38137	-0.220815	-0.675669	0.1605549	-0.294299	-0.066872	down
A_21_P0000862	LINC00856	-2.628094	-2.734024	-2.655916	-0.10593	-0.027822	-0.066876	down
A_24_P284353	TMEM107	2.1785889	2.1128926	2.1105232	-0.065696	-0.068066	-0.066881	down
A_33_P3594654	TMEM185B	2.6212234	2.4512677	2.6573734	-0.169956	0.03615	-0.066903	down
A_24_P286013	UBE2K	6.297447	6.1425786	6.318363	-0.154869	0.020916	-0.066976	down
A_23_P145569	SLC22A1	-1.990654	-2.092026	-2.023236	-0.101372	-0.032582	-0.066977	down
A_22_P00006003	lnc-FAH-1	-2.43148	-2.480667	-2.516347	-0.049188	-0.084867	-0.067027	down
A_23_P38567	SPECC1	2.6470156	2.937965	2.222003	0.2909493	-0.425013	-0.067032	down
A_33_P3283828	CCDC154	0.3411274	0.3469682	0.2010603	0.0058408	-0.140067	-0.067113	down
A_33_P3329834	PRPF38B	1.5780869	1.4377289	1.5842061	-0.140358	0.0061193	-0.067119	down
A_23_P353717	RMI2	4.818961	4.8655868	4.63801	0.0466256	-0.180951	-0.067163	down
A_23_P392384	AIF1L	2.4396324	2.244186	2.5004025	-0.195446	0.06077	-0.067338	down
A_32_P142818	DLX1	-3.173199	-3.265271	-3.215958	-0.092072	-0.042759	-0.067415	down
A_23_P105592	MVK	5.9112806	5.4786983	6.2090282	-0.432582	0.2977476	-0.067417	down
A_23_P368740	HDAC10	-1.633704	-1.543425	-1.858856	0.0902791	-0.225152	-0.067436	down
A_24_P406986	SLC43A3	1.6052008	1.5345092	1.540966	-0.070692	-0.064235	-0.067463	down
A_23_P115149	WDR77	3.295515	3.1326442	3.3234205	-0.162871	0.0279055	-0.067483	down
A_33_P3352449	MTRF1L	2.256072	2.1202989	2.2565994	-0.135773	0.0005274	-0.067623	down
A_33_P3400248	FGF20	5.2244673	4.811682	5.50187	-0.412785	0.2774029	-0.067691	down
A_23_P157766	SPATA6L	-1.206972	-1.14339	-1.406188	0.0635819	-0.199216	-0.067817	down
A_21_P0001364	lnc-DDX20-4	-2.240409	-2.442391	-2.174111	-0.201982	0.0662983	-0.067842	down
A_21_P0010635	LINC01057	-2.648872	-2.709977	-2.723484	-0.061105	-0.074612	-0.067858	down

A_23_P111273	TBC1D7	1.9409456	1.814507	1.9316168	-0.126439	-0.009329	-0.067884	down
A_33_P3295415	ZBTB3	-2.013279	-1.921591	-2.240789	0.0916886	-0.22751	-0.06791	down
A_32_P62863	SCHIP1	4.196866	4.163218	4.094472	-0.033648	-0.102394	-0.068021	down
A_24_P219474	MGAT5B	-1.444371	-1.548552	-1.476265	-0.104181	-0.031893	-0.068037	down
A_21_P0013806	HADHB	3.0188322	2.974214	2.9273186	-0.044618	-0.091514	-0.068066	down
A_33_P3349384	LOC102724074	-2.869046	-2.946448	-2.927809	-0.077402	-0.058763	-0.068082	down
A_21_P0012204	XLOC_l2_009219	-2.298787	-2.129693	-2.604415	0.1690936	-0.305628	-0.068267	down
A_23_P4899	NTF4	0.3704209	0.4467235	0.1573291	0.0763025	-0.213092	-0.068395	down
A_22_P00013887	LOC101927060	-1.657499	-1.5786	-1.873246	0.0788994	-0.215747	-0.068424	down
A_23_P123732	IDNK	1.935564	1.9043832	1.8298941	-0.031181	-0.10567	-0.068425	down
A_23_P427502	ZNRF3	2.4141302	2.3107085	2.3806925	-0.103422	-0.033438	-0.06843	down
A_23_P132438	MSL2	0.9561706	1.0303311	0.7450843	0.0741606	-0.211086	-0.068463	down
A_33_P3227209	PA2G4	8.075082	7.9535155	8.059705	-0.121566	-0.015377	-0.068472	down
A_24_P136161	HNRNPCL1	0.7321997	0.4514518	0.8757677	-0.280748	0.143568	-0.06859	down
A_33_P3249214	CNOT6L	1.6272011	1.4750352	1.642014	-0.152166	0.0148129	-0.068676	down
A_23_P20882	ATP6V1G1	5.6472855	5.557904	5.5993023	-0.089382	-0.047983	-0.068682	down
A_22_P00016741	lnc-TRH-2	-2.535722	-2.607638	-2.601179	-0.071915	-0.065457	-0.068686	down
A_23_P376759	DUSP11	0.9907436	0.8150177	1.0288272	-0.175726	0.0380836	-0.068821	down
A_33_P3315223	HNRNPA0	2.8683033	2.6934896	2.9054585	-0.174814	0.0371552	-0.068829	down
A_33_P3210203	SPIRE2	3.3219738	3.2828631	3.2234163	-0.039111	-0.098557	-0.068834	down
A_21_P0002041	LOC102725386	-3.259744	-3.292898	-3.364523	-0.033154	-0.104779	-0.068967	down
A_24_P109633	ITPK1	-1.930338	-2.127039	-1.871577	-0.1967	0.0587616	-0.068969	down
A_33_P3359157	FAM189B	-0.724653	-1.039756	-0.547498	-0.315103	0.1771555	-0.068974	down
A_22_P00001843	ATXN3	0.0913892	0.18291	-0.138097	0.0915208	-0.229486	-0.068983	down
A_23_P363769	KRT86	0.2114105	0.5169215	-0.232116	0.305511	-0.443526	-0.069008	down
A_33_P3240931	SMIM15	1.8342123	1.8152742	1.7150912	-0.018938	-0.119121	-0.06903	down
A_22_P00016919	LOC101929626	-1.489618	-1.983597	-1.133875	-0.493979	0.3557429	-0.069118	down
A_22_P00015289	CERS6-AS1	-0.575731	-0.771987	-0.517789	-0.196256	0.0579424	-0.069157	down
A_33_P3326225	SAMD12	3.7565098	3.7805047	3.594183	0.0239949	-0.162327	-0.069166	down
A_24_P98411	HSPA5	4.0145884	3.910005	3.9804955	-0.104583	-0.034093	-0.069338	down
A_22_P00015402	KMT2E-AS1	-1.729604	-1.7486	-1.849456	-0.018996	-0.119852	-0.069424	down
A_23_P144458	CAMK2D	2.316019	2.0612855	2.4318361	-0.254734	0.1158171	-0.069458	down
A_23_P415652	GALNT12	-0.412272	-0.385046	-0.578624	0.0272255	-0.166352	-0.069563	down
A_23_P38365	TLK2	2.0600166	2.1691413	1.8116546	0.1091247	-0.248362	-0.069619	down
A_23_P356526	TRIM5	-1.730108	-1.819867	-1.779659	-0.089758	-0.049551	-0.069655	down
A_23_P45831	CHD1L	2.802431	2.767427	2.6980228	-0.035004	-0.104408	-0.069706	down
A_22_P00004376	PCBP3	-0.91006	-0.939143	-1.020457	-0.029083	-0.110397	-0.06974	down
A_23_P3193	GOLGA5	1.0017667	0.9165993	0.9472041	-0.085167	-0.054563	-0.069865	down
A_24_P276932	ATP6V1C2	-0.322548	-0.434278	-0.350689	-0.11173	-0.028141	-0.069936	down
A_33_P3880302	EPHB2	0.0985336	0.1368055	-0.079627	0.0382719	-0.178161	-0.069944	down
A_23_P39910	COA5	1.0478525	1.1312399	0.8245468	0.0833874	-0.223306	-0.069959	down
A_23_P54929	LYRM1	1.7692614	1.9466977	1.4517231	0.1774364	-0.317538	-0.070051	down
A_33_P3330453	PBRM1	2.1085377	2.1685452	1.9082603	0.0600076	-0.200277	-0.070135	down
A_22_P00009098	UGDH-AS1	-0.904116	-1.070335	-0.878275	-0.16622	0.0258408	-0.070189	down
A_23_P142289	GNA11	0.6248894	0.4896455	0.6197395	-0.135244	-0.00515	-0.070197	down
A_22_P00012595	LINC01481	-0.068164	-0.183203	-0.093536	-0.115039	-0.025373	-0.070206	down
A_21_P0010776	PARP8	1.3388119	1.2986169	1.2385788	-0.040195	-0.100233	-0.070214	down
A_24_P130962	TOR3A	0.2650299	0.0522676	0.3373027	-0.212762	0.0722728	-0.070245	down
A_23_P12272	PPP1R8	3.870737	3.7842278	3.8167439	-0.086509	-0.053993	-0.070251	down
A_21_P0009375	lnc-CYB5D2-1	-2.417865	-2.849544	-2.127015	-0.43168	0.2908502	-0.070415	down
A_23_P161634	MUS81	2.845707	2.8056836	2.7447357	-0.040023	-0.100971	-0.070497	down
A_23_P52806	BACE1	0.2907567	0.2525263	0.1878705	-0.03823	-0.102886	-0.070558	down
A_24_P369694	PPP2R5C	1.5262861	1.4592905	1.4521503	-0.066996	-0.074136	-0.070566	down
A_23_P205549	MAX	4.7652445	4.93	4.459343	0.1647553	-0.305902	-0.070573	down
A_33_P3306287	METTL16	-0.242582	-0.34654	-0.279778	-0.103959	-0.037197	-0.070578	down
A_23_P165355	FARSB	2.5138817	2.3897939	2.49677	-0.124088	-0.017112	-0.0706	down

A_33_P3236558	TMEM234	-0.599505	-0.632119	-0.708243	-0.032614	-0.108738	-0.070676	down
A_22_P00018123	lnc-ZNF484-1	-0.405538	-0.29359	-0.658968	0.111948	-0.25343	-0.070741	down
A_21_P0005976	lnc-ASAP1-1	-0.405784	-0.548856	-0.404272	-0.143072	0.0015125	-0.07078	down
A_22_P00008939	lnc-KRT80-3	-1.562065	-1.635181	-1.630626	-0.073115	-0.068561	-0.070838	down
A_33_P3225268	QSOX1	0.4464917	0.4728141	0.2784567	0.0263224	-0.168035	-0.070856	down
A_23_P29422	GYG1	1.9597979	1.9597449	1.8180847	-5.29E-05	-0.141713	-0.070883	down
A_24_P121271	CPSF6	3.5731363	3.5320935	3.4723473	-0.041043	-0.100789	-0.070916	down
A_23_P310911	BLMH	2.2412176	1.7887144	2.5517998	-0.452503	0.3105822	-0.070961	down
A_23_P34045	EDA	-0.735526	-0.884025	-0.72896	-0.148499	0.0065665	-0.070966	down
A_21_P0000038	C2orf61	-0.812434	-1.23186	-0.534957	-0.419426	0.2774773	-0.070974	down
A_23_P254648	FBXW2	1.2069507	1.3122864	0.9596381	0.1053357	-0.247313	-0.070988	down
A_33_P3529859	KDM2B	-0.263978	-0.306957	-0.363088	-0.042978	-0.099109	-0.071044	down
A_33_P3251144	CDCA7L	-0.496479	-0.535533	-0.599744	-0.039053	-0.103265	-0.071159	down
A_33_P3333337	LRCH1	0.1887317	-0.034942	0.27003	-0.223674	0.0812984	-0.071188	down
A_32_P69930	CCDC96	-0.744131	-0.92376	-0.707041	-0.179629	0.0370898	-0.07127	down
A_21_P0013266	LOC101928451	-0.977444	-0.985267	-1.112201	-0.007823	-0.134758	-0.07129	down
A_23_P90565	WDPCP	-1.688908	-1.816329	-1.704093	-0.12742	-0.015184	-0.071302	down
A_24_P135483	TSPAN14	-0.112527	-0.370133	0.002079	-0.257607	0.1146059	-0.0715	down
A_32_P227845	LOC220729	2.985982	2.9245477	2.904397	-0.061434	-0.081585	-0.07151	down
A_23_P35131	SF3B4	-0.383801	-0.638155	-0.272645	-0.254353	0.111156	-0.071599	down
A_21_P0002416	lnc-AC074091.13.1-1	-1.02133	-0.897275	-1.28863	0.1240559	-0.267299	-0.071622	down
A_33_P3212092	PDCD4	2.6035652	2.3931851	2.6706991	-0.21038	0.0671339	-0.071623	down
A_33_P3233580	KIAA1217	-0.344861	-0.351188	-0.481845	-0.006328	-0.136984	-0.071656	down
A_33_P3399634	RASSF10	-2.763629	-3.176403	-2.4942	-0.412775	0.2694287	-0.071673	down
A_23_P93360	AGER	-1.290292	-1.502821	-1.221342	-0.212528	0.0689507	-0.071789	down
A_23_P319895	SETD1B	-0.456474	-0.293138	-0.763453	0.1633363	-0.306979	-0.071821	down
A_33_P3230269	GRHL1	3.6915045	3.6968627	3.5421858	0.0053582	-0.149319	-0.07198	down
A_23_P343808	SOS1	-1.268642	-1.262565	-1.418729	0.0060768	-0.150088	-0.072006	down
A_33_P3287428	LRRC37A4P	-1.386423	-1.273719	-1.64319	0.1127038	-0.256767	-0.072032	down
A_21_P0013289	LOC646588	-2.479993	-2.185053	-2.919291	0.29494	-0.439298	-0.072179	down
A_21_P0014940	lnc-TSC1-1	1.1328888	1.0571628	1.0636945	-0.075726	-0.069194	-0.07246	down
A_23_P405942	LARP4B	3.613865	3.545054	3.5377283	-0.068811	-0.076137	-0.072474	down
A_21_P0009663	lnc-ZNF30-1	-1.213975	-1.264488	-1.308582	-0.050512	-0.094607	-0.07256	down
A_33_P3335042	HSD17B12	1.6542168	1.5114222	1.6518097	-0.142795	-0.002407	-0.072601	down
A_33_P3386775	ADAM11	-2.054404	-2.515205	-1.738936	-0.460801	0.3154683	-0.072666	down
A_21_P0000469	SNORD11B	-1.450322	-1.38289	-1.663153	0.0674319	-0.212831	-0.0727	down
A_33_P3215452	LOC100129361	-1.034798	-1.328571	-0.886644	-0.293773	0.1481543	-0.072809	down
A_33_P3242099	CAMSAP2	1.9513578	1.8879061	1.8691559	-0.063452	-0.082202	-0.072827	down
A_21_P0013434	LINC00174	-3.015992	-2.999284	-3.178416	0.0167084	-0.162424	-0.072858	down
A_23_P89187	LASP1	6.1902304	6.0808063	6.153905	-0.109424	-0.036325	-0.072875	down
A_23_P161615	POLA2	1.0130968	1.0191865	0.8611579	0.0060897	-0.151939	-0.072925	down
A_23_P72068	GMDS	0.3965192	0.3749595	0.2722273	-0.02156	-0.124292	-0.072926	down
A_23_P89410	BECN1	3.589631	3.4740624	3.5592566	-0.115569	-0.030375	-0.072972	down
A_32_P224234	LOC645195	-2.297581	-2.496972	-2.244258	-0.19939	0.0533237	-0.073033	down
A_23_P128408	TRIAP1	3.1488285	2.9260345	3.2255125	-0.222794	0.076684	-0.073055	down
A_32_P57702	LOC641746	5.1697664	5.0893607	5.104044	-0.080406	-0.065722	-0.073064	down
A_19_P00804212	NFU1	1.3071699	1.4438176	1.0240707	0.1366477	-0.283099	-0.073226	down
A_24_P175612	SFXN2	0.065371	-0.332729	0.3169642	-0.3981	0.2515931	-0.073253	down
A_33_P3329652	ZFP90	1.8368149	1.6208019	1.9062967	-0.216013	0.0694819	-0.073266	down
A_23_P500773	MAP3K7	2.1105595	1.967524	2.1069212	-0.143035	-0.003638	-0.073337	down
A_23_P168556	STX1A	3.048357	3.0626597	2.8873281	0.0143027	-0.161029	-0.073363	down
A_23_P368484	LRRC75A	0.5510597	0.3478799	0.6073089	-0.20318	0.0562491	-0.073465	down
A_33_P3336968	TRNT1	0.0725985	-0.019342	0.0173774	-0.091941	-0.055221	-0.073581	down
A_23_P253221	ARHGEF4	3.2721243	3.0651908	3.3318596	-0.206934	0.0597353	-0.073599	down
A_23_P49338	TNFRSF12A	7.10159	7.0173125	7.038329	-0.084278	-0.063261	-0.073769	down

A_33_P3352970	IRAK2	-2.425367	-2.544576	-2.453943	-0.119208	-0.028575	-0.073892	down
A_33_P3383696	SPEG	1.9714355	1.9335933	1.8614788	-0.037842	-0.109957	-0.0739	down
A_33_P3287547	PRKCZ	-0.734352	-0.74104	-0.875758	-0.006688	-0.141406	-0.074047	down
A_23_P2317	DDN	-2.191156	-2.561391	-1.969102	-0.370235	0.2220535	-0.074091	down
A_23_P24709	OSBP	2.1945534	2.2217126	2.0190735	0.0271592	-0.17548	-0.07416	down
A_32_P26330	USP10	0.0042467	-0.124149	-0.015809	-0.128396	-0.020056	-0.074226	down
A_23_P202170	MGEA5	6.134863	6.1956687	5.9255943	0.0608058	-0.209269	-0.074231	down
A_33_P3304888	USP32	0.4295149	0.4820156	0.2284484	0.0525007	-0.201067	-0.074283	down
A_24_P148750	SH3BP5	1.2296643	1.336875	0.973825	0.1072106	-0.255839	-0.074314	down
A_21_P0011126	NUS1	3.9009619	3.6593251	3.9938536	-0.241637	0.0928917	-0.074373	down
A_23_P56709	RNF103	3.0585032	3.053615	2.9145594	-0.004888	-0.143944	-0.074416	down
A_33_P3331588	ATAD3B	5.765995	5.5797086	5.8032303	-0.186286	0.0372353	-0.074526	down
A_23_P346405	MCU	-0.192783	-0.301664	-0.233046	-0.108881	-0.040263	-0.074572	down
A_23_P309619	KIAA1671	3.0189772	2.9471946	2.941495	-0.071783	-0.077482	-0.074632	down
A_23_P100292	ZNF598	6.0711937	5.7173448	6.2755337	-0.353849	0.20434	-0.074754	down
A_23_P34733	FH	3.8759155	3.7800994	3.8220463	-0.095816	-0.053869	-0.074843	down
A_33_P3328511	ARL17B	0.9601054	0.7666616	1.0038118	-0.193444	0.0437064	-0.074869	down
A_23_P152356	ZNF200	2.2180185	2.0746017	2.2116652	-0.143417	-0.006353	-0.074885	down
A_23_P103371	AZIN2	-2.512904	-3.090459	-2.085169	-0.577555	0.4277349	-0.07491	down
A_23_P250283	RAB1A	3.3576202	3.3176112	3.2477531	-0.040009	-0.109867	-0.074938	down
A_23_P347528	TFCP2	2.7000742	2.5898423	2.6603928	-0.110232	-0.039681	-0.074957	down
A_24_P224727	CEBPA	1.212337	1.1373062	1.1373792	-0.075031	-0.074958	-0.074994	down
A_33_P3364884	PDHB	4.2163954	4.162785	4.1199675	-0.05361	-0.096428	-0.075019	down
A_23_P33407	HERC2	1.413888	1.4041057	1.2736235	-0.009782	-0.140265	-0.075023	down
A_23_P103442	MEAF6	3.9233084	3.673614	4.0228806	-0.249694	0.0995722	-0.075061	down
A_23_P39116	LIG1	4.8926306	4.758367	4.876704	-0.134264	-0.015926	-0.075095	down
A_33_P3369781	UBE3D	-0.120573	-0.081268	-0.310443	0.0393052	-0.18987	-0.075283	down
A_33_P3346193	TPM3	0.0210795	-0.191716	0.083283	-0.212795	0.0622034	-0.075296	down
A_32_P36313	VTI1A	1.7380657	1.6575036	1.6679587	-0.080562	-0.070107	-0.075335	down
A_33_P3357964	PDPK1	-2.682694	-2.86865	-2.647521	-0.185956	0.0351734	-0.075391	down
A_22_P00002857	H1FX-AS1	-2.741689	-2.802479	-2.83177	-0.06079	-0.090081	-0.075435	down
A_23_P91930	QTRTD1	3.4152613	3.250235	3.429265	-0.165026	0.0140038	-0.075511	down
A_22_P00006984	lnc-GDPD4-1	-1.06496	-1.240688	-1.040302	-0.175728	0.0246577	-0.075535	down
A_23_P63459	COA6	5.3091593	5.096559	5.3706245	-0.2126	0.0614653	-0.075567	down
A_22_P00010975	CACNA1C-AS1	-2.803048	-2.834472	-2.92315	-0.031424	-0.120101	-0.075763	down
A_24_P68585	SHC1	-0.133367	-0.282447	-0.135823	-0.14908	-0.002457	-0.075768	down
A_33_P3242973	IGF2BP2	-0.538211	-0.687963	-0.540171	-0.149752	-0.001959	-0.075855	down
A_24_P919899	ATPAF1	-2.678292	-2.763237	-2.745194	-0.084944	-0.066902	-0.075923	down
A_24_P106910	PTCH1	-1.503585	-1.419636	-1.739443	0.0839496	-0.235858	-0.075954	down
A_22_P00018081	LOC101929464	-1.735548	-1.766157	-1.856877	-0.03061	-0.121329	-0.075969	down
A_21_P0012310	DGCR5	-2.410244	-2.85032	-2.122157	-0.440077	0.2880864	-0.075995	down
A_23_P45913	SNRNP40	-0.418447	-0.56241	-0.426528	-0.143963	-0.008081	-0.076022	down
A_23_P379794	PIGW	-0.038593	0.0123491	-0.24177	0.0509419	-0.203177	-0.076118	down
A_23_P2582	HDAC7	1.7671576	1.7295599	1.6524329	-0.037598	-0.114725	-0.076161	down
A_33_P3381996	C8orf49	-2.8394	-3.125192	-2.705989	-0.285792	0.1334117	-0.07619	down
A_22_P00005091	DGCR5	2.468278	2.2655196	2.5186472	-0.202758	0.0503693	-0.076195	down
A_23_P83094	TLE4	2.8121748	2.6443534	2.8274765	-0.167821	0.0153017	-0.07626	down
A_23_P45799	ORC1	0.5494728	0.3785434	0.5676827	-0.170929	0.0182099	-0.07636	down
A_33_P3256168	SNX30	-2.35954	-2.17263	-2.699171	0.1869102	-0.339631	-0.07636	down
A_23_P126291	SNRPE	6.586835	6.422301	6.598303	-0.164534	0.0114679	-0.076533	down
A_23_P120472	TFAP2C	-2.40366	-2.458916	-2.501482	-0.055256	-0.097822	-0.076539	down
A_24_P122137	LIF	2.9237032	3.0365071	2.6578035	0.1128039	-0.2659	-0.076548	down
A_24_P87114	USP30	-2.201118	-2.352564	-2.202816	-0.151446	-0.001698	-0.076572	down
A_22_P00007447	LOC100506444	-2.662204	-2.992496	-2.485204	-0.330292	0.1770003	-0.076646	down
A_21_P0002962	lnc-FLNB-1	-1.00897	-1.038412	-1.132861	-0.029442	-0.123891	-0.076666	down
A_33_P3324909	JUND	0.4736013	0.4122906	0.3813381	-0.061311	-0.092263	-0.076787	down

A_23_P304287	PSMC2	4.987975	4.9558635	4.866393	-0.032112	-0.121582	-0.076847	down
A_23_P57709	PCOLCE2	3.298541	3.1233306	3.3198633	-0.17521	0.0213223	-0.076944	down
A_33_P3377239	LOC100270804	-1.642708	-1.570227	-1.869149	0.0724812	-0.226441	-0.07698	down
A_33_P3280694	GSAP	1.4393859	1.2736087	1.4510431	-0.165777	0.0116572	-0.07706	down
A_23_P400945	ETV3	-1.763332	-1.753065	-1.927719	0.0102668	-0.164387	-0.07706	down
A_23_P16469	PLAUR	2.0271854	1.8012385	2.0989285	-0.225947	0.071743	-0.077102	down
A_24_P64233	ALDH1B1	2.1826725	1.8413386	2.3697262	-0.341334	0.1870537	-0.07714	down
A_23_P164468	ADNP2	0.5153804	0.5179725	0.3581719	0.0025921	-0.157208	-0.077308	down
A_23_P50535	DMPK	2.5967312	2.5604339	2.4783735	-0.036297	-0.118358	-0.077327	down
A_33_P3650224	CXorf49B	-1.734222	-1.88123	-1.742029	-0.147008	-0.007807	-0.077407	down
A_33_P3265270	SLC17A5	-0.94289	-1.147481	-0.893197	-0.204591	0.0496931	-0.077449	down
A_23_P374782	SH3KBP1	3.2827177	3.3331504	3.077341	0.0504327	-0.205377	-0.077472	down
A_33_P3336484	NPIPA5	2.0359821	2.1575665	1.7593203	0.1215844	-0.276662	-0.077539	down
A_21_P0012317	FAM230B	-1.576356	-1.498791	-1.809008	0.0775652	-0.232652	-0.077544	down
A_33_P3327108	NLK	2.5756445	2.5484576	2.4474545	-0.027187	-0.12819	-0.077688	down
A_33_P3221498	STK31	-2.526822	-2.410034	-2.798996	0.1167874	-0.272174	-0.077693	down
A_32_P447001	C15orf65	-0.747602	-0.861259	-0.789382	-0.113657	-0.04178	-0.077719	down
A_23_P6119	SEC23B	1.611393	1.6608558	1.4062853	0.0494628	-0.205108	-0.077822	down
A_32_P141238	ANO2	-0.97404	-0.576847	-1.526878	0.3971925	-0.552838	-0.077823	down
A_23_P24535	TTC12	2.1106348	2.1005359	1.9649334	-0.010099	-0.145701	-0.0779	down
A_23_P98345	SNX19	2.3839283	2.2933402	2.3185883	-0.090588	-0.06534	-0.077964	down
A_24_P3249	RARB	-1.45985	-1.64959	-1.426058	-0.18974	0.0337915	-0.077974	down
A_23_P200073	PITHD1	0.4097095	0.3401847	0.3232012	-0.069525	-0.086508	-0.078017	down
A_23_P81212	MRPS18C	6.55766	6.346617	6.612485	-0.211043	0.0548248	-0.078109	down
A_33_P3327956	ZNF605	1.5586243	1.7629647	1.1980515	0.2043405	-0.360573	-0.078116	down
A_23_P167509	CYFIP2	-0.190595	-0.561502	0.0239677	-0.370907	0.2145624	-0.078172	down
A_22_P00003103	LOC158435	-1.509403	-1.294165	-1.881006	0.2152381	-0.371603	-0.078182	down
A_23_P145408	FUCA2	4.36985	4.3799176	4.2033443	0.0100675	-0.166506	-0.078219	down
A_23_P313728	FAM98A	2.3632364	2.1553283	2.4146967	-0.207908	0.0514603	-0.078224	down
A_23_P106544	CMC2	5.6021795	5.250344	5.797407	-0.351836	0.1952276	-0.078304	down
A_33_P3380051	MYADML2	-2.571972	-3.051595	-2.248984	-0.479622	0.322988	-0.078317	down
A_23_P133279	FAM173B	1.0038991	0.926044	0.9250851	-0.077855	-0.078814	-0.078335	down
A_23_P148446	UBE2A	4.86201	4.783262	4.784074	-0.078748	-0.077936	-0.078342	down
A_22_P00012823	LOC728095	-2.483559	-2.508137	-2.615912	-0.024579	-0.132353	-0.078466	down
A_24_P64653	METTL7B	1.8911114	1.4296393	2.195344	-0.461472	0.3042326	-0.07862	down
A_23_P152583	ENGASE	1.1812515	1.0926776	1.1124439	-0.088574	-0.068808	-0.078691	down
A_33_P3216297	NR3C1	1.3282156	1.270195	1.2288184	-0.058021	-0.099397	-0.078709	down
A_21_P0001250	lnc-SAMD11-2	-2.778892	-2.634503	-3.080842	0.1443896	-0.301949	-0.07878	down
A_23_P17870	TMEM184B	6.554351	6.4965835	6.4544764	-0.057767	-0.099875	-0.078821	down
A_22_P00012840	MEF2C-AS1	0.4509234	0.2311635	0.5129185	-0.21976	0.061995	-0.078882	down
A_33_P3357247	USP36	3.8065825	3.8473182	3.6079836	0.0407357	-0.198599	-0.078932	down
A_33_P3358243	KIAA1161	3.5232582	3.151216	3.7374105	-0.372042	0.2141523	-0.078945	down
A_23_P25684	RDH11	4.100648	3.716454	4.3269444	-0.384194	0.2262964	-0.078949	down
A_23_P98763	LRTOMT	-0.978645	-1.264067	-0.851283	-0.285422	0.1273618	-0.07903	down
A_23_P118815	BIRC5	6.528487	6.234182	6.6645994	-0.294305	0.1361122	-0.079097	down
A_22_P00005149	LOC101927978	-1.331121	-1.645534	-1.174921	-0.314413	0.1562009	-0.079106	down
A_23_P131596	PRADC1	1.337287	1.0469217	1.4693761	-0.290365	0.1320891	-0.079138	down
A_21_P0009973	lnc-PTPN1-2	-3.162498	-3.235356	-3.248036	-0.072859	-0.085539	-0.079199	down
A_23_P252681	PCYT1A	2.5338392	2.432819	2.4761438	-0.10102	-0.057695	-0.079358	down
A_21_P0009768	lnc-ZNF404-1	-1.74075	-1.67833	-1.961981	0.0624204	-0.221231	-0.079405	down
A_23_P256455	RPA3	6.2700872	6.179478	6.201844	-0.090609	-0.068243	-0.079426	down
A_24_P55250	HDDC2	4.146633	3.9854789	4.1489286	-0.161154	0.0022955	-0.079429	down
A_24_P347704	CTCF	1.32616	1.3459439	1.1474714	0.019784	-0.178689	-0.079452	down
A_33_P3255434	MEG3	-2.17956	-2.189974	-2.32809	-0.010414	-0.14853	-0.079472	down
A_24_P381962	AP1G1	3.5568037	3.4690013	3.4856453	-0.087802	-0.071158	-0.07948	down
A_32_P198330	SREK1IP1	0.2215452	0.1027818	0.1813388	-0.118763	-0.040206	-0.079485	down

A_24_P257579	EPB41L4A	0.5205874	0.4326353	0.4492993	-0.087952	-0.071288	-0.07962	down
A_32_P198923	YWHAZ	4.895323	4.8770623	4.754284	-0.01826	-0.141039	-0.07965	down
A_21_P0000211	SNORA70	2.52606	2.5105176	2.3822775	-0.015543	-0.143783	-0.079663	down
A_22_P00012501	PAX8-AS1	-1.661411	-1.883555	-1.598613	-0.222144	0.062798	-0.079673	down
A_33_P3287815	DDX21	4.8132105	4.6783576	4.788657	-0.134853	-0.024553	-0.079703	down
A_24_P223163	NAF1	-0.856902	-1.078952	-0.794508	-0.22205	0.0623946	-0.079828	down
A_23_P334123	ITFG1	1.9436712	1.8458548	1.8817825	-0.097816	-0.061889	-0.079853	down
A_22_P00006217	lnc-FAM46D-1	-1.158355	-1.129577	-1.346896	0.0287786	-0.188541	-0.079881	down
A_24_P419028	MOP-1	0.0935812	0.243094	-0.215786	0.1495128	-0.309368	-0.079927	down
A_33_P3340254	KMT2D	2.9048834	2.9576669	2.6921453	0.0527835	-0.212738	-0.079977	down
A_24_P89872	ZNF189	1.7486076	1.783524	1.5535841	0.0349164	-0.195024	-0.080054	down
A_33_P3213432	ARMC10	4.1823893	4.103065	4.1014347	-0.079324	-0.080955	-0.080139	down
A_23_P108785	ACTR3	5.7530394	5.6153083	5.730221	-0.137731	-0.022819	-0.080275	down
A_21_P0000214	SNORD42A	-3.021332	-3.108779	-3.094596	-0.087447	-0.073265	-0.080356	down
A_19_P00321166	LOC100132356	-0.801277	-0.487227	-1.276072	0.3140497	-0.474795	-0.080373	down
A_33_P3410507	CEP170	-0.210993	-0.242105	-0.340733	-0.031111	-0.129739	-0.080425	down
A_23_P39774	SNRNP27	3.057376	2.8879828	3.065898	-0.169393	0.008522	-0.080436	down
A_23_P210330	LGALSL	3.433404	3.299046	3.4066734	-0.134358	-0.026731	-0.080544	down
A_23_P404134	TOX4	2.485695	2.4350457	2.3751478	-0.050649	-0.110547	-0.080598	down
A_23_P2446	TMEM5	3.1015253	2.8327603	3.2090588	-0.268765	0.1075335	-0.080616	down
A_33_P3253799	RUFY1	1.2044692	1.2168145	1.0306468	0.0123453	-0.173822	-0.080739	down
A_33_P3258146	LOC100130539	-2.279033	-2.501148	-2.21855	-0.222115	0.0604832	-0.080816	down
A_33_P3396951	BNIP3L	-0.620154	-0.395789	-1.006204	0.2243648	-0.38605	-0.080842	down
A_23_P2097	TRIM68	0.0523644	-0.128847	0.0717578	-0.181211	0.0193934	-0.080909	down
A_24_P867342	AFMID	-1.640821	-1.637278	-1.806206	0.0035429	-0.165385	-0.080921	down
A_23_P157022	ZNF786	0.6119504	0.51015	0.5518279	-0.1018	-0.060122	-0.080961	down
A_23_P143374	NINL	3.4300423	3.5032516	3.1944504	0.0732093	-0.235592	-0.081191	down
A_23_P146830	SLC25A10	2.0650787	1.8509054	2.1167955	-0.214173	0.0517168	-0.081228	down
A_22_P00012385	lnc-PRKAA2-7	-2.089633	-2.15781	-2.183982	-0.068177	-0.094349	-0.081263	down
A_32_P182511	TLDC1	-0.928046	-1.063455	-0.955227	-0.135409	-0.027182	-0.081295	down
A_23_P107587	NPC1	1.7630348	1.8613777	1.5020938	0.0983429	-0.260941	-0.081299	down
A_21_P0000572	PITPNA-AS1	2.6509628	2.3342576	2.8049917	-0.316705	0.1540289	-0.081338	down
A_23_P40866	ZBTB20	1.0843163	1.2395315	0.7664204	0.1552153	-0.317896	-0.08134	down
A_23_P200216	MAGOH	4.1363764	3.9139342	4.1961327	-0.222442	0.0597563	-0.081343	down
A_33_P3357949	ETV1	-2.385403	-2.524179	-2.409396	-0.138776	-0.023993	-0.081385	down
A_24_P36285	ZNF224	-0.996867	-1.123728	-1.032923	-0.126861	-0.036056	-0.081459	down
A_23_P377376	ACTR2	4.155608	3.9810567	4.1671877	-0.174551	0.0115795	-0.081486	down
A_24_P111242	SVIP	-1.961154	-1.812837	-2.272445	0.1483169	-0.311291	-0.081487	down
A_33_P3394031	UBXN11	-1.570462	-1.565072	-1.738838	0.0053902	-0.168375	-0.081493	down
A_23_P162945	SRP54	0.9746671	0.87742	0.9086266	-0.097247	-0.066041	-0.081644	down
A_23_P259333	C6orf203	2.9579086	2.97409	2.7784185	0.0161815	-0.17949	-0.081654	down
A_33_P3230350	ZNF207	3.453886	3.2915673	3.4528599	-0.162319	-0.001026	-0.081672	down
A_33_P3243618	GPR137B	-0.146721	-0.384832	-0.07208	-0.238111	0.0746417	-0.081734	down
A_24_P294719	FAF2	3.2092915	2.9106822	3.3442345	-0.298609	0.134943	-0.081833	down
A_22_P00011026	lnc-NT5DC2-1	-0.391016	-0.562954	-0.382896	-0.171937	0.0081205	-0.081908	down
A_33_P3236082	NSDHL	5.9196177	5.780522	5.894889	-0.139096	-0.024729	-0.081912	down
A_32_P104063	CRNDE	3.0152712	3.0668664	2.7997303	0.0515952	-0.215541	-0.081973	down
A_21_P0014274	LOC171391	0.7005553	0.6175132	0.6196361	-0.083042	-0.080919	-0.081981	down
A_22_P00002815	LOC101928371	-2.942109	-2.957026	-3.091241	-0.014917	-0.149132	-0.082025	down
A_22_P00010781	NKX6-2	-2.967754	-3.081947	-3.017752	-0.114192	-0.049998	-0.082095	down
A_23_P103601	MAN1C1	-1.056608	-1.336881	-0.940653	-0.280272	0.1159554	-0.082159	down
A_32_P197340	ERICH2	-0.153501	-0.007943	-0.463649	0.1455584	-0.310148	-0.082295	down
A_33_P3344405	MEF2B	3.31946	2.9428535	3.5314035	-0.376606	0.2119436	-0.082331	down
A_23_P404606	CREBRF	0.8097286	1.2948637	0.1598148	0.4851351	-0.649914	-0.082389	down
A_21_P0000266	SNORA72	-0.102525	-0.2744	-0.095634	-0.171875	0.0068913	-0.082492	down
A_23_P143994	FANCD2	0.7970095	0.5864134	0.8426085	-0.210596	0.045599	-0.082499	down

A_23_P75430	SMCO4	4.3664656	3.9799628	4.587901	-0.386503	0.2214356	-0.082534	down
A_33_P3311439	GCH1	-0.649684	-0.733996	-0.730613	-0.084312	-0.080928	-0.08262	down
A_23_P214354	EXOC2	-2.629383	-2.767916	-2.65623	-0.138533	-0.026848	-0.082691	down
A_21_P0000371	SNORD7	-1.260691	-1.038876	-1.647918	0.2218156	-0.387227	-0.082705	down
A_33_P3386099	ELK1	-2.143256	-2.184787	-2.26736	-0.041531	-0.124103	-0.082817	down
A_22_P00008786	LOC101927761	-2.179573	-2.286248	-2.238553	-0.106675	-0.05898	-0.082827	down
A_23_P343366	MAB21L3	-2.741438	-2.800802	-2.847738	-0.059364	-0.1063	-0.082832	down
A_21_P0013322	RABGEF1	-1.514482	-1.51498	-1.679674	-0.000498	-0.165192	-0.082845	down
A_23_P402733	SON	-0.154718	-0.052007	-0.423208	0.1027117	-0.268489	-0.082889	down
A_21_P0011400	lnc-GOLGA8J-3	-1.161126	-1.380833	-1.107218	-0.219707	0.0539079	-0.0829	down
A_23_P101185	NARS	3.9541101	3.9377465	3.8043194	-0.016364	-0.149791	-0.083077	down
A_23_P132417	LSG1	3.1642656	2.8195243	3.3427353	-0.344741	0.1784697	-0.083136	down
A_23_P111672	TES	1.0973935	1.2163439	0.8121429	0.1189504	-0.285251	-0.08315	down
A_23_P366328	VPS37A	1.1441302	1.0225801	1.0991797	-0.12155	-0.04495	-0.08325	down
A_23_P81912	TUBB	4.867625	4.7072864	4.8613853	-0.160339	-0.00624	-0.083289	down
A_33_P3324428	AKAP1	0.039165	0.029098	-0.117366	-0.010067	-0.156531	-0.083299	down
A_33_P3394075	RIT1	0.6003165	0.7360873	0.2978673	0.1357708	-0.302449	-0.083339	down
A_33_P3725227	COBL	2.5491495	2.5849042	2.3466883	0.0357547	-0.202461	-0.083353	down
A_23_P145761	ARL4A	2.0336428	1.9593816	1.9411736	-0.074261	-0.092469	-0.083365	down
A_23_P207537	DUSP14	3.635931	3.5599265	3.5451727	-0.076005	-0.090758	-0.083381	down
A_33_P3847514	C6orf141	-2.136197	-2.125102	-2.314101	0.0110955	-0.177904	-0.083404	down
A_24_P226355	RANBP9	0.3027649	0.2147203	0.2237167	-0.088045	-0.079048	-0.083546	down
A_24_P242820	TSN	3.0035267	2.7955308	3.0439892	-0.207996	0.0404625	-0.083767	down
A_23_P5064	CADM4	0.0499744	0.0679565	-0.135549	0.017982	-0.185524	-0.083771	down
A_32_P216548	LDLRAP1	-0.179355	-0.541935	0.0153108	-0.36258	0.1946654	-0.083957	down
A_23_P77174	PIGB	0.598309	0.6428504	0.3858323	0.0445414	-0.212477	-0.083968	down
A_22_P00012072	lnc-PMEL-1	-1.48411	-1.474129	-1.662058	0.0099816	-0.177948	-0.083983	down
A_21_P0013748	CD99P1	-0.390154	-0.488338	-0.459945	-0.098183	-0.069791	-0.083987	down
A_22_P00019045	MIR181A2HG	-3.206874	-3.134929	-3.446841	0.0719452	-0.239966	-0.08401	down
A_32_P743407	GLYR1	0.5977655	0.3470674	0.6803184	-0.250698	0.0825529	-0.084073	down
A_23_P110624	CTNND2	-3.112872	-3.040839	-3.353159	0.0720334	-0.240287	-0.084127	down
A_23_P100056	RBPMS2	-1.121447	-1.755813	-0.655611	-0.634366	0.4658365	-0.084265	down
A_23_P205738	BCL11B	0.9321685	0.8883157	0.807394	-0.043853	-0.124774	-0.084314	down
A_24_P122921	BCL2L11	3.102847	3.2198176	2.8172035	0.1169705	-0.285644	-0.084337	down
A_24_P323104	DIAPH1	6.4930286	6.357796	6.4595814	-0.135232	-0.033447	-0.08434	down
A_23_P374082	ADAM19	1.8162689	1.3953161	2.0685282	-0.420953	0.2522593	-0.084347	down
A_23_P161686	ARHGAP32	2.3378534	2.2452579	2.2617092	-0.092596	-0.076144	-0.08437	down
A_23_P317324	MECOM	1.3830342	1.3449936	1.2522831	-0.038041	-0.130751	-0.084396	down
A_23_P430381	LYSMD1	1.2415204	0.9147973	1.3992524	-0.326723	0.157732	-0.084496	down
A_23_P476	MPZL1	4.933012	4.727325	4.96953	-0.205687	0.0365181	-0.084584	down
A_33_P3213374	CITED2	-2.303922	-2.030903	-2.746345	0.2730188	-0.442423	-0.084702	down
A_33_P3328426	ANO10	4.0796003	3.8101048	4.179614	-0.269496	0.1000137	-0.084741	down
A_23_P15182	ARL2BP	3.2367983	3.1500306	3.1540012	-0.086768	-0.082797	-0.084782	down
A_22_P00012047	lnc-PLEKHG6-1	1.7874146	1.810852	1.5942879	0.0234375	-0.193127	-0.084845	down
A_33_P3280965	SNHG9	6.3312206	5.876453	6.6160927	-0.454768	0.2848721	-0.084948	down
A_23_P78209	MAFG	3.2181492	3.1409936	3.1254053	-0.077156	-0.092744	-0.08495	down
A_19_P00803226	lnc-TOP1MT-2	3.0510921	2.7131257	3.219017	-0.337966	0.1679249	-0.085021	down
A_22_P00012040	lnc-PLEKHB1-1	-2.107935	-2.213235	-2.172772	-0.1053	-0.064837	-0.085068	down
A_32_P42054	DND1	1.6133957	1.5132823	1.5431952	-0.100113	-0.0702	-0.085157	down
A_23_P50942	RAB3GAP1	3.621976	3.4800649	3.593441	-0.141911	-0.028535	-0.085223	down
A_33_P3270360	OR1S1	-2.651346	-2.838067	-2.635196	-0.186722	0.0161498	-0.085286	down
A_23_P255376	CCDC109B	2.8295488	2.6644368	2.8240776	-0.165112	-0.005471	-0.085292	down
A_22_P00024540	lnc-VN1R2-1	-0.602994	-0.601211	-0.77544	0.0017834	-0.172445	-0.085331	down
A_23_P114221	RBBP7	5.905486	5.825071	5.815177	-0.080415	-0.090309	-0.085362	down
A_33_P3840630	FRYL	0.6584287	0.5147767	0.6312137	-0.143652	-0.027215	-0.085433	down
A_33_P3331376	EPHB2	-0.85014	-0.900178	-0.971084	-0.050039	-0.120945	-0.085492	down

A_24_P419300	PP7080	2.442771	2.2297177	2.4848347	-0.213053	0.0420637	-0.085495	down
A_21_P0014839	STAMBP	-0.891582	-1.345006	-0.609185	-0.453424	0.2823968	-0.085514	down
A_23_P323751	FAM83D	3.7860289	3.5319848	3.868907	-0.254044	0.0828781	-0.085583	down
A_33_P3376434	SETD5	0.9927406	0.7927423	1.0215402	-0.199998	0.0287995	-0.085599	down
A_23_P372771	FAM219B	-1.194259	-1.165895	-1.393841	0.0283642	-0.199583	-0.085609	down
A_23_P102832	CEP250	1.0082846	0.9708071	0.8744831	-0.037477	-0.133801	-0.085639	down
A_33_P3397418	ZC3HAV1	4.345745	4.223826	4.2962494	-0.121919	-0.049496	-0.085707	down
A_33_P3273063	NUP43	-0.338132	-0.470253	-0.37754	-0.132122	-0.039408	-0.085765	down
A_33_P3385436	PLAC8L1	-1.805379	-1.55797	-2.224365	0.2474093	-0.418986	-0.085788	down
A_23_P168812	FAM200A	-0.703323	-0.775861	-0.802508	-0.072538	-0.099185	-0.085862	down
A_21_P0009650	LOC100652911	-1.173332	-1.194535	-1.323977	-0.021203	-0.150645	-0.085924	down
A_21_P0008110	CLYBL	-0.364449	-0.731	-0.169971	-0.366551	0.1944776	-0.086037	down
A_33_P3224105	KNSTRN	5.11154	4.9581056	5.0927715	-0.153434	-0.018768	-0.086101	down
A_33_P3414422	GPHN	3.008071	2.7958732	3.047803	-0.212198	0.039732	-0.086233	down
A_23_P168229	TXNDC5	2.8685703	2.6700387	2.894536	-0.198532	0.0259657	-0.086283	down
A_21_P0013584	LOC102725126	-2.492472	-2.587359	-2.570346	-0.094887	-0.077874	-0.086381	down
A_24_P250922	PTGS2	-1.454847	-1.500515	-1.581976	-0.045667	-0.127129	-0.086398	down
A_23_P218793	XPNPEP3	0.5321584	0.4737649	0.4177165	-0.058393	-0.114442	-0.086418	down
A_21_P0005605	lnc-GUSB-2	-1.241318	-1.36808	-1.287544	-0.126761	-0.046226	-0.086493	down
A_24_P66679	NAA30	-0.429137	-0.390274	-0.641056	0.0388632	-0.211919	-0.086528	down
A_23_P125829	PGK1	7.0131493	7.1004224	6.752777	0.0872731	-0.260372	-0.08655	down
A_22_P00010763	lnc-NKD2-3	1.0207129	0.8179765	1.0502748	-0.202736	0.029562	-0.086587	down
A_22_P00017259	lnc-UNG-1	-2.641471	-2.612903	-2.843326	0.0285678	-0.201855	-0.086644	down
A_33_P3308534	OSBPL1A	-0.55495	-0.735828	-0.547395	-0.180878	0.007555	-0.086662	down
A_23_P256231	FBXO30	2.392005	2.4560466	2.1544418	0.0640416	-0.237563	-0.086761	down
A_23_P28068	GTPBP3	1.4279189	1.3686228	1.313643	-0.059296	-0.114276	-0.086786	down
A_33_P3292596	ERBB2	2.3719769	2.379188	2.1911116	0.0072112	-0.180865	-0.086827	down
A_23_P76684	RTN3	2.5171604	2.4400773	2.4205723	-0.077083	-0.096588	-0.086836	down
A_23_P15639	C17orf75	0.3891792	0.2082148	0.396368	-0.180964	0.0071888	-0.086888	down
A_33_P3352782	TANGO6	0.2219305	0.1798244	0.0902057	-0.042106	-0.131725	-0.086915	down
A_23_P27584	MYADM	4.565116	4.57085	4.3855	0.005734	-0.179616	-0.086941	down
A_33_P3373273	SLC15A1	1.3881202	1.2661486	1.3361301	-0.121972	-0.05199	-0.086981	down
A_23_P18824	PAPD7	3.050932	2.9448771	2.9830074	-0.106055	-0.067925	-0.08699	down
A_33_P3342081	PRDM1	2.1757898	2.1235604	2.0539074	-0.052229	-0.121882	-0.087056	down
A_33_P3738458	TNS1	0.936296	0.7415581	0.9569149	-0.194738	0.0206189	-0.087059	down
A_33_P3263423	TLR5	-0.806109	-0.90309	-0.883381	-0.096981	-0.077271	-0.087126	down
A_33_P3316639	CHMP3	3.4428844	3.4361882	3.2752848	-0.006696	-0.1676	-0.087148	down
A_33_P3232280	ASUN	2.1642523	1.8764539	2.277752	-0.287798	0.1134996	-0.087149	down
A_23_P140748	NDRG4	2.1056795	1.8269806	2.2098951	-0.278699	0.1042156	-0.087242	down
A_23_P65733	ZNF770	0.1841507	0.1666527	0.0270877	-0.017498	-0.157063	-0.087281	down
A_23_P340251	RAB2A	3.8601599	3.5258546	4.0194063	-0.334305	0.1592464	-0.087529	down
A_23_P151093	YARS2	3.0606565	2.717276	3.2289734	-0.34338	0.1683168	-0.087532	down
A_24_P77364	BOLA3	6.948699	6.522942	7.199332	-0.425757	0.2506332	-0.087562	down
A_23_P160729	AP4B1	1.3788757	1.3187199	1.2638569	-0.060156	-0.115019	-0.087587	down
A_23_P146417	TMEM245	3.9149904	3.9271836	3.7275343	0.0121932	-0.187456	-0.087631	down
A_33_P3381245	PDCD5	7.26532	7.0171638	7.3381004	-0.248156	0.0727806	-0.087688	down
A_23_P100203	HSBP1	3.760663	3.555509	3.79043	-0.205154	0.029767	-0.087693	down
A_23_P118693	FOXN1	-1.499737	-1.596042	-1.57882	-0.096305	-0.079083	-0.087694	down
A_22_P00016875	lnc-TSC2-1	0.0857916	-0.236979	0.2329712	-0.322771	0.1471796	-0.087796	down
A_24_P289178	C16orf74	2.4693089	2.4520602	2.3105326	-0.017249	-0.158776	-0.088012	down
A_24_P375322	GGT8P	0.987844	1.2089686	0.5906167	0.2211247	-0.397227	-0.088051	down
A_23_P124476	CLCN3	1.9401236	1.9304242	1.7735562	-0.009699	-0.166567	-0.088133	down
A_19_P00321076	PVT1	-0.230135	-0.290424	-0.34621	-0.060289	-0.116075	-0.088182	down
A_23_P41075	MON1A	2.8008556	2.3430638	3.0822172	-0.457792	0.2813616	-0.088215	down
A_22_P00021779	lnc-C16orf42-1	-1.428423	-1.498922	-1.534491	-0.070499	-0.106069	-0.088284	down
A_32_P40744	LOC101927085	-0.998869	-1.119008	-1.055319	-0.120138	-0.05645	-0.088294	down

A_21_P0013181	LOC100506098	1.9044418	1.5932493	2.0389576	-0.311193	0.1345158	-0.088338	down
A_23_P164528	WDR7	1.9025164	1.7219205	1.9062853	-0.180596	0.0037689	-0.088413	down
A_33_P3258747	EVA1C	0.6311665	0.6926308	0.3927617	0.0614643	-0.238405	-0.08847	down
A_32_P164246	FOXQ1	5.98326	6.0663543	5.7230825	0.0830941	-0.260178	-0.088542	down
A_23_P164148	MLX	2.6536217	2.4267125	2.7033978	-0.226909	0.0497761	-0.088567	down
A_24_P734720	CCNYL1	0.01788	0.0486574	-0.190163	0.0307775	-0.208043	-0.088633	down
A_33_P3378689	WAC	2.5873766	2.5517144	2.4457226	-0.035662	-0.141654	-0.088658	down
A_23_P36753	ALDH2	4.656534	4.803019	4.3326073	0.1464849	-0.323927	-0.088721	down
A_33_P3403459	DHRS4-AS1	0.4233694	0.1226554	0.5465632	-0.300714	0.1231937	-0.08876	down
A_33_P3327822	SH3BGR	-1.306193	-1.17997	-1.609994	0.1262236	-0.303801	-0.088789	down
A_24_P945181	RBM15B	-2.415116	-2.499986	-2.50785	-0.08487	-0.092733	-0.088801	down
A_19_P00315824	PCAT19	0.8341618	0.7104087	0.7801905	-0.123753	-0.053971	-0.088862	down
A_33_P3313889	TOR1A	2.9553976	3.024963	2.708088	0.0695653	-0.24731	-0.088872	down
A_22_P00025883	lnc-FBXO47-1	-1.766467	-1.630479	-2.080208	0.1359878	-0.313741	-0.088877	down
A_23_P214681	PPARD	-0.869635	-1.293831	-0.623223	-0.424196	0.2464118	-0.088892	down
A_23_P110492	MARCH6	3.3107672	3.0886493	3.3549728	-0.222118	0.0442057	-0.088956	down
A_33_P3225587	NAA50	3.8857927	3.7131562	3.8804798	-0.172637	-0.005313	-0.088975	down
A_21_P0008936	LOC101927334	-2.171506	-2.349192	-2.171881	-0.177687	-0.000375	-0.089031	down
A_33_P3335935	HN1L	1.225935	0.988987	1.28475	-0.236948	0.058815	-0.089067	down
A_33_P3423171	WDR6	-2.187045	-2.050773	-2.501564	0.1362724	-0.314518	-0.089123	down
A_32_P110390	TMEM171	0.7827692	0.3852887	1.0019684	-0.397481	0.2191992	-0.089141	down
A_23_P150935	TROAP	4.276018	4.1419387	4.231571	-0.134079	-0.044447	-0.089263	down
A_23_P1361	ALDH18A1	3.0706253	2.9953208	2.9673967	-0.075305	-0.103229	-0.089267	down
A_33_P3420204	CRTC1	1.9469957	1.6884637	2.0268164	-0.258532	0.0798206	-0.089356	down
A_24_P298360	LTBP3	3.4111118	3.3565516	3.2867088	-0.05456	-0.124403	-0.089482	down
A_23_P418413	OXSR1	3.212988	3.3235154	2.923483	0.1105275	-0.289505	-0.089489	down
A_24_P393565	ZNF396	-2.567143	-2.586472	-2.726857	-0.01933	-0.159714	-0.089522	down
A_23_P203137	UBE4A	4.7242937	4.502272	4.767213	-0.222022	0.0429192	-0.089551	down
A_23_P30020	PLA2G12A	3.403349	3.2349973	3.3925323	-0.168352	-0.010817	-0.089584	down
A_32_P134209	ACVR2B	-1.305011	-1.309286	-1.480024	-0.004275	-0.175013	-0.089644	down
A_33_P3311907	TBC1D7	0.391325	0.3521972	0.2508674	-0.039128	-0.140458	-0.089793	down
A_23_P21776	SNAPC5	2.59167	2.2897115	2.7139997	-0.301959	0.1223297	-0.089814	down
A_23_P250948	BRPF1	0.186204	0.0073528	0.1853757	-0.178851	-0.000828	-0.08984	down
A_23_P350754	OR7E14P	1.2322631	1.267983	1.0168271	0.0357199	-0.215436	-0.089858	down
A_24_P341504	RNPC3	0.9849501	0.9859204	0.8040299	0.0009704	-0.18092	-0.089975	down
A_33_P3422679	FAM149B1	0.0340362	0.0090809	-0.121032	-0.024955	-0.155068	-0.090012	down
A_33_P3317321	TPMT	1.0571547	0.8807426	1.0533109	-0.176412	-0.003844	-0.090128	down
A_33_P3273664	ARSK	-2.010595	-2.383008	-1.818468	-0.372413	0.1921268	-0.090143	down
A_24_P201404	C11orf54	2.0175095	2.0379233	1.81667	0.0204139	-0.20084	-0.090213	down
A_23_P154740	KIZ	1.6811485	1.717165	1.4645596	0.0360165	-0.216589	-0.090286	down
A_21_P0010886	XLOC_l2_002033	2.4808693	2.2918172	2.489334	-0.189052	0.0084648	-0.090294	down
A_21_P0000607	ST3GAL4-AS1	0.5078993	0.2736759	0.5614672	-0.234223	0.0535679	-0.090328	down
A_23_P29769	WWTR1	1.0162749	0.7112832	1.1406016	-0.304992	0.1243267	-0.090333	down
A_22_P00011733	lnc-PELI2-5	-1.117125	-1.300094	-1.114904	-0.182969	0.0022206	-0.090374	down
A_22_P00011200	lnc-OLFML2A-1	-2.096966	-1.991827	-2.382903	0.1051393	-0.285937	-0.090399	down
A_21_P0010577	XLOC_l2_000641	-1.513754	-1.686676	-1.521654	-0.172921	-0.007899	-0.09041	down
A_22_P00018906	lnc-TOMM20L-1	-0.937775	-0.875401	-1.181043	0.0623746	-0.243268	-0.090446	down
A_33_P3250356	SH3D21	-2.621028	-2.879995	-2.543304	-0.258966	0.0777242	-0.090621	down
A_22_P00021933	P4HA2	3.6036482	3.5545392	3.4714622	-0.049109	-0.132186	-0.090647	down
A_33_P3220723	KIAA0922	1.8044319	1.6539502	1.7735825	-0.150482	-0.030849	-0.090666	down
A_33_P3404844	SRCIN1	-2.649419	-2.912759	-2.56743	-0.26334	0.0819888	-0.090676	down
A_23_P60565	ZNF354A	-0.681375	-0.703922	-0.840249	-0.022547	-0.158875	-0.090711	down
A_33_P3311956	FEZ2	-0.549435	-0.402653	-0.877705	0.1467814	-0.32827	-0.090745	down
A_23_P87575	CCNT1	-0.810967	-0.955392	-0.848212	-0.144425	-0.037246	-0.090836	down
A_22_P00003933	LOC102723826	2.3492727	2.2622285	2.2546082	-0.087044	-0.094665	-0.090854	down
A_33_P3310674	UBE2D2	5.0801926	4.940745	5.0379314	-0.139448	-0.042261	-0.090854	down

A_23_P71440	GOLGA7	4.5997505	4.3263755	4.6912565	-0.273375	0.091506	-0.090935	down
A_24_P191790	TMEM33	-0.169111	-0.359774	-0.160354	-0.190663	0.0087566	-0.090953	down
A_33_P3337540	IFNAR2	3.1674337	2.98422	3.1686563	-0.183214	0.0012226	-0.090996	down
A_24_P332647	SSH1	-1.267224	-1.679538	-1.036923	-0.412314	0.2303009	-0.091007	down
A_33_P3338733	MITF	-0.886166	-0.947623	-1.006772	-0.061457	-0.120605	-0.091031	down
A_33_P3322328	EPS15	1.7503471	1.5848885	1.7337179	-0.165459	-0.016629	-0.091044	down
A_32_P2738	TCTN3	3.6354818	3.3992658	3.6895885	-0.236216	0.0541067	-0.091055	down
A_24_P233786	FAM129A	2.7533522	3.403174	1.9213781	0.6498218	-0.831974	-0.091076	down
A_23_P136232	IMPAD1	1.328846	1.3250403	1.1503844	-0.003806	-0.178462	-0.091134	down
A_24_P183994	RASEF	-2.016747	-1.937884	-2.27806	0.0788627	-0.261313	-0.091225	down
A_23_P19517	ITPR3	3.4030352	3.3646874	3.2588787	-0.038348	-0.144156	-0.091252	down
A_21_P0010328	LOC101928891	-1.195456	-1.375386	-1.198154	-0.17993	-0.002697	-0.091314	down
A_24_P79712	COX20	2.5916824	2.3111062	2.6894274	-0.280576	0.0977449	-0.091416	down
A_24_P23258	GRAMD4	3.181819	2.925157	3.2555819	-0.256662	0.0737629	-0.09145	down
A_33_P3237125	ANKRD54	-1.25811	-1.278486	-1.420652	-0.020376	-0.162542	-0.091459	down
A_24_P91916	NXT2	0.3906617	0.2841382	0.3142476	-0.106524	-0.076414	-0.091469	down
A_21_P0011309	HERC2P10	-2.236633	-2.202961	-2.453442	0.0336728	-0.216808	-0.091568	down
A_33_P3320772	ATXN1	-1.027839	-0.911899	-1.326969	0.1159401	-0.29913	-0.091595	down
A_33_P3416448	BTBD7	-2.417961	-2.538899	-2.48026	-0.120938	-0.062299	-0.091619	down
A_32_P129752	TMEM30B	4.067958	3.9179873	4.0346622	-0.149971	-0.033296	-0.091633	down
A_23_P170587	SMYD2	3.5473557	3.392014	3.519187	-0.155342	-0.028169	-0.091755	down
A_24_P162293	ERC1	-1.012088	-1.076417	-1.131568	-0.06433	-0.11948	-0.091905	down
A_23_P156327	TGFBI	8.459811	8.424601	8.311089	-0.035211	-0.148723	-0.091967	down
A_33_P3223239	ST7L	-0.218946	-0.243727	-0.378154	-0.024781	-0.159208	-0.091995	down
A_33_P3281940	RPE	2.0879822	1.9455781	2.0463476	-0.142404	-0.041635	-0.092019	down
A_23_P6223	SCAF4	1.4348407	1.3385315	1.3470716	-0.096309	-0.087769	-0.092039	down
A_23_P209978	VSNL1	3.3103027	3.0737996	3.3627071	-0.236503	0.0524044	-0.092049	down
A_33_P3407675	TIPRL	3.0042448	2.8651776	2.959176	-0.139067	-0.045069	-0.092068	down
A_32_P22622	NOP14	-0.294538	-0.533982	-0.239262	-0.239444	0.0552754	-0.092084	down
A_23_P7101	SLBP	4.1692295	4.1245427	4.0296135	-0.044687	-0.139616	-0.092151	down
A_23_P44964	FAM171A1	2.3647099	2.3144994	2.2305956	-0.05021	-0.134114	-0.092162	down
A_33_P3358307	OTUD7B	1.2840376	1.1729326	1.2102017	-0.111105	-0.073836	-0.09247	down
A_33_P3216277	LOC101059976	-2.459773	-2.882851	-2.221659	-0.423079	0.2381136	-0.092482	down
A_23_P401568	MAT2A	4.81279	4.665338	4.775261	-0.147452	-0.037529	-0.09249	down
A_23_P200199	TXNDC12	2.754902	2.5459113	2.7788544	-0.208991	0.0239525	-0.092519	down
A_21_P0000870	SEC24B-AS1	-1.899496	-2.218262	-1.765866	-0.318765	0.1336303	-0.092568	down
A_33_P3259705	SIGIRR	-2.080326	-2.317353	-2.028437	-0.237027	0.0518889	-0.092569	down
A_22_P00013800	lnc-RP4-604K5.1.1-2	0.8251243	0.5903196	0.8747892	-0.234805	0.049665	-0.09257	down
A_33_P3222788	HECTD2-AS1	-0.698982	-0.743122	-0.840445	-0.04414	-0.141463	-0.092802	down
A_23_P397341	PAQR4	0.9210749	0.4357643	1.220727	-0.485311	0.2996521	-0.092829	down
A_24_P149124	NREP	2.44419	2.6991343	2.003563	0.2549443	-0.440627	-0.092841	down
A_23_P89509	SPAG5	4.9578295	4.7930655	4.9367075	-0.164764	-0.021122	-0.092943	down
A_33_P3225630	LOC400706	0.2903972	0.1442123	0.250607	-0.146185	-0.03979	-0.092988	down
A_24_P183864	IMPA1	3.6094599	3.306292	3.7265587	-0.303168	0.1170988	-0.093035	down
A_23_P375549	FUNDC1	2.111741	1.958427	2.0789185	-0.153314	-0.032823	-0.093068	down
A_33_P3214665	MAP2	3.4265137	3.4337668	3.2330656	0.0072532	-0.193448	-0.093097	down
A_23_P80643	SETMAR	1.1241808	0.9632535	1.0988207	-0.160927	-0.02536	-0.093144	down
A_23_P416212	HSPB9	-2.862438	-2.796797	-3.114458	0.0656405	-0.25202	-0.09319	down
A_33_P3347397	AMOT	-1.176188	-1.20081	-1.337947	-0.024622	-0.161759	-0.093191	down
A_33_P3329974	CGN	4.6336718	4.00155	5.0791883	-0.632122	0.4455166	-0.093302	down
A_33_P3416588	RIT2	-2.011545	-1.916259	-2.293936	0.0952859	-0.282391	-0.093553	down
A_24_P940149	C2CD2	0.4370093	0.5465245	0.140377	0.1095152	-0.296632	-0.093559	down
A_23_P50368	OSCAR	0.3166289	0.1422281	0.3038907	-0.174401	-0.012738	-0.09357	down
A_22_P00017514	INTS6-AS1	-1.429586	-1.54364	-1.502803	-0.114053	-0.073217	-0.093635	down

A_21_P0001010	lnc-RP3-377D14.1.1-7	-0.732276	-0.996659	-0.655219	-0.264383	0.0770574	-0.093663	down
A_23_P338603	CHCHD7	2.6551485	2.520883	2.601964	-0.134265	-0.053185	-0.093725	down
A_33_P3210288	FUT6	10.014901	9.97427	9.867891	-0.040631	-0.14701	-0.093821	down
A_24_P250535	TMX4	3.331956	3.2555757	3.2206316	-0.07638	-0.111324	-0.093852	down
A_21_P0005794	lnc-PRAGMIN.1-3	-0.536068	-0.632077	-0.627786	-0.096009	-0.091718	-0.093863	down
A_33_P3241657	CAMKMT	0.5556541	0.5554605	0.3680863	-0.000194	-0.187568	-0.093881	down
A_33_P3210338	RFX7	2.4855518	2.487451	2.2958536	0.0018992	-0.189698	-0.093899	down
A_24_P166094	ARFIP1	4.2351418	3.9666667	4.315586	-0.268475	0.0804443	-0.094015	down
A_23_P163546	TAF1C	3.7412548	3.6753488	3.619113	-0.065906	-0.122142	-0.094024	down
A_23_P112652	CNOT10	3.5041704	3.3454003	3.4747276	-0.15877	-0.029443	-0.094106	down
A_33_P3256920	WNT7B	-2.056466	-2.324354	-1.976831	-0.267888	0.0796351	-0.094126	down
A_24_P380536	CD164	3.254448	3.1851735	3.1354475	-0.069274	-0.119	-0.094137	down
A_33_P3222698	ZNF197	-1.174175	-1.224516	-1.312304	-0.050341	-0.138129	-0.094235	down
A_33_P3341234	UBE2H	2.5501423	2.5152564	2.3962994	-0.034886	-0.153843	-0.094364	down
A_32_P353798	PTDSS2	3.9789524	3.80224	3.9668932	-0.176713	-0.012059	-0.094386	down
A_32_P203528	SYCE2	-1.65507	-1.413602	-2.085813	0.241468	-0.430743	-0.094637	down
A_24_P143076	FAM109A	0.7126579	0.3807306	0.8552809	-0.331927	0.142623	-0.094652	down
A_22_P00003377	CCDC102B	-2.707342	-2.523972	-3.080026	0.1833701	-0.372684	-0.094657	down
A_24_P927716	SLED1	-1.097322	-1.840879	-0.543079	-0.743558	0.5542426	-0.094658	down
A_24_P40721	SPTLC3	-1.574127	-1.654664	-1.6831	-0.080537	-0.108973	-0.094755	down
A_24_P209285	MRPS10	4.734889	4.6681485	4.6120653	-0.066741	-0.122824	-0.094782	down
A_24_P133488	CDCA4	0.1344676	-0.113611	0.1929174	-0.248079	0.0584497	-0.094815	down
A_22_P00008271	CRNDE	1.2054939	1.3199992	0.9010773	0.1145053	-0.304417	-0.094956	down
A_33_P3345294	CCDC162P	-1.845335	-1.819641	-2.061034	0.0256939	-0.215699	-0.095002	down
A_33_P3319760	TAGLN2	5.639385	5.3417172	5.7470255	-0.297668	0.1076403	-0.095014	down
A_22_P00004515	lnc-CPVL-4	-1.405075	-1.529972	-1.470207	-0.124897	-0.065132	-0.095014	down
A_33_P3287883	LOC100133331	1.1703668	1.0989747	1.0516791	-0.071392	-0.118688	-0.09504	down
A_24_P288836	HLA-DPB2	-2.591615	-2.700248	-2.673085	-0.108633	-0.08147	-0.095051	down
A_33_P3251989	CRIPT	3.0873861	2.9584603	3.026205	-0.128926	-0.061181	-0.095053	down
A_23_P209805	NAB1	3.3414373	3.1394496	3.353241	-0.201988	0.0118036	-0.095092	down
A_23_P345591	PSMA2	7.032073	6.963079	6.9108467	-0.068994	-0.121226	-0.09511	down
A_21_P0009241	lnc-MRM1-1	-2.288178	-2.404104	-2.362598	-0.115925	-0.07442	-0.095172	down
A_22_P00022265	lnc-C11orf36-2	-3.070711	-3.257693	-3.074598	-0.186981	-0.003886	-0.095434	down
A_24_P190168	TMEM97	4.463539	4.215902	4.520299	-0.247637	0.0567598	-0.095439	down
A_23_P10870	DOLK	3.2126436	2.9284177	3.3058357	-0.284226	0.0931921	-0.095517	down
A_33_P3228271	CST3	0.2235422	0.0516863	0.2042322	-0.171856	-0.01931	-0.095583	down
A_23_P20248	MAP2K1	3.340539	3.2775111	3.2123413	-0.063028	-0.128198	-0.095613	down
A_21_P0011427	ZNF720	-1.450938	-1.629052	-1.464146	-0.178114	-0.013207	-0.095661	down
A_32_P36694	JAZF1	-1.549468	-1.638711	-1.651555	-0.089242	-0.102087	-0.095665	down
A_22_P00012227	lnc-PPIA-1	3.1777	3.2193246	2.9447002	0.0416245	-0.233	-0.095688	down
A_33_P3287770	TELO2	5.066695	4.795481	5.1464424	-0.271214	0.0797472	-0.095733	down
A_23_P428382	AFTPH	3.0599499	2.998962	2.9294329	-0.060988	-0.130517	-0.095752	down
A_33_P3239512	ZNF836	-1.801668	-1.989728	-1.80523	-0.18806	-0.003561	-0.095811	down
A_22_P00012392	lnc-PRKACG-3	-1.624061	-1.734946	-1.704885	-0.110885	-0.080824	-0.095855	down
A_23_P65674	TMOD3	3.4312534	3.304339	3.3660994	-0.126915	-0.065154	-0.096034	down
A_23_P64617	FZD4	0.5948157	0.4428706	0.5546889	-0.151945	-0.040127	-0.096036	down
A_23_P59950	SLC39A14	3.9807634	3.9338088	3.835329	-0.046955	-0.145434	-0.096195	down
A_23_P116207	IFT46	2.1349268	2.0576777	2.0197592	-0.077249	-0.115168	-0.096208	down
A_21_P0006268	lnc-FAM75A6-1	-2.077742	-2.35661	-1.991317	-0.278868	0.0864244	-0.096222	down
A_23_P162734	RNF6	2.9967175	2.6451669	3.1555815	-0.351551	0.158864	-0.096343	down
A_21_P0007761	MAPKAPK5-AS1	2.3459187	2.1329656	2.365696	-0.212953	0.0197773	-0.096588	down
A_22_P00013094	lnc-RIIAD1-1	-2.271255	-2.348548	-2.387163	-0.077293	-0.115908	-0.0966	down
A_24_P374586	PMS2	0.7543149	0.5278177	0.7876058	-0.226497	0.0332909	-0.096603	down
A_33_P3222937	TMEM214	6.0807323	5.6630483	6.3047915	-0.417684	0.2240591	-0.096812	down
A_23_P68851	KREMEN1	4.152771	3.8609958	4.2509136	-0.291775	0.0981426	-0.096816	down

A_23_P344555	NEDD9	-0.76336	-0.89055	-0.829861	-0.12719	-0.066501	-0.096846	down
A_33_P3417950	AKAP8	4.1895924	3.9733405	4.212021	-0.216252	0.0224285	-0.096912	down
A_24_P77904	HOXA10	1.2876043	1.1942544	1.1870975	-0.09335	-0.100507	-0.096928	down
A_33_P3413808	PABPC1L	-2.544767	-2.118082	-3.165378	0.4266853	-0.620611	-0.096963	down
A_23_P78458	ZNF350	1.4912586	1.3783736	1.4102082	-0.112885	-0.08105	-0.096968	down
A_33_P3278118	CASP3	-0.535265	-0.622966	-0.641622	-0.087701	-0.106357	-0.097029	down
A_24_P41570	H2AFZ	7.712633	7.7404695	7.4907284	0.0278363	-0.221905	-0.097034	down
A_23_P81717	FRMD1	-1.954943	-2.134862	-1.969137	-0.179918	-0.014194	-0.097056	down
A_23_P108437	FZD5	1.4215403	1.48036	1.1685238	0.0588198	-0.253016	-0.097098	down
A_33_P3220020	GOLGA2P9	-2.899679	-2.908659	-3.084976	-0.00898	-0.185297	-0.097138	down
A_23_P11224	MMGT1	4.2510347	4.1010137	4.2066193	-0.150021	-0.044415	-0.097218	down
A_33_P3339397	SH3YL1	2.0826454	1.875287	2.095479	-0.207358	0.0128336	-0.097262	down
A_23_P257895	MAPK1	2.1594048	1.714304	2.4098167	-0.445101	0.250412	-0.097344	down
A_33_P3260026	AIF1L	-1.444651	-1.302235	-1.78181	0.1424155	-0.337159	-0.097372	down
A_33_P3394789	LOC100653296	0.224627	0.2591806	-0.005351	0.0345535	-0.229978	-0.097712	down
A_22_P00003929	lnc-CFH-1	-2.387863	-2.417885	-2.553416	-0.030022	-0.165554	-0.097788	down
A_33_P3333078	ARID1B	0.0356541	-0.090327	-0.03417	-0.125981	-0.069824	-0.097903	down
A_23_P142634	METTL5	5.296382	5.1198297	5.2771196	-0.176552	-0.019262	-0.097907	down
A_33_P3352822	ZNF547	-0.309127	-0.405007	-0.409128	-0.09588	-0.1	-0.09794	down
A_21_P0000624	SLC6A13	-1.426955	-1.455277	-1.594584	-0.028322	-0.167629	-0.097975	down
A_24_P289139	SH3KBP1	1.8850107	1.9536338	1.6203742	0.0686231	-0.264637	-0.098007	down
A_33_P3356392	RGL3	-1.622512	-1.778023	-1.663051	-0.15551	-0.040538	-0.098024	down
A_33_P3212112	SLC12A6	0.4629045	0.5624747	0.1671467	0.0995703	-0.295758	-0.098094	down
A_23_P15493	PTRH2	5.7928324	5.5907464	5.7985277	-0.202086	0.0056953	-0.098195	down
A_33_P3339311	NUP133	-1.087007	-1.288797	-1.081794	-0.201791	0.0052123	-0.098289	down
A_33_P3355071	MLLT10	-0.769547	-0.905383	-0.830304	-0.135836	-0.060758	-0.098297	down
A_33_P3404899	LPHN2	-2.244227	-2.175946	-2.509315	0.0682817	-0.265087	-0.098403	down
A_33_P3388135	MKKS	5.2430105	5.085983	5.203186	-0.157028	-0.039824	-0.098426	down
A_33_P3239317	LINC01144	-0.902346	-0.930163	-1.071412	-0.027818	-0.169066	-0.098442	down
A_24_P381555	SAP18	0.8114648	0.6135807	0.8123865	-0.197884	0.0009217	-0.098481	down
A_23_P360626	PLD6	0.7903605	0.7440872	0.6396232	-0.046273	-0.150737	-0.098505	down
A_23_P96285	REEP1	-0.806502	-0.946208	-0.8641	-0.139706	-0.057599	-0.098652	down
A_21_P0000584	VTRNA2-1	1.8070192	1.6566954	1.7599373	-0.150324	-0.047082	-0.098703	down
A_23_P422981	FBXO36	0.1557369	0.1045723	0.0094042	-0.051165	-0.146333	-0.098749	down
A_23_P501435	CSRP2BP	4.0353184	4.1476626	3.7253838	0.1123443	-0.309935	-0.098795	down
A_33_P3212221	DEPDC5	-1.874587	-1.758491	-2.1883	0.1160955	-0.313714	-0.098809	down
A_23_P93792	PMS2	0.0086155	-0.048454	-0.132089	-0.05707	-0.140705	-0.098887	down
A_21_P0000341	SNORA61	3.0069408	2.9379153	2.8781662	-0.069026	-0.128775	-0.0989	down
A_23_P109427	GSTT2	-0.297057	-0.281077	-0.511013	0.0159798	-0.213956	-0.098988	down
A_33_P3292540	CDKN2C	2.2080193	2.0253758	2.192665	-0.182643	-0.015354	-0.098999	down
A_23_P354591	MVB12B	-1.197642	-1.311793	-1.281574	-0.114151	-0.083931	-0.099041	down
A_33_P3231432	LDHB	6.2885303	6.1574264	6.2214746	-0.131104	-0.067056	-0.09908	down
A_23_P209735	ARMC9	1.022954	0.7531428	1.0944705	-0.269811	0.0715165	-0.099147	down
A_33_P3364433	ACVR1B	-1.706036	-1.793549	-1.816982	-0.087513	-0.110946	-0.09923	down
A_21_P0006606	lnc-GYG2P1-1	0.5863495	0.5674329	0.406693	-0.018917	-0.179657	-0.099287	down
A_21_P0014018	LINC01311	-1.44566	-1.752849	-1.33713	-0.307189	0.10853	-0.09933	down
A_33_P3360942	SCAMP1	2.3580713	2.2074656	2.309866	-0.150606	-0.048205	-0.099406	down
A_33_P3329187	DNMT1	4.1250257	3.9107842	4.1404257	-0.214242	0.0153999	-0.099421	down
A_23_P132536	TRAK1	1.9806786	1.8731313	1.8892622	-0.107547	-0.091416	-0.099482	down
A_24_P253827	AP2B1	2.8287048	2.4761138	2.9823112	-0.352591	0.1536064	-0.099492	down
A_33_P3257903	GSTA4	0.1240945	0.0349622	0.0141711	-0.089132	-0.109923	-0.099528	down
A_23_P376870	C14orf79	-1.744287	-1.634057	-2.053613	0.1102304	-0.309326	-0.099548	down
A_33_P3291882	ARID1B	-2.022679	-2.432509	-1.812218	-0.40983	0.2104616	-0.099684	down
A_23_P123193	ACTR3B	0.9278345	0.8047371	0.8515606	-0.123097	-0.076274	-0.099686	down
A_33_P3766959	TDP1	2.302102	2.2505193	2.1541996	-0.051583	-0.147902	-0.099743	down
A_33_P3363420	FRMD3	-2.194134	-2.556713	-2.031072	-0.362579	0.1630621	-0.099758	down

A_21_P0012985	lnc-FGF10-3	1.9157047	1.7617621	1.8699989	-0.153943	-0.045706	-0.099824	down
A_23_P77160	RNF111	2.3382978	2.2666602	2.2101889	-0.071638	-0.128109	-0.099873	down
A_23_P121356	BBX	3.8153305	3.6476998	3.7831793	-0.167631	-0.032151	-0.099891	down
A_21_P0000158	XPNPEP3	1.9007168	1.4608798	2.1406403	-0.439837	0.2399235	-0.099957	down
A_21_P0010591	XLOC_l2_000727	-1.224004	-1.184019	-1.463967	0.0399852	-0.239963	-0.099989	down
A_23_P357811	MBNL1	3.9875975	3.9244056	3.850748	-0.063192	-0.136849	-0.100021	down
A_23_P92320	NUP54	3.381544	3.321405	3.2415218	-0.060139	-0.140022	-0.100081	down
A_24_P405002	PDIK1L	-0.672261	-0.574675	-0.970285	0.0975866	-0.298024	-0.100219	down
A_23_P166677	MFSD1	4.3569384	4.2703633	4.243065	-0.086575	-0.113873	-0.100224	down
A_23_P83939	SYAP1	4.35577	4.2012258	4.309801	-0.154544	-0.045969	-0.100257	down
A_33_P3299279	ANXA2R	0.9199653	0.7890062	0.8502798	-0.130959	-0.069685	-0.100322	down
A_33_P3234347	AMMECR1	2.9207926	2.8125978	2.8282022	-0.108195	-0.09259	-0.100393	down
A_33_P3252043	P4HB	8.031757	7.606832	8.255821	-0.424925	0.2240639	-0.100431	down
A_23_P301336	R3HCC1L	-1.024314	-1.137868	-1.111804	-0.113554	-0.08749	-0.100522	down
A_23_P92035	SETD5	1.1941242	0.9976535	1.1894908	-0.196471	-0.004633	-0.100552	down
A_23_P129466	ATF7IP2	1.3550634	1.0169501	1.4920692	-0.338113	0.1370058	-0.100554	down
A_33_P3230090	LUZP6	3.2457743	3.0509152	3.2395163	-0.194859	-0.006258	-0.100559	down
A_33_P3268284	GDI2	2.7913942	2.5691676	2.8124294	-0.222227	0.0210352	-0.100596	down
A_23_P58967	ZC3H11A	4.900156	4.942185	4.6567974	0.0420289	-0.243359	-0.100665	down
A_23_P165333	BIN1	0.0980444	0.0998693	-0.105305	0.0018249	-0.203349	-0.100762	down
A_33_P3404779	TCTEX1D4	-3.011218	-2.939662	-3.28443	0.0715563	-0.273212	-0.100828	down
A_33_P3414312	USP32	1.3618646	1.3408608	1.1810131	-0.021004	-0.180851	-0.100928	down
A_24_P854913	METTL21A	2.528925	2.3632264	2.4927626	-0.165699	-0.036162	-0.10093	down
A_33_P3255631	ZNF780A	0.1878896	0.1770134	-0.003246	-0.010876	-0.191136	-0.101006	down
A_23_P24515	ACAT1	4.295973	4.183851	4.2060137	-0.112122	-0.089959	-0.101041	down
A_23_P397899	INIP	1.8317757	1.5859079	1.8755217	-0.245868	0.043746	-0.101061	down
A_33_P3269723	ZSWIM7	1.1123161	1.3296614	0.6927118	0.2173452	-0.419604	-0.10113	down
A_21_P0000267	SNORD72	-3.101142	-3.158633	-3.246167	-0.057491	-0.145026	-0.101258	down
A_24_P322354	SKA1	-0.020045	-0.05151	-0.191136	-0.031466	-0.171091	-0.101278	down
A_23_P345081	ZNF655	1.5121121	1.6844573	1.1370077	0.1723452	-0.375104	-0.10138	down
A_33_P3340682	ZNF276	0.1190667	-0.067183	0.1025047	-0.18625	-0.016562	-0.101406	down
A_24_P301846	GART	1.9843283	1.6453128	2.120325	-0.339015	0.1359968	-0.101509	down
A_24_P254965	HDAC8	0.8023133	0.6891694	0.7123456	-0.113144	-0.089968	-0.101556	down
A_33_P3233947	COX20	4.4253645	4.2524724	4.395006	-0.172892	-0.030358	-0.101625	down
A_23_P81392	WWC1	4.6610937	4.5157523	4.6031313	-0.145341	-0.057962	-0.101652	down
A_32_P126311	SLC38A9	3.2694998	3.2503657	3.0851173	-0.019134	-0.184382	-0.101758	down
A_32_P66020	SNX29	1.0118365	0.9912462	0.8288956	-0.02059	-0.182941	-0.101766	down
A_33_P3324004	CCL15	-0.527461	-0.844674	-0.413899	-0.317214	0.1135612	-0.101826	down
A_22_P00014886	lnc-SLC7A1-2	-2.813627	-2.803984	-3.026982	0.0096426	-0.213355	-0.101856	down
A_23_P349882	PDCL	0.9924264	0.7212348	1.0598812	-0.271192	0.0674548	-0.101868	down
A_33_P3209025	ZNF623	-2.111851	-2.17849	-2.248958	-0.066638	-0.137107	-0.101873	down
A_23_P117782	LARP6	3.129405	3.0463138	3.0087337	-0.083091	-0.120671	-0.101881	down
A_23_P208009	SEC11C	5.785652	5.7442527	5.6232405	-0.041399	-0.162412	-0.101906	down
A_24_P178423	HNRNPC	4.8650255	4.683965	4.8422003	-0.18106	-0.022825	-0.101943	down
A_22_P00024857	LOC100288798	-1.593041	-1.726509	-1.663616	-0.133469	-0.070576	-0.102022	down
A_32_P24585	SH3PXD2B	0.4271278	0.1930375	0.4571314	-0.23409	0.0300035	-0.102043	down
A_23_P211227	POFUT2	-2.422973	-2.377223	-2.672887	0.0457494	-0.249914	-0.102082	down
A_19_P00322533	CRNDE	2.756053	2.810678	2.4969473	0.054625	-0.259106	-0.10224	down
A_21_P0006929	lnc-GPR26-4	-2.061643	-2.145232	-2.182562	-0.083589	-0.12092	-0.102254	down
A_23_P64372	TCN1	-2.216384	-2.139063	-2.498584	0.0773208	-0.2822	-0.102439	down
A_33_P3389178	RAPGEF3	1.0681124	0.9538941	0.9772034	-0.114218	-0.090909	-0.102564	down
A_24_P36890	RAP1GAP	-1.366713	-1.597584	-1.34127	-0.230871	0.0254431	-0.102714	down
A_33_P3223467	VCPIP1	0.3676281	0.2901611	0.2393327	-0.077467	-0.128295	-0.102881	down
A_23_P218158	SMEK1	1.9467201	1.9819627	1.7057061	0.0352426	-0.241014	-0.102886	down
A_23_P214046	FBXW11	4.5794973	4.37682	4.576206	-0.202677	-0.003291	-0.102984	down
A_23_P347198	SP3	2.9162378	2.9255462	2.7009544	0.0093083	-0.215283	-0.102988	down

A_24_P76879	TSPYL1	4.713234	4.519525	4.70092	-0.193709	-0.012314	-0.103011	down
A_23_P5551	NCL	7.898328	7.845986	7.7445183	-0.052342	-0.15381	-0.103076	down
A_33_P3237225	FBXO34	2.7151814	2.5990195	2.625042	-0.116162	-0.090139	-0.103151	down
A_22_P00010713	lnc-NGFR-1	-1.509776	-1.684864	-1.541078	-0.175087	-0.031302	-0.103194	down
A_24_P42681	PSMD2	4.8706064	4.757067	4.7775526	-0.113539	-0.093054	-0.103297	down
A_23_P114095	MBTPS2	0.4364343	0.3172035	0.3490596	-0.119231	-0.087375	-0.103303	down
A_33_P3362886	lnc-COPZ2-1	0.3245463	0.2766867	0.1657109	-0.04786	-0.158835	-0.103348	down
A_24_P271323	SLC2A10	-0.016979	-0.410591	0.1695137	-0.393612	0.1864924	-0.10356	down
A_23_P69100	ARHGEF26	-0.716041	-0.953521	-0.685784	-0.237481	0.0302567	-0.103612	down
A_33_P3353692	MYH9	0.6524744	0.4781017	0.619585	-0.174373	-0.032889	-0.103631	down
A_33_P3235640	RBBP5	-2.483328	-2.787672	-2.38636	-0.304344	0.0969682	-0.103688	down
A_33_P3210986	TYW1	-0.189891	-0.246958	-0.34076	-0.057067	-0.150869	-0.103968	down
A_22_P00000046	HOXA-AS2	-2.378359	-2.634479	-2.33032	-0.25612	0.048039	-0.10404	down
A_33_P3233891	SRP72	2.7498074	2.5888848	2.7025557	-0.160923	-0.047252	-0.104087	down
A_22_P00011759	lnc-PEX7-2	0.5020199	0.3009052	0.4945164	-0.201115	-0.007504	-0.104309	down
A_23_P407684	ZNF598	2.102415	1.8531351	2.1430674	-0.24928	0.0406523	-0.104314	down
A_24_P185986	UPRT	1.7858086	1.6379128	1.7248192	-0.147896	-0.060989	-0.104443	down
A_24_P212539	GALM	-1.494611	-1.68772	-1.510447	-0.193109	-0.015836	-0.104472	down
A_33_P3403598	TMEM120B	-1.792851	-2.204745	-1.589934	-0.411894	0.2029162	-0.104489	down
A_23_P146922	GAS6	-1.284408	-1.296734	-1.48117	-0.012326	-0.196762	-0.104544	down
A_24_P926400	GGA1	-0.122573	-0.099103	-0.355298	0.0234704	-0.232725	-0.104627	down
A_33_P3296940	FNDC3B	1.2301064	1.1701355	1.0808148	-0.059971	-0.149292	-0.104631	down
A_33_P3302681	TLE4	-1.349943	-1.665049	-1.244126	-0.315106	0.1058173	-0.104644	down
A_23_P103503	MDM4	1.0468597	1.1603122	0.7240548	0.1134524	-0.322805	-0.104676	down
A_23_P128215	SOCS2	2.86701	2.4864507	3.038128	-0.380559	0.1711178	-0.104721	down
A_33_P3278455	ANKRD6	-2.551868	-2.942398	-2.370884	-0.390529	0.1809843	-0.104773	down
A_23_P55601	ZNF236	-1.413549	-1.425979	-1.61069	-0.012431	-0.197141	-0.104786	down
A_33_P3255229	SETD7	0.1164093	-0.191561	0.2147927	-0.307971	0.0983834	-0.104794	down
A_23_P159937	SLC6A8	1.7423887	1.5847616	1.6902871	-0.157627	-0.052102	-0.104864	down
A_33_P3395848	VSIG10L	-1.669397	-2.60137	-0.947283	-0.931972	0.7221146	-0.104929	down
A_23_P256641	KCNE5	-2.478626	-2.846161	-2.321432	-0.367536	0.1571934	-0.105171	down
A_21_P0000742	MYLK-AS1	0.3119516	0.4557924	-0.0425	0.1438408	-0.354451	-0.105305	down
A_23_P411881	PIBF1	0.1124392	0.0657864	-0.051521	-0.046653	-0.16396	-0.105306	down
A_33_P3211203	LOC101927919	1.1158996	1.046566	0.9746175	-0.069334	-0.141282	-0.105308	down
A_33_P3303687	PSMC1	3.311676	3.210588	3.2021475	-0.101088	-0.109529	-0.105308	down
A_24_P88554	PEX11B	1.9618282	1.4108124	2.3020897	-0.551016	0.3402615	-0.105377	down
A_33_P3211818	RUNX1	-2.078478	-2.021308	-2.34643	0.0571699	-0.267952	-0.105391	down
A_33_P3328937	TLK2	-1.489254	-1.415924	-1.773478	0.0733299	-0.284225	-0.105447	down
A_23_P304716	HES2	-0.611582	-0.946456	-0.48764	-0.334874	0.1239424	-0.105466	down
A_23_P35045	ARHGAP30	-2.20046	-1.917747	-2.694177	0.2827129	-0.493717	-0.105502	down
A_24_P320880	SNX16	-1.688309	-1.644145	-1.943513	0.0441642	-0.255204	-0.10552	down
A_23_P17855	TRIOBP	1.103581	1.2106814	0.7854266	0.1071005	-0.318154	-0.105527	down
A_33_P3385477	ATAD3B	5.743806	5.4798064	5.796708	-0.263999	0.0529022	-0.105549	down
A_23_P24884	ST5	0.6751585	0.4974132	0.6417203	-0.177745	-0.033438	-0.105592	down
A_24_P633902	RNF115	1.245688	1.0574703	1.2225933	-0.188218	-0.023095	-0.105656	down
A_33_P3241753	NDUFA12	5.8880196	5.653787	5.91084	-0.234232	0.0228205	-0.105706	down
A_22_P00002039	RASSF8-AS1	-1.052033	-1.627869	-0.687749	-0.575836	0.364284	-0.105776	down
A_33_P3349521	SARAF	5.151971	4.919617	5.1727705	-0.232354	0.0207996	-0.105777	down
A_23_P46539	PSRC1	1.3997178	1.0905089	1.4972496	-0.309209	0.0975318	-0.105839	down
A_21_P0000073	OSMR	0.2569213	-0.171453	0.4734387	-0.428374	0.2165175	-0.105928	down
A_33_P3407925	VMP1	4.685176	4.523002	4.635475	-0.162174	-0.049701	-0.105937	down
A_33_P3364520	KRTAP6-1	-2.257575	-2.402381	-2.324655	-0.144806	-0.06708	-0.105943	down
A_23_P127095	USMG5	8.850962	8.610664	8.879091	-0.240297	0.0281296	-0.106084	down
A_22_P00004238	lnc-CMBL-1	0.0630679	-0.155129	0.0689769	-0.218197	0.005909	-0.106144	down
A_22_P00017475	LOC102725381	1.6134624	1.3424444	1.6719179	-0.271018	0.0584555	-0.106281	down
A_24_P39101	KCTD10	1.0038104	0.743485	1.0513067	-0.260325	0.0474963	-0.106415	down

A_24_P254551	ARHGEF9	-1.007189	-0.555613	-1.671618	0.4515762	-0.664429	-0.106426	down
A_23_P123086	PSMG3-AS1	-1.350164	-1.439927	-1.473281	-0.089763	-0.123117	-0.10644	down
A_23_P89123	CFDP1	2.1910858	2.0962014	2.0730581	-0.094884	-0.118028	-0.106456	down
A_32_P524614	C17orf74	-0.879369	-1.091081	-0.88063	-0.211712	-0.00126	-0.106486	down
A_22_P00001203	CAPN10-AS1	-1.911024	-2.291338	-1.743889	-0.380314	0.1671352	-0.106589	down
A_22_P00022727	lnc-TAF6-1	-2.964799	-2.862778	-3.280093	0.1020217	-0.315293	-0.106636	down
A_21_P0009079	lnc-GINS2-1	-0.394039	-0.648662	-0.352982	-0.254623	0.0410576	-0.106783	down
A_24_P49517	ZNF251	0.1848531	0.2933736	-0.137253	0.1085205	-0.322106	-0.106793	down
A_33_P3332744	CYTH1	-0.725288	-0.822864	-0.841312	-0.097575	-0.116024	-0.106799	down
A_23_P121082	GBE1	2.3305979	2.345242	2.1023188	0.0146441	-0.228279	-0.106817	down
A_23_P354705	ST8SIA1	-1.151958	-1.133329	-1.384285	0.0186286	-0.232327	-0.106849	down
A_33_P3364240	PAEP	-2.545222	-2.572841	-2.731331	-0.027619	-0.18611	-0.106864	down
A_23_P25293	NCAPD2	2.7198267	2.5224557	2.703436	-0.197371	-0.016391	-0.106881	down
A_33_P3211956	RGS19	3.6105661	3.1653333	3.8419876	-0.445233	0.2314215	-0.106906	down
A_22_P00009437	SVIL-AS1	0.8814659	0.602705	0.9464002	-0.278761	0.0649343	-0.106913	down
A_23_P51051	ZNF142	1.8402929	1.6994267	1.7672782	-0.140866	-0.073015	-0.106941	down
A_19_P00319721	FLJ37201	-1.13955	-1.191772	-1.301224	-0.052222	-0.161675	-0.106948	down
A_19_P00321765	LINC00630	-3.118928	-3.226736	-3.225197	-0.107808	-0.106268	-0.107038	down
A_23_P356646	TEKT4P2	-1.375231	-1.496037	-1.468549	-0.120806	-0.093318	-0.107062	down
A_22_P00014471	lnc-SHANK2-1	-1.42681	-1.462661	-1.605097	-0.035851	-0.178287	-0.107069	down
A_33_P3252359	BDH1	3.3220844	3.15274	3.2771873	-0.169344	-0.044897	-0.107121	down
A_33_P3244931	DBI	8.2468815	7.9580646	8.321385	-0.288817	0.0745039	-0.107157	down
A_22_P00011668	LINC01489	-1.140717	-1.158606	-1.337246	-0.017889	-0.196529	-0.107209	down
A_24_P168726	NF2	0.7437401	0.6952009	0.5778241	-0.048539	-0.165916	-0.107228	down
A_33_P3296193	MCTS2P	-2.506111	-2.631788	-2.594961	-0.125677	-0.08885	-0.107263	down
A_23_P9319	SPIN1	1.2494612	1.3143706	0.9700236	0.0649095	-0.279438	-0.107264	down
A_23_P17144	GPN1	4.89038	4.654404	4.9117603	-0.235976	0.0213804	-0.107298	down
A_33_P3373750	BRD4	3.9075508	3.895165	3.7053146	-0.012386	-0.202236	-0.107311	down
A_23_P502678	TM2D2	2.229433	2.2206893	2.0235138	-0.008744	-0.205919	-0.107332	down
A_33_P3334205	TMEM184A	-2.089606	-2.065934	-2.32809	0.0236721	-0.238483	-0.107406	down
A_23_P259451	LSM6	4.435727	4.0964255	4.5602007	-0.339302	0.1244736	-0.107414	down
A_21_P0007004	lnc-NDST2-3	-0.41796	-0.660079	-0.390677	-0.242119	0.0272822	-0.107418	down
A_23_P149545	HIST2H2BE	-2.640013	-2.278946	-3.215961	0.3610666	-0.575948	-0.107441	down
A_33_P3258041	GGPS1	-1.562502	-1.802105	-1.537799	-0.239603	0.0247025	-0.10745	down
A_23_P353574	NEK7	-1.354566	-1.27803	-1.646299	0.0765352	-0.291734	-0.107599	down
A_33_P3542599	SNORA2A	-1.391097	-1.3767	-1.620727	0.0143967	-0.22963	-0.107617	down
A_23_P216556	EPB41L4B	3.6036673	3.6495652	3.3424997	0.045898	-0.261168	-0.107635	down
A_23_P502350	RFX2	-1.077267	-1.447528	-0.922304	-0.370261	0.1549635	-0.107649	down
A_23_P64019	MTMR2	2.9985962	2.932795	2.8488464	-0.065801	-0.14975	-0.107775	down
A_33_P3227676	C2orf72	-0.892887	-1.400558	-0.600848	-0.507671	0.2920384	-0.107816	down
A_22_P00015662	SBF2-AS1	-1.074669	-0.953405	-1.411585	0.121264	-0.336916	-0.107826	down
A_33_P3287631	CTSB	4.9612284	4.466727	5.2399235	-0.494502	0.2786951	-0.107903	down
A_23_P212552	TBL1XR1	1.1676617	1.1165547	1.002842	-0.051107	-0.16482	-0.107963	down
A_33_P3273854	NAALADL2	-1.183144	-1.299346	-1.282878	-0.116202	-0.099735	-0.107969	down
A_23_P152055	EFTUD1	3.4237967	3.268351	3.363246	-0.155446	-0.060551	-0.107998	down
A_33_P3246543	MAT2B	-0.303087	-0.433648	-0.388575	-0.13056	-0.085488	-0.108024	down
A_23_P7535	HRH2	-2.685964	-3.046279	-2.541706	-0.360315	0.144258	-0.108028	down
A_23_P92410	CASP3	4.484563	4.3691306	4.383852	-0.115432	-0.100711	-0.108072	down
A_21_P0007374	lnc-PRKRIR-1	-1.318759	-1.260527	-1.593415	0.0582318	-0.274657	-0.108212	down
A_33_P3524912	LOC283038	-1.276936	-1.356243	-1.414115	-0.079307	-0.137179	-0.108243	down
A_32_P130788	SAMD13	-0.401704	-0.475557	-0.544427	-0.073853	-0.142723	-0.108288	down
A_22_P00004186	LOC100507140	-2.063661	-2.169233	-2.174702	-0.105572	-0.111041	-0.108307	down
A_33_P3235410	PTPLA	1.1258006	0.818213	1.216712	-0.307588	0.0909114	-0.108338	down
A_33_P3222203	OXER1	-0.522569	-0.740358	-0.521661	-0.217789	0.0009079	-0.108441	down
A_24_P6467	DENND2C	-1.958911	-1.814395	-2.320371	0.144516	-0.36146	-0.108472	down
A_21_P0006570	lnc-PLS3-2	-1.784835	-1.583514	-2.203155	0.2013211	-0.41832	-0.108499	down

A_23_P57807	MKRN2	2.0917158	1.8411732	2.1252089	-0.250543	0.033493	-0.108525	down
A_24_P343271	RMND1	0.5144301	0.4657688	0.3460355	-0.048661	-0.168395	-0.108528	down
A_33_P3376095	SYPL2	-2.014399	-2.381528	-1.864462	-0.367129	0.1499367	-0.108596	down
A_23_P77286	KATNBL1	-2.374774	-2.501239	-2.46566	-0.126466	-0.090886	-0.108676	down
A_33_P3381771	GID4	2.0578737	1.9552083	1.9431629	-0.102665	-0.114711	-0.108688	down
A_23_P89941	CDKN2D	4.2546434	4.1290383	4.1627064	-0.125605	-0.091937	-0.108771	down
A_33_P3211929	RCOR2	-1.874572	-2.549175	-1.417768	-0.674603	0.4568043	-0.108899	down
A_23_P218282	ZSCAN32	0.4544563	0.2088347	0.4822731	-0.245622	0.0278168	-0.108902	down
A_23_P72840	DPM3	-0.113741	-0.179955	-0.265524	-0.066214	-0.151783	-0.108998	down
A_33_P3424112	DHX40	-0.626075	-0.715538	-0.754806	-0.089463	-0.128731	-0.109097	down
A_23_P214267	GPR110	4.15104	3.8970933	4.186701	-0.253947	0.0356607	-0.109143	down
A_32_P231446	HIPK1	3.857624	3.6356397	3.861309	-0.221984	0.003685	-0.10915	down
A_23_P316472	DNHD1	-2.497956	-2.488712	-2.7256	0.0092447	-0.227643	-0.109199	down
A_24_P943393	AHNAK	7.041664	7.1184497	6.746456	0.0767856	-0.295208	-0.109211	down
A_33_P3289406	LOC91450	-0.469399	-0.873386	-0.283856	-0.403987	0.1855421	-0.109223	down
A_23_P104372	DNAJC9	5.273591	5.1190753	5.2095766	-0.154516	-0.064014	-0.109265	down
A_33_P3261246	RPL37	-0.493247	-0.472683	-0.73241	0.0205631	-0.239163	-0.1093	down
A_32_P76035	PNPT1	0.970027	0.6024351	1.1188631	-0.367592	0.1488361	-0.109378	down
A_24_P245322	PRKAR1A	0.7954669	0.6754365	0.6966529	-0.12003	-0.098814	-0.109422	down
A_23_P28169	ARL6IP6	1.9411926	1.6230388	2.0404654	-0.318154	0.0992727	-0.109441	down
A_23_P344481	STOX1	-1.647329	-1.763077	-1.750552	-0.115748	-0.103223	-0.109486	down
A_24_P380919	HNRNPK	5.6054745	5.471771	5.520088	-0.133704	-0.085386	-0.109545	down
A_21_P0007145	PPP2R5B	-1.49625	-1.519499	-1.692157	-0.023249	-0.195907	-0.109578	down
A_21_P0010306	MIR99AHG	-0.538424	-0.573519	-0.722582	-0.035095	-0.184158	-0.109627	down
A_21_P0010084	LOC100270804	-1.832165	-2.126685	-1.756971	-0.294519	0.0751944	-0.109663	down
A_23_P2355	CBX5	2.0777397	2.018228	1.917737	-0.059512	-0.160003	-0.109757	down
A_24_P65616	PVR	2.5910034	2.4413056	2.5210009	-0.149698	-0.070003	-0.10985	down
A_32_P228414	GAPVD1	0.5188427	0.3310289	0.4868441	-0.187814	-0.031999	-0.109906	down
A_23_P1102	ACTA1	-2.517099	-2.46918	-2.785051	0.0479195	-0.267951	-0.110016	down
A_23_P253723	RRH	-0.199312	-0.29083	-0.327922	-0.091517	-0.12861	-0.110064	down
A_21_P0013269	LOC101928451	1.0494857	0.9881411	0.8905163	-0.061345	-0.158969	-0.110157	down
A_33_P3371055	RAB1A	6.247547	6.0953355	6.1793737	-0.152212	-0.068173	-0.110193	down
A_23_P79259	SH3BP4	0.6634593	0.640255	0.4662743	-0.023204	-0.197185	-0.110195	down
A_23_P45726	HNRNPR	4.2379417	4.069822	4.18557	-0.16812	-0.052372	-0.110246	down
A_24_P11965	MRFAP1	3.407957	3.2932448	3.3020744	-0.114712	-0.105883	-0.110297	down
A_33_P3357332	lnc-C9orf147-1	-1.723321	-2.01754	-1.649716	-0.29422	0.0736041	-0.110308	down
A_24_P151	KCNAB2	-2.198286	-2.143525	-2.473669	0.0547609	-0.275383	-0.110311	down
A_32_P202859	H1FNT	-1.085486	-1.148707	-1.243053	-0.063221	-0.157567	-0.110394	down
A_33_P3372281	MRS2	0.907311	0.6650457	0.9287601	-0.242265	0.0214491	-0.110408	down
A_33_P3367917	SSH2	1.7724762	1.3695631	1.9544992	-0.402913	0.1820231	-0.110445	down
A_33_P3365601	ZNF316	-2.209944	-2.597311	-2.043513	-0.387368	0.1664305	-0.110469	down
A_19_P00316753	PVT1	1.0401411	0.8873162	0.9719229	-0.152825	-0.068218	-0.110522	down
A_23_P24751	TTC9C	2.5252352	1.9920974	2.8372574	-0.533138	0.3120222	-0.110558	down
A_24_P330796	MKS1	-1.713158	-1.979732	-1.667814	-0.266573	0.0453444	-0.110615	down
A_21_P0006351	lnc-RP11-262H14.4.1-4	-1.809745	-1.953217	-1.887519	-0.143472	-0.077774	-0.110623	down
A_33_P3332126	SCLY	1.1161699	0.6869359	1.3240557	-0.429234	0.2078857	-0.110674	down
A_33_P3280094	LRFN4	5.5516567	5.125261	5.756651	-0.426396	0.2049942	-0.110701	down
A_23_P151544	DDX24	0.6019268	0.3426466	0.6392036	-0.25928	0.0372767	-0.111002	down
A_23_P311232	CLASP1	0.8399377	0.7594743	0.6983943	-0.080463	-0.141543	-0.111003	down
A_23_P388670	LTA4H	6.718356	6.524963	6.689474	-0.193393	-0.028882	-0.111138	down
A_23_P133995	PPIL1	4.683153	4.5342784	4.609728	-0.148875	-0.073425	-0.11115	down
A_33_P3228102	NPHP3	0.1158929	0.0578828	-0.048718	-0.05801	-0.164611	-0.111311	down
A_33_P3346032	lnc-GOLGA8J-2	-1.692943	-1.7784	-1.830116	-0.085456	-0.137173	-0.111315	down
A_33_P3327592	RNF13	4.167328	4.003198	4.1087494	-0.16413	-0.058578	-0.111354	down
A_23_P80832	TMEM41A	1.50105	1.2390189	1.5402508	-0.262031	0.0392008	-0.111415	down

A_33_P3302354	CABIN1	-1.701194	-1.907363	-1.718039	-0.206169	-0.016845	-0.111507	down
A_33_P3411145	FAM200B	-1.55533	-1.774149	-1.559586	-0.218819	-0.004256	-0.111537	down
A_23_P168882	TP53INP1	0.2373848	0.3706584	-0.119127	0.1332736	-0.356512	-0.111619	down
A_33_P3416574	AIG1	2.6696587	2.4932857	2.622716	-0.176373	-0.046943	-0.111658	down
A_24_P381494	SLC11A2	0.6540222	0.6304078	0.4542093	-0.023614	-0.199813	-0.111714	down
A_33_P3394494	ELP5	1.5411348	1.487092	1.3716831	-0.054043	-0.169452	-0.111747	down
A_21_P0000882	RNF157-AS1	0.8764944	0.4780216	1.0514522	-0.398473	0.1749578	-0.111758	down
A_21_P0000303	SNORA75	0.2599688	-0.024413	0.3208213	-0.284382	0.0608525	-0.111765	down
A_24_P102920	WDR33	-2.19762	-2.368164	-2.251034	-0.170543	-0.053413	-0.111978	down
A_21_P0012051	SEPT14	-0.011732	0.1219215	-0.369372	0.1336532	-0.35764	-0.111994	down
A_23_P49646	PRPSAP2	2.2452917	2.1159024	2.1506596	-0.129389	-0.094632	-0.112011	down
A_24_P164894	DSCR3	-1.364189	-1.606269	-1.346176	-0.242081	0.0180125	-0.112034	down
A_33_P3648597	LOC283075	-2.173051	-2.370325	-2.200014	-0.197274	-0.026963	-0.112118	down
A_24_P399083	TXNL4B	0.0429087	0.0412493	-0.179865	-0.001659	-0.222774	-0.112217	down
A_23_P162127	CCDC90B	3.7706223	3.6902528	3.6264124	-0.080369	-0.14421	-0.11229	down
A_23_P99360	TRIM13	0.7413182	0.361897	0.8961496	-0.379421	0.1548314	-0.112295	down
A_21_P0013285	LOC101927668	-3.072343	-2.908004	-3.461351	0.1643398	-0.389007	-0.112334	down
A_21_P0012473	VEZF1	-0.403697	-0.508618	-0.523489	-0.104921	-0.119792	-0.112356	down
A_22_P00019021	lnc-SETDB1-1	-2.217573	-2.638254	-2.021813	-0.420681	0.1957603	-0.11246	down
A_22_P00007002	lnc-GGCT-1	-2.086195	-2.036038	-2.361271	0.0501561	-0.275076	-0.11246	down
A_23_P104892	EIF4G2	5.7654247	5.6521153	5.6537724	-0.113309	-0.111652	-0.112481	down
A_23_P56922	HSPE1	8.824333	8.6607685	8.762928	-0.163565	-0.061405	-0.112485	down
A_23_P431319	YIPF6	4.343605	4.251776	4.2103395	-0.091829	-0.133266	-0.112547	down
A_21_P0007038	lnc-BNIP3-2	-0.346906	-0.43188	-0.487032	-0.084974	-0.140127	-0.11255	down
A_24_P155791	ASB7	0.3996058	0.4020186	0.1720901	0.0024128	-0.227516	-0.112551	down
A_22_P00012995	lnc-RFPL3-3	-2.534269	-2.497887	-2.795816	0.0363824	-0.261547	-0.112582	down
A_23_P147845	PDXDC1	4.7632084	4.6979203	4.6032324	-0.065288	-0.159976	-0.112632	down
A_23_P358597	POPDC3	1.2337141	1.0927863	1.1491475	-0.140928	-0.084567	-0.112747	down
A_24_P162226	RIMBP2	-2.981362	-3.202843	-2.985435	-0.221481	-0.004073	-0.112777	down
A_23_P417113	RBM33	0.1349258	0.4135938	-0.369586	0.2786679	-0.504512	-0.112922	down
A_23_P145068	MTO1	2.8166351	2.6818538	2.7254133	-0.134781	-0.091222	-0.113002	down
A_33_P3268695	ZNF503	-0.063058	-0.167196	-0.185094	-0.104138	-0.122036	-0.113087	down
A_24_P167683	ITPK1	-1.269324	-1.426006	-1.338871	-0.156682	-0.069547	-0.113115	down
A_24_P915692	PHLDA1	3.1569738	2.8500314	3.2373285	-0.306942	0.0803547	-0.113294	down
A_23_P30243	ERAP2	2.6518116	2.3382316	2.7387257	-0.31358	0.0869141	-0.113333	down
A_21_P0005950	lnc-MYC-2	-1.537345	-1.731323	-1.570072	-0.193978	-0.032727	-0.113353	down
A_23_P141005	AMFR	0.2425733	-0.074707	0.3331189	-0.31728	0.0905457	-0.113367	down
A_23_P65307	SLITRK6	0.0964327	-0.4033	0.3694043	-0.499733	0.2729716	-0.11338	down
A_23_P255785	NCK1	2.1377068	2.137465	1.9107275	-0.000242	-0.226979	-0.113611	down
A_23_P126057	SCP2	2.6233644	2.4346557	2.5848017	-0.188709	-0.038563	-0.113636	down
A_24_P329597	UBQLN1	1.438374	1.1500912	1.4992528	-0.288283	0.0608788	-0.113702	down
A_23_P94533	CTSL	3.6700592	3.4241233	3.6885662	-0.245936	0.018507	-0.113714	down
A_23_P213153	HNRNPDL	4.048908	4.006495	3.8638525	-0.042413	-0.185056	-0.113734	down
A_33_P3302696	PPM1D	0.6641612	0.4960322	0.6046782	-0.168129	-0.059483	-0.113806	down
A_23_P47426	ACAD8	-1.496416	-1.351733	-1.868847	0.1446834	-0.372431	-0.113874	down
A_24_P410627	MKRN1	2.6871824	2.3656802	2.7809362	-0.321502	0.0937538	-0.113874	down
A_23_P205768	ARPP19	4.0784626	4.054087	3.8750868	-0.024375	-0.203376	-0.113876	down
A_23_P200637	FBXO44	-0.279422	-0.844255	0.0576029	-0.564833	0.3370247	-0.113904	down
A_21_P0006071	lnc-NOL6-2	-0.355468	-0.428132	-0.5107	-0.072664	-0.155232	-0.113948	down
A_23_P89199	COPS3	4.039857	3.7678828	4.0837336	-0.271974	0.0438766	-0.114049	down
A_23_P389907	ATXN2	3.7202024	3.711821	3.5004082	-0.008381	-0.219794	-0.114088	down
A_23_P13065	ZDHHC13	0.2975621	0.1246891	0.2421808	-0.172873	-0.055381	-0.114127	down
A_21_P0013416	lnc-MACC1-1	-2.223389	-2.450874	-2.224238	-0.227485	-0.000849	-0.114167	down
A_33_P3380529	PRTFDC1	-0.369604	-0.313683	-0.653868	0.0559216	-0.284264	-0.114171	down
A_33_P3296198	C5orf63	8.73483	8.451204	8.790097	-0.283626	0.0552673	-0.114179	down
A_23_P429535	GTF3C3	2.5005255	2.1556	2.6168985	-0.344925	0.1163731	-0.114276	down

A_32_P143048	ZFYVE9	0.3022203	0.4157572	-0.039934	0.1135368	-0.342154	-0.114309	down
A_24_P314571	SPC24	2.6487188	2.337295	2.7314463	-0.311424	0.0827274	-0.114348	down
A_19_P00315941	LINC00969	-0.88549	-0.835325	-1.164415	0.0501652	-0.278925	-0.11438	down
A_23_P91487	RWDD2B	0.3382602	0.3015456	0.1461029	-0.036715	-0.192157	-0.114436	down
A_23_P128598	TUBA3C	1.2179742	0.895752	1.3112311	-0.322222	0.093257	-0.114483	down
A_24_P195037	C1orf52	2.2394972	2.1760755	2.0736628	-0.063422	-0.165834	-0.114628	down
A_23_P82823	PINX1	2.1780024	1.9918237	2.1348963	-0.186179	-0.043106	-0.114642	down
A_24_P91094	RUNDC3A	-2.018104	-2.055526	-2.210258	-0.037422	-0.192154	-0.114788	down
A_23_P381261	ADCY4	-2.295709	-2.340207	-2.480869	-0.044498	-0.18516	-0.114829	down
A_19_P00318889	KIAA1671	-2.46113	-2.790886	-2.361111	-0.329756	0.1000187	-0.114869	down
A_24_P113824	TMEM50A	1.8657837	1.6162295	1.8854847	-0.249554	0.019701	-0.114927	down
A_23_P38457	TAOK1	2.8243904	2.7710032	2.6478891	-0.053387	-0.176501	-0.114944	down
A_23_P54758	GDE1	3.966877	3.8252501	3.8785238	-0.141627	-0.088353	-0.11499	down
A_24_P379104	PIM2	1.573802	1.4719844	1.4455972	-0.101818	-0.128205	-0.115011	down
A_33_P3301689	ZNF326	1.5618048	1.6039944	1.2895584	0.0421896	-0.272246	-0.115028	down
A_22_P00025441	lnc-ANKRD42-2	-2.002086	-2.150581	-2.083783	-0.148495	-0.081697	-0.115096	down
A_23_P111753	MRPL32	6.062315	5.9197526	5.9746227	-0.142562	-0.087692	-0.115127	down
A_33_P3331601	THNSL1	0.2149692	0.0993075	0.1003571	-0.115662	-0.114612	-0.115137	down
A_19_P00811196	APTR	3.365284	3.1443677	3.3558044	-0.220916	-0.00948	-0.115198	down
A_33_P3336696	ND4L	8.308639	8.365379	8.021436	0.0567408	-0.287203	-0.115231	down
A_23_P355067	TMCO1	3.4766407	3.1851687	3.5375175	-0.291472	0.0608768	-0.115298	down
A_23_P157007	TMEM176B	-2.737014	-2.905265	-2.799747	-0.168252	-0.062733	-0.115493	down
A_33_P3299232	BMS1P20	-2.657489	-2.605509	-2.940474	0.0519798	-0.282985	-0.115502	down
A_23_P204536	SENP1	2.3832903	2.2260814	2.309452	-0.157209	-0.073838	-0.115524	down
A_33_P3317277	DPF1	-0.684986	-0.973432	-0.627797	-0.288446	0.0571885	-0.115629	down
A_23_P11214	NKRF	3.0959482	2.9134393	3.0471973	-0.182509	-0.048751	-0.11563	down
A_22_P00012829	TMEM161B-AS1	0.3618403	0.1221132	0.3701482	-0.239727	0.0083079	-0.11571	down
A_21_P0014483	FGD5-AS1	4.995945	4.7324786	5.027643	-0.263466	0.0316982	-0.115884	down
A_21_P0012140	LOC101926935	-2.088492	-2.167463	-2.241353	-0.078971	-0.152861	-0.115916	down
A_33_P3376379	CNST	-0.556655	-0.630455	-0.714729	-0.0738	-0.158074	-0.115937	down
A_33_P3284463	ASAH1	4.7482843	4.6319327	4.632663	-0.116352	-0.115622	-0.115987	down
A_23_P211627	NUP50	1.8528223	1.8152857	1.6581097	-0.037537	-0.194713	-0.116125	down
A_33_P3238215	COBLL1	2.9968977	3.181604	2.5799313	0.1847062	-0.416966	-0.11613	down
A_33_P3399061	ADARB1	-2.191477	-2.066865	-2.548354	0.1246119	-0.356878	-0.116133	down
A_33_P3313125	lnc-FAM189A2-1	1.7800226	1.5464807	1.7812223	-0.233542	0.0011997	-0.116171	down
A_21_P0010536	GBP3	0.8502793	0.5955463	0.8726382	-0.254733	0.0223589	-0.116187	down
A_33_P3306715	C6orf132	-2.401578	-2.442243	-2.593367	-0.040664	-0.191788	-0.116226	down
A_23_P213857	C7	-1.762402	-1.675493	-2.081941	0.0869093	-0.319539	-0.116315	down
A_33_P3280066	PTRF	8.770299	8.71125	8.596706	-0.059049	-0.173593	-0.116321	down
A_23_P136635	AP3B1	3.4490604	3.2658896	3.3995113	-0.183171	-0.049549	-0.11636	down
A_33_P3325229	LOC389906	0.3170133	0.1281958	0.2730165	-0.188818	-0.043997	-0.116407	down
A_23_P319572	NR1I3	-2.884219	-2.960805	-3.040519	-0.076586	-0.1563	-0.116443	down
A_22_P00017323	KCTD21-AS1	-2.949053	-2.773057	-3.358004	0.1759965	-0.40895	-0.116477	down
A_24_P839254	RPP14	2.2092667	2.0431104	2.1422472	-0.166156	-0.067019	-0.116588	down
A_23_P408768	DOT1L	0.2153544	0.2531586	-0.055676	0.0378041	-0.27103	-0.116613	down
A_33_P3323742	PNRC2	-0.063811	-0.073077	-0.287853	-0.009266	-0.224041	-0.116654	down
A_23_P362183	ANKS6	4.852766	4.7517667	4.7203608	-0.100999	-0.132405	-0.116702	down
A_33_P3289251	LOC644727	-0.142653	-0.370487	-0.148365	-0.227834	-0.005712	-0.116773	down
A_23_P54605	RSL1D1	4.5572767	4.2337937	4.6470785	-0.323483	0.0898018	-0.116841	down
A_23_P134113	SLC18B1	3.8919725	3.8459468	3.7040882	-0.046026	-0.187884	-0.116955	down
A_22_P00020997	lnc-FAM83H-1	-2.296052	-2.654979	-2.171146	-0.358927	0.1249061	-0.11701	down
A_21_P0010676	XLOC_l2_001331	-2.014433	-2.157006	-2.105909	-0.142573	-0.091475	-0.117024	down
A_23_P56810	SLC4A1AP	3.3419275	3.1955533	3.2542162	-0.146374	-0.087711	-0.117043	down
A_33_P3223749	MED14	2.589179	2.4196239	2.5245066	-0.169555	-0.064672	-0.117114	down
A_21_P0012052	XLOC_l2_007835	0.5690432	0.6029935	0.3008328	0.0339503	-0.26821	-0.11713	down
A_21_P0003940	lnc-CCDC125-1	-1.09271	-1.251911	-1.167774	-0.159201	-0.075064	-0.117132	down

A_23_P115842	CCAR1	3.6275692	3.5410295	3.4797792	-0.08654	-0.14779	-0.117165	down
A_24_P142983	PI4KA	3.1051855	3.0614333	2.914526	-0.043752	-0.19066	-0.117206	down
A_23_P57667	PLXNA1	5.686281	5.4415145	5.69652	-0.244767	0.0102386	-0.117264	down
A_33_P3223713	UBE2E3	2.4673529	2.3701873	2.3299007	-0.097166	-0.137452	-0.117309	down
A_33_P3888485	BCO2	-1.902685	-2.175739	-1.864354	-0.273054	0.0383306	-0.117362	down
A_23_P393645	ADAMTS13	0.7503119	0.6924195	0.5731978	-0.057892	-0.177114	-0.117503	down
A_32_P120638	INTS4	-0.346534	-0.388758	-0.5394	-0.042223	-0.192865	-0.117544	down
A_33_P3233764	LATS1	-1.876877	-2.007997	-1.980892	-0.13112	-0.104015	-0.117568	down
A_24_P816384	UBE2Q2P1	0.7911773	0.8140655	0.5330482	0.0228882	-0.258129	-0.11762	down
A_23_P408095	DSTN	7.729129	7.5450816	7.677861	-0.184047	-0.051268	-0.117657	down
A_32_P182388	ZNF77	0.7371273	0.730228	0.5086932	-0.006899	-0.228434	-0.117667	down
A_23_P91491	C21orf59	5.4186764	5.243912	5.358095	-0.174764	-0.060581	-0.117673	down
A_33_P3258392	EDN1	-1.59763	-1.839576	-1.59116	-0.241947	0.0064697	-0.117738	down
A_24_P389517	HNRNPK	4.461916	4.2680135	4.4202967	-0.193902	-0.041619	-0.117761	down
A_33_P3821660	RABL6	0.8971901	0.8497868	0.7090116	-0.047403	-0.188179	-0.117791	down
A_23_P93881	SYPL1	3.5933952	3.5463996	3.4047155	-0.046996	-0.18868	-0.117838	down
A_33_P3259615	TTC36	-2.579143	-2.840081	-2.553929	-0.260938	0.0252135	-0.117862	down
A_24_P21056	PHF14	2.7638922	2.5527701	2.7390833	-0.211122	-0.024809	-0.117965	down
A_23_P211401	KREMEN1	1.2464604	0.8918161	1.364944	-0.354644	0.1184835	-0.11808	down
A_21_P0007892	lnc-TMEM116-1	-1.514788	-1.639199	-1.626577	-0.124411	-0.111789	-0.1181	down
A_32_P92505	LCLAT1	4.521058	4.2711596	4.5347214	-0.249898	0.0136633	-0.118118	down
A_24_P158314	GARNL3	-0.974021	-1.136506	-1.047862	-0.162485	-0.073841	-0.118163	down
A_33_P6474388	LOC101928053	1.0698414	0.8414078	1.0619402	-0.228434	-0.007901	-0.118167	down
A_24_P223604	KIAA1429	5.331624	5.330931	5.095889	-0.000693	-0.235735	-0.118214	down
A_24_P320526	C6orf106	0.6413899	0.3768292	0.6694427	-0.264561	0.0280528	-0.118254	down
A_23_P414273	SMIM3	6.011985	5.7565713	6.030879	-0.255414	0.0188942	-0.11826	down
A_21_P0012766	EVA1C	1.0073023	1.1244473	0.6535273	0.1171451	-0.353775	-0.118315	down
A_22_P00012339	lnc-PRDM13-4	-2.291224	-2.026904	-2.792187	0.2643204	-0.500963	-0.118321	down
A_33_P3338956	ARL8B	0.5487676	0.5932331	0.2676425	0.0444655	-0.281125	-0.11833	down
A_24_P462899	CENPW	4.5385723	4.227672	4.6127644	-0.3109	0.0741921	-0.118354	down
A_23_P214244	ENPP5	-1.356292	-1.270792	-1.678708	0.0855002	-0.322416	-0.118458	down
A_33_P3646133	NFATC2IP	1.0314775	0.8536916	0.9723001	-0.177786	-0.059177	-0.118482	down
A_21_P0012929	XLOC_l2_012319	-0.557923	-0.972247	-0.380577	-0.414324	0.1773462	-0.118489	down
A_24_P342096	FAM27C	3.8419046	3.6424365	3.804389	-0.199468	-0.037516	-0.118492	down
A_24_P261734	SLC38A1	4.954611	4.962294	4.709753	0.0076833	-0.244858	-0.118587	down
A_33_P3239185	SYT7	2.488596	2.3081927	2.431738	-0.180403	-0.056858	-0.118631	down
A_21_P0000189	DR1	3.4971066	3.3963885	3.3605242	-0.100718	-0.136582	-0.11865	down
A_24_P419120	BICD2	3.1931562	2.9709554	3.1780472	-0.222201	-0.015109	-0.118655	down
A_33_P3279545	HIPK3	-0.654433	-0.849783	-0.696464	-0.19535	-0.042031	-0.11869	down
A_33_P3252612	CYP2W1	10.024449	9.989605	9.82187	-0.034844	-0.20258	-0.118712	down
A_22_P00007283	lnc-GPR20-1	-1.764199	-2.301803	-1.464034	-0.537605	0.3001652	-0.11872	down
A_24_P414999	LAPTM4B	4.964923	4.7822595	4.9101334	-0.182663	-0.05479	-0.118726	down
A_24_P941988	SPAST	1.497675	1.563674	1.1940165	0.065999	-0.303659	-0.11883	down
A_33_P3429242	LOC339988	-0.324359	-0.565371	-0.32113	-0.241012	0.0032291	-0.118891	down
A_22_P00009793	lnc-MEGF11-2	-0.204891	-0.352234	-0.295331	-0.147344	-0.09044	-0.118892	down
A_23_P40174	MMP9	-1.524716	-1.666207	-1.621048	-0.141491	-0.096332	-0.118912	down
A_33_P3245484	LOC100131000	-1.908346	-1.897669	-2.156915	0.0106764	-0.248569	-0.118947	down
A_24_P102053	OCLN	0.6501894	0.4892392	0.5732055	-0.16095	-0.076984	-0.118967	down
A_32_P167076	CAPN14	-2.48774	-2.971121	-2.242362	-0.483381	0.245378	-0.119001	down
A_23_P66777	CDC27	3.0614786	2.9838424	2.9010105	-0.077636	-0.160468	-0.119052	down
A_22_P00021466	lnc-CENPP-1	-0.814952	-1.080189	-0.787918	-0.265237	0.0270338	-0.119102	down
A_21_P0000335	SNORA51	0.6704459	0.295269	0.8073087	-0.375177	0.1368628	-0.119157	down
A_24_P661641	LINC00938	1.4949937	1.3930898	1.3585186	-0.101904	-0.136475	-0.11919	down
A_23_P81880	CTDSP2	6.302945	6.3059335	6.0615416	0.0029883	-0.241404	-0.119208	down
A_33_P3285987	METTL15	0.9878545	0.8422232	0.8948398	-0.145631	-0.093015	-0.119323	down
A_23_P65262	N4BP2L2	0.2401133	0.4483295	-0.206781	0.2082162	-0.446894	-0.119339	down

A_23_P143446	MRPL39	3.2879229	3.1681943	3.1685772	-0.119729	-0.119346	-0.119537	down
A_33_P3268567	NCK2	4.3497057	4.1806207	4.279706	-0.169085	-0.07	-0.119542	down
A_23_P161338	PPA1	7.0859213	6.8907027	7.04185	-0.195219	-0.044071	-0.119645	down
A_23_P118493	TOM1L1	2.3829756	2.4429326	2.0836306	0.059957	-0.299345	-0.119694	down
A_33_P3296592	C11orf30	0.7875485	0.8093066	0.5259271	0.0217581	-0.261621	-0.119932	down
A_23_P368145	GORAB	-1.124064	-1.201074	-1.286982	-0.077011	-0.162919	-0.119965	down
A_24_P726336	PHACTR2	1.5759153	1.3415074	1.5703821	-0.234408	-0.005533	-0.119971	down
A_24_P917026	NF1	1.2430539	1.2425532	1.0033698	-0.000501	-0.239684	-0.120092	down
A_23_P106798	SIAH1	-0.093175	-0.170297	-0.256309	-0.077122	-0.163134	-0.120128	down
A_23_P31844	ATP6V1B2	2.2121553	1.9511504	2.232871	-0.261005	0.0207157	-0.120145	down
A_23_P94128	NEIL2	-0.720004	-0.827346	-0.853017	-0.107342	-0.133014	-0.120178	down
A_21_P0012053	FLJ43681	-1.017088	-0.965967	-1.308603	0.0511203	-0.291516	-0.120198	down
A_24_P269895	HNRNPA3	3.884244	3.6620126	3.8659706	-0.222231	-0.018273	-0.120252	down
A_21_P0000193	SF3A3	4.230319	3.5500474	4.6700697	-0.680272	0.4397507	-0.12026	down
A_22_P00000614	CKMT2-AS1	-1.219935	-1.388702	-1.291825	-0.168767	-0.07189	-0.120329	down
A_22_P00016713	lnc-TRAP1-1	-2.774878	-3.154475	-2.635981	-0.379598	0.1388962	-0.120351	down
A_24_P178093	TOMM40	3.100255	2.8838	3.0758228	-0.216455	-0.024432	-0.120444	down
A_23_P68970	ARFGAP3	2.4539204	2.4637256	2.2030706	0.0098052	-0.25085	-0.120522	down
A_33_P3415087	CLCN5	0.4953303	0.4297171	0.3196969	-0.065613	-0.175633	-0.120623	down
A_23_P16743	MGAT5	1.119216	0.954463	1.0425129	-0.164753	-0.076703	-0.120728	down
A_22_P00003190	LOC100996348	-2.543296	-2.665645	-2.662481	-0.122349	-0.119185	-0.120767	down
A_23_P218442	CEACAM6	4.9029856	4.7003875	4.8639145	-0.202598	-0.039071	-0.120835	down
A_24_P307572	ANKRD13A	-0.427065	-0.67129	-0.42452	-0.244225	0.0025454	-0.12084	down
A_23_P46095	C1orf131	1.5513191	1.3002014	1.5606422	-0.251118	0.0093231	-0.120897	down
A_22_P00012929	PRC1-AS1	-0.883494	-1.064531	-0.944331	-0.181036	-0.060837	-0.120937	down
A_23_P435407	GPC4	-1.855115	-2.163775	-1.788359	-0.30866	0.0667558	-0.120952	down
A_33_P3774867	lnc-AP002478.1-1	-0.903917	-0.887391	-1.162363	0.0165262	-0.258445	-0.12096	down
A_23_P259874	SEC22A	2.3434744	2.1716886	2.273221	-0.171786	-0.070253	-0.12102	down
A_23_P102770	NAA20	4.359973	4.1191945	4.3585253	-0.240778	-0.001448	-0.121113	down
A_21_P0000891	SDCBP2-AS1	-0.908087	-0.928048	-1.130464	-0.019961	-0.222376	-0.121169	down
A_22_P00007020	ZNF337-AS1	-1.627295	-1.80817	-1.688811	-0.180875	-0.061516	-0.121196	down
A_33_P3355232	UBLCP1	3.3332672	3.1787982	3.2451057	-0.154469	-0.088161	-0.121315	down
A_33_P3370132	TNPO3	2.028407	1.8733039	1.9408207	-0.155103	-0.087586	-0.121345	down
A_24_P250815	POF1B	1.6582899	1.4898252	1.5839949	-0.168465	-0.074295	-0.12138	down
A_23_P117734	LYSMD4	1.8882208	1.7244701	1.8089318	-0.163751	-0.079289	-0.12152	down
A_23_P13554	ALG8	5.9044313	5.552996	6.012678	-0.351435	0.1082468	-0.121594	down
A_23_P17021	SCRN3	1.9461203	1.8497896	1.7992496	-0.096331	-0.146871	-0.121601	down
A_33_P3379941	HMGXB3	4.0271273	3.7523503	4.0586634	-0.274777	0.0315361	-0.12162	down
A_22_P00016602	KIAA0040	-1.642228	-2.206879	-1.320867	-0.564651	0.3213611	-0.121645	down
A_23_P66715	PIGS	0.0703607	-0.150102	0.0471993	-0.220463	-0.023161	-0.121812	down
A_23_P70448	HIST1H1A	5.4971733	5.1400847	5.610626	-0.357089	0.1134529	-0.121818	down
A_33_P3320443	MGC57346-CRHR1	-0.26458	-0.505492	-0.267379	-0.240912	-0.002799	-0.121855	down
A_23_P79426	CAB39	3.1211443	3.0423884	2.9561615	-0.078756	-0.164983	-0.121869	down
A_24_P339858	TSPEAR-AS2	0.7557082	0.6904297	0.5771074	-0.065279	-0.178601	-0.12194	down
A_22_P00017841	MATN1-AS1	-2.822713	-2.722635	-3.166793	0.1000783	-0.34408	-0.122001	down
A_32_P99100	PTPRK	5.1124344	5.01802	4.962845	-0.094414	-0.14959	-0.122002	down
A_24_P390060	IQCD	0.4535971	0.2420569	0.421114	-0.21154	-0.032483	-0.122012	down
A_24_P241330	ZC3H14	1.3882437	1.1759429	1.3564024	-0.212301	-0.031841	-0.122071	down
A_24_P331704	KRT80	5.134783	5.2095313	4.8157578	0.0747485	-0.319025	-0.122138	down
A_22_P00002361	lnc-C12orf43-1	-1.954383	-2.218976	-1.934216	-0.264593	0.0201669	-0.122213	down
A_33_P3312519	KPRP	-2.723256	-2.068772	-3.622231	0.6544838	-0.898975	-0.122246	down
A_32_P138004	FAM45A	2.12599	2.0156226	1.9918003	-0.110367	-0.13419	-0.122278	down
A_23_P139099	OR4A15	-2.355858	-2.702909	-2.253389	-0.347051	0.1024685	-0.122291	down
A_23_P48936	SMAD3	2.524211	2.452231	2.3511744	-0.07198	-0.173037	-0.122508	down
A_24_P290163	SMG1P1	0.6623211	0.3037739	0.775723	-0.358547	0.1134019	-0.122573	down
A_33_P3313796	CCDC34	2.4191742	2.0512195	2.5418053	-0.367955	0.1226311	-0.122662	down

A_23_P52311	TAF5	-0.118057	-0.217256	-0.264286	-0.099199	-0.146229	-0.122714	down
A_33_P3240912	MAGT1	0.8885078	0.7909579	0.7405601	-0.09755	-0.147948	-0.122749	down
A_23_P128613	KDELC1	0.9451628	0.575181	1.069468	-0.369982	0.1243053	-0.122838	down
A_23_P37598	NPTN	2.6577444	2.539164	2.5306406	-0.11858	-0.127104	-0.122842	down
A_23_P107051	TCAP	4.2166634	4.2083697	3.97923	-0.008294	-0.237433	-0.122864	down
A_33_P3295283	LPCAT3	4.9038944	4.4784513	5.083501	-0.425443	0.1796064	-0.122918	down
A_24_P254278	SLC23A2	0.2304249	0.4631949	-0.248292	0.23277	-0.478717	-0.122974	down
A_33_P3381191	FOXRED2	-1.365668	-1.369807	-1.607514	-0.004139	-0.241846	-0.122993	down
A_24_P346604	UBE2W	0.4970684	0.4144521	0.333601	-0.082616	-0.163467	-0.123042	down
A_23_P501770	ATRIP	-1.604853	-1.679105	-1.776708	-0.074252	-0.171855	-0.123054	down
A_23_P68007	ATP1B3	8.066806	7.775929	8.111293	-0.290877	0.044487	-0.123195	down
A_33_P3316394	ZNF419	-0.937894	-0.94205	-1.180153	-0.004156	-0.242259	-0.123207	down
A_23_P7636	PTTG1	7.2046156	6.9736676	7.1891317	-0.230948	-0.015484	-0.123216	down
A_23_P419795	SBF2	-1.517827	-1.37323	-1.908872	0.1445971	-0.391046	-0.123224	down
A_23_P35230	CD46	0.9557982	0.6363778	1.0287309	-0.31942	0.0729327	-0.123244	down
A_23_P6963	UBE2E1	-0.601721	-1.115485	-0.33447	-0.513764	0.2672515	-0.123256	down
A_24_P413470	TP73	-2.716914	-3.083189	-2.597167	-0.366276	0.1197469	-0.123264	down
A_23_P202143	NOLC1	5.568692	5.345264	5.5455513	-0.223428	-0.023141	-0.123285	down
A_33_P3399107	PI4KA	1.7626572	1.6190338	1.6596699	-0.143623	-0.102987	-0.123305	down
A_21_P0000476	SNORA70C	1.7653418	1.7645106	1.519557	-0.000831	-0.245785	-0.123308	down
A_23_P129358	SETD6	2.0758963	1.9434991	1.961504	-0.132397	-0.114392	-0.123395	down
A_23_P160214	TTC39A	1.1482477	0.9393997	1.110292	-0.208848	-0.037956	-0.123402	down
A_21_P0012389	XLOC_l2_009867	-1.372473	-1.664876	-1.326977	-0.292403	0.045496	-0.123454	down
A_32_P46840	LOC729680	1.5841303	1.2077813	1.7135639	-0.376349	0.1294336	-0.123458	down
A_23_P128396	RNF34	3.3112392	3.3855443	2.9899454	0.0743051	-0.321294	-0.123494	down
A_33_P3224735	ZC3H12B	-0.358392	-0.614939	-0.348854	-0.256547	0.0095387	-0.123504	down
A_23_P119418	URI1	2.858348	2.7434678	2.7262106	-0.11488	-0.132137	-0.123509	down
A_33_P3378430	NOM1	1.9970179	1.9077444	1.8392429	-0.089273	-0.157775	-0.123524	down
A_33_P3210521	C2orf43	0.8470345	0.6963096	0.7505117	-0.150725	-0.096523	-0.123624	down
A_24_P222911	SRSF7	1.3947563	1.4143925	1.1278691	0.0196362	-0.266887	-0.123626	down
A_24_P218970	EIF4B	1.7724142	1.676733	1.6208329	-0.095681	-0.151581	-0.123631	down
A_33_P3229552	LRFN1	0.3125892	-0.163897	0.5418115	-0.476486	0.2292223	-0.123632	down
A_33_P3391105	LRP1	-2.413166	-2.621653	-2.451958	-0.208487	-0.038792	-0.12364	down
A_23_P58031	MAP3K13	-1.778442	-1.947745	-1.856434	-0.169303	-0.077992	-0.123648	down
A_22_P00007978	IDS	2.1733751	2.2016273	1.897543	0.0282521	-0.275832	-0.12379	down
A_24_P414712	BRPF3	-1.85296	-1.968397	-1.985201	-0.115437	-0.132241	-0.123839	down
A_23_P86632	DCLRE1C	1.819973	1.5388589	1.8533363	-0.281114	0.0333633	-0.123875	down
A_33_P3340342	CMTM3	-0.959738	-0.973645	-1.193615	-0.013906	-0.233876	-0.123891	down
A_33_P3278941	REC8	1.0686617	0.8837266	1.0058041	-0.184935	-0.062858	-0.123896	down
A_33_P3352432	KRBOX4	0.8192482	0.8582568	0.532301	0.0390086	-0.286947	-0.123969	down
A_33_P3475737	ZBTB8A	2.026085	2.0011077	1.802877	-0.024977	-0.223208	-0.124093	down
A_33_P3295108	GANC	1.4571724	1.2300143	1.436131	-0.227158	-0.021041	-0.1241	down
A_23_P171385	PHF6	1.8649302	1.5394559	1.9421644	-0.325474	0.0772343	-0.12412	down
A_33_P3330404	FAM180B	-0.693285	-0.732677	-0.902427	-0.039392	-0.209142	-0.124267	down
A_23_P208706	BAX	4.8214836	4.3288465	5.06557	-0.492637	0.2440863	-0.124275	down
A_24_P43876	C9orf40	0.9458542	0.691711	0.951364	-0.254143	0.0055099	-0.124317	down
A_23_P157726	DENND4C	1.9452162	1.8294144	1.812367	-0.115802	-0.132849	-0.124326	down
A_23_P74162	IQCC	-1.258462	-1.387847	-1.377768	-0.129385	-0.119306	-0.124345	down
A_21_P0008964	lnc-CDH3-1	-1.770782	-2.217928	-1.572347	-0.447147	0.1984348	-0.124356	down
A_33_P3328445	ALOX12-AS1	-1.546831	-1.922919	-1.419515	-0.376088	0.127316	-0.124386	down
A_23_P157607	INTS10	4.303631	4.1674657	4.1909075	-0.136165	-0.112723	-0.124444	down
A_24_P220771	ANKRD17	-1.6963	-2.078634	-1.56288	-0.382334	0.13342	-0.124457	down
A_23_P379614	OIP5	4.60966	4.525227	4.445162	-0.084433	-0.164498	-0.124466	down
A_23_P253301	PFN2	1.3646045	1.3288198	1.1514225	-0.035785	-0.213182	-0.124483	down
A_33_P3339212	TRIP13	4.5757866	4.5592	4.3433533	-0.016587	-0.232433	-0.12451	down
A_24_P365025	SPAG9	2.793954	2.7713866	2.5674887	-0.022567	-0.226465	-0.124516	down

A_23_P58337	FIP1L1	4.4533663	4.250444	4.4070263	-0.202922	-0.04634	-0.124631	down
A_23_P200829	SRGAP2	3.4051018	3.2494197	3.3114996	-0.155682	-0.093602	-0.124642	down
A_23_P102060	SSFA2	3.8592672	3.6084027	3.860818	-0.250865	0.0015507	-0.124657	down
A_23_P348749	AK9	-2.043095	-1.6411	-2.69445	0.4019952	-0.651356	-0.12468	down
A_24_P336705	RABGGTB	3.0631437	3.0185704	2.858241	-0.044573	-0.204903	-0.124738	down
A_24_P73290	ATP2A2	1.90487	1.5498223	2.0103798	-0.355048	0.1055098	-0.124769	down
A_33_P3315410	COIL	4.020152	3.6436124	4.1471167	-0.37654	0.1269646	-0.124788	down
A_23_P414964	ZNF584	1.4406705	1.2558956	1.3756461	-0.184775	-0.065024	-0.1249	down
A_23_P84929	SLC38A5	-2.284089	-2.36669	-2.451325	-0.082601	-0.167236	-0.124919	down
A_23_P391164	ZNF512	1.7682371	1.4756413	1.8109508	-0.292596	0.0427136	-0.124941	down
A_21_P0012669	XLOC_l2_011151	-2.454223	-2.209844	-2.948687	0.2443793	-0.494463	-0.125042	down
A_24_P941505	FAM120A	4.6290503	4.294374	4.7135515	-0.334676	0.0845013	-0.125088	down
A_24_P8220	HS6ST1	3.3907862	3.2093577	3.321516	-0.181428	-0.06927	-0.125349	down
A_23_P164000	SENP3	1.5922451	1.3501782	1.5835552	-0.242067	-0.00869	-0.125378	down
A_24_P940620	CCDC57	-0.985687	-1.169506	-1.052645	-0.183818	-0.066958	-0.125388	down
A_24_P67929	KANSL2	-0.710849	-0.765628	-0.90687	-0.054779	-0.196021	-0.1254	down
A_21_P0013271	SMKR1	0.1921439	-0.121877	0.2553062	-0.314021	0.0631623	-0.125429	down
A_19_P00800206	LOC400958	0.8265176	0.8292255	0.5724754	0.002708	-0.254042	-0.125667	down
A_23_P91891	COPB2	5.5883627	5.365662	5.5596857	-0.222701	-0.028677	-0.125689	down
A_23_P124003	P2RX2	-2.3994	-2.407732	-2.642515	-0.008331	-0.243115	-0.125723	down
A_21_P0000137	BEAN1	-1.862468	-1.837667	-2.138757	0.0248013	-0.276289	-0.125744	down
A_23_P420831	TRIM10	-0.157439	-0.471321	-0.095049	-0.313882	0.0623899	-0.125746	down
A_33_P3239242	SPATA6	-3.089639	-3.149065	-3.28171	-0.059425	-0.192071	-0.125748	down
A_23_P54540	EIF2AK4	2.706684	2.611587	2.5502129	-0.095097	-0.156471	-0.125784	down
A_23_P62276	NKAP	1.1227198	1.2234521	0.770411	0.1007323	-0.352309	-0.125788	down
A_33_P3211604	RAP1B	5.4563246	5.3080974	5.3529425	-0.148227	-0.103382	-0.125805	down
A_21_P0013355	LOC101930608	-2.29473	-2.445576	-2.395624	-0.150846	-0.100894	-0.12587	down
A_23_P66664	TADA2A	-0.555337	-0.634042	-0.728473	-0.078705	-0.173136	-0.125921	down
A_23_P112135	TRAM1	3.724121	3.6099925	3.5863657	-0.114129	-0.137755	-0.125942	down
A_23_P218086	TPCN1	4.224659	4.108993	4.088356	-0.115666	-0.136303	-0.125984	down
A_24_P277367	CXCL5	3.449664	3.295012	3.3523035	-0.154652	-0.097361	-0.126006	down
A_21_P0011536	LRRC37BP1	-0.65616	-0.860739	-0.703821	-0.204579	-0.047661	-0.12612	down
A_33_P3374758	DYNC1LI2	1.5570412	1.3316178	1.5301657	-0.225423	-0.026875	-0.126149	down
A_23_P218597	NPAS2	-1.446796	-1.714547	-1.431415	-0.26775	0.0153813	-0.126184	down
A_24_P76546	WASF2	1.4125934	0.9944043	1.5783095	-0.418189	0.1657162	-0.126236	down
A_21_P0004345	lnc-CNOT6-1	-2.67933	-2.911577	-2.699931	-0.232247	-0.020601	-0.126424	down
A_23_P96641	PRPS2	1.1679301	0.7825513	1.3000102	-0.385379	0.1320801	-0.126649	down
A_33_P3298425	LMO4	1.9347706	2.0334258	1.5827131	0.0986552	-0.352057	-0.126701	down
A_24_P399009	EDC4	-0.47719	-0.988384	-0.219732	-0.511195	0.2574577	-0.126868	down
A_23_P109774	ZBTB11	2.285551	2.2361016	2.0812454	-0.049449	-0.204306	-0.126878	down
A_22_P00010860	SEC22B	0.7416496	0.5486689	0.6808157	-0.192981	-0.060834	-0.126907	down
A_23_P127406	KDM4D	-0.225997	-0.318508	-0.387421	-0.092511	-0.161425	-0.126968	down
A_23_P213137	LNX1	-2.173438	-2.142339	-2.458507	0.0310991	-0.285069	-0.126985	down
A_21_P0011284	BMS1P20	-0.792771	-1.026521	-0.813014	-0.23375	-0.020243	-0.126997	down
A_24_P296070	COG3	1.2768536	1.2019157	1.0976329	-0.074938	-0.179221	-0.127079	down
A_24_P97197	CTDSP1	0.4234939	0.1673727	0.4253016	-0.256121	0.0018077	-0.127157	down
A_21_P0010775	PARP8	2.2851553	2.220327	2.0955868	-0.064828	-0.189569	-0.127198	down
A_23_P153286	ZNF234	3.1252623	2.9925752	3.0035229	-0.132687	-0.121739	-0.127213	down
A_24_P270769	VPS35	5.9682646	5.735995	5.9460287	-0.23227	-0.022236	-0.127253	down
A_32_P4403	C16orf72	2.8083572	2.6172366	2.744957	-0.191121	-0.0634	-0.12726	down
A_23_P141715	TPGS2	4.287258	3.9812608	4.338703	-0.305997	0.051445	-0.127276	down
A_23_P410965	KIAA1522	6.4712257	6.168263	6.519537	-0.302963	0.0483112	-0.127326	down
A_22_P00012743	LOC100996437	-2.097108	-2.185799	-2.263167	-0.088691	-0.166059	-0.127375	down
A_32_P14610	PDLIM5	3.937728	3.7378097	3.8828049	-0.199918	-0.054923	-0.127421	down
A_33_P3400758	ZNF555	-1.12142	-1.11372	-1.383978	0.0077	-0.262558	-0.127429	down
A_33_P3527721	LOC284219	-1.581777	-1.316598	-2.101821	0.2651787	-0.520044	-0.127433	down

A_32_P6832	C5orf51	2.2728271	2.0758414	2.2147284	-0.196986	-0.058099	-0.127542	down
A_33_P3284557	ZAK	3.8060226	3.710607	3.646162	-0.095416	-0.159861	-0.127638	down
A_23_P19938	KDELR2	4.522683	4.2154393	4.5746107	-0.307244	0.0519276	-0.127658	down
A_33_P3279059	RIPK1	2.5488272	2.2884421	2.5538692	-0.260385	0.0050421	-0.127671	down
A_22_P00025777	lnc-NOM1-6	-1.65328	-1.987923	-1.573987	-0.334643	0.0792933	-0.127675	down
A_24_P37441	PDK1	0.716692	0.7015715	0.4764037	-0.015121	-0.240288	-0.127704	down
A_24_P407235	CRY1	1.0278616	0.843976	0.9563022	-0.183886	-0.071559	-0.127723	down
A_24_P920188	ZNF24	0.4840403	0.2939677	0.4185209	-0.190073	-0.065519	-0.127796	down
A_23_P1833	B3GAT1	-2.228289	-2.817592	-1.894631	-0.589303	0.3336577	-0.127823	down
A_33_P3254634	PDIA5	0.2974248	0.2071629	0.1320396	-0.090262	-0.165385	-0.127824	down
A_23_P213699	NRG2	-1.606566	-1.781184	-1.687902	-0.174617	-0.081336	-0.127976	down
A_23_P40307	SNRPB2	4.4470186	4.2788825	4.3591375	-0.168136	-0.087881	-0.128009	down
A_22_P00001709	lnc-ATG16L2-1	-2.863326	-2.89955	-3.083248	-0.036224	-0.219922	-0.128073	down
A_33_P3243364	ERCC4	-1.916985	-1.860107	-2.230203	0.0568776	-0.313219	-0.128171	down
A_33_P3321293	IQGAP3	3.95617	3.8997378	3.756236	-0.056432	-0.199934	-0.128183	down
A_23_P112950	ATF7IP	2.2454615	2.2035213	2.0308533	-0.04194	-0.214608	-0.128274	down
A_33_P3359473	SUV420H2	2.8346796	2.6721911	2.7405462	-0.162488	-0.094133	-0.128311	down
A_23_P94494	TLE4	-0.014867	-0.433567	0.1470776	-0.4187	0.1619444	-0.128378	down
A_24_P109432	NBEAL1	1.2724862	1.3037777	0.9843879	0.0312915	-0.288098	-0.128403	down
A_23_P91081	EPCAM	7.379964	7.2381425	7.2647953	-0.141821	-0.115169	-0.128495	down
A_21_P0014376	LOC101930634	-2.408944	-2.527011	-2.547904	-0.118067	-0.138961	-0.128514	down
A_19_P00319525	TSTD3	1.6039429	1.526423	1.4244294	-0.07752	-0.179513	-0.128517	down
A_21_P0008791	LINC01197	-1.957723	-2.242531	-1.929999	-0.284807	0.0277243	-0.128542	down
A_23_P113471	FAAH2	2.5890694	2.5050912	2.4158354	-0.083978	-0.173234	-0.128606	down
A_33_P3264612	TPCN2	0.942174	0.7241497	0.9029732	-0.218024	-0.039201	-0.128613	down
A_33_P3222744	ZNF117	-2.121823	-1.846632	-2.654385	0.2751913	-0.532562	-0.128685	down
A_32_P139894	ABL2	2.6081867	2.7377534	2.2212029	0.1295667	-0.386984	-0.128709	down
A_23_P338479	CD274	-2.351778	-2.996742	-1.964469	-0.644964	0.3873091	-0.128828	down
A_23_P20697	ADAMTSL2	0.298213	0.4003868	-0.061673	0.1021738	-0.359886	-0.128856	down
A_23_P406438	SRPK2	0.442986	0.3591976	0.2690387	-0.083788	-0.173947	-0.128868	down
A_23_P145541	FIG4	0.6458159	0.5234914	0.5103726	-0.122324	-0.135443	-0.128884	down
A_33_P3808371	LOC283911	2.9850988	2.7433257	2.9685965	-0.241773	-0.016502	-0.129138	down
A_23_P110445	APBB3	0.3993163	0.3850884	0.1552444	-0.014228	-0.244072	-0.12915	down
A_23_P349310	TNRC6A	1.2096977	0.9964991	1.1645899	-0.213199	-0.045108	-0.129153	down
A_23_P142714	SLC25A12	3.3477964	3.0364223	3.4008398	-0.311374	0.0530434	-0.129165	down
A_24_P941930	EAF1	-0.293565	-0.587657	-0.258023	-0.294092	0.035542	-0.129275	down
A_33_P3331906	SNTB2	1.6080427	1.4664893	1.4909792	-0.141553	-0.117064	-0.129308	down
A_24_P308851	PCDHGA7	-2.911918	-2.720546	-3.362159	0.1913719	-0.450241	-0.129434	down
A_23_P119254	ASF1B	3.1691132	2.8879619	3.1913376	-0.281151	0.0222244	-0.129463	down
A_22_P00025539	LOC101930240	-1.797904	-1.885016	-1.969906	-0.087112	-0.172003	-0.129558	down
A_24_P248863	ZCCHC4	0.3921065	0.3415976	0.1832905	-0.050509	-0.208816	-0.129663	down
A_33_P3376449	ZDHHC23	-1.707926	-1.976521	-1.698664	-0.268595	0.0092621	-0.129666	down
A_24_P303594	ACTR3B	-0.361156	-0.598927	-0.382996	-0.237772	-0.021841	-0.129806	down
A_23_P356965	CCDC57	-1.034291	-1.145152	-1.183427	-0.110861	-0.149136	-0.129998	down
A_33_P3293247	GPSM1	-0.185403	-0.522134	-0.108678	-0.336731	0.0767245	-0.130003	down
A_23_P94501	ANXA1	8.28016	8.282143	8.018124	0.0019827	-0.262036	-0.130027	down
A_23_P128166	RAB21	4.036948	3.8596406	3.954198	-0.177308	-0.08275	-0.130029	down
A_33_P3212782	CALM2	5.7763386	5.713959	5.578636	-0.062379	-0.197702	-0.130041	down
A_23_P257198	NDUFS4	5.4428024	5.2436833	5.3818073	-0.199119	-0.060995	-0.130057	down
A_23_P90804	MAP4K4	0.298975	-0.023699	0.3614788	-0.322674	0.0625038	-0.130085	down
A_23_P397019	DNAJC25	-0.71203	-0.903305	-0.781124	-0.191274	-0.069093	-0.130184	down
A_23_P428849	CHCHD4	3.9131079	3.619966	3.9452152	-0.293142	0.0321074	-0.130517	down
A_23_P155288	GFM1	3.5382042	3.2726598	3.5424776	-0.265544	0.0042734	-0.130635	down
A_33_P3396129	KATNB1	-2.312171	-2.48263	-2.403078	-0.170459	-0.090908	-0.130684	down
A_23_P364766	SLX4IP	1.4638596	1.1666493	1.4996953	-0.29721	0.0358357	-0.130687	down
A_24_P98277	GOLGA7	1.9123077	1.7258615	1.8373356	-0.186446	-0.074972	-0.130709	down

A_23_P409553	PPM1A	-1.995582	-1.465768	-2.786933	0.5298138	-0.791351	-0.130769	down
A_23_P50426	KANK2	3.4366245	3.399465	3.2120247	-0.037159	-0.2246	-0.13088	down
A_33_P3783812	C8orf60	-0.354756	-0.547037	-0.424271	-0.192281	-0.069515	-0.130898	down
A_21_P0004548	ZDHHC11	-1.154613	-1.47528	-1.095783	-0.320667	0.0588298	-0.130919	down
A_21_P0012983	LOC100506674	0.1996985	0.2633615	-0.12606	0.063663	-0.325759	-0.131048	down
A_33_P6559875	ZNF518A	0.1028442	0.2538629	-0.310321	0.1510186	-0.413166	-0.131073	down
A_33_P3212650	HDDC2	4.1252384	3.9783273	4.0098925	-0.146911	-0.115346	-0.131129	down
A_24_P252043	PTP4A1	0.0460205	-0.284412	0.1141796	-0.330432	0.0681591	-0.131137	down
A_21_P0000475	SNORA70B	1.6936798	1.682436	1.442606	-0.011244	-0.251074	-0.131159	down
A_23_P254842	HDHD1	2.1942196	1.9664474	2.1596575	-0.227772	-0.034562	-0.131167	down
A_33_P3330991	LOC100134237	-0.391334	-0.400269	-0.644816	-0.008934	-0.253482	-0.131208	down
A_23_P93009	SRP19	0.4187536	0.3183022	0.2566705	-0.100451	-0.162083	-0.131267	down
A_33_P3349947	TCP1	4.020261	3.988501	3.7894201	-0.03176	-0.230841	-0.1313	down
A_24_P364970	DHX33	0.8719521	0.5677738	0.9135227	-0.304178	0.0415707	-0.131304	down
A_23_P5945	DHX35	2.8990965	2.931232	2.6042223	0.0321355	-0.294874	-0.131369	down
A_23_P502641	SEC22C	0.3657689	0.1036477	0.3650699	-0.262121	-0.000699	-0.13141	down
A_33_P3230881	ALG10	-0.186601	-0.074754	-0.56144	0.1118474	-0.374839	-0.131496	down
A_23_P333218	ERGIC1	4.7339563	4.322389	4.8824453	-0.411567	0.148489	-0.131539	down
A_23_P202939	APLP2	6.5605106	6.4881444	6.369467	-0.072366	-0.191044	-0.131705	down
A_23_P113825	NACC2	3.6166935	3.6135497	3.3562422	-0.003144	-0.260451	-0.131798	down
A_33_P3406939	KIF24	0.8936601	0.6445279	0.8791962	-0.249132	-0.014464	-0.131798	down
A_24_P48057	IRX5	0.9932127	0.7699108	0.9528074	-0.223302	-0.040405	-0.131854	down
A_23_P141770	NAPG	6.3570423	6.2497888	6.2005234	-0.107254	-0.156519	-0.131886	down
A_33_P3613688	FER1L6-AS2	-1.149434	-1.41011	-1.152553	-0.260676	-0.003119	-0.131897	down
A_24_P112941	LINGO1	-1.218771	-1.761102	-0.940264	-0.542331	0.2785068	-0.131912	down
A_21_P0006584	NKAPP1	-0.277175	-0.440053	-0.37814	-0.162878	-0.100965	-0.131921	down
A_23_P318039	RQCD1	-0.721485	-0.722055	-0.984856	-0.00057	-0.263371	-0.13197	down
A_32_P13795	DNAJC27	-0.311337	-0.362769	-0.523875	-0.051431	-0.212537	-0.131984	down
A_33_P3229722	PSMF1	2.2759657	1.848207	2.4396381	-0.427759	0.1636725	-0.132043	down
A_22_P00020547	lnc-ATF7IP2-1	-1.666961	-1.972726	-1.625304	-0.305765	0.0416574	-0.132054	down
A_23_P122464	ZSCAN9	-0.225287	-0.523002	-0.191736	-0.297715	0.0335507	-0.132082	down
A_23_P500010	KLK12	-2.072634	-2.54464	-1.864824	-0.472006	0.2078104	-0.132098	down
A_21_P0014327	FTO-IT1	-1.131764	-1.279381	-1.248361	-0.147616	-0.116597	-0.132107	down
A_22_P00012423	lnc-PRM1-1	-2.587946	-2.314735	-3.125449	0.2732115	-0.537503	-0.132146	down
A_21_P0011694	LINC01535	-1.3395	-1.516635	-1.426761	-0.177135	-0.087262	-0.132199	down
A_21_P0010593	XLOC_l2_000727	-1.871946	-2.011387	-1.996996	-0.139441	-0.125051	-0.132246	down
A_33_P3290667	SELT	2.6532955	2.5710502	2.4709034	-0.082245	-0.182392	-0.132319	down
A_32_P148672	SNRPD1	5.8436403	5.649278	5.773321	-0.194362	-0.070319	-0.132341	down
A_19_P00316758	MAP3K13	3.7012472	3.3435845	3.7941847	-0.357663	0.0929375	-0.132363	down
A_24_P411899	RNF19A	1.5541344	1.3998117	1.4436789	-0.154323	-0.110456	-0.132389	down
A_33_P3247659	TMEM87A	0.4579368	0.3507919	0.3000488	-0.107145	-0.157888	-0.132516	down
A_23_P73023	TBC1D1	1.6990709	1.4117589	1.7213221	-0.287312	0.0222511	-0.13253	down
A_23_P84334	TMEM39A	1.2405238	1.3476315	0.8683081	0.1071076	-0.372216	-0.132554	down
A_23_P11543	FUCA1	1.9964466	1.6328864	2.0948677	-0.36356	0.0984211	-0.13257	down
A_24_P294124	SERTAD2	2.8041553	2.6784472	2.6645308	-0.125708	-0.139625	-0.132666	down
A_23_P52176	ZNF124	1.7530689	1.5820608	1.6587329	-0.171008	-0.094336	-0.132672	down
A_22_P00019529	LOC102724096	-1.143199	-1.066012	-1.48585	0.0771866	-0.342651	-0.132732	down
A_23_P250994	ANAPC10	2.3985052	2.16114	2.3703928	-0.237365	-0.028112	-0.132739	down
A_21_P0014494	CTC-338M12.4	2.7279997	2.5907636	2.5997133	-0.137236	-0.128286	-0.132761	down
A_22_P00017056	lnc-TUBB-7	-1.156971	-1.054247	-1.525501	0.1027241	-0.36853	-0.132903	down
A_23_P304991	HLCS	1.8544598	1.4948249	1.9482079	-0.359635	0.0937481	-0.132943	down
A_33_P3284662	EID2	2.007576	1.6565704	2.0926723	-0.351006	0.0850964	-0.132955	down
A_23_P48676	PYGL	4.372279	4.1885467	4.289818	-0.183733	-0.082461	-0.133097	down
A_24_P360722	DIP2C	0.1833911	0.0950289	0.0055299	-0.088362	-0.177861	-0.133112	down
A_23_P73208	GABPB2	1.6507444	1.6984615	1.336668	0.0477171	-0.314076	-0.13318	down
A_23_P334263	SENP8	-0.569148	-0.852379	-0.552388	-0.283231	0.0167599	-0.133236	down

A_23_P145175	ZNF318	0.3748388	0.3309779	0.1521974	-0.043861	-0.222641	-0.133251	down
A_23_P150931	LMBR1L	-0.147559	-0.411208	-0.150484	-0.263649	-0.002925	-0.133287	down
A_23_P32913	ARMC10	4.096513	3.9791083	3.9469357	-0.117404	-0.149577	-0.133491	down
A_23_P217079	DPM2	2.608986	1.9612222	2.9896784	-0.647764	0.3806925	-0.133536	down
A_32_P28402	LRRC37BP1	0.9194551	0.7140169	0.857657	-0.205438	-0.061798	-0.133618	down
A_23_P200001	NEXN	-2.354514	-2.061636	-2.914686	0.2928782	-0.560173	-0.133647	down
A_33_P3367471	PLEKHG1	-1.314139	-1.308546	-1.587386	0.0055938	-0.273246	-0.133826	down
A_21_P0009442	LINC01540	-1.462084	-1.98545	-1.206521	-0.523366	0.2555633	-0.133901	down
A_21_P0006728	LINC00704	-2.08771	-1.951403	-2.491869	0.1363072	-0.404159	-0.133926	down
A_22_P00018022	ZNF207	0.5659671	0.4263191	0.4376993	-0.139648	-0.128268	-0.133958	down
A_23_P210176	ITGA6	4.734874	4.597691	4.603836	-0.137183	-0.131038	-0.13411	down
A_23_P382199	VAPA	3.7508001	3.5521207	3.6811275	-0.198679	-0.069673	-0.134176	down
A_23_P202737	JRKL	0.4394484	0.194407	0.4158559	-0.245041	-0.023592	-0.134317	down
A_33_P3218760	UPF2	4.223441	4.063764	4.1143513	-0.159677	-0.10909	-0.134383	down
A_22_P00008459	LOC101927221	-1.846978	-2.164122	-1.798915	-0.317145	0.0480623	-0.134541	down
A_33_P3374443	L1CAM	-1.438755	-1.593294	-1.553368	-0.15454	-0.114613	-0.134576	down
A_23_P50096	TYMS	6.04432	5.8824267	5.936857	-0.161893	-0.107463	-0.134678	down
A_23_P134109	EPB41L2	1.0070944	1.0545769	0.6900716	0.0474825	-0.317023	-0.13477	down
A_23_P40693	EP300	1.3901529	1.3738465	1.1368942	-0.016306	-0.253259	-0.134783	down
A_32_P234935	TARDBP	6.246503	6.023865	6.1995487	-0.222638	-0.046954	-0.134796	down
A_24_P418637	MACF1	2.307559	2.108293	2.237155	-0.199266	-0.070404	-0.134835	down
A_23_P96165	C11orf80	-0.790758	-0.655848	-1.195341	0.1349101	-0.404583	-0.134837	down
A_24_P242609	KLHL12	2.2542133	2.2102075	2.0284872	-0.044006	-0.225726	-0.134866	down
A_33_P3216955	ANAPC16	1.5425668	1.4391518	1.3761702	-0.103415	-0.166397	-0.134906	down
A_33_P3392250	FAM178A	0.4458132	0.2108207	0.4109521	-0.234993	-0.034861	-0.134927	down
A_33_P3261737	SLC35A2	-0.347842	-0.5219	-0.443989	-0.174058	-0.096148	-0.135103	down
A_33_P3417318	ZNF891	-1.923224	-1.816804	-2.300011	0.106421	-0.376786	-0.135183	down
A_33_P3299977	NAA35	3.1840382	3.175263	2.9223843	-0.008775	-0.261654	-0.135215	down
A_23_P103628	HEATR1	3.869195	3.661553	3.8063908	-0.207642	-0.062804	-0.135223	down
A_23_P202496	NOC3L	3.1283455	2.8810544	3.105177	-0.247291	-0.023169	-0.13523	down
A_23_P213754	PAIP2	3.8314114	3.7278123	3.6643057	-0.103599	-0.167106	-0.135352	down
A_33_P3397658	SYNPO	2.7090225	2.816605	2.3306904	0.1075826	-0.378332	-0.135375	down
A_24_P332926	SUGP2	1.7689562	1.6169205	1.6502357	-0.152036	-0.118721	-0.135378	down
A_33_P3210146	PRR9	-0.713509	-1.065289	-0.632876	-0.35178	0.0806322	-0.135574	down
A_21_P0000917	lnc-CHD1L-1	-1.816352	-2.058064	-1.846043	-0.241711	-0.029691	-0.135701	down
A_23_P80062	TAF4	1.5288529	1.3919835	1.3942938	-0.136869	-0.134559	-0.135714	down
A_33_P3420402	HACE1	-2.189516	-2.28973	-2.360735	-0.100214	-0.171219	-0.135717	down
A_23_P32217	TOPORS	0.5375557	0.428124	0.3754554	-0.109432	-0.1621	-0.135766	down
A_21_P0014174	lnc-PSMG4-1	-1.933705	-2.161509	-1.977448	-0.227804	-0.043743	-0.135773	down
A_33_P3398065	TPD52L1	1.0643044	0.9692435	0.8877583	-0.095061	-0.176546	-0.135803	down
A_33_P3210423	MED1	3.0020514	2.965754	2.7665892	-0.036297	-0.235462	-0.13588	down
A_23_P206059	PRC1	5.245431	4.9320054	5.286991	-0.313426	0.0415602	-0.135933	down
A_24_P942517	TMX4	2.073555	1.9521918	1.9227715	-0.121363	-0.150784	-0.136073	down
A_23_P75204	NSMCE4A	3.917284	3.8389277	3.7231245	-0.078356	-0.19416	-0.136258	down
A_23_P124742	CHKA	4.8008327	4.6582627	4.670782	-0.14257	-0.130051	-0.13631	down
A_23_P92543	SCFD2	2.0640173	1.6274333	2.227971	-0.436584	0.1639538	-0.136315	down
A_24_P245108	USP7	-0.679295	-0.988776	-0.642464	-0.309481	0.0368304	-0.136325	down
A_22_P00016095	SYNPR-AS1	-1.712812	-2.186397	-1.511923	-0.473585	0.2008891	-0.136348	down
A_24_P160874	DUT	4.375613	4.2527585	4.225727	-0.122855	-0.149886	-0.13637	down
A_33_P3424143	AREL1	-0.708864	-0.764949	-0.925709	-0.056086	-0.216846	-0.136466	down
A_21_P0007250	RAB30-AS1	1.9331169	1.6739697	1.9189281	-0.259147	-0.014189	-0.136668	down
A_32_P191503	LINC00265	1.7116079	1.5204835	1.6293764	-0.191124	-0.082232	-0.136678	down
A_33_P3321602	PHACTR4	-1.931729	-2.382338	-1.754552	-0.450608	0.177177	-0.136716	down
A_24_P487736	CXorf23	-0.543109	-0.529461	-0.830225	0.0136476	-0.287116	-0.136734	down
A_23_P169154	ERMP1	1.5133324	1.5152235	1.2379231	0.0018911	-0.275409	-0.136759	down
A_23_P300905	CCNY	1.8149891	1.7193046	1.6371264	-0.095685	-0.177863	-0.136774	down

A_23_P385034	E2F3	2.769081	2.484446	2.7801504	-0.284635	0.0110693	-0.136783	down
A_24_P62615	CAP1	3.9212103	3.7162004	3.8525696	-0.20501	-0.068641	-0.136825	down
A_33_P3317198	GTF2F2	3.9857626	3.802837	3.8948822	-0.182926	-0.09088	-0.136903	down
A_23_P408167	GPRASP2	-2.1628	-2.563808	-2.035692	-0.401008	0.1271076	-0.13695	down
A_23_P53646	NAP1L1	5.7799788	5.993998	5.2919464	0.2140193	-0.488032	-0.137007	down
A_33_P3238052	FAM216A	2.1704798	1.8873	2.1795616	-0.28318	0.0090818	-0.137049	down
A_23_P395534	RSC1A1	-0.00727	-0.233057	-0.055597	-0.225786	-0.048326	-0.137056	down
A_23_P170839	SEPT2	4.539051	4.339148	4.4648333	-0.199903	-0.074218	-0.13706	down
A_32_P156892	ST13	4.6360655	4.4095387	4.5883694	-0.226527	-0.047696	-0.137111	down
A_33_P3411025	ARHGAP19	-0.549202	-0.986574	-0.386424	-0.437372	0.1627784	-0.137297	down
A_24_P409857	HMGN1	6.252616	6.0498347	6.1807413	-0.202781	-0.071875	-0.137328	down
A_22_P00017739	MCM3AP-AS1	-2.194727	-2.523614	-2.140576	-0.328886	0.0541515	-0.137367	down
A_23_P139418	GALNT18	2.0449142	1.68221	2.1328783	-0.362704	0.0879641	-0.13737	down
A_33_P3380612	HSPG2	-3.037781	-3.149068	-3.201266	-0.111287	-0.163484	-0.137385	down
A_33_P3386760	CHEK2	-0.187032	-0.384717	-0.264384	-0.197685	-0.077352	-0.137519	down
A_33_P3407700	TMEM192	-1.155041	-1.029863	-1.555324	0.1251783	-0.400283	-0.137552	down
A_23_P120237	STARD7	5.664733	5.423034	5.63128	-0.241699	-0.033453	-0.137576	down
A_19_P00813482	CRHR1-IT1	-0.313396	-0.585553	-0.316395	-0.272157	-0.002999	-0.137578	down
A_19_P00327035	HAR1A	-1.993302	-2.03751	-2.224315	-0.044208	-0.231013	-0.137611	down
A_23_P359277	ELOVL7	1.4118042	1.2174616	1.3307424	-0.194343	-0.081062	-0.137702	down
A_21_P0008670	LINC00925	-2.189308	-2.201527	-2.452525	-0.012219	-0.263217	-0.137718	down
A_23_P13822	STYK1	0.5488687	0.3996091	0.4224715	-0.14926	-0.126397	-0.137828	down
A_33_P3377619	CACUL1	3.4005032	3.1476283	3.377715	-0.252875	-0.022788	-0.137831	down
A_23_P208325	ZNF235	-0.837934	-0.828394	-1.123181	0.0095391	-0.285247	-0.137854	down
A_24_P107291	PPP2R1B	1.5108395	1.3197174	1.4262509	-0.191122	-0.084589	-0.137855	down
A_22_P00022161	ADNP-AS1	-1.964812	-2.261898	-1.943558	-0.297086	0.0212536	-0.137916	down
A_33_P3214874	KPNA6	4.7788715	4.113624	5.168103	-0.665247	0.3892317	-0.138008	down
A_33_P3883985	LMF1	2.3810291	2.103825	2.3819828	-0.277204	0.0009537	-0.138125	down
A_33_P3403361	TIMM23	3.6822176	3.4979548	3.5900002	-0.184263	-0.092217	-0.13824	down
A_33_P3378880	HIST1H4C	2.0490894	1.8458333	1.9758272	-0.203256	-0.073262	-0.138259	down
A_23_P14157	DZIP1	-1.556505	-2.039764	-1.349765	-0.483258	0.2067399	-0.138259	down
A_33_P3368646	CNKSR3	-2.747105	-2.824629	-2.946184	-0.077524	-0.199079	-0.138301	down
A_33_P3415240	LINC00886	-2.231391	-2.335238	-2.404148	-0.103847	-0.172758	-0.138302	down
A_24_P4661	EI24	4.080287	3.9821668	3.901165	-0.09812	-0.179122	-0.138621	down
A_24_P12626	CAV1	0.5760317	0.3933053	0.4815116	-0.182726	-0.09452	-0.138623	down
A_22_P00015435	LINC00886	-2.186298	-2.241643	-2.40831	-0.055345	-0.222012	-0.138679	down
A_22_P00007826	lnc-HPD-1	-1.494901	-2.026036	-1.241138	-0.531135	0.2537637	-0.138686	down
A_33_P3281816	CAP1	2.159793	1.9343266	2.107853	-0.225466	-0.05194	-0.138703	down
A_24_P916656	CRCP	1.0778103	0.5689669	1.3088675	-0.508843	0.2310572	-0.138893	down
A_33_P3413821	KIRREL	-1.15583	-1.407627	-1.18198	-0.251796	-0.02615	-0.138973	down
A_23_P123727	ZCCHC6	0.1700659	-0.019185	0.0813246	-0.189251	-0.088741	-0.138996	down
A_23_P43226	KCTD9	2.4629211	2.3183303	2.329464	-0.144591	-0.133457	-0.139024	down
A_33_P3327852	NDEL1	-1.479437	-1.557873	-1.679141	-0.078436	-0.199704	-0.13907	down
A_24_P237661	NCOR1	-0.006769	-0.2653	-0.026438	-0.258531	-0.019669	-0.1391	down
A_33_P3264505	FCF1	2.1353788	2.0049434	1.9874954	-0.130435	-0.147883	-0.139159	down
A_32_P99032	LOC100240734	1.3435469	1.2554145	1.1533027	-0.088132	-0.190244	-0.139188	down
A_23_P152807	RSAD1	4.053671	3.7683454	4.060565	-0.285326	0.0068941	-0.139216	down
A_22_P00006179	LOC100507642	-2.000137	-2.027101	-2.251731	-0.026964	-0.251594	-0.139279	down
A_24_P43092	SEPT2	0.7693224	0.6891708	0.570869	-0.080152	-0.198453	-0.139302	down
A_22_P00003932	CFLAR	-0.122613	-0.269351	-0.254563	-0.146738	-0.131949	-0.139344	down
A_33_P3416350	ITSN2	-0.907154	-0.909741	-1.183304	-0.002588	-0.276151	-0.139369	down
A_23_P34757	GPATCH2	0.5921879	0.3931317	0.512413	-0.199056	-0.079775	-0.139416	down
A_22_P00018375	lnc-AVEN-1	-2.786247	-3.004835	-2.84651	-0.218588	-0.060262	-0.139425	down
A_22_P00002000	FBXL19-AS1	1.5136304	1.4444933	1.3038421	-0.069137	-0.209788	-0.139463	down
A_22_P00022512	lnc-OPRL1-1	-2.220281	-2.32177	-2.397838	-0.101489	-0.177557	-0.139523	down
A_33_P3266828	TMEM8B	-0.053336	-0.226296	-0.15944	-0.17296	-0.106104	-0.139532	down

A_33_P3342345	XLOC_l2_004817	-1.879002	-2.1946	-1.842479	-0.315598	0.0365224	-0.139538	down
A_33_P3384710	FAM222B	0.4318733	0.2985139	0.2857385	-0.133359	-0.146135	-0.139747	down
A_21_P0000637	TRAF3IP2-AS1	3.4806185	3.4185338	3.2632027	-0.062085	-0.217416	-0.13975	down
A_21_P0000820	NRAV	-1.522198	-1.779134	-1.544923	-0.256936	-0.022725	-0.139831	down
A_33_P3408722	FAM219A	-3.022396	-2.871254	-3.453296	0.1511412	-0.430901	-0.13988	down
A_23_P110851	TERT	-0.068464	-0.321183	-0.095592	-0.252719	-0.027128	-0.139924	down
A_23_P201287	KIF1B	1.1395221	1.1497645	0.8493528	0.0102425	-0.290169	-0.139963	down
A_33_P3274049	ACOXL	-2.792093	-2.922204	-2.941913	-0.130111	-0.149819	-0.139965	down
A_23_P319598	C4BPB	-0.154176	-0.156952	-0.431396	-0.002776	-0.27722	-0.139998	down
A_33_P3237359	HMGB3	1.4479718	1.357235	1.258502	-0.090737	-0.18947	-0.140103	down
A_21_P0000750	MFI2-AS1	0.6799011	0.3826971	0.6967368	-0.297204	0.0168357	-0.140184	down
A_21_P0005118	lnc-SH3BGRL2-1	-0.128189	-0.608073	0.0713172	-0.479884	0.1995063	-0.140189	down
A_33_P3347132	EXOSC2	2.350216	2.2137265	2.2057838	-0.136489	-0.144432	-0.140461	down
A_33_P3231120	CASD1	-2.801785	-3.067447	-2.817127	-0.265662	-0.015342	-0.140502	down
A_33_P3369530	MAGI1	-2.35223	-2.604809	-2.380688	-0.252579	-0.028458	-0.140518	down
A_23_P162918	SERPINA3	-2.083205	-2.16394	-2.283568	-0.080734	-0.200363	-0.140549	down
A_24_P310894	CAPZA1	6.1333237	5.970323	6.0152044	-0.163001	-0.118119	-0.14056	down
A_33_P3323435	MAP3K9	1.9778824	1.9679265	1.7067146	-0.009956	-0.271168	-0.140562	down
A_24_P56052	ZFP91	3.4116	3.2419114	3.3000298	-0.169689	-0.11157	-0.14063	down
A_23_P212706	ATG3	3.9042168	3.7221026	3.8050556	-0.182114	-0.099161	-0.140638	down
A_22_P00013357	lnc-RP11-166B2.1.1-1	0.697453	0.5084739	0.6051517	-0.188979	-0.092301	-0.14064	down
A_23_P53276	TIMELESS	5.506295	5.318546	5.412716	-0.187749	-0.093579	-0.140664	down
A_19_P00316324	LINC01132	-1.072378	-1.234616	-1.191482	-0.162238	-0.119103	-0.140671	down
A_33_P3399028	TLK2	0.9495711	0.7806649	0.8371053	-0.168906	-0.112466	-0.140686	down
A_33_P3312819	IGF2-AS	-2.246996	-2.516005	-2.259424	-0.269009	-0.012428	-0.140719	down
A_23_P213102	PALLD	3.5195465	3.450398	3.3072367	-0.069149	-0.21231	-0.140729	down
A_33_P3412184	ZNF865	-0.964775	-0.871049	-1.340028	0.0937262	-0.375253	-0.140763	down
A_23_P12526	TP53BP2	2.273983	2.14809	2.1182766	-0.125893	-0.155706	-0.1408	down
A_21_P0008620	SRP14-AS1	-1.597167	-1.516802	-1.959163	0.0803652	-0.361996	-0.140815	down
A_33_P3221708	XLOC_l2_012371	4.3198996	4.1719995	4.1860113	-0.1479	-0.133888	-0.140894	down
A_33_P3292297	COPS7B	-1.278625	-1.744719	-1.094389	-0.466094	0.1842361	-0.140929	down
A_24_P144620	TMEM242	-0.393364	-0.565157	-0.503539	-0.171793	-0.110174	-0.140984	down
A_33_P3252093	TMED10	5.8672495	5.5738235	5.878639	-0.293426	0.0113897	-0.141018	down
A_23_P200489	TMEM63A	0.3770547	0.1198316	0.3521662	-0.257223	-0.024889	-0.141056	down
A_22_P00009460	lnc-MAEA-1	-2.662593	-2.614913	-2.992456	0.0476804	-0.329863	-0.141091	down
A_23_P115703	PCGF6	1.4016614	1.3278089	1.193325	-0.073853	-0.208336	-0.141094	down
A_23_P99927	PTPLAD1	6.073118	5.7155185	6.148528	-0.3576	0.0754099	-0.141095	down
A_23_P434900	HN1L	5.6081343	5.241052	5.692827	-0.367082	0.084693	-0.141195	down
A_23_P110882	TSPYL4	2.872178	2.378882	3.0823078	-0.493296	0.2101297	-0.141583	down
A_23_P110276	AFAP1-AS1	3.156927	2.9476485	3.0829792	-0.209279	-0.073948	-0.141613	down
A_23_P13852	STRAP	7.041316	6.730314	7.069083	-0.311002	0.0277672	-0.141618	down
A_33_P3258274	TFPI	-2.027222	-1.919376	-2.41838	0.1078453	-0.391158	-0.141656	down
A_23_P209726	SP140	-1.868403	-2.016853	-2.003268	-0.14845	-0.134865	-0.141658	down
A_23_P205216	UTP14A	1.0472021	0.8172731	0.9933829	-0.229929	-0.053819	-0.141874	down
A_23_P58002	TCTA	0.0742002	-0.360591	0.2251091	-0.434792	0.150909	-0.141941	down
A_22_P00017320	LOC339803	-0.252378	-0.228358	-0.560504	0.0240197	-0.308126	-0.142053	down
A_33_P3209646	WDFY2	-0.182991	-0.691576	0.0413971	-0.508585	0.2243881	-0.142098	down
A_23_P412392	SEC22B	2.7138767	2.4944615	2.648984	-0.219415	-0.064893	-0.142154	down
A_23_P324523	IQCK	2.437046	2.2832599	2.3064842	-0.153786	-0.130562	-0.142174	down
A_23_P91140	PECR	0.2249317	0.1027894	0.0627155	-0.122142	-0.162216	-0.142179	down
A_23_P200222	LRP8	3.7489157	3.549811	3.6632614	-0.199105	-0.085654	-0.14238	down
A_33_P3380472	HCAR1	-0.268632	-0.545684	-0.276341	-0.277051	-0.007709	-0.14238	down
A_23_P140434	MYO5C	3.3163166	3.3433528	3.0044394	0.0270362	-0.311877	-0.142421	down
A_21_P0011391	CHEK2	0.9528184	0.7803426	0.840384	-0.172476	-0.112434	-0.142455	down
A_33_P3233565	UROS	2.5306082	2.3906322	2.385541	-0.139976	-0.145067	-0.142522	down

A_23_P27013	HOXB9	2.5757923	2.3462958	2.5201836	-0.229496	-0.055609	-0.142553	down
A_33_P3404641	PRR34-AS1	-1.193257	-1.208611	-1.4634	-0.015354	-0.270143	-0.142748	down
A_23_P63655	ATP5C1	4.777712	4.64183	4.628092	-0.135882	-0.14962	-0.142751	down
A_24_P296568	CBX1	5.433814	5.3585725	5.2234535	-0.075242	-0.210361	-0.142801	down
A_32_P122793	MORC2-AS1	0.85148	0.4931121	0.9242392	-0.358368	0.0727592	-0.142804	down
A_23_P363163	CGGBP1	-0.220301	-0.326093	-0.400152	-0.105792	-0.179851	-0.142822	down
A_23_P207614	KIAA0100	4.766144	4.414492	4.8320913	-0.351652	0.0659475	-0.142852	down
A_33_P3416037	FAM96A	5.315073	5.0669	5.2774076	-0.248173	-0.037665	-0.142919	down
A_32_P142028	HNRNPC	6.8352757	6.566748	6.8179283	-0.268528	-0.017347	-0.142937	down
A_22_P00009816	LOC101927632	-0.874845	-1.11657	-0.919115	-0.241725	-0.04427	-0.142998	down
A_24_P212129	ZNF33A	1.4212852	1.3431177	1.213397	-0.078167	-0.207888	-0.143028	down
A_33_P3337771	TGS1	2.9027739	2.7481704	2.7712383	-0.154603	-0.131536	-0.14307	down
A_23_P121141	ASTE1	-0.562104	-0.746433	-0.664033	-0.184329	-0.101929	-0.143129	down
A_33_P3676746	YLPM1	2.5053806	2.3574595	2.3669977	-0.147921	-0.138383	-0.143152	down
A_22_P00006621	RORA-AS1	-1.618213	-1.50602	-2.016733	0.1121931	-0.39852	-0.143163	down
A_21_P0014008	ZNF322	-0.688013	-0.91316	-0.749203	-0.225147	-0.06119	-0.143168	down
A_33_P3353360	BRK1	-2.056658	-2.113725	-2.285944	-0.057067	-0.229286	-0.143176	down
A_33_P3878772	JAK2	-1.526951	-1.504918	-1.8356	0.0220332	-0.308649	-0.143308	down
A_23_P28834	PHACTR3	-1.594194	-1.796928	-1.678266	-0.202734	-0.084072	-0.143403	down
A_19_P00804723	RPS24	-2.642657	-2.476524	-3.095615	0.1661329	-0.452958	-0.143413	down
A_33_P3316800	AHR	2.0906258	1.9505553	1.9438248	-0.14007	-0.146801	-0.143436	down
A_33_P3245977	SHROOM1	-0.057711	0.0308104	-0.433147	0.0885215	-0.375436	-0.143457	down
A_23_P307400	CEP95	3.4886065	3.4849572	3.205286	-0.003649	-0.28332	-0.143485	down
A_22_P00010513	lnc-NBPF6-2	-0.286291	-0.480347	-0.379309	-0.194056	-0.093018	-0.143537	down
A_21_P0014368	PLA2G4C	-1.983173	-2.452184	-1.801393	-0.469011	0.1817803	-0.143615	down
A_33_P3227467	BNIP2	4.5002375	4.4553466	4.2577963	-0.044891	-0.242441	-0.143666	down
A_24_P87036	ANO1	1.445446	1.3977723	1.2057519	-0.047674	-0.239694	-0.143684	down
A_23_P40782	COMMD2	0.4130216	0.4639144	0.0744238	0.0508928	-0.338598	-0.143852	down
A_23_P98085	PTEN	2.350624	2.2490106	2.1645308	-0.101614	-0.186093	-0.143853	down
A_32_P88719	RAB40AL	-0.890759	-1.144448	-0.924834	-0.253689	-0.034075	-0.143882	down
A_23_P326170	CALM2	7.272747	7.1167836	7.1409464	-0.155963	-0.131801	-0.143882	down
A_23_P155463	LRRC2	-2.547087	-2.668964	-2.713152	-0.121878	-0.166066	-0.143972	down
A_21_P0011898	SEPT14	0.9822745	1.1040192	0.5722652	0.1217446	-0.410009	-0.144132	down
A_24_P370096	ZNF230	-1.597381	-1.868435	-1.61485	-0.271055	-0.017469	-0.144262	down
A_21_P0011521	XLOC_l2_005692	-3.09839	-3.182542	-3.302972	-0.084152	-0.204582	-0.144367	down
A_33_P3418120	IPPK	0.7049866	0.7662277	0.3542991	0.0612412	-0.350688	-0.144723	down
A_23_P101342	ATG4D	0.2806101	-0.016492	0.2882109	-0.297102	0.0076008	-0.144751	down
A_23_P74843	IARS2	4.4645205	4.2203193	4.419183	-0.244201	-0.045338	-0.144769	down
A_22_P00017078	lnc-TUSC5-2	-1.128015	-1.227853	-1.317784	-0.099839	-0.18977	-0.144804	down
A_33_P3424057	PEG3	-2.218245	-2.471317	-2.254802	-0.253072	-0.036557	-0.144814	down
A_23_P167444	NSA2	5.861537	5.6565967	5.7766714	-0.20494	-0.084866	-0.144903	down
A_33_P3665777	HSP90AA1	8.286949	8.004637	8.279381	-0.282312	-0.007568	-0.14494	down
A_24_P47988	ELL3	0.5663724	0.2687516	0.5740547	-0.297621	0.0076823	-0.144969	down
A_23_P4294	ZNF232	1.9718342	1.7931552	1.8605709	-0.178679	-0.111263	-0.144971	down
A_23_P28507	MGAT4A	-1.608553	-1.676372	-1.830794	-0.067819	-0.22224	-0.14503	down
A_33_P3394040	ATL2	2.2871923	2.1070056	2.1772108	-0.180187	-0.109982	-0.145084	down
A_24_P373768	GIPR	0.6952496	0.3884416	0.7118149	-0.306808	0.0165653	-0.145121	down
A_24_P120346	NIT2	4.992895	4.464704	5.230835	-0.528191	0.2379398	-0.145126	down
A_23_P74042	LPHN2	1.2140322	1.1582727	0.9794536	-0.055759	-0.234579	-0.145169	down
A_23_P34548	SDCCAG8	-2.085527	-2.409346	-2.052346	-0.323819	0.0331802	-0.14532	down
A_23_P211355	DGCR8	1.9214735	1.7179155	1.8343525	-0.203558	-0.087121	-0.145339	down
A_23_P334955	FAM167A	-1.819613	-1.940754	-1.989247	-0.12114	-0.169634	-0.145387	down
A_23_P148484	RLIM	2.0775795	1.8789668	1.9854069	-0.198613	-0.092173	-0.145393	down
A_23_P152087	RMDN3	3.3946705	3.234243	3.2642832	-0.160428	-0.130387	-0.145407	down
A_19_P00811812	MPRIP	-0.791904	-1.106128	-0.768529	-0.314224	0.023375	-0.145424	down
A_32_P34116	NUS1	2.134471	1.929275	2.0486765	-0.205196	-0.085794	-0.145495	down

A_33_P3380642	FRAS1	-1.395706	-1.632013	-1.450685	-0.236307	-0.054979	-0.145643	down
A_33_P3285018	ZNF888	-2.202547	-2.186192	-2.51036	0.0163546	-0.307813	-0.145729	down
A_23_P136116	AGMO	3.5328283	3.016151	3.757844	-0.516677	0.2250156	-0.145831	down
A_33_P3230658	TSNAX	2.4966335	2.2773004	2.4239025	-0.219333	-0.072731	-0.146032	down
A_33_P3414574	DNAJA2	2.3348608	2.0778413	2.2997684	-0.25702	-0.035092	-0.146056	down
A_23_P169978	ZNF608	-0.269523	-0.361007	-0.470233	-0.091484	-0.20071	-0.146097	down
A_23_P212639	TRA2B	5.8449965	5.6630607	5.734684	-0.181936	-0.110312	-0.146124	down
A_22_P00007426	UBXN8	-2.334584	-2.611571	-2.350027	-0.276987	-0.015443	-0.146215	down
A_33_P3360823	C10orf88	1.2901716	1.0845838	1.2030897	-0.205588	-0.087082	-0.146335	down
A_24_P108291	IMPACT	-0.296154	-0.451655	-0.433328	-0.155501	-0.137175	-0.146338	down
A_24_P280378	AQR	1.0294414	0.7976451	0.9682827	-0.231796	-0.061159	-0.146477	down
A_24_P151692	POF1B	0.0297289	-0.143819	-0.089756	-0.173548	-0.119485	-0.146516	down
A_21_P0011532	XLOC_l2_005714	-0.832171	-0.940279	-1.017218	-0.108107	-0.185047	-0.146577	down
A_33_P3350643	LMX1B	-1.615188	-1.533877	-1.989829	0.0813103	-0.374641	-0.146665	down
A_21_P0000157	ZNF587B	3.5028858	3.4241452	3.2882528	-0.078741	-0.214633	-0.146687	down
A_33_P3404508	NDOR1	-1.768738	-1.981375	-1.849556	-0.212637	-0.080818	-0.146728	down
A_19_P00807328	LOC102724209	-2.220941	-2.461082	-2.274339	-0.240141	-0.053398	-0.14677	down
A_33_P3338423	SPRY4	-2.175877	-2.413581	-2.231726	-0.237704	-0.055849	-0.146777	down
A_32_P114574	CACYBP	5.4042425	5.168677	5.346199	-0.235566	-0.058043	-0.146805	down
A_24_P321511	GOLT1B	0.6808434	0.547864	0.5202045	-0.132979	-0.160639	-0.146809	down
A_23_P1691	MMP1	-0.25059	0.854074	-1.649025	1.1046643	-1.398434	-0.146885	down
A_23_P65558	MGAT2	3.3164053	3.2203736	3.118328	-0.096032	-0.198077	-0.147054	down
A_24_P123245	HNRNPD	3.0543928	2.9593005	2.8550186	-0.095092	-0.199374	-0.147233	down
A_33_P3300273	C1orf52	0.348742	0.024694	0.3781462	-0.324048	0.0294042	-0.147322	down
A_33_P3387463	ATP6V1D	3.323944	3.1577888	3.1953182	-0.166155	-0.128626	-0.147391	down
A_23_P100196	USP10	3.5101833	3.313539	3.4120426	-0.196644	-0.098141	-0.147393	down
A_33_P3677020	IL21R-AS1	-1.959767	-2.25019	-1.964208	-0.290422	-0.004441	-0.147431	down
A_32_P102062	FOXO3	-0.252785	-0.281714	-0.518788	-0.028928	-0.266003	-0.147465	down
A_21_P0000472	SNORD58C	-0.313046	-0.580432	-0.340867	-0.267385	-0.02782	-0.147603	down
A_33_P3231297	CREG1	5.6429396	5.545668	5.4449043	-0.097271	-0.198035	-0.147653	down
A_24_P228149	KRT13	1.2691135	0.9085069	1.3343143	-0.360607	0.0652008	-0.147703	down
A_33_P3341494	ZNF658	-1.838374	-2.0822	-1.889955	-0.243826	-0.051581	-0.147703	down
A_23_P52402	PITRM1	3.3473377	3.287826	3.1114016	-0.059512	-0.235936	-0.147724	down
A_33_P3274332	UBE2J1	2.8780384	2.8802495	2.5798788	0.0022111	-0.29816	-0.147974	down
A_23_P50008	TTC19	2.8406782	2.7615676	2.6238012	-0.079111	-0.216877	-0.147994	down
A_23_P252764	SMARCA2	0.0628877	-0.078535	-0.091689	-0.141422	-0.154577	-0.148	down
A_33_P3256880	LOC642980	-2.425565	-2.621767	-2.525647	-0.196202	-0.100082	-0.148142	down
A_23_P134147	ZBTB24	2.746749	2.5055814	2.6913872	-0.241168	-0.055362	-0.148265	down
A_23_P134426	GPNMB	-2.477339	-2.395661	-2.855668	0.0816777	-0.378329	-0.148326	down
A_21_P0013697	FAM27E3	1.332335	1.2845316	1.0834389	-0.047803	-0.248896	-0.14835	down
A_33_P3419190	AREG	0.2624459	0.3066587	-0.078474	0.0442128	-0.34092	-0.148354	down
A_33_P3227666	AGGF1	1.819499	1.5266633	1.8155069	-0.292836	-0.003992	-0.148414	down
A_32_P56525	TCAF1	0.0759583	-0.365601	0.2206621	-0.441559	0.1447039	-0.148428	down
A_23_P8763	PTPN12	5.1920843	5.188791	4.8980255	-0.003294	-0.294059	-0.148676	down
A_22_P00019837	lnc-GTDC1-6	-1.123469	-1.108155	-1.436171	0.0153141	-0.312702	-0.148694	down
A_23_P372834	AQP1	3.392726	3.0163903	3.4716692	-0.376336	0.0789433	-0.148696	down
A_21_P0007412	lnc-BLID-1	-1.659892	-1.797487	-1.819712	-0.137595	-0.15982	-0.148708	down
A_23_P436476	RBM34	2.9124508	2.7193274	2.8078642	-0.193123	-0.104587	-0.148855	down
A_33_P3240532	RGL1	-0.067707	-0.370191	-0.063004	-0.302484	0.0047026	-0.148891	down
A_33_P7514500	RRP7B	9.418229	9.280472	9.2580805	-0.137757	-0.160149	-0.148953	down
A_24_P22746	ZADH2	0.3157435	0.1031003	0.2303662	-0.212643	-0.085377	-0.14901	down
A_23_P7423	NSUN2	5.1038	4.940746	4.9688053	-0.163054	-0.134995	-0.149024	down
A_22_P00013693	LOC100507464	-1.79315	-1.928971	-1.9554	-0.135822	-0.162251	-0.149036	down
A_33_P3691860	FAM122B	-0.182461	-0.28755	-0.3756	-0.105089	-0.19314	-0.149114	down
A_24_P196117	GTF2H5	5.0865793	4.862203	5.012191	-0.224376	-0.074389	-0.149382	down
A_33_P3287263	COPB1	4.161153	3.9468799	4.076638	-0.214273	-0.084515	-0.149394	down

A_23_P335848	GIT2	2.163271	1.9316382	2.0959358	-0.231633	-0.067335	-0.149484	down
A_24_P225635	HNRNPLL	-1.405824	-1.43437	-1.676263	-0.028546	-0.270439	-0.149492	down
A_24_P11307	AHCYL1	2.1743755	2.0808206	1.9689102	-0.093555	-0.205465	-0.14951	down
A_23_P46131	FAM110D	-1.977943	-2.148671	-2.106265	-0.170729	-0.128322	-0.149526	down
A_23_P345942	NDUFAF2	4.663246	4.2788024	4.7484674	-0.384444	0.0852213	-0.149611	down
A_21_P0002228	lnc-CEP68-1	-2.836444	-2.876382	-3.095765	-0.039938	-0.259321	-0.14963	down
A_24_P387321	ZNF44	-0.287708	-0.222718	-0.652052	0.0649896	-0.364345	-0.149678	down
A_33_P3278856	POF1B	-1.103026	-1.242727	-1.263044	-0.139701	-0.160018	-0.14986	down
A_23_P103511	C1orf226	2.2808294	2.1289077	2.1329775	-0.151922	-0.147852	-0.149887	down
A_23_P134814	THAP1	1.920764	1.5755262	1.9660883	-0.345238	0.0453243	-0.149957	down
A_21_P0013532	XLOC_l2_014098	-1.510877	-1.867426	-1.454469	-0.356549	0.0564075	-0.150071	down
A_21_P0010642	LOC101929023	-1.761198	-2.096074	-1.726511	-0.334876	0.0346866	-0.150095	down
A_33_P3223923	PDIA3	1.4441614	1.1942406	1.3938427	-0.249921	-0.050319	-0.15012	down
A_23_P216693	MLLT3	1.2467518	1.0767193	1.1162095	-0.170033	-0.130542	-0.150287	down
A_23_P88184	BTBD7	-1.607142	-1.729227	-1.785704	-0.122086	-0.178562	-0.150324	down
A_23_P423891	SLC25A16	0.554194	0.4461684	0.3615623	-0.108026	-0.192632	-0.150329	down
A_22_P00003229	lnc-CACNG5-1	-2.099297	-2.178935	-2.320323	-0.079638	-0.221027	-0.150332	down
A_23_P8416	GALNT11	-0.962242	-1.186502	-1.03877	-0.224259	-0.076528	-0.150394	down
A_33_P3764802	SIRT5	1.7525568	1.2556357	1.9486074	-0.496921	0.1960506	-0.150435	down
A_24_P709377	PAX8-AS1	0.7800899	0.5064468	0.7528391	-0.273643	-0.027251	-0.150447	down
A_23_P203949	DDX11	2.941821	2.7761197	2.8065538	-0.165701	-0.135267	-0.150484	down
A_23_P88484	DUT	7.378745	7.19286	7.2633467	-0.185885	-0.115398	-0.150642	down
A_21_P0000491	SNAR-G1	7.638337	7.28575	7.6895065	-0.352587	0.0511694	-0.150709	down
A_24_P408740	CMC1	2.7780066	2.3743749	2.8801956	-0.403632	0.1021891	-0.150721	down
A_24_P269779	CHST3	0.4455328	0.4692226	0.1202574	0.0236897	-0.325275	-0.150793	down
A_23_P201086	ARF1	3.2012606	2.5756745	3.5252237	-0.625586	0.3239632	-0.150811	down
A_33_P3407266	GTF3C4	0.8814731	0.8213086	0.6399698	-0.060164	-0.241503	-0.150834	down
A_32_P83845	HEY1	-1.284399	-1.266895	-1.603831	0.0175042	-0.319432	-0.150964	down
A_33_P3332547	IQCJ-SCHIP1	1.3098288	1.2429757	1.0747247	-0.066853	-0.235104	-0.150979	down
A_23_P156025	IRX2	3.1576262	3.003118	3.0099926	-0.154508	-0.147634	-0.151071	down
A_23_P406702	CAMSAP2	1.3587518	1.2272	1.1880784	-0.131552	-0.170673	-0.151113	down
A_24_P356338	GABARAPL2	4.1958733	3.7289214	4.360588	-0.466952	0.1647148	-0.151119	down
A_23_P203540	EHF	2.9178076	2.62682	2.9063578	-0.290988	-0.01145	-0.151219	down
A_23_P63010	CERS2	3.8788738	3.484305	3.9707985	-0.394569	0.0919247	-0.151322	down
A_23_P337726	ATP6	8.8897705	8.895666	8.581154	0.0058956	-0.308617	-0.151361	down
A_33_P3246863	PRMT7	0.2644486	-0.018194	0.2442141	-0.282642	-0.020235	-0.151438	down
A_21_P0007097	BEND7	-0.528977	-0.657883	-0.702969	-0.128905	-0.173992	-0.151448	down
A_33_P3291097	BCAM	-1.545727	-1.357959	-2.036441	0.1877675	-0.490714	-0.151473	down
A_33_P3256033	CBL	1.0774355	0.8875022	0.9643335	-0.189933	-0.113102	-0.151518	down
A_33_P3344733	NUBPL	2.7094288	2.5315843	2.584217	-0.177845	-0.125212	-0.151528	down
A_23_P43779	COPS4	4.304928	4.1046987	4.2019806	-0.200229	-0.102947	-0.151588	down
A_22_P00010174	FGD5-AS1	-2.130594	-2.381667	-2.18288	-0.251073	-0.052286	-0.15168	down
A_23_P314760	PRKAG2	1.7114801	1.5337682	1.585557	-0.177712	-0.125923	-0.151818	down
A_32_P206293	ZNF322	1.841466	1.6669726	1.7122307	-0.174493	-0.129235	-0.151864	down
A_23_P404821	KIAA1147	0.093544	0.0412917	-0.158377	-0.052252	-0.251921	-0.152087	down
A_33_P3211263	GPATCH4	3.6574755	3.3937058	3.617053	-0.26377	-0.040422	-0.152096	down
A_33_P3285047	ZNF75A	2.099143	1.9732327	1.9207125	-0.12591	-0.178431	-0.15217	down
A_23_P361584	TMEM154	0.4848919	0.3762178	0.2889772	-0.108674	-0.195915	-0.152294	down
A_21_P0005147	lnc-FAM120B-3	-1.986308	-2.217102	-2.060243	-0.230794	-0.073936	-0.152365	down
A_24_P398940	CASC4	4.2611084	4.169474	4.047906	-0.091634	-0.213202	-0.152418	down
A_33_P3228466	FXYD3	-1.279619	-1.488349	-1.375737	-0.20873	-0.096118	-0.152424	down
A_33_P3389060	CDK5RAP2	1.2086086	1.1959748	0.9161072	-0.012634	-0.292501	-0.152568	down
A_33_P3358914	ARHGAP23	-0.989738	-1.163669	-1.121131	-0.173931	-0.131392	-0.152662	down
A_23_P251377	HMCES	2.133028	1.872642	2.088029	-0.260386	-0.044999	-0.152693	down
A_33_P3279715	UHRF2	1.6575985	1.4328451	1.5767989	-0.224753	-0.0808	-0.152776	down
A_24_P88031	C8orf33	1.4194255	1.3928399	1.1403599	-0.026586	-0.279066	-0.152826	down

A_32_P208120	CAMK1D	2.2144165	1.9578481	2.1653233	-0.256568	-0.049093	-0.152831	down
A_23_P5742	CCDC121	-1.404889	-1.64916	-1.466364	-0.244271	-0.061475	-0.152873	down
A_24_P89512	BCLAF1	4.3469152	4.082766	4.305234	-0.264149	-0.041681	-0.152915	down
A_23_P436353	MLLT4	3.8813972	3.6762795	3.7805023	-0.205118	-0.100895	-0.153006	down
A_23_P5389	DPY30	5.155469	4.866406	5.1383524	-0.289063	-0.017117	-0.15309	down
A_23_P160481	DCAF6	1.2938695	1.404973	0.8764763	0.1111035	-0.417393	-0.153145	down
A_23_P42087	BPHL	1.0182137	0.6228223	1.1070452	-0.395391	0.0888314	-0.15328	down
A_33_P3211739	CREB3L2	2.33504	2.0649762	2.298437	-0.270064	-0.036603	-0.153333	down
A_33_P3269779	ZNF3	-2.872677	-3.045057	-3.007005	-0.172381	-0.134329	-0.153355	down
A_23_P132784	FXR1	4.928547	4.860638	4.6895313	-0.067909	-0.239016	-0.153462	down
A_21_P0000210	SNORD4A	0.6365862	0.3652253	0.6009798	-0.271361	-0.035606	-0.153484	down
A_23_P157679	UTP23	3.2841702	2.9607902	3.30058	-0.32338	0.0164099	-0.153485	down
A_23_P210091	SUPT7L	4.240922	4.097655	4.0772	-0.143267	-0.163722	-0.153495	down
A_24_P305938	RAB9BP1	1.04072	0.8876195	0.8866897	-0.1531	-0.15403	-0.153565	down
A_33_P3360363	GATA4	-0.548371	-0.795778	-0.608238	-0.247407	-0.059867	-0.153637	down
A_23_P252641	GOSR1	5.772854	5.5924	5.6459694	-0.180454	-0.126884	-0.153669	down
A_33_P3273258	ZNF701	2.5673027	2.1941743	2.6328707	-0.373128	0.065568	-0.15378	down
A_33_P3237096	INPP5F	1.1505637	1.0438886	0.9494634	-0.106675	-0.2011	-0.153888	down
A_33_P3866631	DKFZP564C152	2.050539	1.5558515	2.2374287	-0.494688	0.1868897	-0.153899	down
A_22_P00009573	lnc-MAP6-3	-1.067157	-1.071372	-1.370829	-0.004215	-0.303672	-0.153944	down
A_24_P194730	ZSCAN22	-1.316963	-1.792319	-1.149596	-0.475356	0.1673675	-0.153994	down
A_23_P202602	SEC23IP	3.1443424	2.8889556	3.0917292	-0.255387	-0.052613	-0.154	down
A_33_P3407195	BOD1L1	1.52809	1.3308878	1.4172769	-0.197202	-0.110813	-0.154008	down
A_23_P253395	UNC13B	0.4254265	0.4969873	0.045754	0.0715609	-0.379673	-0.154056	down
A_33_P3321577	BAZ1B	3.8634872	3.5462775	3.8723736	-0.31721	0.0088863	-0.154162	down
A_22_P00003844	LINC01273	-2.594484	-2.606045	-2.891249	-0.011561	-0.296766	-0.154163	down
A_23_P94636	RC3H2	0.7430873	0.65098	0.5268211	-0.092107	-0.216266	-0.154187	down
A_21_P0014389	ACTR3	5.595933	5.3926444	5.490777	-0.203289	-0.105156	-0.154222	down
A_32_P182394	ZNF77	-2.213151	-2.126843	-2.607904	0.0863078	-0.394753	-0.154223	down
A_19_P00316533	BLCAP	-2.294547	-2.302658	-2.594926	-0.008111	-0.300379	-0.154245	down
A_32_P22338	PDCD6IP	0.5423842	0.2692404	0.5070167	-0.273144	-0.035367	-0.154256	down
A_21_P0011501	LOC101926936	-0.883586	-0.916965	-1.158856	-0.033379	-0.27527	-0.154325	down
A_23_P320887	CDC42SE1	1.6263638	1.4312692	1.5127273	-0.195095	-0.113636	-0.154366	down
A_23_P257003	PCSK5	0.4375205	0.4657269	0.1005745	0.0282063	-0.336946	-0.15437	down
A_24_P769359	WNK1	2.4472046	2.3280997	2.2575674	-0.119105	-0.189637	-0.154371	down
A_23_P321377	C1orf216	-0.503337	-0.64392	-0.671681	-0.140583	-0.168344	-0.154464	down
A_33_P3376239	KMT2E	2.0571346	2.0542474	1.7508512	-0.002887	-0.306283	-0.154585	down
A_23_P99771	PNMA1	4.434496	4.2149158	4.344639	-0.21958	-0.089857	-0.154719	down
A_24_P26897	INPP5A	3.0326014	3.0370307	2.7186651	0.0044293	-0.313936	-0.154753	down
A_23_P56654	MCEE	2.6245146	2.5840259	2.3554335	-0.040489	-0.269081	-0.154785	down
A_24_P943193	PREPL	2.619998	2.7915826	2.1388092	0.1715846	-0.481189	-0.154802	down
A_33_P3319765	FANCC	-0.089818	-0.55327	0.0639725	-0.463453	0.15379	-0.154831	down
A_22_P00007025	lnc-GIP-1	-1.504817	-1.729739	-1.589664	-0.224923	-0.084847	-0.154885	down
A_32_P16007	POTEB3	1.8922901	1.7694392	1.7051682	-0.122851	-0.187122	-0.154986	down
A_33_P3287840	STAM	-0.784323	-0.99442	-0.884359	-0.210097	-0.100037	-0.155067	down
A_33_P3356341	MSL1	1.2473459	1.1271076	1.0571661	-0.120238	-0.19018	-0.155209	down
A_23_P352684	DCAF5	0.4978018	0.6762896	0.0088711	0.1784878	-0.488931	-0.155221	down
A_33_P3342957	STK17B	1.7276878	1.5258694	1.6190209	-0.201818	-0.108667	-0.155243	down
A_21_P0000112	ZNF726	-1.785379	-1.833603	-2.047653	-0.048224	-0.262274	-0.155249	down
A_23_P154457	NOL10	-1.59415	-1.899565	-1.599423	-0.305415	-0.005273	-0.155344	down
A_23_P47991	MED13L	2.4148464	2.2397437	2.2792263	-0.175103	-0.13562	-0.155361	down
A_23_P77776	SRSF2	6.2894554	6.0801196	6.1879683	-0.209336	-0.101487	-0.155411	down
A_23_P131866	AURKA	3.4538498	3.2166753	3.3801908	-0.237175	-0.073659	-0.155417	down
A_22_P00002645	lnc-C1orf192-1	-1.492953	-1.624572	-1.672207	-0.131619	-0.179254	-0.155437	down
A_23_P88731	RAD51	4.820941	4.575351	4.75554	-0.24559	-0.065401	-0.155495	down
A_24_P137376	ATP2C1	1.7621441	1.620481	1.5924158	-0.141663	-0.169728	-0.155696	down

A_23_P77980	SLC4A1	-1.831346	-2.087386	-1.886846	-0.25604	-0.0555	-0.15577	down
A_23_P127557	KBTBD3	-2.379855	-2.352995	-2.718315	0.0268598	-0.33846	-0.1558	down
A_22_P00007503	lnc-GYG2-1	-1.160478	-1.177942	-1.454711	-0.017465	-0.294234	-0.155849	down
A_23_P126075	KCNK1	4.5643616	4.4085975	4.4084053	-0.155764	-0.155956	-0.15586	down
A_22_P00001137	HNRNPKP3	3.6343088	3.424934	3.5319166	-0.209375	-0.102392	-0.155884	down
A_23_P80032	E2F1	3.075098	2.8980203	2.9403858	-0.177078	-0.134712	-0.155895	down
A_22_P00017897	TRHDE-AS1	-0.940948	-1.317902	-0.876064	-0.376955	0.0648832	-0.156036	down
A_32_P70135	PROSER1	3.9265747	3.6907039	3.8503695	-0.235871	-0.076205	-0.156038	down
A_24_P330303	FRMD6	0.2546535	0.0653653	0.1317692	-0.189288	-0.122884	-0.156086	down
A_23_P212728	TBC1D23	2.214281	2.1174474	1.9989176	-0.096834	-0.215364	-0.156099	down
A_23_P146765	RNF139	4.4942913	4.4599357	4.216284	-0.034356	-0.278008	-0.156182	down
A_33_P3389018	OR4F15	-2.655775	-2.975401	-2.648539	-0.319626	0.0072362	-0.156195	down
A_24_P305623	TMEM50B	3.2049227	2.7760882	3.321086	-0.428834	0.1161633	-0.156336	down
A_21_P0010250	LINC00112	-2.139361	-1.878785	-2.712681	0.2605767	-0.573319	-0.156371	down
A_23_P203305	IMMP1L	1.1370864	0.911921	1.0494771	-0.225165	-0.087609	-0.156387	down
A_24_P287473	SAV1	3.046651	2.8736014	2.9068813	-0.173049	-0.13977	-0.15641	down
A_32_P179837	RNF151	-1.345639	-1.548107	-1.456087	-0.202468	-0.110448	-0.156458	down
A_33_P3281930	LRRFIP2	1.8308029	1.7709537	1.5776644	-0.059849	-0.253139	-0.156494	down
A_33_P3248539	LCN1	-2.121077	-2.339666	-2.215488	-0.218589	-0.094411	-0.1565	down
A_33_P3334092	lnc-SHISA8-1	-1.417694	-1.612599	-1.535887	-0.194905	-0.118194	-0.156549	down
A_33_P3422170	HNRNPA3	2.4893227	2.2472224	2.4182663	-0.2421	-0.071056	-0.156578	down
A_23_P128574	ENOX1	-1.321649	-1.842397	-1.114308	-0.520748	0.2073407	-0.156704	down
A_33_P3245183	HRH1	0.0264001	-0.299728	0.0391126	-0.326128	0.0127125	-0.156708	down
A_24_P247106	AK3	1.577178	1.4638109	1.3768411	-0.113367	-0.200337	-0.156852	down
A_33_P3302736	ZSCAN30	-2.393904	-2.331455	-2.770128	0.0624492	-0.376224	-0.156888	down
A_24_P186124	MTERF4	2.5825624	2.5380893	2.3132124	-0.044473	-0.26935	-0.156912	down
A_33_P3298024	ABCC3	2.5274334	2.1449227	2.5960407	-0.382511	0.0686073	-0.156952	down
A_32_P16315	TUBB8	-0.282534	-0.626936	-0.252138	-0.344402	0.030396	-0.157003	down
A_22_P00000873	lnc-AGRP-5	-1.462651	-1.665597	-1.573736	-0.202946	-0.111085	-0.157016	down
A_23_P372925	CIPC	3.1572971	3.0104632	2.9898834	-0.146834	-0.167414	-0.157124	down
A_21_P0001291	lnc-CYP4A22-2	-1.850144	-1.939422	-2.075171	-0.089278	-0.225027	-0.157152	down
A_22_P00007343	NEURL4	-1.524889	-1.697973	-1.666287	-0.173084	-0.141398	-0.157241	down
A_24_P98263	EBAG9	2.6570435	2.170196	2.829401	-0.486847	0.1723576	-0.157245	down
A_21_P0014944	LOC102724156	0.0459156	-0.30055	0.0778766	-0.346466	0.031961	-0.157253	down
A_23_P134078	CDYL	0.874826	0.6537032	0.7812038	-0.221123	-0.093622	-0.157372	down
A_24_P51588	CCNL2	2.0021715	1.9401145	1.7494512	-0.062057	-0.25272	-0.157389	down
A_23_P46470	ERRFI1	2.368719	2.4782538	1.9441662	0.1095347	-0.424553	-0.157509	down
A_33_P3319261	EXD3	-1.546039	-1.397779	-2.009334	0.1482606	-0.463295	-0.157517	down
A_33_P3213029	RNF43	6.8818407	6.592543	6.8560286	-0.289298	-0.025812	-0.157555	down
A_23_P58489	FASTKD3	1.467668	1.2062521	1.4139376	-0.261416	-0.05373	-0.157573	down
A_23_P368558	TMEM131	1.4154735	1.2266245	1.2890496	-0.188849	-0.126424	-0.157636	down
A_33_P3416301	E2F6	-0.286928	-0.470502	-0.418748	-0.183574	-0.13182	-0.157697	down
A_24_P49183	EXD3	-2.001216	-1.944018	-2.373848	0.0571971	-0.372633	-0.157718	down
A_22_P00012464	lnc-PRR15L-2	0.0090394	-0.157823	-0.139715	-0.166862	-0.148755	-0.157809	down
A_23_P56833	ASB3	1.7017164	1.6290784	1.4584556	-0.072638	-0.243261	-0.157949	down
A_24_P169574	YWHAEP1	1.0885825	0.5431962	1.3180442	-0.545386	0.2294617	-0.157962	down
A_23_P420334	PGBD4	-0.192598	-0.327843	-0.373279	-0.135245	-0.180681	-0.157963	down
A_21_P0013831	XLOC_l2_015821	0.1970844	0.1421924	-0.064024	-0.054892	-0.261108	-0.158	down
A_23_P138680	IL15RA	0.9096465	0.6278834	0.875392	-0.281763	-0.034255	-0.158009	down
A_21_P0013643	AQP7P3	0.2607875	0.3948689	-0.189469	0.1340814	-0.450256	-0.158087	down
A_33_P3333982	PDE4DIP	-2.315664	-2.593306	-2.354342	-0.277642	-0.038678	-0.15816	down
A_23_P81825	GUCA1B	-2.088941	-2.384112	-2.110109	-0.295171	-0.021168	-0.15817	down
A_23_P44139	PRIM2	2.4663258	2.2160554	2.4002008	-0.25027	-0.066125	-0.158198	down
A_24_P122682	RAB4A	2.5951319	2.2524428	2.6213722	-0.342689	0.0262403	-0.158224	down
A_21_P0001765	LINC00954	-2.794658	-2.745293	-3.16065	0.0493658	-0.365992	-0.158313	down
A_24_P363087	C5orf45	2.3780985	2.0813222	2.3581047	-0.296776	-0.019994	-0.158385	down

A_23_P138168	CNN3	2.952324	2.7567048	2.8311024	-0.195619	-0.121222	-0.15842	down
A_33_P3234015	LIMD1	2.4284554	2.1148229	2.4252415	-0.313633	-0.003214	-0.158423	down
A_23_P213602	CSNK1G3	0.5651727	0.4349999	0.3784857	-0.130173	-0.186687	-0.15843	down
A_23_P309779	N4BP2	-0.430182	-0.430699	-0.746679	-0.000516	-0.316496	-0.158506	down
A_24_P339611	PDCD5	6.5042696	6.189611	6.5018225	-0.314659	-0.002447	-0.158553	down
A_24_P91405	ZNF709	1.581398	1.5171871	1.3284845	-0.064211	-0.252913	-0.158562	down
A_23_P104876	SPA17	2.600481	2.3052087	2.5786247	-0.295272	-0.021856	-0.158564	down
A_33_P3332438	GINM1	1.9212427	1.9185042	1.6066189	-0.002738	-0.314624	-0.158681	down
A_23_P105730	MDM1	-2.267766	-2.276322	-2.576585	-0.008556	-0.308819	-0.158688	down
A_33_P3247644	MTURN	-0.775452	-0.579942	-1.288391	0.1955094	-0.512939	-0.158715	down
A_21_P0010503	HOTAIR	-0.981943	-1.127542	-1.154008	-0.145598	-0.172065	-0.158832	down
A_33_P3386508	NDUFA6-AS1	-2.151562	-2.322846	-2.297972	-0.171284	-0.14641	-0.158847	down
A_33_P3228931	CASS4	-1.756234	-2.081929	-1.748396	-0.325695	0.0078378	-0.158929	down
A_33_P3217983	ACSL5	3.4973917	3.298263	3.378518	-0.199129	-0.118874	-0.159001	down
A_23_P211212	COL18A1	4.27125	3.9478621	4.2762365	-0.323388	0.0049868	-0.1592	down
A_23_P7791	OGFRL1	1.4512892	1.1170268	1.467134	-0.334262	0.0158448	-0.159209	down
A_23_P153098	HDHD2	3.26515	3.1088347	3.1027966	-0.156315	-0.162354	-0.159334	down
A_24_P339944	PDGFB	-0.834844	-1.020688	-0.967754	-0.185844	-0.13291	-0.159377	down
A_23_P500130	KANK1	3.4053411	2.978561	3.5133448	-0.42678	0.1080036	-0.159388	down
A_19_P00324814	XLOC_l2_015561	-1.129892	-1.122098	-1.456526	0.0077944	-0.326634	-0.15942	down
A_33_P3260322	NR6A1	2.4376755	2.3649049	2.1915617	-0.072771	-0.246114	-0.159442	down
A_33_P3251985	MFSD1	2.6443834	2.4989	2.470973	-0.145484	-0.17341	-0.159447	down
A_24_P301629	ZNF155	-0.61665	-0.778559	-0.773696	-0.161909	-0.157046	-0.159477	down
A_24_P54131	DCLRE1B	0.265552	0.0375175	0.174613	-0.228035	-0.090939	-0.159487	down
A_23_P143662	PISD	0.9312596	0.9412007	0.6020742	0.0099411	-0.329186	-0.159622	down
A_23_P11774	UTP11L	4.345379	4.0411744	4.3303366	-0.304204	-0.015042	-0.159623	down
A_23_P101972	CAPN13	0.1043778	-0.354548	0.2439246	-0.458925	0.1395469	-0.159689	down
A_23_P162525	UTP20	3.664218	3.3297572	3.6792269	-0.334461	0.0150089	-0.159726	down
A_33_P3233978	FBXO18	-0.869403	-1.248081	-0.810204	-0.378678	0.0591989	-0.15974	down
A_33_P3290567	WEE1	1.8688669	2.065292	1.3529086	0.196425	-0.515958	-0.159767	down
A_33_P3358648	LRRFIP1	4.256131	4.194431	3.9979239	-0.0617	-0.258207	-0.159954	down
A_22_P00025517	TLX1NB	-2.22364	-2.176038	-2.59132	0.0476019	-0.36768	-0.160039	down
A_23_P37654	MGA	2.99831	2.8696418	2.8068218	-0.128668	-0.191488	-0.160078	down
A_33_P3297517	TRA2B	2.8352442	2.7089543	2.6412706	-0.12629	-0.193974	-0.160132	down
A_24_P252497	TRIB1	6.3656187	6.2405677	6.1703205	-0.125051	-0.195298	-0.160175	down
A_33_P3274618	LINC01347	-0.759532	-0.699646	-1.139787	0.0598869	-0.380254	-0.160184	down
A_23_P108554	DDX1	6.2658577	6.0502715	6.160905	-0.215586	-0.104953	-0.16027	down
A_23_P30630	MCM9	-2.066066	-2.258651	-2.194097	-0.192585	-0.128031	-0.160308	down
A_24_P252973	CLP1	1.078548	0.9597044	0.8767414	-0.118844	-0.201807	-0.160325	down
A_24_P186943	ELN	-1.915892	-2.047075	-2.105416	-0.131183	-0.189524	-0.160354	down
A_22_P00002044	SCAMP1	0.4288106	0.2475328	0.2892551	-0.181278	-0.139555	-0.160417	down
A_33_P3213362	CASC2	-2.054583	-2.328216	-2.101833	-0.273633	-0.04725	-0.160442	down
A_22_P00015918	lnc-TCIRG1-1	-1.050202	-1.384497	-1.036831	-0.334295	0.013371	-0.160462	down
A_23_P344853	WDR43	3.3156443	3.0332942	3.2767248	-0.28235	-0.038919	-0.160635	down
A_23_P140738	PIEZO1	1.7255316	1.6731787	1.4566016	-0.052353	-0.26893	-0.160641	down
A_33_P3252146	HMX3	-1.98469	-1.816849	-2.474203	0.167841	-0.489513	-0.160836	down
A_23_P141035	CHST4	0.1976085	-0.096938	0.1703897	-0.294546	-0.027219	-0.160882	down
A_21_P0000389	SNORD91B	-1.274869	-1.606288	-1.265253	-0.331419	0.0096154	-0.160902	down
A_23_P134925	BNIP3L	1.7344418	2.0486817	1.0983577	0.31424	-0.636084	-0.160922	down
A_23_P23983	FAM188A	1.6116934	1.3915687	1.5099177	-0.220125	-0.101776	-0.16095	down
A_21_P0012935	XLOC_l2_012323	-1.484094	-1.7477	-1.542508	-0.263606	-0.058414	-0.16101	down
A_23_P97283	PAQR6	1.0842767	0.9900899	0.8564014	-0.094187	-0.227875	-0.161031	down
A_22_P00001508	LCMT1-AS1	-2.407731	-2.374167	-2.763535	0.0335631	-0.355804	-0.161121	down
A_23_P92281	GTPBP8	4.806408	4.6850057	4.6052055	-0.121402	-0.201202	-0.161302	down
A_23_P67785	SPAG16	2.1208725	2.0572133	1.8617535	-0.063659	-0.259119	-0.161389	down
A_23_P2745	GJB6	-0.214485	-0.356401	-0.395355	-0.141917	-0.18087	-0.161393	down

A_23_P216812	CDKN2B	-0.911853	-0.978611	-1.168024	-0.066758	-0.25617	-0.161464	down
A_19_P00810899	SNHG20	1.3742609	1.1235757	1.3018785	-0.250685	-0.072382	-0.161534	down
A_32_P128310	CDKAL1	-2.19178	-2.252935	-2.453709	-0.061155	-0.26193	-0.161543	down
A_21_P0009620	C18orf61	-2.185643	-2.586129	-2.108283	-0.400486	0.0773597	-0.161563	down
A_33_P3549091	LOC100131347	-0.503894	-0.669381	-0.66165	-0.165486	-0.157756	-0.161621	down
A_32_P131031	MACC1	0.977026	0.7287602	0.9019742	-0.248266	-0.075052	-0.161659	down
A_33_P3393650	PLEKHA5	-1.29053	-1.436676	-1.467748	-0.146146	-0.177218	-0.161682	down
A_23_P121806	ENOPH1	3.4890041	3.2246337	3.430008	-0.26437	-0.058996	-0.161683	down
A_33_P3242919	HNRNPM	5.249881	4.759481	5.4168396	-0.4904	0.1669588	-0.161721	down
A_21_P0003311	lnc-LSG1-1	-2.839007	-3.062049	-2.939412	-0.223042	-0.100405	-0.161723	down
A_23_P205713	STXBP6	0.294888	-0.120637	0.3869448	-0.415525	0.0920568	-0.161734	down
A_23_P80122	WRB	0.9231429	0.869071	0.6534057	-0.054072	-0.269737	-0.161905	down
A_23_P145463	SLC35B3	-1.387581	-1.668135	-1.430945	-0.280554	-0.043364	-0.161959	down
A_33_P3215123	RAP1GDS1	2.739604	2.671986	2.483119	-0.067618	-0.256485	-0.162051	down
A_23_P342053	RBBP6	-0.141357	-0.230605	-0.376335	-0.089248	-0.234978	-0.162113	down
A_21_P0005590	lnc-WDR60-1	-2.793436	-2.933339	-2.977803	-0.139903	-0.184367	-0.162135	down
A_23_P144639	TMCO6	1.1194854	0.8855853	1.0290122	-0.2339	-0.090473	-0.162187	down
A_23_P216501	TPM2	6.4015007	6.1843534	6.294112	-0.217147	-0.107389	-0.162268	down
A_24_P658427	NFIB	2.4448147	2.2798457	2.284874	-0.164969	-0.159941	-0.162455	down
A_23_P206018	TPM1	2.591465	2.4479327	2.409998	-0.143532	-0.181467	-0.1625	down
A_24_P121956	TMEM86B	0.5532556	0.426877	0.3546214	-0.126379	-0.198634	-0.162506	down
A_23_P23114	PTP4A2	2.6689014	2.3993516	2.6133547	-0.26955	-0.055547	-0.162548	down
A_24_P941322	QKI	-0.381434	-0.635276	-0.452988	-0.253842	-0.071554	-0.162698	down
A_33_P3255209	MGAT5	7.431753	7.2211175	7.3169613	-0.210636	-0.114792	-0.162714	down
A_33_P3318596	ZNF345	-2.994631	-3.033378	-3.281328	-0.038748	-0.286697	-0.162722	down
A_33_P3372297	ABR	-1.906417	-2.015931	-2.122817	-0.109513	-0.216399	-0.162956	down
A_23_P94552	TMEM2	4.902529	4.654451	4.8246746	-0.248078	-0.077854	-0.162966	down
A_24_P300777	ADAM8	2.4189425	2.2500901	2.2616825	-0.168852	-0.15726	-0.163056	down
A_23_P69908	GLRX	2.6763296	2.5841804	2.441903	-0.092149	-0.234427	-0.163288	down
A_22_P00013027	GACAT1	-2.430395	-2.736548	-2.450877	-0.306153	-0.020482	-0.163317	down
A_33_P3292919	TCOF1	4.962021	4.2947793	5.3026075	-0.667242	0.3405867	-0.163327	down
A_32_P151366	SIRT5	0.9257989	0.5742955	0.9505415	-0.351503	0.0247426	-0.16338	down
A_21_P0012119	XLOC_l2_008622	-2.924901	-3.049709	-3.126886	-0.124808	-0.201985	-0.163397	down
A_32_P25253	ISCA1	2.5588226	2.3421922	2.448596	-0.21663	-0.110227	-0.163429	down
A_22_P00005092	lnc-DGCR6-2	-2.328979	-2.555854	-2.42896	-0.226876	-0.099981	-0.163429	down
A_33_P3332860	STX16	0.339005	0.2741118	0.0769177	-0.064893	-0.262087	-0.16349	down
A_21_P0005793	lnc-ANGPT2-2	-0.289378	-0.661615	-0.244131	-0.372237	0.0452476	-0.163495	down
A_33_P3315600	YY1	4.921153	4.601434	4.9138117	-0.319719	-0.007341	-0.16353	down
A_21_P0000080	FAM122C	-0.771269	-1.020719	-0.848928	-0.24945	-0.077659	-0.163554	down
A_22_P00014180	PDE10A	-1.965059	-2.078019	-2.179237	-0.11296	-0.214179	-0.163569	down
A_24_P162287	CEP250	-1.691011	-1.927961	-1.781204	-0.23695	-0.090192	-0.163571	down
A_21_P0009242	lnc-MRM1-2	-2.338963	-2.345413	-2.659813	-0.00645	-0.320851	-0.163651	down
A_24_P326739	GLS2	-0.821304	-1.473823	-0.496153	-0.652519	0.325151	-0.163684	down
A_24_P208825	MUC4	-0.4238	-0.205544	-0.969472	0.2182555	-0.545672	-0.163708	down
A_33_P3363655	ANAPC1	-0.559741	-0.830773	-0.6162	-0.271033	-0.056459	-0.163746	down
A_24_P48862	SNAP29	-0.817451	-0.816243	-1.146239	0.0012083	-0.328788	-0.16379	down
A_24_P64039	AP3M2	2.7617292	2.4564748	2.7393093	-0.305254	-0.02242	-0.163837	down
A_33_P3301559	CLUAP1	1.0355949	1.007319	0.7361913	-0.028276	-0.299404	-0.16384	down
A_23_P102706	SNPH	-1.624322	-1.956872	-1.619518	-0.33255	0.0048032	-0.163873	down
A_23_P200126	MKNK1	1.0929518	1.0539813	0.8040175	-0.03897	-0.288934	-0.163952	down
A_24_P344961	AMOT	2.3294983	2.1403575	2.1906786	-0.189141	-0.13882	-0.16398	down
A_24_P281988	PLCL2	-2.534256	-2.935201	-2.461352	-0.400945	0.0729034	-0.164021	down
A_33_P3413541	RNF138P1	-0.571086	-0.613442	-0.85679	-0.042356	-0.285703	-0.16403	down
A_33_P3303572	EDA	-0.730459	-0.845761	-0.943493	-0.115302	-0.213034	-0.164168	down
A_21_P0011384	UBE2Q2P1	-0.074946	-0.079525	-0.39878	-0.00458	-0.323834	-0.164207	down
A_21_P0000288	SNORD45B	-0.645065	-0.7717	-0.847114	-0.126635	-0.202048	-0.164342	down

A_24_P8088	RIOK1	1.0100017	0.7539959	0.9370565	-0.256006	-0.072945	-0.164475	down
A_23_P131653	PIGF	0.4188004	0.1215491	0.3870053	-0.297251	-0.031795	-0.164523	down
A_21_P0000051	DISC1	-2.89958	-3.125077	-3.003184	-0.225497	-0.103604	-0.16455	down
A_22_P00008742	TRAF3IP2-AS1	-1.107812	-1.392995	-1.151786	-0.285183	-0.043974	-0.164578	down
A_22_P00013240	LOC100507283	-3.07035	-3.129125	-3.340793	-0.058775	-0.270443	-0.164609	down
A_33_P3403867	PMEPA1	4.6832037	4.4938245	4.543193	-0.189379	-0.140011	-0.164695	down
A_23_P123672	TDRD7	3.1913462	2.8365502	3.2167435	-0.354796	0.0253973	-0.164699	down
A_23_P200792	NOTCH2	4.8287582	4.7656894	4.5623283	-0.063069	-0.26643	-0.164749	down
A_24_P112750	TFCP2	0.172606	-0.061355	0.0770574	-0.233961	-0.095549	-0.164755	down
A_33_P3279379	DSTNP2	-1.240929	-1.318238	-1.493156	-0.077309	-0.252227	-0.164768	down
A_23_P159382	SEC24D	1.7619247	1.6286035	1.5655246	-0.133321	-0.1964	-0.164861	down
A_23_P36305	ATG16L2	0.5918899	0.472784	0.3812513	-0.119106	-0.210639	-0.164872	down
A_33_P3357082	METTL8	1.0405354	0.8075991	0.9436684	-0.232936	-0.096867	-0.164902	down
A_33_P6822021	NFATC3	2.3466501	1.8275371	2.5359392	-0.519113	0.1892891	-0.164912	down
A_23_P129144	MYZAP	-0.215132	-0.328779	-0.431324	-0.113647	-0.216192	-0.16492	down
A_23_P500985	PAX7	-2.500532	-2.717344	-2.613561	-0.216812	-0.113029	-0.16492	down
A_32_P51894	HNRNPU	2.6015997	2.4825568	2.3907442	-0.119043	-0.210855	-0.164949	down
A_24_P88763	LOXL3	1.6166029	1.2007513	1.7023954	-0.415852	0.0857925	-0.16503	down
A_24_P364042	NPY4R	-2.021704	-2.10599	-2.267624	-0.084286	-0.24592	-0.165103	down
A_23_P112596	ADK	3.7665014	3.6649995	3.5377893	-0.101502	-0.228712	-0.165107	down
A_24_P130041	CYP51A1	5.1601057	4.7352986	5.2544775	-0.424807	0.0943718	-0.165218	down
A_24_P382119	MTMR4	3.8530521	3.5011573	3.8745089	-0.351895	0.0214567	-0.165219	down
A_21_P0011937	XLOC_l2_008140	-1.578728	-1.813931	-1.674007	-0.235203	-0.095279	-0.165241	down
A_24_P193582	DEF8	4.3059635	4.011122	4.2701693	-0.294841	-0.035794	-0.165318	down
A_32_P3556	VPS35	3.946351	3.6853452	3.876628	-0.261006	-0.069723	-0.165365	down
A_24_P204214	NCOR1	2.6354542	2.453402	2.486765	-0.182052	-0.148689	-0.165371	down
A_33_P3622802	ABI1	1.2094374	1.1302953	0.9577069	-0.079142	-0.25173	-0.165436	down
A_33_P3298430	FAM171A1	-0.852876	-0.900862	-1.13598	-0.047986	-0.283104	-0.165545	down
A_23_P208210	ZNF432	-0.426044	-0.401299	-0.781895	0.0247445	-0.355851	-0.165553	down
A_22_P00004696	RECQL4	-1.038543	-1.235674	-1.172707	-0.197132	-0.134164	-0.165648	down
A_23_P85543	RNF2	-0.021182	-0.230945	-0.14276	-0.209763	-0.121578	-0.16567	down
A_33_P3296352	PPM1B	1.5406499	1.4889469	1.2604384	-0.051703	-0.280211	-0.165957	down
A_24_P336983	CAPN13	-0.866702	-1.275257	-0.790087	-0.408555	0.0766149	-0.16597	down
A_33_P3231447	ITGA6	3.0502377	2.845408	2.9230728	-0.20483	-0.127165	-0.165997	down
A_33_P3328772	PLEKHA5	-2.433568	-2.461948	-2.737192	-0.02838	-0.303624	-0.166002	down
A_21_P0012944	SMA4	-0.905654	-0.940507	-1.202882	-0.034853	-0.297228	-0.166041	down
A_33_P3327245	SETDB2	0.1188445	-0.020133	-0.074459	-0.138978	-0.193303	-0.16614	down
A_32_P407245	DNAJC22	1.008595	0.7243242	0.9605355	-0.284271	-0.048059	-0.166165	down
A_32_P83465	NBPF10	6.389289	6.261411	6.184699	-0.127878	-0.20459	-0.166234	down
A_24_P148094	LEPROT	2.8497438	2.7092261	2.65761	-0.140518	-0.192134	-0.166326	down
A_23_P401380	DIP2B	1.4798808	1.3073463	1.3196244	-0.172534	-0.160256	-0.166395	down
A_23_P147296	NFU1	2.5776844	2.5677447	2.2546635	-0.00994	-0.323021	-0.16648	down
A_23_P217845	RGS16	0.1689591	-0.369339	0.3742862	-0.538299	0.205327	-0.166486	down
A_22_P00012190	lnc-POU4F2-2	-2.920317	-2.908456	-3.265267	0.0118608	-0.34495	-0.166545	down
A_33_P3349259	TCAF1	-1.192099	-1.535311	-1.182008	-0.343212	0.0100913	-0.16656	down
A_23_P67367	DHDH	1.9100437	1.6568499	1.8300595	-0.253194	-0.079984	-0.166589	down
A_23_P4082	CCT6B	-2.127018	-2.35262	-2.234666	-0.225602	-0.107648	-0.166625	down
A_23_P402287	LNX2	2.5980225	2.2505937	2.6120758	-0.347429	0.0140533	-0.166688	down
A_21_P0013035	LSM14A	0.3943424	0.2496638	0.2055874	-0.144679	-0.188755	-0.166717	down
A_33_P3375665	FOXO3	4.1192226	4.216581	3.688385	0.0973582	-0.430838	-0.16674	down
A_23_P165130	MPV17L2	3.8816996	3.4983802	3.9315329	-0.383319	0.0498333	-0.166743	down
A_33_P3214879	KPNA1	0.004746	-0.413091	0.0886283	-0.417837	0.0838823	-0.166977	down
A_24_P337746	RABGEF1	2.9477463	2.7765489	2.784854	-0.171197	-0.162892	-0.167045	down
A_19_P00316340	LOC100506860	-0.638094	-0.671918	-0.938696	-0.033824	-0.300602	-0.167213	down
A_33_P3377564	PRICKLE3	-1.372312	-1.311277	-1.767907	0.0610342	-0.395595	-0.16728	down
A_23_P251051	NF2	6.2992964	6.01841	6.2455826	-0.280886	-0.053714	-0.1673	down

A_24_P101402	NOP56	7.1106405	6.7413077	7.1453714	-0.369333	0.0347309	-0.167301	down
A_22_P00005923	HOXA11-AS	-1.114862	-1.354933	-1.209773	-0.240071	-0.094911	-0.167491	down
A_19_P00321473	HCG18	0.9033113	0.594008	0.8775959	-0.309303	-0.025715	-0.167509	down
A_23_P73493	CETN2	3.348875	3.1045146	3.2580118	-0.24436	-0.090863	-0.167612	down
A_24_P759477	ITGB8	2.293973	2.1440701	2.108614	-0.149903	-0.185359	-0.167631	down
A_23_P210708	SIRPA	-0.385213	-0.830706	-0.275018	-0.445493	0.1101952	-0.167649	down
A_33_P3237874	TROAP	1.4988542	1.2118387	1.4502902	-0.287015	-0.048564	-0.16779	down
A_21_P0006548	lnc-FAM70A-1	2.8413744	2.6932874	2.6537828	-0.148087	-0.187592	-0.167839	down
A_24_P634768	FLJ22763	-1.538432	-2.308092	-1.104623	-0.76966	0.4338088	-0.167925	down
A_33_P3366146	SRSF5	4.85575	4.755979	4.6192045	-0.099771	-0.236546	-0.168158	down
A_24_P125839	C21orf91	-0.4071	-0.497451	-0.653151	-0.090352	-0.246051	-0.168201	down
A_33_P3390335	RSU1	2.2579384	2.0882688	2.091198	-0.16967	-0.16674	-0.168205	down
A_24_P274640	GTF2H2C_2	3.6113052	3.459148	3.4270105	-0.152157	-0.184295	-0.168226	down
A_23_P170733	ANTXR2	1.1366806	0.6223569	1.3144822	-0.514324	0.1778016	-0.168261	down
A_33_P3422499	FAAHP1	-2.804464	-2.971779	-2.973913	-0.167315	-0.16945	-0.168382	down
A_23_P381102	CCDC74B	0.5449896	0.0055985	0.747427	-0.539391	0.2024374	-0.168477	down
A_24_P349002	POM121	2.6401024	2.1795077	2.763503	-0.460595	0.1234007	-0.168597	down
A_33_P3268334	PGAM1	3.6275263	3.2077394	3.710044	-0.419787	0.0825176	-0.168635	down
A_19_P00803360	LOC730101	2.657094	2.3187242	2.6581345	-0.33837	0.0010405	-0.168665	down
A_23_P10291	CTSE	-0.942618	-1.192209	-1.030643	-0.249591	-0.088025	-0.168808	down
A_23_P201445	DESI2	3.7562742	3.6244626	3.550456	-0.131812	-0.205818	-0.168815	down
A_23_P14482	C14orf166	6.9029226	6.721709	6.746476	-0.181214	-0.156446	-0.16883	down
A_33_P3405728	PKP2	4.3050804	4.000896	4.271409	-0.304184	-0.033671	-0.168928	down
A_23_P213247	FBXL5	1.9961672	1.9581866	1.6962705	-0.037981	-0.299897	-0.168939	down
A_23_P259344	CECR6	-1.761989	-2.235988	-1.62602	-0.473999	0.1359692	-0.169015	down
A_24_P1919	NF1	-0.645608	-0.701678	-0.928002	-0.05607	-0.282394	-0.169232	down
A_23_P167559	RNF145	3.5769148	3.137957	3.6773205	-0.438958	0.1004057	-0.169276	down
A_24_P74064	ZBTB14	0.7268868	0.4270153	0.6881838	-0.299871	-0.038703	-0.169287	down
A_33_P3298356	SH3GLB1	4.5168486	4.319942	4.3751554	-0.196907	-0.141693	-0.1693	down
A_21_P0010331	lnc-NCF4-2	-2.335722	-2.168441	-2.841903	0.1672816	-0.50618	-0.169449	down
A_23_P15414	SCARF1	-2.611616	-2.883737	-2.678401	-0.272121	-0.066785	-0.169453	down
A_23_P205567	PRKCH	2.3981953	2.2456784	2.2115812	-0.152517	-0.186614	-0.169565	down
A_33_P3405444	WASL	3.4135246	3.3934245	3.0944347	-0.0201	-0.31909	-0.169595	down
A_33_P3356406	SOS1	-0.808207	-1.1309	-0.824744	-0.322693	-0.016538	-0.169616	down
A_22_P00003394	lnc-CCDC117-1	-2.694789	-2.988691	-2.740193	-0.293903	-0.045404	-0.169653	down
A_23_P15511	DDX5	6.732009	6.539643	6.5848656	-0.192366	-0.147143	-0.169755	down
A_33_P3358938	CLOCK	0.9488692	0.8132792	0.7449236	-0.13559	-0.203946	-0.169768	down
A_33_P3242873	MPC1	1.005012	0.7776017	0.8925977	-0.22741	-0.112414	-0.169912	down
A_24_P89080	DCK	0.5324326	0.2864103	0.4386287	-0.246022	-0.093804	-0.169913	down
A_33_P3243887	IL11	0.3281884	0.3758597	-0.059421	0.0476713	-0.387609	-0.169969	down
A_21_P0010399	lnc-RGL4-3	-2.218081	-2.665823	-2.110323	-0.447742	0.107758	-0.169992	down
A_23_P162449	SRGAP1	0.3130631	0.2992673	-0.013148	-0.013796	-0.326211	-0.170004	down
A_24_P128205	PSMD1	3.9945288	3.5772023	4.071762	-0.417326	0.0772333	-0.170047	down
A_22_P00002055	lnc-BIVM-1	-2.984504	-2.927931	-3.381185	0.0565734	-0.396681	-0.170054	down
A_23_P133133	ALPK1	0.9971499	0.8554788	0.7984381	-0.141671	-0.198712	-0.170192	down
A_33_P3229215	ATP1A1-AS1	-2.85501	-2.716934	-3.333484	0.1380756	-0.478474	-0.170199	down
A_23_P218751	GNB1L	4.6113977	4.265386	4.616971	-0.346012	0.0055733	-0.170219	down
A_33_P3354796	FLJ42627	-0.036506	-0.284126	-0.129388	-0.247621	-0.092883	-0.170252	down
A_32_P131367	RNF13	1.6320143	1.4534564	1.4700332	-0.178558	-0.161981	-0.170269	down
A_21_P0000069	VEPH1	-0.521751	-0.67975	-0.704321	-0.157999	-0.18257	-0.170284	down
A_33_P3219197	TBC1D10B	-0.333479	-0.468749	-0.539446	-0.135269	-0.205966	-0.170618	down
A_32_P143880	MEMO1	1.565548	1.4322882	1.3575187	-0.13326	-0.208029	-0.170645	down
A_23_P46947	CNNM1	-0.922336	-1.252246	-0.933841	-0.329911	-0.011506	-0.170708	down
A_23_P252362	MRPS30	2.6732616	2.5646443	2.4403334	-0.108617	-0.232928	-0.170773	down
A_33_P3348782	CYP2S1	-2.4822	-2.493486	-2.812477	-0.011286	-0.330278	-0.170782	down
A_24_P28619	ANGEL2	1.9684992	1.7169642	1.8783264	-0.251535	-0.090173	-0.170854	down

A_22_P00006065	LOC101928353	3.4846535	3.4372106	3.1903467	-0.047443	-0.294307	-0.170875	down
A_33_P3245539	PNMA1	0.9616365	0.8097796	0.771668	-0.151857	-0.189969	-0.170913	down
A_24_P353794	GALNT2	1.2306657	1.1162248	1.0031943	-0.114441	-0.227471	-0.170956	down
A_33_P3325306	HSPE1	8.064689	7.7940946	7.993328	-0.270594	-0.071361	-0.170977	down
A_24_P941051	CSTF2T	1.8852577	1.4215441	2.0068226	-0.463714	0.1215649	-0.171074	down
A_21_P0013922	lnc-PPIE-1	-1.734695	-2.098046	-1.713545	-0.36335	0.0211501	-0.1711	down
A_23_P422178	CUL4B	0.7155914	0.5563536	0.5325818	-0.159238	-0.18301	-0.171124	down
A_22_P00002576	lnc-C17orf97-6	-1.931228	-2.03917	-2.165682	-0.107943	-0.234454	-0.171198	down
A_33_P3307495	STRA6	6.1750193	5.9329257	6.0745687	-0.242094	-0.100451	-0.171272	down
A_24_P913115	PTEN	1.1538925	1.1300416	0.8349767	-0.023851	-0.318916	-0.171383	down
A_24_P261169	SEMA4D	-1.309006	-1.622126	-1.338717	-0.31312	-0.029711	-0.171416	down
A_24_P183150	CXCL3	-0.768292	-0.824406	-1.055054	-0.056115	-0.286762	-0.171438	down
A_23_P70384	RNF8	1.1310577	0.8128533	1.1062651	-0.318204	-0.024793	-0.171499	down
A_33_P3247753	TSIX	-0.936516	-1.153728	-1.062407	-0.217211	-0.12589	-0.171551	down
A_23_P50000	FAM57A	1.5231204	1.2375193	1.4653287	-0.285601	-0.057792	-0.171696	down
A_33_P3269149	STX17	-0.080125	-0.28105	-0.222784	-0.200925	-0.142659	-0.171792	down
A_23_P64173	CARD16	0.4304438	0.0794911	0.4377375	-0.350953	0.0072937	-0.171829	down
A_33_P3320888	CREB3L2	-1.512019	-1.839425	-1.528288	-0.327406	-0.01627	-0.171838	down
A_33_P3678883	KIAA1654	7.930949	7.8551154	7.662776	-0.075834	-0.268173	-0.172004	down
A_21_P0007762	MAPKAPK5-AS1	1.8402319	1.5925498	1.7437868	-0.247682	-0.096445	-0.172064	down
A_23_P31389	TRA2A	0.894197	0.6528654	0.7912016	-0.241332	-0.102995	-0.172163	down
A_33_P3418726	ZNF592	-0.064033	-0.353747	-0.118704	-0.289715	-0.054672	-0.172193	down
A_21_P0001545	LOC149351	-1.559103	-1.757767	-1.70499	-0.198664	-0.145887	-0.172276	down
A_24_P381199	TRIM6	-2.359891	-2.024315	-3.040146	0.3355756	-0.680255	-0.17234	down
A_24_P253755	PIGL	-0.202703	-0.391272	-0.358823	-0.188569	-0.15612	-0.172344	down
A_24_P283288	MAPK14	-0.100872	-0.166714	-0.379879	-0.065842	-0.279007	-0.172425	down
A_33_P3300757	DOPEY1	0.7970419	0.5520864	0.6970844	-0.244956	-0.099957	-0.172457	down
A_24_P626931	UBE3D	1.064959	0.8912354	0.8937535	-0.173724	-0.171206	-0.172465	down
A_23_P157361	WDR60	0.3423796	0.2992725	0.0405068	-0.043107	-0.301873	-0.17249	down
A_23_P98631	HPS5	0.8202081	0.6014338	0.6938148	-0.218774	-0.126393	-0.172584	down
A_33_P3280739	RAB3GAP1	3.151534	3.0753531	2.8824482	-0.076181	-0.269086	-0.172633	down
A_24_P319715	PDIA6	4.2019243	4.047357	4.0112123	-0.154567	-0.190712	-0.17264	down
A_21_P0014589	LOC101929112	-0.567964	-0.70757	-0.773684	-0.139606	-0.20572	-0.172663	down
A_23_P216894	MAPKAP1	-1.013529	-1.445563	-0.926851	-0.432034	0.086678	-0.172678	down
A_24_P11587	PEX13	0.8567267	0.8463459	0.5216298	-0.010381	-0.335097	-0.172739	down
A_33_P3394395	FAM73A	1.9556208	1.9749599	1.5906868	0.0193391	-0.364934	-0.172797	down
A_22_P00020832	lnc-TMC5-1	-1.913293	-1.92167	-2.250536	-0.008378	-0.337243	-0.17281	down
A_24_P926960	MEGF6	-0.589729	-0.385408	-1.139716	0.2043209	-0.549986	-0.172833	down
A_21_P0011185	XLOC_l2_003992	-2.180419	-2.446932	-2.259656	-0.266513	-0.079237	-0.172875	down
A_24_P354615	MTMR12	-0.374046	-0.261743	-0.832129	0.1123033	-0.458083	-0.17289	down
A_22_P00004795	lnc-CXCL2-1	-2.918564	-3.248863	-2.934175	-0.330299	-0.01561	-0.172955	down
A_22_P00012468	SP2-AS1	-2.442668	-2.726144	-2.505154	-0.283476	-0.062485	-0.17298	down
A_22_P00007561	OR51B5	-2.788305	-2.84012	-3.082499	-0.051815	-0.294195	-0.173005	down
A_24_P177353	THOC2	1.9403038	1.8388014	1.6957402	-0.101502	-0.244564	-0.173033	down
A_32_P116840	CTAGE5	0.496129	0.4546466	0.1913118	-0.041482	-0.304817	-0.17315	down
A_21_P0007221	LOC101927056	-0.400352	-0.703605	-0.44344	-0.303253	-0.043087	-0.17317	down
A_21_P0010480	lnc-SMC1B-3	-0.758143	-0.579499	-1.28316	0.1786437	-0.525017	-0.173187	down
A_33_P3385361	NOL7	6.245657	5.9629493	6.1814785	-0.282708	-0.064178	-0.173443	down
A_33_P3320152	SNRPE	4.8516207	4.6106057	4.7457085	-0.241015	-0.105912	-0.173464	down
A_33_P3337009	PPCS	-0.754813	-0.844033	-1.012715	-0.08922	-0.257902	-0.173561	down
A_23_P49539	BAHCC1	-0.262869	-0.220824	-0.652149	0.0420456	-0.38928	-0.173617	down
A_23_P122662	GFOD1	2.8575401	2.6921887	2.6754246	-0.165351	-0.182116	-0.173733	down
A_23_P8913	CA2	0.3205218	0.1025147	0.1910501	-0.218007	-0.129472	-0.173739	down
A_32_P180958	PCYOX1	3.2736073	3.034103	3.1655283	-0.239504	-0.108079	-0.173792	down
A_23_P169197	HSDL2	1.7172146	1.6494808	1.4373612	-0.067734	-0.279853	-0.173794	down
A_23_P216355	TONSL	2.0045948	1.7034788	1.9581118	-0.301116	-0.046483	-0.1738	down

A_24_P283320	PCMT1	3.2928991	3.0530105	3.1851702	-0.239889	-0.107729	-0.173809	down
A_23_P2423	MAGOHB	4.893263	4.674933	4.7639656	-0.21833	-0.129297	-0.173814	down
A_22_P00008337	ITPR2	-2.013475	-2.058304	-2.316335	-0.044829	-0.30286	-0.173844	down
A_21_P0009389	lnc-TMEM98-1	-2.526056	-2.440867	-2.958938	0.0851891	-0.432882	-0.173847	down
A_23_P20463	RAD21	6.186206	6.0212026	6.0033693	-0.165003	-0.182837	-0.17392	down
A_33_P3315320	CNTD1	-1.170348	-1.350611	-1.338127	-0.180263	-0.167779	-0.174021	down
A_24_P159227	PAK6	1.1226268	0.9570904	0.9400802	-0.165536	-0.182547	-0.174042	down
A_23_P410507	PSPN	-2.148699	-2.254242	-2.391252	-0.105543	-0.242553	-0.174048	down
A_23_P71855	C5	-2.731518	-2.757005	-3.054343	-0.025487	-0.322825	-0.174156	down
A_23_P110643	CDKL3	-0.423168	-0.445251	-0.749401	-0.022082	-0.326233	-0.174158	down
A_24_P211206	TPM3	3.0880604	2.8319993	2.9957018	-0.256061	-0.092359	-0.17421	down
A_33_P3416687	TRIM16	-1.404669	-1.469031	-1.688782	-0.064362	-0.284112	-0.174237	down
A_24_P376229	UBA2	3.8346987	3.5053535	3.8154898	-0.329345	-0.019209	-0.174277	down
A_24_P203678	ACAT1	1.5239382	1.3566971	1.3426046	-0.167241	-0.181334	-0.174287	down
A_23_P123071	CAV2	-0.876841	-1.003004	-1.099365	-0.126163	-0.222525	-0.174344	down
A_33_P3264528	HOXA11	-0.885434	-1.133995	-0.985642	-0.24856	-0.100208	-0.174384	down
A_24_P9671	DNAJA1	5.5085535	5.0780454	5.590289	-0.430508	0.0817356	-0.174386	down
A_24_P321634	ZMYM5	-0.61323	-0.665687	-0.909659	-0.052457	-0.296429	-0.174443	down
A_22_P00021240	lnc-PSMG4-1	-1.423397	-1.701387	-1.494311	-0.277989	-0.070914	-0.174452	down
A_23_P113972	EXOC1	-0.121426	-0.429293	-0.162498	-0.307867	-0.041072	-0.174469	down
A_23_P314191	ZDHHC17	0.9222693	0.8434448	0.6515875	-0.078825	-0.270682	-0.174753	down
A_24_P111134	POMT2	1.5184827	1.2368355	1.4505811	-0.281647	-0.067902	-0.174774	down
A_23_P11862	C1orf112	4.0245495	3.7931666	3.90617	-0.231383	-0.11838	-0.174881	down
A_23_P391725	DR1	2.9976397	2.7844548	2.8609428	-0.213185	-0.136697	-0.174941	down
A_23_P69840	SLC35A4	2.0993118	1.4537177	2.395011	-0.645594	0.2956991	-0.174947	down
A_23_P23346	MLLT11	2.5274525	2.377685	2.3270016	-0.149767	-0.200451	-0.175109	down
A_24_P612446	C6orf89	-1.477181	-1.785803	-1.51889	-0.308622	-0.041708	-0.175165	down
A_24_P328872	CCT5	-0.405662	-0.384952	-0.776736	0.0207095	-0.371075	-0.175183	down
A_33_P6815667	RBM26-AS1	-1.519003	-1.529456	-1.859072	-0.010452	-0.340069	-0.175261	down
A_24_P362540	ASAP2	3.3200598	3.1985278	3.0908155	-0.121532	-0.229244	-0.175388	down
A_23_P109269	LAMA5	2.0944538	2.082993	1.7550373	-0.011461	-0.339417	-0.175439	down
A_33_P6816152	lnc-GTF2F2-2	-1.272594	-1.518497	-1.377602	-0.245903	-0.105009	-0.175456	down
A_24_P192805	CARD17	-1.525052	-1.723497	-1.677594	-0.198446	-0.152543	-0.175494	down
A_23_P102542	AFTPH	3.4906015	3.39004	3.2401228	-0.100562	-0.250479	-0.17552	down
A_33_P3336642	THOC3	2.5663633	2.2308593	2.5507689	-0.335504	-0.015594	-0.175549	down
A_21_P0000107	MTRNR2L7	2.8279715	2.7489028	2.5554886	-0.079069	-0.272483	-0.175776	down
A_23_P388812	CKAP2L	0.2677107	-0.185363	0.369194	-0.453074	0.1014833	-0.175795	down
A_33_P3232523	APOL4	-2.69584	-2.834499	-2.908771	-0.138659	-0.212931	-0.175795	down
A_21_P0000082	LARP4	3.7936296	3.4828172	3.752759	-0.310812	-0.040871	-0.175842	down
A_23_P69988	RARS	6.587022	6.3620563	6.460251	-0.224966	-0.126771	-0.175868	down
A_33_P3272593	CTPS2	2.7075844	2.3554797	2.7079258	-0.352105	0.0003414	-0.175882	down
A_21_P0001600	lnc-EDEM3-2	-2.64636	-2.671071	-2.973549	-0.024711	-0.327189	-0.17595	down
A_24_P188941	NPM1	9.422414	9.447752	9.044943	0.0253382	-0.377471	-0.176066	down
A_33_P3226465	LRRC37A2	-1.946556	-1.956815	-2.288731	-0.010259	-0.342175	-0.176217	down
A_33_P3365524	SETD3	-1.170716	-1.524985	-1.169048	-0.354269	0.001668	-0.176301	down
A_33_P3383724	TRIM61	-0.744493	-0.691647	-1.150024	0.052846	-0.405531	-0.176342	down
A_33_P3368188	SEPT9	0.1949697	-0.037326	0.0745416	-0.232296	-0.120428	-0.176362	down
A_23_P107412	P4HB	6.0251036	5.4566426	6.2406807	-0.568461	0.2155771	-0.176442	down
A_32_P77977	UTP11L	0.1428142	-0.244372	0.1770411	-0.387187	0.0342269	-0.17648	down
A_33_P3397568	MEMO1	4.345866	4.124533	4.214225	-0.221333	-0.131641	-0.176487	down
A_23_P53476	LDHB	8.204691	7.9361253	8.120262	-0.268566	-0.084429	-0.176497	down
A_33_P3317073	MOB1B	-1.38752	-1.700001	-1.42805	-0.312481	-0.04053	-0.176505	down
A_23_P116743	LINC01089	2.0369053	1.7987523	1.9220066	-0.238153	-0.114899	-0.176526	down
A_23_P168211	VOPP1	3.7079916	3.5282006	3.5346794	-0.179791	-0.173312	-0.176552	down
A_22_P00019662	lnc-ORAOV1-2	1.0541406	0.8735061	0.8815985	-0.180635	-0.172542	-0.176588	down
A_24_P940517	PCYOX1	2.2612658	2.0224385	2.1468687	-0.238827	-0.114397	-0.176612	down

A_23_P122304	HDAC2	5.7743645	5.5129733	5.6824265	-0.261391	-0.091938	-0.176665	down
A_22_P00001744	CENPN	3.9707184	3.5813498	4.0065174	-0.389369	0.035799	-0.176785	down
A_33_P3250268	GOLGA6L7P	-1.323709	-1.610971	-1.390276	-0.287262	-0.066567	-0.176914	down
A_23_P4919	KDELR1	3.658908	3.3267894	3.6370182	-0.332119	-0.02189	-0.177004	down
A_33_P7465707	KRAS	2.0522423	1.9015999	1.8487625	-0.150642	-0.20348	-0.177061	down
A_24_P193600	ZNF174	-1.068123	-1.331108	-1.159288	-0.262984	-0.091165	-0.177075	down
A_32_P16489	MRPL35	2.1678972	1.9183507	2.0632296	-0.249547	-0.104668	-0.177107	down
A_33_P3372705	SRGAP2B	2.1749878	1.957789	2.0378876	-0.217199	-0.1371	-0.17715	down
A_24_P203072	JADE3	1.1321898	0.8363085	1.0737243	-0.295881	-0.058465	-0.177173	down
A_24_P921155	C3orf17	0.2621179	-0.105834	0.2756677	-0.367952	0.0135498	-0.177201	down
A_23_P46964	HIF1AN	-0.747499	-0.39389	-1.455536	0.3536086	-0.708037	-0.177214	down
A_22_P00006999	lnc-GGCT-1	-2.356907	-2.626692	-2.441632	-0.269785	-0.084725	-0.177255	down
A_24_P40594	HOMEZ	-0.247756	-0.224872	-0.62526	0.0228844	-0.377503	-0.17731	down
A_23_P305616	LOC652276	-2.411256	-2.771132	-2.4061	-0.359876	0.0051558	-0.17736	down
A_21_P0014631	LOC101929787	-2.006023	-1.981136	-2.38567	0.0248861	-0.379648	-0.177381	down
A_23_P202565	SHOC2	1.8134289	1.626349	1.6457272	-0.18708	-0.167702	-0.177391	down
A_33_P3402103	STPG1	-1.992879	-2.23493	-2.105635	-0.242051	-0.112756	-0.177403	down
A_32_P163533	ZNF322	1.1522336	0.8763557	1.073225	-0.275878	-0.079009	-0.177443	down
A_21_P0009828	lnc-C20orf187-2	-1.099426	-1.213345	-1.340433	-0.113919	-0.241007	-0.177463	down
A_33_P3226380	LINC01347	-1.943469	-1.637521	-2.604384	0.3059473	-0.660916	-0.177484	down
A_24_P295963	SLC38A2	3.1007586	3.1542468	2.6922817	0.0534883	-0.408477	-0.177494	down
A_33_P3329149	CDK13	-1.049468	-1.297175	-1.156765	-0.247707	-0.107296	-0.177502	down
A_23_P65712	FAM103A1	2.7998857	2.0293345	3.2153587	-0.770551	0.415473	-0.177539	down
A_19_P00330814	HOTAIR	1.1942358	1.1346889	0.8986702	-0.059547	-0.295566	-0.177556	down
A_33_P3324651	RBM39	5.7111883	5.43768	5.6294975	-0.273509	-0.081691	-0.1776	down
A_23_P215070	CEP41	-0.000805	-0.118442	-0.238408	-0.117637	-0.237603	-0.17762	down
A_33_P3390456	YAF2	-0.47296	-0.743801	-0.557405	-0.270841	-0.084445	-0.177643	down
A_22_P00002567	C17orf85	2.1855526	1.9797196	2.036049	-0.205833	-0.149504	-0.177668	down
A_22_P00012128	lnc-POLR1E-2	0.2313604	0.0326815	0.0746212	-0.198679	-0.156739	-0.177709	down
A_24_P317874	KIN	2.2470427	2.1195674	2.0190897	-0.127475	-0.227953	-0.177714	down
A_23_P333498	EEPD1	2.8258276	2.6883273	2.6078424	-0.1375	-0.217985	-0.177743	down
A_33_P3269109	GUCY1A3	-0.89164	-1.271197	-0.867625	-0.379558	0.024015	-0.177771	down
A_24_P841677	CXorf23	-2.676654	-2.45767	-3.251233	0.2189846	-0.574579	-0.177797	down
A_33_P3223088	MRPL19	0.3091936	0.0647893	0.1979885	-0.244404	-0.111205	-0.177805	down
A_33_P3416946	ACTR10	-0.330424	-0.525422	-0.491079	-0.194997	-0.160655	-0.177826	down
A_21_P0012831	DOCK9	-2.109052	-2.328742	-2.245046	-0.21969	-0.135994	-0.177842	down
A_24_P225907	DPH3	1.6301584	1.4092054	1.4954252	-0.220953	-0.134733	-0.177843	down
A_33_P3383611	KCP	0.4544144	0.2970376	0.2560782	-0.157377	-0.198336	-0.177856	down
A_21_P0011551	XLOC_l2_005874	1.670824	1.5163622	1.4695225	-0.154462	-0.201302	-0.177882	down
A_21_P0000064	HPSE	-2.152981	-2.682705	-1.979261	-0.529724	0.1737204	-0.178002	down
A_23_P435697	EDRF1	1.827239	1.5894685	1.7089648	-0.237771	-0.118274	-0.178022	down
A_23_P119923	CNNM4	4.787402	4.322045	4.896468	-0.465357	0.109066	-0.178146	down
A_32_P75094	AIFM2	4.1972685	3.940125	4.0979586	-0.257144	-0.09931	-0.178227	down
A_23_P205074	SLC46A3	-1.45064	-1.603537	-1.65421	-0.152897	-0.20357	-0.178233	down
A_23_P211110	SIM2	0.9788632	0.692378	0.9087181	-0.286485	-0.070145	-0.178315	down
A_33_P3362891	HNRNPA2B1	5.753645	5.4757524	5.6747656	-0.277893	-0.078879	-0.178386	down
A_23_P359655	ZNF664	5.1796274	5.0038304	4.9986296	-0.175797	-0.180998	-0.178397	down
A_23_P417942	FNBP1L	3.4496746	3.5494199	2.9930458	0.0997453	-0.456629	-0.178442	down
A_23_P255653	TNFRSF10A	0.6594391	0.2191987	0.7426934	-0.44024	0.0832543	-0.178493	down
A_23_P305723	MIER1	2.3059397	2.2566333	1.9979258	-0.049306	-0.308014	-0.17866	down
A_32_P157965	EIF2S2	3.7931623	3.8823037	3.346692	0.0891414	-0.44647	-0.178664	down
A_32_P235796	BBS12	0.4232078	0.3917084	0.0970163	-0.031499	-0.326191	-0.178845	down
A_22_P00012089	lnc-PMP2-1	-1.182964	-1.623575	-1.100078	-0.440611	0.0828862	-0.178862	down
A_24_P305345	CD209	-2.650517	-2.881332	-2.777559	-0.230815	-0.127042	-0.178928	down
A_24_P940666	MED1	2.0053215	1.9037318	1.7489972	-0.10159	-0.256324	-0.178957	down
A_32_P213624	RTCA	1.3873749	0.9545298	1.4621773	-0.432845	0.0748024	-0.179021	down

A_23_P153084	RALBP1	3.3667498	3.140923	3.234518	-0.225827	-0.132232	-0.179029	down
A_33_P3353791	ITGA1	0.151659	-0.022748	-0.032047	-0.174407	-0.183706	-0.179056	down
A_21_P0013191	XLOC_l2_013330	-1.584874	-1.804907	-1.723205	-0.220034	-0.138331	-0.179183	down
A_33_P3349269	PIAS2	1.8102636	1.5123734	1.7497869	-0.29789	-0.060477	-0.179183	down
A_23_P23542	RPAP2	0.9018922	0.6061964	0.8391895	-0.295696	-0.062703	-0.179199	down
A_23_P318420	CTTNBP2NL	1.331665	1.1567822	1.1481233	-0.174883	-0.183542	-0.179212	down
A_33_P3330211	AUTS2	-2.021796	-2.289561	-2.112534	-0.267765	-0.090738	-0.179251	down
A_33_P3807268	DNAJA1P5	4.581828	4.1238265	4.68124	-0.458002	0.099412	-0.179295	down
A_22_P00018004	LOC100289333	-1.253639	-1.467681	-1.398187	-0.214042	-0.144548	-0.179295	down
A_24_P49447	RIC1	1.521575	1.3671694	1.3173852	-0.154406	-0.20419	-0.179298	down
A_33_P3416483	HNRNPU-AS1	-1.212141	-1.56339	-1.219697	-0.351249	-0.007556	-0.179403	down
A_23_P141100	ADAT1	1.0984573	0.9336476	0.9044242	-0.16481	-0.194033	-0.179421	down
A_23_P101950	MDH1	7.615103	7.3262143	7.5450764	-0.288888	-0.070026	-0.179457	down
A_24_P365767	CYBB	-0.701759	-0.760323	-1.002241	-0.058563	-0.300481	-0.179522	down
A_33_P3393931	SSBP3-AS1	-2.914085	-3.130276	-3.056951	-0.216191	-0.142866	-0.179529	down
A_24_P143686	SPIDR	0.9520559	0.83218	0.7128539	-0.119876	-0.239202	-0.179539	down
A_24_P148151	TSNAX	1.1349521	0.7495399	1.161284	-0.385412	0.0263319	-0.17954	down
A_33_P3340666	KRTAP19-5	1.3049574	0.8542495	1.3965349	-0.450708	0.0915775	-0.179565	down
A_33_P3422888	CLEC16A	-1.804655	-1.891693	-2.07676	-0.087038	-0.272105	-0.179572	down
A_24_P237486	MECP2	0.2506232	-0.114544	0.2562537	-0.365167	0.0056305	-0.179768	down
A_24_P363548	HIP1	0.7360339	0.1043253	1.0080066	-0.631709	0.2719727	-0.179868	down
A_32_P150030	PPM1D	2.0583162	1.9521289	1.8046913	-0.106187	-0.253625	-0.179906	down
A_21_P0009114	LOC101928659	1.2082963	0.8173413	1.2394152	-0.390955	0.0311189	-0.179918	down
A_33_P3238573	DENND4B	4.3651905	4.2334485	4.136964	-0.131742	-0.228227	-0.179984	down
A_21_P0012058	HS1BP3-IT1	-1.992558	-2.377509	-1.967626	-0.384951	0.0249324	-0.180009	down
A_23_P127995	CLNS1A	5.44119	5.5464272	4.975789	0.1052375	-0.465401	-0.180082	down
A_23_P133665	FRK	0.485683	0.3391485	0.2718372	-0.146534	-0.213846	-0.18019	down
A_24_P406830	MTMR2	2.49621	2.3263726	2.3055258	-0.169837	-0.190684	-0.180261	down
A_23_P47410	ESAM	-0.824143	-0.761569	-1.247469	0.0625749	-0.423326	-0.180376	down
A_21_P0000591	SNORA70F	0.2438426	0.1152091	0.0116506	-0.128634	-0.232192	-0.180413	down
A_23_P132845	CLCN2	0.7321954	0.5553665	0.5481315	-0.176829	-0.184064	-0.180446	down
A_23_P74299	ADORA1	-2.222133	-2.836157	-1.969012	-0.614024	0.2531209	-0.180452	down
A_24_P252794	CUL2	0.5025973	0.3476615	0.2964144	-0.154936	-0.206183	-0.180559	down
A_33_P3383034	UBA2	4.8658066	4.5640464	4.806445	-0.30176	-0.059361	-0.180561	down
A_32_P118372	INTU	-0.385793	-0.340561	-0.792306	0.0452323	-0.406513	-0.18064	down
A_23_P97123	ANKRD36BP1	-0.415858	-0.770354	-0.422711	-0.354496	-0.006853	-0.180674	down
A_23_P69141	DYNC1LI1	-0.123855	-0.363782	-0.245357	-0.239927	-0.121501	-0.180714	down
A_23_P48807	GALK2	3.008954	2.7748885	2.8815374	-0.234066	-0.127417	-0.180741	down
A_23_P120048	BAZ2B	0.3111067	0.4088903	-0.148313	0.0977836	-0.459419	-0.180818	down
A_24_P832426	B3GALTL	1.1362333	1.253881	0.6568942	0.1176477	-0.479339	-0.180846	down
A_33_P3231557	CDC14B	0.0542474	-0.14134	-0.111945	-0.195588	-0.166192	-0.18089	down
A_23_P2203	TAOK3	-1.438542	-1.694417	-1.544603	-0.255875	-0.106061	-0.180968	down
A_23_P330836	LINC01560	-3.127799	-3.096667	-3.520936	0.031132	-0.393137	-0.181002	down
A_33_P3214027	SPECC1L	-1.60928	-1.774427	-1.806248	-0.165147	-0.196968	-0.181057	down
A_21_P0013772	JPX	-2.74706	-2.626655	-3.229633	0.1204047	-0.482573	-0.181084	down
A_23_P32253	NFIL3	2.8890572	2.9409413	2.4747772	0.0518842	-0.41428	-0.181198	down
A_21_P0007168	LOC101927503	-0.971534	-1.167497	-1.137981	-0.195963	-0.166447	-0.181205	down
A_22_P00000250	LOC284009	-1.574998	-1.843267	-1.669285	-0.268269	-0.094286	-0.181278	down
A_33_P3301010	SPIRE1	-0.613287	-0.405881	-1.183286	0.207406	-0.569999	-0.181297	down
A_21_P0001471	lnc-ZBTB17-2	-2.159251	-2.270535	-2.41068	-0.111284	-0.251429	-0.181356	down
A_21_P0000669	EFTUD1	-1.051294	-1.252032	-1.213325	-0.200738	-0.16203	-0.181384	down
A_24_P403459	IFNA4	-2.001387	-2.286808	-2.078735	-0.285421	-0.077349	-0.181385	down
A_19_P00315502	LOC102723721	3.290944	2.9439635	3.2751083	-0.346981	-0.015836	-0.181408	down
A_19_P00809029	GGACT	-2.374366	-2.953424	-2.158527	-0.579058	0.2158384	-0.18161	down
A_23_P400465	GTF3C6	5.560279	5.532581	5.2245865	-0.027698	-0.335692	-0.181695	down
A_23_P121825	TRAPPC13	1.1208801	0.9842773	0.8940816	-0.136603	-0.226799	-0.181701	down

A_33_P3271323	RHOQ	3.728156	3.714954	3.377821	-0.013202	-0.350335	-0.181769	down
A_33_P3287735	KAT6A	2.7981157	2.3907437	2.841897	-0.407372	0.0437813	-0.181795	down
A_33_P3243175	PNPO	4.349681	3.8563552	4.4794054	-0.493326	0.1297245	-0.181801	down
A_23_P377888	FAN1	2.8253326	2.6717563	2.6150732	-0.153576	-0.210259	-0.181918	down
A_22_P00007979	IDS	1.0937052	1.0385323	0.7848196	-0.055173	-0.308886	-0.182029	down
A_22_P00020384	SYNJ2BP-COX16	4.748766	4.581987	4.5514717	-0.166779	-0.197294	-0.182037	down
A_23_P383258	GDA	1.4752631	1.4053836	1.1810403	-0.06988	-0.294223	-0.182051	down
A_24_P943575	CHD6	1.7191305	1.5370889	1.5369997	-0.182042	-0.182131	-0.182086	down
A_33_P3330498	ALDH7A1	-0.962198	-1.250143	-1.038449	-0.287945	-0.076251	-0.182098	down
A_23_P18465	RFC1	1.701745	1.5467334	1.4925089	-0.155012	-0.209236	-0.182124	down
A_24_P345822	TFG	3.7903748	3.6748414	3.5415754	-0.115533	-0.248799	-0.182166	down
A_33_P3375185	TCTEX1D4	-1.110346	-1.281074	-1.304811	-0.170728	-0.194465	-0.182597	down
A_33_P3253975	ACAT1	1.1317773	0.9549923	0.9431691	-0.176785	-0.188608	-0.182697	down
A_33_P3231472	TOR1B	1.0109649	0.7388487	0.917346	-0.272116	-0.093619	-0.182868	down
A_33_P3209537	EBF4	-1.95592	-1.95137	-2.326278	0.0045495	-0.370358	-0.182904	down
A_23_P150741	C2CD3	2.8356056	2.6491466	2.6562548	-0.186459	-0.179351	-0.182905	down
A_32_P10396	WDFY3	0.5406375	0.3569851	0.3584595	-0.183652	-0.182178	-0.182915	down
A_23_P55256	ZNF652	1.8490181	1.8800015	1.4521208	0.0309834	-0.396897	-0.182957	down
A_23_P33187	HIATL1	-0.051754	-0.301993	-0.167432	-0.250239	-0.115678	-0.182959	down
A_23_P401709	C20orf196	-1.480739	-1.674159	-1.653257	-0.19342	-0.172519	-0.18297	down
A_24_P414952	TMEM168	-1.490149	-1.463716	-1.882679	0.0264325	-0.39253	-0.183049	down
A_24_P298545	ZNF257	2.1367168	1.9676757	1.9394102	-0.169041	-0.197307	-0.183174	down
A_33_P3393843	SECISBP2	-0.841808	-0.792997	-1.25736	0.0488105	-0.415552	-0.183371	down
A_23_P96383	SRPX	-0.200764	-0.693908	-0.074481	-0.493144	0.1262827	-0.18343	down
A_24_P27229	CSAD	-0.645557	-0.772241	-0.885756	-0.126684	-0.240199	-0.183441	down
A_24_P94054	STK4	1.7081165	1.3069658	1.7423544	-0.401151	0.0342379	-0.183456	down
A_33_P3347417	SPEN	1.1231141	0.8892674	0.9899426	-0.233847	-0.133172	-0.183509	down
A_21_P0003760	lnc-SEC24D-1	-1.727161	-1.913433	-1.907941	-0.186272	-0.18078	-0.183526	down
A_23_P109420	BMS1	3.5266647	3.1905618	3.4957142	-0.336103	-0.030951	-0.183527	down
A_32_P49848	RHOQ	0.4363542	0.3108258	0.1947432	-0.125528	-0.241611	-0.18357	down
A_33_P3325280	SDHAP1	2.0819473	1.8943462	1.9023209	-0.187601	-0.179626	-0.183614	down
A_33_P3313704	LOC100128374	-2.258868	-2.874278	-2.010769	-0.61541	0.2480989	-0.183656	down
A_23_P45851	HIAT1	3.923297	3.5848832	3.894353	-0.338414	-0.028944	-0.183679	down
A_33_P3223980	TPM3	3.682889	3.3672242	3.6311789	-0.315665	-0.05171	-0.183687	down
A_33_P3339820	LEPR	7.364192	7.212497	7.1482916	-0.151695	-0.2159	-0.183798	down
A_33_P3314446	NF1P2	0.5645871	0.5139413	0.2475953	-0.050646	-0.316992	-0.183819	down
A_24_P363005	UBE2D3	5.739626	5.4703794	5.6412344	-0.269247	-0.098392	-0.183819	down
A_22_P00024722	lnc-GRHL2-1	-1.462314	-1.539506	-1.752973	-0.077192	-0.290659	-0.183926	down
A_33_P3384452	TFDP1	4.835862	4.2984405	5.0054035	-0.537422	0.1695414	-0.18394	down
A_33_P3375809	LOC729970	-0.620615	-0.957	-0.65234	-0.336385	-0.031726	-0.184056	down
A_22_P00007962	lnc-ID1-1	0.0241876	-0.270566	-0.049333	-0.294754	-0.07352	-0.184137	down
A_23_P162288	MYO1A	-2.330054	-2.789842	-2.238561	-0.459788	0.0914931	-0.184147	down
A_24_P67585	VGLL3	-1.982872	-2.088481	-2.245604	-0.105609	-0.262732	-0.184171	down
A_33_P3777584	SERAC1	0.2032399	0.0862799	-0.048169	-0.11696	-0.251409	-0.184184	down
A_21_P0001992	LOC101927534	-2.163606	-2.411911	-2.283689	-0.248305	-0.120084	-0.184194	down
A_23_P22671	VAMP7	1.9696665	2.1402612	1.4305682	0.1705947	-0.539098	-0.184252	down
A_23_P398275	GOLGA2P7	4.72278	4.461204	4.6158476	-0.261576	-0.106933	-0.184254	down
A_33_P3368301	BOLA3	3.925786	3.441378	4.041646	-0.484408	0.11586	-0.184274	down
A_22_P00000555	LOC101926892	-1.461104	-1.561944	-1.728873	-0.10084	-0.267768	-0.184304	down
A_33_P3413597	NDUFB3	7.11553	6.7708707	7.091466	-0.344659	-0.024064	-0.184362	down
A_33_P3302518	CCDC40	-2.965554	-2.998542	-3.301325	-0.032988	-0.335771	-0.18438	down
A_23_P88249	RBM23	1.3646445	0.6720538	1.6883512	-0.692591	0.3237066	-0.184442	down
A_23_P339098	SLC35F2	4.3982687	4.0607486	4.3669014	-0.33752	-0.031367	-0.184444	down
A_32_P12639	PSMD12	3.931058	3.615996	3.8770914	-0.315062	-0.053967	-0.184514	down
A_22_P00023495	NDUFV2-AS1	-1.983496	-2.18306	-2.153033	-0.199564	-0.169537	-0.184551	down
A_24_P261724	RNF10	3.791976	3.5338635	3.68077	-0.258112	-0.111206	-0.184659	down

A_24_P184799	COCH	-1.017483	-1.527631	-0.87674	-0.510148	0.1407433	-0.184702	down
A_33_P3244026	MAVS	1.0096755	0.747191	0.9026575	-0.262485	-0.107018	-0.184751	down
A_23_P24723	TMEM138	2.2505379	1.7938433	2.3376932	-0.456695	0.0871553	-0.18477	down
A_23_P350187	TET2	1.2722211	1.1256733	1.0490847	-0.146548	-0.223136	-0.184842	down
A_23_P401106	PDE2A	-1.850149	-1.786208	-2.283975	0.0639405	-0.433826	-0.184943	down
A_23_P90911	RDH14	3.0517569	2.9275775	2.8059587	-0.124179	-0.245798	-0.184989	down
A_21_P0009727	lnc-EMP3-1	-1.65478	-1.511115	-2.168435	0.1436658	-0.513655	-0.184994	down
A_23_P94319	KBTBD11	3.4348507	2.9143214	3.5853157	-0.520529	0.150465	-0.185032	down
A_33_P3408953	GGTLC2	-0.779017	-0.947106	-0.981011	-0.168088	-0.201994	-0.185041	down
A_33_P3342720	ATP2A1	-2.961499	-3.053037	-3.240145	-0.091539	-0.278646	-0.185092	down
A_33_P6818718	PITRM1-AS1	-0.493357	-0.573353	-0.783568	-0.079996	-0.290211	-0.185103	down
A_23_P250619	ZDHHC14	1.5914268	1.2312846	1.5812502	-0.360142	-0.010177	-0.185159	down
A_24_P382187	IGFBP4	0.1173968	-0.442544	0.3069315	-0.559941	0.1895347	-0.185203	down
A_33_P3361746	TRPS1	3.588377	3.4122596	3.3939943	-0.176117	-0.194383	-0.18525	down
A_33_P3407618	LOC727751	0.4137468	0.3630209	0.0936198	-0.050726	-0.320127	-0.185426	down
A_33_P3378925	RBM14	2.7688732	2.4994903	2.667344	-0.269383	-0.101529	-0.185456	down
A_33_P3261818	MDH1	6.3465643	6.0053897	6.3167543	-0.341175	-0.02981	-0.185492	down
A_23_P321201	DENND5A	0.1668334	-0.047406	0.0100393	-0.214239	-0.156794	-0.185517	down
A_23_P77859	TMEM88	-2.498464	-2.696187	-2.671965	-0.197723	-0.1735	-0.185611	down
A_23_P79725	COPS7B	-0.862664	-1.257663	-0.839191	-0.394999	0.0234728	-0.185763	down
A_33_P3243812	WASF1	-0.630065	-0.664283	-0.967427	-0.034217	-0.337361	-0.185789	down
A_21_P0013176	XLOC_l2_013293	-1.419951	-1.724215	-1.487344	-0.304264	-0.067393	-0.185828	down
A_23_P308032	NUP98	3.1048946	2.830175	3.007905	-0.27472	-0.09699	-0.185855	down
A_33_P3410599	FAM46A	0.6839376	0.4719987	0.5240865	-0.211939	-0.159851	-0.185895	down
A_23_P99996	MAGEL2	-1.817475	-1.863253	-2.143531	-0.045778	-0.326056	-0.185917	down
A_23_P131074	THEG	-2.509332	-2.959189	-2.431409	-0.449856	0.0779235	-0.185966	down
A_23_P131240	UBXN2A	0.8760452	0.571815	0.8082566	-0.30423	-0.067789	-0.186009	down
A_33_P3351566	ETNK1	-0.868127	-1.176283	-0.932144	-0.308156	-0.064017	-0.186086	down
A_33_P3260572	CLIP4	0.8590751	0.7702851	0.5756221	-0.08879	-0.283453	-0.186121	down
A_23_P165783	MLPH	1.9359188	1.9735866	1.5259509	0.0376678	-0.409968	-0.18615	down
A_23_P103756	OVGP1	-0.407887	-0.952615	-0.235859	-0.544728	0.1720276	-0.18635	down
A_23_P1387	ARHGAP19	-0.979699	-1.337038	-0.995312	-0.357339	-0.015614	-0.186476	down
A_32_P50123	SRGAP2B	-2.210368	-2.475978	-2.317809	-0.26561	-0.10744	-0.186525	down
A_22_P00005153	lnc-DHX57-2	-2.845593	-2.911547	-3.152859	-0.065954	-0.307266	-0.18661	down
A_23_P342067	UHMK1	2.8924026	2.8676963	2.5438051	-0.024706	-0.348598	-0.186652	down
A_24_P382319	CEACAM1	1.319077	0.524724	1.7401156	-0.794353	0.4210386	-0.186657	down
A_24_P210513	BTBD7	-1.113849	-1.170996	-1.430088	-0.057147	-0.316238	-0.186693	down
A_23_P47565	LDHA	6.8717785	6.4985156	6.871645	-0.373263	-0.000134	-0.186698	down
A_33_P3406066	ZNF776	2.02534	1.9119086	1.7650933	-0.113431	-0.260247	-0.186839	down
A_23_P71821	PBX3	-0.021057	-0.450905	0.0349727	-0.429848	0.0560298	-0.186909	down
A_33_P3367596	CLCN4	-1.908815	-2.130965	-2.060581	-0.222149	-0.151766	-0.186958	down
A_33_P3335845	WDR33	3.73907	3.4463553	3.657711	-0.292715	-0.081359	-0.187037	down
A_33_P3291998	RALGPS2	-3.22868	-3.292066	-3.539394	-0.063386	-0.310714	-0.18705	down
A_33_P3270034	NCOA4	6.934145	6.7509513	6.743232	-0.183194	-0.190913	-0.187053	down
A_21_P0012654	LOC101928803	1.7356558	1.628406	1.468616	-0.10725	-0.26704	-0.187145	down
A_33_P3257594	CCDC103	-2.404739	-2.304603	-2.879236	0.1001368	-0.474496	-0.18718	down
A_23_P212400	NAT6	-0.626664	-0.813875	-0.813835	-0.187212	-0.187171	-0.187191	down
A_33_P3335621	PSMD10	-0.224927	-0.519986	-0.304317	-0.295059	-0.07939	-0.187224	down
A_21_P0014221	LOC100507464	-2.334513	-2.737948	-2.305687	-0.403435	0.0288255	-0.187305	down
A_23_P7056	SDAD1	0.3459201	0.0895395	0.2273707	-0.256381	-0.118549	-0.187465	down
A_23_P320250	MYO19	4.859212	4.5905013	4.7527695	-0.268711	-0.106442	-0.187577	down
A_24_P112447	ENTPD7	-0.935037	-0.772989	-1.472287	0.1620479	-0.537251	-0.187601	down
A_23_P17955	FBXL2	-0.658536	-1.071233	-0.621085	-0.412697	0.0374513	-0.187623	down
A_33_P3416762	LYPLA1	2.985546	2.6816344	2.9141922	-0.303912	-0.071354	-0.187633	down
A_33_P3313864	lnc-CTIF-1	-0.741108	-0.909098	-0.948482	-0.16799	-0.207374	-0.187682	down
A_23_P35354	CWF19L1	1.1113567	0.9600692	0.8871918	-0.151288	-0.224165	-0.187726	down

A_33_P3349414	ZBTB43	3.4599314	3.5082526	3.0359564	0.0483212	-0.423975	-0.187827	down
A_22_P00003889	ZNF608	-0.469286	-0.377976	-0.936405	0.0913105	-0.467119	-0.187904	down
A_24_P416289	KIAA0195	0.0290699	-0.440774	0.1230669	-0.469844	0.093997	-0.187923	down
A_23_P140907	TMEM8A	3.172346	2.7740455	3.1947517	-0.398301	0.0224056	-0.187948	down
A_22_P00002617	lnc-C1orf122-1	-2.58029	-2.493303	-3.043212	0.0869877	-0.462922	-0.187967	down
A_33_P3300212	BLCAP	1.0446486	0.6445699	1.068615	-0.400079	0.0239663	-0.188056	down
A_23_P167276	PAQR3	2.0655298	1.7873912	1.9674511	-0.278139	-0.098079	-0.188109	down
A_24_P386622	ARRB1	0.678442	0.4348326	0.5457192	-0.243609	-0.132723	-0.188166	down
A_23_P258964	IARS	5.4867487	5.25619	5.3408756	-0.230559	-0.145873	-0.188216	down
A_33_P3378772	FAM98B	-0.75531	-1.16072	-0.726362	-0.40541	0.0289483	-0.188231	down
A_33_P3215277	TTBK2	0.0198817	-0.06349	-0.27344	-0.083372	-0.293322	-0.188347	down
A_33_P3334248	FBRS	-2.113588	-1.964186	-2.6398	0.1494026	-0.526212	-0.188405	down
A_22_P00007300	lnc-GPR39-1	-2.425811	-2.96918	-2.25926	-0.543369	0.1665509	-0.188409	down
A_23_P325501	MORC3	1.124198	1.1540766	0.7173901	0.0298786	-0.406808	-0.188465	down
A_22_P00008272	CRNDE	2.646657	2.665649	2.2506666	0.0189919	-0.39599	-0.188499	down
A_33_P3363637	BLNK	1.4197035	1.1471863	1.3150344	-0.272517	-0.104669	-0.188593	down
A_22_P00014252	lnc-SEMA4D-2	-2.983008	-3.183797	-3.159723	-0.200789	-0.176715	-0.188752	down
A_23_P162300	IRAK3	-0.512651	-0.686503	-0.716442	-0.173852	-0.203791	-0.188822	down
A_21_P0010951	LOC101929506	-0.843356	-1.097578	-0.966911	-0.254221	-0.123555	-0.188888	down
A_33_P3311791	ZNF749	-0.729091	-0.782767	-1.053262	-0.053677	-0.324171	-0.188924	down
A_21_P0011708	XLOC_l2_006804	-2.508054	-2.872417	-2.521651	-0.364363	-0.013597	-0.18898	down
A_33_P3336198	BRICD5	0.7284732	0.407033	0.6718888	-0.32144	-0.056584	-0.189012	down
A_33_P3358745	SEPP1	1.0953422	1.2118125	0.6008277	0.1164703	-0.494514	-0.189022	down
A_23_P385938	PAN3	0.7262187	0.497797	0.5764489	-0.228422	-0.14977	-0.189096	down
A_23_P24244	FAM208B	2.1336126	1.9393096	1.9495525	-0.194303	-0.18406	-0.189182	down
A_23_P19702	TAB2	4.8565893	4.4709563	4.8638496	-0.385633	0.0072603	-0.189186	down
A_33_P3246990	XIAP	3.3651676	3.1244283	3.2275314	-0.240739	-0.137636	-0.189188	down
A_24_P130026	BAG5	3.535674	3.3873477	3.3050432	-0.148326	-0.230631	-0.189479	down
A_23_P105619	TMEM116	1.1017132	0.9656825	0.8586998	-0.136031	-0.243013	-0.189522	down
A_23_P107693	ZNF586	-0.632694	-0.787926	-0.856824	-0.155232	-0.22413	-0.189681	down
A_23_P43326	SPTLC1	3.7597466	3.4845433	3.6552734	-0.275203	-0.104473	-0.189838	down
A_19_P00809558	SSB	5.3089905	5.157965	5.080262	-0.151025	-0.228728	-0.189877	down
A_33_P3807062	HJURP	5.1479425	4.840675	5.0753613	-0.307268	-0.072581	-0.189924	down
A_33_P3388855	LRPPRC	1.4565325	1.2430863	1.290101	-0.213446	-0.166431	-0.189939	down
A_23_P323943	SLC5A12	-1.852166	-2.079079	-2.005185	-0.226913	-0.153019	-0.189966	down
A_23_P18422	MRPL3	5.560214	5.3100085	5.430443	-0.250206	-0.129771	-0.189988	down
A_22_P00017190	lnc-UCHL3-1	-1.944256	-2.148291	-2.120542	-0.204035	-0.176285	-0.19016	down
A_23_P16908	DHX57	0.2972808	-0.010342	0.2244988	-0.307622	-0.072782	-0.190202	down
A_32_P201979	HECTD2	-0.294643	-0.284791	-0.684977	0.0098519	-0.390334	-0.190241	down
A_21_P0004289	lnc-RP11-43D2.2.1-10	-0.640897	-0.801073	-0.861527	-0.160176	-0.220631	-0.190403	down
A_33_P3399453	CAPZA1	1.5026069	1.2125525	1.411797	-0.290054	-0.09081	-0.190432	down
A_22_P00004907	LINC00460	-1.689669	-2.017249	-1.743051	-0.32758	-0.053381	-0.19048	down
A_33_P3333156	C11orf70	0.0920754	-0.435467	0.2385235	-0.527542	0.1464481	-0.190547	down
A_24_P350744	KIAA0907	-0.389771	-0.45634	-0.704357	-0.066568	-0.314585	-0.190577	down
A_33_P3231888	C5orf15	2.1099253	2.017942	1.8206191	-0.091983	-0.289306	-0.190645	down
A_23_P129103	GOLGA2P7	5.447894	5.405687	5.108717	-0.042207	-0.339177	-0.190692	down
A_23_P380965	JKAMP	3.6559982	3.3222451	3.6083212	-0.333753	-0.047677	-0.190715	down
A_23_P208880	UHRF1	3.5835485	3.3675694	3.418065	-0.215979	-0.165483	-0.190731	down
A_32_P8402	SYNCRIP	4.1523886	3.8614326	4.0618067	-0.290956	-0.090582	-0.190769	down
A_21_P0005463	lnc-COX19-1	-0.990312	-1.468574	-0.893664	-0.478261	0.0966482	-0.190807	down
A_23_P110961	MPC1	4.81915	4.5126147	4.743924	-0.306535	-0.075226	-0.190881	down
A_22_P00020809	LOC644277	-2.192847	-2.324838	-2.442627	-0.131991	-0.249779	-0.190885	down
A_23_P59294	RREB1	-2.339224	-2.128357	-2.932031	0.2108674	-0.592807	-0.19097	down
A_21_P0011133	TPTE2	-0.938055	-1.296225	-0.961942	-0.358171	-0.023888	-0.191029	down
A_33_P3410093	LTA4H	2.1679373	1.9311204	2.0225077	-0.236817	-0.14543	-0.191123	down

A_23_P164196	DLX4	-0.556028	-0.730726	-0.763776	-0.174698	-0.207748	-0.191223	down
A_33_P3387901	SLC25A19	0.6100059	0.2643299	0.5732136	-0.345676	-0.036792	-0.191234	down
A_24_P48791	EIF2S2	0.6039376	0.6106877	0.2146373	0.0067501	-0.3893	-0.191275	down
A_33_P3387300	FXR1	4.0921955	3.914719	3.8869267	-0.177476	-0.205269	-0.191373	down
A_23_P48481	RAD51B	-0.642591	-0.705841	-0.962177	-0.06325	-0.319587	-0.191418	down
A_23_P138975	RBM7	0.9234786	0.9099665	0.5538716	-0.013512	-0.369607	-0.19156	down
A_21_P0001284	lnc-AL591845.1-2	-2.794049	-2.67044	-3.300861	0.1236093	-0.506811	-0.191601	down
A_23_P105392	CCT2	8.13555	7.809608	8.078074	-0.325942	-0.057475	-0.191708	down
A_23_P62920	KIFAP3	1.876833	1.8173919	1.5527291	-0.059441	-0.324104	-0.191772	down
A_23_P59410	KIF25	-0.929998	-0.714107	-1.529608	0.2158918	-0.599609	-0.191859	down
A_21_P0000004	SYS1	2.9008064	2.5958076	2.8220778	-0.304999	-0.078729	-0.191864	down
A_24_P236445	DYNC1LI2	1.7029982	1.433598	1.5886397	-0.2694	-0.114358	-0.191879	down
A_24_P201153	TJP2	0.9745789	0.8293295	0.7359705	-0.145249	-0.238608	-0.191929	down
A_33_P3362770	RRN3	-0.038992	-0.344521	-0.117331	-0.305529	-0.078339	-0.191934	down
A_24_P129232	SERINC1	3.6049538	3.4224944	3.4035444	-0.182459	-0.201409	-0.191934	down
A_33_P3422233	UVSSA	1.6256771	1.610434	1.2570448	-0.015243	-0.368632	-0.191938	down
A_33_P3215078	CS	7.037262	6.583652	7.1068926	-0.45361	0.0696306	-0.19199	down
A_24_P96593	EVI5	-0.21784	-0.340237	-0.479463	-0.122397	-0.261622	-0.19201	down
A_24_P302038	FCHSD2	-1.917999	-2.142434	-2.077822	-0.224435	-0.159822	-0.192129	down
A_33_P3318534	PXYLP1	-1.42553	-1.77556	-1.459805	-0.35003	-0.034275	-0.192152	down
A_24_P251534	CTDSPL	3.207532	3.0702815	2.9603386	-0.13725	-0.247193	-0.192222	down
A_23_P146058	ATP6V1C1	4.5280895	4.328881	4.342743	-0.199209	-0.185347	-0.192278	down
A_23_P51508	DUSP12	5.2872086	5.1357627	5.054043	-0.151446	-0.233166	-0.192306	down
A_21_P0002355	lnc-SLC4A3-3	-1.397587	-1.367202	-1.812701	0.030385	-0.415114	-0.192364	down
A_23_P433111	SETD9	0.4252124	0.049046	0.4165807	-0.376166	-0.008632	-0.192399	down
A_23_P25003	CLIP1	3.1314545	2.9306693	2.9472933	-0.200785	-0.184161	-0.192473	down
A_24_P601972	C2orf74	-0.207155	-0.214215	-0.585116	-0.007061	-0.377962	-0.192511	down
A_23_P88522	NMB	6.42196	5.9718795	6.486966	-0.45008	0.0650063	-0.192537	down
A_33_P3313476	ZAR1L	-2.966315	-3.365877	-2.951844	-0.399563	0.0144711	-0.192546	down
A_24_P148043	FAM20B	5.63206	5.4570584	5.421938	-0.175002	-0.210122	-0.192562	down
A_21_P0011476	CHEK2	1.3435979	1.1113315	1.1905737	-0.232266	-0.153024	-0.192645	down
A_22_P00001146	LOC283856	-1.207614	-1.615529	-1.185003	-0.407915	0.0226107	-0.192652	down
A_23_P87532	CAPRIN2	0.8937011	0.6500912	0.7519264	-0.24361	-0.141775	-0.192692	down
A_33_P6455786	ANTXR2	3.7451563	3.5270553	3.577817	-0.218101	-0.167339	-0.19272	down
A_21_P0001467	lnc-MAD2L2-1	-0.298949	-0.676392	-0.306993	-0.377443	-0.008044	-0.192744	down
A_33_P3293202	C8orf37	-1.797247	-1.996651	-1.983598	-0.199405	-0.186352	-0.192878	down
A_33_P3317282	NUS1	1.4671841	1.1968274	1.3517213	-0.270357	-0.115463	-0.19291	down
A_33_P3289218	AAK1	-0.08899	-0.077872	-0.486048	0.0111184	-0.397058	-0.19297	down
A_21_P0014357	ENDOV	-0.626347	-0.814071	-0.824689	-0.187725	-0.198343	-0.193034	down
A_23_P75973	RNF121	0.8238201	0.7567959	0.5047302	-0.067024	-0.31909	-0.193057	down
A_33_P3342111	ZNF169	0.1173615	0.0199327	-0.171446	-0.097429	-0.288808	-0.193118	down
A_33_P3312258	TUBB	4.5200214	4.202969	4.4507504	-0.317052	-0.069271	-0.193162	down
A_33_P3307068	GOLGA2	-1.891913	-2.026498	-2.143823	-0.134585	-0.251911	-0.193248	down
A_22_P00012129	lnc-POLR1E-3	-0.914627	-1.097807	-1.118152	-0.18318	-0.203526	-0.193353	down
A_24_P414719	NFYA	0.8669939	0.7589855	0.5880599	-0.108008	-0.278934	-0.193471	down
A_23_P19619	HIVEP1	0.2533374	0.2426429	-0.122919	-0.010695	-0.376256	-0.193475	down
A_23_P212522	ATP11B	-0.557998	-0.717689	-0.785464	-0.159691	-0.227467	-0.193579	down
A_23_P79661	CCDC93	1.7327957	1.4221907	1.6562133	-0.310605	-0.076582	-0.193594	down
A_23_P15864	MBD2	4.935854	4.6732087	4.811309	-0.262645	-0.124545	-0.193595	down
A_33_P3409904	VAMP4	-1.648121	-1.92342	-1.760071	-0.275299	-0.11195	-0.193624	down
A_23_P50990	CENPO	0.7248664	0.5392447	0.5230951	-0.185622	-0.201771	-0.193697	down
A_23_P416434	JADE2	0.2159405	-0.160306	0.204689	-0.376247	-0.011251	-0.193749	down
A_23_P144071	COL7A1	-2.481403	-2.541529	-2.808919	-0.060126	-0.327516	-0.193821	down
A_23_P157620	FAM160B2	1.1833043	0.6782632	1.3006997	-0.505041	0.1173954	-0.193823	down
A_21_P0009507	lnc-SALL3-2	-2.884049	-2.885541	-3.27041	-0.001492	-0.38636	-0.193926	down
A_23_P87742	IFFO1	-0.883975	-1.153349	-1.002594	-0.269375	-0.118619	-0.193997	down

A_24_P942630	KDM6B	-1.789163	-2.075054	-1.891422	-0.285892	-0.10226	-0.194076	down
A_21_P0014103	LOC101929947	-2.109165	-2.227147	-2.379514	-0.117982	-0.27035	-0.194166	down
A_23_P57293	URB1	2.099843	1.7943096	2.0170412	-0.305533	-0.082802	-0.194168	down
A_24_P222997	ZRANB3	0.2276669	-0.142027	0.2089586	-0.369694	-0.018708	-0.194201	down
A_23_P250118	HSPBAP1	1.1843777	1.2011089	0.7790666	0.0167313	-0.405311	-0.19429	down
A_21_P0007547	LOC100288798	-2.036277	-2.178282	-2.282853	-0.142005	-0.246576	-0.194291	down
A_33_P3343155	GNAQ	0.8424611	0.656847	0.6393905	-0.185614	-0.203071	-0.194342	down
A_32_P169735	TTC8	0.6460195	0.379715	0.5235272	-0.266305	-0.122492	-0.194398	down
A_24_P390403	RTF1	2.7970715	2.451158	2.7540712	-0.345913	-0.043	-0.194457	down
A_23_P72518	SVOP	9.088491	8.924389	8.863624	-0.164103	-0.224868	-0.194485	down
A_23_P24960	NARS2	1.4064093	1.133975	1.2897105	-0.272434	-0.116699	-0.194566	down
A_23_P125042	ZNF222	-0.920795	-1.106761	-1.12416	-0.185966	-0.203364	-0.194665	down
A_23_P68717	PSMG1	6.0744095	6.003058	5.7562904	-0.071352	-0.318119	-0.194735	down
A_21_P0004568	LUCAT1	-1.641642	-0.857769	-2.815084	0.7838731	-1.173442	-0.194784	down
A_33_P3350748	KRT7	8.91478	8.888195	8.551791	-0.026585	-0.362988	-0.194787	down
A_23_P34396	RSRP1	1.2097788	1.1042271	0.9254093	-0.105552	-0.284369	-0.194961	down
A_23_P24215	TBC1D12	0.2838092	-0.04249	0.2201505	-0.326299	-0.063659	-0.194979	down
A_22_P00017827	lnc-ZC3H12D-2	-1.716373	-1.389451	-2.43326	0.3269219	-0.716887	-0.194982	down
A_32_P195401	FAM117B	2.9370413	2.6560855	2.8278189	-0.280956	-0.109222	-0.195089	down
A_24_P340800	ZNF621	-1.061175	-1.003696	-1.508833	0.0574789	-0.447658	-0.19509	down
A_24_P231057	BOD1L1	-1.032584	-0.982755	-1.472734	0.049829	-0.44015	-0.19516	down
A_33_P3311618	RABGGTB	1.0446453	0.8546457	0.8441291	-0.19	-0.200516	-0.195258	down
A_33_P3369258	FGFR1OP2	1.6956949	1.5077453	1.4930415	-0.18795	-0.202653	-0.195302	down
A_23_P35168	ALG6	2.3499413	2.032144	2.277049	-0.317797	-0.072892	-0.195345	down
A_23_P25150	HOXC9	2.7208319	2.494267	2.556652	-0.226565	-0.16418	-0.195372	down
A_33_P3315355	SETD6	1.3979955	1.1521311	1.2529798	-0.245864	-0.145016	-0.19544	down
A_33_P3378644	PHC1	-0.554147	-0.662013	-0.83736	-0.107865	-0.283213	-0.195539	down
A_33_P3549874	LOC255177	0.7574196	0.6375303	0.4862227	-0.119889	-0.271197	-0.195543	down
A_22_P00007751	lnc-HMGB2-3	-2.504171	-2.422405	-2.977296	0.0817666	-0.473125	-0.195679	down
A_23_P67864	ADCY3	4.4687147	4.0739007	4.47178	-0.394814	0.0030651	-0.195874	down
A_23_P64669	ZCCHC8	2.714963	2.4154115	2.6221533	-0.299552	-0.09281	-0.196181	down
A_21_P0005609	KMT2E-AS1	-2.192689	-1.975659	-2.802171	0.2170296	-0.609482	-0.196226	down
A_19_P00803086	lnc-RPRML-3	0.0763607	0.0865088	-0.326306	0.010148	-0.402667	-0.196259	down
A_23_P206324	HSDL1	-0.374335	-0.676783	-0.464555	-0.302448	-0.09022	-0.196334	down
A_21_P0000344	RPL32	3.5823212	3.3633847	3.4084024	-0.218936	-0.173919	-0.196428	down
A_23_P2294	HELB	-1.426182	-1.668231	-1.577057	-0.242048	-0.150875	-0.196462	down
A_23_P325642	PDIA2	-2.389653	-2.537874	-2.634539	-0.148221	-0.244885	-0.196553	down
A_23_P209246	GLI2	-0.121257	-0.231512	-0.404166	-0.110255	-0.282908	-0.196582	down
A_33_P3272402	ADAD2	-1.773051	-2.248782	-1.690575	-0.475731	0.0824761	-0.196627	down
A_22_P00002489	lnc-C16orf45-1	-0.206094	-0.386337	-0.41936	-0.180243	-0.213265	-0.196754	down
A_19_P00317180	PVT1	0.1826081	0.0291038	-0.057561	-0.153504	-0.240169	-0.196837	down
A_33_P3401841	CTDP1	-0.70572	-0.880317	-0.924941	-0.174597	-0.219221	-0.196909	down
A_24_P345498	XRCC5	4.7388916	4.155131	4.92877	-0.583761	0.1898785	-0.196941	down
A_24_P570049	PPARA	-0.763743	-0.617497	-1.303943	0.146246	-0.5402	-0.196977	down
A_24_P170753	EP400NL	-1.692067	-1.98192	-1.796169	-0.289854	-0.104103	-0.196978	down
A_21_P0005873	lnc-KIAA1429-1	0.6911893	0.2621813	0.7262182	-0.429008	0.0350289	-0.19699	down
A_24_P103060	MCFD2	-0.996046	-0.487046	-1.899374	0.5089998	-0.903328	-0.197164	down
A_23_P205228	ATP7B	0.3252935	0.0238667	0.2322378	-0.301427	-0.093056	-0.197241	down
A_23_P208293	PVRL2	6.0994434	5.399017	6.405243	-0.700427	0.3057995	-0.197314	down
A_23_P124164	ISY1	1.6152391	1.36624	1.4692974	-0.248999	-0.145942	-0.19747	down
A_23_P124855	ZCCHC7	-0.431526	-0.809791	-0.448212	-0.378265	-0.016686	-0.197476	down
A_23_P566	FOXJ3	4.308526	4.2886834	3.9332943	-0.019843	-0.375232	-0.197537	down
A_24_P405298	PPP1CB	2.8006496	2.613215	2.5929136	-0.187435	-0.207736	-0.197585	down
A_21_P0007533	lnc-PLEKHA5-4	-2.192763	-2.200041	-2.580765	-0.007278	-0.388002	-0.19764	down
A_33_P3303542	SSC5D	-2.878602	-2.943102	-3.209456	-0.0645	-0.330854	-0.197677	down
A_24_P205130	FNBP1	-2.036768	-2.291812	-2.177204	-0.255045	-0.140436	-0.197741	down

A_23_P383688	AARS2	3.0873947	2.5811648	3.1981087	-0.50623	0.110714	-0.197758	down
A_23_P48056	CKAP4	3.170844	2.9114442	3.0347128	-0.2594	-0.136131	-0.197766	down
A_23_P164674	ZNF225	-1.227876	-1.206069	-1.645306	0.0218067	-0.41743	-0.197812	down
A_21_P0000925	lnc-SCCPDH-1	-2.438407	-2.627102	-2.645386	-0.188695	-0.206979	-0.197837	down
A_32_P140898	FOXN2	3.0587206	2.7851443	2.9365616	-0.273576	-0.122159	-0.197868	down
A_24_P200427	PAICS	5.9302855	5.6566434	5.80803	-0.273642	-0.122255	-0.197949	down
A_23_P85969	ZNF326	3.4737597	3.471724	3.0798073	-0.002036	-0.393952	-0.197994	down
A_22_P00002045	SCAMP1	-0.084132	-0.203665	-0.360771	-0.119533	-0.276639	-0.198086	down
A_23_P320242	KIAA1324L	1.9499006	1.814065	1.6894407	-0.135836	-0.26046	-0.198148	down
A_23_P21092	CALB2	1.041182	0.7483735	0.9374957	-0.292809	-0.103686	-0.198247	down
A_33_P3212027	ELF2	-0.882267	-1.017747	-1.143415	-0.13548	-0.261148	-0.198314	down
A_33_P3231187	XIAP	-0.030409	-0.212595	-0.244868	-0.182186	-0.214458	-0.198322	down
A_23_P402952	CTAGE15	-1.831001	-2.019664	-2.039365	-0.188663	-0.208364	-0.198514	down
A_33_P3320499	RAI1	-2.139915	-2.336711	-2.340219	-0.196796	-0.200304	-0.19855	down
A_24_P106591	BLOC1S5	1.8374891	1.5513401	1.7265038	-0.286149	-0.110985	-0.198567	down
A_33_P3329108	MTAP	2.6502485	2.81352	2.0895967	0.1632714	-0.560652	-0.19869	down
A_24_P175989	VPS29	3.4612074	3.1458402	3.379097	-0.315367	-0.08211	-0.198739	down
A_24_P171983	NDUFAF4	3.6683502	3.284185	3.6550379	-0.384165	-0.013312	-0.198739	down
A_23_P367628	C2CD5	1.3468299	1.2431087	1.0530329	-0.103721	-0.293797	-0.198759	down
A_23_P319792	XRN1	1.2725358	1.1254344	1.0217705	-0.147101	-0.250765	-0.198933	down
A_33_P3292332	KIF5B	4.686887	4.416962	4.558708	-0.269925	-0.128179	-0.199052	down
A_24_P286465	PURB	3.9579926	3.56152	3.9563599	-0.396472	-0.001633	-0.199053	down
A_23_P163353	PIAS1	0.2960782	0.0599237	0.1340747	-0.236155	-0.162004	-0.199079	down
A_33_P3407256	TRIP13	-0.407569	-0.622192	-0.591116	-0.214623	-0.183547	-0.199085	down
A_33_P3355408	STAU2-AS1	-2.130498	-2.526958	-2.132246	-0.396461	-0.001749	-0.199105	down
A_21_P0010430	lnc-MAPK8IP2-1	-2.34977	-2.808826	-2.289043	-0.459056	0.0607266	-0.199165	down
A_23_P70719	LAMA2	-2.734269	-2.868321	-2.998612	-0.134052	-0.264343	-0.199197	down
A_24_P44596	MON1B	3.2086134	2.7930589	3.2255573	-0.415555	0.0169439	-0.199305	down
A_23_P76159	EEA1	1.0743632	0.9566894	0.7934237	-0.117674	-0.28094	-0.199307	down
A_33_P3345504	RC3H2	0.361249	0.1991534	0.1246023	-0.162096	-0.236647	-0.199371	down
A_23_P359616	RPF1	4.066435	3.8513265	3.882636	-0.215108	-0.183799	-0.199454	down
A_23_P134125	MAP3K5	2.3210154	2.0450034	2.198	-0.276012	-0.123015	-0.199514	down
A_22_P00021104	lnc-IL23A-2	0.3308635	0.0345225	0.2281489	-0.296341	-0.102715	-0.199528	down
A_24_P351304	IMMT	1.7429819	1.6334343	1.4532557	-0.109548	-0.289726	-0.199637	down
A_23_P20002	ANKMY2	-0.478057	-0.689762	-0.665711	-0.211704	-0.187654	-0.199679	down
A_33_P3232277	CCNG1	0.0969129	-0.118277	-0.087293	-0.21519	-0.184206	-0.199698	down
A_23_P22350	GRAMD3	1.5787706	1.3560433	1.4020948	-0.222727	-0.176676	-0.199702	down
A_24_P374382	TOP1P2	1.030767	0.7526417	0.9093614	-0.278125	-0.121406	-0.199765	down
A_33_P3405168	MMAA	-1.405877	-1.574022	-1.63749	-0.168145	-0.231614	-0.199879	down
A_24_P416370	HOXB4	-2.13287	-2.296688	-2.369109	-0.163818	-0.23624	-0.200029	down
A_33_P3840512	SLC25A15	1.3400612	1.0154557	1.264431	-0.324605	-0.07563	-0.200118	down
A_23_P66854	KRT20	2.655983	2.144009	2.7676964	-0.511974	0.1117134	-0.20013	down
A_23_P129058	USP8	1.2427554	1.0350232	1.0501027	-0.207732	-0.192653	-0.200192	down
A_23_P36531	TSPAN8	4.5430946	4.55487	4.130472	0.0117755	-0.412622	-0.200423	down
A_22_P00023571	lnc-RP11-677C1.2.1-1	-1.305768	-1.202694	-1.809776	0.1030731	-0.504008	-0.200468	down
A_32_P75792	FAM132A	-1.604647	-1.604978	-2.005446	-0.00033	-0.400799	-0.200565	down
A_23_P218731	CRYZL1	0.0147066	-0.241382	-0.130397	-0.256088	-0.145103	-0.200596	down
A_22_P00011014	lnc-NSUN6-3	-2.052109	-2.273566	-2.231851	-0.221457	-0.179742	-0.2006	down
A_24_P101786	THADA	-1.609307	-1.893041	-1.726852	-0.283734	-0.117545	-0.20064	down
A_33_P3226039	TGFBRAP1	0.7382264	0.6898046	0.3851562	-0.048422	-0.35307	-0.200746	down
A_23_P79572	MGC16025	-2.498442	-2.564549	-2.833944	-0.066108	-0.335502	-0.200805	down
A_21_P0000388	SNORD91A	-1.050723	-1.286734	-1.2166	-0.236011	-0.165876	-0.200944	down
A_23_P137532	PLOD1	1.3125653	0.9565077	1.2666593	-0.356058	-0.045906	-0.200982	down
A_23_P379327	FAM63B	2.801302	2.5846782	2.6159372	-0.216624	-0.185365	-0.200994	down
A_23_P417282	IGF1R	1.195714	1.0841455	0.9052277	-0.111568	-0.290486	-0.201027	down

A_33_P3402725	ASH1L	-0.060502	-0.303247	-0.219858	-0.242744	-0.159356	-0.20105	down
A_23_P156443	KPNA5	-2.274302	-2.471889	-2.47898	-0.197587	-0.204678	-0.201133	down
A_21_P0012873	LOC100507387	-1.637435	-1.711794	-1.965508	-0.074359	-0.328072	-0.201216	down
A_33_P3357062	DDX55	2.866006	2.5072675	2.8222952	-0.358738	-0.043711	-0.201225	down
A_23_P99762	RBM25	2.9999685	2.7596593	2.8378048	-0.240309	-0.162164	-0.201236	down
A_21_P0004921	LOC102724646	-0.753936	-0.992406	-0.917958	-0.23847	-0.164022	-0.201246	down
A_32_P163739	KIAA1257	-2.143249	-2.297392	-2.391845	-0.154143	-0.248596	-0.201369	down
A_33_P3334225	ACACA	5.7978077	5.3465877	5.8462467	-0.45122	0.048439	-0.201391	down
A_33_P3371910	HNRNPLL	0.5702429	0.3384209	0.3991594	-0.231822	-0.171083	-0.201453	down
A_23_P71513	EFR3A	4.6310253	4.546849	4.3121824	-0.084177	-0.318843	-0.20151	down
A_23_P111961	MAK16	2.5708303	2.3784947	2.3601227	-0.192336	-0.210708	-0.201522	down
A_23_P305877	TMEM261	-1.683626	-1.918141	-1.852342	-0.234515	-0.168715	-0.201615	down
A_23_P162807	MRPS31	4.546274	4.111116	4.5780363	-0.435158	0.0317621	-0.201698	down
A_22_P00013705	ZFPM2-AS1	1.2321959	1.1604156	0.9004092	-0.07178	-0.331787	-0.201783	down
A_33_P3236656	SPACA4	-0.425538	-0.680253	-0.574627	-0.254715	-0.14909	-0.201903	down
A_23_P422083	TMEM55A	2.4966106	2.170772	2.418521	-0.325839	-0.07809	-0.201964	down
A_21_P0007484	LINC00941	-0.789849	-1.172992	-0.810639	-0.383143	-0.02079	-0.201967	down
A_32_P100258	FLJ37453	0.6617127	0.3742309	0.5451727	-0.287482	-0.11654	-0.202011	down
A_32_P226205	ZFHX2	-1.703245	-1.730706	-2.079866	-0.027461	-0.376621	-0.202041	down
A_24_P158903	IRAK4	-1.27021	-1.457409	-1.487246	-0.187199	-0.217036	-0.202117	down
A_24_P12539	KBTBD2	1.1728048	1.0292969	0.9119597	-0.143508	-0.260845	-0.202177	down
A_33_P3340224	JAZF1-AS1	-2.072352	-2.095712	-2.453565	-0.02336	-0.381213	-0.202287	down
A_22_P00001368	lnc-AP002478.1-2	-1.54429	-1.180013	-2.313292	0.3642764	-0.769002	-0.202363	down
A_23_P95594	NAT1	0.6829877	0.3423767	0.6188288	-0.340611	-0.064159	-0.202385	down
A_33_P3223759	MED13	3.8191051	3.6749244	3.5580301	-0.144181	-0.261075	-0.202628	down
A_23_P373054	TCAIM	0.2491946	0.2550774	-0.162014	0.0058827	-0.411208	-0.202663	down
A_33_P3265180	LOC400927-CSNK1E	0.6713328	0.5082021	0.4291182	-0.163131	-0.242215	-0.202673	down
A_23_P348383	CC2D2A	0.1445775	0.0190315	-0.135233	-0.125546	-0.279811	-0.202678	down
A_33_P3231653	GOLM1	3.103263	2.9570603	2.8440561	-0.146203	-0.259207	-0.202705	down
A_33_P3244424	SNRPE	-0.903574	-1.289637	-0.922936	-0.386063	-0.019362	-0.202712	down
A_21_P0013421	XLOC_l2_013808	-0.638367	-0.779266	-0.902947	-0.140899	-0.26458	-0.202739	down
A_23_P130735	SLC6A16	-1.184043	-1.189916	-1.583874	-0.005873	-0.399831	-0.202852	down
A_23_P319423	KCNK5	3.985879	3.6565413	3.9093761	-0.329338	-0.076503	-0.20292	down
A_33_P3361707	MDM2	-0.163344	-0.356342	-0.376318	-0.192998	-0.212974	-0.202986	down
A_33_P3256113	GUSBP1	-2.628811	-2.900876	-2.762729	-0.272065	-0.133918	-0.202991	down
A_23_P387000	XKR6	-1.631812	-2.078521	-1.591089	-0.446709	0.0407224	-0.202993	down
A_23_P121250	EIF4A2	5.46418	5.2642217	5.258113	-0.199958	-0.206067	-0.203013	down
A_24_P358245	ATP8B5P	0.1468029	0.0043912	-0.116841	-0.142412	-0.263644	-0.203028	down
A_22_P00014427	lnc-SGSH-1	-2.701214	-2.976786	-2.831935	-0.275572	-0.130721	-0.203147	down
A_33_P3343485	HIP1	-0.685742	-0.750308	-1.027769	-0.064566	-0.342026	-0.203296	down
A_23_P37283	CGRRF1	-0.172533	-0.433291	-0.318432	-0.260758	-0.1459	-0.203329	down
A_23_P52676	CATSPER1	-1.401683	-1.696384	-1.513672	-0.294701	-0.111989	-0.203345	down
A_33_P3422581	WDPCP	-1.812686	-2.222351	-1.809741	-0.409665	0.0029454	-0.20336	down
A_21_P0014273	CYP2R1	-1.839468	-1.985049	-2.100771	-0.145582	-0.261303	-0.203443	down
A_33_P3400828	NXNL2	-2.291503	-2.632608	-2.357354	-0.341105	-0.065851	-0.203478	down
A_24_P943095	SPECC1	-1.320375	-1.479638	-1.568073	-0.159262	-0.247698	-0.20348	down
A_33_P3255354	NT5C2	-0.277018	-0.606695	-0.354357	-0.329677	-0.077339	-0.203508	down
A_33_P3383871	FEM1C	1.5873337	1.247715	1.5198488	-0.339619	-0.067485	-0.203552	down
A_33_P3307500	STRA6	1.6668348	1.3878207	1.5384221	-0.279014	-0.128413	-0.203713	down
A_33_P3329098	WTAP	0.9149795	0.9109621	0.5114188	-0.004017	-0.403561	-0.203789	down
A_23_P68868	TUG1	5.7996445	5.5225635	5.6690826	-0.277081	-0.130562	-0.203821	down
A_23_P31414	ORC5	2.7318916	2.396051	2.6599722	-0.335841	-0.071919	-0.20388	down
A_21_P0000841	PRKAG2-AS1	-0.185539	-0.611107	-0.167752	-0.425568	0.017787	-0.203891	down
A_33_P3212188	LRP3	-2.634093	-3.102575	-2.573479	-0.468483	0.0606141	-0.203934	down
A_23_P93750	LSM5	5.667178	5.426952	5.499218	-0.240226	-0.16796	-0.204093	down

A_23_P210747	TRMT6	2.4636116	2.1798081	2.3390665	-0.283803	-0.124545	-0.204174	down
A_32_P138617	KIAA2018	0.5424399	0.3950458	0.2813563	-0.147394	-0.261084	-0.204239	down
A_23_P106145	ERO1L	3.9754171	3.8700576	3.6722784	-0.10536	-0.303139	-0.204249	down
A_23_P120335	MTX2	3.8174477	3.5042577	3.7220154	-0.31319	-0.095432	-0.204311	down
A_24_P71938	SMAD1	2.9094086	2.4542775	2.9558477	-0.455131	0.0464392	-0.204346	down
A_32_P69465	MORN2	3.1997385	2.9116015	3.0791826	-0.288137	-0.120556	-0.204346	down
A_24_P924591	VEZF1	3.724061	3.5173154	3.5219069	-0.206746	-0.202154	-0.20445	down
A_33_P3255647	RPL36A-HNRNPH2	1.2418561	1.0679765	1.0067453	-0.17388	-0.235111	-0.204495	down
A_24_P588897	SLCO3A1	0.5529599	0.2867923	0.4099503	-0.266168	-0.14301	-0.204589	down
A_33_P3423610	SEP15	4.143506	3.942841	3.9349546	-0.200665	-0.208551	-0.204608	down
A_23_P356004	KCNIP3	-0.417906	-1.074482	-0.170766	-0.656576	0.2471395	-0.204718	down
A_23_P427148	PROCA1	-2.016154	-2.074224	-2.367526	-0.05807	-0.351372	-0.204721	down
A_23_P68922	MICALL1	-0.486403	-1.081489	-0.300779	-0.595086	0.1856237	-0.204731	down
A_33_P3309984	KHK	-0.800484	-1.163241	-0.847195	-0.362758	-0.046711	-0.204735	down
A_33_P3338698	IHH	-2.046321	-2.253032	-2.249086	-0.20671	-0.202764	-0.204737	down
A_33_P3326672	ZNF782	-1.30987	-1.695273	-1.334085	-0.385404	-0.024215	-0.204809	down
A_24_P29401	PIK3R1	2.4243956	2.4104295	2.028369	-0.013966	-0.396027	-0.204996	down
A_22_P00003853	CEBPA-AS1	0.7870812	0.4818983	0.6821971	-0.305183	-0.104884	-0.205034	down
A_23_P348323	PCGF2	0.1164618	-0.093017	-0.084526	-0.209479	-0.200987	-0.205233	down
A_21_P0014257	STK32C	-1.759391	-2.049	-1.880344	-0.289609	-0.120953	-0.205281	down
A_33_P3371493	TOP1	1.4348559	1.1532731	1.3058438	-0.281583	-0.129012	-0.205297	down
A_33_P3397399	PPP3CB	-0.325696	-0.319547	-0.742567	0.0061483	-0.416872	-0.205362	down
A_23_P101048	KRTAP3-2	-2.210091	-2.302327	-2.528631	-0.092236	-0.31854	-0.205388	down
A_21_P0009119	lnc-SPIRE2-1	0.8842025	0.6825662	0.6749969	-0.201636	-0.209206	-0.205421	down
A_23_P150979	MUCL1	4.4979057	4.204635	4.380207	-0.293271	-0.117699	-0.205485	down
A_23_P200741	DPT	-1.497695	-1.595696	-1.810719	-0.098002	-0.313025	-0.205513	down
A_24_P309594	SLC48A1	3.9153423	3.379764	4.039665	-0.535578	0.1243229	-0.205628	down
A_23_P207811	PAIP1	3.6875124	3.6020923	3.361434	-0.08542	-0.326078	-0.205749	down
A_33_P3408943	MCMBP	5.1158953	4.8782516	4.942004	-0.237644	-0.173891	-0.205767	down
A_19_P00322236	ZNF124	-1.020477	-1.216762	-1.235988	-0.196285	-0.215511	-0.205898	down
A_32_P100439	MTURN	2.0696573	2.0720239	1.6554861	0.0023665	-0.414171	-0.205902	down
A_32_P190049	LRRC58	0.0481434	-0.154418	-0.161169	-0.202562	-0.209312	-0.205937	down
A_32_P45493	SRSF10	4.1890965	3.7981334	4.16813	-0.390963	-0.020967	-0.205965	down
A_24_P308029	HSPB6	-2.294692	-2.636232	-2.365226	-0.341541	-0.070535	-0.206038	down
A_33_P3379391	H2AFJ	-2.226018	-2.178634	-2.685545	0.047384	-0.459527	-0.206071	down
A_33_P3293207	C8orf37	0.7647786	0.4659958	0.651341	-0.298783	-0.113438	-0.20611	down
A_21_P0009993	LOC100505771	-0.421825	-0.792384	-0.463555	-0.370559	-0.04173	-0.206144	down
A_23_P301360	ZNF572	1.0143647	0.9082418	0.708107	-0.106123	-0.306258	-0.20619	down
A_33_P3350374	FAM213A	2.5151434	2.1883159	2.4295769	-0.326828	-0.085567	-0.206197	down
A_24_P23411	ARMCX3	-0.644866	-0.639081	-1.063071	0.005785	-0.418206	-0.20621	down
A_23_P159775	GABRE	-2.057338	-2.205948	-2.321158	-0.148611	-0.26382	-0.206215	down
A_33_P3267195	KU-MEL-3	-0.271947	-0.413134	-0.543201	-0.141187	-0.271254	-0.20622	down
A_24_P269062	SPRY4	-0.225101	-0.77244	-0.090304	-0.547338	0.1347971	-0.20627	down
A_23_P143190	MYBL2	2.2149172	1.8889318	2.1280842	-0.325985	-0.086833	-0.206409	down
A_33_P3368610	PPHLN1	2.5627117	2.2535615	2.4588938	-0.30915	-0.103818	-0.206484	down
A_23_P410587	JADE1	0.7280073	0.5837436	0.4591837	-0.144264	-0.268824	-0.206544	down
A_23_P121716	ANXA3	6.175543	6.0250373	5.912876	-0.150506	-0.262667	-0.206586	down
A_33_P3382157	PICALM	5.561634	5.2029386	5.506914	-0.358696	-0.05472	-0.206708	down
A_21_P0010051	lnc-PREX1-3	-1.858363	-1.982592	-2.147572	-0.124228	-0.289209	-0.206719	down
A_33_P3298785	CUX1	-1.094261	-1.466814	-1.135332	-0.372553	-0.041071	-0.206812	down
A_24_P360078	LRBA	3.1692266	3.0268502	2.897461	-0.142376	-0.271766	-0.207071	down
A_23_P114405	MORF4L2	4.5980244	4.3737006	4.408066	-0.224324	-0.189959	-0.207141	down
A_24_P18621	AEBP2	2.578085	2.3316894	2.410077	-0.246396	-0.168008	-0.207202	down
A_23_P87329	NAT10	2.3001919	1.884665	2.3010616	-0.415527	0.0008698	-0.207329	down
A_23_P101392	TMEM38A	1.0945883	0.7955313	0.9789434	-0.299057	-0.115645	-0.207351	down

A_23_P137504	ZBTB37	-2.678416	-3.107328	-2.664279	-0.428912	0.014137	-0.207388	down
A_23_P301925	COX1	8.694934	8.91004	8.065032	0.215106	-0.629902	-0.207398	down
A_23_P37127	FOXA1	1.8641624	1.6752782	1.6381626	-0.188884	-0.226	-0.207442	down
A_23_P13359	NXF1	2.5825357	2.2654185	2.4847507	-0.317117	-0.097785	-0.207451	down
A_23_P410859	ZSWIM6	0.7612205	0.5960426	0.511322	-0.165178	-0.249898	-0.207538	down
A_23_P46333	MTF2	2.766842	2.7322268	2.3860054	-0.034615	-0.380837	-0.207726	down
A_23_P108761	CNOT11	2.7628374	2.7743344	2.3357897	0.011497	-0.427048	-0.207775	down
A_23_P129075	WDR76	-0.779032	-1.073155	-0.900498	-0.294123	-0.121467	-0.207795	down
A_23_P97457	AIDA	-1.163696	-1.388983	-1.354043	-0.225287	-0.190347	-0.207817	down
A_24_P149395	WIPI2	1.5941763	1.1603713	1.6121483	-0.433805	0.017972	-0.207917	down
A_33_P3347482	LACTBL1	-2.332222	-2.521535	-2.55877	-0.189313	-0.226549	-0.207931	down
A_33_P3245348	KRTAP4-1	-0.748286	-1.434878	-0.477582	-0.686592	0.2707038	-0.207944	down
A_23_P164946	FKRP	0.8881912	0.5182996	0.841938	-0.369892	-0.046253	-0.208072	down
A_33_P3377130	MAP3K5	-0.385197	-0.393708	-0.792899	-0.008511	-0.407702	-0.208107	down
A_32_P31182	RPL7	6.364339	6.2870812	6.025358	-0.077258	-0.338981	-0.208119	down
A_21_P0004274	lnc-RASA1-3	1.2585125	1.0578527	1.0428123	-0.20066	-0.2157	-0.20818	down
A_33_P3240757	IGSF3	-2.075667	-2.168387	-2.39942	-0.09272	-0.323752	-0.208236	down
A_23_P251232	TTTY14	1.6929226	1.404973	1.5642905	-0.28795	-0.128632	-0.208291	down
A_32_P93187	SMARCA5	0.4131398	0.2067657	0.2029223	-0.206374	-0.210217	-0.208296	down
A_22_P00005074	lnc-DES-1	-2.676738	-2.791215	-2.979011	-0.114478	-0.302274	-0.208376	down
A_23_P306933	PPTC7	2.941967	2.7701	2.6969223	-0.171867	-0.245045	-0.208456	down
A_23_P21363	AHNAK	5.798196	5.344991	5.833935	-0.453205	0.0357389	-0.208733	down
A_23_P390116	SPATA13	1.2979321	1.188518	0.9897404	-0.109414	-0.308192	-0.208803	down
A_24_P80776	SMIM13	-2.722793	-3.090375	-2.772842	-0.367581	-0.050048	-0.208815	down
A_33_P3359047	LYPD6	-1.020948	-1.495977	-0.963608	-0.47503	0.0573392	-0.208845	down
A_33_P3250845	NIPA2	5.3836308	5.0440335	5.3054914	-0.339597	-0.078139	-0.208868	down
A_23_P140154	TMEM251	0.7798004	0.3998103	0.7419586	-0.37999	-0.037842	-0.208916	down
A_21_P0000725	SAMD12-AS1	-2.355409	-2.932508	-2.196343	-0.577099	0.159066	-0.209017	down
A_24_P210637	CHST14	-2.209735	-2.22433	-2.613213	-0.014595	-0.403477	-0.209036	down
A_23_P112201	KDM4C	1.7833624	1.6490846	1.4995446	-0.134278	-0.283818	-0.209048	down
A_32_P153892	TAF3	2.1224937	1.8780155	1.9488716	-0.244478	-0.173622	-0.20905	down
A_23_P108823	OSBPL6	-1.404732	-1.535042	-1.692775	-0.13031	-0.288043	-0.209177	down
A_23_P426636	AHNAK	2.6727705	2.7365737	2.1905909	0.0638032	-0.48218	-0.209188	down
A_23_P52336	UNC5B	3.65448	3.2021527	3.6883583	-0.452327	0.0338783	-0.209224	down
A_21_P0012276	SLC25A15	0.9713187	0.7061276	0.8179984	-0.265191	-0.15332	-0.209256	down
A_24_P310630	UPF3B	1.0518994	0.8533444	0.831872	-0.198555	-0.220027	-0.209291	down
A_23_P204879	CAB39L	1.698988	1.5635028	1.4158754	-0.135485	-0.283113	-0.209299	down
A_23_P63153	DDX20	3.4274626	3.1468825	3.2894325	-0.28058	-0.13803	-0.209305	down
A_23_P14184	THSD1	1.6892767	1.503056	1.45683	-0.186221	-0.232447	-0.209334	down
A_24_P106357	WDR36	0.5852356	0.1727114	0.5790734	-0.412524	-0.006162	-0.209343	down
A_32_P205637	PARD6B	1.2717123	1.119524	1.0051684	-0.152188	-0.266544	-0.209366	down
A_19_P00807053	LOC100506411	1.6975288	1.439704	1.5363398	-0.257825	-0.161189	-0.209507	down
A_22_P00007442	GSTM5	-0.757679	-0.920508	-1.013868	-0.16283	-0.25619	-0.20951	down
A_23_P139575	PRR13	7.070759	7.299689	6.422777	0.22893	-0.647982	-0.209526	down
A_32_P109522	ZUFSP	1.8604794	1.50242	1.7993836	-0.358059	-0.061096	-0.209578	down
A_24_P396994	ATP6V1A	5.5238323	5.298742	5.3297586	-0.225091	-0.194074	-0.209582	down
A_24_P252130	PPARD	4.098898	3.3892245	4.389182	-0.709673	0.2902842	-0.209695	down
A_33_P3287680	SMCO1	-2.40867	-2.531363	-2.705379	-0.122693	-0.296709	-0.209701	down
A_33_P3361611	PLA2G4D	-2.943602	-2.962279	-3.344883	-0.018677	-0.401281	-0.209979	down
A_33_P3371514	IPPK	0.2149468	0.1877494	-0.177817	-0.027197	-0.392764	-0.20998	down
A_33_P3415092	CLCN5	1.2853723	1.26404	0.8867402	-0.021332	-0.398632	-0.209982	down
A_24_P247536	FAM133B	1.084682	0.7985573	0.9507051	-0.286125	-0.133977	-0.210051	down
A_32_P60223	ING5	1.6805601	1.5494971	1.3914852	-0.131063	-0.289075	-0.210069	down
A_33_P3343785	MGME1	5.1750765	4.9357104	4.9941235	-0.239366	-0.180953	-0.21016	down
A_32_P91902	TSPAN14	2.7522707	2.3133025	2.7706385	-0.438968	0.0183678	-0.2103	down
A_32_P44512	ST13	4.352068	3.9426904	4.3407774	-0.409378	-0.011291	-0.210334	down

A_23_P217778	MSL3	2.0060234	1.9229398	1.6683917	-0.083084	-0.337632	-0.210358	down
A_24_P26073	LRPPRC	4.4434376	4.259958	4.206195	-0.18348	-0.237243	-0.210361	down
A_33_P3363515	XRCC3	8.686723	8.516542	8.436054	-0.17018	-0.250669	-0.210424	down
A_23_P79360	NOSTRIN	2.580143	2.2984643	2.4408894	-0.281679	-0.139254	-0.210466	down
A_32_P129968	ZNF284	1.8086061	1.6144667	1.5816565	-0.194139	-0.22695	-0.210545	down
A_33_P3424062	KCNF1	-2.680491	-2.933351	-2.848768	-0.252859	-0.168277	-0.210568	down
A_33_P3367361	FAM107B	2.218975	2.2304082	1.786262	0.0114331	-0.432713	-0.21064	down
A_33_P3445679	LINC00548	-1.480288	-1.524261	-1.857637	-0.043972	-0.377349	-0.210661	down
A_23_P171054	ELK1	-0.80963	-0.962003	-1.07859	-0.152373	-0.26896	-0.210667	down
A_23_P202860	CYP2R1	2.2118902	2.0509295	1.9514542	-0.160961	-0.260436	-0.210698	down
A_33_P3296308	DCAF16	3.2939482	2.8458467	3.3205318	-0.448102	0.0265837	-0.210759	down
A_21_P0000103	MTRNR2L3	1.7808027	1.6295514	1.5104833	-0.151251	-0.270319	-0.210785	down
A_23_P128375	FAM222A	1.4289846	1.1785731	1.2576714	-0.250412	-0.171313	-0.210862	down
A_24_P370887	VAMP3	2.404935	1.8457837	2.5423326	-0.559151	0.1373978	-0.210877	down
A_33_P3210139	PCF11	2.406868	2.2167058	2.1752367	-0.190162	-0.231631	-0.210897	down
A_21_P0008626	OIP5-AS1	1.6711578	1.3375182	1.5830002	-0.33364	-0.088158	-0.210899	down
A_21_P0011909	XLOC_l2_007967	-2.829214	-2.887416	-3.192881	-0.058202	-0.363667	-0.210934	down
A_22_P00000738	lnc-ADAR-1	-2.312024	-2.41978	-2.626185	-0.107756	-0.31416	-0.210958	down
A_23_P22444	CFP	-0.123229	-0.299865	-0.368642	-0.176636	-0.245414	-0.211025	down
A_32_P305888	SH3TC2	-0.890961	-1.18332	-1.021005	-0.292358	-0.130044	-0.211201	down
A_22_P00002715	MIR646HG	-2.283191	-2.641822	-2.347069	-0.358631	-0.063878	-0.211254	down
A_23_P83931	NET1	4.7784004	4.5339437	4.6002674	-0.244457	-0.178133	-0.211295	down
A_33_P3293114	SRSF11	4.5237894	4.25981	4.365015	-0.263979	-0.158774	-0.211377	down
A_23_P129801	RAB40B	1.7564707	1.2540393	1.8361311	-0.502431	0.0796604	-0.211385	down
A_24_P943106	U2SURP	0.1942854	0.1031456	-0.137417	-0.09114	-0.331702	-0.211421	down
A_24_P289383	CHD7	1.5837259	1.7700391	0.9745646	0.1863132	-0.609161	-0.211424	down
A_22_P00023587	CDK3	3.1722174	2.8916183	3.0299578	-0.280599	-0.14226	-0.211429	down
A_23_P377819	SRSF5	4.9124727	4.707392	4.6945353	-0.205081	-0.217937	-0.211509	down
A_22_P00008083	LOC101929761	-2.577674	-2.739654	-2.838725	-0.161981	-0.261051	-0.211516	down
A_23_P300056	CDC42	-2.34649	-2.90895	-2.207256	-0.562459	0.1392343	-0.211612	down
A_23_P320878	METTL21B	-0.217072	-0.363167	-0.494297	-0.146095	-0.277224	-0.211659	down
A_24_P291426	REV1	0.4177122	0.1005507	0.3114142	-0.317162	-0.106298	-0.21173	down
A_24_P333802	MAGT1	5.0373707	4.819301	4.8318863	-0.21807	-0.205484	-0.211777	down
A_21_P0011251	XLOC_l2_004213	-2.045423	-2.645042	-1.869501	-0.599619	0.1759219	-0.211848	down
A_23_P343935	EGLN1	4.104065	3.655253	4.129101	-0.448812	0.0250359	-0.211888	down
A_24_P282751	SUPT7L	-1.971091	-2.314286	-2.05175	-0.343194	-0.080659	-0.211927	down
A_23_P137765	PAFAH2	-2.358116	-2.572352	-2.567879	-0.214236	-0.209763	-0.212	down
A_33_P3243857	ADAM10	3.6750154	3.4206138	3.5053444	-0.254402	-0.169671	-0.212036	down
A_33_P3393573	PCMT1	3.701931	3.3453774	3.6342077	-0.356554	-0.067723	-0.212138	down
A_23_P209740	PSMD1	6.823947	6.3758044	6.8476534	-0.448143	0.0237064	-0.212218	down
A_32_P74366	VCPIP1	-0.487311	-0.745135	-0.653957	-0.257824	-0.166646	-0.212235	down
A_23_P39517	SRBD1	1.298037	1.1029501	1.068543	-0.195087	-0.229494	-0.212291	down
A_19_P00318883	APTR	2.327487	2.09903	2.131361	-0.228457	-0.196126	-0.212291	down
A_23_P502425	MRPL47	6.2627068	5.98768	6.1129093	-0.275027	-0.149797	-0.212412	down
A_24_P356406	PAFAH1B1	3.5353231	3.341309	3.3045053	-0.194014	-0.230818	-0.212416	down
A_23_P107644	SNRPD1	7.509939	7.28947	7.3052835	-0.220469	-0.204656	-0.212562	down
A_33_P3250133	VSIG10	0.2919641	0.2020788	-0.043385	-0.089885	-0.335349	-0.212617	down
A_24_P331373	GUCD1	-1.097224	-1.012282	-1.607457	0.0849419	-0.510233	-0.212646	down
A_23_P217899	CCNL2	-0.105882	-0.456374	-0.180721	-0.350492	-0.074839	-0.212666	down
A_24_P66027	APOBEC3B	2.7425785	2.8121133	2.2476997	0.0695348	-0.494879	-0.212672	down
A_23_P305507	TOP1	1.5817509	1.4000597	1.3380947	-0.181691	-0.243656	-0.212674	down
A_32_P154830	OSTM1	-0.952041	-1.31456	-1.014906	-0.362519	-0.062865	-0.212692	down
A_23_P1056	GPR89B	1.7048712	1.5773411	1.4069147	-0.12753	-0.297956	-0.212743	down
A_23_P160354	AKT3	-0.654016	-1.048329	-0.685237	-0.394314	-0.031221	-0.212768	down
A_22_P00015054	lnc-SNPH-1	-1.65976	-2.138247	-1.607222	-0.478487	0.0525374	-0.212975	down
A_24_P238543	PHKA1	-1.699985	-2.130737	-1.695216	-0.430753	0.0047684	-0.212992	down

A_23_P146111	NIPAL2	-1.545697	-2.101474	-1.416008	-0.555778	0.1296892	-0.213044	down
A_24_P319736	MEIS1	-0.571464	-0.73641	-0.832609	-0.164947	-0.261146	-0.213046	down
A_32_P196047	DPY19L4	2.3678608	2.2739234	2.0356083	-0.093937	-0.332253	-0.213095	down
A_24_P935103	ADCY9	1.1372738	0.4123492	1.4359698	-0.724925	0.298696	-0.213114	down
A_23_P205007	IPO5	3.1382914	3.0260139	2.824216	-0.112278	-0.314075	-0.213176	down
A_23_P152284	SNRNP25	4.46614	3.9027476	4.6031685	-0.563392	0.1370287	-0.213182	down
A_24_P63799	QRSL1	-2.287199	-2.62541	-2.37537	-0.338212	-0.088171	-0.213191	down
A_21_P0006672	lnc-ZNF37A-3	-2.868122	-3.009425	-3.153441	-0.141303	-0.285319	-0.213311	down
A_24_P183292	CSNK1A1	0.7622585	0.587069	0.51055	-0.17519	-0.251709	-0.213449	down
A_21_P0013225	XLOC_l2_013458	-0.079322	-0.348357	-0.237232	-0.269035	-0.15791	-0.213472	down
A_22_P00004358	LOC101927391	-0.232541	-0.628936	-0.263144	-0.396395	-0.030602	-0.213499	down
A_24_P229531	NABP1	0.6730971	0.6767588	0.2422524	0.0036616	-0.430845	-0.213592	down
A_23_P88740	CENPN	4.776947	4.4279714	4.698701	-0.348976	-0.078246	-0.213611	down
A_23_P25097	LLPH	3.079053	2.7149577	3.0158978	-0.364095	-0.063155	-0.213625	down
A_23_P380998	R3HDM1	2.295082	2.036141	2.1266994	-0.258941	-0.168383	-0.213662	down
A_33_P3358228	VWA2	2.6172	2.2677207	2.5393515	-0.349479	-0.077848	-0.213664	down
A_24_P6517	PLEKHG1	1.5889349	1.4001365	1.3502331	-0.188798	-0.238702	-0.21375	down
A_32_P112493	PKDCC	-2.463963	-2.967932	-2.387767	-0.503969	0.0761955	-0.213887	down
A_33_P3367201	MMAB	0.6710892	0.0870252	0.8268466	-0.584064	0.1557574	-0.214153	down
A_22_P00008012	SEMA3B-AS1	-0.940702	-0.99041	-1.319366	-0.049708	-0.378663	-0.214185	down
A_24_P132518	IKBKB	1.3194041	0.9990602	1.2113366	-0.320344	-0.108068	-0.214206	down
A_22_P00022587	lnc-NUMB-2	-0.461066	-0.73325	-0.617429	-0.272184	-0.156363	-0.214273	down
A_24_P294703	PRRC1	0.1879334	-0.041395	-0.011434	-0.229328	-0.199368	-0.214348	down
A_33_P3261565	ZRANB1	-1.008756	-1.228634	-1.217706	-0.219878	-0.20895	-0.214414	down
A_24_P137897	IFRD1	0.785706	0.8108654	0.3316703	0.0251594	-0.454036	-0.214438	down
A_33_P3317618	SYN2	0.8724289	0.6413903	0.6741972	-0.231039	-0.198232	-0.214635	down
A_22_P00015573	lnc-STT3B-1	0.00457	-0.330882	-0.089311	-0.335452	-0.093881	-0.214666	down
A_33_P3674851	VPS36	2.2402124	1.9005961	2.15032	-0.339616	-0.089892	-0.214754	down
A_33_P3219256	BMPR1A	2.9456787	2.7709813	2.690712	-0.174697	-0.254967	-0.214832	down
A_21_P0000054	PET117	4.0939455	3.816711	3.9413824	-0.277235	-0.152563	-0.214899	down
A_23_P78410	TIMM21	2.5506935	2.2027354	2.4687777	-0.347958	-0.081916	-0.214937	down
A_33_P3759592	FLJ13773	9.400746	9.197205	9.174145	-0.203542	-0.226602	-0.215072	down
A_24_P179044	SNX9	-0.698783	-0.878343	-0.949492	-0.17956	-0.25071	-0.215135	down
A_23_P96990	NVL	4.456645	4.197783	4.2852325	-0.258862	-0.171412	-0.215137	down
A_33_P3414202	MBNL3	-0.404131	-0.755835	-0.482705	-0.351704	-0.078574	-0.215139	down
A_23_P386411	PPID	4.7663307	4.466787	4.635586	-0.299544	-0.130745	-0.215144	down
A_23_P135157	PLAA	1.4935102	1.2781701	1.278255	-0.21534	-0.215255	-0.215298	down
A_24_P302574	OSGEPL1	0.4297895	0.0191917	0.40978	-0.410598	-0.02001	-0.215304	down
A_23_P132915	FAM114A1	-1.073421	-1.332072	-1.245431	-0.258651	-0.17201	-0.215331	down
A_24_P261259	PFKFB3	1.9726639	1.7051029	1.8093424	-0.267561	-0.163322	-0.215441	down
A_33_P3351944	EGFR	-1.068805	-1.382056	-1.186511	-0.313251	-0.117706	-0.215478	down
A_23_P214897	AKAP12	0.8705387	0.5880032	0.7220531	-0.282536	-0.148486	-0.215511	down
A_23_P99853	FAM214A	1.6855869	1.701302	1.2387252	0.0157151	-0.446862	-0.215573	down
A_33_P3412353	ZNF268	2.164524	2.0163302	1.8811188	-0.148194	-0.283405	-0.2158	down
A_33_P3393806	ATP13A2	-1.087013	-1.441505	-1.164204	-0.354492	-0.077191	-0.215841	down
A_33_P3228862	LRRC69	2.3274431	1.9207988	2.30239	-0.406644	-0.025053	-0.215849	down
A_33_P3221253	LPP	1.2306342	1.0026426	1.0267925	-0.227992	-0.203842	-0.215917	down
A_23_P137578	FBXO28	2.3658142	2.0486598	2.2511044	-0.317154	-0.11471	-0.215932	down
A_23_P63026	LGALS8	1.3502407	1.0549245	1.2135258	-0.295316	-0.136715	-0.216016	down
A_32_P107493	SPATA33	0.9550118	0.5274749	0.950511	-0.427537	-0.004501	-0.216019	down
A_23_P394448	DPY19L1	0.5701122	0.1570802	0.5510373	-0.413032	-0.019075	-0.216053	down
A_33_P3395403	LOC100996724	0.0842643	-0.232607	-0.031073	-0.316872	-0.115337	-0.216105	down
A_24_P38815	TPP1	0.1451902	-0.263571	0.1213169	-0.408761	-0.023873	-0.216317	down
A_32_P209624	MKRN7P	-0.526986	-0.675742	-0.811105	-0.148756	-0.284119	-0.216437	down
A_33_P3228355	ERV3-1	-2.213558	-2.219889	-2.640103	-0.00633	-0.426545	-0.216438	down
A_21_P0014146	lnc-RASA1-5	-1.901881	-1.889882	-2.346771	0.0119996	-0.44489	-0.216445	down

A_23_P107724	ZNF112	0.9788399	1.0006661	0.5240665	0.0218263	-0.454773	-0.216474	down
A_23_P23616	PLEKHN1	0.8268008	0.7060528	0.5145164	-0.120748	-0.312284	-0.216516	down
A_33_P3361681	VMA21	5.489418	5.099665	5.445977	-0.389753	-0.043441	-0.216597	down
A_24_P71439	PUM2	2.0398178	1.8042564	1.8421211	-0.235561	-0.197697	-0.216629	down
A_23_P134008	USP45	-0.510036	-0.551892	-0.901557	-0.041856	-0.391521	-0.216689	down
A_24_P135753	PTPLB	3.485631	3.4205709	3.117217	-0.06506	-0.368414	-0.216737	down
A_33_P3343290	PPTC7	0.1175995	-0.003469	-0.194962	-0.121068	-0.312561	-0.216815	down
A_24_P380679	LSMEM1	-1.519487	-1.692788	-1.779827	-0.173301	-0.26034	-0.216821	down
A_21_P0011619	TBC1D3P5	1.6340475	1.6434793	1.1909528	0.0094318	-0.443095	-0.216831	down
A_23_P79911	PSMF1	3.8088017	3.4241843	3.7594347	-0.384617	-0.049367	-0.216992	down
A_24_P945147	RABEP1	0.7023511	0.5171318	0.4535165	-0.185219	-0.248835	-0.217027	down
A_21_P0014626	RNF13	4.763732	4.5216527	4.5715027	-0.242079	-0.192229	-0.217154	down
A_23_P300728	LMBR1	1.5318308	1.2971001	1.3319764	-0.234731	-0.199854	-0.217293	down
A_23_P218988	SLC30A5	2.4023743	2.1423678	2.2277346	-0.260006	-0.17464	-0.217323	down
A_23_P148463	CUL4B	1.4905887	1.4264302	1.1199899	-0.064158	-0.370599	-0.217379	down
A_24_P281975	GNPTAB	3.7386198	3.399179	3.6431732	-0.339441	-0.095447	-0.217444	down
A_23_P56759	KRCC1	1.7581644	1.7063336	1.375031	-0.051831	-0.383133	-0.217482	down
A_23_P22614	SEPT6	-0.491031	-0.831918	-0.585323	-0.340887	-0.094292	-0.217589	down
A_24_P51118	MTAP	0.9594688	0.8041134	0.679492	-0.155355	-0.279977	-0.217666	down
A_23_P157449	POLR2K	3.593834	3.110495	3.641817	-0.483339	0.0479832	-0.217678	down
A_24_P241792	ZNF160	1.4277697	1.1834173	1.2365432	-0.244352	-0.191226	-0.217789	down
A_33_P3380098	PXN	-1.583589	-1.748087	-1.854678	-0.164498	-0.271089	-0.217793	down
A_23_P210358	LIMS1	2.9407291	2.7028909	2.7428274	-0.237838	-0.197902	-0.21787	down
A_24_P291231	PER3	-0.355595	-0.474793	-0.672191	-0.119198	-0.316596	-0.217897	down
A_23_P209232	CLIP4	4.027622	4.076504	3.5429058	0.048882	-0.484716	-0.217917	down
A_23_P323143	ZNF767P	-0.324968	-0.596097	-0.48977	-0.271129	-0.164802	-0.217966	down
A_33_P3449397	METTL3	2.7698889	2.4592023	2.64433	-0.310687	-0.125559	-0.218123	down
A_23_P209426	TRAK2	1.9078665	1.6585603	1.7209125	-0.249306	-0.186954	-0.21813	down
A_21_P0010048	lnc-SULF2-4	-1.923549	-2.116744	-2.166802	-0.193195	-0.243253	-0.218224	down
A_22_P00005863	ERN1	0.7962103	0.5271764	0.6285315	-0.269034	-0.167679	-0.218356	down
A_23_P315836	BAIAP2	1.3148527	1.1554255	1.0374689	-0.159427	-0.277384	-0.218405	down
A_22_P00015282	NBPF1	-1.631047	-1.942408	-1.756504	-0.311361	-0.125456	-0.218409	down
A_22_P00022005	lnc-B4GALT7-1	-2.816496	-3.204294	-2.865687	-0.387797	-0.049191	-0.218494	down
A_32_P1445	PTPN2	1.7674837	1.575675	1.5222502	-0.191809	-0.245234	-0.218521	down
A_23_P60130	MAL2	6.818244	6.678269	6.5208225	-0.139975	-0.297421	-0.218698	down
A_33_P3347772	SEPT2	0.2164311	-0.155211	0.1502042	-0.371642	-0.066227	-0.218935	down
A_23_P97328	C1orf35	4.7081785	3.9795318	4.9989347	-0.728647	0.2907562	-0.218945	down
A_33_P3379017	HIC2	0.523241	0.2765546	0.3319764	-0.246686	-0.191265	-0.218976	down
A_23_P43086	BIN3	1.8708801	1.3644314	1.9390593	-0.506449	0.0681791	-0.219135	down
A_23_P9662	IPP	2.1379452	1.6606832	2.1769304	-0.477262	0.0389853	-0.219138	down
A_33_P3303729	XLOC_l2_013415	-1.805319	-2.162576	-1.886518	-0.357257	-0.081199	-0.219228	down
A_33_P3251685	PDP2	1.2417998	0.7322183	1.3126688	-0.509582	0.070869	-0.219356	down
A_23_P136196	TBC1D19	-1.018604	-1.084944	-1.391068	-0.066339	-0.372463	-0.219401	down
A_33_P3305749	RARB	-1.555279	-1.737629	-1.811747	-0.18235	-0.256468	-0.219409	down
A_22_P00013886	LOC101927060	-0.494091	-0.725869	-0.701192	-0.231778	-0.207101	-0.219439	down
A_32_P81768	TMEM167A	3.308342	2.7530704	3.424693	-0.555272	0.1163511	-0.21946	down
A_23_P158318	ROR2	-2.550409	-3.189496	-2.350248	-0.639086	0.2001615	-0.219462	down
A_33_P3283824	SLC39A8	-1.437857	-1.56461	-1.750102	-0.126753	-0.312245	-0.219499	down
A_23_P15714	NSF	5.153042	4.82439	5.0425005	-0.328652	-0.110541	-0.219597	down
A_33_P3315021	RPL23AP7	0.6045713	0.2313752	0.5385008	-0.373196	-0.066071	-0.219633	down
A_23_P126844	TNFRSF25	2.404642	1.9892116	2.380601	-0.415431	-0.024041	-0.219736	down
A_33_P3399693	LOC100127955	-2.907714	-3.252938	-3.001986	-0.345224	-0.094272	-0.219748	down
A_33_P3358977	RNASEH2C	3.3679	3.0539937	3.2422037	-0.313906	-0.125696	-0.219801	down
A_23_P11507	ZZZ3	2.4862566	2.3019567	2.2309437	-0.1843	-0.255313	-0.219806	down
A_33_P3302280	PDIK1L	-2.427877	-2.696142	-2.599315	-0.268265	-0.171438	-0.219852	down
A_19_P00809423	LINC00423	-2.026147	-2.093975	-2.398193	-0.067828	-0.372046	-0.219937	down

A_21_P0000715	UNK	1.7626228	1.3702693	1.7149353	-0.392354	-0.047688	-0.220021	down
A_24_P158946	FGD4	2.127883	1.8389444	1.9763288	-0.288939	-0.151554	-0.220246	down
A_23_P26094	SLTM	3.5867243	3.4256082	3.3070126	-0.161116	-0.279712	-0.220414	down
A_24_P236522	CD2BP2	2.4790287	1.8164148	2.7007904	-0.662614	0.2217617	-0.220426	down
A_24_P3973	HNRNPA2B1	6.668993	6.3266234	6.570341	-0.34237	-0.098652	-0.220511	down
A_33_P3323347	ZNF417	0.6021524	0.4984655	0.2646995	-0.103687	-0.337453	-0.22057	down
A_21_P0002852	SEMA3B-AS1	-1.106273	-1.305254	-1.348473	-0.198981	-0.2422	-0.22059	down
A_23_P218626	NEU4	-1.612898	-1.877638	-1.789363	-0.26474	-0.176466	-0.220603	down
A_21_P0007073	lnc-SCD-1	-0.399113	-0.630957	-0.608649	-0.231843	-0.209536	-0.22069	down
A_23_P42664	SHFM1	7.4176817	7.1471562	7.2467966	-0.270525	-0.170885	-0.220705	down
A_33_P3386995	TXNDC11	-1.625214	-1.636804	-2.055119	-0.01159	-0.429905	-0.220748	down
A_23_P69586	FAT1	4.8023005	4.7032533	4.459775	-0.099047	-0.342525	-0.220786	down
A_33_P3236921	PARP1	2.4807806	2.2008376	2.318963	-0.279943	-0.161818	-0.22088	down
A_33_P3357748	PVT1	2.4174128	2.2657247	2.1271524	-0.151688	-0.29026	-0.220974	down
A_32_P205241	GJA3	1.4396977	0.9709482	1.4662905	-0.46875	0.0265927	-0.221078	down
A_23_P165879	RAB10	5.729949	5.2600856	5.757655	-0.469863	0.0277061	-0.221079	down
A_22_P00015476	NCK1-AS1	-1.614333	-1.752861	-1.918	-0.138528	-0.303668	-0.221098	down
A_21_P0000561	PCNA-AS1	-2.314487	-2.886777	-2.184558	-0.57229	0.1299286	-0.221181	down
A_22_P00023014	lnc-MFSD9-4	-1.078411	-1.278878	-1.320446	-0.200467	-0.242035	-0.221251	down
A_33_P3223825	SRRM3	-2.253854	-2.181624	-2.768797	0.0722306	-0.514943	-0.221356	down
A_23_P81408	MAT2B	2.5787544	2.4717517	2.2430134	-0.107003	-0.335741	-0.221372	down
A_23_P151851	DUOX2	-0.141906	-0.33993	-0.38664	-0.198024	-0.244733	-0.221379	down
A_24_P192994	FADS1	1.330214	0.9021411	1.315443	-0.428073	-0.014771	-0.221422	down
A_23_P89755	RNF138	1.2981601	1.2430315	0.9103379	-0.055129	-0.387822	-0.221475	down
A_32_P150876	LINC00667	-2.492476	-2.855554	-2.572693	-0.363078	-0.080216	-0.221647	down
A_23_P81770	PTP4A1	5.418969	4.9882417	5.406394	-0.430727	-0.012575	-0.221651	down
A_24_P88565	SLC50A1	1.6627607	1.0632868	1.8187847	-0.599474	0.156024	-0.221725	down
A_21_P0014479	lnc-DNASE1L3-1	5.096264	4.857226	4.8917093	-0.239038	-0.204555	-0.221796	down
A_24_P363745	LRRC14	3.9682322	3.8386407	3.653576	-0.129591	-0.314656	-0.222124	down
A_23_P203829	ZNF10	-0.349022	-0.585663	-0.556648	-0.236641	-0.207626	-0.222133	down
A_24_P49533	ZYG11B	0.5893307	0.3218603	0.4120898	-0.26747	-0.177241	-0.222356	down
A_23_P257201	RNF146	0.3776794	0.1409102	0.1693444	-0.236769	-0.208335	-0.222552	down
A_22_P00010463	lnc-NAIF1-1	-2.400159	-2.851698	-2.393815	-0.451539	0.0063436	-0.222598	down
A_22_P00008226	LLPH-AS1	-2.517709	-2.947925	-2.53282	-0.430216	-0.015111	-0.222664	down
A_32_P188860	IL17RD	0.5848408	0.1658196	0.5583549	-0.419021	-0.026486	-0.222754	down
A_24_P103264	UGT8	2.287057	1.9488902	2.1796007	-0.338167	-0.107456	-0.222811	down
A_32_P70315	TIMP4	-1.282045	-1.06835	-1.941637	0.2136955	-0.659591	-0.222948	down
A_23_P85682	NFIA	1.426065	1.015832	1.3901806	-0.410233	-0.035884	-0.223059	down
A_22_P00007208	MIR92A1	-2.787724	-2.874654	-3.147176	-0.086931	-0.359452	-0.223191	down
A_23_P400066	RALGAPB	1.8844843	1.7748137	1.547677	-0.109671	-0.336807	-0.223239	down
A_23_P5586	MPHOSPH10	3.429597	3.1499763	3.262289	-0.279621	-0.167308	-0.223464	down
A_23_P108376	THADA	1.4997525	1.2954216	1.2571425	-0.204331	-0.24261	-0.22347	down
A_21_P0007430	LOC494141	-1.786271	-2.174648	-1.844847	-0.388378	-0.058577	-0.223477	down
A_23_P361569	SLC35F5	1.7281723	1.5638447	1.4452806	-0.164328	-0.282892	-0.22361	down
A_24_P203622	DOCK7	-0.011646	-0.230427	-0.240138	-0.218781	-0.228491	-0.223636	down
A_33_P3420035	ZNF445	1.5616217	1.4662437	1.209693	-0.095378	-0.351929	-0.223653	down
A_33_P3210343	ETV6	1.2786222	1.0433478	1.0665808	-0.235274	-0.212041	-0.223658	down
A_33_P3228872	R3HCC1L	-0.067089	-0.458604	-0.123148	-0.391515	-0.056059	-0.223787	down
A_24_P237804	POTED	1.7583303	1.5963511	1.4727302	-0.161979	-0.2856	-0.22379	down
A_21_P0014028	PP12719	-0.784329	-0.626652	-1.389611	0.1576767	-0.605282	-0.223803	down
A_23_P73982	NDC1	4.3734217	4.028107	4.271103	-0.345315	-0.102319	-0.223817	down
A_23_P252855	BRIX1	3.6025953	3.1865382	3.5709496	-0.416057	-0.031646	-0.223851	down
A_32_P64096	ZKSCAN2	0.5121746	0.2658043	0.3108397	-0.24637	-0.201335	-0.223853	down
A_23_P328069	HPS1	0.8511014	0.605557	0.6488104	-0.245544	-0.202291	-0.223918	down
A_33_P3277753	ANKRD23	-0.281285	-0.618555	-0.39186	-0.33727	-0.110575	-0.223922	down
A_23_P19987	IGF2BP3	4.5841274	4.399853	4.3203363	-0.184274	-0.263791	-0.224033	down

A_23_P251075	MAMLD1	-1.699861	-2.234218	-1.613986	-0.534357	0.085875	-0.224241	down
A_24_P181149	BBS10	0.1982727	-0.1304	0.0783329	-0.328672	-0.11994	-0.224306	down
A_32_P515431	LOC100132057	-2.460154	-2.679557	-2.68956	-0.219403	-0.229406	-0.224404	down
A_23_P124417	BUB1	3.0894728	2.6701522	3.0598307	-0.419321	-0.029642	-0.224481	down
A_22_P00013393	TMPO-AS1	-1.965496	-2.313658	-2.066504	-0.348162	-0.101007	-0.224585	down
A_33_P3317815	KRAS	2.620181	2.3817945	2.4093895	-0.238387	-0.210792	-0.224589	down
A_23_P322704	FAM177A1	2.5975018	2.4817138	2.2640858	-0.115788	-0.333416	-0.224602	down
A_23_P408271	HSD17B11	2.4031258	2.1470952	2.209878	-0.256031	-0.193248	-0.224639	down
A_33_P3278475	FOXN2	2.7515879	2.4959397	2.5578098	-0.255648	-0.193778	-0.224713	down
A_24_P313109	UFM1	0.7379046	0.6517181	0.3744621	-0.086186	-0.363442	-0.224814	down
A_33_P3254380	SLC9A7	0.1174331	-0.328366	0.1135149	-0.445799	-0.003918	-0.224859	down
A_21_P0000195	FAM208B	3.5049458	3.252256	3.3076906	-0.25269	-0.197255	-0.224972	down
A_24_P98251	VPS13B	2.8608408	2.6465802	2.6250782	-0.214261	-0.235763	-0.225012	down
A_24_P166661	TMEM60	2.0863686	1.6274638	2.0952454	-0.458905	0.0088768	-0.225014	down
A_24_P281801	ZNF732	0.8010612	0.5858231	0.5661721	-0.215238	-0.234889	-0.225064	down
A_32_P1701	POLA1	3.1467972	3.0117364	2.8316975	-0.135061	-0.3151	-0.22508	down
A_33_P3662553	YWHAQ	4.582609	4.3827205	4.3321705	-0.199889	-0.250439	-0.225164	down
A_23_P114414	LONRF3	1.1324039	0.7886291	1.0256596	-0.343775	-0.106744	-0.22526	down
A_23_P206382	C16orf70	2.8834372	2.5983272	2.7179775	-0.28511	-0.16546	-0.225285	down
A_33_P3210866	ZNF808	1.8035212	1.5237951	1.6323404	-0.279726	-0.171181	-0.225453	down
A_33_P3392921	CTTN	0.5114255	0.3612557	0.2106218	-0.15017	-0.300804	-0.225487	down
A_19_P00813124	XLOC_l2_010511	-2.745403	-2.804655	-3.137187	-0.059252	-0.391784	-0.225518	down
A_23_P252106	RIPK2	3.4339943	3.1437993	3.2730322	-0.290195	-0.160962	-0.225579	down
A_23_P318296	PLEKHA8	-0.707909	-0.78199	-1.085114	-0.07408	-0.377205	-0.225643	down
A_32_P184279	CCDC6	6.368105	6.0207486	6.264159	-0.347356	-0.103946	-0.225651	down
A_33_P3702055	SLC11A1	-0.867396	-1.087941	-1.098321	-0.220545	-0.230926	-0.225735	down
A_23_P118300	FAHD1	2.8574562	2.4611402	2.8019638	-0.396316	-0.055492	-0.225904	down
A_21_P0005119	lnc-SH3BGRL2-1	-1.769357	-1.743708	-2.246955	0.0256486	-0.477598	-0.225975	down
A_23_P65918	ITPKA	3.6921988	3.2474551	3.6849823	-0.444744	-0.007216	-0.22598	down
A_33_P3403044	MAT2A	1.5623436	1.3384118	1.3342967	-0.223932	-0.228047	-0.225989	down
A_24_P17453	TTC30A	-2.419279	-3.003528	-2.287115	-0.584249	0.132164	-0.226042	down
A_33_P3362641	CISD2	3.540122	3.1423984	3.4850798	-0.397724	-0.055042	-0.226383	down
A_24_P93741	RFT1	-0.670081	-0.641756	-1.15141	0.0283251	-0.481329	-0.226502	down
A_24_P161293	NBPF9	5.33778	5.08919	5.133212	-0.24859	-0.204568	-0.226579	down
A_22_P00020023	PDP2	2.9778385	2.3540444	3.148344	-0.623794	0.1705055	-0.226644	down
A_23_P12336	PRMT6	1.9409189	1.7550817	1.6732049	-0.185837	-0.267714	-0.226776	down
A_19_P00808033	STRN	-2.263438	-2.491874	-2.488576	-0.228436	-0.225138	-0.226787	down
A_23_P31407	AGR2	5.0653915	5.3022103	4.374977	0.2368188	-0.690414	-0.226798	down
A_23_P258002	CDKN2AIP	1.9643173	1.7386079	1.736381	-0.225709	-0.227936	-0.226823	down
A_23_P251293	SNCG	-2.252282	-2.142985	-2.815424	0.1092963	-0.563142	-0.226923	down
A_33_P3888629	MECOM	2.1557732	1.9716458	1.8859615	-0.184127	-0.269812	-0.226969	down
A_23_P140978	ZNF263	-0.172685	-0.393234	-0.406149	-0.22055	-0.233465	-0.227007	down
A_32_P194779	ZBTB34	1.489583	1.2235494	1.3015966	-0.266034	-0.187986	-0.22701	down
A_23_P40072	MTIF2	2.5075054	2.3421493	2.2188206	-0.165356	-0.288685	-0.227021	down
A_24_P303199	ARL15	-0.165222	-0.427984	-0.356507	-0.262762	-0.191285	-0.227024	down
A_23_P256956	KIF20A	1.6988735	1.5052447	1.4383407	-0.193629	-0.260533	-0.227081	down
A_21_P0000316	SNORA20	0.105607	-0.300382	0.0574307	-0.405989	-0.048176	-0.227083	down
A_23_P154217	ITGB6	0.1633973	-0.236861	0.1093583	-0.400258	-0.054039	-0.227149	down
A_19_P00320362	NBAT1	-2.575436	-2.731245	-2.873954	-0.155809	-0.298518	-0.227163	down
A_23_P25989	SETD3	-0.058291	-0.428574	-0.142597	-0.370282	-0.084305	-0.227294	down
A_23_P359214	LINC00842	1.0504432	0.7719822	0.8742795	-0.278461	-0.176164	-0.227312	down
A_33_P3659678	NR6A1	3.0080671	2.8421655	2.7193365	-0.165902	-0.288731	-0.227316	down
A_23_P147199	ZNF271P	1.0018296	0.7913046	0.7576623	-0.210525	-0.244167	-0.227346	down
A_24_P838448	CASC15	-1.981401	-1.937975	-2.479583	0.043426	-0.498182	-0.227378	down
A_24_P494454	SPIN3	-0.672968	-0.900902	-0.899836	-0.227933	-0.226867	-0.2274	down
A_23_P14892	IGFALS	-2.618458	-2.869621	-2.822136	-0.251162	-0.203678	-0.22742	down

A_33_P3390284	FAM122B	-1.303744	-1.534725	-1.527771	-0.23098	-0.224027	-0.227504	down
A_23_P35916	ATM	-1.433512	-1.393125	-1.929172	0.0403872	-0.49566	-0.227636	down
A_33_P3889862	LOC151475	-1.474427	-1.456554	-1.948201	0.0178738	-0.473774	-0.22795	down
A_22_P00009172	LOC100507577	0.826313	0.8024683	0.394228	-0.023845	-0.432085	-0.227965	down
A_33_P3230103	RNFT2	-1.406053	-1.856235	-1.411867	-0.450182	-0.005814	-0.227998	down
A_32_P16854	ANAPC13	2.048665	1.7772565	1.863842	-0.271409	-0.184823	-0.228116	down
A_23_P34093	G6PD	2.1976757	1.9186492	2.020382	-0.279027	-0.177294	-0.22816	down
A_23_P14105	RCBTB2	-0.056953	-0.411592	-0.158679	-0.354638	-0.101725	-0.228182	down
A_32_P94	LHFPL4	-2.584438	-2.682702	-2.942678	-0.098264	-0.35824	-0.228252	down
A_23_P115482	UBE2T	5.968849	5.5766234	5.904003	-0.392226	-0.064846	-0.228536	down
A_22_P00013376	lnc-RP11-17M16.1.1-1	-2.528421	-2.509098	-3.004953	0.0193236	-0.476532	-0.228604	down
A_23_P143734	CYP2D6	-2.443533	-2.837962	-2.50658	-0.394428	-0.063046	-0.228737	down
A_23_P2661	RAP1B	5.858409	5.642732	5.6165733	-0.215677	-0.241836	-0.228756	down
A_33_P3281066	ZBTB21	1.116982	0.9106336	0.8657937	-0.206348	-0.251188	-0.228768	down
A_33_P3305254	PSMD5	0.2103705	-0.182719	0.145906	-0.39309	-0.064465	-0.228777	down
A_21_P0014949	NPEPPS	1.0771866	0.9478741	0.7488623	-0.129313	-0.328324	-0.228818	down
A_23_P152218	E2F4	1.6880121	1.1388087	1.7795606	-0.549203	0.0915484	-0.228827	down
A_32_P174908	WAPAL	3.87597	3.5739923	3.7201834	-0.301978	-0.155787	-0.228882	down
A_19_P00322906	C17orf51	-1.296752	-1.356824	-1.694821	-0.060072	-0.398069	-0.22907	down
A_23_P500542	DMBX1	0.1047139	-0.218926	-0.029905	-0.32364	-0.134619	-0.22913	down
A_21_P0012676	GK3P	-1.882962	-1.831127	-2.393205	0.0518351	-0.510243	-0.229204	down
A_33_P3230176	EPT1	0.5592251	0.1808028	0.4790945	-0.378422	-0.080131	-0.229276	down
A_23_P120744	MCM3AP	1.432909	0.9097967	1.497447	-0.523112	0.064538	-0.229287	down
A_24_P364296	STX2	0.3620267	0.0486703	0.216558	-0.313356	-0.145469	-0.229413	down
A_21_P0013163	RAET1K	0.4545169	0.0826101	0.3675418	-0.371907	-0.086975	-0.229441	down
A_22_P00009057	lnc-LENG9-1	-0.254627	-0.462712	-0.505486	-0.208085	-0.250859	-0.229472	down
A_23_P167595	UBE2B	0.5398521	0.1933084	0.4274368	-0.346544	-0.112415	-0.22948	down
A_33_P3222892	SLC10A3	4.096155	3.5283275	4.2048874	-0.567828	0.1087322	-0.229548	down
A_32_P37592	SCARNA17	3.358573	2.833178	3.4248667	-0.525395	0.0662937	-0.229551	down
A_23_P107116	RNF112	-1.789203	-1.927079	-2.110481	-0.137876	-0.321278	-0.229577	down
A_23_P212844	TACC3	1.7208595	1.3842506	1.5983081	-0.336609	-0.122551	-0.22958	down
A_23_P92842	SAR1B	3.5338535	3.176085	3.4324408	-0.357769	-0.101413	-0.229591	down
A_23_P59714	MGC16142	-2.391985	-2.781718	-2.461451	-0.389733	-0.069466	-0.2296	down
A_33_P3340769	ADAM9	-1.968355	-2.138425	-2.257486	-0.170069	-0.289131	-0.2296	down
A_23_P25605	KBTBD7	0.602242	0.3880391	0.357204	-0.214203	-0.245038	-0.22962	down
A_24_P194886	EHBP1	1.0177293	0.7175441	0.8586626	-0.300185	-0.159067	-0.229626	down
A_24_P405621	NISCH	1.2518988	0.9669609	1.077548	-0.284938	-0.174351	-0.229644	down
A_23_P138105	MED18	1.1584516	1.5006304	0.3568244	0.3421788	-0.801627	-0.229724	down
A_23_P23966	ZNF488	-0.854045	-1.09964	-1.067932	-0.245595	-0.213887	-0.229741	down
A_33_P3407742	PPM1B	1.1765614	1.000556	0.8930073	-0.176005	-0.283554	-0.22978	down
A_24_P171873	FBXO4	-0.366781	-0.422402	-0.771023	-0.055621	-0.404243	-0.229932	down
A_33_P3361422	CYP27A1	-2.22631	-2.672696	-2.239792	-0.446386	-0.013482	-0.229934	down
A_21_P0000921	LINC01352	-2.057695	-2.275998	-2.299393	-0.218303	-0.241698	-0.23	down
A_23_P53217	EED	5.030553	4.6701016	4.9309607	-0.360451	-0.099592	-0.230022	down
A_23_P45294	FAM199X	4.3526754	4.290523	3.9547014	-0.062152	-0.397974	-0.230063	down
A_24_P192434	TERF1	3.211053	3.0822797	2.8796463	-0.128773	-0.331407	-0.23009	down
A_33_P3479999	LOC494150	8.834554	8.598228	8.610556	-0.236325	-0.223998	-0.230162	down
A_24_P98975	ANKRD13A	1.8323927	1.5300975	1.6742425	-0.302295	-0.15815	-0.230223	down
A_19_P00318085	HCG18	-0.46107	-0.790726	-0.591995	-0.329656	-0.130926	-0.230291	down
A_23_P407142	LUZP1	-0.427602	-0.691289	-0.624518	-0.263687	-0.196917	-0.230302	down
A_22_P00024593	C5orf66-AS1	-1.265822	-1.450831	-1.541534	-0.185009	-0.275712	-0.230361	down
A_21_P0009976	lnc-TSHZ2-3	-1.807803	-2.096931	-1.979637	-0.289128	-0.171834	-0.230481	down
A_24_P75920	CCDC159	-2.674315	-3.054737	-2.754889	-0.380422	-0.080574	-0.230498	down
A_21_P0003733	lnc-CCKAR-1	-0.778918	-1.004963	-1.013926	-0.226045	-0.235008	-0.230526	down
A_33_P3256425	BICD1	-2.24397	-2.185027	-2.764323	0.0589428	-0.520353	-0.230705	down

A_32_P113508	ATG16L1	2.832118	2.505876	2.6966524	-0.326242	-0.135466	-0.230854	down
A_33_P3262138	ANAPC1	0.2078438	-0.101182	0.0550656	-0.309025	-0.152778	-0.230902	down
A_23_P252711	LACTB2	1.7805586	1.3562613	1.7429004	-0.424297	-0.037658	-0.230978	down
A_23_P157600	DDHD2	-0.397621	-0.886492	-0.370737	-0.488871	0.0268846	-0.230993	down
A_33_P3215720	PPP1R2	-0.344403	-0.574314	-0.576606	-0.229911	-0.232203	-0.231057	down
A_23_P59877	FABP5	6.577772	6.198478	6.4949446	-0.379294	-0.082828	-0.231061	down
A_33_P3293456	GATA4	-2.035468	-2.334163	-2.198927	-0.298696	-0.16346	-0.231078	down
A_23_P84651	CNBP	5.7193356	5.444822	5.531563	-0.274514	-0.187773	-0.231143	down
A_22_P00011609	LOC101929066	-2.475561	-2.732733	-2.681192	-0.257172	-0.205632	-0.231402	down
A_21_P0008858	lnc-PEAK1.1-1	-1.482789	-1.728847	-1.699668	-0.246058	-0.216879	-0.231469	down
A_22_P00007797	lnc-HOMER1-1	-3.007992	-3.030011	-3.448938	-0.02202	-0.440946	-0.231483	down
A_21_P0013751	XLOC_l2_015441	-0.549918	-0.746504	-0.816319	-0.196586	-0.266401	-0.231493	down
A_23_P115885	MINPP1	0.0855336	-0.241409	-0.050527	-0.326943	-0.136061	-0.231502	down
A_33_P3352103	LYPLAL1	1.6418056	1.4073095	1.4127884	-0.234496	-0.229017	-0.231757	down
A_33_P3412538	ANKRD17	3.2147226	2.9112554	3.0546608	-0.303467	-0.160062	-0.231765	down
A_24_P194748	ZNF275	-0.549855	-0.844578	-0.718665	-0.294724	-0.16881	-0.231767	down
A_21_P0003072	lnc-EIF2B5-2	-2.581558	-2.909013	-2.717657	-0.327455	-0.1361	-0.231777	down
A_23_P258321	MRPS17	5.3320026	4.8698945	5.3302364	-0.462108	-0.001766	-0.231937	down
A_19_P00322333	LOC101059954	-1.209808	-1.412612	-1.47099	-0.202804	-0.261182	-0.231993	down
A_23_P342641	SLC44A5	-1.216269	-1.252049	-1.644593	-0.03578	-0.428324	-0.232052	down
A_22_P00010482	lnc-NAV1-1	-2.629481	-2.66192	-3.061225	-0.032439	-0.431744	-0.232092	down
A_32_P3914	WIPF3	-0.178881	-0.213419	-0.608629	-0.034539	-0.429748	-0.232143	down
A_23_P50897	NIFK	3.513711	3.2440991	3.3189726	-0.269612	-0.194738	-0.232175	down
A_33_P3218772	UPF2	-0.741198	-0.987281	-0.959486	-0.246083	-0.218288	-0.232186	down
A_32_P416161	XKRX	-1.125738	-1.449338	-1.266649	-0.323599	-0.140911	-0.232255	down
A_23_P345361	C18orf25	0.5178485	0.1081915	0.4627824	-0.409657	-0.055066	-0.232362	down
A_33_P3322307	DDX11	-0.991641	-1.171758	-1.276301	-0.180117	-0.28466	-0.232389	down
A_33_P3351765	KLHDC10	-0.918407	-0.957472	-1.344519	-0.039065	-0.426112	-0.232589	down
A_23_P386320	MFI2	2.389309	2.1208816	2.1925468	-0.268427	-0.196762	-0.232595	down
A_23_P132316	APOBEC3D	-2.853366	-2.833539	-3.338394	0.0198264	-0.485028	-0.232601	down
A_24_P924202	PCNXL3	0.6265011	-0.114363	0.9021134	-0.740864	0.2756124	-0.232626	down
A_33_P3359900	EMB	2.5593042	2.2992692	2.3540096	-0.260035	-0.205295	-0.232665	down
A_33_P3291541	TMCO4	-1.77875	-1.760566	-2.26231	0.0181847	-0.483559	-0.232687	down
A_33_P3299435	SART1	-0.217427	-0.473619	-0.426685	-0.256192	-0.209258	-0.232725	down
A_22_P00009162	LMO7	-2.046799	-2.245457	-2.313663	-0.198658	-0.266864	-0.232761	down
A_21_P0009323	lnc-NR1D1-1	0.0351257	-0.326905	-0.068496	-0.362031	-0.103621	-0.232826	down
A_23_P371011	ZNF227	-0.474826	-0.702313	-0.713275	-0.227487	-0.238448	-0.232968	down
A_23_P395595	FNBP4	2.104497	1.9448867	1.7979951	-0.15961	-0.306502	-0.233056	down
A_32_P86118	PM20D2	2.4509516	2.156057	2.279561	-0.294895	-0.171391	-0.233143	down
A_33_P3217804	CAMSAP1	-2.332948	-2.361841	-2.770698	-0.028894	-0.437751	-0.233322	down
A_32_P219368	WTAP	1.7195997	1.4213519	1.5511966	-0.298248	-0.168403	-0.233325	down
A_21_P0005492	lnc-C7orf11-2	-1.832456	-1.779048	-2.352593	0.0534077	-0.520137	-0.233365	down
A_21_P0006870	lnc-ANTXRL-3	1.5586176	1.2300391	1.4202094	-0.328578	-0.138408	-0.233493	down
A_22_P00022663	lnc-RPL30-1	-1.151687	-1.500929	-1.269557	-0.349242	-0.11787	-0.233556	down
A_21_P0007419	lnc-NFRKB-1	-2.772522	-2.952412	-3.059773	-0.17989	-0.287251	-0.23357	down
A_23_P319133	DNAJC10	3.4364471	3.2868829	3.1185017	-0.149564	-0.317945	-0.233755	down
A_33_P3293668	DENND4C	2.803506	2.6683917	2.4710102	-0.135114	-0.332496	-0.233805	down
A_23_P88351	ATL1	0.0530629	-0.124222	-0.237387	-0.177285	-0.29045	-0.233867	down
A_23_P151791	LTB4R	-0.935382	-1.028856	-1.309734	-0.093474	-0.374352	-0.233913	down
A_23_P92154	MBD4	2.1050148	1.7682233	1.9738846	-0.336792	-0.13113	-0.233961	down
A_33_P3340180	PPIP5K1	-1.827454	-2.255927	-1.86691	-0.428473	-0.039455	-0.233964	down
A_24_P935009	CRKL	1.4699073	1.3978076	1.0737424	-0.0721	-0.396165	-0.234132	down
A_23_P121396	DNAJC19	2.0117655	1.9468732	1.6082721	-0.064892	-0.403493	-0.234193	down
A_33_P3403549	CNEP1R1	-1.847131	-2.132128	-2.030566	-0.284997	-0.183435	-0.234216	down
A_23_P304237	RAPGEF1	0.8574295	0.1869788	1.0594258	-0.670451	0.2019963	-0.234227	down
A_24_P285501	UBR3	0.199142	-0.093137	0.0229597	-0.292279	-0.176182	-0.234231	down

A_23_P129956	DUSP3	2.640668	2.2814164	2.5313568	-0.359252	-0.109311	-0.234281	down
A_23_P76078	IL23A	2.217411	2.158566	1.8075886	-0.058845	-0.409822	-0.234334	down
A_33_P3308263	DIRC2	0.4513311	0.5042057	-0.070217	0.0528746	-0.521548	-0.234337	down
A_21_P0013814	XLOC_l2_015722	0.820518	0.486486	0.6858058	-0.334032	-0.134712	-0.234372	down
A_22_P00013946	lnc-RSL1D1-2	-2.94182	-3.147255	-3.205131	-0.205435	-0.263311	-0.234373	down
A_23_P152651	DDX42	3.566678	3.3798041	3.2847967	-0.186874	-0.281881	-0.234378	down
A_21_P0008759	PEAK1	-1.340684	-1.898361	-1.251799	-0.557677	0.0888844	-0.234396	down
A_23_P73530	MTMR1	3.1342535	2.7613015	3.0384035	-0.372952	-0.09585	-0.234401	down
A_24_P6674	WDR31	-2.191033	-2.409815	-2.441304	-0.218782	-0.25027	-0.234526	down
A_32_P134968	SPTB	-2.560331	-2.986681	-2.603071	-0.426349	-0.042739	-0.234544	down
A_33_P3250463	PMS1	1.068747	0.7946739	0.8736916	-0.274073	-0.195055	-0.234564	down
A_33_P3307957	ABCD3	1.4709082	0.8100162	1.6624498	-0.660892	0.1915417	-0.234675	down
A_33_P3322909	CUL4A	1.232687	0.8760138	1.1199718	-0.356673	-0.112715	-0.234694	down
A_21_P0013562	EBLN3	-0.588639	-0.948211	-0.698498	-0.359572	-0.109859	-0.234716	down
A_23_P99942	LRRK1	0.1921673	-0.028913	-0.056235	-0.221081	-0.248403	-0.234742	down
A_21_P0004696	lnc-TULP4-1	5.8198843	5.5949445	5.5753164	-0.22494	-0.244568	-0.234754	down
A_32_P77502	HNRNPA3	6.7097397	6.2993274	6.6504183	-0.410412	-0.059321	-0.234867	down
A_24_P71468	QPCT	3.959797	3.7627902	3.686823	-0.197007	-0.272974	-0.23499	down
A_22_P00005924	HOXA10-AS	3.0062952	2.6859403	2.8566122	-0.320355	-0.149683	-0.235019	down
A_23_P168771	CCDC146	-0.647293	-0.684765	-1.079943	-0.037472	-0.43265	-0.235061	down
A_33_P3589722	ATM	-0.32197	-0.646474	-0.467697	-0.324504	-0.145727	-0.235116	down
A_33_P3288774	RC3H1	0.8668971	0.6745186	0.5890145	-0.192379	-0.277883	-0.235131	down
A_23_P120103	KCNS3	4.366064	4.005489	4.2563276	-0.360575	-0.109736	-0.235156	down
A_23_P125639	ZFX	-1.547015	-1.848981	-1.715367	-0.301966	-0.168352	-0.235159	down
A_23_P57941	RBM6	-0.033014	-0.455971	-0.080443	-0.422957	-0.047429	-0.235193	down
A_33_P3332130	PGRMC1	6.4059477	6.0677505	6.2736263	-0.338197	-0.132321	-0.235259	down
A_23_P26865	MYH3	-2.863355	-2.716133	-3.481113	0.1472213	-0.617758	-0.235268	down
A_23_P148984	DARS2	2.3462286	1.8796544	2.3417892	-0.466574	-0.004439	-0.235507	down
A_23_P9415	ACO1	2.632471	2.3805223	2.4133997	-0.251949	-0.219071	-0.23551	down
A_23_P77310	ZNF106	3.4383001	3.127873	3.2776356	-0.310427	-0.160665	-0.235546	down
A_33_P3321150	CUL3	5.695307	5.2399106	5.679592	-0.455396	-0.015715	-0.235555	down
A_33_P3299510	SCX	0.05828	-0.178682	-0.17596	-0.236962	-0.23424	-0.235601	down
A_23_P218841	NGLY1	4.269308	4.0802846	3.9870796	-0.189024	-0.282228	-0.235626	down
A_33_P3279940	TM2D1	2.4804268	2.0856757	2.4037876	-0.394751	-0.076639	-0.235695	down
A_33_P3269728	ANKRD10	-0.218352	-0.427926	-0.480313	-0.209574	-0.261961	-0.235768	down
A_21_P0000635	NCAM1-AS1	-0.750794	-0.975269	-0.99788	-0.224475	-0.247086	-0.23578	down
A_33_P3322283	AZI2	-0.467202	-0.617876	-0.788105	-0.150674	-0.320902	-0.235788	down
A_21_P0010884	LINC00948	-2.734781	-3.109204	-2.832198	-0.374424	-0.097418	-0.235921	down
A_33_P3317725	LINC00243	-1.069365	-1.260614	-1.350015	-0.191249	-0.28065	-0.23595	down
A_33_P3375002	SOCS2-AS1	-0.631977	-1.102753	-0.633208	-0.470777	-0.001231	-0.236004	down
A_22_P00006061	lnc-FAM131B-1	-1.625189	-1.92642	-1.796003	-0.301231	-0.170815	-0.236023	down
A_32_P95729	FANCI	3.9875965	3.613708	3.8891926	-0.373889	-0.098404	-0.236146	down
A_33_P3341676	MEF2A	3.2095385	3.0836034	2.8631067	-0.125935	-0.346432	-0.236183	down
A_22_P00024090	lnc-GBP5-2	-0.540607	-0.819199	-0.734433	-0.278592	-0.193826	-0.236209	down
A_23_P209200	CCNE1	4.4419928	4.5463853	3.8650894	0.1043925	-0.576903	-0.236255	down
A_23_P307328	WHSC1	1.6853304	1.7600813	1.138042	0.0747509	-0.547288	-0.236269	down
A_23_P311010	SPRY3	0.1679368	0.3285661	-0.465348	0.1606293	-0.633285	-0.236328	down
A_24_P240242	MAP4	-2.023787	-2.442441	-2.077955	-0.418655	-0.054169	-0.236412	down
A_21_P0012236	NF1	0.5657029	0.5312705	0.1272378	-0.034432	-0.438465	-0.236449	down
A_24_P280029	PDXP	0.7662129	0.2362175	0.8232083	-0.529995	0.0569954	-0.2365	down
A_21_P0007153	LOC143666	-2.522496	-3.063014	-2.45503	-0.540518	0.0674653	-0.236526	down
A_33_P3277728	ITSN1	-0.084561	-0.33532	-0.30699	-0.250758	-0.222429	-0.236593	down
A_23_P385500	RNF166	-2.237658	-2.435571	-2.513053	-0.197913	-0.275395	-0.236654	down
A_32_P168464	CASK	2.0551224	1.9855733	1.6513419	-0.069549	-0.40378	-0.236665	down
A_21_P0004275	lnc-RASA1-3	-1.793605	-1.898556	-2.162103	-0.104951	-0.368498	-0.236724	down
A_23_P217938	SPHAR	2.065359	1.7334805	1.9237671	-0.331879	-0.141592	-0.236735	down

A_24_P943957	PIKFYVE	0.3991962	0.3462663	-0.021369	-0.05293	-0.420565	-0.236747	down
A_24_P117029	LDLR	5.9230604	5.387961	5.9841423	-0.5351	0.0610819	-0.237009	down
A_24_P355944	EFNB2	2.731513	2.4425159	2.5464716	-0.288997	-0.185041	-0.237019	down
A_23_P81811	TJAP1	2.1222734	2.0771346	1.6931419	-0.045139	-0.429132	-0.237135	down
A_32_P192823	PRPS1L1	0.9730845	0.7167378	0.7549963	-0.256347	-0.218088	-0.237217	down
A_33_P3415097	CLCN4	1.0155725	0.807096	0.7494726	-0.208477	-0.2661	-0.237288	down
A_22_P00008908	lnc-KLHL8-1	-2.462155	-2.54408	-2.854825	-0.081924	-0.39267	-0.237297	down
A_23_P91019	PRKRA	3.945774	3.8025632	3.6143808	-0.143211	-0.331393	-0.237302	down
A_33_P3337318	HNRNPA2B1	-2.208032	-2.332452	-2.558259	-0.12442	-0.350227	-0.237324	down
A_23_P203920	SSPN	-1.398885	-1.695949	-1.57653	-0.297064	-0.177645	-0.237355	down
A_33_P3257784	TSPAN17	-0.410464	-0.776748	-0.518972	-0.366283	-0.108508	-0.237396	down
A_33_P3406706	EXOG	0.8457904	0.5876951	0.6282506	-0.258095	-0.21754	-0.237818	down
A_21_P0008570	lnc-USP8-2	0.8296785	0.6749487	0.5087695	-0.15473	-0.320909	-0.237819	down
A_21_P0013234	XLOC_l2_013460	2.3444538	2.029655	2.1835957	-0.314799	-0.160858	-0.237828	down
A_21_P0014314	lnc-ZSCAN10-3	0.4940772	-0.051324	0.5637331	-0.545401	0.0696559	-0.237873	down
A_23_P90612	MCM6	5.208168	4.8957343	5.0446424	-0.312434	-0.163526	-0.23798	down
A_21_P0012246	XLOC_l2_009285	1.1538811	0.9725089	0.8592835	-0.181372	-0.294598	-0.237985	down
A_23_P11800	CAMK2N1	3.5093422	3.403503	3.1391792	-0.105839	-0.370163	-0.238001	down
A_33_P3351664	CCDC57	1.4598913	1.298295	1.1454186	-0.161596	-0.314473	-0.238034	down
A_33_P3271490	RBMS1	2.4177923	2.1015859	2.257886	-0.316206	-0.159906	-0.238056	down
A_23_P136172	TMEM68	0.4159517	0.329237	0.0265412	-0.086715	-0.389411	-0.238063	down
A_19_P00318297	APTR	-1.70789	-1.994642	-1.897289	-0.286752	-0.1894	-0.238076	down
A_23_P20683	KIAA0020	3.976385	3.5340486	3.9425554	-0.442337	-0.03383	-0.238083	down
A_33_P3354858	COQ10B	0.0192971	-0.288626	-0.149167	-0.307923	-0.168464	-0.238194	down
A_23_P202334	FGFR2	0.0922265	-0.151334	-0.140612	-0.243561	-0.232839	-0.2382	down
A_33_P3245321	CENPP	2.0523806	1.90063	1.7277303	-0.151751	-0.32465	-0.2382	down
A_24_P97001	PRPF38A	3.5630522	3.255899	3.3937273	-0.307153	-0.169325	-0.238239	down
A_23_P380298	LZTS3	-0.951075	-1.301641	-1.077532	-0.350565	-0.126457	-0.238511	down
A_33_P3832857	HAUS2	1.1593204	0.9101348	0.9313116	-0.249186	-0.228009	-0.238597	down
A_24_P406714	MLLT10	0.8243094	0.6938949	0.4775109	-0.130414	-0.346798	-0.238606	down
A_33_P3369520	MAGI3	1.5115423	1.3858781	1.1599326	-0.125664	-0.35161	-0.238637	down
A_23_P60354	SMARCA2	-0.214298	-0.294507	-0.611434	-0.080209	-0.397135	-0.238672	down
A_21_P0007247	RAB30-AS1	2.5380287	2.2119126	2.386673	-0.326116	-0.151356	-0.238736	down
A_24_P191781	PARM1	-1.926837	-2.282659	-2.048493	-0.355821	-0.121655	-0.238738	down
A_33_P3287972	RASA2	-1.886218	-2.040844	-2.209167	-0.154626	-0.322949	-0.238788	down
A_23_P41487	TBC1D9	0.9081717	1.0029583	0.3357358	0.0947866	-0.572436	-0.238825	down
A_32_P5480	CERS6	4.601886	4.34172	4.384368	-0.260166	-0.217518	-0.238842	down
A_23_P116173	COLCA2	-0.679464	-1.047741	-0.788988	-0.368277	-0.109524	-0.2389	down
A_23_P350234	UBE2NL	2.412469	2.0015483	2.3455734	-0.410921	-0.066895	-0.238908	down
A_33_P3362631	GUF1	1.8461657	1.647335	1.5671115	-0.198831	-0.279054	-0.238942	down
A_23_P311087	ZNF281	1.6754179	1.3752031	1.4977365	-0.300215	-0.177681	-0.238948	down
A_33_P3421325	ZNF782	-1.128692	-1.42156	-1.31374	-0.292868	-0.185048	-0.238958	down
A_33_P3234168	MTDH	6.550355	6.251537	6.371233	-0.298818	-0.179122	-0.23897	down
A_33_P3366120	FLNA	5.538707	5.3867774	5.212512	-0.151929	-0.326195	-0.239062	down
A_23_P423926	SS18L1	-0.054348	-0.266562	-0.320299	-0.212214	-0.265951	-0.239083	down
A_33_P3408212	HSP90B1	2.5769491	2.3227792	2.352869	-0.25417	-0.22408	-0.239125	down
A_23_P159237	GPR20	-0.085342	-0.758774	0.1097693	-0.673432	0.1951113	-0.239161	down
A_33_P3265224	CEP68	-0.70466	-0.745616	-1.142097	-0.040956	-0.437436	-0.239196	down
A_33_P3296479	APP	2.0460386	1.8516321	1.7618694	-0.194407	-0.284169	-0.239288	down
A_33_P3393796	TRMT5	3.5800934	3.212912	3.4686155	-0.367181	-0.111478	-0.23933	down
A_33_P3397693	AGPS	2.2103767	1.948998	1.9930706	-0.261379	-0.217306	-0.239342	down
A_33_P3308740	RANBP2	-1.029836	-1.144305	-1.394063	-0.114469	-0.364227	-0.239348	down
A_22_P00016601	KIAA0040	-0.412825	-1.095442	-0.209063	-0.682617	0.2037616	-0.239428	down
A_33_P3362981	KCTD5	-0.191515	-0.570182	-0.292055	-0.378667	-0.10054	-0.239604	down
A_23_P201939	PPM1J	-1.608552	-1.590162	-2.106244	0.0183902	-0.497692	-0.239651	down
A_24_P655268	OIP5-AS1	1.4962649	1.1499977	1.3630562	-0.346267	-0.133209	-0.239738	down

A_19_P00805548	BMPR1A	2.9118567	2.64986	2.694334	-0.261997	-0.217523	-0.23976	down
A_33_P3221353	VWA8	-0.147366	-0.534679	-0.239577	-0.387313	-0.092211	-0.239762	down
A_33_P3668839	LOC644656	0.0425491	-0.147573	-0.246922	-0.190122	-0.289471	-0.239796	down
A_33_P3307775	DENR	-1.485335	-1.929033	-1.521244	-0.443698	-0.035909	-0.239803	down
A_22_P00016650	lnc-TP53I11-6	-2.831659	-3.292388	-2.850582	-0.46073	-0.018923	-0.239826	down
A_23_P359897	KDM7A	-0.728942	-0.871097	-1.066624	-0.142155	-0.337682	-0.239918	down
A_23_P122615	PNISR	-0.272969	-0.458741	-0.567066	-0.185772	-0.294097	-0.239935	down
A_23_P200325	RABGAP1L	3.3131485	2.9114232	3.234828	-0.401725	-0.078321	-0.240023	down
A_23_P140069	FBXL3	1.7405043	1.6834955	1.3172579	-0.057009	-0.423246	-0.240128	down
A_33_P3421365	ZNF169	0.7771964	0.515842	0.5582256	-0.261354	-0.218971	-0.240163	down
A_23_P25868	NAA30	2.6160965	2.325582	2.4262276	-0.290514	-0.189869	-0.240192	down
A_21_P0000553	MTERF4	-0.641448	-1.048645	-0.71465	-0.407197	-0.073202	-0.240199	down
A_23_P155666	NAAA	-1.170737	-1.480397	-1.341553	-0.30966	-0.170815	-0.240238	down
A_23_P373119	HMGB3P1	3.483492	3.1518836	3.334509	-0.331608	-0.148983	-0.240296	down
A_23_P302654	CEP72	1.6271467	1.5187211	1.2548366	-0.108426	-0.37231	-0.240368	down
A_24_P94651	ARIH2	3.7324715	3.29878	3.685255	-0.433692	-0.047216	-0.240454	down
A_33_P3296777	PSMF1	-0.658532	-0.897008	-0.901217	-0.238476	-0.242685	-0.240581	down
A_23_P127676	CTR9	1.9023113	1.8233285	1.4999042	-0.078983	-0.402407	-0.240695	down
A_33_P3271755	SIRT5	-0.832361	-1.407455	-0.738729	-0.575094	0.0936322	-0.240731	down
A_21_P0000686	BACE1-AS	-2.854881	-3.222212	-2.969058	-0.367332	-0.114177	-0.240754	down
A_23_P250404	RAD50	1.9395905	1.787065	1.6101918	-0.152525	-0.329399	-0.240962	down
A_32_P161762	RUNX2	1.0321684	0.6967907	0.8855925	-0.335378	-0.146576	-0.240977	down
A_24_P944040	RRP1B	1.7071733	1.3847451	1.5473461	-0.322428	-0.159827	-0.241128	down
A_19_P00315753	LINC00883	-0.237981	-0.541592	-0.416791	-0.303611	-0.17881	-0.241211	down
A_33_P3245575	COLGALT1	0.4678431	0.4102159	0.0430474	-0.057627	-0.424796	-0.241211	down
A_33_P3269338	HAUS5	3.3796902	3.1800742	3.0968332	-0.199616	-0.282857	-0.241236	down
A_24_P239076	IGLL1	-0.014467	-0.216994	-0.294752	-0.202527	-0.280285	-0.241406	down
A_32_P22257	HMGN1	4.2338486	3.991014	3.9938374	-0.242835	-0.240011	-0.241423	down
A_33_P3318746	CNIH4	1.8109446	1.2811131	1.8577805	-0.529831	0.0468359	-0.241498	down
A_33_P3325110	SOX13	1.8526936	1.5506768	1.6717114	-0.302017	-0.180982	-0.241499	down
A_21_P0005461	LOC102723672	-0.793593	-0.924296	-1.145895	-0.130703	-0.352302	-0.241503	down
A_23_P98310	CSTF3	2.8478756	2.5073686	2.7053432	-0.340507	-0.142532	-0.24152	down
A_24_P366082	MICAL3	1.3752241	1.0512466	1.2159829	-0.323977	-0.159241	-0.241609	down
A_33_P3234697	LXN	3.3964415	3.1086726	3.2009382	-0.287769	-0.195503	-0.241636	down
A_23_P429082	SPTY2D1	-0.024067	-0.280627	-0.250829	-0.25656	-0.226761	-0.241661	down
A_23_P57306	CHAF1B	2.8677416	2.7593226	2.4924412	-0.108419	-0.3753	-0.24186	down
A_23_P69826	DHX15	4.25191	4.0395627	3.9805222	-0.212348	-0.271388	-0.241868	down
A_23_P47885	LRIG3	1.1330004	0.8174758	0.9644852	-0.315525	-0.168515	-0.24202	down
A_19_P00316857	LINC00969	0.6081071	0.49615	0.2358766	-0.111957	-0.372231	-0.242094	down
A_33_P3289705	GOLGB1	1.5568161	1.2862468	1.3428655	-0.270569	-0.213951	-0.24226	down
A_33_P3229272	BCAR1	-2.403481	-2.587866	-2.70369	-0.184385	-0.300209	-0.242297	down
A_24_P175460	CAMSAP1	0.1378179	0.1279035	-0.336975	-0.009914	-0.474793	-0.242354	down
A_33_P3233659	RPP30	0.0010214	-0.244505	-0.238165	-0.245526	-0.239186	-0.242356	down
A_33_P3316293	CAB39	1.3004861	1.0457935	1.0701871	-0.254693	-0.230299	-0.242496	down
A_24_P212443	UBE3C	-0.690225	-0.89216	-0.973304	-0.201935	-0.283079	-0.242507	down
A_22_P00005330	KANK1	1.1810446	0.8217201	1.0553436	-0.359324	-0.125701	-0.242513	down
A_33_P3242508	ZNF365	-0.894967	-1.577274	-0.697767	-0.682307	0.1971998	-0.242553	down
A_23_P166135	XRN2	1.774848	1.5378408	1.5266986	-0.237007	-0.248149	-0.242578	down
A_23_P317184	LRRFIP2	-0.018549	-0.222187	-0.30012	-0.203638	-0.281571	-0.242604	down
A_23_P121120	GPR87	0.865098	0.6822434	0.5624909	-0.182855	-0.302607	-0.242731	down
A_23_P51487	GBP3	2.1921396	1.9170566	1.9817581	-0.275083	-0.210382	-0.242732	down
A_33_P3283420	RBM12B	-0.415278	-0.429856	-0.886184	-0.014578	-0.470906	-0.242742	down
A_23_P98963	CCNT1	2.4366207	2.0951838	2.2925682	-0.341437	-0.144053	-0.242745	down
A_24_P354337	ARL6	-1.454166	-1.958351	-1.43572	-0.504185	0.018446	-0.242869	down
A_22_P00012243	lnc-PPM1D-1	-1.227269	-1.434421	-1.506058	-0.207152	-0.278789	-0.24297	down
A_21_P0011262	XPO6	-1.561686	-2.161298	-1.448052	-0.599613	0.1136336	-0.242989	down

A_33_P3224380	DLG1	3.9795952	3.809895	3.663187	-0.1697	-0.316408	-0.243054	down
A_23_P124108	ITGAM	-2.648832	-2.778415	-3.00539	-0.129584	-0.356559	-0.243071	down
A_21_P0003882	lnc-CTBP1-1	6.2113886	6.091295	5.845254	-0.120094	-0.366135	-0.243114	down
A_32_P93391	NUDT19	2.3265085	2.2078252	1.9587307	-0.118683	-0.367778	-0.243231	down
A_24_P238333	NSA2	4.702099	4.44043	4.4772215	-0.261669	-0.224877	-0.243273	down
A_23_P1145	MTPAP	2.4093742	2.1190352	2.212924	-0.290339	-0.19645	-0.243395	down
A_24_P56363	CAB39L	2.4175262	2.1656108	2.182581	-0.251915	-0.234945	-0.24343	down
A_23_P34578	GNL2	4.516577	4.0957904	4.4504395	-0.420786	-0.066137	-0.243462	down
A_33_P3380056	MSANTD3	-0.06529	-0.289144	-0.328375	-0.223854	-0.263085	-0.243469	down
A_24_P941441	GNA13	3.056548	2.9490018	2.6771154	-0.107546	-0.379433	-0.24349	down
A_33_P3456341	LARP4	0.1791048	-0.072845	-0.055951	-0.25195	-0.235056	-0.243503	down
A_24_P127928	RAB3GAP2	3.5198383	3.2405543	3.3120298	-0.279284	-0.207809	-0.243546	down
A_33_P3390027	EXOC7	1.3438578	0.9531436	1.247314	-0.390714	-0.096544	-0.243629	down
A_33_P3813684	FLJ31662	2.4878445	2.1042514	2.3841705	-0.383593	-0.103674	-0.243634	down
A_23_P417331	RPS6KA3	1.2859678	1.1647124	0.9198976	-0.121255	-0.36607	-0.243663	down
A_22_P00006567	lnc-FLYWCH2-1	0.5353851	0.2857671	0.2976437	-0.249618	-0.237741	-0.24368	down
A_23_P200260	PCNXL2	2.3507252	2.2040133	2.0098028	-0.146712	-0.340922	-0.243817	down
A_24_P363100	RGMB	-2.248578	-2.549199	-2.435831	-0.300621	-0.187253	-0.243937	down
A_33_P3413083	RHEB	2.641818	2.4979262	2.2978058	-0.143892	-0.344012	-0.243952	down
A_33_P3234989	IFT81	1.326026	1.0392327	1.124814	-0.286793	-0.201212	-0.244003	down
A_23_P68610	TPX2	4.0032597	3.5172858	4.0012207	-0.485974	-0.002039	-0.244006	down
A_22_P00013824	lnc-RPGRIP1-1	-2.084414	-2.534043	-2.123376	-0.449629	-0.038962	-0.244295	down
A_24_P93754	KDM4C	-2.090446	-2.340128	-2.329474	-0.249682	-0.239029	-0.244356	down
A_21_P0000485	SNORD77	-1.546516	-1.71269	-1.869087	-0.166174	-0.322571	-0.244373	down
A_21_P0009332	lnc-COL1A1-4	-2.54825	-2.234291	-3.351074	0.3139582	-0.802824	-0.244433	down
A_32_P32653	SENP5	4.210802	3.768497	4.1639643	-0.442305	-0.046838	-0.244571	down
A_24_P377277	HSPA4	4.708544	4.323767	4.604088	-0.384777	-0.104456	-0.244616	down
A_23_P16915	QPCT	2.6689901	2.5315843	2.3171215	-0.137406	-0.351869	-0.244637	down
A_23_P42257	IER3	4.9255457	4.831393	4.5303993	-0.094153	-0.395146	-0.24465	down
A_33_P3411414	FAM156B	-0.192403	-0.704697	-0.169487	-0.512293	0.0229168	-0.244688	down
A_33_P3306948	LRP6	2.913703	2.6181054	2.719717	-0.295598	-0.193986	-0.244792	down
A_24_P201064	PPP1R3B	0.469027	0.2264295	0.2218804	-0.242598	-0.247147	-0.244872	down
A_33_P3266873	MSL1	5.106146	4.910127	4.812294	-0.196019	-0.293852	-0.244935	down
A_33_P3350828	EPS15L1	1.7903547	1.3520703	1.738759	-0.438284	-0.051596	-0.24494	down
A_24_P916496	PRKCA	2.1127367	1.715909	2.0195026	-0.396828	-0.093234	-0.245031	down
A_24_P372553	EDRF1	0.0688591	-0.300963	-0.051805	-0.369822	-0.120664	-0.245243	down
A_23_P115597	ANKRD26	-1.48735	-1.747315	-1.718121	-0.259964	-0.230771	-0.245368	down
A_24_P206047	SLC25A4	3.846465	3.517696	3.6842604	-0.328769	-0.162205	-0.245487	down
A_24_P130363	LDLRAD4	-2.821774	-2.675976	-3.458552	0.1457973	-0.636778	-0.245491	down
A_23_P133284	RIOK2	2.532999	2.2024302	2.3725605	-0.330569	-0.160439	-0.245504	down
A_21_P0011142	NAP1L1	5.1029043	5.1045156	4.610236	0.0016112	-0.492668	-0.245528	down
A_23_P24784	TNNI2	3.1254644	2.7911477	2.9686852	-0.334317	-0.156779	-0.245548	down
A_24_P363679	ATP6V1H	0.1877031	-0.161371	0.0455613	-0.349074	-0.142142	-0.245608	down
A_23_P19084	HNRNPAB	4.246106	3.807344	4.1935453	-0.438762	-0.052561	-0.245662	down
A_23_P35467	CISD1	5.229291	4.9178863	5.0492887	-0.311405	-0.180002	-0.245703	down
A_23_P128084	ITGA7	1.4331436	1.2682853	1.1064239	-0.164858	-0.32672	-0.245789	down
A_23_P57588	GTSE1	2.07201	1.6884274	1.9638205	-0.383583	-0.10819	-0.245886	down
A_24_P280983	HOXA11-AS	0.4076476	0.0946512	0.2286806	-0.312996	-0.178967	-0.245982	down
A_23_P401014	ARIH1	0.9322238	0.4342794	0.9381847	-0.497944	0.0059609	-0.245992	down
A_33_P3303086	HNRNPLL	2.6200857	2.35045	2.3974895	-0.269636	-0.222596	-0.246116	down
A_33_P3404448	SNRNP48	-0.051055	-0.23265	-0.361733	-0.181595	-0.310678	-0.246136	down
A_23_P162199	PIP4K2C	0.7867503	0.3232188	0.7578955	-0.463532	-0.028855	-0.246193	down
A_23_P420256	C2orf43	1.351192	1.2872539	0.9226375	-0.063938	-0.428555	-0.246246	down
A_24_P408457	NAGA	-1.195523	-1.463535	-1.420138	-0.268012	-0.224615	-0.246313	down
A_23_P1552	CTSC	1.1945438	0.7792711	1.1170559	-0.415273	-0.077488	-0.24638	down
A_32_P140139	F13A1	-2.219039	-2.300103	-2.630932	-0.081065	-0.411893	-0.246479	down

A_33_P3269879	FAM160B2	-1.465376	-1.981172	-1.44265	-0.515795	0.0227265	-0.246534	down
A_24_P332739	GUSBP1	2.9167795	2.7324166	2.6079435	-0.184363	-0.308836	-0.246599	down
A_22_P00025545	CECR7	-1.230286	-1.573939	-1.380036	-0.343653	-0.14975	-0.246702	down
A_21_P0001371	ZNF717	-2.072877	-2.363092	-2.2763	-0.290215	-0.203423	-0.246819	down
A_23_P144202	EEFSEC	-1.19634	-1.810019	-1.076398	-0.613678	0.1199417	-0.246868	down
A_21_P0004517	TMEM161B-AS1	-0.861277	-1.240807	-0.975555	-0.37953	-0.114278	-0.246904	down
A_23_P384329	DENND4A	-2.973327	-3.305504	-3.135024	-0.332176	-0.161697	-0.246937	down
A_23_P368393	SF3B1	1.9015961	1.6786747	1.6303849	-0.222921	-0.271211	-0.247066	down
A_33_P3372924	ADCK3	-2.168017	-2.423019	-2.407269	-0.255003	-0.239252	-0.247127	down
A_24_P231173	GIGYF1	-1.819947	-1.491807	-2.642618	0.3281403	-0.82267	-0.247265	down
A_23_P204609	VEZT	2.8833961	2.6958547	2.5763807	-0.187541	-0.307015	-0.247278	down
A_24_P297098	PHF20	2.092002	1.839056	1.8503819	-0.252946	-0.24162	-0.247283	down
A_33_P7278111	LAPTM4B	5.9050617	5.574635	5.740794	-0.330427	-0.164268	-0.247347	down
A_33_P3370634	CNIH1	0.8911443	0.6627936	0.6247597	-0.228351	-0.266385	-0.247368	down
A_33_P3265104	PITRM1	0.8069472	0.7456226	0.3735323	-0.061325	-0.433415	-0.24737	down
A_21_P0012208	lnc-C21orf58-1	-0.790212	-1.015042	-1.060156	-0.22483	-0.269944	-0.247387	down
A_33_P3280003	HERC2	-0.491521	-0.824387	-0.653452	-0.332866	-0.161932	-0.247399	down
A_23_P130089	IFT20	1.8007116	1.0883751	2.0179996	-0.712337	0.217288	-0.247524	down
A_32_P131143	CECR5-AS1	-0.803318	-1.031961	-1.069779	-0.228644	-0.266462	-0.247553	down
A_23_P66732	GSG2	-0.650633	-0.905859	-0.890515	-0.255225	-0.239882	-0.247553	down
A_21_P0000393	SNORD99	0.5246878	0.2457075	0.3083892	-0.27898	-0.216299	-0.247639	down
A_33_P3392245	FAM178A	-2.679352	-3.124491	-2.729674	-0.445138	-0.050322	-0.24773	down
A_33_P3235690	THOC2	0.4888005	0.2499294	0.2317824	-0.238871	-0.257018	-0.247945	down
A_33_P3234571	MAP2K3	0.4761415	-0.050241	0.5066004	-0.526383	0.0304589	-0.247962	down
A_23_P390148	RALGPS1	0.0743747	-0.115862	-0.231431	-0.190237	-0.305806	-0.248022	down
A_24_P303589	CUL1	1.744484	1.4780416	1.5145841	-0.266442	-0.2299	-0.248171	down
A_33_P3266265	ABHD17B	3.2646904	2.6454701	3.3874788	-0.61922	0.1227884	-0.248216	down
A_33_P3886707	PPA2	6.234522	6.039243	5.93336	-0.195279	-0.301162	-0.24822	down
A_23_P59397	RSPH3	2.2060728	1.9086366	2.0067377	-0.297436	-0.199335	-0.248386	down
A_23_P103672	NES	-0.836715	-1.169435	-1.000917	-0.33272	-0.164203	-0.248461	down
A_23_P80008	MYLK2	-0.387115	-0.643057	-0.628499	-0.255943	-0.241384	-0.248663	down
A_33_P3390357	SPIRE1	2.6176348	2.5705028	2.1673937	-0.047132	-0.450241	-0.248687	down
A_33_P3209866	KANK1	1.2206888	0.7816687	1.162272	-0.43902	-0.058417	-0.248719	down
A_32_P456537	TCP1	3.3263168	3.162106	2.993042	-0.164211	-0.333275	-0.248743	down
A_33_P3557274	LOC100996924	-0.197817	-0.430921	-0.462262	-0.233104	-0.264445	-0.248775	down
A_23_P166508	LINC01315	3.0193405	2.4869266	3.0542011	-0.532414	0.0348606	-0.248777	down
A_33_P3234643	TMCO3	-0.9922	-0.996525	-1.485452	-0.004325	-0.493252	-0.248789	down
A_23_P78134	EMC6	5.126279	4.3637595	5.3910656	-0.762519	0.2647867	-0.248866	down
A_23_P109143	PRNP	4.5478506	4.384668	4.2131786	-0.163183	-0.334672	-0.248927	down
A_21_P0011877	LOC102725407	-1.10714	-1.346787	-1.365515	-0.239647	-0.258375	-0.249011	down
A_23_P56798	ACP1	4.763794	4.4136653	4.615796	-0.350129	-0.147998	-0.249063	down
A_33_P3371402	FAM223A	-2.653958	-2.86479	-2.941306	-0.210832	-0.287347	-0.24909	down
A_24_P7594	APOL6	-1.368365	-1.652813	-1.582106	-0.284449	-0.213741	-0.249095	down
A_23_P408285	PRICKLE1	1.0724883	0.9265833	0.7196355	-0.145905	-0.352853	-0.249379	down
A_23_P22214	PRKAR2A	-1.400437	-1.858948	-1.44076	-0.458511	-0.040323	-0.249417	down
A_33_P3370812	TBC1D1	0.8032622	0.4774914	0.629992	-0.325771	-0.17327	-0.249521	down
A_22_P00007340	LOC100506314	-2.819399	-3.225916	-2.912015	-0.406517	-0.092617	-0.249567	down
A_21_P0002445	lnc-BCL11A-1	-2.461587	-2.879955	-2.542462	-0.418368	-0.080875	-0.249622	down
A_33_P3394727	KHDRBS3	-0.909213	-1.231031	-1.086643	-0.321818	-0.17743	-0.249624	down
A_24_P361158	ZNF839	-1.067161	-1.090219	-1.543352	-0.023058	-0.476192	-0.249625	down
A_33_P3369190	PAM	2.298664	2.0922542	2.0056753	-0.20641	-0.292989	-0.249699	down
A_23_P20275	PLEKHF2	3.8057709	3.6018233	3.5102158	-0.203948	-0.295555	-0.249751	down
A_22_P00023023	FLJ32255	-1.658289	-1.861422	-1.954846	-0.203133	-0.296557	-0.249845	down
A_23_P348911	LINC01126	-1.443597	-1.459177	-1.92787	-0.01558	-0.484273	-0.249926	down
A_24_P317450	OSTCP1	1.7810526	1.5664406	1.495728	-0.214612	-0.285325	-0.249968	down
A_21_P0007176	lnc-CTR9-5	-1.599544	-1.595929	-2.103154	0.0036144	-0.503611	-0.249998	down

A_33_P3333805	ZNF337	-1.151493	-1.596368	-1.206688	-0.444875	-0.055196	-0.250036	down
A_24_P11791	KPNA4	5.4747143	5.2740884	5.1751986	-0.200626	-0.299516	-0.250071	down
A_32_P61684	PAG1	-1.78225	-1.750658	-2.31404	0.0315919	-0.531791	-0.250099	down
A_33_P3238993	AGFG1	1.8858452	1.7355504	1.5358319	-0.150295	-0.350013	-0.250154	down
A_33_P3345186	CHST13	-2.162348	-2.189492	-2.635531	-0.027144	-0.473182	-0.250163	down
A_22_P00000407	lnc-AC040934.1-3	-2.876105	-3.118109	-3.134735	-0.242004	-0.25863	-0.250317	down
A_23_P501634	BTN2A1	-0.482713	-0.857748	-0.608362	-0.375035	-0.125649	-0.250342	down
A_23_P105833	BIVM	0.4763327	-0.036983	0.4884548	-0.513315	0.0121222	-0.250597	down
A_24_P319942	SSR3	1.2863469	1.0163116	1.0550146	-0.270035	-0.231332	-0.250684	down
A_33_P3402526	CSGALNACT2	-1.394913	-1.518409	-1.772984	-0.123496	-0.378071	-0.250783	down
A_24_P192197	WRNIP1	1.1414995	0.830348	0.9509673	-0.311152	-0.190532	-0.250842	down
A_21_P0000302	SNORA8	1.6700182	1.3303847	1.5079055	-0.339633	-0.162113	-0.250873	down
A_19_P00811048	ZEB1-AS1	-1.141697	-1.398218	-1.387036	-0.256521	-0.245339	-0.25093	down
A_24_P413126	PMEPA1	6.8705883	6.6196923	6.619624	-0.250896	-0.250964	-0.25093	down
A_23_P211207	ADARB1	0.9961515	0.793438	0.6969543	-0.202713	-0.299197	-0.250955	down
A_22_P00012830	TMEM161B-AS1	1.4756317	1.0508566	1.3984551	-0.424775	-0.077177	-0.250976	down
A_23_P401904	PHF19	4.854451	4.3436513	4.863167	-0.5108	0.0087156	-0.251042	down
A_22_P00017804	ZBTB20	1.6165571	1.6741114	1.0568371	0.0575542	-0.55972	-0.251083	down
A_33_P3416366	CHAF1A	0.2046132	-0.077753	-0.015244	-0.282366	-0.219857	-0.251111	down
A_24_P271014	PPP4R1	0.2542248	0.0511794	-0.045191	-0.203045	-0.299416	-0.25123	down
A_19_P00318174	LOC100506014	-1.67413	-1.964159	-1.886708	-0.290029	-0.212578	-0.251303	down
A_23_P209360	KLHL29	1.8213301	1.7475839	1.3924026	-0.073746	-0.428927	-0.251337	down
A_19_P00321361	LINC-PINT	-1.455658	-1.303236	-2.110897	0.152422	-0.655239	-0.251409	down
A_23_P354805	KLF12	2.0431404	1.9240775	1.6592889	-0.119063	-0.383852	-0.251457	down
A_23_P165707	LYG1	-0.275414	-0.432488	-0.621435	-0.157074	-0.346021	-0.251547	down
A_23_P436117	HMG20A	0.2657852	-0.052361	0.0807858	-0.318146	-0.184999	-0.251573	down
A_33_P3387621	RHPN2	5.6776104	5.3358502	5.5160303	-0.34176	-0.16158	-0.25167	down
A_23_P133095	RAPGEF2	3.2523565	3.14601	2.8550625	-0.106347	-0.397294	-0.25182	down
A_23_P308097	PPP2CB	2.2821817	1.9872813	2.0734205	-0.2949	-0.208761	-0.251831	down
A_23_P143047	ATP6V1E2	1.5454044	1.1296096	1.4575038	-0.415795	-0.087901	-0.251848	down
A_32_P31618	GSR	1.0880117	0.9229159	0.7493024	-0.165096	-0.338709	-0.251903	down
A_23_P112531	FAM102A	5.9779177	5.6894994	5.7623854	-0.288418	-0.215532	-0.251975	down
A_22_P00013798	LOC101928433	-2.682881	-2.508271	-3.361491	0.1746099	-0.67861	-0.252	down
A_32_P49844	RHOQ	3.033575	2.929833	2.6332912	-0.103742	-0.400284	-0.252013	down
A_32_P95462	STXBP5L	-2.700618	-2.84612	-3.059259	-0.145502	-0.358642	-0.252072	down
A_24_P33444	YWHAE	1.034668	0.3810139	1.1840429	-0.653654	0.149375	-0.25214	down
A_23_P218358	CDRT1	-0.632504	-0.189563	-1.580037	0.4429412	-0.947533	-0.252296	down
A_32_P28685	SNRPA1	4.0708523	3.7127938	3.9242058	-0.358058	-0.146647	-0.252352	down
A_33_P3350892	LOC651337	-2.531991	-2.761059	-2.807842	-0.229068	-0.275851	-0.25246	down
A_23_P324994	KLHL7	-0.259419	-0.599636	-0.424173	-0.340217	-0.164754	-0.252485	down
A_21_P0010750	LOC102725199	0.254499	0.0697165	-0.065737	-0.184783	-0.320236	-0.252509	down
A_23_P72961	PRPS1	1.9557123	1.8522058	1.5540242	-0.103507	-0.401688	-0.252597	down
A_23_P85800	CD52	-1.699747	-2.837718	-1.066999	-1.137971	0.6327486	-0.252611	down
A_24_P238143	LRRC37A2	0.5218482	0.2695189	0.2687674	-0.252329	-0.253081	-0.252705	down
A_33_P3273272	ZNF701	-1.974171	-2.275029	-2.178811	-0.300858	-0.20464	-0.252749	down
A_33_P3327200	ATP2B4	-0.478998	-0.655856	-0.807845	-0.176858	-0.328847	-0.252853	down
A_23_P327069	KIAA0232	1.3714743	1.185813	1.051322	-0.185661	-0.320152	-0.252907	down
A_23_P72568	SNX4	1.9452105	1.7592373	1.6251884	-0.185973	-0.320022	-0.252998	down
A_32_P109794	STYX	2.7727423	2.5818572	2.4574518	-0.190885	-0.31529	-0.253088	down
A_22_P00015190	LOC101929441	-2.417734	-2.826282	-2.515577	-0.408548	-0.097843	-0.253196	down
A_23_P63681	IDE	2.1859398	1.8049579	2.060132	-0.380982	-0.125808	-0.253395	down
A_33_P3249354	SPDL1	0.6527166	0.4085193	0.3896275	-0.244197	-0.263089	-0.253643	down
A_21_P0009685	TMEM167A	3.344348	2.7842965	3.3970976	-0.560051	0.0527496	-0.253651	down
A_23_P149975	FAM107B	2.9764242	2.9547477	2.4906006	-0.021677	-0.485824	-0.25375	down
A_24_P5305	SPG7	1.0146341	0.7018161	0.819941	-0.312818	-0.194693	-0.253756	down
A_23_P359854	BEND3	2.8236656	2.4695334	2.6702747	-0.354132	-0.153391	-0.253762	down

A_24_P383076	AASDH	-0.918085	-1.114773	-1.229028	-0.196689	-0.310944	-0.253816	down
A_33_P3357620	PAGR1	1.2701745	1.020926	1.0116911	-0.249249	-0.258483	-0.253866	down
A_33_P3224891	LOC729275	-1.965651	-2.32915	-2.11	-0.3635	-0.144349	-0.253924	down
A_33_P3381666	ABLIM2	-0.958499	-1.299856	-1.125359	-0.341358	-0.16686	-0.254109	down
A_22_P00014451	lnc-SH3BGRL2-1	-0.103717	-0.623305	-0.092432	-0.519588	0.0112848	-0.254152	down
A_24_P566701	SKP1	3.720273	3.464992	3.467085	-0.255281	-0.253188	-0.254235	down
A_24_P191664	GOLIM4	2.4357033	1.9240623	2.438857	-0.511641	0.0031538	-0.254244	down
A_24_P113221	ZNF24	2.5814066	2.2481408	2.406064	-0.333266	-0.175343	-0.254304	down
A_33_P3369550	SRSF11	3.8351908	3.567329	3.5943604	-0.267862	-0.24083	-0.254346	down
A_33_P3235034	PP13	-1.244172	-1.304007	-1.693056	-0.059834	-0.448884	-0.254359	down
A_33_P3234855	UTRN	-1.899458	-2.216765	-2.090877	-0.317307	-0.191418	-0.254362	down
A_24_P916378	HNRNPLL	-0.349762	-0.562731	-0.645645	-0.212968	-0.295883	-0.254426	down
A_23_P12572	CASP7	3.8629866	3.4326482	3.7842102	-0.430338	-0.078776	-0.254557	down
A_23_P53370	RND1	-2.264362	-2.424577	-2.614168	-0.160215	-0.349805	-0.25501	down
A_22_P00011907	lnc-PIK3R1-5	-1.176937	-1.19799	-1.666102	-0.021054	-0.489165	-0.25511	down
A_33_P3220445	SMAD5	1.1138496	0.9258528	0.7915154	-0.187997	-0.322334	-0.255166	down
A_23_P162874	HSP90AA1	6.7610464	6.332359	6.67937	-0.428688	-0.081676	-0.255182	down
A_24_P97836	RAD17	-1.857555	-1.877881	-2.347865	-0.020325	-0.490309	-0.255317	down
A_33_P3300965	HOXC6	3.3110542	3.1914897	2.9199	-0.119565	-0.391154	-0.255359	down
A_33_P3380932	ZNF800	-1.83046	-2.362975	-1.808838	-0.532515	0.0216217	-0.255447	down
A_32_P840463	TIMM23B	0.5259581	0.038589	0.5022802	-0.487369	-0.023678	-0.255523	down
A_32_P163169	VDAC1	1.8942547	1.8009934	1.4763494	-0.093261	-0.417905	-0.255583	down
A_23_P23719	VPS45	1.3391881	1.1604338	1.0067501	-0.178754	-0.332438	-0.255596	down
A_33_P3339066	RNPC3	2.1098423	1.9122744	1.7961216	-0.197568	-0.313721	-0.255644	down
A_21_P0011529	RPS6KB1	-0.372675	-0.424152	-0.8325	-0.051477	-0.459825	-0.255651	down
A_32_P230547	DOCK7	2.2704191	1.9770722	2.0524569	-0.293347	-0.217962	-0.255655	down
A_24_P288722	CASK	0.4047618	0.0894756	0.2086868	-0.315286	-0.196075	-0.255681	down
A_23_P113793	ZBED2	6.1194944	5.6901584	6.0372686	-0.429336	-0.082226	-0.255781	down
A_33_P3337981	FIG4	-1.190436	-1.215502	-1.677153	-0.025065	-0.486716	-0.255891	down
A_33_P6663603	PRIM2	-1.029428	-1.472486	-1.098462	-0.443058	-0.069035	-0.256046	down
A_33_P3328928	BMP2K	-1.272148	-1.642356	-1.414042	-0.370209	-0.141894	-0.256052	down
A_24_P583225	LINC00265	-1.87159	-1.829755	-2.425549	0.0418348	-0.553959	-0.256062	down
A_32_P229746	DNAJB6	5.426549	4.7349687	5.605895	-0.69158	0.1793461	-0.256117	down
A_33_P3293432	LRRC37A3	-0.468447	-0.62883	-0.8203	-0.160383	-0.351853	-0.256118	down
A_24_P196704	KMT2E	1.3555913	1.2529192	0.9460263	-0.102672	-0.409565	-0.256119	down
A_33_P3226202	FAM179B	1.4708266	1.2710352	1.158339	-0.199791	-0.312488	-0.25614	down
A_23_P156180	SLC22A4	-2.059054	-2.102965	-2.527566	-0.043911	-0.468513	-0.256212	down
A_19_P00316225	FAM107A	-2.576423	-2.681119	-2.984154	-0.104696	-0.407732	-0.256214	down
A_23_P110212	ACSL1	3.4321938	3.139051	3.2127686	-0.293143	-0.219425	-0.256284	down
A_33_P3329301	LOC646014	-1.131657	-1.153888	-1.62213	-0.022231	-0.490473	-0.256352	down
A_23_P166910	DNAJC13	1.9143467	1.8065214	1.5094142	-0.107825	-0.404933	-0.256379	down
A_33_P3262431	KREMEN1	-0.926387	-1.476203	-0.889342	-0.549817	0.037045	-0.256386	down
A_23_P206532	PHKB	3.81981	3.5586696	3.5680885	-0.26114	-0.251721	-0.256431	down
A_23_P6490	TNRC6B	2.5252228	2.2735353	2.2640228	-0.251688	-0.2612	-0.256444	down
A_33_P3230324	COPS8	0.26015	-0.13991	0.1473122	-0.40006	-0.112838	-0.256449	down
A_23_P258944	DNAJB9	2.4760332	2.8653436	1.5735822	0.3893104	-0.902451	-0.25657	down
A_23_P26905	POLG2	2.7990208	2.4434896	2.641366	-0.355531	-0.157655	-0.256593	down
A_23_P206441	FANCA	1.1573048	0.6537185	1.1476836	-0.503586	-0.009621	-0.256604	down
A_33_P3292679	SNX6	2.186184	1.7371135	2.122016	-0.44907	-0.064168	-0.256619	down
A_22_P00019908	lnc-WNT1-2	2.5144787	2.112308	2.403201	-0.402171	-0.111278	-0.256724	down
A_19_P00811706	LPP	-2.536978	-2.804779	-2.782734	-0.267801	-0.245756	-0.256779	down
A_21_P0014428	LOC101927372	-0.399275	-0.511016	-0.801143	-0.111741	-0.401868	-0.256805	down
A_33_P6501835	LOC101929128	-0.241286	-0.593795	-0.402602	-0.352509	-0.161315	-0.256912	down
A_21_P0010962	XLOC_l2_002204	-1.3126	-1.770268	-1.368942	-0.457668	-0.056342	-0.257005	down
A_33_P3229161	UTP14C	2.28656	1.9729371	2.0861425	-0.313623	-0.200418	-0.25702	down
A_33_P3226439	GLIDR	1.3899236	1.0979943	1.1678052	-0.291929	-0.222118	-0.257024	down

A_23_P47941	HOXC11	-2.322507	-2.344014	-2.815061	-0.021507	-0.492554	-0.257031	down
A_23_P366125	EME2	-1.665252	-1.904895	-1.939796	-0.239643	-0.274544	-0.257093	down
A_21_P0011819	lnc-ZNF2-1	-1.086198	-1.374779	-1.311926	-0.28858	-0.225728	-0.257154	down
A_24_P341019	TMEM230	1.7413273	1.4695086	1.4986944	-0.271819	-0.242633	-0.257226	down
A_23_P395555	ZNF226	2.880537	2.6854262	2.560975	-0.195111	-0.319562	-0.257336	down
A_33_P3370109	IMPA1	-1.199861	-1.696382	-1.218285	-0.496521	-0.018424	-0.257472	down
A_33_P3278318	MSRB2	-0.392252	-0.836292	-0.46316	-0.444039	-0.070908	-0.257473	down
A_23_P379746	DENND5B	-1.750833	-2.075194	-1.941448	-0.324361	-0.190615	-0.257488	down
A_33_P3280030	FRA10AC1	2.2380924	2.0627465	1.8984394	-0.175346	-0.339653	-0.257499	down
A_33_P3592015	HABP4	2.4174566	2.0993037	2.22056	-0.318153	-0.196897	-0.257525	down
A_22_P00021338	GPATCH2L	0.502111	0.1130428	0.3760233	-0.389068	-0.126088	-0.257578	down
A_19_P00318361	KIF9-AS1	-1.339541	-1.602161	-1.592123	-0.26262	-0.252582	-0.257601	down
A_23_P157268	CLDN12	1.8217325	1.9161954	1.2120547	0.0944629	-0.609678	-0.257607	down
A_21_P0012869	LOC728554	-1.524396	-1.880098	-1.683909	-0.355703	-0.159513	-0.257608	down
A_24_P405190	USP33	3.3974314	3.0020547	3.277381	-0.395377	-0.12005	-0.257714	down
A_33_P3365586	VAPB	0.2064233	-0.07759	-0.025031	-0.284013	-0.231454	-0.257733	down
A_23_P118427	MAP2K3	1.5524769	1.0059266	1.5835533	-0.54655	0.0310764	-0.257737	down
A_21_P0014534	LOC101928504	-1.640358	-2.110878	-1.685337	-0.470519	-0.044979	-0.257749	down
A_33_P7883685	TSIX	1.0487757	0.8865113	0.6954155	-0.162264	-0.35336	-0.257812	down
A_33_P3323803	PDE4DIP	-0.546817	-0.567886	-1.041393	-0.02107	-0.494576	-0.257823	down
A_23_P62807	SCCPDH	4.3351974	3.9572282	4.1974993	-0.377969	-0.137698	-0.257834	down
A_23_P5611	RIF1	0.2765975	0.0496783	-0.012583	-0.226919	-0.28918	-0.25805	down
A_32_P150632	ANXA11	-1.063666	-1.595332	-1.048139	-0.531666	0.0155272	-0.258069	down
A_33_P3283744	LOC100132249	-1.811228	-2.105485	-2.033176	-0.294257	-0.221948	-0.258102	down
A_33_P3276282	PTPRF	-0.264574	-0.59757	-0.448206	-0.332997	-0.183632	-0.258315	down
A_23_P121686	AIMP1	4.933546	4.631087	4.719102	-0.302459	-0.214444	-0.258452	down
A_33_P3275350	NCS1	0.6826782	0.2288175	0.6194801	-0.453861	-0.063198	-0.258529	down
A_33_P3358462	GLI2	-1.282204	-1.519915	-1.561596	-0.23771	-0.279392	-0.258551	down
A_22_P00004046	lnc-CHST1-4	-1.972035	-2.062676	-2.398512	-0.090641	-0.426478	-0.258559	down
A_24_P280113	IL13RA1	4.8951397	4.450937	4.822175	-0.444203	-0.072965	-0.258584	down
A_24_P73943	COX11	-0.436404	-0.789226	-0.600853	-0.352822	-0.164448	-0.258635	down
A_23_P108641	FAM136A	5.6981115	5.3048263	5.5740366	-0.393285	-0.124075	-0.25868	down
A_32_P170397	lnc-JMJD7-PLA2G4B-1	-2.41981	-2.576965	-2.780291	-0.157156	-0.360481	-0.258818	down
A_22_P00007605	lnc-HEATR1-1	-0.132517	-0.482771	-0.299974	-0.350254	-0.167457	-0.258855	down
A_23_P213863	FAM193B	-0.194414	-0.478632	-0.428046	-0.284218	-0.233632	-0.258925	down
A_32_P159400	UBR5	1.2769408	0.972352	1.0636578	-0.304589	-0.213283	-0.258936	down
A_24_P126060	DDX3X	2.4854336	2.1964498	2.256219	-0.288984	-0.229215	-0.259099	down
A_23_P360245	NUDCD2	4.8561993	4.426762	4.767288	-0.429437	-0.088911	-0.259174	down
A_33_P3389133	MINPP1	-0.710891	-1.006223	-0.934031	-0.295332	-0.223139	-0.259236	down
A_23_P387057	TUBB	3.74605	3.3705144	3.6030407	-0.375536	-0.143009	-0.259272	down
A_33_P3276153	GOLGA8J	-1.451966	-1.508336	-1.914319	-0.05637	-0.462353	-0.259361	down
A_23_P6786	TRNT1	-0.525703	-0.784745	-0.785451	-0.259042	-0.259748	-0.259395	down
A_33_P3373144	SZT2	-0.227678	-0.366199	-0.60802	-0.138521	-0.380342	-0.259432	down
A_24_P377269	HSPA4	7.0985174	6.6534286	7.024626	-0.445089	-0.073892	-0.25949	down
A_23_P138693	NMT2	0.3917117	0.090198	0.1740804	-0.301514	-0.217631	-0.259573	down
A_21_P0012458	LOC100506990	-1.349361	-1.728972	-1.489011	-0.379612	-0.139651	-0.259631	down
A_24_P136551	NPLOC4	0.135807	0.1168375	-0.365026	-0.01897	-0.500833	-0.259901	down
A_23_P217637	TIMM8A	3.3822489	2.9296622	3.3145542	-0.452587	-0.067695	-0.260141	down
A_23_P111361	PPIL6	-1.911279	-2.333306	-2.009577	-0.422027	-0.098298	-0.260162	down
A_33_P3400224	DZIP1	-1.103222	-1.410748	-1.316153	-0.307525	-0.21293	-0.260228	down
A_23_P166663	APPL1	2.5868359	2.3332162	2.3197927	-0.25362	-0.267043	-0.260331	down
A_23_P113656	TUBB4A	-2.448188	-2.845172	-2.572135	-0.396985	-0.123948	-0.260466	down
A_23_P157628	DEFB4A	-2.509523	-2.783189	-2.756808	-0.273666	-0.247286	-0.260476	down
A_23_P104798	IL18	3.32646	3.316401	2.8154612	-0.010059	-0.510999	-0.260529	down
A_22_P00020359	CTTNBP2NL	0.4638314	0.3182831	0.0882955	-0.145548	-0.375536	-0.260542	down

A_24_P901986	LOC102725300	-1.189431	-1.552709	-1.3473	-0.363277	-0.157869	-0.260573	down
A_23_P25974	TTC7B	0.6674838	0.4264374	0.3872795	-0.241046	-0.280204	-0.260625	down
A_23_P10518	TFDP3	-1.697721	-2.457737	-1.458989	-0.760017	0.2387319	-0.260642	down
A_32_P226567	UPF3A	0.584394	0.3723936	0.2749662	-0.212	-0.309428	-0.260714	down
A_23_P134755	TRPS1	2.3718405	2.0528207	2.1693926	-0.31902	-0.202448	-0.260734	down
A_32_P123088	TMED7-TICAM2	0.8717642	0.5299583	0.691905	-0.341806	-0.179859	-0.260833	down
A_23_P50591	KCNK6	0.3119488	0.098083	0.0038171	-0.213866	-0.308132	-0.260999	down
A_24_P207150	UBE3A	1.9631224	1.7365351	1.6675868	-0.226587	-0.295536	-0.261061	down
A_33_P3422330	KLHL17	2.2978354	1.9715924	2.1017761	-0.326243	-0.196059	-0.261151	down
A_23_P122906	AUTS2	3.7258244	3.4314237	3.4977417	-0.294401	-0.228083	-0.261242	down
A_23_P82941	ARFGEF1	1.6279764	1.3938842	1.339531	-0.234092	-0.288445	-0.261269	down
A_23_P102571	SLC2A4RG	6.4749365	5.9784303	6.4486103	-0.496506	-0.026326	-0.261416	down
A_33_P3269069	HS2ST1	1.3087082	1.1368399	0.9576449	-0.171868	-0.351063	-0.261466	down
A_24_P286935	ARL3	0.9764257	0.5563412	0.8733597	-0.420084	-0.103066	-0.261575	down
A_21_P0004815	FOXCUT	-1.802756	-2.093121	-2.035896	-0.290365	-0.23314	-0.261752	down
A_33_P3364646	RASGEF1B	-1.985399	-2.028333	-2.466093	-0.042934	-0.480694	-0.261814	down
A_23_P43726	NUP160	3.4140892	3.1650329	3.1394854	-0.249056	-0.274604	-0.26183	down
A_23_P118150	ARL6IP1	2.6739836	2.496961	2.3272858	-0.177022	-0.346698	-0.26186	down
A_21_P0005453	lnc-NOM1-3	-2.184274	-2.350932	-2.541423	-0.166658	-0.357148	-0.261903	down
A_21_P0012993	NAIP	-1.710677	-1.923701	-2.021661	-0.213024	-0.310984	-0.262004	down
A_33_P3267740	ZNF546	-1.58979	-1.70759	-1.996241	-0.1178	-0.406451	-0.262125	down
A_24_P70117	DTWD1	0.9581533	0.6284676	0.7635675	-0.329686	-0.194586	-0.262136	down
A_33_P3251108	SLC33A1	0.1664295	-0.100688	-0.090738	-0.267118	-0.257168	-0.262143	down
A_33_P3354539	CHURC1	-0.626347	-0.81514	-0.961921	-0.188793	-0.335575	-0.262184	down
A_23_P137097	SLC16A2	-1.20788	-1.385752	-1.554516	-0.177873	-0.346636	-0.262254	down
A_33_P3228023	SCPEP1	-2.318017	-2.904046	-2.256528	-0.586029	0.0614889	-0.26227	down
A_23_P214766	HIVEP2	2.296811	2.0449176	2.0240612	-0.251894	-0.27275	-0.262322	down
A_23_P204079	NPFF	1.1497955	1.0028844	0.772006	-0.146911	-0.37779	-0.26235	down
A_24_P8109	ANO9	-0.797036	-0.921166	-1.197693	-0.124131	-0.400658	-0.262394	down
A_23_P140450	SLC27A2	3.162693	2.7753758	3.0250778	-0.387317	-0.137615	-0.262466	down
A_24_P299996	SHQ1	-0.664225	-1.052546	-0.800839	-0.388321	-0.136614	-0.262467	down
A_33_P3228902	LOC100133306	-1.209252	-1.767217	-1.176652	-0.557965	0.0326004	-0.262682	down
A_21_P0011707	XLOC_l2_006797	-1.67623	-1.931988	-1.945888	-0.255759	-0.269658	-0.262708	down
A_33_P3414907	NPL	-2.288606	-2.573797	-2.52887	-0.285192	-0.240264	-0.262728	down
A_24_P64167	PTGS1	-2.070307	-2.500623	-2.165496	-0.430315	-0.095189	-0.262752	down
A_33_P3367565	FNBP1	1.4212313	1.133925	1.1828661	-0.287306	-0.238365	-0.262836	down
A_24_P273157	OBSCN	-1.47427	-1.753995	-1.720347	-0.279725	-0.246077	-0.262901	down
A_23_P365844	EID2	4.1008673	3.5423608	4.133544	-0.558507	0.0326767	-0.262915	down
A_23_P340722	XPOT	1.5806093	1.640914	0.9943271	0.0603046	-0.586282	-0.262989	down
A_33_P3277808	LOC101928125	-2.913553	-2.944867	-3.408418	-0.031314	-0.494865	-0.263089	down
A_24_P221575	RUFY3	-2.049405	-2.204197	-2.420805	-0.154792	-0.3714	-0.263096	down
A_33_P3288017	PDE12	2.3341703	1.880084	2.2618532	-0.454086	-0.072317	-0.263202	down
A_33_P3369286	IPO8	3.392662	3.1432385	3.1155214	-0.249424	-0.277141	-0.263282	down
A_23_P155765	HMGB2	5.3942003	5.331074	4.930685	-0.063126	-0.463515	-0.263321	down
A_32_P213330	ARHGEF28	-1.254002	-1.463841	-1.570872	-0.209839	-0.316871	-0.263355	down
A_33_P3327673	COBL	-2.030157	-2.567074	-2.020049	-0.536917	0.010108	-0.263405	down
A_24_P29686	ZMIZ2	2.9983234	2.7292895	2.7402668	-0.269034	-0.258057	-0.263545	down
A_21_P0014801	KLHDC4	2.4823523	2.1003356	2.3372364	-0.382017	-0.145116	-0.263566	down
A_23_P412577	ANKRD29	0.3964462	0.0823488	0.1832242	-0.314097	-0.213222	-0.26366	down
A_33_P3404331	TAB2	1.7468548	1.1468072	1.819529	-0.600048	0.0726743	-0.263687	down
A_33_P3211924	RCOR1	2.9669485	2.619821	2.7866335	-0.347127	-0.180315	-0.263721	down
A_21_P0011549	XLOC_l2_005871	-0.001922	-0.413112	-0.118196	-0.411191	-0.116275	-0.263733	down
A_33_P3234939	ZNF266	-1.052675	-1.530946	-1.101875	-0.478271	-0.049201	-0.263736	down
A_33_P3301331	CEACAM3	-0.099204	-0.529914	-0.196138	-0.43071	-0.096934	-0.263822	down
A_33_P3763846	MAPK12	-1.591831	-2.187651	-1.523763	-0.59582	0.068068	-0.263876	down
A_33_P3239736	WDR44	-0.321743	-0.664966	-0.506426	-0.343224	-0.184683	-0.263953	down

A_33_P3345796	SNX3	-2.700884	-2.739311	-3.190374	-0.038427	-0.48949	-0.263958	down
A_33_P3394517	SMG1	0.3065128	0.2247815	-0.139689	-0.081731	-0.446202	-0.263967	down
A_21_P0011462	XLOC_l2_005438	-1.166887	-1.200224	-1.661516	-0.033338	-0.494629	-0.263983	down
A_33_P3423979	PALLD	1.7413168	1.4160976	1.5383525	-0.325219	-0.202964	-0.264092	down
A_24_P306136	RIC1	-0.242744	-0.693936	-0.319948	-0.451192	-0.077204	-0.264198	down
A_33_P3297205	C22orf29	4.1312695	3.7004151	4.033347	-0.430854	-0.097922	-0.264388	down
A_33_P3330264	CXCL1	-1.118738	-1.425528	-1.341099	-0.30679	-0.222361	-0.264575	down
A_23_P202587	KIAA1598	2.431614	2.2933583	2.0405083	-0.138256	-0.391106	-0.264681	down
A_33_P3404286	KIAA0368	3.3719149	3.278666	2.935711	-0.093249	-0.436204	-0.264726	down
A_33_P3358626	TAF4B	-0.677707	-0.953344	-0.93167	-0.275638	-0.253963	-0.264801	down
A_24_P145911	TRA2B	3.2559137	2.980825	3.0013885	-0.275089	-0.254525	-0.264807	down
A_23_P385861	CDCA2	3.7515316	3.3317027	3.6417427	-0.419829	-0.109789	-0.264809	down
A_33_P3253596	KIF4A	0.0679383	-0.27212	-0.121648	-0.340059	-0.189586	-0.264822	down
A_23_P171143	TSPAN6	3.6693506	3.1069398	3.7018623	-0.562411	0.0325117	-0.26495	down
A_24_P396702	CD302	0.0045238	-0.528236	0.0073609	-0.53276	0.0028372	-0.264961	down
A_33_P3373805	LOC255187	-0.884898	-1.345528	-0.954664	-0.460629	-0.069766	-0.265198	down
A_33_P3233666	RPP30	1.5776424	1.1984363	1.4263163	-0.379206	-0.151326	-0.265266	down
A_22_P00002468	lnc-C15orf48-1	-2.434331	-2.434686	-2.964932	-0.000355	-0.530601	-0.265478	down
A_24_P268786	MYNN	1.7433672	1.3922143	1.563529	-0.351153	-0.179838	-0.265496	down
A_23_P132874	CMSS1	6.302432	6.0292697	6.044482	-0.273162	-0.25795	-0.265556	down
A_21_P0012127	XLOC_l2_008667	-0.855777	-1.302812	-0.939872	-0.447035	-0.084095	-0.265565	down
A_23_P250164	HGD	3.4160967	2.9653	3.3357496	-0.450797	-0.080347	-0.265572	down
A_33_P3400477	STIL	2.9684496	2.5891652	2.816246	-0.379284	-0.152204	-0.265744	down
A_22_P00020454	lnc-CHPF-1	-2.911551	-2.919517	-3.435343	-0.007966	-0.523793	-0.265879	down
A_24_P263672	BMS1	3.7221775	3.264443	3.6481094	-0.457735	-0.074068	-0.265901	down
A_24_P45379	CACYBP	-0.547166	-0.894582	-0.731773	-0.347416	-0.184608	-0.266012	down
A_21_P0000328	SNORA40	0.9587584	0.7164731	0.6689782	-0.242285	-0.28978	-0.266033	down
A_23_P151870	GLCE	0.9188743	0.5651517	0.7404366	-0.353723	-0.178438	-0.26608	down
A_23_P44781	CDKAL1	-0.548728	-0.733576	-0.896082	-0.184848	-0.347354	-0.266101	down
A_23_P10206	HAS2	-0.059722	-0.810109	0.15834	-0.750387	0.2180619	-0.266163	down
A_24_P82957	RNF114	1.4774947	1.4022112	1.0203118	-0.075284	-0.457183	-0.266233	down
A_33_P3262191	CPNE7	3.9491272	3.666288	3.6992607	-0.282839	-0.249866	-0.266353	down
A_23_P64204	ZW10	2.707056	2.343943	2.5371895	-0.363113	-0.169867	-0.26649	down
A_33_P3235766	QSER1	2.6609945	2.4047112	2.3842773	-0.256283	-0.276717	-0.2665	down
A_23_P151159	TCHP	0.3464904	-0.078594	0.2385669	-0.425084	-0.107924	-0.266504	down
A_33_P3334535	GIN1	0.0788188	-0.320933	-0.054484	-0.399752	-0.133303	-0.266527	down
A_23_P161624	FOSL1	1.0721412	1.1099291	0.5011568	0.0377879	-0.570984	-0.266598	down
A_23_P22433	RP2	2.1523342	1.889319	1.8819256	-0.263015	-0.270409	-0.266712	down
A_23_P131348	THUMPD2	0.8388495	0.4944167	0.649796	-0.344433	-0.189054	-0.266743	down
A_23_P162782	ARGLU1	2.9679346	2.777247	2.6250086	-0.190688	-0.342926	-0.266807	down
A_23_P92672	OCLN	0.713728	0.3287592	0.5647707	-0.384969	-0.148957	-0.266963	down
A_21_P0011308	LOC100288637	-1.195007	-1.44766	-1.47631	-0.252653	-0.281302	-0.266978	down
A_33_P3253723	AQP1	-1.108379	-1.423514	-1.327356	-0.315135	-0.218977	-0.267056	down
A_23_P84995	MTMR8	-1.990621	-2.550002	-1.965429	-0.559381	0.0251918	-0.267094	down
A_23_P88602	MEIS2	-0.340844	-0.356135	-0.85987	-0.015291	-0.519025	-0.267158	down
A_24_P301547	C17orf80	-0.323125	-0.692501	-0.488239	-0.369375	-0.165113	-0.267244	down
A_24_P201531	ARCN1	2.357254	2.035193	2.1447964	-0.322061	-0.212458	-0.267259	down
A_23_P206612	USP31	0.4164634	0.2099743	0.0882969	-0.206489	-0.328166	-0.267328	down
A_33_P3650353	SNORA33	-0.909516	-1.304406	-1.049312	-0.39489	-0.139796	-0.267343	down
A_33_P3244433	FAM188B	-2.636677	-2.942819	-2.865288	-0.306142	-0.228611	-0.267377	down
A_23_P110243	METTL14	0.6056223	0.5048943	0.1714869	-0.100728	-0.434135	-0.267432	down
A_23_P203841	BAZ2A	0.9543557	0.6734462	0.7002549	-0.28091	-0.254101	-0.267505	down
A_23_P214821	EDN1	1.0379062	0.6380882	0.9026895	-0.399818	-0.135217	-0.267517	down
A_21_P0000500	SNORD86	4.4521036	3.9281192	4.4409695	-0.523984	-0.011134	-0.267559	down
A_33_P3273969	ZC3H11A	3.2502413	2.9975247	2.9678364	-0.252717	-0.282405	-0.267561	down
A_23_P203023	RDX	-0.007205	-0.509359	-0.040232	-0.502154	-0.033027	-0.26759	down

A_33_P6476600	LOC101929128	-0.003346	-0.265108	-0.276812	-0.261761	-0.273466	-0.267613	down
A_23_P23006	NRD1	5.203727	5.0531745	4.818782	-0.150552	-0.384945	-0.267749	down
A_33_P3319491	AZIN1	5.0807953	4.784656	4.8413877	-0.296139	-0.239408	-0.267773	down
A_23_P98022	SIRT1	0.0179529	-0.069251	-0.430405	-0.087204	-0.448358	-0.267781	down
A_23_P7172	PGM2	3.284422	2.7850413	3.2481346	-0.499381	-0.036287	-0.267834	down
A_23_P433791	SPRTN	-0.747764	-1.252996	-0.778201	-0.505232	-0.030437	-0.267835	down
A_23_P130515	CEACAM3	-0.119198	-0.530381	-0.243701	-0.411183	-0.124503	-0.267843	down
A_33_P3216357	KDM5C	-2.602706	-2.7627	-2.978534	-0.159995	-0.375829	-0.267912	down
A_24_P940125	CNOT6	2.8512745	2.610066	2.556469	-0.241209	-0.294806	-0.268007	down
A_32_P34589	RSRC1	2.195056	1.9325948	1.9212627	-0.262461	-0.273793	-0.268127	down
A_33_P3400389	SPATS2L	4.3313723	4.053968	4.072462	-0.277404	-0.25891	-0.268157	down
A_23_P60227	CCIN	-1.900844	-2.191049	-2.146979	-0.290205	-0.246135	-0.26817	down
A_24_P132950	NAP1L4	0.6465235	0.4036398	0.3530049	-0.242884	-0.293519	-0.268201	down
A_33_P8086411	SMKR1	-0.322762	-0.96957	-0.212484	-0.646808	0.1102781	-0.268265	down
A_32_P231493	DOCK9-AS2	-0.601159	-1.030432	-0.708555	-0.429273	-0.107397	-0.268335	down
A_23_P29365	RYBP	-0.433023	-0.664257	-0.738764	-0.231234	-0.305741	-0.268487	down
A_33_P3325384	LOC100130992	-1.71016	-1.922226	-2.035185	-0.212066	-0.325025	-0.268545	down
A_23_P33607	TEFM	2.0039158	1.666265	1.8042345	-0.337651	-0.199681	-0.268666	down
A_33_P3401428	TMEM38B	0.0154734	-0.316734	-0.189652	-0.332208	-0.205126	-0.268667	down
A_24_P69691	ZNF25	-1.089237	-1.215349	-1.500559	-0.126112	-0.411322	-0.268717	down
A_23_P77145	RAB11A	2.4816732	2.2519393	2.173853	-0.229734	-0.30782	-0.268777	down
A_23_P391778	MACF1	4.572648	4.2903285	4.3171587	-0.28232	-0.255489	-0.268904	down
A_23_P258367	GLE1	-0.839387	-1.102059	-1.114626	-0.262672	-0.275238	-0.268955	down
A_33_P3304133	GLB1L2	1.6331167	1.4595604	1.2685895	-0.173556	-0.364527	-0.269042	down
A_33_P3323945	CEND1	-1.819959	-2.236328	-1.941723	-0.416369	-0.121764	-0.269067	down
A_33_P3389188	TFAM	5.116334	4.909495	4.7850027	-0.206839	-0.331331	-0.269085	down
A_22_P00001768	lnc-ATP13A4-4	-2.756095	-2.95658	-3.093803	-0.200485	-0.337708	-0.269097	down
A_33_P3258339	LRRC75A	-0.848718	-1.216672	-1.018963	-0.367954	-0.170245	-0.2691	down
A_23_P215819	ZNF655	0.7817903	0.5052581	0.5199046	-0.276532	-0.261886	-0.269209	down
A_23_P56553	METTL8	3.1472235	2.8569808	2.898962	-0.290243	-0.248261	-0.269252	down
A_33_P3271445	COL4A3BP	3.3942213	3.0813813	3.1684885	-0.31284	-0.225733	-0.269286	down
A_33_P3291325	INO80	-2.25186	-2.340799	-2.7015	-0.088938	-0.44964	-0.269289	down
A_23_P136460	FAM13B	1.0080695	0.7380886	0.7393737	-0.269981	-0.268696	-0.269338	down
A_24_P348861	TTTY15	-0.47646	-0.806015	-0.68564	-0.329556	-0.20918	-0.269368	down
A_24_P212481	MCTP1	-0.397478	-0.580239	-0.753481	-0.182761	-0.356003	-0.269382	down
A_33_P3248325	EPB41L5	0.5002375	0.2680111	0.1935597	-0.232226	-0.306678	-0.269452	down
A_33_P3373358	GJC1	-2.568456	-2.571583	-3.104528	-0.003127	-0.536072	-0.2696	down
A_23_P213518	CAST	5.31332	5.046428	5.04101	-0.266892	-0.27231	-0.269601	down
A_24_P935881	SERF1B	4.787978	4.3979707	4.6383934	-0.390008	-0.149585	-0.269796	down
A_23_P320739	MEF2C	0.2758761	0.1447763	-0.132783	-0.1311	-0.408659	-0.269879	down
A_21_P0011113	LOC100240735	-1.560775	-1.883519	-1.777793	-0.322744	-0.217017	-0.269881	down
A_33_P3210885	ATP7A	1.1975441	0.9007359	0.9544625	-0.296808	-0.243082	-0.269945	down
A_33_P3380063	ADAMTSL4	-0.543097	-0.735208	-0.890881	-0.192111	-0.347785	-0.269948	down
A_24_P415624	MLXIP	0.5400796	0.1515012	0.3886995	-0.388578	-0.15138	-0.269979	down
A_32_P21474	IRAK1BP1	0.1652775	-0.143033	-0.066541	-0.30831	-0.231818	-0.270064	down
A_23_P122007	C5orf30	2.731946	2.203178	2.720563	-0.528768	-0.011383	-0.270076	down
A_33_P3309919	RWDD1	4.611001	4.4143567	4.267353	-0.196644	-0.343648	-0.270146	down
A_21_P0000272	SNORD51	-2.038511	-2.273581	-2.343912	-0.235069	-0.305401	-0.270235	down
A_33_P3328154	ZNF527	-2.457548	-2.587252	-2.868577	-0.129704	-0.411029	-0.270367	down
A_33_P3285235	ZNF507	-2.335455	-2.491468	-2.720185	-0.156013	-0.384731	-0.270372	down
A_23_P126241	EIF4G3	1.4723563	1.2348485	1.1690755	-0.237508	-0.303281	-0.270394	down
A_33_P3254606	WDR62	3.8492422	3.4126086	3.7448883	-0.436634	-0.104354	-0.270494	down
A_33_P3412945	SRSF2	3.3084745	3.0362115	3.0396156	-0.272263	-0.268859	-0.270561	down
A_32_P160972	ABRACL	6.2993326	5.8780427	6.1793566	-0.42129	-0.119976	-0.270633	down
A_23_P61466	CD163L1	-0.544695	-0.867826	-0.762918	-0.323131	-0.218222	-0.270676	down
A_23_P157580	SDCBP	2.9420385	2.6540675	2.6884527	-0.287971	-0.253586	-0.270778	down

A_33_P3228072	NPHP3	0.4184675	0.2325597	0.0625849	-0.185908	-0.355883	-0.270895	down
A_23_P95930	HMGA2	1.7149305	1.2467504	1.6412072	-0.46818	-0.073723	-0.270952	down
A_23_P334282	BMP2K	-0.489221	-0.933782	-0.586712	-0.444561	-0.097491	-0.271026	down
A_22_P00023870	lnc-CARHSP1-1	-2.500516	-2.958018	-2.585125	-0.457501	-0.084609	-0.271055	down
A_23_P202594	MCMBP	4.0206347	3.7220407	3.7769184	-0.298594	-0.243716	-0.271155	down
A_22_P00014015	LOC389247	-2.192877	-2.237318	-2.69083	-0.044441	-0.497952	-0.271196	down
A_23_P64854	KRT75	-1.148552	-1.649517	-1.190062	-0.500965	-0.04151	-0.271237	down
A_33_P3400943	CDIP1	4.0356503	3.358585	4.170083	-0.677065	0.1344328	-0.271316	down
A_23_P325075	RNGTT	1.4566312	1.0980582	1.2725501	-0.358573	-0.184081	-0.271327	down
A_23_P337753	SP140L	-1.208855	-1.328914	-1.631473	-0.120059	-0.422618	-0.271338	down
A_22_P00003428	LOC102723673	-0.541236	-0.676344	-0.948821	-0.135108	-0.407585	-0.271347	down
A_24_P131392	RMDN2	-0.334036	-0.590718	-0.620186	-0.256682	-0.28615	-0.271416	down
A_24_P97526	CMTM6	2.59939	2.2688031	2.3867702	-0.330587	-0.21262	-0.271603	down
A_33_P3233160	CFAP36	2.422492	2.1182065	2.183527	-0.304286	-0.238965	-0.271625	down
A_23_P112004	LRRC6	-1.890247	-1.990432	-2.333378	-0.100185	-0.443131	-0.271658	down
A_33_P3344406	MEF2B	1.2303452	0.6708465	1.2464981	-0.559499	0.0161529	-0.271673	down
A_33_P3355208	CROT	-1.659032	-2.710645	-1.150925	-1.051613	0.5081077	-0.271752	down
A_33_P3378291	IL17REL	-2.031844	-2.241167	-2.366135	-0.209323	-0.334291	-0.271807	down
A_21_P0009781	UCA1	-2.424474	-2.773294	-2.619684	-0.34882	-0.195209	-0.272014	down
A_24_P8454	SPAG9	-0.406634	-0.645203	-0.712266	-0.238568	-0.305631	-0.2721	down
A_33_P3273298	ZNF813	0.6617417	0.074688	0.7045937	-0.587054	0.0428519	-0.272101	down
A_33_P3430391	LOC389273	-1.69989	-2.043059	-1.900966	-0.343169	-0.201076	-0.272122	down
A_24_P271049	C18orf32	-0.464519	-0.923333	-0.550027	-0.458814	-0.085508	-0.272161	down
A_22_P00016500	lnc-TMPRSS11E-1	-0.608416	-0.630044	-1.131196	-0.021629	-0.52278	-0.272204	down
A_22_P00014988	C3orf80	-1.587539	-2.17975	-1.539774	-0.592211	0.0477643	-0.272223	down
A_33_P3265355	SNRPE	6.3141766	5.980766	6.1030903	-0.333411	-0.211086	-0.272249	down
A_33_P3404546	OBSCN	-0.563588	-0.92818	-0.743869	-0.364592	-0.180281	-0.272436	down
A_21_P0003595	lnc-PCDH7-13	3.8495235	3.622644	3.5315275	-0.22688	-0.317996	-0.272438	down
A_23_P125837	PBDC1	0.9617138	0.5915599	0.7865696	-0.370154	-0.175144	-0.272649	down
A_23_P148297	SH3BGRL	3.1191607	2.7745318	2.9183989	-0.344629	-0.200762	-0.272695	down
A_23_P48166	TWF1	4.2091885	4.008575	3.8642263	-0.200614	-0.344962	-0.272788	down
A_24_P407323	PPM1A	0.575592	0.365375	0.2400937	-0.210217	-0.335498	-0.272858	down
A_33_P3284939	TMEM189	4.7530766	4.478995	4.481352	-0.274082	-0.271725	-0.272903	down
A_23_P209337	METTL21A	2.4554234	2.0709872	2.29393	-0.384436	-0.161493	-0.272965	down
A_33_P3267230	TBC1D3B	0.6433954	0.5518451	0.1890001	-0.09155	-0.454395	-0.272973	down
A_33_P3381410	ORC2	0.4124551	-0.029344	0.3080654	-0.441799	-0.10439	-0.273094	down
A_32_P121085	DOK3	-1.16227	-1.584899	-1.285923	-0.422628	-0.123653	-0.273141	down
A_33_P3305974	SNAP23	-1.770681	-2.183459	-1.90424	-0.412778	-0.133558	-0.273168	down
A_33_P3234267	LOC100130442	-2.223457	-2.357182	-2.636386	-0.133725	-0.412929	-0.273327	down
A_22_P00003484	JRKL-AS1	-2.761132	-3.02913	-3.039815	-0.267998	-0.278683	-0.27334	down
A_22_P00018413	LINC00858	-2.786721	-3.024939	-3.09532	-0.238218	-0.308599	-0.273408	down
A_23_P11295	CMC4	2.02989	1.6187625	1.8940821	-0.411128	-0.135808	-0.273468	down
A_32_P123629	TTC39C	2.7245111	2.5403867	2.361661	-0.184124	-0.36285	-0.273487	down
A_23_P413193	SFR1	3.3869705	3.2154937	3.0114079	-0.171477	-0.375563	-0.27352	down
A_23_P257538	RBBP9	1.4384999	0.9374876	1.3924122	-0.501012	-0.046088	-0.27355	down
A_32_P19752	FAM76B	1.5945816	1.5912766	1.0507584	-0.003305	-0.543823	-0.273564	down
A_24_P23522	THAP5	-1.152645	-1.547765	-1.304757	-0.39512	-0.152112	-0.273616	down
A_23_P257111	FBP1	-2.00856	-2.227543	-2.336836	-0.218983	-0.328275	-0.273629	down
A_21_P0013312	XLOC_l2_013853	-1.294427	-1.459934	-1.676191	-0.165507	-0.381764	-0.273636	down
A_33_P3405004	NFXL1	1.2904959	1.0637465	0.969923	-0.226749	-0.320573	-0.273661	down
A_23_P99579	C14orf142	-0.896598	-0.916039	-1.424593	-0.01944	-0.527994	-0.273717	down
A_24_P101114	CNOT1	0.8396211	0.520114	0.6116743	-0.319507	-0.227947	-0.273727	down
A_23_P162037	ARNTL	0.0048313	-0.446024	-0.091897	-0.450855	-0.096728	-0.273792	down
A_33_P3254751	ST6GALNAC6	-1.482372	-1.715613	-1.796756	-0.233241	-0.314384	-0.273812	down
A_23_P214544	GPX5	-2.381827	-2.845641	-2.465773	-0.463814	-0.083946	-0.27388	down
A_32_P119197	TPM3	-0.232768	-1.048913	0.0354762	-0.816145	0.2682438	-0.273951	down

A_23_P385217	ARL8B	4.877513	4.7685285	4.438568	-0.108984	-0.438945	-0.273965	down
A_21_P0014730	lnc-C7orf23-1	0.3847909	-0.018705	0.2403045	-0.403496	-0.144486	-0.273991	down
A_33_P3341716	SP4	0.2561021	0.0477891	-0.083793	-0.208313	-0.339895	-0.274104	down
A_21_P0013236	GTF2I	0.5826945	0.2214065	0.3956871	-0.361288	-0.187007	-0.274148	down
A_23_P210554	SPATA2	-0.959026	-1.367059	-1.09932	-0.408033	-0.140294	-0.274163	down
A_23_P144337	CCRN4L	-2.048504	-2.675774	-1.969563	-0.62727	0.0789413	-0.274164	down
A_22_P00022750	LOC101929901	-0.654476	-0.764205	-1.09317	-0.109729	-0.438694	-0.274211	down
A_24_P64100	SLC25A37	3.2413387	2.8192916	3.1147556	-0.422047	-0.126583	-0.274315	down
A_22_P00007632	USP3-AS1	-1.671917	-2.524311	-1.368414	-0.852395	0.3035026	-0.274446	down
A_21_P0011593	WDR55	-2.348788	-2.674557	-2.57194	-0.325768	-0.223151	-0.27446	down
A_24_P342829	SLC16A14	-0.748377	-0.85339	-1.192295	-0.105013	-0.443918	-0.274465	down
A_33_P3767927	NR3C1	4.532609	4.387666	4.1285906	-0.144943	-0.404018	-0.274481	down
A_23_P170626	CHCHD7	4.311964	4.069323	4.0055666	-0.242641	-0.306397	-0.274519	down
A_33_P3341144	BAX	0.4224134	-0.275213	0.5709686	-0.697626	0.1485553	-0.274535	down
A_33_P3247803	RBM12B	0.6625123	0.6591988	0.1167488	-0.003314	-0.545764	-0.274539	down
A_23_P158925	GPR125	3.4489918	3.121101	3.2274199	-0.327891	-0.221572	-0.274731	down
A_33_P3285911	KIAA1919	-0.201376	-0.508578	-0.443899	-0.307201	-0.242522	-0.274862	down
A_22_P00008487	lnc-KCNC2-1	-2.60581	-3.011538	-2.750004	-0.405728	-0.144194	-0.274961	down
A_21_P0005503	lnc-SBDS-2	-2.722825	-3.00199	-2.993625	-0.279165	-0.2708	-0.274983	down
A_24_P409042	CDC42SE2	4.6014223	4.2325616	4.419812	-0.368861	-0.18161	-0.275235	down
A_23_P20615	ANP32B	5.5724535	5.790834	4.803403	0.2183805	-0.769051	-0.275335	down
A_22_P00007694	lnc-HIST1H3F-1	-0.812876	-1.264917	-0.91151	-0.452042	-0.098634	-0.275338	down
A_32_P74955	ARID2	1.5317731	1.2506661	1.2619553	-0.281107	-0.269818	-0.275462	down
A_23_P155332	PCNP	4.6130114	4.3801045	4.2949	-0.232907	-0.318111	-0.275509	down
A_21_P0000487	SNORD94	-2.429433	-2.433656	-2.976317	-0.004223	-0.546884	-0.275553	down
A_23_P219013	ABCC10	0.4938183	-0.088439	0.5248547	-0.582257	0.0310364	-0.27561	down
A_33_P3306510	NUP153	2.9305334	2.4500203	2.859806	-0.480513	-0.070727	-0.27562	down
A_21_P0006594	TTTY15	0.6624222	0.410718	0.3628087	-0.251704	-0.299613	-0.275659	down
A_24_P346587	MTFR1	0.4952908	0.2465034	0.1926146	-0.248787	-0.302676	-0.275732	down
A_23_P253012	GRAMD1C	1.426228	1.1855302	1.1154165	-0.240698	-0.310812	-0.275755	down
A_21_P0001706	PLEKHO1	-2.479308	-2.76031	-2.749981	-0.281002	-0.270673	-0.275838	down
A_22_P00015661	LOC101930316	-2.176889	-2.392353	-2.513176	-0.215464	-0.336287	-0.275875	down
A_23_P148475	KIF4A	2.375104	2.0671344	2.131032	-0.30797	-0.244072	-0.276021	down
A_33_P3400152	ENTPD1-AS1	-1.253391	-1.704355	-1.354962	-0.450964	-0.101571	-0.276268	down
A_24_P208998	TRIM23	-1.784073	-1.831141	-2.28968	-0.047067	-0.505607	-0.276337	down
A_21_P0005917	LOC100507316	0.5888996	-0.031302	0.6562247	-0.620202	0.0673251	-0.276438	down
A_24_P636332	CCDC84	4.326951	4.1663337	3.9346638	-0.160617	-0.392287	-0.276452	down
A_24_P40417	FMR1	1.2853141	0.9592471	1.0584722	-0.326067	-0.226842	-0.276454	down
A_24_P185604	NDRG3	1.0434752	0.4357147	1.0982289	-0.60776	0.0547538	-0.276503	down
A_33_P3267665	ZDHHC2	3.4819307	3.264833	3.1459608	-0.217098	-0.33597	-0.276534	down
A_23_P55073	NOL11	3.6620007	3.323255	3.4476633	-0.338746	-0.214337	-0.276541	down
A_23_P151059	FAM90A1	-1.165186	-1.453202	-1.430395	-0.288017	-0.265209	-0.276613	down
A_19_P00322762	ZNF83	-0.928859	-1.235901	-1.175118	-0.307043	-0.24626	-0.276651	down
A_33_P3325018	ERLEC1	3.5010958	3.01059	3.4380589	-0.490506	-0.063037	-0.276771	down
A_23_P156445	DDX43	2.2958632	2.264027	1.7740788	-0.031836	-0.521784	-0.27681	down
A_33_P3282261	XLOC_l2_000001	-2.066462	-1.87451	-2.812088	0.1919522	-0.745626	-0.276837	down
A_23_P200801	PDE4DIP	0.0872688	-0.040613	-0.338568	-0.127882	-0.425837	-0.276859	down
A_23_P55388	KLHL11	1.981864	1.6509075	1.7590995	-0.330956	-0.222764	-0.27686	down
A_19_P00319376	UBA6-AS1	-2.569103	-3.007053	-2.684935	-0.43795	-0.115832	-0.276891	down
A_19_P00320927	LINC00942	-2.449958	-2.548663	-2.905206	-0.098705	-0.455247	-0.276976	down
A_33_P3424566	FLJ38717	-2.04587	-2.028925	-2.616776	0.0169454	-0.570906	-0.27698	down
A_21_P0010861	LINC00202-1	-1.86497	-2.062622	-2.22132	-0.197652	-0.35635	-0.277001	down
A_23_P111194	SPDEF	-1.813133	-2.289195	-1.891297	-0.476061	-0.078164	-0.277113	down
A_33_P3263841	RCHY1	-1.128422	-1.534258	-1.27687	-0.405837	-0.148448	-0.277143	down
A_24_P636882	STK17B	0.0444512	-0.252861	-0.21266	-0.297312	-0.257112	-0.277212	down
A_33_P3423874	MYZAP	2.5461216	2.2478948	2.289916	-0.298227	-0.256206	-0.277216	down

A_23_P254797	BPIFB2	-2.065567	-2.445737	-2.240118	-0.38017	-0.174551	-0.27736	down
A_22_P00011891	LOC101927326	-2.735346	-2.813528	-3.212042	-0.078182	-0.476696	-0.277439	down
A_24_P481783	UBAP1L	-1.248253	-1.500924	-1.550471	-0.252671	-0.302218	-0.277444	down
A_24_P360763	CREM	-0.618334	-0.990735	-0.800891	-0.372401	-0.182557	-0.277479	down
A_24_P167642	GCH1	-0.031747	-0.322458	-0.296003	-0.290711	-0.264256	-0.277483	down
A_33_P3360204	XLOC_l2_006958	0.3354697	-0.005655	0.1214628	-0.341125	-0.214007	-0.277566	down
A_22_P00023143	lnc-POLE3-1	-2.645915	-2.683804	-3.163174	-0.037889	-0.517259	-0.277574	down
A_33_P3267208	CSNK1G1	0.3696146	-0.04334	0.2273407	-0.412955	-0.142274	-0.277614	down
A_23_P405707	BCOR	0.0867143	-0.251209	-0.130737	-0.337923	-0.217451	-0.277687	down
A_24_P220454	CUX1	2.9287806	2.5224404	2.779519	-0.40634	-0.149261	-0.277801	down
A_21_P0005703	lnc-RAB2A-1	-0.520554	-1.005575	-0.591497	-0.485022	-0.070944	-0.277983	down
A_33_P3339231	UBN2	-0.222334	-0.397269	-0.603456	-0.174935	-0.381122	-0.278028	down
A_33_P3380783	LOC100128184	-1.95756	-2.02575	-2.44546	-0.06819	-0.487901	-0.278045	down
A_24_P664995	CBX5	3.4476566	3.2127538	3.1263514	-0.234903	-0.321305	-0.278104	down
A_24_P346126	SERF1A	4.0121174	3.5706058	3.8973846	-0.441512	-0.114733	-0.278122	down
A_24_P156288	GZF1	-0.149373	-0.500743	-0.354454	-0.35137	-0.205081	-0.278226	down
A_23_P201567	PRPF38B	1.2744651	0.9481411	1.0441928	-0.326324	-0.230272	-0.278298	down
A_24_P189533	ENDOD1	4.6734695	4.2601743	4.5300484	-0.413295	-0.143421	-0.278358	down
A_21_P0000282	SNORD56	0.4850364	0.3228664	0.0904155	-0.16217	-0.394621	-0.278395	down
A_23_P30495	HMGCR	4.5658627	4.222427	4.352497	-0.343436	-0.213366	-0.278401	down
A_23_P14255	GUCY1B2	-2.202104	-2.108896	-2.85239	0.0932074	-0.650286	-0.27854	down
A_23_P137427	ZMPSTE24	6.2097874	5.862539	5.9999523	-0.347249	-0.209835	-0.278542	down
A_33_P3289491	LOC100129069	-2.366488	-2.345699	-2.944577	0.0207891	-0.578089	-0.27865	down
A_23_P141894	PVR	-0.711365	-1.100065	-0.880101	-0.3887	-0.168736	-0.278718	down
A_24_P56317	MBNL2	3.2060146	3.1071305	2.747407	-0.098884	-0.458608	-0.278746	down
A_23_P210210	EPAS1	5.713045	5.232556	5.6360283	-0.480489	-0.077017	-0.278753	down
A_22_P00009062	lnc-LEO1-2	0.0960183	-0.043839	-0.321729	-0.139857	-0.417748	-0.278802	down
A_24_P69379	CASD1	-1.460111	-1.796836	-1.681019	-0.336726	-0.220909	-0.278817	down
A_33_P3211229	SLC35F2	0.3269329	-0.289404	0.3854561	-0.616337	0.0585232	-0.278907	down
A_23_P111343	BCLAF1	3.460453	3.0597987	3.3031254	-0.400654	-0.157328	-0.278991	down
A_33_P8814326	CATSPER2P1	9.180836	8.904018	8.899582	-0.276817	-0.281254	-0.279036	down
A_33_P3413038	PLXNB3	-0.831224	-0.990682	-1.229865	-0.159459	-0.398641	-0.27905	down
A_24_P784765	CD59	-2.033932	-2.743749	-1.882234	-0.709817	0.1516986	-0.279059	down
A_22_P00012531	lnc-PSPC1-1	-1.164661	-1.425972	-1.461621	-0.261311	-0.29696	-0.279136	down
A_33_P3296687	KRT33A	-2.166996	-2.23981	-2.652741	-0.072814	-0.485745	-0.279279	down
A_23_P214977	SEC63	3.3757734	3.2097507	2.9830523	-0.166023	-0.392721	-0.279372	down
A_33_P3901921	PARPBP	-0.827105	-1.329325	-0.883637	-0.50222	-0.056533	-0.279377	down
A_33_P3336925	PDLIM5	2.6927395	2.1240916	2.7024279	-0.568648	0.0096884	-0.27948	down
A_23_P212545	RAB5A	3.8802233	3.5298724	3.6715755	-0.350351	-0.208648	-0.279499	down
A_22_P00009250	lnc-LRP11-1	-3.264963	-3.346086	-3.742855	-0.081124	-0.477892	-0.279508	down
A_21_P0003719	lnc-SLC2A9-1	-1.061444	-1.284153	-1.398068	-0.222709	-0.336624	-0.279666	down
A_24_P39724	CCDC43	-0.138865	-0.496503	-0.340666	-0.357639	-0.201802	-0.27972	down
A_23_P252748	ZNF195	0.5772719	0.400269	0.1947842	-0.177003	-0.382488	-0.279745	down
A_22_P00003954	lnc-CHADL-2	-2.370926	-2.476708	-2.82479	-0.105782	-0.453864	-0.279823	down
A_33_P3219770	CDC73	-0.815012	-1.183486	-1.00645	-0.368474	-0.191438	-0.279956	down
A_22_P00004067	lnc-CHSY1-5	-2.870416	-2.842122	-3.458735	0.0282946	-0.588319	-0.280012	down
A_24_P161973	ATP11A	2.3917093	2.0054784	2.2177925	-0.386231	-0.173917	-0.280074	down
A_33_P3332302	MTVR2	-2.084104	-2.380254	-2.348279	-0.29615	-0.264175	-0.280163	down
A_23_P54006	HECTD1	4.0484333	3.7930484	3.7434635	-0.255385	-0.30497	-0.280177	down
A_32_P95823	UBXN4	0.7317753	0.4146094	0.4885511	-0.317166	-0.243224	-0.280195	down
A_23_P58321	CCNA2	2.9895658	2.4774842	2.9412193	-0.512082	-0.048347	-0.280214	down
A_33_P3277447	SLC26A2	1.7895079	1.555716	1.4627995	-0.233792	-0.326708	-0.28025	down
A_21_P0013046	XLOC_l2_012802	-3.020148	-3.261577	-3.339267	-0.24143	-0.31912	-0.280275	down
A_22_P00002363	lnc-C12orf48-1	-1.493023	-1.764539	-1.782137	-0.271515	-0.289114	-0.280314	down
A_23_P301476	C3orf33	-1.015971	-1.306842	-1.285734	-0.290871	-0.269763	-0.280317	down
A_33_P3215239	KIF20B	1.0743055	0.8752933	0.7124853	-0.199012	-0.36182	-0.280416	down

A_19_P00322977	LINC-PINT	-1.875807	-2.289451	-2.023033	-0.413644	-0.147226	-0.280435	down
A_23_P308954	BHLHB9	-1.611027	-1.741738	-2.041199	-0.130711	-0.430172	-0.280441	down
A_33_P3214491	PDE4DIP	-2.603638	-2.472032	-3.296269	0.1316063	-0.692631	-0.280512	down
A_23_P34877	RBM15	0.1821823	-0.127007	-0.069772	-0.309189	-0.251955	-0.280572	down
A_23_P23947	MAP3K8	-0.392174	-0.505093	-0.840622	-0.112918	-0.448448	-0.280683	down
A_21_P0001480	lnc-C1orf201-2	-1.891649	-2.331059	-2.01381	-0.43941	-0.122161	-0.280786	down
A_23_P27404	ZNF841	0.0679627	-0.1853	-0.240375	-0.253263	-0.308338	-0.2808	down
A_33_P3232955	F2RL3	0.2001615	-0.336777	0.1754584	-0.536939	-0.024703	-0.280821	down
A_33_P3279851	ANAPC1P1	-1.202604	-1.514631	-1.452256	-0.312027	-0.249652	-0.280839	down
A_21_P0010778	XLOC_l2_001134	-0.418949	-0.618912	-0.780923	-0.199963	-0.361974	-0.280969	down
A_33_P3258702	SEC22C	0.5909028	0.1386614	0.4811707	-0.452241	-0.109732	-0.280987	down
A_21_P0009329	lnc-COPZ2-1	-0.757074	-0.966435	-1.110448	-0.209362	-0.353374	-0.281368	down
A_23_P157495	PPP3CC	1.0098987	0.8849692	0.5720325	-0.124929	-0.437866	-0.281398	down
A_23_P146456	CTSV	2.6611109	2.474421	2.2849112	-0.18669	-0.3762	-0.281445	down
A_33_P3366246	UGGT1	2.0559912	1.724947	1.8238049	-0.331044	-0.232186	-0.281615	down
A_21_P0014738	LOC101930282	-1.658104	-1.752403	-2.127505	-0.094299	-0.4694	-0.28185	down
A_22_P00011346	lnc-OTUD7B-1	-1.257744	-1.36459	-1.714672	-0.106846	-0.456928	-0.281887	down
A_32_P129950	NHLRC3	1.5426588	1.1457939	1.3755045	-0.396865	-0.167154	-0.28201	down
A_33_P3228366	XPO5	1.9082756	1.2996583	1.9527378	-0.608617	0.0444622	-0.282078	down
A_23_P321473	FAM76A	-0.514922	-0.932345	-0.661742	-0.417423	-0.14682	-0.282121	down
A_33_P3323298	JUN	3.188034	2.9840636	2.8277178	-0.20397	-0.360316	-0.282143	down
A_33_P3363061	ERLIN2	-1.737457	-2.175504	-1.864214	-0.438047	-0.126757	-0.282402	down
A_21_P0011018	LOC101928620	-1.146513	-1.406222	-1.451651	-0.25971	-0.305139	-0.282424	down
A_21_P0013325	XLOC_l2_013873	-1.992127	-2.078925	-2.470234	-0.086799	-0.478108	-0.282453	down
A_23_P83453	SMARCC1	2.2221413	1.8348155	2.0444946	-0.387326	-0.177647	-0.282486	down
A_21_P0000829	RAB30-AS1	2.3540993	1.950275	2.1927567	-0.403824	-0.161343	-0.282583	down
A_23_P382045	TULP4	2.8945131	2.6482024	2.5756025	-0.246311	-0.318911	-0.282611	down
A_24_P145316	DTNBP1	-0.750671	-1.138931	-0.927843	-0.38826	-0.177172	-0.282716	down
A_23_P134809	NSMAF	0.8260779	0.5354061	0.5512266	-0.290672	-0.274851	-0.282762	down
A_33_P3271241	PPA1	7.29716	6.959843	7.068698	-0.337317	-0.228462	-0.28289	down
A_33_P3343996	LMBR1	-0.313865	-0.70758	-0.486053	-0.393714	-0.172187	-0.282951	down
A_33_P3247848	ZNF814	0.9090424	0.6358433	0.6162348	-0.273199	-0.292808	-0.283003	down
A_23_P103919	B4GALT3	4.1866875	3.6250873	4.1819544	-0.5616	-0.004733	-0.283167	down
A_33_P3642648	ZNF460	-0.030148	-0.111406	-0.515416	-0.081258	-0.485268	-0.283263	down
A_33_P3308105	GGH	5.7480946	5.5479407	5.3814974	-0.200154	-0.366597	-0.283376	down
A_23_P95029	SNTB1	3.1724176	2.770533	3.0075417	-0.401885	-0.164876	-0.28338	down
A_23_P99693	ZBTB1	-2.563182	-2.854368	-2.838774	-0.291187	-0.275593	-0.28339	down
A_33_P3242623	SLC7A11	4.000887	4.305221	3.1295433	0.3043342	-0.871344	-0.283505	down
A_21_P0012138	XLOC_l2_008783	-2.017116	-1.844434	-2.75704	0.1726818	-0.739924	-0.283621	down
A_23_P334727	NRG4	0.5708394	0.2056775	0.368742	-0.365162	-0.202097	-0.28363	down
A_22_P00009482	LINC00894	-2.512149	-2.423019	-3.168693	0.0891302	-0.656544	-0.283707	down
A_23_P73114	PROS1	0.6553059	0.2931581	0.449986	-0.362148	-0.20532	-0.283734	down
A_23_P148916	OSBPL9	2.9347363	2.7553077	2.5463886	-0.179429	-0.388348	-0.283888	down
A_23_P100420	ZCCHC14	3.206419	2.6930947	3.151948	-0.513324	-0.054471	-0.283898	down
A_23_P502832	RBM12	2.5563316	2.2482352	2.296566	-0.308096	-0.259766	-0.283931	down
A_23_P213959	PPARGC1B	0.5816245	0.2063537	0.3889728	-0.375271	-0.192652	-0.283961	down
A_33_P3415957	GTF2A2	6.3643446	5.788634	6.3720503	-0.575711	0.0077057	-0.284003	down
A_23_P343594	USP40	-0.250525	-0.397027	-0.672163	-0.146502	-0.421639	-0.28407	down
A_23_P11286	HNRNPH2	3.464242	3.0869355	3.273367	-0.377306	-0.190875	-0.284091	down
A_23_P24623	ELP4	2.1137676	1.7782874	1.8810196	-0.33548	-0.232748	-0.284114	down
A_23_P202004	PRTFDC1	1.5471463	1.4097462	1.116303	-0.1374	-0.430843	-0.284122	down
A_33_P3221489	KIRREL	-2.599147	-2.651155	-3.115392	-0.052008	-0.516245	-0.284127	down
A_23_P26358	SMG1	4.109644	3.9004655	3.7505522	-0.209178	-0.359092	-0.284135	down
A_24_P140391	PIGY	4.507015	4.083838	4.361764	-0.423177	-0.145251	-0.284214	down
A_22_P00022799	lnc-RIMS3-4	-0.499598	-1.138654	-0.429011	-0.639057	0.0705862	-0.284235	down
A_24_P359545	RBMXL1	1.6518288	1.2777791	1.4573708	-0.37405	-0.194458	-0.284254	down

A_23_P94762	ZNF354B	-0.247545	-0.649606	-0.414176	-0.402061	-0.166631	-0.284346	down
A_21_P0006910	lnc-ADD3-2	-2.14357	-2.3943	-2.461845	-0.25073	-0.318276	-0.284503	down
A_33_P3319026	ZZZ3	2.0226173	1.7920971	1.684093	-0.23052	-0.338524	-0.284522	down
A_23_P81898	UBD	0.5186873	0.1439776	0.324306	-0.37471	-0.194381	-0.284545	down
A_23_P60387	NOTCH1	-1.234816	-1.709248	-1.329536	-0.474432	-0.094719	-0.284576	down
A_33_P3247392	TPTE	0.8984613	0.7140622	0.5137019	-0.184399	-0.384759	-0.284579	down
A_23_P136964	RPGR	-0.126207	-0.595623	-0.225957	-0.469415	-0.09975	-0.284582	down
A_33_P3251821	TRAPPC13	0.4314776	0.2647076	0.0288925	-0.16677	-0.402585	-0.284678	down
A_23_P29723	SGOL1	-0.806748	-1.216648	-0.966303	-0.4099	-0.159555	-0.284728	down
A_23_P416608	LAMP2	1.2234492	0.8088303	1.068532	-0.414619	-0.154917	-0.284768	down
A_23_P303155	TMEM87B	-0.582682	-0.588962	-1.145961	-0.00628	-0.56328	-0.28478	down
A_21_P0007545	lnc-ARID2-3	-1.778199	-1.99685	-2.129552	-0.218651	-0.351354	-0.285002	down
A_33_P3333777	GABPB1-AS1	-0.269455	-0.645195	-0.463797	-0.37574	-0.194342	-0.285041	down
A_22_P00017727	KIZ	-1.107575	-1.29042	-1.494972	-0.182845	-0.387397	-0.285121	down
A_33_P3415663	MBLAC2	-0.138097	-0.664117	-0.182334	-0.52602	-0.044237	-0.285129	down
A_22_P00017651	lnc-WRAP73-2	-2.374015	-2.502818	-2.815616	-0.128803	-0.441601	-0.285202	down
A_24_P11506	KYNU	-2.543542	-2.597379	-3.060179	-0.053838	-0.516638	-0.285238	down
A_33_P3236632	MIA3	-0.319993	-0.725364	-0.485099	-0.405371	-0.165106	-0.285239	down
A_23_P215318	VPS41	1.0937419	0.6055174	1.0113168	-0.488225	-0.082425	-0.285325	down
A_21_P0008156	lnc-TPT1-1	0.3828402	0.4833798	-0.288758	0.1005397	-0.671598	-0.285529	down
A_24_P16950	ZNF160	-0.693485	-1.108139	-0.849929	-0.414653	-0.156444	-0.285549	down
A_23_P117852	KIAA0101	6.173585	5.940766	5.83529	-0.232819	-0.338295	-0.285557	down
A_33_P6817530	PCBP1-AS1	-1.691188	-1.548328	-2.405191	0.1428599	-0.714003	-0.285572	down
A_23_P434347	ITSN2	-1.214651	-1.405185	-1.595529	-0.190534	-0.380878	-0.285706	down
A_33_P3358641	LRRFIP1	1.7267523	1.541892	1.3399892	-0.18486	-0.386763	-0.285812	down
A_33_P3243332	ARHGAP42	0.0219827	-0.437971	-0.08981	-0.459953	-0.111793	-0.285873	down
A_33_P3321225	lnc-SLA2-3	-2.447205	-2.743696	-2.722489	-0.296492	-0.275284	-0.285888	down
A_23_P155257	FOXP1	4.712144	4.3333125	4.5191956	-0.378831	-0.192948	-0.28589	down
A_21_P0011914	XLOC_l2_008009	-0.100425	-0.453687	-0.318999	-0.353262	-0.218575	-0.285918	down
A_23_P139958	CDK8	0.1022029	-0.366098	-0.001483	-0.468301	-0.103686	-0.285994	down
A_33_P3510335	WNK1	-2.58596	-2.820638	-2.92328	-0.234678	-0.33732	-0.285999	down
A_23_P309837	STON2	-1.918689	-2.31425	-2.095192	-0.395561	-0.176503	-0.286032	down
A_33_P3393734	RPS27	-0.922461	-1.366168	-1.050952	-0.443707	-0.128491	-0.286099	down
A_23_P43079	INTS8	1.6653738	1.3857985	1.3726282	-0.279575	-0.292746	-0.28616	down
A_23_P25403	HCFC2	0.1512299	-0.189065	-0.080828	-0.340294	-0.232058	-0.286176	down
A_23_P364465	GPBP1	4.0532475	3.8226638	3.711318	-0.230584	-0.341929	-0.286257	down
A_23_P103864	TTC13	0.5652647	-0.041925	0.5993729	-0.60719	0.0341082	-0.286541	down
A_33_P3352253	MTUS1	-0.399534	-0.468976	-0.903327	-0.069442	-0.503792	-0.286617	down
A_23_P139388	RSF1	0.8364625	0.7773948	0.3221979	-0.059068	-0.514265	-0.286666	down
A_23_P343398	CCR7	-0.112409	-0.810954	0.012773	-0.698545	0.1251817	-0.286682	down
A_33_P3732466	LOC100506282	0.8744464	0.5731545	0.6022186	-0.301292	-0.272228	-0.28676	down
A_23_P37424	SPATA5L1	2.8053875	2.4091382	2.6280308	-0.396249	-0.177357	-0.286803	down
A_23_P144165	DZIP3	0.7911911	0.6541243	0.3545995	-0.137067	-0.436592	-0.286829	down
A_33_P3409077	TBC1D32	-2.238572	-2.368728	-2.682523	-0.130156	-0.443952	-0.287054	down
A_23_P59349	HECA	3.800293	3.8754601	3.150797	0.0751672	-0.649496	-0.287164	down
A_22_P00005635	LOC102724805	-2.65956	-3.025139	-2.868405	-0.365578	-0.208845	-0.287211	down
A_24_P142095	AKAP13	3.5710602	3.3958583	3.1718044	-0.175202	-0.399256	-0.287229	down
A_22_P00005915	HOTAIRM1	-0.854374	-1.328199	-0.955154	-0.473825	-0.10078	-0.287303	down
A_23_P129014	EFCAB11	1.6553669	1.3449059	1.3911915	-0.310461	-0.264175	-0.287318	down
A_23_P201845	ZMYM6	-0.819939	-1.036502	-1.17814	-0.216563	-0.358201	-0.287382	down
A_23_P111811	ING3	2.1583233	2.0475993	1.6942139	-0.110724	-0.464109	-0.287417	down
A_33_P3220470	SMAD6	1.710823	1.282845	1.5639672	-0.427978	-0.146856	-0.287417	down
A_23_P146997	TXLNG	2.6850548	2.3471627	2.4477272	-0.337892	-0.237328	-0.28761	down
A_32_P50431	ST13	-2.124837	-2.81316	-2.011776	-0.688323	0.1130614	-0.287631	down
A_24_P106112	PKD2	1.2686634	1.0766397	0.8854146	-0.192024	-0.383249	-0.287636	down
A_33_P3404879	LPHN2	-0.761754	-1.153664	-0.945138	-0.39191	-0.183384	-0.287647	down

A_21_P0014351	SP2-AS1	-2.415725	-2.967234	-2.439827	-0.551509	-0.024102	-0.287806	down
A_23_P54144	BMP4	4.7552776	4.356978	4.5778656	-0.3983	-0.177412	-0.287856	down
A_22_P00023374	INAFM2	2.625327	2.1624331	2.5124454	-0.462894	-0.112882	-0.287888	down
A_24_P61864	CCDC47	3.2546253	2.945414	2.987977	-0.309211	-0.266648	-0.28793	down
A_23_P325924	GAREML	0.0773659	-0.531545	0.1102805	-0.608911	0.0329146	-0.287998	down
A_32_P117464	MB21D2	1.0168996	1.1070814	0.350697	0.0901818	-0.666203	-0.28801	down
A_24_P228302	CEACAM7	3.0834846	2.6757588	2.9150705	-0.407726	-0.168414	-0.28807	down
A_23_P84140	IQCB1	2.0278835	1.8729243	1.6065316	-0.154959	-0.421352	-0.288156	down
A_23_P42096	MAP3K4	1.0326929	0.7901068	0.6988912	-0.242586	-0.333802	-0.288194	down
A_22_P00012741	lnc-RABEPK-1	-1.846567	-2.451928	-1.817625	-0.605362	0.0289416	-0.28821	down
A_33_P3230017	AURKAPS1	2.326045	1.8491292	2.2264318	-0.476916	-0.099613	-0.288265	down
A_33_P3780901	SBNO1	2.2286463	1.8092275	2.0714283	-0.419419	-0.157218	-0.288318	down
A_33_P3841621	MYO5B	-1.032763	-1.246414	-1.396118	-0.213651	-0.363355	-0.288503	down
A_21_P0012277	XLOC_l2_009448	-1.482539	-1.552363	-1.989735	-0.069824	-0.507196	-0.28851	down
A_23_P142994	RBM45	1.9329538	1.6451058	1.6435704	-0.287848	-0.289383	-0.288616	down
A_33_P3397051	EXOC8	-0.266737	-0.602012	-0.508767	-0.335274	-0.24203	-0.288652	down
A_23_P213620	PPP2R2B	-1.376257	-1.96051	-1.369383	-0.584253	0.0068741	-0.288689	down
A_23_P76705	TUBGCP3	0.4716268	0.0300736	0.3357625	-0.441553	-0.135864	-0.288709	down
A_23_P256632	PGGT1B	-0.844454	-1.201307	-1.065066	-0.356853	-0.220612	-0.288732	down
A_23_P76951	TMX1	5.3605433	5.020512	5.123047	-0.340031	-0.237496	-0.288764	down
A_22_P00022123	lnc-TK2-2	-1.893844	-2.205236	-2.15999	-0.311393	-0.266147	-0.28877	down
A_22_P00023585	LOC101929610	-0.973024	-1.133445	-1.390196	-0.16042	-0.417172	-0.288796	down
A_22_P00019571	SNAPC1	-0.078526	-0.274661	-0.460072	-0.196135	-0.381546	-0.288841	down
A_33_P3321533	TMEM178B	-2.148135	-2.226971	-2.647013	-0.078835	-0.498878	-0.288857	down
A_33_P3464555	CAMK1D	2.2086878	1.7050457	2.134554	-0.503642	-0.074134	-0.288888	down
A_24_P937325	FGD4	-2.501066	-2.947672	-2.632264	-0.446606	-0.131198	-0.288902	down
A_32_P20523	TIGD2	1.8519182	1.6379361	1.4879556	-0.213982	-0.363963	-0.288972	down
A_23_P165691	PSMD14	6.5828295	6.123175	6.464445	-0.459654	-0.118384	-0.289019	down
A_21_P0009037	lnc-IRX3-4	-2.588134	-2.618314	-3.13602	-0.03018	-0.547885	-0.289033	down
A_23_P205584	JKAMP	0.0426641	-0.227175	-0.265831	-0.269839	-0.308496	-0.289167	down
A_23_P13364	NUCB2	1.6299982	1.4510293	1.2300186	-0.178969	-0.39998	-0.289474	down
A_23_P144916	GFPT2	-1.454104	-1.37856	-2.108604	0.0755434	-0.6545	-0.289478	down
A_33_P3303951	NUPL2	1.1122723	0.8269615	0.818604	-0.285311	-0.293668	-0.28949	down
A_23_P8055	NRM	0.2356896	-0.129209	0.0215669	-0.364899	-0.214123	-0.289511	down
A_32_P199252	HSP90AA1	4.685052	4.1851516	4.605898	-0.4999	-0.079154	-0.289527	down
A_33_P3386581	ANKRD40	3.1482887	2.8790774	2.8384256	-0.269211	-0.309863	-0.289537	down
A_33_P3303940	BRCC3	3.1165638	2.7912965	2.8627138	-0.325267	-0.25385	-0.289559	down
A_24_P115511	RAB14	-0.408324	-1.128456	-0.267362	-0.720131	0.1409621	-0.289585	down
A_24_P264166	GOLGA8M	-2.536216	-2.616639	-3.03497	-0.080422	-0.498754	-0.289588	down
A_24_P157424	NCBP2	2.7538662	2.231566	2.6969376	-0.5223	-0.056929	-0.289614	down
A_23_P134058	GFOD1	-0.958652	-0.998888	-1.497716	-0.040236	-0.539065	-0.289651	down
A_33_P3374205	MKI67	2.8977346	2.4813018	2.7347937	-0.416433	-0.162941	-0.289687	down
A_23_P44295	CLASP2	1.9376421	1.6438456	1.6519756	-0.293797	-0.285666	-0.289732	down
A_33_P3355185	BCL2	-1.519263	-1.836714	-1.781578	-0.317451	-0.262315	-0.289883	down
A_24_P220058	MAPRE1	4.9110126	4.182265	5.059985	-0.728748	0.1489725	-0.289888	down
A_33_P3272165	NDUFAF6	3.1758833	2.8255725	2.9463778	-0.350311	-0.229506	-0.289908	down
A_33_P3327277	VWA2	0.5340066	0.2073197	0.2808714	-0.326687	-0.253135	-0.289911	down
A_23_P98930	KIAA1551	1.7599211	1.5152278	1.4247026	-0.244693	-0.335218	-0.289956	down
A_24_P419177	ZNF638	-1.329853	-1.500367	-1.739267	-0.170514	-0.409414	-0.289964	down
A_21_P0011461	XLOC_l2_005438	1.1276617	0.9952512	0.6800995	-0.132411	-0.447562	-0.289986	down
A_33_P3327479	ZDHHC3	3.504613	2.9082208	3.5204525	-0.596392	0.0158396	-0.290276	down
A_24_P74329	ZNF493	0.6683559	0.3656435	0.3904409	-0.302712	-0.277915	-0.290314	down
A_23_P250385	HIST1H1B	2.6537724	2.1819654	2.544839	-0.471807	-0.108933	-0.29037	down
A_24_P194931	SMEK2	2.1281977	2.0783248	1.5973191	-0.049873	-0.530879	-0.290376	down
A_33_P3304533	RNF207	-2.211329	-2.148857	-2.854788	0.0624716	-0.643459	-0.290494	down
A_33_P3345479	MIRLET7BHG	-0.919848	-1.034743	-1.385973	-0.114895	-0.466125	-0.29051	down

A_33_P3288904	ARHGEF7	-0.399548	-0.726942	-0.653237	-0.327394	-0.253689	-0.290542	down
A_21_P0011314	INAFM2	2.2661314	1.9239192	2.027092	-0.342212	-0.239039	-0.290626	down
A_33_P3418611	ZNF729	1.5751152	1.3505282	1.2183619	-0.224587	-0.356753	-0.29067	down
A_32_P54018	KIAA1143	1.9532862	1.6429319	1.6822071	-0.310354	-0.271079	-0.290717	down
A_33_P3261197	NCBP1	-1.484037	-1.784952	-1.764734	-0.300915	-0.280697	-0.290806	down
A_22_P00011035	lnc-NTHL1-1	-2.425068	-2.700527	-2.73137	-0.275459	-0.306301	-0.29088	down
A_23_P157109	ADCYAP1R1	-3.240402	-3.340248	-3.722351	-0.099847	-0.481949	-0.290898	down
A_24_P39639	EXOSC6	1.7782526	1.4147997	1.5598264	-0.363453	-0.218426	-0.29094	down
A_23_P133470	PJA2	0.9115071	0.6943216	0.5465961	-0.217186	-0.364911	-0.291048	down
A_23_P207666	USP32	0.1224289	-0.225599	-0.111695	-0.348028	-0.234124	-0.291076	down
A_33_P3371320	BRE	2.3402433	1.3789926	2.7192268	-0.961251	0.3789835	-0.291134	down
A_23_P376239	PAPOLA	2.3630867	1.9586625	2.1851091	-0.404424	-0.177978	-0.291201	down
A_23_P164022	MYO1C	7.944318	7.654989	7.651164	-0.289329	-0.293154	-0.291241	down
A_32_P711043	LOC100507191	-2.658165	-2.466	-3.43283	0.1921654	-0.774665	-0.29125	down
A_33_P3289356	CD58	3.260107	3.0212836	2.9164047	-0.238823	-0.343702	-0.291263	down
A_23_P99424	TPP2	2.5316944	2.2602391	2.2204876	-0.271455	-0.311207	-0.291331	down
A_23_P3204	MAPK6	3.8934813	3.8649	3.339347	-0.028581	-0.554134	-0.291358	down
A_24_P128563	KPNA6	1.8930464	1.0996366	2.1035805	-0.79341	0.2105341	-0.291438	down
A_32_P121303	NUP50	3.5932636	3.3871694	3.2162447	-0.206094	-0.377019	-0.291557	down
A_23_P312174	ALMS1	2.2619467	1.9617696	1.9789333	-0.300177	-0.283013	-0.291595	down
A_33_P3309636	C11orf58	4.010378	3.803742	3.6337814	-0.206636	-0.376596	-0.291616	down
A_33_P3216008	SKA3	2.6311808	2.250814	2.428218	-0.380367	-0.202963	-0.291665	down
A_23_P145529	PKIB	-0.364669	-0.867684	-0.445168	-0.503015	-0.080499	-0.291757	down
A_23_P130182	AURKB	0.5325017	0.2245112	0.2569237	-0.307991	-0.275578	-0.291784	down
A_33_P3221234	IPP	-1.306707	-1.741216	-1.455823	-0.434509	-0.149116	-0.291812	down
A_22_P00002461	lnc-C15orf41-5	-1.606844	-1.944481	-1.852866	-0.337637	-0.246022	-0.29183	down
A_22_P00020962	LOC100996579	0.2783041	-0.11715	0.089951	-0.395454	-0.188353	-0.291903	down
A_32_P9963	HSF2	2.4208202	1.8003364	2.4574413	-0.620484	0.0366211	-0.291931	down
A_21_P0012220	ANKRD20A11P	-0.731529	-1.052058	-0.994877	-0.320529	-0.263348	-0.291939	down
A_23_P102262	MRPL19	5.2291775	4.811113	5.063287	-0.418065	-0.165891	-0.291978	down
A_19_P00319398	ERVMER34-1	1.4982433	0.9903884	1.4221115	-0.507855	-0.076132	-0.291993	down
A_33_P3313258	ATAD3B	1.6909552	1.2765212	1.5213504	-0.414434	-0.169605	-0.292019	down
A_32_P93996	LOC100128361	-1.654339	-2.363588	-1.52914	-0.709249	0.1251988	-0.292025	down
A_19_P00322276	LINC01091	-2.84372	-3.070943	-3.200762	-0.227223	-0.357041	-0.292132	down
A_24_P301186	WDR89	-1.492082	-1.947411	-1.621091	-0.455328	-0.129009	-0.292169	down
A_21_P0000056	TMEM41B	0.6401811	0.4023609	0.2936077	-0.23782	-0.346573	-0.292197	down
A_23_P109055	TP53RK	-0.425727	-0.662451	-0.773446	-0.236724	-0.347719	-0.292222	down
A_23_P133365	TCERG1	0.8847528	0.5456028	0.639432	-0.33915	-0.245321	-0.292235	down
A_24_P68019	ZNF551	1.7553082	1.4805641	1.4455137	-0.274744	-0.309794	-0.292269	down
A_23_P419764	CCDC149	-1.846711	-2.159353	-2.118635	-0.312642	-0.271924	-0.292283	down
A_23_P4036	TEX2	2.1926146	1.6478677	2.1523695	-0.544747	-0.040245	-0.292496	down
A_24_P259922	LANCL1	1.5560799	1.1512456	1.3758616	-0.404834	-0.180218	-0.292526	down
A_21_P0004569	LUCAT1	-2.193657	-1.671165	-3.30137	0.5224924	-1.107713	-0.29261	down
A_23_P252642	BBS5	6.293401	6.019197	5.982339	-0.274204	-0.311062	-0.292633	down
A_23_P7761	C5orf22	-0.51989	-0.908461	-0.716628	-0.388571	-0.196738	-0.292655	down
A_33_P3268124	ZNF654	-1.188631	-1.40594	-1.556639	-0.217309	-0.368008	-0.292659	down
A_23_P216568	FAM206A	2.4151897	2.112184	2.1327648	-0.303006	-0.282425	-0.292715	down
A_33_P3539345	MYO6	2.0164003	1.642839	1.8045263	-0.373561	-0.211874	-0.292718	down
A_23_P47704	UCP2	2.2448282	1.6100712	2.2940407	-0.634757	0.0492125	-0.292772	down
A_23_P63751	PRDX3	3.4162455	3.150457	3.0963144	-0.265789	-0.319931	-0.29286	down
A_33_P3244669	TMEM30A	4.083559	3.8228192	3.7585258	-0.26074	-0.325033	-0.292887	down
A_24_P383850	EIF4G3	-0.712047	-1.117875	-0.892108	-0.405828	-0.180061	-0.292945	down
A_33_P3306545	EIF4G2	3.2758045	2.986671	2.9790344	-0.289134	-0.29677	-0.292952	down
A_33_P3284247	GYG2P1	-1.527479	-1.929829	-1.711451	-0.40235	-0.183972	-0.293161	down
A_23_P3212	DTWD1	1.5311341	1.2030358	1.2728705	-0.328098	-0.258264	-0.293181	down
A_33_P3396159	CCDC6	2.404437	2.0070415	2.2152176	-0.397396	-0.189219	-0.293308	down

A_23_P35309	TAF5L	1.113615	0.6009493	1.039465	-0.512666	-0.07415	-0.293408	down
A_23_P82108	ZBTB2	1.5113935	1.0355949	1.3994217	-0.475799	-0.111972	-0.293885	down
A_33_P3223648	CEBPZOS	0.3969078	-0.045613	0.2515421	-0.442521	-0.145366	-0.293943	down
A_23_P118203	ZG16B	-0.426123	-1.006084	-0.434058	-0.579961	-0.007935	-0.293948	down
A_23_P368259	EID2B	-0.957427	-1.110367	-1.392612	-0.15294	-0.435185	-0.294063	down
A_23_P406330	SMAP2	1.0349545	0.4660277	1.0152483	-0.568927	-0.019706	-0.294317	down
A_33_P3312842	ZNRF2	0.3259406	0.112371	-0.049155	-0.21357	-0.375096	-0.294333	down
A_23_P79628	PSME4	1.4634471	1.2335758	1.1043711	-0.229871	-0.359076	-0.294474	down
A_24_P140827	PCMT1	4.421097	3.9955297	4.2574797	-0.425567	-0.163617	-0.294592	down
A_23_P80648	MFN1	1.9120245	1.6340408	1.6007671	-0.277984	-0.311257	-0.294621	down
A_24_P309360	TM9SF3	3.8594427	3.6333127	3.4961157	-0.22613	-0.363327	-0.294729	down
A_23_P89601	KRT32	-2.746498	-3.090131	-2.992397	-0.343633	-0.245899	-0.294766	down
A_32_P220696	TERF1	3.13128	2.9033532	2.7696505	-0.227927	-0.36163	-0.294778	down
A_23_P424878	ARHGAP39	0.2161675	-0.036801	-0.120508	-0.252969	-0.336676	-0.294822	down
A_23_P9603	PRKDC	1.2845054	1.0988607	0.8803153	-0.185645	-0.40419	-0.294917	down
A_21_P0008871	FLJ42627	0.4260531	0.1159096	0.1460176	-0.310143	-0.280036	-0.295089	down
A_23_P336565	FAM185A	-0.185622	-0.568533	-0.392908	-0.382911	-0.207286	-0.295099	down
A_33_P3247175	C4orf47	-0.067186	-0.22205	-0.502573	-0.154864	-0.435387	-0.295125	down
A_33_P3252635	ZNF254	0.3517699	0.1690526	-0.055831	-0.182717	-0.407601	-0.295159	down
A_33_P3422258	C8orf59	6.235648	5.861545	6.0193224	-0.374103	-0.216326	-0.295214	down
A_23_P356122	ZNF451	-2.125147	-2.31604	-2.524712	-0.190893	-0.399565	-0.295229	down
A_21_P0012274	LINC01296	0.28862	-0.253286	0.2400322	-0.541906	-0.048588	-0.295247	down
A_33_P3267410	KLLN	-0.474034	-0.964619	-0.574251	-0.490585	-0.100217	-0.295401	down
A_33_P3309526	PTPRC	-1.695676	-1.772095	-2.210229	-0.076419	-0.514553	-0.295486	down
A_22_P00022216	lnc-PAPSS2-2	-1.773429	-1.842659	-2.295189	-0.06923	-0.52176	-0.295495	down
A_21_P0012011	ANKRD36BP2	-2.780109	-2.835993	-3.315511	-0.055884	-0.535402	-0.295643	down
A_23_P214037	NPM1	7.334243	7.299253	6.7778788	-0.03499	-0.556364	-0.295677	down
A_33_P3237235	FBXO44	-1.282569	-1.68609	-1.470485	-0.403521	-0.187916	-0.295718	down
A_23_P86550	EIF3A	6.869195	6.719477	6.4274235	-0.149718	-0.441772	-0.295745	down
A_23_P96325	ERCC6L	0.766427	0.3667207	0.5745101	-0.399706	-0.191917	-0.295812	down
A_23_P167828	RWDD1	4.7298098	4.4257684	4.442094	-0.304041	-0.287716	-0.295879	down
A_33_P3330468	RBM41	1.3767886	1.2123356	0.9494739	-0.164453	-0.427315	-0.295884	down
A_24_P311604	PACRGL	-0.733501	-1.119929	-0.938904	-0.386428	-0.205402	-0.295915	down
A_33_P3290909	SMC1A	2.9716492	2.445066	2.9062252	-0.526583	-0.065424	-0.296004	down
A_33_P3502311	LOC102723564	-0.944852	-1.334645	-1.147173	-0.389793	-0.202322	-0.296057	down
A_23_P254079	STBD1	-0.66753	-1.06088	-0.866307	-0.393351	-0.198778	-0.296064	down
A_23_P23924	CAPN2	6.673332	6.2883735	6.4660244	-0.384959	-0.207308	-0.296133	down
A_23_P251316	CTNND1	2.305996	1.6873817	2.3322735	-0.618614	0.0262775	-0.296168	down
A_23_P329198	NABP1	3.1401405	3.0753608	2.6125584	-0.06478	-0.527582	-0.296181	down
A_32_P107002	SNX29	-1.700936	-1.826995	-2.16739	-0.126059	-0.466454	-0.296256	down
A_23_P34510	PHC2	1.4865346	1.1317616	1.2486925	-0.354773	-0.237842	-0.296308	down
A_33_P3357322	SMC2	2.0418415	1.7213874	1.7693586	-0.320454	-0.272483	-0.296468	down
A_24_P82135	SECISBP2L	-0.504015	-0.822083	-0.7789	-0.318068	-0.274886	-0.296477	down
A_21_P0008857	lnc-PEAK1.1-1	-0.119002	-0.463931	-0.367173	-0.344928	-0.248171	-0.29655	down
A_33_P3400171	AKT2	-0.486033	-0.509344	-1.055843	-0.023311	-0.569811	-0.296561	down
A_33_P3267305	ANKRD52	5.2344656	4.8509803	5.024727	-0.383485	-0.209739	-0.296612	down
A_23_P19543	SRPK1	4.840762	4.4242597	4.6640263	-0.416502	-0.176736	-0.296619	down
A_33_P3722568	LINC00685	-1.127581	-1.510716	-1.337728	-0.383135	-0.210147	-0.296641	down
A_22_P00021349	LOC100288748	0.236598	-0.161777	0.0416265	-0.398375	-0.194972	-0.296673	down
A_22_P00019560	lnc-SFN-1	-3.056322	-3.160647	-3.545354	-0.104325	-0.489032	-0.296679	down
A_23_P44760	SETD2	-0.159857	-0.302407	-0.610878	-0.14255	-0.451022	-0.296786	down
A_23_P154025	SSB	5.2869434	5.061856	4.9179573	-0.225088	-0.368986	-0.297037	down
A_23_P34946	DIEXF	-0.258745	-0.392888	-0.718959	-0.134143	-0.460215	-0.297179	down
A_23_P57199	GGTLC1	-0.33235	-0.629039	-0.630114	-0.29669	-0.297764	-0.297227	down
A_24_P66932	LOC729080	4.7731686	4.539002	4.412838	-0.234167	-0.360331	-0.297249	down
A_33_P3255274	CNNM3	2.2717657	1.5683885	2.3805923	-0.703377	0.1088266	-0.297275	down

A_24_P943472	NR1D2	0.5895476	0.589047	-0.004507	-0.000501	-0.594055	-0.297278	down
A_23_P96331	WAS	-1.779446	-2.061955	-2.091495	-0.282509	-0.312049	-0.297279	down
A_33_P3398181	LOC100127909	1.4085598	1.2914858	0.9310627	-0.117074	-0.477497	-0.297286	down
A_23_P82047	STXBP5	-0.057641	-0.330387	-0.379498	-0.272746	-0.321857	-0.297302	down
A_23_P212696	FSTL1	1.5254364	1.3185792	1.1376634	-0.206857	-0.387773	-0.297315	down
A_23_P93464	BCKDHB	1.6845875	1.2995024	1.4748063	-0.385085	-0.209781	-0.297433	down
A_23_P7282	ELMOD2	3.2824087	3.1056857	2.8642092	-0.176723	-0.4182	-0.297461	down
A_23_P35148	TAF13	1.5144138	1.3251276	1.1087193	-0.189286	-0.405694	-0.29749	down
A_33_P3215193	NMNAT1	-0.617026	-0.708512	-1.12058	-0.091486	-0.503554	-0.29752	down
A_23_P40240	CTSZ	3.9070559	2.9848762	4.234149	-0.92218	0.3270931	-0.297543	down
A_21_P0004622	lnc-AL035696.1-5	-1.890273	-2.319229	-2.056437	-0.428957	-0.166164	-0.29756	down
A_22_P00005391	DSC2	0.8304634	0.7133994	0.3521919	-0.117064	-0.478271	-0.297668	down
A_24_P382401	ZNF791	-1.462342	-1.760994	-1.759053	-0.298652	-0.296711	-0.297682	down
A_24_P117323	KLHL22	0.0706105	-0.300612	-0.153818	-0.371223	-0.224428	-0.297826	down
A_23_P420348	POTED	1.2204456	1.0731273	0.7718439	-0.147318	-0.448602	-0.29796	down
A_33_P3392952	FOXK2	3.4643564	3.1912932	3.1413813	-0.273063	-0.322975	-0.298019	down
A_23_P342727	STARD13	-1.402784	-1.672605	-1.729207	-0.269821	-0.326423	-0.298122	down
A_21_P0014396	IPO5P1	1.7519484	1.5089245	1.3986306	-0.243024	-0.353318	-0.298171	down
A_24_P126651	TCEA1	0.3202219	0.2661705	-0.222089	-0.054051	-0.542311	-0.298181	down
A_23_P37994	TP53TG3	-1.276943	-1.624686	-1.525763	-0.347743	-0.248819	-0.298281	down
A_33_P3278410	MLLT4	0.9321976	0.7485352	0.5192061	-0.183662	-0.412992	-0.298327	down
A_33_P3210585	AAK1	3.9250755	3.6898322	3.563631	-0.235243	-0.361444	-0.298344	down
A_23_P65068	EID3	-1.757188	-1.239227	-2.871924	0.5179606	-1.114736	-0.298388	down
A_23_P207507	ABCC3	4.309863	4.09808	3.924736	-0.211783	-0.385127	-0.298455	down
A_33_P3360887	HELZ2	4.303172	3.9592957	4.0500536	-0.343876	-0.253119	-0.298497	down
A_23_P24176	CCNJ	1.4095383	0.9523659	1.2694659	-0.457172	-0.140072	-0.298622	down
A_23_P1343	STAM	0.5063586	0.2078776	0.2075462	-0.298481	-0.298812	-0.298647	down
A_23_P217659	BRCC3	4.3352423	4.0139956	4.0586033	-0.321247	-0.276639	-0.298943	down
A_33_P3399101	CTSL	0.8895917	0.6353045	0.5459318	-0.254287	-0.34366	-0.298974	down
A_24_P317907	SORBS1	0.5239291	0.2256012	0.224175	-0.298328	-0.299754	-0.299041	down
A_33_P3290179	DEGS1	2.0932617	1.480515	2.1076126	-0.612747	0.0143509	-0.299198	down
A_33_P3406661	TMEM63C	0.3063998	0.0215902	-0.007216	-0.28481	-0.313616	-0.299213	down
A_23_P90220	ZNF585A	-0.785179	-0.985059	-1.183806	-0.199881	-0.398628	-0.299254	down
A_33_P3263061	AK3	5.3516235	5.1203094	4.984313	-0.231314	-0.367311	-0.299312	down
A_24_P136402	KMT2A	-2.173424	-2.531525	-2.414085	-0.358101	-0.240662	-0.299381	down
A_32_P80255	DDX6	2.9053059	2.3345342	2.8771	-0.570772	-0.028206	-0.299489	down
A_19_P00324399	LINC01481	1.5644455	0.9833403	1.5464358	-0.581105	-0.01801	-0.299557	down
A_33_P6559880	ZNF518A	-1.293299	-1.244531	-1.941547	0.048768	-0.648248	-0.29974	down
A_21_P0011075	XLOC_l2_003260	-1.756167	-1.778197	-2.333683	-0.02203	-0.577515	-0.299772	down
A_33_P3877728	SMCR6	1.2024393	0.9548326	0.8504448	-0.247607	-0.351995	-0.299801	down
A_22_P00002737	NRSN2-AS1	-1.502403	-1.634863	-1.969554	-0.132461	-0.467152	-0.299806	down
A_33_P3250671	TCF7	1.0836983	0.6828442	0.8847489	-0.400854	-0.198949	-0.299902	down
A_24_P349965	TCF19	-0.61594	-1.114524	-0.717339	-0.498584	-0.101398	-0.299991	down
A_23_P424582	EGFL8	-0.696578	-1.024025	-0.969207	-0.327447	-0.272629	-0.300038	down
A_21_P0013441	RABGEF1	1.2627964	0.9458604	0.9793515	-0.316936	-0.283445	-0.30019	down
A_24_P134392	HSPA13	1.1639771	1.036025	0.6915264	-0.127952	-0.472451	-0.300201	down
A_23_P316064	ZACN	-2.797612	-3.076184	-3.119516	-0.278572	-0.321904	-0.300238	down
A_33_P3396692	SSR1	3.4070253	3.046307	3.1671448	-0.360718	-0.239881	-0.300299	down
A_33_P3415500	TRIO	2.998932	2.759603	2.6374483	-0.239329	-0.361484	-0.300406	down
A_33_P3405500	SACS	-0.902516	-1.275899	-1.129988	-0.373383	-0.227471	-0.300427	down
A_24_P403417	PTGES	-1.123097	-1.561177	-1.286058	-0.438081	-0.162962	-0.300521	down
A_23_P70843	BPGM	1.5895863	1.2203856	1.3577261	-0.369201	-0.23186	-0.30053	down
A_24_P268015	MEAF6	1.5760183	1.046535	1.5040517	-0.529483	-0.071967	-0.300725	down
A_23_P2397	RASSF3	-0.98727	-1.376865	-1.199158	-0.389595	-0.211887	-0.300741	down
A_23_P99741	CDKL1	0.2638655	-0.284838	0.2110548	-0.548703	-0.052811	-0.300757	down
A_21_P0012158	lnc-C20orf197-4	-2.588236	-2.506147	-3.271858	0.0820892	-0.683622	-0.300766	down

A_21_P0000095	HDHD1	-0.447004	-1.124631	-0.371029	-0.677626	0.0759754	-0.300825	down
A_23_P88691	CHRNA5	2.8371391	2.4040322	2.6685886	-0.433107	-0.16855	-0.300829	down
A_23_P117515	ARID4A	-2.832193	-3.130981	-3.135277	-0.298788	-0.303084	-0.300936	down
A_32_P197524	EIF2A	0.6529284	0.3234739	0.380404	-0.329454	-0.272524	-0.300989	down
A_21_P0001340	lnc-ABCD3-2	-3.018605	-3.284033	-3.355176	-0.265429	-0.336572	-0.301	down
A_23_P36658	MGST1	6.902195	6.583959	6.6182594	-0.318236	-0.283936	-0.301086	down
A_33_P3393836	NT5C3A	2.666339	2.3109374	2.4194145	-0.355402	-0.246924	-0.301163	down
A_23_P95764	PRPS1	5.11285	4.868691	4.754629	-0.244159	-0.358221	-0.30119	down
A_23_P389118	ANO6	2.9203758	2.4477544	2.7905836	-0.472621	-0.129792	-0.301207	down
A_32_P34552	POLB	0.4498701	0.1774097	0.1197181	-0.27246	-0.330152	-0.301306	down
A_24_P287756	NUDT21	2.3537426	1.9772363	2.1275558	-0.376506	-0.226187	-0.301347	down
A_23_P2601	HSP90B1	7.720269	7.3660264	7.4717817	-0.354243	-0.248487	-0.301365	down
A_32_P116206	RELL1	2.1841393	1.9830337	1.7824855	-0.201106	-0.401654	-0.30138	down
A_23_P71328	MATN2	-0.708456	-0.907201	-1.112483	-0.198745	-0.404027	-0.301386	down
A_24_P940135	CTBS	0.8172078	0.5373049	0.4941912	-0.279903	-0.323017	-0.30146	down
A_33_P3212716	LOC101929998	-0.793453	-1.069249	-1.120584	-0.275796	-0.32713	-0.301463	down
A_22_P00016779	lnc-TRIM41-3	0.3459382	-0.329486	0.4177012	-0.675424	0.071763	-0.301831	down
A_24_P402438	TGFB2	1.3320246	1.2001066	0.8601542	-0.131918	-0.47187	-0.301894	down
A_22_P00018367	EPHA1-AS1	-0.459064	-0.851962	-0.670276	-0.392898	-0.211212	-0.302055	down
A_33_P3313421	UHRF1BP1	2.409896	2.5765023	1.639041	0.1666064	-0.770855	-0.302124	down
A_23_P64792	KCNMB4	3.3621464	3.0563712	3.0635529	-0.305775	-0.298594	-0.302184	down
A_33_P3415207	MPV17	-1.974711	-2.36222	-2.191779	-0.387509	-0.217068	-0.302289	down
A_23_P321261	ME2	1.2288547	0.8316503	1.0214748	-0.397204	-0.20738	-0.302292	down
A_24_P298877	C1orf174	3.9832067	3.9717498	3.389821	-0.011457	-0.593386	-0.302421	down
A_33_P3303748	PDPR	-3.022663	-3.195716	-3.454684	-0.173053	-0.43202	-0.302536	down
A_33_P3309064	UQCC1	-1.328929	-2.076662	-1.186404	-0.747733	0.1425252	-0.302604	down
A_24_P304449	MLEC	1.5688238	0.9089513	1.6234698	-0.659873	0.054646	-0.302613	down
A_24_P173746	RALGPS2	-0.505147	-0.626912	-0.988813	-0.121764	-0.483665	-0.302715	down
A_23_P59547	NT5C3A	3.585432	3.212335	3.3529253	-0.373097	-0.232507	-0.302802	down
A_23_P256473	SEMA3C	-2.192448	-2.510298	-2.480217	-0.31785	-0.287769	-0.302809	down
A_22_P00000421	LINC00969	-0.483055	-0.520286	-1.05157	-0.037231	-0.568516	-0.302873	down
A_23_P86660	HNRNPA3	2.9990587	2.661489	2.730689	-0.33757	-0.26837	-0.30297	down
A_23_P73429	HCLS1	-1.824165	-1.720183	-2.534124	0.103982	-0.709959	-0.302988	down
A_23_P50919	SERPINE2	-0.817363	-1.323592	-0.917357	-0.506228	-0.099994	-0.303111	down
A_33_P3272563	NMT2	0.5289283	0.2147245	0.236845	-0.314204	-0.292083	-0.303144	down
A_33_P3346292	UBAP2	-2.012181	-2.179535	-2.451192	-0.167354	-0.439011	-0.303183	down
A_23_P120557	TASP1	1.4044266	1.1361356	1.066124	-0.268291	-0.338303	-0.303297	down
A_21_P0005653	LOC101929128	-1.784337	-2.040599	-2.134909	-0.256262	-0.350572	-0.303417	down
A_22_P00013456	USP2-AS1	-1.015756	-1.396092	-1.242397	-0.380336	-0.226641	-0.303489	down
A_32_P209208	CAPS2	-0.917411	-0.837085	-1.604801	0.0803261	-0.68739	-0.303532	down
A_33_P3416286	RBM12	2.0953083	1.8411069	1.7421975	-0.254201	-0.353111	-0.303656	down
A_23_P205098	PDS5B	0.3923197	0.3148837	-0.137652	-0.077436	-0.529972	-0.303704	down
A_32_P46191	ZNF727	4.1514187	3.969338	3.7258549	-0.182081	-0.425564	-0.303822	down
A_21_P0012245	CECR7	0.2364574	0.0037498	-0.138613	-0.232708	-0.37507	-0.303889	down
A_23_P426140	ZYG11B	-1.550028	-1.79847	-1.90944	-0.248442	-0.359412	-0.303927	down
A_23_P47839	DDX55	2.1277113	1.7404809	1.9070826	-0.38723	-0.220629	-0.30393	down
A_23_P345065	SCLY	3.0437574	2.45618	3.0234175	-0.587577	-0.02034	-0.303959	down
A_24_P333716	XPO4	1.0422215	0.746881	0.7292953	-0.295341	-0.312926	-0.304133	down
A_24_P208909	TRIM2	2.0703993	1.825077	1.707386	-0.245322	-0.363013	-0.304168	down
A_33_P3229156	SLC17A9	3.671895	3.087439	3.6478348	-0.584456	-0.02406	-0.304258	down
A_23_P352426	CDRT1	-1.762568	-1.340923	-2.792835	0.4216447	-1.030267	-0.304311	down
A_21_P0002805	LOC102724479	-1.121808	-1.514089	-1.338321	-0.392281	-0.216513	-0.304397	down
A_23_P57474	OSBP2	-1.725956	-1.929903	-2.130873	-0.203946	-0.404917	-0.304431	down
A_21_P0005971	lnc-RBM12B-2	-2.217003	-2.383348	-2.659524	-0.166345	-0.44252	-0.304433	down
A_32_P142440	PCSK9	1.862174	1.0893493	2.0261097	-0.772825	0.1639357	-0.304445	down
A_23_P217737	ATP7A	-0.980572	-1.033514	-1.536643	-0.052942	-0.556071	-0.304506	down

A_21_P0007508	lnc-C12orf49-1	-2.823848	-2.901793	-3.355015	-0.077945	-0.531167	-0.304556	down
A_23_P429689	BOD1L1	-0.447178	-0.683773	-0.81977	-0.236595	-0.372592	-0.304594	down
A_32_P85813	RAP1GDS1	2.7657537	2.3148952	2.607417	-0.450859	-0.158337	-0.304598	down
A_24_P73669	GSPT1	2.2020206	1.8238115	1.9710102	-0.378209	-0.23101	-0.30461	down
A_23_P30162	MFSD8	0.3985863	0.2177248	-0.029817	-0.180861	-0.428403	-0.304632	down
A_22_P00020246	lnc-GPR55-2	0.6572509	0.58989	0.1152549	-0.067361	-0.541996	-0.304678	down
A_23_P18939	RASA1	0.817687	0.5479512	0.4780536	-0.269736	-0.339633	-0.304685	down
A_33_P3327847	SGPL1	5.6825905	5.319966	5.4353228	-0.362625	-0.247268	-0.304946	down
A_21_P0013327	LOC493754	0.5784655	0.2211995	0.3258205	-0.357266	-0.252645	-0.304955	down
A_33_P3381647	AKT2	0.4972725	0.0674186	0.3171239	-0.429854	-0.180149	-0.305001	down
A_21_P0000149	GCFC2	1.0772247	0.6425638	0.9014354	-0.434661	-0.175789	-0.305225	down
A_21_P0000207	RPS3A	0.9989324	0.5307322	0.8566637	-0.4682	-0.142269	-0.305234	down
A_24_P393571	GDA	3.69872	3.5119224	3.2747393	-0.186798	-0.423981	-0.305389	down
A_23_P82478	PUS7	3.1374369	2.9014401	2.7623978	-0.235997	-0.375039	-0.305518	down
A_33_P3409154	SLC22A23	-1.077331	-1.320359	-1.445426	-0.243028	-0.368095	-0.305562	down
A_33_P3335966	TPM1	2.1864815	1.8057733	1.9559269	-0.380708	-0.230555	-0.305631	down
A_33_P3415962	LOC100506947	-2.611081	-2.886483	-2.947447	-0.275401	-0.336366	-0.305884	down
A_33_P3325753	TRIOBP	-0.800293	-1.000763	-1.211928	-0.20047	-0.411635	-0.306053	down
A_23_P140029	UBL3	1.5941315	1.44384	1.1321006	-0.150291	-0.462031	-0.306161	down
A_24_P56388	HIF1A	2.976325	2.5349383	2.8053703	-0.441387	-0.170955	-0.306171	down
A_24_P923757	ATF7IP	1.3476229	0.9950318	1.0878196	-0.352591	-0.259803	-0.306197	down
A_23_P52885	MSANTD2	-1.847164	-2.098058	-2.208712	-0.250894	-0.361548	-0.306221	down
A_23_P217570	CAPN6	1.6809597	0.8267074	1.9226952	-0.854252	0.2417355	-0.306258	down
A_23_P336513	GEMIN5	2.956603	2.4591966	2.8413858	-0.497406	-0.115217	-0.306312	down
A_23_P82748	ENY2	6.275915	5.94863	5.989978	-0.327285	-0.285937	-0.306611	down
A_21_P0007030	lnc-UROS-1	-0.794437	-1.091759	-1.110631	-0.297322	-0.316194	-0.306758	down
A_23_P32577	DACH1	-2.110902	-2.520105	-2.315515	-0.409203	-0.204613	-0.306908	down
A_23_P101374	CYP2S1	-0.763333	-1.181711	-0.958824	-0.418378	-0.195491	-0.306935	down
A_24_P227831	ABCC1	1.4983177	0.7478247	1.6349411	-0.750493	0.1366234	-0.306935	down
A_24_P331655	KIAA0895L	2.2977228	1.912456	2.0690584	-0.385267	-0.228664	-0.306966	down
A_33_P3220390	WTAP	-1.25918	-1.683688	-1.448868	-0.424508	-0.189688	-0.307098	down
A_33_P3344816	ZNF813	-2.242417	-2.656039	-2.443116	-0.413621	-0.200698	-0.30716	down
A_23_P31671	UQCRB	7.14884	6.8278785	6.8548594	-0.320961	-0.293981	-0.307471	down
A_33_P3375234	RMDN3	0.4539228	0.1494646	0.1432872	-0.304458	-0.310636	-0.307547	down
A_24_P370970	NSL1	-0.515832	-0.682206	-0.964569	-0.166374	-0.448737	-0.307555	down
A_23_P164141	PSME3	4.1614876	3.5886216	4.1192265	-0.572866	-0.042261	-0.307564	down
A_23_P38677	SLMO1	2.9157743	2.5156493	2.7007494	-0.400125	-0.215025	-0.307575	down
A_23_P51853	ZDHHC18	-0.025132	-0.646515	-0.019	-0.621383	0.0061321	-0.307626	down
A_33_P3331711	LOC100130463	-1.673554	-2.022497	-1.93992	-0.348943	-0.266365	-0.307654	down
A_23_P112113	KIAA0196	4.400098	4.1158996	4.0689707	-0.284198	-0.331127	-0.307663	down
A_33_P3418833	FLRT3	1.2676253	0.9314613	0.9884243	-0.336164	-0.279201	-0.307683	down
A_21_P0000271	SNORA4	0.7103891	0.3062515	0.4990554	-0.404138	-0.211334	-0.307736	down
A_23_P41424	SLC39A8	4.513179	4.159064	4.251814	-0.354115	-0.261365	-0.30774	down
A_24_P88921	XPC	0.1677966	-0.106183	-0.173911	-0.273979	-0.341707	-0.307843	down
A_23_P104705	SLC29A2	0.3593907	-0.177759	0.2807841	-0.53715	-0.078607	-0.307878	down
A_33_P3374085	LRRFIP1	2.502408	2.3524327	2.0365305	-0.149975	-0.465878	-0.307926	down
A_33_P3365010	ELK4	1.065105	0.5572004	0.9570155	-0.507905	-0.108089	-0.307997	down
A_33_P3307955	ABCD3	2.6932697	1.9252534	2.8450432	-0.768016	0.1517735	-0.308121	down
A_33_P3384322	METTL22	-0.04384	-0.433828	-0.270158	-0.389988	-0.226318	-0.308153	down
A_33_P3281139	ZC3H13	-1.424137	-1.988337	-1.476291	-0.5642	-0.052154	-0.308177	down
A_23_P118042	LRRC36	-0.7058	-1.109033	-0.918933	-0.403234	-0.213134	-0.308184	down
A_23_P204967	MPHOSPH8	2.3807087	2.0670857	2.0779467	-0.313623	-0.302762	-0.308192	down
A_33_P3222565	TSPAN14	-1.437712	-1.665804	-1.826086	-0.228092	-0.388374	-0.308233	down
A_22_P00023783	lnc-MKI67-2	-2.460609	-2.816477	-2.721225	-0.355867	-0.260616	-0.308242	down
A_22_P00012004	PLCG2	-2.585414	-2.586575	-3.200837	-0.001161	-0.615423	-0.308292	down
A_32_P73045	CMAS	2.0105848	1.7713223	1.6331916	-0.239263	-0.377393	-0.308328	down

A_24_P288685	IL13RA1	0.5183888	0.1132941	0.3068104	-0.405095	-0.211578	-0.308336	down
A_24_P926195	MAN1A2	2.0504236	1.5356855	1.9482775	-0.514738	-0.102146	-0.308442	down
A_32_P92281	PMS2P9	-1.278607	-1.407653	-1.766517	-0.129046	-0.487909	-0.308478	down
A_23_P93562	SESN1	-0.813398	-1.015695	-1.228149	-0.202296	-0.41475	-0.308523	down
A_23_P117933	GCSH	5.983096	5.685739	5.663377	-0.297357	-0.319719	-0.308538	down
A_23_P121898	PARP8	1.1289997	0.8567481	0.7838745	-0.272252	-0.345125	-0.308688	down
A_21_P0007916	DNAJC3-AS1	0.0167165	-0.309345	-0.274671	-0.326062	-0.291388	-0.308725	down
A_33_P3257451	SRRM3	-2.112036	-2.245153	-2.596563	-0.133117	-0.484528	-0.308822	down
A_24_P21985	FOXJ2	-1.490507	-1.789213	-1.80958	-0.298706	-0.319073	-0.30889	down
A_33_P3396607	UGDH	1.6665707	1.4368868	1.2783837	-0.229684	-0.388187	-0.308935	down
A_22_P00007547	LOC101929147	-1.64792	-1.866719	-2.047251	-0.218799	-0.399331	-0.309065	down
A_24_P91852	DYNLT3	0.0564733	-0.173887	-0.331423	-0.23036	-0.387897	-0.309128	down
A_23_P304511	ZNF397	-2.684705	-3.037647	-2.950054	-0.352942	-0.265349	-0.309145	down
A_32_P96807	RC3H1	2.9672775	2.691729	2.6245184	-0.275548	-0.342759	-0.309154	down
A_23_P393727	NRP2	-0.696453	-0.832929	-1.178306	-0.136476	-0.481853	-0.309165	down
A_23_P29985	TRMT44	1.807787	1.584929	1.4120932	-0.222858	-0.395694	-0.309276	down
A_23_P86822	CEP57	1.5655918	1.4648762	1.0476875	-0.100716	-0.517904	-0.30931	down
A_33_P3243683	DIAPH1	0.1759682	-0.181894	-0.084995	-0.357862	-0.260963	-0.309412	down
A_33_P3816266	LOC91548	-0.368065	-0.606132	-0.748828	-0.238067	-0.380764	-0.309415	down
A_24_P406006	LPCAT1	0.5908036	-0.245675	0.8083367	-0.836478	0.2175331	-0.309473	down
A_23_P154022	C2orf44	0.9856467	0.4376965	0.9146261	-0.54795	-0.071021	-0.309485	down
A_22_P00002171	LINC00958	-2.587911	-2.687275	-3.107607	-0.099364	-0.519696	-0.30953	down
A_23_P92786	RBM27	4.2627945	3.9118485	3.9945831	-0.350946	-0.268211	-0.309579	down
A_24_P305764	SMS	3.6908607	3.2983394	3.4641924	-0.392521	-0.226668	-0.309595	down
A_33_P3628481	MGC27345	0.410449	0.1004853	0.1011744	-0.309964	-0.309275	-0.309619	down
A_33_P3288942	FAM107B	1.7314272	1.3979068	1.4455495	-0.33352	-0.285878	-0.309699	down
A_23_P209564	CYBRD1	0.717319	0.5871992	0.2278228	-0.13012	-0.489496	-0.309808	down
A_19_P00807384	LOC101927151	-0.029815	-0.40126	-0.278043	-0.371445	-0.248228	-0.309836	down
A_24_P215407	DDX6	2.7700567	2.218346	2.7020674	-0.551711	-0.067989	-0.30985	down
A_33_P3265920	PTPN4	1.7901087	1.3657012	1.59479	-0.424407	-0.195319	-0.309863	down
A_24_P218001	ZNF273	0.1344476	-0.071122	-0.279748	-0.20557	-0.414195	-0.309882	down
A_23_P383986	CHST15	2.035492	1.6059918	1.8449135	-0.4295	-0.190578	-0.310039	down
A_23_P215341	FKBP14	2.3432693	1.9001546	2.165742	-0.443115	-0.177527	-0.310321	down
A_21_P0007215	lnc-PPP1R32-1	-1.510075	-1.49557	-2.145275	0.0145049	-0.6352	-0.310348	down
A_23_P259328	PEX3	1.3189569	0.9682398	1.0488315	-0.350717	-0.270125	-0.310421	down
A_33_P3299872	HINT3	2.7968006	2.102593	2.869915	-0.694208	0.0731144	-0.310547	down
A_33_P3416420	ZUFSP	-0.882735	-1.274438	-1.112224	-0.391703	-0.229489	-0.310596	down
A_23_P68031	STAT4	-1.32987	-1.377791	-1.903341	-0.047921	-0.573472	-0.310696	down
A_33_P3222501	FAM157A	-2.195979	-2.279263	-2.734405	-0.083284	-0.538425	-0.310855	down
A_22_P00007202	lnc-GPBP1L1-1	-2.764555	-3.32739	-2.823506	-0.562835	-0.058952	-0.310894	down
A_23_P163027	PARP2	3.2735434	2.9722552	2.9530287	-0.301288	-0.320515	-0.310901	down
A_23_P145984	TSPAN12	-1.202326	-1.583619	-1.443083	-0.381293	-0.240757	-0.311025	down
A_24_P388703	LOC439933	-1.679682	-1.924396	-2.057251	-0.244714	-0.377569	-0.311141	down
A_24_P324814	CEP95	1.7464504	1.5399652	1.3304434	-0.206485	-0.416007	-0.311246	down
A_21_P0001107	lnc-ID3-4	-1.972412	-2.299228	-2.2681	-0.326816	-0.295688	-0.311252	down
A_22_P00003095	LOC102724710	-1.779028	-2.018191	-2.162384	-0.239162	-0.383355	-0.311259	down
A_23_P92954	HSD17B4	3.4459457	3.0140748	3.2548866	-0.431871	-0.191059	-0.311465	down
A_23_P75288	ATAD1	5.155736	4.7961802	4.8921995	-0.359556	-0.263536	-0.311546	down
A_21_P0014793	OXR1	1.385376	0.9993377	1.148221	-0.386038	-0.237155	-0.311597	down
A_23_P105571	CHPT1	0.8061962	0.4122582	0.5767446	-0.393938	-0.229452	-0.311695	down
A_33_P3343715	EMX1	-2.077322	-2.12859	-2.64966	-0.051268	-0.572338	-0.311803	down
A_23_P33364	SH3D19	4.800726	4.4672484	4.510233	-0.333478	-0.290493	-0.311985	down
A_24_P377499	OSBPL3	0.9500332	0.6340318	0.6419888	-0.316001	-0.308044	-0.312023	down
A_33_P3369262	FGFR1OP2	-1.485385	-1.782495	-1.812337	-0.29711	-0.326952	-0.312031	down
A_19_P00331853	LOC100131564	-1.95011	-2.122357	-2.402075	-0.172247	-0.451965	-0.312106	down
A_23_P65789	MCTP2	-1.423597	-2.022453	-1.449098	-0.598856	-0.0255	-0.312178	down

A_23_P98898	CDK2	0.2213383	-0.102833	-0.078855	-0.324172	-0.300193	-0.312182	down
A_33_P3349325	RAB28	-2.069611	-2.691369	-2.072494	-0.621758	-0.002882	-0.31232	down
A_23_P253932	FAIM	4.475028	4.0295625	4.2957754	-0.445466	-0.179253	-0.312359	down
A_19_P00315543	LINC01512	-1.752556	-1.993474	-2.136532	-0.240918	-0.383976	-0.312447	down
A_23_P53267	RSRC2	4.423424	4.0651507	4.1566877	-0.358273	-0.266736	-0.312505	down
A_33_P3309804	EID1	-1.591432	-1.867679	-1.940264	-0.276247	-0.348832	-0.312539	down
A_23_P337917	PPFIBP1	0.7140789	0.3926449	0.4103241	-0.321434	-0.303755	-0.312594	down
A_24_P56252	PPP1R12A	3.8516712	3.5043216	3.5738173	-0.34735	-0.277854	-0.312602	down
A_33_P3376116	SPC24	-0.994607	-1.455104	-1.159327	-0.460497	-0.16472	-0.312608	down
A_33_P3367392	FAM167B	-1.888341	-2.544194	-1.858236	-0.655853	0.0301046	-0.312874	down
A_33_P3273599	INTU	-1.67899	-1.847029	-2.136717	-0.168039	-0.457727	-0.312883	down
A_33_P3228757	ZNF814	0.6651282	0.4681864	0.2362342	-0.196942	-0.428894	-0.312918	down
A_32_P148796	UBXN2B	1.1602817	1.0178175	0.6769052	-0.142464	-0.483377	-0.31292	down
A_23_P211047	BACH1	0.9599266	0.9431748	0.350699	-0.016752	-0.609228	-0.31299	down
A_23_P97265	GPATCH4	4.6131	4.1269174	4.473281	-0.486183	-0.139819	-0.313001	down
A_23_P63908	TRUB1	2.8169136	2.377274	2.630515	-0.43964	-0.186399	-0.313019	down
A_22_P00024092	SLC38A6	-1.36482	-1.562543	-1.79315	-0.197723	-0.428331	-0.313027	down
A_23_P316974	SYNJ2	2.5505686	2.1540837	2.3207798	-0.396485	-0.229789	-0.313137	down
A_33_P3336527	PDXDC1	2.1952963	1.8046317	1.9595861	-0.390665	-0.23571	-0.313187	down
A_22_P00003261	lnc-CALHM3-2	-1.251895	-1.557499	-1.572768	-0.305604	-0.320874	-0.313239	down
A_24_P261383	TAF1D	3.3101416	3.1702523	2.823491	-0.139889	-0.48665	-0.31327	down
A_33_P3244279	MAGT1	-1.145575	-1.461319	-1.456378	-0.315744	-0.310802	-0.313273	down
A_32_P186474	RACGAP1	3.733571	3.2367854	3.6036224	-0.496786	-0.129949	-0.313367	down
A_33_P3356075	LPIN1	-0.838685	-1.403552	-0.90062	-0.564867	-0.061935	-0.313401	down
A_33_P3221458	ZNF204P	-1.093427	-1.249196	-1.564459	-0.155769	-0.471033	-0.313401	down
A_33_P3349536	CHEK1	2.448842	2.1524472	2.1183395	-0.296395	-0.330503	-0.313449	down
A_23_P5757	TPRKB	4.3188896	3.8665986	4.1441526	-0.452291	-0.174737	-0.313514	down
A_22_P00007397	lnc-GRIK2-2	0.9563818	0.3277273	0.9575062	-0.628655	0.0011244	-0.313765	down
A_33_P3304382	NDUFAF6	1.7879047	1.5389676	1.4088135	-0.248937	-0.379091	-0.314014	down
A_21_P0013984	AK4	0.8853159	0.5568395	0.5854631	-0.328476	-0.299853	-0.314165	down
A_21_P0000296	SNORD63	-1.49711	-1.845125	-1.777576	-0.348015	-0.280466	-0.31424	down
A_23_P133582	ETF1	4.125305	3.890183	3.7318573	-0.235122	-0.393448	-0.314285	down
A_32_P35800	ICE1	1.6514368	1.2808995	1.3933921	-0.370537	-0.258045	-0.314291	down
A_23_P69683	SEC24B	1.5285921	1.0390525	1.3890457	-0.48954	-0.139546	-0.314543	down
A_32_P165116	WDR92	1.5252743	1.0940905	1.3273611	-0.431184	-0.197913	-0.314548	down
A_33_P3253707	LRR1	4.7838125	4.456757	4.4816427	-0.327055	-0.30217	-0.314613	down
A_33_P3384462	THSD4	-1.417667	-1.95232	-1.512362	-0.534653	-0.094695	-0.314674	down
A_24_P313096	TUBGCP3	1.8238859	1.4906259	1.5277848	-0.33326	-0.296101	-0.314681	down
A_22_P00011160	lnc-OBSCN-1	-1.86743	-2.17688	-2.187417	-0.309449	-0.319987	-0.314718	down
A_32_P190461	LOC101926894	-0.64815	-0.857875	-1.068143	-0.209724	-0.419993	-0.314859	down
A_32_P105195	DDX46	1.7800531	1.4816513	1.4487181	-0.298402	-0.331335	-0.314868	down
A_33_P3321697	EPB41L4A	2.7972593	2.440659	2.5240154	-0.3566	-0.273244	-0.314922	down
A_23_P377965	LINC00526	-0.423622	-0.763516	-0.713598	-0.339894	-0.289977	-0.314935	down
A_23_P217384	AP1S2	-0.169624	-0.282873	-0.686266	-0.113249	-0.516642	-0.314945	down
A_24_P403303	PHF20L1	-1.716353	-1.949606	-2.11332	-0.233253	-0.396967	-0.31511	down
A_33_P3326545	LRP5L	0.8194523	0.3973537	0.6112742	-0.422099	-0.208178	-0.315138	down
A_33_P3295655	APBA1	-1.765424	-2.534775	-1.62648	-0.769351	0.1389437	-0.315204	down
A_23_P1775	DPAGT1	1.6606045	0.9669638	1.7238188	-0.693641	0.0632143	-0.315213	down
A_22_P00006449	lnc-FBXW5-1	-1.487588	-1.923767	-1.682044	-0.43618	-0.194456	-0.315318	down
A_23_P391443	PPM1H	-1.902154	-2.428636	-2.006506	-0.526482	-0.104351	-0.315416	down
A_23_P11859	HSD17B7	1.6360855	0.9329138	1.7082663	-0.703172	0.0721808	-0.315495	down
A_24_P165656	PRKD3	2.2256708	1.9303346	1.8896093	-0.295336	-0.336061	-0.315699	down
A_23_P205389	MOAP1	0.7421241	0.5307608	0.3220592	-0.211363	-0.420065	-0.315714	down
A_33_P3292337	KIF5B	1.300302	0.9761515	0.9930186	-0.324151	-0.307283	-0.315717	down
A_23_P145397	CCNC	2.2758112	1.9031138	2.0170364	-0.372697	-0.258775	-0.315736	down
A_33_P3318465	SUMO1	5.47622	5.13359	5.187114	-0.34263	-0.289106	-0.315868	down

A_23_P48886	ADAM10	2.6545935	2.2108693	2.4664955	-0.443724	-0.188098	-0.315911	down
A_23_P66881	RGS9	-0.421348	-0.892648	-0.581986	-0.4713	-0.160638	-0.315969	down
A_33_P3209351	IARS	3.0708723	2.6817908	2.828004	-0.389081	-0.242868	-0.315975	down
A_32_P119616	ETFA	2.6770515	2.314362	2.4076176	-0.36269	-0.269434	-0.316062	down
A_23_P382705	TMTC2	1.6982765	0.9934378	1.7708421	-0.704839	0.0725656	-0.316137	down
A_33_P3296871	ZNF33B	2.289689	1.9781737	1.9688759	-0.311515	-0.320813	-0.316164	down
A_33_P3305243	PSMD5	-1.846874	-2.064261	-2.26208	-0.217388	-0.415206	-0.316297	down
A_22_P00010686	CYB5B	1.1060987	0.6450424	0.9344058	-0.461056	-0.171693	-0.316375	down
A_23_P87769	PARPBP	3.0297642	2.5841994	2.8420963	-0.445565	-0.187668	-0.316616	down
A_22_P00003977	lnc-CHD9-1	-2.749939	-2.730488	-3.402632	0.0194507	-0.652693	-0.316621	down
A_23_P150325	TMEM133	-0.836391	-1.178925	-1.1271	-0.342534	-0.290709	-0.316621	down
A_21_P0007249	RAB30-AS1	-1.590069	-1.764974	-2.048552	-0.174905	-0.458483	-0.316694	down
A_33_P3395688	MYZAP	-1.768116	-2.163843	-2.005818	-0.395727	-0.237703	-0.316715	down
A_24_P75220	MAGI1	-2.284676	-2.443589	-2.75928	-0.158913	-0.474604	-0.316758	down
A_33_P3364112	FRS2	0.8332987	0.5223765	0.5105872	-0.310922	-0.322711	-0.316817	down
A_33_P3224595	OFD1	0.6034393	0.349792	0.2234478	-0.253647	-0.379992	-0.316819	down
A_23_P166779	LINC00312	-2.657313	-3.04613	-2.902293	-0.388816	-0.244979	-0.316898	down
A_33_P3273238	ZNF320	0.4604912	0.0696325	0.2175202	-0.390859	-0.242971	-0.316915	down
A_23_P69877	ZFP62	4.3569164	4.3312955	3.7485857	-0.025621	-0.608331	-0.316976	down
A_24_P778836	RSL1D1	3.3807478	2.9960456	3.1313477	-0.384702	-0.2494	-0.317051	down
A_23_P403521	YAE1D1	2.096365	1.7664208	1.7921805	-0.329944	-0.304184	-0.317064	down
A_23_P434890	CARD10	5.5066404	5.071545	5.307431	-0.435095	-0.199209	-0.317152	down
A_24_P50458	TERF1	5.019828	4.78151	4.6236353	-0.238318	-0.396193	-0.317255	down
A_32_P196115	ZSCAN25	-2.096674	-2.619655	-2.208381	-0.522981	-0.111707	-0.317344	down
A_23_P45185	FIGF	-2.692469	-2.752238	-3.267398	-0.059769	-0.57493	-0.317349	down
A_24_P310756	FBXO28	2.5313063	2.1011405	2.326686	-0.430166	-0.20462	-0.317393	down
A_23_P24365	ANKRD49	2.3778524	2.0395427	2.0812654	-0.33831	-0.296587	-0.317448	down
A_23_P159125	SLC16A5	5.8486824	5.424621	5.637785	-0.424061	-0.210897	-0.317479	down
A_24_P100551	SH3RF1	4.3387794	3.8599133	4.1819916	-0.478866	-0.156788	-0.317827	down
A_24_P117138	ZNF626	0.7374463	0.4526234	0.386395	-0.284823	-0.351051	-0.317937	down
A_33_P3373046	ZNF772	-2.925911	-3.088738	-3.398959	-0.162827	-0.473048	-0.317937	down
A_24_P795594	ALG9	-0.469857	-0.958214	-0.617375	-0.488357	-0.147519	-0.317938	down
A_23_P69452	DBR1	0.7954202	0.495183	0.4595785	-0.300237	-0.335842	-0.318039	down
A_24_P57170	PROSER3	-0.453382	-0.636549	-0.906334	-0.183167	-0.452952	-0.318059	down
A_33_P3260722	UVRAG	0.5003042	-0.029137	0.3933272	-0.529441	-0.106977	-0.318209	down
A_33_P3372979	LINC00294	0.4150152	0.2306032	-0.037059	-0.184412	-0.452075	-0.318243	down
A_33_P3832852	HAUS2	1.9808302	1.603364	1.7215443	-0.377466	-0.259286	-0.318376	down
A_21_P0010774	lnc-PPIAL4G-4	-1.390409	-1.872516	-1.545117	-0.482107	-0.154708	-0.318408	down
A_23_P84872	SECISBP2	-0.237655	-0.274457	-0.837678	-0.036802	-0.600023	-0.318413	down
A_33_P3409159	SLC22A23	-1.413514	-1.648823	-1.815442	-0.23531	-0.401928	-0.318619	down
A_23_P7342	UGT2B10	-1.332073	-1.738998	-1.562411	-0.406925	-0.230339	-0.318632	down
A_33_P3316983	BBIP1	2.0408602	2.0743566	1.3699245	0.0334964	-0.670936	-0.31872	down
A_23_P207280	APPBP2	1.5821867	1.3158331	1.2108531	-0.266354	-0.371334	-0.318844	down
A_21_P0001652	lnc-AKT3-2	-2.364858	-2.240896	-3.126651	0.1239619	-0.761794	-0.318916	down
A_32_P16258	EXOC6B	-0.882657	-1.155603	-1.247679	-0.272946	-0.365022	-0.318984	down
A_23_P6914	OSBPL11	1.6827459	1.3381042	1.3893347	-0.344642	-0.293411	-0.319026	down
A_23_P435002	SRFBP1	1.7178373	1.3410106	1.4562263	-0.376827	-0.261611	-0.319219	down
A_33_P3325439	USP16	0.8871255	0.6493497	0.4864516	-0.237776	-0.400674	-0.319225	down
A_23_P259357	SLC35E4	9.296105	8.9779625	8.975751	-0.318143	-0.320354	-0.319249	down
A_22_P00015892	lnc-TCF19-1	-1.737523	-2.195793	-1.917759	-0.45827	-0.180236	-0.319253	down
A_24_P416346	ETV4	0.6672568	-0.020594	0.7163367	-0.687851	0.0490799	-0.319385	down
A_19_P00805523	MIR4458HG	-2.15727	-2.696794	-2.25665	-0.539524	-0.09938	-0.319452	down
A_23_P40989	USP13	1.2594142	0.8218823	1.0580416	-0.437532	-0.201373	-0.319452	down
A_23_P81529	ISL1	-0.067662	-0.799106	0.0248089	-0.731444	0.0924706	-0.319487	down
A_32_P44568	LDHA	4.7289066	4.206635	4.612153	-0.522272	-0.116754	-0.319513	down
A_23_P91076	TMEM87B	0.7332783	0.5919132	0.2356091	-0.141365	-0.497669	-0.319517	down

A_23_P12053	SPEN	1.4619694	1.2426147	1.0421166	-0.219355	-0.419853	-0.319604	down
A_33_P3261902	ENOSF1	3.2675009	2.9711776	2.92453	-0.296323	-0.342971	-0.319647	down
A_33_P3256738	ARHGEF39	-2.235277	-2.569308	-2.540871	-0.334031	-0.305595	-0.319813	down
A_33_P3245389	TMEM260	1.7898607	1.3311896	1.608849	-0.458671	-0.181012	-0.319841	down
A_22_P00016361	lnc-TMEM179-1	-2.60893	-3.074326	-2.78326	-0.465396	-0.17433	-0.319863	down
A_23_P205255	WDR20	-1.200185	-1.559162	-1.481138	-0.358977	-0.280953	-0.319965	down
A_23_P500000	SCEL	3.8020105	3.634293	3.3295574	-0.167717	-0.472453	-0.320085	down
A_33_P3289192	HBS1L	4.211622	3.8188996	3.9640121	-0.392723	-0.24761	-0.320166	down
A_21_P0000155	PSMC2	-0.813064	-1.053915	-1.213323	-0.24085	-0.400259	-0.320555	down
A_22_P00008175	lnc-INPP4B-2	-2.80338	-2.982262	-3.265741	-0.178882	-0.462361	-0.320621	down
A_33_P3305731	NINL	-1.765284	-2.010717	-2.161176	-0.245434	-0.395893	-0.320663	down
A_23_P80827	FYTTD1	2.6066504	2.1275544	2.4443502	-0.479096	-0.1623	-0.320698	down
A_33_P3414695	HP1BP3	2.151536	1.9314799	1.7300086	-0.220056	-0.421527	-0.320792	down
A_33_P3217218	AGPAT5	2.1922665	1.9123507	1.8305349	-0.279916	-0.361732	-0.320824	down
A_22_P00001465	lnc-AQP8-1	-1.226598	-1.425283	-1.669779	-0.198684	-0.443181	-0.320932	down
A_23_P82206	ECHDC1	3.359662	2.8804545	3.1968145	-0.479208	-0.162848	-0.321028	down
A_22_P00001788	lnc-ATP4B-1	-0.675727	-1.162984	-0.830531	-0.487257	-0.154804	-0.32103	down
A_33_P3292812	GPATCH2L	0.8361316	0.4995546	0.5305371	-0.336577	-0.305594	-0.321086	down
A_32_P114896	PTGES3	2.9215431	2.6389737	2.561903	-0.282569	-0.35964	-0.321105	down
A_23_P2683	RPAP3	1.835906	1.4201164	1.6094193	-0.41579	-0.226487	-0.321138	down
A_21_P0014553	DUXAP10	-1.748387	-2.001122	-2.137961	-0.252735	-0.389574	-0.321155	down
A_32_P204676	FABP5	5.7683363	5.310992	5.5833073	-0.457345	-0.185029	-0.321187	down
A_33_P3279049	UPF2	-0.829235	-1.263167	-1.037693	-0.433932	-0.208458	-0.321195	down
A_33_P3242820	LOC115110	-1.056248	-1.379881	-1.37514	-0.323633	-0.318892	-0.321262	down
A_23_P104762	YAP1	3.510005	3.0792508	3.2978497	-0.430754	-0.212155	-0.321455	down
A_23_P216149	TERF1	5.010315	4.7688346	4.608884	-0.24148	-0.401431	-0.321456	down
A_21_P0005354	lnc-OCM-1	-0.887105	-1.138423	-1.278699	-0.251318	-0.391595	-0.321457	down
A_23_P7873	MCM3	5.834037	5.635863	5.389185	-0.198174	-0.444852	-0.321513	down
A_32_P194246	CLEC16A	2.8541946	2.3277574	2.7375307	-0.526437	-0.116664	-0.321551	down
A_33_P3220728	RREB1	-1.035583	-1.357687	-1.356704	-0.322104	-0.321121	-0.321612	down
A_23_P65208	ZMYM5	-0.112914	-0.466406	-0.40328	-0.353492	-0.290366	-0.321929	down
A_23_P212617	TFRC	6.390396	5.9724736	6.16426	-0.417923	-0.226136	-0.322029	down
A_32_P193822	ZC3H11A	1.8482752	1.6155968	1.4366231	-0.232678	-0.411652	-0.322165	down
A_33_P3213857	RNASEH2B	3.444004	3.0695214	3.1741447	-0.374483	-0.269859	-0.322171	down
A_33_P3350758	RASAL2	0.6167522	0.2404647	0.3486214	-0.376287	-0.268131	-0.322209	down
A_24_P303989	BMI1	1.0827174	0.8259497	0.6948457	-0.256768	-0.387872	-0.32232	down
A_24_P169048	RFPL3S	-1.905054	-2.058309	-2.396521	-0.153255	-0.491467	-0.322361	down
A_22_P00002332	LINC01493	-2.743766	-3.210045	-2.922412	-0.46628	-0.178647	-0.322463	down
A_23_P217109	SLC31A2	1.5489254	1.3626146	1.090291	-0.186311	-0.458634	-0.322473	down
A_23_P214222	MARCKS	5.3985653	4.9918275	5.1602554	-0.406738	-0.23831	-0.322524	down
A_24_P193592	CCNF	-0.588144	-1.011509	-0.809943	-0.423365	-0.221799	-0.322582	down
A_32_P170003	TRAF3IP1	1.8708296	1.2286415	1.8677855	-0.642188	-0.003044	-0.322616	down
A_23_P7697	SNX2	4.7675724	4.397424	4.492465	-0.370148	-0.275107	-0.322628	down
A_33_P3404097	PGM5P2	7.193158	6.943028	6.7979965	-0.25013	-0.395162	-0.322646	down
A_24_P64918	ZBTB1	-0.46882	-0.641966	-0.940981	-0.173146	-0.472161	-0.322654	down
A_33_P3240392	FOXO3	4.6017	4.4255376	4.132393	-0.176162	-0.469307	-0.322735	down
A_21_P0000017	RPS20	-2.833227	-3.206691	-3.105419	-0.373463	-0.272192	-0.322827	down
A_24_P254949	PGM5	-2.094163	-2.381289	-2.452737	-0.287126	-0.358575	-0.32285	down
A_22_P00013864	LINC00593	-2.364939	-2.477304	-2.898379	-0.112365	-0.53344	-0.322902	down
A_23_P99172	CCDC77	2.133397	1.8300862	1.7907195	-0.303311	-0.342678	-0.322994	down
A_21_P0002591	lnc-SIX3-3	-1.399889	-1.397512	-2.04844	0.0023766	-0.648551	-0.323087	down
A_23_P218131	INF2	1.5510979	0.8750739	1.5807195	-0.676024	0.0296216	-0.323201	down
A_21_P0000581	PARGP1	-0.478837	-0.955851	-0.648333	-0.477014	-0.169496	-0.323255	down
A_33_P3743508	LOC158435	3.0853786	2.7523646	2.7716475	-0.333014	-0.313731	-0.323373	down
A_32_P76720	NT5DC3	-0.677002	-0.938522	-1.062229	-0.261519	-0.385227	-0.323373	down
A_21_P0000098	CARD8	-1.863403	-2.321401	-2.052332	-0.457998	-0.188929	-0.323464	down

A_24_P357709	VPS8	0.7406168	0.3811245	0.4531775	-0.359492	-0.287439	-0.323466	down
A_23_P422851	CABLES1	0.5904355	0.4705782	0.0632057	-0.119857	-0.52723	-0.323544	down
A_23_P77201	C15orf41	0.2949405	0.2059245	-0.263164	-0.089016	-0.558105	-0.32356	down
A_23_P43157	MYBL1	-2.336746	-2.955188	-2.365455	-0.618442	-0.02871	-0.323576	down
A_33_P3771067	LOC283140	-1.601347	-1.901967	-1.947924	-0.300621	-0.346577	-0.323599	down
A_21_P0000909	LOC284600	-0.77058	-0.831463	-1.35713	-0.060883	-0.586549	-0.323716	down
A_33_P3382709	LEMD3	3.0192919	2.7939706	2.5971556	-0.225321	-0.422136	-0.323729	down
A_33_P3211260	DBNL	-0.50058	-0.591507	-1.057571	-0.090927	-0.55699	-0.323959	down
A_33_P3588134	PANX2	-1.734814	-2.131037	-1.986544	-0.396223	-0.25173	-0.323976	down
A_33_P3272231	MFSD2A	1.511168	0.8610821	1.5132494	-0.650086	0.0020814	-0.324002	down
A_33_P3424257	NAA50	7.177759	6.764897	6.9423943	-0.412862	-0.235365	-0.324114	down
A_24_P353638	SLAMF7	-0.477083	-0.007128	-1.595592	0.469955	-1.118509	-0.324277	down
A_21_P0014806	LOC100507131	-0.156803	-0.333275	-0.62916	-0.176473	-0.472357	-0.324415	down
A_24_P8318	AFF4	0.108736	-0.205113	-0.226327	-0.313849	-0.335063	-0.324456	down
A_22_P00019692	lnc-HIST4H4-1	-1.811434	-2.458952	-1.813054	-0.647518	-0.00162	-0.324569	down
A_24_P649747	BMS1	2.3503866	1.9536242	2.0974588	-0.396762	-0.252928	-0.324845	down
A_23_P214411	GLO1	2.053917	1.5628443	1.8951836	-0.491073	-0.158733	-0.324903	down
A_22_P00012025	TTN-AS1	-1.169548	-1.339365	-1.649611	-0.169817	-0.480063	-0.32494	down
A_19_P00316985	LINC00472	-1.294061	-1.383421	-1.854812	-0.08936	-0.560751	-0.325056	down
A_33_P3851513	LIN54	1.8410234	1.5759907	1.4555182	-0.265033	-0.385505	-0.325269	down
A_23_P135061	CORO2A	2.8849497	2.3247266	2.7944975	-0.560223	-0.090452	-0.325338	down
A_21_P0012336	GGTLC1	0.2008448	-0.182071	-0.067164	-0.382916	-0.268009	-0.325462	down
A_23_P105276	ZNF84	1.1296568	0.9636602	0.6446834	-0.165997	-0.484973	-0.325485	down
A_23_P124252	CAMK1D	-0.51516	-1.019391	-0.661932	-0.504231	-0.146772	-0.325502	down
A_23_P398770	PWWP2A	1.9175587	1.6065631	1.577344	-0.310996	-0.340215	-0.325605	down
A_19_P00804020	TLE1	-0.52958	-0.744024	-0.966576	-0.214445	-0.436996	-0.32572	down
A_23_P106922	CHST6	-0.648479	-1.538729	-0.409842	-0.890249	0.2386375	-0.325806	down
A_24_P167614	INTS6	1.5008731	1.3101778	1.039896	-0.190695	-0.460977	-0.325836	down
A_33_P3727762	SET	7.38573	7.3069367	6.81275	-0.078793	-0.57298	-0.325886	down
A_24_P247732	SLC5A6	-2.151609	-2.623852	-2.331156	-0.472243	-0.179547	-0.325895	down
A_33_P3314161	YAF2	1.5601282	0.979135	1.4890327	-0.580993	-0.071095	-0.326044	down
A_23_P325625	SCAI	-1.310042	-1.911872	-1.360317	-0.60183	-0.050275	-0.326052	down
A_23_P25019	PRIM1	2.7730799	2.4882932	2.4054308	-0.284787	-0.367649	-0.326218	down
A_24_P728006	XLOC_l2_011874	-1.258342	-1.465729	-1.703643	-0.207387	-0.445301	-0.326344	down
A_23_P59677	RINT1	0.3877153	-0.046059	0.1685014	-0.433774	-0.219214	-0.326494	down
A_23_P41541	PRMT9	6.437E-05	-0.357635	-0.29523	-0.357699	-0.295294	-0.326497	down
A_22_P00024589	lnc-KB-1980E6.3.1-1	-0.593502	-1.139262	-0.700755	-0.54576	-0.107253	-0.326506	down
A_33_P3320393	MRPS16	7.472039	7.1084056	7.1826077	-0.363634	-0.289432	-0.326533	down
A_22_P00019374	lnc-RDH5-1	-2.137014	-2.486841	-2.440524	-0.349827	-0.30351	-0.326668	down
A_21_P0014019	LGALS2	-0.623945	-0.798121	-1.103374	-0.174176	-0.479429	-0.326802	down
A_33_P3340040	GINS4	4.25935	3.782887	4.08208	-0.476463	-0.17727	-0.326866	down
A_22_P00005914	HOXA-AS2	-2.467793	-3.017704	-2.571794	-0.549911	-0.104001	-0.326956	down
A_33_P3577671	DPP8	3.7388105	3.2996678	3.5239248	-0.439143	-0.214886	-0.327014	down
A_21_P0014594	lnc-PDCD11-1	-2.329871	-2.922068	-2.391848	-0.592198	-0.061977	-0.327088	down
A_23_P102471	MSH2	4.388157	3.9791675	4.1429243	-0.408989	-0.245233	-0.327111	down
A_33_P3360718	CDC27	0.0475116	-0.19897	-0.36028	-0.246482	-0.407791	-0.327137	down
A_33_P3297562	IRX2	4.611779	4.2385	4.3304157	-0.373279	-0.281364	-0.327321	down
A_33_P3233700	HPN-AS1	-2.373844	-2.943002	-2.459479	-0.569158	-0.085635	-0.327397	down
A_21_P0011550	LINC00674	0.5288529	-0.028755	0.4315052	-0.557608	-0.097348	-0.327478	down
A_21_P0013556	XLOC_l2_014711	3.0560293	2.7718177	2.6846542	-0.284212	-0.371375	-0.327793	down
A_33_P3324383	FIGNL2	-0.505545	-0.871744	-0.794976	-0.366199	-0.289431	-0.327815	down
A_33_P3304372	TMEM144	1.5544395	1.1753788	1.2774167	-0.379061	-0.277023	-0.328042	down
A_33_P3343145	MAP1B	-0.514976	-0.700821	-0.98522	-0.185845	-0.470244	-0.328045	down
A_23_P301896	PRDM2	1.0927782	0.8256345	0.7038136	-0.267144	-0.388965	-0.328054	down
A_33_P3326588	TNFRSF10D	2.1931553	1.82237	1.9076567	-0.370785	-0.285499	-0.328142	down

A_33_P3269598	ERCC6L2	-0.23354	-0.506747	-0.616624	-0.273207	-0.383084	-0.328146	down
A_24_P917833	TMED10	3.7766008	3.3457236	3.551176	-0.430877	-0.225425	-0.328151	down
A_23_P92424	UTP3	4.897703	4.4774117	4.661639	-0.420291	-0.236064	-0.328178	down
A_24_P318939	ZNF337	-0.914227	-1.273061	-1.21178	-0.358834	-0.297553	-0.328193	down
A_21_P0014736	LOC100506538	-2.468942	-2.766615	-2.827659	-0.297673	-0.358717	-0.328195	down
A_23_P119857	TTC32	1.8047724	1.4037185	1.5493155	-0.401054	-0.255457	-0.328255	down
A_23_P75516	PPFIA1	0.8432932	0.6030812	0.4267216	-0.240212	-0.416572	-0.328392	down
A_21_P0004439	LOC101927488	-2.852299	-2.864	-3.497392	-0.011701	-0.645093	-0.328397	down
A_33_P3335725	INSR	1.3050098	0.883574	1.0695319	-0.421436	-0.235478	-0.328457	down
A_33_P3259403	PAN3	-2.077409	-2.392838	-2.419088	-0.315429	-0.341679	-0.328554	down
A_33_P3320994	SYDE2	-0.189583	-0.357666	-0.678629	-0.168082	-0.489046	-0.328564	down
A_33_P3342285	RAP1A	4.8048735	4.4409785	4.51151	-0.363895	-0.293364	-0.328629	down
A_33_P3368471	NT5DC1	0.944942	0.5411553	0.6913686	-0.403787	-0.253573	-0.32868	down
A_24_P81298	PPP6C	0.3379116	-0.076361	0.0947776	-0.414272	-0.243134	-0.328703	down
A_23_P135977	CKAP5	3.3253574	2.797048	3.1961956	-0.528309	-0.129162	-0.328736	down
A_23_P57697	DHX36	4.345703	4.0335927	3.9999971	-0.31211	-0.345706	-0.328908	down
A_33_P3336700	SHROOM3	4.038642	3.5026631	3.9167757	-0.535979	-0.121866	-0.328923	down
A_32_P108655	AK4	4.3734894	3.9992433	4.0897436	-0.374246	-0.283746	-0.328996	down
A_23_P3956	C1QTNF1	-1.000275	-1.146222	-1.51285	-0.145947	-0.512575	-0.329261	down
A_33_P3240263	POMP	2.0787964	1.6676507	1.8312359	-0.411146	-0.247561	-0.329353	down
A_33_P3355371	TTC9C	-1.635489	-1.792446	-2.13724	-0.156957	-0.501751	-0.329354	down
A_24_P215628	DCUN1D2	-0.410424	-0.679275	-0.800373	-0.26885	-0.389948	-0.329399	down
A_33_P3287862	LOC101060524	-2.651233	-3.096941	-2.864578	-0.445708	-0.213345	-0.329527	down
A_33_P3249574	SCAMP1	0.7188706	0.4751964	0.30337	-0.243674	-0.415501	-0.329587	down
A_33_P3349693	LCLAT1	0.5911393	0.1093631	0.4135389	-0.481776	-0.1776	-0.329688	down
A_24_P98555	FAM45A	0.0131712	-0.254163	-0.378968	-0.267334	-0.392139	-0.329736	down
A_33_P3330666	FLJ36777	-0.683013	-0.882637	-1.142878	-0.199625	-0.459865	-0.329745	down
A_33_P3389653	PDE4D	1.570096	0.9698682	1.5107899	-0.600228	-0.059306	-0.329767	down
A_33_P3369393	NCF1	-2.39884	-2.546067	-2.91125	-0.147226	-0.512409	-0.329818	down
A_22_P00013078	LINC01089	-1.562264	-1.960347	-1.823909	-0.398083	-0.261645	-0.329864	down
A_23_P83149	ABHD17B	-1.164025	-1.603726	-1.384079	-0.439701	-0.220053	-0.329877	down
A_33_P3423631	WHSC1	-0.101717	-0.362807	-0.50041	-0.26109	-0.398693	-0.329891	down
A_23_P107612	RAB27B	-1.051332	-1.39749	-1.365096	-0.346158	-0.313764	-0.329961	down
A_23_P42718	NFE2L3	3.5799236	3.087422	3.4124765	-0.492502	-0.167447	-0.329974	down
A_33_P3257513	FAT3	2.2331371	1.9896445	1.816453	-0.243493	-0.416684	-0.330088	down
A_33_P3420247	LINC01501	-1.383672	-1.908667	-1.518892	-0.524995	-0.13522	-0.330107	down
A_32_P141488	XLOC_l2_009859	-1.067223	-1.67774	-1.117086	-0.610518	-0.049863	-0.33019	down
A_33_P3281358	ARF3	-1.137929	-1.423723	-1.512518	-0.285794	-0.37459	-0.330192	down
A_23_P21162	TCTEX1D2	3.430666	3.004108	3.1967936	-0.426558	-0.233872	-0.330215	down
A_23_P137434	RNF11	5.163827	4.700908	4.9662886	-0.462919	-0.197538	-0.330229	down
A_23_P3681	NETO2	3.0542889	2.582542	2.8654385	-0.471747	-0.18885	-0.330299	down
A_21_P0010724	LOC645166	-0.984593	-1.105094	-1.524725	-0.120501	-0.540132	-0.330316	down
A_33_P3284029	CSE1L	5.0201283	4.5589633	4.8204155	-0.461165	-0.199713	-0.330439	down
A_33_P3293169	AMBRA1	-2.187469	-2.382275	-2.653569	-0.194806	-0.4661	-0.330453	down
A_23_P30972	ASCC3	1.7692289	1.3767533	1.5007505	-0.392476	-0.268478	-0.330477	down
A_32_P141612	ORC4	-0.090071	-0.425597	-0.415574	-0.335526	-0.325503	-0.330515	down
A_22_P00015941	LOC101927104	-0.748134	-1.411511	-0.745894	-0.663378	0.0022397	-0.330569	down
A_23_P503200	PHF10	2.3225489	1.8195729	2.1641064	-0.502976	-0.158443	-0.330709	down
A_33_P3420747	YTHDC2	2.0942745	1.8128548	1.7141623	-0.28142	-0.380112	-0.330766	down
A_19_P00320119	TUG1	2.9826632	2.4588532	2.8448248	-0.52381	-0.137838	-0.330824	down
A_24_P251841	SETD7	1.885519	1.6578393	1.4514985	-0.22768	-0.434021	-0.33085	down
A_24_P10137	RGCC	-0.349662	-0.486238	-0.87494	-0.136576	-0.525278	-0.330927	down
A_33_P3335730	FLJ16171	-2.234333	-2.455066	-2.675873	-0.220734	-0.441541	-0.331137	down
A_33_P3315425	METTL4	0.6715908	0.4891419	0.1917095	-0.182449	-0.479881	-0.331165	down
A_23_P69573	GUCY1A3	2.6928558	2.3899207	2.333373	-0.302935	-0.359483	-0.331209	down
A_23_P74115	RAD54L	3.8261719	3.499083	3.4908075	-0.327089	-0.335364	-0.331227	down

A_22_P00002555	lnc-C17orf75-2	-2.653001	-2.85827	-3.110372	-0.20527	-0.457372	-0.331321	down
A_23_P8311	TDP2	3.6655874	3.417601	3.250822	-0.247986	-0.414765	-0.331376	down
A_21_P0003853	lnc-FAM160A1-1	2.1846552	1.794136	1.9123173	-0.390519	-0.272338	-0.331429	down
A_21_P0000548	PDCD6IP	-2.23259	-2.725523	-2.402686	-0.492933	-0.170096	-0.331514	down
A_33_P3272580	FUCA2	1.1949854	0.9196057	0.807312	-0.27538	-0.387673	-0.331527	down
A_23_P14734	RPS27L	6.3711014	5.833405	6.2456512	-0.537696	-0.12545	-0.331573	down
A_21_P0007248	lnc-PCF11-1	0.9794087	0.4982429	0.7973013	-0.481166	-0.182107	-0.331637	down
A_21_P0014601	SFPQ	3.7144709	3.3894048	3.3761492	-0.325066	-0.338322	-0.331694	down
A_32_P2452	TMTC1	-1.241429	-1.741097	-1.405156	-0.499667	-0.163726	-0.331697	down
A_23_P70785	AIM1	1.4503784	1.046246	1.1907544	-0.404132	-0.259624	-0.331878	down
A_32_P88635	NBPF3	-1.244425	-1.68304	-1.469747	-0.438615	-0.225322	-0.331969	down
A_24_P364057	C11orf70	-0.107103	-0.878346	0.0001831	-0.771244	0.107286	-0.331979	down
A_22_P00012088	lnc-PMM2-6	2.712596	2.073524	2.6876774	-0.639072	-0.024919	-0.331995	down
A_24_P76995	ZDHHC17	-2.196564	-2.594376	-2.462751	-0.397813	-0.266187	-0.332	down
A_24_P38081	FKBP5	-0.414602	-0.719389	-0.773893	-0.304787	-0.359291	-0.332039	down
A_33_P3305851	SELT	-1.203733	-1.430318	-1.64137	-0.226585	-0.437637	-0.332111	down
A_33_P3317937	KRTAP20-1	-2.082857	-2.305178	-2.525071	-0.222321	-0.442214	-0.332267	down
A_24_P73730	CCDC113	-1.262912	-1.410503	-1.779866	-0.147591	-0.516954	-0.332273	down
A_24_P158421	SAR1A	4.0905123	3.720574	3.7957268	-0.369938	-0.294786	-0.332362	down
A_32_P82475	SNHG10	1.1268439	0.5679817	1.0208526	-0.558862	-0.105991	-0.332427	down
A_23_P54079	OSGEP	1.352159	1.0560699	0.9832792	-0.296089	-0.36888	-0.332484	down
A_23_P16562	SUGP2	1.6846399	1.3138294	1.3904395	-0.370811	-0.2942	-0.332505	down
A_33_P3331345	DUSP11	-0.232955	-0.616607	-0.514442	-0.383651	-0.281487	-0.332569	down
A_33_P3375646	GTPBP6	-1.455706	-1.67672	-1.899899	-0.221014	-0.444192	-0.332603	down
A_33_P3224680	TRMT13	0.2493706	0.0740757	-0.240718	-0.175295	-0.490089	-0.332692	down
A_21_P0013695	EBLN3	-0.199342	-0.62524	-0.43891	-0.425899	-0.239568	-0.332733	down
A_23_P20045	PEX1	0.8191457	0.4571767	0.5152016	-0.361969	-0.303944	-0.332957	down
A_23_P431268	PLEKHA6	3.064763	2.5461802	2.917408	-0.518583	-0.147355	-0.332969	down
A_23_P53957	COX16	5.88385	5.6283774	5.473299	-0.255473	-0.410551	-0.333012	down
A_23_P70047	MATR3	3.4269342	3.1566644	3.0311213	-0.27027	-0.395813	-0.333041	down
A_24_P944616	HP1BP3	4.2068586	4.0149903	3.7323322	-0.191868	-0.474526	-0.333197	down
A_23_P88303	HSPA2	2.7713547	2.3825402	2.4929342	-0.388814	-0.27842	-0.333617	down
A_22_P00016093	LOC101928402	-0.394197	-0.838126	-0.61771	-0.44393	-0.223513	-0.333721	down
A_22_P00020072	lnc-BAG3-1	-1.551377	-1.970292	-1.799974	-0.418915	-0.248597	-0.333756	down
A_23_P85188	ARMCX5	2.450696	1.7357502	2.4980001	-0.714946	0.0473042	-0.333821	down
A_33_P3278755	SULT6B1	-1.734232	-2.168272	-1.96784	-0.434039	-0.233608	-0.333824	down
A_24_P141786	ATP2A2	1.3260899	1.0544372	0.9299607	-0.271653	-0.396129	-0.333891	down
A_33_P3214650	TAP2	3.2035685	2.6780257	3.061307	-0.525543	-0.142262	-0.333902	down
A_23_P61406	SHC3	-1.398132	-1.947279	-1.516924	-0.549147	-0.118792	-0.333969	down
A_23_P104201	YME1L1	5.2140512	4.8878655	4.8722296	-0.326186	-0.341822	-0.334004	down
A_33_P3412035	UBA6	-1.295692	-1.60496	-1.654503	-0.309268	-0.35881	-0.334039	down
A_22_P00007883	HSD17B11	2.118618	1.7183704	1.8505011	-0.400248	-0.268117	-0.334182	down
A_23_P435610	EMC1	0.333797	-0.177274	0.1764021	-0.511071	-0.157395	-0.334233	down
A_23_P132526	PIK3R4	1.100831	0.6443777	0.8888164	-0.456453	-0.212015	-0.334234	down
A_33_P3387145	SH3KBP1	-0.869242	-0.870316	-1.536734	-0.001074	-0.667492	-0.334283	down
A_23_P84821	MRPL1	0.3893633	-0.096333	0.2064381	-0.485696	-0.182925	-0.334311	down
A_21_P0000326	SNORA38	-2.000024	-2.418585	-2.250109	-0.418561	-0.250085	-0.334323	down
A_33_P3379775	TLE1	0.0374146	-0.321661	-0.272697	-0.359076	-0.310112	-0.334594	down
A_33_P3346498	OPA1	0.2937737	-0.083995	0.0020356	-0.377769	-0.291738	-0.334753	down
A_23_P41327	LYAR	3.4572706	2.9292722	3.3157587	-0.527998	-0.141512	-0.334755	down
A_33_P3325023	ERLEC1	2.4427385	1.7432146	2.4725952	-0.699524	0.0298567	-0.334834	down
A_19_P00319537	LINC01091	-2.463083	-2.799754	-2.796108	-0.336671	-0.333025	-0.334848	down
A_23_P46819	BTRC	-0.368476	-0.86734	-0.539351	-0.498864	-0.170875	-0.334869	down
A_23_P143952	CEP97	-1.847938	-2.269547	-2.096368	-0.42161	-0.248431	-0.33502	down
A_23_P165788	PHOSPHO2	2.6784267	2.2005782	2.4861917	-0.477849	-0.192235	-0.335042	down
A_33_P3373775	BCL2L15	1.3194413	1.1115446	0.8570471	-0.207897	-0.462394	-0.335145	down

A_33_P3284152	LOC285191	-2.356716	-2.624813	-2.758911	-0.268097	-0.402195	-0.335146	down
A_32_P222961	SPIN4	2.2393713	1.8842249	1.9242067	-0.355146	-0.315165	-0.335155	down
A_23_P101281	ZNF587	3.4852514	3.0424342	3.257576	-0.442817	-0.227675	-0.335246	down
A_21_P0007826	lnc-MGST1-2	-1.32813	-1.857638	-1.46935	-0.529509	-0.141221	-0.335365	down
A_23_P92042	ITPR1	-1.597013	-1.828771	-2.036144	-0.231758	-0.439132	-0.335445	down
A_33_P3337599	PRKDC	-1.538905	-1.873446	-1.875294	-0.334541	-0.336389	-0.335465	down
A_24_P358868	ZNF728	-0.998483	-1.270579	-1.397334	-0.272096	-0.398851	-0.335474	down
[truncated: 473,090 more chars]
